# Supplementary material for: Polarizability matters in enantio-selection
Source: Nat Commun. 2024 Apr 22;15:3394. doi: 10.1038/s41467-024-47813-4 (PMC11035643; doi:10.1038/s41467-024-47813-4)

Supplementary Information

Polarizability Matters in Enantio-selection

Fumin Chen, [1,2]† Yu Chen, [2]† Xiao-Yong Chang,[2] Dongxu He,[2] Qingjing Yang,[2] David Zhigang Wang,[3] Chen Xu,*[2] Peiyuan Yu,*[2] and Xiangyou Xing*[2]

[1]School of Chemistry and Chemical Engineering, Harbin Institute of Technology, Harbin 150001, China

[2]Shenzhen Grubbs Institute and Department of Chemistry, Guangdong Provincial Key Laboratory of Catalysis, Southern University of Science and Technology, Shenzhen 518055, China

[3]Shenzhen Youwei Tech Group, Shenzhen 518057, China

Corresponding author: xingxy@sustech.edu.cn; yupy@sustech.edu.cn; xuc@sustech.edu.cn.

Table of Contents

Supplementary Methods S2
Supplementary Discussion S2
1. Qualitative correlation of local polarizability on enantio-selection S2
1.1 Preparation of Ru-catalysts S2
1.2 Preparation of ketones S5
1.3 General procedure for the asymmetric transfer hydrogenation of ketones and determination of the absolute stereochemistry of alcohol products S18
2. Linear free energy relationship (LFER) studies of substrate local polarizability on enantio-selection S161
2.1 System 1: Noyori-type Ru-catalyzed transfer hydrogenation of di-aryl ketones S161
2.2 System 2: Noyori-Ikariya Ru-catalyzed transfer hydrogenation of ketones S167
2.3 System 3: Oxazaborolidines-catalyzed reductions of ketones with Corey-Bakshi-Shibata (CBS) catalyst S168
2.4 System 4: Sharpless asymmetric dihydroxylation (SAD) of alkenes with a cinchona alkaloidderived ligand S186
3. Computational detail S215
3.1 Procedure for the calculation of local polarizabilities. S215
3.2 Analysis of key transition states. S216
3.3 Additional computational results S216
3.3.1 Grimme's D4 dispersion model S216
3.3.2 Linear free energy relationship (LFER) studies with Hammett constants S217
3.3.3 Linear free energy relationship (LFER) studies with whole ring polarizability and electrostatic potential S219
3.3.4 Density functional theory (DFT) calculations S220
Supplementary References S222
NMR Spectra S223

Supplementary Methods

All reactions were carried out under argon atmosphere with dry solvents, unless otherwise noted. All the chemicals were purchased commercially, and used without further purification. Anhydrous tetrahydrofuran (THF), toluene (PhMe) and hexane were distilled from sodium-benzophenone. Dichloromethane (CH_2Cl_2) and trimethylamine (Et_3N) were distilled from calcium hydride. Extra dry isopropanol (^{i}PrOH) and acetonitrile (MeCN) were purchased from J&K Scientific. Thin-layer chromatography (TLC) was conducted with 0.25 mm Tsingdao silica gel plates (60F-254) and visualized by exposure to UV light (254 nm). Flash column chromatography was performed using Tsingdao silica gel (60, particle size 0.040–0.063 mm). Reagents were purchased at the highest commercial quality and used without further purification, unless otherwise stated. ^{1}H NMR (400 MHz and 600 MHz) and ^{13}C NMR (101 MHz and 151 MHz) spectra were recorded on a Bruker Avance NEO 400 MHz or Avance NEO 600 MHz spectrometer, and were reported in terms of chemical shift relative to residual $CDCl_3$ (δ 7.26 and δ 77.0 ppm, respectively) or MeOD (δ 4.87 and δ 49.0 ppm, respectively). Data for ^{1}H NMR spectra are reported as follows: chemical shift (δ ppm) (multiplicity, coupling constant (Hz), integration). Abbreviations are used as follows: s = singlet, bs = broad singlet, d = doublet, t = triplet, q = quartet, m = multiplet. Data for ^{13}C NMR spectra are reported in terms of chemical shift. High-resolution mass spectra (HRMS) data was obtained by using Thermo Scientific™ Q Exactive™ Quadrupole-Orbitrap Mass Spectrometer. X-ray analysis was conducted on Bruker D8 VENTURE. Electronic circular dichroism (ECD) analysis was conducted on Chirascan Spectrophotometer. HPLC analysis was conducted on Shimadzu LC-20A or Agilent 1260 instrument. Enantio-pure alcohol products (ee = 100%) for crystal growing were further purified by Waters 2545 Binary Gradient Module equipping with CHIRALPAK® AD-H or OD-H, Waters 2489 UV/Visible Detector, Waters Fraction Collector Ⅲ.

Supplementary Discussion

1. Qualitative correlation of local polarizability on enantio-selection

1.1 Preparation of Ru-catalysts

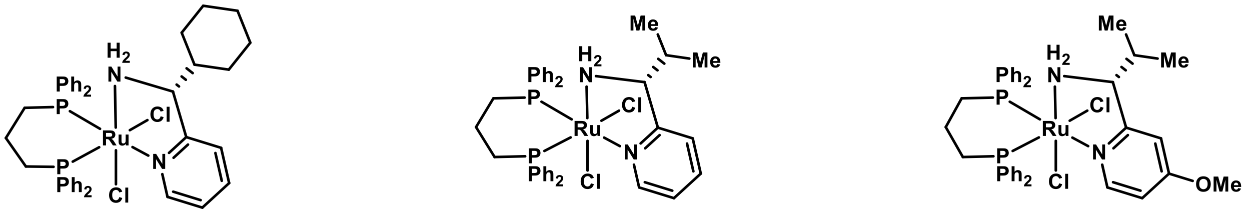

B B1 B2

The Ru-catalysts used in this work were developed by us recently, in which **B1** and **B2** have been reported previously[1,2]. Catalyst **B** was synthesized in this work according to the known procedure.

Characterization of catalyst **B**:

^{1}H NMR (400 MHz, $CDCl_3$): δ 9.01 (d, *J* = 5.6 Hz, 1H), 8.02 (t, *J* = 8.6 Hz, 2H), 7.72 (t, *J* = 7.5 Hz, 2H), 7.66 – 7.57 (m, 3H), 7.42 (t, *J* = 6.8 Hz, 1H), 7.33 – 7.28 (m, 1H), 7.26 – 7.22 (m, 3H), 7.20 – 7.14 (m, 1H), 7.11 – 6.99 (m, 3H), 6.96 – 6.85 (m, 5H), 6.78 (t, *J* = 8.6 Hz, 2H), 6.72 (t, *J* = 6.8 Hz, 1H), 4.23 (t, *J* = 11.2 Hz, 1H), 3.41 (t, *J* = 14.8 Hz, 1H), 3.14 (d, *J* = 12.4 Hz, 1H), 2.64 (t, *J* = 14.8 Hz, 1H), 2.54 – 2.44 (m, 1H), 2.32 – 2.16 (m, 1H), 1.91 – 1.70 (m, 7H), 1.46 (d, *J* = 12.4 Hz, 1H), 1.30 – 1.14 (m, 2H), 1.13 – 1.02 (m, 2H), 0.95 – 0.87 (m, 1H), 0.85 – 0.69 (m, 1H) ppm

^{13}C NMR (101 MHz, $CDCl_3$): δ 158.4, 151.1, 138.0 (d, *J* = 36.6 Hz), 137.1 (d, *J* = 36.8 Hz), 136.8 (d, *J* = 41.2 Hz), 135.9 (d, *J* = 42.9 Hz), 134.6 (d, *J* = 9.3 Hz), 133.1 (d, *J* = 9.9 Hz), 130.4 (d, *J* = 7.6 Hz), 129.6 (d, *J* = 75.0 Hz), 128.6 (d, *J* = 8.3 Hz), 128.6 (d, *J* = 81.8 Hz), 128.3 (d, *J* = 86.6 Hz), 128.0 (d, *J* = 8.7 Hz), 127.3 (d, *J* = 9.4 Hz), 127.0 (d, *J* = 8.7 Hz), 125.3, 123.2, 120.0, 77.2, 66.6, 39.5, 30.0, 29.3 (d, *J* = 28.2 Hz), 26.6, 26.4 (d, *J* = 23.5 Hz), 25.6 (d, *J* = 29.6 Hz), 21.4, 20.5 ppm

^{31}P NMR (162 MHz, $CDCl_3$): δ 52.37 (d, *J* = 46.2 Hz), 36.54 (d, *J* = 46.2 Hz) ppm

HRMS (ESI+): calculated for $C_{39}H_{44}N_2P_2ClRu$ $[M-Cl]^-$: 739.1706, found 739.1706.

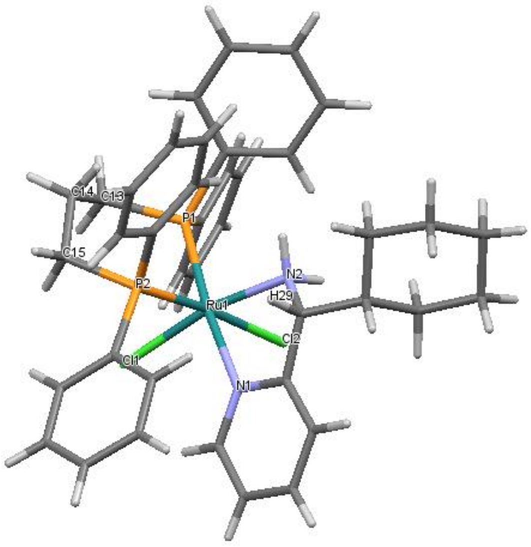

Supplementary Figure 1. The single crystal configuration of catalyst **B** is demonstrated in a capped sticks mode.

Supplementary Table 1. Crystal data and structure refinement for the catalyst **B**

Identification code	2017208
Empirical formula	$C_{39}H_{44}Cl_2N_2P_2Ru$
Formula weight	774.67
Temperature/K	100
Crystal system	tetragonal
Space group	$P4_12_12$
a/Å	12.7372(6)
b/Å	12.7372(6)
c/Å	45.259(2)
α/°	90
β/°	90
γ/°	90
Volume/Å^3	7342.7(8)
Z	8
ρ_{calc}g/cm^3	1.402
μ/mm^{-1}	0.690
F(000)	3200.0
Crystal size/mm^3	0.38 × 0.37 × 0.32
Radiation	MoKα (λ = 0.71073)
2Θ range for data collection/°	4.186 to 56.748
Index ranges	$-17 \le h \le 16$, $-16 \le k \le 17$, $-60 \le l \le 60$
Reflections collected	135812
Independent reflections	9150 [R_{int} = 0.0698, R_{sigma} = 0.0272]
Data/restraints/parameters	9150/0/415
Goodness-of-fit on F^2	1.205
Final R indexes [I>=2σ (I)]	R_1 = 0.0426, wR_2 = 0.0800
Final R indexes [all data]	R_1 = 0.0464, wR_2 = 0.0812
Largest diff. peak/hole / e Å^{-3}	0.53/-0.71
Flack parameter	-0.015(9)

1.2 Preparation of ketones

Supplementary Table 2. Ketone substrates for Ru-catalyzed asymmetric transfer hydrogenation

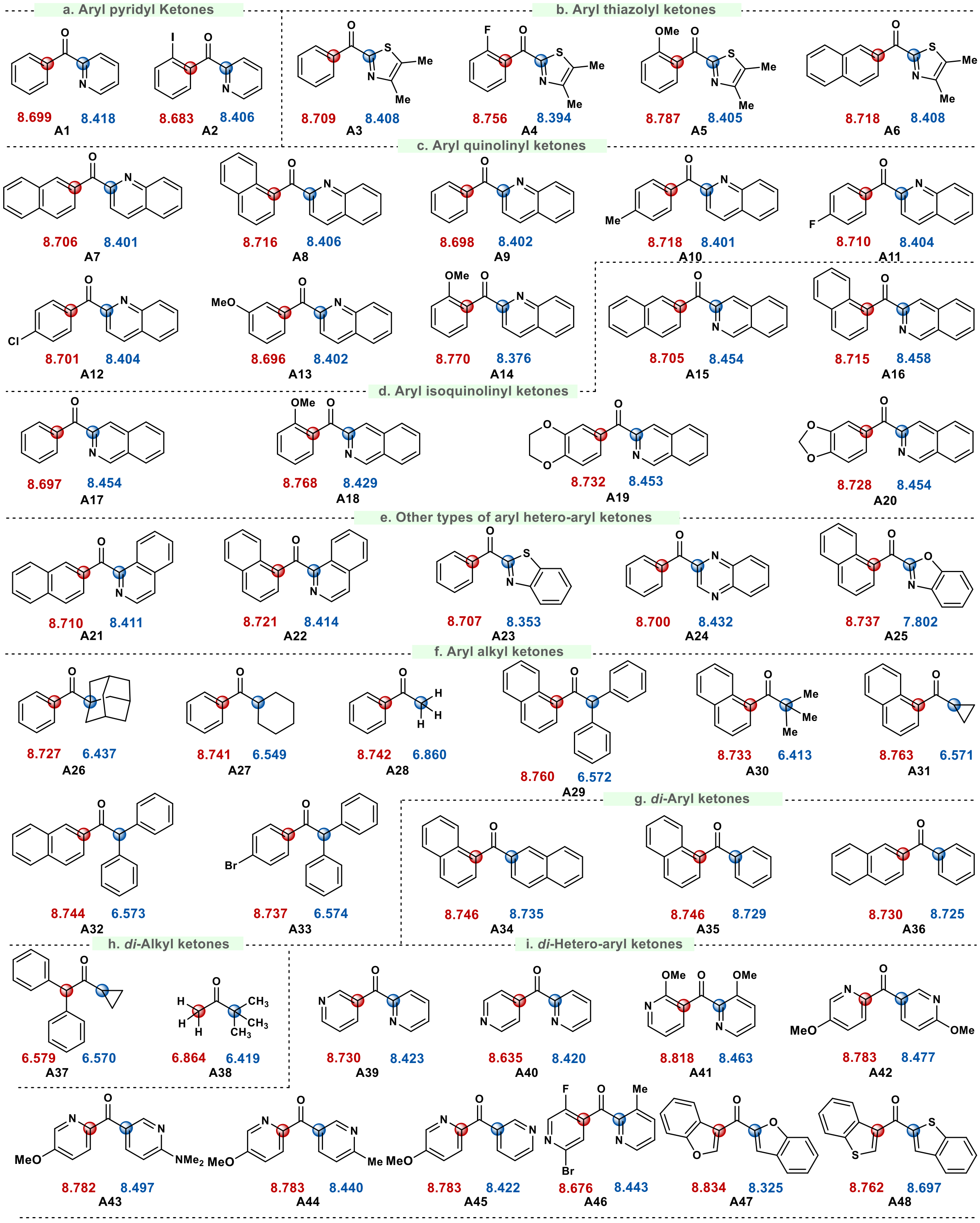

The red and blue numbers underneath each ketone substrates respectively denote the local polarizability values of the corresponding red and blue carbon atoms. Ketones **A1**, **A27**, **A28**, **A35**, **A36** and **A38** were commercially available. Ketones **A2, A39-41** and **A46-48** have been reported previously[1,2]. Other ketones were unknown and synthesized.

Preparation of ketones **A3-A6**, **A23** and **A25**

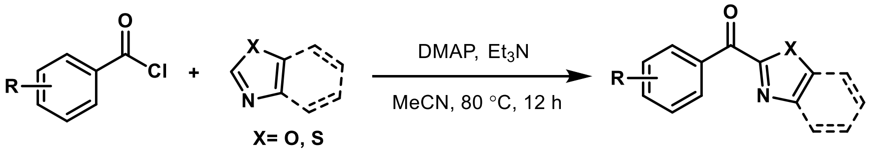

DMAP (1.5 mmol), aryl chloride (10 mmol) and azole (5 mmol) were added in a sealable tube. The tube was sealed and flushed with a stream of dry argon. MeCN (10 mL) and Et_3N ((15 mmo) were added sequentially. The mixture was stirred at 80 °C for 24 hours, and then cooled to room temperature, diluted with saturated $NaHCO_3$ solution, and extracted with ethyl acetate (3 × 50 mL). The combined organic layers were washed with brine, dried over Na_2SO_4 and concentrated under reduced pressure. The crude ketone product was purified by flash chromatography with petroleum ether and ethyl acetate.

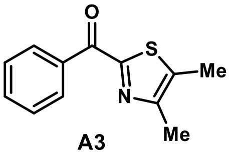

A3

^{1}H NMR (600 MHz, $CDCl_3$): δ 8.43 (d, *J* = 7.7 Hz, 2H), 7.60 (t, *J* = 7.4 Hz, 1H), 7.50 (t, *J* = 7.7 Hz, 2H), 2.47 (s, 3H), 2.45 (s, 3H) ppm
^{13}C NMR (151 MHz, $CDCl_3$): δ 184.0, 162.5, 151.7, 135.8, 135.5, 133.2, 131.0, 128.2, 15.1, 11.9 ppm
HRMS (ESI+): calculated for $C_{12}H_{12}NOS$ $[M+H]^+$: 218.0634, found 218.0634.

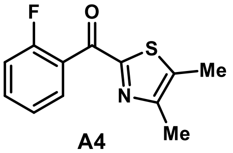

A4

^{1}H NMR (600 MHz, $CDCl_3$): δ 7.87 – 7.84 (m, 1H), 7.55 – 7.50 (m, 1H), 7.25 (t, *J* = 8.0 Hz, 1H), 7.19 – 7.13 (m, 1H), 2.48 (s, 3H), 2.40 (s, 3H) ppm
^{13}C NMR (151 MHz, $CDCl_3$): δ 183.8, 161.5, 160.8 (d, *J* = 256.0 Hz), 152.1, 136.7, 133.6 (d, *J* = 8.7 Hz), 131.6, 125.3 (d, *J* = 12.6 Hz), 123.8 (d, *J* = 3.8 Hz), 116.4 (d, *J* = 21.7 Hz), 15.0, 12.1 ppm
^{19}F NMR (565 MHz, $CDCl_3$): δ -111.30 (s) ppm
HRMS (ESI+): calculated for $C_{12}H_{11}FNOS$ $[M+H]^+$: 236.0540, found 236.0538.

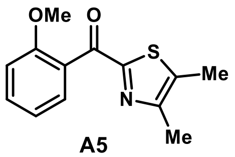

A5

^{1}H NMR (600 MHz, CDCl$_3$): δ 7.62 (dd, *J* = 7.6 Hz, 1H), 77.47 (t, *J* = 8.4 Hz, 1H), 7.03 (t, *J* = 7.6 Hz, 1H), 7.00 (d, *J* = 8.4 Hz, 1H), 3.80 (s, 3H), 2.45 (s, 3H), 2.37 (s, 3H) ppm
^{13}C NMR (151 MHz, CDCl$_3$): δ 186.4, 162.4, 158.3, 151.7, 135.8, 132.7, 130.8, 126.8, 120.0, 111.9, 55.8, 15.0, 12.0 pp
HRMS (ESI+): calculated for $C_{13}H_{14}NO_2S$ [M+H]$^+$: 248.0740, found 248.0738.

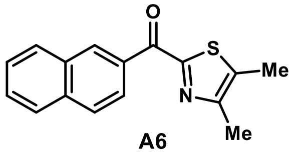

A6

^{1}H NMR (600 MHz, CDCl$_3$): δ 9.18 (s, 1H), 8.37 (d, *J* = 8.6 Hz, 1H), 8.02 (d, *J* = 8.1 Hz, 1H), 7.92 (d, *J* = 8.6 Hz, 1H), 7.88 (d, *J* = 8.1 Hz, 1H), 7.60 (t, *J* = 7.4 Hz, 1H), 7.55 (t, *J* = 7.4 Hz, 1H), 2.49 (s, 3H), 2.48 (s, 3H) ppm
^{13}C NMR (151 MHz, CDCl$_3$): δ 183.7, 162.7, 151.7, 135.8, 135.6, 133.6, 132.7, 132.4, 130.0, 128.5, 128.0, 127.6, 126.4, 126.0, 15.1, 11.9 ppm
HRMS (ESI+): calculated for $C_{16}H_{14}NOS$ [M+H]$^+$: 268.0791, found 268.0789.

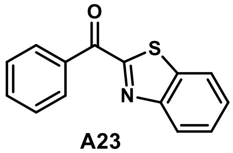

A23

^{1}H NMR (600 MHz, CDCl$_3$): δ8.57 (d, *J* = 7.4 Hz, 2H), 8.25 (d, *J* = 8.0 Hz, 1H), 8.02 (d, *J* = 8.0 Hz, 1H), 7.68 (t, *J* = 7.4 Hz, 1H), 7.61 – 7.53 (m, 4H) ppm
^{13}C NMR (151 MHz, CDCl$_3$): δ 185.3, 167.1, 153.9, 137.0, 134.9, 133.9, 131.3, 128.5, 127.6, 126.9, 125.7, 122.1 ppm
HRMS (ESI+): calculated for $C_{14}H_{10}NOS$ [M+H]$^+$: 240.0478, found 240.0476.

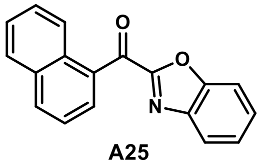

A25

^{1}H NMR (600 MHz, CDCl$_3$): δ 8.61 (d, *J* = 8.4 Hz, 1H), 8.41 (d, *J* = 7.2 Hz, 1H), 8.13 (d, *J* = 8.4 Hz, 1H), 7.93 (t, *J* = 9.0 Hz, 2H), 7.74 (d, *J* = 8.4 Hz, 1H), 7.66 – 7.61 (m, 2H), 7.59 – 7.55 (m, 2H), 7.47 (t, *J* = 7.8 Hz, 1H) ppm
^{13}C NMR (151 MHz, CDCl$_3$): δ 182.9, 158.2, 150.8, 140.8, 134.2, 133.9, 132.3, 132.1, 131.0, 128.7, 128.5, 128.4, 126.7, 125.7, 125.2, 124.2, 122.5, 111.9 ppm
HRMS (ESI+): calculated for $C_{18}H_{12}NO_2$ [M+H]$^+$: 274.0863, found 274.0861.

Preparation of ketones **A7-A14**

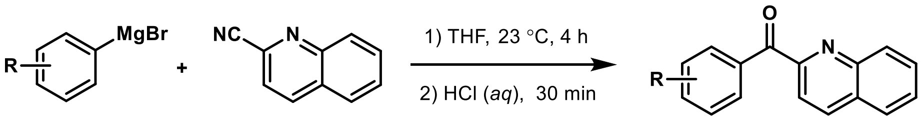

A solution of freshly prepared aryl magnesium bromide (6 mmol) was slowly added into the solution of the 2-cyanoquinoline (5 mmol) in THF (8 mL) at 0 °C. The reaction mixture was stirred at 23 °C for 4 hours, which was quenched by saturated NH_4Cl. The organic layer was separated and the aqueous layer was extracted twice with CH_2Cl_2. After removing of the solvent by high vacuum, the crude imine product was dissolved in Et_2O (40 mL). 6 M HCl (*aq)* (5 mL) was then added to promote hydrolysis of the imine to the corresponding ketone. After 30 minutes, the organic layer was separated. The aqueous layer was basified with saturated $NaHCO_3$ and extracted with CH_2Cl_2 (3 × 50 mL). The combined organic layers were dried over Na_2SO_4 and concentrated under reduce pressure. The crude ketone product was purified by flash chromatography with petroleum ether and ethyl acetate.

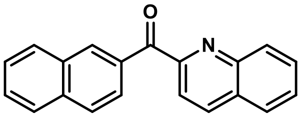

A7

^{1}H NMR (600 MHz, $CDCl_3$): δ 8.82 (s, 1H), 8.39 (d, *J* = 8.4 Hz, 1H), 8.28 (d, *J* = 8.4 Hz, 1H), 8.23 (d, *J* = 8.4 Hz, 1H), 8.16 (d, *J* = 8.4 Hz, 1H), 7.99 – 7.90 (m, 4H), 7.81 (t, *J* = 7.6 Hz, 1H), 7.69 (t, *J* = 7.6 Hz, 1H), 7.62 (t, *J* = 7.4 Hz, 1H), 7.55 (t, *J* = 7.4 Hz, 1H) ppm
^{13}C NMR (151 MHz, $CDCl_3$): δ 193.7, 155.0, 146.8, 137.1, 135.6, 134.1, 133.4, 132.4, 130.6, 130.1, 129.9, 128.9, 128.5, 128.4, 127.9, 127.7, 127.7, 126.5, 126.4, 120.9 ppm
HRMS (ESI+): calculated for $C_{20}H_{14}NO$ $[M+H]^+$: 284.1070, found 284.1068.

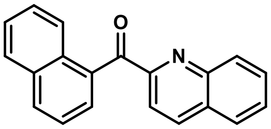

A8

^{1}H NMR (600 MHz, $CDCl_3$): δ 8.44 (d, *J* = 8.2 Hz, 1H), 8.37 (d, *J* = 8.4 Hz, 1H), 8.24 (d, *J* = 8.4 Hz, 1H), 8.10 (d, *J* = 8.4 Hz, 1H), 8.07 (d, *J* = 8.2 Hz, 1H), 7.94 (d, *J* = 8.4 Hz, 1H), 7.92 (d, *J* = 8.2 Hz, 1H), 7.87 (d, *J* = 7.0 Hz, 1H), 7.74 (t, *J* = 7.4 Hz, 1H), 7.65 (t, *J* = 7.4 Hz, 1H), 7.62 – 7.47 (m, 3H) ppm
^{13}C NMR (151 MHz, $CDCl_3$): δ 196.6, 155.3, 147.1, 137.0, 134.3, 133.9, 132.5, 131.6, 131.3, 130.8, 130.0, 129.1, 128.5, 128.4, 127.6, 126.3, 125.9, 124.1, 120.7 ppm
HRMS (ESI+): calculated for $C_{20}H_{14}NO$ $[M+H]^+$: 284.1070, found 284.1068.

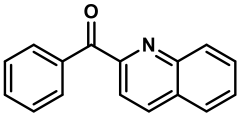

A9

^{1}H NMR (600 MHz, CDCl$_3$): δ 8.35 (d, *J* = 8.5 Hz, 1H), 8.24 (d, *J* = 8.2 Hz, 2H), 8.21 (d, *J* = 8.5 Hz, 1H), 8.11 (d, *J* = 8.5 Hz, 1H), 7.91 (d, *J* = 8.2 Hz, 1H), 7.79 (t, *J* = 7.6 Hz, 1H), 7.66 (t, *J* = 7.4 Hz, 1H), 7.63 (t, *J* = 7.4 Hz, 1H), 7.52 (t, *J* = 7.6 Hz, 2H) ppm
^{13}C NMR (151 MHz, CDCl$_3$): δ 193.8, 154.7, 146.7, 137.1, 136.1, 133.0, 131.4, 130.5, 130.1, 128.9, 128.4, 128.1, 127.6, 120.7 ppm
HRMS (ESI+): calculated for $C_{16}H_{12}NO$ [M+H]$^+$: 234.0913, found 234.0912.

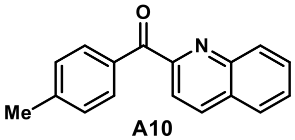

A10

^{1}H NMR (600 MHz, CDCl$_3$): δ 8.26 (d, *J* = 8.5 Hz, 1H), 8.19 (d, *J* = 8.2 Hz, 1H), 8.17 (d, *J* = 8.0 Hz, 2H), 8.06 (d, *J* = 8.5 Hz, 1H), 7.83 (d, *J* = 8.2 Hz, 1H), 7.73 (t, *J* = 7.6 Hz, 1H), 7.59 (t, *J* = 7.6 Hz, 1H), 7.29 (d, *J* = 8.0 Hz, 2H), 2.41 (s, 3H) ppm
^{13}C NMR (151 MHz, CDCl$_3$): δ 193.2, 154.8, 146.5, 143.7, 136.8, 133.4, 131.4, 130.2, 129.8, 128.7, 128.6, 128.1, 127.4, 120.6, 21.5 ppm
HRMS (ESI+): calculated for $C_{17}H_{14}NO$ [M+H]$^+$: 248.1070, found 248.1069.

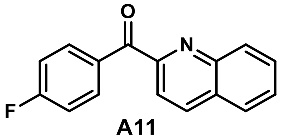

A11

^{1}H NMR (600 MHz, CDCl$_3$): δ 8.38 – 8.32 (m, 3H), 8.20 (d, *J* = 8.5 Hz, 1H), 8.12 (d, *J* = 8.5 Hz, 1H), 7.92 (d, *J* = 8.1 Hz, 1H), 7.80 (t, *J* = 7.1 Hz, 1H), 7.68 (t, *J* = 7.1 Hz, 1H), 7.19 (t, *J* = 8.7 Hz, 2H) ppm
^{13}C NMR (151 MHz, CDCl$_3$): δ 192.0, 165.8 (d, *J* = 255.1 Hz), 154.5, 146.6, 137.2, 134.2 (d, *J* = 9.5 Hz), 132.4 (d, *J* = 2.4 Hz), 130.5, 130.2, 128.9, 128.5, 127.7, 120.8, 115.3 (d, *J* = 21.8 Hz) ppm
^{19}F NMR (565 MHz, CDCl$_3$): δ -105.09 (s) ppm
HRMS (ESI+): calculated for $C_{16}H_{11}FNO$ [M+H]$^+$: 252.0819, found 252.0817.

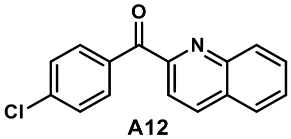

A12

^{1}H NMR (600 MHz, CDCl$_3$): δ 8.35 (d, *J* = 8.2 Hz, 1H), 8.24 (d, *J* = 7.8 Hz, 2H), 8.19 (d, *J* = 7.8 Hz, 1H), 8.13 (d, *J* = 8.2 Hz, 1H), 7.91 (d, *J* = 7.8 Hz, 1H), 7.79 (t, *J* = 6.4 Hz, 1H), 7.67 (t, *J* = 6.4 Hz, 1H), 7.49 (d, *J* = 7.8 Hz, 2H) ppm
^{13}C NMR (151 MHz, CDCl$_3$): δ 192.3, 154.2, 146.6, 139.5, 137.2, 134.5, 132.9, 130.5, 130.2, 129.0, 128.6, 128.4, 127.6, 120.7 ppm
HRMS (ESI+): calculated for $C_{16}H_{11}ClNO$ [M+H]$^+$: 268.0524, found 268.0522.

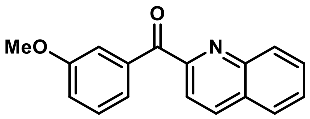

A13

^{1}H NMR (600 MHz, CDCl$_3$): δ 8.35 (d, *J* = 8.4 Hz, 1H), 8.21 (d, *J* = 8.4 Hz, 1H), 8.09 (d, *J* = 8.4 Hz, 1H), 7.91 (d, *J* = 8.4 Hz, 1H), 7.81 (d, *J* = 7.4 Hz, 1H), 7.80 (s, 1H), 7.78 (d, *J* = 8.3 Hz, 1H), 7.66 (t, *J* = 7.8 Hz, 1H), 7.42 (t, *J* = 7.8 Hz, 1H), 7.18 (d, *J* = 7.4 Hz, 1H), 3.88 (s, 3H) ppm
^{13}C NMR (151 MHz, CDCl$_3$): δ 193.5, 159.4, 154.7, 146.7, 137.3, 137.1, 130.5, 130.1, 129.1, 128.9, 128.4, 127.6, 124.4, 120.8, 119.8, 115.3, 55.5 ppm
HRMS (ESI+): calculated for $C_{17}H_{14}NO_2$ $[M+H]^+$: 264.1019, found 264.1017.

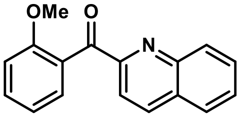

A14

^{1}H NMR (600 MHz, CDCl$_3$): δ 8.30 (d, *J* = 8.4 Hz, 1H), 8.10 (d, *J* = 8.4 Hz, 1H), 8.06 (d, *J* = 8.4 Hz, 1H), 7.87 (d, *J* = 7.8 Hz, 1H), 7.72 (t, *J* = 7.8 Hz, 1H), 7.66 (d, *J* = 7.8 Hz, 1H), 7.61 (t, *J* = 7.5 Hz, 1H), 7.53 (t, *J* = 7.8 Hz, 1H), 7.10 (t, *J* = 7.5 Hz, 1H), 7.01 (d, *J* = 8.4 Hz, 1H), 3.61 (s, 3H) ppm
^{13}C NMR (151 MHz, CDCl$_3$): δ 196.2, 158.85 (s), 155.2, 147.2, 136.7, 132.9, 131.0, 130.6, 129.7, 129.0, 128.10 (d, *J* = 3.6 Hz), 127.5, 120.5, 119.7, 111.9, 55.8 ppm
HRMS (ESI+): calculated for $C_{17}H_{14}NO_2$ $[M+H]^+$: 264.1019, found 264.1017.

Preparation of ketones **A15-A20**

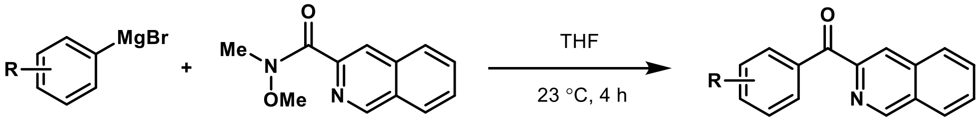

A solution of freshly prepared aryl magnesium bromide (6 mmol) was slowly added into the solution of the N-methoxy-N-methylisoquinoline-3-carboxyamide[6] (5 mmol) in THF (8 mL) at 0 °C. The reaction mixture was stirred at 23 °C for 4 hours, which was quenched by saturated NH_4Cl and extracted with ethyl acetate (3 × 50 mL). The combined organic layers were dried over Na_2SO_4 and concentrated under reduce pressure. The crude ketone product was purified by flash chromatography with petroleum ether and ethyl acetate.

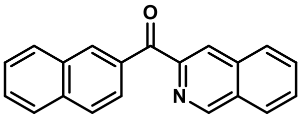

A15

^{1}H NMR (600 MHz, CDCl$_3$): δ 9.38 (s, 1H), 8.66 (s, 1H), 8.52 (s, 1H), 8.16 (d, *J* = 8.4 Hz, 1H), 8.09 (d, *J* = 8.0 Hz, 1H), 8.01 (d, *J* = 8.0 Hz, 1H), 7.95 (d, *J* = 8.4 Hz, 2H), 7.90 (d, *J* = 8.4 Hz, 1H), 7.80 (t, *J* = 7.4 Hz, 1H), 7.76 (t, *J* = 7.4 Hz, 1H), 7.60 (t, *J* = 7.4 Hz, 1H), 7.54 (t, *J* = 7.4 Hz, 1H) ppm

^{13}C NMR (151 MHz, CDCl$_3$): δ 194.3, 151.7, 149.2, 135.7, 135.44, 134.3, 133.1, 132.4, 131.1, 129.7, 129.6, 129.5, 128.3, 128.2, 128.0, 127.7, 127.7, 126.5, 126.3, 123.5 ppm
HRMS (ESI+): calculated for $C_{20}H_{14}NO$ [M+H]$^+$: 284.1070, found 284.1068.

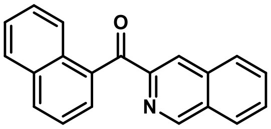

A16

^{1}H NMR (600 MHz, CDCl$_3$): δ 9.31 (s, 1H), 8.52 (s, 1H), 8.20 (d, *J* = 8.2 Hz, 1H), 8.06 (d, *J* = 8.0 Hz, 1H), 8.04 (d, *J* = 8.2 Hz, 1H), 7.99 (d, *J* = 8.0 Hz, 1H), 7.93 (d, *J* = 7.5 Hz, 1H), 7.82 – 7.73 (m, 3H), 7.58 – 7.48 (m, 3H) ppm
^{13}C NMR (151 MHz, CDCl$_3$): δ 196.9, 152.4, 149.1, 135.7, 135.5, 133.8, 131.8, 131.2, 131.1, 129.8, 129.7, 129.1, 128.4, 128.4, 127.7, 127.3, 126.3, 125.7, 124.3, 124.0 ppm
HRMS (ESI+): calculated for $C_{20}H_{14}NO$ [M+H]$^+$: 284.1070, found 284.1069.

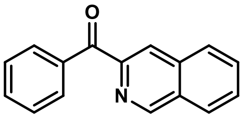

A17

^{1}H NMR (600 MHz, CDCl$_3$): δ 9.34 (s, 1H), 8.48 (s, 1H), 8.09 – 8.05 (m, 3H), 8.00 (dd, *J* = 13.2, 7.2 Hz, 1H), 7.83 – 7.72 (m, 2H), 7.60 (dd, *J* = 13.2, 7.2 Hz, 1H), 7.56 – 7.47 (m, 2H) ppm
^{13}C NMR (151 MHz, CDCl$_3$): δ 194.4, 151.6, 148.9, 137.0, 135.6, 132.7, 131.1, 130.9, 129.6, 129.4, 128.2, 128.1, 127.6, 123.5 ppm
HRMS (ESI+): calculated for $C_{16}H_{12}NO$ [M+H]$^+$: 234.0913, found 234.0912.

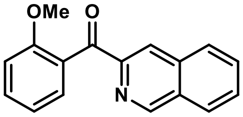

A18

^{1}H NMR (600 MHz, CDCl$_3$): δ 9.27 (s, 1H), 8.41 (s, 1H), 8.03 (d, *J* = 8.4Hz, 1H), 7.98 (d, *J* = 7.8 Hz, 1H), 7.77 (t, *J* = 7.4 Hz, 1H), 7.73 (t, *J* = 7.4 Hz, 1H), 7.56 (d, *J* = 7.8 Hz, 1H), 7.51 (t, *J* = 7.4 Hz, 1H), 7.09 (t, *J* = 7.4 Hz, 1H), 7.01 (d, *J* = 8.4 Hz, 1H), 3.66 (s, 3H) ppm
^{13}C NMR (151 MHz, CDCl$_3$): δ 196.2, 158.1, 151.9, 149.2, 135.5, 132.5, 130.8, 130.2, 129.7, 129.2, 128.8, 128.3, 127.6, 122.2, 120.6, 111.6, 55.6 ppm
HRMS (ESI+): calculated for $C_{17}H_{14}NO_2$ [M+H]$^+$: 264.1019, found 264.1017.

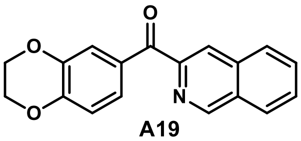

A19

^{1}H NMR (600 MHz, CDCl$_3$): δ 9.32 (s, 1H), 8.42 (s, 1H), 8.06 (d, *J* = 8.0 Hz, 1H), 7.99 (d, *J* = 8.0 Hz, 1H), 7.79 (t, *J* = 7.4 Hz, 1H), 7.74 (t, *J* = 7.4 Hz, 1H), 7.71 (s, 1H), 7.68 (d, *J* = 8.4 Hz, 1H), 6.96 (d, *J* = 8.4 Hz, 1H), 4.33 (s, 2H), 4.30 (s, 2H) ppm

^{13}C NMR (151 MHz, CDCl$_3$): δ 192.6, 151.4, 149.3, 147.9, 142.9, 135.7, 131.0, 130.4, 129.4, 129.2, 128.1, 127.6, 125.3, 123.1, 120.7, 117.0, 64.7, 64.1 ppm
HRMS (ESI+): calculated for $C_{18}H_{14}NO_3$ [M+H]$^+$: 292.0968, found 292.0966.

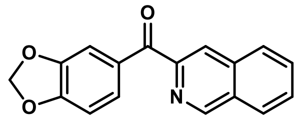

A20

^{1}H NMR (600 MHz, CDCl$_3$): δ 9.32 (s, 1H), 8.42 (s, 1H), 8.07 (d, *J* = 8.0 Hz, 1H), 7.99 (d, *J* = 8.2 Hz, 1H), 7.79 – 7.71 (m, 3H), 7.63 (s, 1H), 6.90 (d, *J* = 8.2 Hz, 1H), 6.06 (s, 2H) ppm
^{13}C NMR (151 MHz, CDCl$_3$): δ 192.3, 151.6, 151.4, 149.3, 147.6, 135.7, 131.3, 131.0, 129.40, 129.3, 128.1, 127.8, 127.6, 123.2, 110.8, 107.8, 101.7 ppm
HRMS (ESI+): calculated for $C_{17}H_{12}NO_3$ [M+H]$^+$: 278.0812, found 278.0811.

Preparation of ketones **A21-A22**

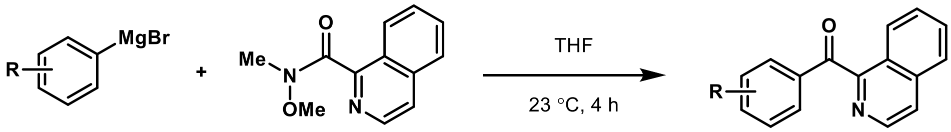

A solution of freshly prepared aryl magnesium bromide (6 mmol) was slowly added into the solution of the N-methoxy-N-methylisoquinoline-1-carboxyamide[6] (5 mmol) in THF (8 mL) at 0 °C. The reaction mixture was stirred at 23 °C for 4 hours, which was quenched by saturated NH_4Cl and extracted with ethyl acetate (3 × 50 mL). The combined organic layers were dried over Na_2SO_4 and concentrated under reduce pressure. The crude ketone product was purified by flash chromatography with petroleum ether and ethyl acetate.

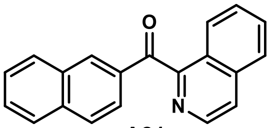

A21

^{1}H NMR (600 MHz, CDCl$_3$): δ 8.65 (d, *J* = 5.6 Hz, 1H), 8.36 (s, 1H), 8.25 (d, *J* = 8.4 Hz, 1H), 8.16 (d, *J* = 8.4 Hz, 1H), 7.95 (d, *J* = 8.4 Hz, 2H), 7.89 (d, *J* = 8.4 Hz, 1H), 7.85 (d, *J* = 6.5 Hz, 2H), 7.76 (t, *J* = 7.5 Hz, 1H), 7.64 – 7.59 (m, 2H), 7.51 (t, *J* = 7.5 Hz, 1H) ppm
^{13}C NMR (151 MHz, CDCl$_3$): δ 194.7, 156.7, 141.2, 136.7, 135.9, 133.9, 133.7, 132.3, 130.7, 129.8, 128.8, 128.4, 128.3, 127.8, 127.1, 126.7, 126.5, 126.2, 125.2, 122.6 ppm
HRMS (ESI+): calculated for $C_{20}H_{14}NO$ [M+H]$^+$: 284.1070, found 284.1068.

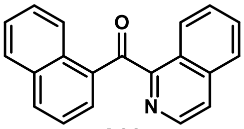

A22

^{1}H NMR (400 MHz, $CDCl_3$): δ 8.86 (d, *J* = 8.4 Hz, 1H), 8.57 (d, *J* = 5.6 Hz, 1H), 8.43 (d, *J* = 8.4 Hz, 1H), 8.05 (d, *J* = 8.4 Hz, 1H), 7.95 (d, *J* = 5.2 Hz, 1H), 7.93 (d, *J* = 4.4 Hz, 1H), 7.81 (d, *J* = 5.6 Hz, 1H), 7.77 (t, *J* = 7.6 Hz, 1H), 7.68 – 7.56 (m, 4H), 7.43 (t, *J* = 7.6 Hz, 1H) ppm
^{13}C NMR (101 MHz, $CDCl_3$): δ 197.3, 157.5, 141.4, 136.7, 134.5, 134.0, 133.8, 132.6, 2131.4, 130.7, 128.5, 128.3, 127.1, 126.6, 126.5, 126.3, 126.0, 124.2, 122.8 ppm
HRMS (ESI+): calculated for $C_{20}H_{14}NO$ $[M+H]^+$: 284.1070, found 284.1067.

Preparation of ketone **A24**

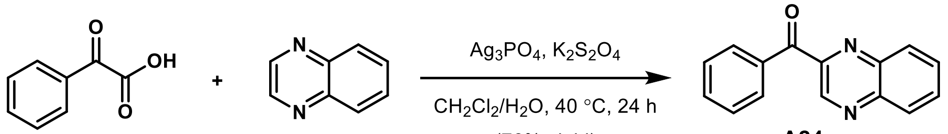

The quinoxaline (5 mmol, 650 mg), benzoylformic acid (10 mmol, 1.50 g), $K_2S_2O_8$ (10 mmol, 2.06 g), Ag_3PO_4 (10 mol%, 415 mg), 30mL of CH_2Cl_2 and 12 mL of H_2O was added in a dry reaction tube. The mixture was stirred at 40 °C for 24 hours and then cooled to ambient temperature, which was quenched by saturated NH_4Cl and extracted with CH_2Cl_2 (3 × 50 mL). The combined organic layers were dried over Na_2SO_4 and concentrated under reduce pressure. The crude mixture was purified by flash chromatography with petroleum ether and ethyl acetate (5/1) to provide **A24** as yellow solid (893 mg, 3.82 mmol, 76% yield).

^{1}H NMR (600 MHz, $CDCl_3$): δ 9.49 (s, 1H), 8.24 (d, *J* = 8.2 Hz, 2H), 8.20 (d, *J* = 8.2 Hz, 2H), 7.90 (t, *J* = 7.6 Hz, 1H), 7.86 (t, *J* = 7.6 Hz, 1H), 7.66 (t, *J* = 7.4 Hz, 1H), 7.54 (t, *J* = 7.4 Hz, 2H) ppm
^{13}C NMR (151 MHz, $CDCl_3$): δ 192.3, 148.6, 145.3, 143.1, 140.4, 135.5, 133.6, 132.0, 131.2, 130.8, 130.4, 129.4, 128.4 ppm
HRMS (ESI+): calculated for $C_{15}H_{11}N_2O$ $[M+H]^+$: 235.0866, found 235.0865.

Preparation of ketones **A26**, **A30**, **A31** and **A34**

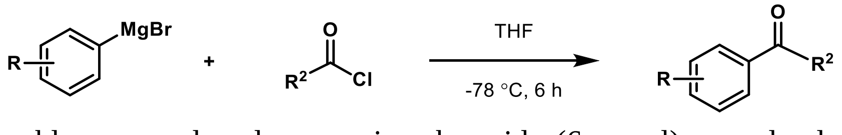

A solution of freshly prepared aryl magnesium bromide (6 mmol) was slowly added into the solution of acid chloride compound (5 mmol) in THF (10 mL) at -78 °C. The reaction mixture was stirred at -78 °C for 6 hours, which was quenched by saturated NH_4Cl, and then warmed to ambient temperature and extracted with CH_2Cl_2 (3 × 50 mL). The combined organic layers were dried over Na_2SO_4 and concentrated under reduce pressure. The crude ketone product was purified by flash chromatography with petroleum ether and ethyl acetate.

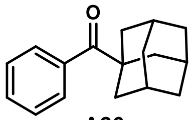

A26

^{1}H NMR (600 MHz, CDCl$_3$): δ 7.54 (d, *J* = 7.4 Hz, 2H), 7.43 (t, *J* = 7.4 Hz, 1H), 7.38 (t, *J* = 7.4 Hz, 2H), 2.09 – 2.05 (m, 3H), 2.03 – 1.99 (m, 6H), 1,79 – 1.70 (m, 6H) ppm
^{13}C NMR (151 MHz, CDCl$_3$): δ 210.1, 139.6, 130.1, 127.9, 127.1, 46.9, 39.0, 36.5, 28.1 ppm
HRMS (ESI+): calculated for $C_{17}H_{21}O$ [M+H]$^+$: 241.1587, found 241.1585.

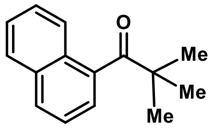

A30

^{1}H NMR (600 MHz, CDCl$_3$): δ 7.90 – 7.84 (m, 2H), 7.66 – 7.60 (m, 1H), 7.52 – 7.48 (m, 2H), 7.47 – 7.43 (m, 1H), 7.36 (d, *J* = 7.0 Hz, 1H), 1.33 (s, 9H) ppm
^{13}C NMR (151 MHz, CDCl$_3$): δ 214.5, 138.9, 133.5, 129.9, 128.9, 128.4, 126.7, 126.2, 125.4, 124.2, 122.2, 45.5, 27.2 ppm
HRMS (ESI+): calculated for $C_{15}H_{17}O$ [M+H]$^+$: 213.1274, found 213.1273.

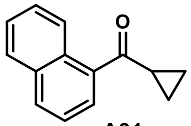

A31

^{1}H NMR (600 MHz, CDCl$_3$): δ 8.48 (d, *J* = 8.4 Hz, 1H), 7.97 (d, *J* = 8.2 Hz, 1H), 7.95 (d, *J* = 7.2 Hz, 1H), 7.89 (d, *J* = 8.2 Hz, 1H), 7.58 (t, *J* = 7.2 Hz, 1H), 7.54 – 7.51 (m, 2H), 2.62 – 2.56 (m, 1H), 1.42 – 1.38 (m, 2H), 1.15 – 1.11 (m, 2H) ppm
^{13}C NMR (151 MHz, CDCl$_3$): δ 204.7 137.8, 133.7, 131.8, 129.8, 128.3, 127.4, 127.1, 126.3, 125.7, 124.5, 21.5, 12.4 ppm
HRMS (ESI+): calculated for $C_{14}H_{13}O$ [M+H]$^+$: 197.0966, found 197.0961.

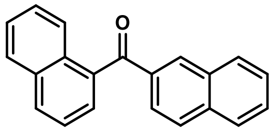

A34

^{1}H NMR (600 MHz, CDCl$_3$): δ 8.27 (s, 1H), 8.14 (d, *J* = 8.4 Hz, 1H), 8.10 (d, *J* = 8.4 Hz, 1H), 8.05 (d, *J* = 8.2 Hz, 1H), 7.96 (t, *J* = 8.2 Hz, 2H), 7.91 (d, *J* = 8.2 Hz, 1H), 7.84 (d, *J* = 8.2 Hz, 1H), 7.66 (d, *J* = 7.2 Hz, 1H), 7.61 (t, *J* = 7.6 Hz, 1H), 7.56 (t, *J* = 7.6 Hz, 2H), 7.54 – 7.50 (m, 2H) ppm
^{13}C NMR (151 MHz, CDCl$_3$): δ 198.0, 136.6, 135.7, 135.6, 133.7, 132.9, 132.3, 131.2, 131.0, 129.6, 128.6, 128.4, 128.4, 127.8, 127.7, 127.2, 126.8, 126.5, 125.7, 125.3, 124.4 ppm
HRMS (ESI+): calculated for $C_{21}H_{15}O$ [M+H]$^+$: 283.1117, found 283.1115.

Preparation of ketones **A29**, **A32**, **A33**

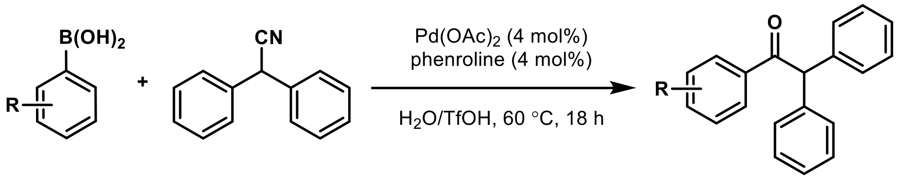

Arylboronic acid (6 mmol), nitrile (5 mmol), Pd(OAc)$_2$ (4 mol%), phenroline (4 mol%), H_2O (6 mL) and triflic acid (2 mL) were added to a 100 mL round-bottomed flask. The reaction mixture was stirred at 60 °C under air for 18 hours, which was then neutralized with saturated $NaHCO_3$ solution and extracted with ethyl acetate (3 × 50 mL). The combined organic layers were washed with brine, dried by Na_2SO_4 and concentrated. The crude ketone product was purified by flash chromatography with petroleum ether and CH_2Cl_2.

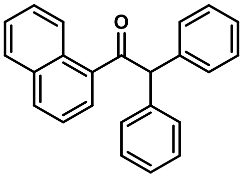

A29

^{1}H NMR (600 MHz, CDCl$_3$): δ 8.56 (d, J = 8.4 Hz, 1H), 7.95 (t, J = 9.0 Hz, 2H), 7.86 (d, J = 8.4 Hz, 1H), 7.57 (t, J = 7.6Hz, 1H), 7.53 (t, J = 7.6 Hz, 1H), 7.47 – 7.38 (m, 5H), 7.35 (t, J = 7.5 Hz, 4H), 7.29 – 7.26 (m, 2H), 6.09 (s, 1H) ppm
^{13}C NMR (151 MHz, CDCl$_3$): δ 202.0, 139.1, 136.4, 133.9, 132.7, 130.5, 129.1, 128.7, 128.4, 128.0, 127.8, 127.2, 126.4, 125.8, 124.3, 62.5 ppm
HRMS (ESI+): calculated for $C_{24}H_{19}O$ [M+H]$^+$: 323.1430, found 323.1429.

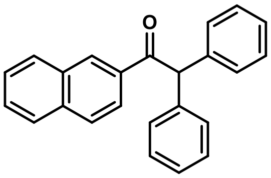

A32

^{1}H NMR (600 MHz, CDCl$_3$): δ 8.53 (s, 1H), 8.05 (d, J = 8.6 Hz, 1H), 7.87 (d, J = 8.0 Hz, 1H), 7.84 – 7.75 (m, 2H), 7.52 (dd, J = 11.0, 4.0 Hz, 1H), 7.47 (dd, J = 11.0, 4.0 Hz, 1H), 7.42 – 7.18 (m, 10H), 6.20 (s, 1H) ppm
^{13}C NMR (151 MHz, CDCl$_3$): δ 198.1, 139.1, 135.4, 134.1, 132.4, 130.6, 129.6, 129.1, 128.7, 128.5, 128.4, 127.6, 127.1, 126.7, 124.6, 59.4 ppm
HRMS (ESI+): calculated for $C_{24}H_{19}O$ [M+H]$^+$: 323.1430, found 323.1429.

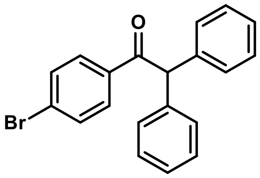

A33

¹H NMR (600 MHz, CDCl₃): δ 7.84 (d, J = 8.2 Hz, 2H), 7.51 (d, J = 8.2 Hz, 2H), 7.31 (t, J = 7.5 Hz, 4H), 7.24 (d, J = 7.8 Hz, 6H), 5.95 (s, 1H) ppm
¹³C NMR (151 MHz, CDCl₃): δ 197.1, 138.7, 135.4, 131.9, 130.4, 129.0, 128.8, 128.2, 127.2, 59.5 ppm
HRMS (ESI+): calculated for $C_{20}H_{16}BrO$ $[M+H]^+$: 351.0379, found 351.0378.

Preparation of ketone **A37**

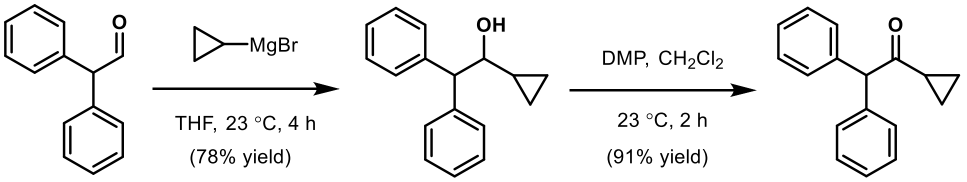

A37

A solution of cyclopropylmagnesium bromide (1.0 M, 12 mL, 12 mmol) was slowly added into the solution of the diphenylacetaldehyde (10 mmol, 1.96 g) in THF (20 mL) at 0 °C. The reaction mixture was stirred at 23 °C for 4 hours, then quenched by saturated NH_4Cl and extracted with CH_2Cl_2 (3 × 50 mL). Organic layers were washed with brine, dried over Na_2SO_4, and concentrated under reduce pressure. The mixture was purified by flash chromatography with petroleum ether and ethyl acetate (5/1) to afford pure alcohol as white solid (1.85 g, 7.8 mmol, 78% yield).

Next, the alcohol (5 mmol, 1.19 g) was added into the solution of Dess- Martin Periodinane (DPM) (7.5 mmol, 1.46 g) in CH_2Cl_2 (20 mL) at 23 °C and stirred for 2 hours. Addition of a solution of saturated Na_2CO_3 afforded white precipitates. The solid was filtered off and the resulting liquid was diluted with water, and extracted by ethyl acetate (3 × 50 mL). The combined organic layers were dried over Na_2SO_4 and concentrated under reduce pressure. The crude mixture was purified by flash chromatography with petroleum ether and ethyl acetate (10/1) afford ketone **A37** as colorless liquid (1.07 g, 4.5 mmol, 91% yield).

¹H NMR (600 MHz, CDCl₃): δ 7.35 – 7.30 (m, 4H), 7.28 – 7.22 (m, 6H), 5.27 (s, 1H), 2.01 (tt, J = 7.6, 4.5 Hz, 1H), 1.11 (dt, J = 7.6, 4.5 Hz, 2H), 0.86 (dt, J = 7.6, 4.5 Hz, 2H) ppm
¹³C NMR (151 MHz, CDCl₃): δ 208.6, 138.6, 129.2, 128.6, 127.1, 65.3, 21.1, 12.0 ppm
HRMS (ESI+): calculated for $C_{17}H_{17}O$ $[M+H]^+$: 237.1274, found 237.1273.

Preparation of ketones **A42-A45**

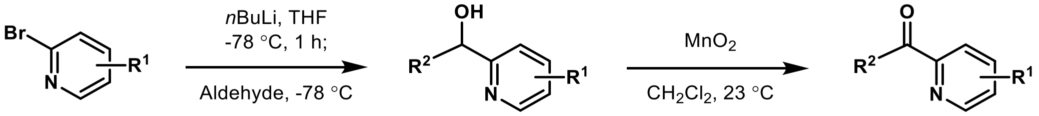

A solution of n-BuLi (2.4 M, 4.4 mL, 1.05 equiv.) was slowly added into the solution of 2-bromide pyridine compound (10 mmol) in THF (20 mL) at -78 °C under argon atmosphere. The mixture was kept at -78 °C for 1 hour, and then the corresponding aldehyde (10 mmol, 1.0 equiv.) was added. The reaction mixture was stirred about 30 minutes and warmed to room temperature and monitored by TLC. After the corresponding aldehyde was fully consumed, the resulting solution

was quenched with saturated NH_4Cl solution, and extracted with ethyl acetate (3 × 50 mL). The combined organic layers were dried over Na_2SO_4, concentrated under reduce pressure. and further purified by flash chromatography on silica gel to afford the corresponding alcohol. Next, a 100 mL of round-bottom flask charged with the corresponding alcohol and CH_2Cl_2 (20 mL) was added MnO_2 (50 mmol). The mixture was stirred at ambient temperature and monitored by TLC. After the corresponding alcohol was fully consumed, the resulted suspension was filtered through a pad of silica gel and washed with CH_2Cl_2. The solvent was removed by rotary evaporation and the residue was further purified by flash chromatography with petroleum ether and ethyl acetate on silica gel to afford the corresponding ketone.

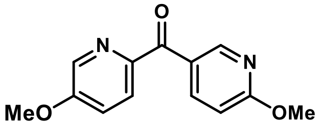

A42

^{1}H NMR (400 MHz, CDCl$_3$): δ 9.08 (d, *J* = 2.4 Hz, 1H), 8.40 – 8.30 (m, 2H), 8.14 (dd, *J* = 8.7, 3.3 Hz, 1H), 7.38 – 7.30 (m, 1H), 6.81 (dd, *J* = 8.7, 2.4 Hz, 1H), 4.01 (s, 3H), 3.94 (s, 3H) ppm
^{13}C NMR (151 MHz, CDCl$_3$): δ 189.8, 166.2, 157.9, 152.0, 147.4, 140.9, 136.5, 126.4, 126.15 (s), 120.3, 110.4, 55.8, 54.0 ppm
HRMS (ESI+): calculated for $C_{13}H_{13}N_2O_3$ $[M+H]^+$: 245.0921, found 245.0920.

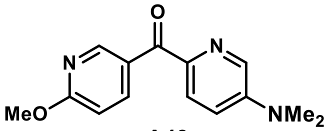

A43

^{1}H NMR (600 MHz, CDCl$_3$): δ 9.08 (s, 1H), 8.35 (d, *J* = 8.6 Hz, 1H), 8.13 (s, 1H), 8.08 (d, *J* = 8.9 Hz, 1H), 7.03 (d, *J* = 8.9 Hz, 1H), 6.80 (d, *J* = 8.6 Hz, 1H), 4.01 (s, 3H), 3.11 (s, 6H) ppm
^{13}C NMR (101 MHz, CDCl$_3$): δ 189.8, 165.8, 151.5, 147.4, 142.4, 140.9, 132.2, 127.2, 126.0, 117.2, 110.1, 53.8, 39.7 ppm
HRMS (ESI+): calculated for $C_{14}H_{16}N_3O_2$ $[M+H]^+$: 258.1237, found 258.1237.

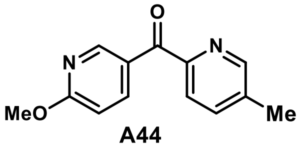

A44

^{1}H NMR (400 MHz, CDCl$_3$): δ 9.07 (d, *J* = 2.2 Hz, 1H), 8.52 (s, 1H), 8.35 (dd, *J* = 8.7, 2.2 Hz, 1H), 8.00 (d, *J* = 8.0 Hz, 1H), 7.69 (d, *J* = 8.0 Hz, 1H), 6.81 (d, *J* = 8.7 Hz, 1H), 4.01 (s, 3H), 2.44 (s, 3H) ppm
^{13}C NMR (101 MHz, CDCl$_3$): δ 190.9, 166.3, 152.3, 152.2, 148.9, 140.8, 137.5, 136.9, 126.2, 124.2, 110.5, 54.0, 18.7 ppm
HRMS (ESI+): calculated for $C_{13}H_{13}N_2O_2$ $[M+H]^+$: 229.0972, found 229.0971.

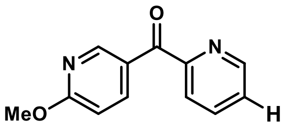

A45

^{1}H NMR (400 MHz, $CDCl_3$): δ 9.08 (s, 1H), 8.71 (d, *J* = 4.8 Hz, 1H), 8.37 (d, *J* = 8.7 Hz, 1H), 8.08 (d, *J* = 7.9 Hz, 1H), 7.91 (t, *J* = 7.7 Hz, 1H), 7.49 (dd, *J* = 7.6, 4.8 Hz, 1H), 6.83 (d, *J* = 8.7 Hz, 1H), 4.02 (s, 3H) ppm

^{13}C NMR (101 MHz, $CDCl_3$): δ 191.0, 166.4, 154.8, 152.4, 148.4, 140.8, 137.2, 126.4, 125.9, 124.5, 110.7, 54.0 ppm

HRMS (ESI+): calculated for $C_{12}H_{11}N_2O_2$ $[M+H]^+$: 215.0815, found 215.0814.

1.3 General procedure for the asymmetric transfer hydrogenation of ketones and determination of the absolute stereochemistry of alcohol products

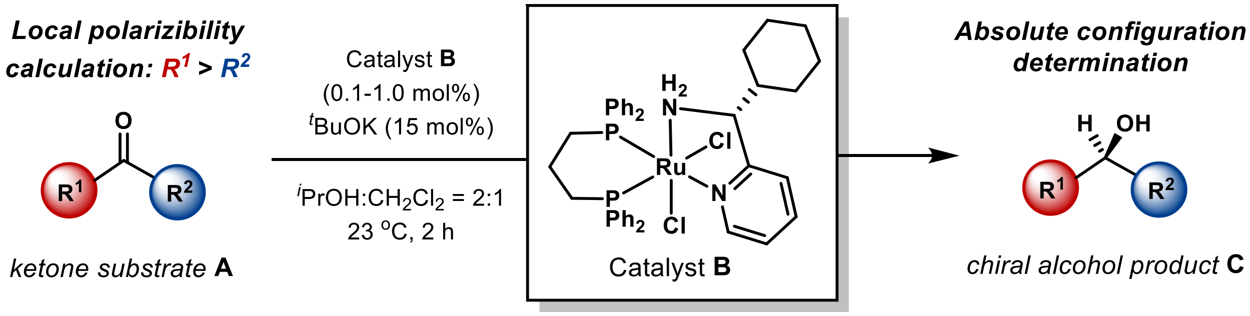

In an argon filled-glovebox, the catalyst **B** (0.2 – 2.0 μmol, 0.1- 1.0 mol%) was suspended in ^{i}PrOH (2.0 mL) in a 4-mL vial, and potassium *tert*-butanolate (30 μL, 1M in *tert*-butanol, 15 mol%) was added. After the mixture was stirred for 20 minutes, ketone **A** (0.2 mmol) dissolved in CH_2Cl_2 (1.0 mL) was added. After stirring at 23 °C for 2 hours, the reaction mixture was concentrated under reduced pressure and the crude product was purified by silica gel column chromatography with petroleum ether and ethyl acetate to give the alcohol **C.** In order to determine the absolute configuration of the major enantiomer of product **C**, its minor enantiomer was removed by preparative HPLC (OD-H or AD-H 2.0*25 cm, 5μm). Finally, the *X*-ray crystal of the predominant enantiomer of the alcohol **C** (100% ee) or its chiral auxiliary-derived compound was obtained by liquid/liquid diffusion with CH_2Cl_2/hexane or THF/hexane.

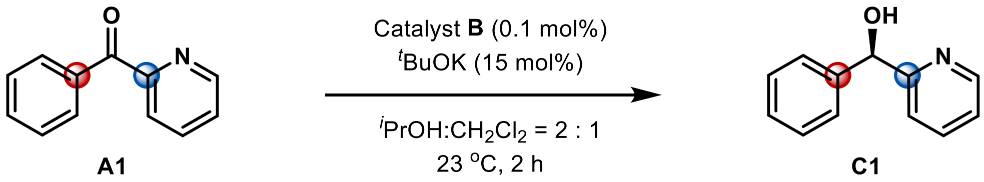

Following the general procedure, **C1** (34.0 mg, 92% yield, 90% ee) was obtained as white solid. Its major pure enantiomer was purified by chiral preparative HPLC (OD-H, 2.0*25 cm, 5μm) and the *X*-ray crystal of the optically pure enantiomer (100% ee) was obtained by liquid/liquid diffusion with CH_2Cl_2/hexane system.

^{1}H NMR (600 MHz, $CDCl_3$): δ 8.54 (d, J = 4.8 Hz, 1H), 7.59 (t, J = 7.8 Hz, 1H), 7.35 (d, J = 7.2 Hz, 2H), 7.31 (t, J = 7.2 Hz, 2H), 7.24 (d, J = 9.6 Hz, 1H), 7.17 (t, J = 5.4 Hz, 1H), 7.12 (d, J = 7.8 Hz, 1H), 5.72 (s, 1H), 5.27 (s, 1H) ppm

^{13}C NMR (151 MHz, $CDCl_3$): δ 160.8, 147.8, 143.2, 136.8, 128.6, 127.8, 127.1, 122.4, 121.3, 74.9 ppm

HRMS (ESI+): calculated for $C_{12}H_{12}NO$ $[M+H]^+$: 186.0913, found 186.0913.

HPLC (OD-H, 0.46*25 cm, 5μm, hexane / ethanol = 95/5, flow 1 mL/min, detection at 254 nm) retention time = 10.401 min (minor) and 11.261 min (major).

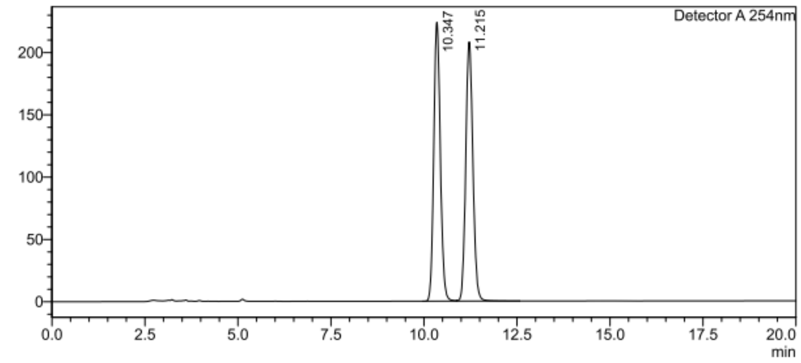

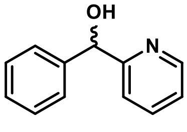

(±)-**C1**

<Peak Table>

Detector A 254nm

Peak#	Ret. Time	Area	Height	Conc.	Unit	Mark	Name
1	10.347	2755045	223774	49.880			
2	11.215	2768288	208035	50.120		V	
Total		5523334	431809				

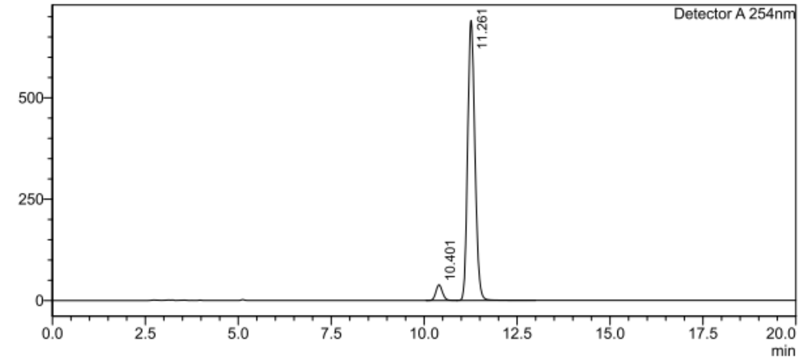

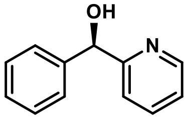

C1
92% yield, 90% ee
obtained by catalyst **B**

<Peak Table>

Detector A 254nm

Peak#	Ret. Time	Area	Height	Conc.	Unit	Mark	Name
1	10.401	476807	38601	4.840			
2	11.261	9374759	690018	95.160		V	
Total		9851565	728620				

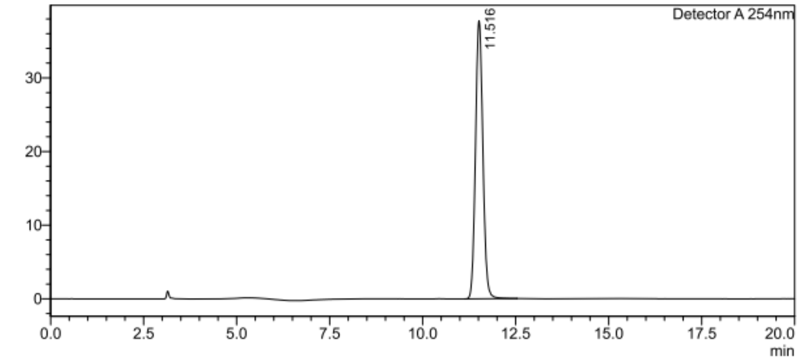

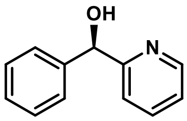

C1
> 99.9% ee

<Peak Table>

Detector A 254nm

Peak#	Ret. Time	Area	Height	Conc.	Unit	Mark	Name
1	11.516	519515	37738	100.000			
Total		519515	37738				

Supplementary Table 3. Crystal data and structure refinement for **C1**

CCDC Number	2017192
Empirical formula	$C_{12}H_{11}NO$
Formula weight	185.22
Temperature/K	200.0
Crystal system	orthorhombic
Space group	$P2_12_12_1$
a/Å	7.3874(2)
b/Å	9.8509(3)
c/Å	13.7207(5)
α/°	90
β/°	90
γ/°	90
Volume/Å^3	998.49(5)
Z	4
ρ_{calc}g/cm^3	1.232
μ/mm^{-1}	0.626
F(000)	392.0
Crystal size/mm^3	0.36 × 0.35 × 0.29
Radiation	CuKα (λ = 1.54178)
2Θ range for data collection/°	11.056 to 137.42
Index ranges	$-8 \le h \le 8$, $-9 \le k \le 11$, $-16 \le l \le 16$
Reflections collected	4925
Independent reflections	1836 [R_{int} = 0.0460, R_{sigma} = 0.0473]
Data/restraints/parameters	1836/0/129
Goodness-of-fit on F^2	1.061
Final R indexes [I>=2σ (I)]	R_1 = 0.0318, wR_2 = 0.0739
Final R indexes [all data]	R_1 = 0.0340, wR_2 = 0.0759
Largest diff. peak/hole / e Å^{-3}	0.16/-0.17
Flack parameter	-0.12(17)

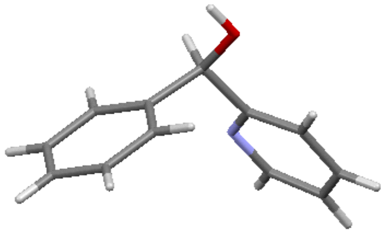

Supplementary Figure 2. The single crystal configuration of **C1** is demonstrated in a capped sticks mode.

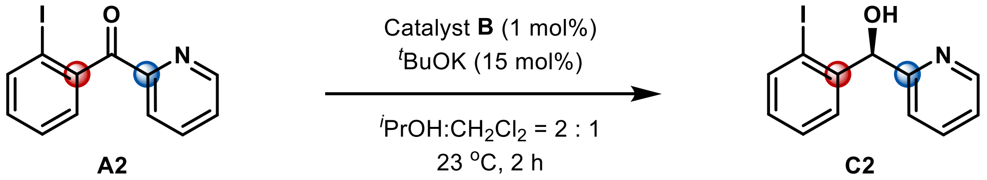

Following the general procedure, **C2** (60.3 mg, 97% yield, 95% ee) was obtained as white solid. The retention time in HPLC of the major enantiomer is consistent with that obtained by Ru-catalyst **B1,** and the *X*-ray crystal of the optically pure major enantiomer (100% ee) has been previously reported[1].

^{1}H NMR (400 MHz, CDCl$_3$): δ 8.59 (d, *J* = 4.8 Hz, 1H), 7.86 (d, *J* = 7.9 Hz, 1H), 7.62 (dd, *J* = 7.6, 6.0 Hz, 1H), 7.32 – 7.27 (m, 2H), 7.26 – 7.20 (m, 2H), 6.97 (dd, *J* = 7.9, 4.8 Hz, 1H), 6.10 (s, 1H), 5.57 (s, 1H) ppm

^{13}C NMR (101 MHz, CDCl$_3$): δ 159.8, 147.8, 145.3, 139.5, 137.0, 129.6, 129.0, 128.7, 122.7, 121.5, 99.0, 77.6 ppm

HRMS (ESI+): calculated for $C_{12}H_{11}INO$ $[M+H]^+$: 311.9880, found 311.9876.

HPLC (AD-H, 0.46*25 cm, 5μm, hexane / ethanol = 95/5, flow 1 mL/min, detection at 254 nm) retention time = 14.346 min (major) and 16.841 min (minor).

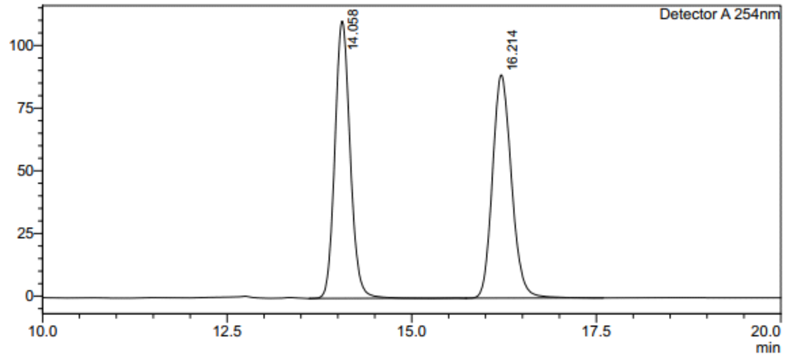

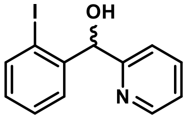

(±)-**C2**

<Peak Table>

Detector A 254nm

Peak#	Ret. Time	Area	Height	Conc.	Unit	Mark	Name
1	14.058	1594909	110649	49.940		S	
2	16.214	1598741	89045	50.060		V	
Total		3193650	199694				

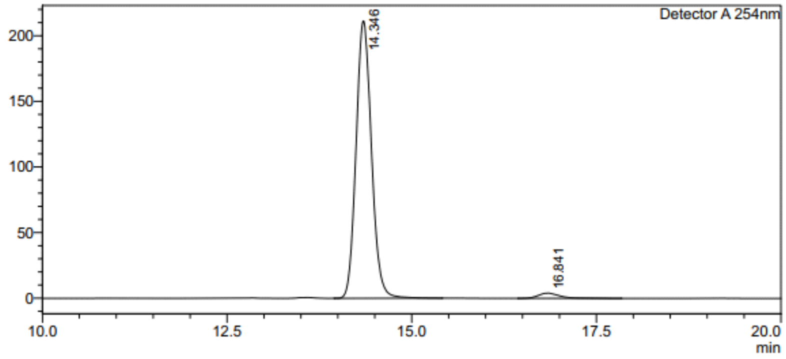

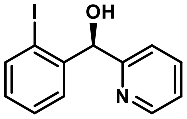

C2
97% yield, 95% ee
obtained by catalyst **B**

<Peak Table>

Detector A 254nm

Peak#	Ret. Time	Area	Height	Conc.	Unit	Mark	Name
1	14.346	3070178	211368	97.473			
2	16.841	79588	4019	2.527			
Total		3149766	215387				

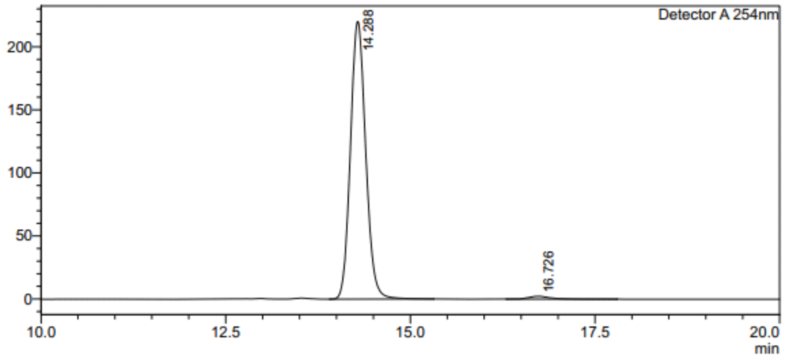

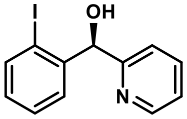

C2
96% yield, 97% ee
obtained by Ru-catalyst **B1**

<Peak Table>

Detector A 254nm

Peak#	Ret. Time	Area	Height	Conc.	Unit	Mark	Name
1	14.288	3169724	220175	98.553			
2	16.726	46546	2267	1.447			
Total		3216270	222442				

Supplementary Table 4. Crystal data and structure refinement for **C2**[1]

CCDC Number	1897334
Empirical formula	$C_{12}H_{10}INO$
Formula weight	311.11
Temperature/K	100
Crystal system	monoclinic
Space group	$P2_1/c$
a/Å	16.0930(13)
b/Å	9.8036(6)
c/Å	6.9586(6)
α/°	90
β/°	93.020(2)
γ/°	90
Volume/Å^3	1096.33(15)
Z	4
ρ_{calc}g/cm^3	1.885
μ/mm^{-1}	2.892
F(000)	600.0
Crystal size/mm^3	0.42 × 0.38 × 0.29
Radiation	MoKα (λ = 0.71073)
2Θ range for data collection/°	7.518 to 55.166
Index ranges	$0 \leq h \leq 20, -12 \leq k \leq 0, -9 \leq l \leq 9$
Reflections collected	2477
Independent reflections	2477 [R_{int} = ?, R_{sigma} = 0.0372]
Data/restraints/parameters	2477/6/139
Goodness-of-fit on F^2	1.108
Final R indexes [I>=2σ (I)]	R_1 = 0.0253, wR_2 = 0.0686
Final R indexes [all data]	R_1 = 0.0258, wR_2 = 0.0692
Largest diff. peak/hole / e Å^{-3}	0.84/-1.19

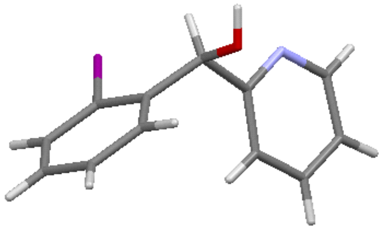

Supplementary Figure 3. The single crystal configuration of **C2** is demonstrated in a capped sticks mode.

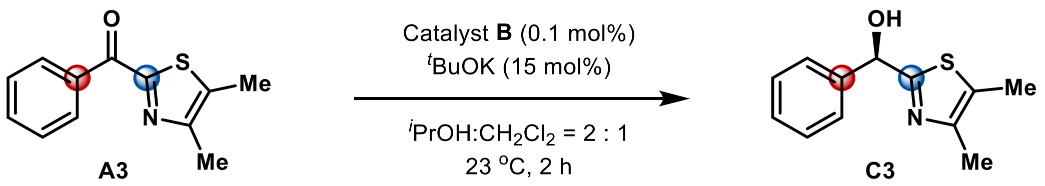

Following the general procedure, **C3** (29.3 mg, 67% yield, 87% ee) was obtained as white solid. Its major pure enantiomer was purified by chiral preparative HPLC (OD-H, 2.0*25 cm, 5μm) and the *X*-ray crystal of the optically pure enantiomer (100% ee) was obtained by liquid/liquid diffusion with CH_2Cl_2/hexane system.

^{1}H NMR (600 MHz, $CDCl_3$): δ 7.47 (d, *J* = 7.4 Hz, 2H), 7.35 (t, *J* = 7.4 Hz, 2H), 7.30 (t, *J* = 7.4 Hz, 1H), 5.94 (s, 1H), 4.54 (bs, 1H), 2.27 (s, 3H), 2.25 (s, 3H) ppm
^{13}C NMR (151 MHz, $CDCl_3$): δ 169.4, 147.4, 141.7, 128.5, 128.2, 127.1, 126.5, 73.4, 14.4, 11.2 ppm
HRMS (ESI+): calculated for $C_{12}H_{14}NOS$ $[M+H]^+$: 220.0791, found 220.0794.
HPLC (OD-H, 0.46*25 cm, 5μm, hexane / ethanol = 90/10, flow 1 mL/min, detection at 254 nm) retention time = 5.303 min (minor) and 6.593 min (major).

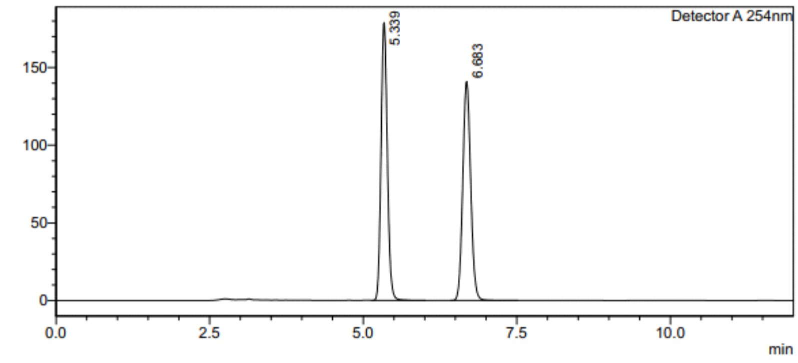

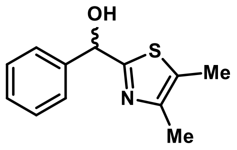

(±)-**C3**

<Peak Table>

Detector A 254nm

Peak#	Ret. Time	Area	Height	Conc.	Unit	Mark	Name
1	5.339	1275922	178980	50.022			
2	6.683	1274775	140954	49.978		V	
Total		2550696	319934				

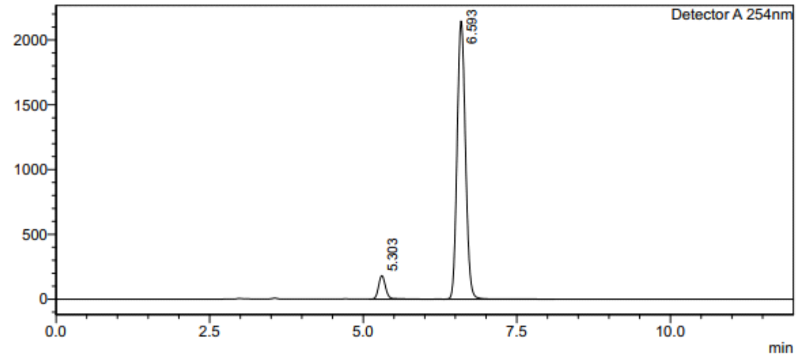

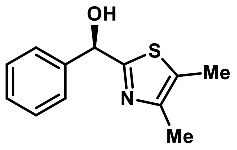

C3
67% yield, 87% ee
obtained by catalyst **B**

<Peak Table>

Detector A 254nm

Peak#	Ret. Time	Area	Height	Conc.	Unit	Mark	Name
1	5.303	1465840	181090	6.752		S	
2	6.593	20244583	2145976	93.248		V	
Total		21710423	2327066				

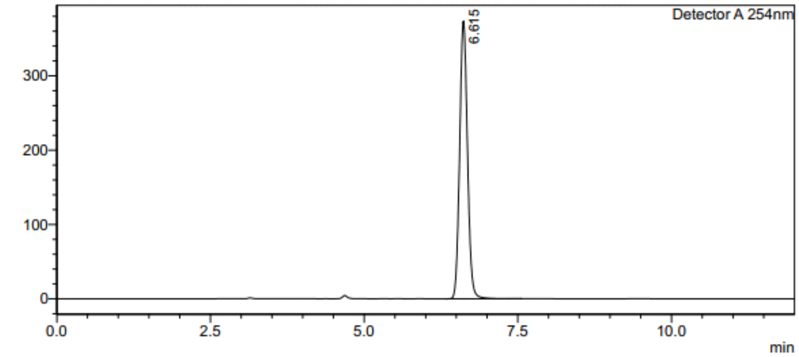

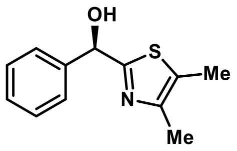

C3
> 99.9% ee

<Peak Table>

Detector A 254nm

Peak#	Ret. Time	Area	Height	Conc.	Unit	Mark	Name
1	6.615	3309366	373903	100.000			
Total		3309366	373903				

Supplementary Table 5. Crystal data and structure refinement for **C3**

CCDC Number	2017080
Empirical formula	$C_{12}H_{13}NOS$
Formula weight	219.29
Temperature/K	100.0
Crystal system	orthorhombic
Space group	$P2_12_12_1$
a/Å	7.0063(18)
b/Å	8.606(2)
c/Å	18.823(4)
α/°	90
β/°	90
γ/°	90
Volume/Å^3	1134.9(5)
Z	4
ρ_{calc}g/cm^3	1.283
μ/mm^{-1}	0.257
F(000)	464.0
Crystal size/mm^3	0.36 × 0.31 × 0.28
Radiation	MoKα (λ = 0.71073)
2Θ range for data collection/°	5.204 to 61.102
Index ranges	$-10 \le h \le 8$, $-12 \le k \le 12$, $-19 \le l \le 26$
Reflections collected	16000
Independent reflections	3448 [R_{int} = 0.0362, R_{sigma} = 0.0267]
Data/restraints/parameters	3448/0/140
Goodness-of-fit on F^2	1.083
Final R indexes [I>=2σ (I)]	R_1 = 0.0255, wR_2 = 0.0670
Final R indexes [all data]	R_1 = 0.0275, wR_2 = 0.0682
Largest diff. peak/hole / e Å^{-3}	0.26/-0.27
Flack parameter	-0.02(2)

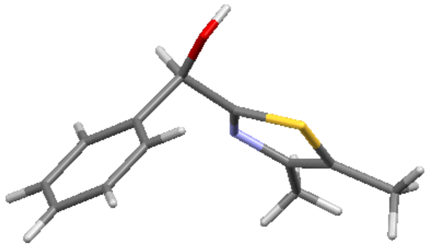

Supplementary Figure 4. The single crystal configuration of **C3** is demonstrated in a capped sticks mode.

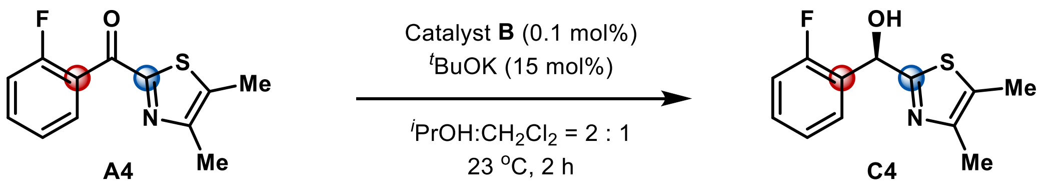

Following the general procedure, **C4** (46.9 mg, 99% yield, 92% ee) was obtained as white solid. Its major pure enantiomer was purified by chiral preparative HPLC (OD-H, 2.0*25 cm, 5μm) and the *X*-ray crystal of the optically pure enantiomer (100% ee) was obtained by liquid/liquid diffusion with CH_2Cl_2/hexane system.

^{1}H NMR (600 MHz, $CDCl_3$): δ 7.58 – 7.55 (m, 1H), 7.32 – 7.27 (m, 1H), 7.17 (t, *J* = 7.5 Hz, 1H), 7.05 (t, *J* = 9.3 Hz, 1H), 6.26 (s, 1H), 4.62 (s, 1H), 2.28 (s, 3H), 2.27 (s, 3H) ppm
^{13}C NMR (151 MHz, $CDCl_3$): δ 167.8, 156.0 (d, *J* = 247.3 Hz), 147.5, 129.8 (d, *J* = 8.4 Hz), 129.0 (d, *J* = 13.0 Hz), 128.1 (d, *J* = 3.4 Hz), 127.2, 124.4 (d, *J* = 3.2 Hz), 115.4 (d, *J* = 21.4 Hz), 67.2 (d, *J* = 3.5 Hz), 14.5, 11.3 ppm
^{19}F NMR (565 MHz, $CDCl_3$): δ -118.18 (s) ppm
HRMS (ESI+): calculated for $C_{12}H_{13}FNOS$ $[M+H]^+$: 238.0796, found 238.0700.
HPLC (OD-H, 0.46*25 cm, 5μm, hexane / ethanol = 95/5, flow 1 mL/min, detection at 254 nm) retention time = 6.481 min (minor) and 7.343 min (major).

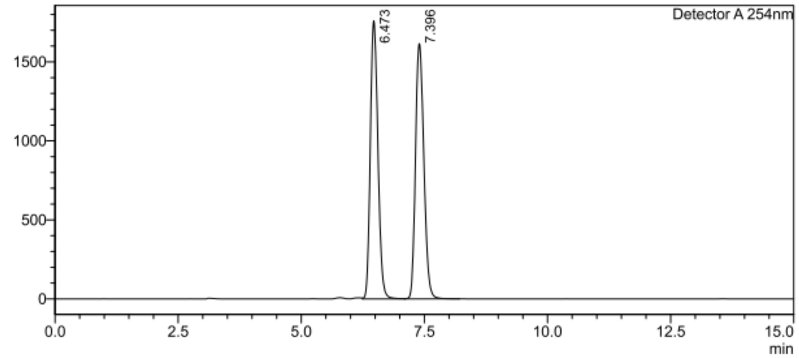

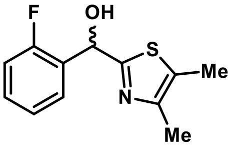

(±)-**C4**

<Peak Table>

Detector A 254nm

Peak#	Ret. Time	Area	Height	Conc.	Unit	Mark	Name
1	6.473	18982890	1758688	49.923			
2	7.396	19041621	1613761	50.077		V M	
Total		38024510	3372449				

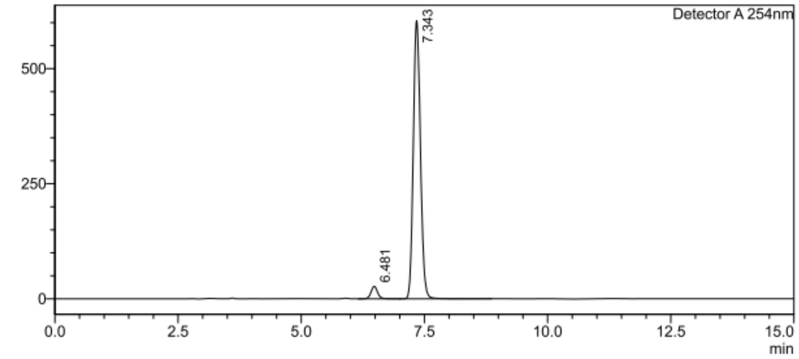

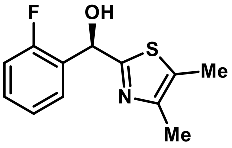

C4
99% yield, 92% ee
obtained by catalyst **B**

<Peak Table>

Detector A 254nm

Peak#	Ret. Time	Area	Height	Conc.	Unit	Mark	Name
1	6.481	251899	27015	4.001		S	
2	7.343	6043549	603852	95.999		SV	
Total		6295447	630867				

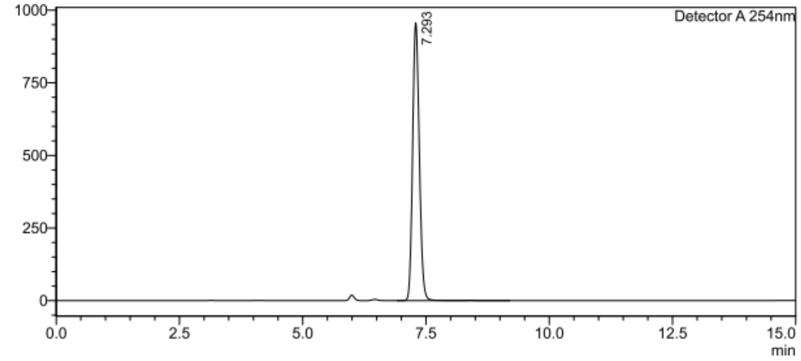

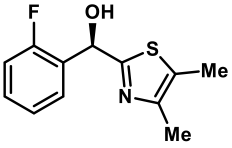

C4
> 99.9% ee

<Peak Table>

Detector A 254nm

Peak#	Ret. Time	Area	Height	Conc.	Unit	Mark	Name
1	7.293	9027770	955974	100.000		S	
Total		9027770	955974				

Supplementary Table 6. Crystal data and structure refinement for **C4**

CCDC Number	2017098
Empirical formula	$C_{12}H_{12}FNOS$
Formula weight	237.29
Temperature/K	100.03
Crystal system	orthorhombic
Space group	$P2_12_12_1$
a/Å	8.5288(8)
b/Å	9.0978(8)
c/Å	14.5650(13)
α/°	90
β/°	90
γ/°	90
Volume/Å^3	1130.15(18)
Z	4
ρ_{calc}g/cm^3	1.395
μ/mm^{-1}	0.276
F(000)	496.0
Crystal size/mm^3	0.41 × 0.38 × 0.36
Radiation	MoKα (λ = 0.71073)
2Θ range for data collection/°	5.28 to 56.694
Index ranges	$-11 \le h \le 9$, $-12 \le k \le 11$, $-18 \le l \le 19$
Reflections collected	14506
Independent reflections	2808 [R_{int} = 0.0457, R_{sigma} = 0.0323]
Data/restraints/parameters	2808/0/149
Goodness-of-fit on F^2	1.085
Final R indexes [I>=2σ (I)]	R_1 = 0.0270, wR_2 = 0.0692
Final R indexes [all data]	R_1 = 0.0287, wR_2 = 0.0702
Largest diff. peak/hole / e Å^{-3}	0.27/-0.18
Flack parameter	0.02(3)

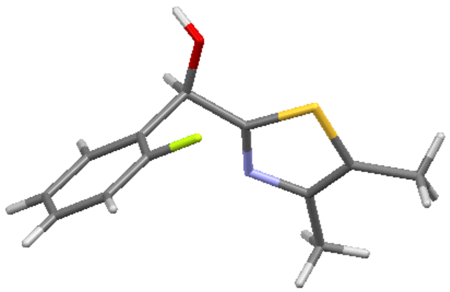

Supplementary Figure 5. The single crystal configuration of **C4** is demonstrated in a capped sticks mode.

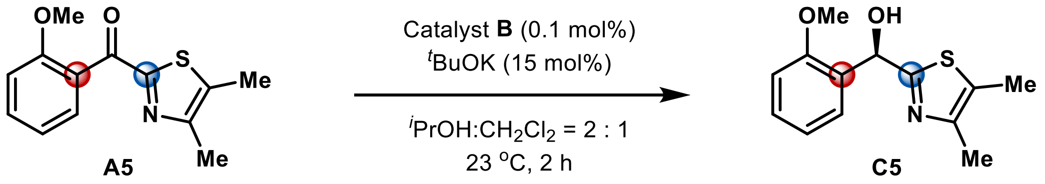

Following the general procedure, **C5** (49.3 mg, 99% yield, 94% ee) was obtained as white solid.

^{1}H NMR (600 MHz, $CDCl_3$): δ 7.42 (d, *J* = 7.5 Hz, 1H), 7.29 (t, *J* = 8.0 Hz, 1H), 6.97 (t, *J* = 7.5 Hz, 1H), 6.90 (d, *J* = 8.0 Hz, 1H), 6.20 (s, 1H), 4.15 (s, 1H), 3.85 (s, 3H), 2.28 (s, 3H), 2.28 (s, 3H) ppm
^{13}C NMR (151 MHz, $CDCl_3$): δ 168.5, 156.5, 147.1, 130.0, 129.2, 127.5, 126.6, 120.9, 110.6, 69.5, 55.4, 14.6, 11.3 ppm
HRMS (ESI+): calculated for $C_{13}H_{16}NO_2S$ $[M+H]^+$: 250.0896, found 250.0899.
HPLC (OD-H, 0.46*25 cm, 5μm, hexane / ethanol = 95/5, flow 1 mL/min, detection at 254 nm) retention time = 9.875 min (minor) and 10.916 min (major).

The major enantiomer of **C5** (94% ee) was purified by chiral preparative HPLC (OD-H, 2.0*25 cm, 5μm). Because we were unable to grow *X*-ray qualified crystal of the optically pure **C5** (100% ee), we derivatize it with (1*S*)-(-)-camphanic acid chloride to afford **D5** as a single diastereomer. Then the *X*-ray crystal of the optically pure **D5** was obtained by liquid/liquid diffusion with CH_2Cl_2/hexane system.

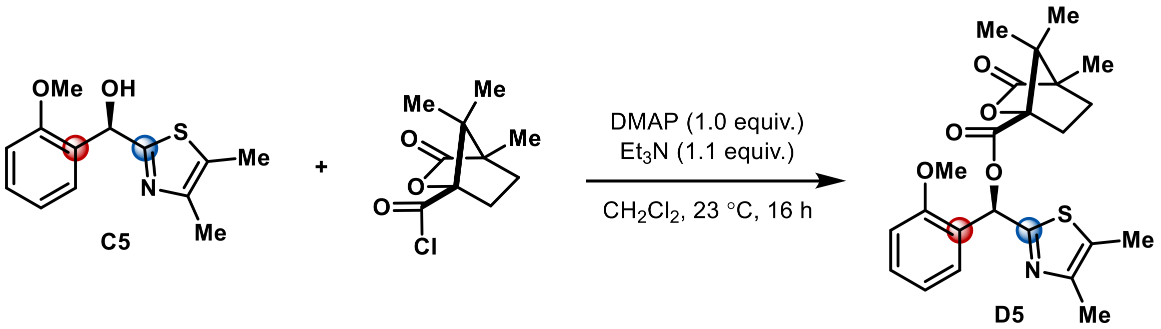

^{1}H NMR (600 MHz, $CDCl_3$): δ 7.49 (s, 1H), 7.41 (d, *J* = 7.6 Hz, 1H), 7.32 (t, *J* = 7.8 Hz, 1H), 6.96 (t, *J* = 7.8 Hz, 1H), 6.89 (d, *J* = 8.4 Hz, 1H), 3.83 (s, 3H), 2.52 – 2.45 (m, 1H), 2.30 (s, 3H), 2.28 (s, 3H), 2.08 – 2.02 (m, 1H), 1.94– 1.88 (m, 1H), 1.71 – 1.64 (m, 1H), 1.10 (s, 3H), 1.03 (s, 3H), 0.96 (s, 3H) ppm
^{13}C NMR (151 MHz, $CDCl_3$): δ 178.3, 166.1 162.9, 156.7, 148.5, 130.2, 128.15 (s), 127.2, 125.9, 120.7, 110.8, 91.1, 70.2, 55.6, 54.9, 54.5, 30.65 (s), 28.9, 16.7, 16.6, 14.7, 11.2, 9.7 ppm
HRMS (ESI+): calculated for $C_{23}H_{28}NO_5S$ $[M+H]^+$: 430.1683, found 430.1682.

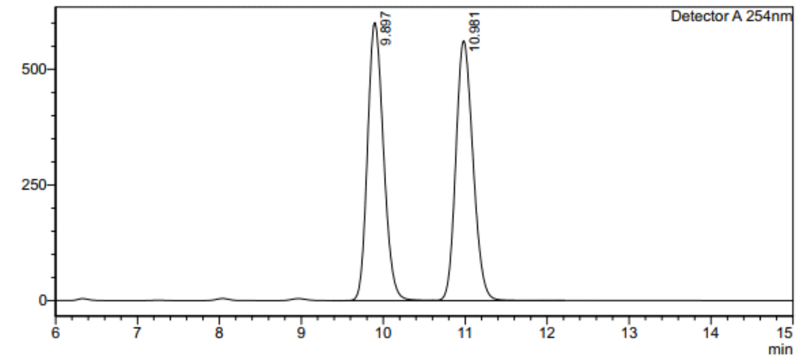

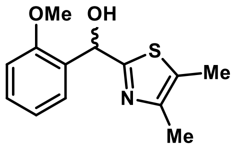

(±)-**C5**

<Peak Table>

Detector A 254nm

Peak#	Ret. Time	Area	Height	Conc.	Unit	Mark	Name
1	9.897	8148996	600717	49.935			
2	10.981	8170076	561559	50.065		V	
Total		16319072	1162276				

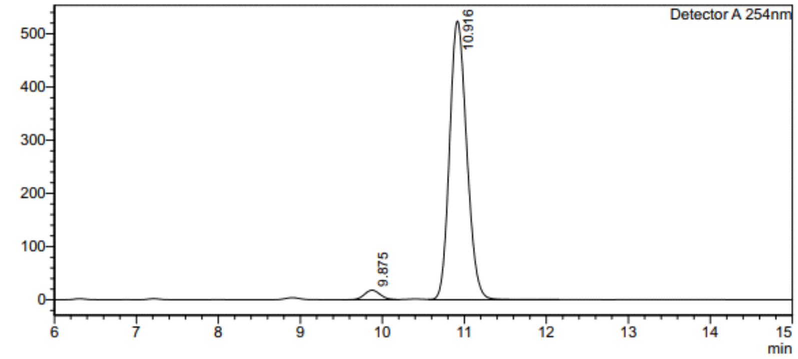

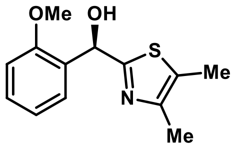

C5
99% yield, 94% ee
obtained by catalyst **B**

<Peak Table>

Detector A 254nm

Peak#	Ret. Time	Area	Height	Conc.	Unit	Mark	Name
1	9.875	240286	17834	3.047			
2	10.916	7646988	524051	96.953			
Total		7887274	541885				

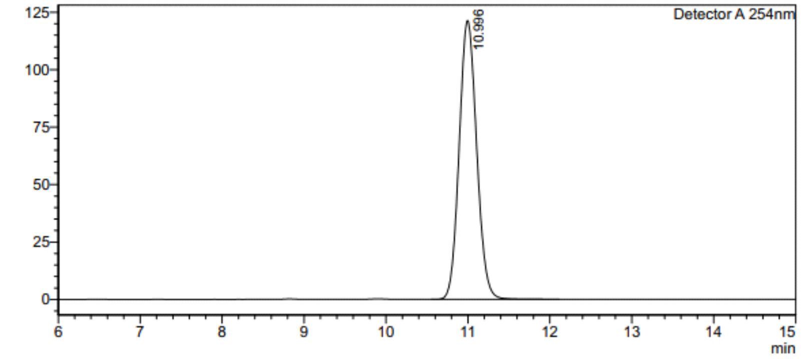

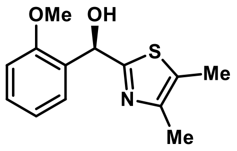

C5
> 99.9% ee

<Peak Table>

Detector A 254nm

Peak#	Ret. Time	Area	Height	Conc.	Unit	Mark	Name
1	10.996	1801901	121233	100.000			
Total		1801901	121233				

Supplementary Table 7. Crystal data and structure refinement for **D5**

CCDC Number	2017150
Empirical formula	$C_{23}H_{27}NO_5S$
Formula weight	429.51
Temperature/K	100
Crystal system	orthorhombic
Space group	$P2_12_12_1$
a/Å	7.7220(4)
b/Å	16.1361(8)
c/Å	17.8123(9)
α/°	90
β/°	90
γ/°	90
Volume/Å^3	2219.47(19)
Z	4
ρ_{calc}g/cm^3	1.285
μ/mm^{-1}	0.179
F(000)	912.0
Crystal size/mm^3	0.35 × 0.32 × 0.28
Radiation	MoKα (λ = 0.71073)
2Θ range for data collection/°	4.574 to 55.128
Index ranges	$-10 \leq h \leq 10$, $-20 \leq k \leq 20$, $-23 \leq l \leq 23$
Reflections collected	21637
Independent reflections	5100 [R_{int} = 0.0469, R_{sigma} = 0.0382]
Data/restraints/parameters	5100/0/278
Goodness-of-fit on F^2	1.024
Final R indexes [I>=2σ (I)]	R_1 = 0.0338, wR_2 = 0.0833
Final R indexes [all data]	R_1 = 0.0382, wR_2 = 0.0859
Largest diff. peak/hole / e Å^{-3}	0.25/-0.22
Flack parameter	-0.01(3)

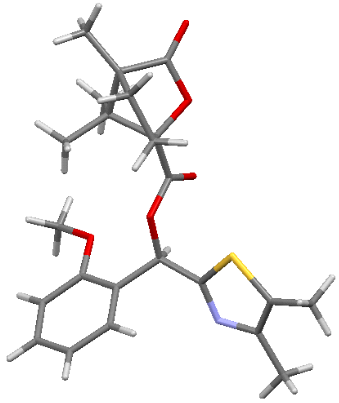

Supplementary Figure 6. The single crystal configuration of **C5** is demonstrated in a capped sticks mode.

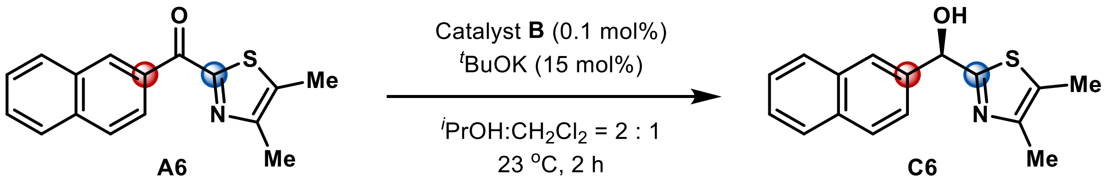

Following the general procedure, **C6** (39.3 mg, 73% yield, 90% ee) was obtained as white solid. Its major pure enantiomer was purified by chiral preparative HPLC (OD-H, 2.0*25 cm, 5μm) and the *X*-ray crystal of the optically pure enantiomer (100% ee) was obtained by liquid/liquid diffusion with CH_2Cl_2/hexane system.

^{1}H NMR (600 MHz, $CDCl_3$): δ 7.95 (s, 1H), 7.87 – 7.80 (m, 3H), 7.55 (d, *J* = 8.4 Hz, 1H), 7.50 – 7.46 (m, 2H), 6.10 (s, 1H), 3.77 (s, 1H), 2.30 (s, 3H), 2.28 (s, 3H) ppm
^{13}C NMR (151 MHz, $CDCl_3$): δ 168.6, 147.5, 139.00, 133.3, 133.2, 128.6, 128.2, 127.7, 127.4, 126.3, 126.2, 125.6, 124.3, 73.9, 14.6, 11.3 ppm
HRMS (ESI+): calculated for $C_{16}H_{16}NOS$ $[M+H]^+$: 270.0947, found 270.0950.
HPLC (OD-H, 0.46*25 cm, 5μm, hexane / ethanol = 90/10, flow 1 mL/min, detection at 254 nm) retention time = 7.020 min (minor) and 7.607 min (major).

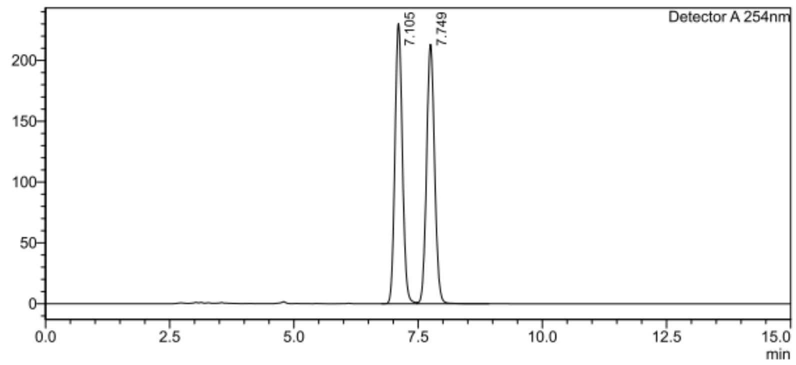

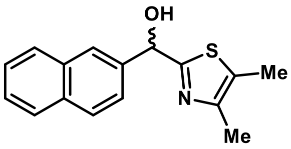

(±)-**C6**

<Peak Table>

Detector A 254nm

Peak#	Ret. Time	Area	Height	Conc.	Unit	Mark	Name
1	7.105	2391140	230338	49.885			
2	7.749	2402150	213325	50.115		SV	
Total		4793291	443663				

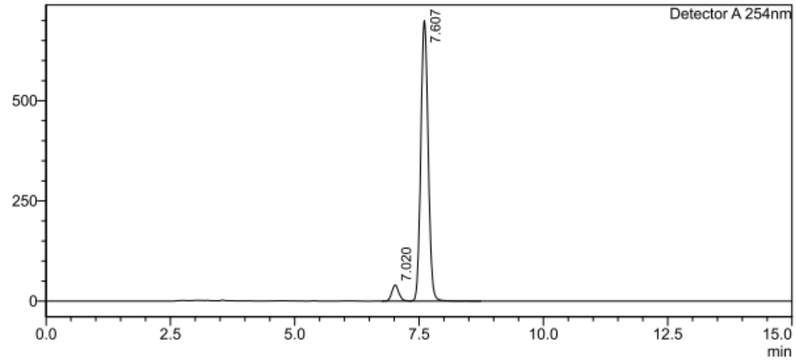

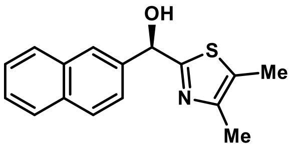

C6
73% yield, 90% ee
obtained by catalyst **B**

<Peak Table>

Detector A 254nm

Peak#	Ret. Time	Area	Height	Conc.	Unit	Mark	Name
1	7.020	400234	40018	5.278			
2	7.607	7183044	699520	94.722		V	
Total		7583278	739539				

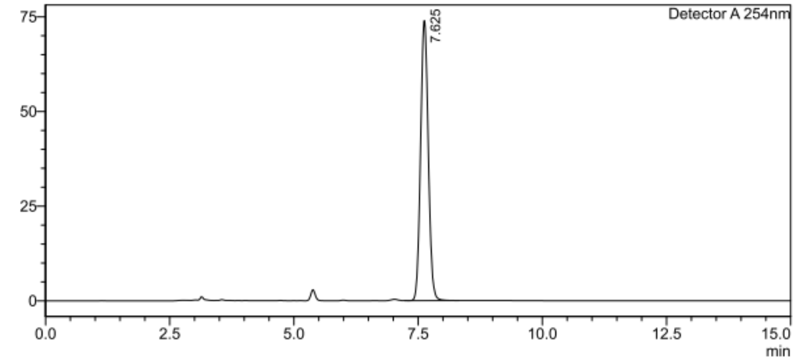

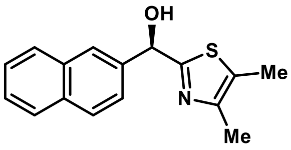

C6
> 99.9% ee

<Peak Table>

Detector A 254nm

Peak#	Ret. Time	Area	Height	Conc.	Unit	Mark	Name
1	7.625	794093	73923	100.000			
Total		794093	73923				

Supplementary Table 8. Crystal data and structure refinement for **C6**

CCDC Number	2017187
Empirical formula	$C_{16}H_{15}NOS$
Formula weight	269.35
Temperature/K	100
Crystal system	monoclinic
Space group	$P2_1$
a/Å	13.8799(8)
b/Å	5.9358(4)
c/Å	16.5889(10)
α/°	90
β/°	103.625(2)
γ/°	90
Volume/Å^3	1328.27(14)
Z	4
ρ_{calc}g/cm^3	1.347
μ/mm^{-1}	0.234
F(000)	568.0
Crystal size/mm^3	0.38 × 0.05 × 0.05
Radiation	MoKα (λ = 0.71073)
2Θ range for data collection/°	4.37 to 54.964
Index ranges	$-18 \le h \le 17$, $-7 \le k \le 7$, $-21 \le l \le 21$
Reflections collected	21743
Independent reflections	6096 [R_{int} = 0.0914, R_{sigma} = 0.0882]
Data/restraints/parameters	6096/1/349
Goodness-of-fit on F^2	1.050
Final R indexes [I>=2σ (I)]	R_1 = 0.0814, wR_2 = 0.1992
Final R indexes [all data]	R_1 = 0.0943, wR_2 = 0.2105
Largest diff. peak/hole / e Å^{-3}	0.82/-0.64
Flack parameter	0.05(15)

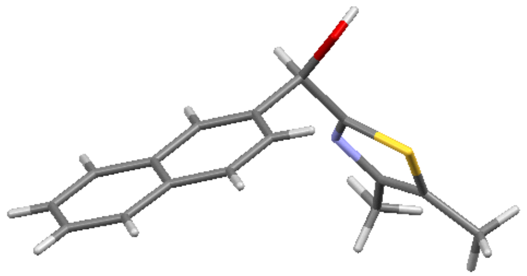

Supplementary Figure 7. The single crystal configuration of **C6** is demonstrated in a capped sticks mode.

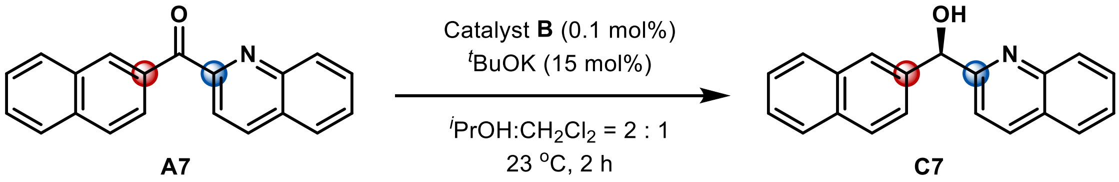

Following the general procedure, **C7** (39.2 mg, 68% yield, 84% ee) was obtained as white solid. Its major pure enantiomer was purified by chiral preparative HPLC (OD-H, 2.0*25 cm, 5μm) and the *X*-ray crystal of the optically pure enantiomer (100% ee) was obtained by liquid/liquid diffusion with CH_2Cl_2/hexane system.

^{1}H NMR (600 MHz, CDCl$_3$): δ 8.19 (d, *J* = 8.4 Hz, 1H), 8.03 (d, *J* = 8.4 Hz, 1H), 7.96 (s, 1H), 7.86 (d, *J* = 7.8 Hz, 1H), 7.83 – 7.76 (m, 4H), 7.56 (t, *J* = 7.8 Hz, 1H), 7.52 – 7.45 (m, 2H), 7.44 (d, *J* = 8.5 Hz, 1H), 7.21 (d, *J* = 8.5 Hz, 1H), 6.23 (d, *J* = 2.8 Hz, 1H), 6.04 (d, *J* = 2.8 Hz, 1H) ppm

^{13}C NMR (151 MHz, CDCl$_3$): δ 160.3, 146.0, 140.1, 137.0, 133.3, 133.2, 129.9, 128.8, 128.6, 128.0, 127.7, 127.6, 127.5, 126.7, 126.7, 126.2, 126.1, 125.0, 119.3, 75.3 ppm

HRMS (ESI+): calculated for $C_{20}H_{16}NO$ [M+H]$^+$: 286.1226, found 286.1231.

HPLC (OD-H, 0.46*25 cm, 5μm, hexane / ethanol = 80/20, flow 1 mL/min, detection at 254 nm) retention time = 7.347 min (minor) and 11.502 min (major).

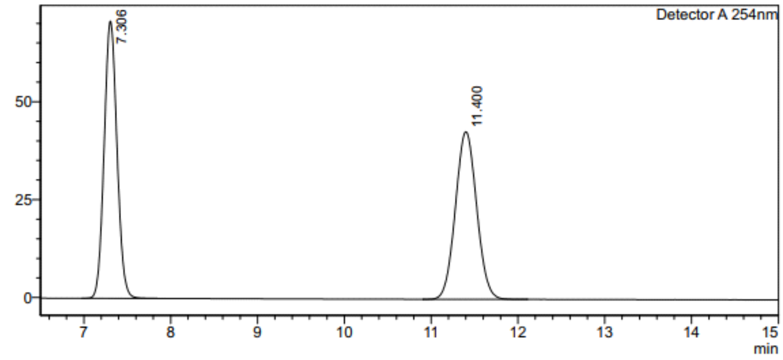

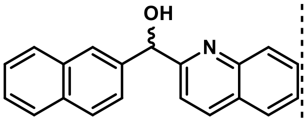

(±)-**C7**

<Peak Table>

Detector A 254nm

Peak#	Ret. Time	Area	Height	Conc.	Unit	Mark	Name
1	7.306	729362	70796	50.007			
2	11.400	729153	42805	49.993			
Total		1458515	113600				

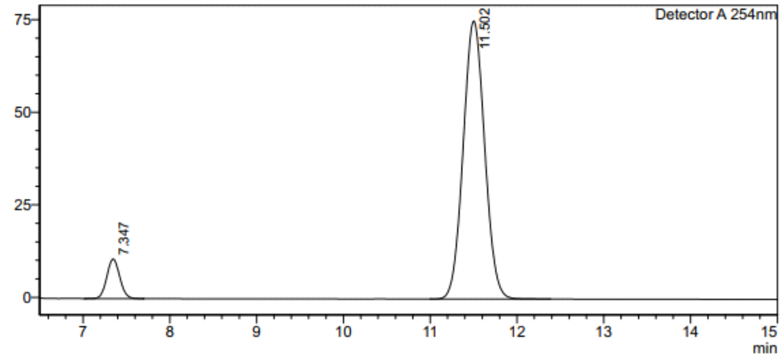

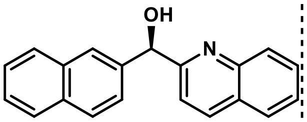

C7
68% yield, 84% ee
obtained by catalyst **B**

<Peak Table>

Detector A 254nm

Peak#	Ret. Time	Area	Height	Conc.	Unit	Mark	Name
1	7.347	110832	10692	7.902			
2	11.502	1291722	75151	92.098			
Total		1402553	85842				

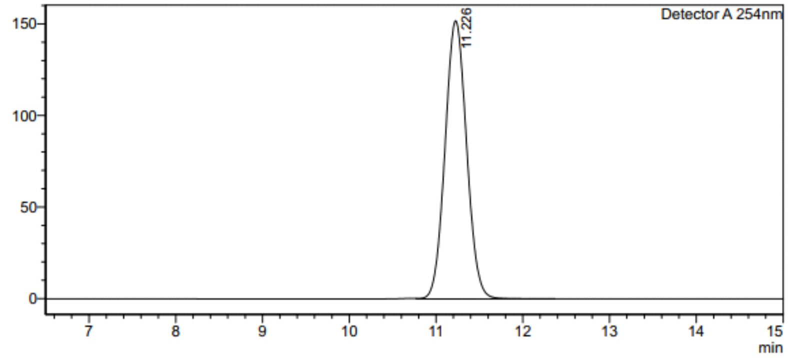

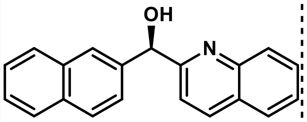

C7
> 99.9% ee

<Peak Table>

Detector A 254nm

Peak#	Ret. Time	Area	Height	Conc.	Unit	Mark	Name
1	11.226	2629017	151966	100.000			
Total		2629017	151966				

Supplementary Table 9. Crystal data and structure refinement for **C7**

CCDC Number	2017094
Empirical formula	$C_{20}H_{15}NO$
Formula weight	285.33
Temperature/K	100
Crystal system	orthorhombic
Space group	$P2_12_12_1$
a/Å	6.0321(4)
b/Å	8.1905(5)
c/Å	27.9244(17)
α/°	90
β/°	90
γ/°	90
Volume/Å^3	1379.63(15)
Z	4
ρ_{calc}g/cm^3	1.374
μ/mm^{-1}	0.662
F(000)	600.0
Crystal size/mm^3	0.36 × 0.32 × 0.29
Radiation	CuKα (λ = 1.54178)
2Θ range for data collection/°	6.33 to 136.604
Index ranges	$-6 \le h \le 7$, $-9 \le k \le 9$, $-33 \le l \le 33$
Reflections collected	21593
Independent reflections	2525 [R_{int} = 0.0466, R_{sigma} = 0.0259]
Data/restraints/parameters	2525/0/201
Goodness-of-fit on F^2	1.077
Final R indexes [I>=2σ (I)]	R_1 = 0.0307, wR_2 = 0.0857
Final R indexes [all data]	R_1 = 0.0309, wR_2 = 0.0859
Largest diff. peak/hole / e Å^{-3}	0.25/-0.17
Flack parameter	0.10(15)

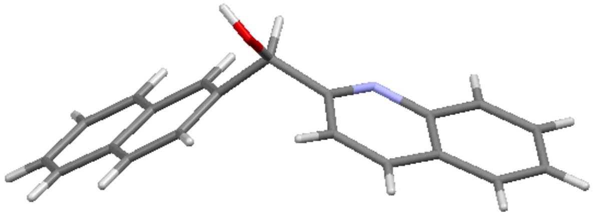

Supplementary Figure 8. The single crystal configuration of **C7** is demonstrated in a capped sticks mode.

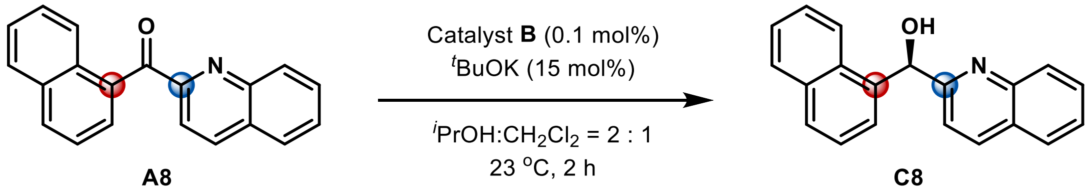

Following the general procedure, **C8** (51.9 mg, 91% yield, 94% ee) was obtained as white solid.

^{1}H NMR (600 MHz, CDCl$_3$): δ 8.28 – 8.15 (m, 2H), 7.98 (d, *J* = 8.4 Hz, 1H), 7.87 (d, *J* = 8.0 Hz, 1H), 7.84 (d, *J* = 8.0 Hz, 1H), 7.81 – 7.78 (m, 2H), 7.57 (t, *J* = 7.5 Hz, 1H), 7.51 (d, *J* = 7.2 Hz, 1H), 7.45 (t, *J* = 7.2 Hz, 2H), 7.44 – 7.37 (m, 1H), 7.11 (d, *J* = 8.4 Hz, 1H), 6.53 (s, 1H), 6.15 (s, 1H) ppm

^{13}C NMR (151 MHz, CDCl$_3$): δ 160.8, 146.1, 137.7, 137.1, 134.3, 131.5, 129.9, 129.0, 128.8, 128.7, 127.6, 127.5, 127.0, 126., 126.3, 125.6, 125.3, 124.3, 119.1, 73.9 ppm

HRMS (ESI+): calculated for $C_{20}H_{16}NO$ $[M+H]^+$: 286.1226, found 286.1230.

HPLC (AD-H, 0.46*25 cm, 5μm, hexane / ethanol = 80/20, flow 1 mL/min, detection at 254 nm) retention time = 14.399 min (minor) and 15.808 min (major).

The major enantiomer of **C8** (94% ee) was purified by chiral preparative HPLC (AD-H, 2.0*25 cm, 5μm). Because we were unable to grow *X*-ray qualified crystal of the optically pure **C8** (100% ee), we derivatize it with (1*S*)-(-)-camphanic acid chloride to afford **D8** as a single diastereomer. Then the *X*-ray crystal of the optically pure **D8** was obtained by liquid/liquid diffusion with CH_2Cl_2/hexane system.

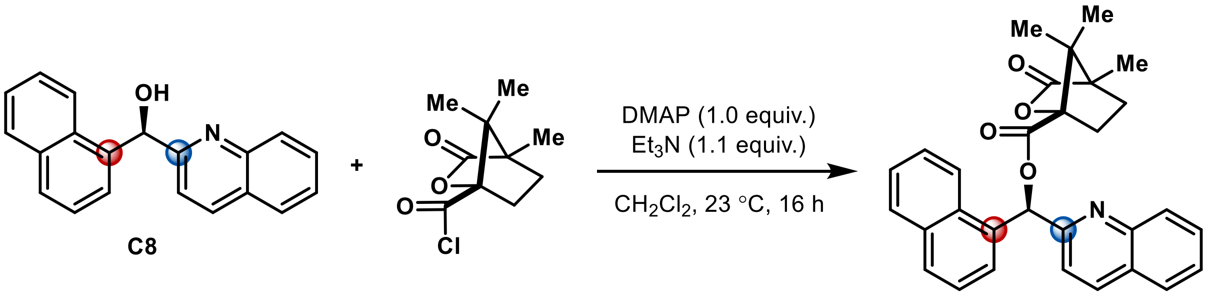

^{1}H NMR (600 MHz, CDCl$_3$): δ 8.35 (d, *J* = 8.4 Hz, 1H), 8.10 (d, *J* = 8.4 Hz, 1H), 8.07 (d, *J* = 8.4 Hz, 1H), 7.89 (s, 1H), 7.86 (d, *J* = 8.1 Hz, 2H), 7.77 (d, *J* = 8.1 Hz, 1H), 7.71 (t, *J* = 7.5 Hz, 1H), 7.68 (d, *J* = 7.2 Hz, 1H), 7.53 (t, *J* = 7.5 Hz, 1H), 7.51 – 7.45 (m, 3H), 7.44 (d, *J* = 8.4 Hz, 1H), 2.64 – 2.56 (m, 1H), 2.19 – 2.14 (m, 1H), 1.98 – 1.90 (m, 1H), 1.74 – 1.70 (m, 1H), 1.11 (s, 3H), 1.01 (s, 3H) , 1.01 (s, 3H) ppm

^{13}C NMR (151 MHz, CDCl$_3$): δ 178.4, 166.7, 157.8, 147.4, 136.9, 133.9, 133.7, 130.9, 129.7, 129.5, 128.7, 127.5, 127.4, 126.8, 126.6, 125.9, 125.9, 125.3, 124.2, 119.1, 91.3, 77.2, 55.0, 54.5, 31.0, 28.9, 16.8, 16.8, 9.7 ppm

HRMS (ESI+): calculated for $C_{30}H_{28}NO_4$ $[M+H]^+$: 466.2013, found 466.2013.

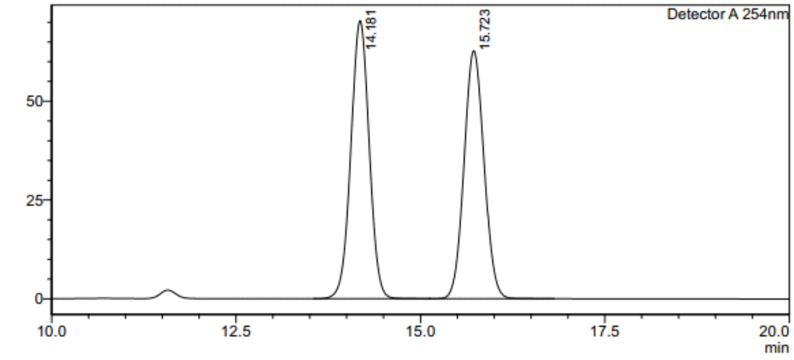

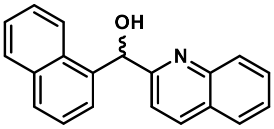

(±)-**C8**

<Peak Table>

Detector A 254nm

Peak#	Ret. Time	Area	Height	Conc.	Unit	Mark	Name
1	14.181	1219243	70369	50.653			
2	15.723	1187821	62766	49.347		V	
Total		2407063	133135				

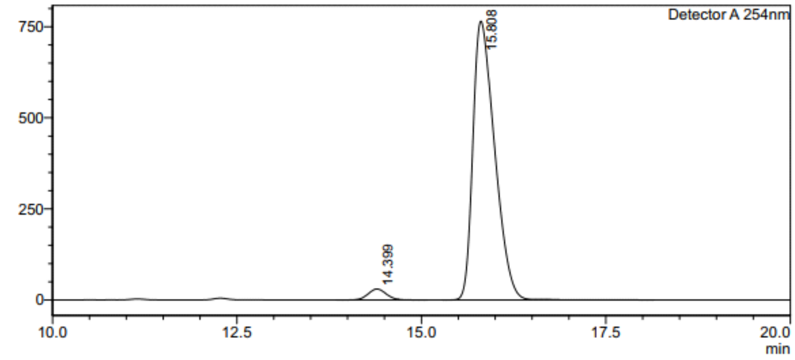

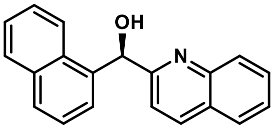

C8
91% yield, 94% ee
obtained by catalyst **B**

<Peak Table>

Detector A 254nm

Peak#	Ret. Time	Area	Height	Conc.	Unit	Mark	Name
1	14.399	514857	30025	3.173			
2	15.808	15710385	765283	96.827			
Total		16225241	795308				

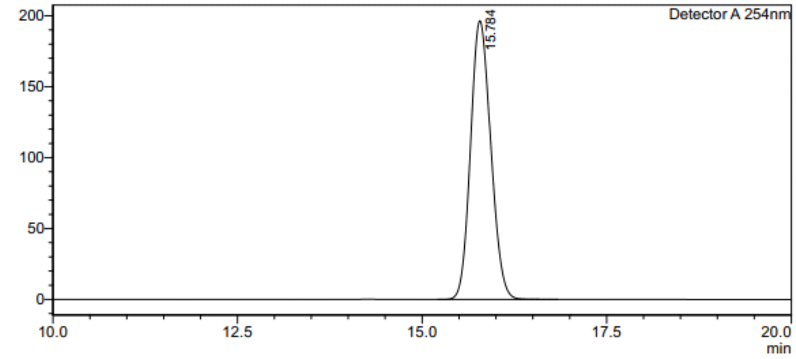

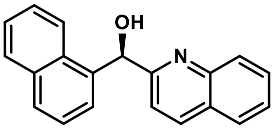

C8
> 99.9% ee

<Peak Table>

Detector A 254nm

Peak#	Ret. Time	Area	Height	Conc.	Unit	Mark	Name
1	15.784	3756223	196498	100.000			
Total		3756223	196498				

Supplementary Table 10. Crystal data and structure refinement for **D8**

CCDC Number	2017193
Empirical formula	$C_{30}H_{27}NO_4$
Formula weight	465.52
Temperature/K	100
Crystal system	orthorhombic
Space group	$P2_12_12_1$
a/Å	9.0790(3)
b/Å	10.2723(4)
c/Å	24.5579(8)
α/°	90
β/°	90
γ/°	90
Volume/Å^3	2290.32(14)
Z	4
ρ_{calc}g/cm^3	1.350
μ/mm^{-1}	0.717
F(000)	984.0
Crystal size/mm^3	0.36 × 0.3 × 0.28
Radiation	CuKα (λ = 1.54178)
2Θ range for data collection/°	7.198 to 137.432
Index ranges	-10 ≤ h ≤ 10, -12 ≤ k ≤ 12, -29 ≤ l ≤ 29
Reflections collected	44318
Independent reflections	4214 [R_{int} = 0.0408, R_{sigma} = 0.0187]
Data/restraints/parameters	4214/0/320
Goodness-of-fit on F^2	1.085
Final R indexes [I>=2σ (I)]	R_1 = 0.0302, wR_2 = 0.0773
Final R indexes [all data]	R_1 = 0.0303, wR_2 = 0.0775
Largest diff. peak/hole / e Å^{-3}	0.34/-0.14
Flack parameter	0.06(7)

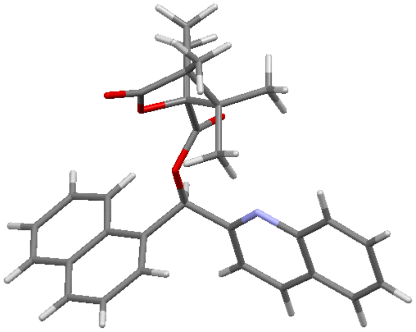

Supplementary Figure 9. The single crystal configuration of **D8** is demonstrated in a capped sticks mode..

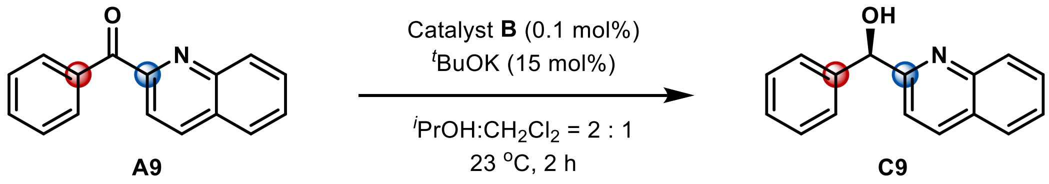

Following the general procedure, **C9** (45.6 mg, 97% yield, 85% ee) was obtained as white solid. Its major pure enantiomer was purified by chiral preparative HPLC (AD-H, 2.0*25 cm, 5μm) and the *X*-ray crystal of the optically pure enantiomer (100% ee) was obtained by liquid/liquid diffusion with CH_2Cl_2/hexane system.

^{1}H NMR (600 MHz, $CDCl_3$): δ 8.15 (d, *J* = 8.4 Hz, 1H), 8.05 (d, *J* = 8.4 Hz, 1H), 7.80 (d, *J* = 8.4 Hz, 1H), 7.76 (t, *J* = 7.6 Hz, 1H), 7.56 (t, *J* = 7.6 Hz, 1H), 7.42 (d, *J* = 7.4 Hz, 2H), 7.35 (t, *J* = 7.4 Hz, 2H), 7.32 – 7.27 (m, 1H), 7.19 (d, *J* = 8.4 Hz, 1H), 6.11 (s, 1H), 5.88 (s, 1H) ppm
^{13}C NMR (151 MHz, $CDCl_3$): δ 160.4, 146.0, 142.7, 137.0, 129.9, 128.8, 128.6, 128.0, 127.6, 127.5, 127.4, 126.6, 119.2, 75.2 ppm
HRMS (ESI+): calculated for $C_{16}H_{14}NO$ $[M+H]^+$: 236.1070, found 236.1073.
HPLC (AD-H, 0.46*25 cm, 5μm, hexane / ethanol = 90/10, flow 1 mL/min, detection at 254 nm) retention time = 18.624 min (major) and 23.649 min (minor).

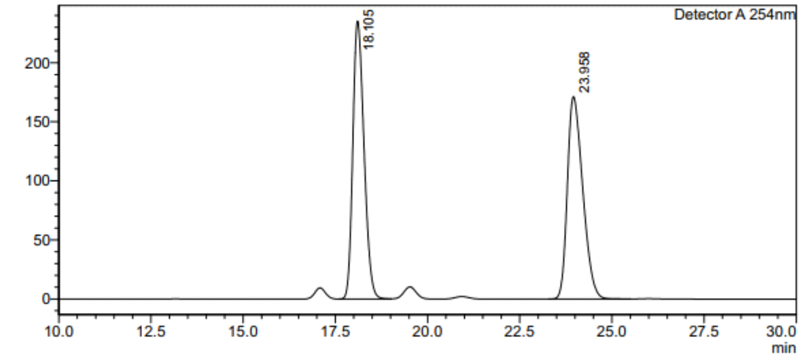

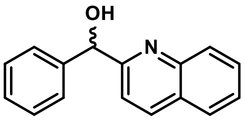

(±)-**C9**

<Peak Table>

Detector A 254nm

Peak#	Ret. Time	Area	Height	Conc.	Unit	Mark	Name
1	18.105	4977761	235420	49.960			
2	23.958	4985772	171428	50.040			
Total		9963533	406848				

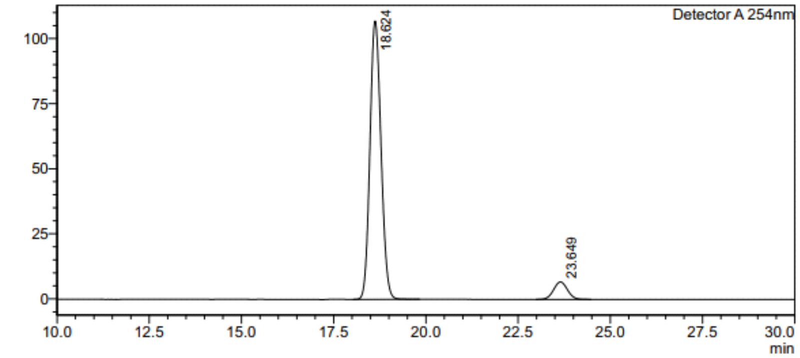

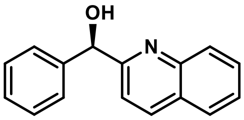

C9
97% yield, 85% ee
obtained by catalyst **B**

<Peak Table>

Detector A 254nm

Peak#	Ret. Time	Area	Height	Conc.	Unit	Mark	Name
1	18.624	2225376	106941	92.732			
2	23.649	174409	6693	7.268			
Total		2399785	113633				

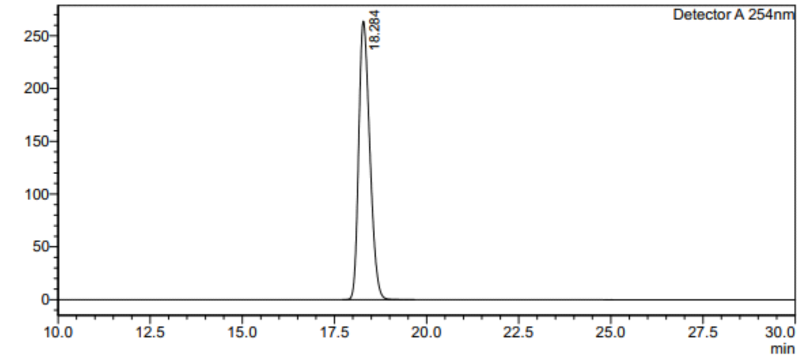

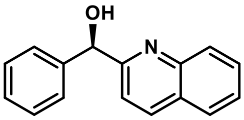

C9
> 99.9% ee

<Peak Table>

Detector A 254nm

Peak#	Ret. Time	Area	Height	Conc.	Unit	Mark	Name
1	18.284	5528568	263987	100.000			
Total		5528568	263987				

Supplementary Table 11. Crystal data and structure refinement for **C9**

CCDC Number	2017022
Empirical formula	$C_{16}H_{15}NO_2$
Formula weight	253.29
Temperature/K	100.01
Crystal system	monoclinic
Space group	$P2_1$
a/Å	8.7024(3)
b/Å	6.0158(2)
c/Å	12.8342(5)
α/°	90
β/°	106.7840(10)
γ/°	90
Volume/Å^3	643.27(4)
Z	2
ρ_{calc}g/cm^3	1.308
μ/mm^{-1}	0.693
F(000)	268.0
Crystal size/mm^3	0.45 × 0.42 × 0.38
Radiation	CuKα (λ = 1.54178)
2Θ range for data collection/°	7.194 to 137.528
Index ranges	$-9 \leq h \leq 10, -7 \leq k \leq 6, -15 \leq l \leq 15$
Reflections collected	8814
Independent reflections	2327 [R_{int} = 0.0339, R_{sigma} = 0.0324]
Data/restraints/parameters	2327/1/177
Goodness-of-fit on F^2	1.077
Final R indexes [I>=2σ (I)]	R_1 = 0.0401, wR_2 = 0.1076
Final R indexes [all data]	R_1 = 0.0406, wR_2 = 0.1081
Largest diff. peak/hole / e Å^{-3}	0.16/-0.17
Flack parameter	0.3(3)

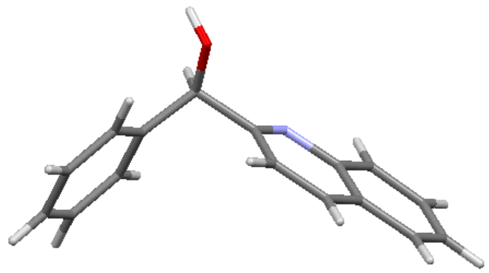

Supplementary Figure 10. The single crystal configuration of **D9** is demonstrated in a capped sticks mode.

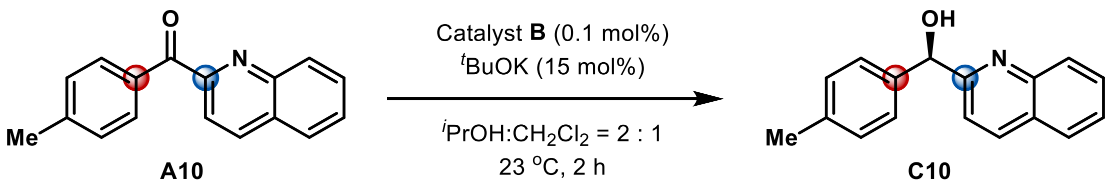

Following the general procedure, **C10** (40.7 mg, 82% yield, 83% ee) was obtained as white solid. Its major pure enantiomer was purified by chiral preparative HPLC (AD-H, 2.0*25 cm, 5μm) and the *X*-ray crystal of the optically pure enantiomer (100% ee) was obtained by liquid/liquid diffusion with CH_2Cl_2/hexane system.

^{1}H NMR (600 MHz, $CDCl_3$): δ 8.15 (d, *J* = 8.4 Hz, 1H), 8.05 (d, *J* = 8.4 Hz, 1H), 7.80 (d, *J* = 8.4 Hz, 1H), 7.75 (t, *J* = 7.8 Hz, 1H), 7.55 (t, *J* = 7.5 Hz, 1H), 7.29 (d, *J* = 7.8 Hz, 2H), 7.18 (d, *J* = 8.4 Hz, 1H), 7.15 (d, *J* = 7.5 Hz, 2H), 6.03 (s, 1H), 5.84 (s, 1H), 2.33 (s, 3H) ppm
^{13}C NMR (151 MHz, $CDCl_3$): δ 160.7, 146.0, 139.9, 137.7, 137.0, 129.9, 129.3, 128.8, 127.6, 127.6, 127.4, 126.6, 119.3, 75.0, 21.2 ppm
HRMS (ESI+): calculated for $C_{17}H_{16}NO$ $[M+H]^+$: 250.1226, found 250.1229.
HPLC (AD-H, 0.46*25 cm, 5μm, hexane / ethanol = 80/20, flow 1 mL/min, detection at 254 nm) retention time = 10.479 min (major) and 14.533 min (minor).

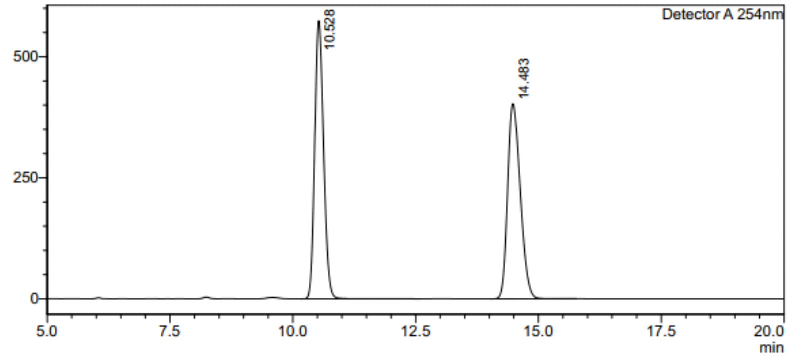

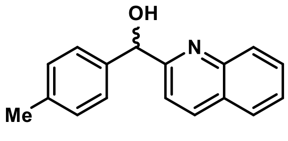

(±)-**C10**

<Peak Table>

Detector A 254nm

Peak#	Ret. Time	Area	Height	Conc.	Unit	Mark	Name
1	10.528	7174468	574345	49.978			
2	14.483	7180675	403138	50.022			
Total		14355143	977483				

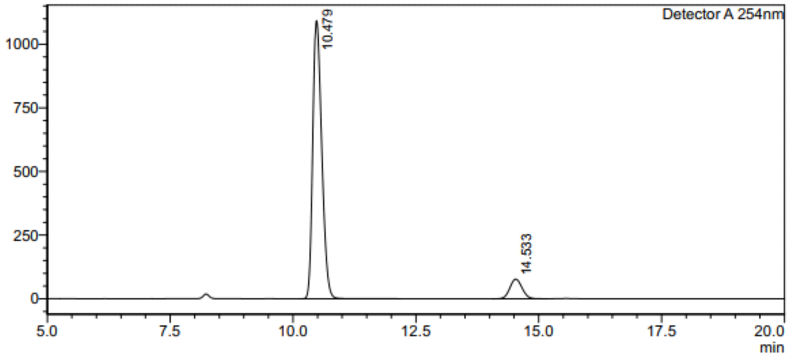

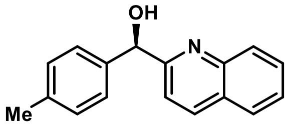

C10
82% yield, 83% ee
obtained by catalyst **B**

<Peak Table>

Detector A 254nm

Peak#	Ret. Time	Area	Height	Conc.	Unit	Mark	Name
1	10.479	13800414	1092384	91.260			
2	14.533	1321587	77016	8.740			
Total		15122002	1169400				

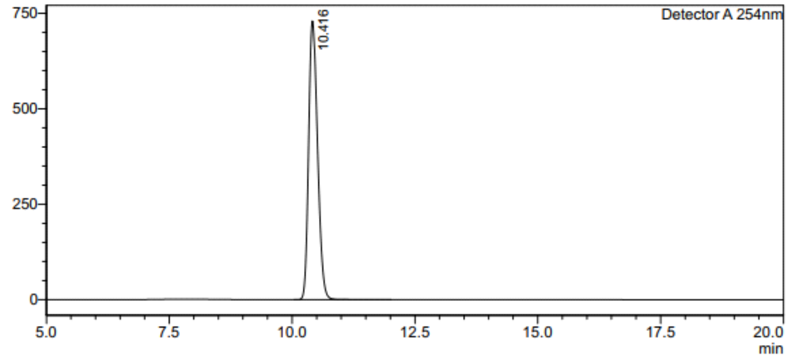

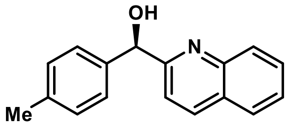

C10
> 99.9% ee

<Peak Table>

Detector A 254nm

Peak#	Ret. Time	Area	Height	Conc.	Unit	Mark	Name
1	10.416	9160920	729974	100.000			
Total		9160920	729974				

Supplementary Table 12. Crystal data and structure refinement for **C10**

CCDC Number	2017093
Empirical formula	$C_{17}H_{15}NO$
Formula weight	249.30
Temperature/K	100.0
Crystal system	orthorhombic
Space group	$P2_12_12_1$
a/Å	8.1606(2)
b/Å	12.2489(3)
c/Å	13.1244(3)
α/°	90
β/°	90
γ/°	90
Volume/Å^3	1311.89(5)
Z	4
ρ_{calc}g/cm^3	1.262
μ/mm^{-1}	0.614
F(000)	528.0
Crystal size/mm^3	0.42 × 0.41 × 0.39
Radiation	CuKα (λ = 1.54178)
2Θ range for data collection/°	15.968 to 136.532
Index ranges	$-7 \le h \le 9$, $-14 \le k \le 12$, $-12 \le l \le 15$
Reflections collected	7783
Independent reflections	2366 [R_{int} = 0.0260, R_{sigma} = 0.0236]
Data/restraints/parameters	2366/0/175
Goodness-of-fit on F^2	1.058
Final R indexes [I>=2σ (I)]	R_1 = 0.0280, wR_2 = 0.0717
Final R indexes [all data]	R_1 = 0.0285, wR_2 = 0.0720
Largest diff. peak/hole / e Å^{-3}	0.14/-0.15
Flack parameter	0.00(8)

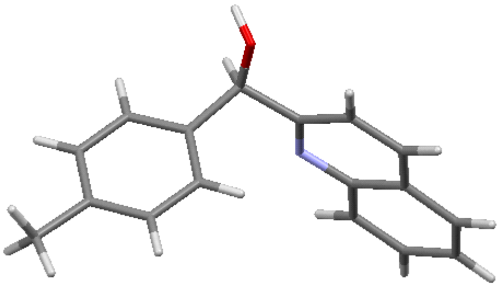

Supplementary Figure 11. The single crystal configuration of **C10** is demonstrated in a capped sticks mode..

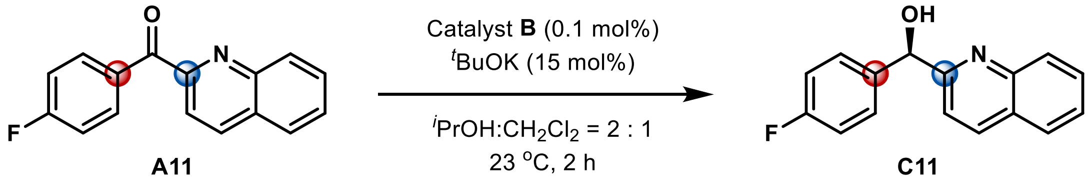

Following the general procedure, **C11** (43.5 mg, 86% yield, 85% ee) was obtained as white solid. Its major pure enantiomer was purified by chiral preparative HPLC (AD-H, 2.0*25 cm, 5μm) and the *X*-ray crystal of the optically pure enantiomer (100% ee) was obtained by liquid/liquid diffusion with CH_2Cl_2/hexane system.

^{1}H NMR (600 MHz, $CDCl_3$): δ 8.14 (d, *J* = 8.4 Hz, 1H), 8.05 (d, *J* = 8.4 Hz, 1H), 7.80 (d, *J* = 7.8 Hz, 1H), 7.76 (t, *J* = 7.2 Hz, 1H), 7.56 (t, *J* = 7.2 Hz, 1H), 7.38 (dd, *J* = 8.4, 5.6 Hz, 2H), 7.15 (d, *J* = 8.4 Hz, 1H), 7.02 (t, *J* = 8.4 Hz, 2H), 6.14 (s, 1H), 5.87 (s, 1H) ppm
^{13}C NMR (151 MHz, $CDCl_3$): δ 162.4 (d, *J* = 246.0 Hz), 160.2, 145.9, 138.6 (d, *J* = 2.3 Hz), 137.1, 130.0, 129.1 (d, *J* = 8.4 Hz), 128.7, 127.6, 127.4, 126.7, 119.0, 115.4 (d, *J* = 21.6 Hz), 74.4 ppm
^{19}F NMR (565 MHz, $CDCl_3$): δ -114.31 (s) ppm
HRMS (ESI+): calculated for $C_{16}H_{13}FNO$ $[M+H]^+$: 254.0976, found 254.0979.
HPLC (AD-H, 0.46*25 cm, 5μm, hexane / ethanol = 90/10, flow 1 mL/min, detection at 254 nm) retention time = 18.394 min (major) and 22.635 min (minor).

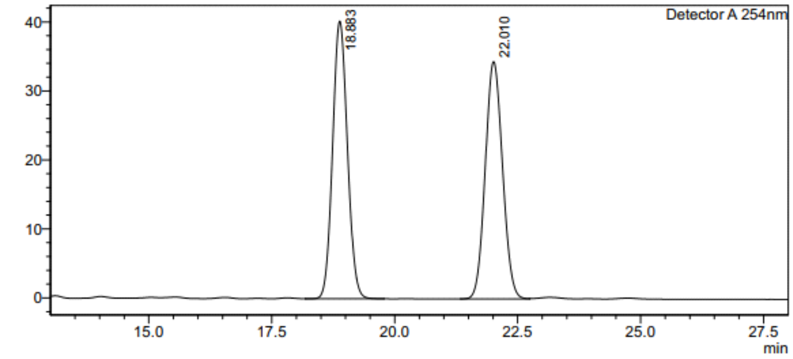

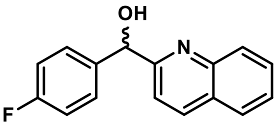

(±)-**C11**

<Peak Table>

Detector A 254nm

Peak#	Ret. Time	Area	Height	Conc.	Unit	Mark	Name
1	18.883	844672	40251	50.110			
2	22.010	840959	34397	49.890			
Total		1685631	74648				

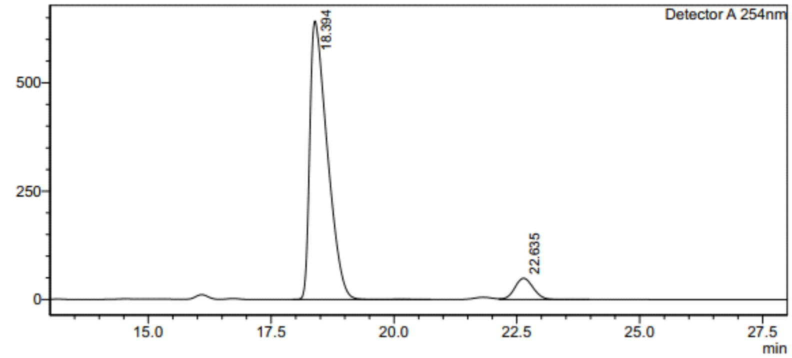

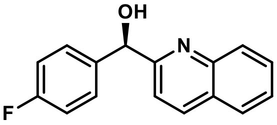

C11
86% yield, 85% ee
obtained by catalyst **B**

<Peak Table>

Detector A 254nm

Peak#	Ret. Time	Area	Height	Conc.	Unit	Mark	Name
1	18.394	15887031	642881	92.535			
2	22.635	1281683	49093	7.465			
Total		17168714	691975				

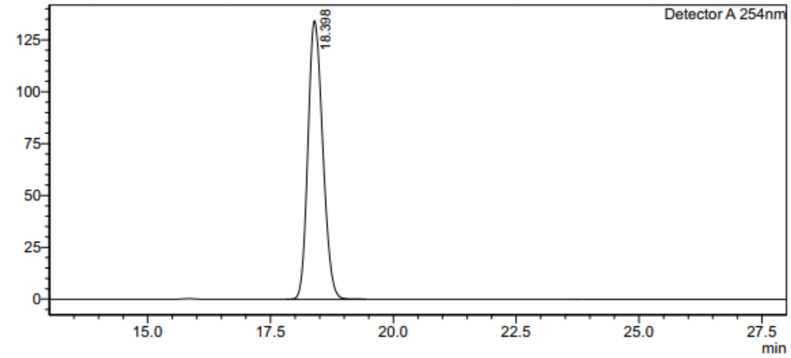

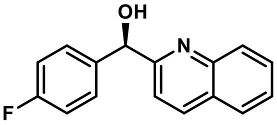

C11
> 99.9% ee

<Peak Table>

Detector A 254nm

Peak#	Ret. Time	Area	Height	Conc.	Unit	Mark	Name
1	18.398	2802299	134330	100.000			
Total		2802299	134330				

Supplementary Table 13. Crystal data and structure refinement for **C11**

CCDC Number	2017091
Empirical formula	$C_{16}H_{12}FNO$
Formula weight	253.27
Temperature/K	100.01
Crystal system	orthorhombic
Space group	$P2_12_12_1$
a/Å	5.9679(4)
b/Å	8.5208(6)
c/Å	24.1164(18)
α/°	90
β/°	90
γ/°	90
Volume/Å^3	1226.35(15)
Z	4
ρ_{calc}g/cm^3	1.372
μ/mm^{-1}	0.790
F(000)	528.0
Crystal size/mm^3	0.38 × 0.38 × 0.36
Radiation	CuKα (λ = 1.54178)
2Θ range for data collection/°	11.012 to 136.606
Index ranges	$-7 \le h \le 6$, $-10 \le k \le 9$, $-29 \le l \le 23$
Reflections collected	7243
Independent reflections	2227 [R_{int} = 0.0369, R_{sigma} = 0.0417]
Data/restraints/parameters	2227/0/174
Goodness-of-fit on F^2	1.167
Final R indexes [I>=2σ (I)]	R_1 = 0.0379, wR_2 = 0.0969
Final R indexes [all data]	R_1 = 0.0385, wR_2 = 0.0973
Largest diff. peak/hole / e Å^{-3}	0.21/-0.20
Flack parameter	0.11(14)

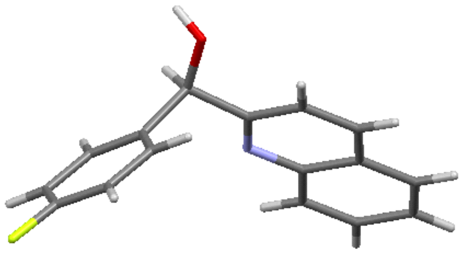

Supplementary Figure 12. The single crystal configuration of **C11** is demonstrated in a capped sticks mode..

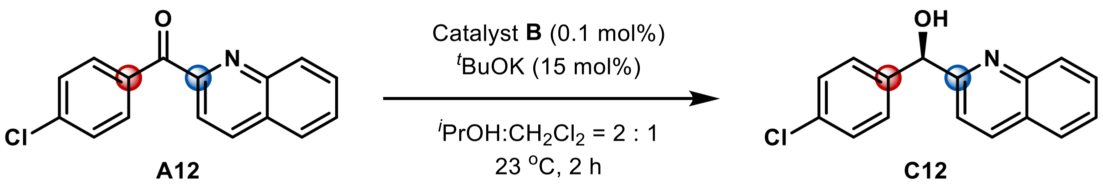

Following the general procedure, **C12** (44.7 mg, 83% yield, 81% ee) was obtained as white solid. Its major pure enantiomer was purified by chiral preparative HPLC (AD-H, 2.0*25 cm, 5μm) and the *X*-ray crystal of the optically pure enantiomer (100% ee) was obtained by liquid/liquid diffusion with CH_2Cl_2/hexane system.

^{1}H NMR (600 MHz, $CDCl_3$): δ 8.14 (d, J = 8.4 Hz, 1H), 8.07 (d, J = 8.4 Hz, 1H), 7.81 (d, J = 8.4 Hz, 1H), 7.77 (t, J = 7.6 Hz, 1H), 7.57 (t, J = 7.6 Hz, 1H), 7.36 (d, J = 8.4 Hz, 2H), 7.31 (d, J = 8.4 Hz, 2H), 7.15 (d, J = 8.4 Hz, 1H), 6.11 (s, 1H), 5.85 (s, 1H) ppm
^{13}C NMR (151 MHz, $CDCl_3$): δ 159.9, 146.0, 141.3, 137.2, 133.8, 130.0, 128.8, 128.8, 127.6, 127.5, 126.8, 119.0, 74.5 ppm
HRMS (ESI+): calculated for $C_{16}H_{13}ClNO$ $[M+H]^+$: 270.0680, found 270.0684.
HPLC (AD-H, 0.46*25 cm, 5μm, hexane / ethanol = 90/10, flow 1 mL/min, detection at 254 nm) retention time = 16.765 min (major) and 22.876 min (minor).

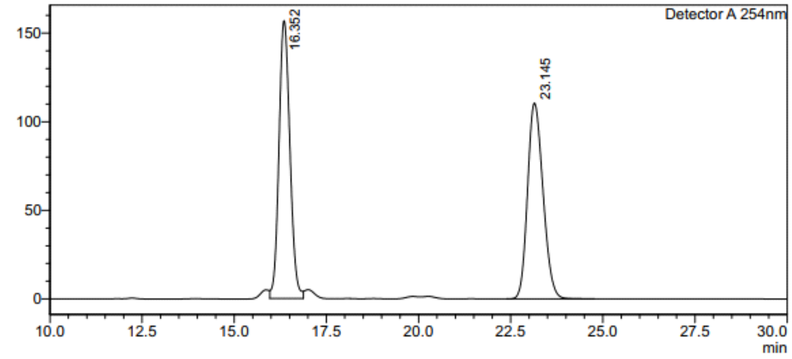

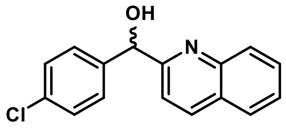

(±)-**C12**

<Peak Table>

Detector A 254nm

Peak#	Ret. Time	Area	Height	Conc.	Unit	Mark	Name
1	16.352	3238174	156807	50.042		M	
2	23.145	3232706	110501	49.958			
Total		6470880	267308				

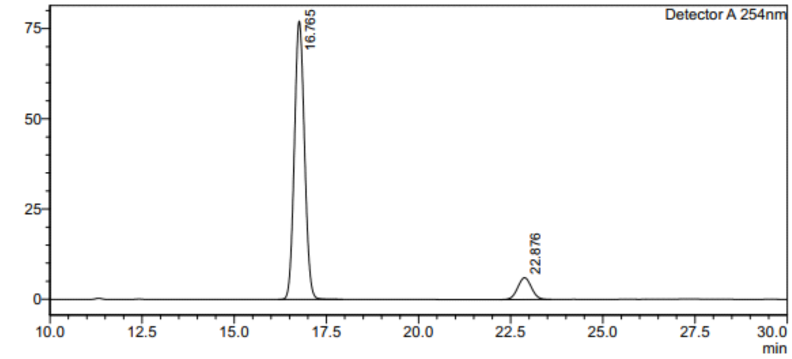

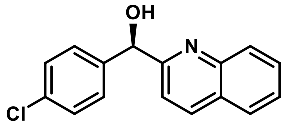

C12
83% yield, 81% ee
obtained by catalyst **B**

<Peak Table>

Detector A 254nm

Peak#	Ret. Time	Area	Height	Conc.	Unit	Mark	Name
1	16.765	1470499	77132	90.422			
2	22.876	155766	6037	9.578			
Total		1626265	83169				

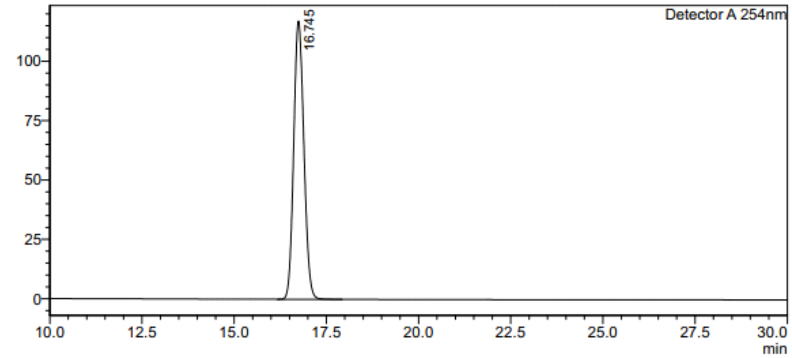

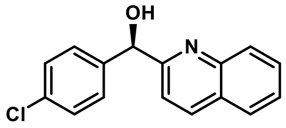

C12
> 99.9% ee

<Peak Table>

Detector A 254nm

Peak#	Ret. Time	Area	Height	Conc.	Unit	Mark	Name
1	16.745	2252821	117179	100.000			
Total		2252821	117179				

Supplementary Table 14. Crystal data and structure refinement for **C12**

CCDC Number	2017092
Empirical formula	$C_{16}H_{12}ClNO$
Formula weight	269.72
Temperature/K	100.0
Crystal system	orthorhombic
Space group	$P2_12_12_1$
a/Å	8.0730(3)
b/Å	12.2356(5)
c/Å	13.0445(6)
α/°	90
β/°	90
γ/°	90
Volume/Å^3	1288.51(9)
Z	4
ρ_{calc}g/cm^3	1.390
μ/mm^{-1}	0.286
F(000)	560.0
Crystal size/mm^3	0.45 × 0.42 × 0.29
Radiation	MoKα (λ = 0.71073)
2Θ range for data collection/°	5.934 to 56.586
Index ranges	$-10 \leq h \leq 8$, $-14 \leq k \leq 16$, $-17 \leq l \leq 17$
Reflections collected	16939
Independent reflections	3199 [R_{int} = 0.0345, R_{sigma} = 0.0231]
Data/restraints/parameters	3199/0/174
Goodness-of-fit on F^2	1.072
Final R indexes [I>=2σ (I)]	R_1 = 0.0260, wR_2 = 0.0641
Final R indexes [all data]	R_1 = 0.0283, wR_2 = 0.0653
Largest diff. peak/hole / e Å^{-3}	0.26/-0.18
Flack parameter	-0.041(17)

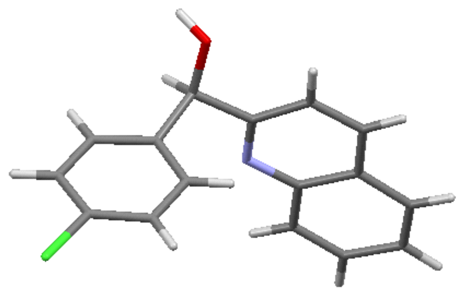

Supplementary Figure 13. The single crystal configuration of **C12** is demonstrated in a capped sticks mode.

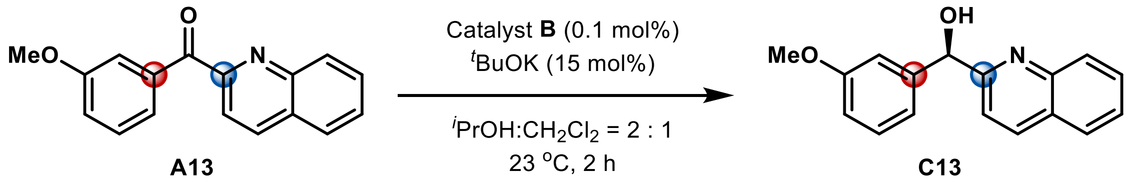

Following the general procedure, **C13** (44.5 mg, 84% yield, 84% ee) was obtained as white solid.

^{1}H NMR (600 MHz, CDCl$_3$): δ 8.06 (d, J = 8.4 Hz, 1H), 7.97 (d, J = 8.4 Hz, 1H), 7.72 (d, J = 8.2 Hz, 1H), 7.67 (t, J = 7.8 Hz, 1H), 7.47 (t, J = 7.4 Hz, 1H), 7.18 (t, J = 7.8 Hz, 1H), 7.13 (d, J = 8.4 Hz, 1H), 6.93 (d, J = 7.4 Hz, 1H), 6.88 (s, 1H), 6.75 (d, J = 8.2 Hz, 1H), 6.01 (s, 1H), 5.76 (s, 1H), 3.68 (s, 3H) ppm

^{13}C NMR (151 MHz, CDCl$_3$): δ 160.3, 159.8, 146.0, 144.3, 137.0, 129.9, 129.6, 128.8, 127.6, 127.5,126.6, 119.8, 119.2, 113.6, 112.8, 75.1, 55.2 ppm

HRMS (ESI+): calculated for $C_{17}H_{16}NO_2$ [M+H]$^+$: 266.1176, found 266.1178.

HPLC (AD-H, 0.46*25 cm, 5μm, hexane / ethanol = 80/20, flow 1 mL/min, detection at 254 nm) retention time = 15.552 min (major) and 24.790 min (minor).

The major enantiomer of **C13** (84% ee) was purified by chiral preparative HPLC (AD-H, 2.0*25 cm, 5μm). Because we were unable to grow *X*-ray qualified crystal of the optically pure **C13** (100% ee), we derivatize it with (1*S*)-(-)-camphanic acid chloride to afford **D13** as a single diastereomer. Then the *X*-ray crystal of the optically pure **D13** was obtained by liquid/liquid diffusion with CH_2Cl_2/hexane system.

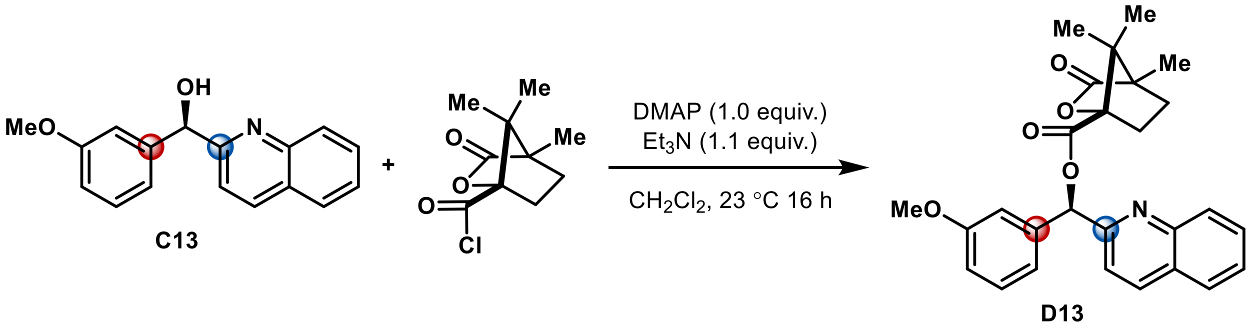

^{1}H NMR (600 MHz, CDCl$_3$): δ 8.13 (d, J = 8.5 Hz, 1H), 8.07 (d, J = 8.5 Hz, 1H), 7.78 (d, J = 8.2 Hz, 1H), 7.70 (t, J = 7.8 Hz, 1H), 7.55 – 7.50 (m, 2H), 7.26 (t, J = 7.8 Hz, 1H), 7.12 (d, J = 7.8 Hz, 1H), 7.10 (s, 1H), 7.07 (s, 1H), 6.83 (d, J = 8.2 Hz, 1H), 3.77 (s, 3H), 2.59 – 2.51 (m, 1H), 2.17 – 2.11 (m, 1H), 1.98 – 1.91 (m, 1H), 1.76 – 1.71 (m, 1H), 1.13 (s, 3H), 1.06 (s, 3H), 1.02 (s, 3H) ppm

^{13}C NMR (151 MHz, CDCl$_3$): δ 178.4, 166.5, 159.7, 158.2, 147.4, 139.5, 137.1, 129.8, 129.7, 129.4, 127.6, 127.5, 126.7, 119.4, 118.6, 113.8, 113.0, 91.1, 79.5, 55.2, 54.9, 54.4, 30.9, 28.9, 16.8, 16.7, 9.7 ppm

HRMS (ESI+): calculated for $C_{27}H_{28}NO_5$ [M+H]$^+$: 446.1962, found 446.1963.

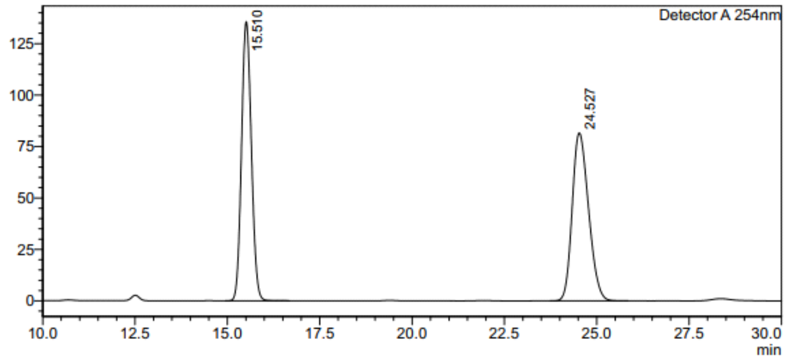

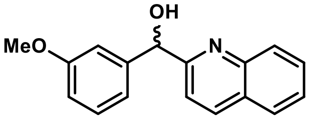

(±)-**C13**

<Peak Table>

Detector A 254nm

Peak#	Ret. Time	Area	Height	Conc.	Unit	Mark	Name
1	15.510	2508727	135650	49.936			
2	24.527	2515181	81594	50.064			
Total		5023908	217244				

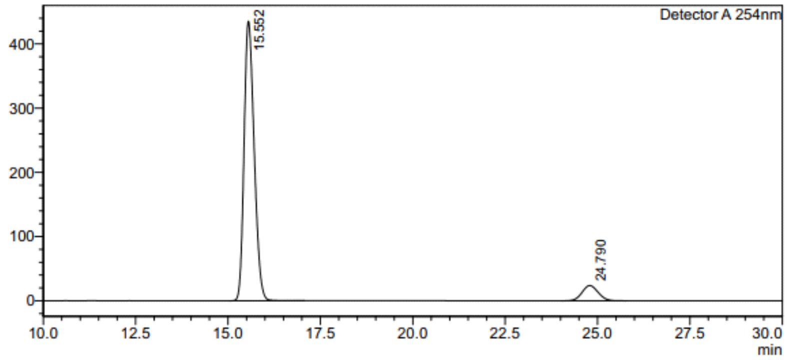

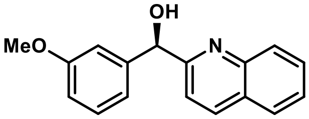

C13
84% yield, 84% ee
obtained by catalyst **B**

<Peak Table>

Detector A 254nm

Peak#	Ret. Time	Area	Height	Conc.	Unit	Mark	Name
1	15.552	8255579	435371	92.029			
2	24.790	715037	23691	7.971			
Total		8970617	459062				

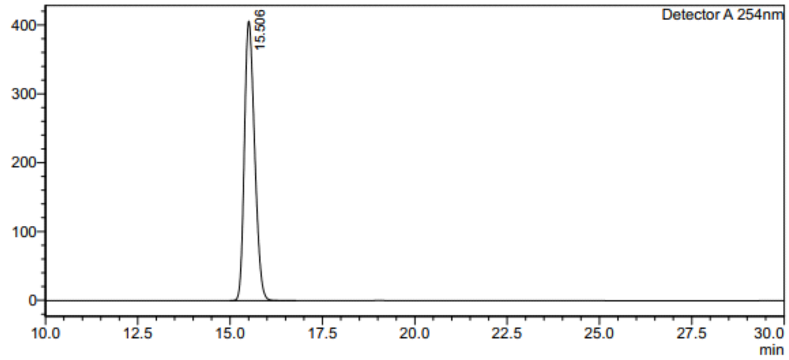

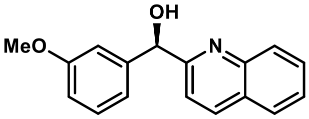

C13
> 99.9% ee

<Peak Table>

Detector A 254nm

Peak#	Ret. Time	Area	Height	Conc.	Unit	Mark	Name
1	15.506	7804654	406043	100.000			
Total		7804654	406043				

Supplementary Table 15. Crystal data and structure refinement for **D13**

CCDC Number	2017194
Empirical formula	$C_{27}H_{27}NO_5$
Formula weight	445.49
Temperature/K	100.04
Crystal system	hexagonal
Space group	$P6_1$
a/Å	10.8512(3)
b/Å	10.8512(3)
c/Å	33.8307(10)
α/°	90
β/°	90
γ/°	120
Volume/Å^3	3449.8(2)
Z	6
ρ_{calc}g/cm^3	1.287
μ/mm^{-1}	0.720
F(000)	1416.0
Crystal size/mm^3	0.39 × 0.26 × 0.25
Radiation	CuKα (λ = 1.54178)
2Θ range for data collection/°	9.41 to 138.202
Index ranges	$-13 \leq h \leq 13$, $-10 \leq k \leq 12$, $-40 \leq l \leq 40$
Reflections collected	71289
Independent reflections	4268 [R_{int} = 0.0745, R_{sigma} = 0.0207]
Data/restraints/parameters	4268/1/303
Goodness-of-fit on F^2	1.048
Final R indexes [I>=2σ (I)]	R_1 = 0.0215, wR_2 = 0.0545
Final R indexes [all data]	R_1 = 0.0231, wR_2 = 0.0549
Largest diff. peak/hole / e Å^{-3}	0.16/-0.11
Flack parameter	-0.03(3)

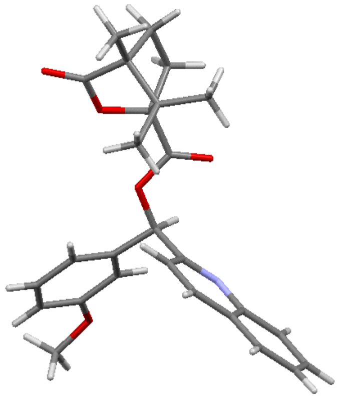

Supplementary Figure 14. The single crystal configuration of **D13** is demonstrated in a capped sticks mode.

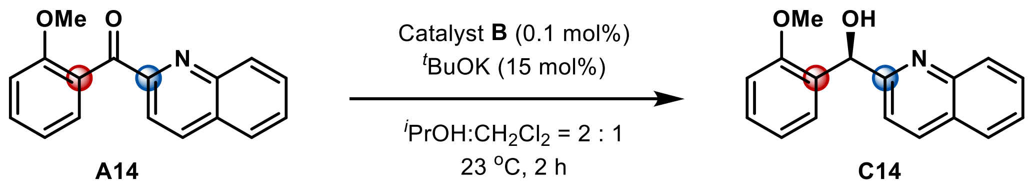

Following the general procedure, **C14** (40.3 mg, 76% yield, 91% ee) was obtained as white solid. Its major pure enantiomer was purified by chiral preparative HPLC (OD-H, 2.0*25 cm, 5μm) and the *X*-ray crystal of the optically pure enantiomer (100% ee) was obtained by liquid/liquid diffusion with CH_2Cl_2/hexane system.

^{1}H NMR (600 MHz, $CDCl_3$): δ 8.13 (d, J = 8.4 Hz, 1H), 8.03 (d, J = 8.4 Hz, 1H), 7.79 (d, J = 8.2 Hz, 1H), 7.73 (t, J = 7.4 Hz, 1H), 7.53 (t, J = 7.4 Hz, 1H), 7.33 (d, J = 8.4 Hz, 1H), 7.28 (d, J = 7.6 Hz, 1H), 7.25 (t, J = 7.6 Hz, 1H), 6.95 (d, J = 8.2 Hz, 1H), 6.91 (t, J = 7.6 Hz, 1H), 6.40 (s, 1H), 6.00 (s, 1H), 3.92 (s, 3H) ppm
^{13}C NMR (151 MHz, $CDCl_3$): δ 161.1, 156.8, 146.0, 136.8, 131.3, 129.6, 128.8, 128.8, 128.2, 127.5, 127.5, 126.3, 121.0, 119.3, 110.8, 68.6, 55.6 ppm
HRMS (ESI+): calculated for $C_{17}H_{16}NO_2$ $[M+H]^+$: 266.1176, found 266.1179.
HPLC (OD-H, 0.46*25 cm, 5μm, hexane / ethanol = 80/20, flow 1 mL/min, detection at 254 nm) retention time = 6.460 min (minor) and 8.078 min (major).

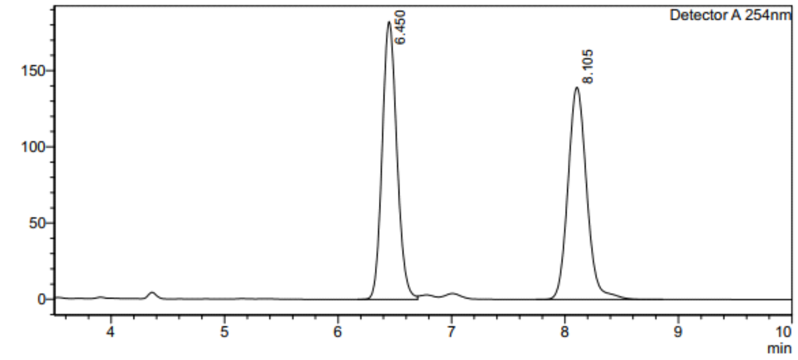

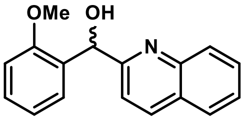

(±)-**C14**

<Peak Table>

Detector A 254nm

Peak#	Ret. Time	Area	Height	Conc.	Unit	Mark	Name
1	6.450	1640648	182250	50.692		V	
2	8.105	1595846	139103	49.308			
Total		3236495	321353				

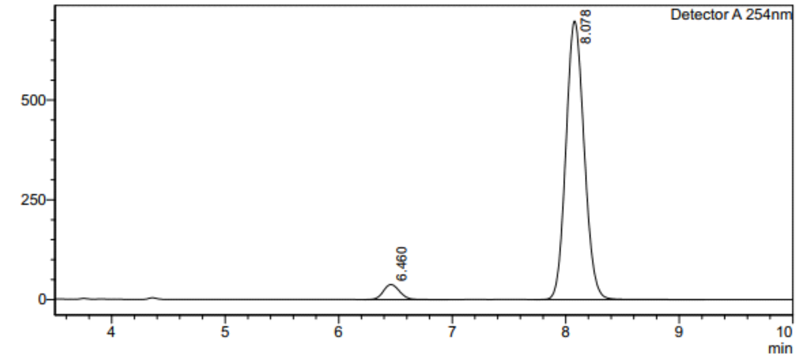

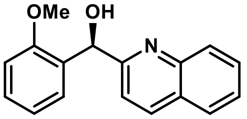

C14
76% yield, 91% ee
obtained by catalyst **B**

<Peak Table>

Detector A 254nm

Peak#	Ret. Time	Area	Height	Conc.	Unit	Mark	Name
1	6.460	371570	37526	4.552		V	
2	8.078	7790430	698009	95.448			
Total		8162001	735535				

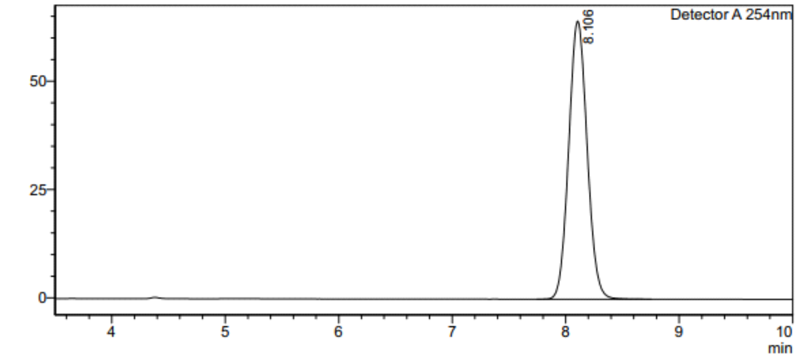

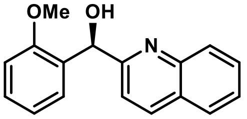

C14
> 99.9% ee

<Peak Table>

Detector A 254nm

Peak#	Ret. Time	Area	Height	Conc.	Unit	Mark	Name
1	8.106	724999	64185	100.000			
Total		724999	64185				

Supplementary Table 16. Crystal data and structure refinement for **C14**

CCDC Number	2017096
Empirical formula	$C_{17}H_{15}NO_2$
Formula weight	265.30
Temperature/K	100.01
Crystal system	orthorhombic
Space group	$P2_12_12_1$
a/Å	7.7162(4)
b/Å	8.2194(4)
c/Å	20.2800(10)
α/°	90
β/°	90
γ/°	90
Volume/Å^3	1286.21(11)
Z	4
ρ_{calc}g/cm^3	1.370
μ/mm^{-1}	0.721
F(000)	560.0
Crystal size/mm^3	0.42 × 0.31 × 0.26
Radiation	CuKα (λ = 1.54178)
2Θ range for data collection/°	8.72 to 136.8
Index ranges	$-9 \le h \le 9$, $-9 \le k \le 9$, $-24 \le l \le 24$
Reflections collected	14209
Independent reflections	2359 [R_{int} = 0.0278, R_{sigma} = 0.0183]
Data/restraints/parameters	2359/0/184
Goodness-of-fit on F^2	1.079
Final R indexes [I>=2σ (I)]	R_1 = 0.0221, wR_2 = 0.0566
Final R indexes [all data]	R_1 = 0.0223, wR_2 = 0.0567
Largest diff. peak/hole / e Å^{-3}	0.15/-0.12
Flack parameter	-0.01(5)

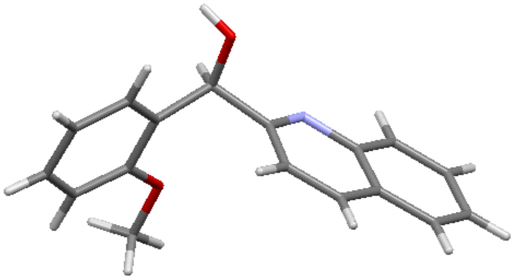

Supplementary Figure 15. The single crystal configuration of **C14** is demonstrated in a capped sticks mode.

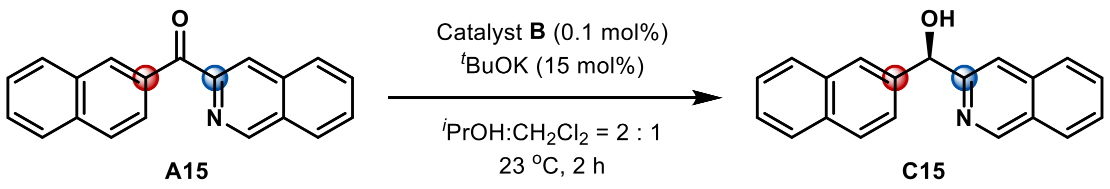

Following the general procedure, **C15** (44.0 mg, 77% yield, 93% ee) was obtained as white solid. Its major pure enantiomer was purified by chiral preparative HPLC (OD-H, 2.0*25 cm, 5μm) and the *X*-ray crystal of the optically pure enantiomer (100% ee) was obtained by liquid/liquid diffusion with CH_2Cl_2/hexane system.

^{1}H NMR (600 MHz, $CDCl_3$): δ 9.25 (s, 1H), 7.98 (d, *J* = 7.4 Hz, 1H), δ 7.98 (s, 1H)., 7.86 (d, *J* = 8.2 Hz, 1H), 7.81 (d, *J* = 8.2 Hz, 2H), 7.73 (d, *J* = 8.4 Hz, 1H), 7.66 (t, *J* = 7.5 Hz, 1H), 7.58 (t, *J* = 7.5 Hz, 1H), 7.57 (s, 1H), 7.53 (d, *J* = 8.4 Hz, 1H), 7.50 – 7.44 (m, 2H), 6.12 (s, 1H), 4.84 (s, 1H) ppm

^{13}C NMR (151 MHz, $CDCl_3$): δ 154.6, 151.5, 140.8, 136.4, 133.3, 133.1, 130.7, 128.4, 128.1, 128.0, 127.7, 127.6, 127.2, 126.7, 126.1, 126.0, 126.0, 124.9, 117.5, 75.6 ppm

HRMS (ESI+): calculated for $C_{20}H_{16}NO$ $[M+H]^+$: 286.1226, found 286.1230.

HPLC (OD-H, 0.46*25 cm, 5μm, hexane / ethanol = 80/20, flow 1 mL/min, detection at 254 nm) retention time = 7.948 min (minor) and 11.124 min (major).

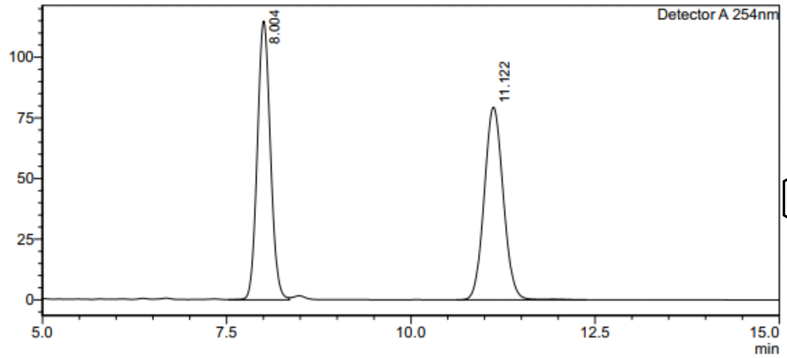

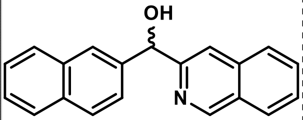

(±)-**C15**

<Peak Table>

Detector A 254nm

Peak#	Ret. Time	Area	Height	Conc.	Unit	Mark	Name
1	8.004	1423595	114906	49.863			
2	11.122	1431403	79448	50.137			
Total		2854998	194354				

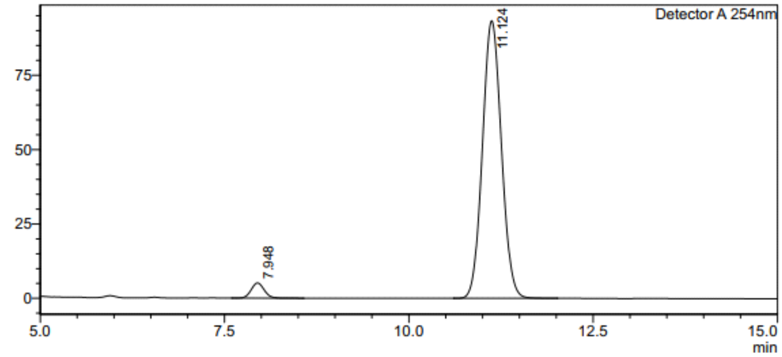

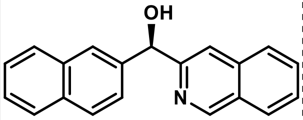

C15
77% yield, 93% ee
obtained by catalyst **B**

<Peak Table>

Detector A 254nm

Peak#	Ret. Time	Area	Height	Conc.	Unit	Mark	Name
1	7.948	63715	5105	3.664		S	
2	11.124	1675131	93318	96.336			
Total		1738846	98422				

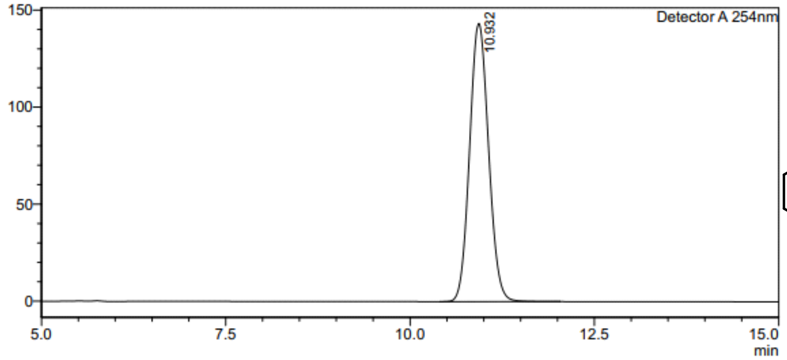

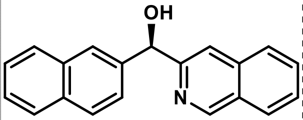

C15
> 99.9% ee

<Peak Table>

Detector A 254nm

Peak#	Ret. Time	Area	Height	Conc.	Unit	Mark	Name
1	10.932	2562503	143406	100.000			
Total		2562503	143406				

Supplementary Table 17. Crystal data and structure refinement for **C15**

CCDC Number	2017134
Empirical formula	$C_{20}H_{15}NO$
Formula weight	285.33
Temperature/K	100
Crystal system	orthorhombic
Space group	$P2_12_12_1$
a/Å	7.9077(8)
b/Å	11.8952(12)
c/Å	14.8962(15)
α/°	90
β/°	90
γ/°	90
Volume/Å^3	1401.2(2)
Z	4
ρ_{calc}g/cm^3	1.353
μ/mm^{-1}	0.652
F(000)	600.0
Crystal size/mm^3	0.35 × 0.31 × 0.26
Radiation	CuKα (λ = 1.54178)
2Θ range for data collection/°	11.882 to 137.06
Index ranges	$-9 \le h \le 9$, $-13 \le k \le 14$, $-17 \le l \le 17$
Reflections collected	14593
Independent reflections	2560 [R_{int} = 0.0438, R_{sigma} = 0.0293]
Data/restraints/parameters	2560/0/201
Goodness-of-fit on F^2	1.067
Final R indexes [I>=2σ (I)]	R_1 = 0.0278, wR_2 = 0.0697
Final R indexes [all data]	R_1 = 0.0284, wR_2 = 0.0704
Largest diff. peak/hole / e Å^{-3}	0.13/-0.12
Flack parameter	0.10(12)

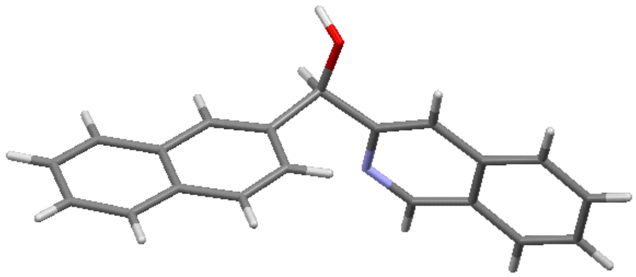

Supplementary Figure 16. The single crystal configuration of **C15** is demonstrated in a capped sticks mode.

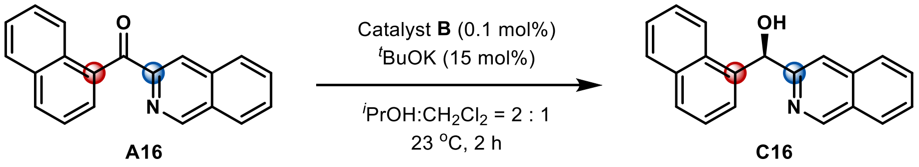

Following the general procedure, **C16** (44.1 mg, 78% yield, 93% ee) was obtained as white solid. Its major pure enantiomer was purified by chiral preparative HPLC (OD-H, 2.0*25 cm, 5μm) and the *X*-ray crystal of the optically pure enantiomer (100% ee) was obtained by liquid/liquid diffusion with CH_2Cl_2/hexane system.

^{1}H NMR (600 MHz, $CDCl_3$): δ 9.31 (s, 1H), 8.17 (d, J = 8.2 Hz, 1H), 7.99 (d, J = 8.2 Hz, 1H), 7.88 (d, J = 8.2 Hz, 1H), 7.85 (d, J = 8.2 Hz, 1H), 7.66 – 7.55 (m, 4H), 7.52 – 7.40 (m, 4H), 6.65 (s, 1H), 4.86 (s, 1H) ppm

^{13}C NMR (151 MHz, $CDCl_3$): δ 154.7, 151.4, 138.4, 136.4, 134.1, 131.2, 130.6, 128.7, 128.7, 127.9, 127.6, 127.2, 126.8, 126.1, 125.8, 125.5, 125.4, 124.4, 117.6, 73.7 ppm

HRMS (ESI+): calculated for $C_{20}H_{16}NO$ $[M+H]^+$: 286.1226, found 286.1230.

HPLC (OD-H, 0.46*25 cm, 5μm, hexane / ethanol = 90/10, flow 1 mL/min, detection at 254 nm) retention time = 15.362 min (minor) and 17.612 min (major).

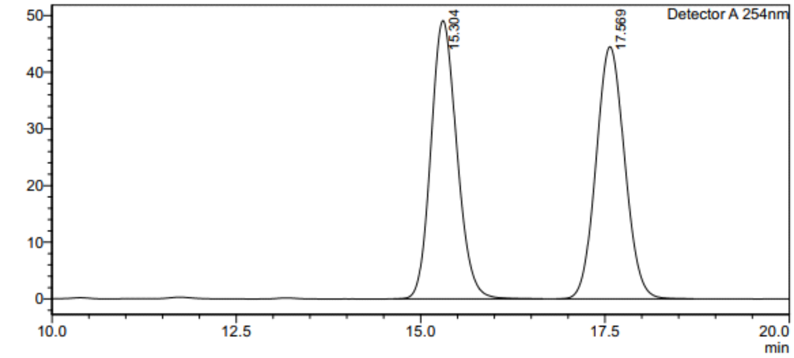

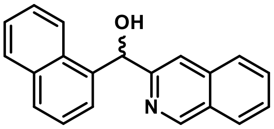

(±)-**C16**

<Peak Table>

Detector A 254nm

Peak#	Ret. Time	Area	Height	Conc.	Unit	Mark	Name
1	15.304	1203125	49088	50.028			
2	17.569	1201764	44502	49.972			
Total		2404889	93589				

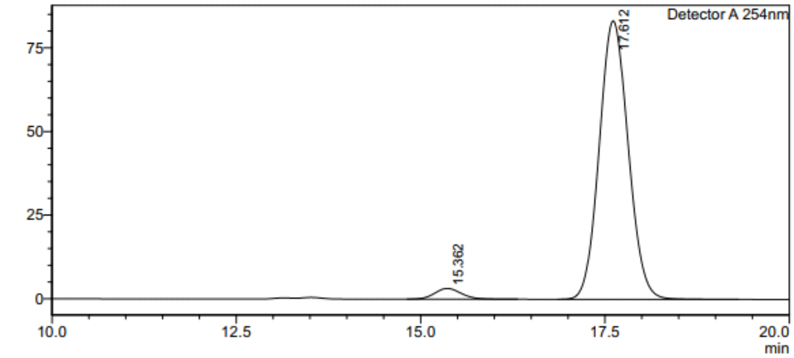

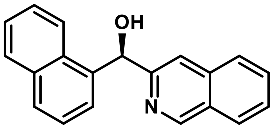

C16
78% yield, 93% ee
obtained by catalyst **B**

<Peak Table>

Detector A 254nm

Peak#	Ret. Time	Area	Height	Conc.	Unit	Mark	Name
1	15.362	80164	3189	3.401			
2	17.612	2276875	83254	96.599			
Total		2357039	86444				

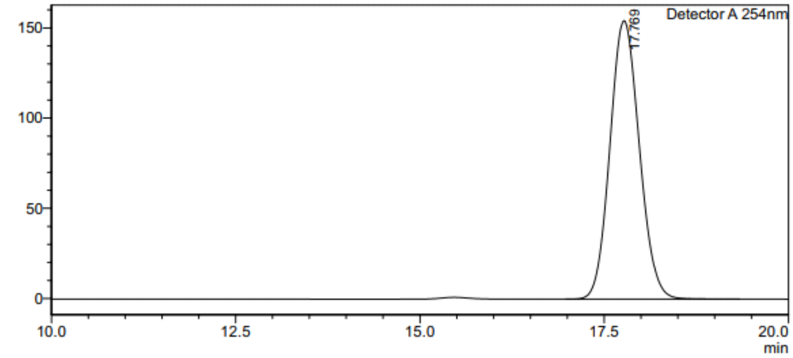

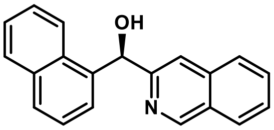

C16
> 99.9% ee

<Peak Table>

Detector A 254nm

Peak#	Ret. Time	Area	Height	Conc.	Unit	Mark	Name
1	17.769	4281195	154347	100.000			
Total		4281195	154347				

Supplementary Table 18. Crystal data and structure refinement for **C16**

CCDC Number	2017106
Empirical formula	$C_{20}H_{15}NO$
Formula weight	285.33
Temperature/K	100
Crystal system	orthorhombic
Space group	$P2_12_12_1$
a/Å	8.3540(9)
b/Å	11.3460(12)
c/Å	15.0481(16)
α/°	90
β/°	90
γ/°	90
Volume/Å^3	1426.3(3)
Z	4
ρ_{calc}g/cm^3	1.329
μ/mm^{-1}	0.641
F(000)	600.0
Crystal size/mm^3	0.37 × 0.37 × 0.35
Radiation	CuKα (λ = 1.54178)
2Θ range for data collection/°	11.762 to 137.162
Index ranges	$-10 \leq h \leq 10$, $-13 \leq k \leq 13$, $-18 \leq l \leq 17$
Reflections collected	11951
Independent reflections	2593 [R_{int} = 0.0420, R_{sigma} = 0.0338]
Data/restraints/parameters	2593/0/201
Goodness-of-fit on F^2	1.067
Final R indexes [I>=2σ (I)]	R_1 = 0.0291, wR_2 = 0.0730
Final R indexes [all data]	R_1 = 0.0294, wR_2 = 0.0733
Largest diff. peak/hole / e Å^{-3}	0.16/-0.12
Flack parameter	-0.07(12)

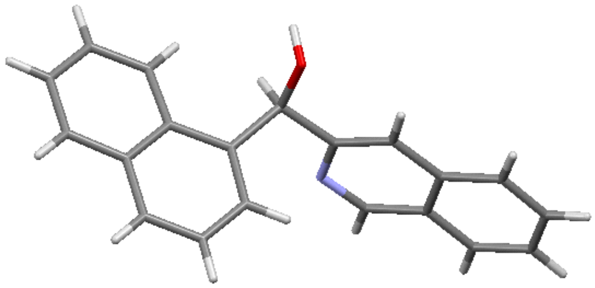

Supplementary Figure 17. The single crystal configuration of **C16** is demonstrated in a capped sticks mode..

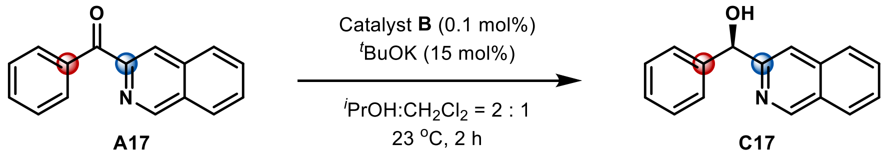

Following the general procedure, **C17** (45.2 mg, 96% yield, 91% ee) was obtained as white solid.

^{1}H NMR (600 MHz, $CDCl_3$): δ 9.22 (s, 1H), 7.96 (d, *J* = 8.2 Hz, 1H), 7.75 (d, *J* = 8.2 Hz, 1H), 7.67 (t, *J* = 7.5 Hz, 1H), 7.57 (t, *J* = 7.5 Hz, 1H), 7.57 (s, 1H), 7.47 (d, *J* = 7.4 Hz, 2H), 7.36 (t, *J* = 7.4 Hz, 2H), 7.29 (t, *J* = 7.4 Hz, 1H), 5.97 (s, 1H), 4.77 (s, 1H) ppm
^{13}C NMR (151 MHz, $CDCl_3$): δ 154.8, 151.5, 143.4, 136.3, 130.7, 128.5, 127.9, 127.7, 127.6, 127.1, 127.0, 126.7, 117.2, 75.5 ppm
HRMS (ESI+): calculated for $C_{16}H_{14}NO$ $[M+H]^+$: 236.1070, found 236.1073.
HPLC (OD-H, 0.46*25 cm, 5μm, hexane / ethanol = 90/10, flow 1 mL/min, detection at 254 nm) retention time = 8.992 min (minor) and 16.604 min (major).

The major enantiomer of **C17** (91% ee) was purified by chiral preparative HPLC (OD-H, 2.0*25 cm, 5μm). Because we were unable to grow *X*-ray qualified crystal of the optically pure **C17** (100% ee), we derivatize it with (1*S*)-(-)-camphanic acid chloride to afford **D17** as a single diastereomer. Then the *X*-ray crystal of the optically pure **D17** was obtained by liquid/liquid diffusion with CH_2Cl_2/hexane system.

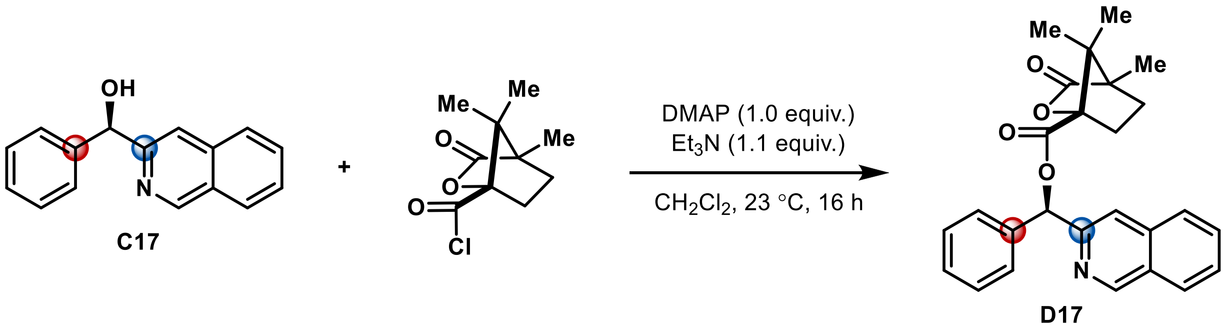

^{1}H NMR (600 MHz, $CDCl_3$): δ 9.20 (s, 1H), 7.94 (d, *J* = 8.2 Hz, 1H), 7.83 (d, *J* = 8.2 Hz, 1H), 7.81 (s, 1H), 7.70 (t, *J* = 7.6 Hz, 1H), 7.60 (t, *J* = 7.6 Hz, 1H), 7.53 (d, *J* = 7.8 Hz, 2H), 7.36 (t, *J* = 7.5 Hz, 2H), 7.30 (t, *J* = 7.5 Hz, 1H), 7.20 (s, 1H), 2.56 – 2.47 (m, 1H), 2.13– 2.08 (m, 1H), 1.98 – 1.91 (m, 1H), 1.74 – 1.69 (m, 1H), 1.12 (s, 3H), 1.05 (s, 3H), 0.96 (s, 3H) ppm
^{13}C NMR (151 MHz, $CDCl_3$): δ 178.4, 166.6, 152.6, 152.0, 138.5, 136.1, 130.7, 128.6, 128.4, 128.0, 127.6, 127.6, 126.9, 117.3, 91.1, 78.9, 54.9, 54.5, 30.8, 29.0, 16.8, 16.7, 9.7 ppm
HRMS (ESI+): calculated for $C_{26}H_{26}NO_4$ $[M+H]^+$: 416.1856, found 416.1853.

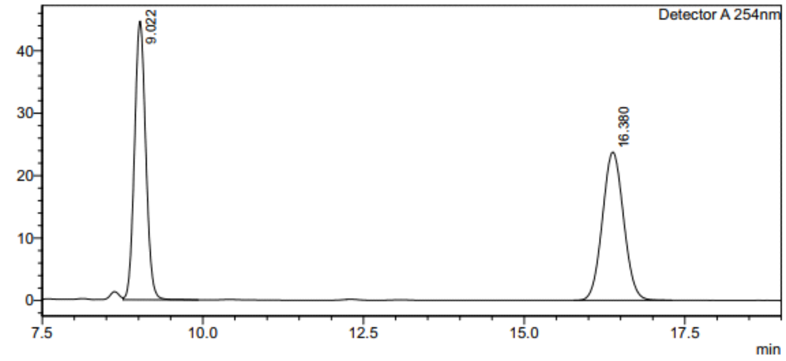

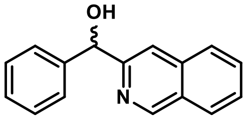

(±)-**C17**

<Peak Table>

Detector A 254nm

Peak#	Ret. Time	Area	Height	Conc.	Unit	Mark	Name
1	9.022	543463	44591	49.949		S	
2	16.380	544569	23715	50.051			
Total		1088032	68306				

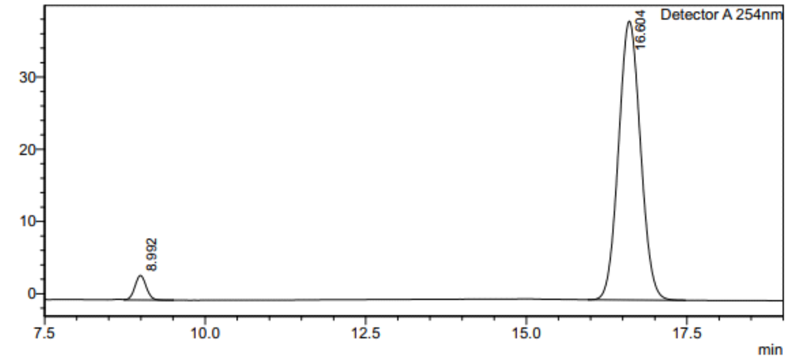

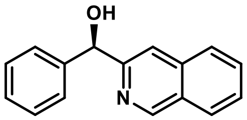

C17
96% yield, 91% ee
obtained by catalyst **B**

<Peak Table>

Detector A 254nm

Peak#	Ret. Time	Area	Height	Conc.	Unit	Mark	Name
1	8.992	41581	3398	4.332		V	
2	16.604	918239	38600	95.668			
Total		959820	41997				

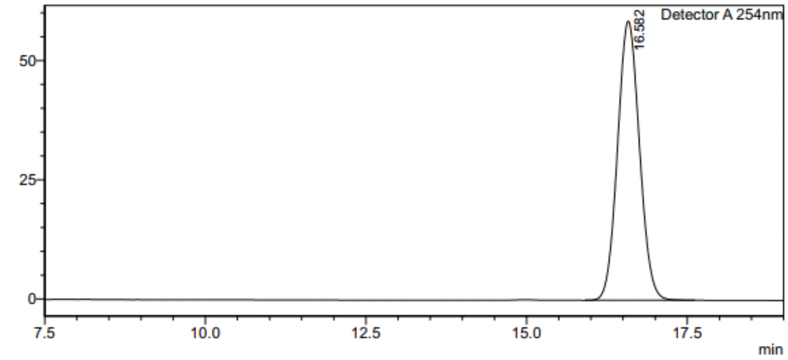

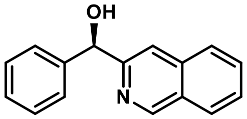

C17
> 99.9% ee

<Peak Table>

Detector A 254nm

Peak#	Ret. Time	Area	Height	Conc.	Unit	Mark	Name
1	16.582	1389090	58550	100.000			
Total		1389090	58550				

Supplementary Table 19. Crystal data and structure refinement for **D17**

CCDC Number	2017196
Empirical formula	$C_{26}H_{25}NO_4$
Formula weight	415.47
Temperature/K	100
Crystal system	monoclinic
Space group	$P2_1$
a/Å	17.5745(7)
b/Å	6.7175(3)
c/Å	18.3732(7)
α/°	90
β/°	93.474(2)
γ/°	90
Volume/Å^3	2165.09(15)
Z	4
ρ_{calc}g/cm^3	1.275
μ/mm^{-1}	0.691
F(000)	880.0
Crystal size/mm^3	0.41 × 0.22 × 0.15
Radiation	CuKα (λ = 1.54178)
2Θ range for data collection/°	4.818 to 136.694
Index ranges	$-21 \leq h \leq 21$, $-8 \leq k \leq 8$, $-21 \leq l \leq 17$
Reflections collected	29467
Independent reflections	7820 [R_{int} = 0.0392, R_{sigma} = 0.0361]
Data/restraints/parameters	7820/1/566
Goodness-of-fit on F^2	1.033
Final R indexes [I>=2σ (I)]	R_1 = 0.0338, wR_2 = 0.0879
Final R indexes [all data]	R_1 = 0.0349, wR_2 = 0.0891
Largest diff. peak/hole / e Å^{-3}	0.15/-0.17
Flack parameter	-0.09(12)

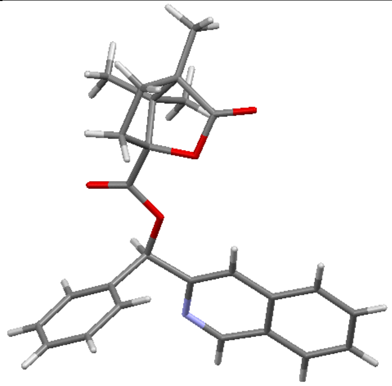

Supplementary Figure 18. The single crystal configuration of **D17** is demonstrated in a capped sticks mode.

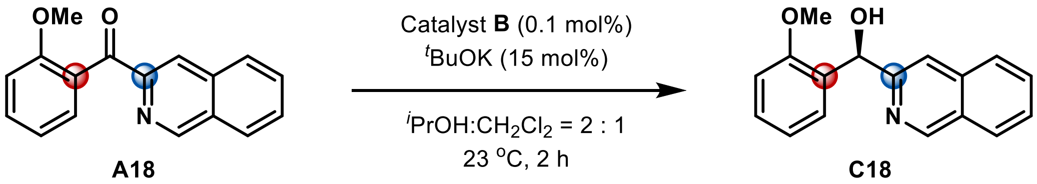

Following the general procedure, **C18** (49.3mg, 93% yield, 96% ee) was obtained as white solid.

^{1}H NMR (600 MHz, CDCl$_3$): δ 9.14 (s, 1H), 7.87 (d, *J* = 8.0 Hz, 1H), 7.68 (d, *J* = 8.2 Hz, 1H), 7.59 (s, 1H), 7.57 (t, *J* = 7.5 Hz, 1H), 7.48 (t, *J* = 7.5 Hz, 1H), 7.28 (d, *J* = 8.0 Hz, 1H), 7.18 (t, *J* = 7.5 Hz, 1H), 6.87 (t, *J* = 7.5 Hz, 1H), 6.84 (d, *J* = 8.2 Hz, 1H), 6.29 (s, 1H), 4.62 (s, 1H), 3.79 (s, 3H) ppm
^{13}C NMR (151 MHz, CDCl$_3$): δ 156.7, 154.8, 151.3, 136.4, 131.7, 130.4, 128.6, 127.8, 127.8, 127.5, 126.8, 126.7, 120.8, 117.0, 110.6, 70.1, 55.5 ppm
HRMS (ESI+): calculated for $C_{17}H_{16}NO_2$ [M+H]$^+$: 266.1176, found 266.1179.
HPLC (AD-H, 0.46*25 cm, 5μm, hexane / ethanol = 80/20, flow 1 mL/min, detection at 254 nm) retention time = 17.948 min (major) and 27.458 min (minor).

The major enantiomer of **C18** (96% ee) was purified by chiral preparative HPLC (AD-H, 2.0*25 cm, 5μm). Because we were unable to grow *X*-ray qualified crystal of the optically pure **C18** (100% ee), we derivatize it with (1*S*)-(-)-camphanic acid chloride to afford **D18** as a single diastereomer. Then the *X*-ray crystal of the optically pure **D18** was obtained by liquid/liquid diffusion with CH_2Cl_2/hexane system.

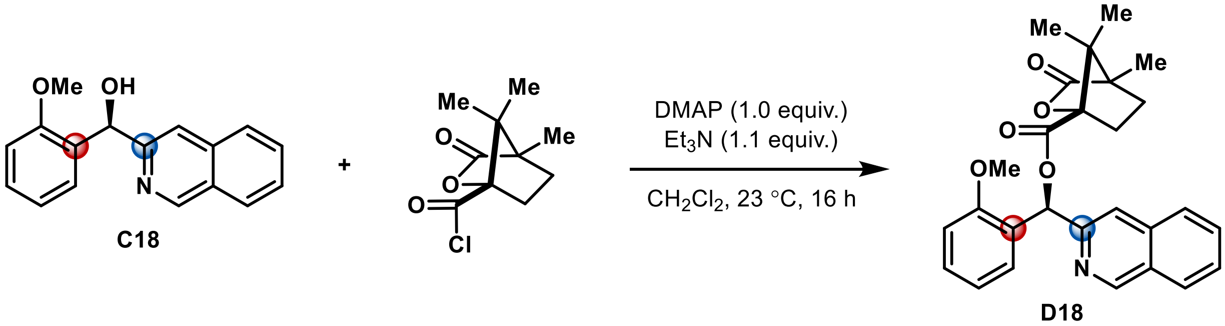

^{1}H NMR (600 MHz, CDCl$_3$): δ 9.21 (s, 1H), 7.94 (d, *J* = 8.2 Hz, 1H), 7.80 (d, *J* = 8.2 Hz, 1H), 7.71 (s, 1H), 7.68 (t, *J* = 7.5 Hz, 1H), 7.60 (s, 1H), δ 7.59 (t, *J* = 7.5 Hz, 1H), 7.40 (d, *J* = 7.6 Hz, 1H), 7.31 (t, *J* = 7.8 Hz, 1H), 6.95 (t, *J* = 7.8 Hz, 1H), 6.91 (d, *J* = 8.4 Hz, 1H), 3.84 (s, 3H), 2.56 – 2.51 (m, 1H), 2.11 – 2.07 (m, 1H), 1.95 – 1.90 (m, 1H), 1.72 – 1.67 (m, 1H), 1.11 (s, 3H), 1.04 (s, 3H), 0.98 (s, 3H) ppm
^{13}C NMR (151 MHz, CDCl$_3$): δ 178.4, 166.5, 156.9, 152.4, 151.5, 136.0, 130.5, 129.6, 128.7, 127.9, 127.5, 127.4, 126.8, 126.7, 120.7, 118.2, 110.7, 91.3, 73.4, 55.6, 54.9, 54.4, 30.7, 29.0, 16.7, 16.6, 9.7 ppm
HRMS (ESI+): calculated for $C_{27}H_{28}NO_5$ [M+H]$^+$: 446.1962, found 446.1960.

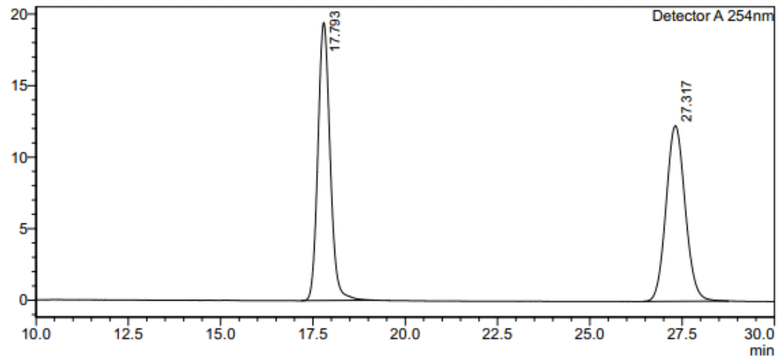

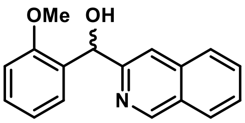

(±)-**C18**

<Peak Table>

Detector A 254nm

Peak#	Ret. Time	Area	Height	Conc.	Unit	Mark	Name
1	17.793	445755	19435	50.189			
2	27.317	442400	12272	49.811			
Total		888155	31707				

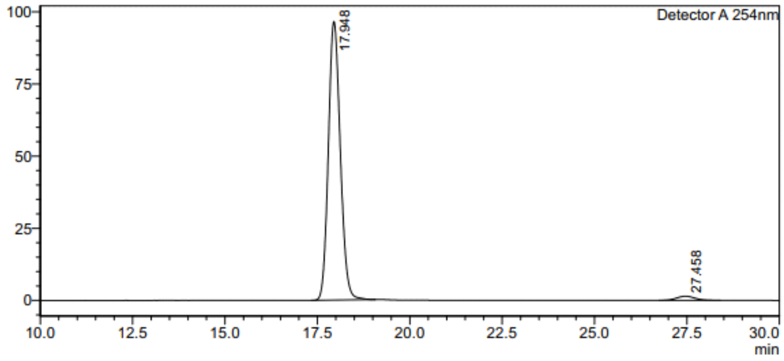

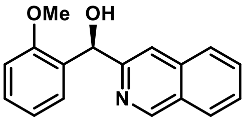

C18
93% yield, 96% ee
obtained by catalyst **B**

<Peak Table>

Detector A 254nm

Peak#	Ret. Time	Area	Height	Conc.	Unit	Mark	Name
1	17.948	2171288	96525	97.767			
2	27.458	49604	1450	2.233			
Total		2220892	97975				

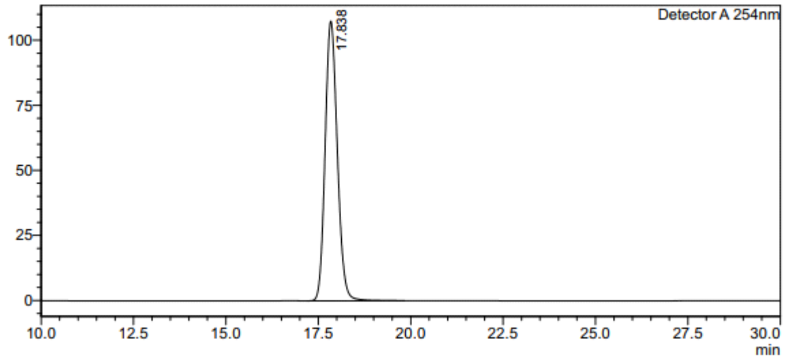

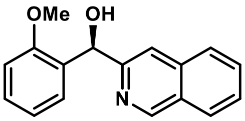

C18
> 99.9% ee

<Peak Table>

Detector A 254nm

Peak#	Ret. Time	Area	Height	Conc.	Unit	Mark	Name
1	17.838	2466021	107489	100.000			
Total		2466021	107489				

Supplementary Table 20. Crystal data and structure refinement for **D18**

CCDC Number	2017157
Empirical formula	$C_{27}H_{27}NO_5$
Formula weight	445.49
Temperature/K	100
Crystal system	orthorhombic
Space group	$P2_12_12_1$
a/Å	7.5843(2)
b/Å	16.3462(5)
c/Å	18.6209(6)
α/°	90
β/°	90
γ/°	90
Volume/Å^3	2308.52(12)
Z	4
ρ_{calc}g/cm^3	1.282
μ/mm^{-1}	0.717
F(000)	944.0
Crystal size/mm^3	0.32 × 0.19 × 0.18
Radiation	CuKα (λ = 1.54178)
2Θ range for data collection/°	7.196 to 137.264
Index ranges	$-9 \leq h \leq 7$, $-19 \leq k \leq 15$, $-19 \leq l \leq 22$
Reflections collected	20029
Independent reflections	4236 [R_{int} = 0.0445, R_{sigma} = 0.0313]
Data/restraints/parameters	4236/0/303
Goodness-of-fit on F^2	1.076
Final R indexes [I>=2σ (I)]	R_1 = 0.0311, wR_2 = 0.0781
Final R indexes [all data]	R_1 = 0.0317, wR_2 = 0.0785
Largest diff. peak/hole / e Å^{-3}	0.27/-0.26
Flack parameter	-0.05(7)

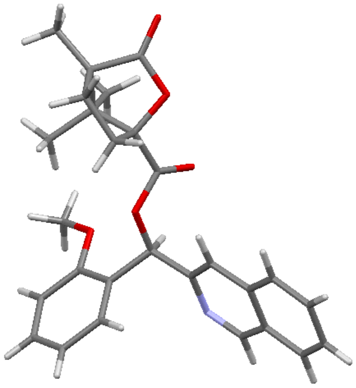

Supplementary Figure 19. The single crystal configuration of **D18** is demonstrated in a capped sticks mode.

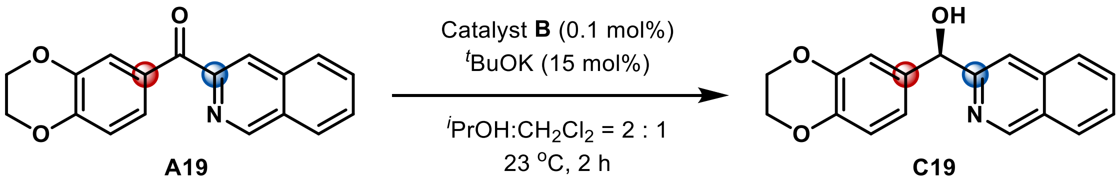

Following the general procedure, **C19** (51.0 mg, 87% yield, 87% ee) was obtained as white solid.

^{1}H NMR (600 MHz, CDCl$_3$): δ 9.22 (s, 1H), 7.96 (d, *J* = 8.2 Hz, 1H), 7.77 (d, *J* = 8.2 Hz, 1H), 7.67 (t, *J* = 7.5 Hz, 1H), 7.58 (t, *J* = 7.5 Hz, 1H), 7.58 (s, 1H), 6.94 (s, 1H), 6.93 (d, *J* = 8.4 Hz, 1H), 6.84 (d, *J* = 8.4 Hz, 1H), 5.85 (s, 1H), 4.58 (s, 1H), 4.22 (s, 2H) , 4.22 (s, 2H) ppm
^{13}C NMR (151 MHz, CDCl$_3$): δ 155.0, 151.4, 143.4, 143.1, 136.9, 136.3, 130.6, 127.8, 127.6, 127.0, 126.7, 120.1, 117.2, 117.0, 116.0, 75.0, 64.3, 64.2 ppm
HRMS (ESI+): calculated for $C_{18}H_{16}NO_3$ [M+H]$^+$: 294.1130, found 294.1129.
HPLC (OD-H, 0.46*25 cm, 5μm, hexane / ethanol = 85/15, flow 1 mL/min, detection at 254 nm) retention time = 12.321 min (minor) and 18.977 min (major).

The major enantiomer of **C19** (87% ee) was purified by chiral preparative HPLC (OD-H, 2.0*25 cm, 5μm). Because we were unable to grow *X*-ray qualified crystal of the optically pure **C19** (100% ee), we derivatize it with (1*S*)-(-)-camphanic acid chloride to afford **D19** as a single diastereomer. Then the *X*-ray crystal of the optically pure **D19** was obtained by liquid/liquid diffusion with CH_2Cl_2/hexane system.

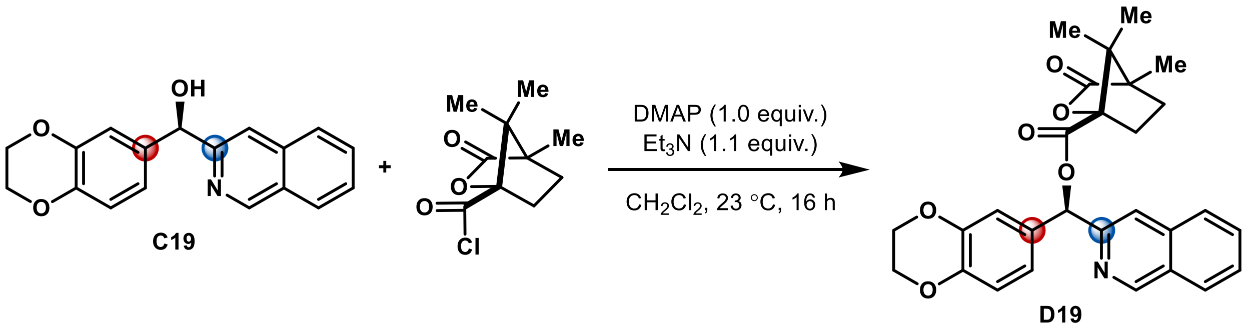

^{1}H NMR (600 MHz, CDCl$_3$): δ 9.19 (s, 1H), 7.94 (d, *J* = 8.2 Hz, 1H), 7.83 (d, *J* = 8.2 Hz, 1H), 7.79 (s, 1H), 7.70 (t, *J* = 7.5 Hz, 1H), 7.59 (t, *J* = 7.5 Hz, 1H), 7.09 (s, 1H), 7.00 (s, 1H), 6.99 (d, *J* = 6.0 Hz, 1H), 6.83 (d, *J* = 8.9 Hz, 1H), 4.21 (s, 2H), 4.21 (s, 2H), 2.55 – 2.48 (m, 1H), 2.14 – 2.07 (m, 1H), 1.98 – 1.91 (m, 1H), 1.73– 1.69 (m, 1H), 1.12 (s, 3H), 1.05 (s, 3H), 0.97 (s, 3H) ppm
^{13}C NMR (151 MHz, CDCl$_3$): δ 178.4, 166.6, 152.5, 152.0, 143.7, 143.5, 136.1, 131.8, 130.7, 127.9, 127.5, 127.5, 126.9, 121.1, 117.4, 117.0, 116.9, 91.1, 78.5, 64.3, 64.2, 54.9, 54.4, 30.8, 29.0, 16.8, 16.7, 9.7 ppm
HRMS (ESI+): calculated for $C_{28}H_{28}NO_6$ [M+H]$^+$: 474.1911, found 474.1910.

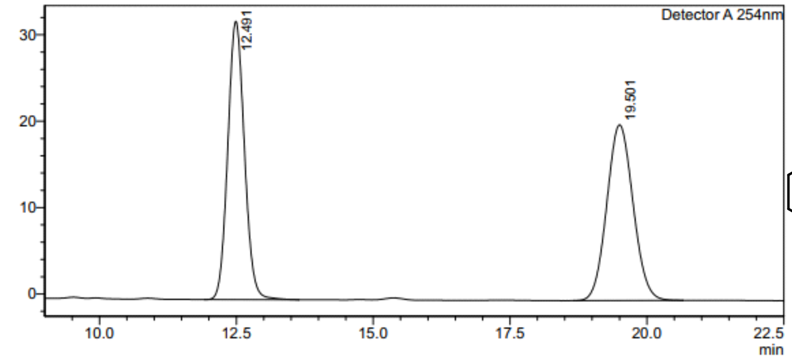

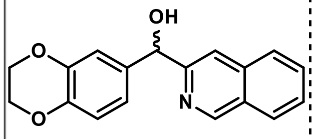

(±)-**C19**

<Peak Table>

Detector A 254nm

Peak#	Ret. Time	Area	Height	Conc.	Unit	Mark	Name
1	12.491	682890	32208	50.138			
2	19.501	679140	20332	49.862			
Total		1362029	52540				

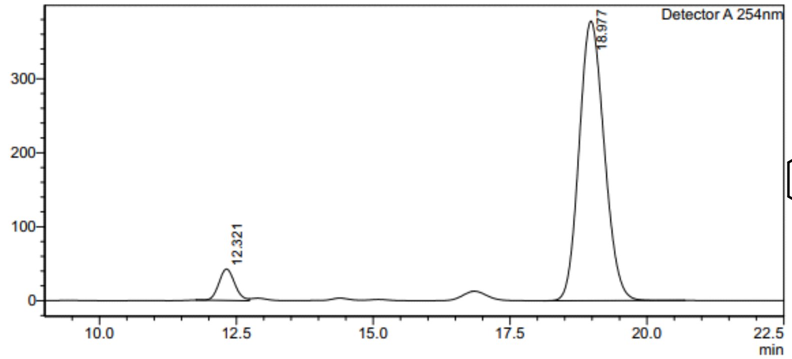

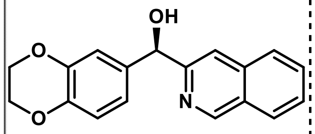

C19
87% yield, 87% ee
obtained by catalyst **B**

<Peak Table>

Detector A 254nm

Peak#	Ret. Time	Area	Height	Conc.	Unit	Mark	Name
1	12.321	854607	42452	6.616		M	
2	18.977	12062890	377888	93.384		M	
Total		12917496	420340				

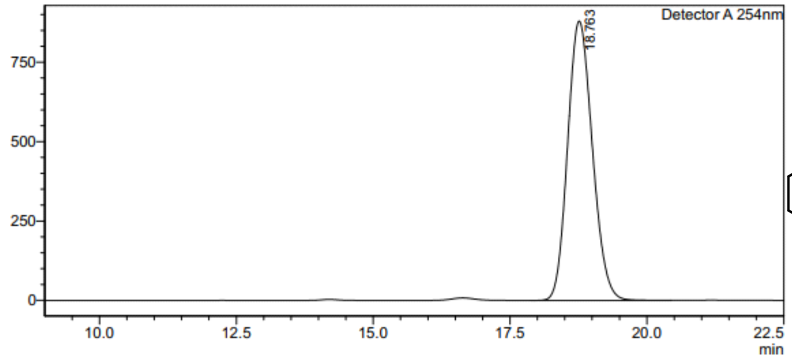

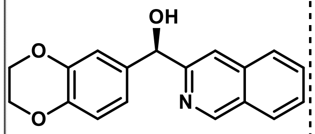

C19
> 99.9% ee

<Peak Table>

Detector A 254nm

Peak#	Ret. Time	Area	Height	Conc.	Unit	Mark	Name
1	18.763	27702340	879485	100.000			
Total		27702340	879485				

Supplementary Table 21. Crystal data and structure refinement for **D19**

CCDC Number	2017161
Empirical formula	$C_{28}H_{27}NO_6$
Formula weight	473.50
Temperature/K	100
Crystal system	triclinic
Space group	P1
a/Å	6.7753(2)
b/Å	9.6107(3)
c/Å	9.7616(3)
α/°	113.7830(10)
β/°	90.0690(10)
γ/°	92.6130(10)
Volume/Å^3	580.91(3)
Z	1
ρ_{calc}g/cm^3	1.354
μ/mm^{-1}	0.780
F(000)	250.0
Crystal size/mm^3	0.42 × 0.41 × 0.1
Radiation	CuKα (λ = 1.54178)
2Θ range for data collection/°	10.07 to 137.542
Index ranges	$-8 \le h \le 8$, $-11 \le k \le 11$, $-11 \le l \le 11$
Reflections collected	15483
Independent reflections	4125 [R_{int} = 0.0348, R_{sigma} = 0.0318]
Data/restraints/parameters	4125/3/320
Goodness-of-fit on F^2	1.067
Final R indexes [I>=2σ (I)]	R_1 = 0.0393, wR_2 = 0.1049
Final R indexes [all data]	R_1 = 0.0396, wR_2 = 0.1054
Largest diff. peak/hole / e Å^{-3}	0.23/-0.20
Flack parameter	-0.02(19)

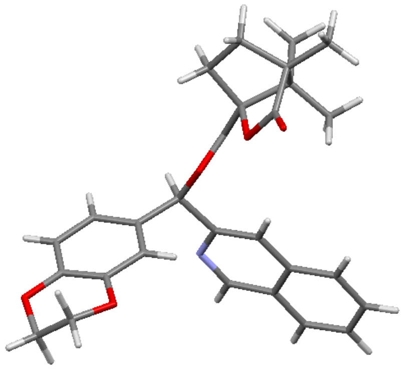

Supplementary Figure 20. The single crystal configuration of **D19** is demonstrated in a capped sticks mode.

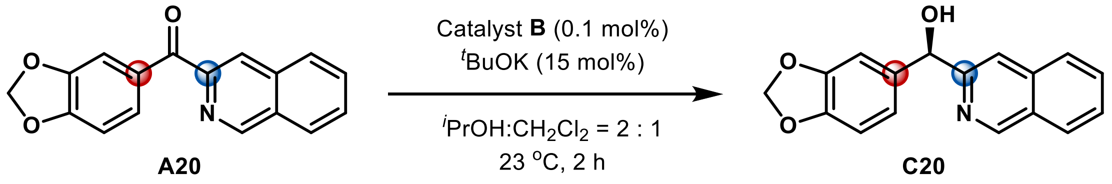

Following the general procedure, **C20** (46.9 mg, 84% yield, 88% ee) was obtained as white solid. Its major pure enantiomer was purified by chiral preparative HPLC (OD-H, 2.0*25 cm, 5μm) and the *X*-ray crystal of the optically pure enantiomer (100% ee) was obtained by liquid/liquid diffusion with THF/hexane system.

^{1}H NMR (600 MHz, CDCl$_3$): δ 9.21 (s, 1H), 7.96 (d, *J* = 8.2 Hz, 1H), 7.76 (d, *J* = 8.2 Hz, 1H), 7.67 (t, *J* = 7.5 Hz, 1H), 7.58 (t, *J* = 7.5 Hz, 1H), 7.56 (s, 1H), 6.95 (d, *J* = 8.0 Hz, 1H), 6.90 (s, 1H), 6.79 (d, *J* = 8.0 Hz, 1H), 5.92 (d, *J* = 5.5 Hz, 2H), 5.87 (s, 1H), 4.74 (s, 1H). ppm
^{13}C NMR (151 MHz, CDCl$_3$): δ 154.8, 151.5, 147.8, 147.1, 137.6, 136.3, 130.7, 127.9, 127.6, 127.2, 126.7, 120.6, 117.1, 108.1, 107.5, 101.0, 75.2 ppm
HRMS (ESI+): calculated for $C_{17}H_{14}NO_3$ [M+H]$^+$: 280.0968, found 280.0972.
HPLC (OD-H, 0.46*25 cm, 5μm, hexane / ethanol = 85/15, flow 1 mL/min, detection at 254 nm) retention time = 9.360 min (minor) and 14.573 min (major).

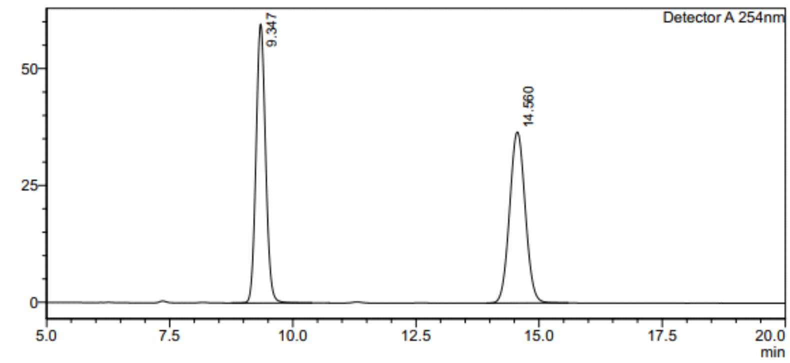

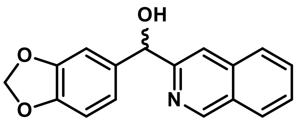

(±)-**C20**

<Peak Table>

Detector A 254nm

Peak#	Ret. Time	Area	Height	Conc.	Unit	Mark	Name
1	9.347	806669	59686	50.042			
2	14.560	805307	36598	49.958			
Total		1611976	96284				

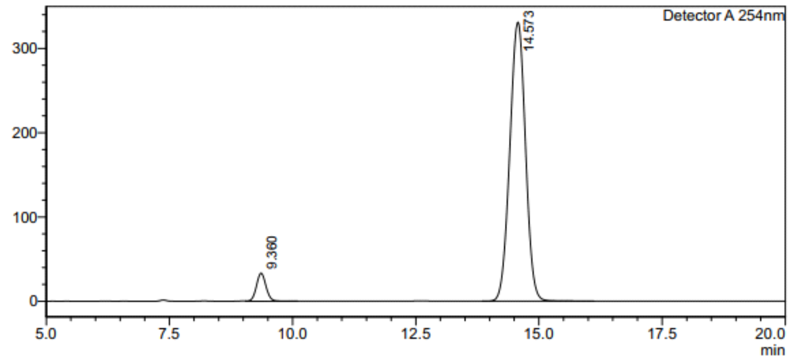

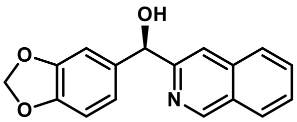

C20
84% yield, 88% ee
obtained by catalyst **B**

<Peak Table>

Detector A 254nm

Peak#	Ret. Time	Area	Height	Conc.	Unit	Mark	Name
1	9.360	451309	33271	5.833			
2	14.573	7286332	330957	94.167			
Total		7737641	364228				

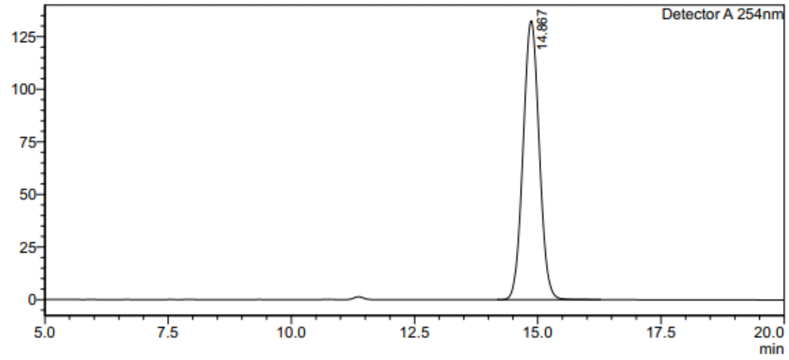

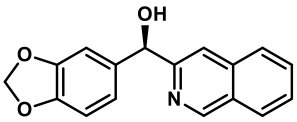

C20
> 99.9% ee

<Peak Table>

Detector A 254nm

Peak#	Ret. Time	Area	Height	Conc.	Unit	Mark	Name
1	14.867	3029524	132540	100.000			
Total		3029524	132540				

Supplementary Table 22. Crystal data and structure refinement for **C20**

CCDC Number	2017154
Empirical formula	$C_{17}H_{13}NO_3$
Formula weight	279.28
Temperature/K	100
Crystal system	orthorhombic
Space group	$P2_12_12_1$
a/Å	7.0712(2)
b/Å	10.2476(2)
c/Å	18.3156(4)
α/°	90
β/°	90
γ/°	90
Volume/Å^3	1327.20(5)
Z	4
ρ_{calc}g/cm^3	1.398
μ/mm^{-1}	0.790
F(000)	584.0
Crystal size/mm^3	0.38 × 0.38 × 0.06
Radiation	CuKα (λ = 1.54178)
2Θ range for data collection/°	9.658 to 136.59
Index ranges	$-8 \le h \le 8$, $-11 \le k \le 12$, $-22 \le l \le 22$
Reflections collected	21131
Independent reflections	2414 [R_{int} = 0.0350, R_{sigma} = 0.0171]
Data/restraints/parameters	2414/0/192
Goodness-of-fit on F^2	1.085
Final R indexes [I>=2σ (I)]	R_1 = 0.0247, wR_2 = 0.0635
Final R indexes [all data]	R_1 = 0.0251, wR_2 = 0.0638
Largest diff. peak/hole / e Å^{-3}	0.14/-0.13
Flack parameter	-0.10(6)

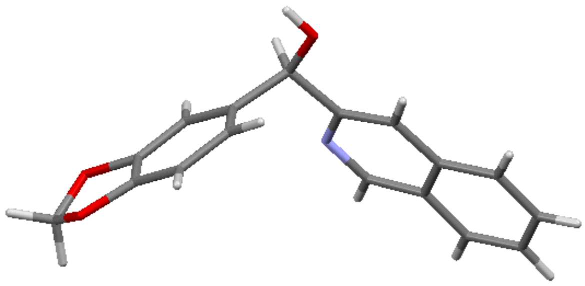

Supplementary Figure 21. The single crystal configuration of **C20** is demonstrated in a capped sticks mode.

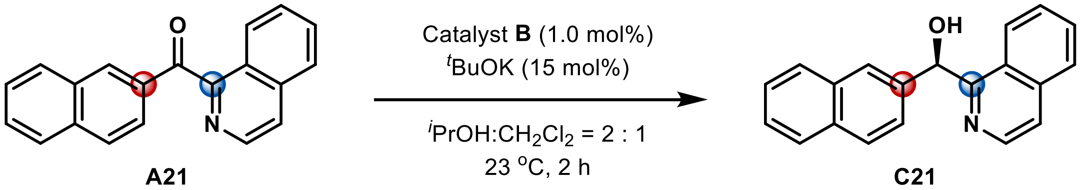

Following the general procedure, **C21** (34.1 mg, 60% yield, 81% ee) was obtained as white solid. Its major pure enantiomer was purified by chiral preparative HPLC (OD-H, 2.0*25 cm, 5μm) and the *X*-ray crystal of the optically pure enantiomer (100% ee) was obtained by liquid/liquid diffusion with CH_2Cl_2/hexane system.

^{1}H NMR (600 MHz, $CDCl_3$): δ 8.59 (d, J = 5.7 Hz, 1H), 8.02 (d, J = 8.4 Hz, 1H), 7.88 (s, 1H), 7.84 (d, J = 8.4 Hz, 1H), 7.80 (d, J = 7.6 Hz, 1H), 7.76 (d, J = 7.6 Hz, 1H), 7.74 (d, J = 8.4 Hz, 1H), 7.69 (d, J = 5.7 Hz, 1H), 7.62 (t, J = 7.5 Hz, 1H), 7.48 – 7.40 (m, 3H), 7.37 (d, J = 8.4 Hz, 1H), 6.53 (s, 1H), 6.36 (s, 1H) ppm
^{13}C NMR (151 MHz, $CDCl_3$): δ 159.0, 140.7, 140.0, 136.6, 133.4, 133.0, 130.3, 128.7, 128.0, 127.6, 127.5, 127.4, 126.9, 126.1, 126.0, 125.2, 124.8, 121.2, 72.7 ppm
HRMS (ESI+): calculated for $C_{20}H_{16}NO$ $[M+H]^+$: 286.1226, found 286.1231.
HPLC (OD-H, 0.46*25 cm, 5μm, hexane / ethanol = 80/20, flow 1 mL/min, detection at 254 nm) retention time = 7.675 min (minor) and 16.251 min (major).

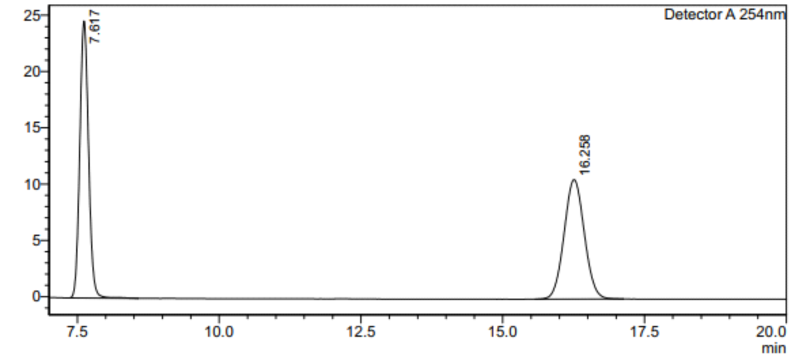

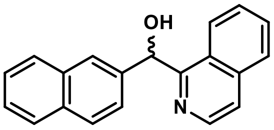

(±)-**C21**

<Peak Table>

Detector A 254nm

Peak#	Ret. Time	Area	Height	Conc.	Unit	Mark	Name
1	7.617	264601	24623	50.063		S	
2	16.258	263932	10609	49.937			
Total		528533	35232				

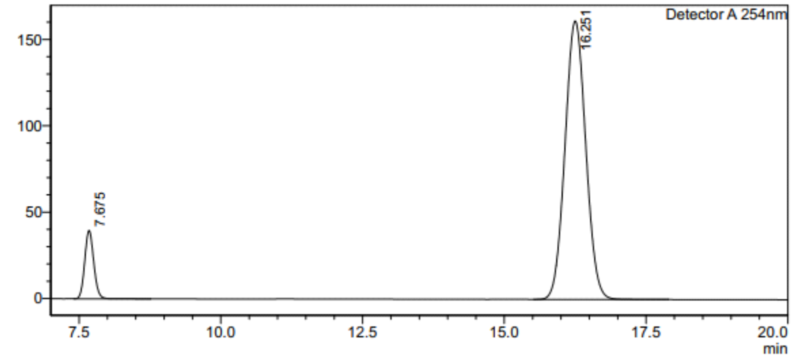

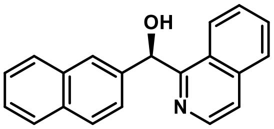

C21
60% yield, 81% ee
obtained by catalyst **B**

<Peak Table>

Detector A 254nm

Peak#	Ret. Time	Area	Height	Conc.	Unit	Mark	Name
1	7.675	440801	39605	9.678		SV	
2	16.251	4113837	161277	90.322			
Total		4554638	200882				

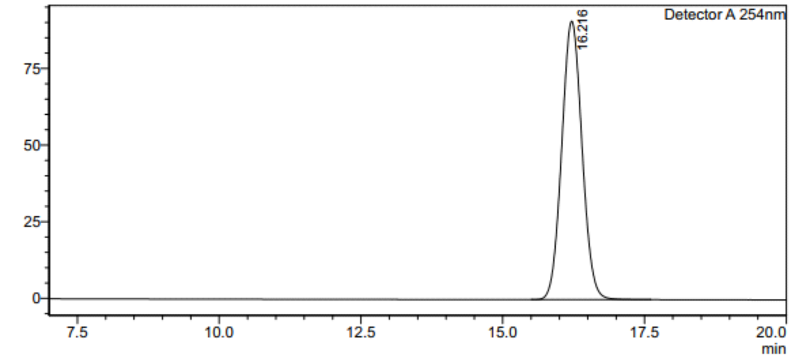

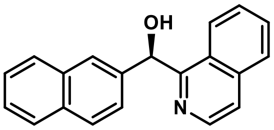

C21
> 99.9% ee

<Peak Table>

Detector A 254nm

Peak#	Ret. Time	Area	Height	Conc.	Unit	Mark	Name
1	16.216	2266605	90872	100.000			
Total		2266605	90872				

Supplementary Table 23. Crystal data and structure refinement for **C21**

CCDC Number	2017185
Empirical formula	$C_{20}H_{15}NO$
Formula weight	285.33
Temperature/K	100
Crystal system	monoclinic
Space group	$P2_1$
a/Å	8.0845(4)
b/Å	5.6020(3)
c/Å	15.5985(7)
α/°	90
β/°	90.2940(10)
γ/°	90
Volume/Å^3	706.44(6)
Z	2
ρ_{calc}g/cm^3	1.341
μ/mm^{-1}	0.647
F(000)	300.0
Crystal size/mm^3	0.41 × 0.36 × 0.35
Radiation	CuKα (λ = 1.54178)
2Θ range for data collection/°	5.666 to 149.39
Index ranges	$-10 \le h \le 10$, $-6 \le k \le 7$, $-19 \le l \le 19$
Reflections collected	11062
Independent reflections	2808 [R_{int} = 0.0285, R_{sigma} = 0.0272]
Data/restraints/parameters	2808/1/201
Goodness-of-fit on F^2	1.049
Final R indexes [I>=2σ (I)]	R_1 = 0.0269, wR_2 = 0.0698
Final R indexes [all data]	R_1 = 0.0272, wR_2 = 0.0700
Largest diff. peak/hole / e Å^{-3}	0.14/-0.12
Flack parameter	-0.02(10)

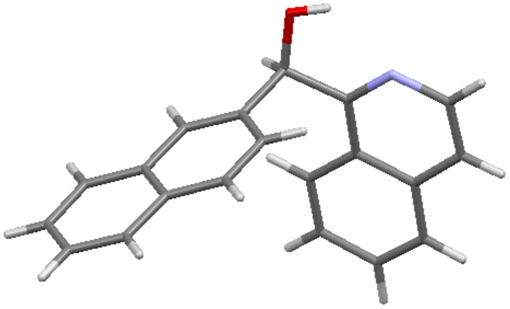

Supplementary Figure 22. The single crystal configuration of **C21** is demonstrated in a capped sticks mode.

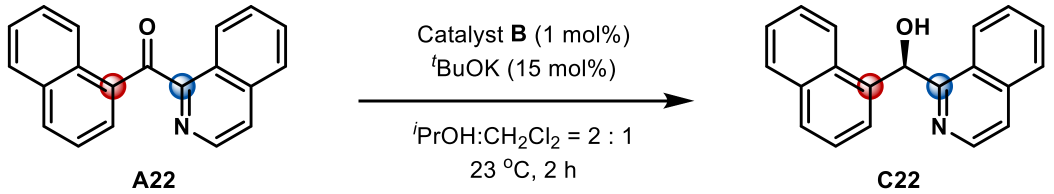

Following the general procedure, **C22** (52.4 mg, 92% yield, 87% ee) was obtained as white solid.
^{1}H NMR (400 MHz, CDCl$_3$): δ 8.68 – 8.59 (m, 2H), 7.89 (d, *J* = 8.2 Hz, 1H), 7.85 (d, *J* = 8.4 Hz, 1H), 7.75 (d, *J* = 8.2 Hz, 1H), 7.71 (d, *J* = 5.8 Hz, 1H), 7.67 – 7.33 (m, 2H), 7.60 (t, *J* = 7.5 Hz, 1H), 7.55 (t, *J* = 7.5 Hz, 1H), 7.31 (t, *J* = 7.7 Hz, 1H), 7.19 (t, *J* = 7.7 Hz, 1H), 7.12 (s, 1H), 6.75 (s, 1H) ppm
^{13}C NMR (101 MHz, CDCl$_3$): δ 159.4, 140.0, 139.1, 136.4, 134.2, 131.9, 130.3, 128.8, 128.7, 127.4, 127.3, 126.7, 125.9, 125.8, 125.7, 125.3, 124.9, 123.9, 121.1, 69.4 ppm
HRMS (ESI+): calculated for $C_{20}H_{16}NO$ [M+H]$^+$: 286.1226, found 286.1223.
HPLC (OD-H, 0.46*25 cm, 5μm, hexane / ethanol = 75/25, flow 1 mL/min, detection at 254 nm) retention time = 6.555 min (minor) and 16.565 min (major).

The major enantiomer of **C22** (87% ee) was purified by chiral preparative HPLC (OD-H, 2.0*25 cm, 5μm). Because we were unable to grow *X*-ray qualified crystal of the optically pure **C22** (100% ee), we derivatize it with (1*S*)-(-)-camphanic acid chloride to afford **D22** as a single diastereomer. Then the *X*-ray crystal of the optically pure **D22** was obtained by liquid/liquid diffusion with CH_2Cl_2/hexane system.

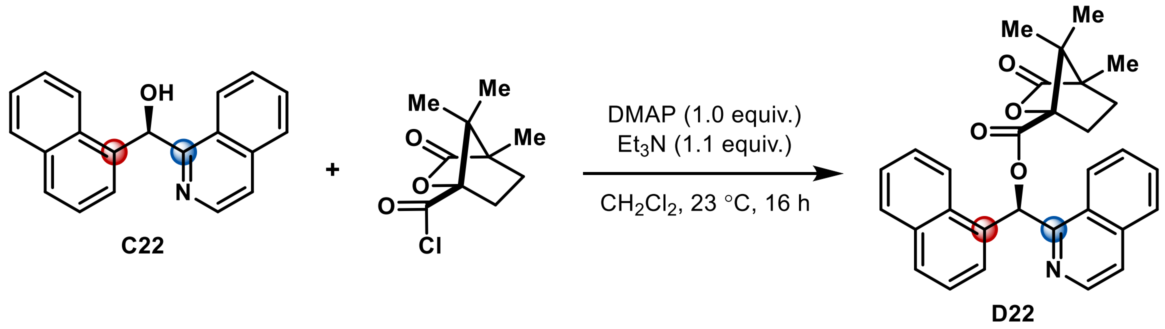

^{1}H NMR (400 MHz, CDCl$_3$): δ 8.56 (d, *J* = 5.6 Hz, 1H), 8.44 (s, 1H), 8.27 (d, *J* = 8.4 Hz, 1H), 8.02 (d, *J* = 8.4 Hz, 1H), 7.91 (d, *J* = 7.8 Hz, 1H), 7.85 (d, *J* = 8.4 Hz, 2H), 7.67 – 7.51 (m, 4H), 7.45 (t, *J* = 7.2 Hz, 1H), 7.38 – 7.30 (m, 2H), 2.68 – 2.60 (m, 1H), 2.29 – 2.21 (m, 1H), 1.93 – 1.86 (m, 1H), 1.72 – 1.62 (m, 1H), 1.07 (s, 3H), 0.96 (s, 3H), 0.90 (s, 3H) ppm
^{13}C NMR (101 MHz, CDCl$_3$): δ 178.2, 167.1, 155.7, 141.6, 136.6, 133.9, 133.1, 131.2, 130.0, 129.7, 129.0, 127.6, 127.5, 127.4, 126.9, 126.1, 126.0, 125.3, 124.6, 123.2, 121.2, 91.4, 73.8, 55.0, 54.4, 31.0, 28.9, 16.7, 16.7, 9.7 ppm
HRMS (ESI+): calculated for $C_{30}H_{28}NO_4$ [M+H]$^+$: 466.2013, found 466.2011.

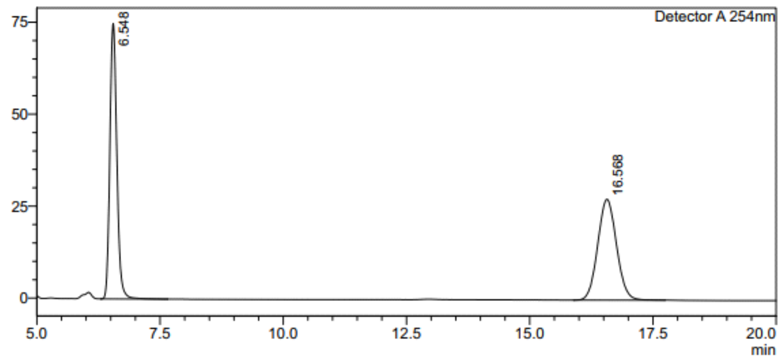

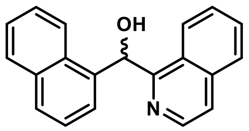

(±)-**C22**

<Peak Table>

Detector A 254nm

Peak#	Ret. Time	Area	Height	Conc.	Unit	Mark	Name
1	6.548	732618	74898	50.206		S	
2	16.568	726594	27425	49.794			
Total		1459212	102324				

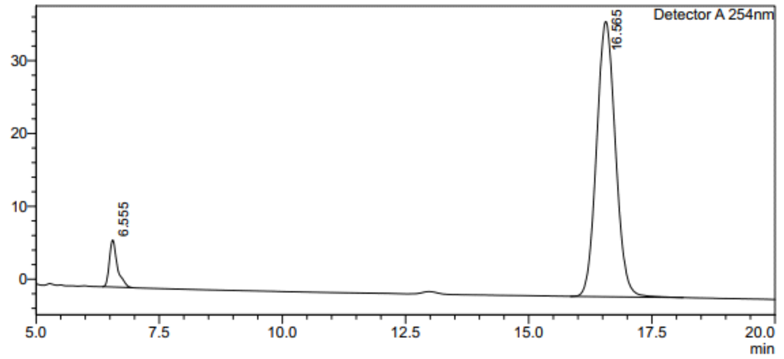

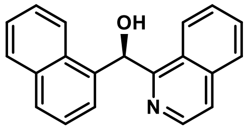

C22
92% yield, 87% ee
obtained by catalyst **B**

<Peak Table>

Detector A 254nm

Peak#	Ret. Time	Area	Height	Conc.	Unit	Mark	Name
1	6.555	72246	6443	6.681		M	
2	16.565	1009071	37786	93.319			
Total		1081316	44229				

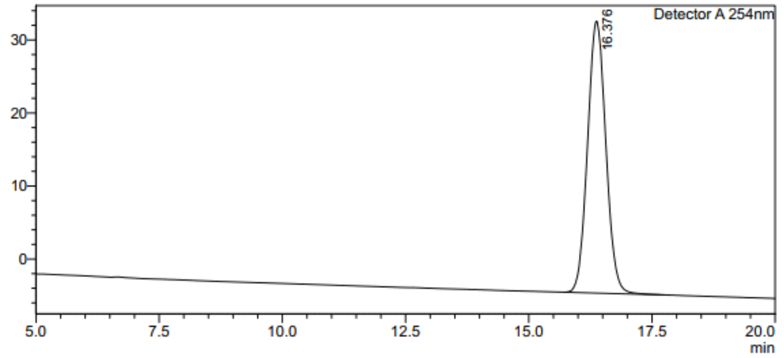

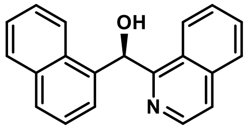

C22
> 99.9% ee

<Peak Table>

Detector A 254nm

Peak#	Ret. Time	Area	Height	Conc.	Unit	Mark	Name
1	16.376	964344	37246	100.000			
Total		964344	37246				

Supplementary Table 24. Crystal data and structure refinement for **D22**

Identification code	2183982
Empirical formula	$C_{30}H_{27}NO_4$
Formula weight	465.52
Temperature/K	100.0(2)
Crystal system	monoclinic
Space group	$P2_1$
a/Å	10.8891(9)
b/Å	16.8386(14)
c/Å	13.4455(11)
α/°	90
β/°	108.194(3)
γ/°	90
Volume/Å^3	2342.1(3)
Z	4
ρ_{calc}g/cm^3	1.320
μ/mm^{-1}	0.701
F(000)	984.0
Crystal size/mm^3	0.35 × 0.33 × 0.24
Radiation	CuKα (λ = 1.54178)
2Θ range for data collection/°	6.92 to 136.52
Index ranges	$-13 \leq h \leq 12$, $-20 \leq k \leq 20$, $-16 \leq l \leq 16$
Reflections collected	43281
Independent reflections	8541 [R_{int} = 0.0529, R_{sigma} = 0.0372]
Data/restraints/parameters	8541/39/675
Goodness-of-fit on F^2	1.034
Final R indexes [I>=2σ (I)]	R_1 = 0.0279, wR_2 = 0.0718
Final R indexes [all data]	R_1 = 0.0287, wR_2 = 0.0722
Largest diff. peak/hole / e Å^{-3}	0.25/-0.17
Flack parameter	-0.01(5)

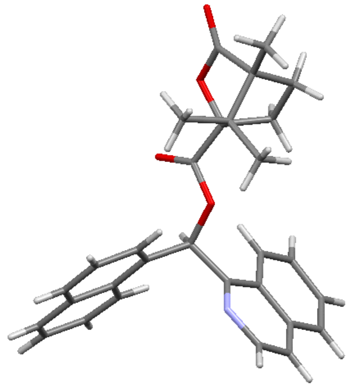

Supplementary Figure 23. The single crystal configuration of **D22** is demonstrated in a capped sticks mode.

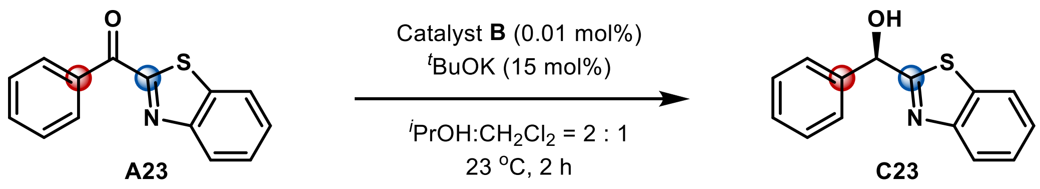

Following the general procedure, **C23** (25.1 mg, 52% yield, 76% ee) was obtained as white solid. Its major pure enantiomer was purified by chiral preparative HPLC (OD-H, 2.0*25 cm, 5μm) and the *X*-ray crystal of the optically pure enantiomer (100% ee) was obtained by liquid/liquid diffusion with CH_2Cl_2/hexane system.

^{1}H NMR (600 MHz, $CDCl_3$): δ 7.95 (t, *J* = 8.2 Hz, 1H), 7.81 (t, *J* = 8.2 Hz, 1H), 7.63 – 7.29 (m, 7H), 6.14 (s, 1H), 4.62 (s, 1H) ppm
^{13}C NMR (151 MHz, $CDCl_3$): δ 175.4, 152.5, 140.9, 135.1, 128.7, 128.6, 126.7, 126.1, 125.1, 122.9, 121.7, 74.2 ppm
HRMS (ESI+): calculated for $C_{14}H_{12}NOS$ $[M+H]^+$: 242.0634, found 242.0637.
HPLC (OD-H, 0.46*25 cm, 5μm, hexane / ethanol = 90/10, flow 1 mL/min, detection at 254 nm) retention time = 7.065 min (minor) and 13.612 min (major)

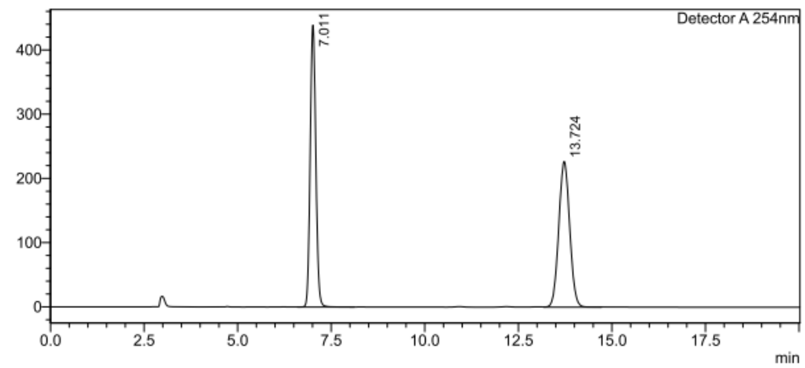

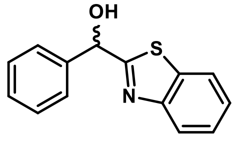

(±)-**C23**

<Peak Table>

Detector A 254nm

Peak#	Ret. Time	Area	Height	Conc.	Unit	Mark	Name
1	7.011	4610410	438717	49.978		V	
2	13.724	4614525	226730	50.022			
Total		9224935	665447				

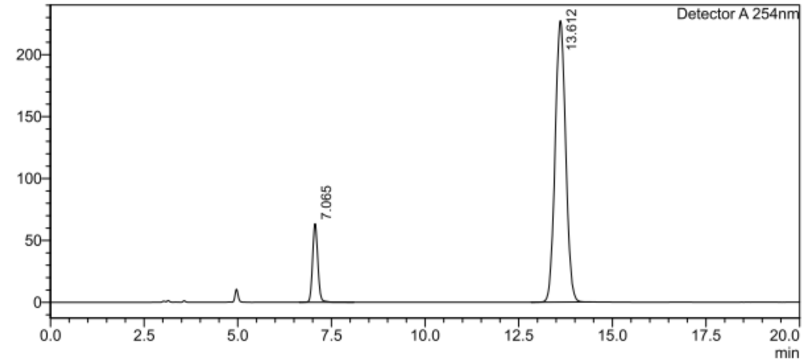

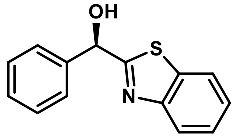

C23
52% yield, 76% ee
obtained by catalyst **B**

<Peak Table>

Detector A 254nm

Peak#	Ret. Time	Area	Height	Conc.	Unit	Mark	Name
1	7.065	613756	63439	12.124		S	
2	13.612	4448400	227231	87.876			
Total		5062156	290670				

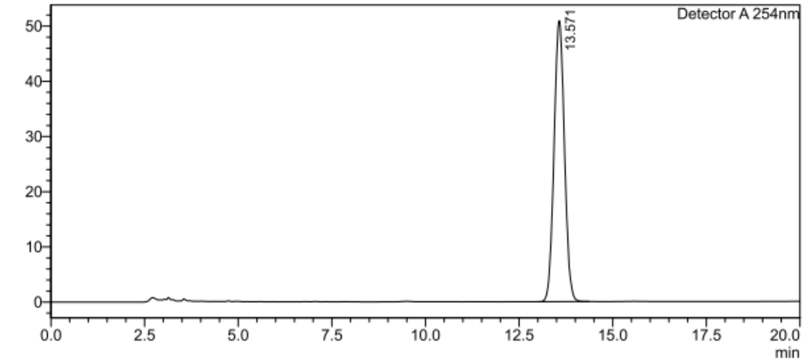

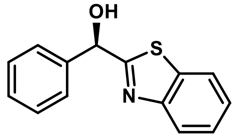

C**23**
> 99.9% ee

<Peak Table>

Detector A 254nm

Peak#	Ret. Time	Area	Height	Conc.	Unit	Mark	Name
1	13.571	984532	50900	100.000			
Total		984532	50900				

Supplementary Table 25. Crystal data and structure refinement for **C23**

CCDC Number	2017100
Empirical formula	$C_{14}H_{10}NOS$
Formula weight	240.29
Temperature/K	100.04
Crystal system	monoclinic
Space group	$P2_1$
a/Å	8.1762(9)
b/Å	8.4247(9)
c/Å	9.1055(11)
α/°	90
β/°	108.890(4)
γ/°	90
Volume/Å^3	593.42(12)
Z	2
ρ_{calc}g/cm^3	1.345
μ/mm^{-1}	0.253
F(000)	250.0
Crystal size/mm^3	0.35 × 0.29 × 0.28
Radiation	MoKα (λ = 0.71073)
2Θ range for data collection/°	4.728 to 56.752
Index ranges	$-10 \le h \le 10$, $-11 \le k \le 11$, $-12 \le l \le 12$
Reflections collected	4976
Independent reflections	4976 [R_{int} = ?, R_{sigma} = 0.0629]
Data/restraints/parameters	4976/1/157
Goodness-of-fit on F^2	1.094
Final R indexes [I>=2σ (I)]	R_1 = 0.0634, wR_2 = 0.1672
Final R indexes [all data]	R_1 = 0.0781, wR_2 = 0.1786
Largest diff. peak/hole / e Å^{-3}	0.88/-0.94
Flack parameter	-0.49(5)

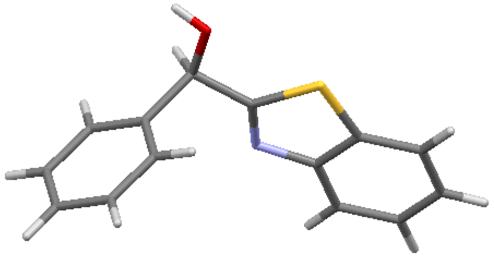

Supplementary Figure 24. The single crystal configuration of **D23** is demonstrated in a capped sticks mode.

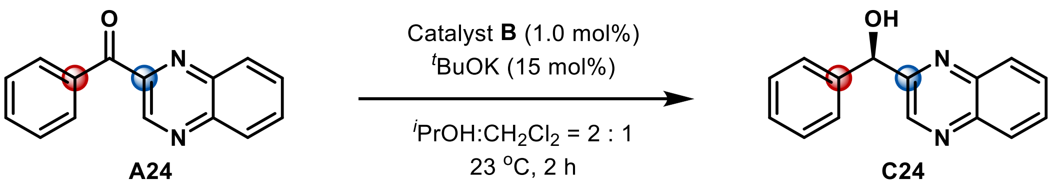

Following the general procedure, **C24** (28.3 mg, 60% yield, 75% ee) was obtained as yellow solid. Its major pure enantiomer was purified by chiral preparative HPLC (OD-H, 2.0*25 cm, 5μm) and the *X*-ray crystal of the optically pure enantiomer (100% ee) was obtained by liquid/liquid diffusion with CH_2Cl_2/hexane system.

^{1}H NMR (600 MHz, $CDCl_3$): δ 8.73 (s, 1H), 8.13 (d, J = 8.2 Hz, 1H), 8.10 (d, J = 8.2 Hz, 1H), 7.80 (t, J = 6.9 Hz, 1H), 7.76 (t, J = 6.9 Hz, 1H), 7.43 (d, J = 7.4 Hz, 2H), 7.36 (t, J = 7.4 Hz, 2H), 7.31 (t, J = 7.4 Hz, 1H), 6.02 (s, 1H), 5.22 (s, 1H) ppm

^{13}C NMR (151 MHz, $CDCl_3$): δ 155.5, 144.2, 141.9, 141.3, 140.4, 130.5, 129.8, 129.2, 128.9, 128.8, 128.4, 127.2, 74.4 ppm

HRMS (ESI+): calculated for $C_{15}H_{13}N_2O$ $[M+H]^+$: 237.1022, found 237.1026.

HPLC (OD-H, 0.46*25 cm, 5μm, hexane / ethanol = 90/10, flow 1 mL/min, detection at 254 nm) retention time = 8.079 min (minor) and 9.178 min (major).

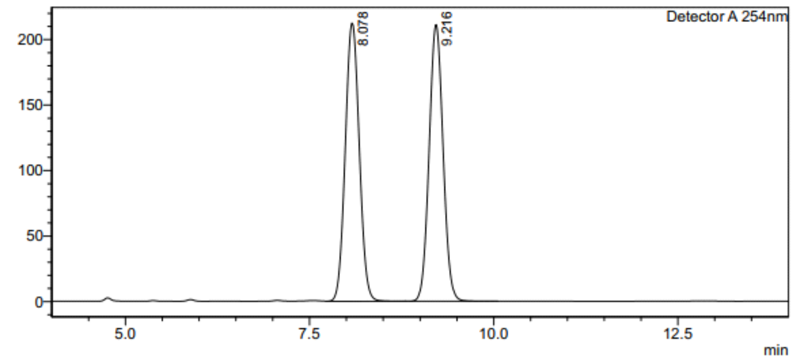

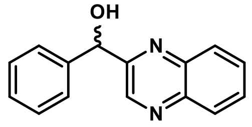

(±)-**C24**

<Peak Table>

Detector A 254nm

Peak#	Ret. Time	Area	Height	Conc.	Unit	Mark	Name
1	8.078	2795106	212456	49.970			
2	9.216	2798407	211298	50.030		V	
Total		5593513	423754				

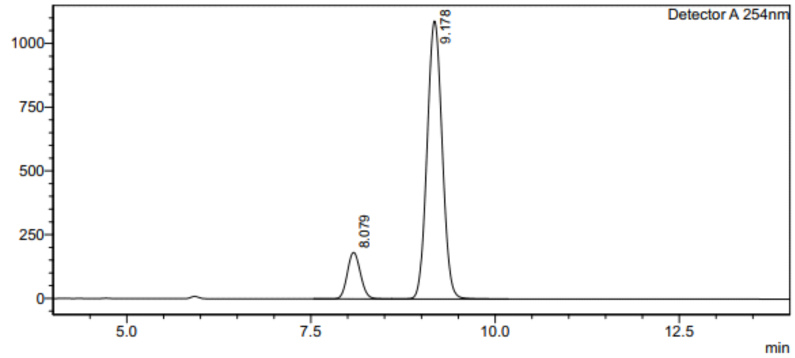

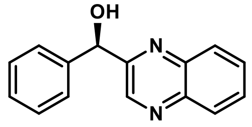

C24
60% yield, 75% ee
obtained by catalyst **B**

<Peak Table>

Detector A 254nm

Peak#	Ret. Time	Area	Height	Conc.	Unit	Mark	Name
1	8.079	2253219	181829	12.638			
2	9.178	15576397	1090038	87.362		V	
Total		17829617	1271867				

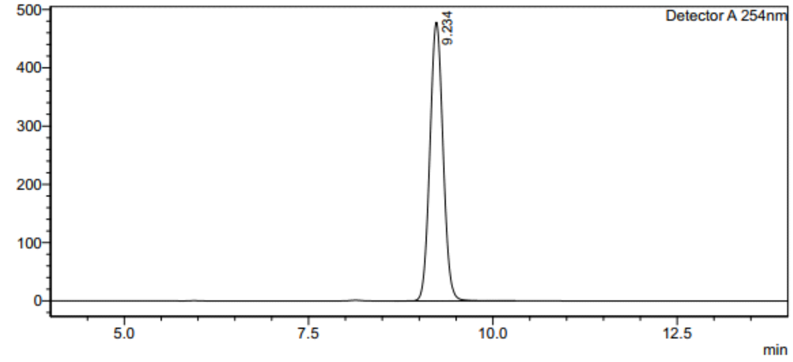

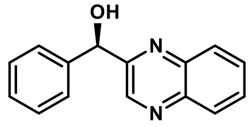

C24
> 99.9% ee

<Peak Table>

Detector A 254nm

Peak#	Ret. Time	Area	Height	Conc.	Unit	Mark	Name
1	9.234	5961620	478274	100.000			
Total		5961620	478274				

Supplementary Table 26. Crystal data and structure refinement for **C24**

CCDC Number	2017097
Empirical formula	$C_{15}H_{12}N_2O$
Formula weight	236.27
Temperature/K	100
Crystal system	monoclinic
Space group	$P2_1$
a/Å	10.1355(3)
b/Å	10.4919(3)
c/Å	10.9913(4)
α/°	90
β/°	91.5730(10)
γ/°	90
Volume/Å^3	1168.38(6)
Z	4
ρ_{calc}g/cm^3	1.343
μ/mm^{-1}	0.687
F(000)	496.0
Crystal size/mm^3	0.34 × 0.33 × 0.29
Radiation	CuKα (λ = 1.54178)
2Θ range for data collection/°	8.728 to 136.572
Index ranges	$-12 \le h \le 12, -12 \le k \le 12, -11 \le l \le 13$
Reflections collected	13842
Independent reflections	4234 [R_{int} = 0.0264, R_{sigma} = 0.0258]
Data/restraints/parameters	4234/1/328
Goodness-of-fit on F^2	1.061
Final R indexes [I>=2σ (I)]	R_1 = 0.0262, wR_2 = 0.0664
Final R indexes [all data]	R_1 = 0.0268, wR_2 = 0.0668
Largest diff. peak/hole / e Å^{-3}	0.19/-0.14
Flack parameter	-0.07(7)

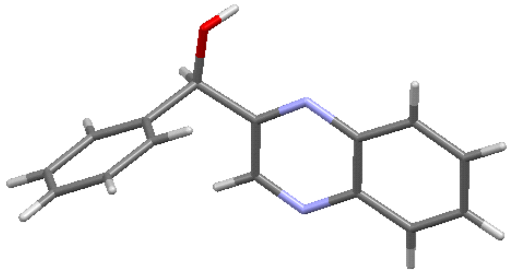

Supplementary Figure 25. The single crystal configuration of **C24** is demonstrated in a capped sticks mode.

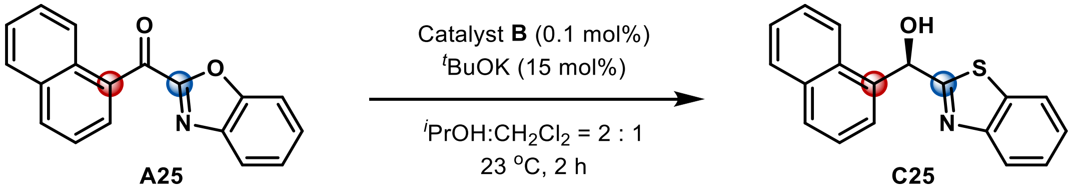

Following the general procedure, **C25** (28.1 mg, 51% yield, 86% ee) was obtained as white solid. Its major pure enantiomer was purified by chiral preparative HPLC (OD-H, 2.0*25 cm, 5μm) and the *X*-ray crystal of the optically pure enantiomer (100% ee) was obtained by liquid/liquid diffusion with CH_2Cl_2/hexane system.

^{1}H NMR (600 MHz, $CDCl_3$): δ 8.32 (d, *J* = 8.4 Hz, 1H), 7.90 (d, *J* = 8.1 Hz, 1H), 7.87 (d, *J* = 8.1 Hz, 1H), 7.66 (d, *J* = 7.6 Hz, 1H), 7.60 (d, *J* = 7.2 Hz, 1H), 7.56 (t, *J* = 7.5 Hz, 1H), 7.52 (t, *J* = 7.5 Hz, 1H), 7.47 (t, *J* = 7.6 Hz, 1H), 7.43 (d, *J* = 8.4 Hz, 1H), 7.35 – 7.28 (m, 2H), 6.75 (s, 1H), 3.98 (s, 1H) ppm

^{13}C NMR (151 MHz, $CDCl_3$): δ 166.6, 151.0, 140.4, 134.3, 134.0, 130.8, 129.7, 128.9, 126.8, 126.0, 125.3, 125.3, 124.6, 123.5, 120.1, 110.9, 68.6 ppm

HRMS (ESI+): calculated for $C_{18}H_{14}NO_2$ $[M+H]^+$: 276.1019, found 276.1022.

HPLC (OD-H, 0.46*25 cm, 5μm, hexane / ethanol = 90/10, flow 1 mL/min, detection at 254 nm) retention time = 10.563 min (major) and 12.356 min (minor).

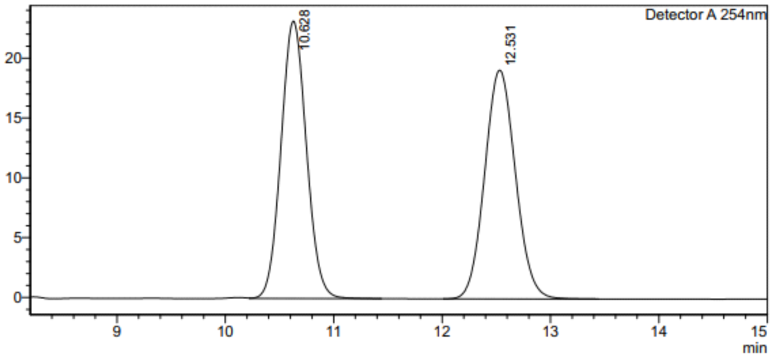

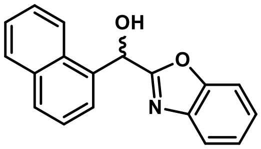

(±)-**C25**

<Peak Table>

Detector A 254nm

Peak#	Ret. Time	Area	Height	Conc.	Unit	Mark	Name
1	10.628	378366	23182	50.019		V	
2	12.531	378081	19122	49.981			
Total		756447	42304				

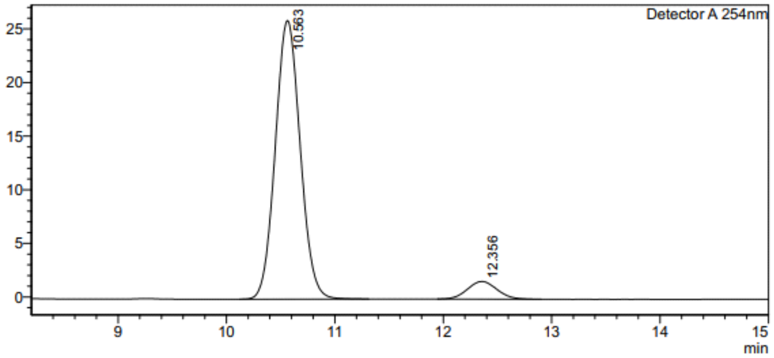

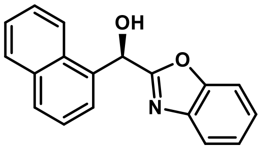

C25
51% yield, 86% ee
obtained by catalyst **B**

<Peak Table>

Detector A 254nm

Peak#	Ret. Time	Area	Height	Conc.	Unit	Mark	Name
1	10.563	415155	25961	92.993			
2	12.356	31282	1642	7.007			
Total		446437	27604				

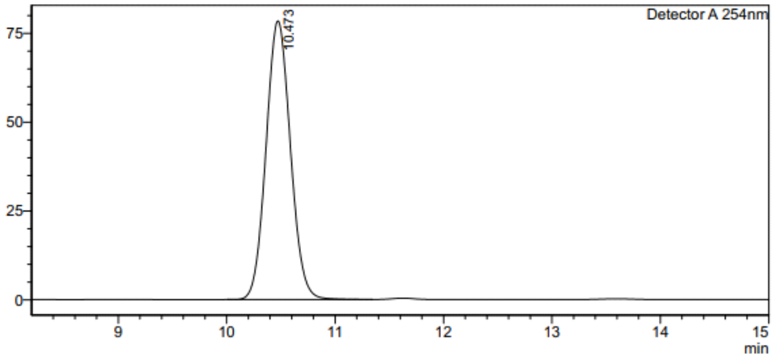

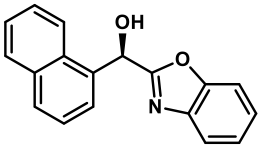

C25
> 99.9% ee

<Peak Table>

Detector A 254nm

Peak#	Ret. Time	Area	Height	Conc.	Unit	Mark	Name
1	10.473	1228158	78410	100.000			
Total		1228158	78410				

Supplementary Table 27. Crystal data and structure refinement for **C25**

CCDC Number	2017199
Empirical formula	$C_{18}H_{13}NO_2$
Formula weight	275.29
Temperature/K	100.0
Crystal system	orthorhombic
Space group	$P2_12_12_1$
a/Å	4.4535(2)
b/Å	12.7601(6)
c/Å	22.7875(10)
α/°	90
β/°	90
γ/°	90
Volume/Å^3	1294.95(10)
Z	4
ρ_{calc}g/cm^3	1.412
μ/mm^{-1}	0.744
F(000)	576.0
Crystal size/mm^3	0.42 × 0.12 × 0.08
Radiation	CuKα (λ = 1.54178)
2Θ range for data collection/°	7.76 to 137.388
Index ranges	$-5 \leq h \leq 5$, $-15 \leq k \leq 15$, $-26 \leq l \leq 27$
Reflections collected	22459
Independent reflections	2395 [R_{int} = 0.0600, R_{sigma} = 0.0269]
Data/restraints/parameters	2395/0/192
Goodness-of-fit on F^2	1.077
Final R indexes [I>=2σ (I)]	R_1 = 0.0318, wR_2 = 0.0823
Final R indexes [all data]	R_1 = 0.0324, wR_2 = 0.0829
Largest diff. peak/hole / e Å^{-3}	0.25/-0.23
Flack parameter	0.07(7)

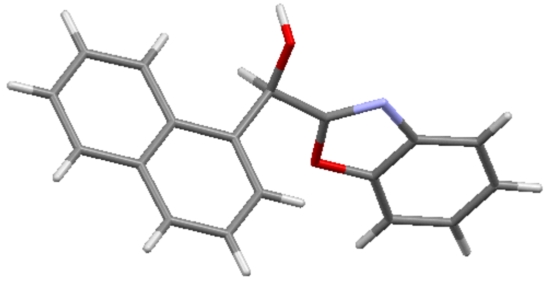

Supplementary Figure 26. The single crystal configuration of **C25** is demonstrated in a capped sticks mode.

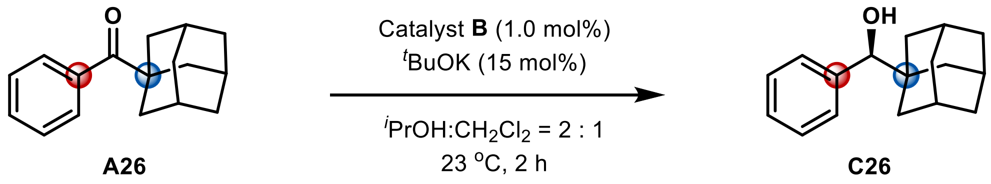

Following the general procedure, **C26** (29.0 mg, 60% yield, 70% ee) was obtained as colorless liquid.

^{1}H NMR (600 MHz, $CDCl_3$): δ 7.33 – 7.27 (m, 2H), 7.26 – 7.24 (m, 3H), 4.19 (s, 1H), 1.96 – 1.91 (m, 3H), 1.66 – 1.54 (m, 10H), 1.50 – 1.45 (m, 3H) ppm
^{13}C NMR (151 MHz, $CDCl_3$): δ 141.1, 127.8, 127.4, 127.2, 83.0, 38.1, 37.1, 37.0, 28.3 ppm
HRMS (ESI+): calculated for $C_{17}H_{21}$ $[M+H-H_2O]^+$: 225.1638, found 225.1640.
HPLC (AD-H, 0.46*25 cm, 5μm, hexane / ethanol = 95/5, flow 1 mL/min, detection at 254 nm) retention time = 7.642 min (major) and 11.331 min (minor).

The major enantiomer of **C26** (70% ee) was purified by chiral preparative HPLC (AD-H, 2.0*25 cm, 5μm). Because we were unable to grow *X*-ray qualified crystal of the optically pure **C26** (100% ee), we derivatize it with (1*S*)-(-)-camphanic acid chloride to afford **D26** as a single diastereomer. Then the *X*-ray crystal of the optically pure **D26** was obtained by liquid/liquid diffusion with CH_2Cl_2/hexane system.

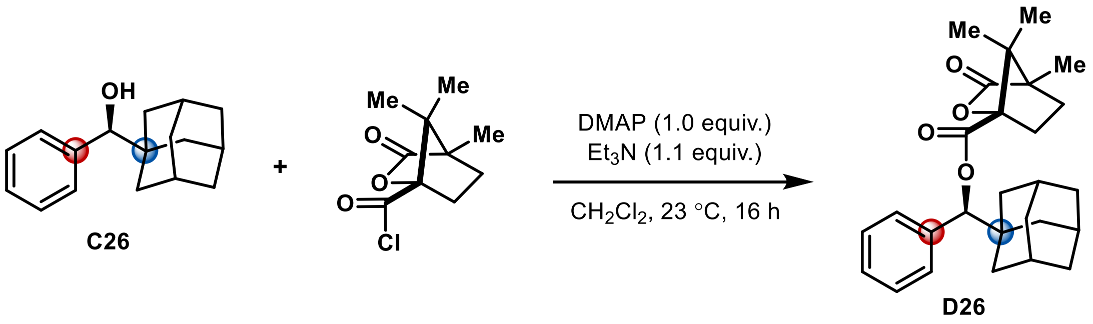

^{1}H NMR (600 MHz, $CDCl_3$): δ 7.34 – 7.27 (m, 3H), 7.25 (d, *J* = 7.8 Hz, 2H), 5.43 (s, 1H), 2.43 – 2.35 (m, 1H), 2.04 –2.00 (m, 1H), 1.93 – 1.88 (m, 1H), 1.72 – 1.54 (m, 13H), 1.51 (d, *J* = 11.8 Hz, 3H), 1.12 (s, 3H), 1.09 (s, 3H), 0.97 (s, 3H) ppm
^{13}C NMR (151 MHz, $CDCl_3$): δ 178.5, 166.9, 136.6, 127.9, 127.8, 127.6, 91.4, 85.2, 54.9, 54.1, 38.2, 36.7, 36.5, 30.9, 29.0, 28.0, 16.9, 9.7 ppm
HRMS (ESI+): calculated for $C_{27}H_{34}NaO_4$ $[M+Na]^+$: 445.2349, found 445.2347.

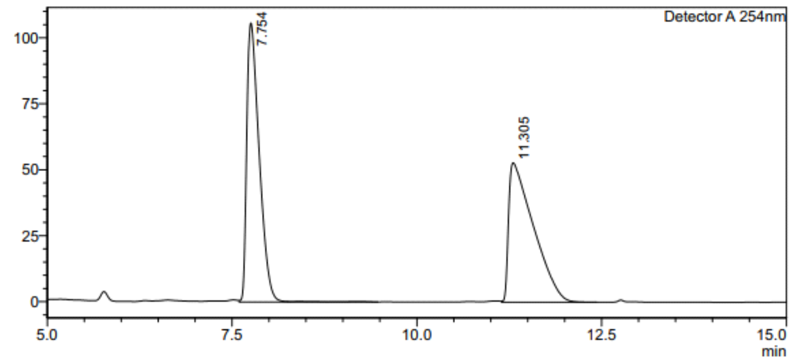

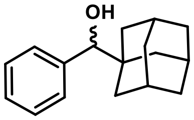

(±)-**C26**

<Peak Table>

Detector A 254nm

Peak#	Ret. Time	Area	Height	Conc.	Unit	Mark	Name
1	7.754	1234365	105687	50.024		S	
2	11.305	1233197	52818	49.976			
Total		2467562	158505				

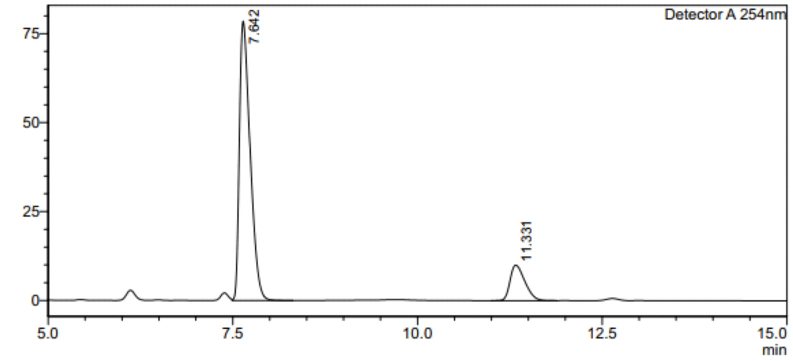

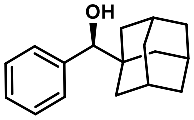

C26
60% yield, 70% ee
obtained by catalyst **B**

<Peak Table>

Detector A 254nm

Peak#	Ret. Time	Area	Height	Conc.	Unit	Mark	Name
1	7.642	791758	78536	85.157			
2	11.331	138009	9977	14.843			
Total		929767	88513				

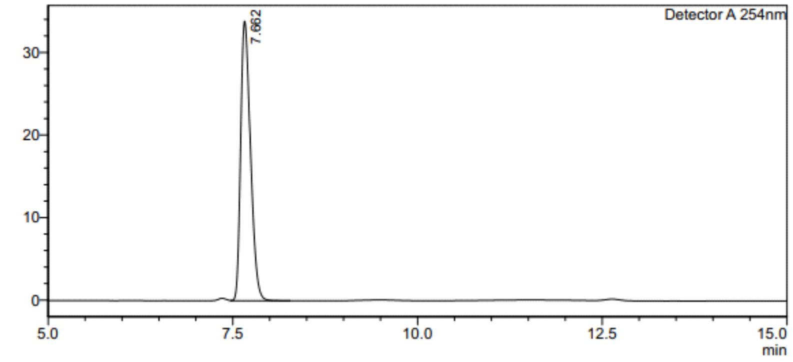

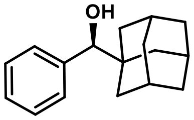

C26
> 99.9% ee

<Peak Table>

Detector A 254nm

Peak#	Ret. Time	Area	Height	Conc.	Unit	Mark	Name
1	7.662	312149	33859	100.000			
Total		312149	33859				

Supplementary Table 28. Crystal data and structure refinement for **D26**

CCDC Number	2017197
Empirical formula	$C_{27}H_{34}O_4$
Formula weight	422.54
Temperature/K	100
Crystal system	monoclinic
Space group	$P2_1$
a/Å	11.0212(4)
b/Å	6.7112(2)
c/Å	15.7585(5)
α/°	90
β/°	107.5500(10)
γ/°	90
Volume/Å^3	1111.33(6)
Z	2
ρ_{calc}g/cm^3	1.263
μ/mm^{-1}	0.660
F(000)	456.0
Crystal size/mm^3	0.32 × 0.32 × 0.29
Radiation	CuKα (λ = 1.54178)
2Θ range for data collection/°	5.882 to 137.286
Index ranges	$-13 \leq h \leq 13$, $-8 \leq k \leq 8$, $-18 \leq l \leq 19$
Reflections collected	7058
Independent reflections	7058 [R_{int} = ?, R_{sigma} = 0.0352]
Data/restraints/parameters	7058/1/285
Goodness-of-fit on F^2	1.049
Final R indexes [I>=2σ (I)]	R_1 = 0.0396, wR_2 = 0.1154
Final R indexes [all data]	R_1 = 0.0469, wR_2 = 0.1344
Largest diff. peak/hole / e Å^{-3}	0.20/-0.15
Flack parameter	-0.07(10)

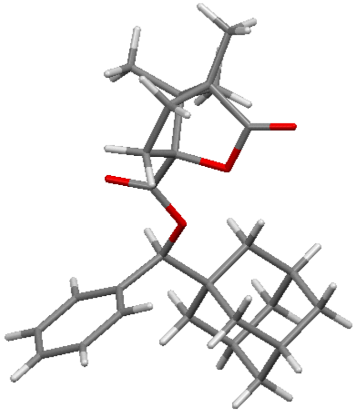

Supplementary Figure 27. The single crystal configuration of **D26** is demonstrated in a capped sticks mode.

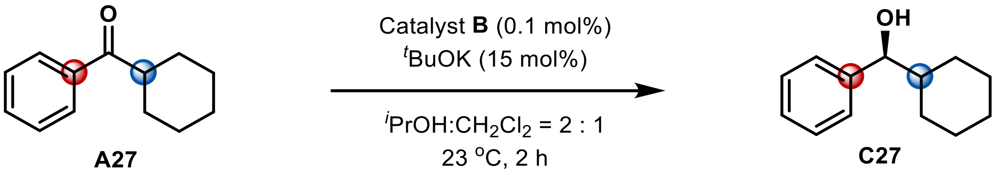

Following the general procedure, **C27** (32.2 mg, 85% yield, 92% ee) was obtained as white solid. Its major pure enantiomer was purified by chiral preparative HPLC (OD-H, 2.0*25 cm, 5μm) and the *X*-ray crystal of the optically pure enantiomer (100% ee) was obtained by liquid/liquid diffusion with dichloromethane / *n*-hexane system.

^{1}H NMR (400 MHz, CDCl$_3$): δ 7.38 – 7.22 (m, 5H), 4.35 (d, *J* = 7.2 Hz, 1H), 1.99 – 1.96 (m, 1H), 1.85 (bs, 1H), 1.80 – 1.73 (m, 1H), 1.70 – 1.56 (m, 3H), 1.41 – 1.33 (m, 1H), 1.27 – 0.99 (m, 4H), 0.97 – 0.84 (m, 1H) ppm
^{13}C NMR (101 MHz, CDCl$_3$): δ 143.6, 128.1, 127.4, 126.6, 79.3, 44.9, 29.3, 28.8, 26.4, 26.1, 26.0 ppm
HRMS (ESI+): calculated for $C_{13}H_{17}$ $[M+H-H_2O]^+$: 173.1325, found 173.1324.
HPLC (OD-H, 0.46*25 cm, 5μm, hexane / ethanol = 97/3, flow 1 mL/min, detection at 210 nm) retention time = 6.946 min (minor) and 7.350 min (major).

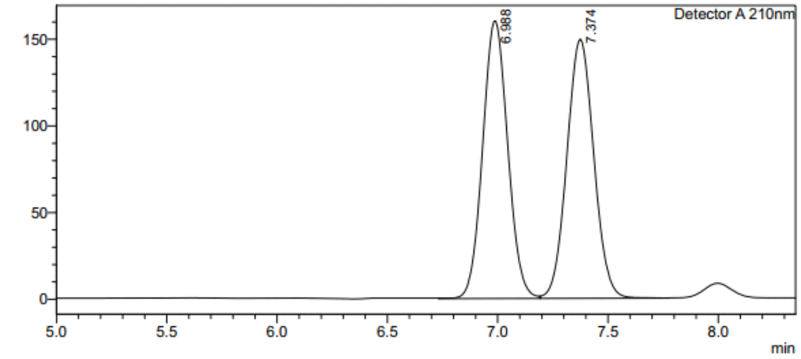

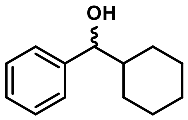

(±)-**C27**

<Peak Table>

Detector A 210nm

Peak#	Ret. Time	Area	Height	Conc.	Unit	Mark	Name
1	6.988	1268375	160176	49.983			
2	7.374	1269255	149596	50.017		V	
Total		2537630	309772				

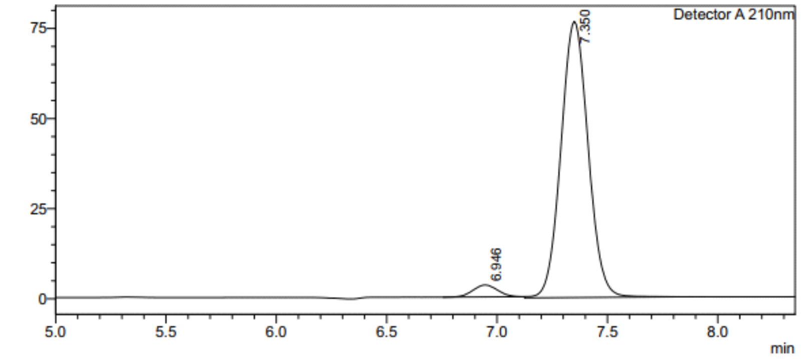

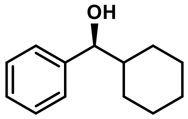

C27
85% yield, 92% ee
obtained by catalyst **B**

<Peak Table>

Detector A 210nm

Peak#	Ret. Time	Area	Height	Conc.	Unit	Mark	Name
1	6.946	25894	3309	3.790		M	
2	7.350	657321	76644	96.210			
Total		683215	79953				

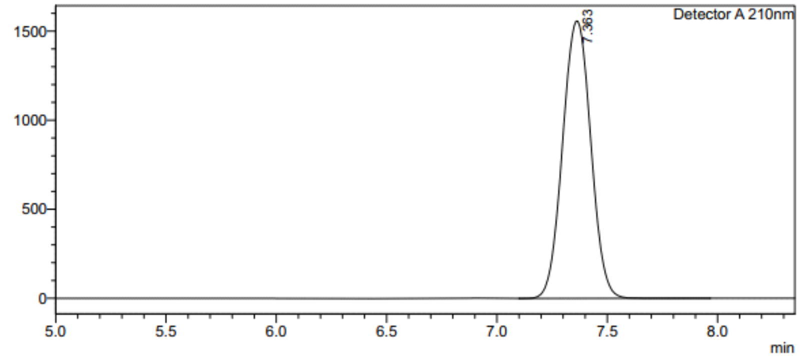

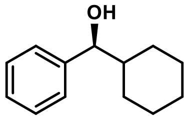

C27
> 99.9% ee

<Peak Table>

Detector A 210nm

Peak#	Ret. Time	Area	Height	Conc.	Unit	Mark	Name
1	7.363	13881305	1556947	100.000			
Total		13881305	1556947				

Supplementary Table 29. Crystal data and structure refinement for **C27**

Identification code	2183980
Empirical formula	$C_{13}H_{18}O$
Formula weight	190.27
Temperature/K	100.0(2)
Crystal system	monoclinic
Space group	$P2_1$
a/Å	5.3491(6)
b/Å	16.5493(19)
c/Å	12.3422(14)
α/°	90
β/°	93.991(4)
γ/°	90
Volume/Å^3	1089.9(2)
Z	4
ρ_{calc}g/cm^3	1.160
μ/mm^{-1}	0.545
F(000)	416.0
Crystal size/mm^3	0.33 × 0.31 × 0.24
Radiation	CuKα (λ = 1.54178)
2Θ range for data collection/°	7.18 to 144.522
Index ranges	$-6 \le h \le 6$, $-20 \le k \le 20$, $-15 \le l \le 15$
Reflections collected	29898
Independent reflections	4273 [R_{int} = 0.0605, R_{sigma} = 0.0338]
Data/restraints/parameters	4273/1/256
Goodness-of-fit on F^2	1.052
Final R indexes [I>=2σ (I)]	R_1 = 0.0305, wR_2 = 0.0804
Final R indexes [all data]	R_1 = 0.0307, wR_2 = 0.0806
Largest diff. peak/hole / e Å^{-3}	0.19/-0.14
Flack parameter	0.08(6)

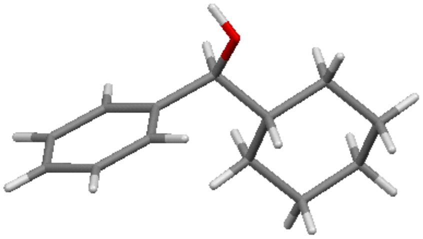

Supplementary Figure 28. The single crystal configuration of **C27** is demonstrated in a capped sticks mode.

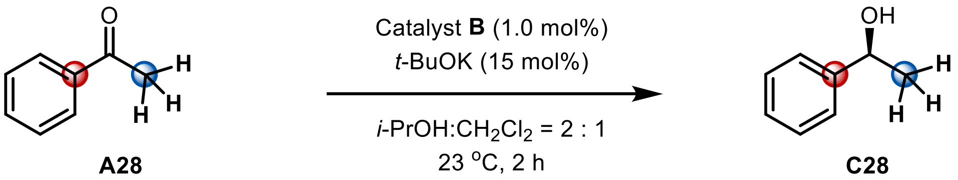

Following the general procedure, **C28** (21.5 mg, 88% yield, 86% ee) was obtained as colorless liquid.

^{1}H NMR (400 MHz, CDCl$_3$): δ 7.41 – 7.30 (m, 4H), 7.29 – 7.23 (m, 1H), 4.88 (q, J = 6.5 Hz, 1H), 1.89 (bs, 1H), 1.49 (d, J = 6.5 Hz, 3H). ppm
^{13}C NMR (101 MHz, CDCl$_3$): δ 145.8, 128.5, 127.4, 125.4, 70.4, 25.1 ppm
HRMS (ESI+): calculated for C_8H_9 [M+H-H_2O]$^+$: 105.0699, found 105.0702.
HPLC (OD-H, 0.46*25 cm, 5μm, hexane / ethanol = 95/5, flow 1 mL/min, detection at 210 nm) retention time = 6.838 min (minor) and 7.668 min (major).

The major enantiomer of **C28** (86% ee) was purified by chiral preparative HPLC (AD-H, 2.0*25 cm, 5μm). Because we were unable to grow *X*-ray qualified crystal of the optically pure **C28** (100% ee), we derivatize it with (1*S*)-(-)-camphanic acid chloride to afford **D28** as a single diastereomer. Then the *X*-ray crystal of the optically pure **D28** was obtained by liquid/liquid diffusion with CH_2Cl_2/hexane system.

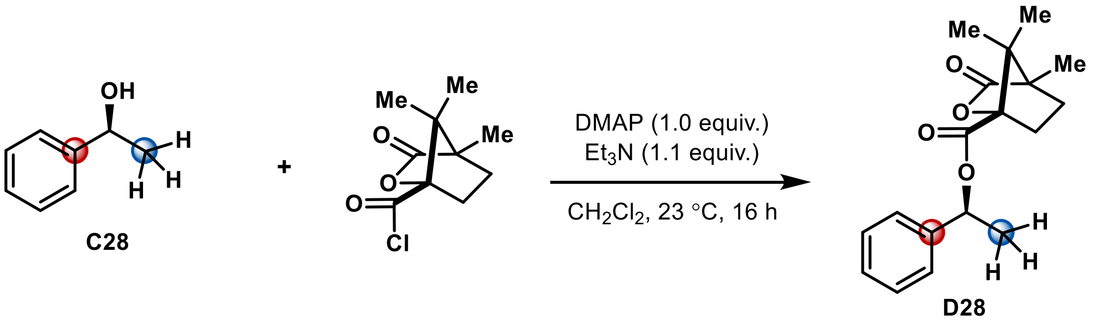

^{1}H NMR (600 MHz, CDCl$_3$): δ 7.41 – 7.33 (m, 4H), 7.31 (t, J = 7.2 Hz, 1H), 6.03 (q, J = 6.6 Hz, 1H), 2.43 – 2.38 (m, 1H), 2.04 – 1.99 (m, 1H), 1.93 – 1.88 (m, 1H), 1.70 – 1.60 (m, 1H), 1.60 (d, J = 6.6 Hz, 3H), 1.11 (s, 3H), 1.02 (s, 3H), 0.93 (s, 3H) ppm
^{13}C NMR (151 MHz, CDCl$_3$): δ 178.3, 166.8, 140.7, 128.6, 128.2, 126.2, 91.0, 73.9, 54.8, 54.2, 30.5, 28.9, 22.3, 16.8, 16.6, 9.7 ppm
HRMS (ESI+): calculated for $C_{18}H_{23}O_4$ [M+Na]$^+$: 303.1591, found 303.1587.

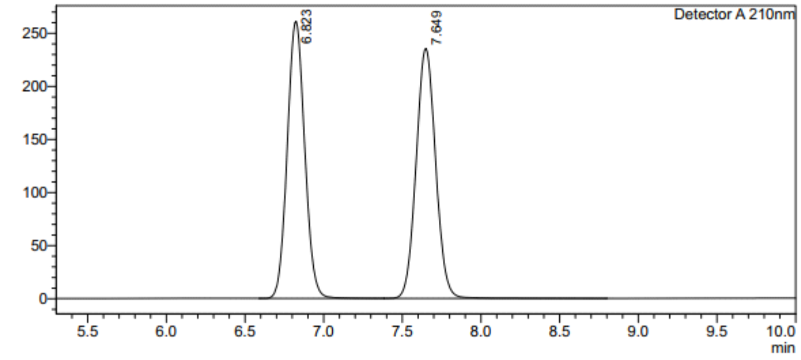

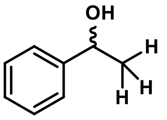

(±)-**C28**

<Peak Table>

Detector A 210nm

Peak#	Ret. Time	Area	Height	Conc.	Unit	Mark	Name
1	6.823	2013643	261186	49.762			
2	7.649	2032879	235625	50.238		V	
Total		4046522	496811				

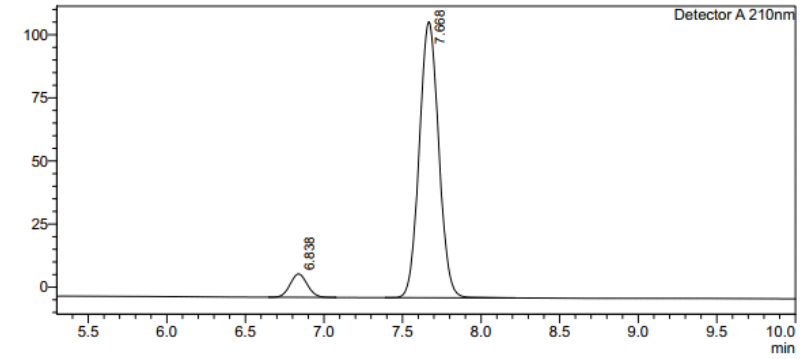

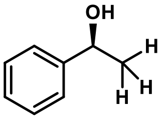

C28
88% yield, 86% ee
obtained by catalyst **B**

<Peak Table>

Detector A 210nm

Peak#	Ret. Time	Area	Height	Conc.	Unit	Mark	Name
1	6.838	70710	9242	7.020		M	
2	7.668	936522	109374	92.980		M	
Total		1007232	118616				

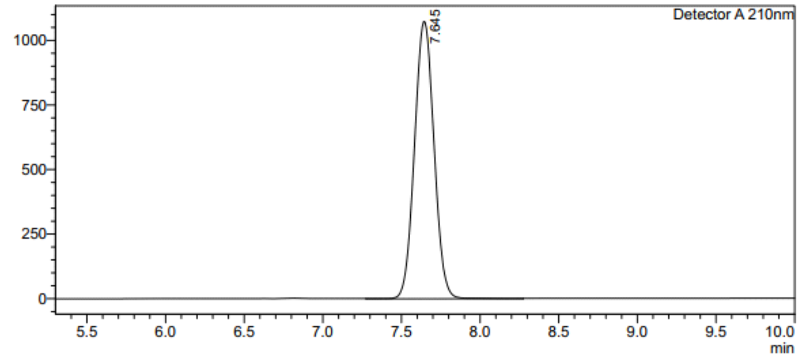

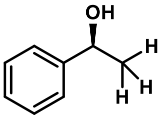

C28
> 99.9% ee

<Peak Table>

Detector A 210nm

Peak#	Ret. Time	Area	Height	Conc.	Unit	Mark	Name
1	7.645	9311173	1074560	100.000			
Total		9311173	1074560				

Supplementary Table 30. Crystal data and structure refinement for **D28**

Identification code	2255377
Empirical formula	$C_{18}H_{22}O_4$
Formula weight	302.35
Temperature/K	298.0(2)
Crystal system	orthorhombic
Space group	$P2_12_12_1$
a/Å	6.4629(6)
b/Å	12.1791(13)
c/Å	21.094(3)
α/°	90
β/°	90
γ/°	90
Volume/Å^3	1660.4(3)
Z	4
ρ_{calc}g/cm^3	1.210
μ/mm^{-1}	0.686
F(000)	648.0
Crystal size/mm^3	0.26 × 0.25 × 0.22
Radiation	CuKα (λ = 1.54178)
2Θ range for data collection/°	8.382 to 144.618
Index ranges	$-7 \leq h \leq 7$, $-15 \leq k \leq 15$, $-26 \leq l \leq 26$
Reflections collected	54315
Independent reflections	3248 [R_{int} = 0.0534, R_{sigma} = 0.0236]
Data/restraints/parameters	3248/0/204
Goodness-of-fit on F^2	1.035
Final R indexes [I>=2σ (I)]	R_1 = 0.0329, wR_2 = 0.0869
Final R indexes [all data]	R_1 = 0.0336, wR_2 = 0.0878
Largest diff. peak/hole / e Å^{-3}	0.18/-0.17
Flack parameter	0.07(4)

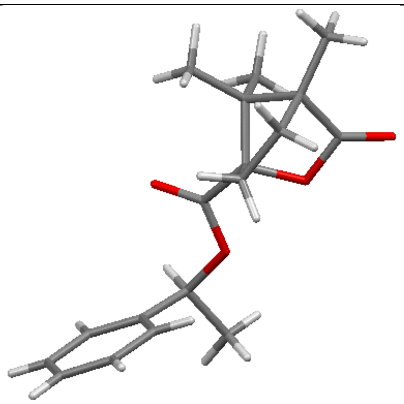

Supplementary Figure 29. The single crystal configuration of **D28** is demonstrated in a capped sticks mode.

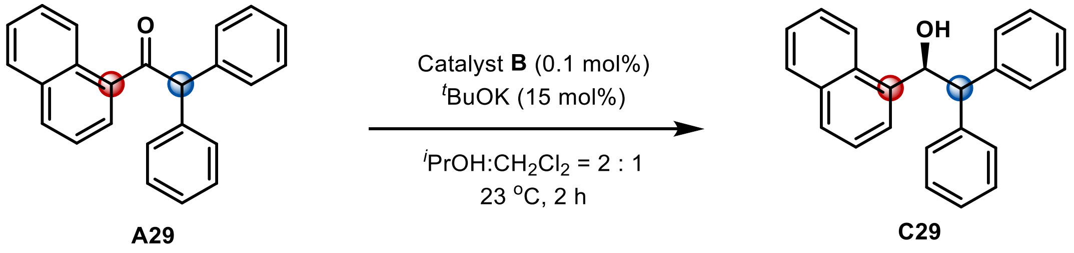

Following the general procedure, **C29** (49.9 mg, 77% yield, 98% ee) was obtained as white solid. Its major pure enantiomer was purified by chiral preparative HPLC (AD-H, 2.0*25 cm, 5μm) and the *X*-ray crystal of the optically pure enantiomer (100% ee) was obtained by liquid/liquid diffusion with CH_2Cl_2/hexane system.

^{1}H NMR (600 MHz, $CDCl_3$): δ 8.17 (d, *J* = 8.7 Hz, 1H), 7.87 (d, *J* = 8.7 Hz, 1H), 7.75 (d, *J* = 8.1 Hz, 1H), 7.53 – 7.48 (m, 2H), 7.35 – 7.23 (m, 7H), 7.22 (d, *J* = 7.5 Hz, 2H), 7.18 (t, *J* = 7.5 Hz, 2H), 7.12 (t, *J* = 7.5 Hz, 1H), 6.22 (t, *J* = 5.4 Hz, 1H), 4.67 (d, *J* = 6.4 Hz, 1H), 2.20 (d, *J* = 4.4 Hz, 1H) ppm
^{13}C NMR (151 MHz, $CDCl_3$): δ 142.1, 140.0, 137.9, 133.7, 130.7, 129.6, 128.9, 128.5, 128.4, 128.3, 128.1, 126.9, 126.4, 126.0, 125.4, 125.0, 124.7, 123.3, 73.3, 57.8 ppm
HRMS (ESI+): calculated for $C_{24}H_{19}$ $[M+H-H_2O]^+$: 307.1487, found 307.1486.
HPLC (AD-H, 0.46*25 cm, 5μm, hexane / ethanol = 90/10, flow 1 mL/min, detection at 254 nm) retention time = 11.065 min (major) and 13.909 min (minor).

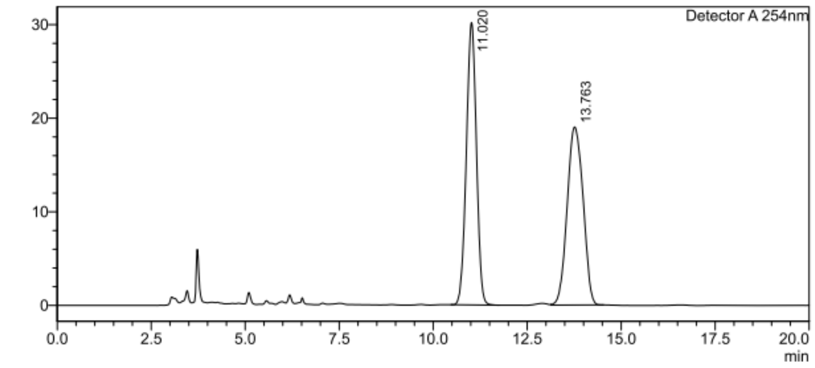

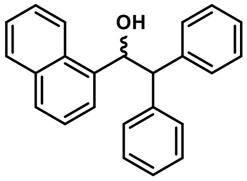

(±)-**C29**

<Peak Table>

Detector A 254nm

Peak#	Ret. Time	Area	Height	Conc.	Unit	Mark	Name
1	11.020	562681	30165	50.151			
2	13.763	559299	19024	49.849			
Total		1121980	49189				

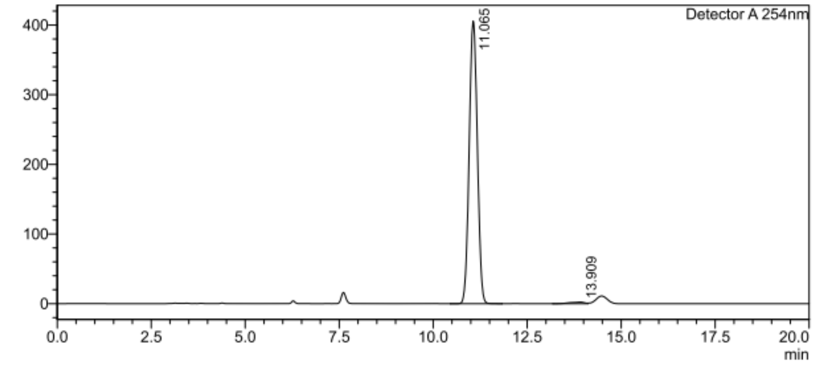

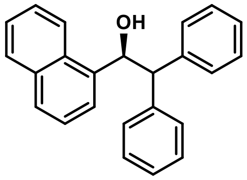

C29
77% yield, 98% ee
obtained by catalyst **B**

<Peak Table>

Detector A 254nm

Peak#	Ret. Time	Area	Height	Conc.	Unit	Mark	Name
1	11.065	6138805	405701	99.029			
2	13.909	60203	2252	0.971			
Total		6199009	407953				

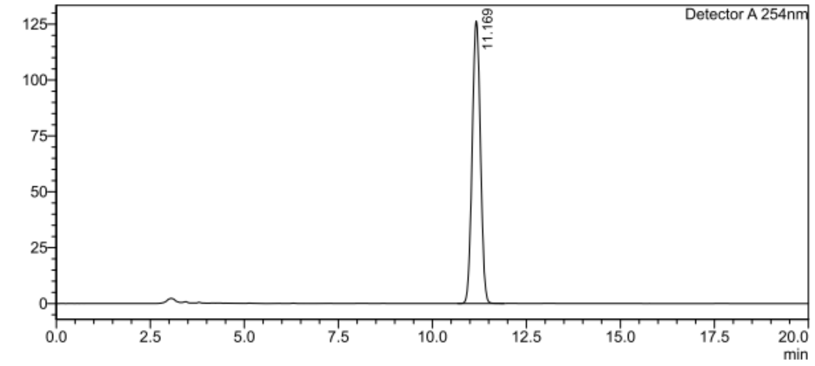

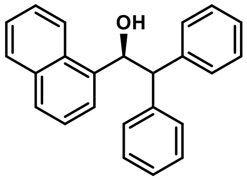

C29
> 99.9% ee

<Peak Table>

Detector A 254nm

Peak#	Ret. Time	Area	Height	Conc.	Unit	Mark	Name
1	11.169	1923565	126358	100.000			
Total		1923565	126358				

Supplementary Table 31. Crystal data and structure refinement for **C29**

CCDC Number	2017205
Empirical formula	$C_{24}H_{20}O$
Formula weight	324.40
Temperature/K	100
Crystal system	orthorhombic
Space group	$P2_12_12_1$
a/Å	5.8841(7)
b/Å	14.2509(17)
c/Å	20.523(2)
α/°	90
β/°	90
γ/°	90
Volume/Å^3	1721.0(4)
Z	4
ρ_{calc}g/cm^3	1.252
μ/mm^{-1}	0.575
F(000)	688.0
Crystal size/mm^3	0.36 × 0.28 × 0.24
Radiation	CuKα (λ = 1.54178)
2Θ range for data collection/°	7.552 to 137.402
Index ranges	$-7 \le h \le 7$, $-17 \le k \le 17$, $-24 \le l \le 24$
Reflections collected	23812
Independent reflections	3167 [R_{int} = 0.0684, R_{sigma} = 0.0364]
Data/restraints/parameters	3167/0/228
Goodness-of-fit on F^2	1.046
Final R indexes [I>=2σ (I)]	R_1 = 0.0299, wR_2 = 0.0738
Final R indexes [all data]	R_1 = 0.0305, wR_2 = 0.0742
Largest diff. peak/hole / e Å^{-3}	0.18/-0.14
Flack parameter	0.19(14)

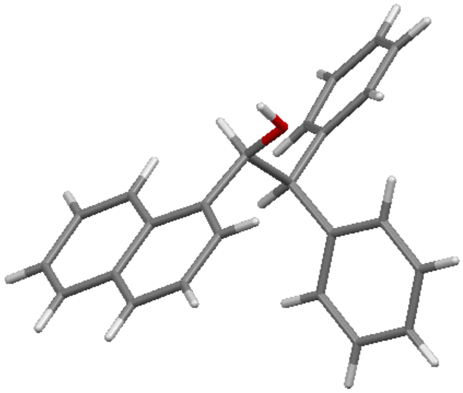

Supplementary Figure 30. The single crystal configuration of **C29** is demonstrated in a capped sticks mode.

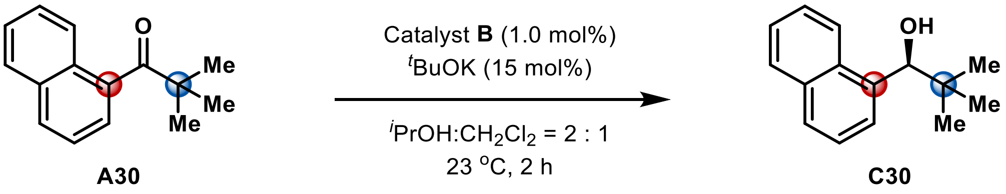

Following the general procedure, **C30** (37.6 mg, 86% yield, 89% ee) was obtained as colorless liquid.

¹H NMR (600 MHz, $CDCl_3$): δ 8.17 (s, 1H), 7.86 (d, *J* = 8.0 Hz, 1H), 7.79 (d, *J* = 8.0 Hz, 1H), 7.71 (d, *J* = 6.8 Hz, 1H), 7.54 – 7.44 (m, 3H), 5.43 (s, 1H), 1.91 (s, 1H), 1.01 (s, 9H) ppm
¹³C NMR (151 MHz, $CDCl_3$): δ 138.8, 133.4, 131.8, 128.8, 127.8, 125.5, 125.1, 125.0, 123.9, 37.0, 26.5 ppm
HRMS (ESI+): calculated for $C_{15}H_{17}$ $[M+H-H_2O]^+$: 197.1325, found 197.1329.
HPLC (AD-H, 0.46*25 cm, 5μm, hexane / ethanol = 90/10, flow 1 mL/min, detection at 254 nm) retention time = 10.401 min (major) and 11.261 min (minor).

The major enantiomer of **C30** (89% ee) was purified by chiral preparative HPLC (AD-H, 2.0*25 cm, 5μm). Because we were unable to grow *X*-ray qualified crystal of the optically pure **C30** (100% ee), we derivatize it with (1*S*)-(-)-camphanic acid chloride to afford **D30** as a single diastereomer. Then the *X*-ray crystal of the optically pure **D30** was obtained by liquid/liquid diffusion with CH_2Cl_2/hexane system.

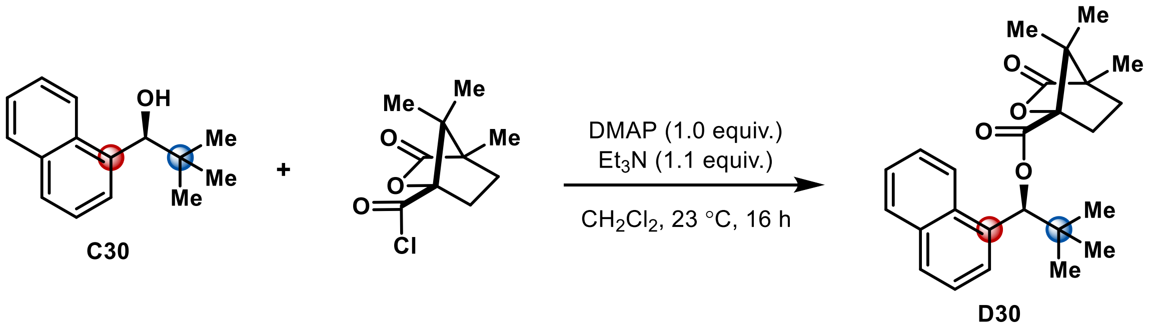

¹H NMR (600 MHz, $CDCl_3$): δ 8.21 (d, *J* = 8.4 Hz, 1H), 7.85 (d, *J* = 8.4 Hz, 1H), 7.80 (d, *J* = 8.4 Hz, 1H), 7.59 (d, *J* = 7.2 Hz, 1H), 7.54 (t, *J* = 7.6 Hz, 1H), 7.48 (d, *J* = 7.2 Hz, 1H), 7.46 (d, *J* = 7.4 Hz, 1H), 6.61 (s, 1H), 2.39 – 2.28 (m, 1H), 2.01 –1.94 (m, 1H), 1.93 – 1.87 (m, 1H), 1.71 – 1.65 (m, 1H), 1.12 (s, 3H), 1.08 (s, 3H), 1.03 (s, 9H), 0.99 (s, 3H) ppm
¹³C NMR (151 MHz, $CDCl_3$): δ 178.5, 167.0, 134.4, 133.3, 131.8, 128.9, 128.5, 126.2, 125.4, 125.4, 124.7, 123.4, 91.3, 78.7, 54.9, 54.1, 36.3, 30.8, 29.0, 26.5, 16.8, 16.8, 9.7 ppm
HRMS (ESI+): calculated for $C_{25}H_{30}NaO_4$ $[M+Na]^+$: 417.2036, found 417.2035.

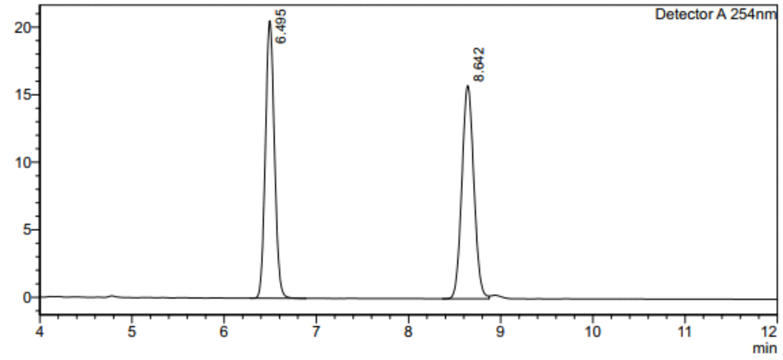

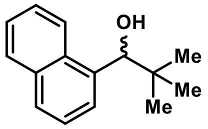

(±)-**C30**

<Peak Table>

Detector A 254nm

Peak#	Ret. Time	Area	Height	Conc.	Unit	Mark	Name
1	6.495	140354	20556	49.539			
2	8.642	142964	15782	50.461			
Total		283318	36337				

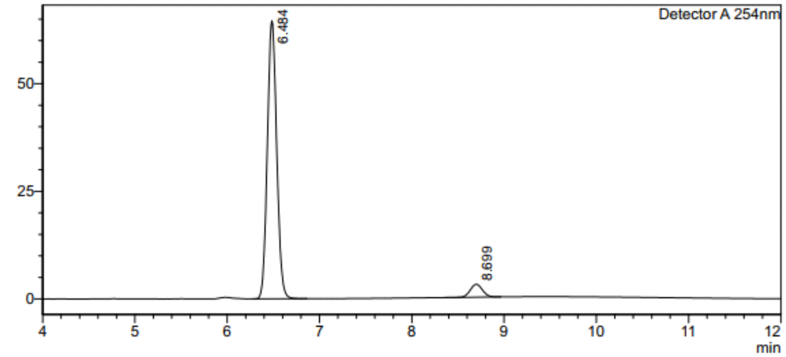

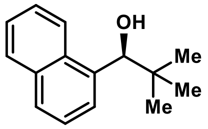

C30
88% yield, 89% ee
obtained by catalyst **B**

<Peak Table>

Detector A 254nm

Peak#	Ret. Time	Area	Height	Conc.	Unit	Mark	Name
1	6.484	455980	64635	94.283			
2	8.699	27647	2989	5.717			
Total		483627	67624				

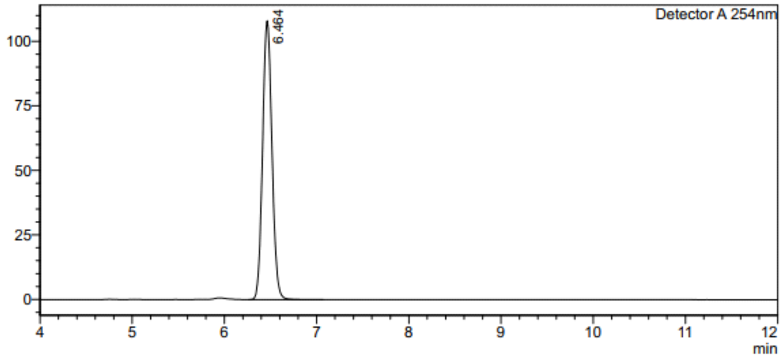

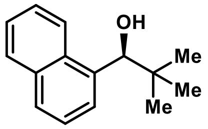

C30
> 99.9% ee

<Peak Table>

Detector A 254nm

Peak#	Ret. Time	Area	Height	Conc.	Unit	Mark	Name
1	6.464	764783	108049	100.000			
Total		764783	108049				

Supplementary Table 32. Crystal data and structure refinement for **D30**

CCDC Number	2017173
Empirical formula	$C_{25}H_{30}O_4$
Formula weight	394.49
Temperature/K	100
Crystal system	orthorhombic
Space group	$P2_12_12_1$
a/Å	11.1524(3)
b/Å	13.7927(4)
c/Å	14.3217(4)
α/°	90
β/°	90
γ/°	90
Volume/Å^3	2202.99(11)
Z	4
ρ_{calc}g/cm^3	1.189
μ/mm^{-1}	0.632
F(000)	848.0
Crystal size/mm^3	0.42 × 0.31 × 0.28
Radiation	CuKα (λ = 1.54178)
2Θ range for data collection/°	8.9 to 137.114
Index ranges	$-13 \leq h \leq 13$, $-16 \leq k \leq 16$, $-17 \leq l \leq 17$
Reflections collected	17064
Independent reflections	4049 [R_{int} = 0.0318, R_{sigma} = 0.0242]
Data/restraints/parameters	4049/0/269
Goodness-of-fit on F^2	1.039
Final R indexes [I>=2σ (I)]	R_1 = 0.0259, wR_2 = 0.0652
Final R indexes [all data]	R_1 = 0.0265, wR_2 = 0.0657
Largest diff. peak/hole / e Å^{-3}	0.17/-0.11
Flack parameter	-0.01(6)

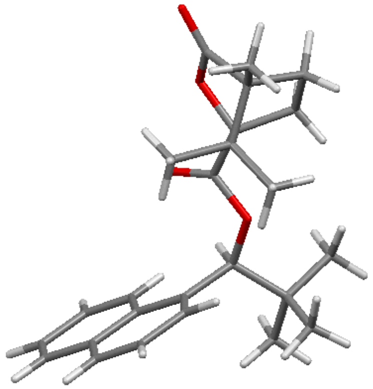

Supplementary Figure 31. The single crystal configuration of **D30** is demonstrated in a capped sticks mode.

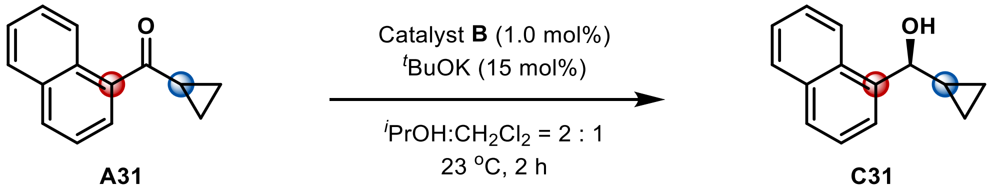

Following the general procedure, **C31** (24.9 mg, 63% yield, 88% ee) was obtained as colorless liquid.

^{1}H NMR (600 MHz, CDCl$_3$): δ 8.26 (d, *J* = 8.4 Hz, 1H), 7.88 (d, *J* = 7.5 Hz, 1H), 7.81 (d, *J* = 8.4 Hz, 1H), 7.69 (d, *J* = 7.5 Hz, 1H), 7.57 – 7.42 (m, 3H), 4.86 (d, *J* = 7.6 Hz, 1H), 2.14 (s, 1H), 1.60 – 1.49 (m, 1H), 0.73 – 0.65 (m, 1H), 0.59 – 0.54 (m, 1H), 0.50 (td, *J* = 9.8, 5.0 Hz, 1H), 0.39 (td, *J* = 9.8, 5.0 Hz, 1H) ppm
^{13}C NMR (151 MHz, CDCl$_3$): δ 138.9, 133.9, 131.0, 128.7, 128.2, 125.9, 125.5, 125.3, 124.0, 123.87, 75.0, 17.7, 3.98, 2.8 ppm
HRMS (ESI+): calculated for $C_{14}H_{13}$ [M+H-H$_2$O]$^+$: 181.1012, found 181.1015.
HPLC (AD-H, 0.46*25 cm, 5μm, hexane / ethanol = 90/10, flow 1 mL/min, detection at 254 nm) retention time = 8.509 min (major) and 9.174 min (minor).

The major enantiomer of **C31** (88% ee) was purified by chiral preparative HPLC (AD-H, 2.0*25 cm, 5μm). Because we were unable to grow *X*-ray qualified crystal of the optically pure **C31** (100% ee), we derivatize it with (1*S*)-(-)-camphanic acid chloride to afford **D31** as a single diastereomer. Then the *X*-ray crystal of the optically pure **D31** was obtained by liquid/liquid diffusion with CH_2Cl_2/hexane system.

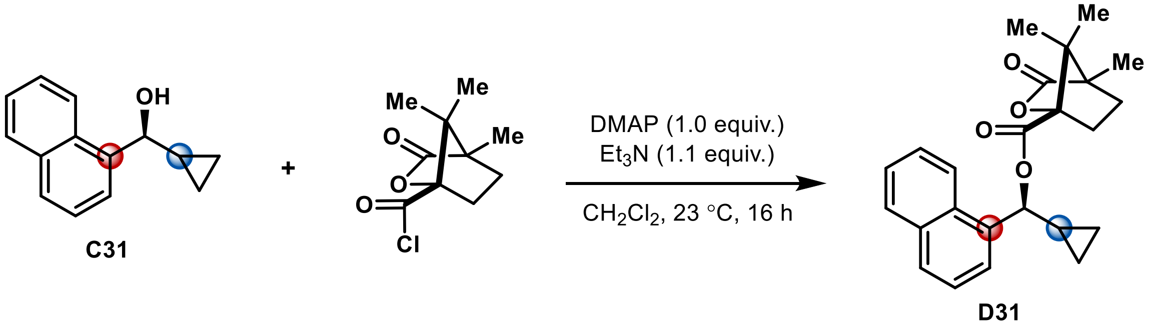

^{1}H NMR (600 MHz, CDCl$_3$): δ 8.17 (d, *J* = 8.4 Hz, 1H), 7.87 (d, *J* = 8.2 Hz, 1H), 7.83 (d, *J* = 8.2 Hz, 1H), 7.68 (d, *J* = 7.1 Hz, 1H), 7.54 (t, *J* = 7.1 Hz, 1H), 7.50 (t, *J* = 7.4 Hz, 1H), 7.48 (t, *J* = 7.4 Hz, 1H), 6.15 (d, *J* = 8.4 Hz, 1H), 2.43 – 2.36 (m, 1H), 2.02 – 1.95 (m, 1H), 1.92 – 1.85 (m, 1H), 1.69 – 1.63 (m, 2H), 1.10 (s, 3H), 0.98 (s, 3H), 0.95 (s, 3H), 0.72 – 0.66 (m, 1H), 0.63 – 0.53 (m, 2H), 0.47 – 0.41 (m, 1H) ppm
^{13}C NMR (151 MHz, CDCl$_3$): δ 178.4, 167.1, 134.8, 133.8, 130.7, 129.0, 128.9, 126.4, 125.7, 125.2, 125.2, 123.6, 91.2, 78.7, 54.8, 54.2, 30.6, 28.9, 16.7, 16.7, 16.0, 9.7, 4.7, 3.5 ppm
HRMS (ESI+): calculated for $C_{24}H_{26}NaO_4$ [M+Na]$^+$: 401.1723, found 401.1722.

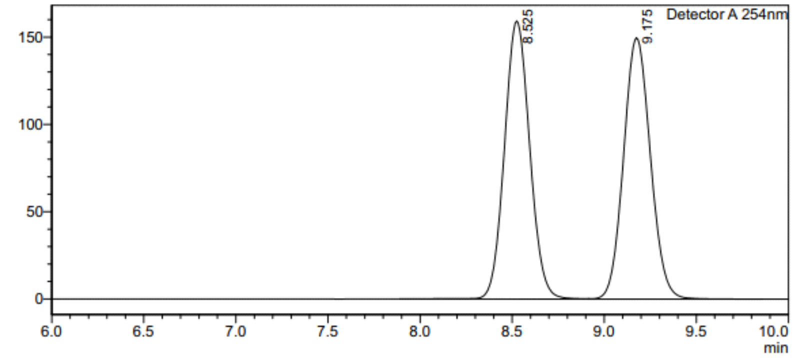

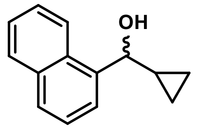

(±)-**C31**

<Peak Table>

Detector A 254nm

Peak#	Ret. Time	Area	Height	Conc.	Unit	Mark	Name
1	8.525	1521150	159343	50.062		V	
2	9.175	1517399	149690	49.938		V	
Total		3038549	309034				

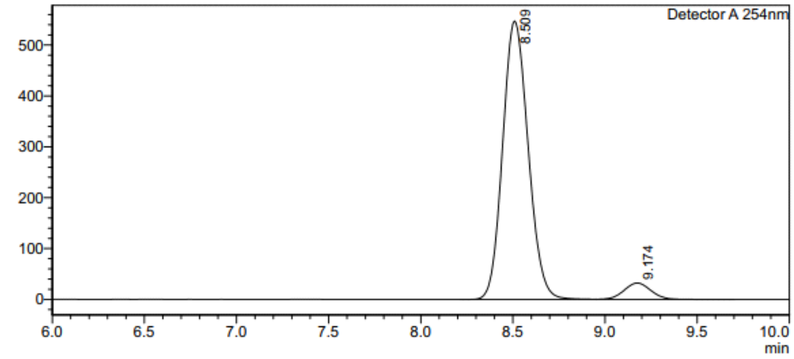

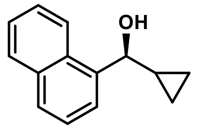

C31
63% yield, 88% ee
obtained by catalyst **B**

<Peak Table>

Detector A 254nm

Peak#	Ret. Time	Area	Height	Conc.	Unit	Mark	Name
1	8.509	5271426	547435	94.151			
2	9.174	327486	32173	5.849		V	
Total		5598912	579607				

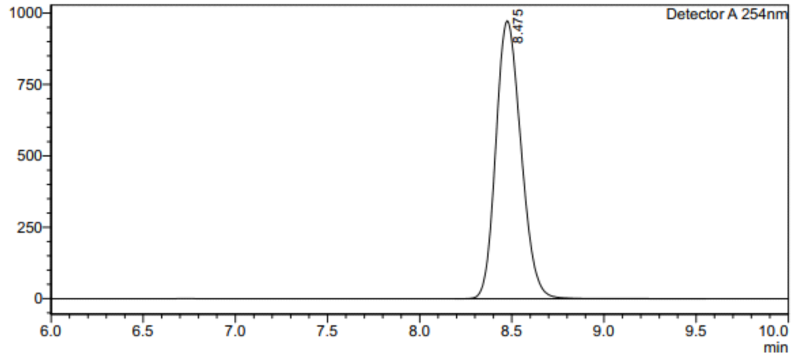

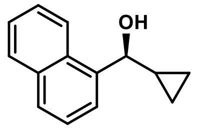

C31
> 99.9% ee

<Peak Table>

Detector A 254nm

Peak#	Ret. Time	Area	Height	Conc.	Unit	Mark	Name
1	8.475	9245934	973502	100.000			
Total		9245934	973502				

Supplementary Table 33. Crystal data and structure refinement for **D31**

CCDC Number	2018330
Empirical formula	$C_{24}H_{26}O_4$
Formula weight	378.45
Temperature/K	100
Crystal system	monoclinic
Space group	$P2_1$
a/Å	11.7092(3)
b/Å	6.9159(2)
c/Å	12.1078(4)
α/°	90
β/°	102.3500(10)
γ/°	90
Volume/Å^3	957.80(5)
Z	2
ρ_{calc}g/cm^3	1.312
μ/mm^{-1}	0.708
F(000)	404.0
Crystal size/mm^3	0.4 × 0.18 × 0.08
Radiation	CuKα (λ = 1.54178)
2Θ range for data collection/°	7.474 to 136.846
Index ranges	$-13 \le h \le 14$, $-8 \le k \le 8$, $-12 \le l \le 14$
Reflections collected	15965
Independent reflections	3487 [R_{int} = 0.0478, R_{sigma} = 0.0348]
Data/restraints/parameters	3487/1/257
Goodness-of-fit on F^2	1.035
Final R indexes [I>=2σ (I)]	R_1 = 0.0447, wR_2 = 0.1118
Final R indexes [all data]	R_1 = 0.0460, wR_2 = 0.1136
Largest diff. peak/hole / e Å^{-3}	0.24/-0.20
Flack parameter	-0.2(3)

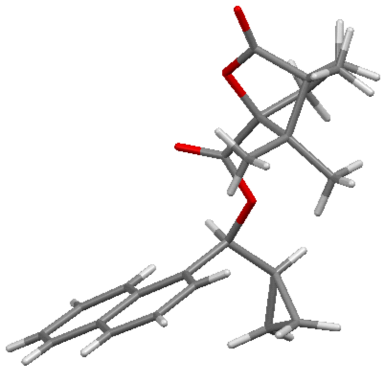

Supplementary Figure 32. The single crystal configuration of **D31** is demonstrated in a capped sticks mode.

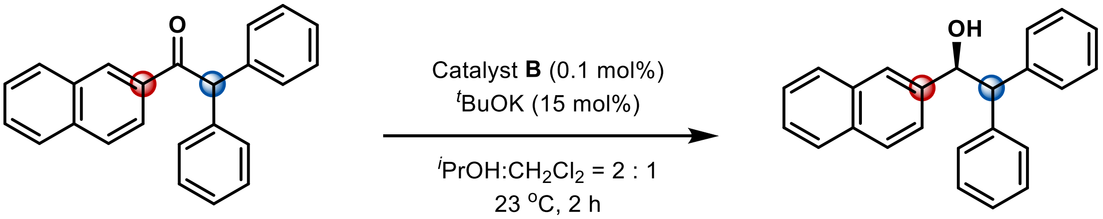

A32 **C32**

Following the general procedure, **C32** (63.5 mg, 98% yield, 94% ee) was obtained as white solid. Its major pure enantiomer was purified by chiral preparative HPLC (AD-H, 2.0*25 cm, 5μm) and the *X*-ray crystal of the optically pure enantiomer (100% ee) was obtained by liquid/liquid diffusion with CH_2Cl_2/hexane system.

^{1}H NMR (600 MHz, $CDCl_3$): δ 7.76 (d, *J* = 8.2 Hz, 1H), 7.73 – 7.69 (m, 2H), 7.64 (s, 1H), 7.44 – 7.40 (m, 4H), 7.34 (t, *J* = 7.3 Hz, 3H), 7.25 (t, *J* = 7.3 Hz, 1H), 7.15 (d, *J* = 7.4 Hz, 2H), 7.11 (t, *J* = 7.4 Hz, 2H), 7.05 (t, *J* = 7.4 Hz, 1H), 5.56 (dd, *J* = 8.6, 2.4 Hz, 1H), 4.36 (d, *J* = 8.6 Hz, 1H), 2.19 (d, *J* = 2.4 Hz, 1H) ppm
^{13}C NMR (151 MHz, $CDCl_3$): δ 141.4, 140.8, 139.7, 133.0, 132.9, 129.0, 128.8, 128.6, 128.3, 128.0, 127.7, 127.6, 127.0, 126.4, 126.0, 125.9, 125.7, 124.8, 76.9, 60.1 ppm
HRMS (ESI+): calculated for $C_{24}H_{19}$ $[M+H-H_2O]^+$: 307.1487, found 307.1486.
HPLC (AD-H, 0.46*25 cm, 5μm, hexane / ethanol = 85/15, flow 1 mL/min, detection at 254 nm) retention time = 12.916 min (major) and 18.002 min (minor).

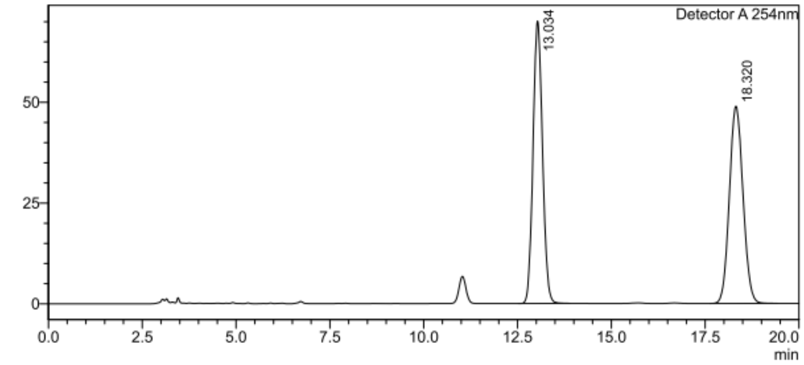

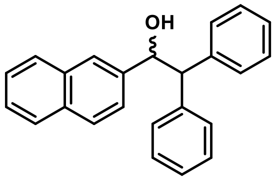

(±)-**C32**

<Peak Table>

Detector A 254nm

Peak#	Ret. Time	Area	Height	Conc.	Unit	Mark	Name
1	13.034	1232703	70061	49.925			
2	18.320	1236404	48913	50.075			
Total		2469107	118973				

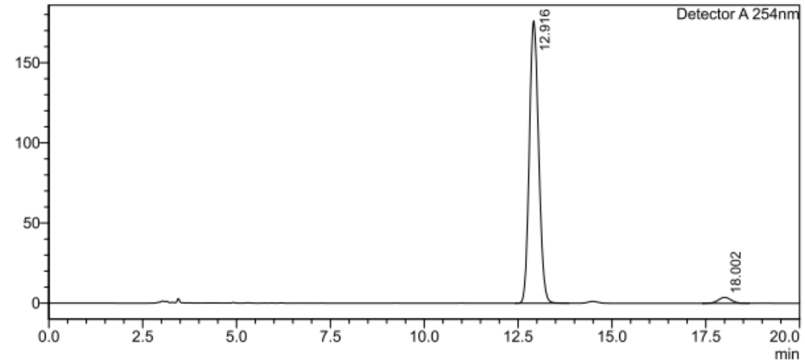

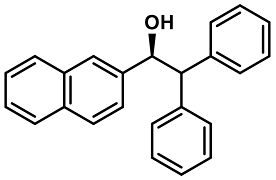

C32
98% yield, 94% ee
obtained by catalyst **B**

<Peak Table>

Detector A 254nm

Peak#	Ret. Time	Area	Height	Conc.	Unit	Mark	Name
1	12.916	3040769	176058	97.168			
2	18.002	88622	3687	2.832			
Total		3129391	179746				

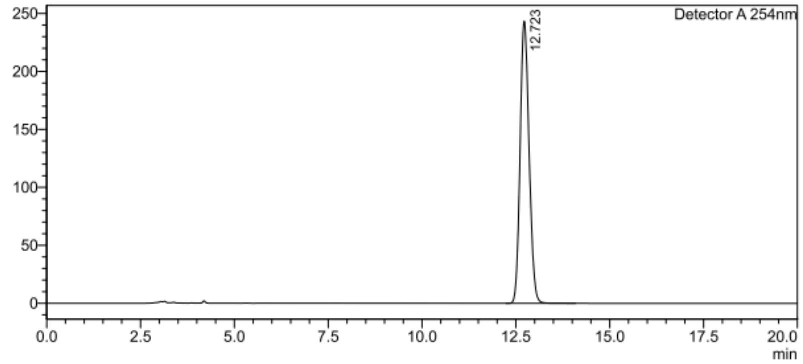

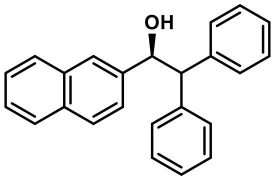

C32
> 99.9% ee

<Peak Table>

Detector A 254nm

Peak#	Ret. Time	Area	Height	Conc.	Unit	Mark	Name
1	12.723	4052129	243424	100.000		S	
Total		4052129	243424				

Supplementary Table 34. Crystal data and structure refinement for **C32**

CCDC Number	2017130
Empirical formula	$C_{24}H_{20}O$
Formula weight	324.40
Temperature/K	100
Crystal system	monoclinic
Space group	$P2_1$
a/Å	5.7661(3)
b/Å	17.4438(8)
c/Å	16.8996(8)
α/°	90
β/°	92.892(2)
γ/°	90
Volume/Å^3	1697.64(14)
Z	4
ρ_{calc}g/cm^3	1.269
μ/mm^{-1}	0.583
F(000)	688.0
Crystal size/mm^3	0.32 × 0.32 × 0.29
Radiation	CuKα (λ = 1.54178)
2Θ range for data collection/°	5.236 to 137.168
Index ranges	$-6 \le h \le 6$, $-21 \le k \le 21$, $-20 \le l \le 20$
Reflections collected	57552
Independent reflections	6177 [R_{int} = 0.0423, R_{sigma} = 0.0211]
Data/restraints/parameters	6177/1/453
Goodness-of-fit on F^2	1.061
Final R indexes [I>=2σ (I)]	R_1 = 0.0308, wR_2 = 0.0787
Final R indexes [all data]	R_1 = 0.0317, wR_2 = 0.0795
Largest diff. peak/hole / e Å^{-3}	0.17/-0.21
Flack parameter	-0.13(6)

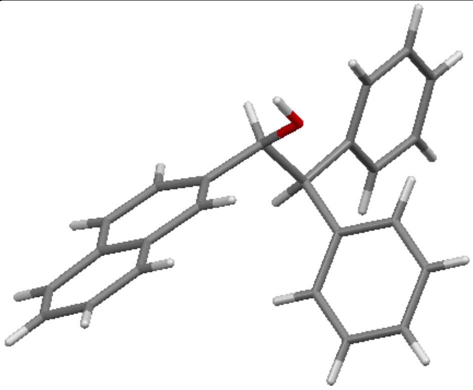

Supplementary Figure 33. The single crystal configuration of **C32** is demonstrated in a capped sticks mode.

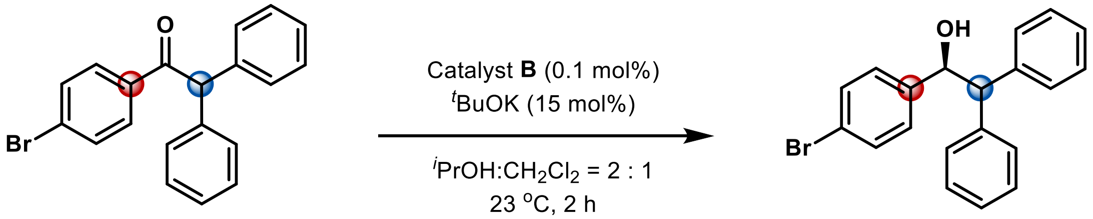

Following the general procedure, **C33** (59.8 mg, 85% yield, 95% ee) was obtained as white solid. Its major pure enantiomer was purified by chiral preparative HPLC (AD-H, 2.0*25 cm, 5μm) and the *X*-ray crystal of the optically pure enantiomer (100% ee) was obtained by liquid/liquid diffusion with CH_2Cl_2/hexane system.

^{1}H NMR (600 MHz, $CDCl_3$): δ 7.40 (d, *J* = 7.5 Hz, 2H), 7.37 – 7.32 (m, 4H), 7.28 – 7.25 (m, 2H), 7.18 – 7.14 (m, 2H), 7.13 – 7.10 (m, 3H), 7.07 (d, *J* = 8.4 Hz, 2H), 5.36 (dd, *J* = 8.8, 2.4 Hz, 1H), 4.17 (d, *J* = 8.8 Hz, 1H), 2.14 (d, *J* = 2.4 Hz, 1H) ppm
^{13}C NMR (151 MHz, $CDCl_3$): δ 141.2, 141.1, 140.4, 131.1, 128.9, 128.8, 128.6, 128.5, 128.4, 127.1, 126.6, 121.4, 76.2, 60.4 ppm
HRMS (ESI+): calculated for $C_{20}H_{16}Br$ $[M+H-H_2O]^+$: 335.0435, found 335.0435.
HPLC (AD-H, 0.46*25 cm, 5μm, hexane / ethanol = 85/15, flow 1 mL/min, detection at 254 nm) retention time = 10.889 min (major) and 15.754 min (minor).

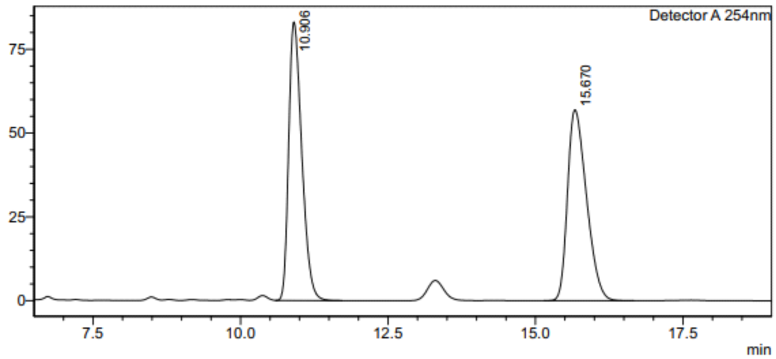

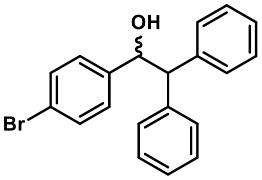

(±)-**C33**

<Peak Table>

Detector A 254nm

Peak#	Ret. Time	Area	Height	Conc.	Unit	Mark	Name
1	10.906	1283961	83137	50.149			
2	15.670	1276317	56971	49.851			
Total		2560278	140109				

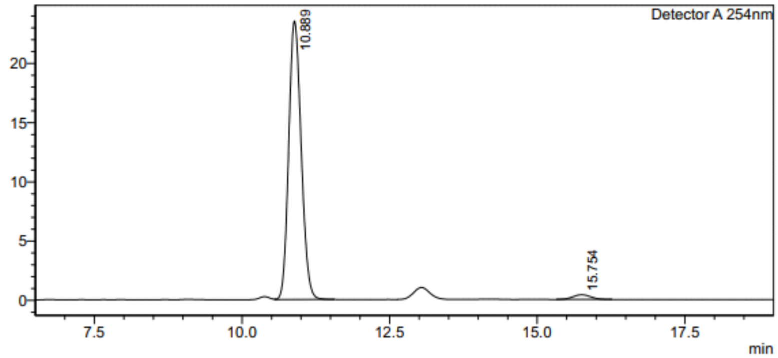

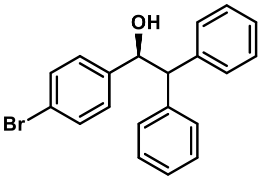

C33
85% yield, 95% ee
obtained by catalyst **B**

<Peak Table>

Detector A 254nm

Peak#	Ret. Time	Area	Height	Conc.	Unit	Mark	Name
1	10.889	345589	23498	97.628			
2	15.754	8397	396	2.372			
Total		353986	23895				

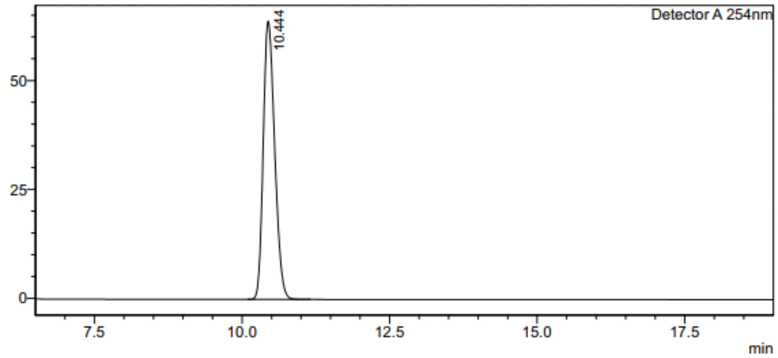

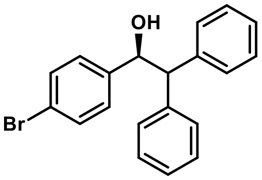

C33
> 99.9% ee

<Peak Table>

Detector A 254nm

Peak#	Ret. Time	Area	Height	Conc.	Unit	Mark	Name
1	10.444	861050	63907	100.000			
Total		861050	63907				

Supplementary Table 35. Crystal data and structure refinement for **C33**

CCDC Number	2017175
Empirical formula	$C_{20}H_{17}BrO$
Formula weight	353.24
Temperature/K	100
Crystal system	monoclinic
Space group	$P2_1$
a/Å	5.7069(2)
b/Å	16.9735(6)
c/Å	15.9483(6)
α/°	90
β/°	97.400(2)
γ/°	90
Volume/Å^3	1531.98(10)
Z	4
ρ_{calc}g/cm^3	1.532
μ/mm^{-1}	2.682
F(000)	720.0
Crystal size/mm^3	0.41 × 0.32 × 0.29
Radiation	MoKα (λ = 0.71073)
2Θ range for data collection/°	4.8 to 61.338
Index ranges	$-8 \le h \le 8$, $-24 \le k \le 24$, $-22 \le l \le 22$
Reflections collected	36672
Independent reflections	9445 [R_{int} = 0.0478, R_{sigma} = 0.0434]
Data/restraints/parameters	9445/1/399
Goodness-of-fit on F^2	1.031
Final R indexes [I>=2σ (I)]	R_1 = 0.0363, wR_2 = 0.0838
Final R indexes [all data]	R_1 = 0.0435, wR_2 = 0.0868
Largest diff. peak/hole / e Å^{-3}	0.89/-0.53
Flack parameter	0.010(7)

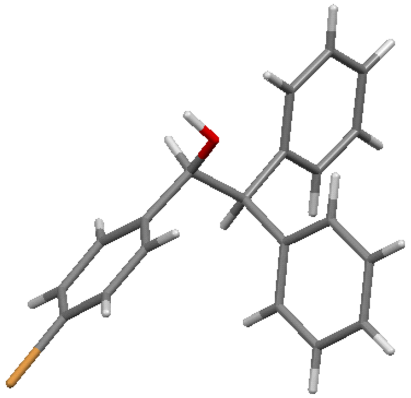

Supplementary Figure 34. The single crystal configuration of **C33** is demonstrated in a capped sticks mode.

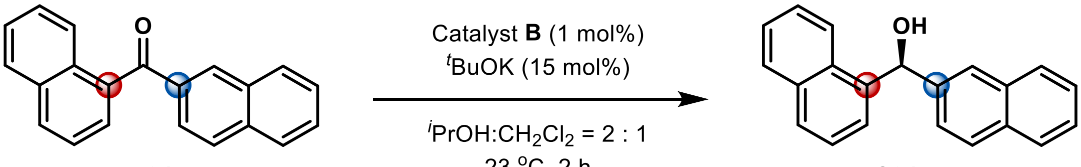

Following the general procedure, **C34** (47.7 mg, 84% yield, 83% ee) was obtained as white solid. The retention time in HPLC of the major enantiomer is consistent with that obtained by Ru-catalyst **B2,** and the *X*-ray crystal of the optically pure major enantiomer (100% ee) has been previously reported[2].

^{1}H NMR (400 MHz, $CDCl_3$): δ 8.12 (d, *J* = 7.8 Hz, 1H), 7.94 (s, 1H), 7.92 – 7.75 (m, 5H), 7.66 (d, *J* = 7.2 Hz, 1H), 7.56 – 7.37 (m, 6H), 6.71 (d, *J* = 3.6 Hz, 1H), 2.49 (d, *J* = 3.6 Hz, 1H) ppm
^{13}C NMR (101 MHz, $CDCl_3$): δ 140.5, 138.6, 134.0, 133.3, 132.9, 130.8, 128.8, 128.6, 128.3, 128.1, 127.6, 126.2, 126.1, 126.0, 125.7, 125.6, 125.3, 125.1, 124.9, 124.0, 73.8 ppm
HRMS (ESI+): calculated for $C_{21}H_{15}$ $[M+H-H_2O]^+$: 267.1168, found 267.1166.
HPLC (OD-H, 0.46*25 cm, 5μm, hexane / ethanol = 75/25, flow 1 mL/min, detection at 254 nm) retention time = 7.863 min (major) and 12.278 min (minor).

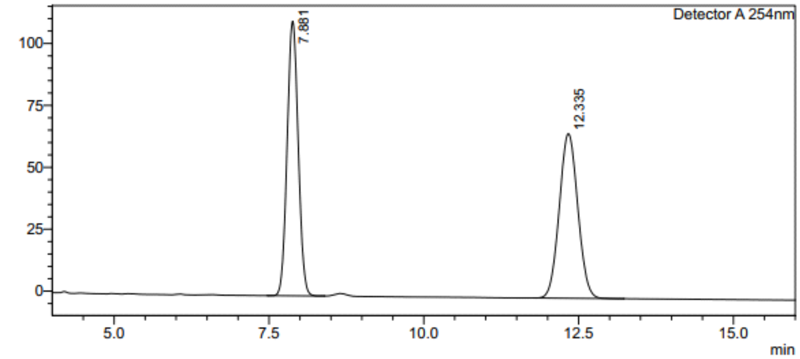

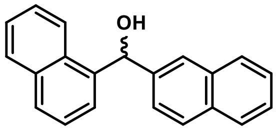

(±)-**C34**

<Peak Table>

Detector A 254nm

Peak#	Ret. Time	Area	Height	Conc.	Unit	Mark	Name
1	7.881	1377741	110901	49.881		M	
2	12.335	1384318	66452	50.119			
Total		2762059	177353				

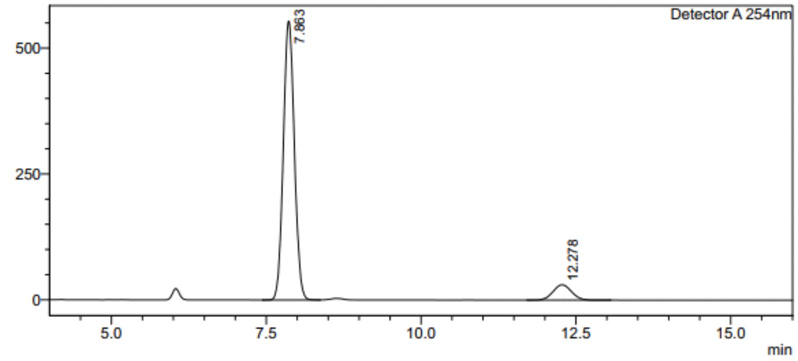

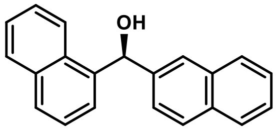

C34
84% yield, 83% ee
obtained by catalyst **B**

<Peak Table>

Detector A 254nm

Peak#	Ret. Time	Area	Height	Conc.	Unit	Mark	Name
1	7.863	6853114	553882	91.564			
2	12.278	631406	30590	8.436			
Total		7484520	584472				

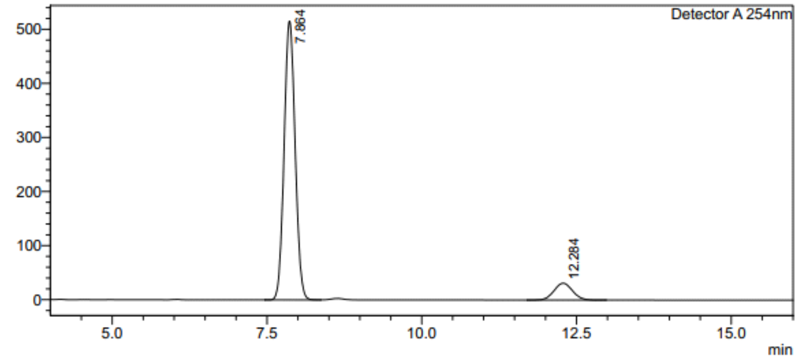

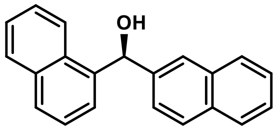

C34
88% yield, 82% ee
obtained by Ru-catalyst **B2**

<Peak Table>

Detector A 254nm

Peak#	Ret. Time	Area	Height	Conc.	Unit	Mark	Name
1	7.864	6382495	515723	90.830			
2	12.284	644397	31174	9.170			
Total		7026892	546896				

Supplementary Table 36. Crystal data and structure refinement for **C34**[2]

CCDC number	2017293
Empirical formula	$C_{21}H_{16}O$
Formula weight	284.34
Temperature/K	100
Crystal system	monoclinic
Space group	P21
a/Å	7.9063(5)
b/Å	5.6177(4)
c/Å	16.2503(11)
α/°	90
β/°	92.950(2)
γ/°	90
Volume/Å^3	720.80(8)
Z	2
ρcalcg/cm^3	1.310
μ/mm^{-1}	0.611
F(000)	300.0
Crystal size/mm^3	0.39 × 0.21 × 0.19
Radiation	CuKα (λ = 1.54178)
2Θ range for data collection/°	5.446 to 136.476
Index ranges	-8 ≤ h ≤ 9, -6 ≤ k ≤ 6, -19 ≤ l ≤ 19
Reflections collected	10705
Independent reflections	2618 [R_{int} = 0.0375, R_{sigma} = 0.0336]
Data/restraints/parameters	2618/1/201
Goodness-of-fit on F^2	1.042
Final R indexes [I>=2σ (I)]	R_1 = 0.0289, wR_2 = 0.0770
Final R indexes [all data]	R_1 = 0.0291, wR_2 = 0.0772
Largest diff. peak/hole / e Å^{-3}	0.15/-0.18
Flack parameter	-0.04(11)

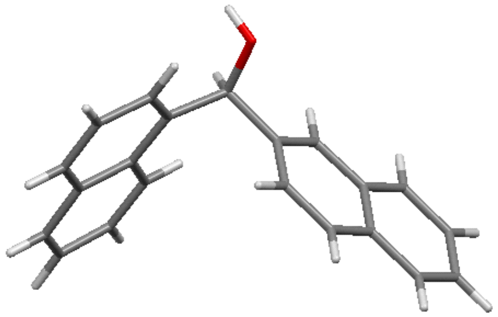

Supplementary Figure 35. The single crystal configuration of **C34** is demonstrated in a capped sticks mode.

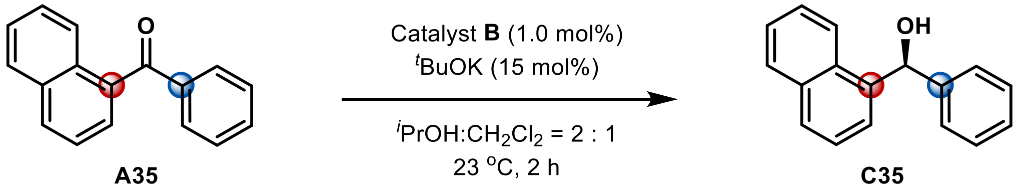

Following the general procedure, **C35** (35.6 mg, 76% yield, 84% ee) was obtained as white solid. Its major pure enantiomer was purified by chiral preparative HPLC (AD-H, 2.0*25 cm, 5μm) and the *X*-ray crystal of the optically pure enantiomer (100% ee) was obtained by liquid/liquid diffusion with CH_2Cl_2/hexane system.

^{1}H NMR (600 MHz, $CDCl_3$): δ 8.04 (d, *J* = 8.1 Hz, 1H), 7.88 (d, *J* = 8.4 Hz, 1H), 7.83 (d, *J* = 8.1 Hz, 1H), 7.64 (d, *J* = 7.2 Hz, 1H), 7.50 (d, *J* = 7.6 Hz, 1H), 7.48 (d, *J* = 7.6 Hz, 1H), 7.45 (d, *J* = 8.4 Hz, 1H), 7.42 (d, *J* = 7.5 Hz, 2H), 7.34 (t, *J* = 7.5 Hz, 2H), 7.29 (t, *J* = 7.2 Hz, 1H), 6.54 (s, 1H), 2.43 (s, 1H) ppm
^{13}C NMR (151 MHz, $CDCl_3$): δ 143.1, 138.8, 133.9, 130.7, 128.7, 128.5, 128.5, 127.6, 127.0, 126.1, 125.6, 125.3, 124.6, 124.0, 73.6 ppm
HRMS (ESI+): calculated for $C_{17}H_{13}$ $[M+H-H_2O]^+$: 217.1012, found 217.1013.
HPLC (AD-H, 0.46*25 cm, 5μm, hexane / ethanol = 95/5, flow 1 mL/min, detection at 254 nm) retention time = 19.906 min (major) and 23.469 min (minor).

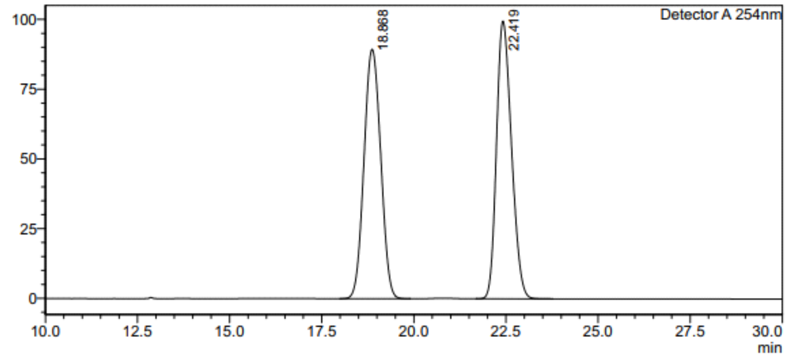

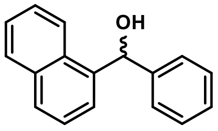

(±)-**C35**

<Peak Table>

Detector A 254nm

Peak#	Ret. Time	Area	Height	Conc.	Unit	Mark	Name
1	18.868	2823058	89453	50.010			
2	22.419	2821937	99610	49.990			
Total		5644996	189063				

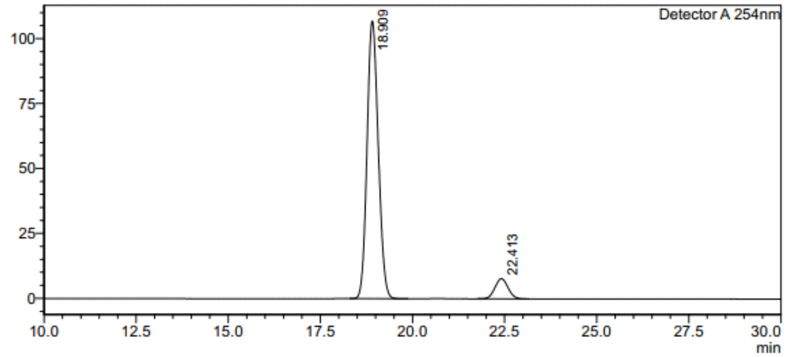

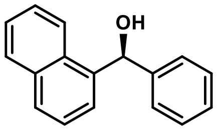

C35

76% yield, 84% ee

obtained by catalyst **B**

<Peak Table>

Detector A 254nm

Peak#	Ret. Time	Area	Height	Conc.	Unit	Mark	Name
1	18.909	2264680	106945	92.132			
2	22.413	193400	7762	7.868			
Total		2458080	114708				

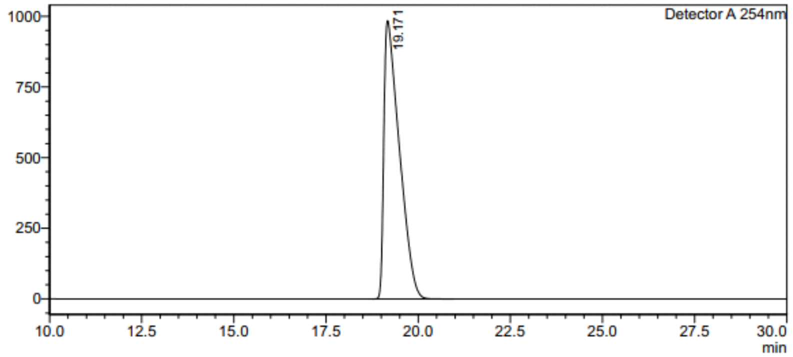

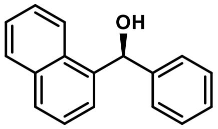

C35

> 99.9% ee

<Peak Table>

Detector A 254nm

Peak#	Ret. Time	Area	Height	Conc.	Unit	Mark	Name
1	19.171	28748525	984836	100.000			
Total		28748525	984836				

Supplementary Table 37. Crystal data and structure refinement for **C35**

CCDC Number	2017162
Empirical formula	$C_{17}H_{14}O$
Formula weight	234.28
Temperature/K	100
Crystal system	monoclinic
Space group	$P2_1$
a/Å	13.3585(5)
b/Å	5.7422(2)
c/Å	16.6807(6)
α/°	90
β/°	107.3710(10)
γ/°	90
Volume/Å^3	1221.17(8)
Z	4
ρ_{calc}g/cm^3	1.274
μ/mm^{-1}	0.603
F(000)	496.0
Crystal size/mm^3	0.36 × 0.35 × 0.28
Radiation	CuKα (λ = 1.54178)
2Θ range for data collection/°	5.552 to 137.182
Index ranges	$-16 \leq h \leq 16$, $-6 \leq k \leq 6$, $-19 \leq l \leq 20$
Reflections collected	19509
Independent reflections	4464 [R_{int} = 0.0275, R_{sigma} = 0.0226]
Data/restraints/parameters	4464/1/328
Goodness-of-fit on F^2	1.078
Final R indexes [I>=2σ (I)]	R_1 = 0.0254, wR_2 = 0.0673
Final R indexes [all data]	R_1 = 0.0256, wR_2 = 0.0674
Largest diff. peak/hole / e Å^{-3}	0.21/-0.17
Flack parameter	0.03(6)

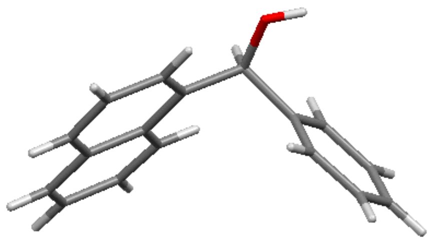

Supplementary Figure 36. The single crystal configuration of **C35** is demonstrated in a capped sticks mode.

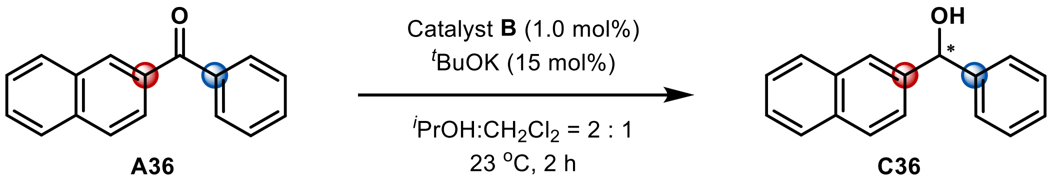

Following the general procedure, **C36** (46.3 mg, 99% yield, 1% ee) was obtained as white solid.

^{1}H NMR (600 MHz, CDCl$_3$): δ 7.90 (s, 1H), 7.88 – 7.74 (m, 3H), 7.53 – 7.39 (m, 5H), 7.35 (t, *J* = 7.2 Hz, 2H), 7.31 – 7.26 (m, 1H), 6.01 (s, 1H), 2.40 (s, 1H) ppm

^{13}C NMR (151 MHz, CDCl$_3$): δ 143.6, 141.1, 133.2, 132.9, 128.5, 128.3, 128.0, 127.7, 126.7, 126.2, 126.0, 125.0, 124.7, 76.4 ppm

HRMS (ESI+): calculated for $C_{17}H_{13}$ $[M+H-H_2O]^+$: 217.1012, found 217.1012.

HPLC (AD-H, 0.46*25 cm, 5μm, hexane / ethanol = 90/10, flow 1 mL/min, detection at 254 nm) retention time = 21.728 min (minor) and 25.353 min (major).

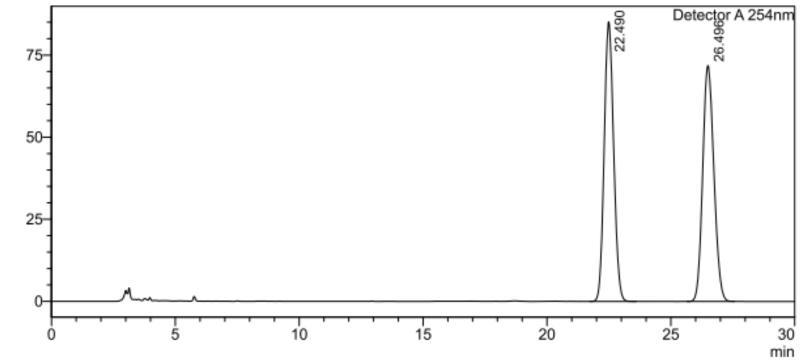

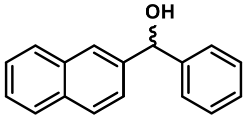

(±)-**C36**

<Peak Table>

Detector A 254nm

Peak#	Ret. Time	Area	Height	Conc.	Unit	Mark	Name
1	22.490	2260397	85143	50.037			
2	26.496	2257015	71824	49.963			
Total		4517412	156968				

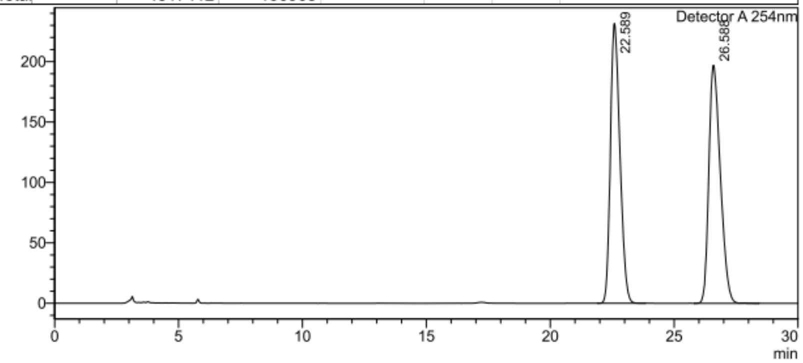

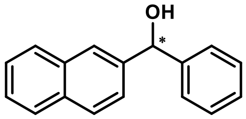

C36
92% yield, 1% ee
obtained by catalyst **B**

<Peak Table>

Detector A 254nm

Peak#	Ret. Time	Area	Height	Conc.	Unit	Mark	Name
1	22.589	6211270	231628	49.437			
2	26.588	6352676	197084	50.563			
Total		12563946	428711				

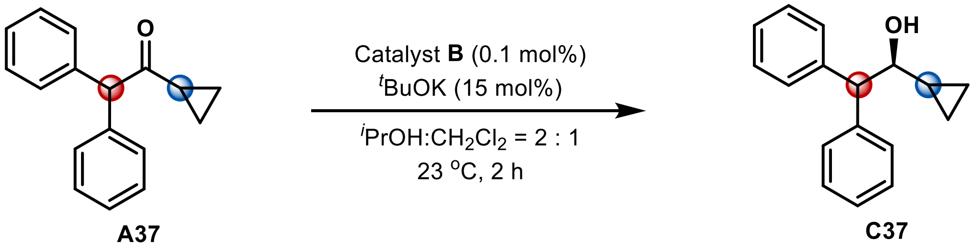

Following the general procedure, **C37** (17.6 mg, 37% yield, 31% ee) was obtained as white solid. Its major pure enantiomer was purified by chiral preparative HPLC (AD-H, 2.0*25 cm, 5μm) and the *X*-ray crystal of the optically pure enantiomer (100% ee) was obtained by liquid/liquid diffusion with CH_2Cl_2/hexane system.

^{1}H NMR (600 MHz, $CDCl_3$): δ 7.41 (d, *J* = 7.2 Hz, 2H), 7.32 (d, *J* = 7.2 Hz, 4H), 7.26 (t, *J* = 7.0 Hz, 2H), 7.24 – 7.20 (m, 1H), 7.18 (t, *J* = 7.0 Hz, 1H), 4.11 (d, *J* = 6.8 Hz, 1H), 3.69 (t, *J* = 7.2 Hz, 1H), 1.68 (s, 1H), 0.93 – 0.82 (m, 1H), 0.50 – 0.30 (m, 3H), 0.07 (s, 1H) ppm
^{13}C NMR (151 MHz, $CDCl_3$): δ 142.5, 141.2, 129.1, 128.5, 128.3, 126.7, 126.3, 77.7, 58.6, 16.4, 3.6, 2.4 ppm
HRMS (ESI+): calculated for $C_{17}H_{17}$ $[M+H-H_2O]^+$: 221.1330, found 221.1329.
HPLC (AD-H, 0.46*25 cm, 5μm, hexane / ethanol = 90/10, flow 1 mL/min, detection at 210 nm) retention time = 7.369 min (major) and 8.669 min (minor).

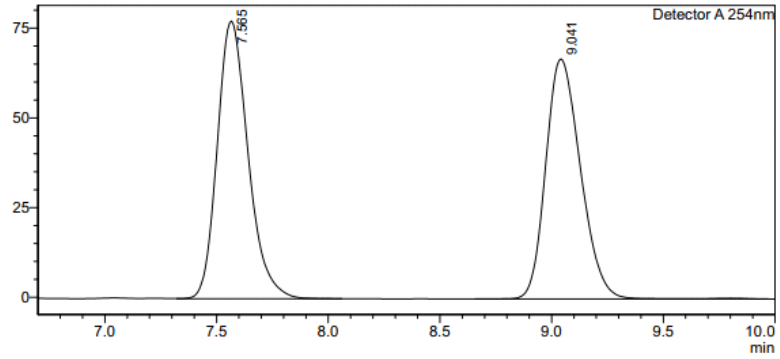

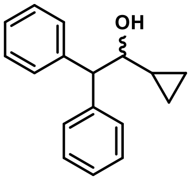

(±)-**C37**

<Peak Table>

Detector A 254nm

Peak#	Ret. Time	Area	Height	Conc.	Unit	Mark	Name
1	7.565	742881	77357	50.601		V	
2	9.041	725241	66917	49.399			
Total		1468122	144274				

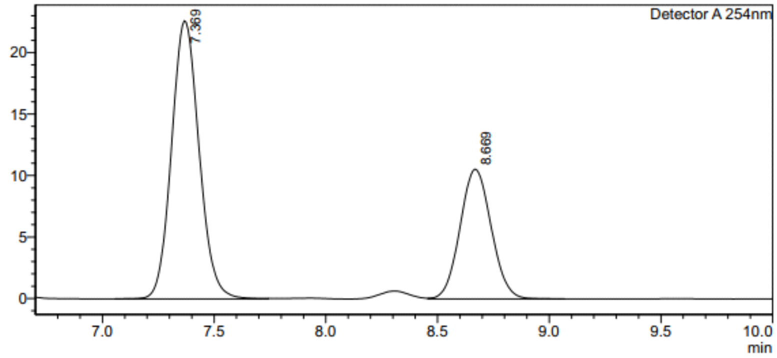

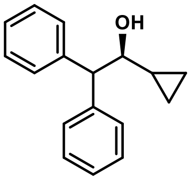

C37
37% yield, 31% ee
obtained by catalyst **B**

<Peak Table>

Detector A 254nm

Peak#	Ret. Time	Area	Height	Conc.	Unit	Mark	Name
1	7.369	194349	22637	65.427			
2	8.669	102696	10558	34.573			
Total		297045	33195				

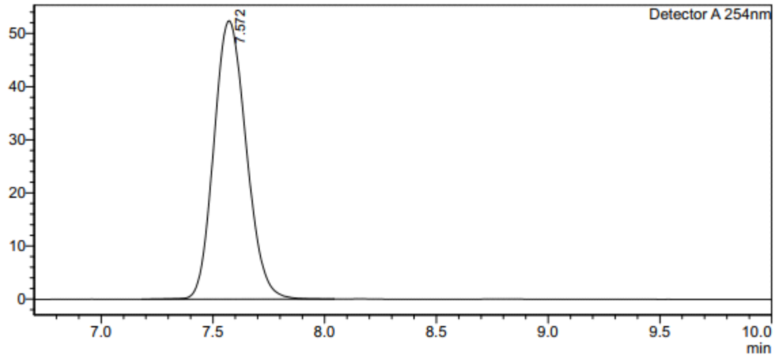

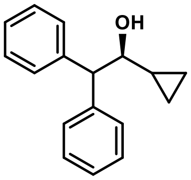

C37
> 99.9% ee

<Peak Table>

Detector A 254nm

Peak#	Ret. Time	Area	Height	Conc.	Unit	Mark	Name
1	7.572	528387	52388	100.000		V	
Total		528387	52388				

Supplementary Table 38. Crystal data and structure refinement for **C37**

Identification code	2181768
Empirical formula	$C_{17}H_{18}O$
Formula weight	238.31
Temperature/K	100.0
Crystal system	monoclinic
Space group	$P2_1$
a/Å	9.2580(3)
b/Å	5.2817(2)
c/Å	13.3649(5)
α/°	90
β/°	92.8830(10)
γ/°	90
Volume/Å^3	652.69(4)
Z	2
ρ_{calc}g/cm^3	1.213
μ/mm^{-1}	0.565
F(000)	256.0
Crystal size/mm^3	0.35 × 0.26 × 0.19
Radiation	CuKα (λ = 1.54178)
2Θ range for data collection/°	6.622 to 136.692
Index ranges	$-11 \leq h \leq 11$, $-6 \leq k \leq 6$, $-16 \leq l \leq 15$
Reflections collected	11166
Independent reflections	2374 [R_{int} = 0.0272, R_{sigma} = 0.0209]
Data/restraints/parameters	2374/1/165
Goodness-of-fit on F^2	1.053
Final R indexes [I>=2σ (I)]	R_1 = 0.0244, wR_2 = 0.0604
Final R indexes [all data]	R_1 = 0.0250, wR_2 = 0.0609
Largest diff. peak/hole / e Å^{-3}	0.16/-0.13
Flack parameter	-0.08(9)

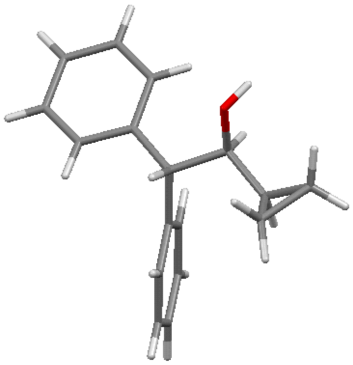

Supplementary Figure 37. The single crystal configuration of **C37** is demonstrated in a capped sticks mode.

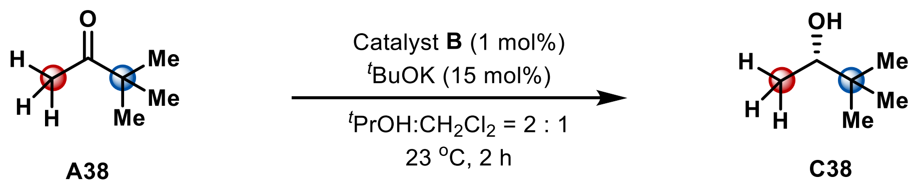

Following the general procedure, **C38** (10.8 mg, 53% yield) was obtained as colorless liquid.

^{1}H NMR (400 MHz, CDCl$_3$): δ 3.47 (qd, J = 6.4, 5.0 Hz, 1H), 1.33 (d, J = 5.0 Hz, 1H), 1.12 (d, J = 6.4 Hz, 3H), 0.89 (s, 9H) ppm
^{13}C NMR (151 MHz, CDCl$_3$): δ 75.7, 34.9, 25.4, 17.8 ppm

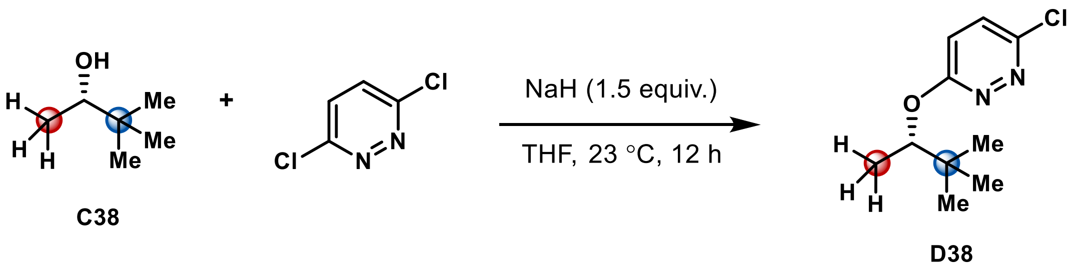

Since **C38** cannot be detected by UV, **C38** was derived with 3,6-dichloropyridazine to afford **D38**(86% yield, 66% ee). The major pure enantiomer of **D38** (66% ee) was purified by chiral preparative HPLC (OD-H, 2.0*25 cm, 5μm) and the *X*-ray crystal of the optically pure enantiomer (100% ee) was obtained by liquid/liquid diffusion with CH_2Cl_2/hexane system.

^{1}H NMR (600 MHz, CDCl$_3$): δ 7.33 (d, J = 9.1 Hz, 1H), 6.89 (d, J = 9.1 Hz, 1H), 5.22 (q, J = 6.3 Hz, 1H), 1.28 (d, J = 6.3 Hz, 3H), 0.98 (s, 9H) ppm
^{13}C NMR (151 MHz, CDCl$_3$): δ 164.5, 150.2, 130.7, 120.5, 80.8, 34.7, 25.7, 14.1 ppm
HRMS (ESI+): calculated for $C_{10}H_{16}ClN_2O$ $[M+H]^+$: 215.0946, found 215.0945.
HPLC (OD-H, 0.46*25 cm, 5μm, hexane / isopropanol = 95/5, flow 1 mL/min, detection at 220 nm) retention time = 4.418 min (minor) and 4.802 min (major).

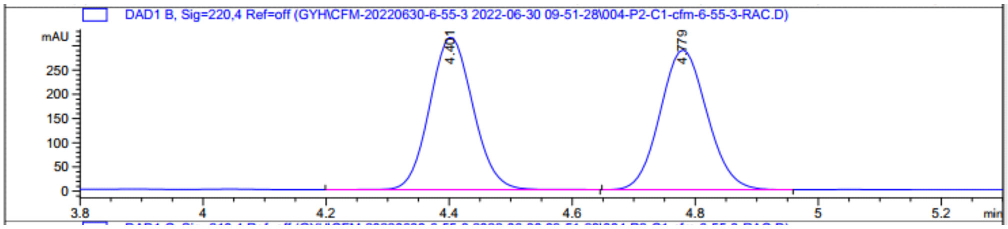

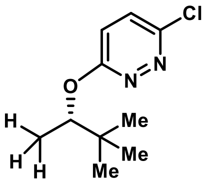

(±)-**D38**

Peak #	RetTime [min]	Type	Width [min]	Area [mAU*s]	Height [mAU]	Area %
1	4.401	BB	0.0769	1539.06421	315.73105	50.2129
2	4.779	BB	0.0836	1526.01563	288.81989	49.7871
Totals :				3065.07983	604.55093	

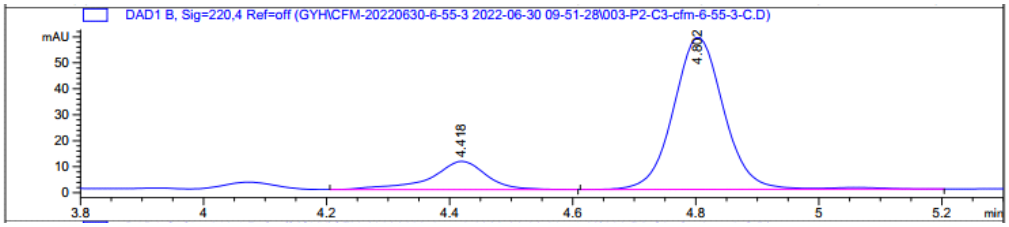

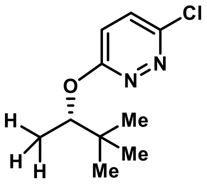

D38
53% yield, 66% ee
obtained by catalyst **B**

Peak #	RetTime [min]	Type	Width [min]	Area [mAU*s]	Height [mAU]	Area %
1	4.418	BB	0.0915	68.70208	10.89782	17.0835
2	4.802	BV R	0.0863	333.45279	58.68587	82.9165
Totals :				402.15487	69.58370	

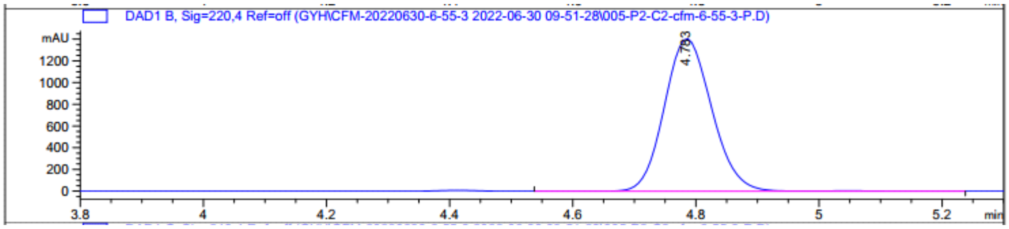

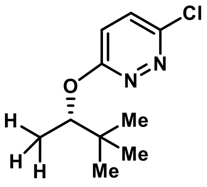

D38
> 99.9% ee

Peak #	RetTime [min]	Type	Width [min]	Area [mAU*s]	Height [mAU]	Area %
1	4.783	BV R	0.0822	7535.41992	1408.01416	100.0000
Totals :				7535.41992	1408.01416	

Supplementary Table 39. Crystal data and structure refinement for **D38**

Identification code	2183983
Empirical formula	$C_{10}H_{15}ClN_2O$
Formula weight	214.69
Temperature/K	100.0(2)
Crystal system	orthorhombic
Space group	$P2_12_12_1$
a/Å	5.7948(4)
b/Å	11.8811(9)
c/Å	16.8487(13)
α/°	90
β/°	90
γ/°	90
Volume/Å^3	1160.01(15)
Z	4
ρ_{calc}g/cm^3	1.229
μ/mm^{-1}	2.689
F(000)	456.0
Crystal size/mm^3	0.4 × 0.12 × 0.09
Radiation	CuKα (λ = 1.54178)
2Θ range for data collection/°	9.108 to 144.518
Index ranges	$-7 \leq h \leq 7, -14 \leq k \leq 14, -20 \leq l \leq 20$
Reflections collected	18427
Independent reflections	2287 [R_{int} = 0.0422, R_{sigma} = 0.0238]
Data/restraints/parameters	2287/0/131
Goodness-of-fit on F^2	1.056
Final R indexes [I>=2σ (I)]	R_1 = 0.0227, wR_2 = 0.0591
Final R indexes [all data]	R_1 = 0.0230, wR_2 = 0.0593
Largest diff. peak/hole / e Å^{-3}	0.18/-0.16
Flack parameter	-0.001(5)

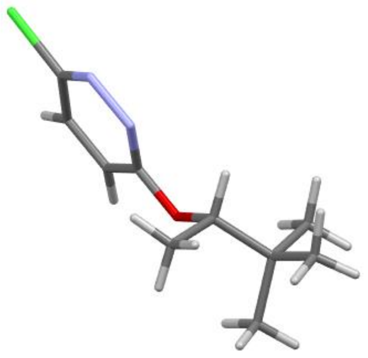

Supplementary Figure 38. The single crystal configuration of **D38** is demonstrated in a capped sticks mode.

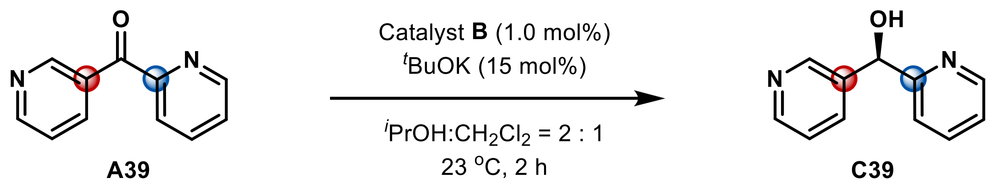

Following the general procedure, **C39** (35.4 mg, 95% yield, 87% ee) was obtained as white solid. Its major pure enantiomer was purified by chiral preparative HPLC (AD-H, 2.0*25 cm, 5μm) and the *X*-ray crystal of the optically pure enantiomer (100% ee) was obtained by liquid/liquid diffusion with CH_2Cl_2/hexane system.

^{1}H NMR (600 MHz, $CDCl_3$): δ 8.66 (s, 1H), 8.59 (d, *J* = 4.8 Hz, 1H), 8.53 (d, *J* = 6.4 Hz, 1H), 7.69 – 7.64 (m, 2H), 7.26 – 7.22 (m, 2H), 7.14 (d, *J* = 8.0 Hz, 1H), 5.79 (d, *J* = 4.4 Hz, 1H), 5.37 (d, *J* = 4.4 Hz, 1H) ppm
^{13}C NMR (151 MHz, $CDCl_3$): δ 159.8, 149.3, 148.8, 148.1, 138.7, 137.1, 134.6, 123.6, 122.8, 121.3, 72.8 ppm
HRMS (ESI+): calculated for $C_{11}H_{11}N_2O$ [M+H]$^+$: 187.0866, found 187.0866.
HPLC (AD-H, 0.46*25 cm, 5μm, hexane / ethanol = 80/20, flow 1 mL/min, detection at 254 nm) retention time = 13.000 min (major) and 16.636 min (minor).

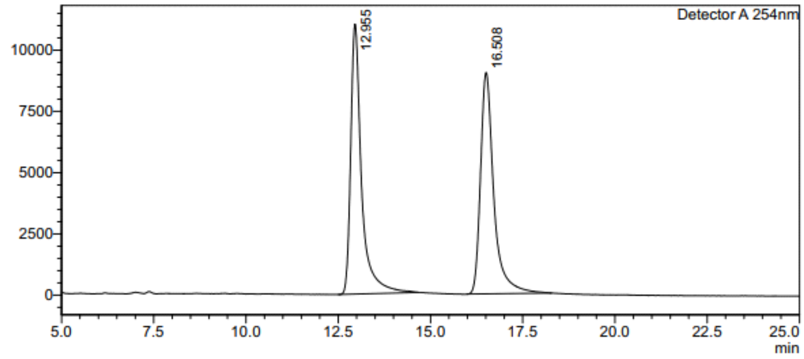

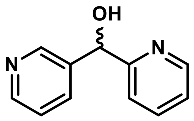

(±)-**C39**

<Peak Table>

Detector A 254nm

Peak#	Ret. Time	Area	Height	Conc.	Unit	Mark	Name
1	12.955	225428	11016	49.931		S	
2	16.508	226051	9040	50.069			
Total		451478	20056				

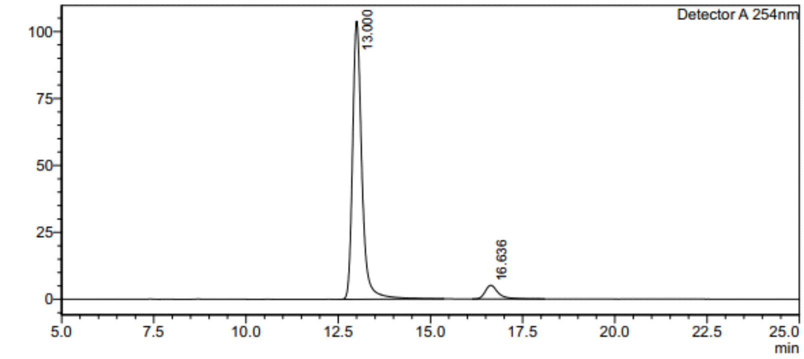

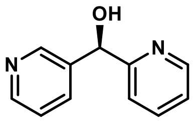

C39
95% yield, 87% ee
obtained by catalyst **B**

<Peak Table>

Detector A 254nm

Peak#	Ret. Time	Area	Height	Conc.	Unit	Mark	Name
1	13.000	1828309	103906	93.495		S	
2	16.636	127203	5123	6.505		S	
Total		1955512	109028				

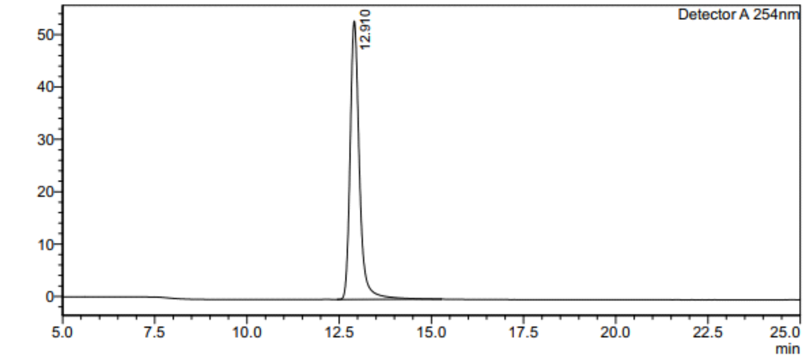

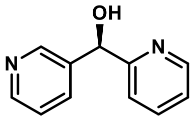

C39
> 99.9% ee

<Peak Table>

Detector A 254nm

Peak#	Ret. Time	Area	Height	Conc.	Unit	Mark	Name
1	12.910	937513	53185	100.000			
Total		937513	53185				

Supplementary Table 40. Crystal data and structure refinement for **C39**

CCDC Number	2017188
Empirical formula	$C_{11}H_{10}N_2O$
Formula weight	186.21
Temperature/K	100
Crystal system	orthorhombic
Space group	$P2_12_12_1$
a/Å	7.3916(2)
b/Å	9.2036(3)
c/Å	13.8817(4)
α/°	90
β/°	90
γ/°	90
Volume/Å^3	944.36(5)
Z	4
ρ_{calc}g/cm^3	1.310
μ/mm^{-1}	0.697
F(000)	392.0
Crystal size/mm^3	0.4 × 0.36 × 0.36
Radiation	CuKα (λ = 1.54178)
2Θ range for data collection/°	11.536 to 136.998
Index ranges	$-8 \leq h \leq 8, -11 \leq k \leq 11, -16 \leq l \leq 16$
Reflections collected	11979
Independent reflections	1720 [R_{int} = 0.0308, R_{sigma} = 0.0186]
Data/restraints/parameters	1720/0/129
Goodness-of-fit on F^2	1.045
Final R indexes [I>=2σ (I)]	R_1 = 0.0231, wR_2 = 0.0591
Final R indexes [all data]	R_1 = 0.0234, wR_2 = 0.0593
Largest diff. peak/hole / e Å^{-3}	0.15/-0.12
Flack parameter	-0.02(7)

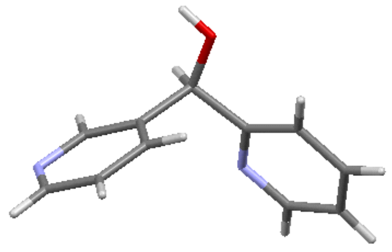

Supplementary Figure 39. The single crystal configuration of **C39** is demonstrated in a capped sticks mode.

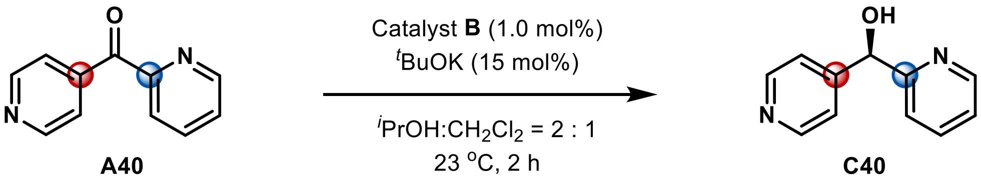

Following the general procedure, **C40** (36.1 mg, 97% yield, 82% ee) was obtained as white solid.

^{1}H NMR (400 MHz, $CDCl_3$): δ 8.56 (d, J = 4.8 Hz, 1H), 8.53 (d, J = 6.0 Hz, 2H), 7.66 (t, J = 7.6 Hz, 1H), 7.34 (d, J = 6.0 Hz, 2H), 7.26 – 7.19 (m, 2H), 5.74 (s, 1H), 5.49 (s, 1H) ppm

^{13}C NMR (101 MHz, $CDCl_3$): δ 159.4, 152.0, 149.9, 148.3, 137.2, 123.0, 121.7, 121.1, 73.8 ppm

HRMS (ESI+): calculated for $C_{11}H_{11}N_2O$ $[M+H]^+$: 187.0866, found 187.0866.

HPLC (AD-H, 0.46*25 cm, 5μm, hexane / ethanol = 90/10, flow 1 mL/min, detection at 254 nm) retention time = 18.918 min (major) and 21.733 min (minor).

The major enantiomer of **C40** (82% ee) was purified by chiral preparative HPLC (AD-H, 2.0*25 cm, 5μm). Because we were unable to grow *X*-ray qualified crystal of the optically pure **C40** (100% ee), we derivatize it with (1*S*)-(-)-camphanic acid chloride to afford **D40** as a single diastereomer. Then the *X*-ray crystal of the optically pure **D40** was obtained by liquid/liquid diffusion with THF/hexane system.

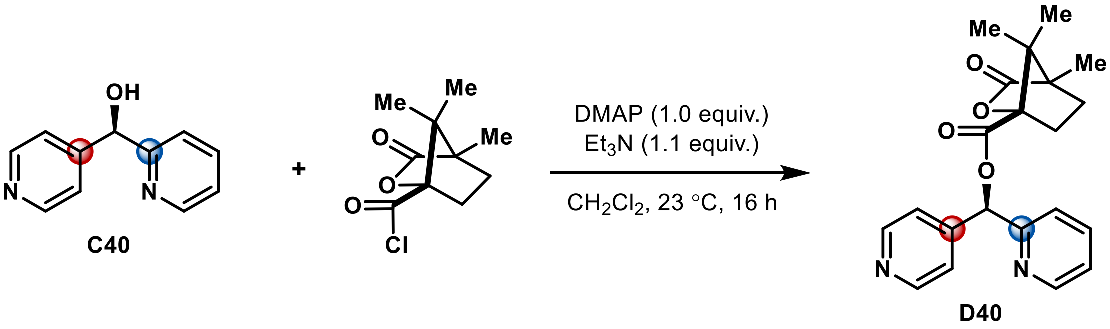

^{1}H NMR (600 MHz, $CDCl_3$): δ 8.57 (d, J = 5.8 Hz, 2H), 8.55 (d, J = 4.8 Hz, 1H), 7.70 (t, J = 7.0 Hz, 1H), 7.45 (d, J = 7.9 Hz, 1H), 7.39 (d, J = 5.8 Hz, 2H), 7.22 (dd, J = 7.0, 4.8 Hz, 1H), 6.92 (s, 1H), 2.50 – 2.43 (m, 1H), 2.13 – 2.02 (m, 2H), 1.97 – 1.89 (m, 1H), 1.74 – 1.68 (m, 1H), 1.11 (s, 3H), 1.03 (s, 3H), 0.93 (s, 3H) ppm

^{13}C NMR (151 MHz, $CDCl_3$): δ 178.1, 166.4, 157.1, 150.1, 149.6, 146.9, 137.2, 123.4, 121.5, 120.9, 90.8, 77.4, 54.9, 54.5, 30.8, 28.8, 16.7, 16.6, 9.6 ppm

HRMS (ESI+): calculated for $C_{21}H_{23}N_2O_4$ $[M+H]^+$: 367.1652, found 367.1653.

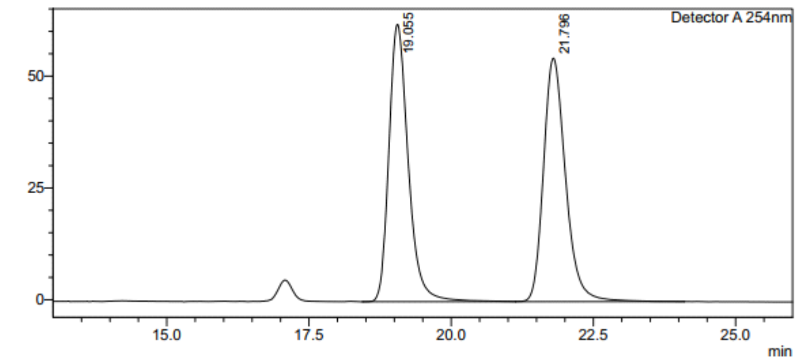

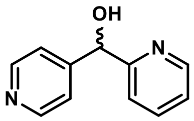

(±)-**C40**

<Peak Table>

Detector A 254nm

Peak#	Ret. Time	Area	Height	Conc.	Unit	Mark	Name
1	19.055	1470164	61996	49.989		S	
2	21.796	1470791	54408	50.011		SV	
Total		2940955	116404				

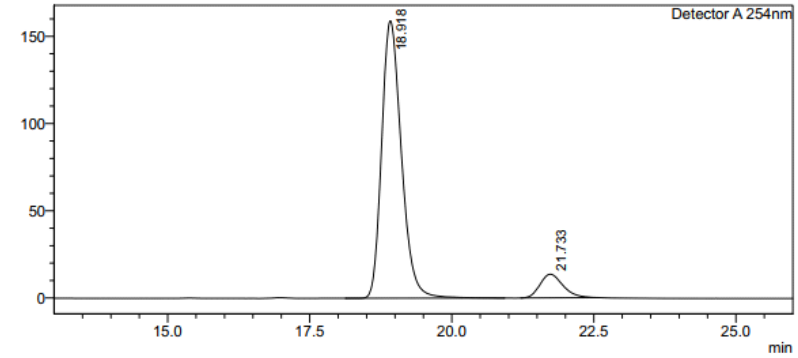

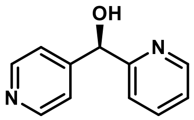

C40
97% yield, 82% ee
obtained by catalyst **B**

<Peak Table>

Detector A 254nm

Peak#	Ret. Time	Area	Height	Conc.	Unit	Mark	Name
1	18.918	3841043	159012	90.916		M	
2	21.733	383806	13592	9.084		M	
Total		4224849	172604				

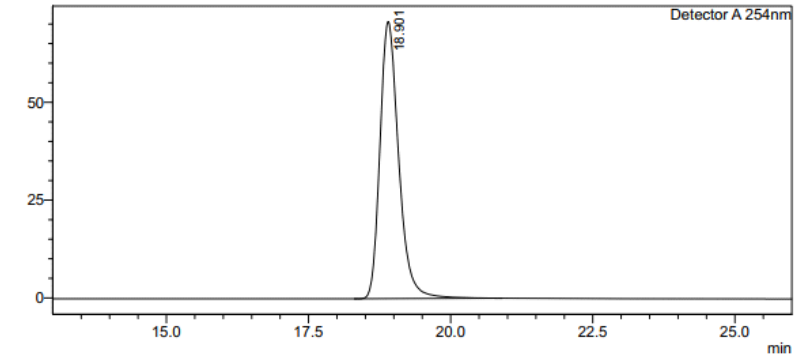

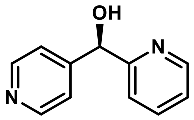

C40
> 99.9% ee

<Peak Table>

Detector A 254nm

Peak#	Ret. Time	Area	Height	Conc.	Unit	Mark	Name
1	18.901	1626179	70823	100.000		S	
Total		1626179	70823				

Supplementary Table 41. Crystal data and structure refinement for **D40**

CCDC Number	2208981
Empirical formula	$C_{21}H_{22}N_2O_4$
Formula weight	366.40
Temperature/K	100.0(2)
Crystal system	orthorhombic
Space group	P212121
a/Å	9.3662(8)
b/Å	9.6760(8)
c/Å	19.9192(17)
α/°	90
β/°	90
γ/°	90
Volume/Å^3	1805.2(3)
Z	4
ρcalcg/cm^3	1.348
μ/mm^{-1}	0.489
F(000)	776.0
Crystal size/mm^3	0.25 × 0.24 × 0.19
Radiation	GaKα (λ = 1.34139)
2Θ range for data collection/°	8.84 to 126.996
Index ranges	$-12 \le h \le 12$, $-12 \le k \le 12$, $-26 \le l \le 25$
Reflections collected	49948
Independent reflections	4460 [R_{int} = 0.0537, R_{sigma} = 0.0234]
Data/restraints/parameters	4460/0/247
Goodness-of-fit on F^2	1.037
Final R indexes [I>=2σ (I)]	R_1 = 0.0282, wR_2 = 0.0750
Final R indexes [all data]	R_1 = 0.0286, wR_2 = 0.0754
Largest diff. peak/hole / e Å^{-3}	0.28/-0.17
Flack parameter	0.00(6)

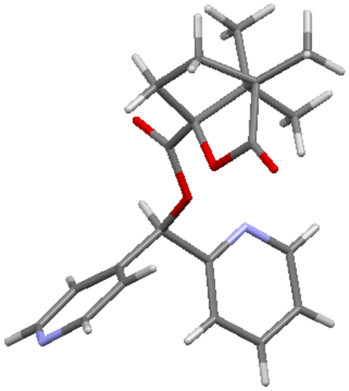

Supplementary Figure 40. The single crystal configuration of **D40** is demonstrated in a capped sticks mode.

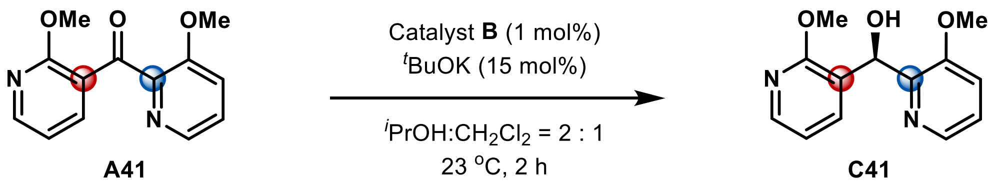

Following the general procedure, **C41** (45.8 mg, 93% yield, 95% ee) was obtained as white solid. The retention time in HPLC of the major enantiomer is consistent with that obtained by Ru-catalyst **B2,** and the *X*-ray crystal of the optically pure major enantiomer (100% ee) has been previously reported[2].

^{1}H NMR (400 MHz, CDCl$_3$): δ 8.24 (d, *J* = 4.8 Hz, 1H), 8.08 (d, *J* = 4.8 Hz, 1H), 7.29 (dd, *J* = 7.6, 2.8 Hz, 1H), 7.25 (dd, *J* = 7.6, 2.8 Hz, 1H), 7.16 (d, *J* = 7.4 Hz, 1H), 6.79 (d, *J* = 7.4 Hz, 1H), 6.21 (s, 1H), 5.32 (s, 1H), 4.01 (s, 3H), 3.73 (s, 3H) ppm

^{13}C NMR (101 MHz, CDCl$_3$): δ 161.8, 152.4, 149.1, 145.7, 139.2, 136.6, 125.4, 123.4, 117.6, 116.6, 65.6, 55.3, 53.4 ppm

HRMS (ESI+): calculated for $C_{13}H_{15}N_2O_3$ [M+H]$^+$: 247.1077, found 247.1076.

HPLC (AD-H, 0.46*25 cm, 5μm, hexane / ethanol = 90/10, flow 1 mL/min, detection at 254 nm) retention time = 16.365 min (major) and 19.940 min (minor).

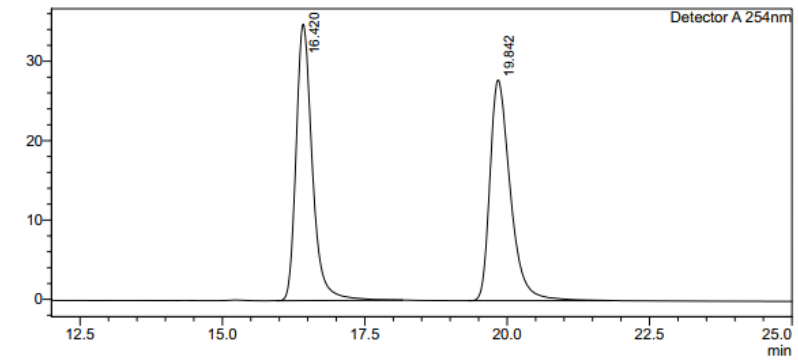

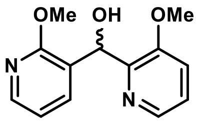

(±)-**C41**

<Peak Table>

Detector A 254nm

Peak#	Ret. Time	Area	Height	Conc.	Unit	Mark	Name
1	16.420	691153	34832	49.945			
2	19.842	692666	27814	50.055		S	
Total		1383819	62646				

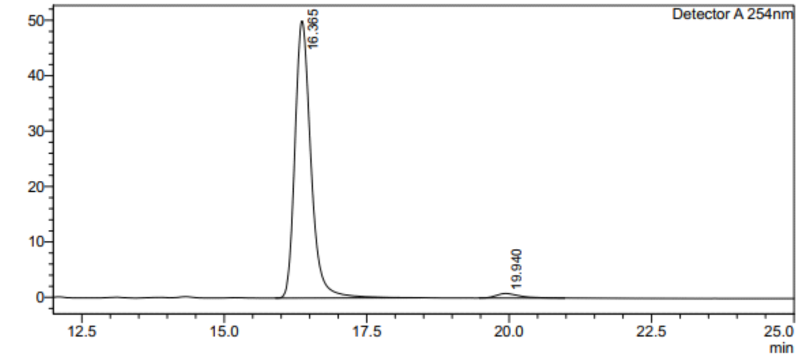

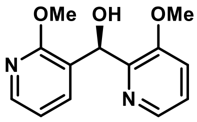

C41
93% yield, 95% ee
obtained by catalyst **B**

<Peak Table>

Detector A 254nm

Peak#	Ret. Time	Area	Height	Conc.	Unit	Mark	Name
1	16.365	977802	50000	97.625		S	
2	19.940	23789	819	2.375		S	
Total		1001590	50820				

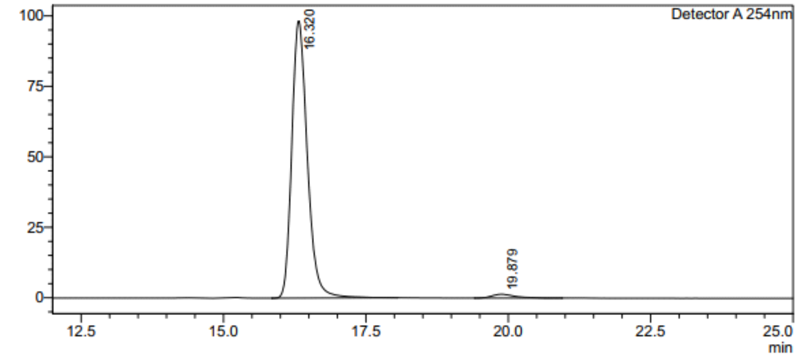

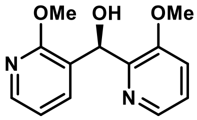

C41
98% yield, 96% ee
obtained by Ru-catalyst **B2**

<Peak Table>

Detector A 254nm

Peak#	Ret. Time	Area	Height	Conc.	Unit	Mark	Name
1	16.320	1869290	98316	98.011			
2	19.879	37942	1345	1.989			
Total		1907233	99661				

Supplementary Table 42. Crystal data and structure refinement for **C41**[2]

CCDC number	2019561
Empirical formula	$C_{13}H_{14}N_2O_3$
Formula weight	246.26
Temperature/K	100
Crystal system	orthorhombic
Space group	$P2_12_12_1$
a/Å	7.5491(5)
b/Å	10.7683(7)
c/Å	14.2993(9)
α/°	90
β/°	90
γ/°	90
Volume/Å^3	1162.40(13)
Z	4
ρcalcg/cm^3	1.407
μ/mm^{-1}	0.838
F(000)	520.0
Crystal size/mm^3	0.38 × 0.34 × 0.26
Radiation	CuKα (λ = 1.54178)
2Θ range for data collection/°	10.284 to 137.42
Index ranges	$-8 \leq h \leq 9$, $-12 \leq k \leq 12$, $-17 \leq l \leq 17$
Reflections collected	23927
Independent reflections	2142 [R_{int} = 0.0314, R_{sigma} = 0.0139]
Data/restraints/parameters	2142/0/167
Goodness-of-fit on F^2	1.079
Final R indexes [I>=2σ (I)]	R_1 = 0.0224, wR_2 = 0.0603
Final R indexes [all data]	R_1 = 0.0225, wR_2 = 0.0604
Largest diff. peak/hole / e Å^{-3}	0.18/-0.14
Flack parameter	-0.01(4)

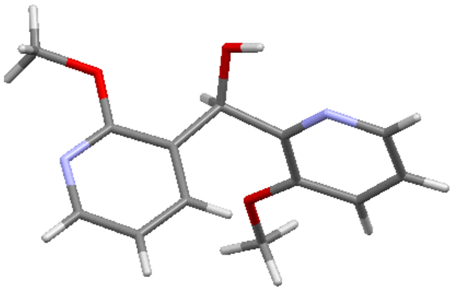

Supplementary Figure 41. The single crystal configuration of **C41** is demonstrated in a capped sticks mode.

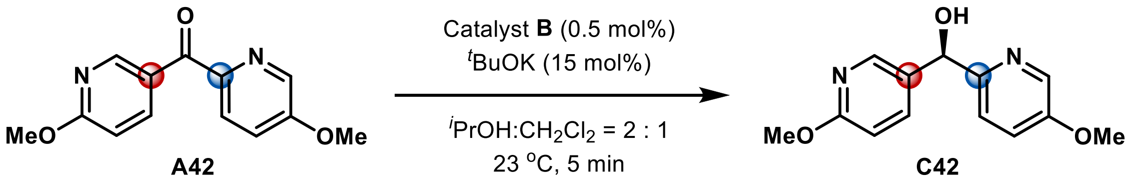

Following the general procedure, **C42** (48.2 mg, 98% yield, 95% ee) was obtained as white solid. Its major pure enantiomer was purified by chiral preparative HPLC (AD-H, 2.0*25 cm, 5μm) and the *X*-ray crystal of the optically pure enantiomer (100% ee) was obtained by liquid/liquid diffusion with CH_2Cl_2/hexane system.

^{1}H NMR (400 MHz, $CDCl_3$): δ 8.24 (s, 1H), 8.16 (s, 1H), 7.49 (d, *J* = 8.6 Hz, 1H), 7.17 (d, *J* = 8.6 Hz, 1H), 7.04 (d, *J* = 8.6 Hz, 1H), 6.69 (d, *J* = 8.6 Hz, 1H), 5.69 (s, 1H), 3.91 (s, 3H), 3.85 (s, 3H) ppm

^{13}C NMR (101 MHz, $CDCl_3$): δ 163.9, 155.1, 152.5, 145.5, 137.5, 134.8, 131.8, 122.2, 121.4, 111.1, 72.0, 55.7, 53.4 ppm

HRMS (ESI+): calculated for $C_{13}H_{15}N_2O_3$ $[M+H]^+$: 247.1077, found 247.1076.

HPLC (AD-H, 0.46*25 cm, 5μm, hexane / isopropanol = 90/10, flow 1 mL/min, detection at 210nm) retention time = 31.852 min (major) and 36.790 min (minor).

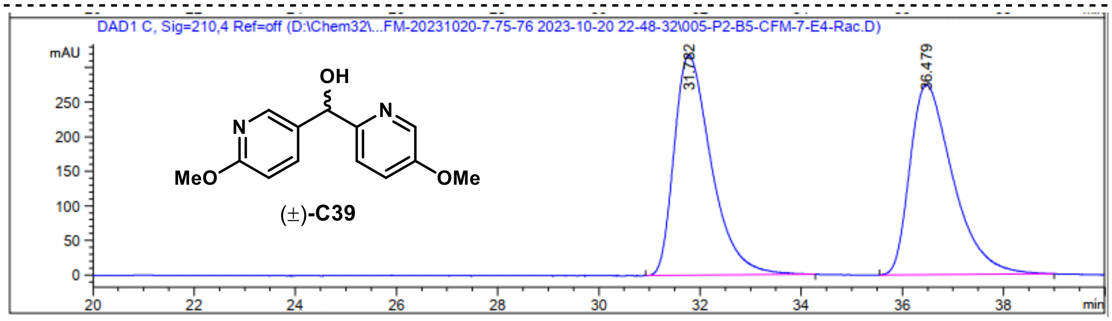

Peak #	RetTime [min]	Type	Width [min]	Area [mAU*s]	Height [mAU]	Area %
1	31.782	BB	0.7549	1.63097e4	318.77853	50.1066
2	36.479	BB	0.8547	1.62402e4	274.33054	49.8934

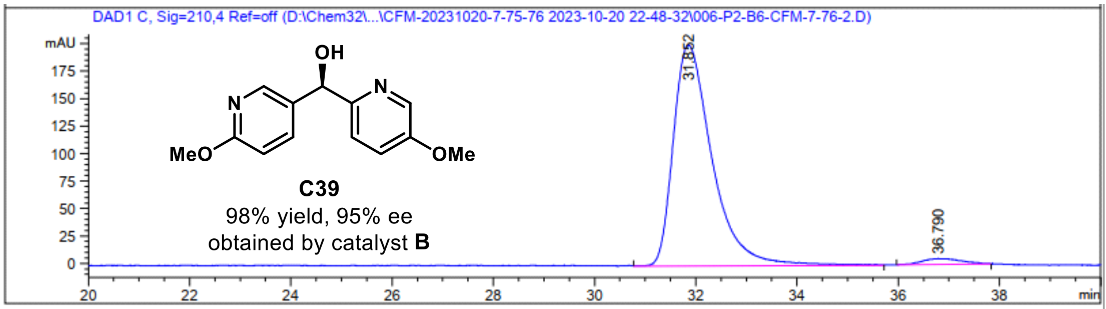

Peak #	RetTime [min]	Type	Width [min]	Area [mAU*s]	Height [mAU]	Area %
1	31.852	MM R	0.8926	1.07782e4	201.25880	97.3976
2	36.790	VB R	0.6369	287.98926	5.34249	2.6024

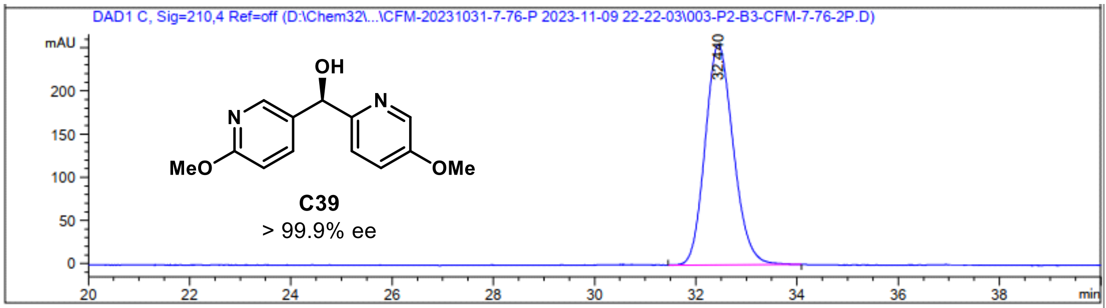

Peak #	RetTime [min]	Type	Width [min]	Area [mAU*s]	Height [mAU]	Area %
1	32.440	VV R	0.5984	9872.25098	254.60970	100.0000

Supplementary Table 43. Crystal data and structure refinement for **C42**

CCDC Number	2311153
Empirical formula	$C_{13}H_{14}N_2O_3$
Formula weight	246.26
Temperature/K	100.0(2)
Crystal system	orthorhombic
Space group	P212121
a/Å	7.3403(9)
b/Å	17.127(2)
c/Å	29.115(4)
α/°	90
β/°	90
γ/°	90
Volume/Å^3	3660.2(8)
Z	12
ρcalcg/cm^3	1.341
μ/mm^{-1}	0.508
F(000)	1560.0
Crystal size/mm^3	0.24 × 0.19 × 0.18
Radiation	GaKα (λ = 1.34138)
2Θ range for data collection/°	5.208 to 140.426
Index ranges	$-10 \le h \le 10$, $-23 \le k \le 23$, $-40 \le l \le 40$
Reflections collected	192158
Independent reflections	10517 [R_{int} = 0.0592, R_{sigma} = 0.0205]
Data/restraints/parameters	10517/0/497
Goodness-of-fit on F^2	1.027
Final R indexes [I>=2σ (I)]	R_1 = 0.0287, wR_2 = 0.0761
Final R indexes [all data]	R_1 = 0.0303, wR_2 = 0.0773
Largest diff. peak/hole / e Å^{-3}	0.32/-0.17
Flack parameter	0.02(3)

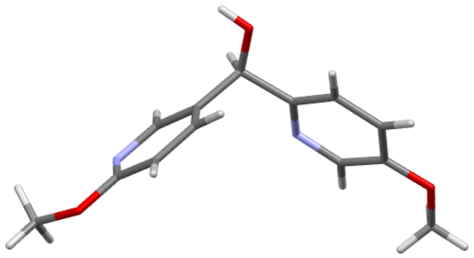

Supplementary Figure 42. The single crystal configuration of **C42** is demonstrated in a capped sticks mode.

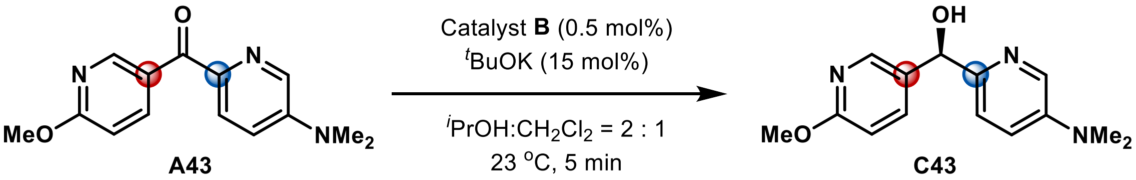

Following the general procedure, **C43** (46.1 mg, 89% yield, 94% ee) was obtained as white solid. Its major pure enantiomer was purified by chiral preparative HPLC (OD-H, 2.0*25 cm, 5μm) and the *X*-ray crystal of the optically pure enantiomer (100% ee) was obtained by liquid/liquid diffusion with CH_2Cl_2/hexane system.

^{1}H NMR (400 MHz, CDCl$_3$): δ 8.16 (s, 1H), 8.05 (s, 1H), 7.50 (d, *J* = 8.6 Hz, 1H), 7.01 – 6.88 (m, 2H), 6.68 (d, *J* = 8.6 Hz, 1H), 5.65 (s, 1H), 3.91 (s, 3H), 2.96 (s, 6H) ppm
^{13}C NMR (101 MHz, CDCl$_3$): δ 163.8, 147.8, 145.6, 145.5, 137.6, 132.3, 121.0, 120.1, 111.0, 71.8, 53.4, 40.2 ppm
HRMS (ESI+): calculated for $C_{14}H_{18}N_3O_2$ $[M+H]^+$: 260.1394, found 260.1392.
HPLC (OD-H, 0.46*25 cm, 5μm, hexane / isopropanol = 80/20, flow 1 mL/min, detection at 210 nm) retention time = 13.942 min (minor) and 22.285 min (major).

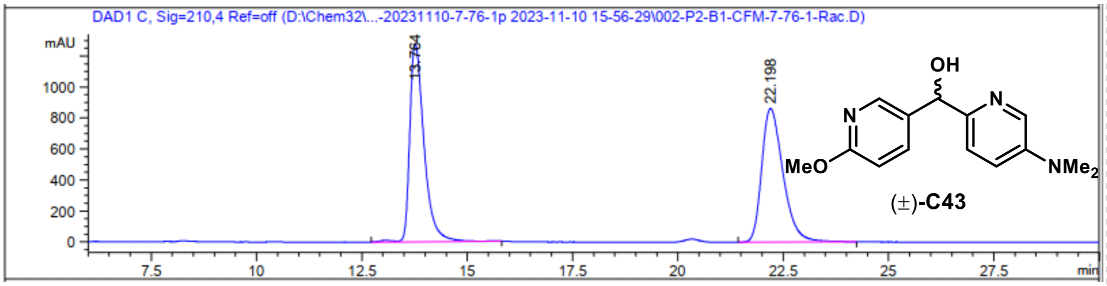

Peak #	RetTime [min]	Type	Width [min]	Area [mAU*s]	Height [mAU]	Area %
1	13.764	MM R	0.3965	3.04074e4	1278.14661	50.0583
2	22.198	BV R	0.5343	3.03366e4	862.54254	49.9417

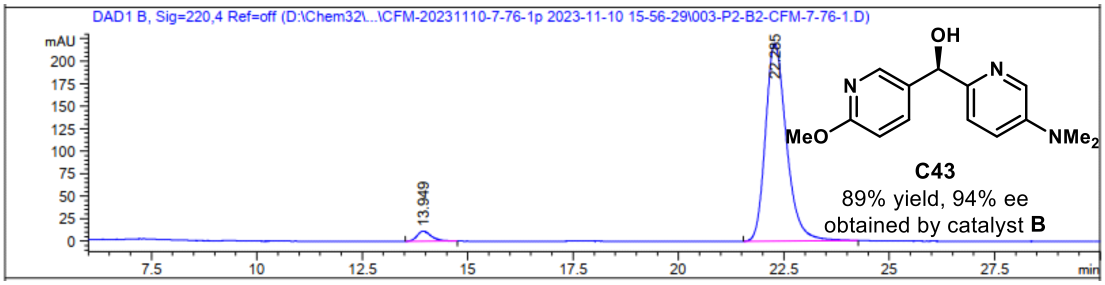

Peak #	RetTime [min]	Type	Width [min]	Area [mAU*s]	Height [mAU]	Area %
1	13.942	MM R	0.3852	253.62076	10.97360	3.1797
2	22.285	MM R	0.5930	7722.50391	217.04951	96.8203

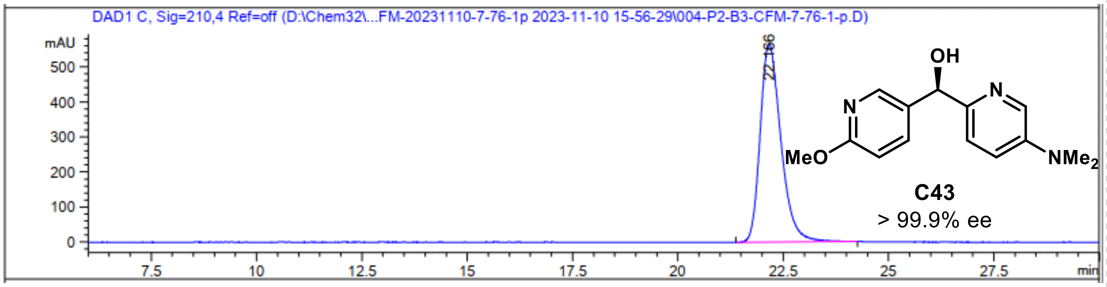

Peak #	RetTime [min]	Type	Width [min]	Area [mAU*s]	Height [mAU]	Area %
1	22.166	VV R	0.5097	1.93756e4	565.58502	100.0000

Supplementary Table 44. Crystal data and structure refinement for **C43**

CCDC Number	2311152
Empirical formula	$C_{14}H_{17}N_3O_2$
Formula weight	259.30
Temperature/K	100.0(2)
Crystal system	orthorhombic
Space group	P212121
a/Å	7.2013(7)
b/Å	10.2793(10)
c/Å	17.6460(18)
α/°	90
β/°	90
γ/°	90
Volume/$Å^3$	1306.2(2)
Z	4
ρcalcg/cm^3	1.319
μ/mm-1	0.467
F(000)	552.0
Crystal size/mm^3	0.23 × 0.18 × 0.18
Radiation	GaKα (λ = 1.34138)
2Θ range for data collection/°	8.662 to 146.77
Index ranges	-10 ≤ h ≤ 9, -14 ≤ k ≤ 14, -25 ≤ l ≤ 25
Reflections collected	58087
Independent reflections	3983 [R_{int} = 0.0530, R_{sigma} = 0.0259]
Data/restraints/parameters	3983/0/176
Goodness-of-fit on F^2	1.026
Final R indexes [I>=2σ (I)]	R_1 = 0.0335, wR_2 = 0.0932
Final R indexes [all data]	R_1 = 0.0338, wR_2 = 0.0935
Largest diff. peak/hole / e $Å^{-3}$	0.31/-0.20
Flack parameter	0.05(5)

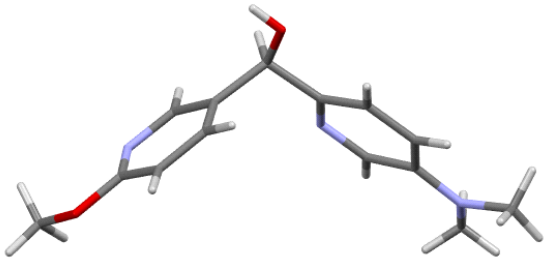

Supplementary Figure 43. The single crystal configuration of **C43** is demonstrated in a capped sticks mode.

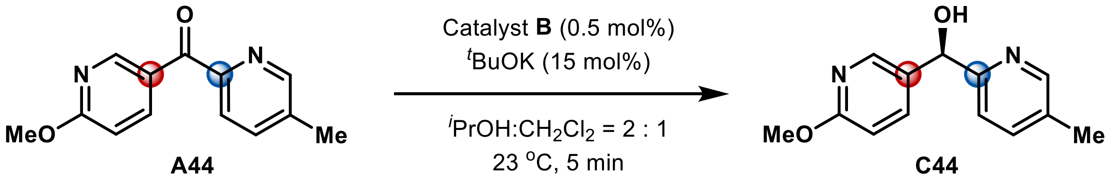

Following the general procedure, **C44** (45.1 mg, 98% yield, 92% ee) was obtained as white solid. Its major pure enantiomer was purified by chiral preparative HPLC (AD-H, 2.0*25 cm, 5μm) and the *X*-ray crystal of the optically pure enantiomer (100% ee) was obtained by liquid/liquid diffusion with CH_2Cl_2/hexane system.

^{1}H NMR (400 MHz, CDCl$_3$): δ 8.37 (s, 1H), 8.17 (s, 1H), 7.48 (d, *J* = 8.6 Hz, 1H), 7.44 (d, *J* = 8.0 Hz, 1H), 7.00 (d, *J* = 8.0 Hz, 1H), 6.68 (d, *J* = 8.6 Hz, 1H), 5.68 (s, 1H), 3.91 (s, 3H), 2.32 (s, 3H) ppm

^{13}C NMR (101 MHz, CDCl$_3$): δ 163.9, 157.5, 148.0, 145.6, 137.7, 137.6, 132.2, 131.8, 120.7, 111.1, 72.1, 53.4, 18.0 ppm

HRMS (ESI+): calculated for $C_{13}H_{15}N_2O_2$ [M+H]$^+$: 231.1128, found 231.1127.

HPLC (AD-H, 0.46*25 cm, 5μm, hexane / isopropanol = 90/10, flow 1 mL/min, detection at 210 nm) retention time = 21.789 min (major) and 24.235 min (minor).

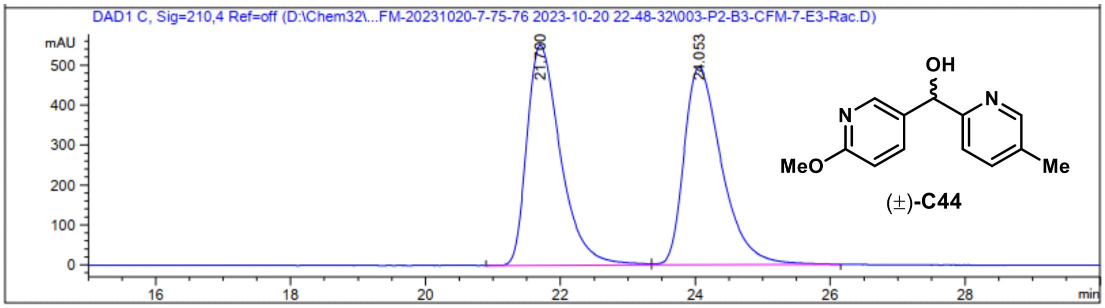

Peak #	RetTime [min]	Type	Width [min]	Area [mAU*s]	Height [mAU]	Area %
1	21.700	VV R	0.5287	1.91383e4	551.65631	49.9239
2	24.053	VB	0.5785	1.91967e4	494.95386	50.0761

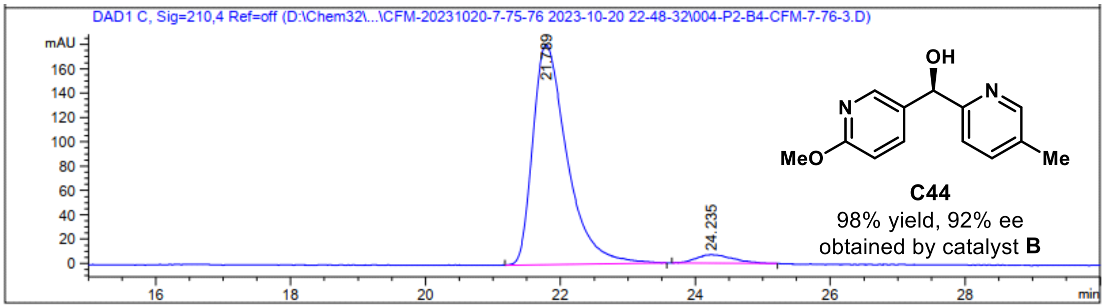

Peak #	RetTime [min]	Type	Width [min]	Area [mAU*s]	Height [mAU]	Area %
1	21.789	BV R	0.5313	6363.39014	180.50874	95.9357
2	24.235	VV R	0.4614	269.58466	7.03018	4.0643

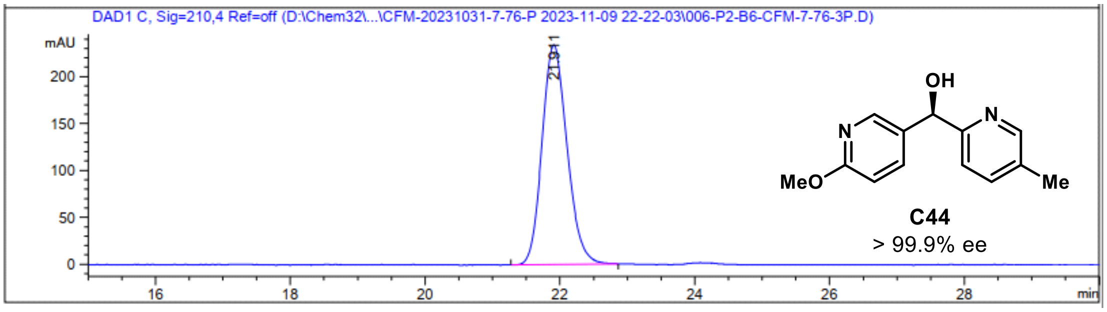

Peak #	RetTime [min]	Type	Width [min]	Area [mAU*s]	Height [mAU]	Area %
1	21.911	BV R	0.3983	6023.99756	233.99663	100.0000

Supplementary Table 45. Crystal data and structure refinement for **C44**

CCDC Number	2311154
Empirical formula	$C_{13}H_{14}N_2O_2$
Formula weight	230.26
Temperature/K	100.0(2)
Crystal system	orthorhombic
Space group	P212121
a/Å	7.3579(10)
b/Å	10.0523(13)
c/Å	15.610(2)
α/°	90
β/°	90
γ/°	90
Volume/Å^3	1154.6(3)
Z	4
ρcalcg/cm^3	1.325
μ/mm-1	0.470
F(000)	488.0
Crystal size/mm^3	0.24 × 0.22 × 0.14
Radiation	GaKα (λ = 1.34138)
2Θ range for data collection/°	9.104 to 126.708
Index ranges	-9 ≤ h ≤ 9, -13 ≤ k ≤ 13, -20 ≤ l ≤ 20
Reflections collected	61683
Independent reflections	2869 [R_{int} = 0.0477, R_{sigma} = 0.0192]
Data/restraints/parameters	2869/0/158
Goodness-of-fit on F^2	1.091
Final R indexes [I>=2σ (I)]	R_1 = 0.0269, wR_2 = 0.0723
Final R indexes [all data]	R_1 = 0.0272, wR_2 = 0.0724
Largest diff. peak/hole / e Å^{-3}	0.26/-0.18
Flack parameter	0.05(4)

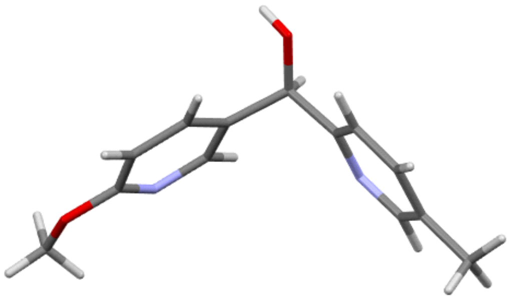

Supplementary Figure 44. The single crystal configuration of **C44** is demonstrated in a capped sticks mode.

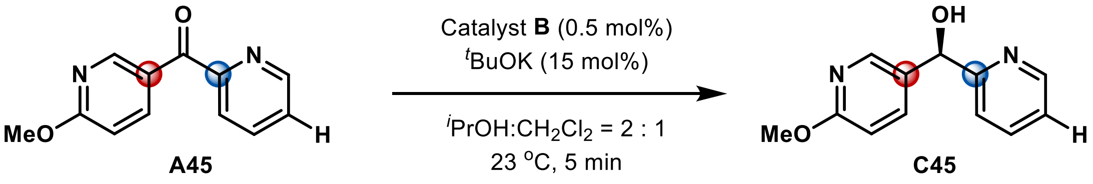

Following the general procedure, **C45** (40.2 mg, 98% yield, 93% ee) was obtained as white solid. Its major pure enantiomer was purified by chiral preparative HPLC (AD-H, 2.0*25 cm, 5μm) and the *X*-ray crystal of the optically pure enantiomer (100% ee) was obtained by liquid/liquid diffusion with CH_2Cl_2/hexane system.

^{1}H NMR (400 MHz, $CDCl_3$): δ 8.55 (s, 1H), 8.17 (s, 1H), 7.68 – 7.57 (m, 1H), 7.49 (d, *J* = 8.6 Hz, 1H), 7.25 – 7.17 (m, 1H), 7.12 (d, *J* = 7.9 Hz, 1H), 6.69 (d, *J* = 8.6 Hz, 1H), 5.71 (s, 1H), 3.91 (s, 3H) ppm
^{13}C NMR (101 MHz, $CDCl_3$): δ 164.0, 160.3, 147.9, 145.7, 137.6, 137.0, 131.6, 122.6, 121.2, 111.2, 72.3, 53.4 ppm
HRMS (ESI+): calculated for $C_{12}H_{13}N_2O_2$ $[M+H]^+$: 217.0972, found 217.0970.
HPLC (AD-H, 0.46*25 cm, 5μm, hexane / isopropanol = 90/10, flow 1 mL/min, detection at 210 nm) retention time = 21.735 min (major) and 25.331 min (minor).

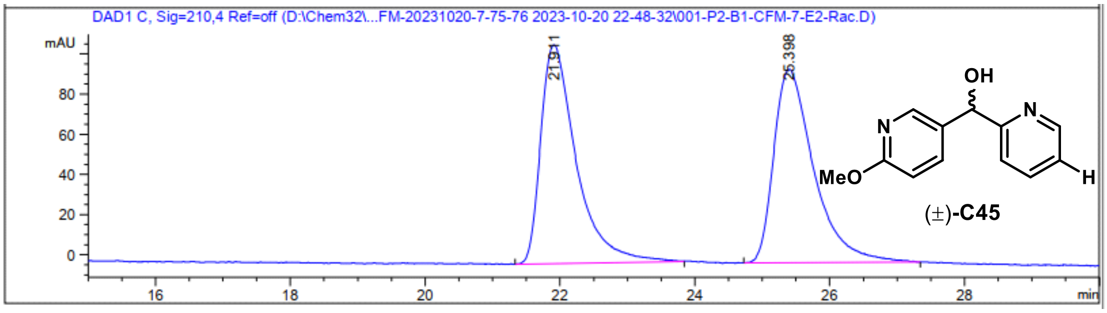

Peak #	RetTime [min]	Type	Width [min]	Area [mAU*s]	Height [mAU]	Area %
1	21.911	BB	0.5218	3970.60522	108.79224	50.0074
2	25.398	BV R	0.5911	3969.42358	96.25262	49.9926

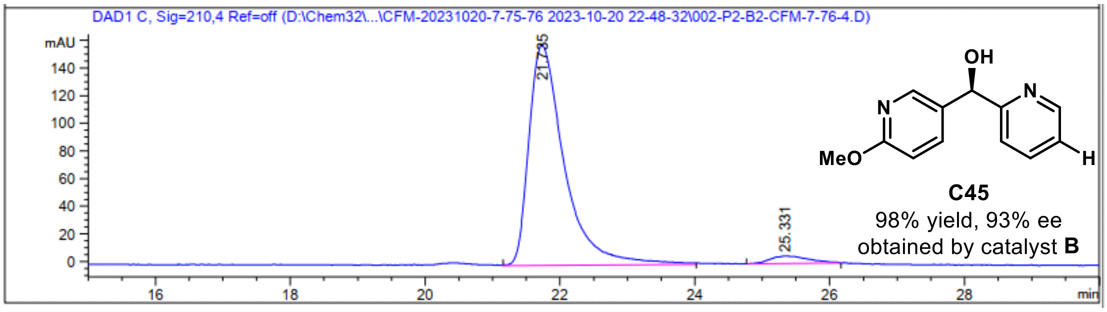

Peak #	RetTime [min]	Type	Width [min]	Area [mAU*s]	Height [mAU]	Area %
1	21.735	MM R	0.6072	5802.25391	159.26407	96.3576
2	25.331	BB	0.4767	219.32822	5.53064	3.6424

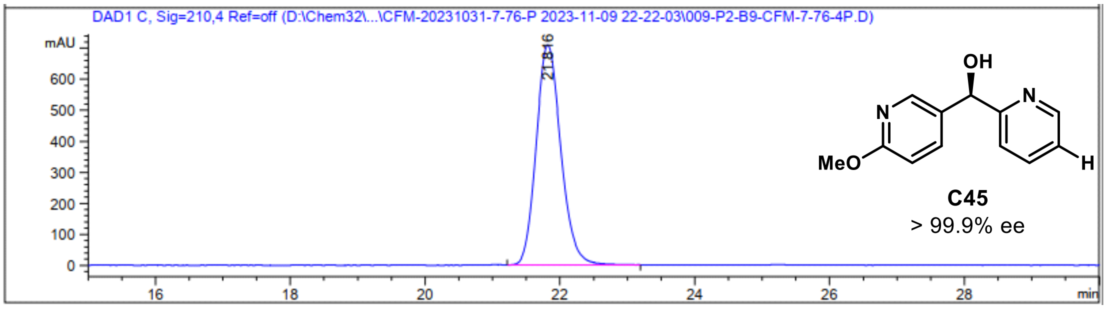

Peak #	RetTime [min]	Type	Width [min]	Area [mAU*s]	Height [mAU]	Area %
1	21.816	BV R	0.3940	1.79851e4	708.93860	100.0000

Supplementary Table 46. Crystal data and structure refinement for **C45**

CCDC Number	2311155
Empirical formula	$C_{12}H_{12}N_2O_2$
Formula weight	216.24
Temperature/K	100.0(2)
Crystal system	orthorhombic
Space group	P212121
a/Å	7.4325(11)
b/Å	9.7760(14)
c/Å	14.772(2)
α/°	90
β/°	90
γ/°	90
Volume/Å^3	1073.4(3)
Z	4
ρcalcg/cm^3	1.338
μ/mm-1	0.484
F(000)	456.0
Crystal size/mm^3	0.18 × 0.16 × 0.16
Radiation	GaKα (λ = 1.34139)
2Θ range for data collection/°	9.438 to 126.77
Index ranges	$-9 \le h \le 9$, $-13 \le k \le 13$, $-19 \le l \le 19$
Reflections collected	50248
Independent reflections	2649 [R_{int} = 0.0432, R_{sigma} = 0.0204]
Data/restraints/parameters	2649/0/148
Goodness-of-fit on F^2	1.050
Final R indexes [I>=2σ (I)]	R_1 = 0.0265, wR_2 = 0.0701
Final R indexes [all data]	R_1 = 0.0266, wR_2 = 0.0702
Largest diff. peak/hole / e Å^{-3}	0.27/-0.17
Flack parameter	0.03(3)

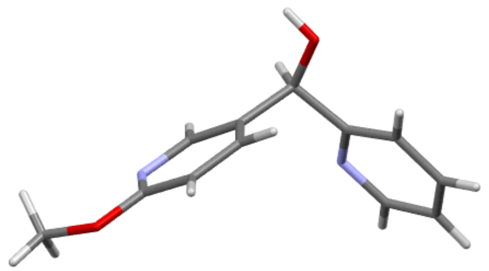

Supplementary Figure 45. The single crystal configuration of **C45** is demonstrated in a capped sticks mode.

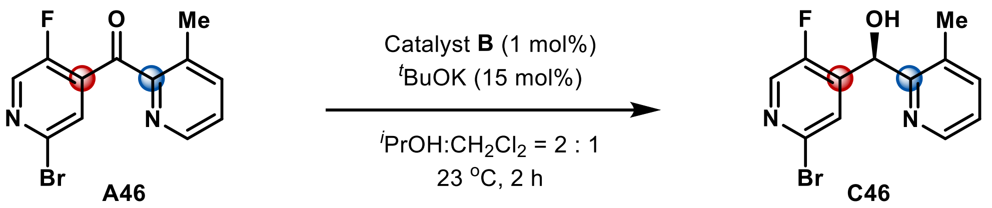

Following the general procedure, **C46** (51.5 mg, 87% yield, 72% ee) was obtained as white solid. The retention time in HPLC of the major enantiomer is consistent with that obtained by Ru-catalyst **B2,** and the *X*-ray crystal of the optically pure major enantiomer (100% ee) has been previously reported[2].

^{1}H NMR (400 MHz, CDCl$_3$): δ 8.50 (s, 1H), 8.22 (s, 1H), 7.52 (d, *J* = 7.6 Hz, 1H), 7.26 (dd, *J* = 7.6, 4.8 Hz, 1H), 7.22 (d, *J* = 4.8 Hz, 1H), 6.06 (s, 1H), 5.93 (s, 1H), 2.16 (s, 3H) ppm
^{13}C NMR (101 MHz, CDCl$_3$): δ 157.0 (d, *J* = 256.7 Hz), 154.9, 145.7, 141.1 (d, *J* = 13.5 Hz), 139.2, 138.4 (d, *J* = 27.1 Hz), 136.4 (d, *J* = 2.9 Hz), 130.4, 127.6 (d, *J* = 1.2 Hz), 123.6, 64.3, 17.1 ppm
^{19}F NMR (376 MHz, CDCl3): δ -136.05 (s) ppm
HRMS (ESI+): calculated for $C_{12}H_{11}BrFN_2O$ [M+H]$^+$: 297.0033, found 297.0030.
HPLC (AD-H, 0.46*25 cm, 5μm, hexane / ethanol = 90/10, flow 1 mL/min, detection at 254 nm) retention time = 9.100 min (major) and 11.552 min (minor).

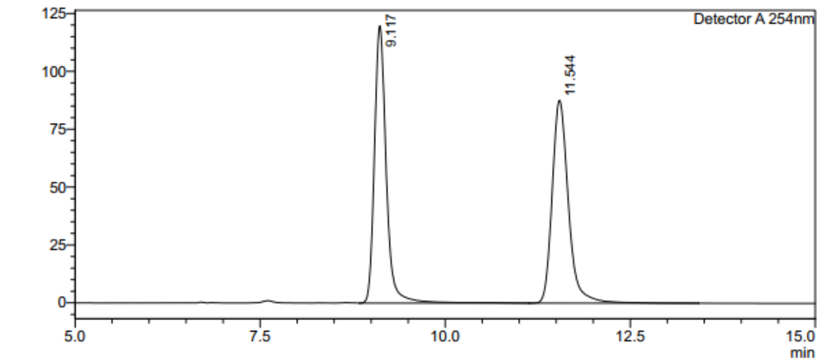

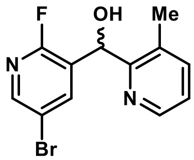

(±)-**C46**

<Peak Table>

Detector A 254nm

Peak#	Ret. Time	Area	Height	Conc.	Unit	Mark	Name
1	9.117	1326953	119791	49.856			
2	11.544	1334603	87652	50.144			
Total		2661556	207444				

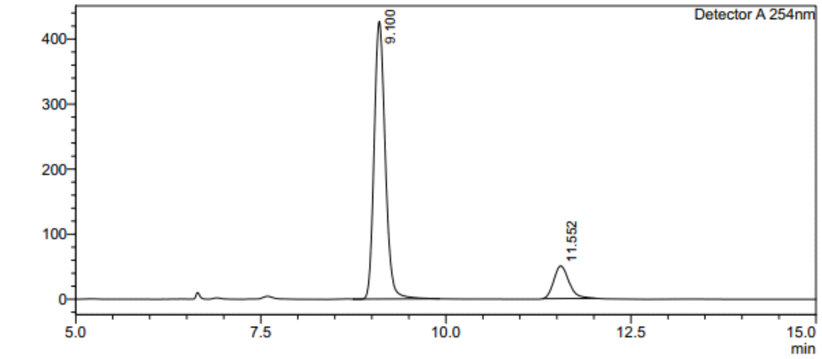

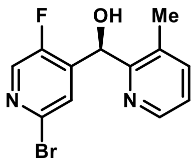

C46
87% yield, 72% ee
obtained by catalyst **B**

<Peak Table>

Detector A 254nm

Peak#	Ret. Time	Area	Height	Conc.	Unit	Mark	Name
1	9.100	4447847	426911	86.164		M	
2	11.552	714202	50190	13.836		M	
Total		5162050	477101				

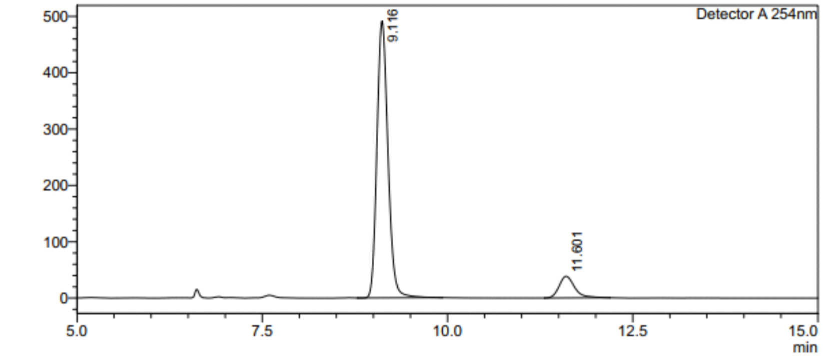

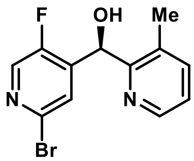

C46
90% yield, 80% ee
obtained byRu-catalyst **B2**

<Peak Table>

Detector A 254nm

Peak#	Ret. Time	Area	Height	Conc.	Unit	Mark	Name
1	9.116	5094106	491680	90.077		M	
2	11.601	561200	38356	9.923		M	
Total		5655306	530035				

Supplementary Table 47. Crystal data and structure refinement for **C46** [2]

CCDC number	2017295
Empirical formula	$C_{12}H_{10}BrFN_2O$
Formula weight	297.13
Temperature/K	100
Crystal system	monoclinic
Space group	$P2_1$
a/Å	9.2874(5)
b/Å	6.1502(3)
c/Å	10.0325(5)
α/°	90
β/°	102.2135(17)
γ/°	90
Volume/Å^3	560.08(5)
Z	2
ρ_{calc}g/cm^3	1.762
μ/mm^{-1}	3.666
F(000)	296.0
Crystal size/mm^3	0.42 × 0.38 × 0.38
Radiation	MoKα (λ = 0.71073)
2Θ range for data collection/°	5.432 to 61.192
Index ranges	-13 ≤ h ≤ 13, -8 ≤ k ≤ 8, -14 ≤ l ≤ 14
Reflections collected	11314
Independent reflections	3420 [R_{int} = 0.0545, R_{sigma} = 0.0509]
Data/restraints/parameters	3420/1/156
Goodness-of-fit on F^2	1.085
Final R indexes [I>=2σ (I)]	R_1 = 0.0350, wR_2 = 0.0853
Final R indexes [all data]	R_1 = 0.0372, wR_2 = 0.0866
Largest diff. peak/hole / e Å^{-3}	0.46/-0.79
Flack parameter	-0.011(11)

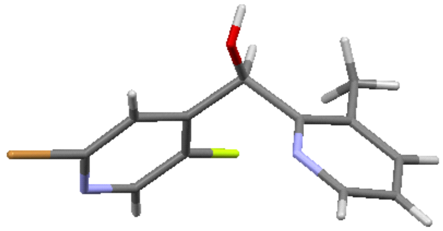

Supplementary Figure 46. The single crystal configuration of **C46** is demonstrated in a capped sticks mode.

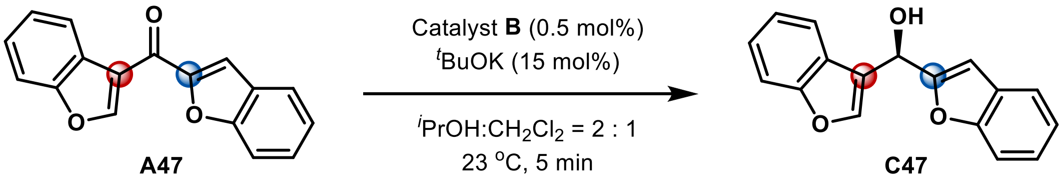

Following the general procedure, **C47** (45.8 mg, 87% yield, 77% ee) was obtained as white solid.

^{1}H NMR (400 MHz, CDCl$_3$): δ 7.72 (s, 1H), 7.61 (d, J = 7.6 Hz, 1H), 7.53 (t, J = 8.4 Hz, 2H), 7.48 (d, J = 7.6 Hz, 1H), 7.35 – 7.27 (m, 2H), 7.26 – 7.19 (m, 2H), 6.68 (s, 1H), 6.21 (s, 1H) ppm

^{13}C NMR (101 MHz, CDCl$_3$): δ 156.9, 155.7, 155.0, 142.9, 127.9, 125.8, 124.7, 124.6, 123.0, 122.9, 121.3, 120.6, 120.5, 111.7, 111.4, 104.2, 63.7 ppm

HRMS (ESI+): calculated for $C_{17}H_{11}O_2$ [M+H-H_2O]$^+$: 247.0753, found 247.0754.

HPLC (OD-H, 0.46*25 cm, 5μm, hexane / isopropanol = 80/20, flow 1 mL/min, detection at 254nm) retention time = 16.123 min (major) and 17.536 min (minor).

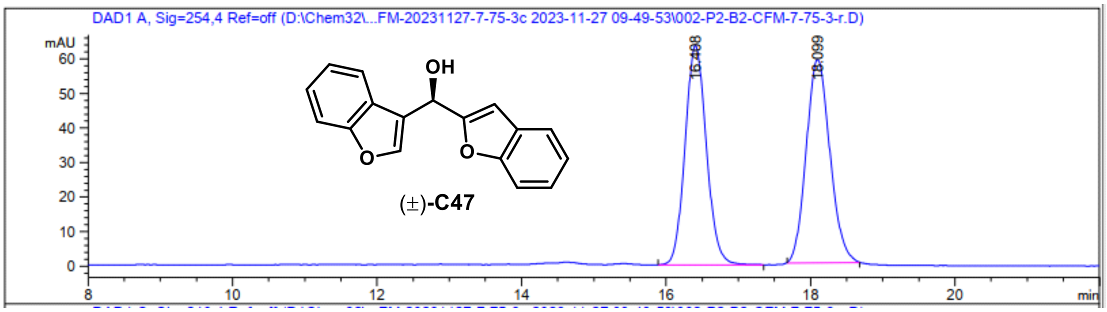

Peak #	RetTime [min]	Type	Width [min]	Area [mAU*s]	Height [mAU]	Area %
1	16.408	BB	0.3190	1300.63452	63.49693	49.2126
2	18.099	PM R	0.3801	1342.25439	58.84896	50.7874

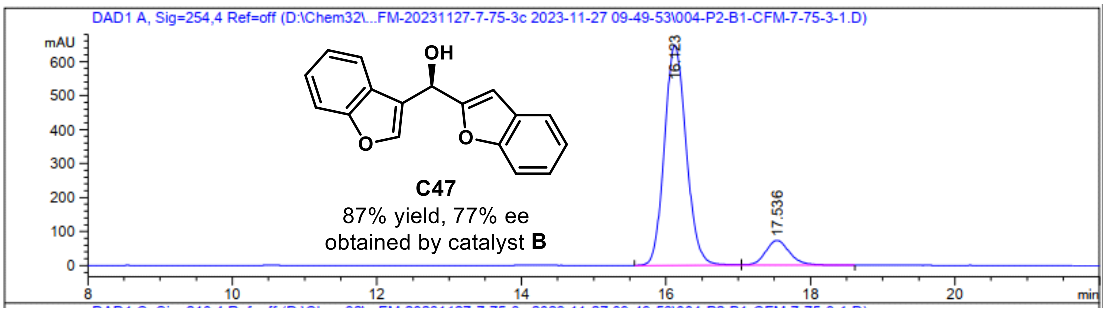

Peak #	RetTime [min]	Type	Width [min]	Area [mAU*s]	Height [mAU]	Area %
1	16.123	BV	0.3246	1.35773e4	647.62219	88.7772
2	17.536	VB	0.3594	1716.38464	73.24908	11.2228

The absolute configuration of **C47** was determined by ECD. The initial structures were searched using Grimme's programs xTB 6.3, and then were optimized at the level of M06-2X/Def2TZVP/IEFPCM($CHCl_3$). The obtained conformations were performing ECD calculations using TD-M06-2X/Def2TZVP/IEFPCM($CHCl_3$). The final ECD spectrum were formed based on Boltzmann populations. The calculated ECD spectrums indicate that the major product **C47** has ***R*** configuration.

Samples of **C47** for ECD were dissolved in MeCN, and spectra were acquired in a 1.0-mm pathlength cuvette, respectively. The ECD spectra were recorded using a Chirascan Spectrophotometer with the following instrumental parameters: 205–310 nm with a 1 nm step and a 2 nm bandwidth with data averaging over 1.0 sec per point. Three spectral acquisitions were taken for each sample and were averaged and smoothed thereafter.

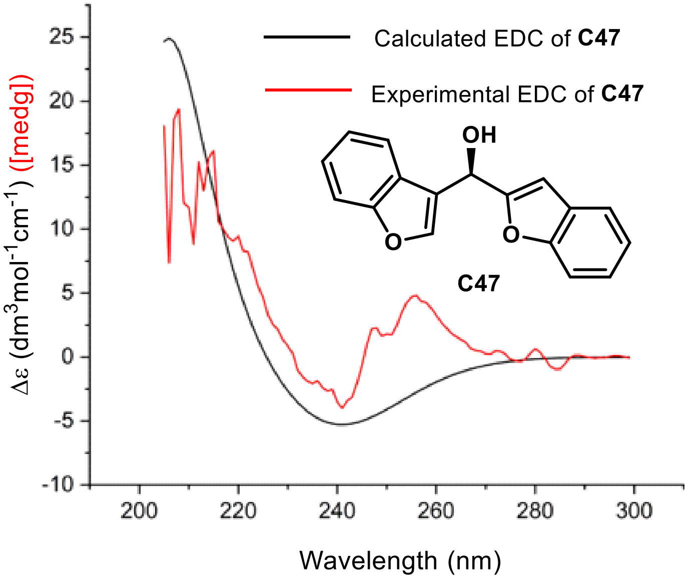

Supplementary Figure 47. The experimental and calculated ECD spectrum of **C47** at the level of TD-M06-2X/Def2TZVP/IEFPCM($CHCl_3$)//M06-2X/Def2TZVP/IEFPCM($CHCl_3$).

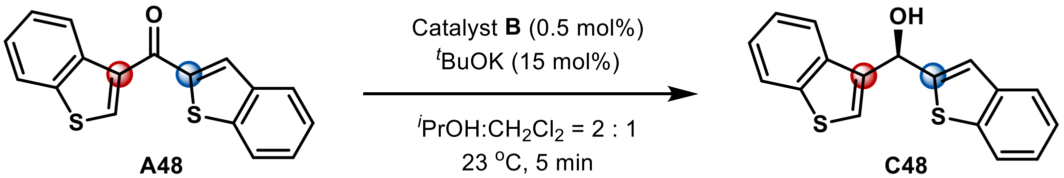

A48 **C48**

Following the general procedure, **C48** (49.2 mg, 83% yield, 91% ee) was obtained as white solid.

^{1}H NMR (400 MHz, CDCl$_3$): δ 7.90 (d, J = 7.0 Hz, 1H), 7.82 (t, J = 9.0 Hz, 2H), 7.71 (d, J = 7.0 Hz, 1H), 7.60 (s, 1H), 7.41 – 7.29 (m, 4H), 7.25 (s, 1H), 6.50 (s, 1H) ppm

^{13}C NMR (101 MHz, CDCl$_3$): δ 146.9, 140.9, 139.9, 139.4, 137.3, 136.9, 124.6, 124.4, 124.3, 124.3, 124.2, 123.7, 122.9, 122.5, 122.5, 121.7, 68.7 ppm

HRMS (ESI+): calculated for $C_{17}H_{11}S_2$ [M+H-H$_2$O]$^+$: 279.0297, found 279.0297.

HPLC (AD-H, 0.46*25 cm, 5μm, hexane / isopropanol = 90/10, flow 1 mL/min, detection at 210nm) retention time = 42.820 min (minor) and 45.651 min (major).

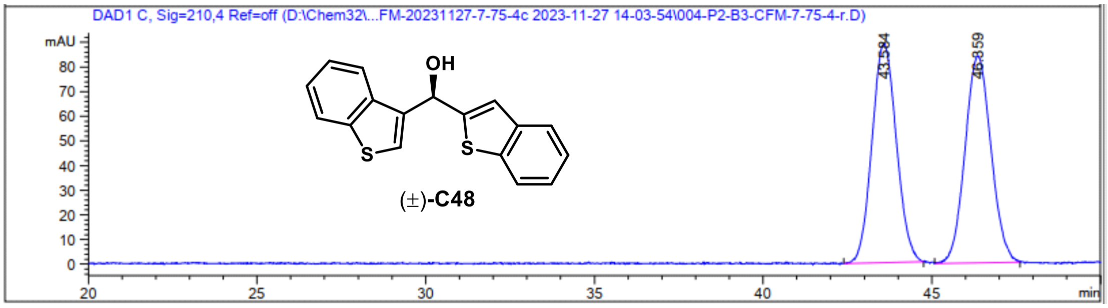

Peak #	RetTime [min]	Type	Width [min]	Area [mAU*s]	Height [mAU]	Area %
1	43.584	VB R	0.6061	4461.24854	88.80682	49.9191
2	46.359	VB R	0.6747	4475.71582	83.85410	50.0809

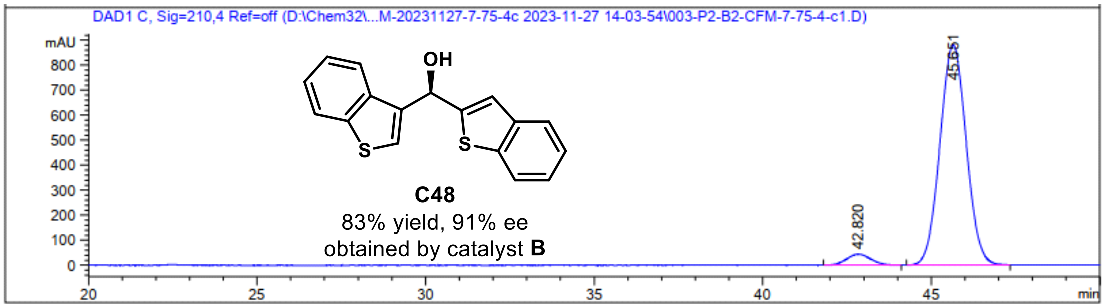

Peak #	RetTime [min]	Type	Width [min]	Area [mAU*s]	Height [mAU]	Area %
1	42.820	VV R	0.5969	2194.01025	44.06166	4.3996
2	45.651	BB	0.6673	4.76740e4	881.03253	95.6004

The absolute configuration of **C48** was determined by ECD. The initial structures were searched using Grimme's programs xTB 6.3, and then were optimized at the level of M06-2X/Def2TZVP/IEFPCM($CHCl_3$). The obtained conformations were performing ECD calculations using TD-M06-2X/Def2TZVP/IEFPCM($CHCl_3$). The final ECD spectrum were formed based on Boltzmann populations. The calculated ECD spectrum indicate that the major product **C48** has ***R*** configuration.

Samples of **C48** for ECD were dissolved in MeCN, and spectra were acquired in a 1.0-mm pathlength cuvette, respectively. The ECD spectra were recorded using a Chirascan Spectrophotometer with the following instrumental parameters: 205–310 nm with a 1 nm step and a 2 nm bandwidth with data averaging over 1.0 sec per point. Three spectral acquisitions were taken for each sample and were averaged and smoothed thereafter.

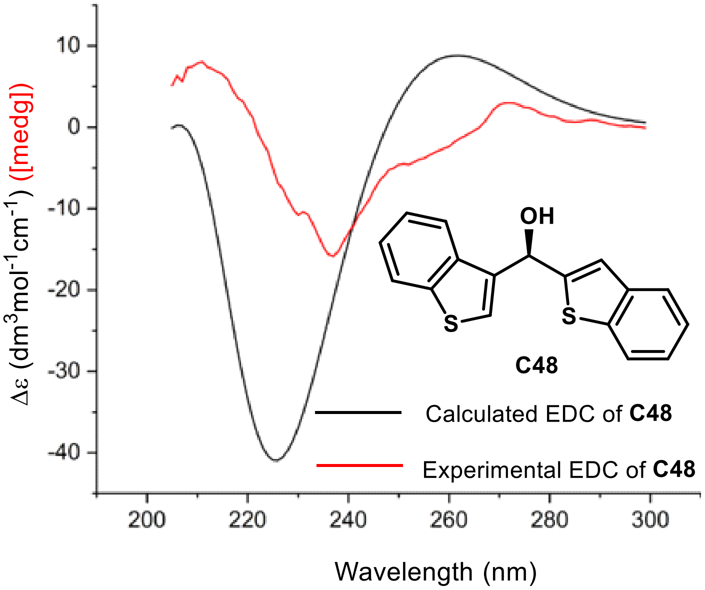

Supplementary Figure 48. The experimental and calculated ECD spectrum of **C48** at the level of TD-M06-2X/Def2TZVP/IEFPCM($CHCl_3$)//M06-2X/Def2TZVP/IEFPCM($CHCl_3$).

2. Linear free energy relationship (LFER) studies of substrate local polarizability on enantio-selection

2.1 System 1: Noyori-type Ru-catalyzed transfer hydrogenation of di-aryl ketones

Asymmetric catalytic systems that are suitable for this direct and quantitative study should be irreversible, highly reproducible and mechanistically clear. Although our Noyori-type Ru-catalyzed asymmetric transfer hydrogenation with *i*-PrOH as hydrogen source are partially reversible[3], a quantitative LFER between substrates' local polarizabilities and enantioselectivities is demonstrated here in a series of *di*-aryl ketones where one of the aryl rings are substituted by both electron-donating and withdrawing groups.

The reactions were run at relatively low conversions (standard conditions for 5 minutes) to prevent reversibility that could erode alcohol products' enantioselectivities (%ee) to some extents. For the quantitative studies, the local polarizabilities were calculated using a scheme based on Tkatchenko-Scheffler model[4], which appears to be more accurate than the simple D4-based method in constructing quantitative correlations with free energies. As shown in **Supplementary Figure 49**, the decrease in the substrates' local polarizabilities of the carbon marked in blue correlated well ($R^2 = 0.905$) with the increase in the difference of free energies of activation ($\Delta\Delta G^{\ddagger}$) derived from experimentally observed reaction enantioselectivities (%ee).

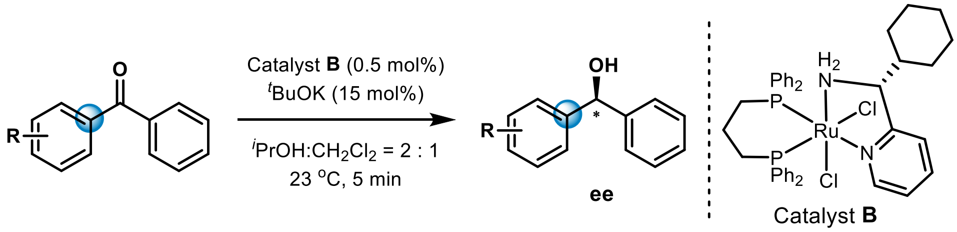

	R	●	%ee
1	4-OMe	9.956	32
2	4-Me	9.934	23
3	4-H	9.917	0
4	4-Br	9.888	-7
5	4-CF_3	9.862	-10
6	3,5-*di*-CF3	9.809	-28

● *blue carbon's local polarizability*

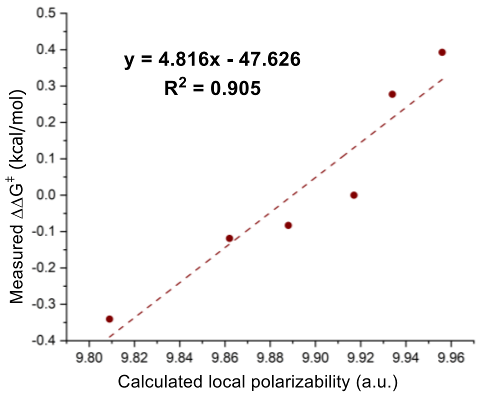

Supplementary Figure 49. Linear free energy relationship (LFER) analysis of substrate local polarizabilities on enantio-selections in the Ru-catalyzed asymmetric transfer hydrogenation of *di*-aryl ketones.

HPLC of the alcohol products

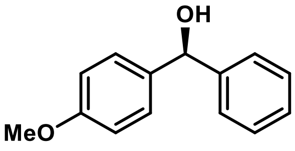

C49

HPLC (AD-H, 0.46*25 cm, 5μm, hexane / isopropanol = 90/10, flow 1 mL/min, detection at 210 nm) retention time = 19.196 min (major) and 20.825 min (minor).

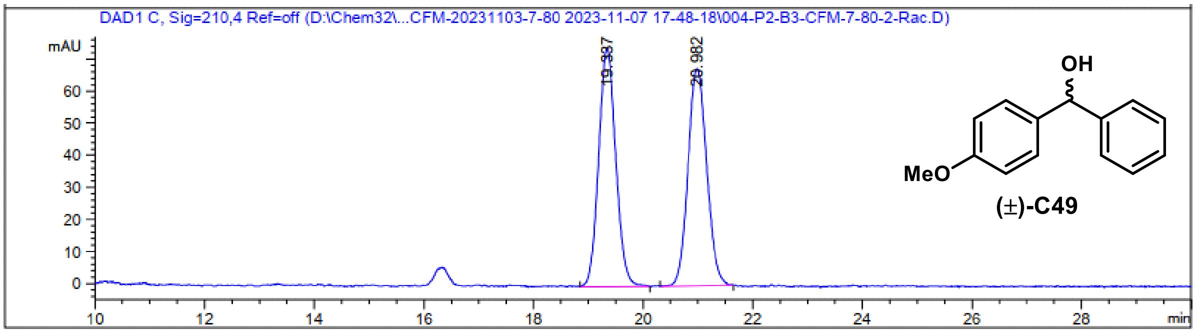

Peak #	RetTime [min]	Type	Width [min]	Area [mAU*s]	Height [mAU]	Area %
1	19.337	BB	0.3283	1621.09143	74.35900	50.2913
2	20.982	VV R	0.3540	1602.30920	67.74187	49.7087

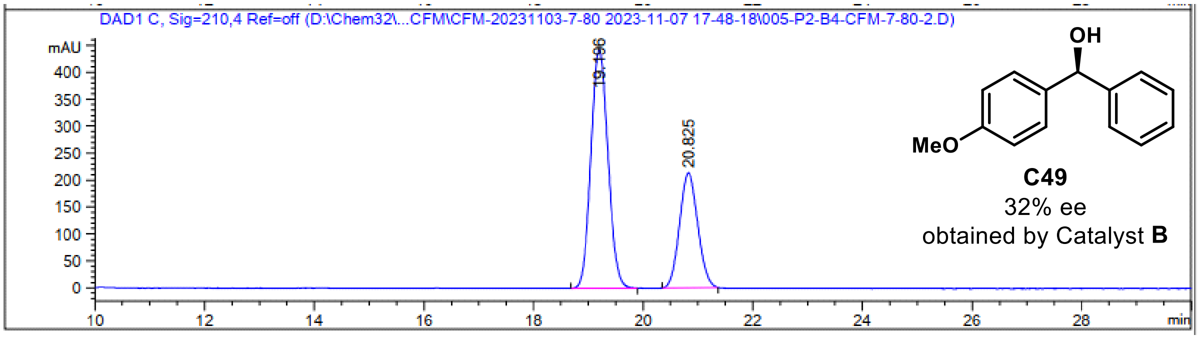

Peak #	RetTime [min]	Type	Width [min]	Area [mAU*s]	Height [mAU]	Area %
1	19.196	BB	0.3379	9560.39355	442.75183	65.9137
2	20.825	MM R	0.3851	4944.01709	213.95280	34.0863

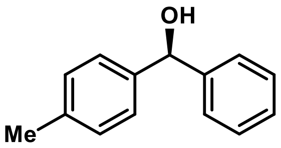

C50

HPLC (AD-H, 0.46*25 cm, 5μm, hexane / isopropanol = 95/5, flow 1 mL/min, detection at 210 nm) retention time = 20.469 min (major) and 22.146 min (minor).

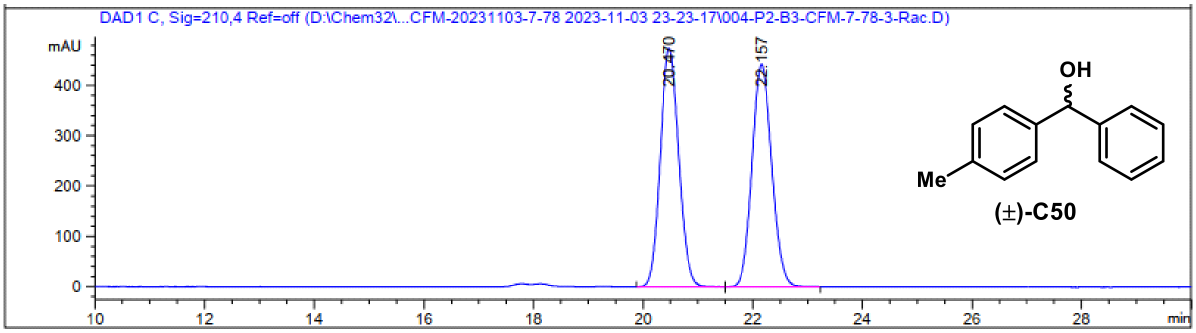

Peak #	RetTime [min]	Type	Width [min]	Area [mAU*s]	Height [mAU]	Area %
1	20.470	BV R	0.3675	1.11113e4	474.04703	49.9864
2	22.157	VB	0.3972	1.11173e4	442.40363	50.0136

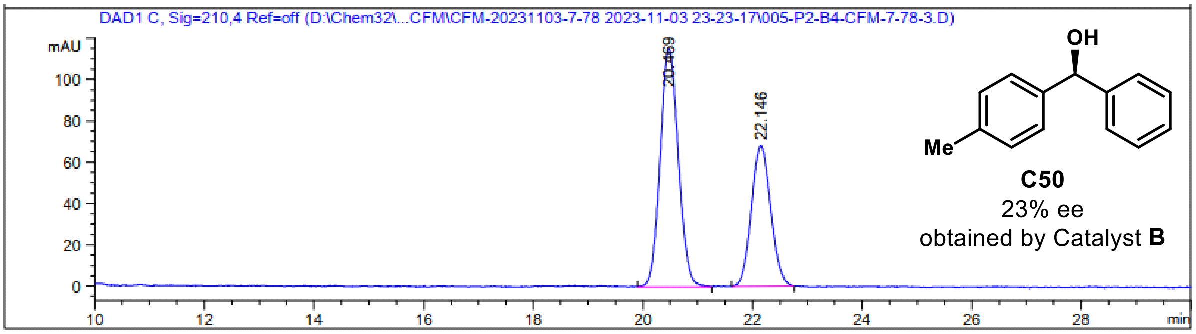

Peak #	RetTime [min]	Type	Width [min]	Area [mAU*s]	Height [mAU]	Area %
1	20.469	MM R	0.3867	2678.93335	115.45055	61.5030
2	22.146	BB	0.3730	1676.84021	68.17115	38.4970

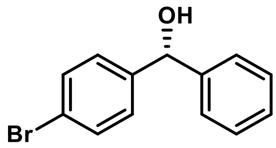

C52

HPLC (AD-H, 0.46*25 cm, 5μm, hexane / isopropanol = 95/5, flow 1 mL/min, detection at 210 nm) retention time = 21.964 min (major) and 25.088 min (minor).

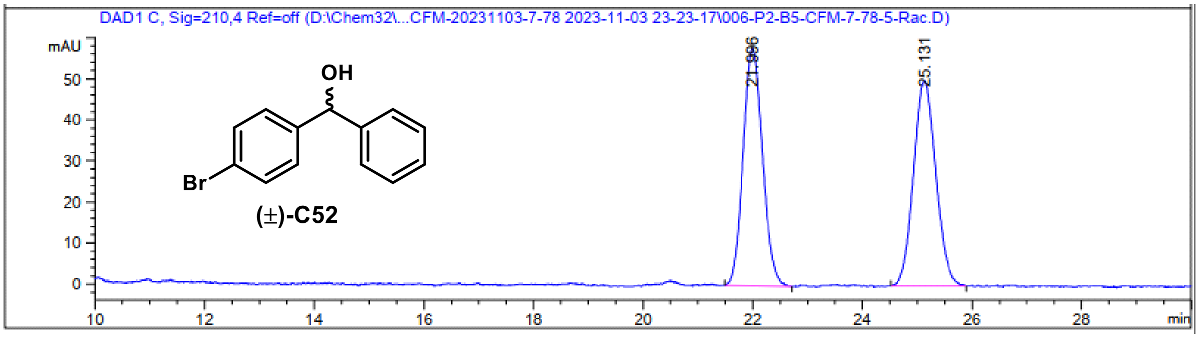

Peak #	RetTime [min]	Type	Width [min]	Area [mAU*s]	Height [mAU]	Area %
1	21.996	BB	0.3664	1419.30005	57.81200	49.9273
2	25.131	BB	0.3979	1423.43359	50.04085	50.0727

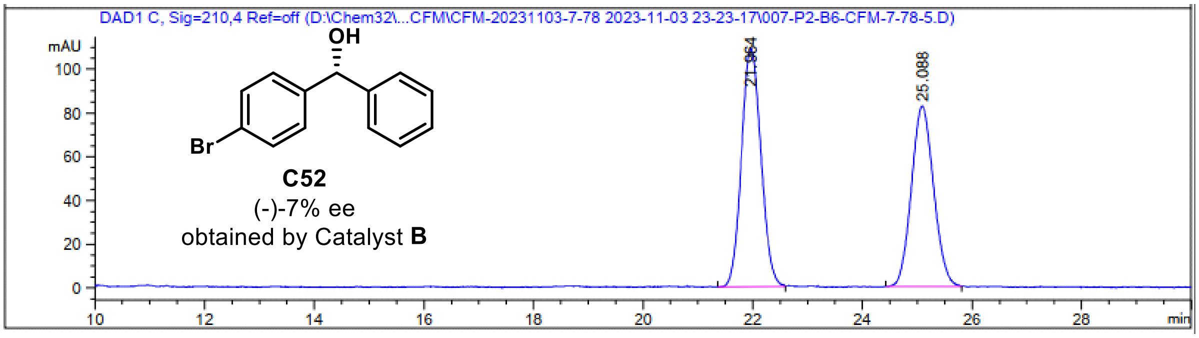

Peak #	RetTime [min]	Type	Width [min]	Area [mAU*s]	Height [mAU]	Area %
1	21.964	MM R	0.4104	2679.78906	108.83952	53.5031
2	25.088	MM R	0.4700	2328.87183	82.58098	46.4969

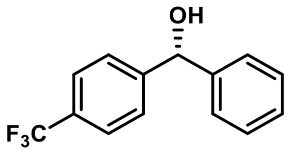

C54

HPLC (AD-H, 0.46*25 cm, 5μm, hexane / isopropanol = 90/10, flow 1 mL/min, detection at 210 nm) retention time = 9.785 min (minor) and 11.758 min (major).

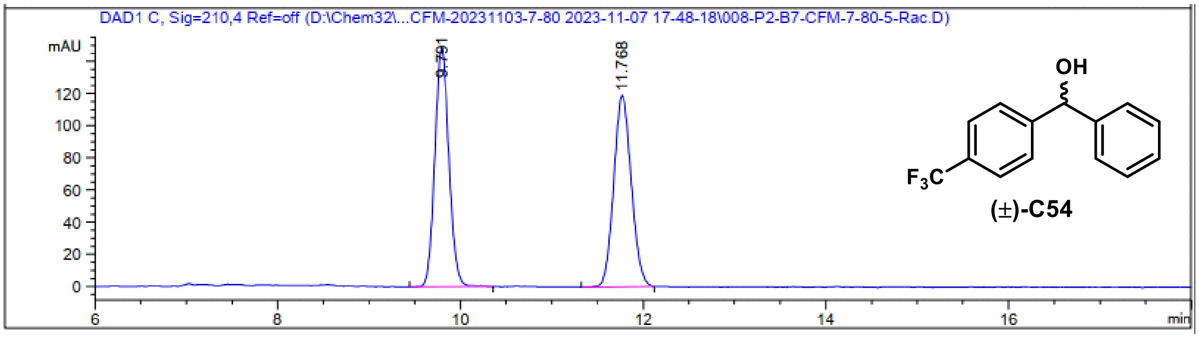

Peak #	RetTime [min]	Type	Width [min]	Area [mAU*s]	Height [mAU]	Area %
1	9.791	BV R	0.1683	1604.36353	148.16185	50.1511
2	11.768	BB	0.2083	1594.69788	118.99476	49.8489

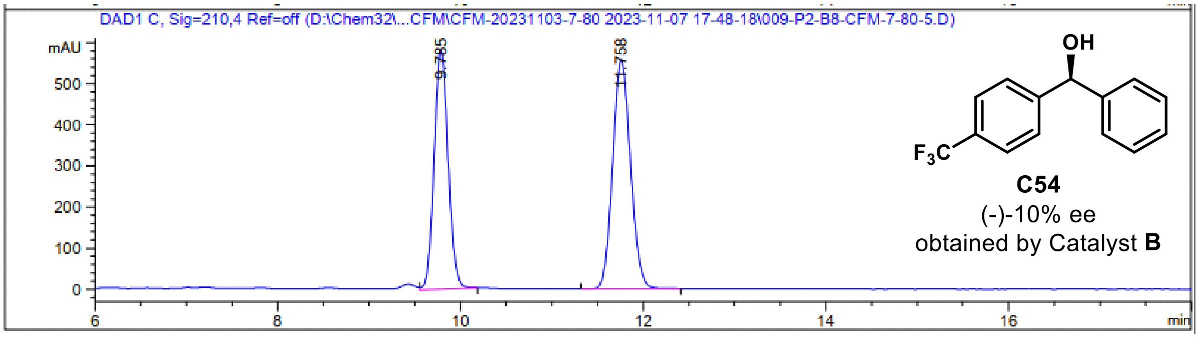

Peak #	RetTime [min]	Type	Width [min]	Area [mAU*s]	Height [mAU]	Area %
1	9.785	VB	0.1656	188.79750	17.82530	44.9750
2	11.758	BB	0.2120	230.98526	17.05358	55.0250

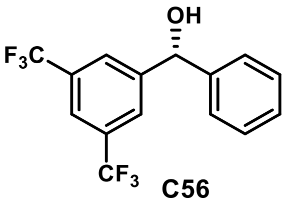

C56

HPLC (AD-H, 0.46*25 cm, 5μm, hexane / isopropanol = 98/2, flow 1 mL/min, detection at 210 nm) retention time = 12.536 min (minor) and 13.393 min (major).

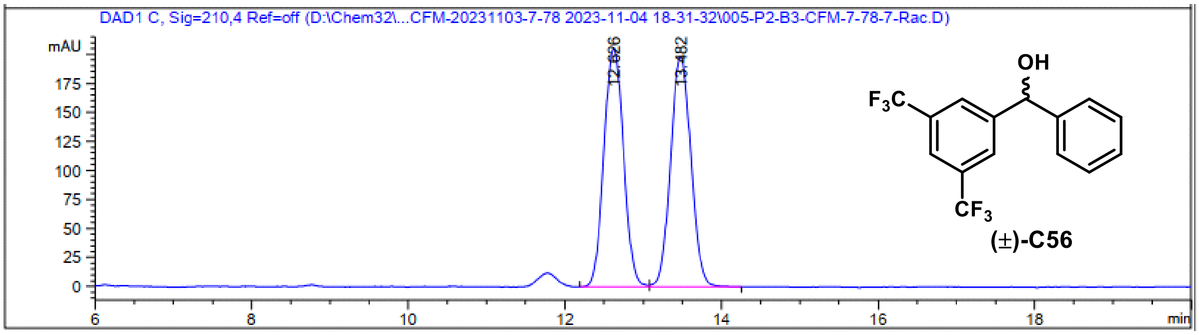

Peak #	RetTime [min]	Type	Width [min]	Area [mAU*s]	Height [mAU]	Area %
1	12.626	BV	0.2751	3576.65942	205.41006	49.8457
2	13.482	VB	0.2836	3598.80371	198.38995	50.1543

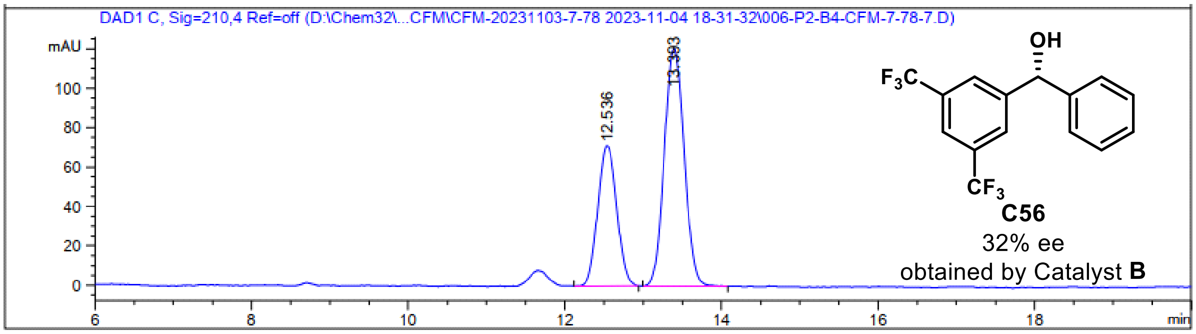

Peak #	RetTime [min]	Type	Width [min]	Area [mAU*s]	Height [mAU]	Area %
1	12.536	MM R	0.2815	1202.11243	71.16537	36.0760
2	13.393	BB	0.2779	2130.05151	120.68185	63.9240

2.2 System 2: Noyori-Ikariya Ru-catalyzed transfer hydrogenation of ketones

Supplementary Table 47. Noyori-Ikariya Ru-catalyzed transfer hydrogenation of ketones

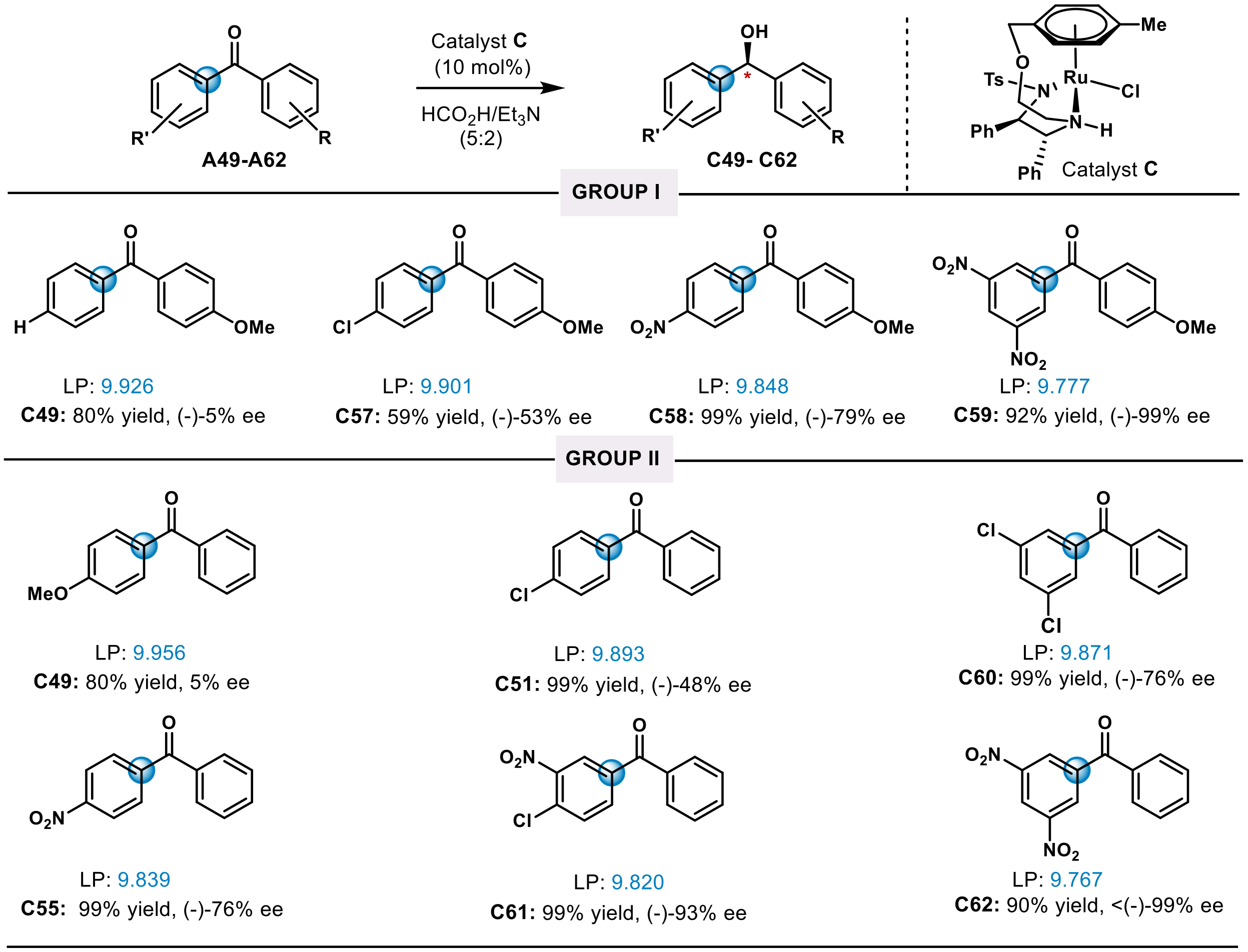

LP: blue carbon's local polarizability

The experimental results were reported by Ikariya[4] and the carbonyl substrates' local polarizabilities were calculated by using Tkatchenko-Scheffler model[5]. Based on these experimental and computational data, the quantitative linear free energy correlations between the substrate local polarizabilities and the observed magnitudes of reaction enantio-selection ($\Delta\Delta G^{‡}$ values) in Noyori-Ikariya system were conducted as below (**Supplementary Figure 50**).

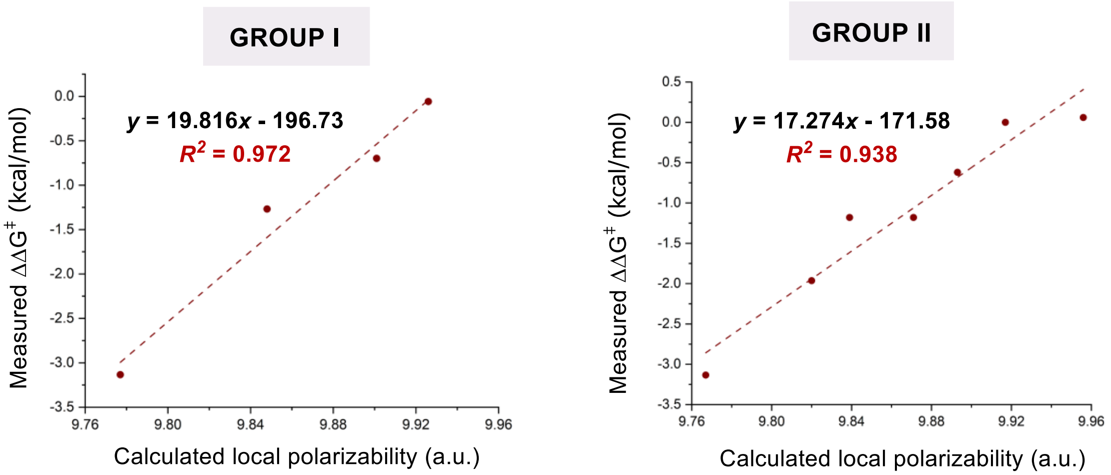

Supplementary Figure 50. LFER for ATH of ketones in Noyori-Ikariya system

2.3 System 3: Oxazaborolidines-catalyzed reductions of ketones with Corey-Bakshi-Shibata (CBS) catalyst

Supplementary Table 48. Corey-Bakshi-Shibata (CBS) reduction of ketones with catalyst **D1**

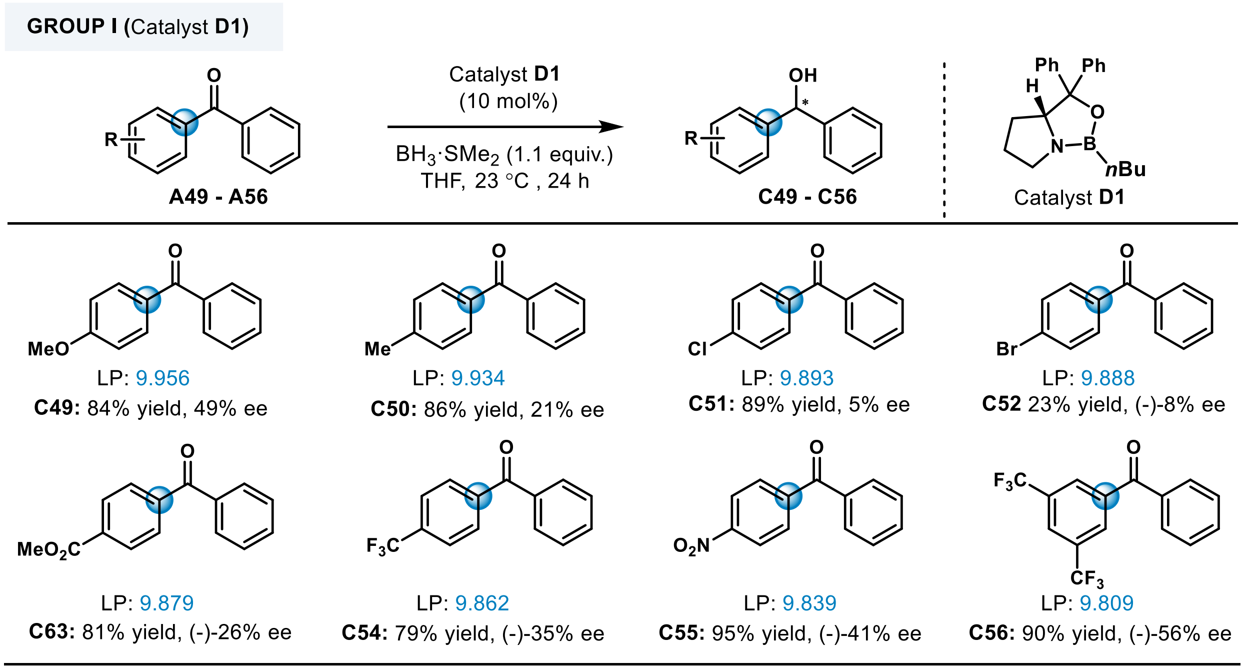

LP: blue carbon's local polarizability

Supplementary Table 49. Corey-Bakshi-Shibata (CBS) reduction of ketones with catalyst **D2**

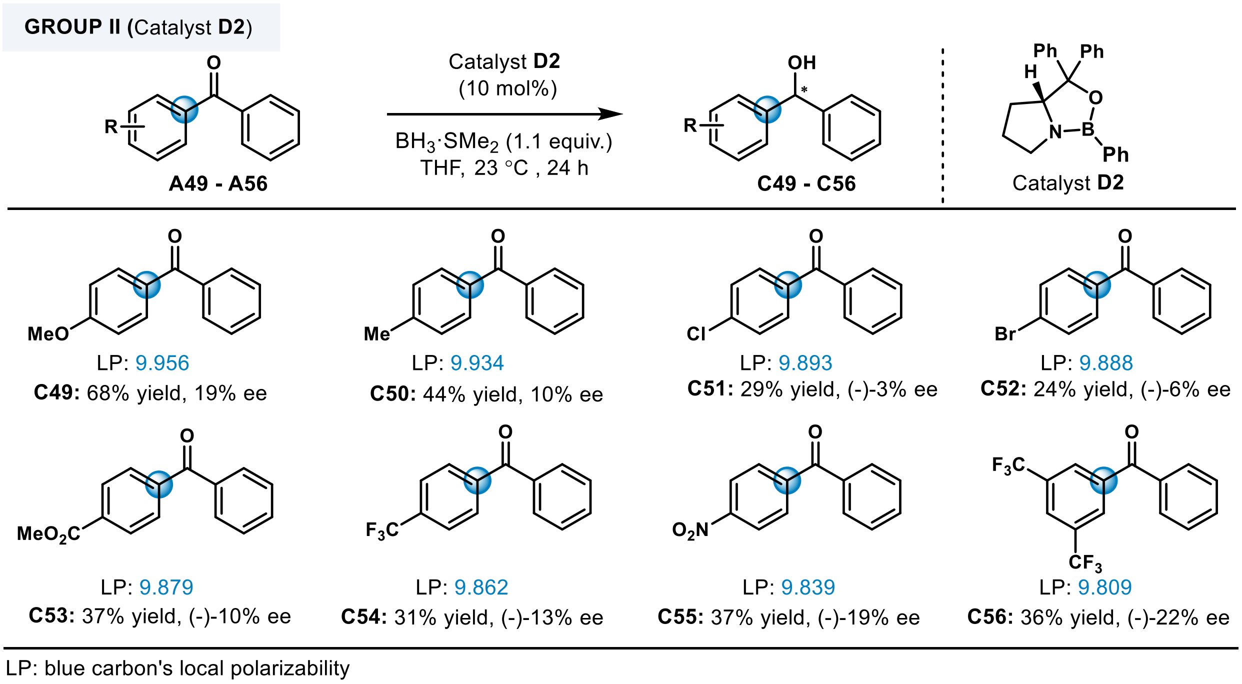

The CBS reduction was conducted under standard conditions[6] and the results with catalyst **D1** and catalyst **D2** were showed in **Supplementary Table 48** and **Supplementary Table 49**, respectively. The carbonyl substrates' local polarizabilities were calculated by using Tkatchenko-Scheffler model[5]. Based on these experimental and computational data, the quantitative linear free energy correlations between the substrate local polarizabilities and the observed magnitudes of reaction enantio-selection ($\Delta\Delta G^{‡}$ values) in CBS system were conducted as below (**Supplementary Figure 51**).

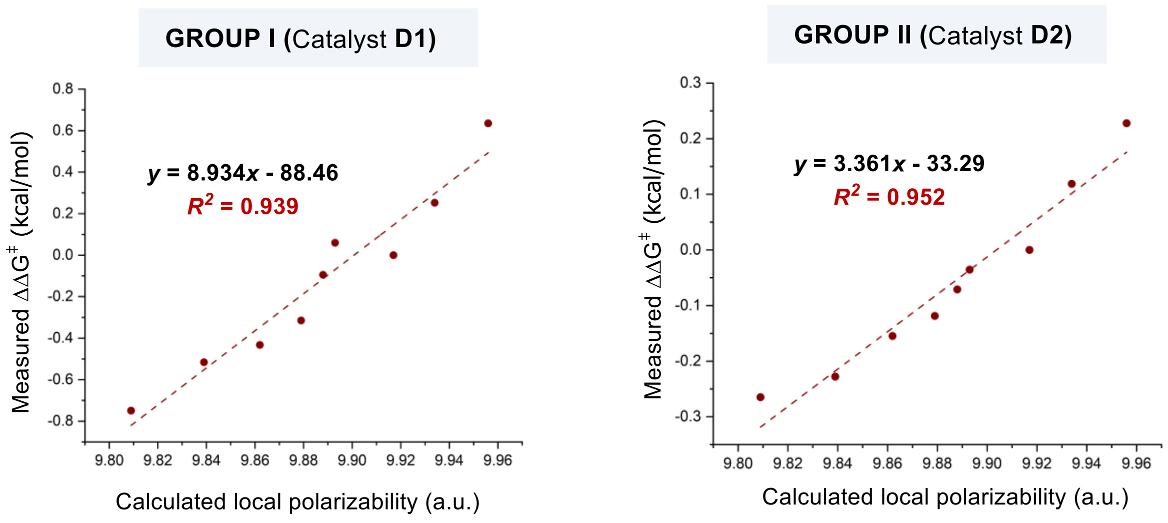

Supplementary Figure 51. LFER for Corey-Bakshi-Shibata (CBS) reduction of ketones

Characterization of the alcohol products

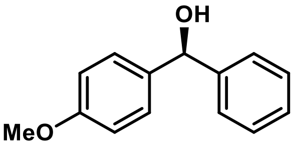

C49

Following the reported procedure with catalyst **D1**, **C49** (35.9 mg, 84% yield, 49% ee) was obtained as white solid.

^{1}H NMR (400 MHz, $CDCl_3$): δ 7.43 - 7.34 (m, 4H), 7.34 - 7.27 (m, 3H), 6.92 - 6.89 (m, 2H), 5.79 (s, 1H), 3.81 (s, 1H), 2.61 (s, 1H) ppm

^{13}C NMR (101 MHz, $CDCl_3$): δ 158.9, 144.0, 136.1, 128.3, 127.8, 127.3, 126.3, 113.8, 75.7, 55.2 ppm

HPLC (OD-H, 0.46*25 cm, 5μm, hexane / isopropanol = 90/10, flow 1 mL/min, detection at 210 nm) retention time = 14.280 min (minor) for enantiomer (*R*) and 15.496 min (major) for enantiomer (*S*).

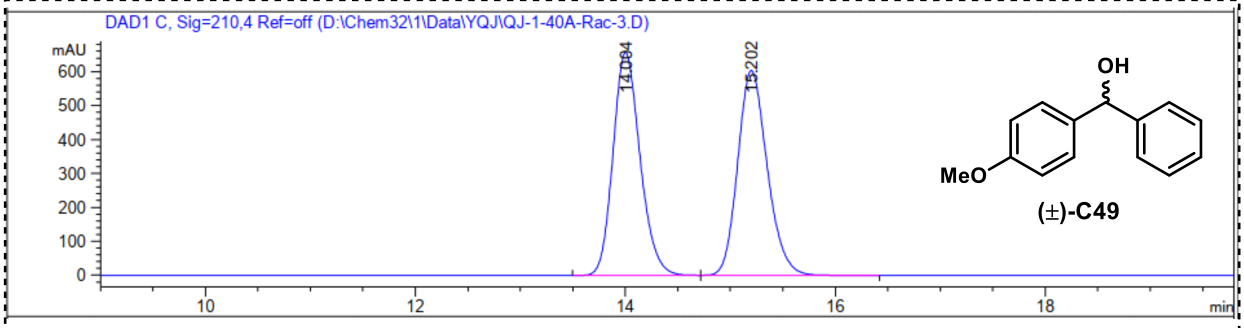

Peak #	RetTime [min]	Type	Width [min]	Area [mAU*s]	Height [mAU]	Area %
1	14.004	BB	0.2742	1.17405e4	657.83673	49.9500
2	15.202	BB	0.2994	1.17640e4	603.62738	50.0500

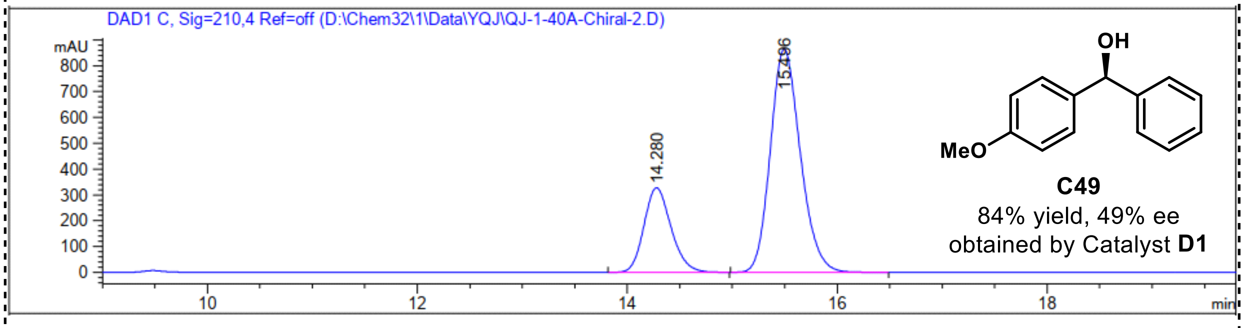

Peak #	RetTime [min]	Type	Width [min]	Area [mAU*s]	Height [mAU]	Area %
1	14.280	BB	0.2776	5873.27246	326.92300	25.4982
2	15.496	BB	0.3055	1.71608e4	864.92615	74.5018

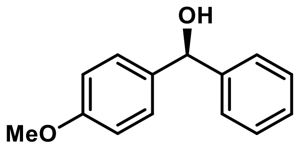

C49

Following the reported procedure with catalyst **D2**, **C49** (29.1 mg, 68% yield, 19% ee) was obtained as white solid.

HPLC (OD-H, 0.46*25 cm, 5μm, hexane / isopropanol = 90/10, flow 1 mL/min, detection at 210 nm) retention time = 14.641 min (minor) for enantiomer (*R*) and 15.876 min (major) for enantiomer (*S*).

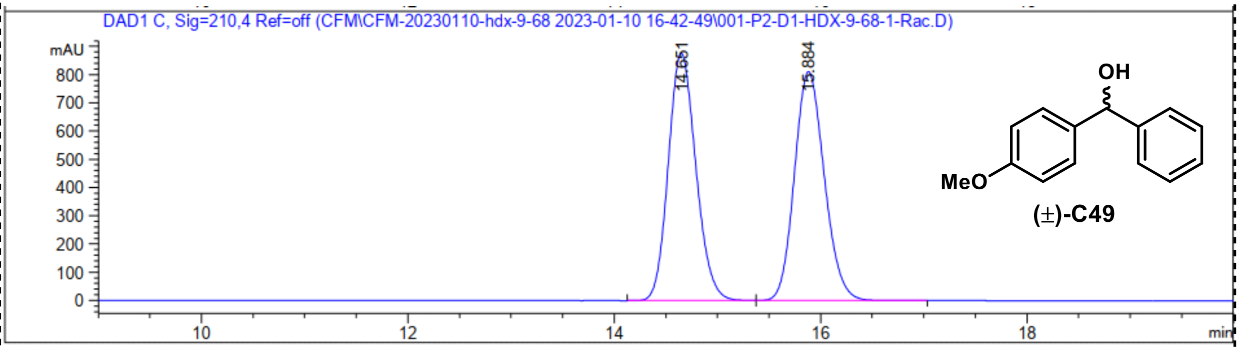

Peak #	RetTime [min]	Type	Width [min]	Area [mAU*s]	Height [mAU]	Area %
1	14.651	BB	0.2885	1.62758e4	876.94525	49.9535
2	15.884	BB	0.3106	1.63061e4	810.82074	50.0465

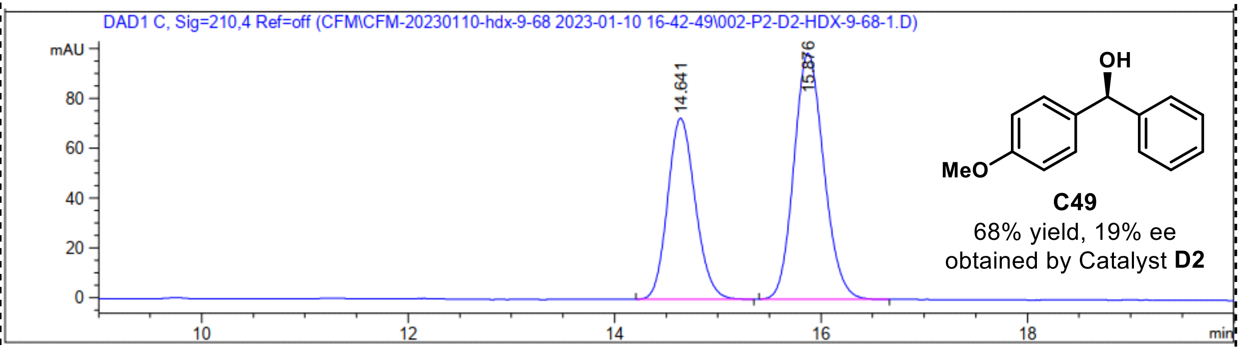

Peak #	RetTime [min]	Type	Width [min]	Area [mAU*s]	Height [mAU]	Area %
1	14.641	BB	0.2871	1351.32678	72.61492	40.4369
2	15.876	BB	0.3133	1990.49072	98.69794	59.5631

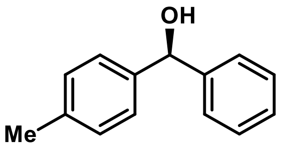

C50

Following the reported procedure with catalyst **D1**, **C50** (34.0 mg, 86% yield, 21% ee) was obtained as white solid.

^{1}H NMR (400 MHz, $CDCl_3$): δ 7.45 – 7.37 (m, 4H), 7.37 – 7.28 (m, 3H), 7.22 (d, *J* = 8.0 Hz, 2H), 5.78 (d, *J* = 3.6 Hz, 1H), 2.88 (d, *J* = 3.6 Hz, 1H), 2.43 (s, 3H) ppm

^{13}C NMR (101 MHz, $CDCl_3$): δ 143.9, 140.9, 137.0, 129.0, 128.2, 127.2, 126.4, 126.4, 75.8, 21.0 ppm

HPLC (AD-H, 0.46*25 cm, 5μm, hexane / isopropanol = 95/5, flow 1 mL/min, detection at 210 nm) retention time = 16.127 min (minor) for enantiomer (*R*) and 17.410 min (major) for enantiomer (*S*).

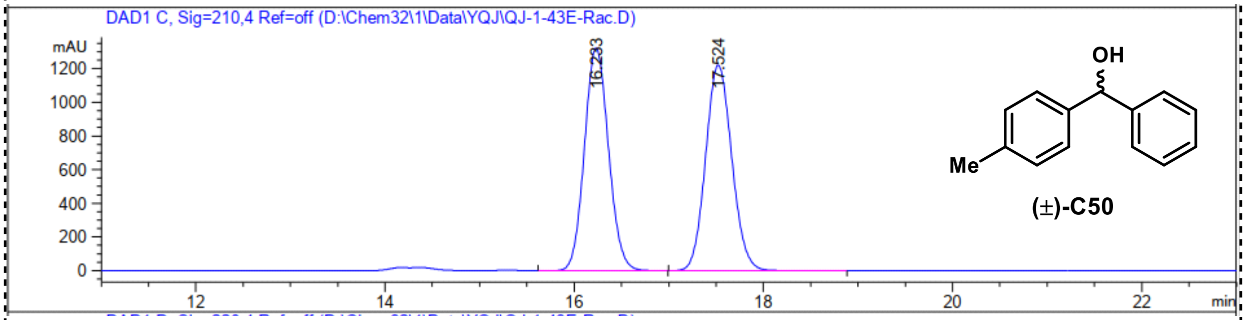

Peak #	RetTime [min]	Type	Width [min]	Area [mAU*s]	Height [mAU]	Area %
1	16.233	BB	0.2767	2.34328e4	1322.66418	49.8726
2	17.524	BB	0.3010	2.35525e4	1221.41626	50.1274

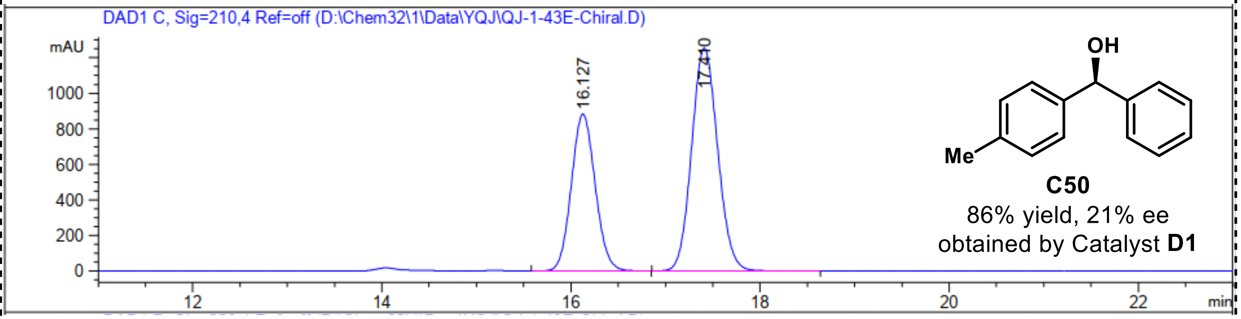

Peak #	RetTime [min]	Type	Width [min]	Area [mAU*s]	Height [mAU]	Area %
1	16.127	BB	0.2745	1.54898e4	883.82111	39.2047
2	17.410	BB	0.2993	2.40202e4	1254.78589	60.7953

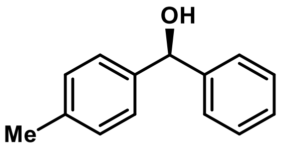

C50

Following the reported procedure with catalyst **D2**, **C50** (17.4 mg, 44% yield, 10% ee) was obtained as white solid.

HPLC (AD-H, 0.46*25 cm, 5μm, hexane / isopropanol = 95/5, flow 1 mL/min, detection at 210 nm) retention time = 12.123 min (minor) for enantiomer (*R*) and 13.101 min (major) for enantiomer (*S*).

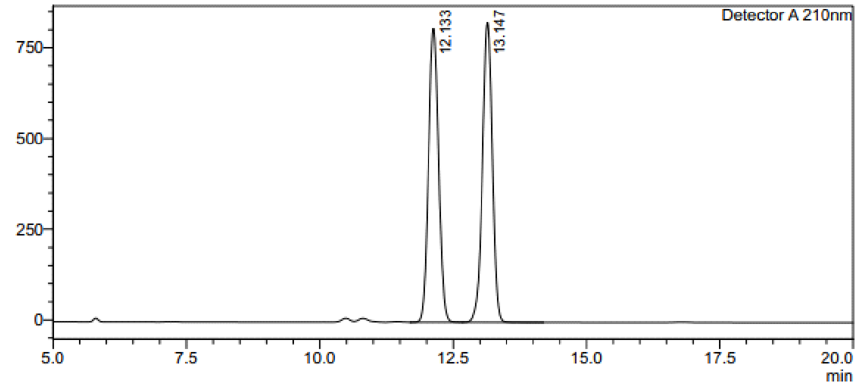

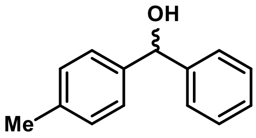

(±)-C50

<Peak Table>

Detector A 210nm

Peak#	Ret. Time	Area	Height	Conc.	Unit	Mark	Name
1	12.133	10654678	810011	49.991			
2	13.147	10658627	825997	50.009		V	
Total		21313305	1636008				

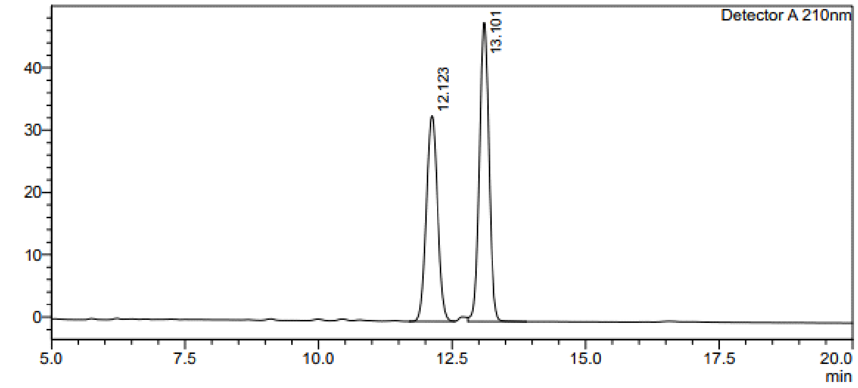

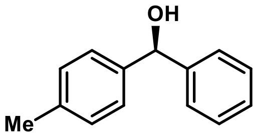

C50

44% yield, 10% ee

obtained by Catalyst **D2**

<Peak Table>

Detector A 210nm

Peak#	Ret. Time	Area	Height	Conc.	Unit	Mark	Name
1	12.123	483809	33023	44.800			
2	13.101	596117	47981	55.200		S	
Total		1079925	81004				

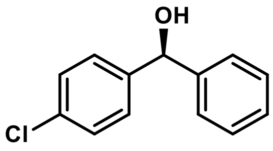

C51

Following the reported procedure with catalyst **D1**, **C51**(38.8 mg, 89% yield, 5% ee) was obtained as white solid.

^{1}H NMR (400 MHz, $CDCl_3$): δ 7.36 - 7.25 (m, 9H), 5.79 (d, *J* = 3.4 Hz, 1H), 2.32 (d, *J* = 3.5 Hz, 1H) ppm

^{13}C NMR (101 MHz, $CDCl_3$): δ 143.4, 142.2, 133.2, 128.6, 128.5, 127.8, 127.8, 126.5, 75.5 ppm

HPLC (OD-H, 0.46*25 cm, 5μm, hexane / isopropanol = 93/7, flow 1 mL/min, detection at 210 nm) retention time = 9.804 min (minor) for enantiomer (*R*) and 10.776 min (major) for enantiomer (*S*).

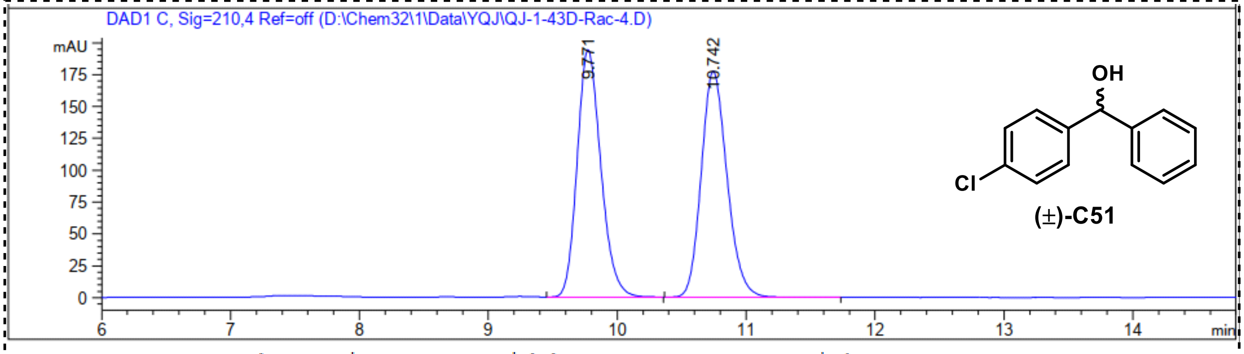

Peak #	RetTime [min]	Type	Width [min]	Area [mAU*s]	Height [mAU]	Area %
1	9.771	BB	0.1969	2474.33008	193.92586	50.1046
2	10.742	BB	0.2141	2464.00146	177.33247	49.8954

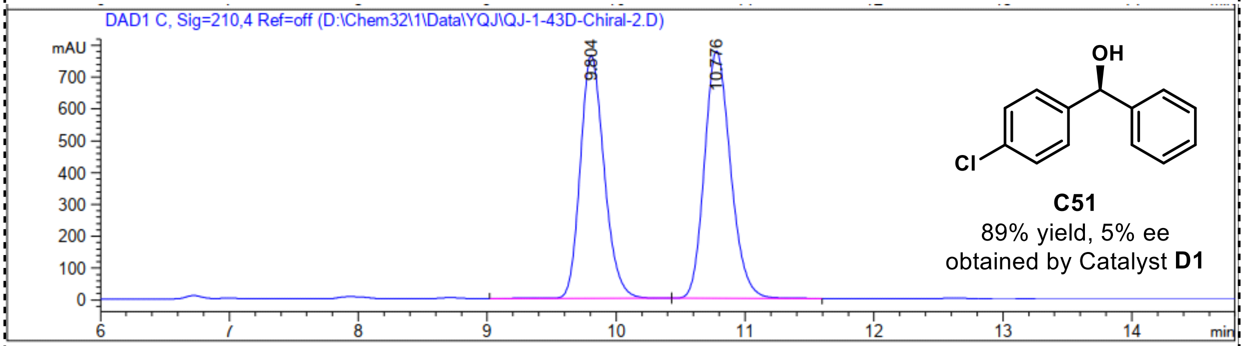

Peak #	RetTime [min]	Type	Width [min]	Area [mAU*s]	Height [mAU]	Area %
1	9.804	BB	0.2005	9845.76563	763.35913	47.4144
2	10.776	BB	0.2161	1.09196e4	776.42584	52.5856

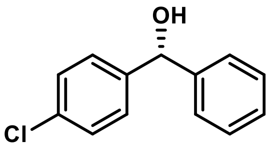

C51

Following the reported procedure with catalyst **D2**, **C51**(12.6 mg, 29% yield, (-)-3% ee) was obtained as white solid.

HPLC (OD-H, 0.46*25 cm, 5μm, hexane / isopropanol = 93/7, flow 1 mL/min, detection at 210 nm) retention time = 15.268 min (major) for enantiomer (*R*) and 17.023 min (minor) for enantiomer (*S*).

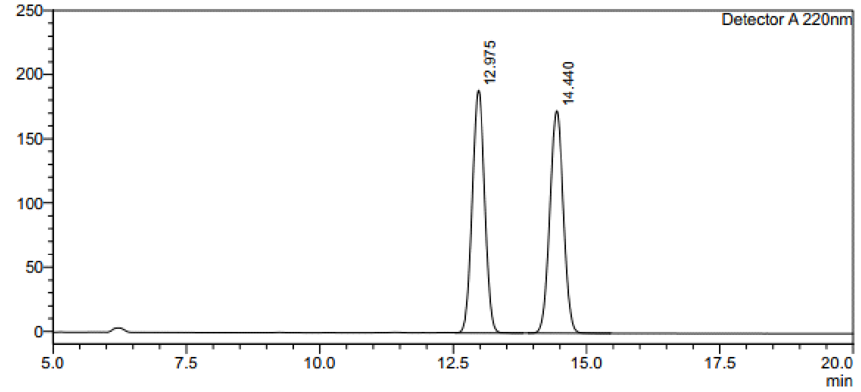

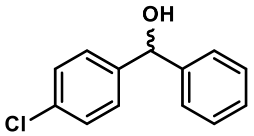

(±)-C51

<Peak Table>

Detector A 220nm

Peak#	Ret. Time	Area	Height	Conc.	Unit	Mark	Name
1	12.975	3086366	189052	50.115			
2	14.440	3072169	173169	49.885			
Total		6158535	362221				

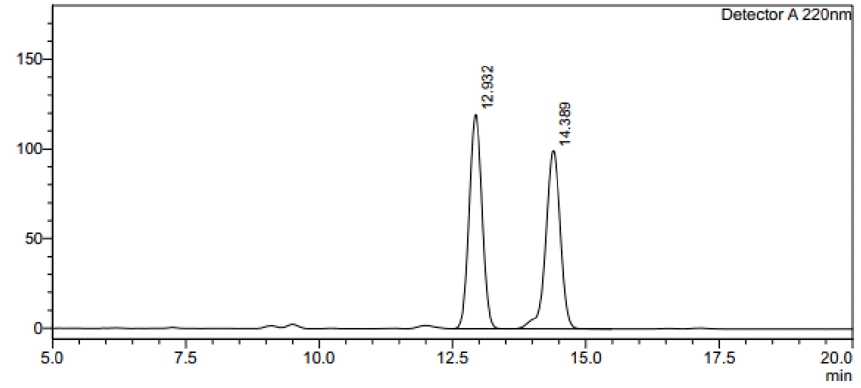

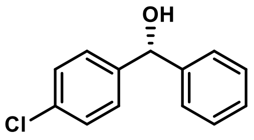

C51
29% yield, (-)-3% ee
obtained by Catalyst **D2**

<Peak Table>

Detector A 220nm

Peak#	Ret. Time	Area	Height	Conc.	Unit	Mark	Name
1	12.932	2022911	119569	51.591			
2	14.389	1898133	99374	48.409			
Total		3921044	218943				

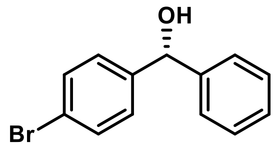

C52

Following the reported procedure with catalyst **D1**, **C52** (12.1 mg, 23% yield, (-)-8% ee) was obtained as colorless oil.

^{1}H NMR (400 MHz, $CDCl_3$): δ 7.43 (d, J = 8.4 Hz, 2H), 7.36 - 7.24 (m, 5H), 7.22 (d, J = 8.4 Hz, 2H), 5.73 (d, J = 3.4 Hz, 1H), 2.42 (d, J = 3.4 Hz, 1H) ppm

^{13}C NMR (101 MHz, $CDCl_3$): δ 143.3, 142.7, 131.5, 128.6, 128.2, 127.8, 126.5, 121.4, 75.6 ppm

HPLC (AD-H, 0.46*25 cm, 5μm, hexane / isopropanol = 95/5, flow 1 mL/min, detection at 210 nm) retention time = 17.352 min (major) for enantiomer (*R*) and 19.872 min (minor) for enantiomer (*S*).

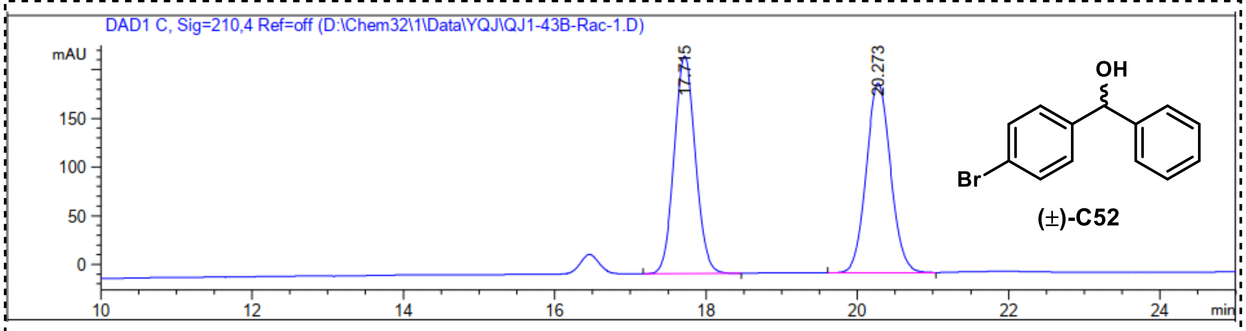

Peak #	RetTime [min]	Type	Width [min]	Area [mAU*s]	Height [mAU]	Area %
1	17.715	BB	0.3031	4351.65674	223.57890	50.0573
2	20.273	BB	0.3462	4341.70117	194.68953	49.9427

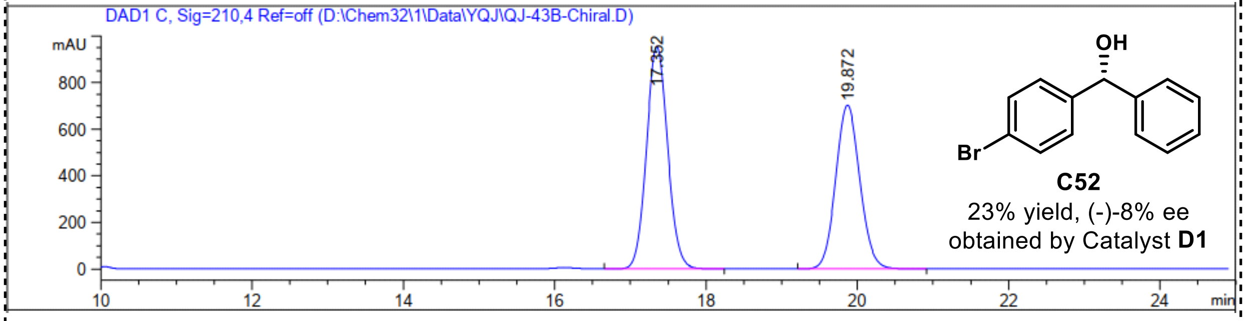

Peak #	RetTime [min]	Type	Width [min]	Area [mAU*s]	Height [mAU]	Area %
1	17.352	BB	0.3025	1.85119e4	953.70404	54.3669
2	19.872	BB	0.3443	1.55381e4	701.85730	45.6331

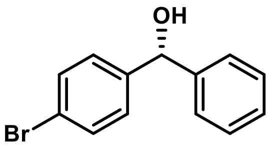

C52

Following the reported procedure with catalyst **D2**, **C52** (12.6 mg, 24% yield, (-)-6% ee) was obtained as colorless oil.

HPLC (AD-H, 0.46*25 cm, 5μm, hexane / isopropanol = 95/5, flow 1 mL/min, detection at 210 nm) retention time = 13.183 min (major) for enantiomer (*R*) and 14.656 min (minor) for enantiomer (*S*).

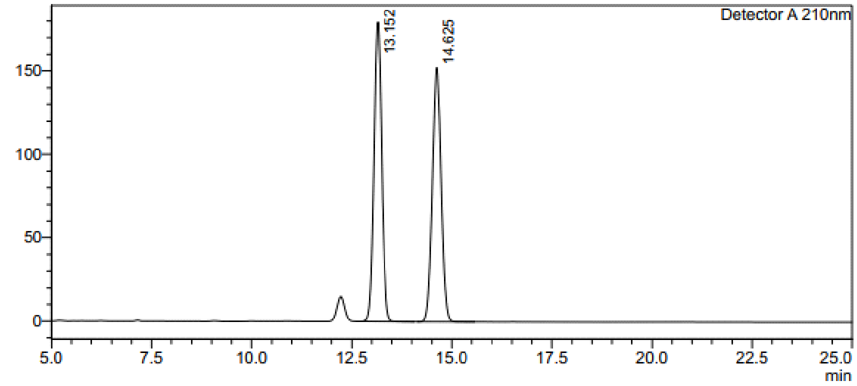

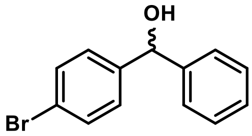

(±)-**C52**

<Peak Table>

Detector A 210nm

Peak#	Ret. Time	Area	Height	Conc.	Unit	Mark	Name
1	13.152	2408845	179603	50.042			
2	14.625	2404846	152369	49.958			
Total		4813691	331972				

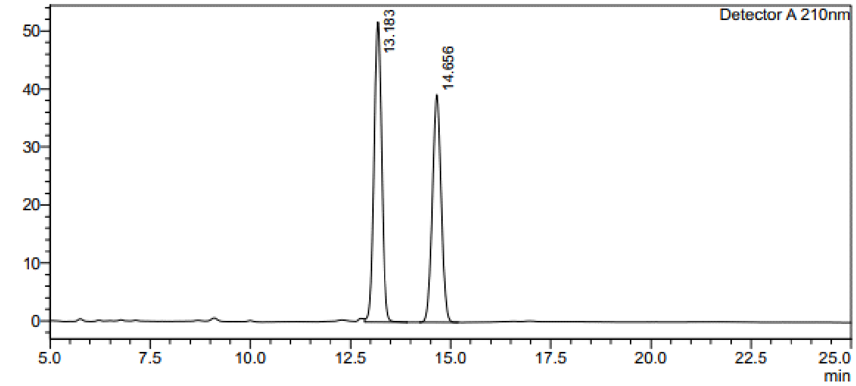

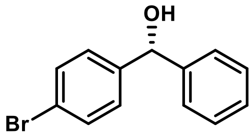

C52
24% yield, (-)-6% ee
obtained by Catalyst **D2**

<Peak Table>

Detector A 210nm

Peak#	Ret. Time	Area	Height	Conc.	Unit	Mark	Name
1	13.183	687169	51697	52.749			
2	14.656	615557	39183	47.251			
Total		1302726	90880				

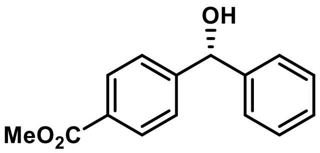

C53

Following the reported procedure with catalyst **D1**, **C53** (39.2 mg, 81% yield, (-)-26% ee) was obtained as white solid.

^{1}H NMR (400 MHz, CDCl$_3$): δ 8.00 (d, *J* = 8.4 Hz, 2H), 7.47 (d, *J* = 8.4 Hz, 2H), 7.40 – 7.26 (m, 5H), 5.86 (s, 1H), 3.90 (s, 3H), 2.84 (s, 1H) ppm

^{13}C NMR (101 MHz, CDCl$_3$): δ 166.9, 148.7, 143.2, 129.7, 129.1, 128.6, 127.8, 126.6, 126.3, 75.8, 52.0 ppm

HPLC (IA-H, 0.46*25 cm, 5μm, hexane / isopropanol = 80/20, flow 1 mL/min, detection at 254 nm) retention time = 7.86 min (major) for enantiomer (*R*) and 8.51 min (minor) for enantiomer (*S*).

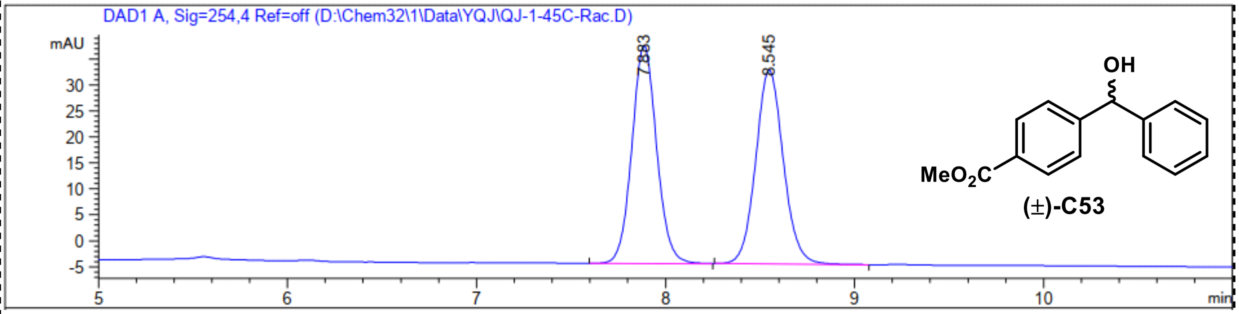

Peak #	RetTime [min]	Type	Width [min]	Area [mAU*s]	Height [mAU]	Area %
1	7.883	BB	0.1404	838.30621	91.88605	50.2404
2	8.545	BB	0.1564	830.28296	81.83249	49.7596

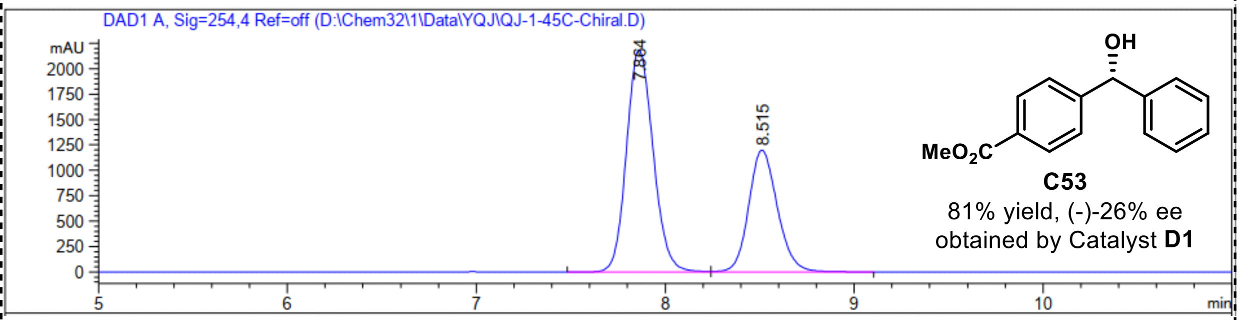

Peak #	RetTime [min]	Type	Width [min]	Area [mAU*s]	Height [mAU]	Area %
1	7.864	BV	0.1547	2.14763e4	2184.59058	62.8936
2	8.515	VB	0.1614	1.26708e4	1198.07544	37.1064

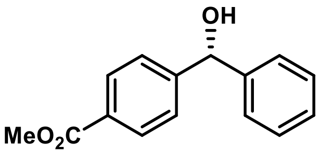

C53

Following the reported procedure with catalyst **D2**, **C53** (17.9 mg, 37% yield, (-)-10% ee) was obtained as white solid.

HPLC (IA-H, 0.46*25 cm, 5μm, hexane / isopropanol = 80/20, flow 1 mL/min, detection at 254 nm) retention time = 6.798 min (major) for enantiomer (*R*) and 7.328 min (minor) for enantiomer (*S*).

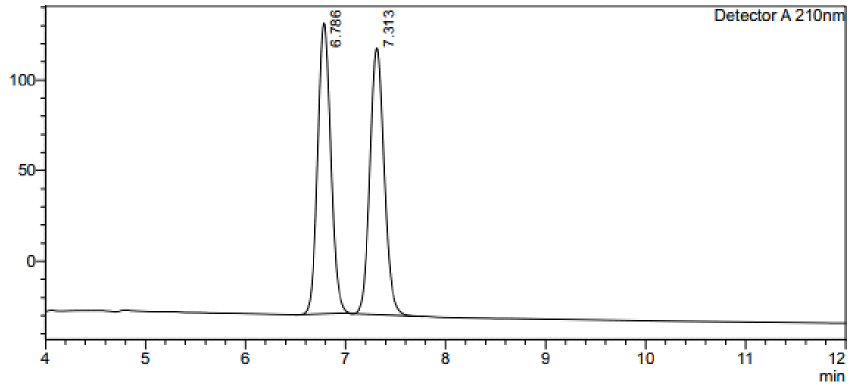

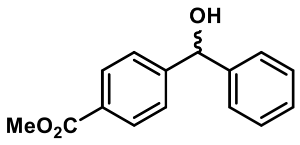

(±)-C53

<Peak Table>

Detector A 210nm

Peak#	Ret. Time	Area	Height	Conc.	Unit	Mark	Name
1	6.786	1454640	160464	50.044		M	
2	7.313	1452089	147021	49.956		M	
Total		2906729	307485				

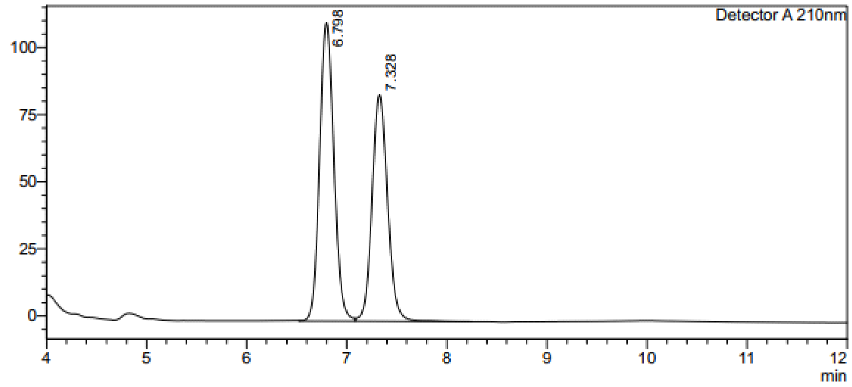

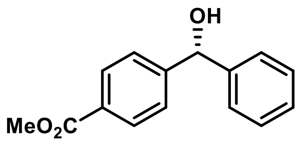

C53

37% yield, (-)-10% ee

obtained by Catalyst **D2**

<Peak Table>

Detector A 210nm

Peak#	Ret. Time	Area	Height	Conc.	Unit	Mark	Name
1	6.798	1094599	111247	54.740			
2	7.328	905024	84370	45.260		V	
Total		1999623	195617				

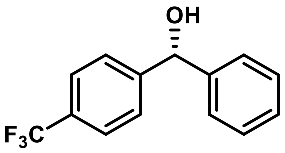

C54

Following the reported procedure with catalyst **D1**, **C54** (39.8 mg, 79% yield, (-)-35% ee) was obtained as white solid.

^{1}H NMR (400 MHz, $CDCl_3$): δ 7.60 (d, *J* = 8.2 Hz, 2H), 7.50 (d, *J* = 8.2 Hz, 2H), 7.42 – 7.27 (m, 5H), 5.86 (s, 1H), 2.53 (s, 1H) ppm

^{13}C NMR (101 MHz, $CDCl_3$): δ 147.5, 143.1, 129.6 (q, *J* = 32.3 Hz), 128.7, 128.0, 126.6 (q, *J* = 2.4 Hz), 125.4 (q, *J* = 3.7 Hz), 124.1 (q, *J* = 270.1 Hz), 75.7 ppm

^{19}F NMR (376 MHz, $CDCl_3$): δ -62.39 (s) ppm

HPLC (AD-H, 0.46*25 cm, 5μm, hexane / isopropanol = 85/15, flow 1 mL/min, detection at 220 nm) retention time = 11.949 min (major) for enantiomer (*R*) and 13.910 min (minor) for enantiomer (*S*).

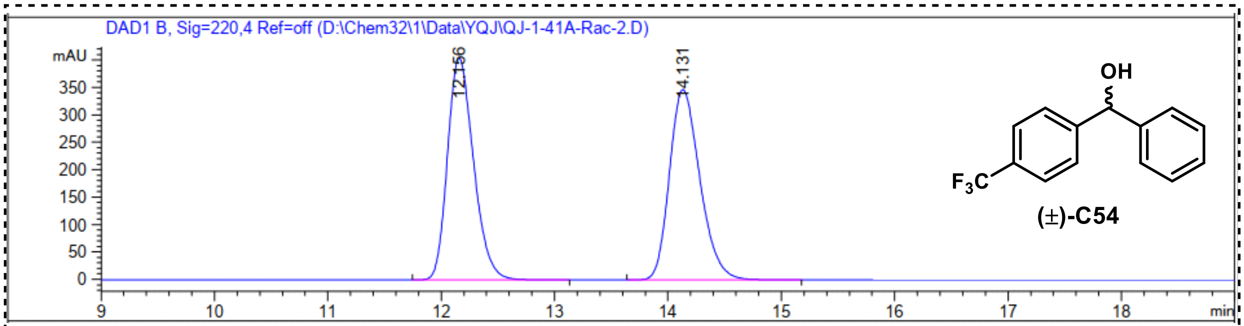

Peak #	RetTime [min]	Type	Width [min]	Area [mAU*s]	Height [mAU]	Area %
1	12.156	BB	0.2459	6458.11475	405.20505	49.9688
2	14.131	BB	0.2875	6466.17334	346.79123	50.0312

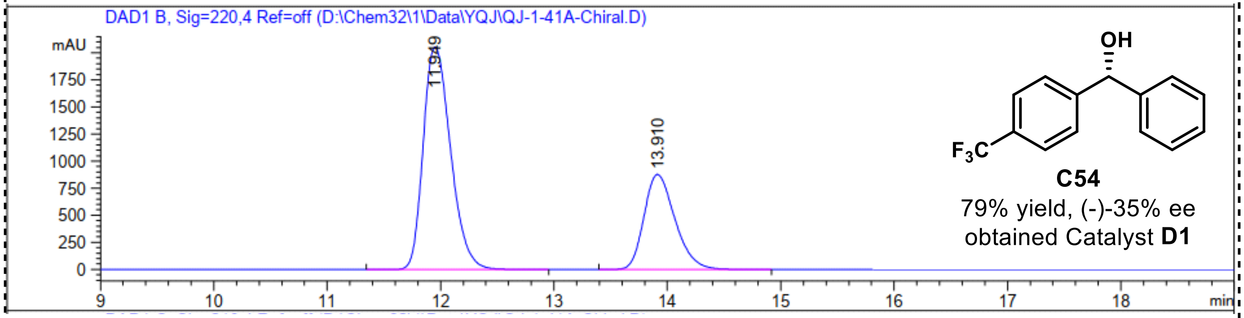

Peak #	RetTime [min]	Type	Width [min]	Area [mAU*s]	Height [mAU]	Area %
1	11.949	BB	0.2616	3.44579e4	2055.15552	67.3728
2	13.910	BB	0.2915	1.66872e4	878.78796	32.6272

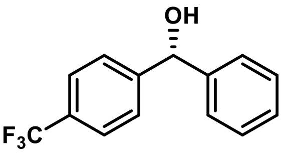

C54

Following the reported procedure with catalyst **D2**, **C54** (15.6 mg, 31% yield, (-)-13% ee) was obtained as white solid.

HPLC (AD-H, 0.46*25 cm, 5μm, hexane / isopropanol = 85/15, flow 1 mL/min, detection at 220 nm) retention time = 5.047 min (major) for enantiomer (*R*) and 5.760 min (minor) for enantiomer (*S*).

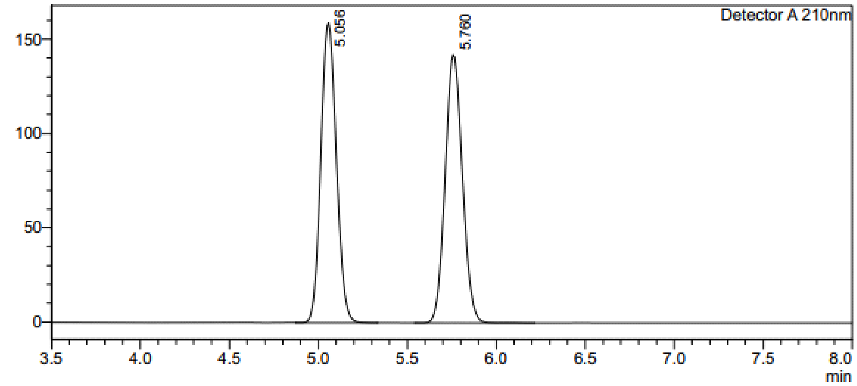

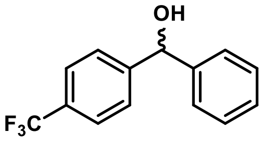

(±)-C54

<Peak Table>

Detector A 210nm

Peak#	Ret. Time	Area	Height	Conc.	Unit	Mark	Name
1	5.056	960078	159267	49.971		V	
2	5.760	961185	142426	50.029		S	
Total		1921263	301693				

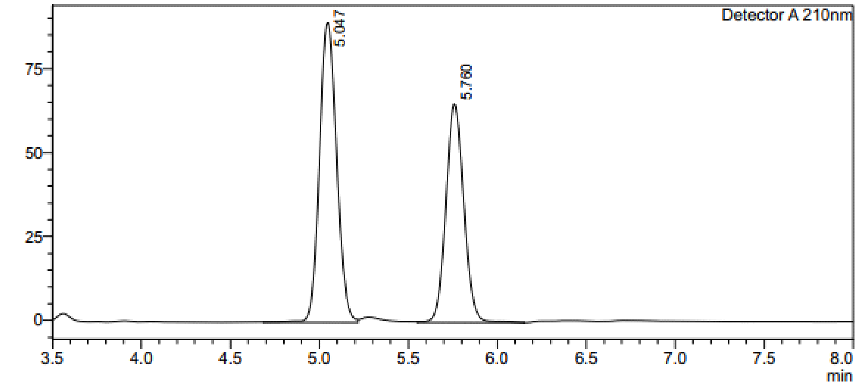

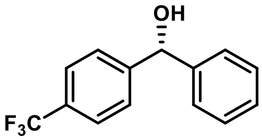

C54

31% yield, (-)-13% ee

obtained Catalyst **D2**

<Peak Table>

Detector A 210nm

Peak#	Ret. Time	Area	Height	Conc.	Unit	Mark	Name
1	5.047	599625	89377	56.652		V	
2	5.760	458814	65142	43.348			
Total		1058440	154520				

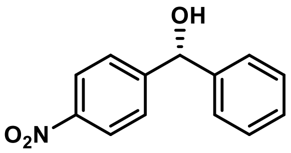

C55

Following the reported procedure with catalyst **D1**, **C55** (43.5 mg, 95% yield, (-)-41% ee) was obtained as white solid

^{1}H NMR (400 MHz, $CDCl_3$): δ 8.27 – 8.07 (m, 2H), 7.55 (d, *J* = 8.6 Hz, 2H), 7.44 – 7.27 (m, 5H), 5.88 (s, 1H), 2.75 (s, 1H) ppm

^{13}C NMR (101 MHz, $CDCl_3$): δ 150.7, 147.0, 142.6, 128.8, 128.3, 127.0, 126.6, 123.6, 75.4 ppm

HPLC (AD-H, 0.46*25 cm, 5μm, hexane / isopropanol = 90/10, flow 1 mL/min, detection at 210 nm) retention time = 14.858 min (major) for enantiomer (*R*) and 19.194 min (minor) for enantiomer (*S*).

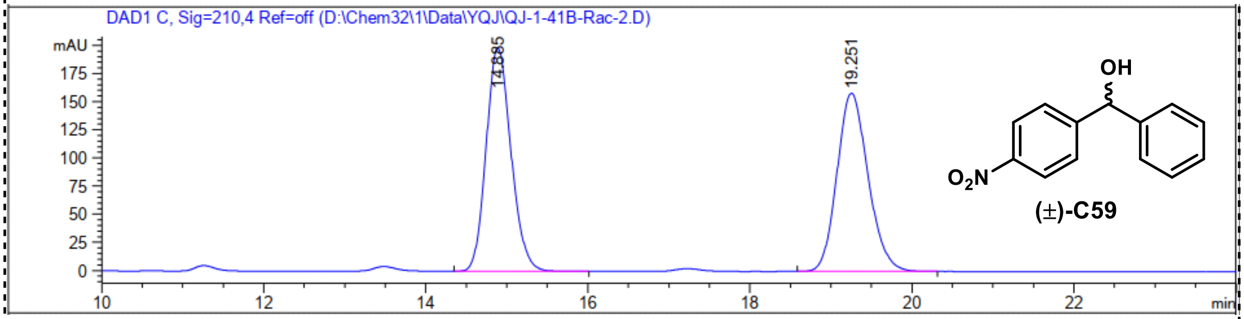

Peak #	RetTime [min]	Type	Width [min]	Area [mAU*s]	Height [mAU]	Area %
1	14.885	BB	0.3309	4241.87939	198.83987	50.0949
2	19.251	BB	0.4116	4225.79932	158.22090	49.9051

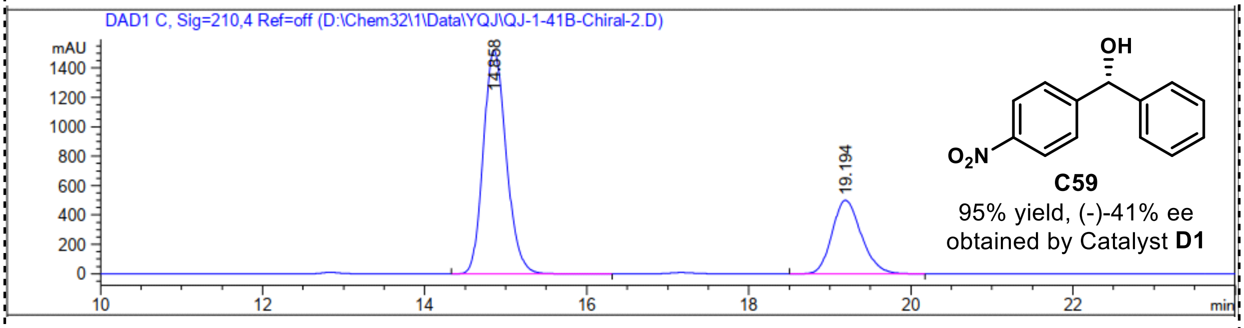

Peak #	RetTime [min]	Type	Width [min]	Area [mAU*s]	Height [mAU]	Area %
1	14.858	BB	0.3051	2.98843e4	1522.00452	70.4200
2	19.194	BB	0.3866	1.25529e4	500.58481	29.5800

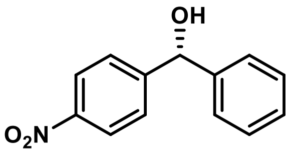

C55

Following the reported procedure with catalyst **D2**, **C55** (17.0 mg, 37% yield, (-)-19% ee) was obtained as white solid

HPLC (AD-H, 0.46*25 cm, 5μm, hexane / isopropanol = 90/10, flow 1 mL/min, detection at 210 nm) retention time = 12.680 min (major) for enantiomer (*R*) and 15.859 min (minor) for enantiomer (*S*).

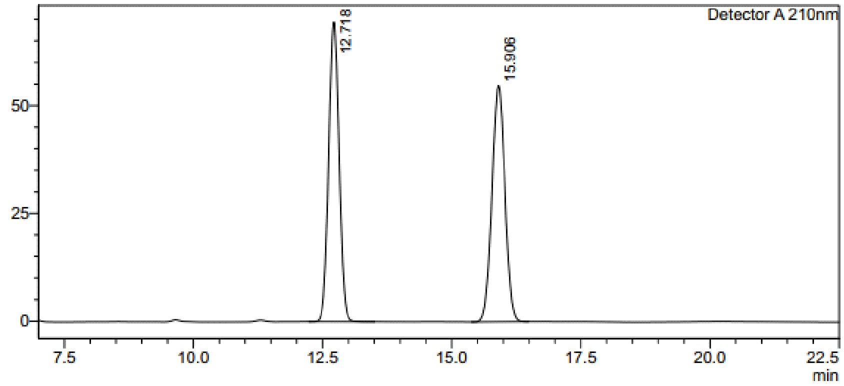

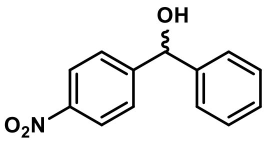

(±)-C55

<Peak Table>

Detector A 210nm

Peak#	Ret. Time	Area	Height	Conc.	Unit	Mark	Name
1	12.718	966609	69493	50.082			
2	15.906	963458	54811	49.918			
Total		1930066	124304				

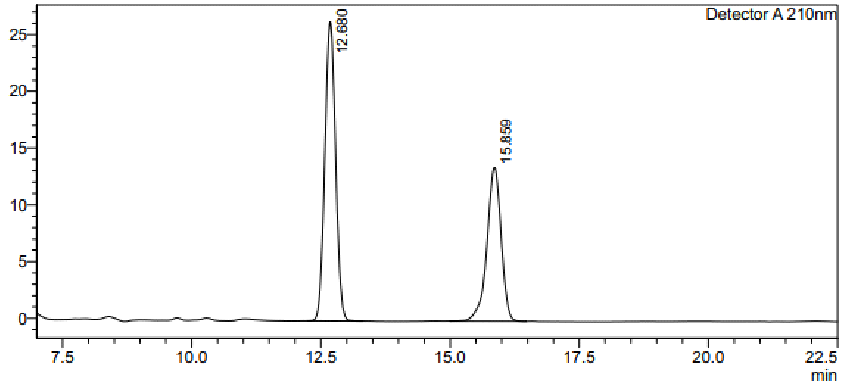

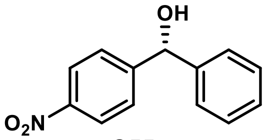

C55
37% yield, (-)-19% ee
obtained by Catalyst **D2**

<Peak Table>

Detector A 210nm

Peak#	Ret. Time	Area	Height	Conc.	Unit	Mark	Name
1	12.680	377631	26361	59.412			
2	15.859	257980	13544	40.588			
Total		635611	39905				

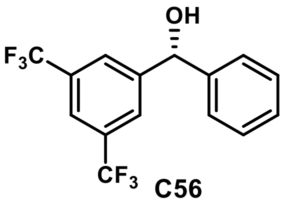

C56

Following the reported procedure with catalyst **D1**, **C56** (57.6 mg, 90% yield, (-)-56% ee) was obtained as white solid.

HPLC (AD-H, 0.46*25 cm, 5μm, hexane / isopropanol = 98/2, flow 1 mL/min, detection at 210 nm) retention time = 9.820 min (minor) for enantiomer (*S*) and 10.484 min (major) for enantiomer (*R*).

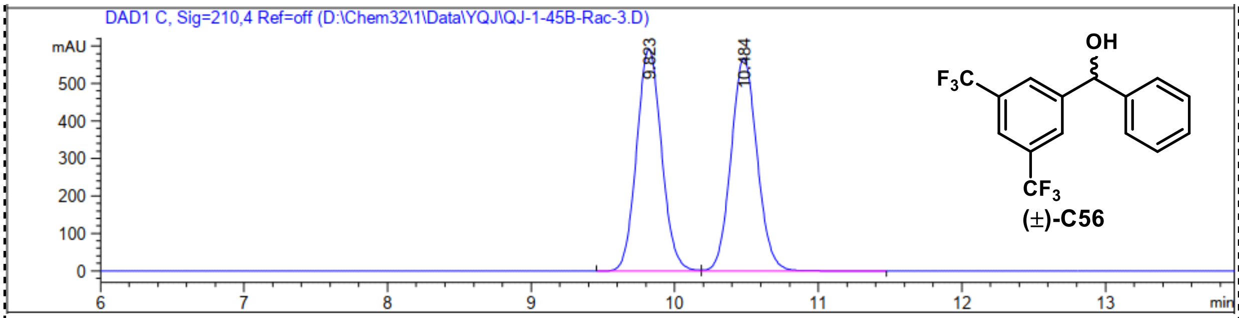

Peak #	RetTime [min]	Type	Width [min]	Area [mAU*s]	Height [mAU]	Area %
1	9.823	BV	0.1864	7148.17871	593.88605	49.8985
2	10.484	VB	0.1973	7177.25146	568.45221	50.1015

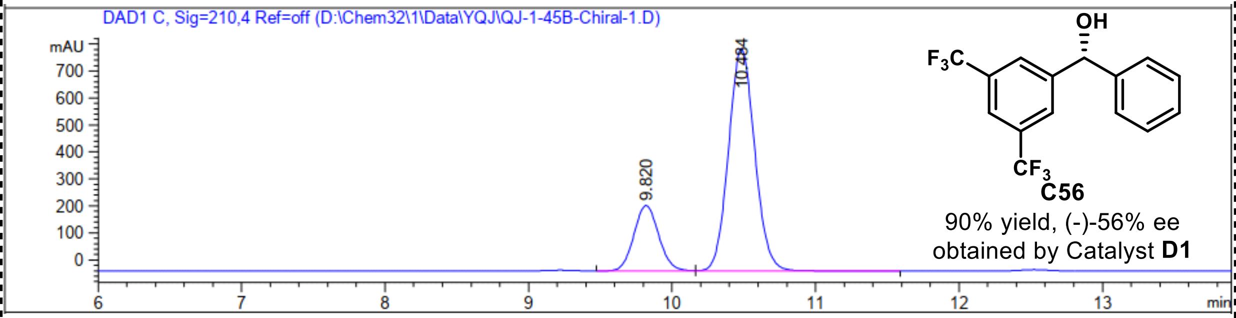

Peak #	RetTime [min]	Type	Width [min]	Area [mAU*s]	Height [mAU]	Area %
1	9.820	BV	0.1884	2918.02222	242.46301	21.8266
2	10.484	VB	0.1979	1.04511e4	824.31580	78.1734

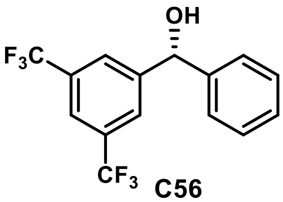

C56

Following the reported procedure with catalyst **D2**, **C56** (23.0 mg, 36% yield, (-)-22% ee) was obtained as white solid.

HPLC (AD-H, 0.46*25 cm, 5μm, hexane / isopropanol = 98/2, flow 1 mL/min, detection at 210 nm) retention time = 36.037 min (minor) for enantiomer (*S*) and 40.224 min (major) for enantiomer (*R*).

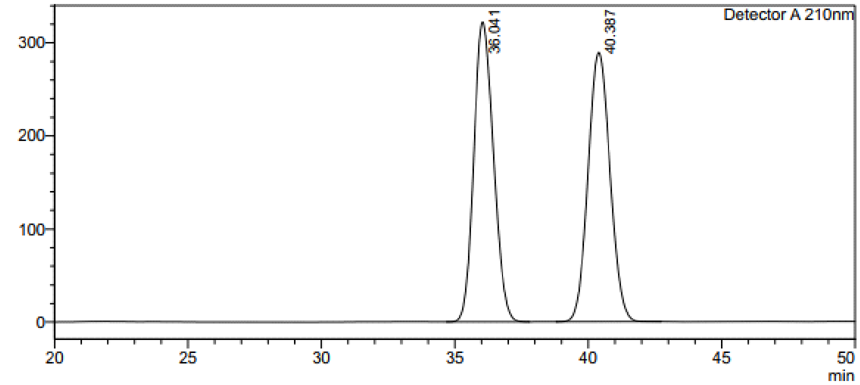

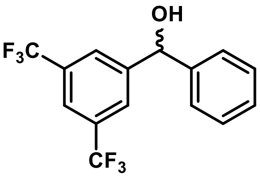

(±)-C56

<Peak Table>

Detector A 210nm

Peak#	Ret. Time	Area	Height	Conc.	Unit	Mark	Name
1	36.041	16578685	322149	49.964			
2	40.387	16602318	289307	50.036			
Total		33181004	611456				

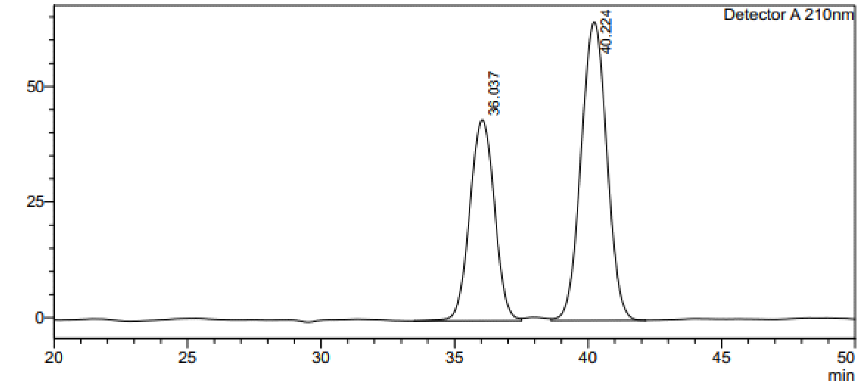

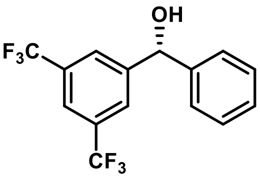

C56

36% yield, (-)-22% ee

obtained by Catalyst **D2**

<Peak Table>

Detector A 210nm

Peak#	Ret. Time	Area	Height	Conc.	Unit	Mark	Name
1	36.037	2791662	43377	39.132			
2	40.224	4342335	64505	60.868			
Total		7133997	107881				

2.4 System 4: Sharpless asymmetric dihydroxylation (SAD) of alkenes with a cinchona alkaloidderived ligand

2.4.1 Synthesis and characterization of the ligands

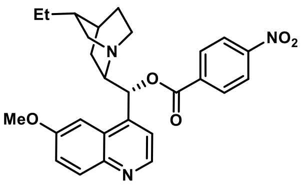

E

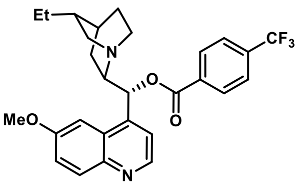

F

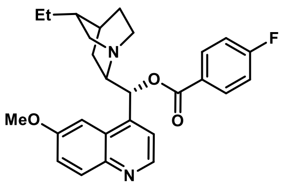

G

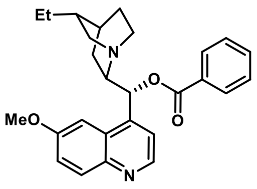

H

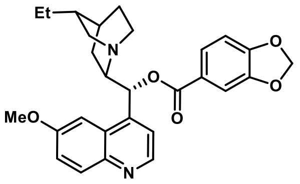

I

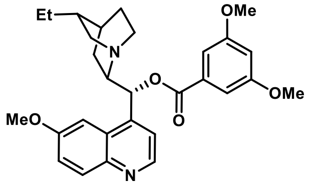

J

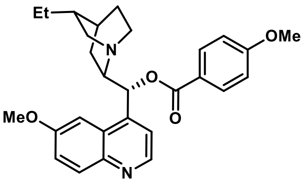

K

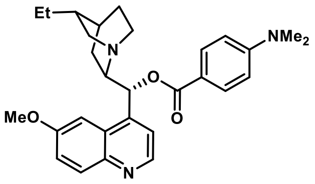

L

All the cinchona alkaloid ligands used in this section were synthesized according to the literature, in which **F**, **H**, **I** and **J** have been reported. Ligands **E**, **G**, **K** and **L** were unknown and synthesized.

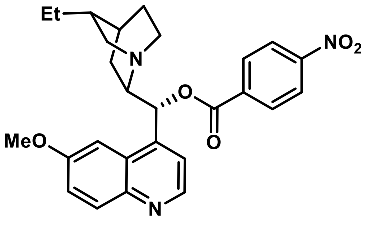

E

According to the literature, ligand **E** was obtained as solid (1.88 g, 79% yield)

^{1}H NMR (600 MHz, $CDCl_3$): δ 8.73 (d, *J* = 4.4 Hz, 1H), 8.32 (d, *J* = 8.2 Hz, 2H), 8.25 (d, *J* = 8.2 Hz, 2H), 8.03 (d, *J* = 9.2 Hz, 1H), 7.52 (s, 1H), 7.44 – 7.36 (m, 2H), 6.79 (s, 1H), 4.00 (s, 3H), 3.51 (dd, *J* = 15.6, 7.4 Hz, 1H), 3.27 – 3.01 (m 2H), 2.71 (s, 1H), 2.40 (d, *J* = 9.4 Hz, 1H), 1.93 – 1.86 (m, 2H), 1.78 (s, 1H), 1.72 – 1.62 (m, 1H), 1.59 – 1.42 (m, 2H), 1.43 – 1.30 (m, 2H), 0.87 (t, *J* = 7.4 Hz, 3H) ppm

^{13}C NMR (101 MHz, $CDCl_3$): δ 163.7, 158.2, 150.8, 147.4, 144.8, 142.9, 135.0, 132.0, 130.7, 126.9, 123.8, 122.0, 118.5, 101.3, 75.4, 59.2, 58.3, 55.8, 42.7 37.3, 28.5, 27.7, 25.2, 24.1, 12.1 ppm

HRMS (ESI+): calculated for $C_{27}H_{30}N_3O_5$ $[M+H]^-$: 476.2180, found 476.2181.

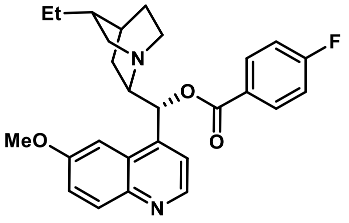

G

According to the literature, ligand **G** was obtained as solid (1.70 g, 76% yield)

^{1}H NMR (600 MHz, $CDCl_3$): δ 8.71 (d, *J* = 4.5 Hz, 1H), 8.17 – 8.08 (m, 2H), 8.01 (d, *J* = 9.2 Hz, 1H), 7.53 (s, 1H), 7.44 – 7.36 (m, 2H), 7.18 – 7.12 (m, 2H), 6.80 (s, 1H), 4.00 (s, 3H), 3.47 (dd, *J* = 15.5, 7.4 Hz, 1H), 3.22 (s, 1H), 3.09 (dd, *J* = 12.8, 10.0 Hz, 1H), 2.71 (t, *J* = 10.0 Hz, 1H), 2.41 (d, *J* = 12.8 Hz, 1H), 1.90 – 1.69 (m, 4H), 1.58 – 1.44 (m, 2H), 1.40 – 1.28 (m, 2H), 0.85 (t, *J* = 7.4 Hz, 3H) ppm

^{13}C NMR (151 MHz, $CDCl_3$): δ 166.0 (d, *J* = 255.1 Hz), 164.5, 158.1, 147.4, 144.8, 143.4, 132.2 (d, *J* = 9.1 Hz), 131.8, 126.8, 126.0 (d, *J* = 2.4 Hz), 122.0, 118.4, 115.8 (d, *J* = 22.3 Hz), 101.3, 74.4, 59.2, 58.3, 55.8, 42.7, 37.2, 28.3, 27.7, 25.2, 23.6, 12.0 ppm

^{19}F NMR (376 MHz, $CDCl_3$): δ -104.45 (s) ppm

HRMS (ESI+): calculated for $C_{27}H_{30}N_2O_3F$ $[M+H]^-$: 449.2235, found 449.2234.

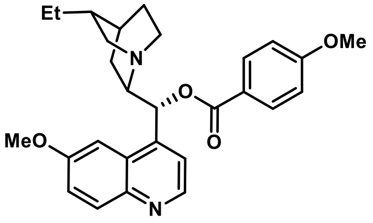

K

According to the literature, ligand **K** was obtained as solid (1.91 g, 83% yield)

^{1}H NMR (400 MHz, $CDCl_3$): δ 8.66 (d, *J* = 4.5 Hz, 1H), 8.04 (d, *J* = 8.9 Hz, 2H), 7.99 (d, *J* = 9.2 Hz, 1H), 7.69 (s, 1H), 7.40 – 7.32 (m, 2H), 7.16 (s, 1H), 6.95 (d, *J* = 8.9 Hz, 2H), 4.07 (s, 3H), 3.85 (s, 3H), 3.58 – 3.38 (m, 2H), 3.33 – 3.20 (m, 1H), 3.00 – 2.81 (m, 1H), 2.58 (d, *J* = 12.5 Hz, 1H), 2.11 – 1.92 (m, 3H), 1.86 – 1.59 (m, 3H), 1.35 – 1.28 (m, 2H), 0.83 (t, *J* = 7.3 Hz, 3H) ppm

^{13}C NMR (101 MHz, $CDCl_3$): δ 164.2, 163.9, 158.65 (s), 147.0, 144.7, 142.4, 131.6, 131.6, 126.3, 122.7, 121.6, 117.7, 114.0, 101.2, 72.0, 58.8, 57.6, 56.7, 55.5, 45.7, 42.8, 36.2, 27.3, 25.0, 11.7, 8.6 ppm

HRMS (ESI+): calculated for $C_{28}H_{33}N_2O_4$ $[M+H]^-$: 461.2435, found 461.2432.

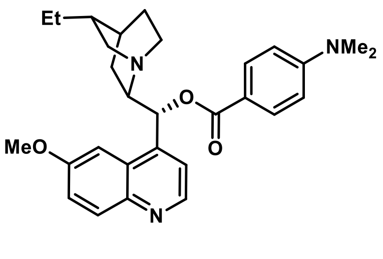

L

According to the literature, ligand **L** was obtained as solid (1.68 g, 71% yield)

^{1}H NMR (600 MHz, $CDCl_3$): δ 8.66 (d, J = 4.5 Hz, 1H), 7.99 (d, J = 9.2 Hz, 1H), 7.96 (d, J = 8.8 Hz, 2H), 7.68 (s, 1H), 7.41 – 7.34 (m, 2H), 7.08 (s, 1H), 6.67 (d, J = 8.8 Hz, 2H), 4.07 (s, 3H), 3.51 – 3.41 (m, 2H), 3.31 – 3.22 (m, 1H), 3.05 (s, 6H), 2.94 – 2.81 (m, 1H), 2.63 – 2.51 (m, 1H), 2.12 – 1.91 (m, 3H), 1.81 – 1.74 (m, 1H), 1.73 – 1.56 (m, 2H), 1.37 – 1.26 (m, 2H), 0.84 (t, J = 7.3 Hz, 3H) ppm

^{13}C NMR (151 MHz, $CDCl_3$): δ 164.9, 158.5, 153.7, 147.1, 144.7, 131.6, 131.3, 128.4, 126.5, 122.5, 117.9, 115.8, 110.9, 101.3, 71.8, 58.9, 57.8, 56.6, 42.9, 40.0, 36.5, 27.4, 27.1, 25.1, 21.9, 11.8 ppm

HRMS (ESI+): calculated for $C_{29}H_{36}N_3O_3$ $[M+H]^-$: 474.2751, found 474.2752.

2.4.2 Preparation of alkenes

Supplementary Table 50. Alkenes for LFER studies

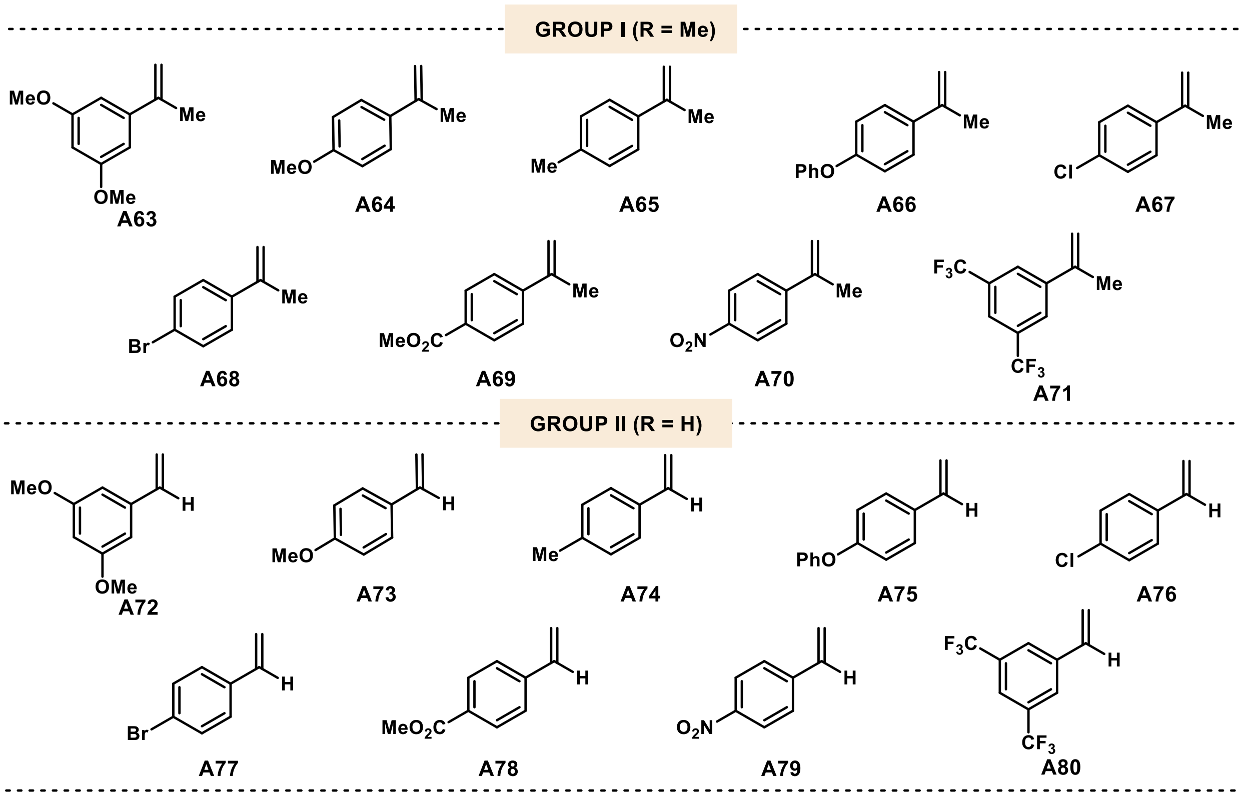

Except alkenes **A63**, **A66**, **A69**, and **A71** that were unknown and synthesized according to a known literature procedure[6], other alkenes were commercially available.

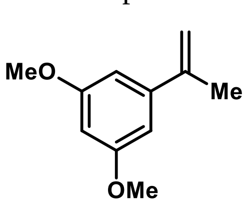

A63

^{1}H NMR (400 MHz, CDCl$_3$): δ 6.62 (s, 2H), 6.40 (s, 1H), 5.36 (s, 1H), 5.12 – 5.06 (m, 1H), 3.81 (s, 3H), 3.81 (s, 3H), 2.13 (s, 3H).
^{13}C NMR (101 MHz, CDCl$_3$): δ 160.6, 143.5, 143.3, 112.8, 104.0, 99.3, 55.3, 21.9 ppm
HRMS (ESI+): calculated for $C_{11}H_{15}O_2$ [M+H]$^-$: 179.1067, found 179.1067.

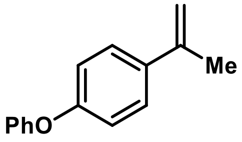

A66

^{1}H NMR (400 MHz, CDCl$_3$): δ 7.45 (d, *J* = 8.4 Hz, 2H), 7.35 (t, *J* = 7.5 Hz, 2H), 7.12 (t, *J* = 7.5 Hz, 1H), 7.03 (d, *J* = 8.4 Hz, 2H), 6.98 (d, *J* = 8.4 Hz, 2H), 5.34 (d, *J* = 1.3 Hz, 1H), 5.06 (d, *J* = 1.3 Hz, 1H), 2.16 (s, 3H) ppm
^{13}C NMR (101 MHz, CDCl$_3$): δ 157.2, 156.6, 142.4, 136.3, 129.7, 126.8, 123.2, 118.9, 118.5, 111.7, 21.9 ppm
HRMS (ESI+): calculated for $C_{15}H_{15}O$ [M+H]$^-$: 211.1117, found 211.1117.

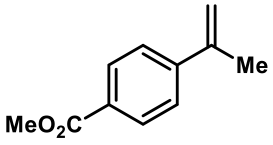

A69

^{1}H NMR (400 MHz, CDCl$_3$): δ 7.99 (d, *J* = 8.5 Hz, 2H), 7.52 (d, *J* = 8.5 Hz, 2H), 5.47 (s, 1H), 5.19 (s, 1H), 3.92 (s, 3H), 2.17 (s, 3H) ppm
^{13}C NMR (101 MHz, CDCl$_3$): δ 166.9, 145.6, 142.4, 129.6, 128.9, 125.4, 114.5, 52.0, 21.6 ppm
HRMS (ESI+): calculated for $C_{11}H_{13}O_2$ [M+H]$^-$: 177.0910, found 177.0911.

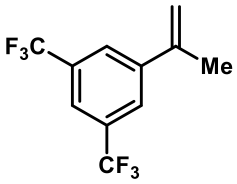

A71

^{1}H NMR (400 MHz, CDCl$_3$): δ 7.87 (s, 2H), 7.77 (s, 1H), 5.50 (s, 1H), 5.29 (s, 1H), 2.20 (s, 3H) ppm

^{13}C NMR (101 MHz, CDCl$_3$): δ 143.3, 140.9, 131.6 (q, *J* = 33.2 Hz), 125.5, 123.4 (q, *J* = 272.7 Hz), 121.09 – 120.90 (m), 115.8, 21.5 ppm
^{19}F NMR (377 MHz, CDCl$_3$): δ -62.93 ppm
HRMS (ESI+): calculated for $C_{11}H_9F_6$ [M+H]$^-$: 255.0603, found 255.0603.

2.4.3 Identification of the optimal ligand for LFER studies

For each ligand, we chose three alkene substrates with substituents of different electronic properties on the aryl rings (**A73**, R = OMe; **A76**, R = Cl, **A79**, R = NO_2). The asymmetric dihydroxylations were conducted under standard conditions[8], and the corresponding ees were recorded as below (**Supplementary Table 51**). We found that ligand **E** can provide largest ee differences between the three substrates, thus would be potentially the optimal ligand to investigate LFER between local carbon polarizability and observed ees.

Supplementary Table 51. Identification of the optimal ligand or LFER studies

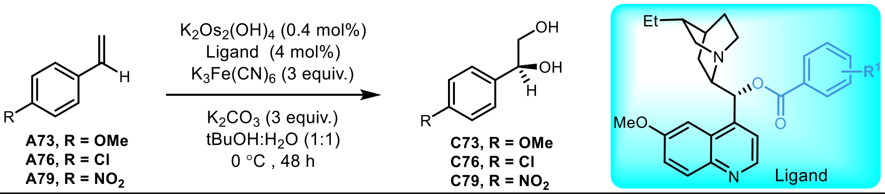

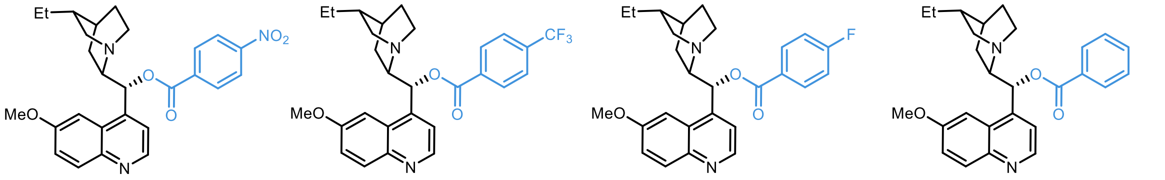

	E	F	G	H
**C73, R = OMe:**	99% conv, **81.4**% ee	99% conv, **84.0**% ee	99% conv, **83.4**% ee	99% conv, **84.1**% ee
**C76, R = Cl:**	99% conv, **72.1**% ee	99% conv, **78.0**% ee	99% conv, **77.6**% ee	99% conv, **79.4**% ee
**C79, R = NO_2:**	99% conv, **62.2**% ee	99% conv, **69.1**% ee	99% conv, **69.8**% ee	99% conv, **70.4**% ee
**Δ ee :**	**9.3%; 9.9%; 19.2%**	**6%; 8.9%;14.9%**	**5.8%; 7.8%; 13.6%**	**4.7%; 9%; 13.7%**

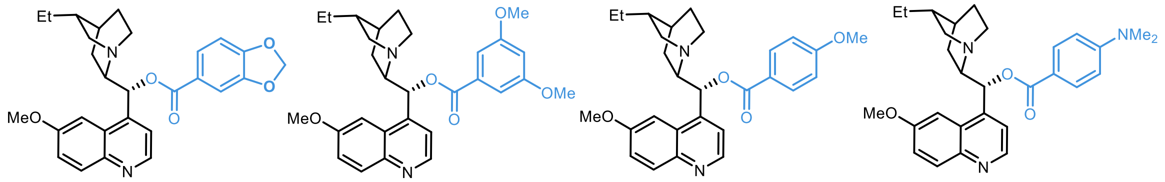

	I	J	K	L
**C73, R = OMe:**	99% conv, **84.2**% ee	99% conv, **85.2**% ee	99% conv, **84.5**% ee	99% conv, **85.4**% ee
**C76, R = Cl:**	99% conv, **78.3**% ee	99% conv, **79.3**% ee	99% conv, **79.2**% ee	99% conv, **80.2**% ee
**C79, R = NO_2:**	99% conv, **69.4**% ee	99% conv, **72.5**% ee	99% conv, **67.6**% ee	99% conv, **76.0**% ee
**Δ ee :**	**5.9%; 8.9%; 14.8%**	**5.9%; 6.8%; 12.7%**	**5.3%; 11.6%; 16.8%**	**5.2%; 4.2%; 9.4%**

.

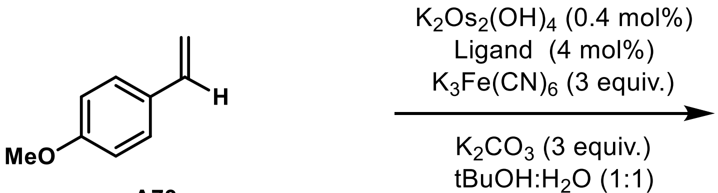

A73 **C73**

The HPLCs of alcohol product **C73** with ligands **E** to **L**:

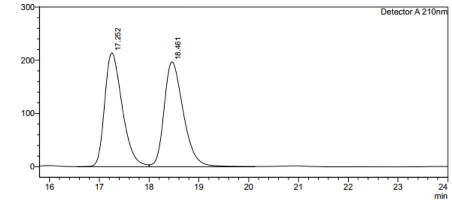

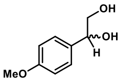

(±)-**C73**

<Peak Table>
Detector A 210nm

Peak#	Ret. Time	Area	Height	Conc.	Unit	Mark	Name
1	17.252	5257605	213847	49.785			
2	18.461	5302935	197044	50.215		V	
Total		10560540	410891				

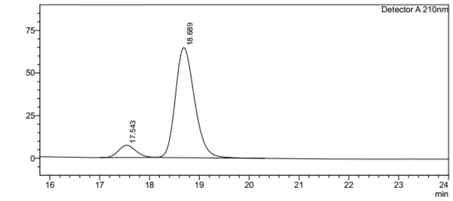

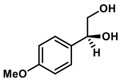

C73
With Ligand **E**

<Peak Table>
Detector A 210nm

Peak#	Ret. Time	Area	Height	Conc.	Unit	Mark	Name
1	17.543	177133	7186	9.317		M	
2	18.689	1724132	64550	90.683		M	
Total		1901265	71736				

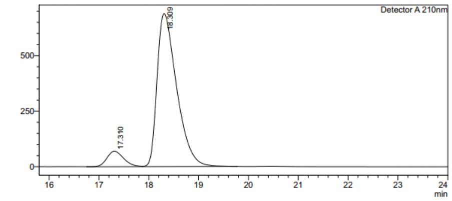

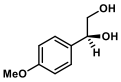

C73
With Ligand **F**

<Peak Table>
Detector A 210nm

Peak#	Ret. Time	Area	Height	Conc.	Unit	Mark	Name
1	17.310	1628911	69788	7.979			
2	18.309	18785813	688857	92.021		V M	
Total		20414724	758645				

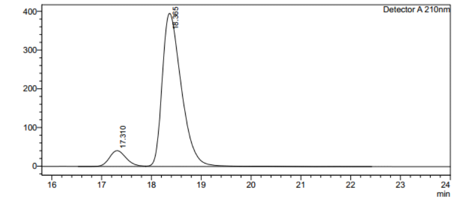

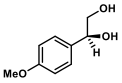

C73
With Ligand **G**

<Peak Table>
Detector A 210nm

Peak#	Ret. Time	Area	Height	Conc.	Unit	Mark	Name
1	17.310	966738	41088	8.374			
2	18.365	10578065	395759	91.626		V	
Total		11544803	436847				

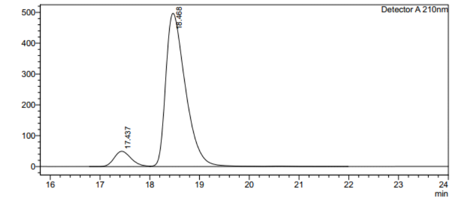

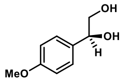

C73
With Ligand **H**

<Peak Table>
Detector A 210nm

Peak#	Ret. Time	Area	Height	Conc.	Unit	Mark	Name
1	17.437	1180625	49605	7.946			
2	18.468	13677249	497073	92.054		V	
Total		14857874	546678				

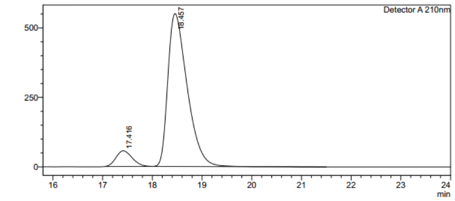

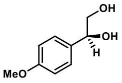

C73
With Ligand **I**

<Peak Table>
Detector A 210nm

Peak#	Ret. Time	Area	Height	Conc.	Unit	Mark	Name
1	17.416	1279292	56598	7.906		M	
2	18.457	14902030	550207	92.094		M	
Total		16181322	606805				

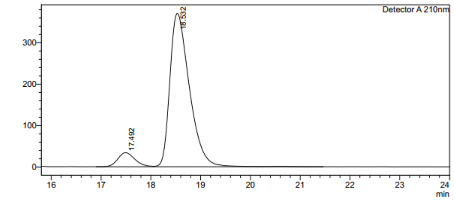

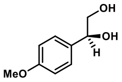

C73
With Ligand **J**

<Peak Table>
Detector A 210nm

Peak#	Ret. Time	Area	Height	Conc.	Unit	Mark	Name
1	17.492	808882	33942	7.417			
2	18.532	10097021	370240	92.583		V	
Total		10905904	404182				

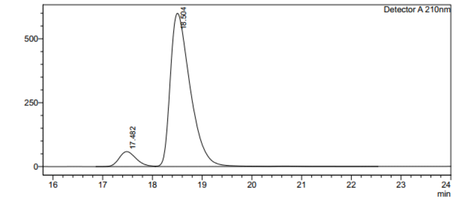

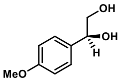

C73
With Ligand **K**

<Peak Table>
Detector A 210nm

Peak#	Ret. Time	Area	Height	Conc.	Unit	Mark	Name
1	17.482	1404710	58852	7.737			
2	18.504	16750516	599706	92.263		V	
Total		18155226	658558				

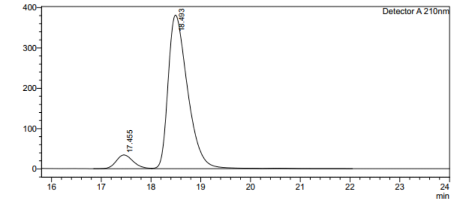

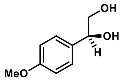

C73
With Ligand **L**

<Peak Table>
Detector A 210nm

Peak#	Ret. Time	Area	Height	Conc.	Unit	Mark	Name
1	17.455	817417	34278	7.290			
2	18.493	10395588	380723	92.710		SV	
Total		11213005	415001				

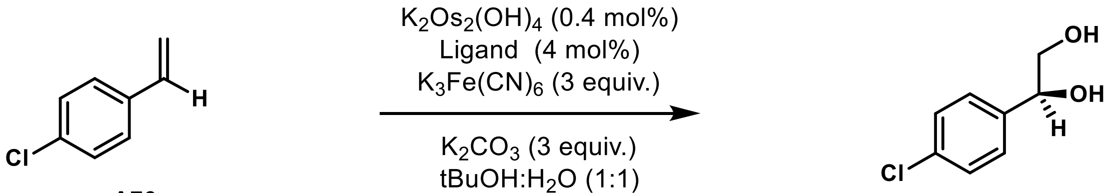

A76 **C76**

The HPLCs of alcohol product **C76** with ligands **E** to **L**:

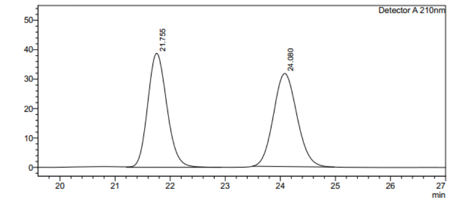

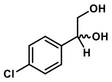

(±)-**C76**

<Peak Table>

Detector A 210nm

Peak#	Ret. Time	Area	Height	Conc.	Unit	Mark	Name
1	21.755	939848	38744	49.977			
2	24.080	940720	31640	50.023		M	
Total		1880568	70384				

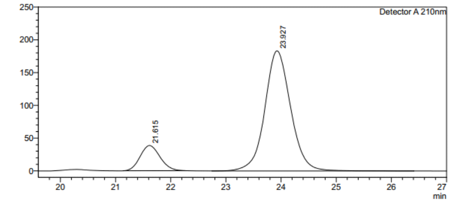

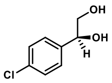

C76
With Ligand **E**

<Peak Table>

Detector A 210nm

Peak#	Ret. Time	Area	Height	Conc.	Unit	Mark	Name
1	21.615	965339	38465	13.926		M	
2	23.927	5966813	183380	86.074			
Total		6932152	221845				

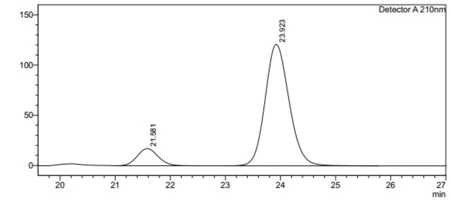

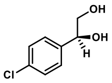

C76
With Ligand **F**

<Peak Table>

Detector A 210nm

Peak#	Ret. Time	Area	Height	Conc.	Unit	Mark	Name
1	21.581	452102	17105	11.014			
2	23.923	3652523	120947	88.986			
Total		4104626	138052				

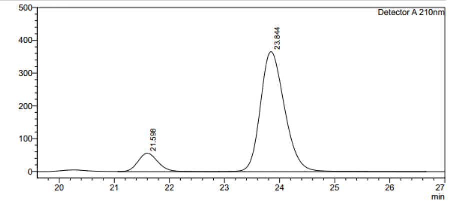

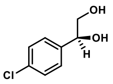

C76
With Ligand **G**

<Peak Table>

Detector A 210nm

Peak#	Ret. Time	Area	Height	Conc.	Unit	Mark	Name
1	21.598	1404127	56127	11.215			
2	23.844	11116303	366086	88.785		V	
Total		12520430	422213				

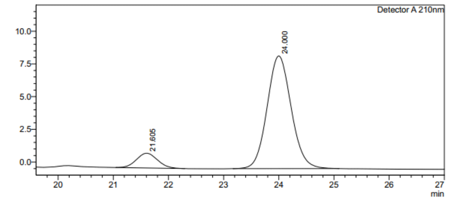

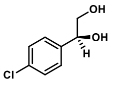

C76
With Ligand **H**

<Peak Table>

Detector A 210nm

Peak#	Ret. Time	Area	Height	Conc.	Unit	Mark	Name
1	21.605	29998	1127	10.291			
2	24.000	261515	8608	89.709			
Total		291513	9735				

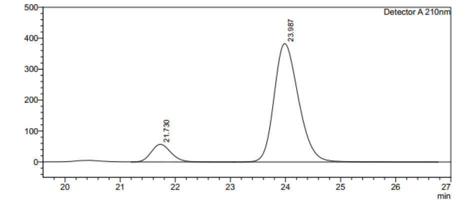

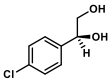

C76
With Ligand **I**

<Peak Table>

Detector A 210nm

Peak#	Ret. Time	Area	Height	Conc.	Unit	Mark	Name
1	21.730	1434494	57193	10.839			
2	23.987	11800036	382732	89.161		V	
Total		13234530	439924				

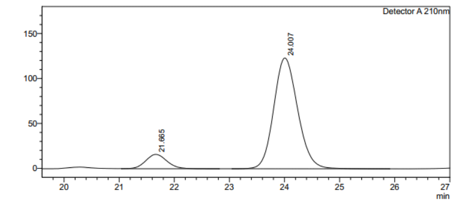

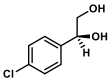

C76
With Ligand **J**

<Peak Table>

Detector A 210nm

Peak#	Ret. Time	Area	Height	Conc.	Unit	Mark	Name
1	21.665	429518	15981	10.338			
2	24.007	3725135	123214	89.662			
Total		4154653	139196				

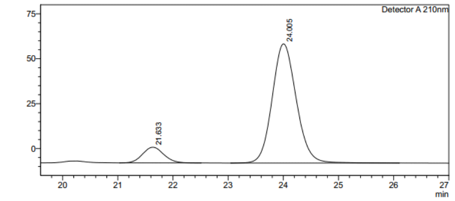

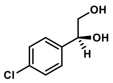

C76
With Ligand **K**

<Peak Table>

Detector A 210nm

Peak#	Ret. Time	Area	Height	Conc.	Unit	Mark	Name
1	21.633	233640	8767	10.381			
2	24.005	2017058	66358	89.619			
Total		2250698	75125				

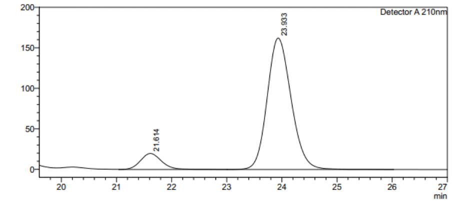

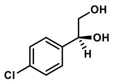

C76
With Ligand **L**

<Peak Table>

Detector A 210nm

Peak#	Ret. Time	Area	Height	Conc.	Unit	Mark	Name
1	21.614	538127	19808	9.888			
2	23.933	4903853	162298	90.112		V	
Total		5441980	182105				

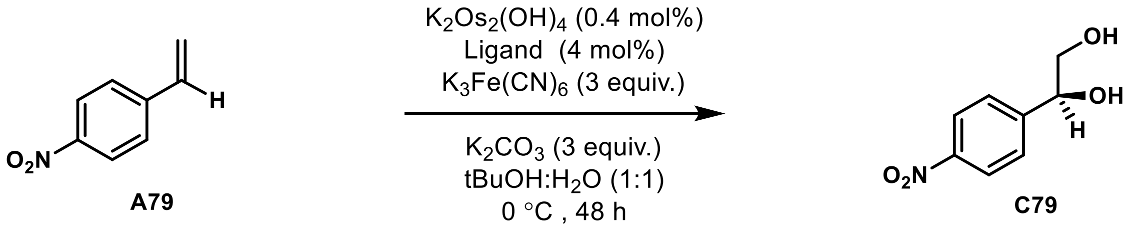

A79 **C79**

The HPLCs of alcohol product **C79** with ligands **E** to **L**:

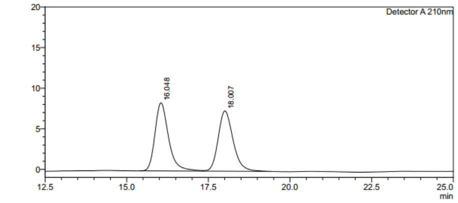

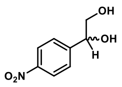

(±)-**C79**

<Peak Table>

Detector A 210nm

Peak#	Ret. Time	Area	Height	Conc.	Unit	Mark	Name
1	16.048	244045	8402	50.792			
2	18.007	236434	7433	49.208		V	
Total		480479	15835				

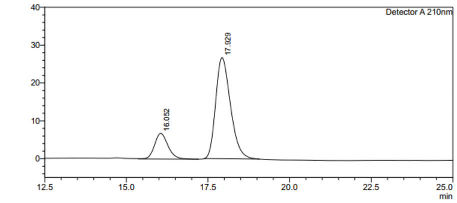

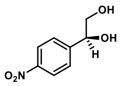

C79
With Ligand **E**

<Peak Table>

Detector A 210nm

Peak#	Ret. Time	Area	Height	Conc.	Unit	Mark	Name
1	16.052	196511	6824	18.892		M	
2	17.929	843686	26717	81.108		M	
Total		1040197	33542				

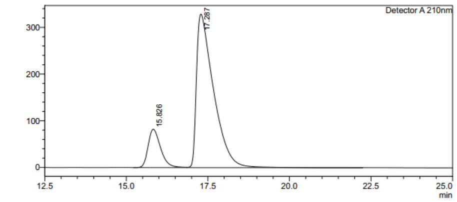

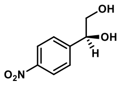

C79
With Ligand **F**

<Peak Table>

Detector A 210nm

Peak#	Ret. Time	Area	Height	Conc.	Unit	Mark	Name
1	15.826	2187094	82237	15.445			
2	17.287	11973743	328763	84.555		V	
Total		14160837	411000				

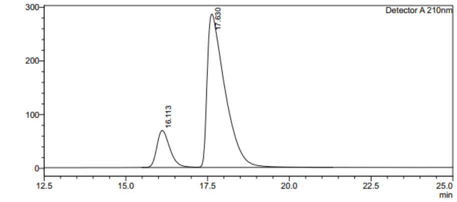

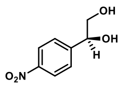

C79
With Ligand **G**

<Peak Table>

Detector A 210nm

Peak#	Ret. Time	Area	Height	Conc.	Unit	Mark	Name
1	16.113	1930191	69239	15.121			
2	17.630	10834519	285942	84.879		V	
Total		12764710	355182				

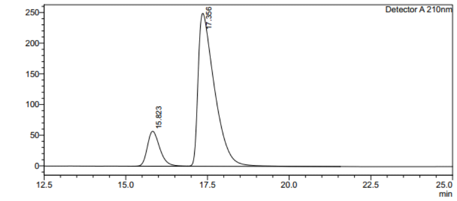

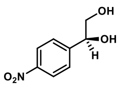

C79
With Ligand **H**

<Peak Table>

Detector A 210nm

Peak#	Ret. Time	Area	Height	Conc.	Unit	Mark	Name
1	15.823	1515860	57031	14.778			
2	17.356	8741675	249448	85.222		V	
Total		10257535	306479				

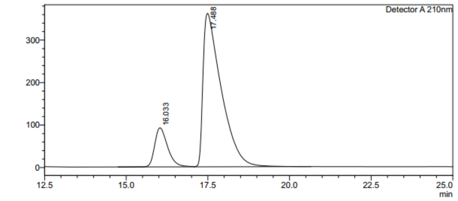

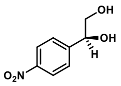

C79
With Ligand **I**

<Peak Table>

Detector A 210nm

Peak#	Ret. Time	Area	Height	Conc.	Unit	Mark	Name
1	16.033	2532155	91925	15.285			
2	17.488	14034029	361108	84.715		V	
Total		16566184	453033				

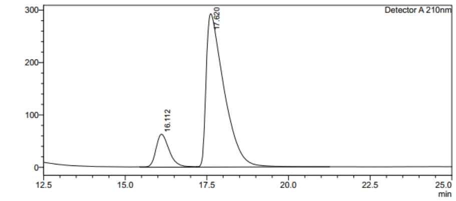

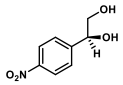

C79
With Ligand **J**

<Peak Table>

Detector A 210nm

Peak#	Ret. Time	Area	Height	Conc.	Unit	Mark	Name
1	16.112	1784967	62937	13.734			
2	17.620	11211947	292556	86.266		V	
Total		12996913	355494				

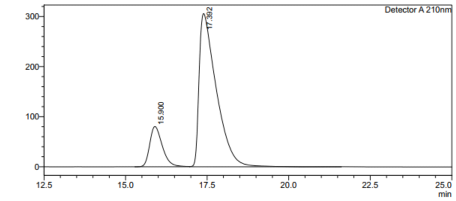

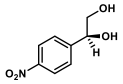

C79
With Ligand **K**

<Peak Table>

Detector A 210nm

Peak#	Ret. Time	Area	Height	Conc.	Unit	Mark	Name
1	15.900	2163666	80510	16.176			
2	17.392	11211768	305950	83.824		V	
Total		13375434	386461				

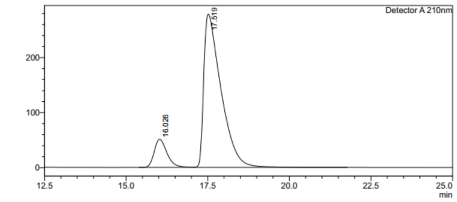

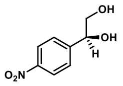

C79
With Ligand **L**

<Peak Table>

Detector A 210nm

Peak#	Ret. Time	Area	Height	Conc.	Unit	Mark	Name
1	16.026	1392622	51176	11.990			
2	17.519	10222301	278366	88.010		V	
Total		11614923	329542				

2.3.4 Investigation of LFER between local carbon polarizability and observed ees with ligand E

Supplementary Table 52. LFER for Sharpless asymmetric dihydroxylation of substituted terminal alkenes

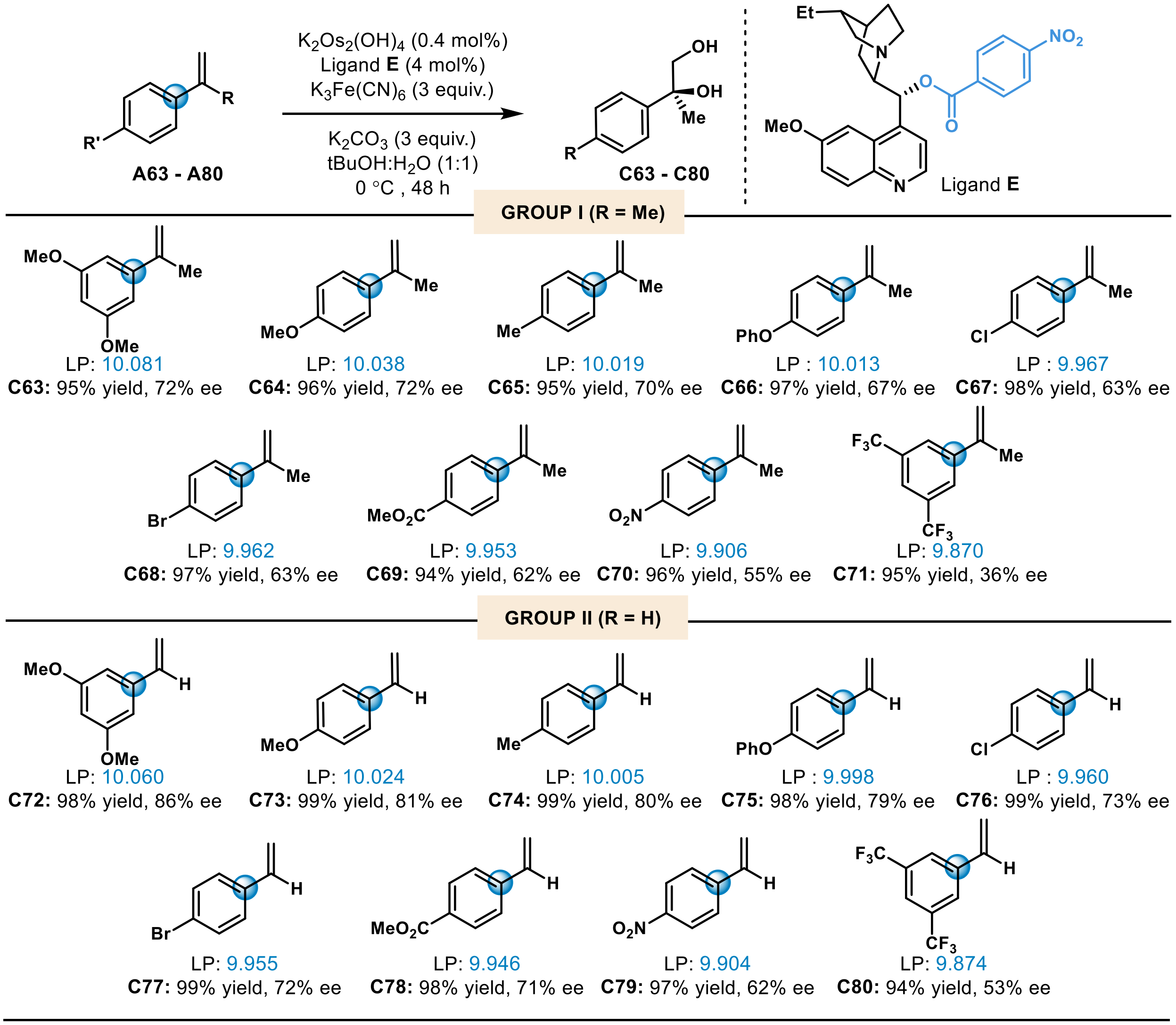

LP: blue carbon's local polarizability

The Sharpless asymmetric dihydroxylation were conducted under standard conditions[8] and the results were showed in **Supplementary Table 52**. The carbonyl substrates' local polarizabilities were calculated by using Tkatchenko-Scheffler model[5]. Based on these experimental and computational data, the quantitative linear free energy correlations between the substrate local polarizabilities and the observed magnitudes of reaction enantio-selection ($\Delta\Delta G^{\ddagger}$ values) in SAD system with substituted terminal alkenes were conducted as below (**Supplementary Figure 52**).

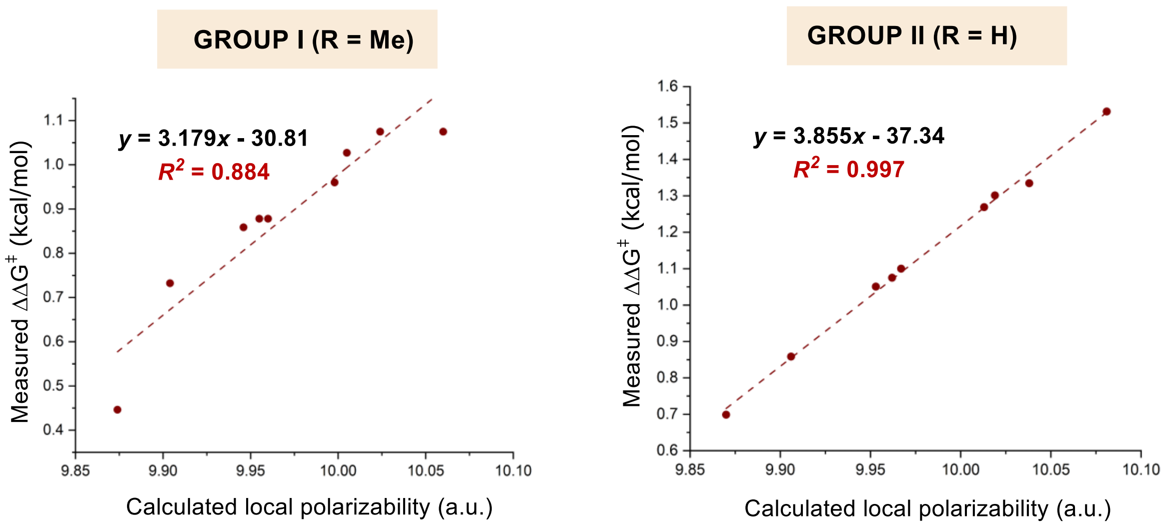

Supplementary Figure 52. LFER for Sharpless asymmetric dihydroxylation of substituted terminal alkenes

Characterization of the dihydroxylation products

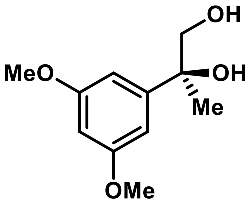

C63

C63 (20.2 mg, 95% yield, 72% ee) was obtained as white solid.

^{1}H NMR (400 MHz, $CDCl_3$): δ 6.59 (d, J = 2.2 Hz, 2H), 6.36 (t, J = 2.2 Hz, 1H), 3.78 (s, 6H), 3.76 (d, J = 11.2 Hz, 1H), 3.59 (d, J = 11.2 Hz, 1H), 2.78 (s, 1H), 2.03 (s, 1H), 1.48 (s, 3H) ppm

^{13}C NMR (101 MHz, $CDCl_3$): δ 160.8, 147.7, 103.5, 98.7, 74.9, 70.9, 55.3, 26.0 ppm

HRMS (ESI+): calculated for $C_{11}H_{16}O_4Na$ $[M+Na]^+$: 235.0941, found 235.0940.

HPLC (OD-H, 0.46*25 cm, 5μm, hexane / ethanol = 95/5, flow 1 mL/min, detection at 210 nm) retention time = 15.756 min (minor) and 18.806 min (major).

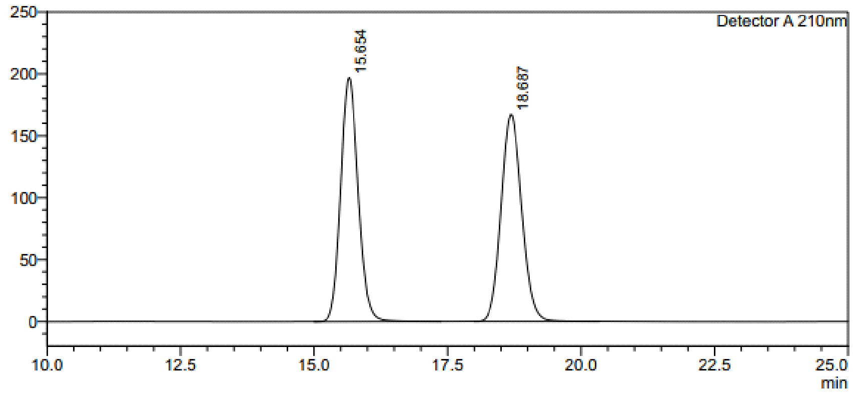

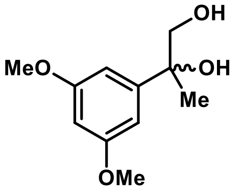

(±)-**C63**

<Peak Table>

Detector A 210nm

Peak#	Ret. Time	Area	Height	Conc.	Unit	Mark	Name
1	15.654	4496859	196981	49.943			
2	18.687	4507065	166962	50.057			
Total		9003924	363942				

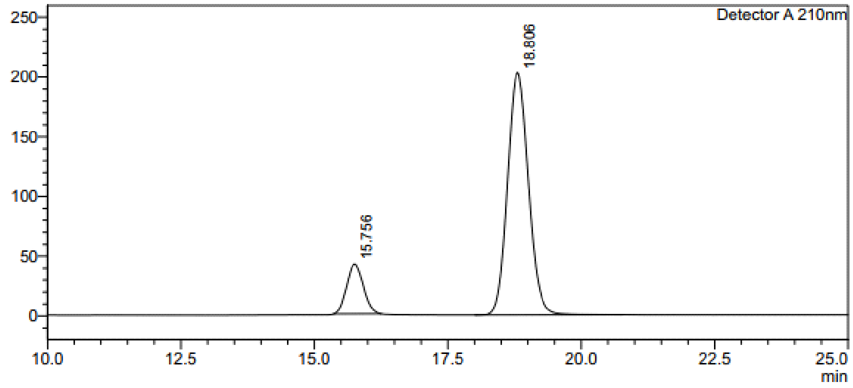

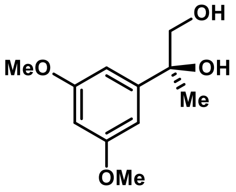

C63

95% yield, 72% ee

obtained by ligand **E**

<Peak Table>

Detector A 210nm

Peak#	Ret. Time	Area	Height	Conc.	Unit	Mark	Name
1	15.756	908525	41736	14.085		M	
2	18.806	5541852	202837	85.915			
Total		6450377	244573				

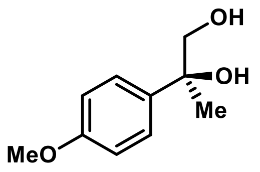

C64

C64 (17.5 mg, 96% yield, 72% ee) was obtained as white solid.

^{1}H NMR (400 MHz, $CDCl_3$): δ 7.36 (d, *J* = 8.7 Hz, 2H), 6.89 (d, *J* = 8.7 Hz, 2H), 3.80 (s, 3H), 3.74 (d, *J* = 11.0 Hz, 1H), 3.59 (d, *J* = 11.0 Hz, 1H), 2.64 (s, 1H), 2.03 (s, 1H), 1.51 (s, 3H) ppm
^{13}C NMR (101 MHz, $CDCl_3$): δ 158.7, 137.0, 126.3, 113.7, 74.5, 71.1, 55.2, 26.0 ppm
HRMS (ESI+): calculated for $C_{10}H_{13}O_2$ $[M+H-H_2O]^+$: 165.0910, found 165.0910.
HPLC (AD-H, 0.46*25 cm, 5μm, hexane / ethanol = 95/5, flow 1 mL/min, detection at 210 nm) retention time = 15.723 min (major) and 21.004 min (minor).

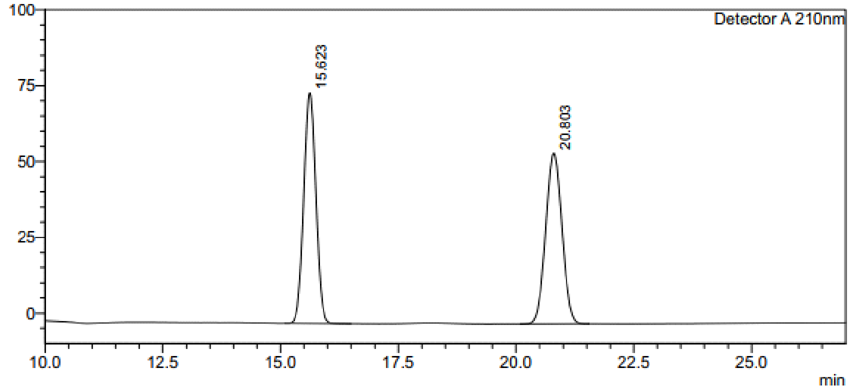

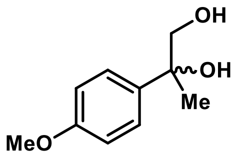

(±)-**C64**

<Peak Table>

Detector A 210nm

Peak#	Ret. Time	Area	Height	Conc.	Unit	Mark	Name
1	15.623	1350881	76023	50.081			
2	20.803	1346534	56170	49.919			
Total		2697415	132192				

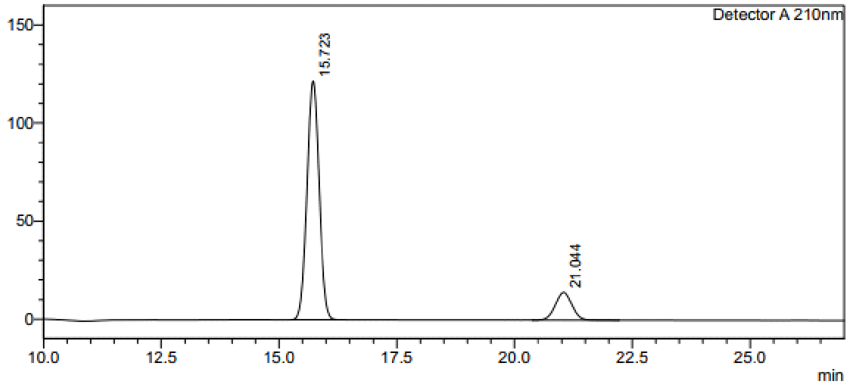

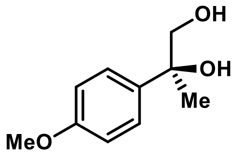

C64
96% yield, 72% ee
obtained by ligand **E**

<Peak Table>

Detector A 210nm

Peak#	Ret. Time	Area	Height	Conc.	Unit	Mark	Name
1	15.723	2168858	121707	86.205		M	
2	21.044	347061	14154	13.795		S	
Total		2515919	135861				

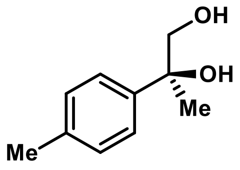

C65

C65 (15.7 mg, 95% yield, 70% ee) was obtained as white solid.

^{1}H NMR (400 MHz, CDCl$_3$): δ 7.33 (d, J = 8.1 Hz, 2H), 7.17 (d, J = 8.1 Hz, 2H), 3.74 (d, J = 11.0 Hz, 1H), 3.58 (d, J = 11.0 Hz, 1H), 2.83 (s, 1H), 2.34 (s, 3H), 2.29 (s, 1H), 1.50 (s, 3H) ppm
^{13}C NMR (101 MHz, CDCl$_3$): δ 142.0, 136.8, 129.1, 125.0, 74.7, 71.0, 26.0, 20.9 ppm
HRMS (ESI+): calculated for $C_{10}H_{14}O_2Na$ [M+Na]$^+$: 189.0886, found 189.0886
HPLC (AD-H, 0.46*25 cm, 5μm, hexane / isopropanol = 95/5, flow 1 mL/min, detection at 210 nm) retention time = 10.704 min (major) and 14.127 min (minor).

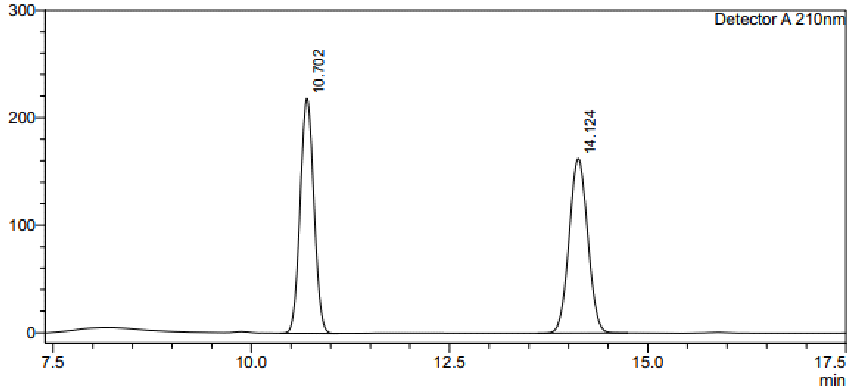

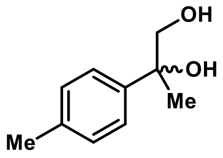

(±)-**C65**

<Peak Table>

Detector A 210nm

Peak#	Ret. Time	Area	Height	Conc.	Unit	Mark	Name
1	10.702	2623940	218327	49.869		M	
2	14.124	2637725	162233	50.131		M	
Total		5261666	380560				

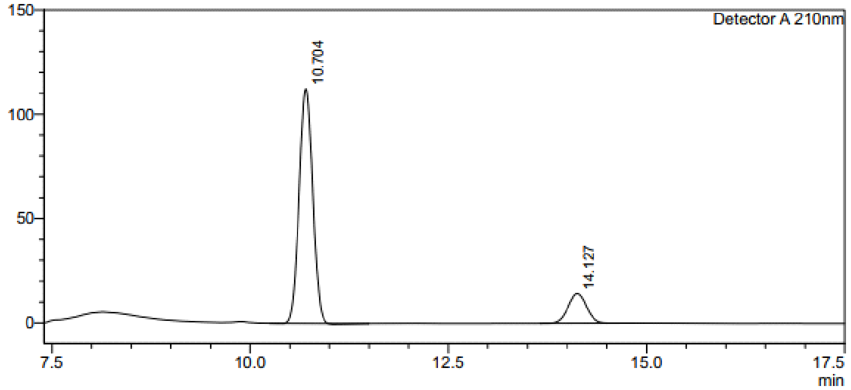

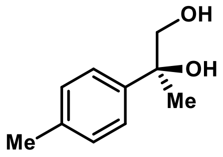

C65
95% yield, 70% ee
obtained by ligand **E**

<Peak Table>

Detector A 210nm

Peak#	Ret. Time	Area	Height	Conc.	Unit	Mark	Name
1	10.704	1323305	112347	85.191		M	
2	14.127	230037	14267	14.809			
Total		1553342	126615				

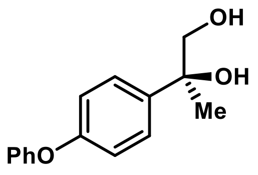

C66

C66 (23.6 mg, 97% yield, 67% ee) was obtained as white solid.

^{1}H NMR (400 MHz, CDCl$_3$): δ 7.41 (d, *J* = 8.4 Hz, 2H), 7.34 (t, *J* = 7.6 Hz, 2H), 7.11 (t, *J* = 7.6 Hz, 1H), 7.00 (t, *J* = 8.4 Hz, 4H), 3.78 (d, *J* = 11.1 Hz, 1H), 3.63 (d, *J* = 11.1 Hz, 1H), 2.64 (s, 1H), 1.98 (s, 1H), 1.54 (s, 3H) ppm

^{13}C NMR (101 MHz, CDCl$_3$): δ 157.0, 156.4, 139.6, 129.7, 126.6, 123.4, 119.0, 118.5, 74.6, 71.1, 26.1 ppm

HRMS (ESI+): calculated for $C_{15}H_{16}O_3Na$ [M+Na]$^+$: 267.0992, found 267.0990.

HPLC (AD-H, 0.46*25 cm, 5μm, hexane / isopropanol = 95/5, flow 1 mL/min, detection at 210 nm) retention time = 12.214 min (major) and 12.925 min (minor).

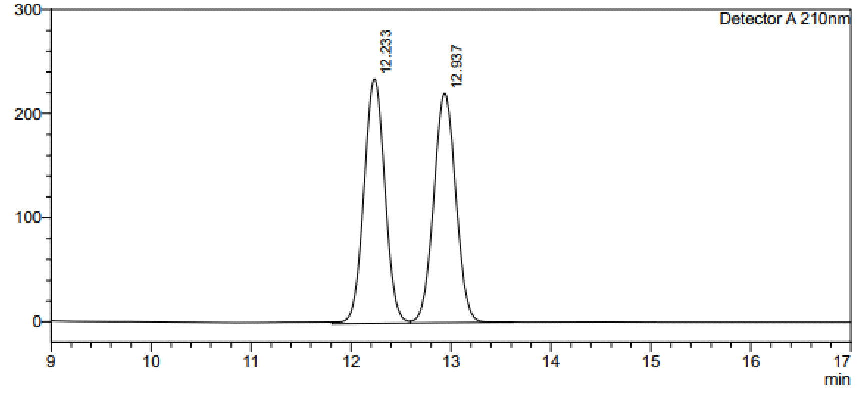

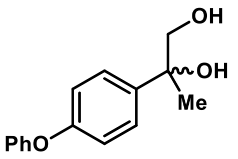

(±)-**C66**

<Peak Table>

Detector A 210nm

Peak#	Ret. Time	Area	Height	Conc.	Unit	Mark	Name
1	12.233	3428875	235276	50.192			
2	12.937	3402638	220495	49.808		V	
Total		6831514	455771				

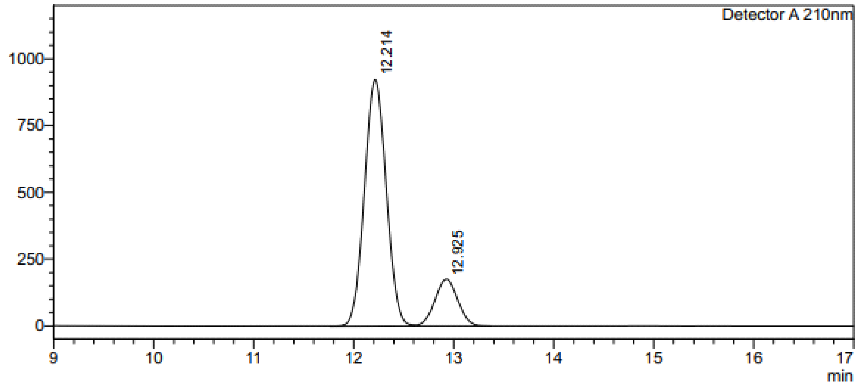

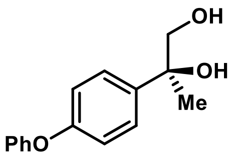

C66
97% yield, 67% ee
obtained by ligand **E**

<Peak Table>

Detector A 210nm

Peak#	Ret. Time	Area	Height	Conc.	Unit	Mark	Name
1	12.214	14022259	923637	83.512		M	
2	12.925	2768492	174331	16.488		M	
Total		16790752	1097968				

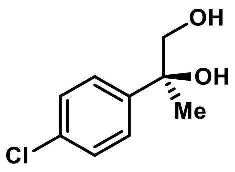

C67

C67 (18.2 mg, 98% yield, 63% ee) was obtained as white solid.

^{1}H NMR (460 MHz, $CDCl_3$): δ 7.35 (d, J = 8.7 Hz, 2H), 7.31 (d, J = 8.7 Hz, 2H), 3.69 (d, J = 11.2 Hz, 1H), 3.56 (d, J = 11.2 Hz, 1H), 3.03 (s, 1H), 2.50 (s, 1H), 1.47 (s, 3H) ppm

^{13}C NMR (151 MHz, $CDCl_3$): δ 143.5, 133.0, 128.4, 126.6, 74.6, 70.7, 25.9 ppm

HRMS (ESI+): calculated for $C_9H_{11}O_2Cl_2$ [M+Cl]$^-$: 221.0136, found 221.0138.

HPLC (AD-H, 0.46*25 cm, 5μm, hexane / ethanol = 90/10, flow 1 mL/min, detection at 210 nm) retention time = 12.645 min (major) and 14.429 min (minor).

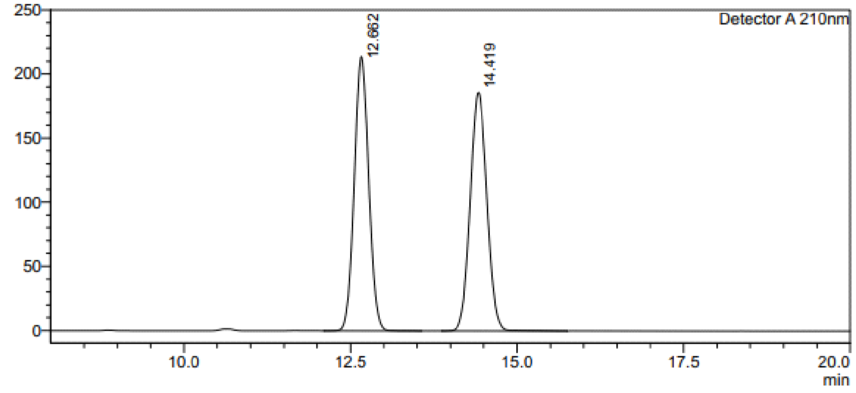

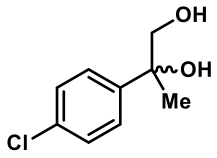

(±)-**C67**

<Peak Table>

Detector A 210nm

Peak#	Ret. Time	Area	Height	Conc.	Unit	Mark	Name
1	12.662	3257597	213957	50.047			
2	14.419	3251439	185957	49.953			
Total		6509036	399914				

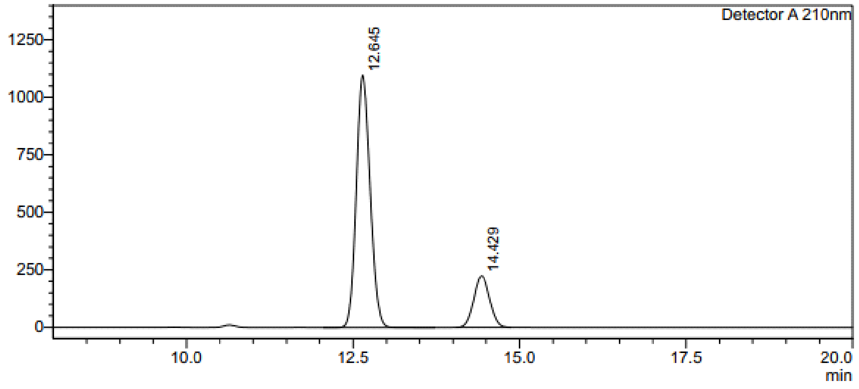

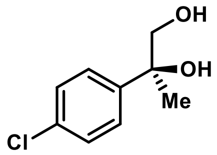

C67
98% yield, 63% ee
obtained by ligand **E**

<Peak Table>

Detector A 210nm

Peak#	Ret. Time	Area	Height	Conc.	Unit	Mark	Name
1	12.645	15981339	1095993	81.320			
2	14.429	3671021	223069	18.680		M	
Total		19652360	1319062				

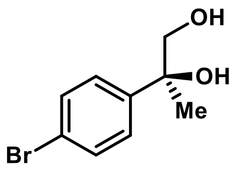

C68

C68 (22.5 mg, 97% yield, 63% ee) was obtained as white solid.

^{1}H NMR (400 MHz, $CDCl_3$): δ 7.48 (d, *J* = 8.5 Hz, 2H), 7.31 (d, *J* = 8.5 Hz, 2H), 3.72 (d, *J* = 11.1 Hz, 1H), 3.59 (d, *J* = 11.1 Hz, 1H), 2.38 (s, 2H), 1.49 (s, 3H) ppm

^{13}C NMR (101 MHz, $CDCl_3$): δ 144.0, 131.4, 127.0, 121.2, 74.6, 70.8, 25.9 ppm

HRMS (ESI+): calculated for $C_9H_{11}O_2NaBr$ $[M+Na]^+$: 252.9835, found 252.9834.

HPLC (OJ-H, 0.46*25 cm, 5μm, hexane / ethanol = 95/5, flow 1 mL/min, detection at 210 nm) retention time = 15.912 min (major) and 18.589 min (minor).

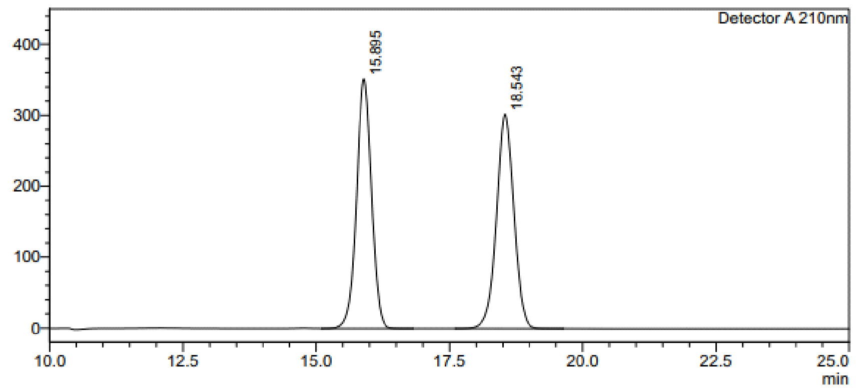

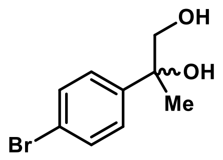

(±)-**C68**

<Peak Table>

Detector A 210nm

Peak#	Ret. Time	Area	Height	Conc.	Unit	Mark	Name
1	15.895	6906207	351529	49.982			
2	18.543	6911187	302239	50.018			
Total		13817394	653768				

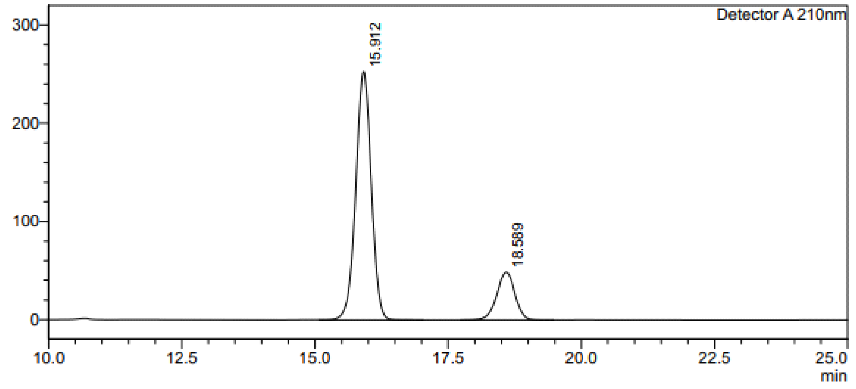

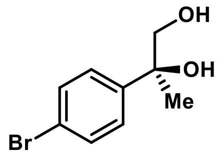

C68
97% yield, 63% ee
obtained by ligand **E**

<Peak Table>

Detector A 210nm

Peak#	Ret. Time	Area	Height	Conc.	Unit	Mark	Name
1	15.912	5131579	252883	81.683			
2	18.589	1150763	48885	18.317			
Total		6282342	301768				

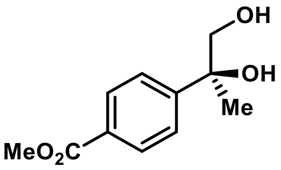

C69

C69 (19.7 mg, 94% yield, 62% ee) was obtained as white solid.

^{1}H NMR (400 MHz, $CDCl_3$): δ 8.01 (d, *J* = 8.4 Hz, 2H), 7.53 (d, *J* = 8.4 Hz, 2H), 3.91 (s, 3H), 3.80 (d, *J* = 11.1 Hz, 1H), 3.66 (d, *J* = 11.1 Hz, 1H), 2.24 (s, 2H), 1.54 (s, 3H) ppm

^{13}C NMR (101 MHz, $CDCl_3$): δ 166.9, 150.2, 129.7, 129.0, 125.2, 74.9, 70.8, 52.1, 26.0 ppm

HRMS (ESI+): calculated for $C_{11}H_{14}O_4Na$ [M+Na]$^+$: 233.0784, found233.0785.

HPLC (OD-H, 0.46*25 cm, 5μm, hexane / ethanol = 95/5, flow 1 mL/min, detection at 210 nm) retention time = 19.161 min (minor) and 22.574 min (major).

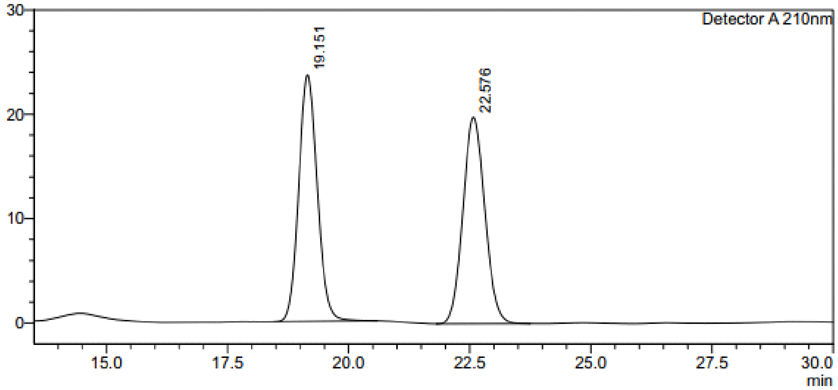

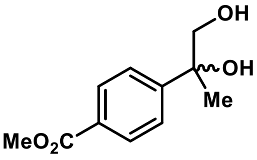

(±)-**C70**

<Peak Table>

Detector A 210nm

Peak#	Ret. Time	Area	Height	Conc.	Unit	Mark	Name
1	19.151	632434	23602	50.277		M	
2	22.576	625458	19806	49.723			
Total		1257892	43408				

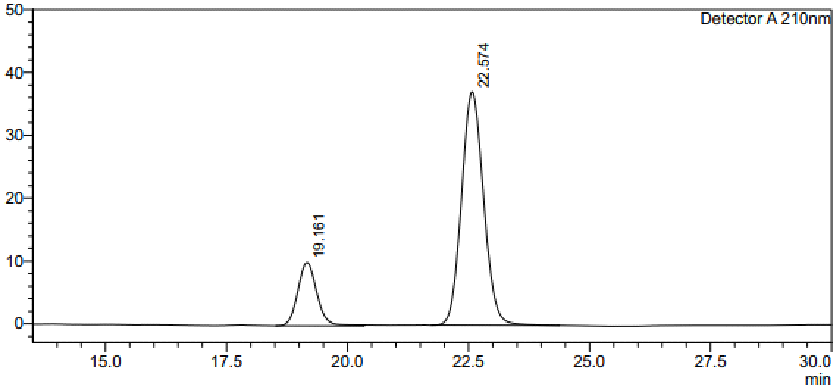

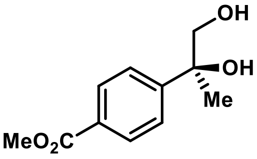

C70
94% yield, 62% ee
obtained by ligand **E**

<Peak Table>

Detector A 210nm

Peak#	Ret. Time	Area	Height	Conc.	Unit	Mark	Name
1	19.161	278778	10106	19.093		M	
2	22.574	1181314	37146	80.907			
Total		1460092	47251				

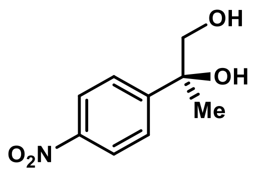

C70

C70 (19.0 mg, 96% yield, 55% ee) was obtained as white solid.

^{1}H NMR (400 MHz, CDCl$_3$): δ 8.19 (d, *J* = 8.9 Hz, 2H), 7.63 (d, *J* = 8.9 Hz, 2H), 3.80 (d, *J* = 11.0 Hz, 1H), 3.70 (d, *J* = 11.0 Hz, 1H), 2.32 (s, 2H), 1.55 (s, 3H) ppm

^{13}C NMR (101 MHz, CDCl$_3$): δ 152.6, 147.0, 126.2, 123.5, 74.8, 70.6, 26.0 ppm

HRMS (ESI+): calculated for $C_9H_{11}NO_4Na$ $[M+Na]^+$: 220.0580, found 220.0581.

HPLC (OJ-H, 0.46*25 cm, 5μm, hexane / ethanol = 95/5, flow 1 mL/min, detection at 210 nm) retention time = 38.520 min (major) and 46.411 min (minor).

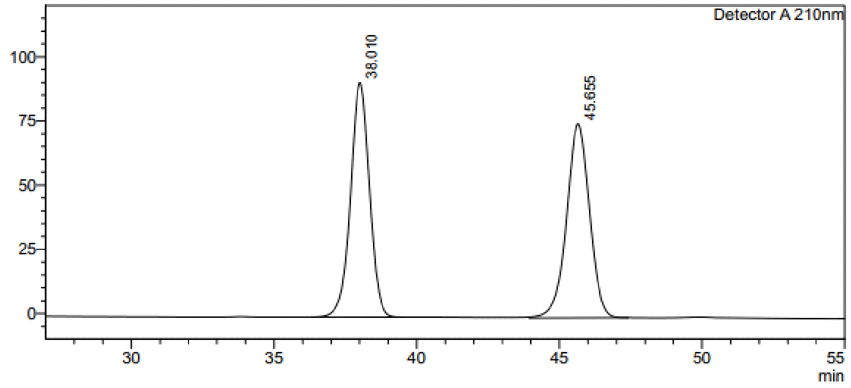

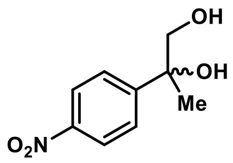

(±)-**C70**

<Peak Table>

Detector A 210nm

Peak#	Ret. Time	Area	Height	Conc.	Unit	Mark	Name
1	38.010	4223636	91351	50.001			
2	45.655	4223533	75488	49.999		M	
Total		8447169	166839				

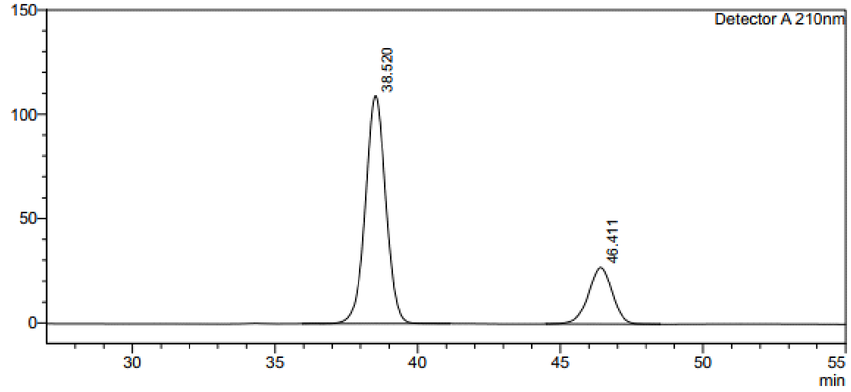

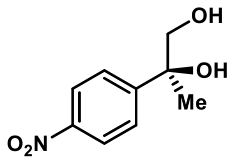

C70
96% yield, 55% ee
obtained by ligand **E**

<Peak Table>

Detector A 210nm

Peak#	Ret. Time	Area	Height	Conc.	Unit	Mark	Name
1	38.520	5424541	109042	77.379		M	
2	46.411	1585804	27053	22.621			
Total		7010345	136095				

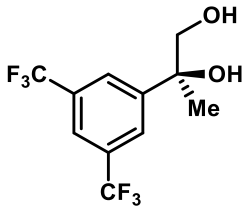

C71

C71 (27.3 mg, 98% yield, 50% ee) was obtained as white solid.

^{1}H NMR (600 MHz, MeOD): δ 8.10 (s, 2H), 7.84 (s, 1H), 3.68 (d, J = 11.2 Hz, 1H), 3.62 (d, J = 11.2 Hz, 1H), 1.56 (s, 3H) ppm

^{13}C NMR (151 MHz, MeOD): δ 151.5, 132.2 (q, J = 32.9 Hz), 127.5, 125.1 (q, J = 271.6 Hz), 121.4 (q, J = 4.1 Hz), 75.2, 71.4, 26.2 ppm

^{19}F NMR (565 MHz, MeOD): δ -64.26 (s) ppm

HRMS (ESI+): calculated for $C_{11}H_{10}O_2F_6Cl$ $[M+Cl]^+$: 323.0274, found 323.0279.

HPLC (OD-H, 0.46*25 cm, 5μm, hexane / ethanol = 97/3, flow 1 mL/min, detection at 210 nm) retention time = 9.424 min (major) and 9.938 min (minor).

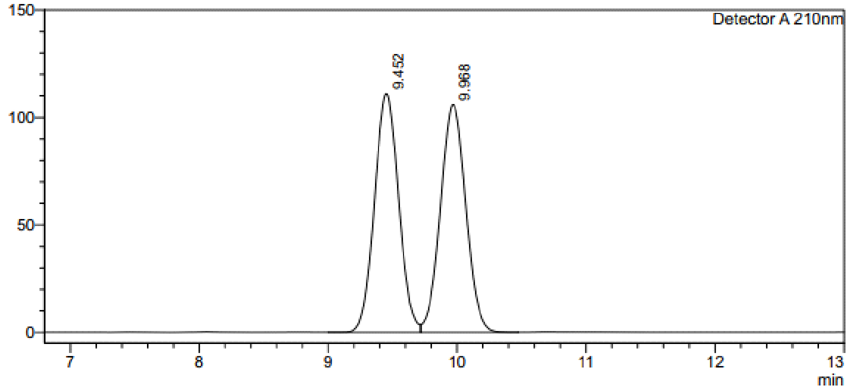

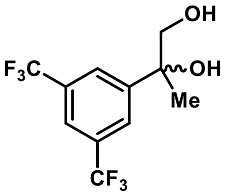

(±)-**C71**

<Peak Table>

Detector A 210nm

Peak#	Ret. Time	Area	Height	Conc.	Unit	Mark	Name
1	9.452	1450348	110950	49.994			
2	9.968	1450707	105953	50.006		V	
Total		2901055	216903				

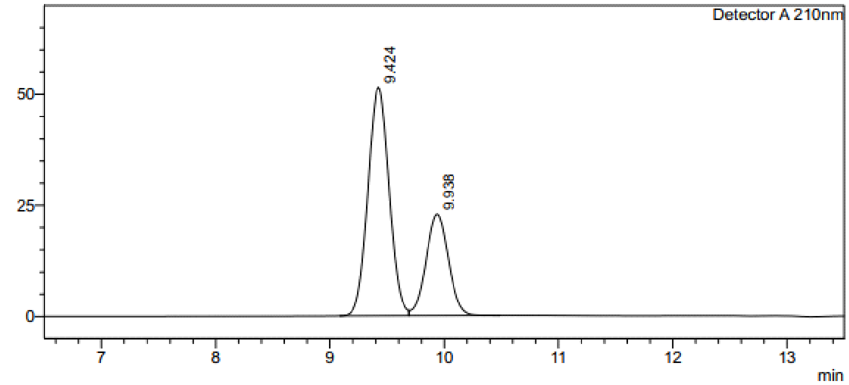

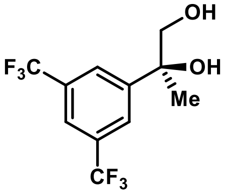

C71

95% yield, 36% ee
obtained by ligand **E**

<Peak Table>

Detector A 210nm

Peak#	Ret. Time	Area	Height	Conc.	Unit	Mark	Name
1	9.424	664126	51429	68.155		M	
2	9.938	310304	22803	31.845		V M	
Total		974430	74231				

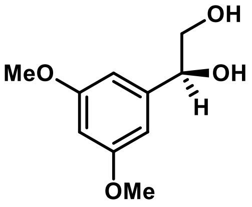

C72

C72 (19.4 mg, 98% yield, 86% ee) was obtained as white solid.
HPLC (OD-H, 0.46*25 cm, 5μm, hexane / ethanol = 95/5, flow 1 mL/min, detection at 210 nm) retention time = 9.804 min (minor) and 10.673 min (major).

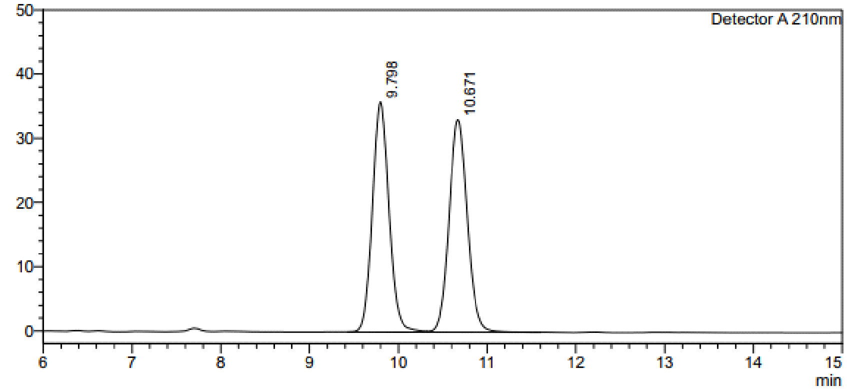

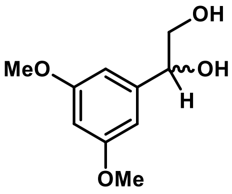

(±)-**C72**

<Peak Table>

Detector A 210nm

Peak#	Ret. Time	Area	Height	Conc.	Unit	Mark	Name
1	9.798	470216	35837	49.847		V	
2	10.671	473106	33074	50.153		V	
Total		943322	68910				

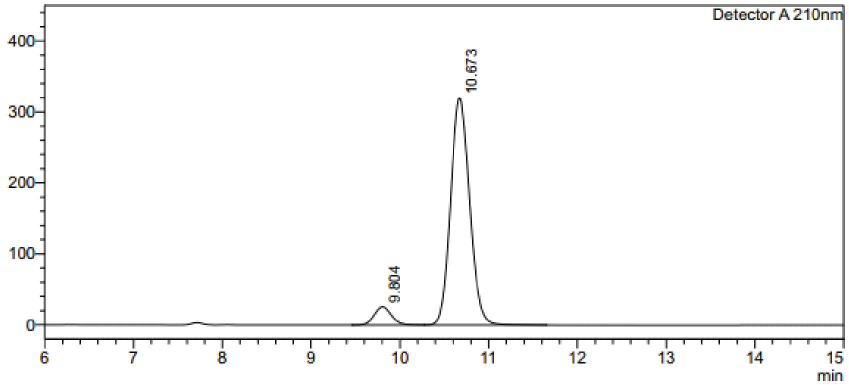

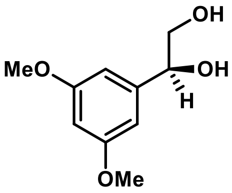

C72
98% yield, 86% ee
obtained by ligand **E**

<Peak Table>

Detector A 210nm

Peak#	Ret. Time	Area	Height	Conc.	Unit	Mark	Name
1	9.804	353390	25928	6.887			
2	10.673	4777734	320030	93.113		V M	
Total		5131125	345958				

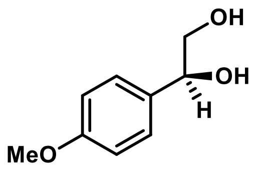

C73

C73 (16.6 mg, 99% yield, 81% ee) was obtained as white solid.

^{1}H NMR (400 MHz, $CDCl_3$): δ 7.25 (d, *J* = 8.7 Hz, 2H), 6.87 (d, *J* = 8.7 Hz, 2H), 4.73 (dd, *J* = 7.9, 3.2 Hz, 1H), 3.79 (s, 3H), 3.73 – 3.57 (m, 2H), 2.78 (s, 2H) ppm

^{13}C NMR (101 MHz, $CDCl_3$): δ 159.3, 132.6, 127.3, 113.9, 74.3, 68.0, 55.2 ppm

HRMS (ESI+): calculated for $C_9H_{12}O_3Na$ $[M+Na]^+$: 191.0679, found 191.0679.

HPLC (OD-H, 0.46*25 cm, 5μm, hexane / ethanol = 95/5, flow 1 mL/min, detection at 210 nm) retention time = 17.449 min (minor) and 18.596 min (major).

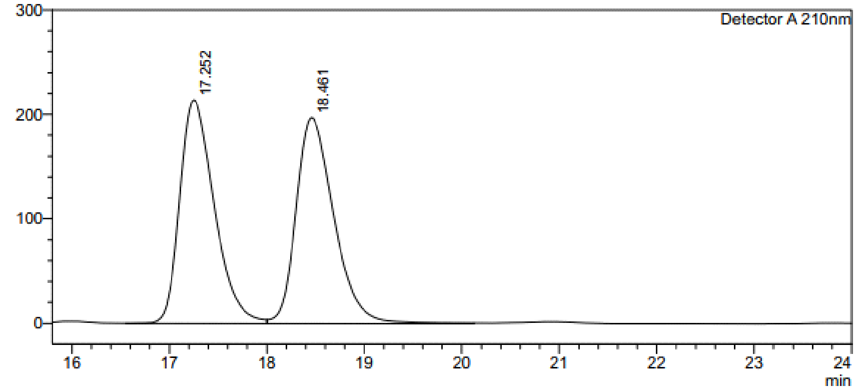

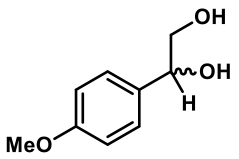

(±)-**C73**

<Peak Table>

Detector A 210nm

Peak#	Ret. Time	Area	Height	Conc.	Unit	Mark	Name
1	17.252	5257605	213847	49.785			
2	18.461	5302935	197044	50.215		V	
Total		10560540	410891				

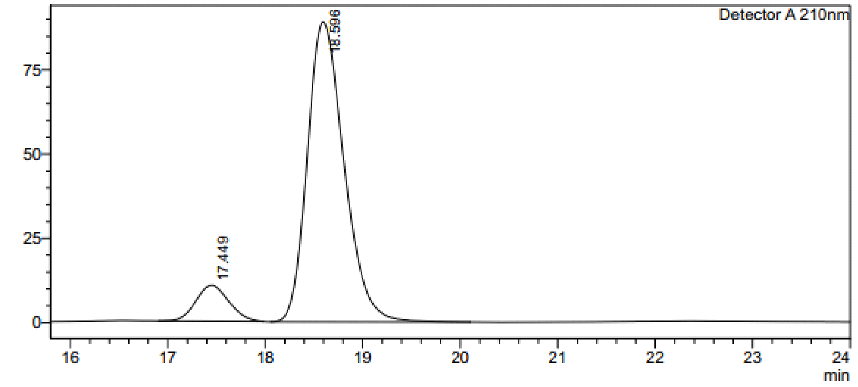

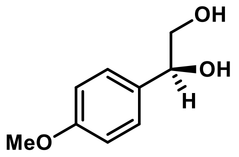

C73
99% yield, 81% ee
obtained by ligand **E**

<Peak Table>

Detector A 210nm

Peak#	Ret. Time	Area	Height	Conc.	Unit	Mark	Name
1	17.449	249253	10612	9.649		M	
2	18.596	2333958	88990	90.351		M	
Total		2583210	99602				

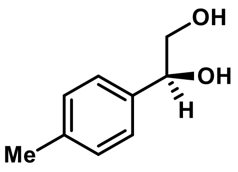

C74

C74 (15.0 mg, 99% yield, 80% ee) was obtained as white solid.

^{1}H NMR (400 MHz, CDCl$_3$): δ 7.23 (d, J = 8.0 Hz, 1H), 7.15 (d, J = 8.0 Hz, 1H), 4.76 (dd, J = 8.2, 3.5 Hz, 1H), 3.75 – 3.57 (m, 2H), 3.25 – 2.57 (m, 2H), 2.34 (s, 2H) ppm
^{13}C NMR (101 MHz, CDCl$_3$): δ 137.7, 137.5, 129.2, 126.0, 74.5, 68.0, 21.1 ppm
HRMS (ESI+): calculated for $C_9H_{12}O_2Na$ [M+Na]$^+$: 175.0730, found 175.0730.
HPLC (OD-H, 0.46*25 cm, 5μm, hexane / ethanol = 95/5, flow 1 mL/min, detection at 210 nm) retention time = 11.098 min (minor) and 12.193 min (major).

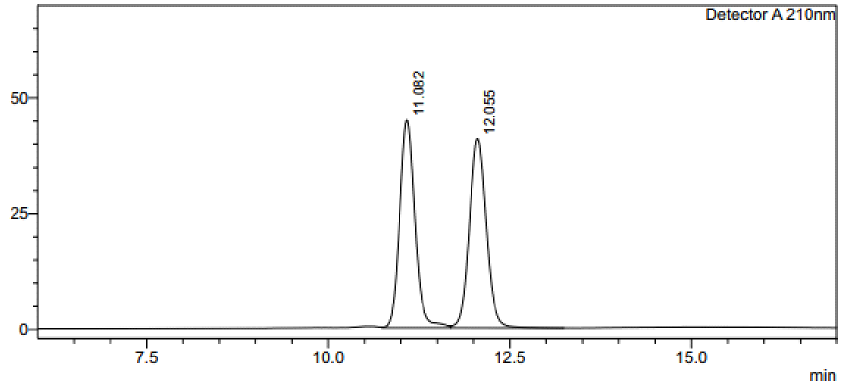

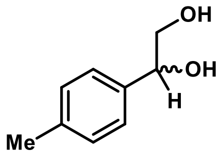

(±)-**C74**

<Peak Table>
Detector A 210nm

Peak#	Ret. Time	Area	Height	Conc.	Unit	Mark	Name
1	11.082	659238	44841	49.847			
2	12.055	663278	40869	50.153		V	
Total		1322516	85711				

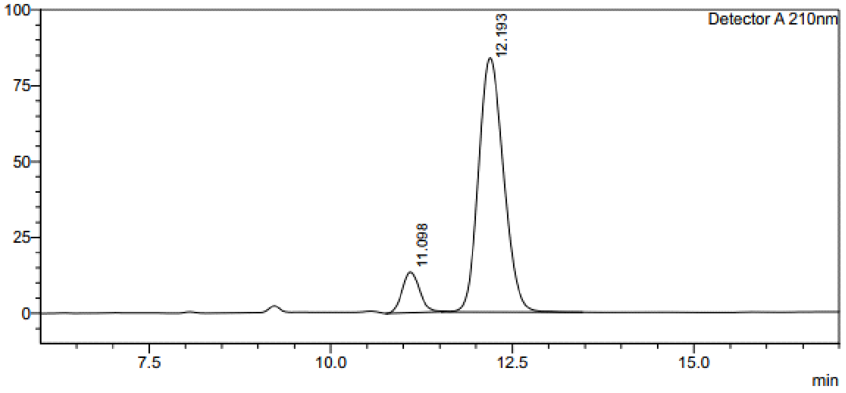

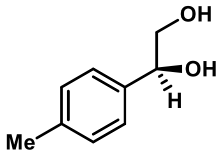

C74
99% yield, 80% ee
obtained by ligand **E**

<Peak Table>
Detector A 210nm

Peak#	Ret. Time	Area	Height	Conc.	Unit	Mark	Name
1	11.098	225505	13457	10.074		M	
2	12.193	2012876	83847	89.926		M	
Total		2238382	97305				

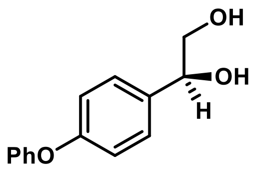

C75

Following the general procedure, **C75** (22.6 mg, 98% yield, 79% ee) was obtained as white solid.
^{1}H NMR (400 MHz, CDCl$_3$): δ 7.32 (dd, *J* = 12.4, 5.1 Hz, 4H), 7.11 (t, *J* = 7.4 Hz, 1H), 7.05 – 6.94 (m, 4H), 4.80 (dd, *J* = 8.2, 3.4 Hz, 1H), 3.74 (dd, *J* = 11.3, 3.4 Hz, 1H), 3.65 (dd, *J* = 11.3, 8.2 Hz, 1H), 2.51 (s, 2H) ppm
^{13}C NMR (101 MHz, CDCl$_3$): δ 157.1, 157.0, 135.2, 129.8, 127.5, 123.4, 119.0, 118.7, 74.2, 68.0 ppm
HRMS (ESI+): calculated for $C_{14}H_{14}O_3Na$ [M+Na]$^+$: 253.0835, found 253.0834.
HPLC (OD-H, 0.46*25 cm, 5μm, hexane / ethanol = 95/5, flow 1 mL/min, detection at 210 nm) retention time = 16.858 min (minor) and 18.145 min (major).

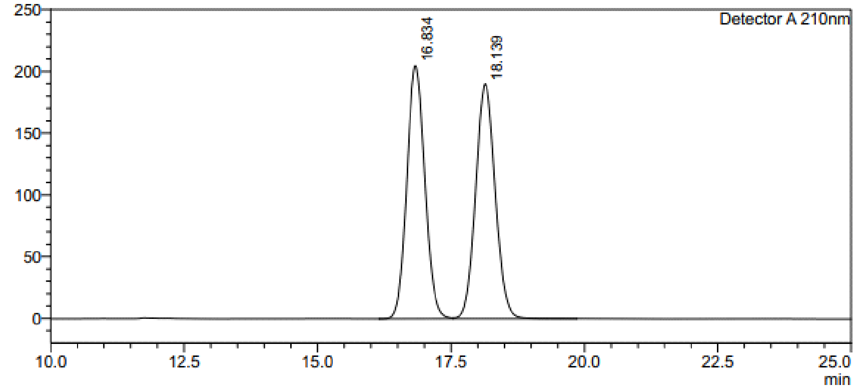

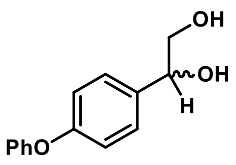

(±)-**C75**

<Peak Table>

Detector A 210nm

Peak#	Ret. Time	Area	Height	Conc.	Unit	Mark	Name
1	16.834	4834695	204933	49.903			
2	18.139	4853437	189950	50.097		V	
Total		9688132	394883				

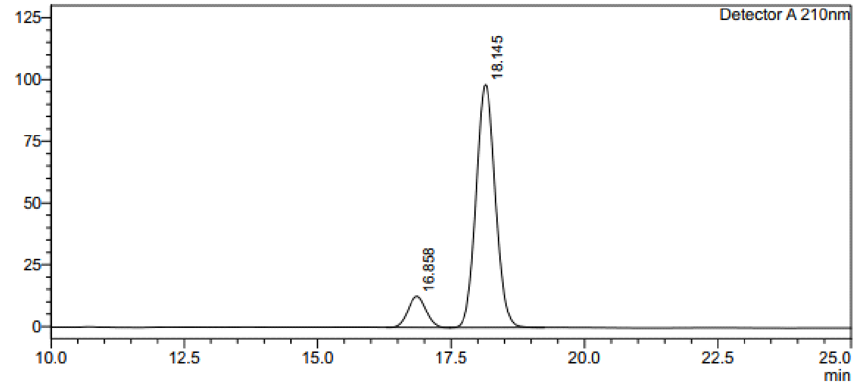

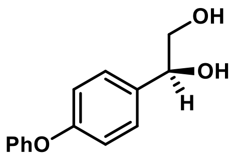

C75
98% yield, 79% ee
obtained by ligand **E**

<Peak Table>

Detector A 210nm

Peak#	Ret. Time	Area	Height	Conc.	Unit	Mark	Name
1	16.858	296327	12570	10.702			
2	18.145	2472687	98371	89.298			
Total		2769014	110941				

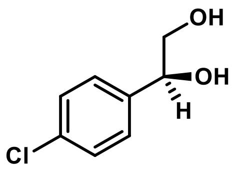

C76

C76 (17.1 mg, 99% yield, 73% ee) was obtained as white solid.

^{1}H NMR (400 MHz, CDCl$_3$): δ 7.32 (d, *J* = 8.5 Hz, 2H), 7.27 (d, *J* = 8.5 Hz, 2H), , 4.77 (dd, *J* = 8.2, 2.4 Hz, 1H), 3.71 (dd, *J* = 11.0, 2.4 Hz, 1H), 3.59 (dd, *J* = 11.0, 8.2 Hz, 1H), 2.72 (s, 2H) ppm
^{13}C NMR (101 MHz, CDCl$_3$): δ 138.9, 133.7, 128.7, 127.4, 74.0, 67.9 ppm
HRMS (ESI+): calculated for $C_8H_9O_2Cl_2$ [M+ Cl]$^-$: 206.9980, found 206.9980.
HPLC (OD-H, 0.46*25 cm, 5μm, hexane / ethanol = 97/3, flow 1 mL/min, detection at 210 nm) retention time = 21.855 min (minor) and 24.099 min (major).

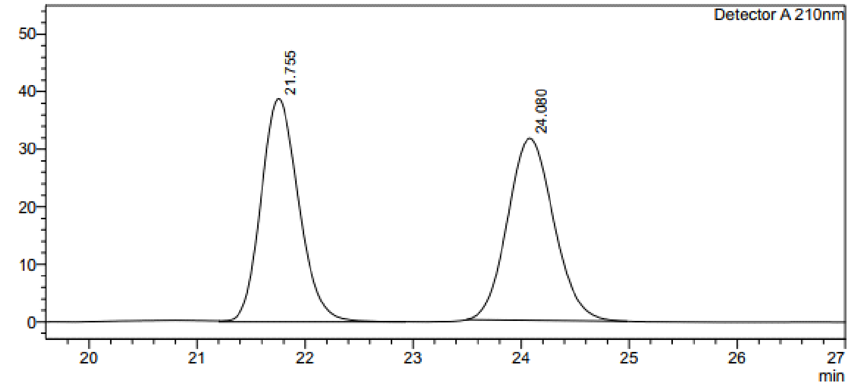

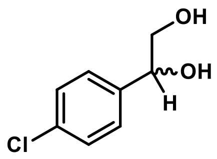

(±)-**C76**

<Peak Table>

Detector A 210nm

Peak#	Ret. Time	Area	Height	Conc.	Unit	Mark	Name
1	21.755	939848	38744	49.977			
2	24.080	940720	31640	50.023		M	
Total		1880568	70384				

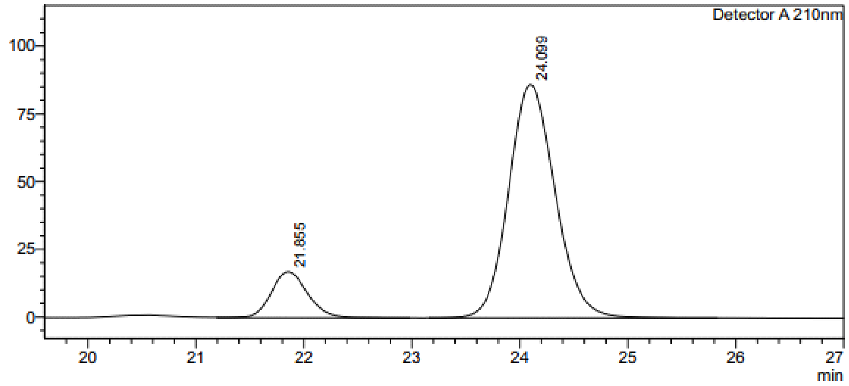

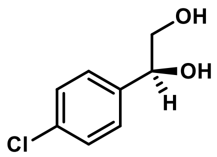

C76
99% yield, 73% ee
obtained by ligand **E**

<Peak Table>

Detector A 210nm

Peak#	Ret. Time	Area	Height	Conc.	Unit	Mark	Name
1	21.855	398298	16997	13.345			
2	24.099	2586240	86145	86.655			
Total		2984538	103142				

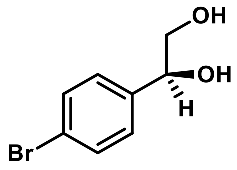

C77

C77 (21.3 mg, 99% yield, 72% ee) was obtained as white solid.
^{1}H NMR (400 MHz, $CDCl_3$): δ 7.49 (d, *J* = 8.4 Hz, 2H), 7.25 (d, *J* = 8.4 Hz, 2H), 4.79 (dd, *J* = 8.1, 3.5 Hz, 1H), 3.75 (dd, *J* = 11.3, 3.5 Hz, 1H), 3.61 (dd, *J* = 11.3, 8.1 Hz, 1H), 1.76 (s, 2H) ppm
^{13}C NMR (101 MHz, $CDCl_3$): δ 139.4, 131.6, 127.8, 121.8, 74.0, 67.9 ppm
HRMS (ESI+): calculated for $C_8H_9O_2NaBrCl$ $[M+Cl]^-$: 250.9474, found 250.9479.
HPLC (OD-H, 0.46*25 cm, 5μm, hexane / ethanol = 97/3, flow 1 mL/min, detection at 210 nm) retention time = 13.676 min (minor) and 14.880 min (major).

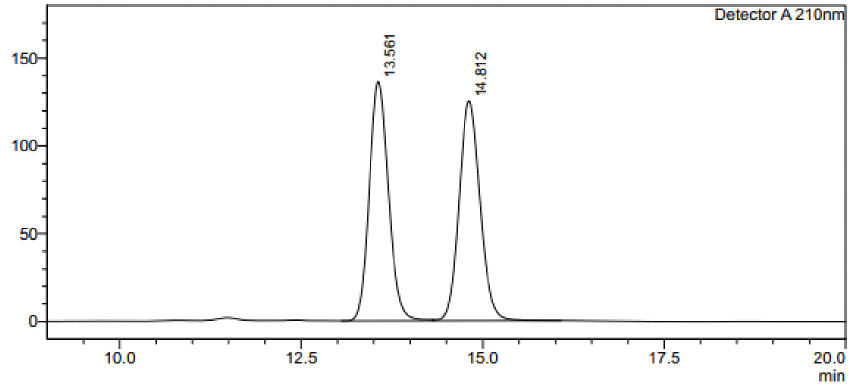

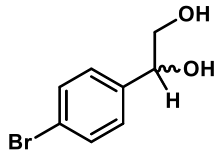

(±)-**C77**

<Peak Table>
Detector A 210nm

Peak#	Ret. Time	Area	Height	Conc.	Unit	Mark	Name
1	13.561	2511415	136279	49.990			
2	14.812	2512441	125119	50.010		V	
Total		5023856	261398				

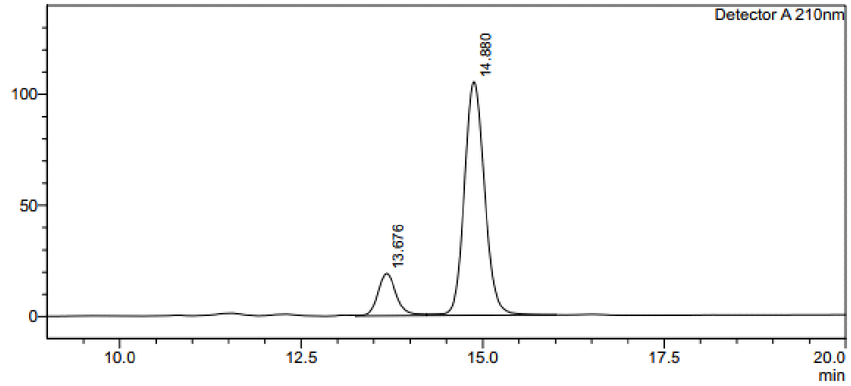

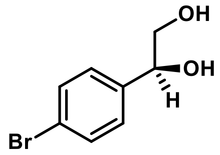

C77
99% yield, 72% ee
obtained by ligand **E**

<Peak Table>
Detector A 210nm

Peak#	Ret. Time	Area	Height	Conc.	Unit	Mark	Name
1	13.676	336475	19118	14.190			
2	14.880	2034807	105079	85.810		V	
Total		2371282	124197				

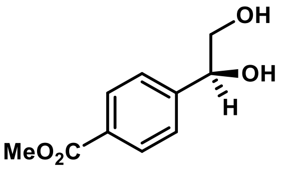

C78

C78 (19.2 mg, 98% yield, 71% ee) was obtained as white solid.

^{1}H NMR (400 MHz, CDCl$_3$): δ 8.01 (d, J = 8.2 Hz, 2H), 7.43 (d, J = 8.2 Hz, 2H), 4.87 (dd, J = 8.1, 3.4 Hz, 1H), 3.90 (s, 3H), 3.78 (dd, J = 11.3, 3.4 Hz, 1H), 3.63 (dd, J = 11.3, 8.1 Hz, 1H), 2.55 (s, 2H) ppm

^{13}C NMR (101 MHz, CDCl$_3$): δ 166.9, 145.6, 129.8, 129.7, 126.0, 74.3, 67.9, 52.2 ppm

HRMS (ESI+): calculated for $C_{10}H_{12}O_4Na$ [M+Na]$^+$: 219.0628, found 219.0628.

HPLC (OD-H, 0.46*25 cm, 5μm, hexane / ethanol = 95/5, flow 1 mL/min, detection at 210 nm) retention time = 20.957 min (minor) and 22.748 min (major).

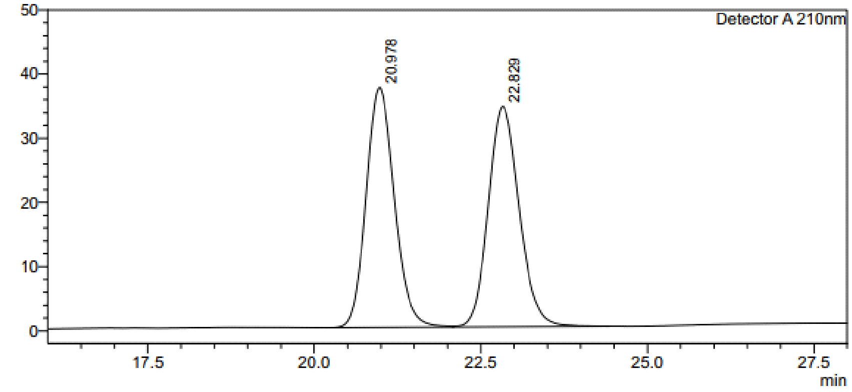

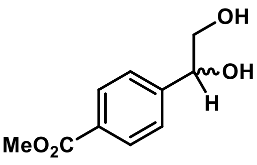

(±)-**C78**

<Peak Table>

Detector A 210nm

Peak#	Ret. Time	Area	Height	Conc.	Unit	Mark	Name
1	20.978	1100999	37371	49.864			
2	22.829	1106989	34298	50.136		V	
Total		2207988	71668				

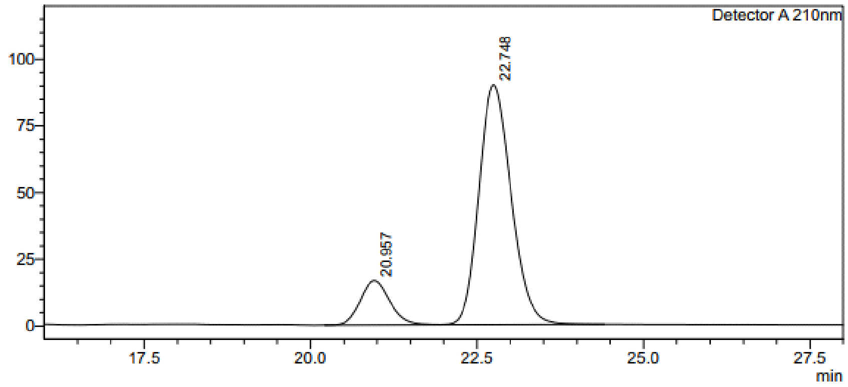

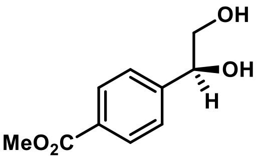

C78
98% yield, 71% ee
obtained by ligand **E**

<Peak Table>

Detector A 210nm

Peak#	Ret. Time	Area	Height	Conc.	Unit	Mark	Name
1	20.957	512793	16579	14.462			
2	22.748	3032888	89902	85.538		V	
Total		3545682	106481				

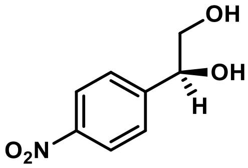

C79

C79 (17.8 mg, 97% yield, 62% ee) was obtained as white solid.

^{1}H NMR (600 MHz, MeOD): δ 8.23 (d, *J* = 8.4 Hz, 2H), 7.65 (d, *J* = 8.4 Hz, 2H), 4.84 – 4.80 (m, 1H), 3.69 (dd, *J* = 11.3, 4.9 Hz, 1H), 3.66 (dd, *J* = 11.3, 6.7 Hz, 1H) ppm
^{13}C NMR (151 MHz, MeOD): δ 151.4, 148.7, 128.5, 124.2, 74.9, 68.3 ppm
HRMS (ESI+): calculated for $C_8H_9NO_4Cl$ [M+Cl]$^-$: 218.0220, found 218.0222.
HPLC (OD-H, 0.46*25 cm, 5μm, hexane / isopropanol = 90/10, flow 1 mL/min, detection at 210 nm) retention time = 16.072 min (minor) and 18.008 min (major).

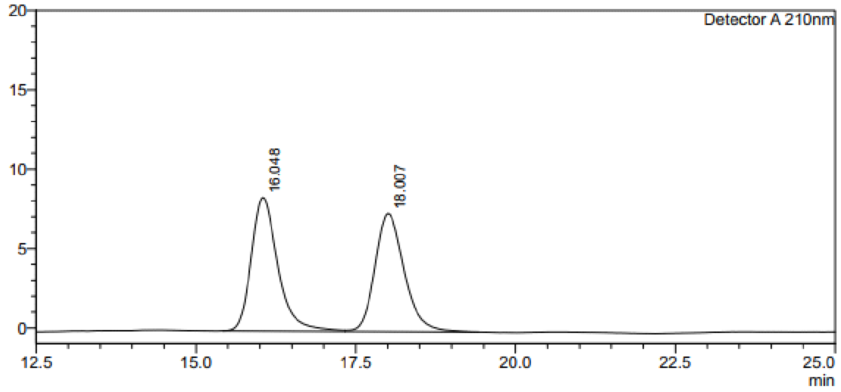

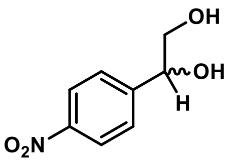

(±)-**C79**

<Peak Table>

Detector A 210nm

Peak#	Ret. Time	Area	Height	Conc.	Unit	Mark	Name
1	16.048	244045	8402	50.792			
2	18.007	236434	7433	49.208		V	
Total		480479	15835				

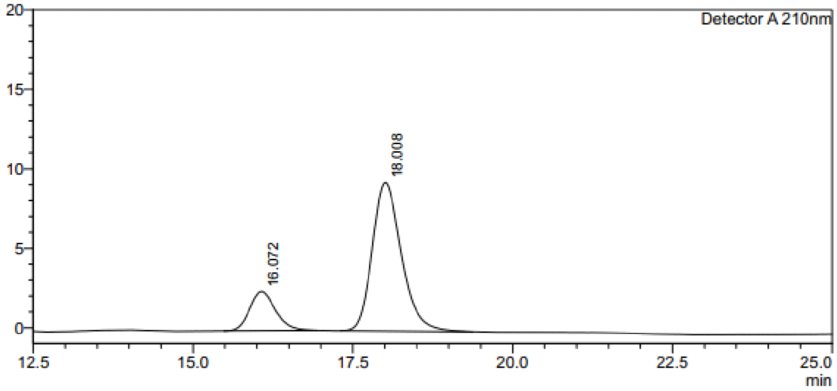

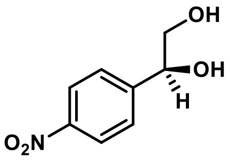

C79
97% yield, 62% ee
obtained by ligand **E**

<Peak Table>

Detector A 210nm

Peak#	Ret. Time	Area	Height	Conc.	Unit	Mark	Name
1	16.072	68888	2461	18.836			
2	18.008	296840	9344	81.164			
Total		365728	11805				

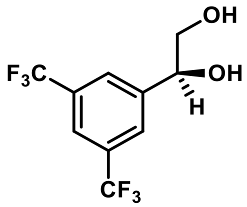

C80

C80 (25.8 mg, 94% yield, 53% ee) was obtained as white solid.

^{1}H NMR (400 MHz, MeOD): δ 8.00 (s, 2H), 7.86 (s, 1H), 4.86 (d, *J* = 5.6 Hz, 1H), 3.69 (d, *J* = 5.6 Hz, 2H) ppm

^{13}C NMR (101 MHz, MeOD): δ 147.7 132.4 (q, *J* = 33.2 Hz), 128.1, 125.0 (q, *J* = 271.7 Hz), 121.9 (q, *J* = 3.8 Hz), 74.3, 68.1 ppm

^{19}F NMR (376 MHz, MeOD): δ -64.33 (s) ppm

HRMS (ESI+): calculated for $C_{10}H_8O_2ClF_6$ $[M+Cl]^-$: 309.0117, found 309.0124.

HPLC (OD-H, 0.46*25 cm, 5μm, hexane / ethanol = 97/3, flow 1 mL/min, detection at 210 nm) retention time = 11.488 min (minor) and 13.150 min (major).

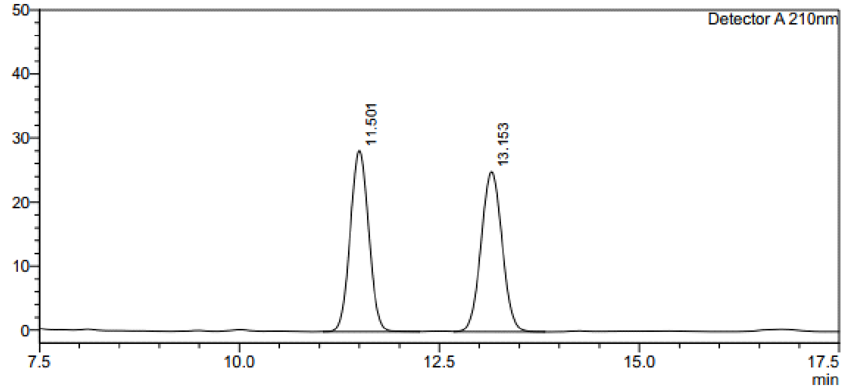

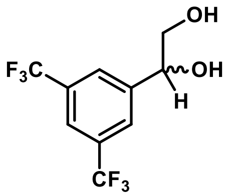

(±)-**C80**

<Peak Table>

Detector A 210nm

Peak#	Ret. Time	Area	Height	Conc.	Unit	Mark	Name
1	11.501	455197	28303	49.868			
2	13.153	457601	24997	50.132			
Total		912799	53300				

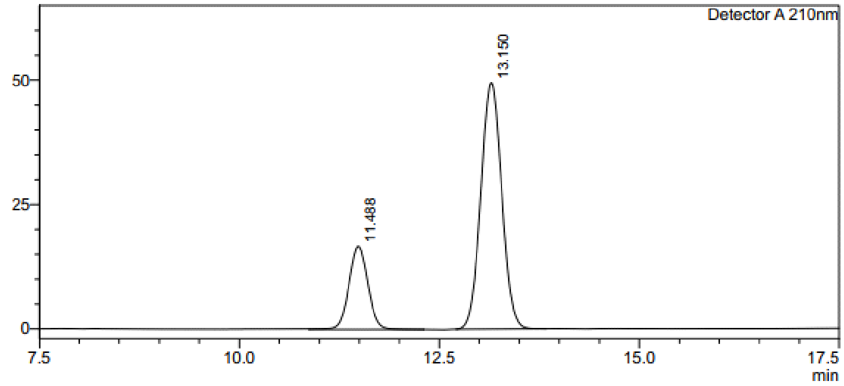

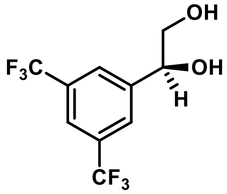

C80
94% yield, 53% ee
obtained by ligand **E**

<Peak Table>

Detector A 210nm

Peak#	Ret. Time	Area	Height	Conc.	Unit	Mark	Name
1	11.488	274863	16675	23.419		M	
2	13.150	898803	49507	76.581		M	
Total		1173666	66182				

2.3.5 Determination of the absolute stereochemistry of the dihydroxylation product

Supplementary Table 53. Crystal data and structure refinement for **C74**

Identification code	2235591
Empirical formula	$C_9H_{12}O_2$
Formula weight	152.19
Temperature/K	100.0(2)
Crystal system	monoclinic
Space group	$P2_1$
a/Å	5.5388(3)
b/Å	25.3783(15)
c/Å	5.7980(3)
α/°	90
β/°	90.0360(10)
γ/°	90
Volume/Å^3	815.00(8)
Z	4
ρ_{calc}g/cm^3	1.240
μ/mm^{-1}	0.446
F(000)	328.0
Crystal size/mm^3	0.18 × 0.18 × 0.16
Radiation	GaKα (λ = 1.34139)
2Θ range for data collection/°	3.028 to 147.212
Index ranges	$-7 \leq h \leq 7$, $-36 \leq k \leq 36$, $-8 \leq l \leq 8$
Reflections collected	24752
Independent reflections	4938 [R_{int} = 0.0442, R_{sigma} = 0.0302]
Data/restraints/parameters	4938/1/202
Goodness-of-fit on F^2	1.039
Final R indexes [I>=2σ (I)]	R_1 = 0.0265, wR_2 = 0.0724
Final R indexes [all data]	R_1 = 0.0267, wR_2 = 0.0725
Largest diff. peak/hole / e Å^{-3}	0.26/-0.15
Flack parameter	-0.01(5)

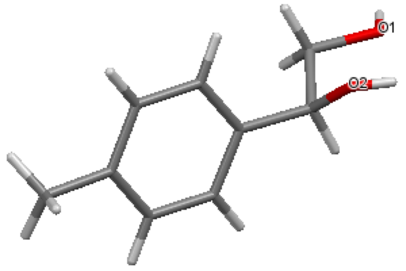

Supplementary Figure 53. The single crystal configuration of **C74** is demonstrated in a capped sticks mode.

3. Computational detail

3.1 Procedure for the calculation of local polarizabilities.

Molecular geometries of substrates were optimized with *Gaussian 16*[9] using the M06-2X functional[10]. The SDD effective core potential (ECP) was chosen to describe iodine, and for other atoms the 6-311+G(d,p) basis set was used. Frequency calculations were carried out at the same level of theory to verify that all the stationary points are minima (zero imaginary frequency) on the potential energy surface. For molecules with multiple conformations, the conformation having the lowest free energy was selected for the subsequent calculation of polarizabilities. Two different schemes for the calculation of local polarizabilities were used in this study. The first scheme involves the direct calculation of atomic polarizabilities in molecules based on Grimme's D4 dispersion model[11] using the GFN2-xTB 6.3 program[12](**Supplementary Figure 54a**). This operationally simple and computationally efficient method was demonstrated to give accurate molecular polarizabilities and correct trends of local polarizabilities for simple hydrocarbons (**Supplementary Figure 55**). The second scheme is based on the effective volume of atom-in-molecules proposed by Tkatchenko-Scheffler (TS)[5] for the calculation of atomic polarizabilities. In the latter scheme, electron densities were first calculated at the M06-2X/aug-cc-pVDZ level of theory with *Gaussian 16*[9], then the atomic polarizabilities were subsequently calculated with Hirshfeld partitioning using the Multiwfn program[13](**Supplementary Figure 54b**). The latter scheme, albeit being more operationally tedious, gave better correlation as compared to the D4-based method (**Supplementary Figure 56**).

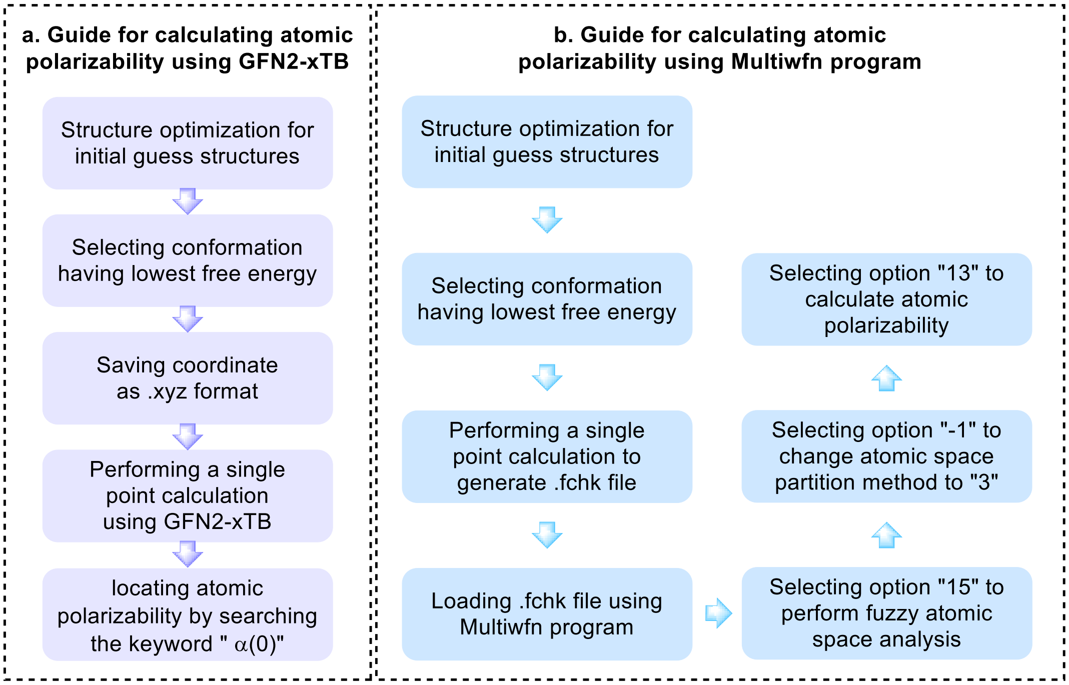

Supplementary Figure 54. Step-by-step guide for calculating atomic polarizabilities using GFN2-xTB and Multiwfn programs, respectively.

3.2 Analysis of key transition states.

The initial structures of key transition states were constructed by performing conformational searches using Grimme's programs xTB 6.3[12] and CREST 2.10.2[14]. The generated conformations were further optimized using the wB97X-D functional with *Gaussian 16*[9]. The SDD effective core potential (ECP) was chosen to describe Ru and Os, and for other atoms the 6-31G(d) basis set was used. Frequency calculations were carried out at the same level of theory to verify that all the stationary points are transition states (one imaginary frequency) on the potential energy surface. Truhlar's quasiharmonic correction[13] was used to obtain thermal correction to free energies of every transition state by setting all positive frequencies that are less than 100 cm^{-1} to 100 cm^{-1}. The higher accuracy single-point energies were calculated using the wB97X-D functional with SDD for Ru and Os, and 6-311+(d,p) basis set for all other atoms, using the SMD solvation model[16] with the corresponding solvents used in experiments. For every transition state, the conformation having the lowest free energy was selected to perform distortion/interaction analysis[17] for exploring the origin of stereoselectivity in each reaction system at the level of wB97X-D/6-311+G(d,p)/SDD(Ru, Os). Furthermore, the independent gradient model based on the Hirshfeld partition (IGMH)[18] was used to display the possible types of non-covalent interactions between catalyst and substrate using Multiwfn and VMD programs, in which the electron densities were obtained at level of wB97X-D/6-311+G(d,p)/SDD(Ru, Os).

3.3 Additional computational results

3.3.1 Grimme's D4 dispersion model

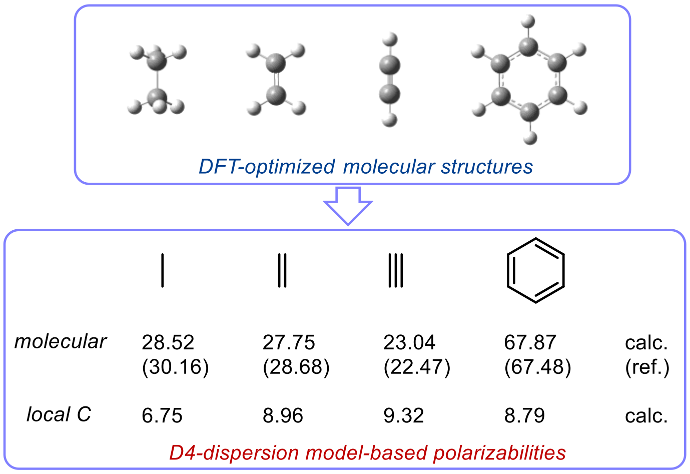

Supplementary Figure 55. Illustration of the computational protocol used in this study for the calculation of molecular and local polarizabilities. The reference value shown in parentheses (ref.) were taken from Miller, T. M., Atomic and Molecular Polarizabilities, *CRC Handbook of Chemistry and Physics* (ed. Lide, D. R.) 10-166 (CRC Press, 2000).

Supplementary Figure 55 illustrates the molecular and local polarizabilities for several representative hydrocarbon molecules calculated based on Grimme's D4 dispersion model. It is worth noting that although molecular polarizability decreases from ethane (C_2H_6) to ethyne (C_2H_2) as molecular volume becomes smaller, an increase of π-character enhances the corresponding local polarizability of carbon atoms. We have also considered to construct quantitative correlations with free energies based on the local polarizabilities calculated using the most efficient D4-based method. The reaction system for the Ru-catalyzed transfer hydrogenation of aryl ketones reported by Ikariya was tested (**Supplementary Figure 56**). In group I (R = *p*-OMe), the coefficient of determination (R^2 = 0.767) is smaller as compared to that obtained using the Tkatchenko-Scheffler (TS) method (R^2 =0.972). Similarly, in group II (R = H), a worse correlation (R^2 = 0.711) between local polarizabilities and $\Delta\Delta G^‡$ was also observed. Thus, for constructing quantitative correlations with free energies, all local polarizabilities were calculated using the Tkatchenko-Scheffler (TS) method.

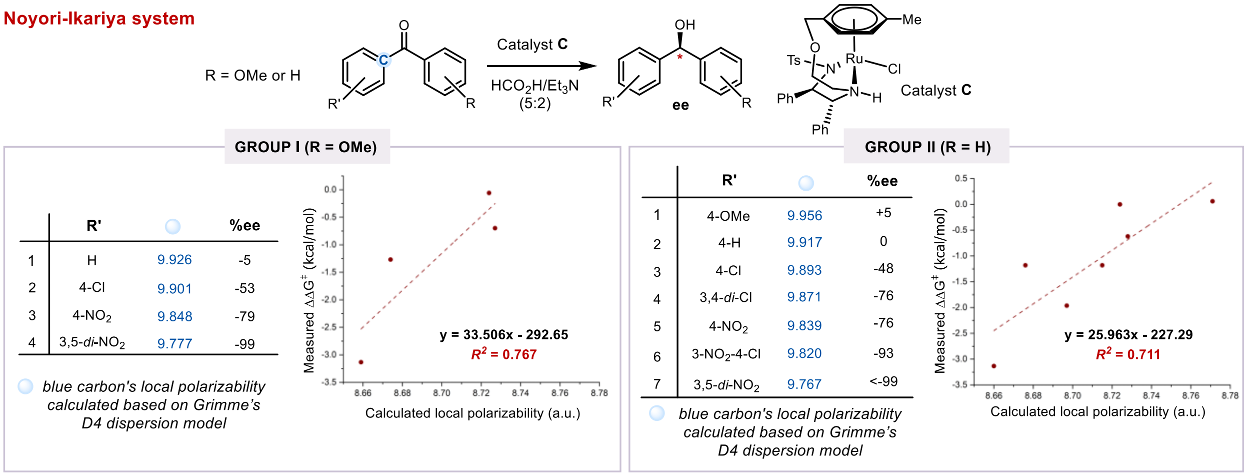

Supplementary Figure 56. Linear free energy relationship (LFER) analysis of substrate local polarizabilities calculated based on Grimme's D4 dispersion model on enantio-selections for the asymmetric transfer hydrogenation of di-aryl ketones by means of a Noyori-Ikariya catalyst.

3.3.2 Linear free energy relationship (LFER) studies with Hammett constants

Hammett constants have been also employed to establish linear free energy relationship with observed enantioselectivities (**Supplementary Figure 57**). For Noyori-Ikariya and Corey-Bakshi-Shibata system, the fine correlations are observed. But for Sharpless dihydroxylation system, the correlations dramatically decline due to the outlier value obtained by 3,5-*di*-OMe substituted substrate.

a. Noyori-Ikariya system

R = OMe or H

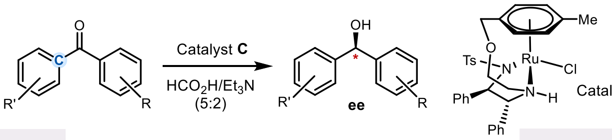

Catalyst **C**

GROUP I (R = OMe)

	R'	Sigma(σ)	%ee
1	H	0.00	-5
2	4-Cl	0.23	-53
3	4-NO_2	0.78	-79
4	3,5-*di*-NO_2	1.42	-99

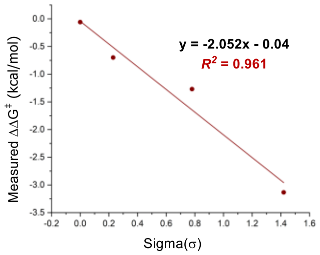

GROUP II (R = H)

	R'	Sigma(σ)	%ee
1	4-OMe	-0.27	+5
2	4-H	0.00	0
3	4-Cl	0.23	-48
4	3,4-*di*-Cl	0.60	-76
5	4-NO_2	0.78	-76
6	3-NO_2-4-Cl	0.94	-93
7	3,5-*di*-NO_2	1.42	<-99

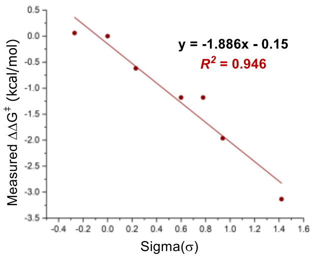

b. Corey-Bakshi-Shibata system

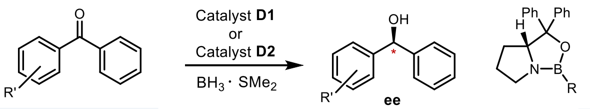

Catalyst **D1:** R = *n*Bu
Catalyst **D2:** R = Ph

GROUP I (Catalyst **D1**)

	R'	Sigma(σ)	%ee
1	4-OMe	-0.27	+49
2	4-Me	-0.17	+21
3	H	0.00	0
4	4-Cl	0.23	+5
5	4-Br	0.23	-8
6	4-CO_2Me	0.45	-26
7	4-CF_3	0.54	-35
8	4-NO_2	0.78	-41
9	3,5-*di*-CF_3	0.86	-56

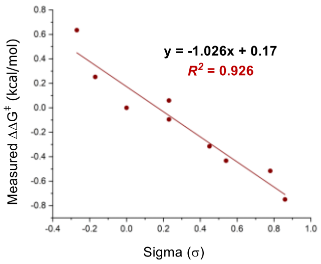

GROUP II (Catalyst **D2**)

	R'	Sigma(σ)	%ee
1	4-OMe	-0.27	+19
2	4-Me	-0.17	+10
3	H	0.00	0
4	4-Cl	0.23	-3
5	4-Br	0.23	-6
6	4-CO_2Me	0.45	-10
7	4-CF_3	0.54	-13
8	4-NO_2	0.78	-19
9	3,5-*di*-CF_3	0.86	-22

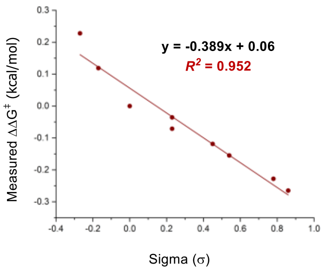

c. Sharpless dihydroxylation system

R = Me, H

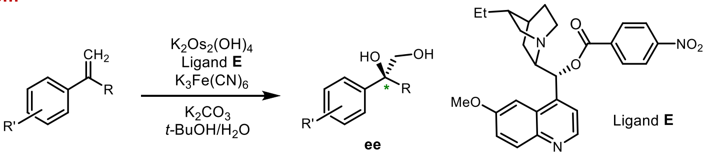

Ligand **E**

GROUP I (R = Me)

	R'	Sigma(σ)	%ee
1	3,5-*di*-OMe	0.24	72
2	4-OMe	-0.27	72
3	4-Me	-0.17	70
4	4-OPh	-0.03	67
5	4-Cl	0.23	63
6	4-Br	0.23	63
7	4-CO_2Me	0.45	62
8	4-NO_2	0.78	55
9	3,5-*di*-CF_3	0.86	36

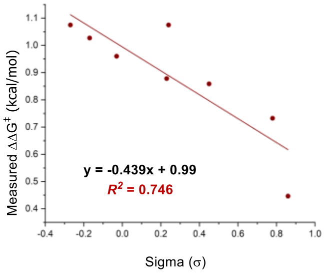

GROUP II (R = H)

	R'	Sigma(σ)	%ee
1	3,5-*di*-OMe	0.24	86
2	4-OMe	-0.27	81
3	4-Me	-0.17	80
4	4-OPh	-0.03	79
5	4-Cl	0.23	73
6	4-Br	0.23	72
7	4-CO_2Me	0.45	71
8	4-NO_2	0.78	62
9	3,5-*di*-CF_3	0.86	53

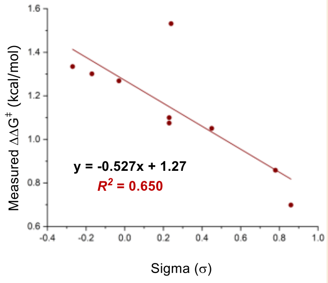

Supplementary Figure 57. The linear free energy relationship between Hammett constants and observed enantioselectivities for given reaction systems.

3.3.3 Linear free energy relationship (LFER) studies with whole ring polarizability and electrostatic potential

we also have evaluated the performances of the polarizability of the entire aryl ring and the electrostatic potential at a fixed distance above the ring on their correlations with stereoselectivity (**Supplementary Figure 58**). Taking CBS system as example, the results indicate that the electrostatic potential also has a good linear correlation with stereoselectivity (R^2 = 0.903), but the polarizability of the entire aryl ring does not (R^2 = 0.093).

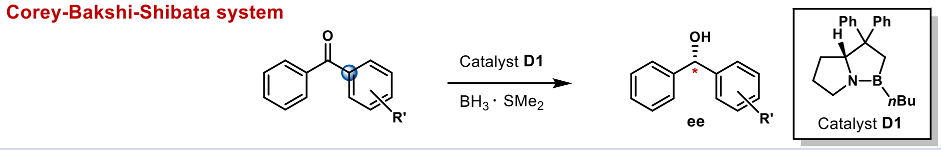

	R'	● (blue)	● (red)	%ee
1	4-OMe	86.77	0.1079	+49
2	4-Me	81.52	0.1129	+21
3	4-H	68.03	0.1180	0
4	4-Cl	82.10	0.1283	+5
5	4-Br	89.69	0.1299	-8
6	4-CO_2Me	100.59	0.1317	-26
7	4-CF_3	80.71	0.1392	-35
8	4-NO_2	84.70	0.1507	-41
9	3,5-*di*-CF_3	93.78	0.1552	-56

● (blue) *The polarizability including entire aryl ring*

● (red) *The electrostatic potential at one angstrom distance above the C atom*

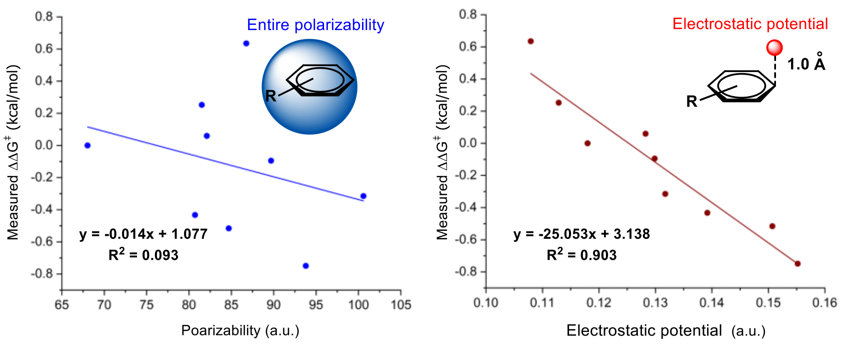

Supplementary Figure 58. The linear free energy relationship between whole ring polarizability or electrostatic potential and observed enantioselectivities for the CBS reduction with catalyst **D1**.

3.3.4 Density functional theory (DFT) calculations

In general, the stereoselectivities of catalytic asymmetric reactions are determined by the relative energies of the key transition states that lead to the corresponding stereoisomeric products. The origin of this energy difference is a combined result of various subtle interactions between the catalyst and the different approaching orientations of the substrate(s) in the stereo-determining transition states. To unravel what kinds of interactions are present in the catalytic systems that demonstrated qualitative/quantitative relationship between local polarizability and enantio-selection, we analyzed the corresponding transition states of the four reactions studied here (one representative substrate for each reaction) through density functional theory (DFT) calculations. The results are summarized in **Supplementary Figure 59**. The calculated differences in the relative free energies of activation ($\Delta\Delta G_{calc.}$) provide good agreement with those derived from experimentally measured enantioselectivities ($\Delta\Delta G_{expt.}$). The relative electronic energies of the transition states ($\Delta\Delta E_{calc.}$) were further dissected into the relative distortion energies of catalysts and substrates ($\Delta\Delta E_{dist\text{-}cat}$ and $\Delta\Delta E_{dist\text{-}sub}$, respectively), and the relative interaction energies between them ($\Delta\Delta E_{int}$) through distortion/interaction analysis. The results indicated a dominating role played by the interaction energies for the asymmetric transfer hydrogenation of ketone catalyzed by the Ru-catalyst (**TS1**/**TS1'**), CBS reduction of ketone (**TS3**/**TS3'**) and SAD reaction of alkene (**TS4**/**TS4'**). For the transfer hydrogenation catalyzed by the Noyori-Ikariya type catalyst (**TS2**/**TS2'**), while substrate distortion ($\Delta\Delta E_{dist\text{-}sub}$ = -0.6 kcal) also contributed to the relative destabilization of the higher-energy/disfavored transition state (**TS2'**), interaction energies ($\Delta\Delta E_{int}$ = -0.7 kcal) still played an important role in stabilizing of the lower-energy/favored transition state **TS2**. To further identify and visualize the key non-covalent interactions between the catalysts and the substrates, we performed the IGMH analysis of these transition states. The green color-coded maps shown in **Supplementary Figure 59** revealed three major types of non-covalent interactions present in these transition states, namely π-π, CH-π, and lone pair-π interactions, in accordance with previous studies on related systems. Specifically, for the asymmetric transfer hydrogenation reactions with two structurally distinct catalysts, π-π stacking interaction is solely present in **TS1**/**TS1'**, while CH-π and lone pair-π interactions are instead found in **TS2**/**TS2'**. For the CBS reduction of ketone by the *n*-butyl substituted oxazaborolidine catalyst, two sets of CH-π interactions are present in **TS3**/**TS3'**. For the SAD reaction, the CH-π interactions are uncovered in **TS4** and **TS4'**. The rich variety of weak interactions found in these transition states demonstrate the potential generality of using local polarizability as a simple determinant in constructing a qualitative or even a quantitative relationship with the sense and magnitude of enantio-selection, regardless of the exact types of weak interactions and their relative contributions to transition state energies in the course of chiral induction processes.

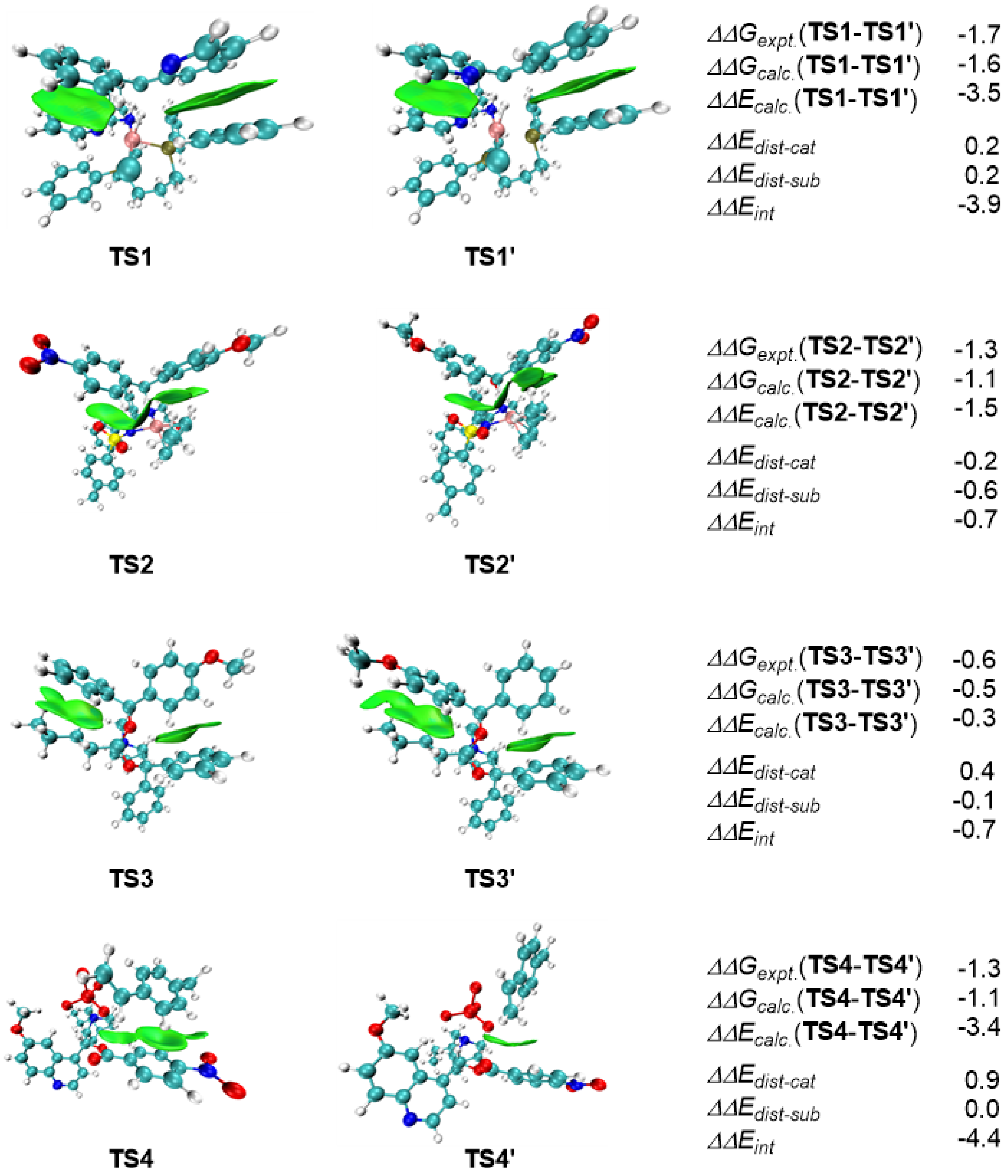

Supplementary Figure 59. DFT-computed transition state (TS) structures and their relative energies of four representative reactions in this study. **TS1-4** are the favored TSs that lead to the corresponding major enantiomer in each reaction, while **TS1'-4'** are the disfavored TSs that lead to the minor enantiomers accordingly. The distortion/interaction energies and IGMH analysis are also shown. Energy values are given in kcal/mol. The isosurface of IGMH analysis is 0.003.

Supplementary References

1. Chen, F., He, D., Chen, L., Chang, X., Wang, D. Z., Xu, C. & Xing, X. Chirality-Economy Catalysis: Asymmetric transfer hydrogenation of ketones by Ru-catalysts of minimal stereogenicity. *ACS Catal.* **9**, 5562–5566 (2019).
2. He, D., Xu, X., Lu, Y., Zhou, M. & Xing, X. Asymmetric transfer hydrogenation of densely functionalized diheteroaryl and diaryl ketones by a Ru-catalyst of minimal stereogenicity. *Org. Lett.* **22**, 8458–8463 (2020).
3. Chen, F. et al. Simultaneous access to two enantio-enriched alcohols by a single Ru-catalyst: asymmetric hydrogen transfer from racemic alcohols to matching ketones. *ACS Catal.* **12**, 14429 (2022).
4. Lassalas, P., Marsais, F. & Hoarau, C. DMAP-catalyzed regel-type direct C-2 (hetero)aroylation of oxazoles and thiazoles derivatives with acid chlorides. *Synlett* **24**, 2233-2240 (2013).
5. Yang, H., Wang, E., Yang, P., Lv, H. & Zhang, X. Pyridine-directed asymmetric hydrogenation of 1,1-diarylalkenes. *Org. Lett.* ***19***, 5062-5065 (2017).
6. Guanti, G. & Riva, R. Homochiral isoquinolines by lipase-catalysed resolution and their diastereoselective functionalization. *Tetrahedron: Asymmetry* **12**, 1185–1200 (2001).
7. Friel, D., Snapper, M. & Hoveyda, A. Aluminum-catalyzed asymmetric alkylations of pyridyl-substituted alkynyl ketones with dialkylzinc reagents. *J. Am. Chem. Soc.* **130**, 9942-9951 (2008).
8. Li, Y., Lai, M., Wu, Z., Zhao, M. & Zhang, M. Synthesis of 2-acyl-substituted pyrazine derivatives through silver-catalyzed decarboxylative coupling reactions. *ChemistrySelect* **3**, 5588-5592 (2018).
9. Tuluma, D., Amarnath, C. & Amitabha, S. Palladium catalyzed addition of arylboronic acid or indole to nitriles: synthesis of aryl ketones. *Tetrahedron Lett.* **55**, 7198-7202 (2014).
10. Schwerdtfegera, P. & Nagleb, J. K. 2018 Table of static dipole polarizabilities of the neutral elements in the periodic table. *Mol. Phys.* **117**, 1200-1225 (2019).
11. Grimme, S., Ehrlich, S. & Goerigk, L. Effect of the damping function in dispersion corrected density functional theory. *J. Comput. Chem*. **32**, 1456-1465 (2011).
12. Adamo, C. & Barone, V. Toward reliable density functional methods without adjustable parameters: The PBE0 model. *J. Chem. Phys*. **110**, 6158-6170 (1999).
13. Zhao, Y. & Truhlar, D. G. The M06 suite of density functionals for main group thermochemistry, thermochemical kinetics, noncovalent interactions, excited states, and transition elements: two new functionals and systematic testing of four M06-class functionals and 12 other functionals. *Theor. Chem. Acc*. **120**, 215-241 (2008).
14. Weigend, F. & Ahlrichs, R. Balanced basis sets of split valence, triple zeta valence and quadruple zeta valence quality for H to Rn: Design and assessment of accuracy. *Phys. Chem. Chem. Phys*. **7**, 3297-3305 (2005).

15. Weigend, F. Accurate Coulomb-fitting basis sets for H to Rn. *Phys. Chem. Chem. Phys*. **8**, 1057-1065 (2006).
16. Chai, J. D. & Head-Gordon, M. Long-range corrected hybrid density functionals with damped atom–atom dispersion corrections. *Phys. Chem. Chem. Phys*. **10**, 6615-6620 (2008).
17. Marenich, A. V., Cramer, C. J. & Truhlar, D. G. Universal Solvation Model Based on Solute Electron Density and on a Continuum Model of the Solvent Defined by the Bulk Dielectric Constant and Atomic Surface Tensions. *J. Phys. Chem. B*. **113**, 6378−6396 (2009).
18. Ribeiro, R. F., Marenich, A. V., Cramer, C. J. & Truhlar, D. G. Use of Solution-Phase Vibrational Frequencies in Continuum Models for the Free Energy of Solvation. *J. Phys. Chem. B*. **115**, 14556–14562 (2011).

NMR Spectra

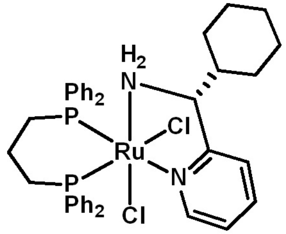

B

	Parameter	Value
1	Title	CFM-Cat.b-070301
2	Comment	
3	Origin	Bruker BioSpin GmbH
4	Owner	nmrsu
5	Site	
6	Spectrometer	AVANCE NEO 400 MHZ DIGITAL NMR SPECTROMETER
7	Author	
8	Solvent	CDCl3
9	Temperature	298.2
10	Pulse Sequence	zg30
11	Experiment	1D
12	Number of Scans	8
13	Receiver Gain	101
14	Spectrometer Frequency	400.13
15	Spectral Width	8196.7
16	Lowest Frequency	-1637.3
17	Nucleus	1H
18	Acquired Size	32768
19	Spectral Size	65536

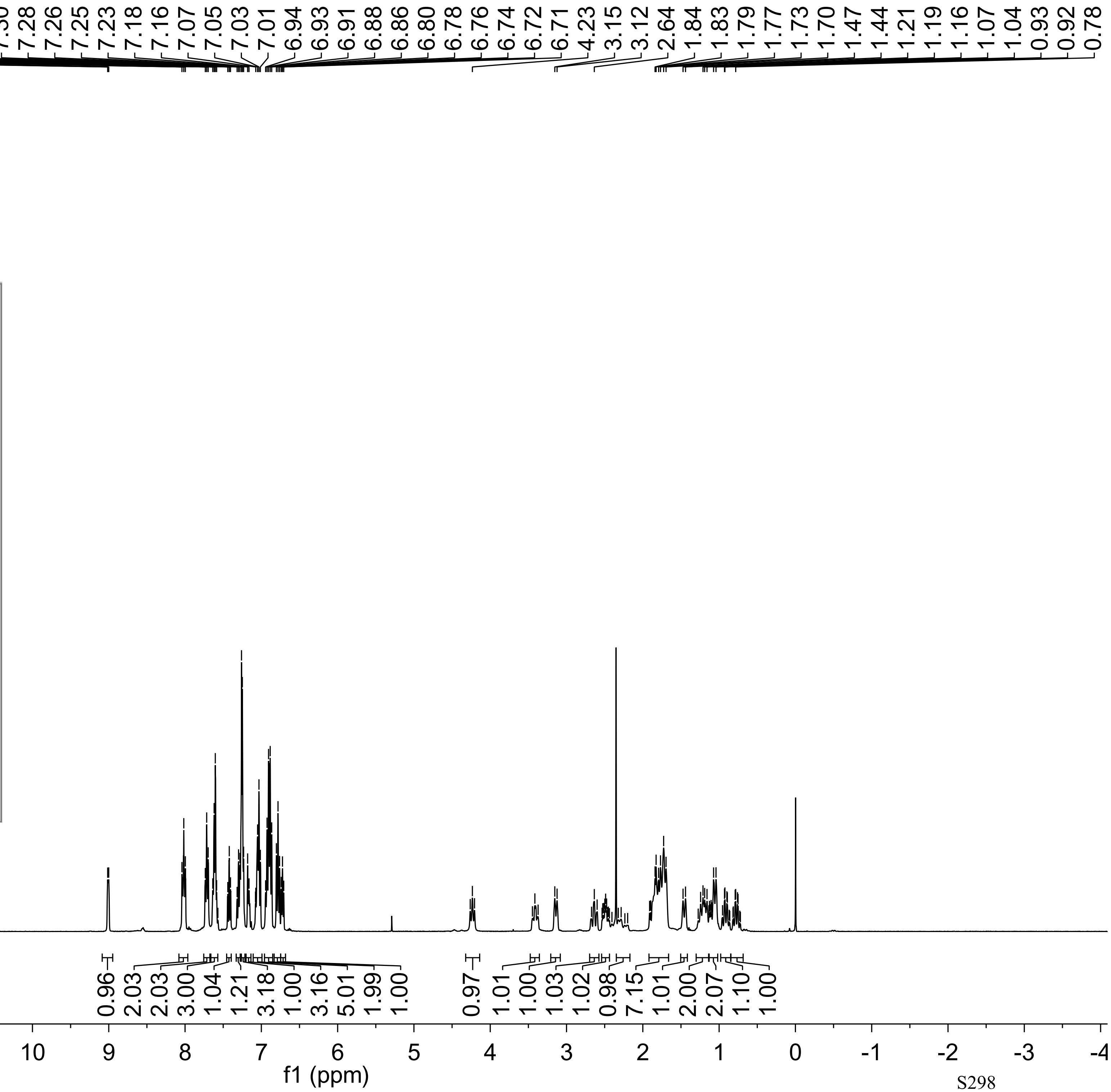

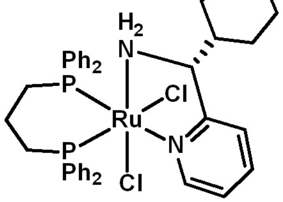

B

	Parameter	Value
1	Title	CFM-Cat.b-070301
2	Comment	
3	Origin	Bruker BioSpin GmbH
4	Owner	nmrsu
5	Site	
6	Spectrometer	AVANCE NEO 400 MHZ DIGITAL NMR SPECTROMETER
7	Author	
8	Solvent	CDCl3
9	Temperature	298.2
10	Pulse Sequence	zgpg30
11	Experiment	1D
12	Number of Scans	1352
13	Receiver Gain	62
14	Spectrometer Frequency	100.61
15	Spectral Width	23809.5
16	Lowest Frequency	-1848.2
17	Nucleus	13C
18	Acquired Size	32768
19	Spectral Size	32768

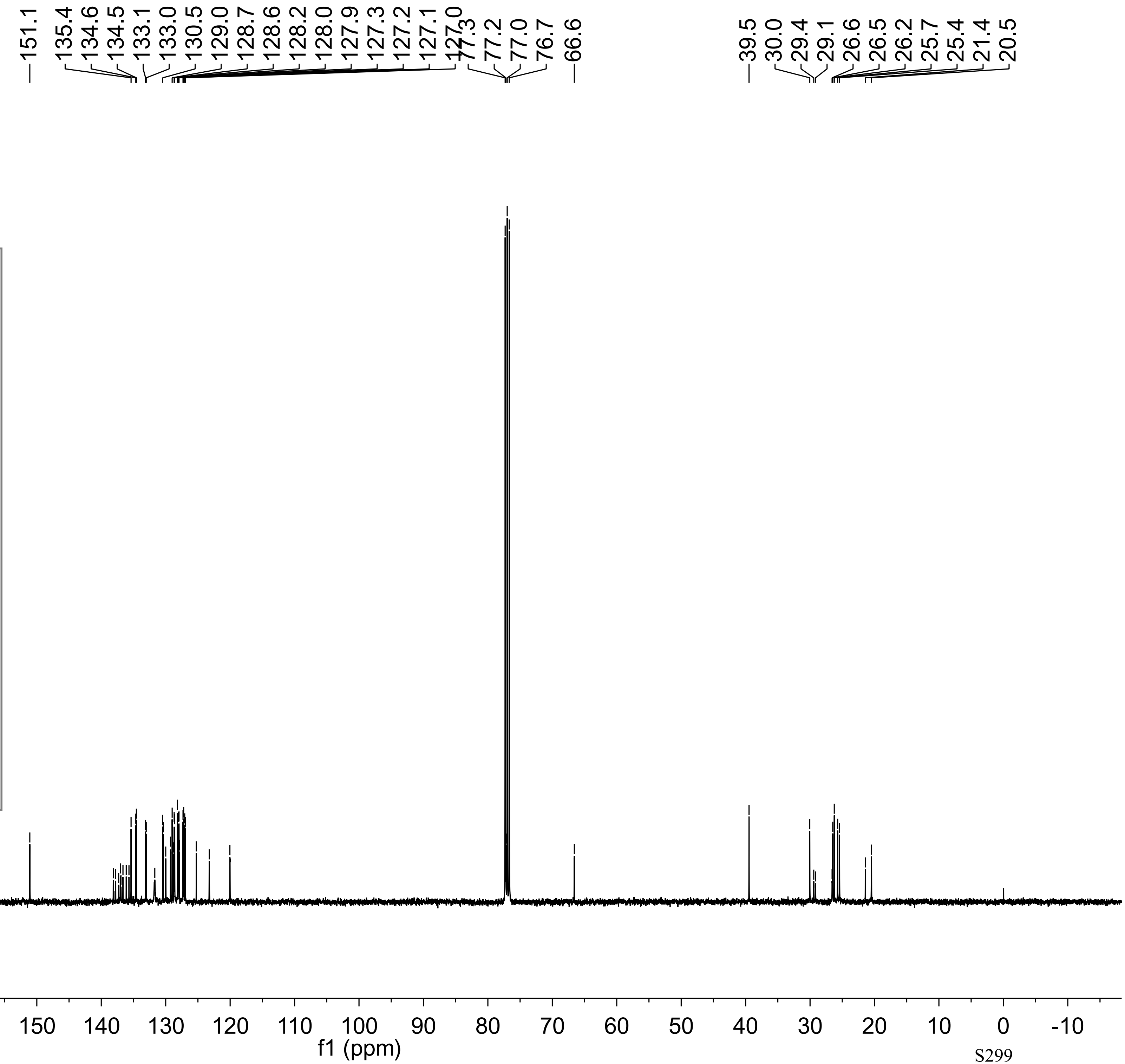

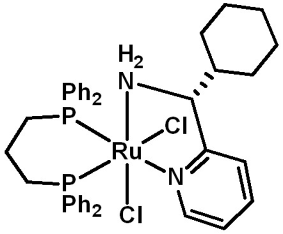

B

Parameter	Value
1 Title	CFM-Cat.b-070301
2 Comment	
3 Origin	Bruker BioSpin GmbH
4 Owner	nmrsu
5 Site	
6 Spectrometer	AVANCE NEO 400 MHZ DIGITAL NMR SPECTROMETER
7 Author	
8 Solvent	CDCl3
9 Temperature	298.2
10 Pulse Sequence	zgpg30
11 Experiment	1D
12 Number of Scans	16
13 Receiver Gain	101
14 Spectrometer Frequency	161.98
15 Spectral Width	65789.5
16 Lowest Frequency	-40993.5
17 Nucleus	31P
18 Acquired Size	32768
19 Spectral Size	32768

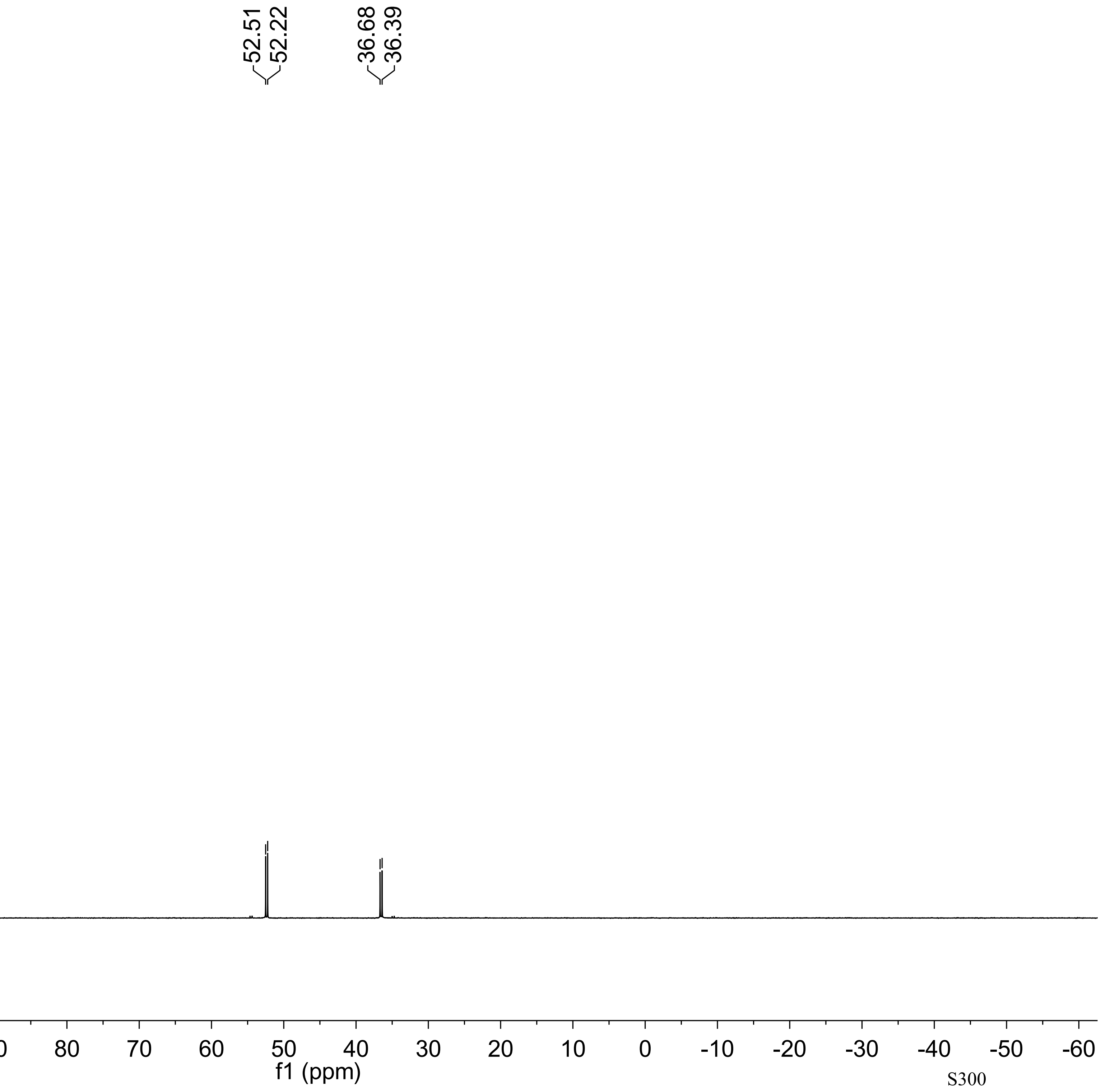

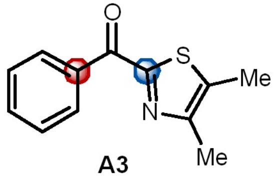

A3

Parameter	Value
1 Title	CFM-C1
2 Comment	
3 Origin	Bruker BioSpin GmbH
4 Owner	nmrsu
5 Site	
6 Spectrometer	Avance NEO 600
7 Author	
8 Solvent	CDCl3
9 Temperature	296.9
10 Pulse Sequence	zg30
11 Experiment	1D
12 Number of Scans	16
13 Receiver Gain	101
14 Relaxation Delay	1.0000
15 Pulse Width	10.0000
16 Acquisition Time	2.7525
17 Acquisition Date	2019-12-09T23:49:47
18 Modification Date	2019-12-10T11:38:11
19 Spectrometer Frequency	600.15
20 Spectral Width	11904.8
21 Lowest Frequency	-2261.1
22 Nucleus	1H
23 Acquired Size	32768
24 Spectral Size	65536

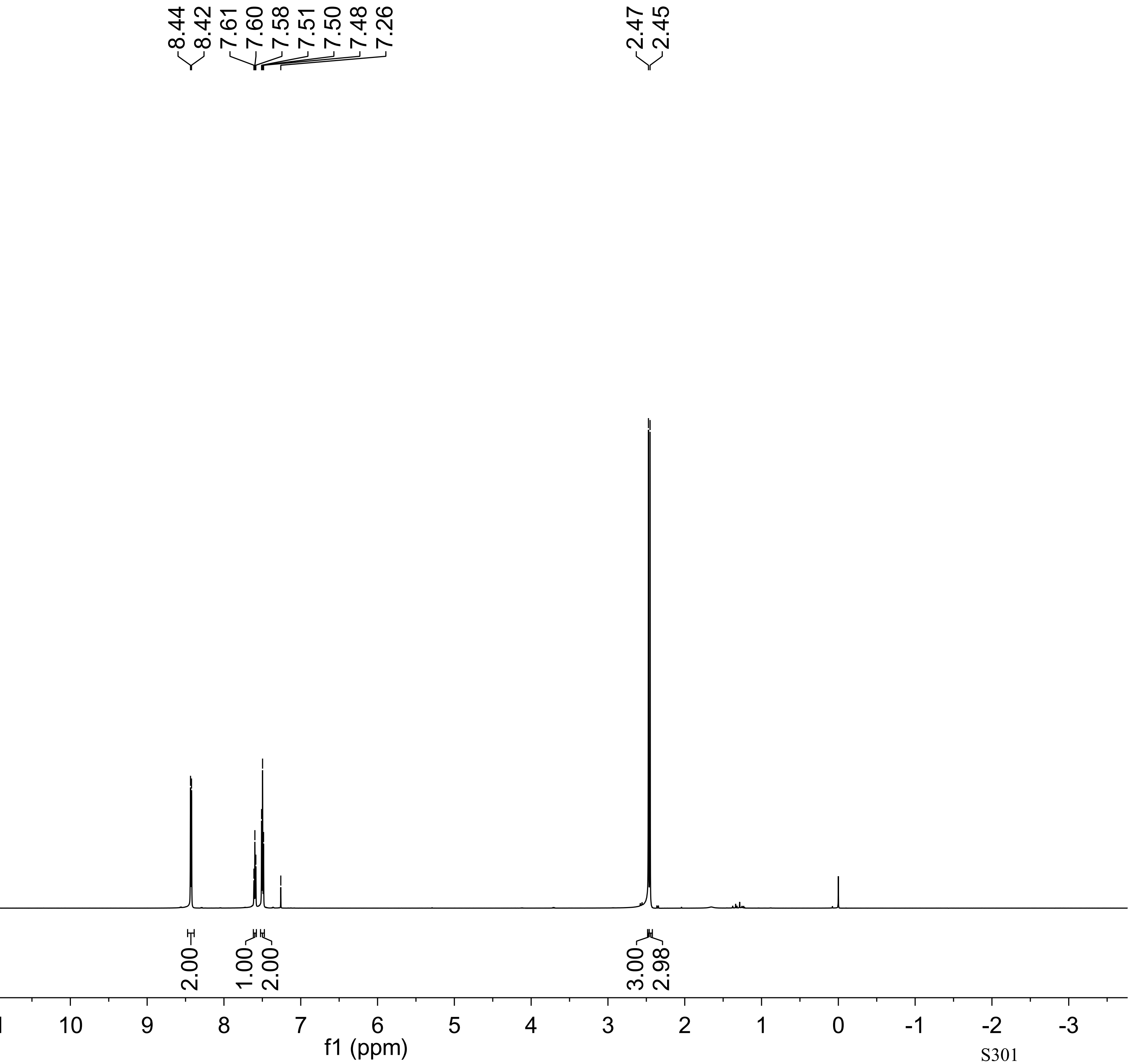

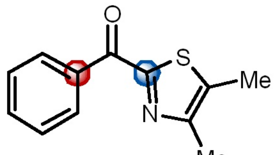

A3

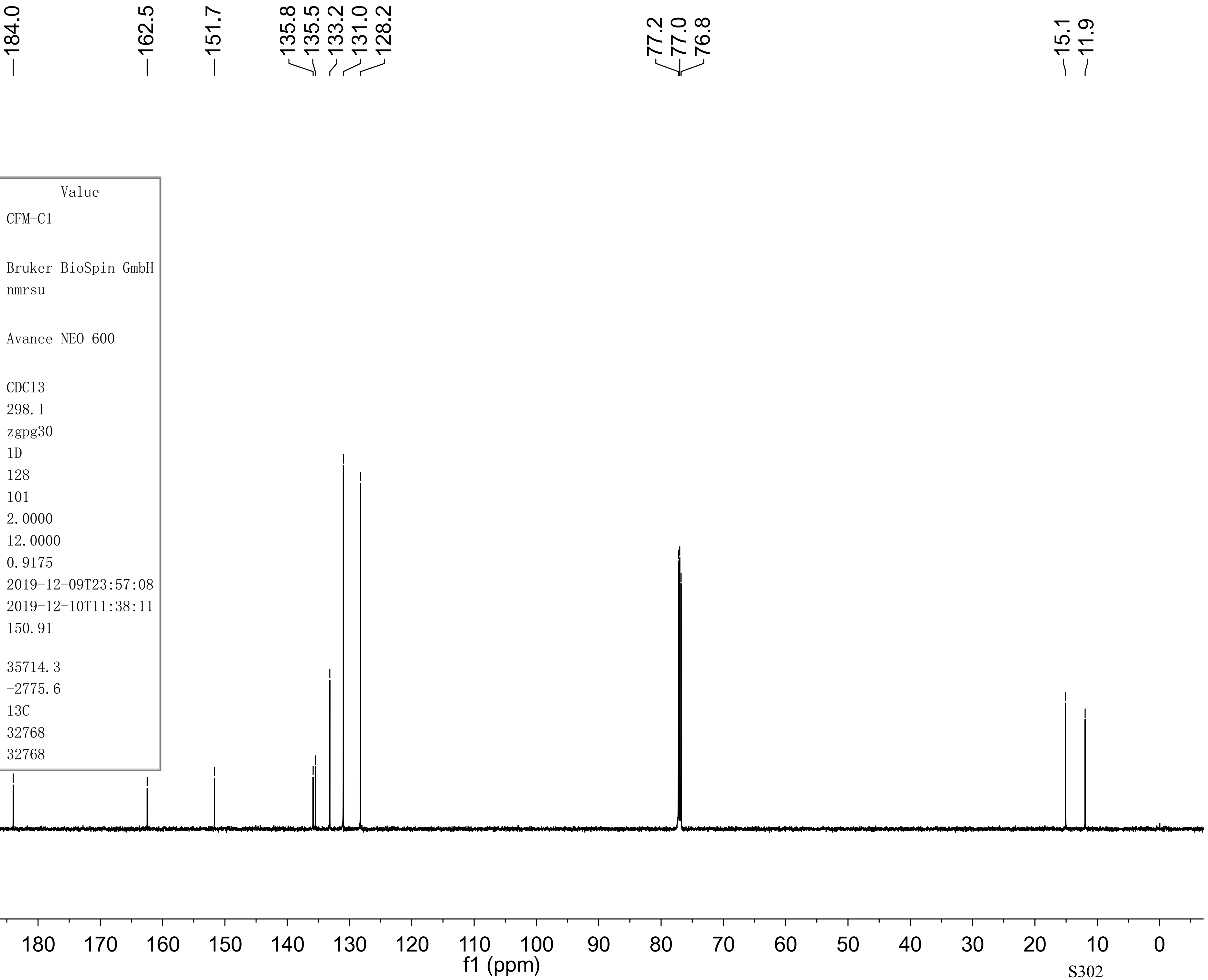

	Parameter	Value
1	Title	CFM-C1
2	Comment	
3	Origin	Bruker BioSpin GmbH
4	Owner	nmrsu
5	Site	
6	Spectrometer	Avance NEO 600
7	Author	
8	Solvent	CDCl3
9	Temperature	298.1
10	Pulse Sequence	zgpg30
11	Experiment	1D
12	Number of Scans	128
13	Receiver Gain	101
14	Relaxation Delay	2.0000
15	Pulse Width	12.0000
16	Acquisition Time	0.9175
17	Acquisition Date	2019-12-09T23:57:08
18	Modification Date	2019-12-10T11:38:11
19	Spectrometer Frequency	150.91
20	Spectral Width	35714.3
21	Lowest Frequency	-2775.6
22	Nucleus	13C
23	Acquired Size	32768
24	Spectral Size	32768

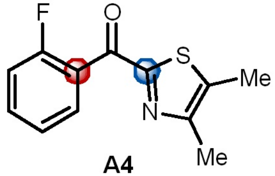

A4

Parameter	Value
1 Title	CFM-C2
2 Comment	
3 Origin	Bruker BioSpin GmbH
4 Owner	nmrsu
5 Site	
6 Spectrometer	Avance NEO 600
7 Author	
8 Solvent	CDCl3
9 Temperature	296.9
10 Pulse Sequence	zg30
11 Experiment	1D
12 Number of Scans	16
13 Receiver Gain	101
14 Relaxation Delay	1.0000
15 Pulse Width	10.0000
16 Acquisition Time	2.7525
17 Acquisition Date	2019-12-10T00:02:04
18 Modification Date	2019-12-10T11:38:10
19 Spectrometer Frequency	600.15
20 Spectral Width	11904.8
21 Lowest Frequency	-2258.8
22 Nucleus	1H
23 Acquired Size	32768
24 Spectral Size	65536

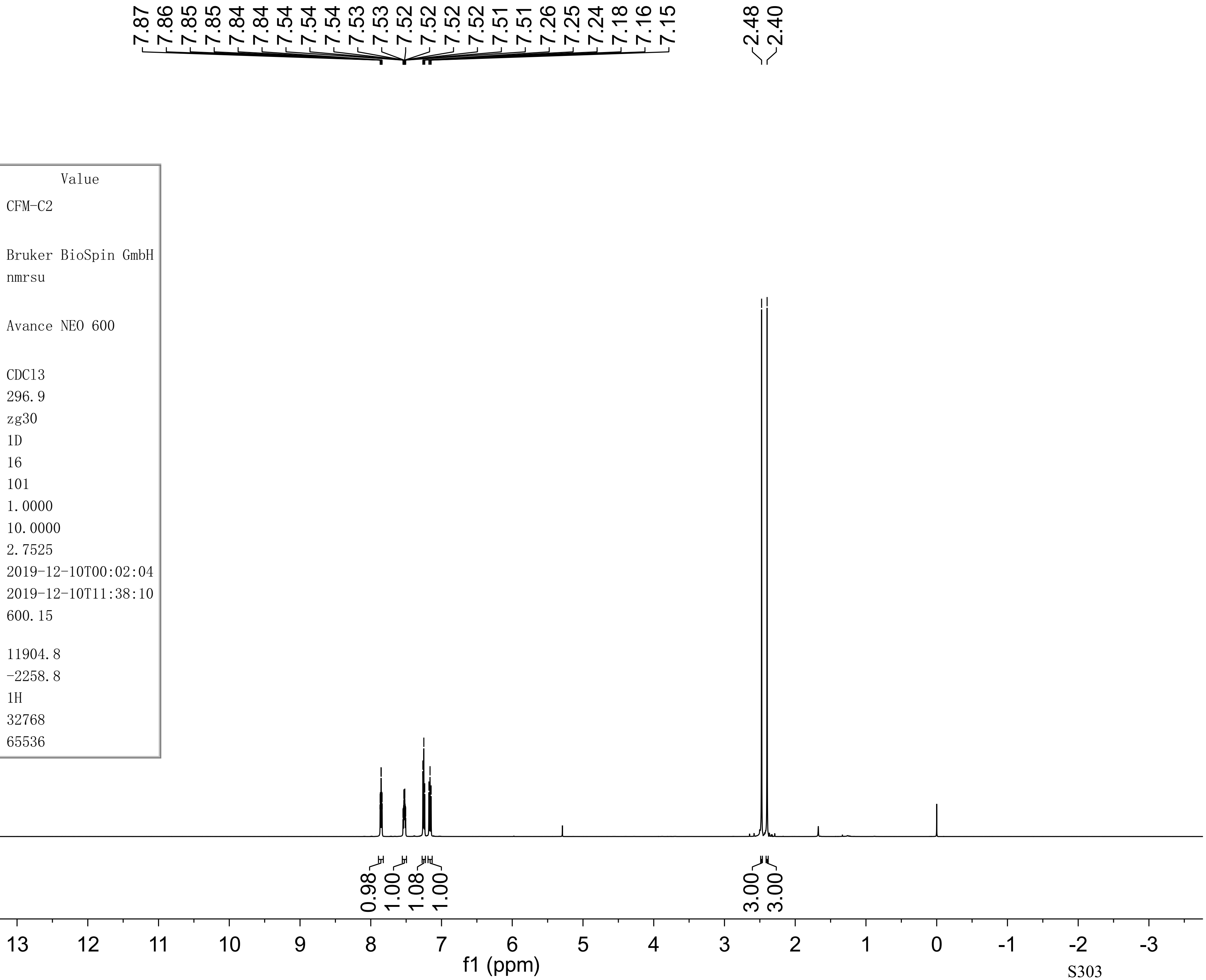

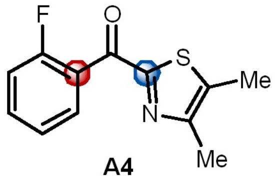

A4

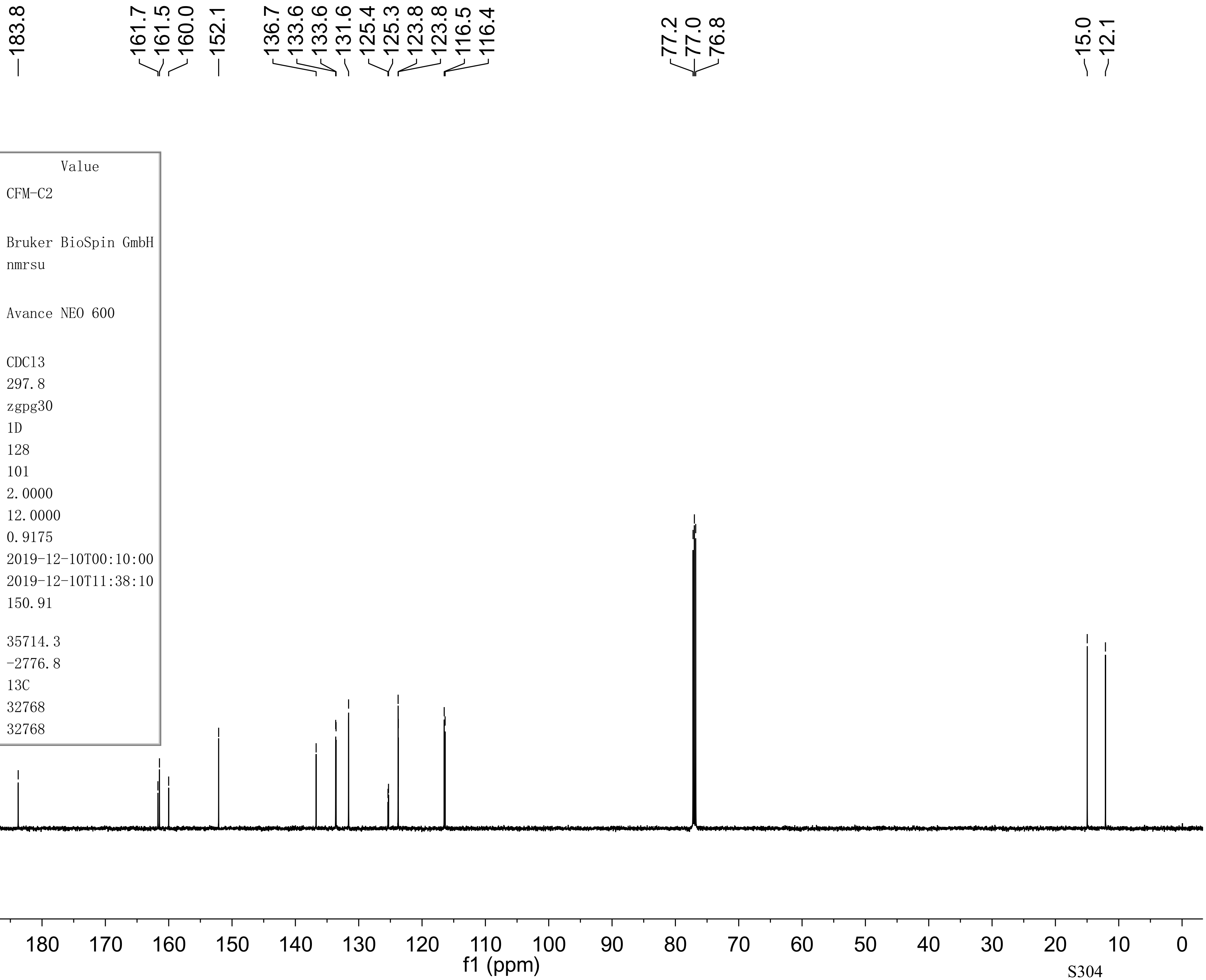

	Parameter	Value
1	Title	CFM-C2
2	Comment	
3	Origin	Bruker BioSpin GmbH
4	Owner	nmrsu
5	Site	
6	Spectrometer	Avance NEO 600
7	Author	
8	Solvent	CDCl3
9	Temperature	297.8
10	Pulse Sequence	zgpg30
11	Experiment	1D
12	Number of Scans	128
13	Receiver Gain	101
14	Relaxation Delay	2.0000
15	Pulse Width	12.0000
16	Acquisition Time	0.9175
17	Acquisition Date	2019-12-10T00:10:00
18	Modification Date	2019-12-10T11:38:10
19	Spectrometer Frequency	150.91
20	Spectral Width	35714.3
21	Lowest Frequency	-2776.8
22	Nucleus	13C
23	Acquired Size	32768
24	Spectral Size	32768

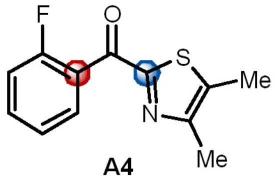

A4

Parameter	Value
1 Title	CFM-C2
2 Comment	
3 Origin	Bruker BioSpin GmbH
4 Owner	nmrsu
5 Site	
6 Spectrometer	Avance NEO 600
7 Author	
8 Solvent	CDCl3
9 Temperature	297.1
10 Pulse Sequence	zg
11 Experiment	1D
12 Number of Scans	16
13 Receiver Gain	101
14 Relaxation Delay	1.0000
15 Pulse Width	12.0000
16 Acquisition Time	0.4981
17 Acquisition Date	2019-12-10T00:11:31
18 Modification Date	2019-12-10T11:38:10
19 Spectrometer Frequency	564.71
20 Spectral Width	131579.0
21 Lowest Frequency	-122260.0
22 Nucleus	19F
23 Acquired Size	65536
24 Spectral Size	65536

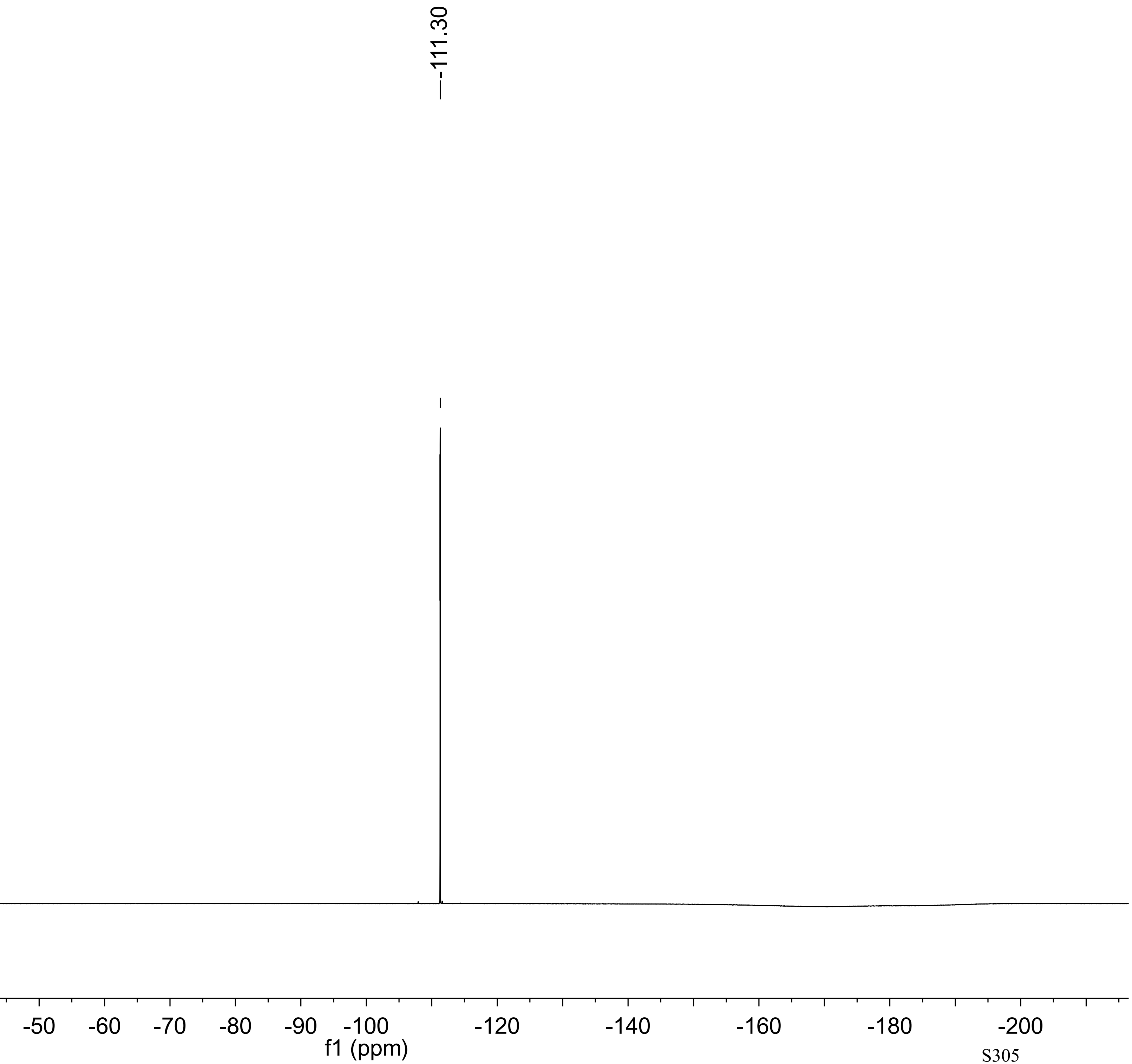

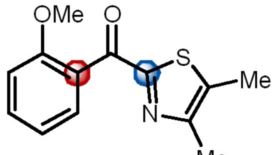

A5

Parameter	Value
1 Title	CFM-C3
2 Comment	
3 Origin	Bruker BioSpin GmbH
4 Owner	nmrsu
5 Site	
6 Spectrometer	Avance NEO 600
7 Author	
8 Solvent	CDCl3
9 Temperature	296.8
10 Pulse Sequence	zg30
11 Experiment	1D
12 Number of Scans	16
13 Receiver Gain	100
14 Relaxation Delay	1.0000
15 Pulse Width	10.0000
16 Acquisition Time	2.7525
17 Acquisition Date	2019-12-10T00:16:34
18 Modification Date	2019-12-10T11:38:12
19 Spectrometer Frequency	600.15
20 Spectral Width	11904.8
21 Lowest Frequency	-2261.1
22 Nucleus	1H
23 Acquired Size	32768
24 Spectral Size	65536

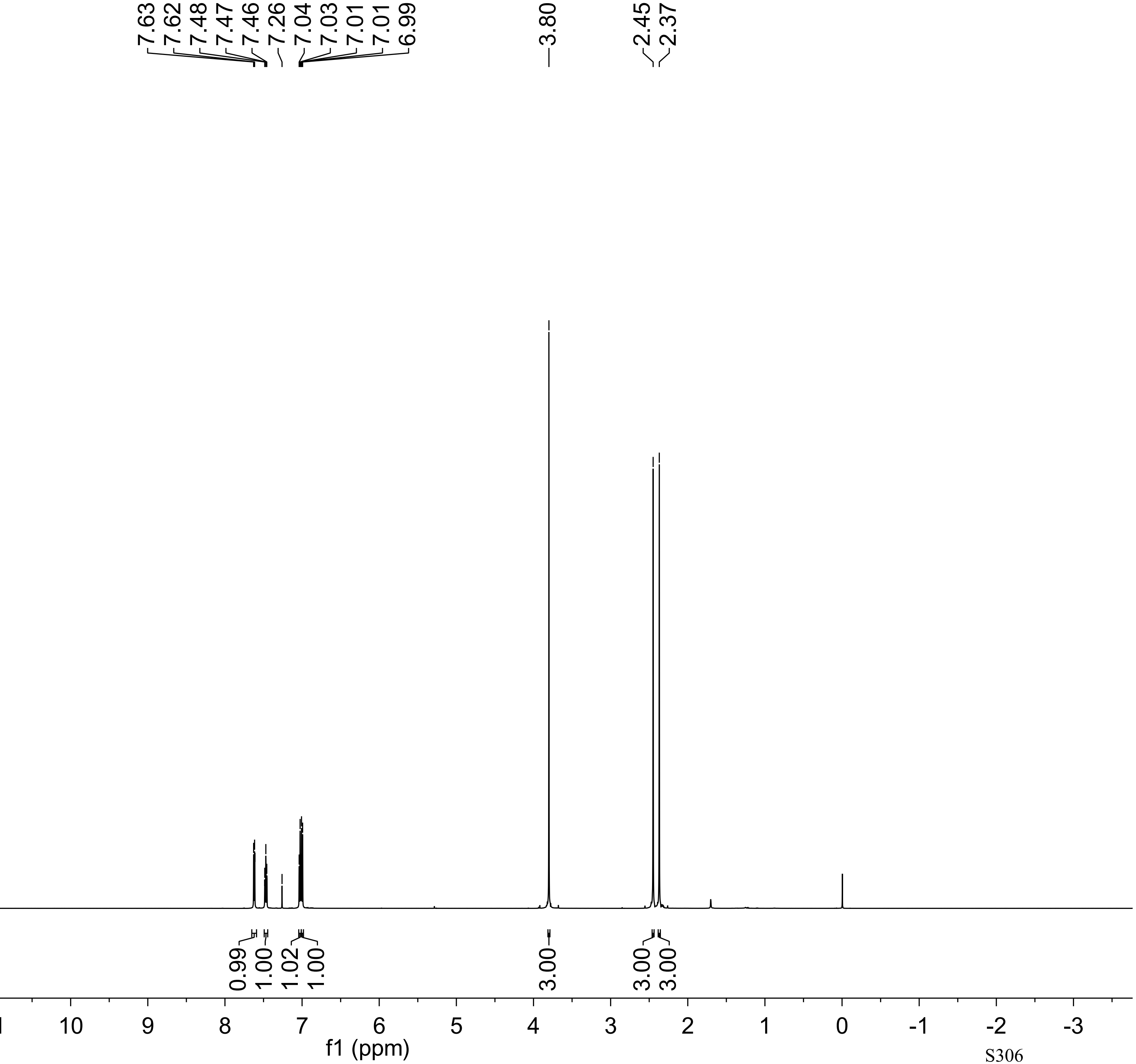

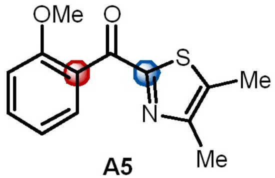

A5

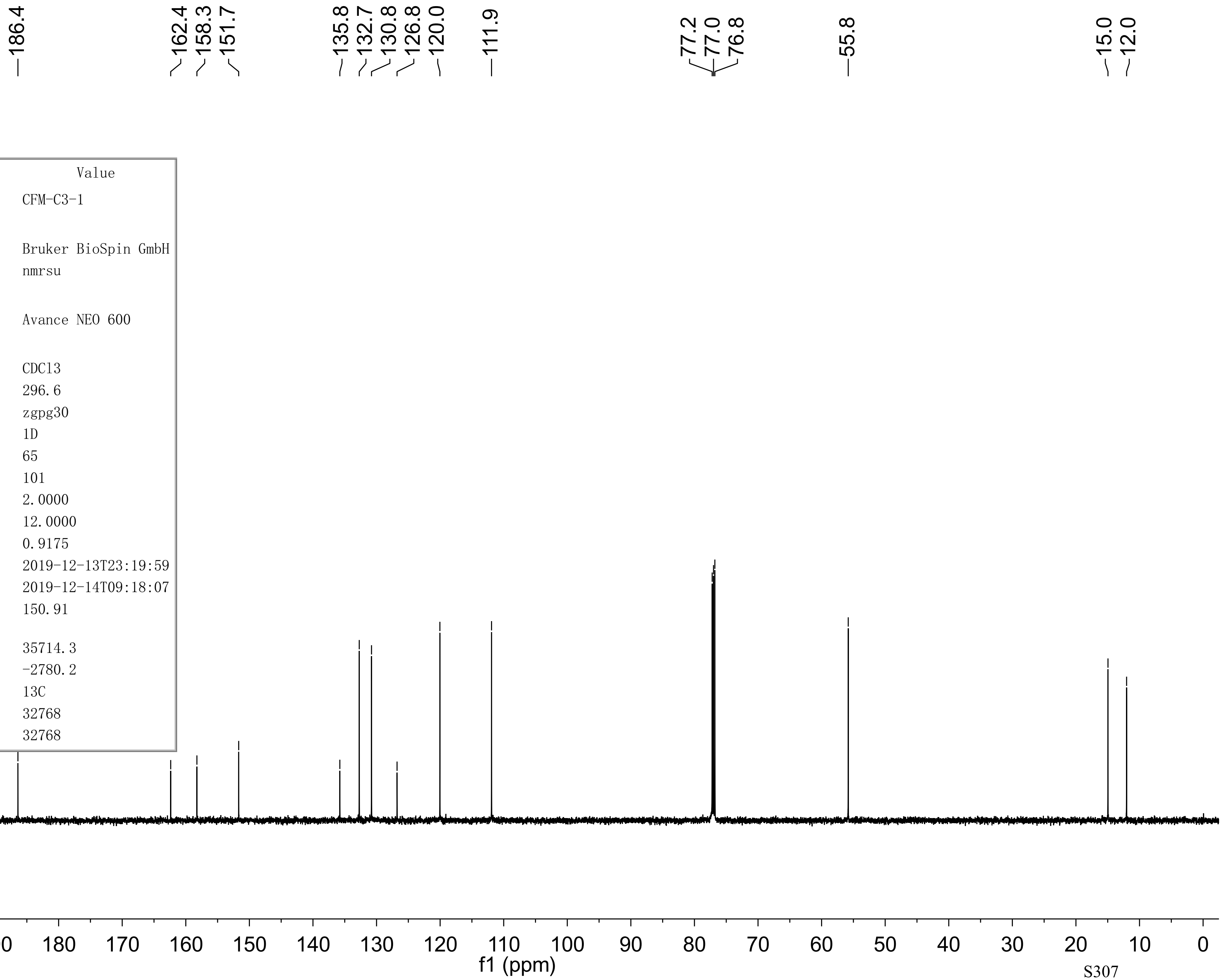

Parameter	Value
1 Title	CFM-C3-1
2 Comment	
3 Origin	Bruker BioSpin GmbH
4 Owner	nmrsu
5 Site	
6 Spectrometer	Avance NEO 600
7 Author	
8 Solvent	CDCl3
9 Temperature	296.6
10 Pulse Sequence	zgpg30
11 Experiment	1D
12 Number of Scans	65
13 Receiver Gain	101
14 Relaxation Delay	2.0000
15 Pulse Width	12.0000
16 Acquisition Time	0.9175
17 Acquisition Date	2019-12-13T23:19:59
18 Modification Date	2019-12-14T09:18:07
19 Spectrometer Frequency	150.91
20 Spectral Width	35714.3
21 Lowest Frequency	-2780.2
22 Nucleus	13C
23 Acquired Size	32768
24 Spectral Size	32768

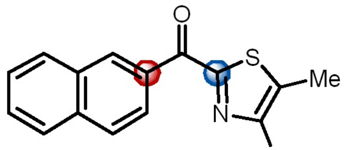

A6

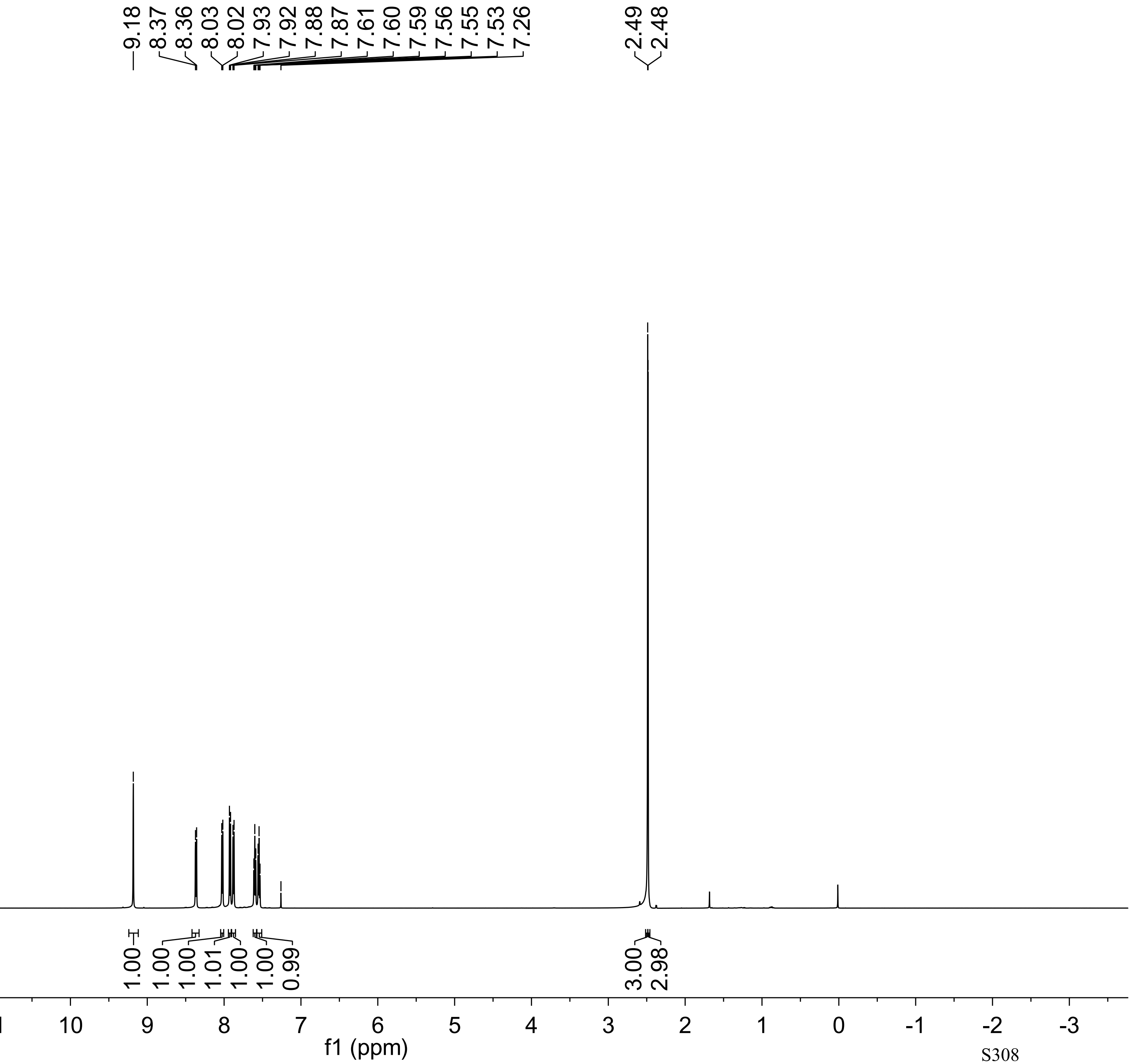

	Parameter	Value
1	Title	CFM-C4
2	Comment	
3	Origin	Bruker BioSpin GmbH
4	Owner	nmrsu
5	Site	
6	Spectrometer	Avance NEO 600
7	Author	
8	Solvent	CDCl3
9	Temperature	296.4
10	Pulse Sequence	zg30
11	Experiment	1D
12	Number of Scans	16
13	Receiver Gain	83
14	Relaxation Delay	1.0000
15	Pulse Width	10.0000
16	Acquisition Time	2.7525
17	Acquisition Date	2019-12-10T00:29:47
18	Modification Date	2019-12-10T11:38:11
19	Spectrometer Frequency	600.15
20	Spectral Width	11904.8
21	Lowest Frequency	-2260.8
22	Nucleus	1H
23	Acquired Size	32768
24	Spectral Size	65536

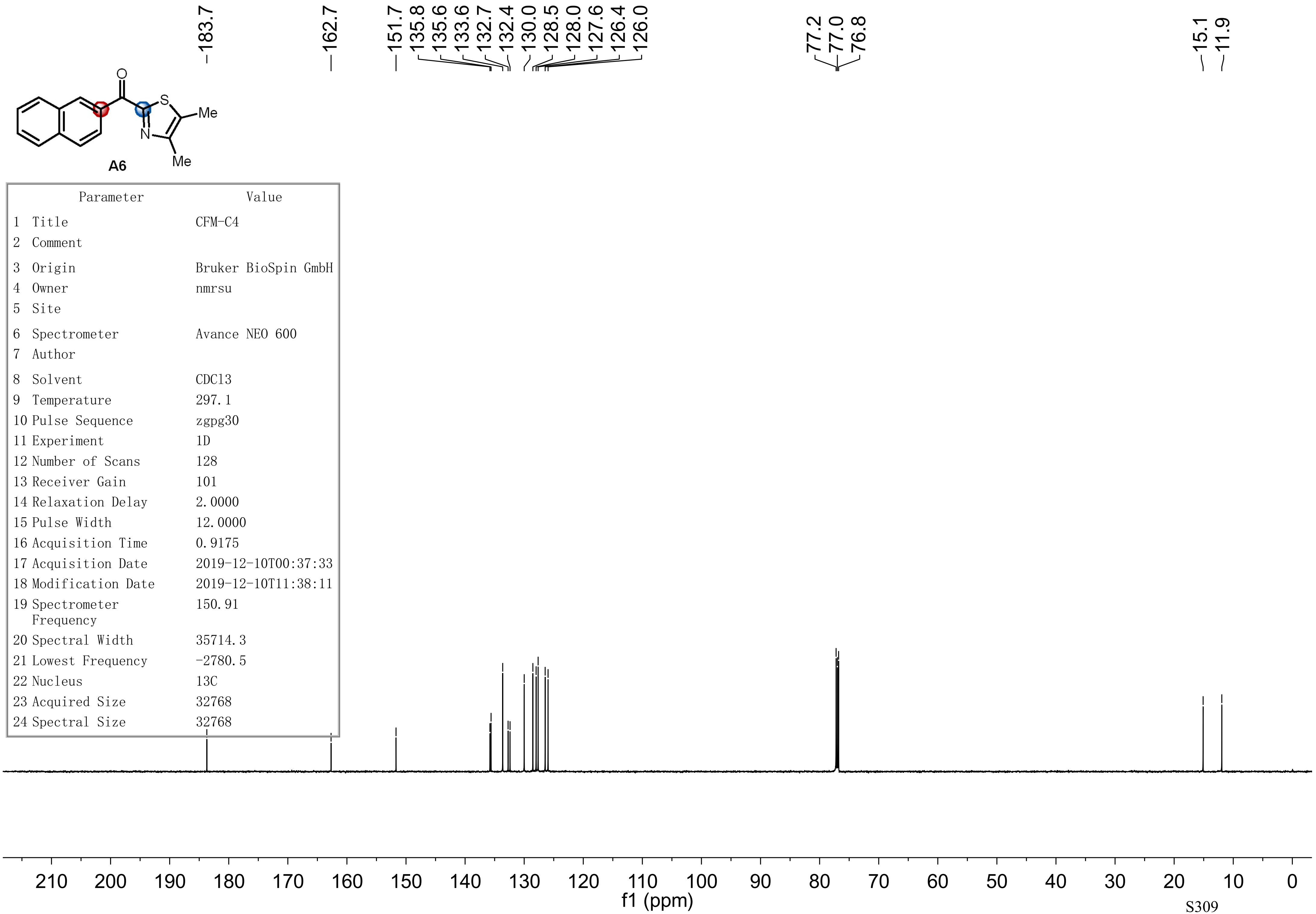

	Parameter	Value
1	Title	CFM-C4
2	Comment	
3	Origin	Bruker BioSpin GmbH
4	Owner	nmrsu
5	Site	
6	Spectrometer	Avance NEO 600
7	Author	
8	Solvent	CDCl3
9	Temperature	297.1
10	Pulse Sequence	zgpg30
11	Experiment	1D
12	Number of Scans	128
13	Receiver Gain	101
14	Relaxation Delay	2.0000
15	Pulse Width	12.0000
16	Acquisition Time	0.9175
17	Acquisition Date	2019-12-10T00:37:33
18	Modification Date	2019-12-10T11:38:11
19	Spectrometer Frequency	150.91
20	Spectral Width	35714.3
21	Lowest Frequency	-2780.5
22	Nucleus	13C
23	Acquired Size	32768
24	Spectral Size	32768

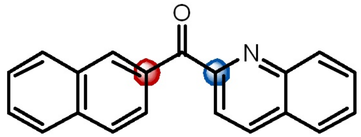

A7

Parameter	Value
1 Title	CFM-E9
2 Comment	
3 Origin	Bruker BioSpin GmbH
4 Owner	nmrsu
5 Site	
6 Spectrometer	Avance NEO 600
7 Author	
8 Solvent	CDCl3
9 Temperature	297.0
10 Pulse Sequence	zg30
11 Experiment	1D
12 Number of Scans	16
13 Receiver Gain	101
14 Relaxation Delay	1.0000
15 Pulse Width	10.0000
16 Acquisition Time	2.7525
17 Acquisition Date	2019-12-10T09:13:13
18 Modification Date	2019-12-10T11:38:48
19 Spectrometer Frequency	600.15
20 Spectral Width	11904.8
21 Lowest Frequency	-2261.2
22 Nucleus	1H
23 Acquired Size	32768
24 Spectral Size	65536

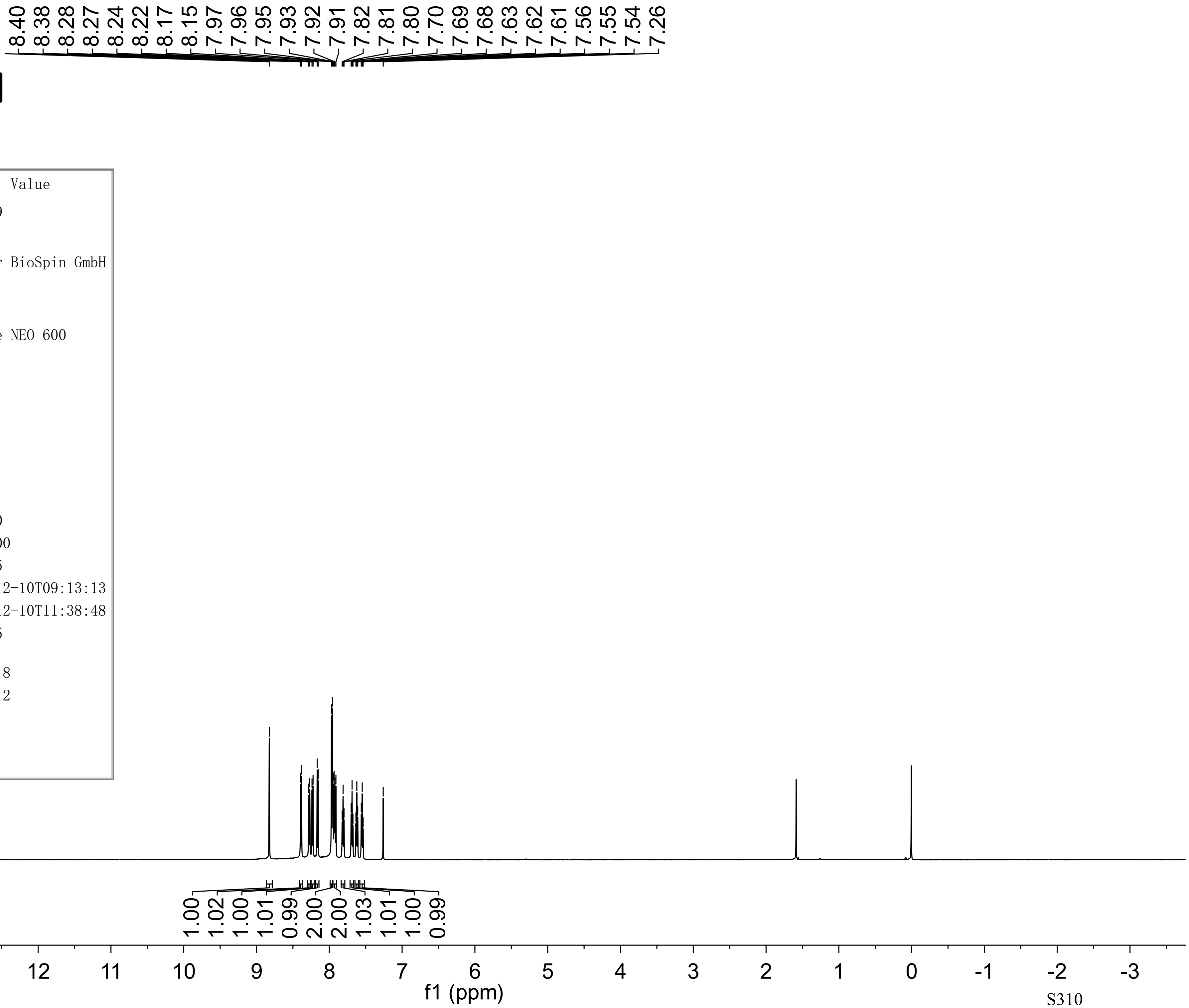

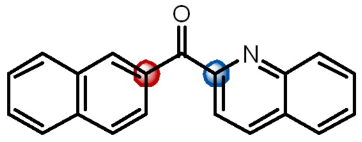

A7

Parameter	Value
1 Title	CFM-E9
2 Comment	
3 Origin	Bruker BioSpin GmbH
4 Owner	nmrsu
5 Site	
6 Spectrometer	Avance NEO 600
7 Author	
8 Solvent	CDCl3
9 Temperature	298.2
10 Pulse Sequence	zgpg30
11 Experiment	1D
12 Number of Scans	128
13 Receiver Gain	101
14 Relaxation Delay	2.0000
15 Pulse Width	12.0000
16 Acquisition Time	0.9175
17 Acquisition Date	2019-12-10T09:20:29
18 Modification Date	2019-12-10T11:38:48
19 Spectrometer Frequency	150.91
20 Spectral Width	35714.3
21 Lowest Frequency	-2772.2
22 Nucleus	13C
23 Acquired Size	32768
24 Spectral Size	32768

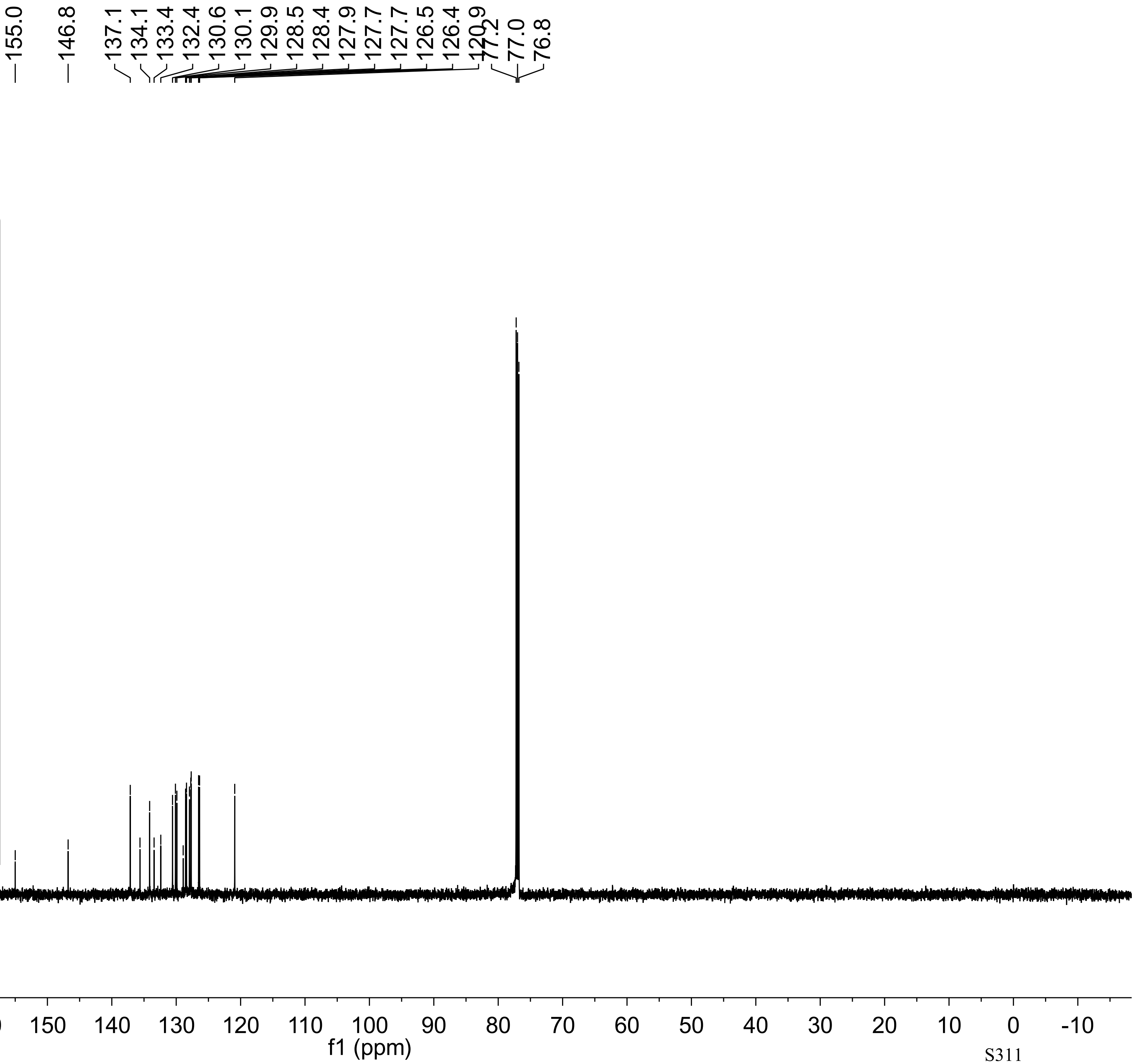

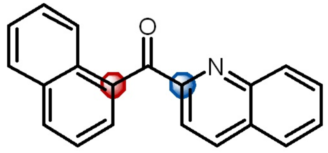

A8

Parameter	Value
1 Title	CFM-E10
2 Comment	
3 Origin	Bruker BioSpin GmbH
4 Owner	nmrsu
5 Site	
6 Spectrometer	Avance NEO 600
7 Author	
8 Solvent	CDCl3
9 Temperature	297.9
10 Pulse Sequence	zg30
11 Experiment	1D
12 Number of Scans	16
13 Receiver Gain	101
14 Relaxation Delay	1.0000
15 Pulse Width	10.0000
16 Acquisition Time	2.7525
17 Acquisition Date	2019-12-30T08:10:41
18 Modification Date	2019-12-30T09:23:56
19 Spectrometer Frequency	600.15
20 Spectral Width	11904.8
21 Lowest Frequency	-2261.3
22 Nucleus	1H
23 Acquired Size	32768
24 Spectral Size	65536

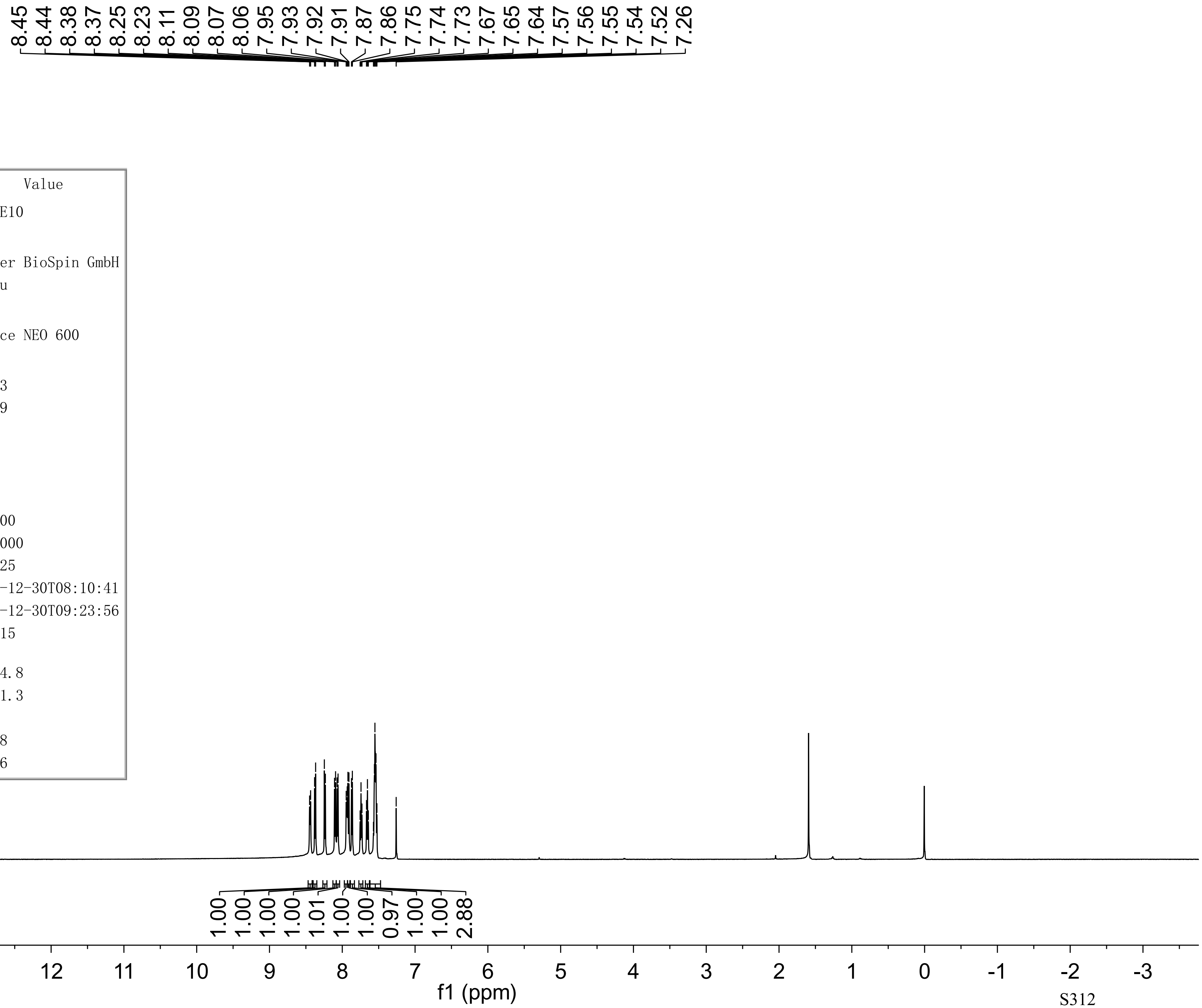

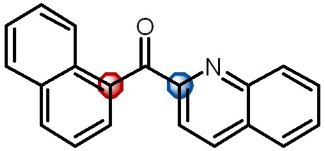

A8

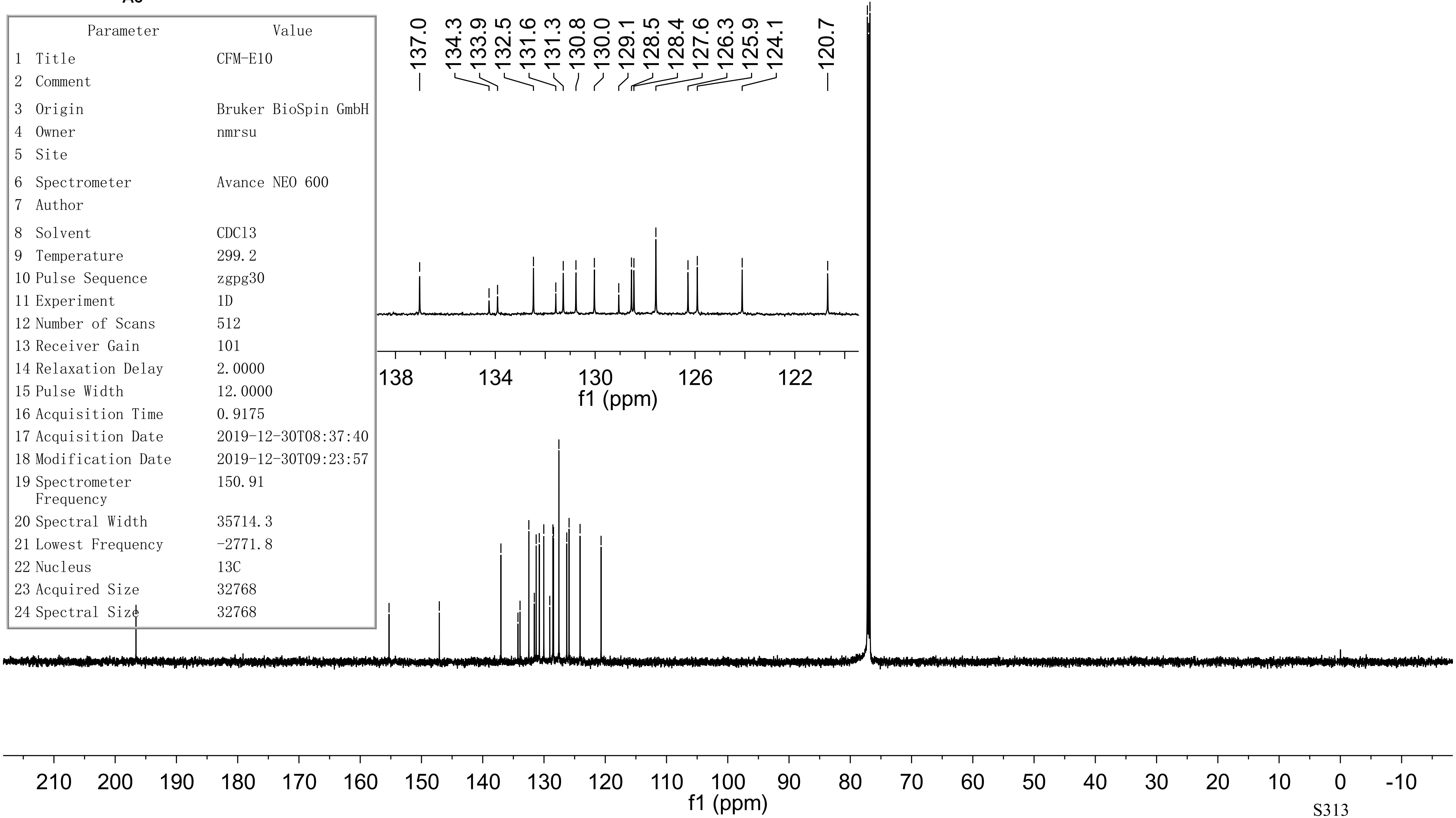

	Parameter	Value
1	Title	CFM-E10
2	Comment	
3	Origin	Bruker BioSpin GmbH
4	Owner	nmrsu
5	Site	
6	Spectrometer	Avance NEO 600
7	Author	
8	Solvent	CDCl3
9	Temperature	299.2
10	Pulse Sequence	zgpg30
11	Experiment	1D
12	Number of Scans	512
13	Receiver Gain	101
14	Relaxation Delay	2.0000
15	Pulse Width	12.0000
16	Acquisition Time	0.9175
17	Acquisition Date	2019-12-30T08:37:40
18	Modification Date	2019-12-30T09:23:57
19	Spectrometer Frequency	150.91
20	Spectral Width	35714.3
21	Lowest Frequency	-2771.8
22	Nucleus	13C
23	Acquired Size	32768
24	Spectral Size	32768

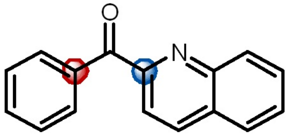

A9

Parameter	Value
1 Title	CFM-E1
2 Comment	
3 Origin	Bruker BioSpin GmbH
4 Owner	nmrsu
5 Site	
6 Spectrometer	Avance NEO 600
7 Author	
8 Solvent	CDCl3
9 Temperature	297.2
10 Pulse Sequence	zg30
11 Experiment	1D
12 Number of Scans	16
13 Receiver Gain	101
14 Relaxation Delay	1.0000
15 Pulse Width	10.0000
16 Acquisition Time	2.7525
17 Acquisition Date	2019-12-10T07:36:46
18 Modification Date	2019-12-10T11:38:43
19 Spectrometer Frequency	600.15
20 Spectral Width	11904.8
21 Lowest Frequency	-2261.0
22 Nucleus	1H
23 Acquired Size	32768
24 Spectral Size	65536

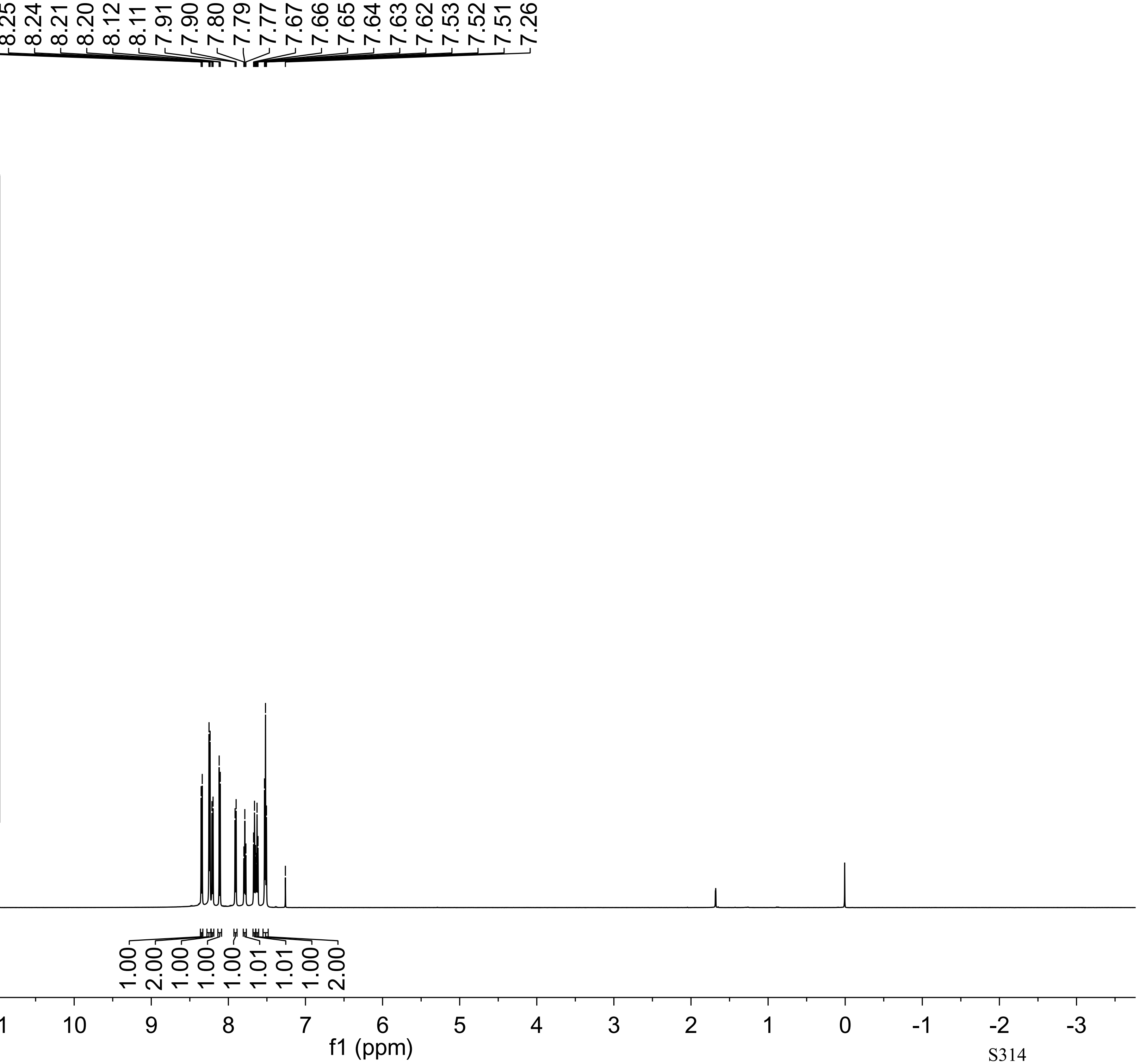

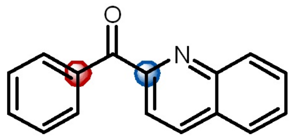

A9

	Parameter	Value
1	Title	CFM-E1
2	Comment	
3	Origin	Bruker BioSpin GmbH
4	Owner	nmrsu
5	Site	
6	Spectrometer	Avance NEO 600
7	Author	
8	Solvent	CDCl3
9	Temperature	298.3
10	Pulse Sequence	zgpg30
11	Experiment	1D
12	Number of Scans	128
13	Receiver Gain	101
14	Relaxation Delay	2.0000
15	Pulse Width	12.0000
16	Acquisition Time	0.9175
17	Acquisition Date	2019-12-10T07:44:18
18	Modification Date	2019-12-10T11:38:44
19	Spectrometer Frequency	150.91
20	Spectral Width	35714.3
21	Lowest Frequency	-2776.2
22	Nucleus	13C
23	Acquired Size	32768
24	Spectral Size	32768

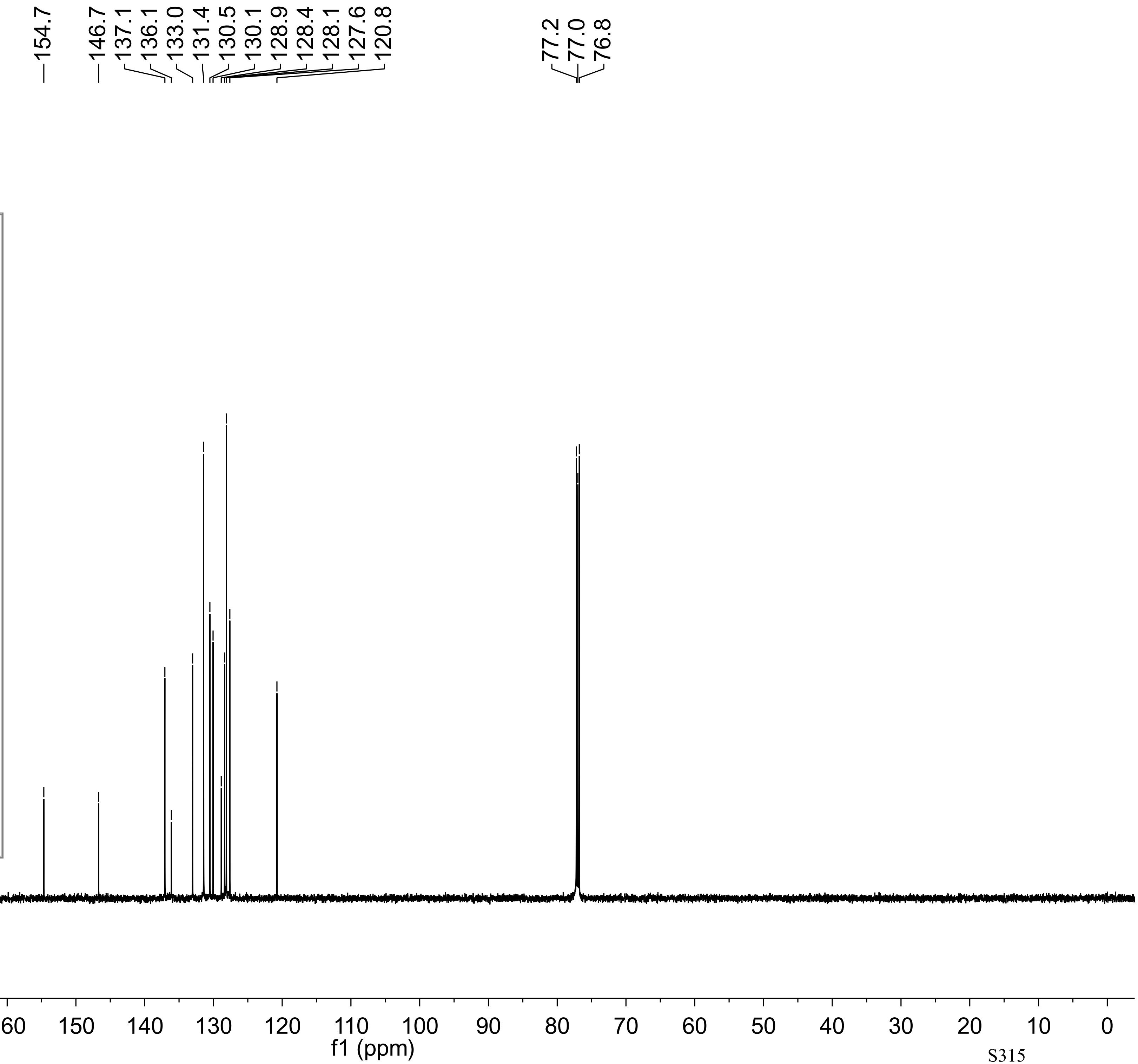

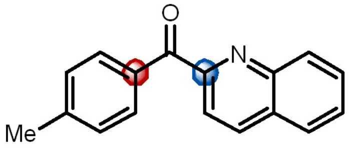

A10

	Parameter	Value
1	Title	CFM-E5
2	Comment	
3	Origin	Bruker BioSpin GmbH
4	Owner	nmrsu
5	Site	
6	Spectrometer	Avance NEO 600
7	Author	
8	Solvent	CDCl3
9	Temperature	297.8
10	Pulse Sequence	zg30
11	Experiment	1D
12	Number of Scans	8
13	Receiver Gain	30
14	Relaxation Delay	1.0000
15	Pulse Width	10.0000
16	Acquisition Time	2.7525
17	Acquisition Date	2020-08-05T23:18:18
18	Modification Date	2020-08-06T09:02:00
19	Spectrometer Frequency	600.15
20	Spectral Width	11904.8
21	Lowest Frequency	-2273.8
22	Nucleus	1H
23	Acquired Size	32768
24	Spectral Size	65536

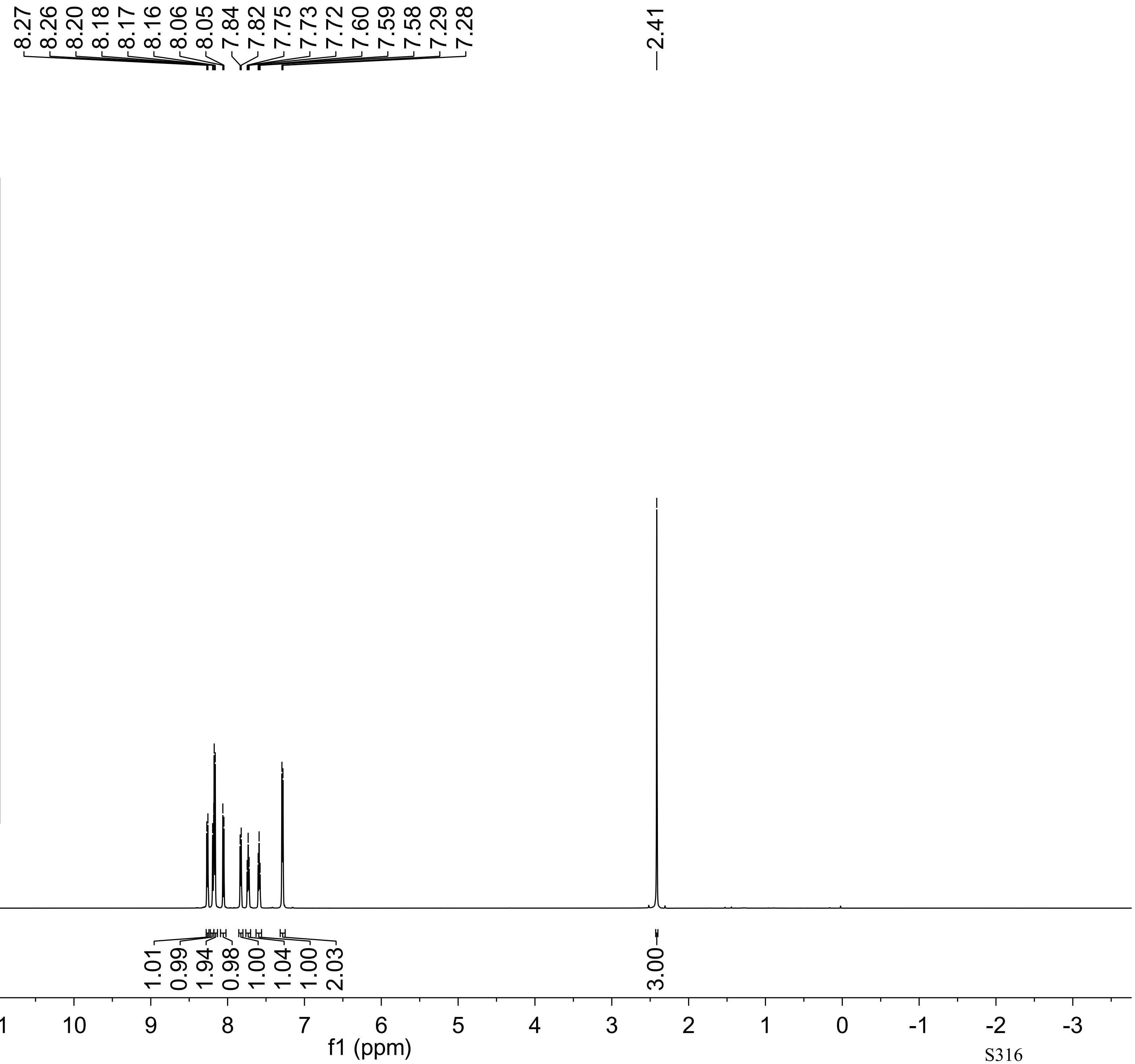

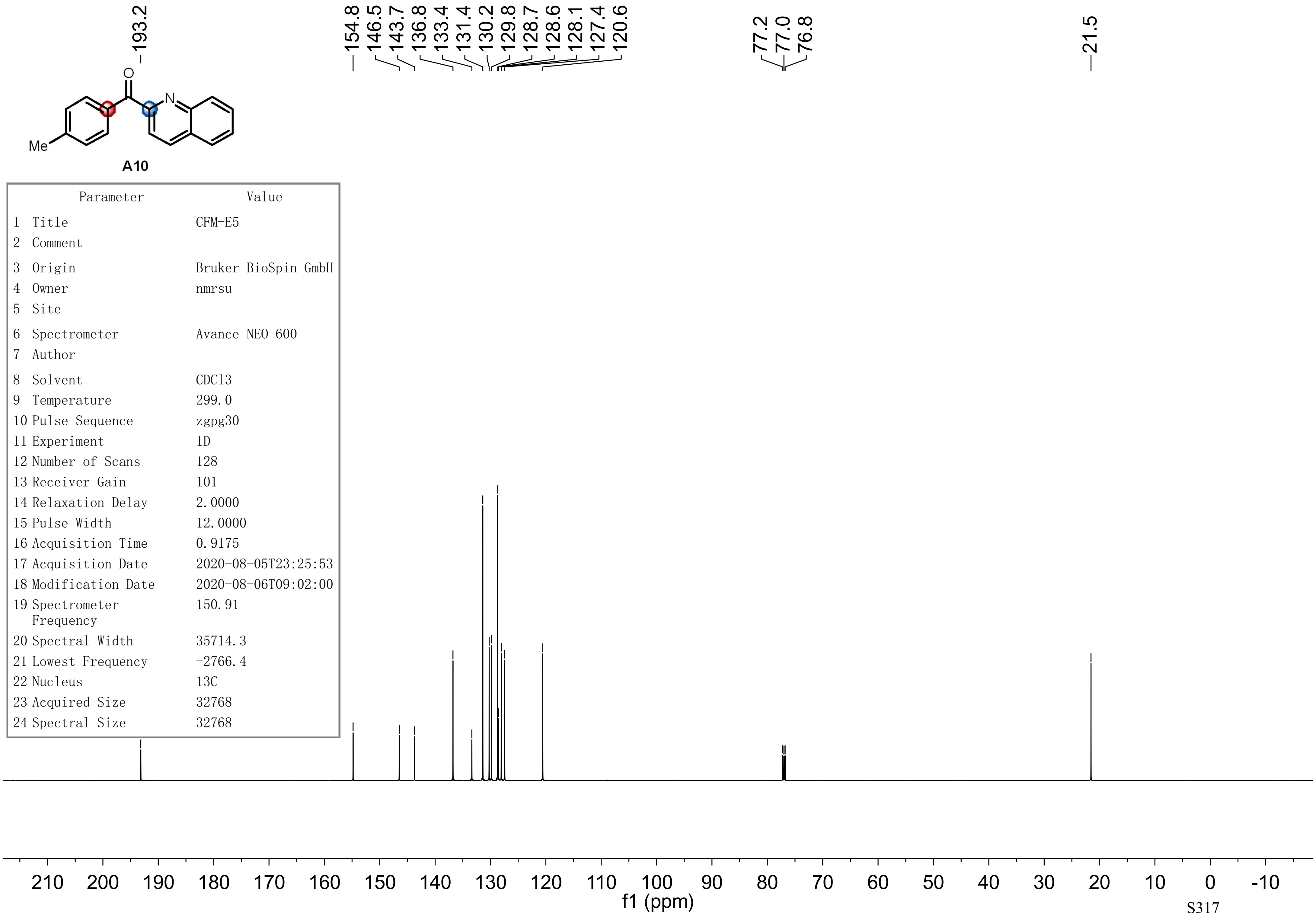

A10

	Parameter	Value
1	Title	CFM-E5
2	Comment	
3	Origin	Bruker BioSpin GmbH
4	Owner	nmrsu
5	Site	
6	Spectrometer	Avance NEO 600
7	Author	
8	Solvent	CDCl3
9	Temperature	299.0
10	Pulse Sequence	zgpg30
11	Experiment	1D
12	Number of Scans	128
13	Receiver Gain	101
14	Relaxation Delay	2.0000
15	Pulse Width	12.0000
16	Acquisition Time	0.9175
17	Acquisition Date	2020-08-05T23:25:53
18	Modification Date	2020-08-06T09:02:00
19	Spectrometer Frequency	150.91
20	Spectral Width	35714.3
21	Lowest Frequency	-2766.4
22	Nucleus	13C
23	Acquired Size	32768
24	Spectral Size	32768

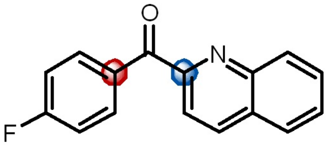

A11

Parameter	Value
1 Title	CFM-E2
2 Comment	
3 Origin	Bruker BioSpin GmbH
4 Owner	nmrsu
5 Site	
6 Spectrometer	Avance NEO 600
7 Author	
8 Solvent	CDCl3
9 Temperature	297.2
10 Pulse Sequence	zg30
11 Experiment	1D
12 Number of Scans	16
13 Receiver Gain	101
14 Relaxation Delay	1.0000
15 Pulse Width	10.0000
16 Acquisition Time	2.7525
17 Acquisition Date	2019-12-10T07:49:21
18 Modification Date	2019-12-10T11:38:47
19 Spectrometer Frequency	600.15
20 Spectral Width	11904.8
21 Lowest Frequency	-2260.9
22 Nucleus	1H
23 Acquired Size	32768
24 Spectral Size	65536

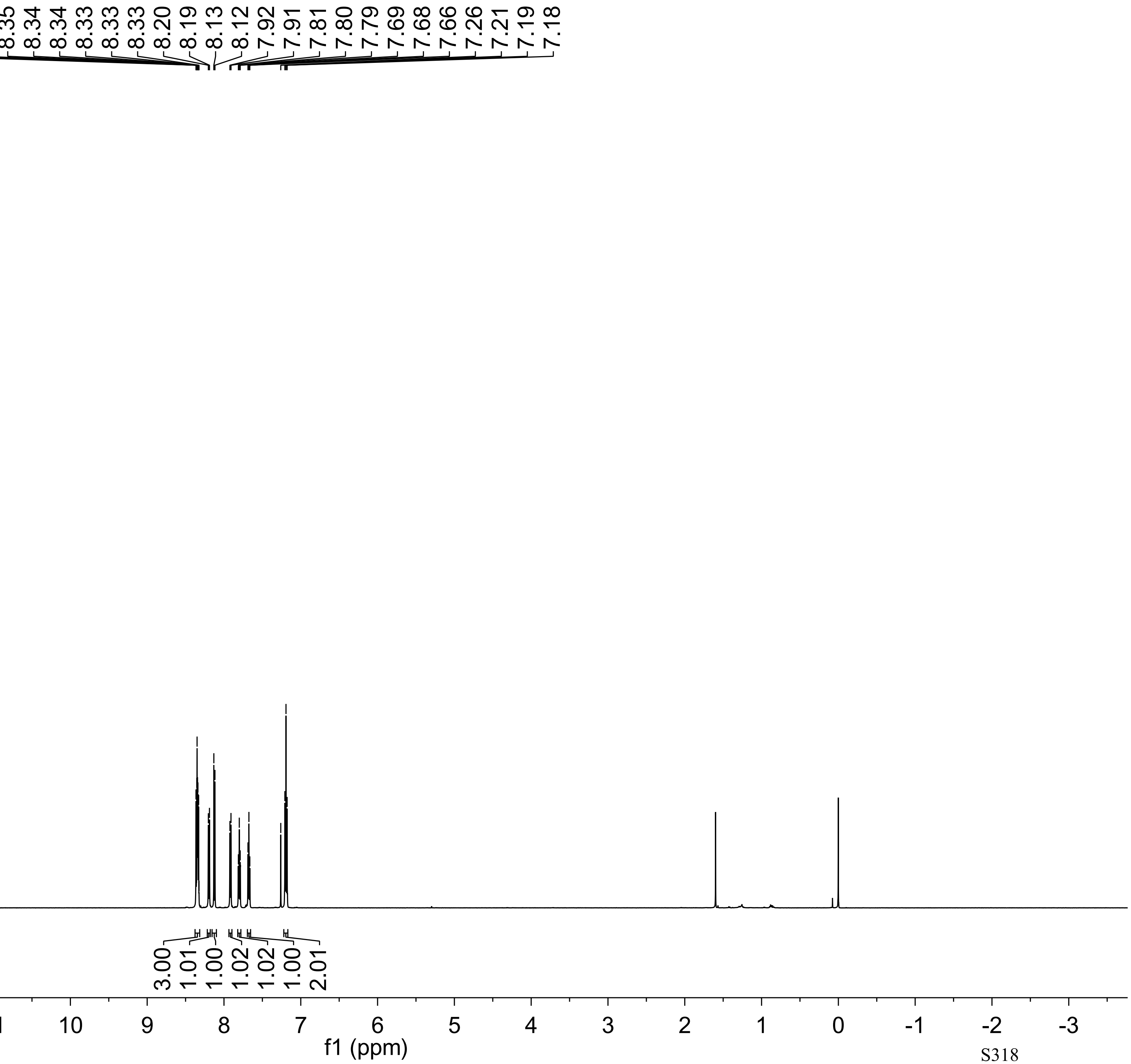

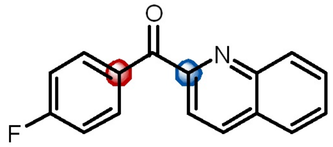

A11

Parameter	Value
1 Title	CFM-E2
2 Comment	
3 Origin	Bruker BioSpin GmbH
4 Owner	nmrsu
5 Site	
6 Spectrometer	Avance NEO 600
7 Author	
8 Solvent	CDCl3
9 Temperature	298.3
10 Pulse Sequence	zgpg30
11 Experiment	1D
12 Number of Scans	128
13 Receiver Gain	101
14 Relaxation Delay	2.0000
15 Pulse Width	12.0000
16 Acquisition Time	0.9175
17 Acquisition Date	2019-12-10T07:57:06
18 Modification Date	2019-12-10T11:38:47
19 Spectrometer Frequency	150.91
20 Spectral Width	35714.3
21 Lowest Frequency	-2771.9
22 Nucleus	13C
23 Acquired Size	32768
24 Spectral Size	32768

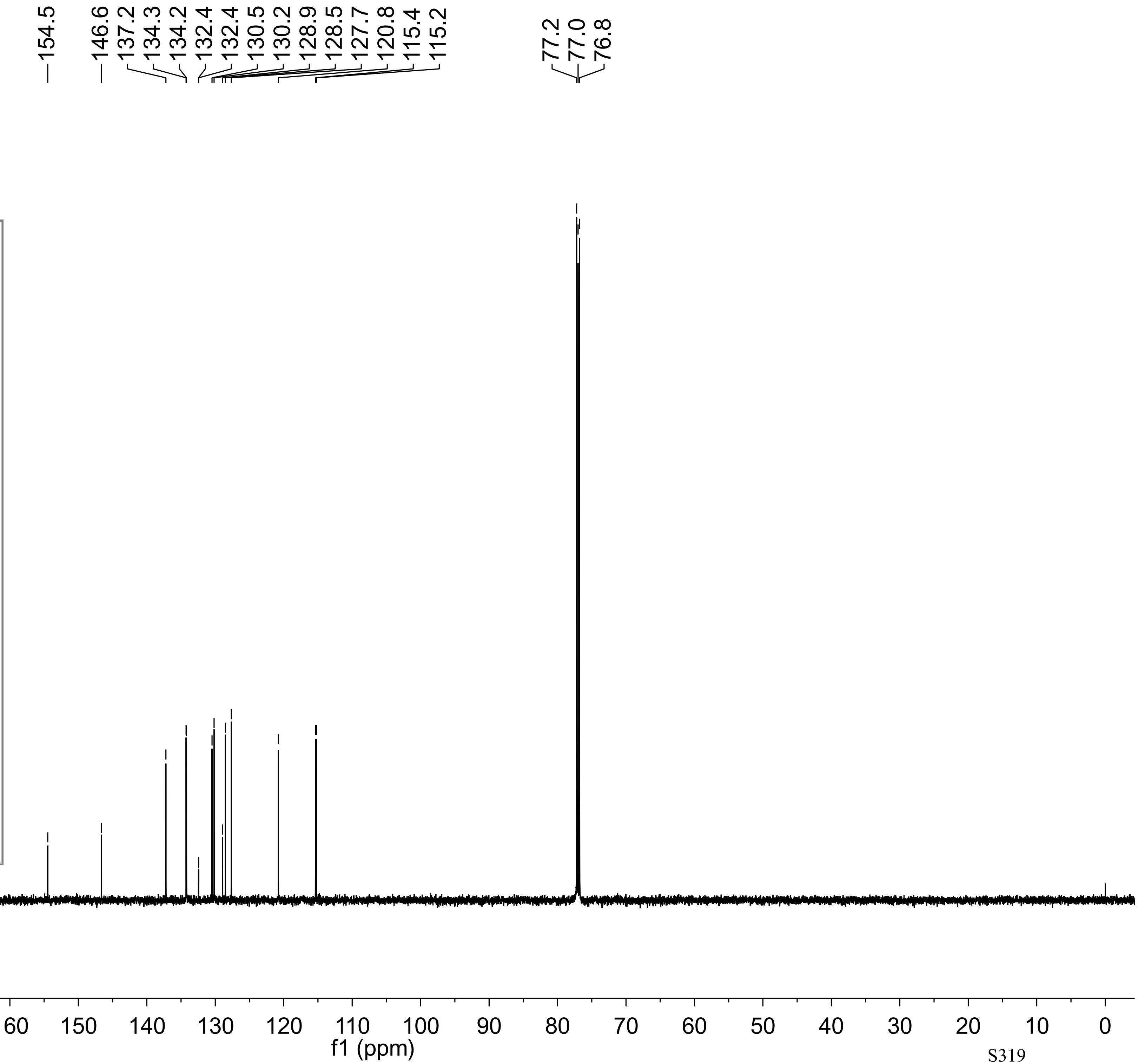

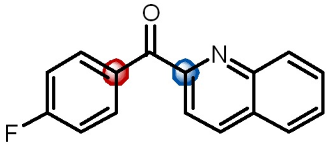

A11

Parameter	Value
1 Title	CFM-E1
2 Comment	
3 Origin	Bruker BioSpin GmbH
4 Owner	nmrsu
5 Site	
6 Spectrometer	Avance NEO 600
7 Author	
8 Solvent	CDCl3
9 Temperature	296.2
10 Pulse Sequence	zg
11 Experiment	1D
12 Number of Scans	2
13 Receiver Gain	101
14 Relaxation Delay	1.0000
15 Pulse Width	12.0000
16 Acquisition Time	0.4981
17 Acquisition Date	2019-12-13T14:37:13
18 Modification Date	2019-12-13T14:50:26
19 Spectrometer Frequency	564.71
20 Spectral Width	131579.0
21 Lowest Frequency	-122260.0
22 Nucleus	19F
23 Acquired Size	65536
24 Spectral Size	65536

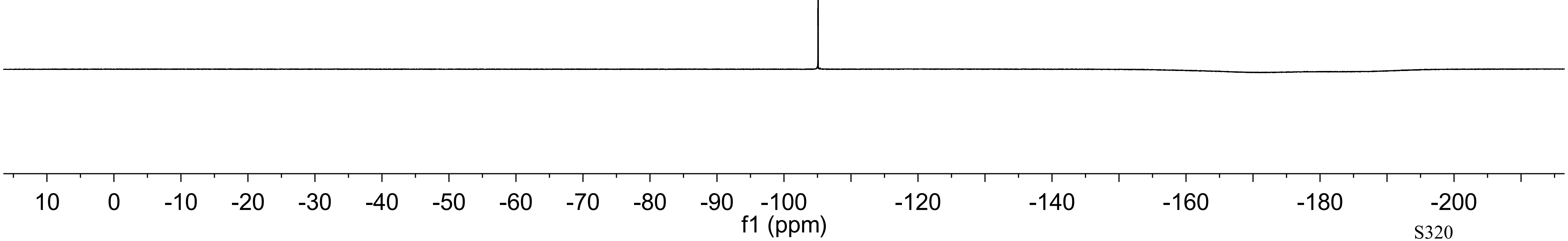

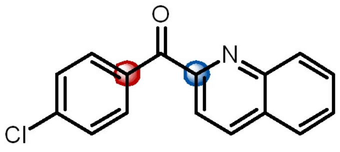

A12

Parameter	Value
1 Title	CFM-E3
2 Comment	
3 Origin	Bruker BioSpin GmbH
4 Owner	nmrsu
5 Site	
6 Spectrometer	Avance NEO 600
7 Author	
8 Solvent	CDCl3
9 Temperature	297.1
10 Pulse Sequence	zg30
11 Experiment	1D
12 Number of Scans	16
13 Receiver Gain	101
14 Relaxation Delay	1.0000
15 Pulse Width	10.0000
16 Acquisition Time	2.7525
17 Acquisition Date	2019-12-10T08:05:09
18 Modification Date	2019-12-10T11:38:46
19 Spectrometer Frequency	600.15
20 Spectral Width	11904.8
21 Lowest Frequency	-2260.9
22 Nucleus	1H
23 Acquired Size	32768
24 Spectral Size	65536

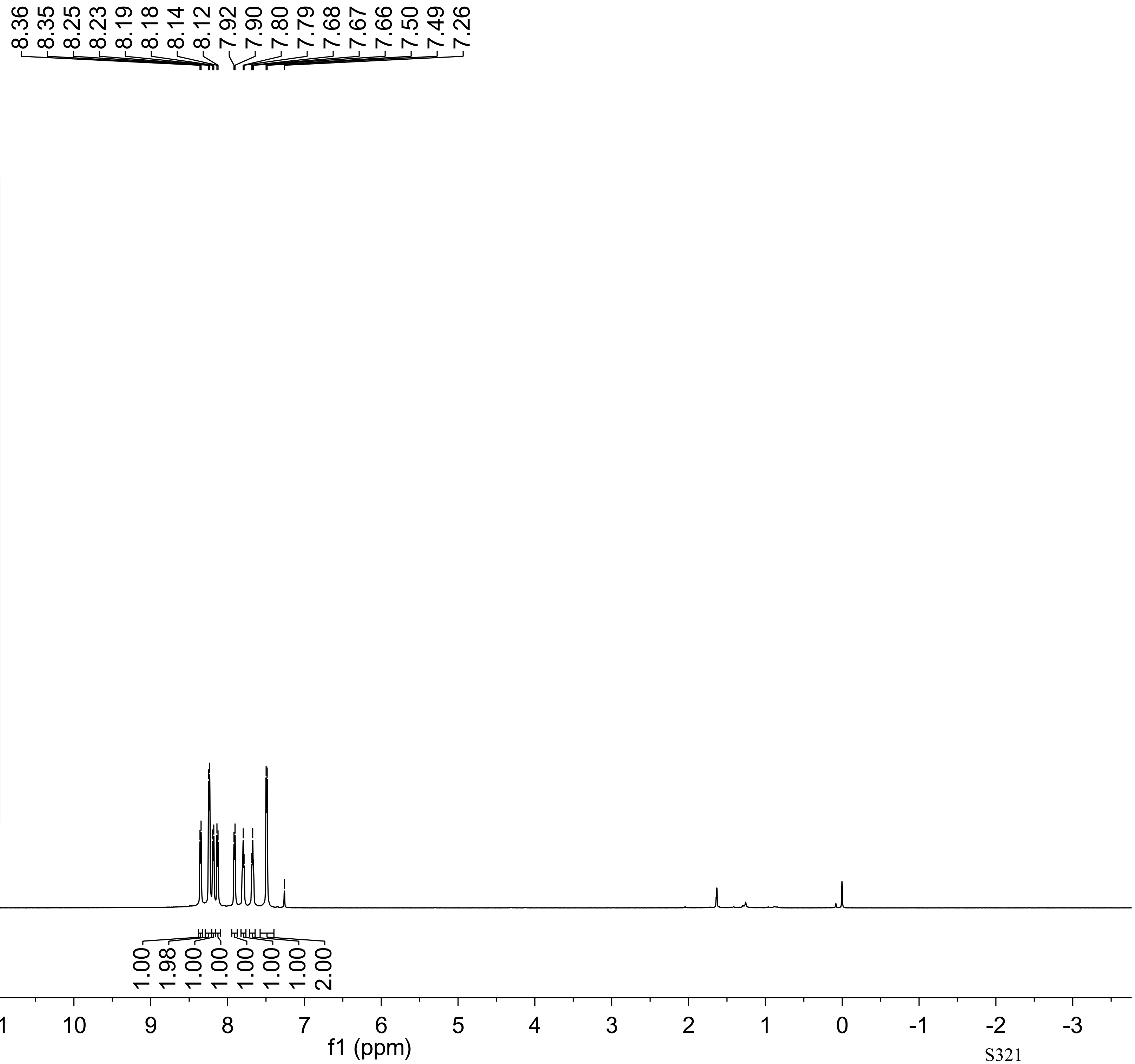

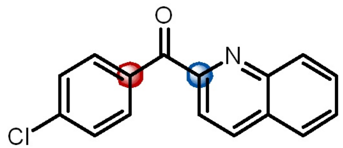

A12

	Parameter	Value
1	Title	CFM-E3
2	Comment	
3	Origin	Bruker BioSpin GmbH
4	Owner	nmrsu
5	Site	
6	Spectrometer	Avance NEO 600
7	Author	
8	Solvent	CDCl3
9	Temperature	298.5
10	Pulse Sequence	zgpg30
11	Experiment	1D
12	Number of Scans	128
13	Receiver Gain	101
14	Relaxation Delay	2.0000
15	Pulse Width	12.0000
16	Acquisition Time	0.9175
17	Acquisition Date	2019-12-10T08:12:59
18	Modification Date	2019-12-10T11:38:46
19	Spectrometer Frequency	150.91
20	Spectral Width	35714.3
21	Lowest Frequency	-2774.0
22	Nucleus	13C
23	Acquired Size	32768
24	Spectral Size	32768

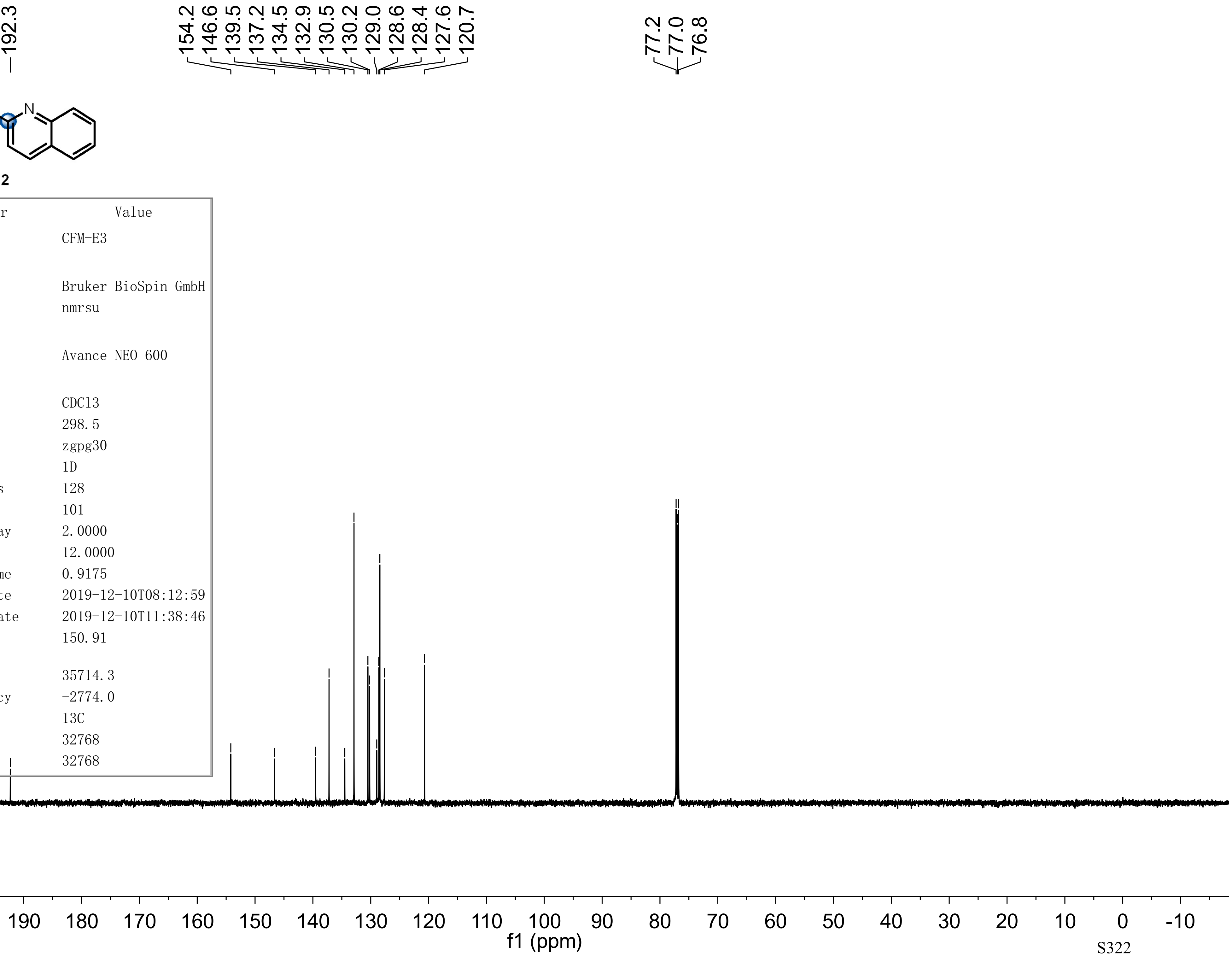

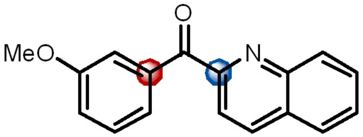

A13

Parameter	Value
1 Title	CFM-E7
2 Comment	
3 Origin	Bruker BioSpin GmbH
4 Owner	nmrsu
5 Site	
6 Spectrometer	Avance NEO 600
7 Author	
8 Solvent	CDCl3
9 Temperature	296.3
10 Pulse Sequence	zg30
11 Experiment	1D
12 Number of Scans	8
13 Receiver Gain	101
14 Relaxation Delay	1.0000
15 Pulse Width	10.0000
16 Acquisition Time	2.7525
17 Acquisition Date	2020-03-19T10:01:10
18 Modification Date	2020-03-19T14:58:36
19 Spectrometer Frequency	600.15
20 Spectral Width	11904.8
21 Lowest Frequency	-2261.4
22 Nucleus	1H
23 Acquired Size	32768
24 Spectral Size	65536

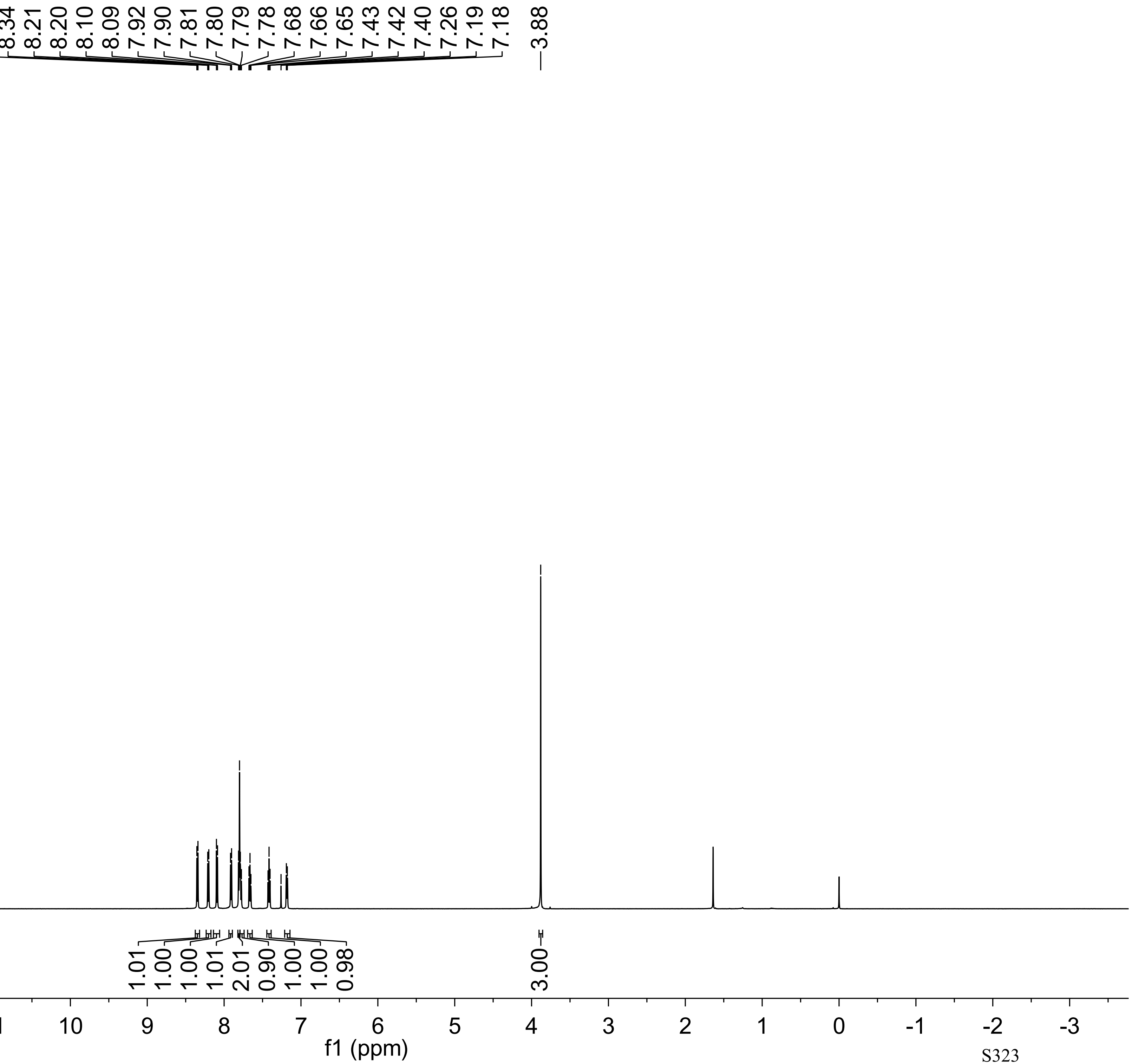

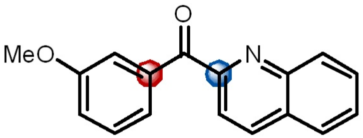

A13

Parameter	Value
1 Title	CFM-E7
2 Comment	
3 Origin	Bruker BioSpin GmbH
4 Owner	nmrsu
5 Site	
6 Spectrometer	Avance NEO 600
7 Author	
8 Solvent	CDCl3
9 Temperature	297.1
10 Pulse Sequence	zgpg30
11 Experiment	1D
12 Number of Scans	128
13 Receiver Gain	101
14 Relaxation Delay	2.0000
15 Pulse Width	12.0000
16 Acquisition Time	0.9175
17 Acquisition Date	2020-03-19T10:09:08
18 Modification Date	2020-03-19T14:58:37
19 Spectrometer Frequency	150.91
20 Spectral Width	35714.3
21 Lowest Frequency	-2773.7
22 Nucleus	13C
23 Acquired Size	32768
24 Spectral Size	32768

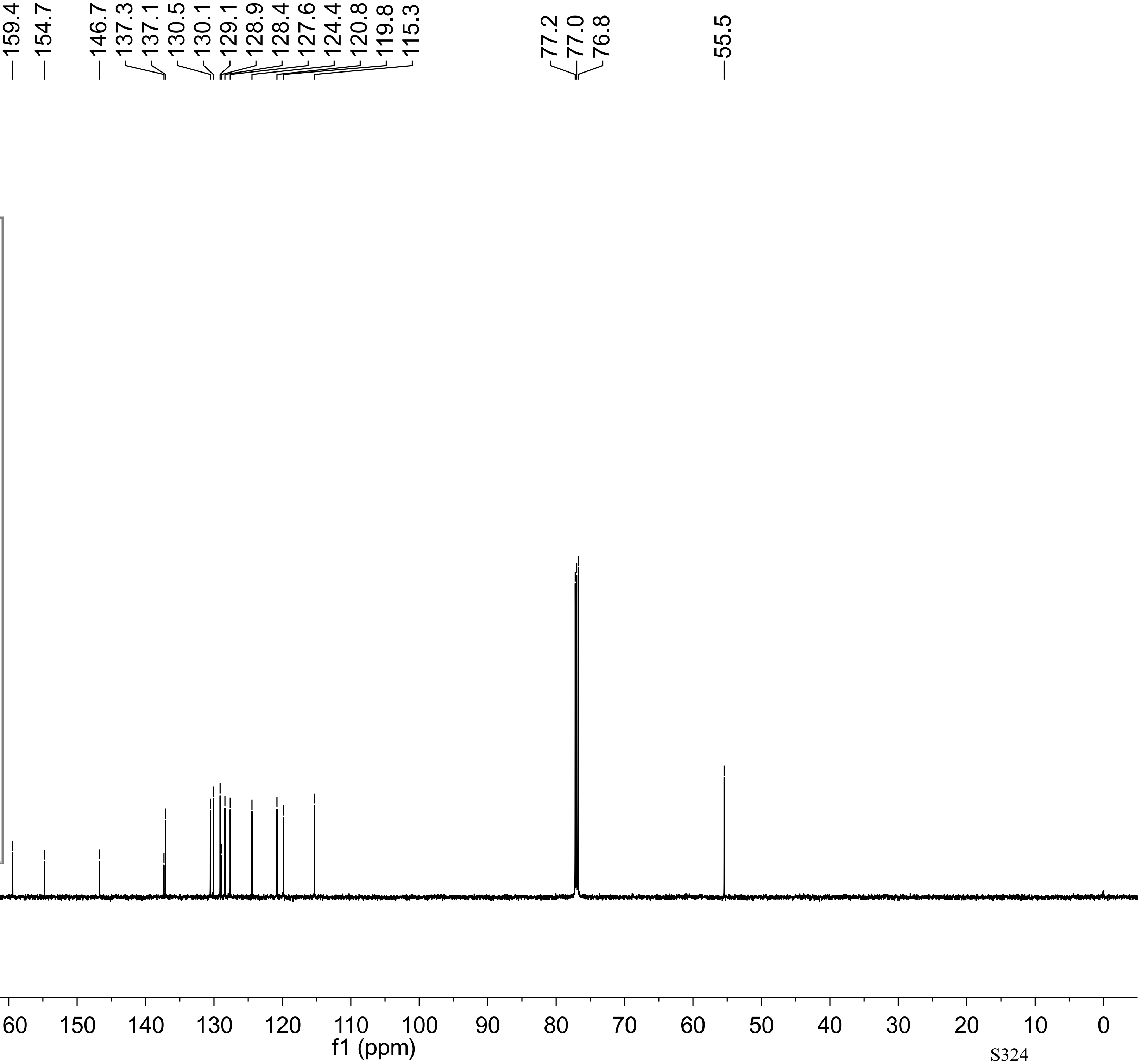

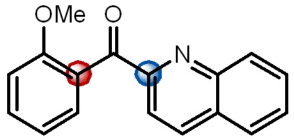

A14

Parameter	Value
1 Title	CFM-E8
2 Comment	
3 Origin	Bruker BioSpin GmbH
4 Owner	nmrsu
5 Site	
6 Spectrometer	Avance NEO 600
7 Author	
8 Solvent	CDCl3
9 Temperature	298.9
10 Pulse Sequence	zg30
11 Experiment	1D
12 Number of Scans	16
13 Receiver Gain	101
14 Relaxation Delay	1.0000
15 Pulse Width	10.0000
16 Acquisition Time	2.7525
17 Acquisition Date	2019-12-30T07:48:02
18 Modification Date	2019-12-30T08:57:57
19 Spectrometer Frequency	600.15
20 Spectral Width	11904.8
21 Lowest Frequency	-2261.0
22 Nucleus	1H
23 Acquired Size	32768
24 Spectral Size	65536

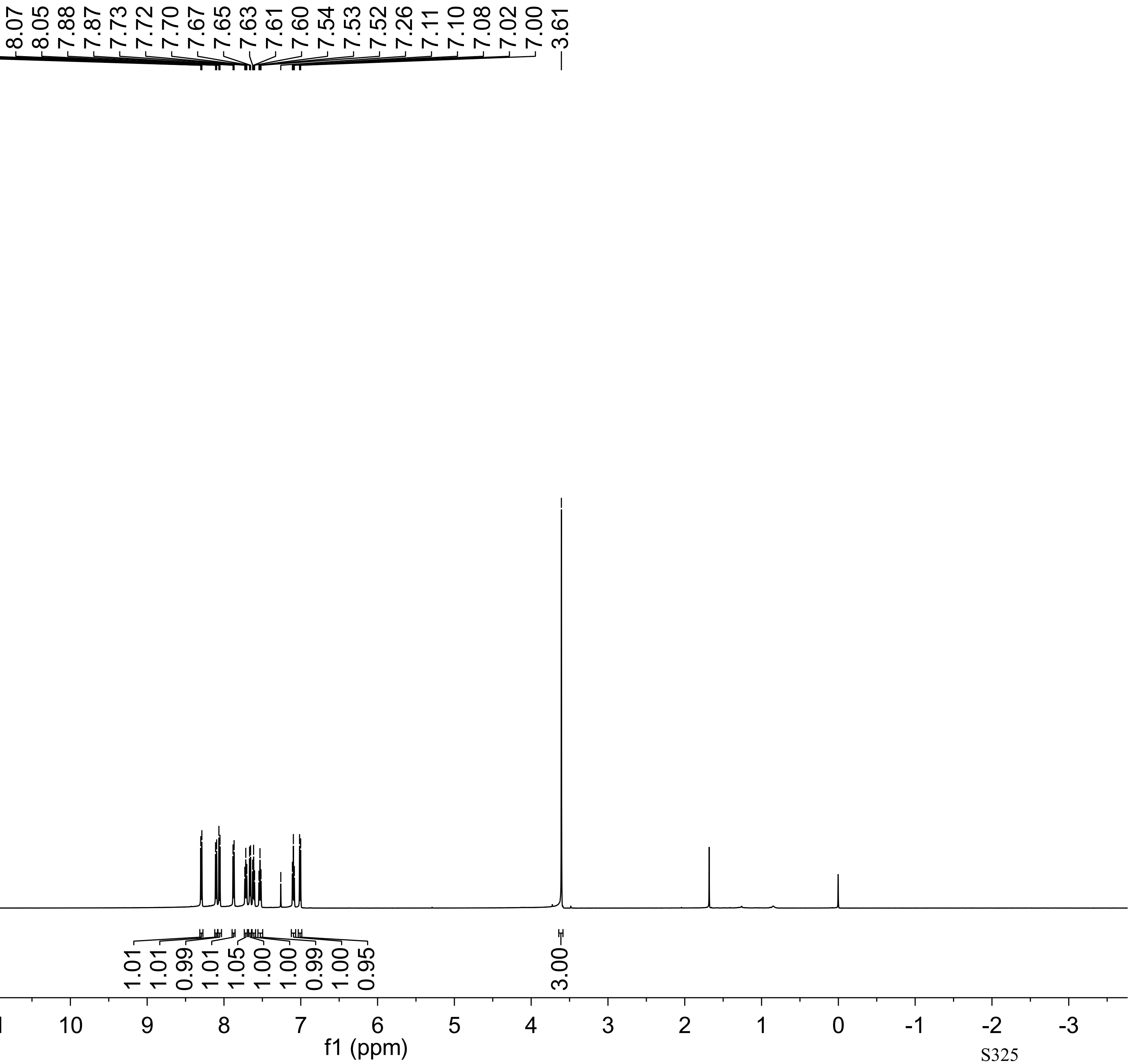

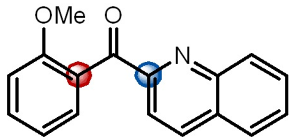

A14

Parameter		Value
1	Title	CFM-E8
2	Comment	
3	Origin	Bruker BioSpin GmbH
4	Owner	nmrsu
5	Site	
6	Spectrometer	Avance NEO 600
7	Author	
8	Solvent	CDCl3
9	Temperature	299.3
10	Pulse Sequence	zgpg30
11	Experiment	1D
12	Number of Scans	318
13	Receiver Gain	101
14	Relaxation Delay	2.0000
15	Pulse Width	12.0000
16	Acquisition Time	0.9175
17	Acquisition Date	2019-12-30T08:05:15
18	Modification Date	2019-12-30T08:57:57
19	Spectrometer Frequency	150.91
20	Spectral Width	35714.3
21	Lowest Frequency	-2773.4
22	Nucleus	13C
23	Acquired Size	32768
24	Spectral Size	32768

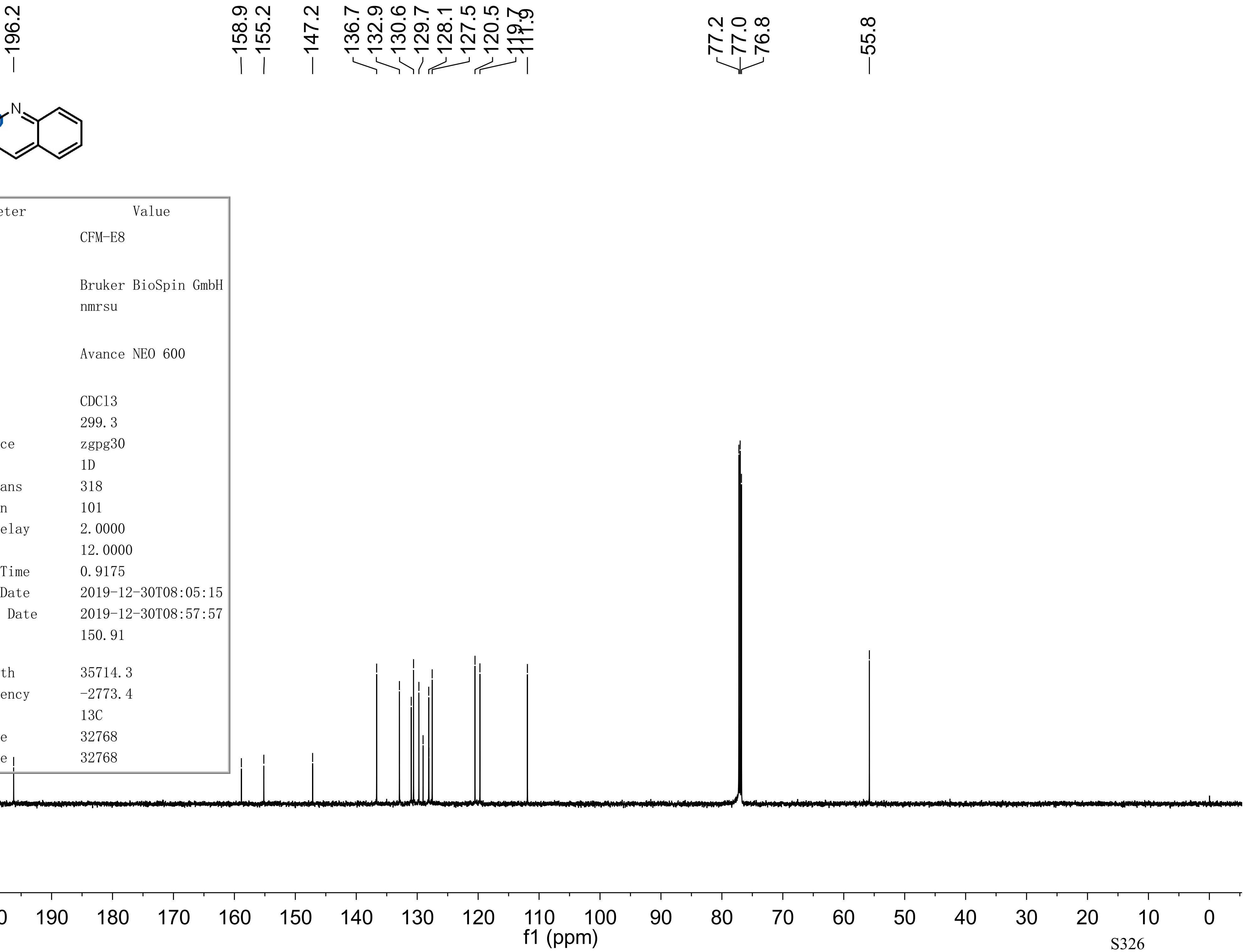

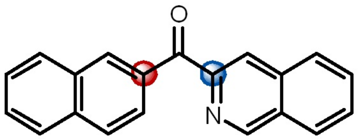

A15

Parameter	Value
1 Title	CFM-A2
2 Comment	
3 Origin	Bruker BioSpin GmbH
4 Owner	nmrsu
5 Site	
6 Spectrometer	Avance NEO 600
7 Author	
8 Solvent	CDCl3
9 Temperature	297.8
10 Pulse Sequence	zg30
11 Experiment	1D
12 Number of Scans	8
13 Receiver Gain	101
14 Relaxation Delay	1.0000
15 Pulse Width	10.0000
16 Acquisition Time	2.7525
17 Acquisition Date	2020-08-05T23:05:07
18 Modification Date	2020-08-06T09:01:59
19 Spectrometer Frequency	600.15
20 Spectral Width	11904.8
21 Lowest Frequency	-2260.8
22 Nucleus	1H
23 Acquired Size	32768
24 Spectral Size	65536

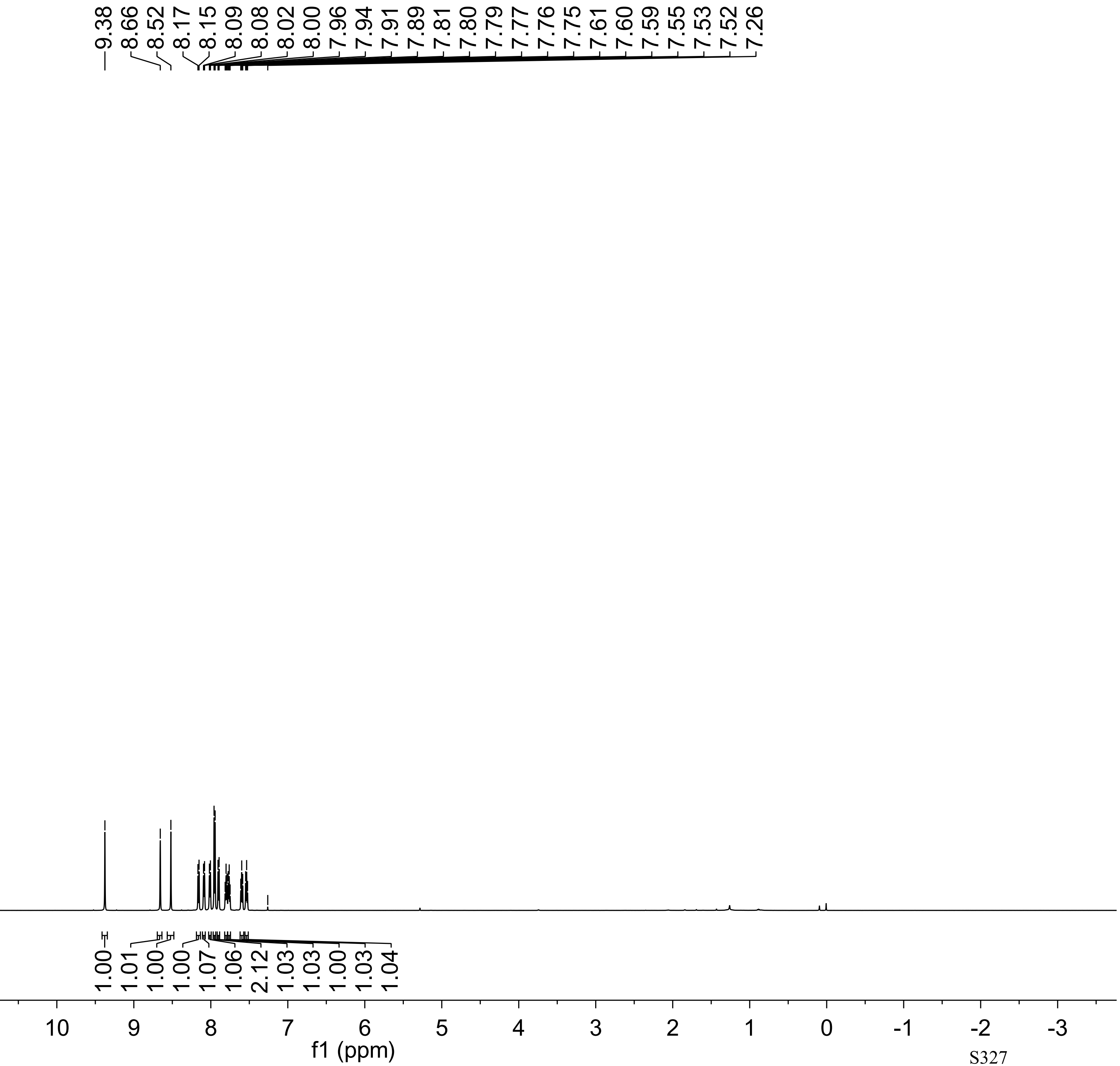

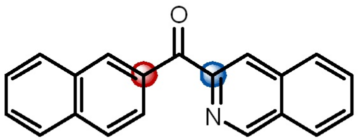

A15

Parameter		Value
1	Title	2-S16-0325
2	Comment	
3	Origin	Bruker BioSpin GmbH
4	Owner	nmrsu
5	Site	
6	Spectrometer	Avance NEO 600
7	Author	
8	Solvent	CDCl3
9	Temperature	298.2
10	Pulse Sequence	zgpg30
11	Experiment	1D
12	Number of Scans	128
13	Receiver Gain	101
14	Relaxation Delay	2.0000
15	Pulse Width	12.0000
16	Acquisition Time	0.9175
17	Acquisition Date	2019-03-26T11:10:05
18	Modification Date	2019-03-26T11:12:25
19	Spectrometer Frequency	150.91
20	Spectral Width	35714.3
21	Lowest Frequency	-2771.3
22	Nucleus	13C
23	Acquired Size	32768
24	Spectral Size	32768

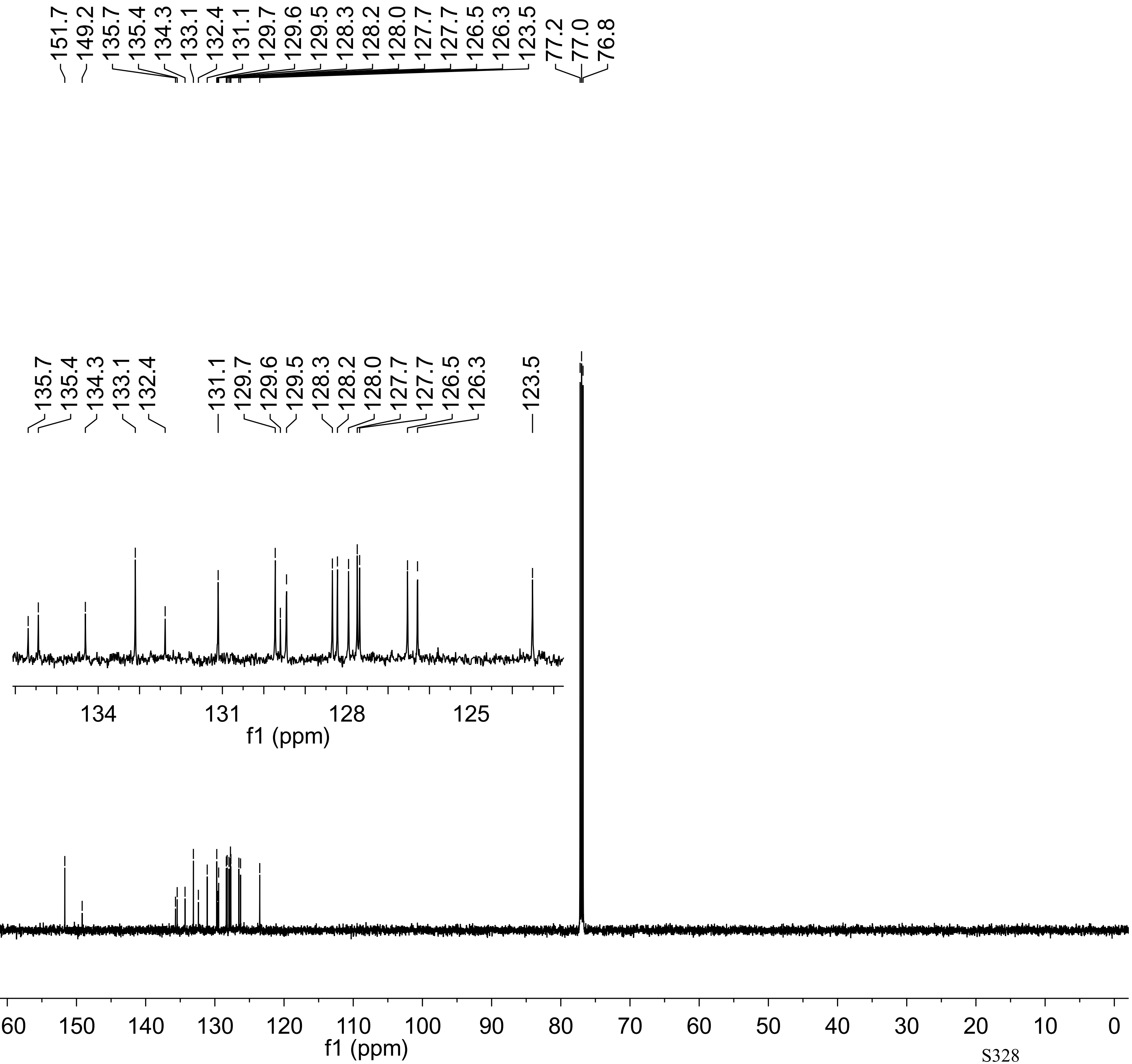

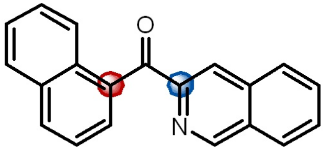

A16

Parameter	Value
1 Title	cfm-A3
2 Comment	
3 Origin	Bruker BioSpin GmbH
4 Owner	nmrsu
5 Site	
6 Spectrometer	Avance NEO 600
7 Author	
8 Solvent	CDCl3
9 Temperature	296.8
10 Pulse Sequence	zg30
11 Experiment	1D
12 Number of Scans	16
13 Receiver Gain	101
14 Relaxation Delay	1.0000
15 Pulse Width	10.0000
16 Acquisition Time	2.7525
17 Acquisition Date	2019-12-09T20:08:11
18 Modification Date	2019-12-09T21:26:32
19 Spectrometer Frequency	600.15
20 Spectral Width	11904.8
21 Lowest Frequency	-2261.0
22 Nucleus	1H
23 Acquired Size	32768
24 Spectral Size	65536

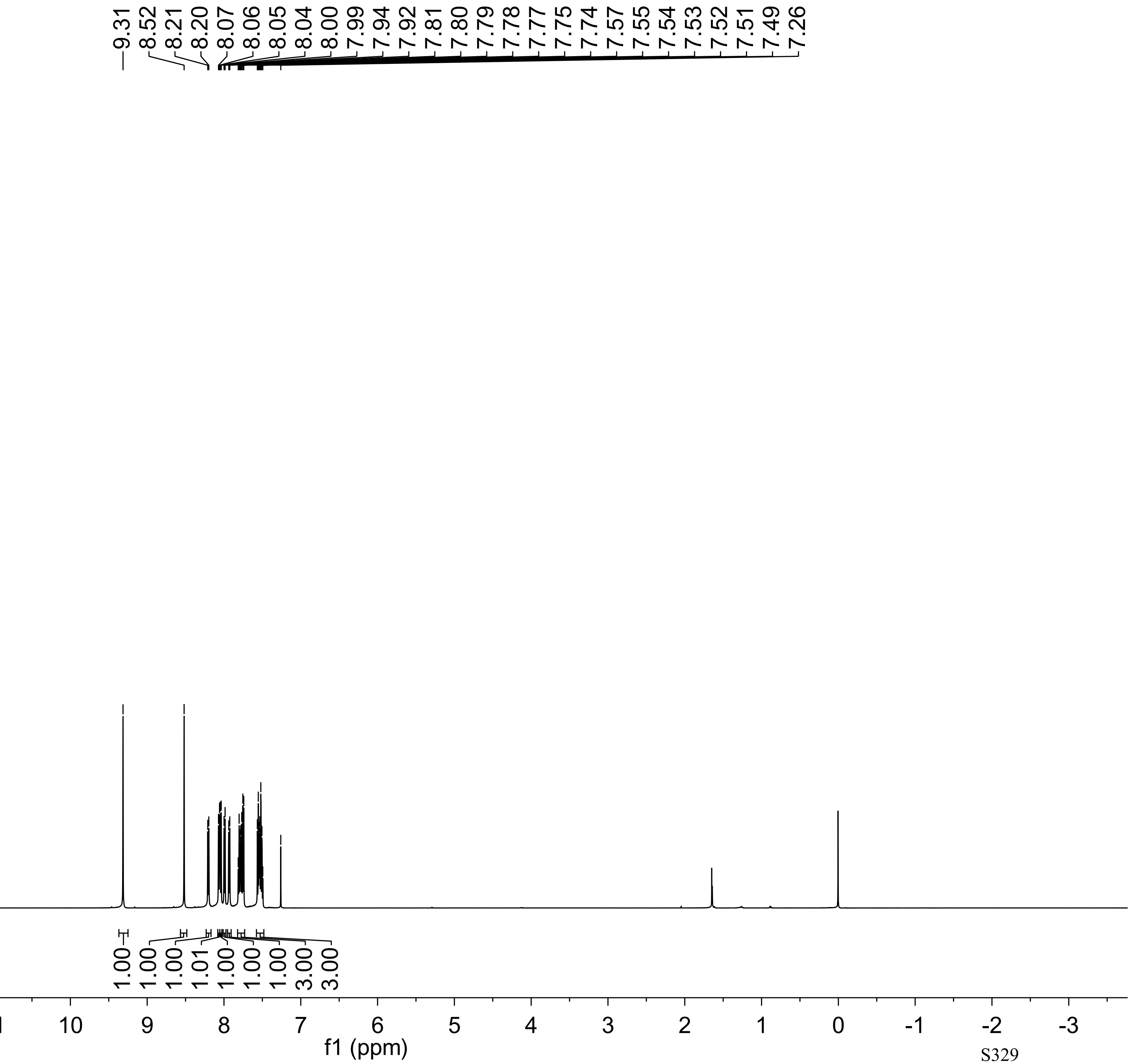

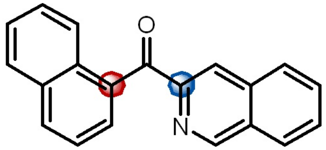

A16

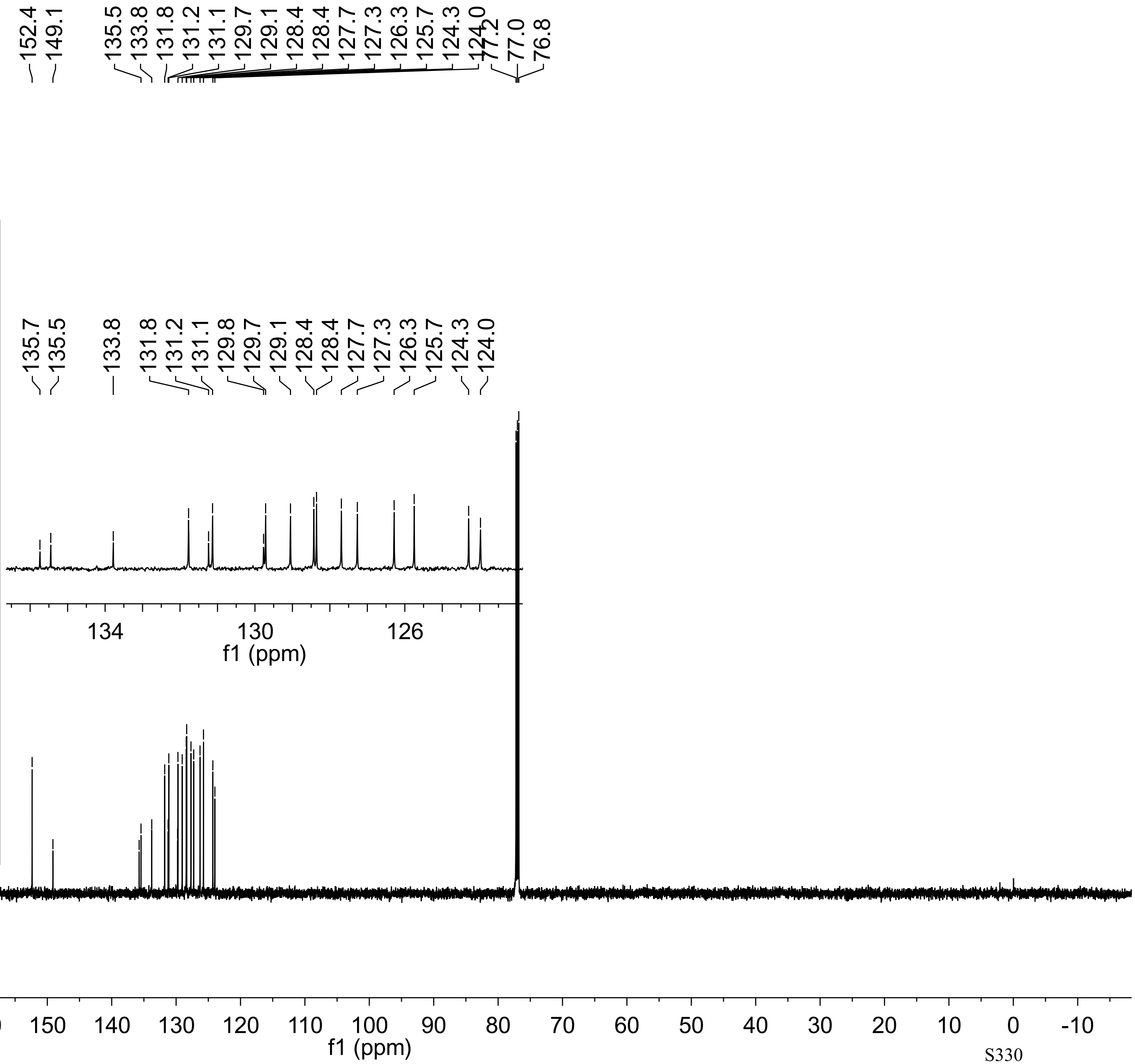

	Parameter	Value
1	Title	cfm-A3
2	Comment	
3	Origin	Bruker BioSpin GmbH
4	Owner	nmrsu
5	Site	
6	Spectrometer	Avance NEO 600
7	Author	
8	Solvent	CDCl3
9	Temperature	297.9
10	Pulse Sequence	zgpg30
11	Experiment	1D
12	Number of Scans	64
13	Receiver Gain	101
14	Relaxation Delay	2.0000
15	Pulse Width	12.0000
16	Acquisition Time	0.9175
17	Acquisition Date	2019-12-09T20:12:11
18	Modification Date	2019-12-09T21:26:33
19	Spectrometer Frequency	150.91
20	Spectral Width	35714.3
21	Lowest Frequency	-2774.8
22	Nucleus	13C
23	Acquired Size	32768
24	Spectral Size	32768

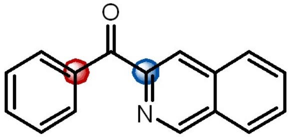

A17

Parameter	Value
1 Title	CFM-A1
2 Comment	
3 Origin	Bruker BioSpin GmbH
4 Owner	nmrsu
5 Site	
6 Spectrometer	Avance NEO 600
7 Author	
8 Solvent	CDCl3
9 Temperature	296.4
10 Pulse Sequence	zg30
11 Experiment	1D
12 Number of Scans	16
13 Receiver Gain	101
14 Relaxation Delay	1.0000
15 Pulse Width	10.0000
16 Acquisition Time	2.7525
17 Acquisition Date	2019-12-09T19:47:06
18 Modification Date	2019-12-09T21:26:34
19 Spectrometer Frequency	600.15
20 Spectral Width	11904.8
21 Lowest Frequency	-2261.0
22 Nucleus	1H
23 Acquired Size	32768
24 Spectral Size	65536

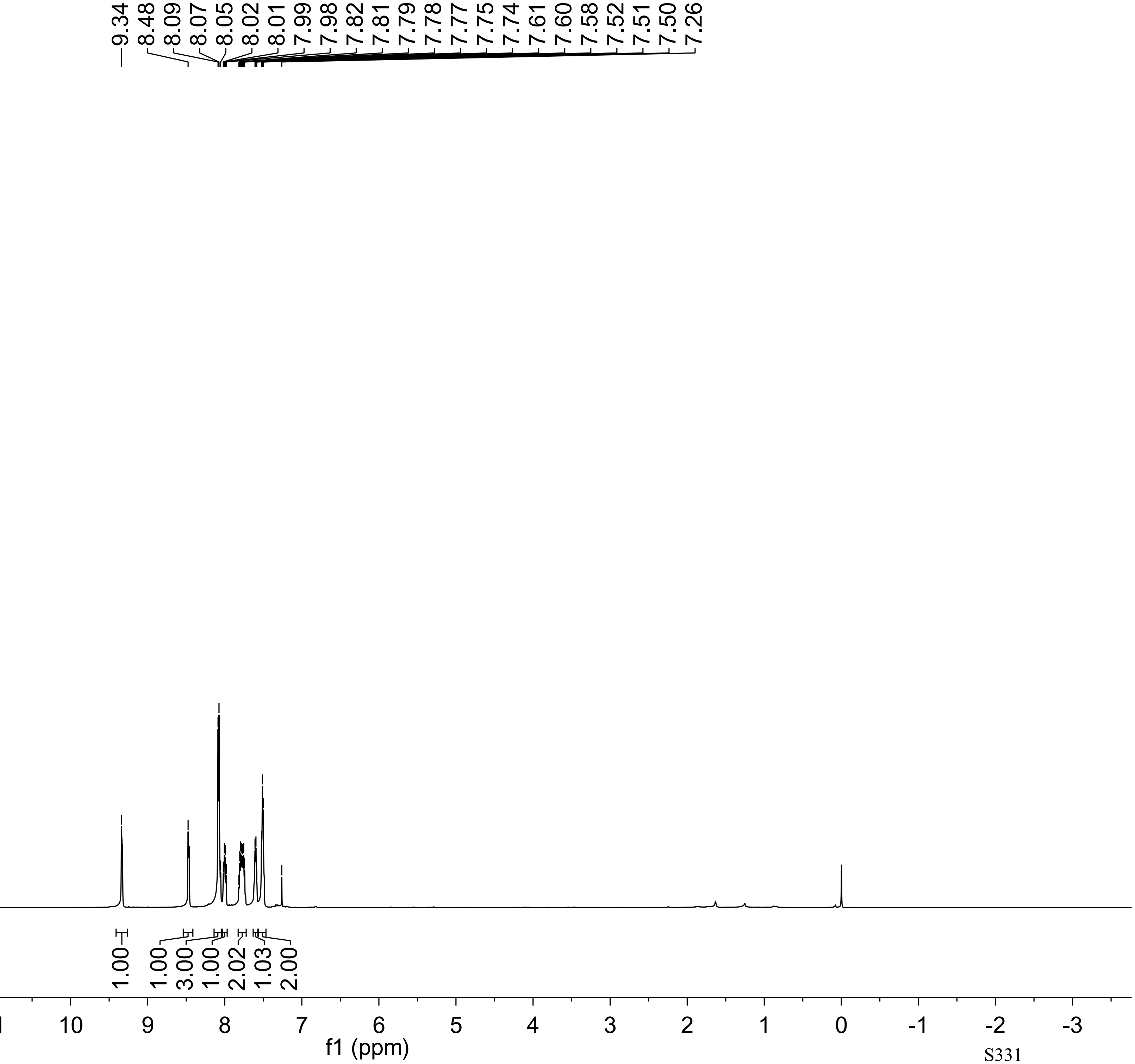

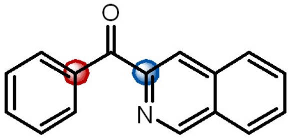

A17

Parameter	Value
1 Title	CFM-A1
2 Comment	
3 Origin	Bruker BioSpin GmbH
4 Owner	nmrsu
5 Site	
6 Spectrometer	Avance NEO 600
7 Author	
8 Solvent	CDCl3
9 Temperature	297.4
10 Pulse Sequence	zgpg30
11 Experiment	1D
12 Number of Scans	64
13 Receiver Gain	101
14 Relaxation Delay	2.0000
15 Pulse Width	12.0000
16 Acquisition Time	0.9175
17 Acquisition Date	2019-12-09T19:51:34
18 Modification Date	2019-12-09T21:26:35
19 Spectrometer Frequency	150.91
20 Spectral Width	35714.3
21 Lowest Frequency	-2773.6
22 Nucleus	13C
23 Acquired Size	32768
24 Spectral Size	32768

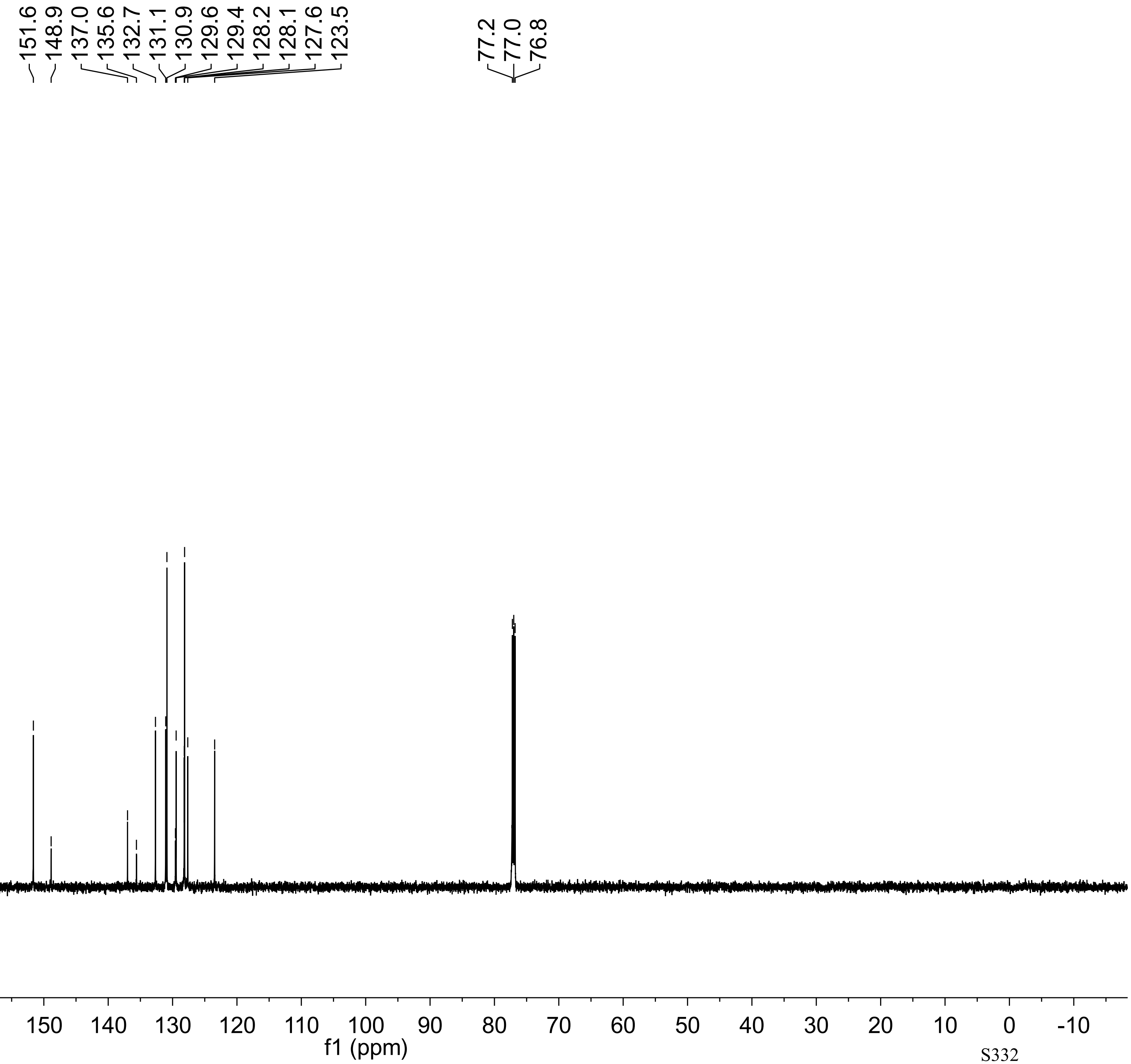

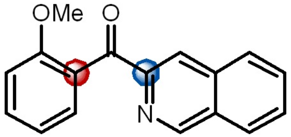

A18

Parameter	Value
1 Title	cfm-A4
2 Comment	
3 Origin	Bruker BioSpin GmbH
4 Owner	nmrsu
5 Site	
6 Spectrometer	Avance NEO 600
7 Author	
8 Solvent	CDCl3
9 Temperature	296.8
10 Pulse Sequence	zg30
11 Experiment	1D
12 Number of Scans	16
13 Receiver Gain	101
14 Relaxation Delay	1.0000
15 Pulse Width	10.0000
16 Acquisition Time	2.7525
17 Acquisition Date	2019-12-09T20:18:24
18 Modification Date	2019-12-09T21:26:31
19 Spectrometer Frequency	600.15
20 Spectral Width	11904.8
21 Lowest Frequency	-2261.5
22 Nucleus	1H
23 Acquired Size	32768
24 Spectral Size	65536

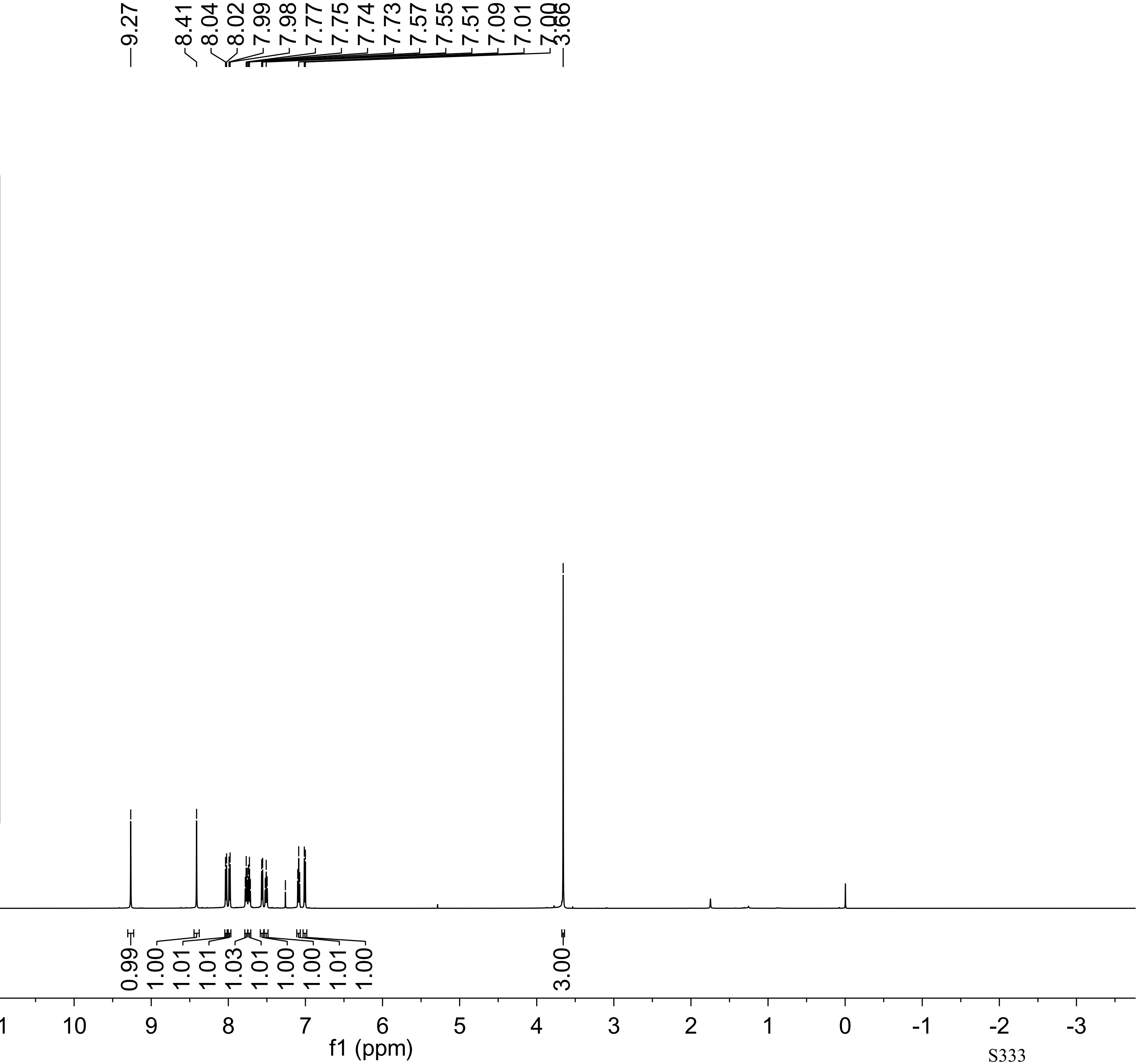

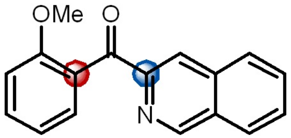

A18

	Parameter	Value
1	Title	cfm-A4
2	Comment	
3	Origin	Bruker BioSpin GmbH
4	Owner	nmrsu
5	Site	
6	Spectrometer	Avance NEO 600
7	Author	
8	Solvent	CDCl3
9	Temperature	297.8
10	Pulse Sequence	zgpg30
11	Experiment	1D
12	Number of Scans	64
13	Receiver Gain	101
14	Relaxation Delay	2.0000
15	Pulse Width	12.0000
16	Acquisition Time	0.9175
17	Acquisition Date	2019-12-09T20:22:48
18	Modification Date	2019-12-09T21:26:32
19	Spectrometer Frequency	150.91
20	Spectral Width	35714.3
21	Lowest Frequency	-2777.1
22	Nucleus	13C
23	Acquired Size	32768
24	Spectral Size	32768

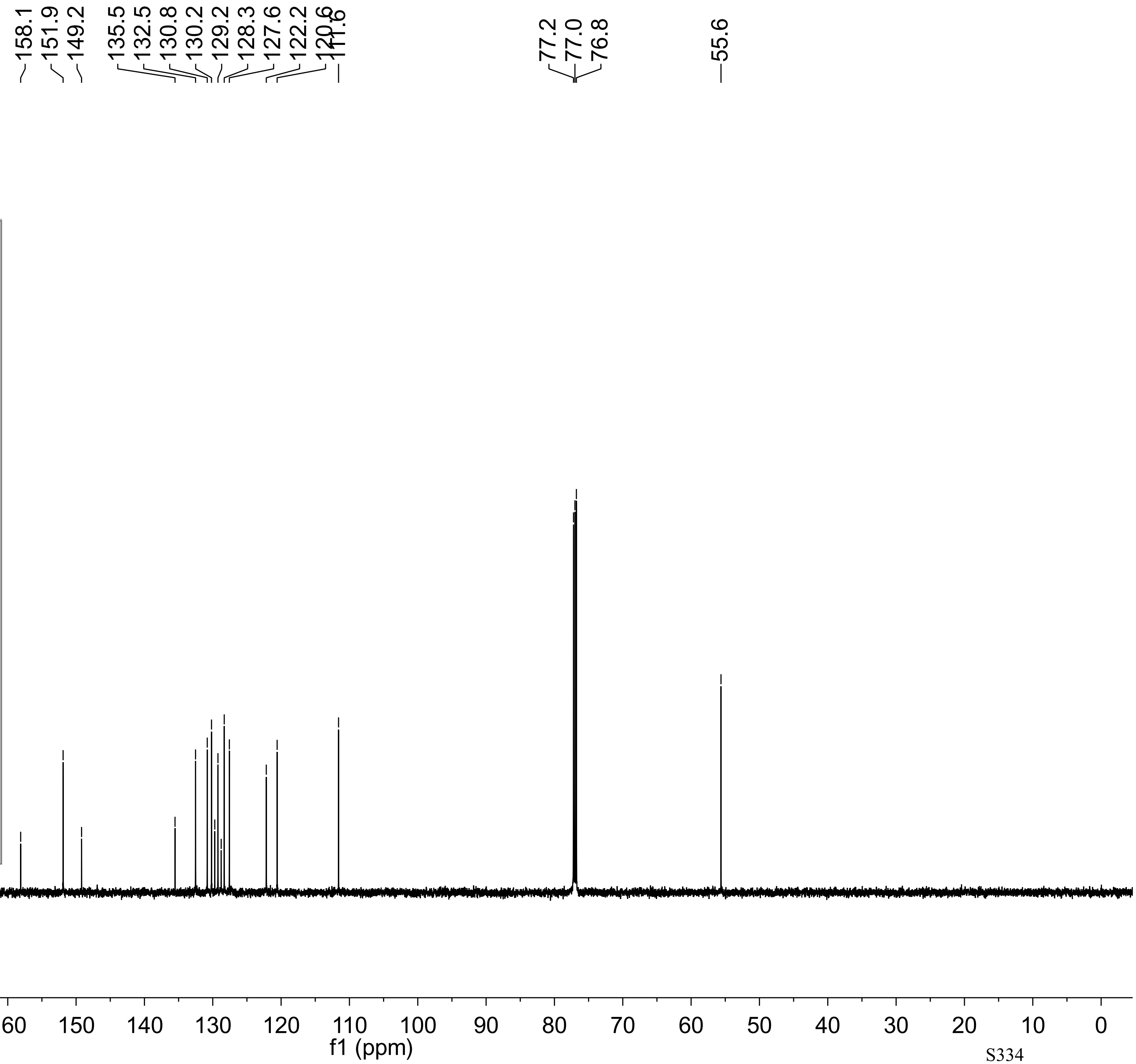

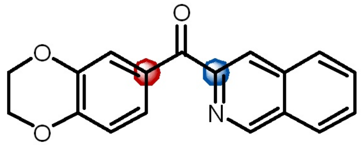

A19

Parameter	Value
1 Title	CFM-A5
2 Comment	
3 Origin	Bruker BioSpin GmbH
4 Owner	nmrsu
5 Site	
6 Spectrometer	Avance NEO 600
7 Author	
8 Solvent	CDCl3
9 Temperature	296.8
10 Pulse Sequence	zg30
11 Experiment	1D
12 Number of Scans	16
13 Receiver Gain	101
14 Relaxation Delay	1.0000
15 Pulse Width	10.0000
16 Acquisition Time	2.7525
17 Acquisition Date	2019-12-09T23:37:33
18 Modification Date	2019-12-10T11:38:09
19 Spectrometer Frequency	600.15
20 Spectral Width	11904.8
21 Lowest Frequency	-2261.2
22 Nucleus	1H
23 Acquired Size	32768
24 Spectral Size	65536

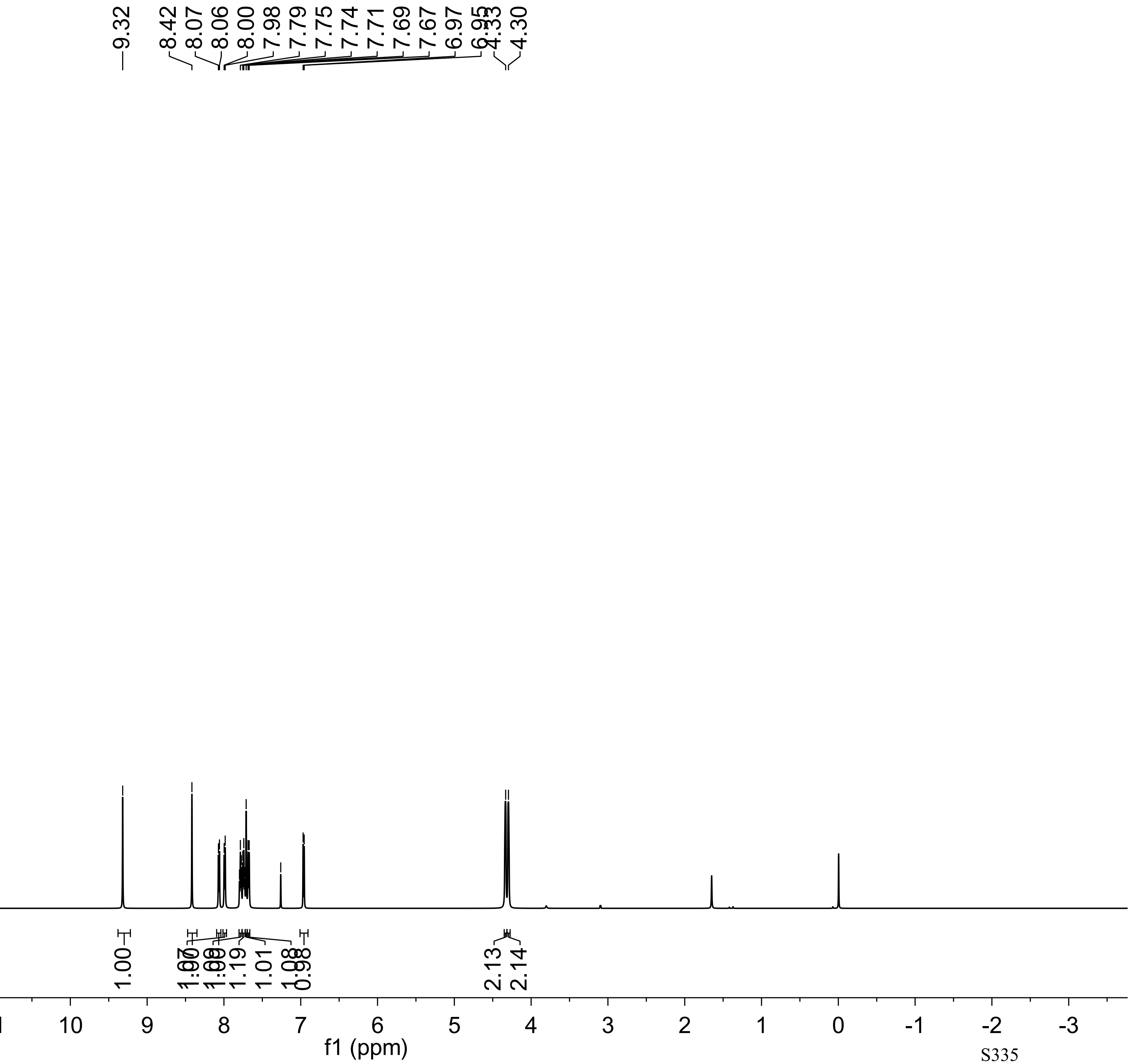

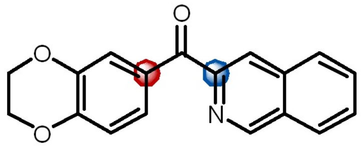

A19

	Parameter	Value
1	Title	CFM-A5
2	Comment	
3	Origin	Bruker BioSpin GmbH
4	Owner	nmrsu
5	Site	
6	Spectrometer	Avance NEO 600
7	Author	
8	Solvent	CDCl3
9	Temperature	298.0
10	Pulse Sequence	zgpg30
11	Experiment	1D
12	Number of Scans	128
13	Receiver Gain	101
14	Relaxation Delay	2.0000
15	Pulse Width	12.0000
16	Acquisition Time	0.9175
17	Acquisition Date	2019-12-09T23:45:00
18	Modification Date	2019-12-10T11:38:09
19	Spectrometer Frequency	150.91
20	Spectral Width	35714.3
21	Lowest Frequency	-2773.6
22	Nucleus	13C
23	Acquired Size	32768
24	Spectral Size	32768

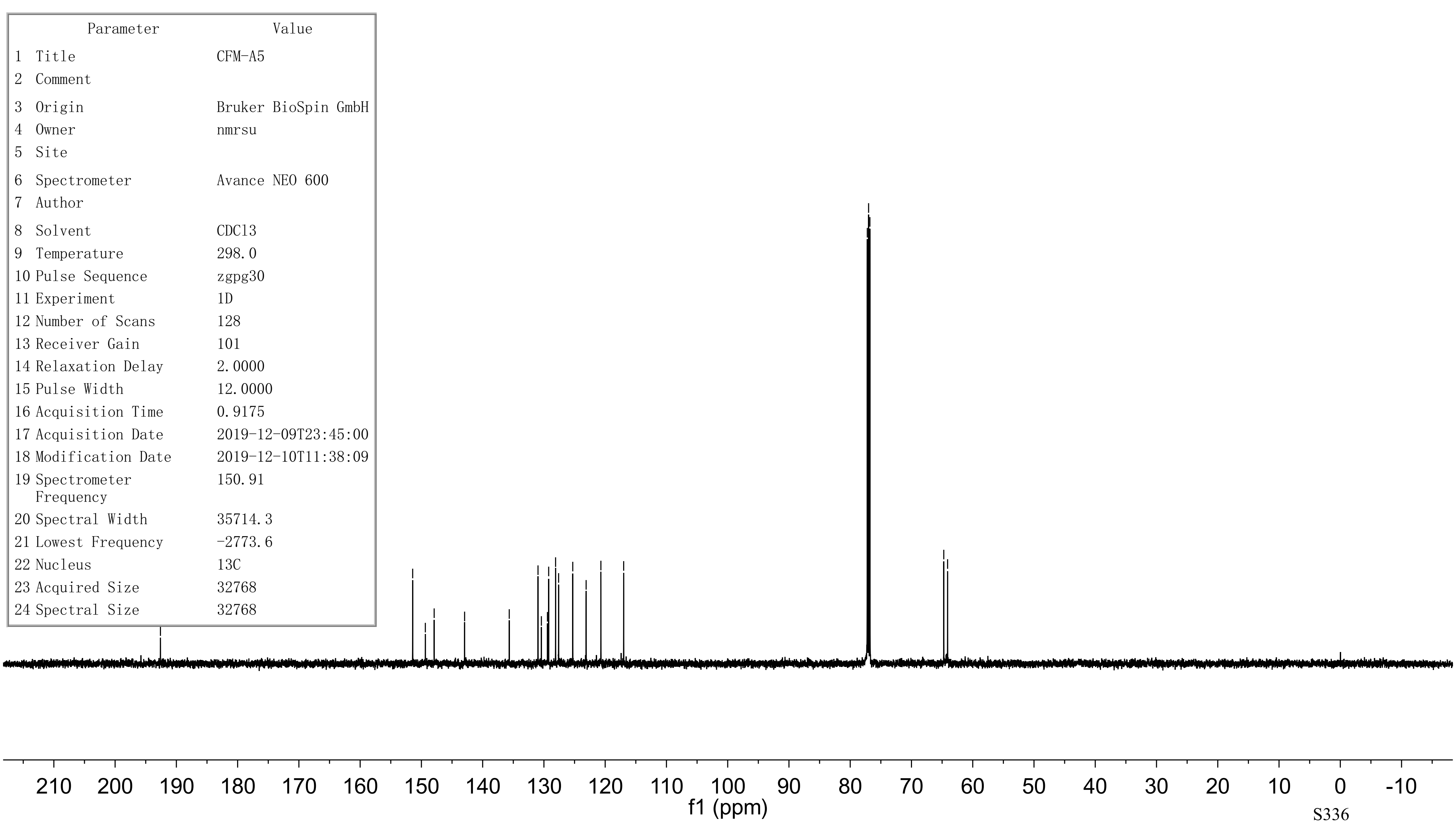

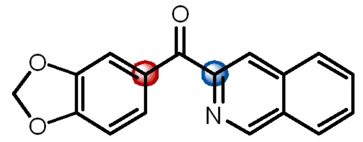

A20

Parameter	Value
1 Title	CFM-A6
2 Comment	
3 Origin	Bruker BioSpin GmbH
4 Owner	nmrsu
5 Site	
6 Spectrometer	Avance NEO 600
7 Author	
8 Solvent	CDCl3
9 Temperature	296.6
10 Pulse Sequence	zg30
11 Experiment	1D
12 Number of Scans	16
13 Receiver Gain	101
14 Relaxation Delay	1.0000
15 Pulse Width	10.0000
16 Acquisition Time	2.7525
17 Acquisition Date	2019-12-09T23:21:56
18 Modification Date	2019-12-10T11:38:08
19 Spectrometer Frequency	600.15
20 Spectral Width	11904.8
21 Lowest Frequency	-2261.1
22 Nucleus	1H
23 Acquired Size	32768
24 Spectral Size	65536

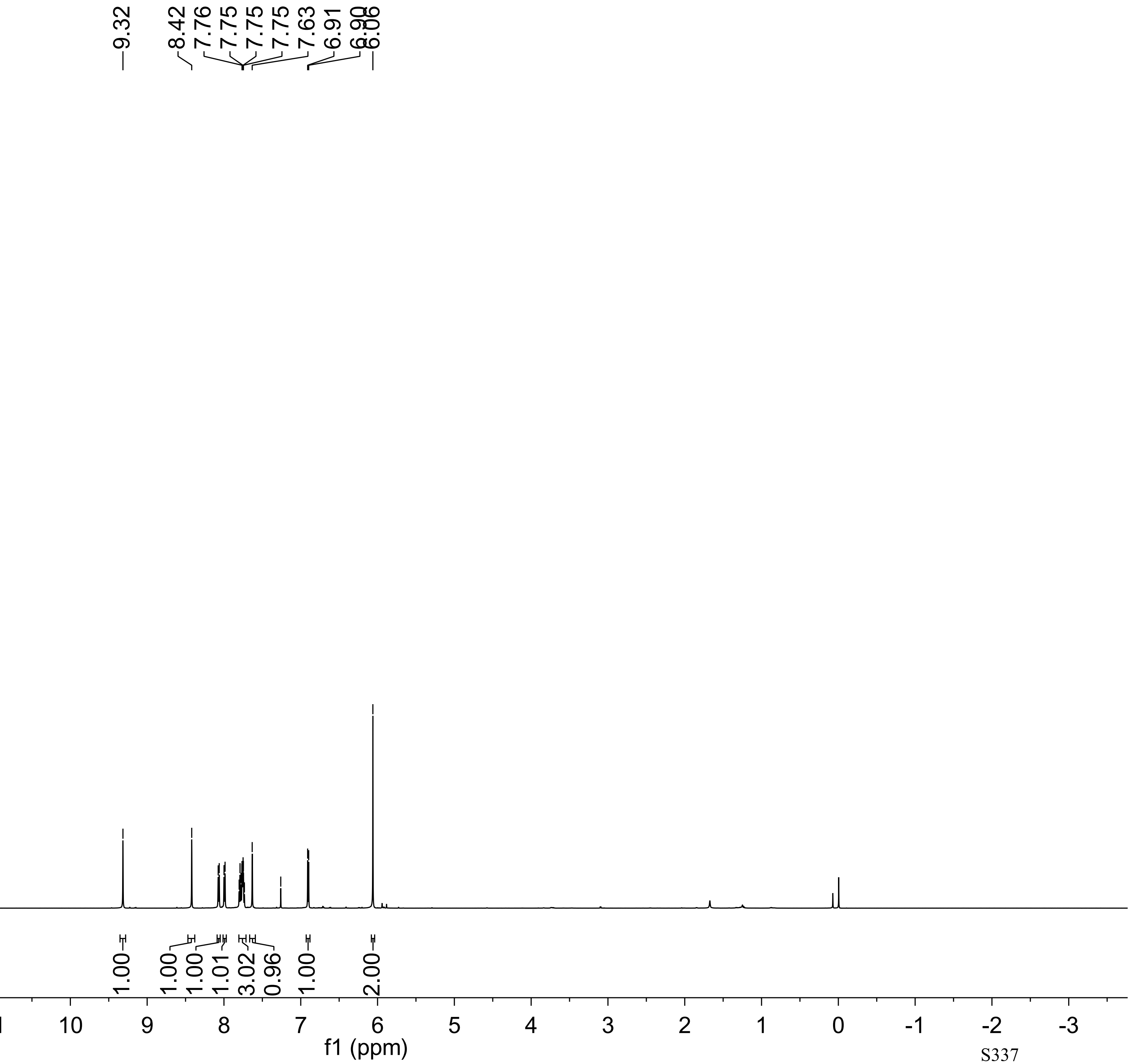

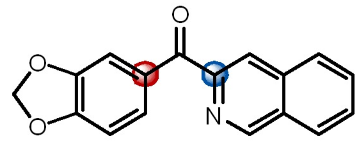

A20

Parameter	Value
1 Title	CFM-A6
2 Comment	
3 Origin	Bruker BioSpin GmbH
4 Owner	nmrsu
5 Site	
6 Spectrometer	Avance NEO 600
7 Author	
8 Solvent	CDCl3
9 Temperature	297.4
10 Pulse Sequence	zgpg30
11 Experiment	1D
12 Number of Scans	128
13 Receiver Gain	101
14 Relaxation Delay	2.0000
15 Pulse Width	12.0000
16 Acquisition Time	0.9175
17 Acquisition Date	2019-12-09T23:29:20
18 Modification Date	2019-12-10T11:38:09
19 Spectrometer Frequency	150.91
20 Spectral Width	35714.3
21 Lowest Frequency	-2774.5
22 Nucleus	13C
23 Acquired Size	32768
24 Spectral Size	32768

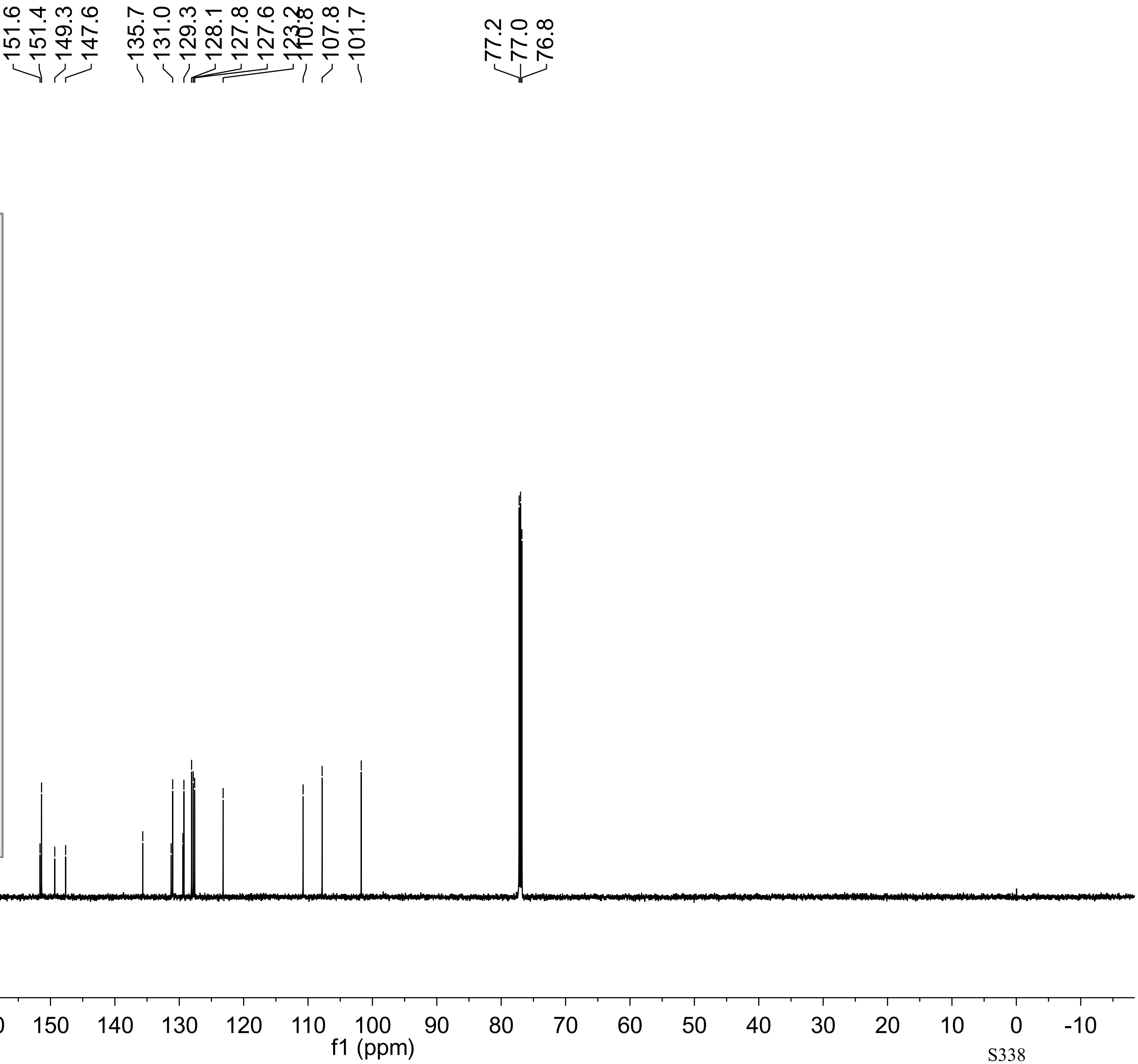

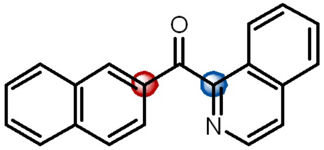

A21

Parameter	Value
1 Title	CFM-F3-1
2 Comment	
3 Origin	Bruker BioSpin GmbH
4 Owner	nmrsu
5 Site	
6 Spectrometer	Avance NEO 600
7 Author	
8 Solvent	CDCl3
9 Temperature	296.5
10 Pulse Sequence	zg30
11 Experiment	1D
12 Number of Scans	8
13 Receiver Gain	101
14 Relaxation Delay	1.0000
15 Pulse Width	10.0000
16 Acquisition Time	2.7525
17 Acquisition Date	2020-03-19T18:38:18
18 Modification Date	2020-03-19T18:51:44
19 Spectrometer Frequency	600.15
20 Spectral Width	11904.8
21 Lowest Frequency	-2260.8
22 Nucleus	1H
23 Acquired Size	32768
24 Spectral Size	65536

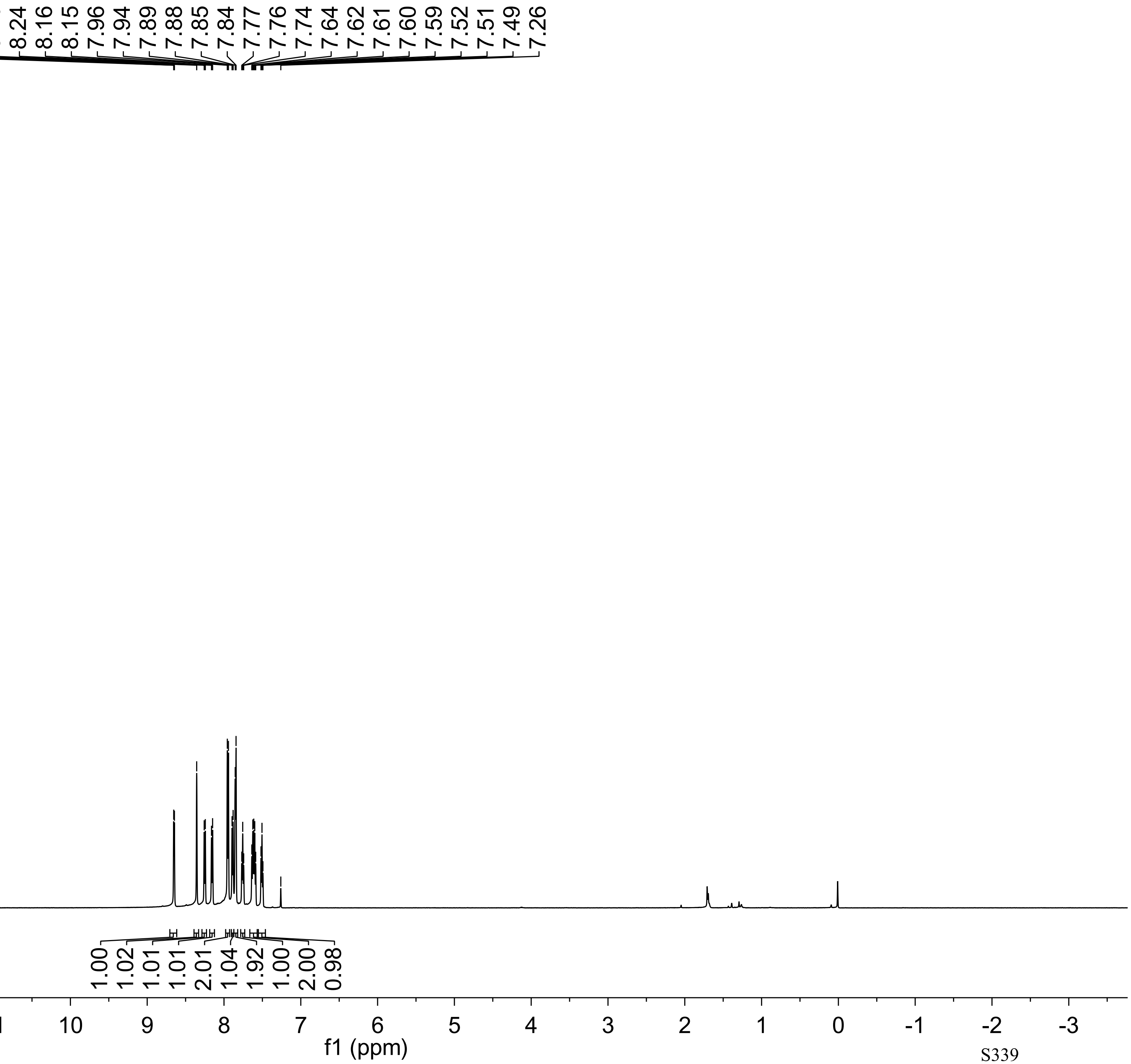

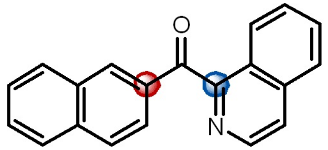

A21

	Parameter	Value
1	Title	CFM-F3-1
2	Comment	
3	Origin	Bruker BioSpin GmbH
4	Owner	nmrsu
5	Site	
6	Spectrometer	Avance NEO 600
7	Author	
8	Solvent	CDCl3
9	Temperature	296.8
10	Pulse Sequence	zgpg30
11	Experiment	1D
12	Number of Scans	128
13	Receiver Gain	101
14	Relaxation Delay	2.0000
15	Pulse Width	12.0000
16	Acquisition Time	0.9175
17	Acquisition Date	2020-03-19T18:46:04
18	Modification Date	2020-03-19T18:51:44
19	Spectrometer Frequency	150.91
20	Spectral Width	35714.3
21	Lowest Frequency	-2778.0
22	Nucleus	13C
23	Acquired Size	32768
24	Spectral Size	32768

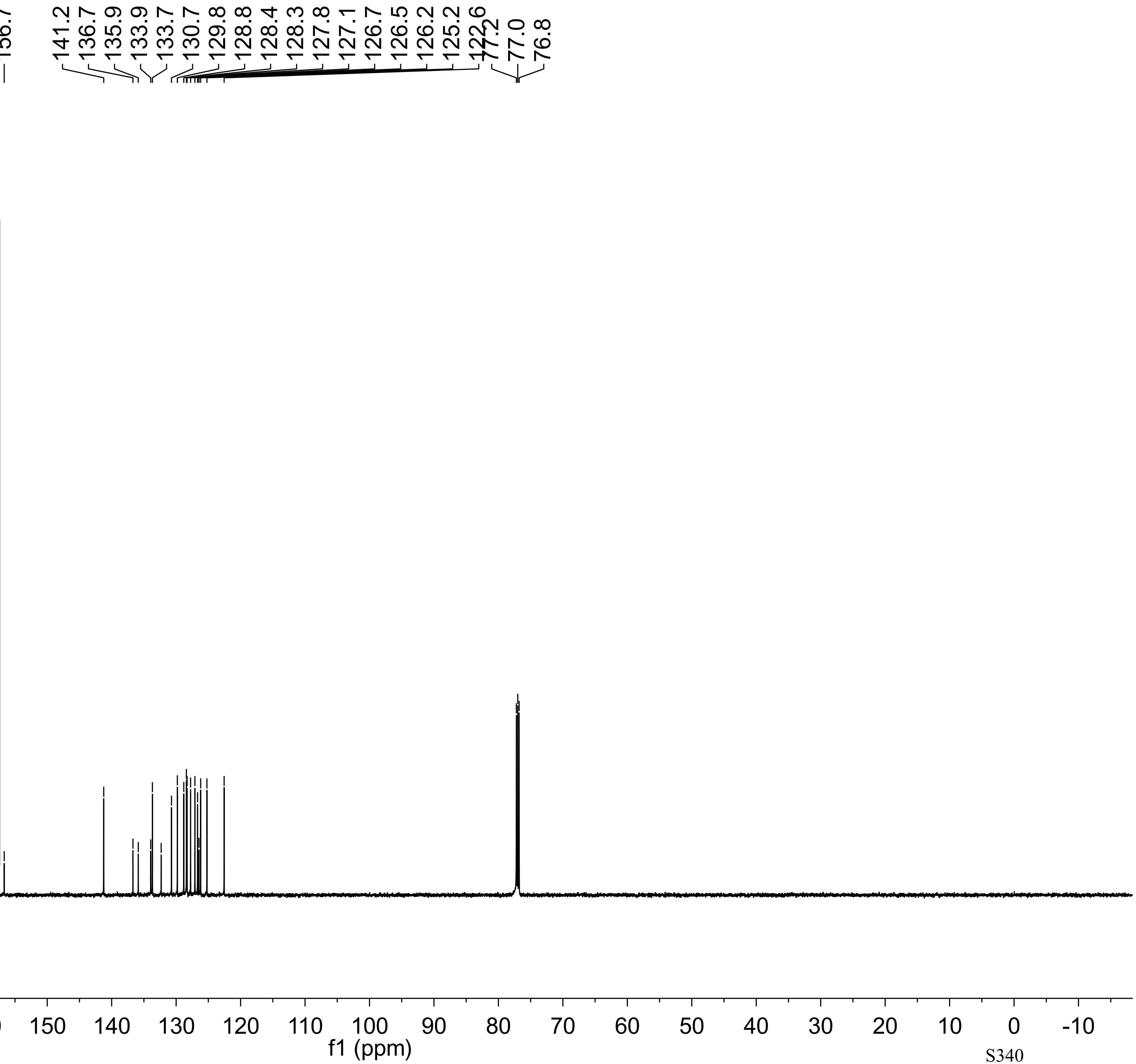

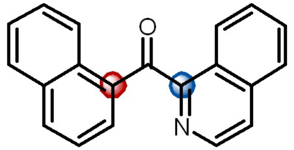

A22

Parameter	Value
1 Title	CFM-6-48-K
2 Comment	
3 Origin	Bruker BioSpin GmbH
4 Owner	nmrsu
5 Site	
6 Spectrometer	Avance Neo 400M
7 Author	
8 Solvent	CDCl3
9 Temperature	298.1
10 Pulse Sequence	zg30
11 Experiment	1D
12 Number of Scans	9
13 Receiver Gain	101
14 Spectrometer Frequency	400.18
15 Spectral Width	8196.7
16 Lowest Frequency	-1636.9
17 Nucleus	1H
18 Acquired Size	32768
19 Spectral Size	65536

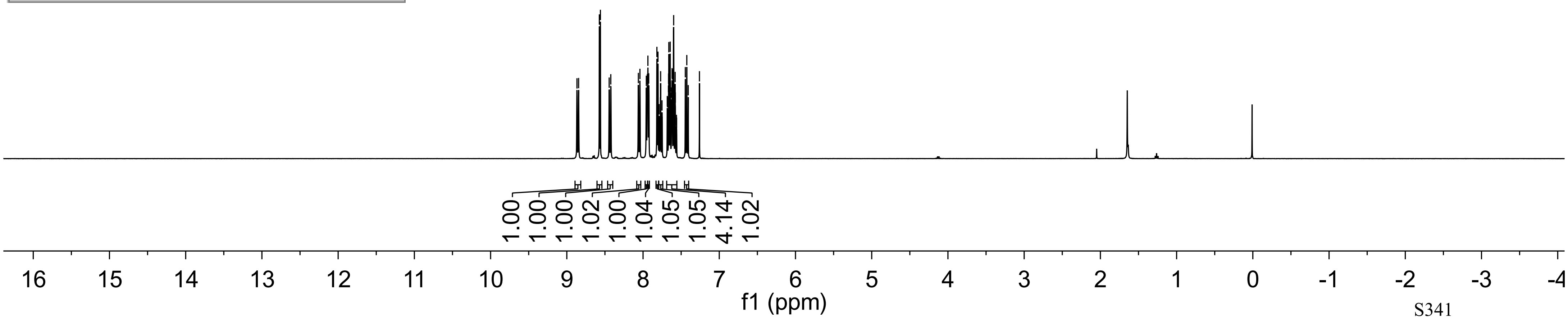

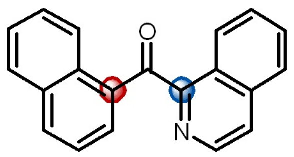

A22

	Parameter	Value
1	Title	CFM-6-48-K
2	Comment	
3	Origin	Bruker BioSpin GmbH
4	Owner	nmrsu
5	Site	
6	Spectrometer	Avance Neo 400M
7	Author	
8	Solvent	CDCl3
9	Temperature	298.2
10	Pulse Sequence	zgpg30
11	Experiment	1D
12	Number of Scans	62
13	Receiver Gain	32
14	Spectrometer Frequency	100.63
15	Spectral Width	23809.5
16	Lowest Frequency	-1848.6
17	Nucleus	13C
18	Acquired Size	32768
19	Spectral Size	32768

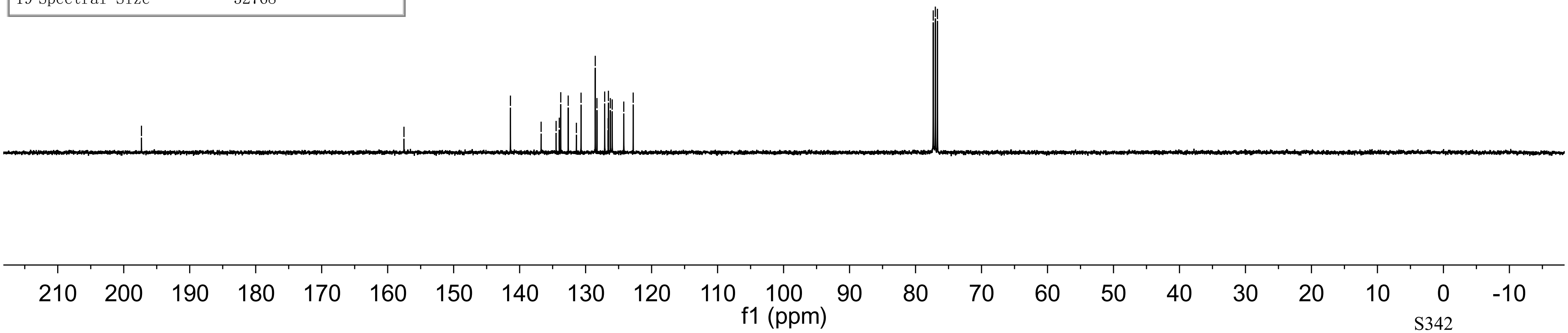

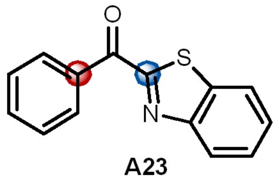

A23

Parameter	Value
1 Title	CFM-D1
2 Comment	
3 Origin	Bruker BioSpin GmbH
4 Owner	nmrsu
5 Site	
6 Spectrometer	Avance NEO 600
7 Author	
8 Solvent	CDCl3
9 Temperature	297.0
10 Pulse Sequence	zg30
11 Experiment	1D
12 Number of Scans	16
13 Receiver Gain	101
14 Relaxation Delay	1.0000
15 Pulse Width	10.0000
16 Acquisition Time	2.7525
17 Acquisition Date	2019-12-10T06:26:27
18 Modification Date	2019-12-10T11:38:14
19 Spectrometer Frequency	600.15
20 Spectral Width	11904.8
21 Lowest Frequency	-2261.3
22 Nucleus	1H
23 Acquired Size	32768
24 Spectral Size	65536

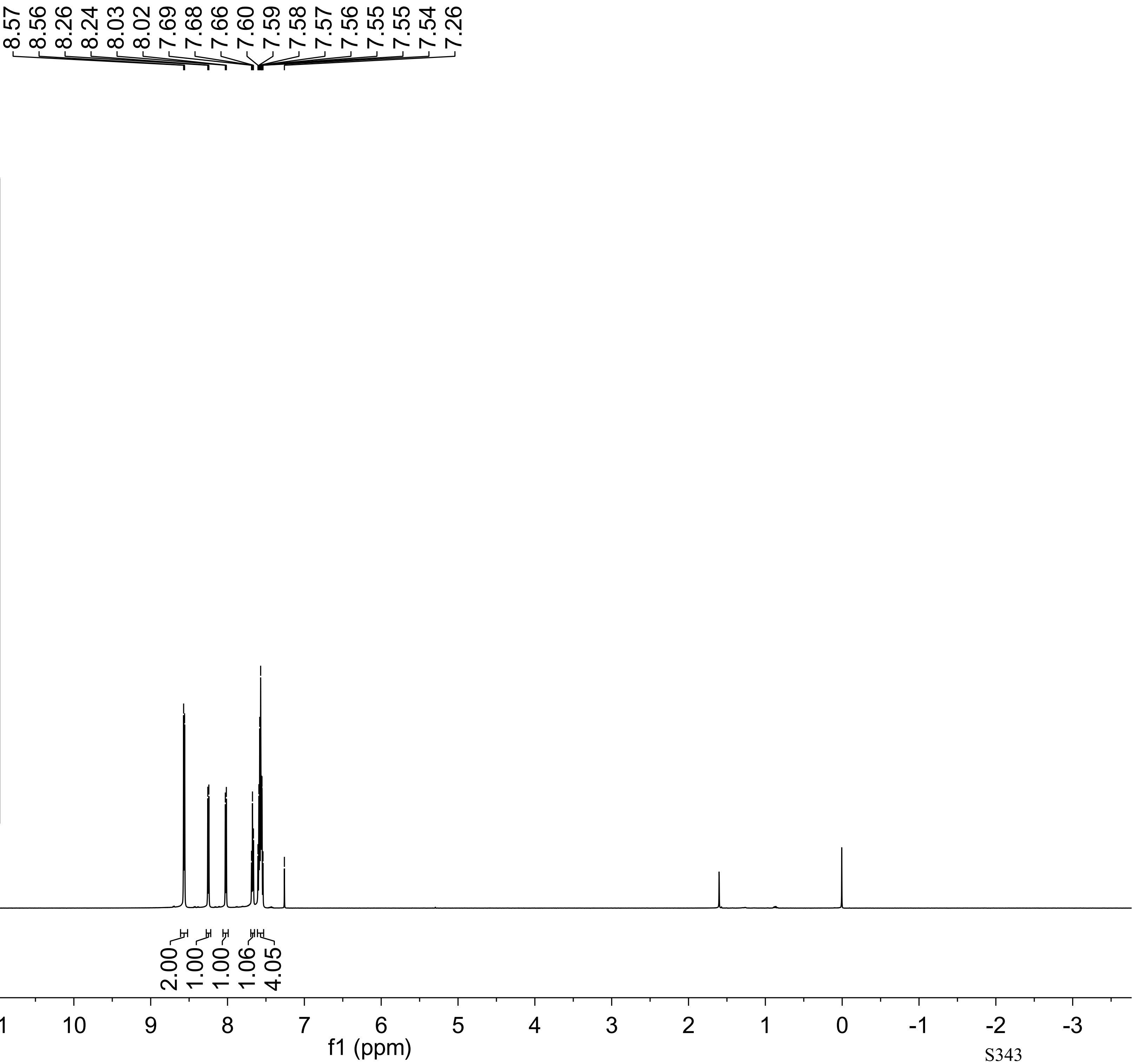

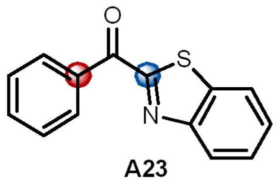

A23

	Parameter	Value
1	Title	CFM-D1
2	Comment	
3	Origin	Bruker BioSpin GmbH
4	Owner	nmrsu
5	Site	
6	Spectrometer	Avance NEO 600
7	Author	
8	Solvent	CDCl3
9	Temperature	297.9
10	Pulse Sequence	zgpg30
11	Experiment	1D
12	Number of Scans	128
13	Receiver Gain	101
14	Relaxation Delay	2.0000
15	Pulse Width	12.0000
16	Acquisition Time	0.9175
17	Acquisition Date	2019-12-10T06:34:28
18	Modification Date	2019-12-10T11:38:15
19	Spectrometer Frequency	150.91
20	Spectral Width	35714.3
21	Lowest Frequency	-2774.7
22	Nucleus	13C
23	Acquired Size	32768
24	Spectral Size	32768

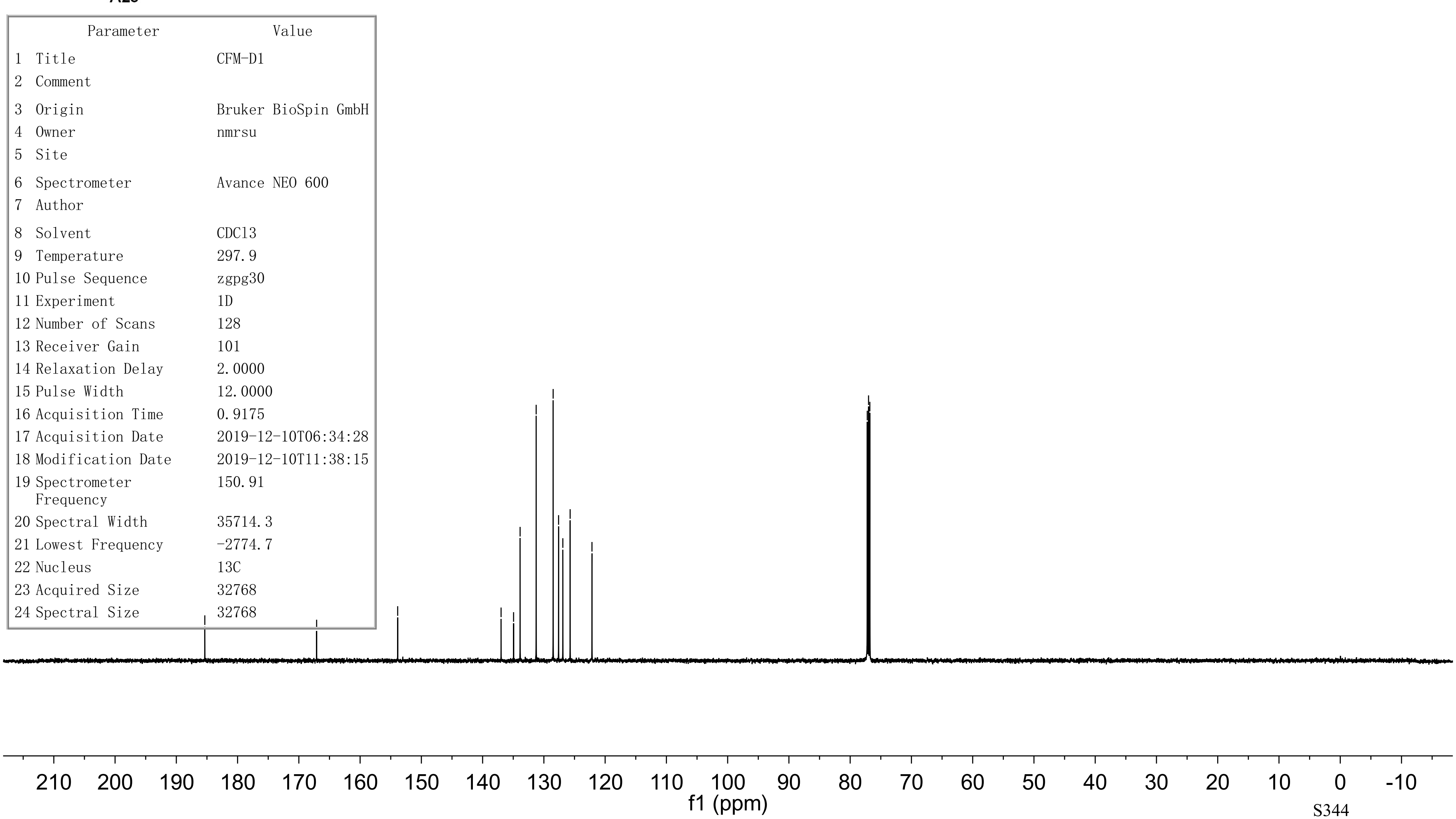

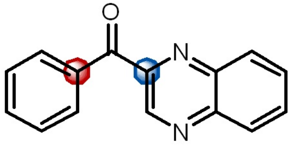

A24

Parameter	Value
1 Title	CFM-F4-1
2 Comment	
3 Origin	Bruker BioSpin GmbH
4 Owner	nmrsu
5 Site	
6 Spectrometer	Avance NEO 600
7 Author	
8 Solvent	CDCl3
9 Temperature	295.8
10 Pulse Sequence	zg30
11 Experiment	1D
12 Number of Scans	8
13 Receiver Gain	101
14 Relaxation Delay	1.0000
15 Pulse Width	10.0000
16 Acquisition Time	2.7525
17 Acquisition Date	2020-03-19T15:54:50
18 Modification Date	2020-03-19T18:07:54
19 Spectrometer Frequency	600.15
20 Spectral Width	11904.8
21 Lowest Frequency	-2260.8
22 Nucleus	1H
23 Acquired Size	32768
24 Spectral Size	65536

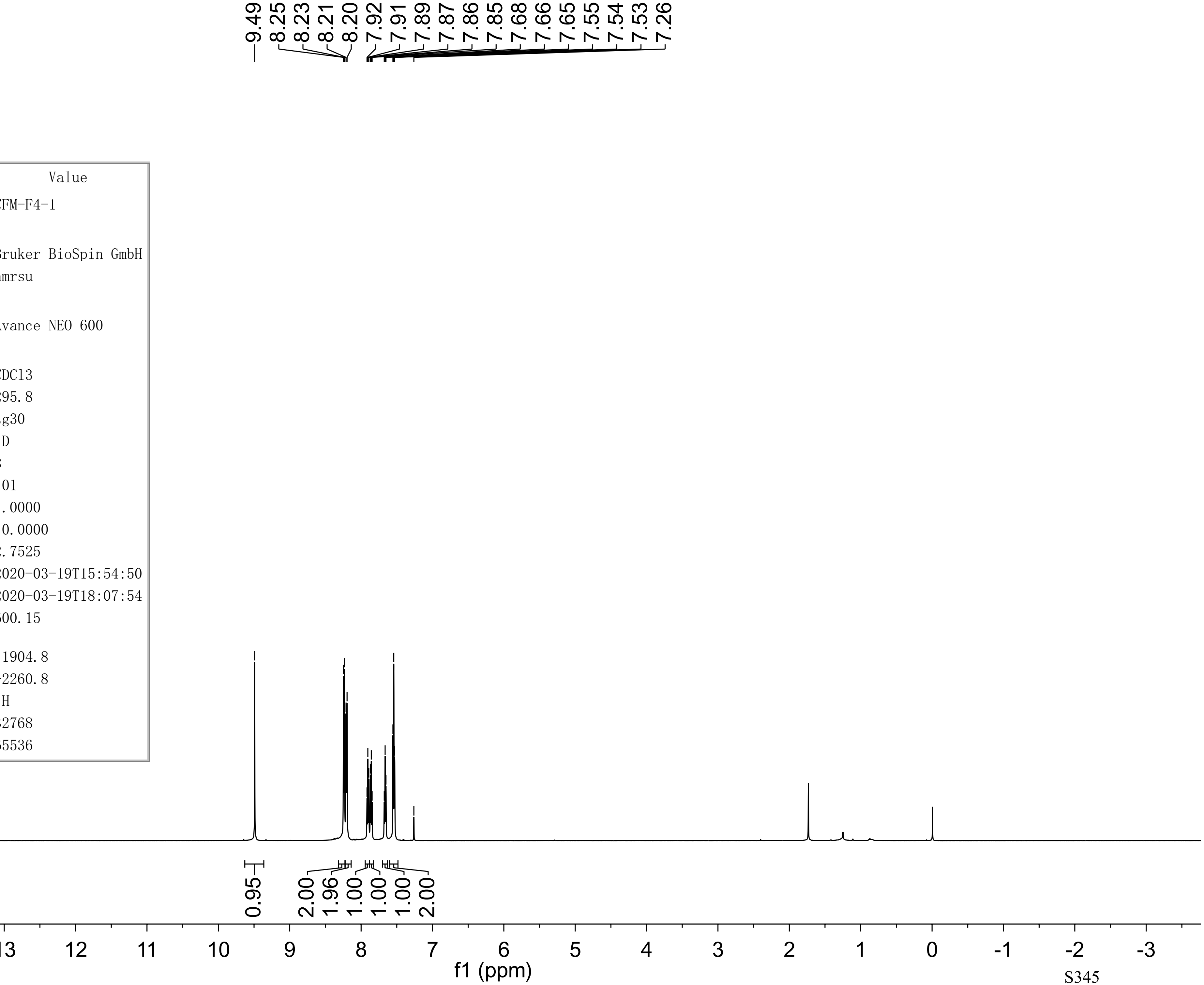

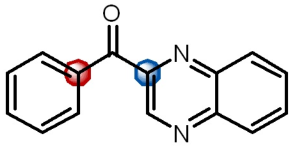

A24

	Parameter	Value
1	Title	CFM-F4-1
2	Comment	
3	Origin	Bruker BioSpin GmbH
4	Owner	nmrsu
5	Site	
6	Spectrometer	Avance NEO 600
7	Author	
8	Solvent	CDCl3
9	Temperature	296.7
10	Pulse Sequence	zgpg30
11	Experiment	1D
12	Number of Scans	29
13	Receiver Gain	101
14	Relaxation Delay	2.0000
15	Pulse Width	12.0000
16	Acquisition Time	0.9175
17	Acquisition Date	2020-03-19T15:57:48
18	Modification Date	2020-03-19T18:07:54
19	Spectrometer Frequency	150.91
20	Spectral Width	35714.3
21	Lowest Frequency	-2775.7
22	Nucleus	13C
23	Acquired Size	32768
24	Spectral Size	32768

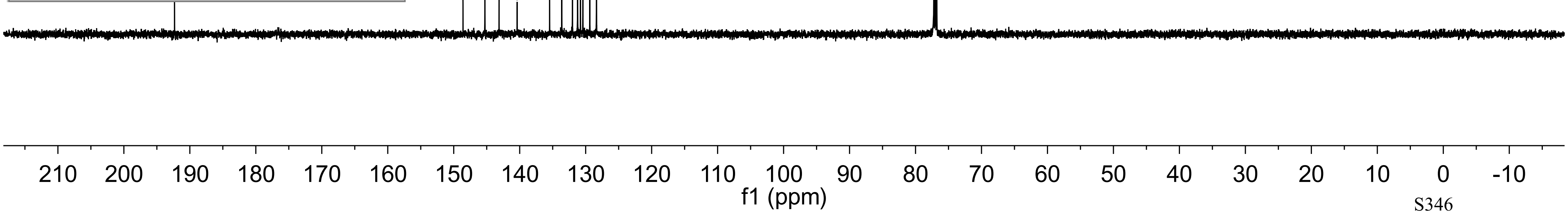

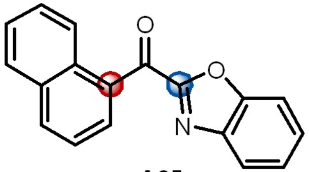

A25

Parameter	Value
1 Title	CFM-D4
2 Comment	
3 Origin	Bruker BioSpin GmbH
4 Owner	nmrsu
5 Site	
6 Spectrometer	Avance NEO 600
7 Author	
8 Solvent	CDCl3
9 Temperature	297.8
10 Pulse Sequence	zg30
11 Experiment	1D
12 Number of Scans	8
13 Receiver Gain	101
14 Relaxation Delay	1.0000
15 Pulse Width	10.0000
16 Acquisition Time	2.7525
17 Acquisition Date	2020-08-05T23:14:34
18 Modification Date	2020-08-06T09:02:02
19 Spectrometer Frequency	600.15
20 Spectral Width	11904.8
21 Lowest Frequency	-2261.1
22 Nucleus	1H
23 Acquired Size	32768
24 Spectral Size	65536

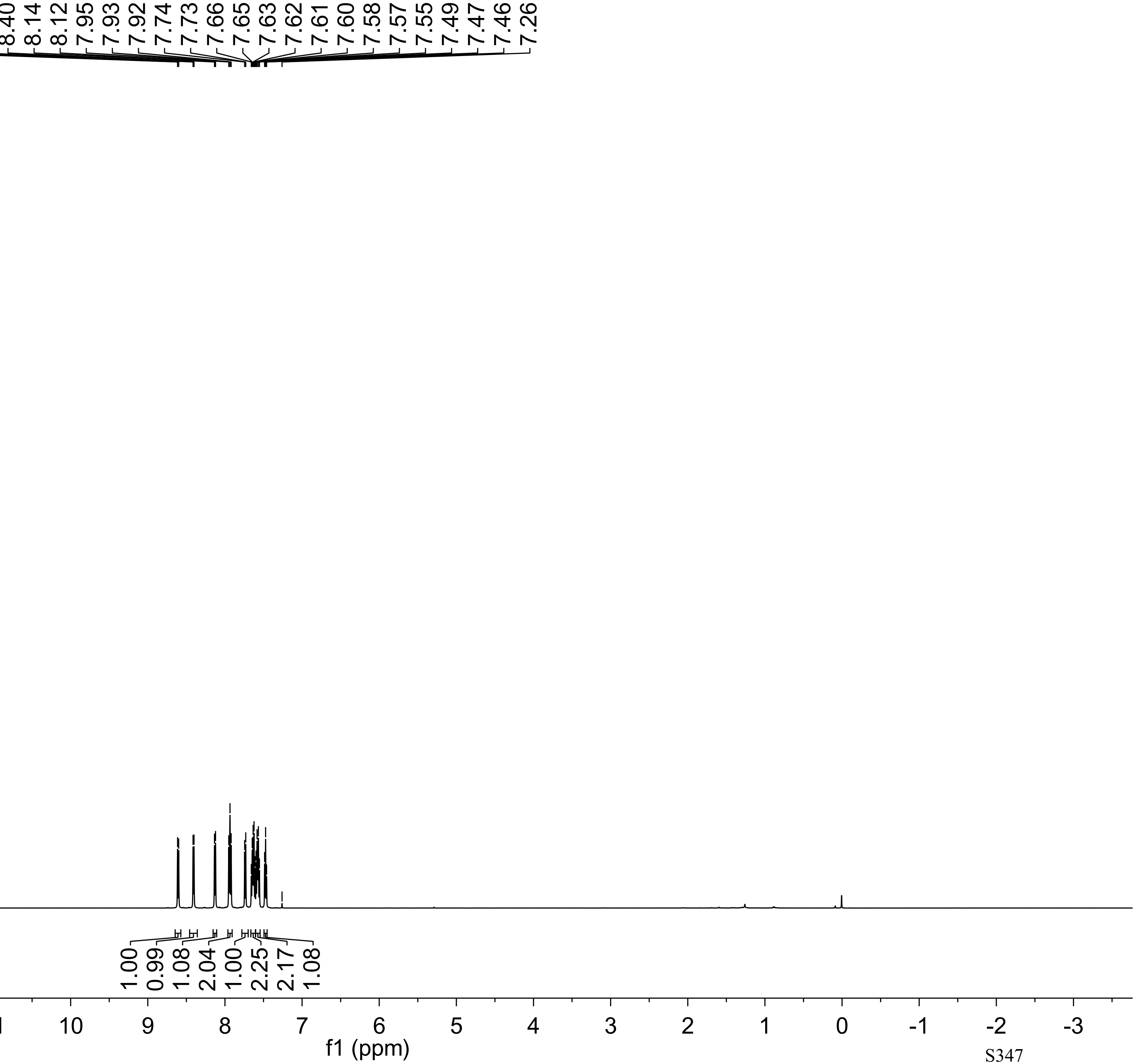

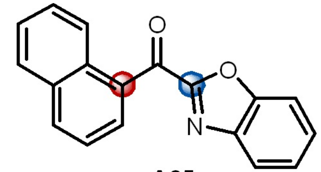

A25

Parameter		Value
1	Title	CFM-D4
2	Comment	
3	Origin	Bruker BioSpin GmbH
4	Owner	nmrsu
5	Site	
6	Spectrometer	Avance NEO 600
7	Author	
8	Solvent	CDCl3
9	Temperature	298.1
10	Pulse Sequence	zgpg30
11	Experiment	1D
12	Number of Scans	128
13	Receiver Gain	101
14	Relaxation Delay	2.0000
15	Pulse Width	12.0000
16	Acquisition Time	0.9175
17	Acquisition Date	2019-12-10T07:17:19
18	Modification Date	2019-12-10T11:38:45
19	Spectrometer Frequency	150.91
20	Spectral Width	35714.3
21	Lowest Frequency	-2774.7
22	Nucleus	13C
23	Acquired Size	32768
24	Spectral Size	32768

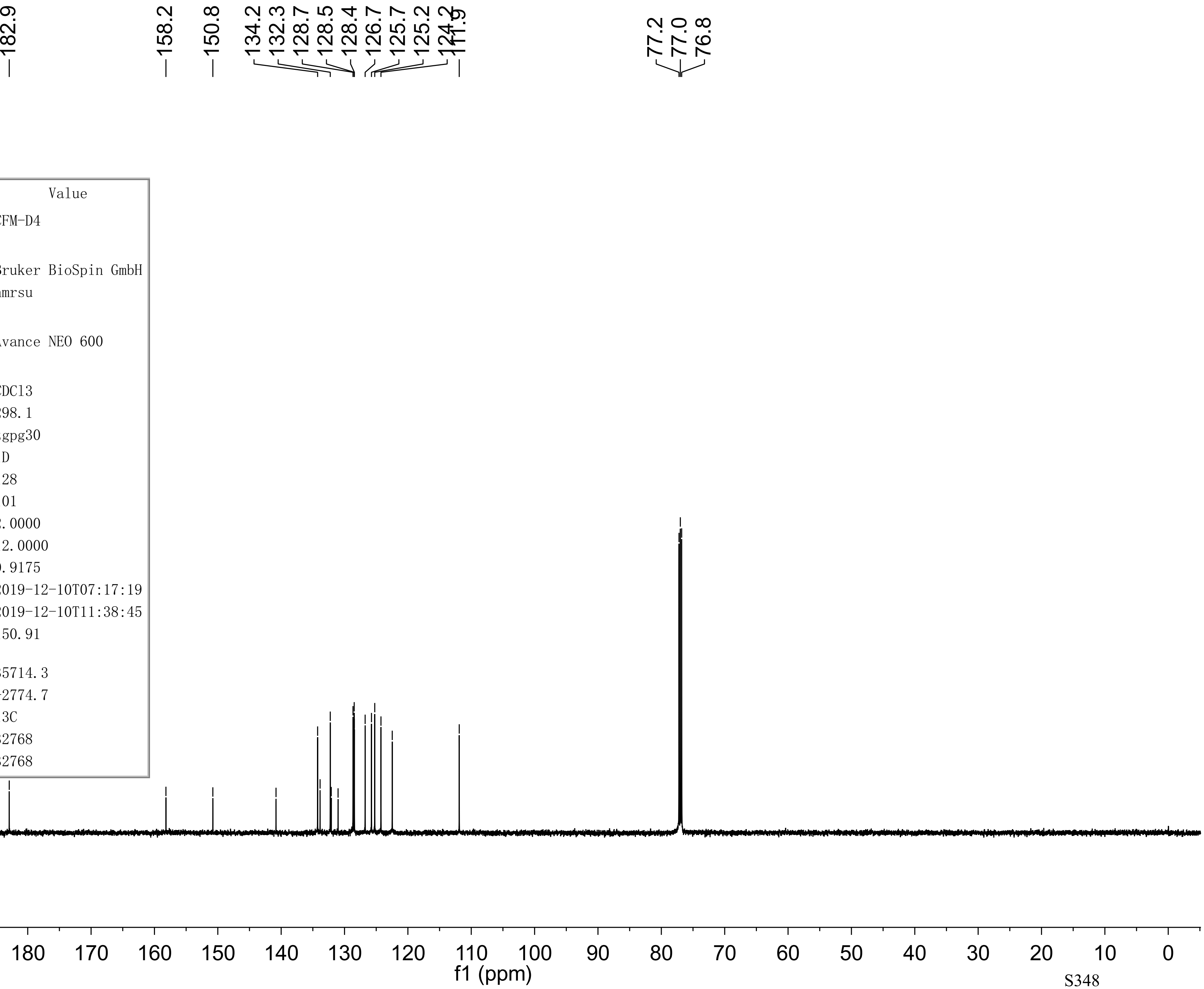

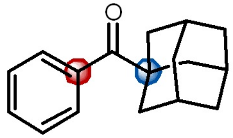

A26

Parameter	Value
1 Title	CFM-G6-4
2 Comment	
3 Origin	Bruker BioSpin GmbH
4 Owner	nmrsu
5 Site	
6 Spectrometer	Avance NEO 600
7 Author	
8 Solvent	CDCl3
9 Temperature	298.1
10 Pulse Sequence	zg30
11 Experiment	1D
12 Number of Scans	8
13 Receiver Gain	64
14 Relaxation Delay	1.0000
15 Pulse Width	10.0000
16 Acquisition Time	2.7525
17 Acquisition Date	2020-08-18T19:44:31
18 Modification Date	2020-08-18T19:54:00
19 Spectrometer Frequency	600.15
20 Spectral Width	11904.8
21 Lowest Frequency	-2261.1
22 Nucleus	1H
23 Acquired Size	32768
24 Spectral Size	65536

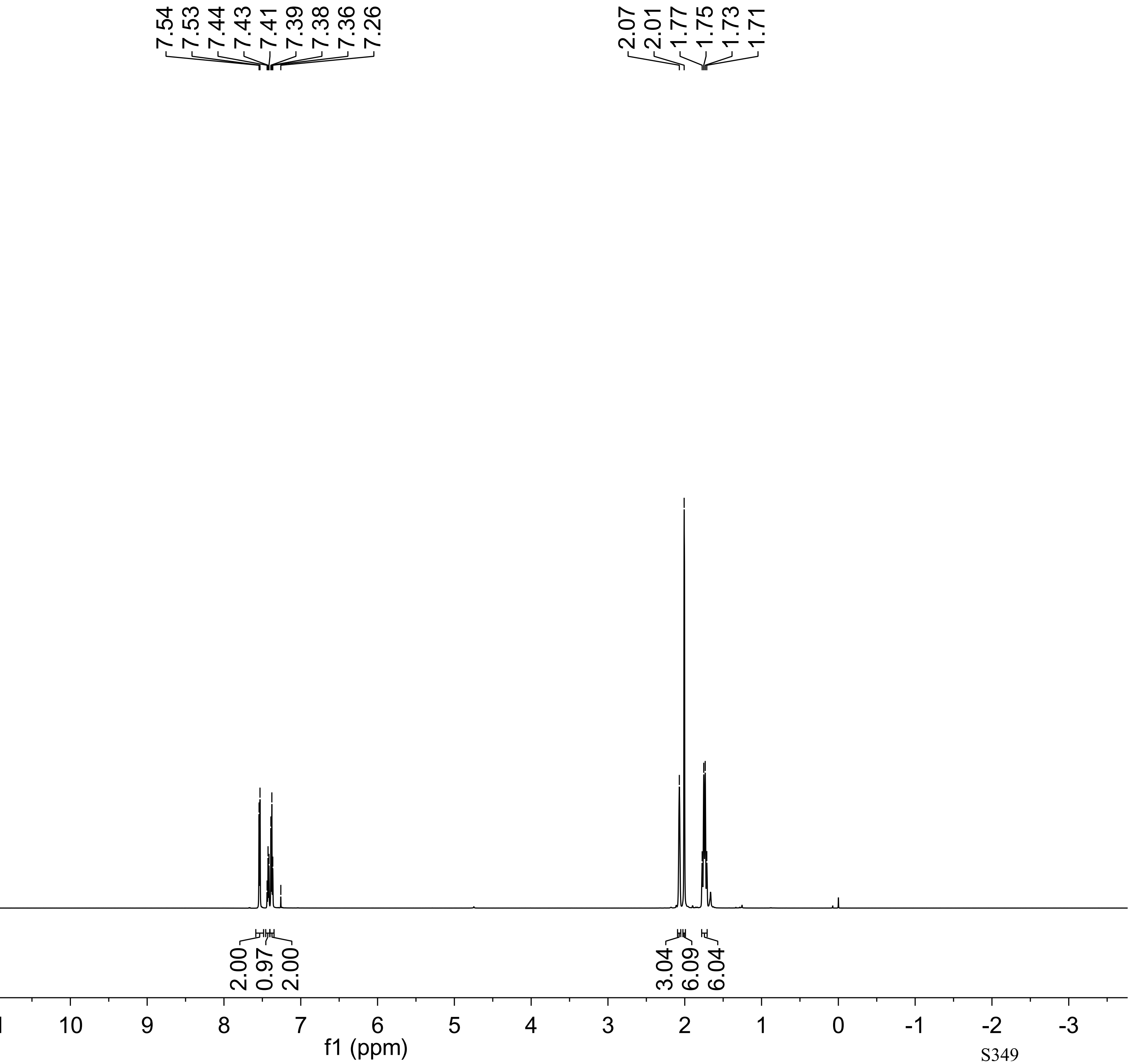

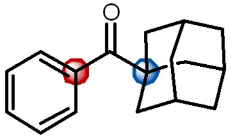

A26

	Parameter	Value
1	Title	CFM-G6-4
2	Comment	
3	Origin	Bruker BioSpin GmbH
4	Owner	nmrsu
5	Site	
6	Spectrometer	Avance NEO 600
7	Author	
8	Solvent	CDCl3
9	Temperature	298.7
10	Pulse Sequence	zgpg30
11	Experiment	1D
12	Number of Scans	16
13	Receiver Gain	101
14	Relaxation Delay	2.0000
15	Pulse Width	12.0000
16	Acquisition Time	0.9175
17	Acquisition Date	2020-08-18T19:46:13
18	Modification Date	2020-08-18T19:54:01
19	Spectrometer Frequency	150.91
20	Spectral Width	35714.3
21	Lowest Frequency	-2766.4
22	Nucleus	13C
23	Acquired Size	32768
24	Spectral Size	32768

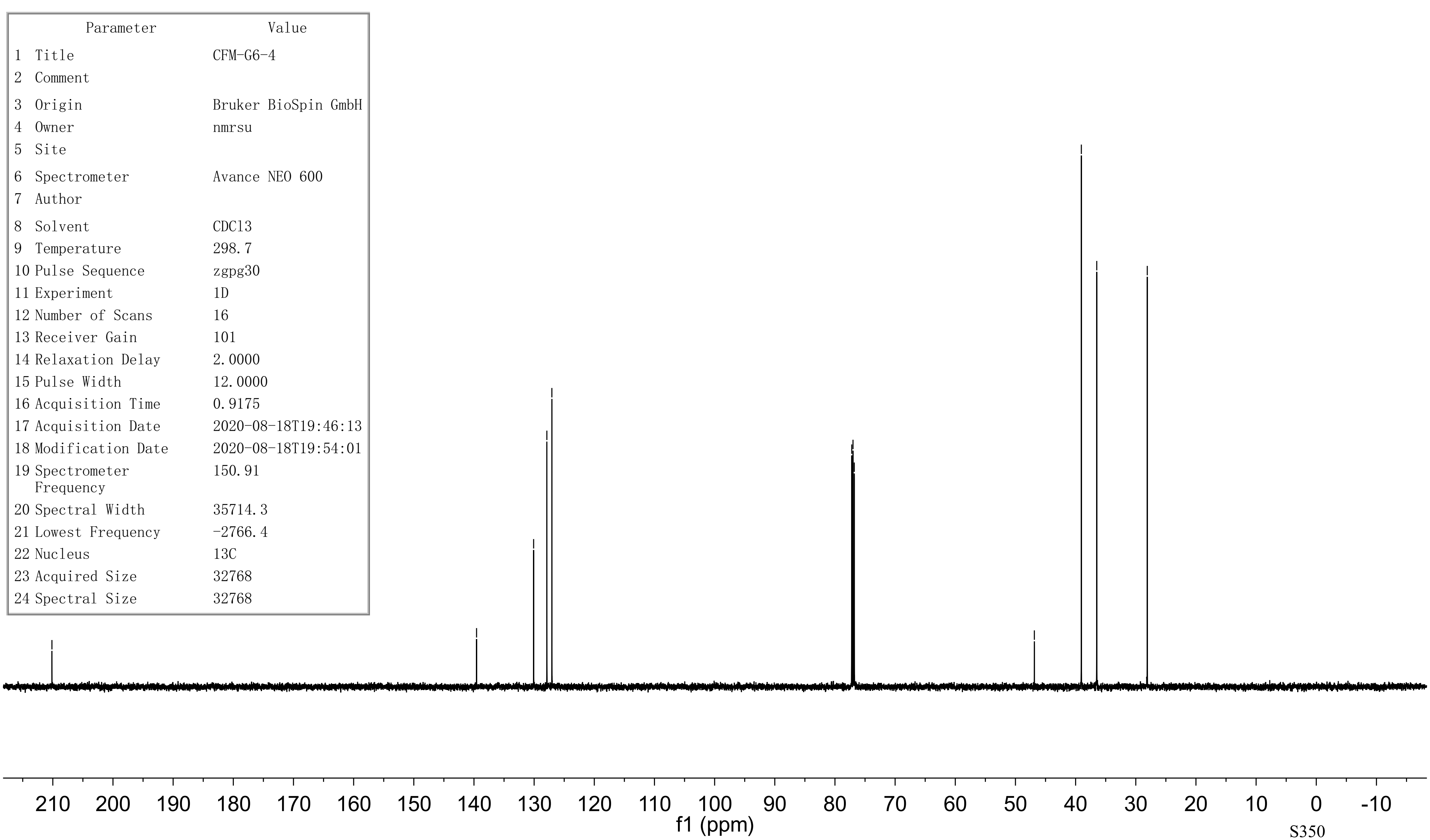

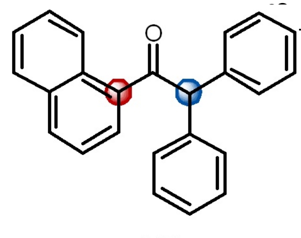

A29

Parameter	Value
1 Title	CFM-B3
2 Comment	
3 Origin	Bruker BioSpin GmbH
4 Owner	nmrsu
5 Site	
6 Spectrometer	Avance NEO 600
7 Author	
8 Solvent	CDCl3
9 Temperature	296.6
10 Pulse Sequence	zg30
11 Experiment	1D
12 Number of Scans	16
13 Receiver Gain	101
14 Relaxation Delay	1.0000
15 Pulse Width	10.0000
16 Acquisition Time	2.7525
17 Acquisition Date	2019-12-09T08:23:22
18 Modification Date	2019-12-09T09:11:57
19 Spectrometer Frequency	600.15
20 Spectral Width	11904.8
21 Lowest Frequency	-2260.9
22 Nucleus	1H
23 Acquired Size	32768
24 Spectral Size	65536

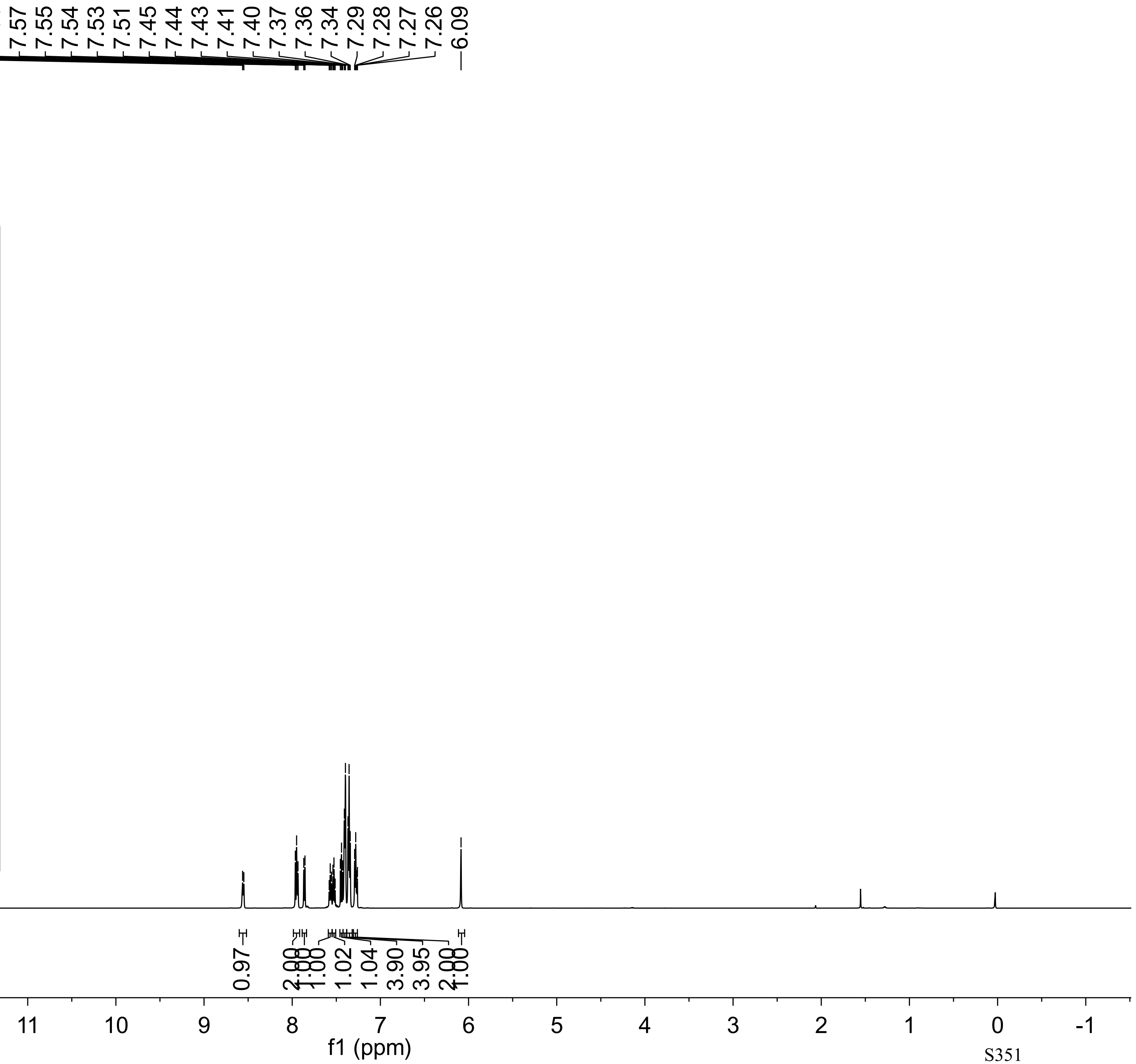

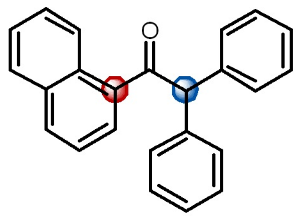

A29

	Parameter	Value
1	Title	CFM-B3
2	Comment	
3	Origin	Bruker BioSpin GmbH
4	Owner	nmrsu
5	Site	
6	Spectrometer	Avance NEO 600
7	Author	
8	Solvent	CDCl3
9	Temperature	297.8
10	Pulse Sequence	zgpg30
11	Experiment	1D
12	Number of Scans	128
13	Receiver Gain	101
14	Relaxation Delay	2.0000
15	Pulse Width	12.0000
16	Acquisition Time	0.9175
17	Acquisition Date	2019-12-09T08:30:51
18	Modification Date	2019-12-09T09:11:57
19	Spectrometer Frequency	150.91
20	Spectral Width	35714.3
21	Lowest Frequency	-2776.8
22	Nucleus	13C
23	Acquired Size	32768
24	Spectral Size	32768

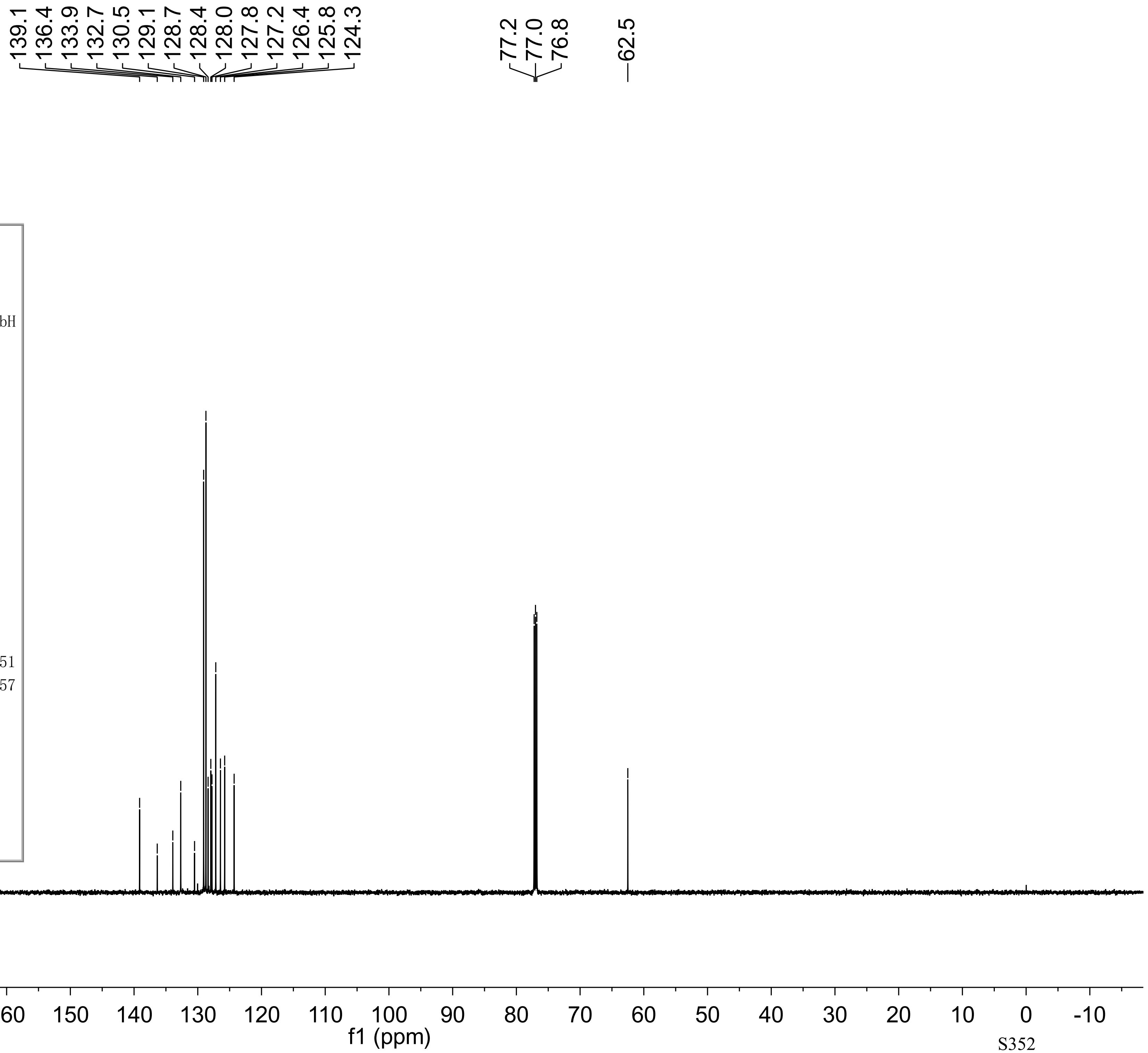

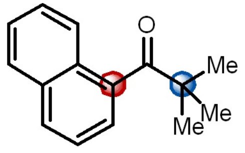

A30

Parameter	Value
1 Title	CFM-G4
2 Comment	
3 Origin	Bruker BioSpin GmbH
4 Owner	nmrsu
5 Site	
6 Spectrometer	Avance NEO 600
7 Author	
8 Solvent	CDCl3
9 Temperature	296.5
10 Pulse Sequence	zg30
11 Experiment	1D
12 Number of Scans	16
13 Receiver Gain	74
14 Relaxation Delay	1.0000
15 Pulse Width	10.0000
16 Acquisition Time	2.7525
17 Acquisition Date	2019-12-28T22:43:42
18 Modification Date	2019-12-29T10:16:04
19 Spectrometer Frequency	600.15
20 Spectral Width	11904.8
21 Lowest Frequency	-2260.9
22 Nucleus	1H
23 Acquired Size	32768
24 Spectral Size	65536

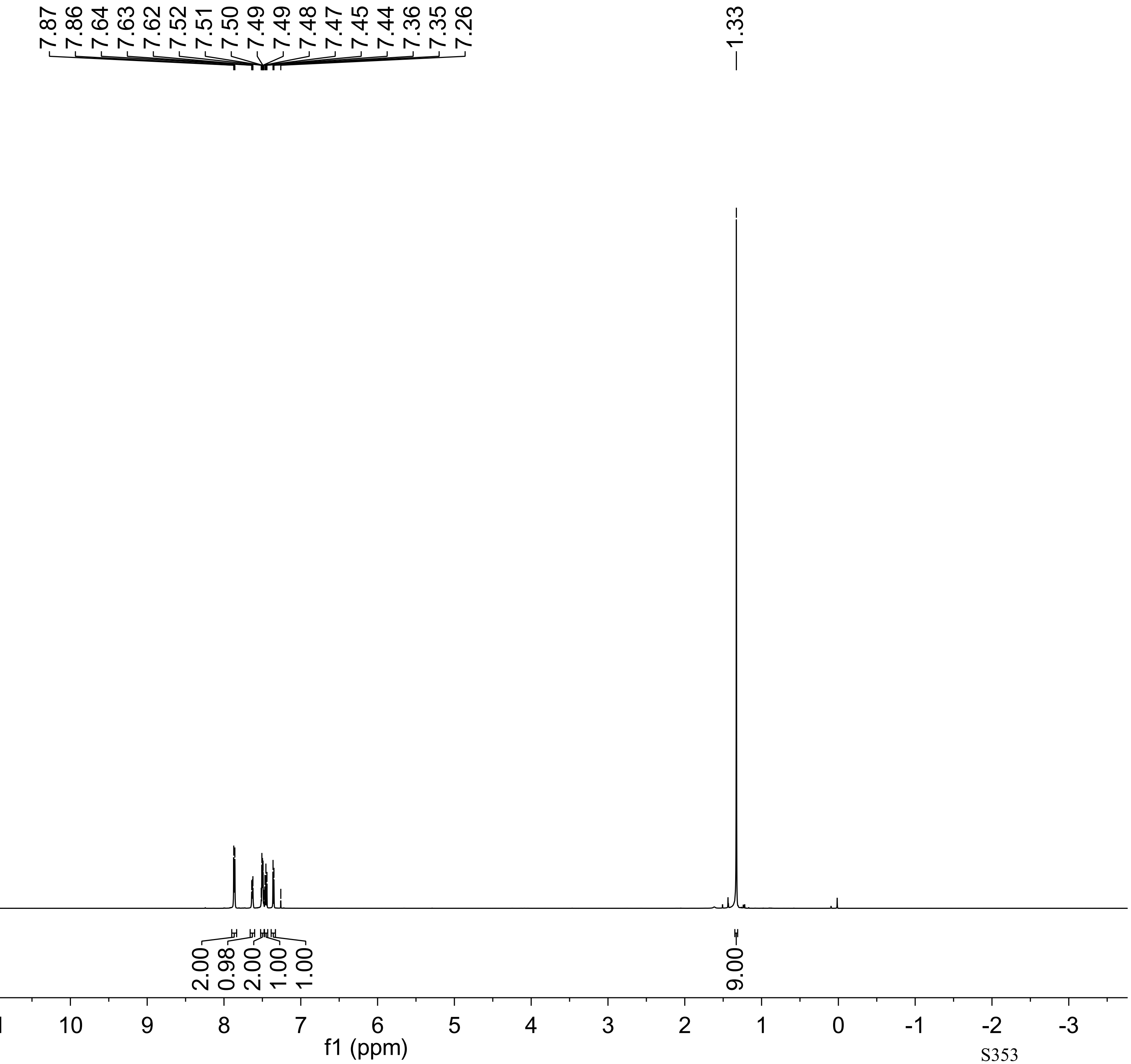

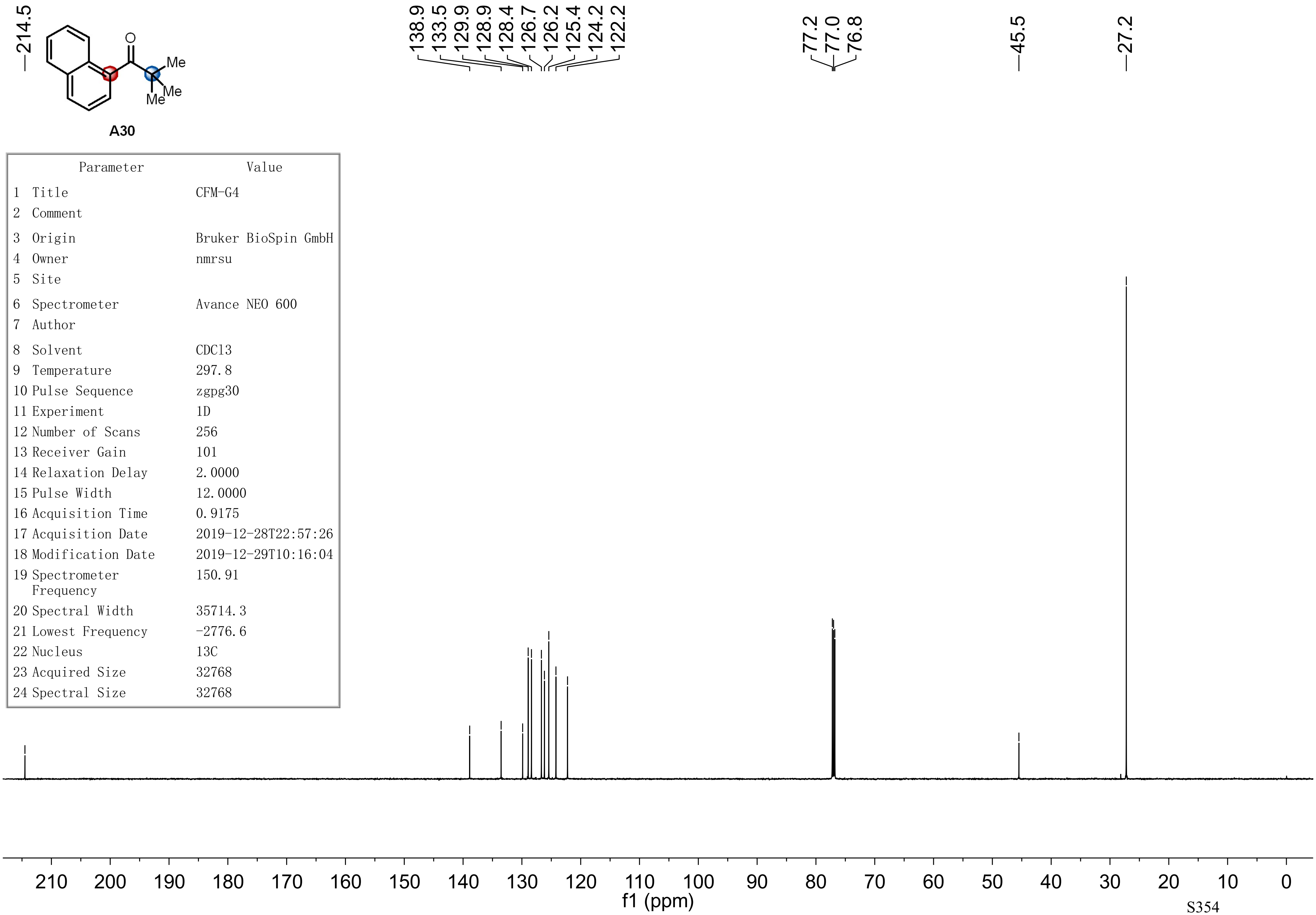

	Parameter	Value
1	Title	CFM-G4
2	Comment	
3	Origin	Bruker BioSpin GmbH
4	Owner	nmrsu
5	Site	
6	Spectrometer	Avance NEO 600
7	Author	
8	Solvent	CDCl3
9	Temperature	297.8
10	Pulse Sequence	zgpg30
11	Experiment	1D
12	Number of Scans	256
13	Receiver Gain	101
14	Relaxation Delay	2.0000
15	Pulse Width	12.0000
16	Acquisition Time	0.9175
17	Acquisition Date	2019-12-28T22:57:26
18	Modification Date	2019-12-29T10:16:04
19	Spectrometer Frequency	150.91
20	Spectral Width	35714.3
21	Lowest Frequency	-2776.6
22	Nucleus	13C
23	Acquired Size	32768
24	Spectral Size	32768

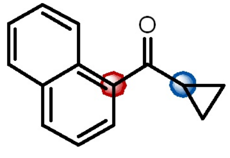

A31

Parameter	Value
1 Title	CFM-G3
2 Comment	
3 Origin	Bruker BioSpin GmbH
4 Owner	nmrsu
5 Site	
6 Spectrometer	Avance NEO 600
7 Author	
8 Solvent	CDCl3
9 Temperature	296.4
10 Pulse Sequence	zg30
11 Experiment	1D
12 Number of Scans	16
13 Receiver Gain	74
14 Relaxation Delay	1.0000
15 Pulse Width	10.0000
16 Acquisition Time	2.7525
17 Acquisition Date	2019-12-28T22:25:07
18 Modification Date	2019-12-29T10:16:04
19 Spectrometer Frequency	600.15
20 Spectral Width	11904.8
21 Lowest Frequency	-2260.8
22 Nucleus	1H
23 Acquired Size	32768
24 Spectral Size	65536

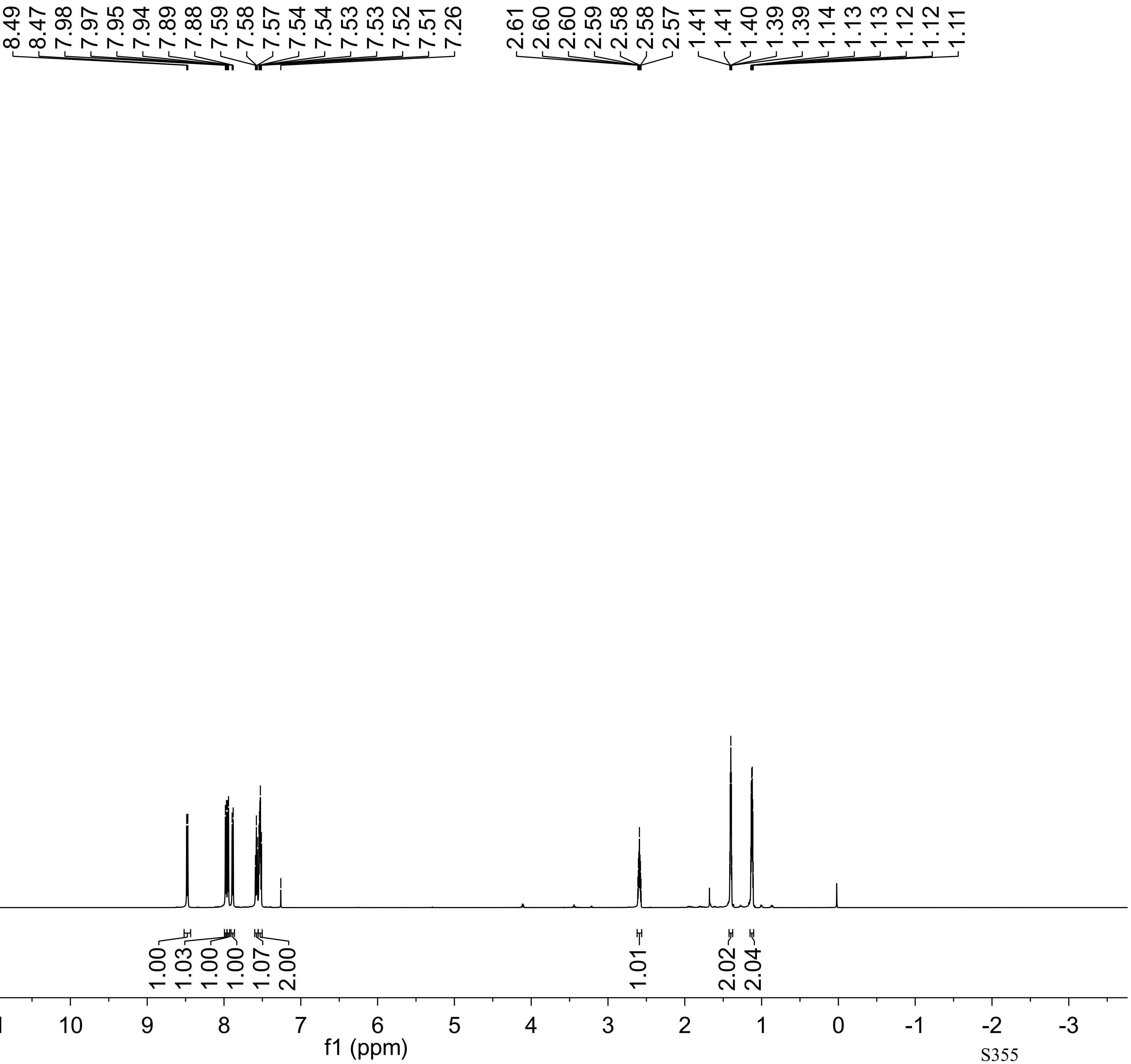

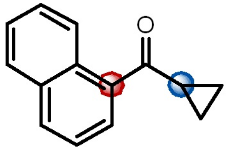

A31

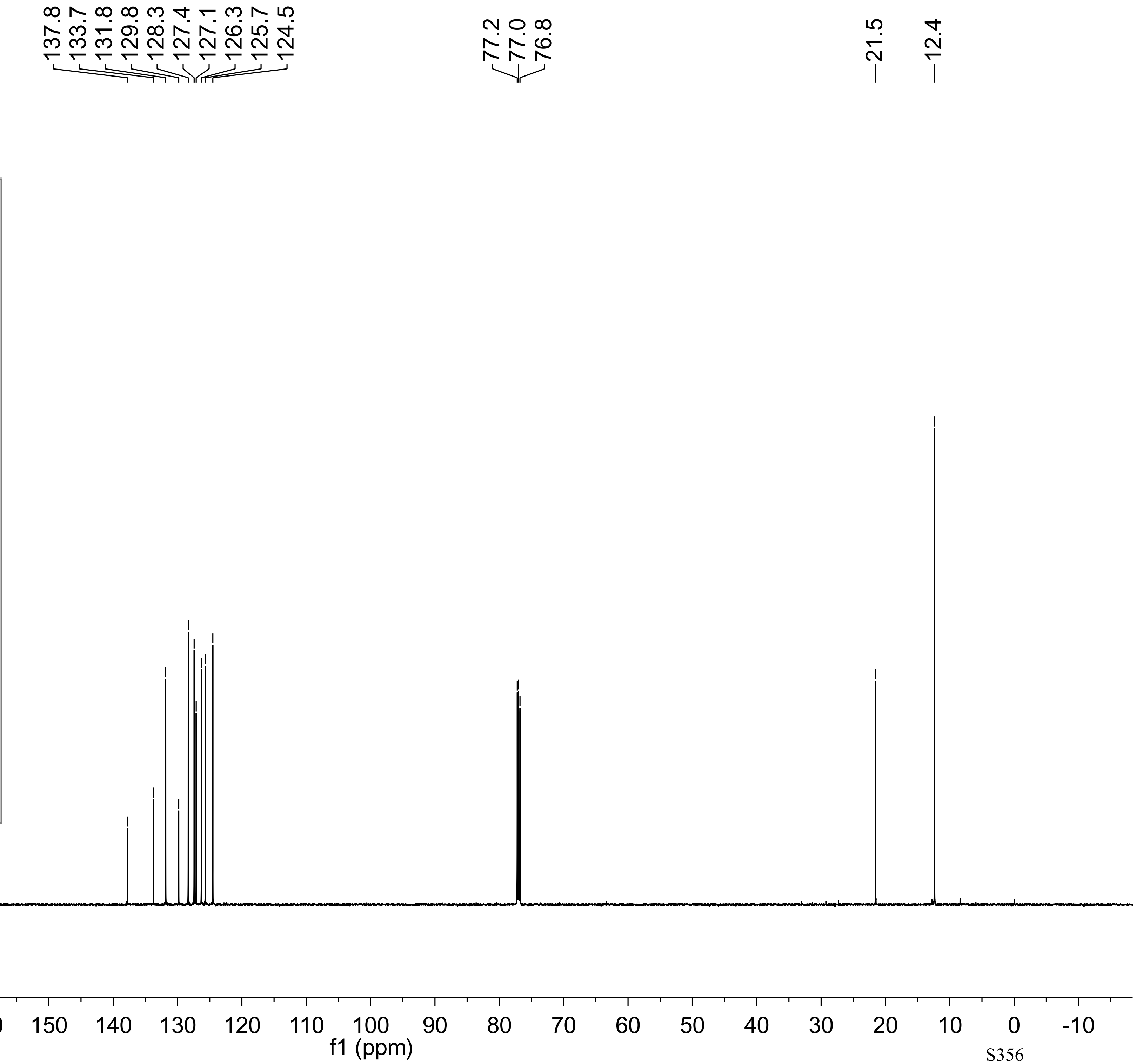

Parameter		Value
1	Title	CFM-G3
2	Comment	
3	Origin	Bruker BioSpin GmbH
4	Owner	nmrsu
5	Site	
6	Spectrometer	Avance NEO 600
7	Author	
8	Solvent	CDCl3
9	Temperature	297.5
10	Pulse Sequence	zgpg30
11	Experiment	1D
12	Number of Scans	256
13	Receiver Gain	101
14	Relaxation Delay	2.0000
15	Pulse Width	12.0000
16	Acquisition Time	0.9175
17	Acquisition Date	2019-12-28T22:38:54
18	Modification Date	2019-12-29T10:16:04
19	Spectrometer Frequency	150.91
20	Spectral Width	35714.3
21	Lowest Frequency	-2779.8
22	Nucleus	13C
23	Acquired Size	32768
24	Spectral Size	32768

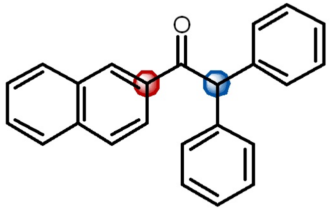

A32

	Parameter	Value
1	Title	CFM-B2
2	Comment	
3	Origin	Bruker BioSpin GmbH
4	Owner	nmrsu
5	Site	
6	Spectrometer	Avance NEO 600
7	Author	
8	Solvent	CDCl3
9	Temperature	296.4
10	Pulse Sequence	zg30
11	Experiment	1D
12	Number of Scans	16
13	Receiver Gain	67
14	Relaxation Delay	1.0000
15	Pulse Width	10.0000
16	Acquisition Time	2.7525
17	Acquisition Date	2019-12-09T08:10:39
18	Modification Date	2019-12-09T09:11:57
19	Spectrometer Frequency	600.15
20	Spectral Width	11904.8
21	Lowest Frequency	-2302.6
22	Nucleus	1H
23	Acquired Size	32768
24	Spectral Size	65536

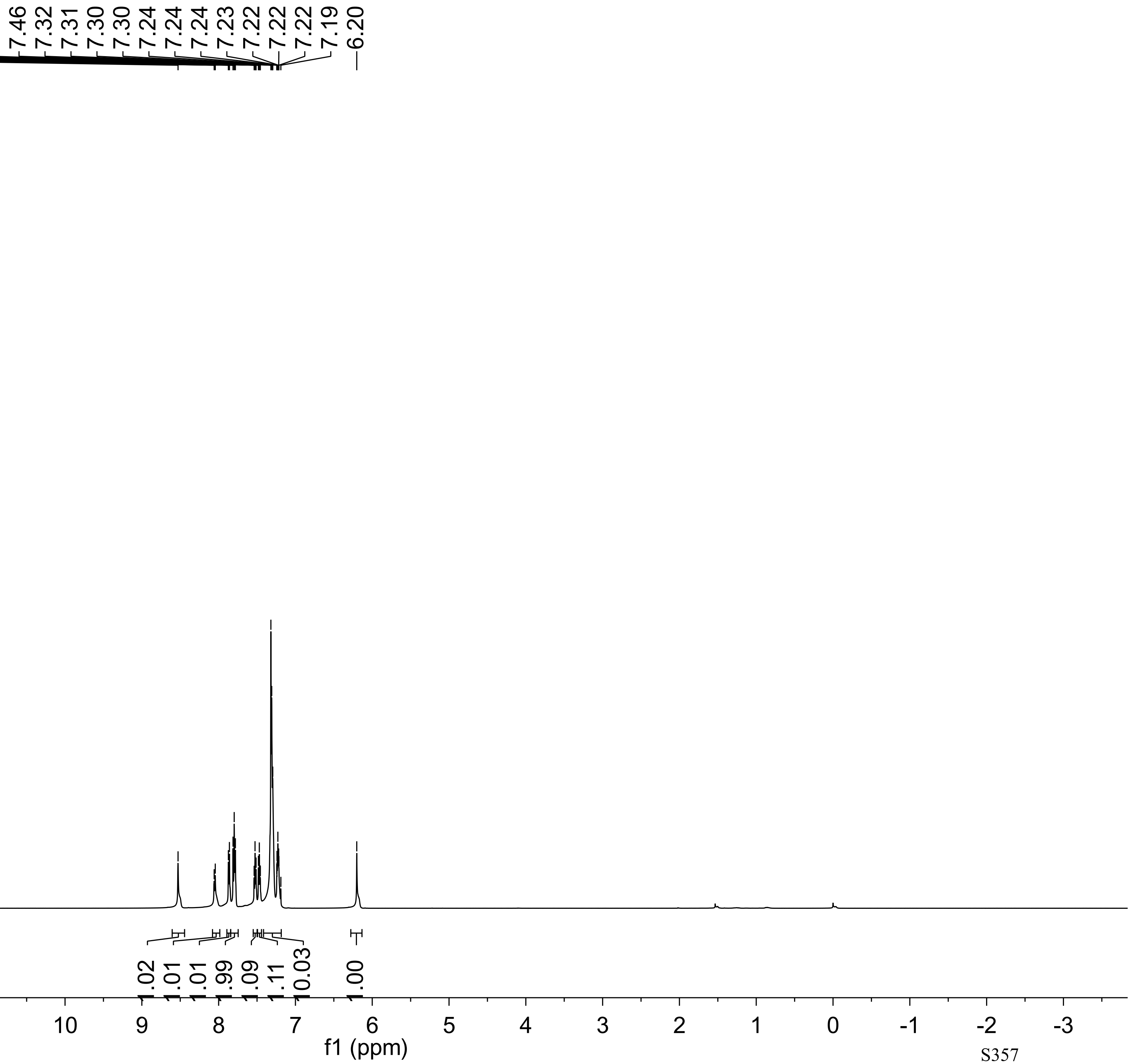

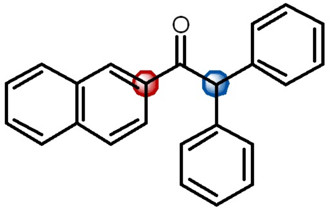

A32

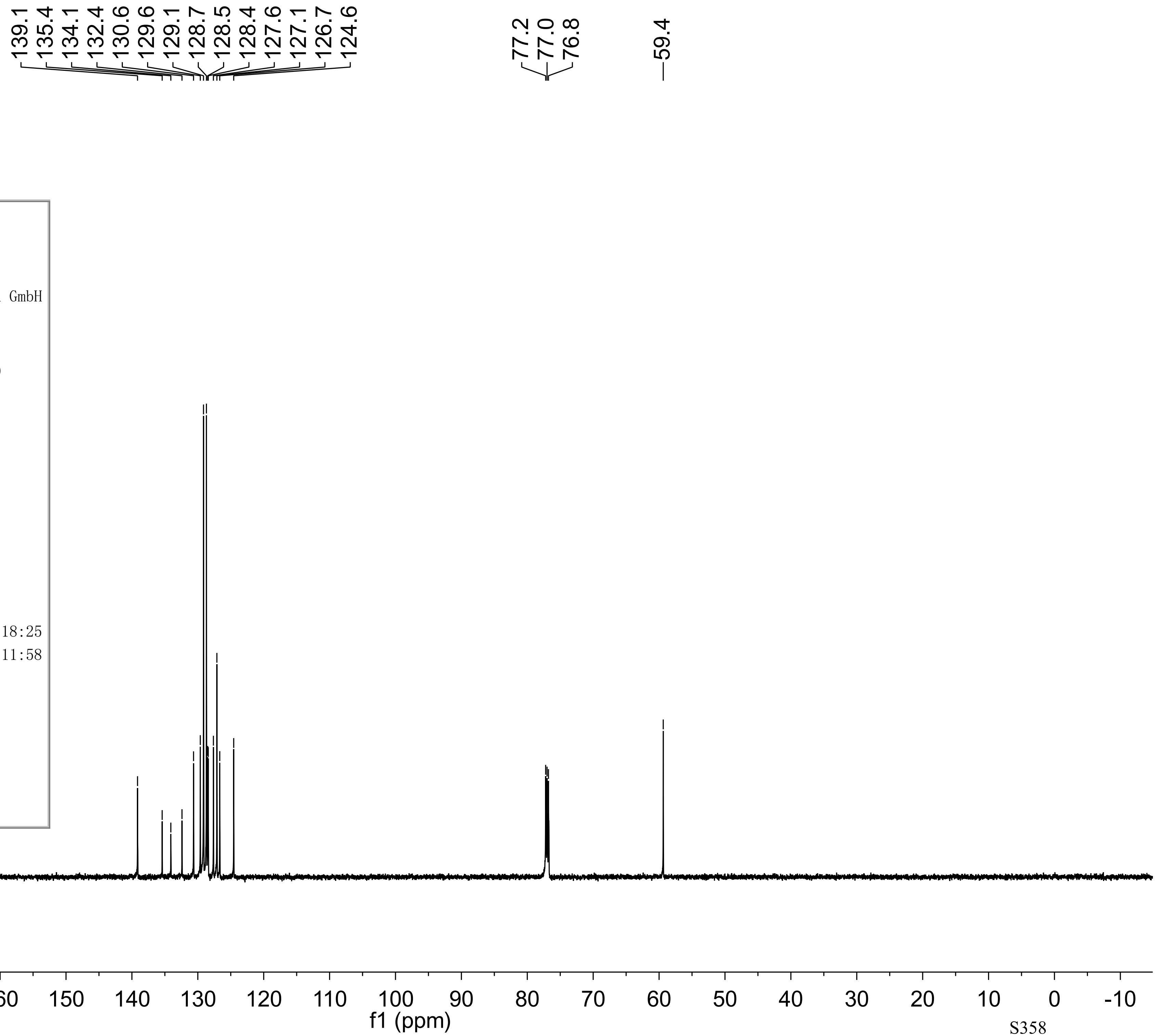

	Parameter	Value
1	Title	CFM-B2
2	Comment	
3	Origin	Bruker BioSpin GmbH
4	Owner	nmrsu
5	Site	
6	Spectrometer	Avance NEO 600
7	Author	
8	Solvent	CDCl3
9	Temperature	297.6
10	Pulse Sequence	zgpg30
11	Experiment	1D
12	Number of Scans	128
13	Receiver Gain	101
14	Relaxation Delay	2.0000
15	Pulse Width	12.0000
16	Acquisition Time	0.9175
17	Acquisition Date	2019-12-09T08:18:25
18	Modification Date	2019-12-09T09:11:58
19	Spectrometer Frequency	150.91
20	Spectral Width	35714.3
21	Lowest Frequency	-2787.8
22	Nucleus	13C
23	Acquired Size	32768
24	Spectral Size	32768

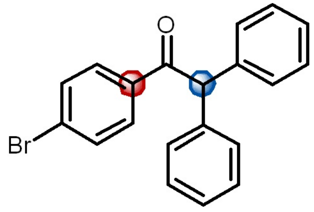

A33

	Parameter	Value
1	Title	CFM-B6-1
2	Comment	
3	Origin	Bruker BioSpin GmbH
4	Owner	nmrsu
5	Site	
6	Spectrometer	Avance NEO 600
7	Author	
8	Solvent	CDCl3
9	Temperature	298.6
10	Pulse Sequence	zg30
11	Experiment	1D
12	Number of Scans	8
13	Receiver Gain	73
14	Relaxation Delay	1.0000
15	Pulse Width	10.0000
16	Acquisition Time	2.7525
17	Acquisition Date	2020-03-19T22:10:23
18	Modification Date	2020-03-19T22:16:56
19	Spectrometer Frequency	600.15
20	Spectral Width	11904.8
21	Lowest Frequency	-2283.2
22	Nucleus	1H
23	Acquired Size	32768
24	Spectral Size	65536

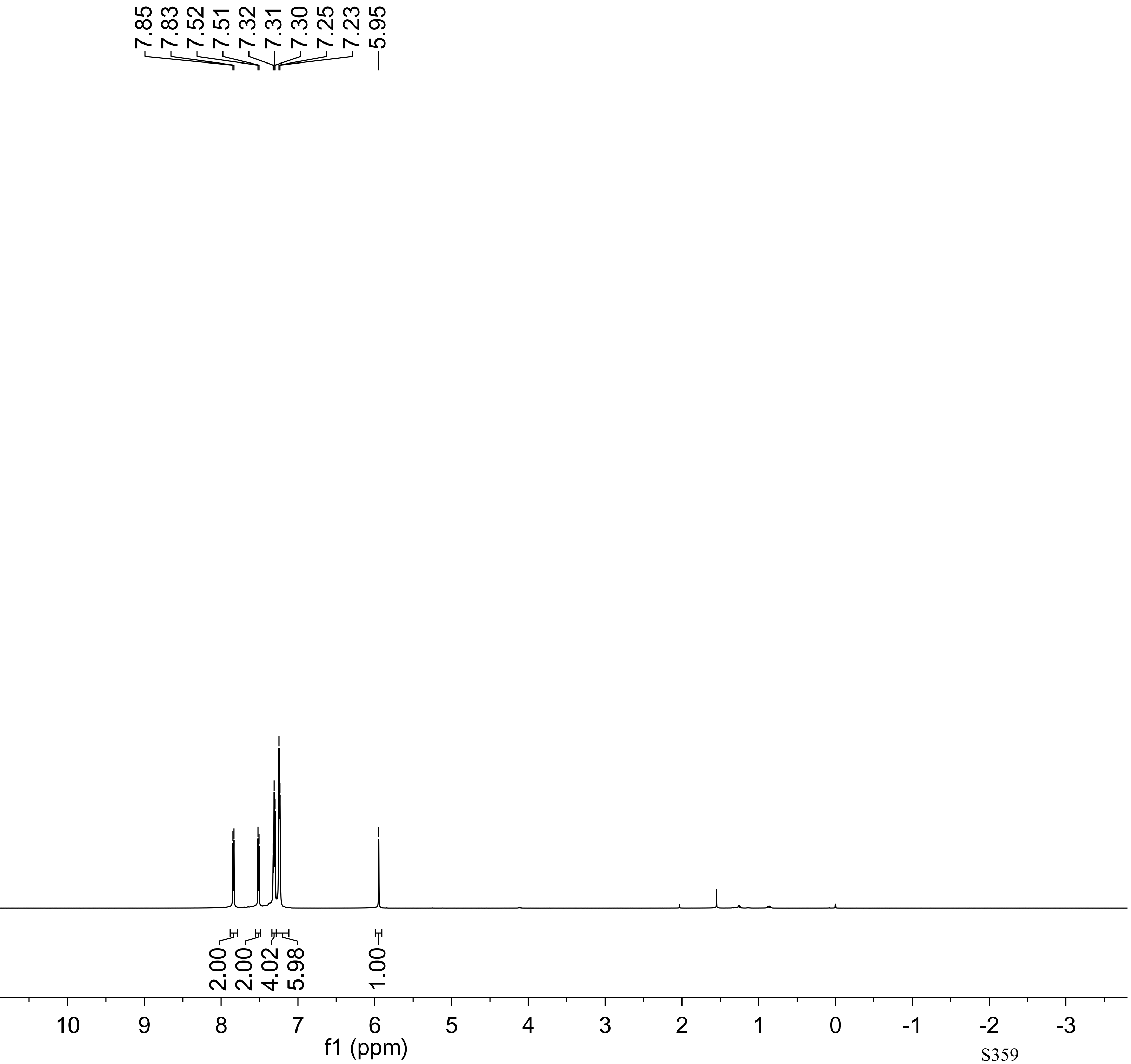

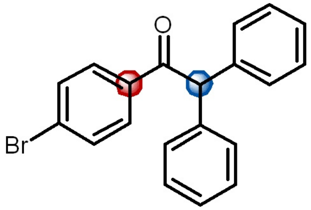

A33

Parameter	Value
1 Title	CFM-B6-1
2 Comment	
3 Origin	Bruker BioSpin GmbH
4 Owner	nmrsu
5 Site	
6 Spectrometer	Avance NEO 600
7 Author	
8 Solvent	CDCl3
9 Temperature	299.5
10 Pulse Sequence	zgpg30
11 Experiment	1D
12 Number of Scans	49
13 Receiver Gain	101
14 Relaxation Delay	2.0000
15 Pulse Width	12.0000
16 Acquisition Time	0.9175
17 Acquisition Date	2020-03-19T22:13:58
18 Modification Date	2020-03-19T22:16:56
19 Spectrometer Frequency	150.91
20 Spectral Width	35714.3
21 Lowest Frequency	-2781.4
22 Nucleus	13C
23 Acquired Size	32768
24 Spectral Size	32768

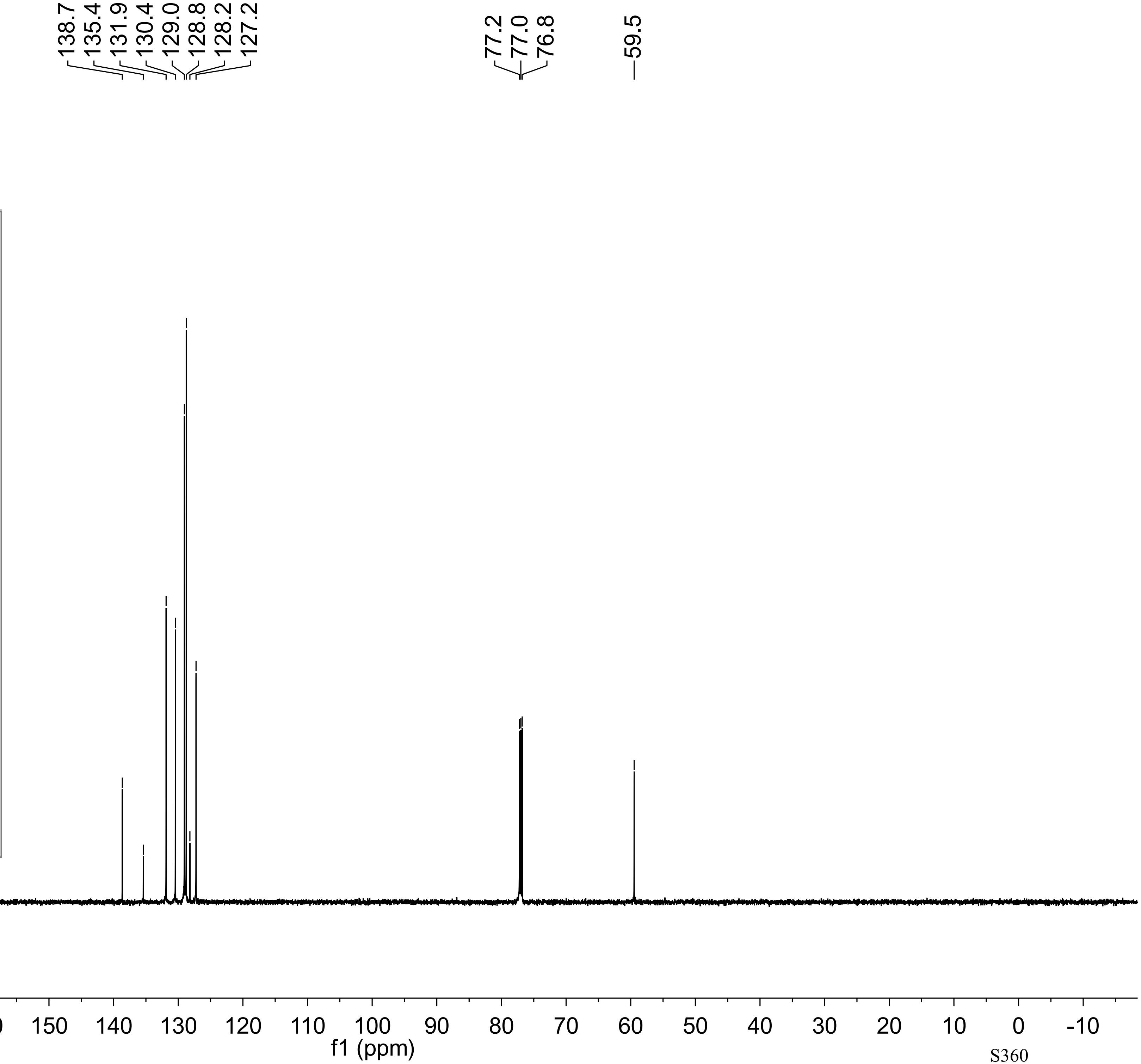

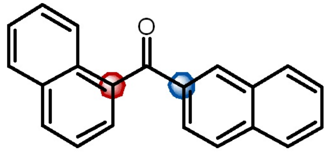

A34

	Parameter	Value
1	Title	CFM-5-196-2
2	Comment	
3	Origin	Bruker BioSpin GmbH
4	Owner	nmrsu
5	Site	
6	Spectrometer	Avance NEO 600
7	Author	
8	Solvent	CDCl3
9	Temperature	298.2
10	Pulse Sequence	zg30
11	Experiment	1D
12	Number of Scans	8
13	Receiver Gain	101
14	Spectrometer Frequency	600.15
15	Spectral Width	11904.8
16	Lowest Frequency	-2261.9
17	Nucleus	1H
18	Acquired Size	32768
19	Spectral Size	65536

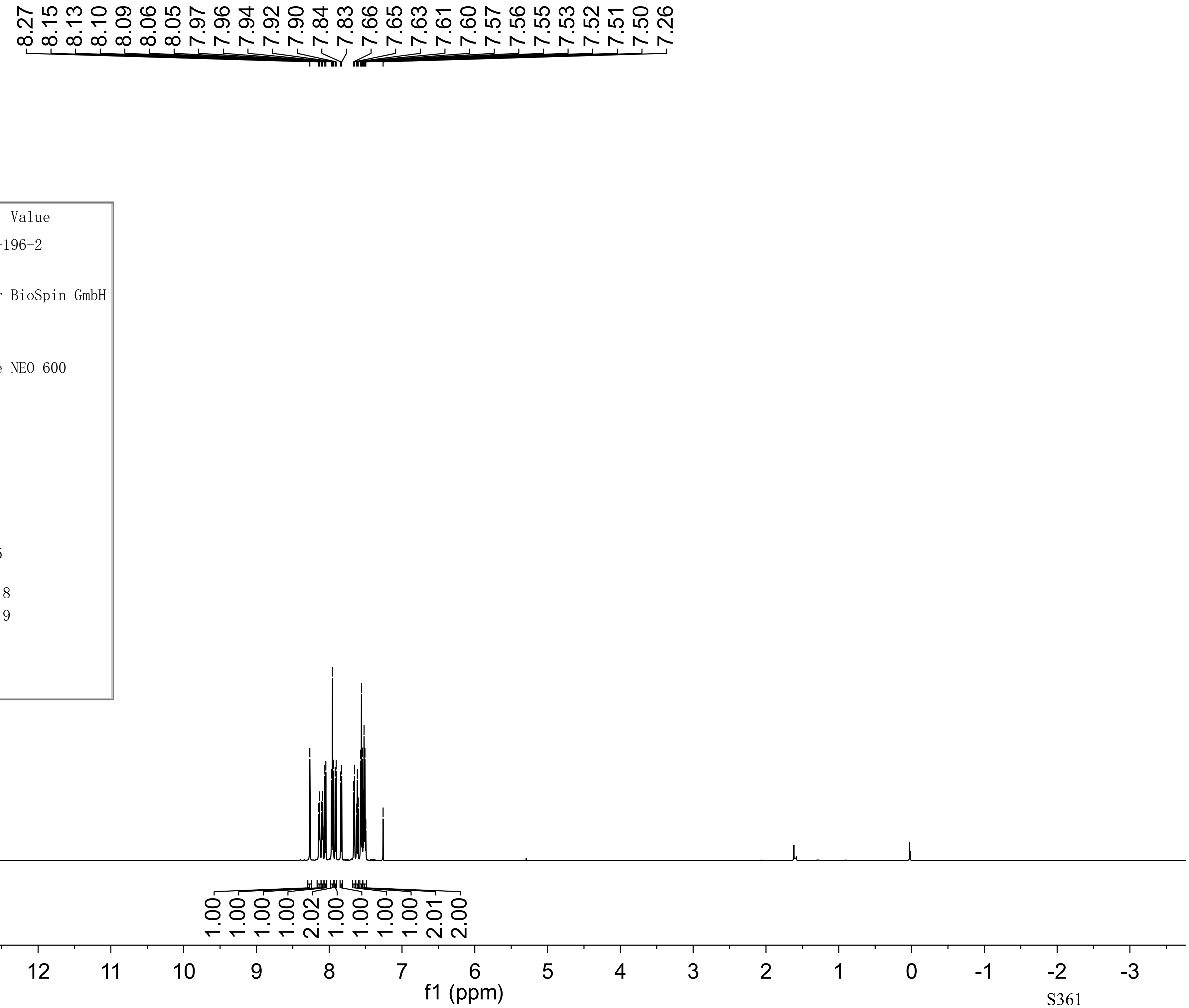

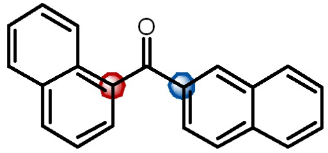

A34

Parameter	Value
1 Title	CFM-5-196-2
2 Comment	
3 Origin	Bruker BioSpin GmbH
4 Owner	nmrsu
5 Site	
6 Spectrometer	Avance NEO 600
7 Author	
8 Solvent	CDCl3
9 Temperature	298.2
10 Pulse Sequence	zgpg30
11 Experiment	1D
12 Number of Scans	256
13 Receiver Gain	101
14 Spectrometer Frequency	150.91
15 Spectral Width	35714.3
16 Lowest Frequency	-2778.3
17 Nucleus	13C
18 Acquired Size	32768
19 Spectral Size	32768

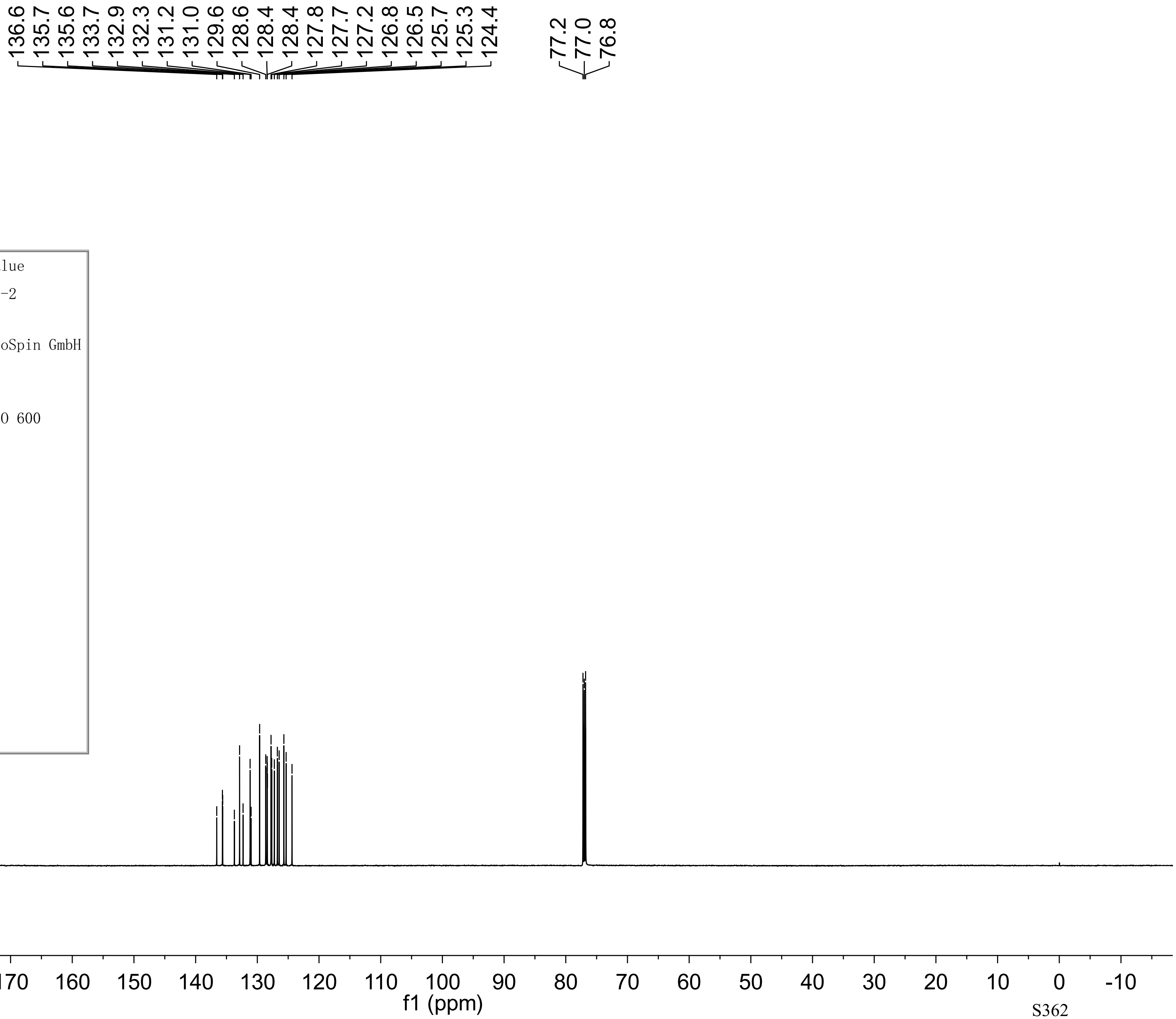

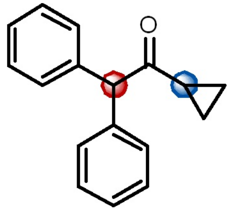

A37

	Parameter	Value
1	Title	ZYLF0118
2	Comment	
3	Origin	Bruker BioSpin GmbH
4	Owner	nmrsu
5	Site	
6	Spectrometer	Avance NEO 600
7	Author	
8	Solvent	CDCl3
9	Temperature	296.4
10	Pulse Sequence	zg30
11	Experiment	1D
12	Number of Scans	8
13	Receiver Gain	101
14	Relaxation Delay	1.0000
15	Pulse Width	10.0000
16	Acquisition Time	2.7525
17	Acquisition Date	2020-01-17T18:32:23
18	Modification Date	2020-03-18T21:21:58
19	Spectrometer Frequency	600.15
20	Spectral Width	11904.8
21	Lowest Frequency	-2265.1
22	Nucleus	1H
23	Acquired Size	32768
24	Spectral Size	65536

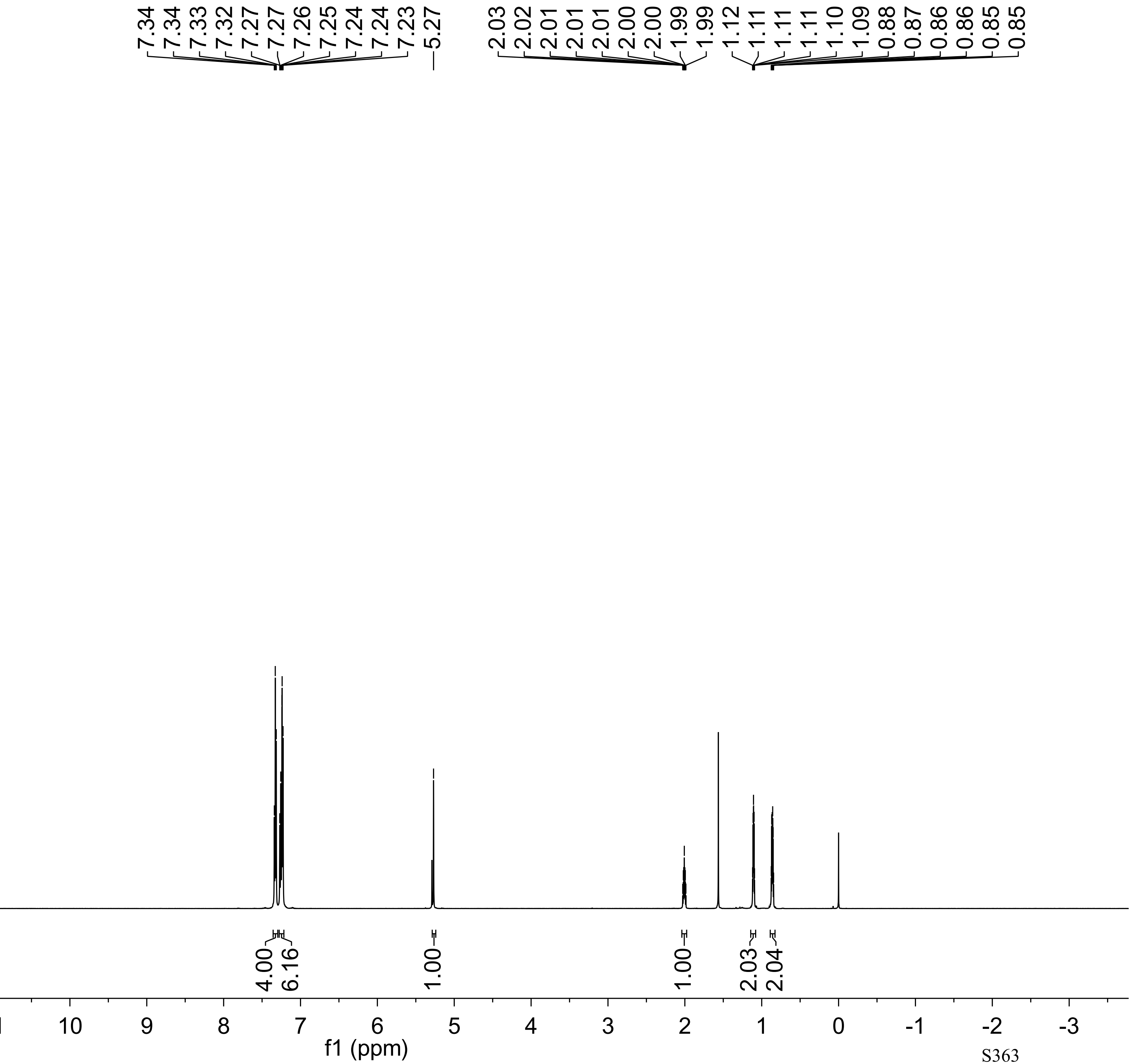

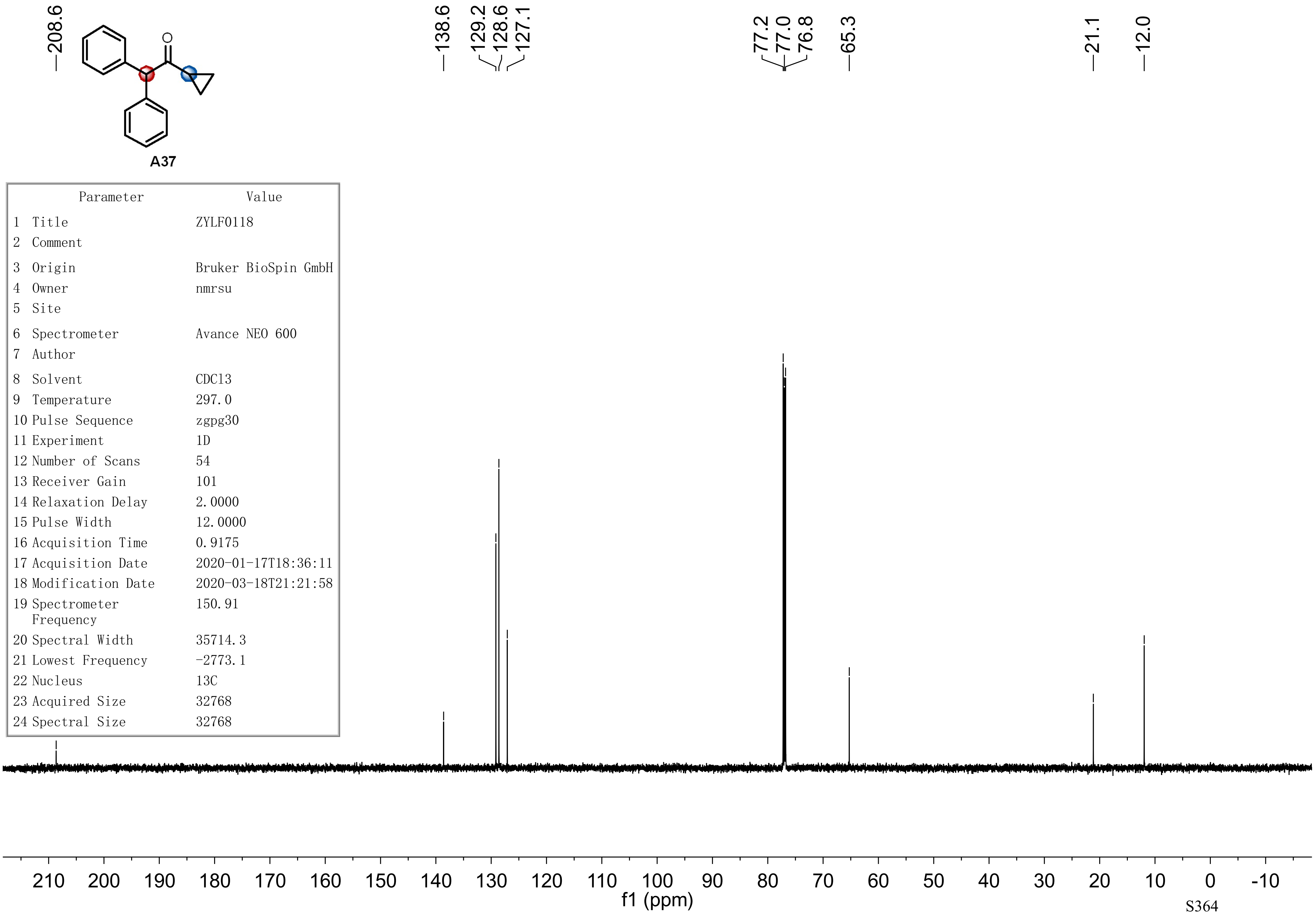

A37

	Parameter	Value
1	Title	ZYLF0118
2	Comment	
3	Origin	Bruker BioSpin GmbH
4	Owner	nmrsu
5	Site	
6	Spectrometer	Avance NEO 600
7	Author	
8	Solvent	CDCl3
9	Temperature	297.0
10	Pulse Sequence	zgpg30
11	Experiment	1D
12	Number of Scans	54
13	Receiver Gain	101
14	Relaxation Delay	2.0000
15	Pulse Width	12.0000
16	Acquisition Time	0.9175
17	Acquisition Date	2020-01-17T18:36:11
18	Modification Date	2020-03-18T21:21:58
19	Spectrometer Frequency	150.91
20	Spectral Width	35714.3
21	Lowest Frequency	-2773.1
22	Nucleus	13C
23	Acquired Size	32768
24	Spectral Size	32768

CFM-7-71

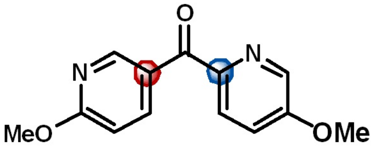

A42

Parameter	Value
1 Title	CFM-7-71
2 Comment	
3 Origin	Bruker BioSpin GmbH
4 Owner	nmrsu
5 Site	
6 Spectrometer	AVANCE NEO 400 MHZ DIGITAL NMR SPECTROMETER
7 Author	
8 Solvent	CDCl3
9 Temperature	298.2
10 Pulse Sequence	zg30
11 Experiment	1D
12 Number of Scans	8
13 Receiver Gain	101
14 Spectrometer Frequency	400.13
15 Spectral Width	8196.7
16 Lowest Frequency	-1637.2
17 Nucleus	1H
18 Acquired Size	32768
19 Spectral Size	65536

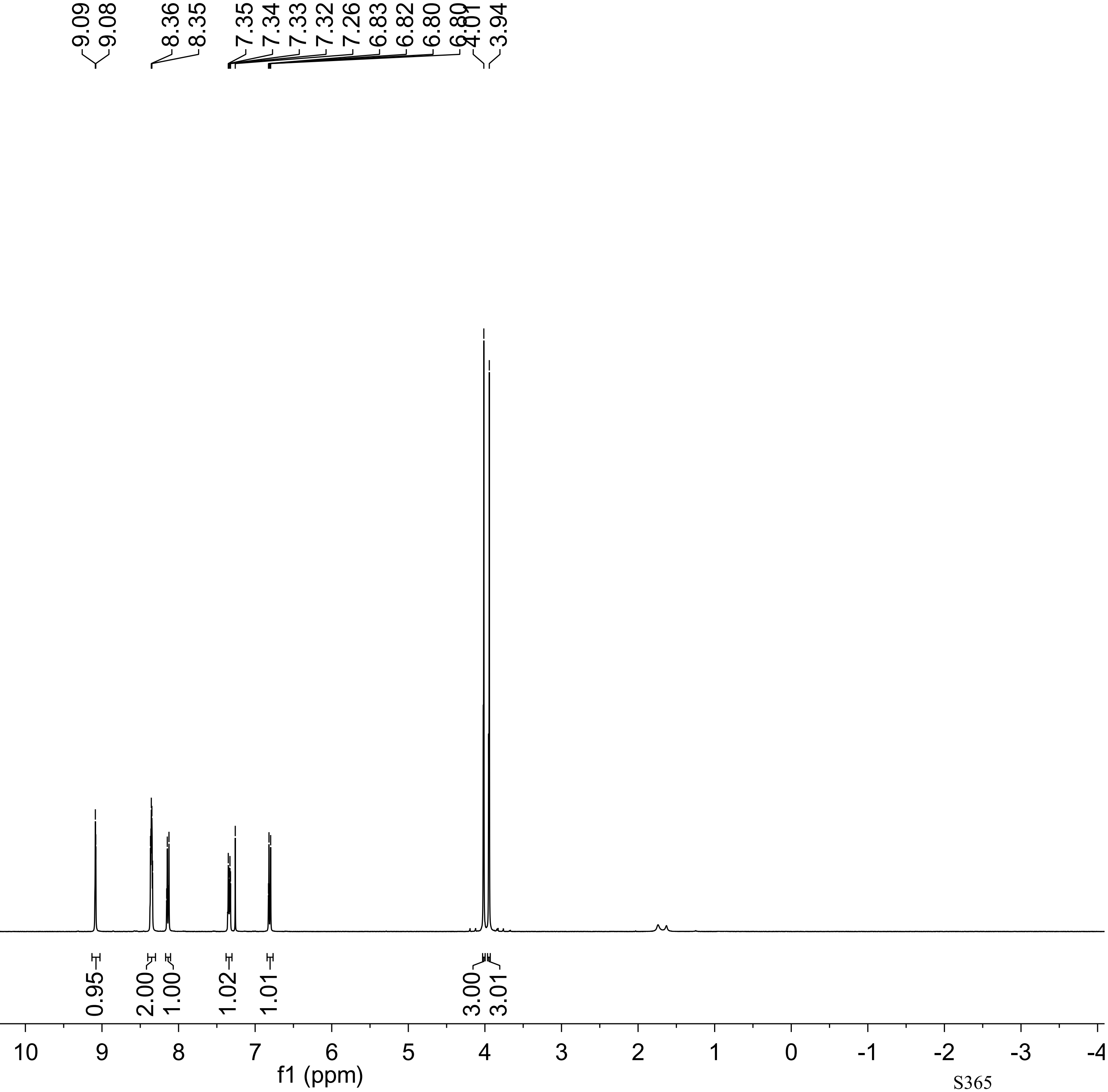

CFM-7-71-C

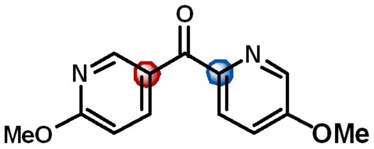

A42

Parameter	Value
1 Title	CFM-7-71-C
2 Comment	
3 Origin	Bruker BioSpin GmbH
4 Owner	nmrsu
5 Site	
6 Spectrometer	Avance NEO 600
7 Author	
8 Solvent	CDCl3
9 Temperature	298.1
10 Pulse Sequence	zgpg30
11 Experiment	1D
12 Number of Scans	128
13 Receiver Gain	101
14 Spectrometer Frequency	150.91
15 Spectral Width	35714.3
16 Lowest Frequency	-2771.3
17 Nucleus	13C
18 Acquired Size	32768
19 Spectral Size	32768

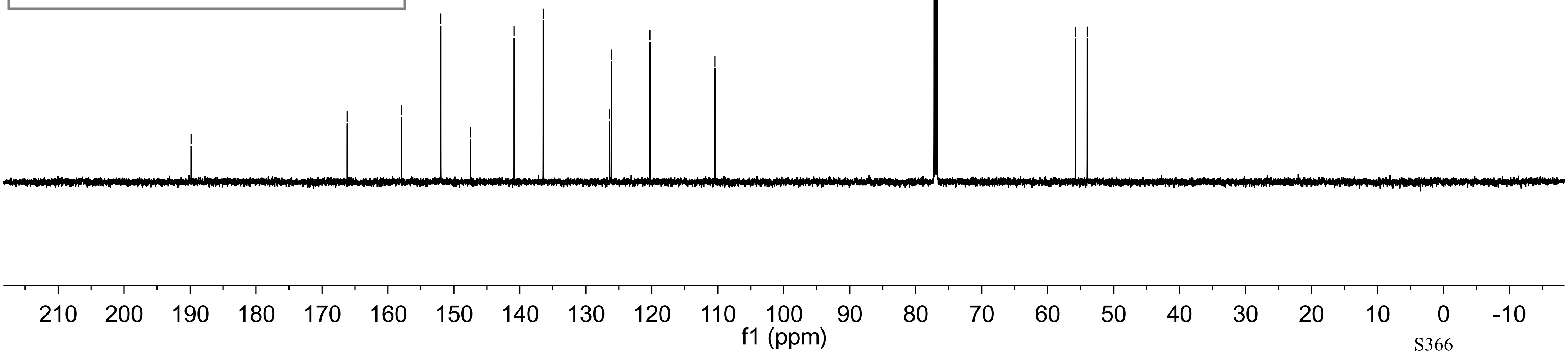

A173-2

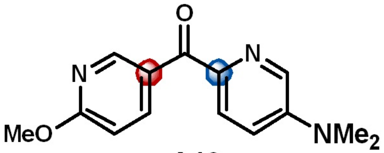

A43

Parameter	Value
1 Title	A173-2
2 Comment	
3 Origin	Bruker BioSpin GmbH
4 Owner	nmrsu
5 Site	
6 Spectrometer	Avance NEO 600
7 Author	
8 Solvent	CDCl3
9 Temperature	298.2
10 Pulse Sequence	zg30
11 Experiment	1D
12 Number of Scans	8
13 Receiver Gain	101
14 Spectrometer Frequency	600.15
15 Spectral Width	11904.8
16 Lowest Frequency	-2260.9
17 Nucleus	1H
18 Acquired Size	32768
19 Spectral Size	65536

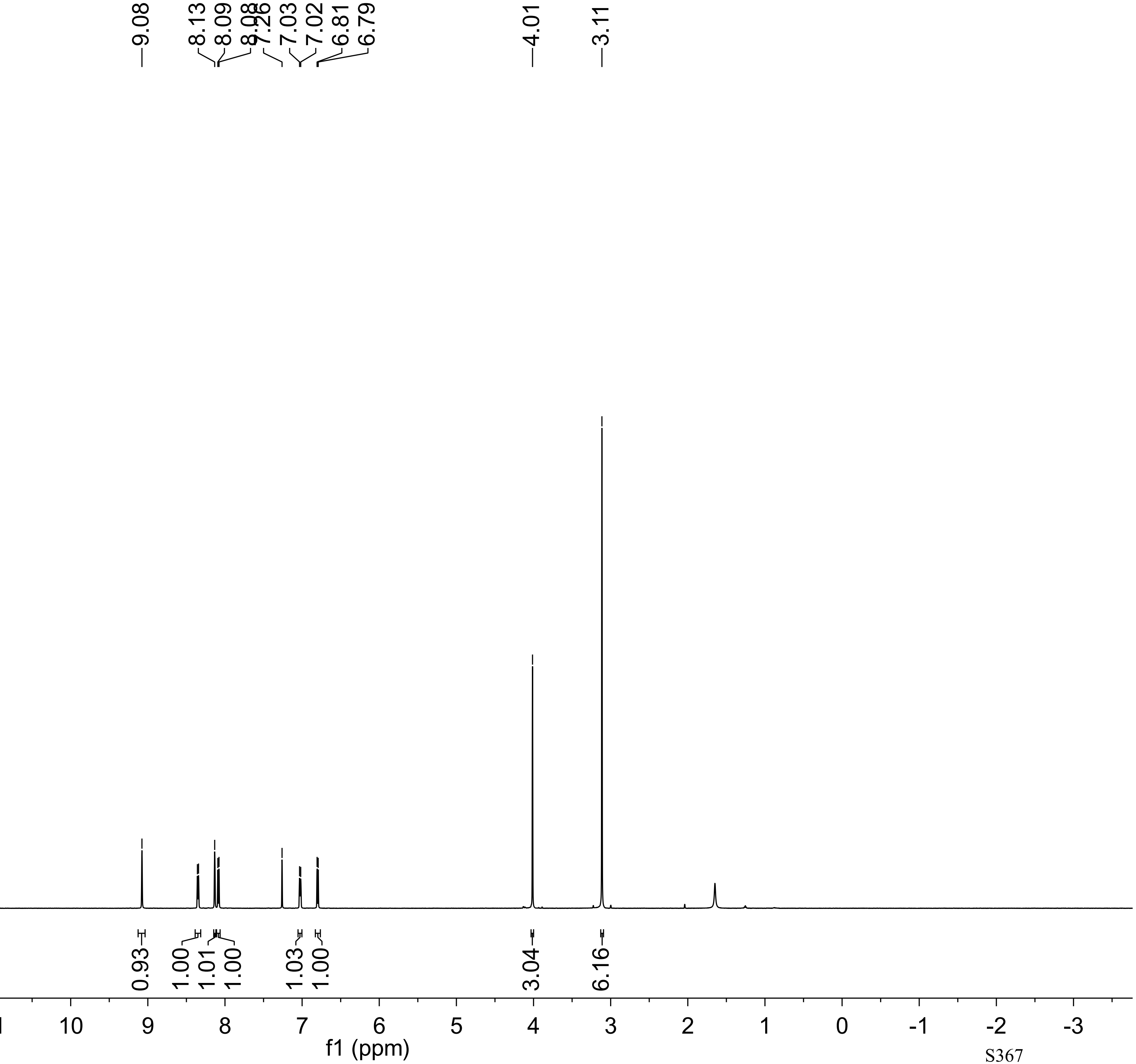

A173-2-C

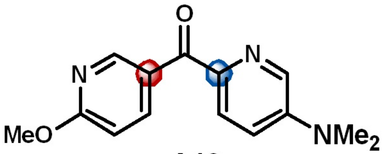

A43

Parameter	Value
1 Title	A173-2-C
2 Comment	
3 Origin	Bruker BioSpin GmbH
4 Owner	nmrsu
5 Site	
6 Spectrometer	AVANCE NEO 400 MHZ DIGITAL NMR SPECTROMETER
7 Author	
8 Solvent	CDCl3
9 Temperature	298.2
10 Pulse Sequence	zgpg30
11 Experiment	1D
12 Number of Scans	256
13 Receiver Gain	57
14 Spectrometer Frequency	100.61
15 Spectral Width	23809.5
16 Lowest Frequency	-1848.5
17 Nucleus	13C
18 Acquired Size	32768
19 Spectral Size	32768

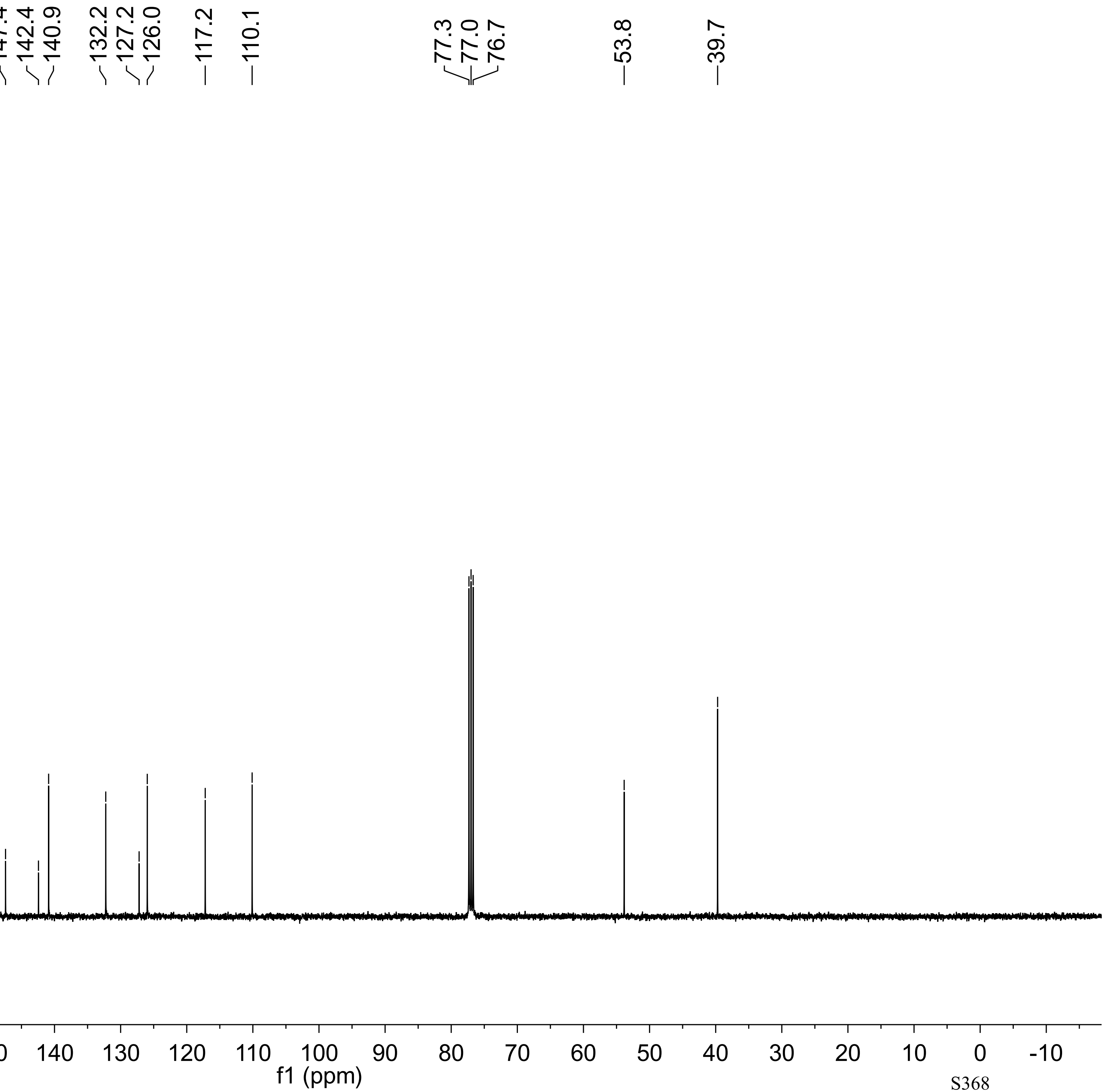

plx-A172

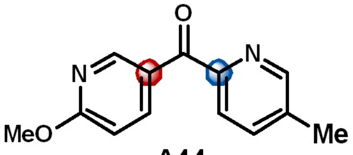

A44

	Parameter	Value
1	Title	plx-A172
2	Comment	
3	Origin	Bruker BioSpin GmbH
4	Owner	nmrsu
5	Site	
6	Spectrometer	Avance Neo 400M
7	Author	
8	Solvent	CDCl3
9	Temperature	298.0
10	Pulse Sequence	zg30
11	Experiment	1D
12	Number of Scans	13
13	Receiver Gain	101
14	Spectrometer Frequency	400.18
15	Spectral Width	8196.7
16	Lowest Frequency	-1636.9
17	Nucleus	1H
18	Acquired Size	32768
19	Spectral Size	65536

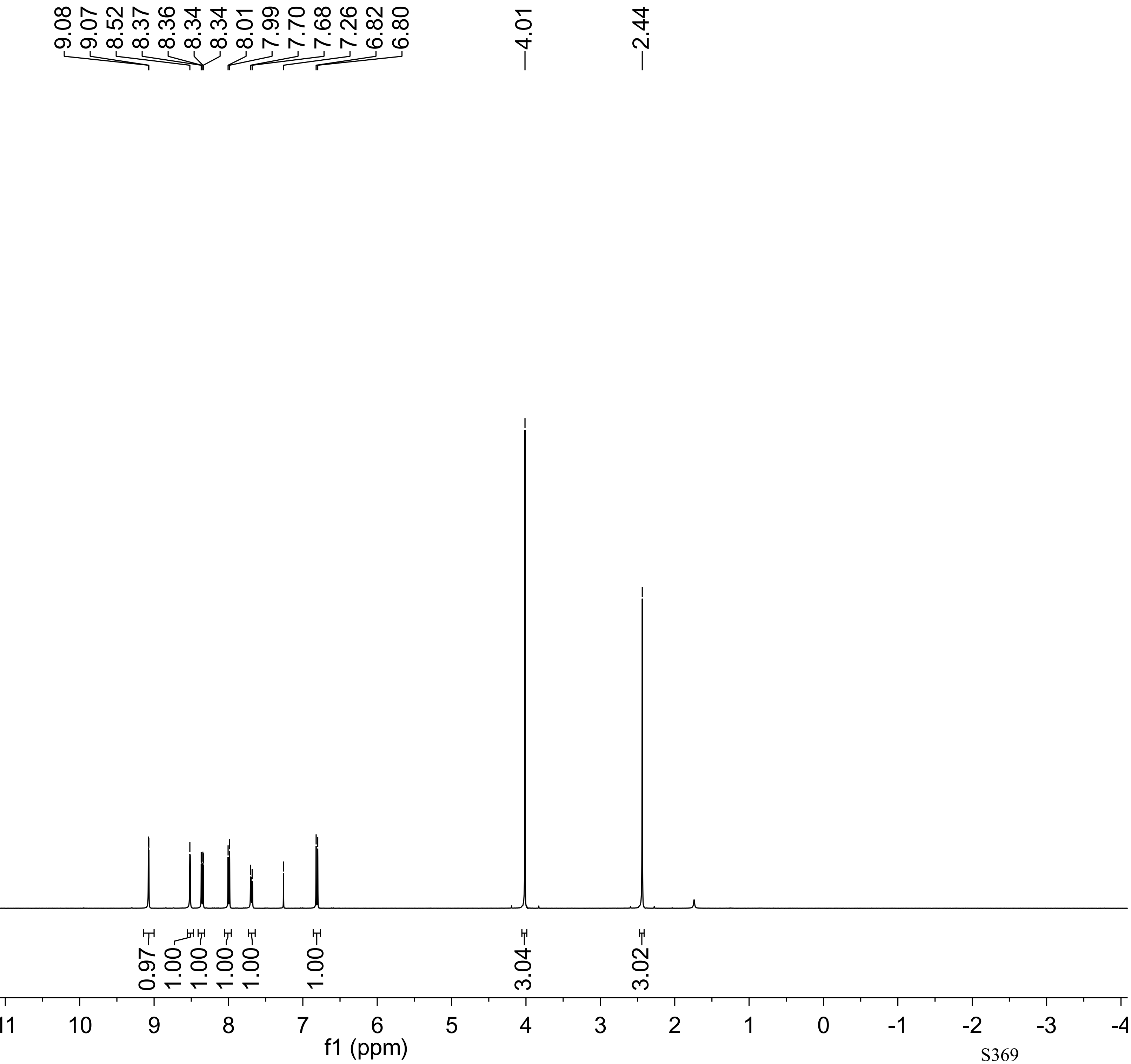

plx-A172

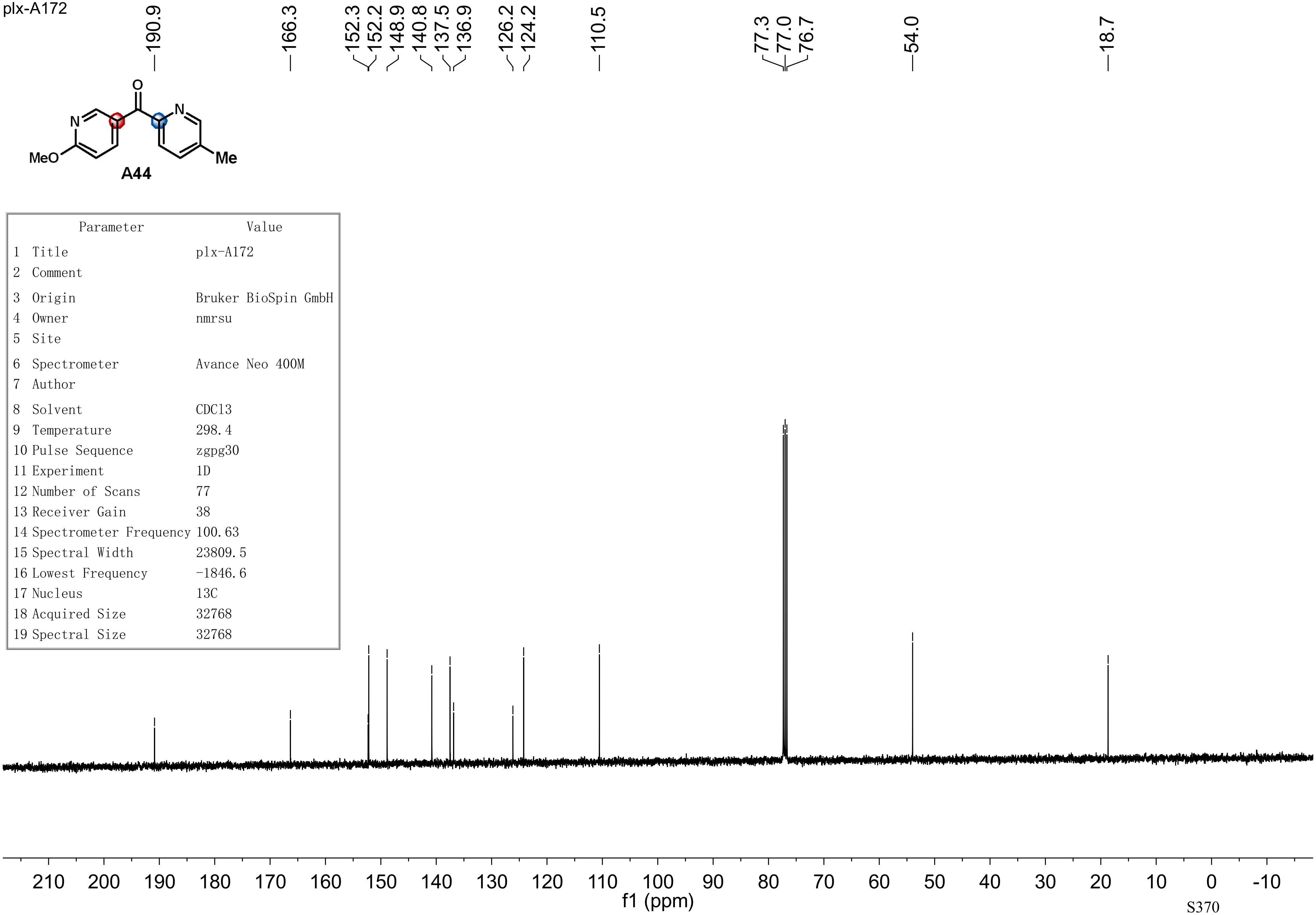

	Parameter	Value
1	Title	plx-A172
2	Comment	
3	Origin	Bruker BioSpin GmbH
4	Owner	nmrsu
5	Site	
6	Spectrometer	Avance Neo 400M
7	Author	
8	Solvent	CDCl3
9	Temperature	298.4
10	Pulse Sequence	zgpg30
11	Experiment	1D
12	Number of Scans	77
13	Receiver Gain	38
14	Spectrometer Frequency	100.63
15	Spectral Width	23809.5
16	Lowest Frequency	-1846.6
17	Nucleus	13C
18	Acquired Size	32768
19	Spectral Size	32768

plx-A174-2

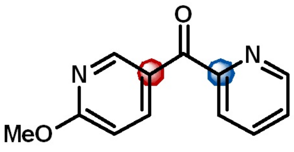
A45

Parameter	Value
1 Title	plx-A174-2
2 Comment	
3 Origin	Bruker BioSpin GmbH
4 Owner	nmrsu
5 Site	
6 Spectrometer	AVANCE NEO 400 MHZ DIGITAL NMR SPECTROMETER
7 Author	
8 Solvent	CDCl3
9 Temperature	298.1
10 Pulse Sequence	zg30
11 Experiment	1D
12 Number of Scans	3
13 Receiver Gain	101
14 Spectrometer Frequency	400.13
15 Spectral Width	8196.7
16 Lowest Frequency	-1637.2
17 Nucleus	1H
18 Acquired Size	32768
19 Spectral Size	65536

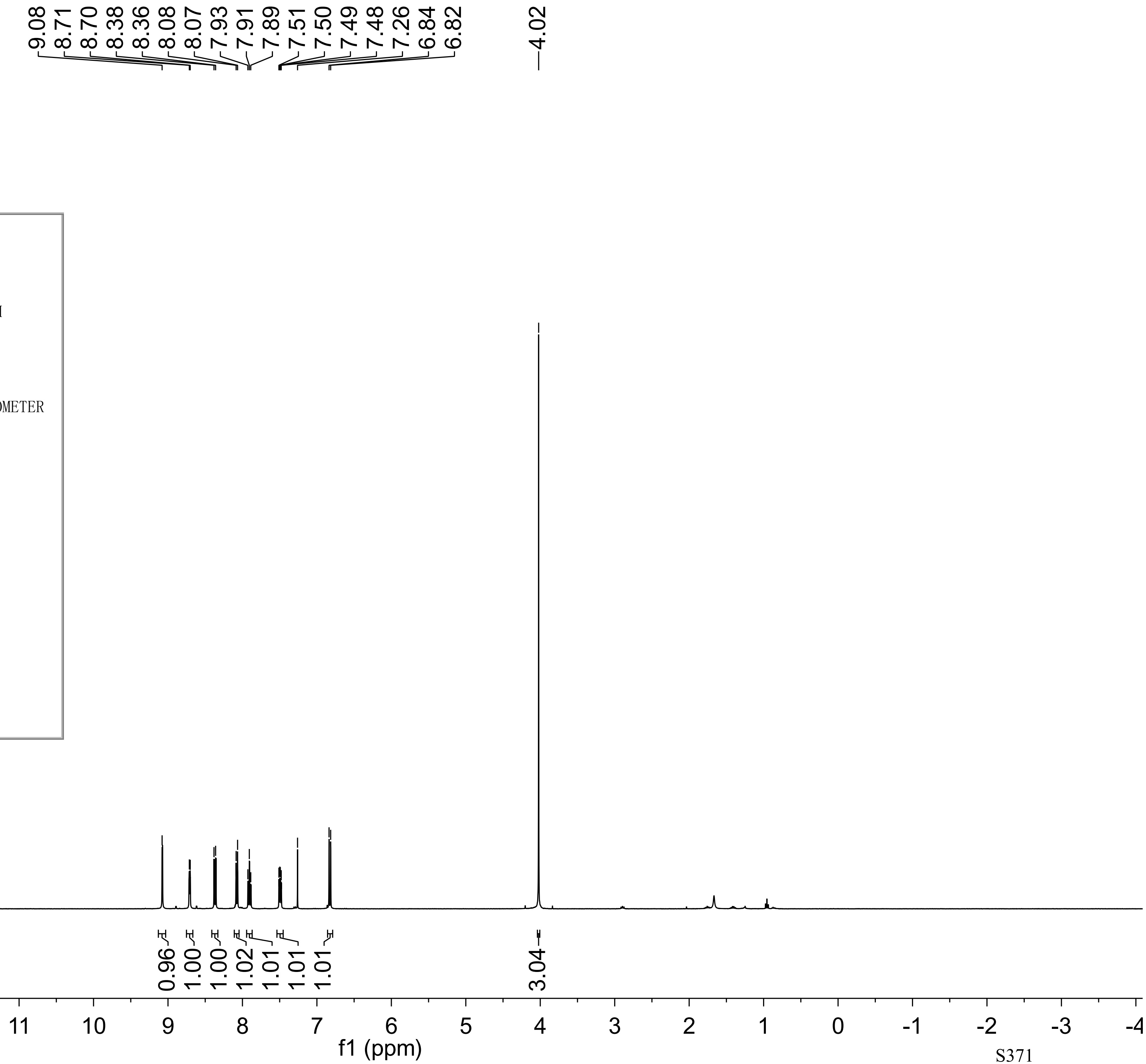

A174-2

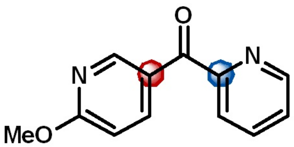

A45

Parameter		Value
1	Title	A174-2
2	Comment	
3	Origin	Bruker BioSpin GmbH
4	Owner	nmrsu
5	Site	
6	Spectrometer	Avance Neo 400M
7	Author	
8	Solvent	CDCl3
9	Temperature	295.1
10	Pulse Sequence	zgpg30
11	Experiment	1D
12	Number of Scans	124
13	Receiver Gain	65
14	Spectrometer Frequency	100.63
15	Spectral Width	23809.5
16	Lowest Frequency	-1846.5
17	Nucleus	13C
18	Acquired Size	32768
19	Spectral Size	32768

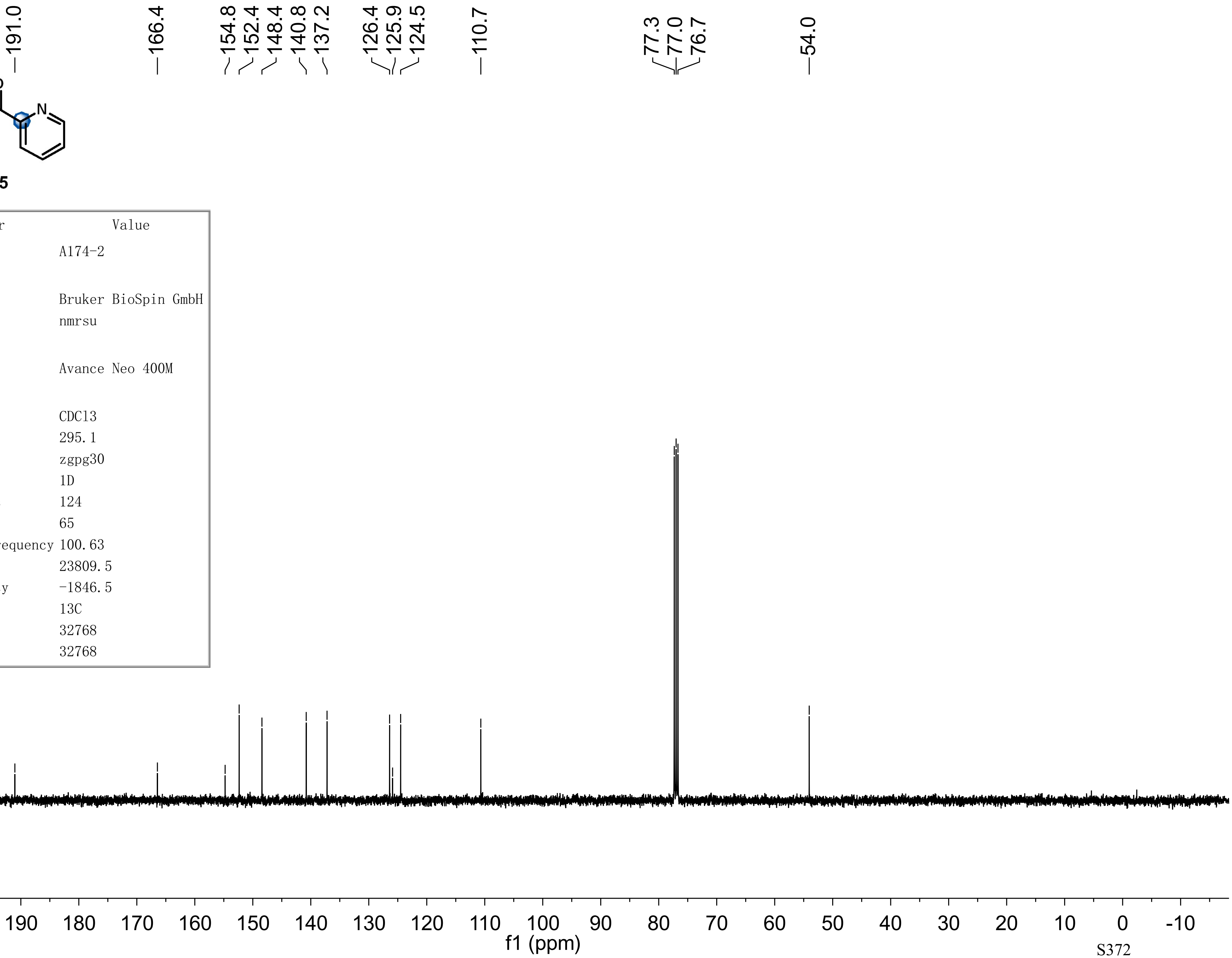

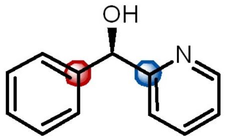

C1

Parameter	Value
1 Title	R-CFM-G13-2
2 Comment	
3 Origin	Bruker BioSpin GmbH
4 Owner	nmrsu
5 Site	
6 Spectrometer	Avance NEO 600
7 Author	
8 Solvent	CDCl3
9 Temperature	296.6
10 Pulse Sequence	zg30
11 Experiment	1D
12 Number of Scans	8
13 Receiver Gain	101
14 Relaxation Delay	1.0000
15 Pulse Width	10.0000
16 Acquisition Time	2.7525
17 Acquisition Date	2020-04-17T18:13:18
18 Modification Date	2020-04-17T19:39:12
19 Spectrometer Frequency	600.15
20 Spectral Width	11904.8
21 Lowest Frequency	-2279.1
22 Nucleus	1H
23 Acquired Size	32768
24 Spectral Size	65536

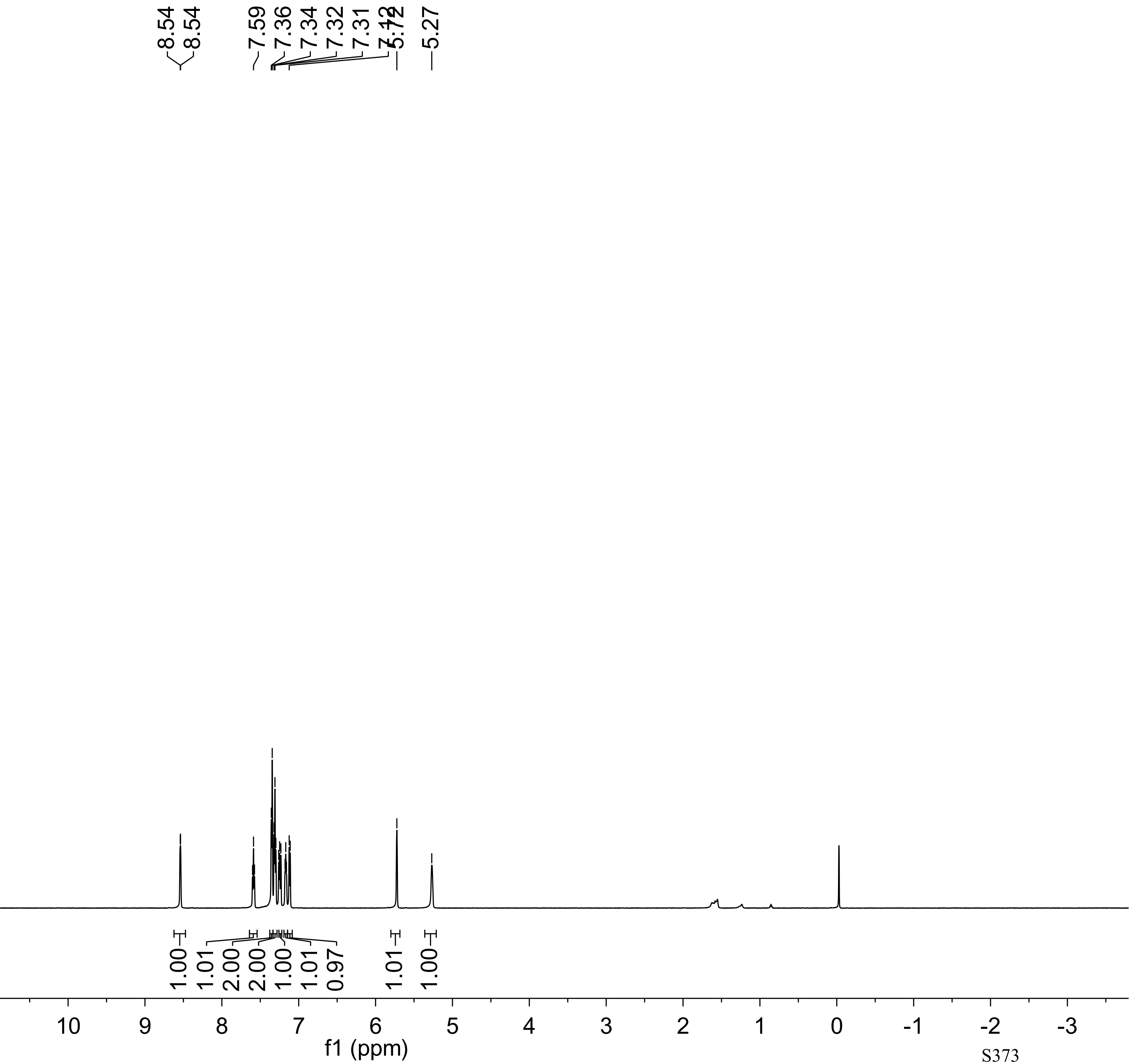

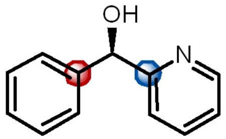

C1

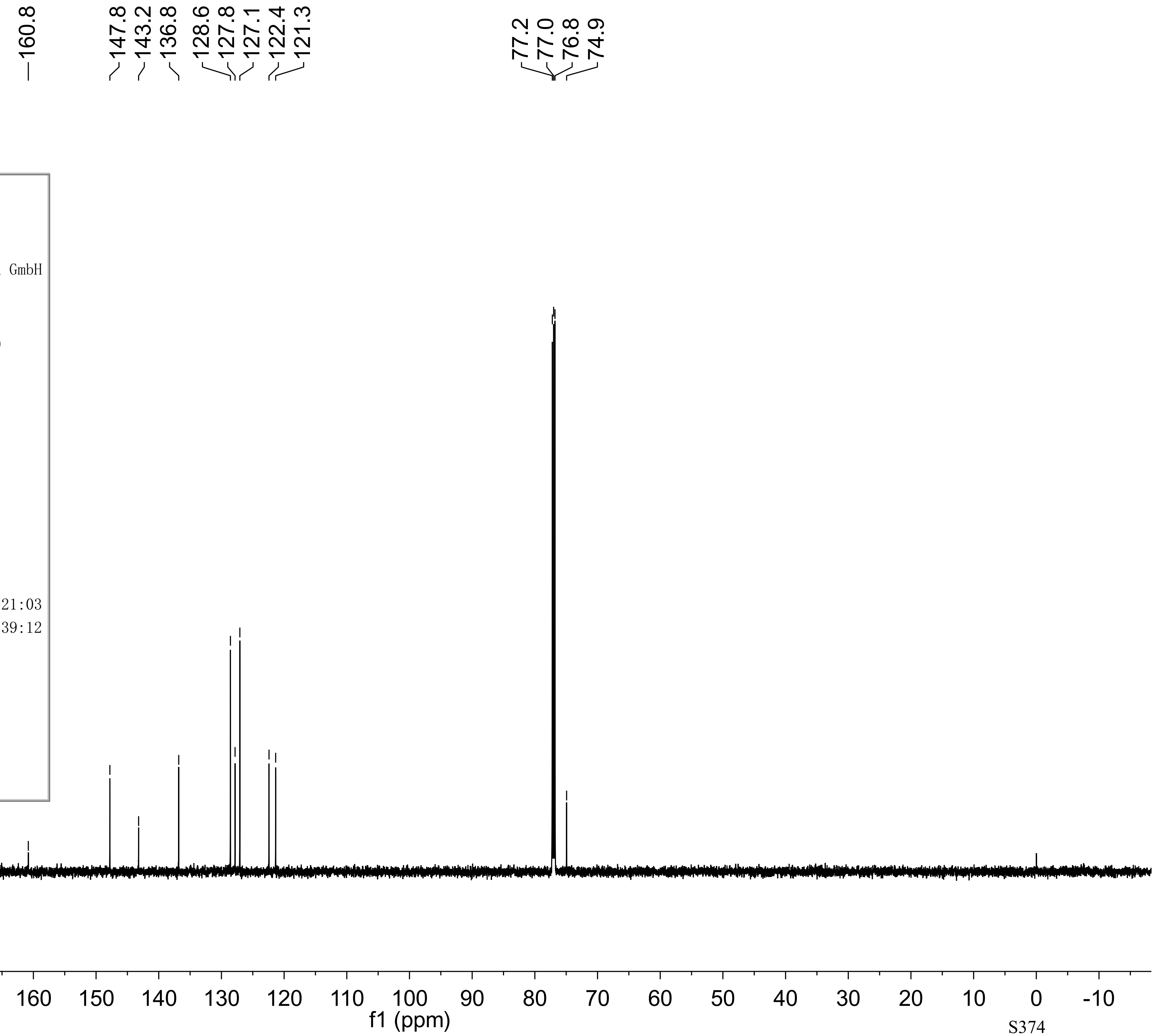

Parameter	Value
1 Title	R-CFM-G13-2
2 Comment	
3 Origin	Bruker BioSpin GmbH
4 Owner	nmrsu
5 Site	
6 Spectrometer	Avance NEO 600
7 Author	
8 Solvent	CDCl3
9 Temperature	297.9
10 Pulse Sequence	zgpg30
11 Experiment	1D
12 Number of Scans	128
13 Receiver Gain	101
14 Relaxation Delay	2.0000
15 Pulse Width	12.0000
16 Acquisition Time	0.9175
17 Acquisition Date	2020-04-17T18:21:03
18 Modification Date	2020-04-17T19:39:12
19 Spectrometer Frequency	150.91
20 Spectral Width	35714.3
21 Lowest Frequency	-2771.6
22 Nucleus	13C
23 Acquired Size	32768
24 Spectral Size	32768

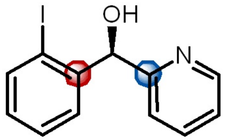

C2

Parameter	Value
1 Title	CFM-C2-0624
2 Comment	
3 Origin	Bruker BioSpin GmbH
4 Owner	nmrsu
5 Site	
6 Spectrometer	AVANCE NEO 400 MHZ DIGITAL NMR SPECTROMETER
7 Author	
8 Solvent	CDCl3
9 Temperature	298.1
10 Pulse Sequence	zg30
11 Experiment	1D
12 Number of Scans	8
13 Receiver Gain	101
14 Spectrometer Frequency	400.13
15 Spectral Width	8196.7
16 Lowest Frequency	-1636.8
17 Nucleus	1H
18 Acquired Size	32768
19 Spectral Size	65536

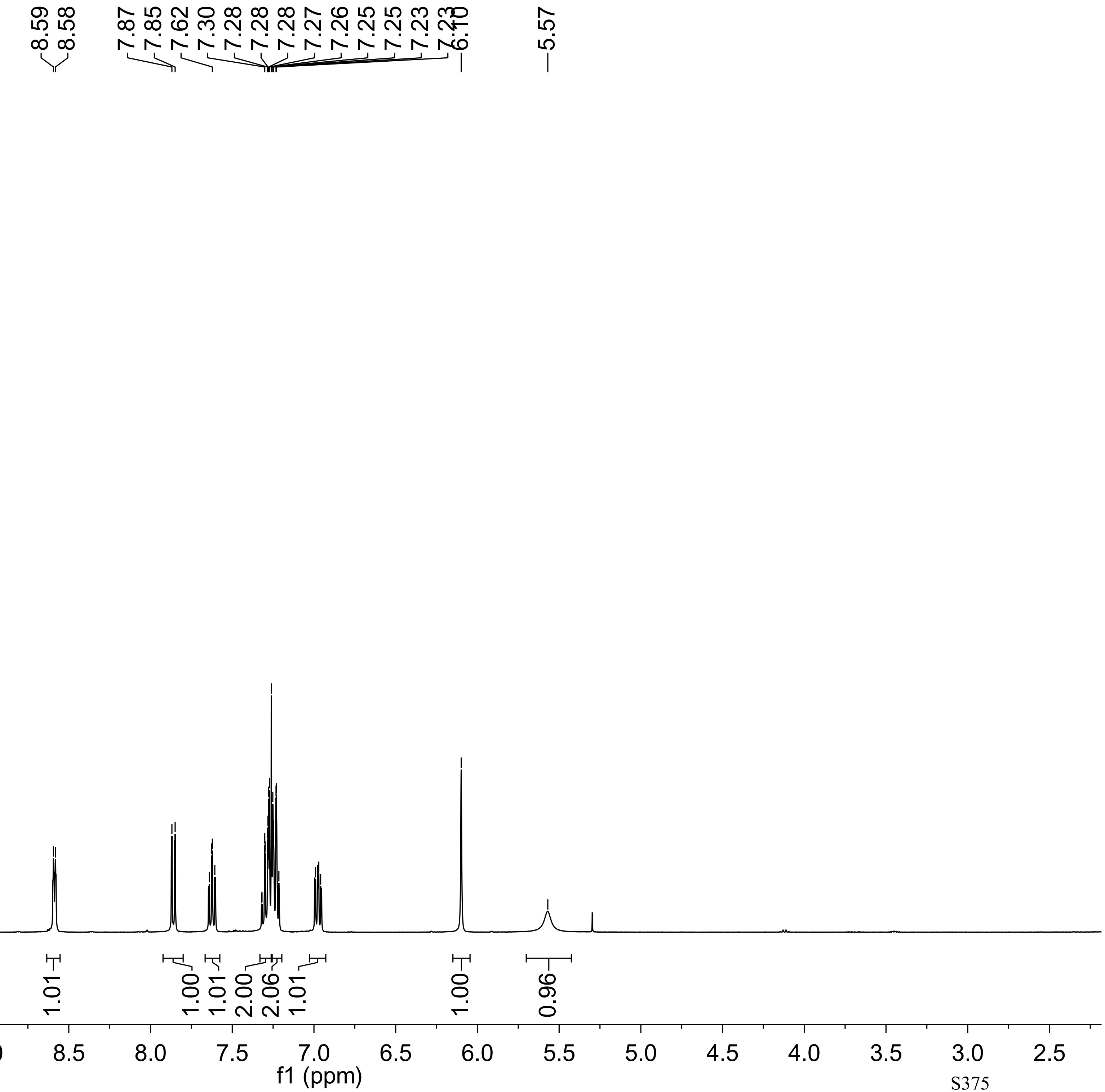

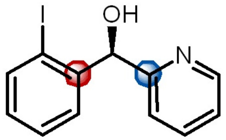

C2

Parameter	Value
1 Title	CFM-C2-0624
2 Comment	
3 Origin	Bruker BioSpin GmbH
4 Owner	nmrsu
5 Site	
6 Spectrometer	AVANCE NEO 400 MHZ DIGITAL NMR SPECTROMETER
7 Author	
8 Solvent	CDCl3
9 Temperature	298.1
10 Pulse Sequence	zgpg30
11 Experiment	1D
12 Number of Scans	256
13 Receiver Gain	53
14 Spectrometer Frequency	100.61
15 Spectral Width	23809.5
16 Lowest Frequency	-1847.0
17 Nucleus	13C
18 Acquired Size	32768
19 Spectral Size	32768

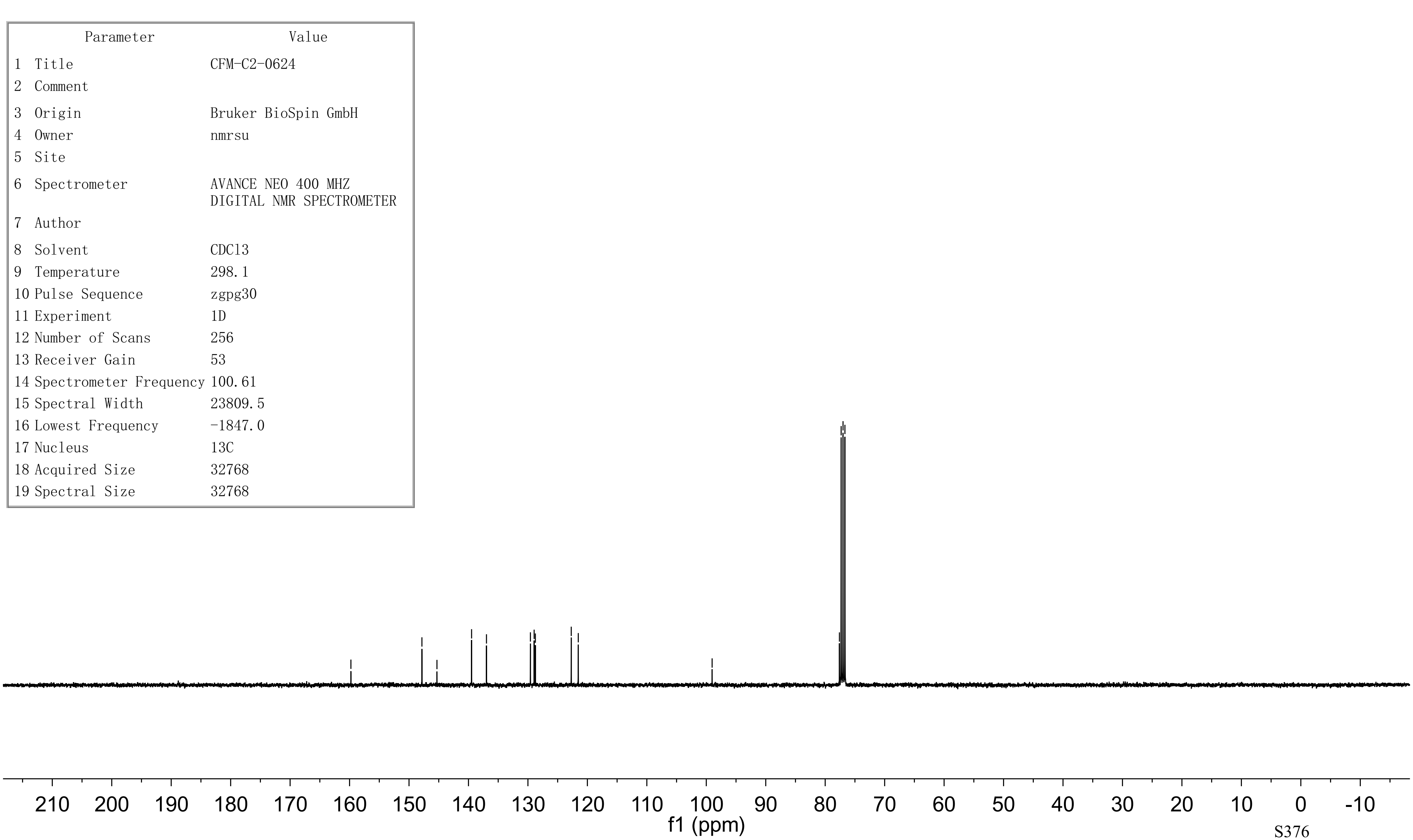

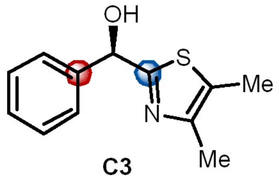

C3

Parameter	Value
1 Title	CFM-1106-9
2 Comment	
3 Origin	Bruker BioSpin GmbH
4 Owner	nmrsu
5 Site	
6 Spectrometer	Avance NEO 600
7 Author	
8 Solvent	CDCl3
9 Temperature	296.8
10 Pulse Sequence	zg30
11 Experiment	1D
12 Number of Scans	16
13 Receiver Gain	87
14 Relaxation Delay	1.0000
15 Pulse Width	10.0000
16 Acquisition Time	2.7525
17 Acquisition Date	2019-11-07T03:18:18
18 Modification Date	2019-11-07T09:53:10
19 Spectrometer Frequency	600.15
20 Spectral Width	11904.8
21 Lowest Frequency	-2261.3
22 Nucleus	1H
23 Acquired Size	32768
24 Spectral Size	65536

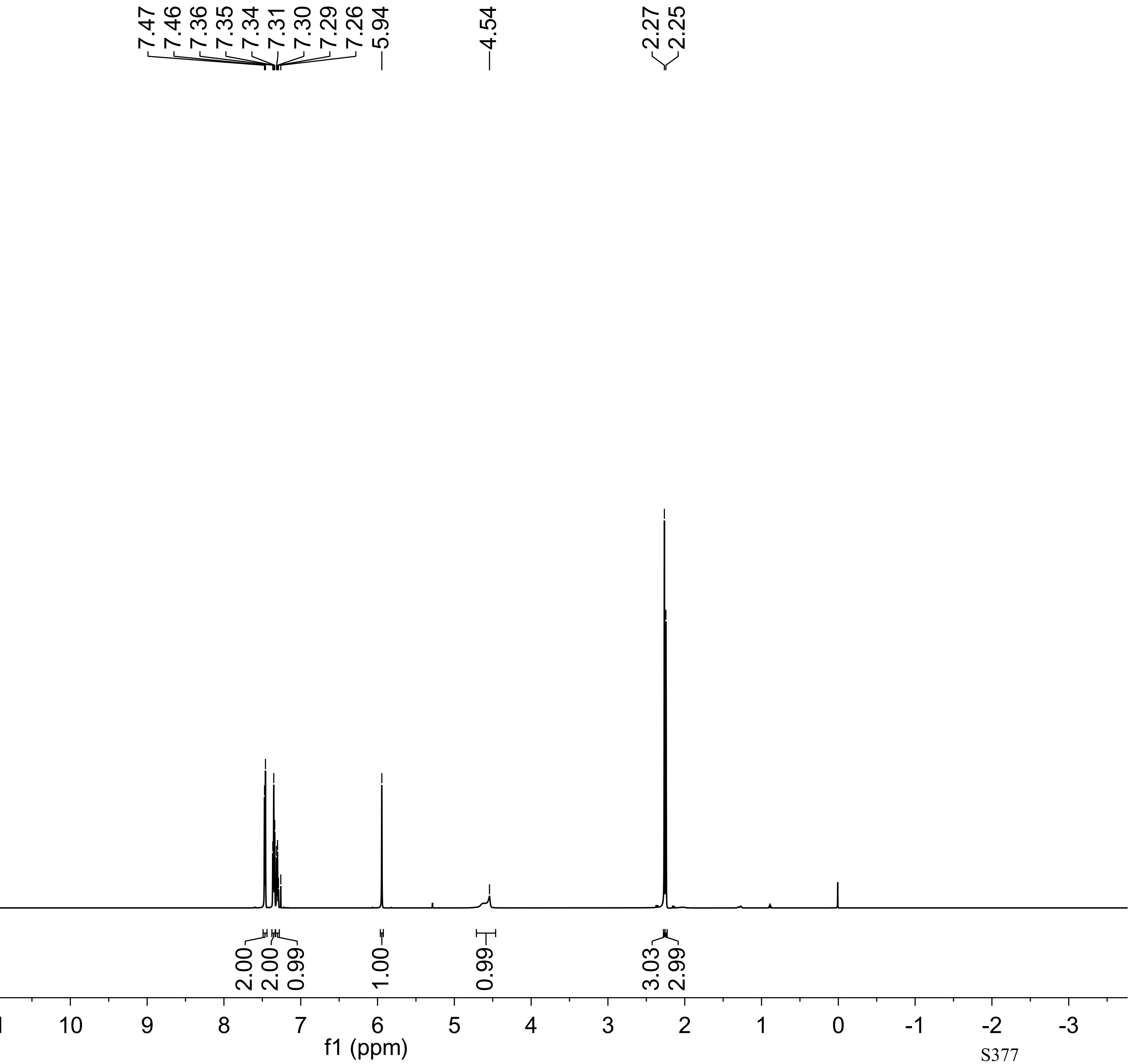

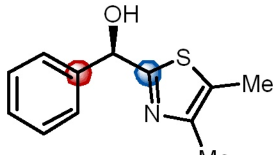

C3

Parameter	Value
1 Title	CFM-1106-9
2 Comment	
3 Origin	Bruker BioSpin GmbH
4 Owner	nmrsu
5 Site	
6 Spectrometer	Avance NEO 600
7 Author	
8 Solvent	CDCl3
9 Temperature	297.8
10 Pulse Sequence	zgpg30
11 Experiment	1D
12 Number of Scans	256
13 Receiver Gain	101
14 Relaxation Delay	2.0000
15 Pulse Width	12.0000
16 Acquisition Time	0.9175
17 Acquisition Date	2019-11-07T03:32:30
18 Modification Date	2019-11-07T09:53:10
19 Spectrometer Frequency	150.91
20 Spectral Width	35714.3
21 Lowest Frequency	-2777.8
22 Nucleus	13C
23 Acquired Size	32768
24 Spectral Size	32768

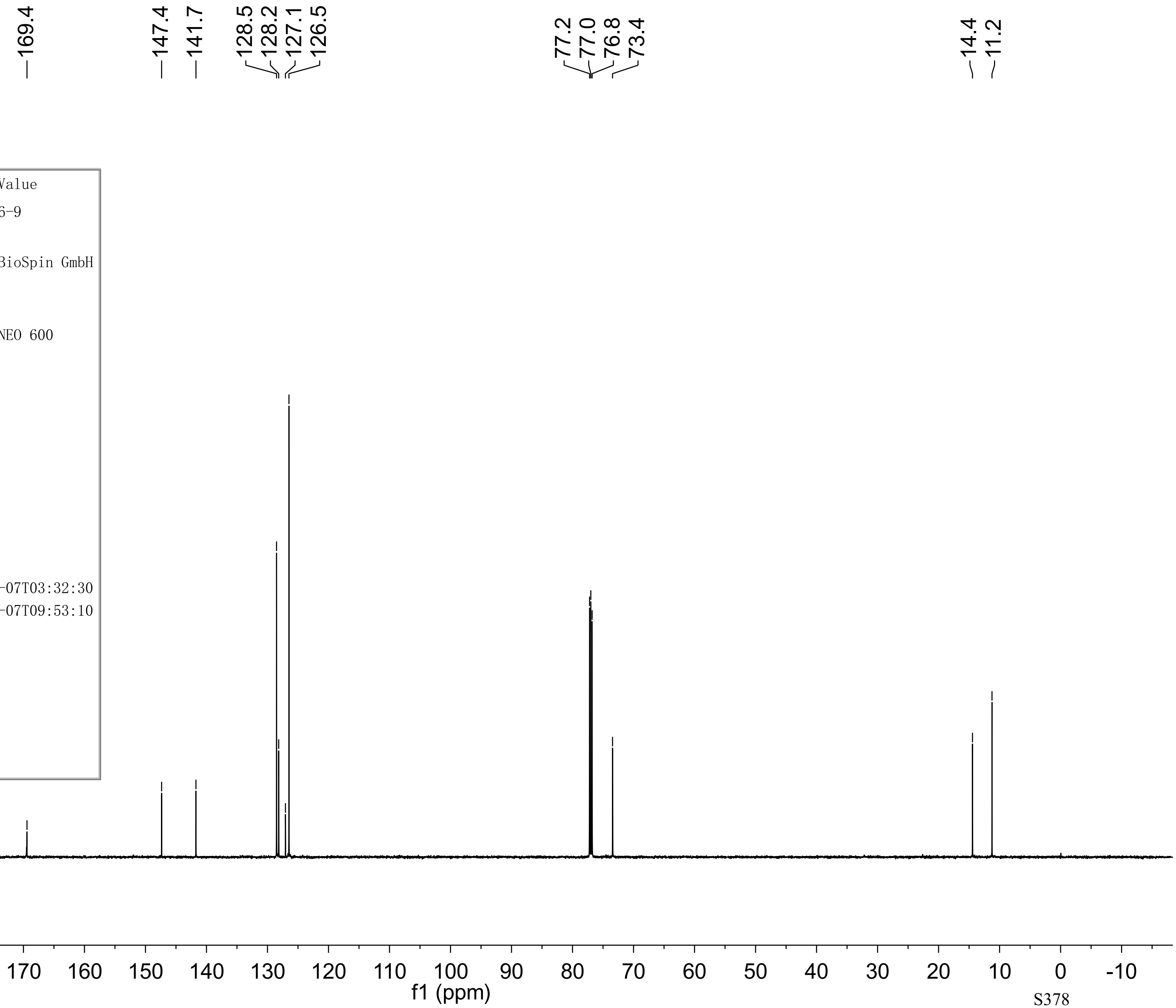

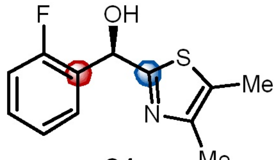

C4

Parameter	Value
1 Title	CFM-1106-10
2 Comment	
3 Origin	Bruker BioSpin GmbH
4 Owner	nmrsu
5 Site	
6 Spectrometer	Avance NEO 600
7 Author	
8 Solvent	CDCl3
9 Temperature	296.5
10 Pulse Sequence	zg30
11 Experiment	1D
12 Number of Scans	16
13 Receiver Gain	101
14 Relaxation Delay	1.0000
15 Pulse Width	10.0000
16 Acquisition Time	2.7525
17 Acquisition Date	2019-11-07T03:36:59
18 Modification Date	2019-11-07T09:53:11
19 Spectrometer Frequency	600.15
20 Spectral Width	11904.8
21 Lowest Frequency	-2261.0
22 Nucleus	1H
23 Acquired Size	32768
24 Spectral Size	65536

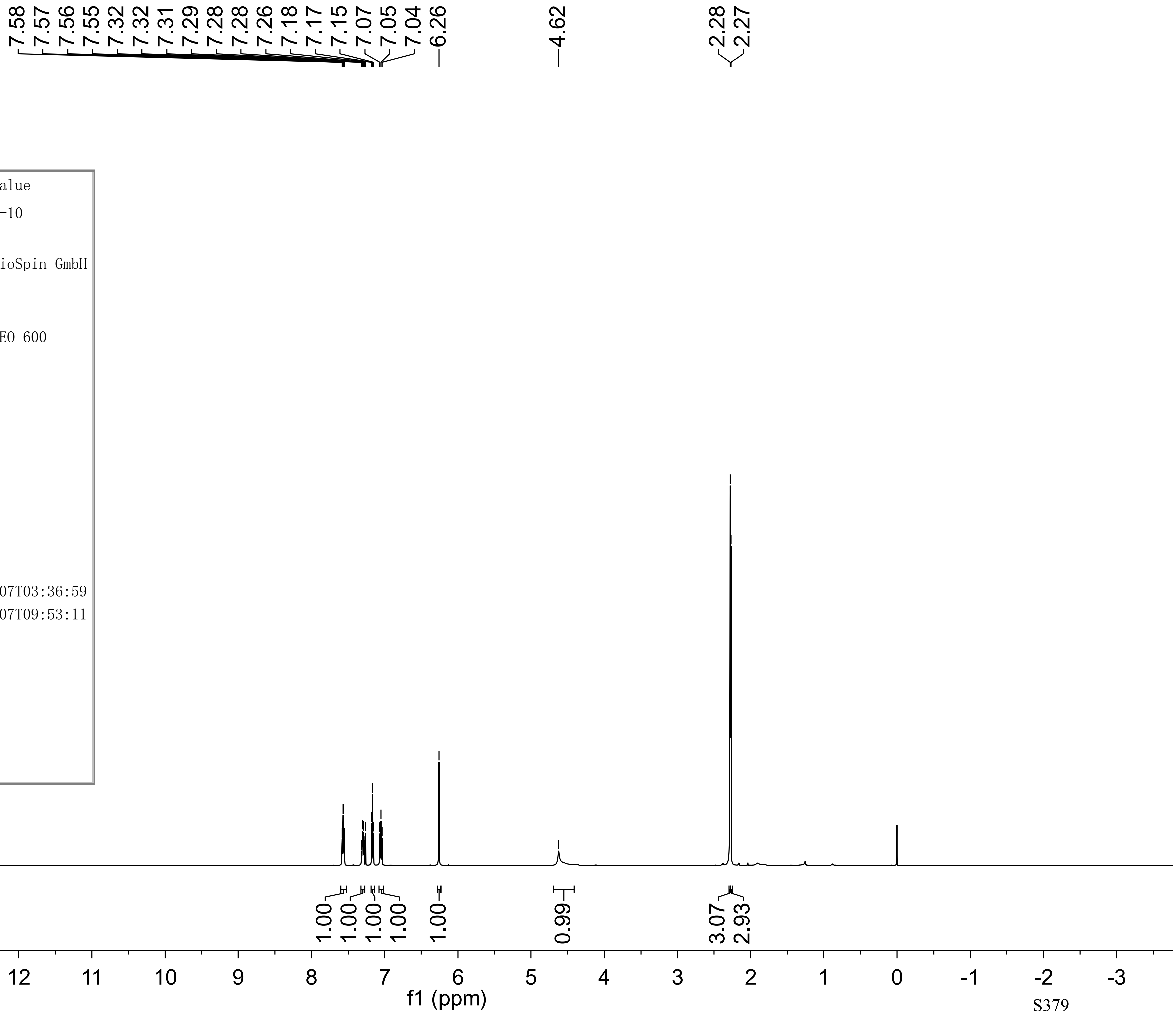

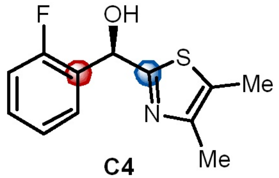

C4

Parameter		Value
1	Title	CFM-1106-10
2	Comment	
3	Origin	Bruker BioSpin GmbH
4	Owner	nmrsu
5	Site	
6	Spectrometer	Avance NEO 600
7	Author	
8	Solvent	CDCl3
9	Temperature	297.7
10	Pulse Sequence	zgpg30
11	Experiment	1D
12	Number of Scans	256
13	Receiver Gain	101
14	Relaxation Delay	2.0000
15	Pulse Width	12.0000
16	Acquisition Time	0.9175
17	Acquisition Date	2019-11-07T03:51:10
18	Modification Date	2019-11-07T09:53:11
19	Spectrometer Frequency	150.91
20	Spectral Width	35714.3
21	Lowest Frequency	-2773.9
22	Nucleus	13C
23	Acquired Size	32768
24	Spectral Size	32768

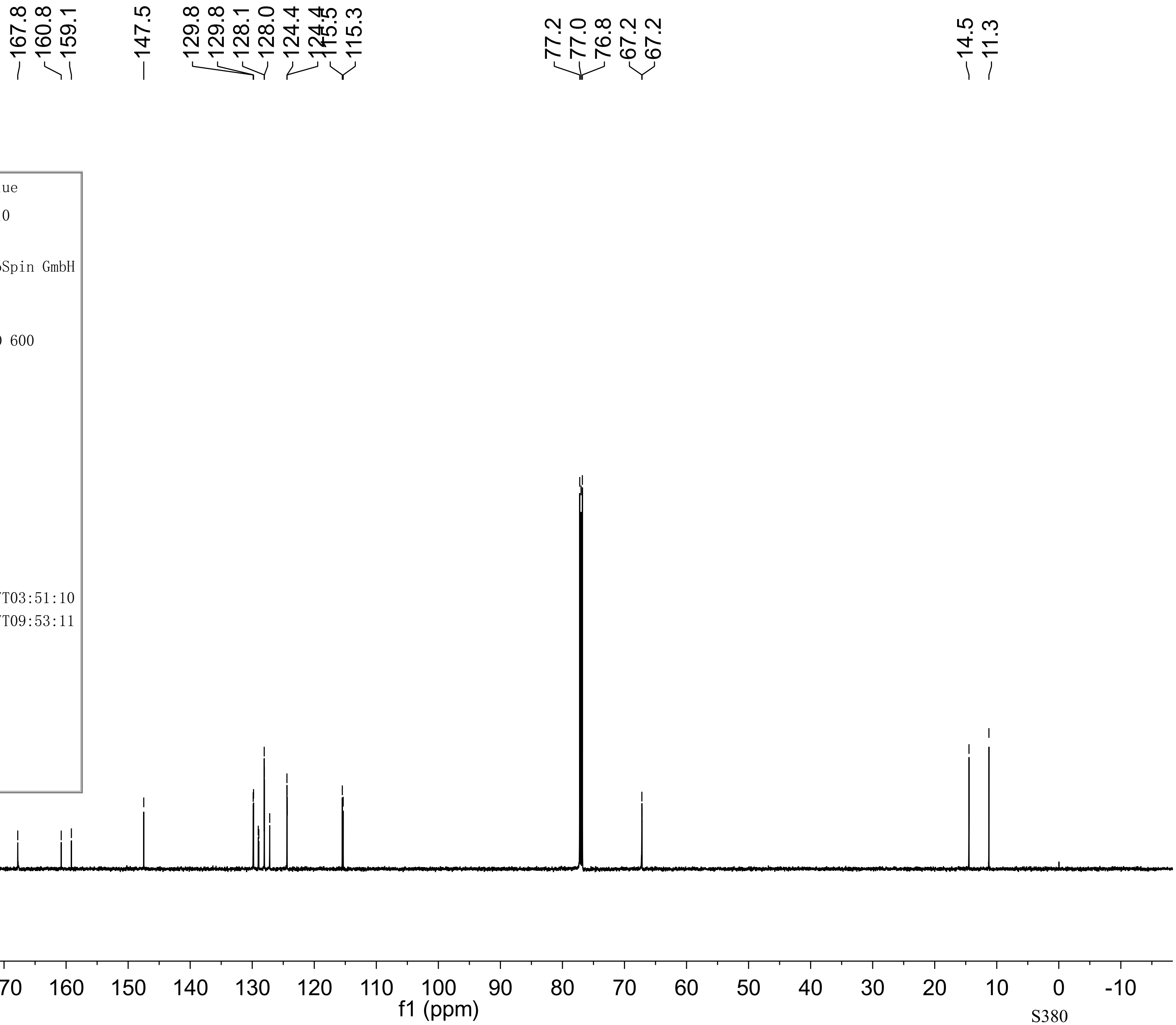

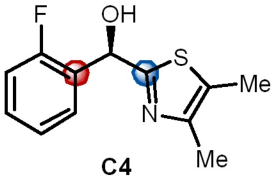

C4

	Parameter	Value
1	Title	CFM-1106-10
2	Comment	
3	Origin	Bruker BioSpin GmbH
4	Owner	nmrsu
5	Site	
6	Spectrometer	Avance NEO 600
7	Author	
8	Solvent	CDCl3
9	Temperature	296.9
10	Pulse Sequence	zg
11	Experiment	1D
12	Number of Scans	16
13	Receiver Gain	101
14	Relaxation Delay	1.0000
15	Pulse Width	12.0000
16	Acquisition Time	0.4981
17	Acquisition Date	2019-11-07T03:52:30
18	Modification Date	2019-11-07T09:53:11
19	Spectrometer Frequency	564.71
20	Spectral Width	131579.0
21	Lowest Frequency	-122260.0
22	Nucleus	19F
23	Acquired Size	65536
24	Spectral Size	65536

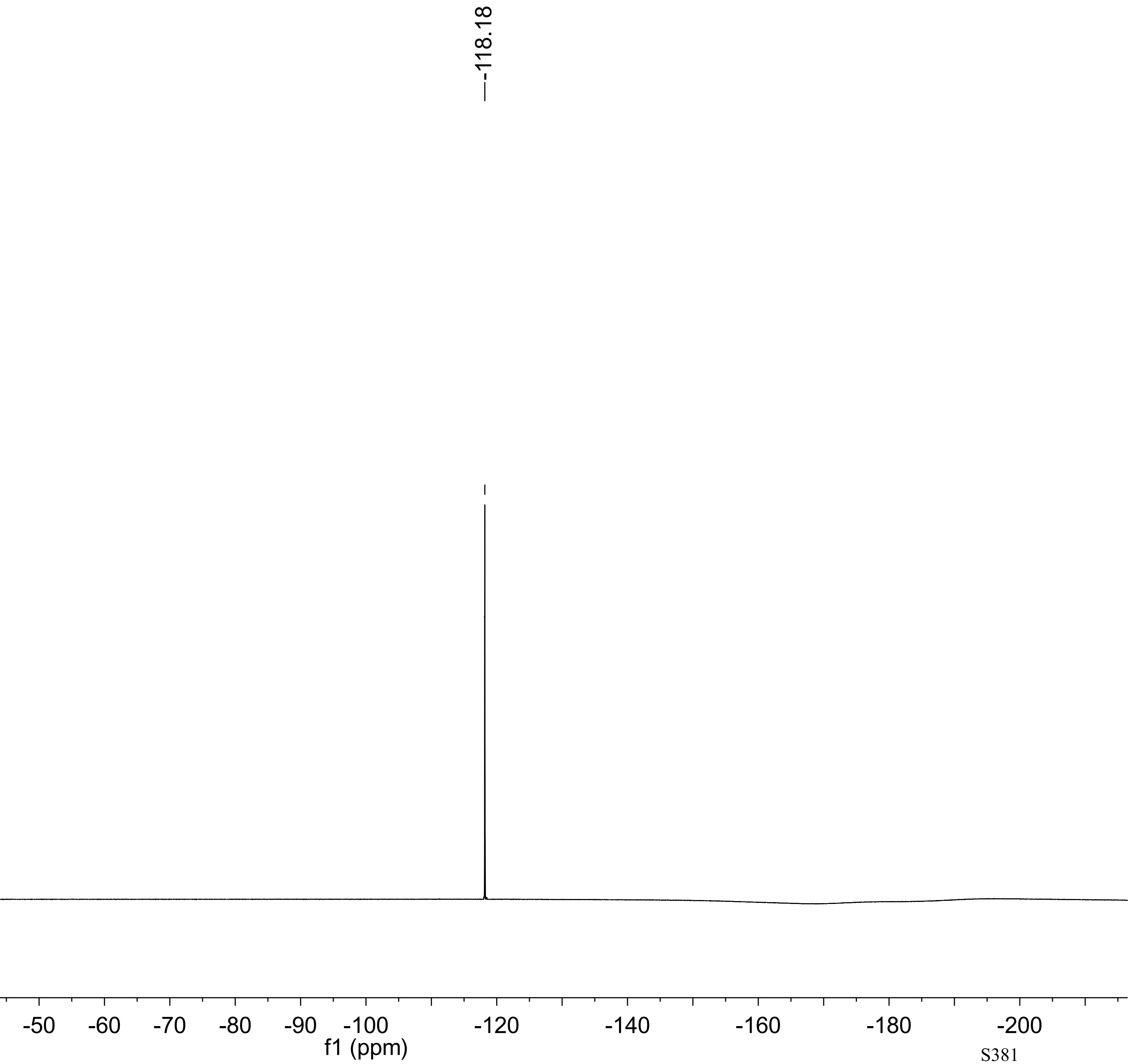

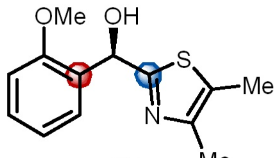

C5

Parameter	Value
1 Title	R-CFM-C3
2 Comment	
3 Origin	Bruker BioSpin GmbH
4 Owner	nmrsu
5 Site	
6 Spectrometer	Avance NEO 600
7 Author	
8 Solvent	CDCl3
9 Temperature	296.8
10 Pulse Sequence	zg30
11 Experiment	1D
12 Number of Scans	16
13 Receiver Gain	101
14 Relaxation Delay	1.0000
15 Pulse Width	10.0000
16 Acquisition Time	2.7525
17 Acquisition Date	2019-12-14T02:58:18
18 Modification Date	2019-12-14T09:18:10
19 Spectrometer Frequency	600.15
20 Spectral Width	11904.8
21 Lowest Frequency	-2261.1
22 Nucleus	1H
23 Acquired Size	32768
24 Spectral Size	65536

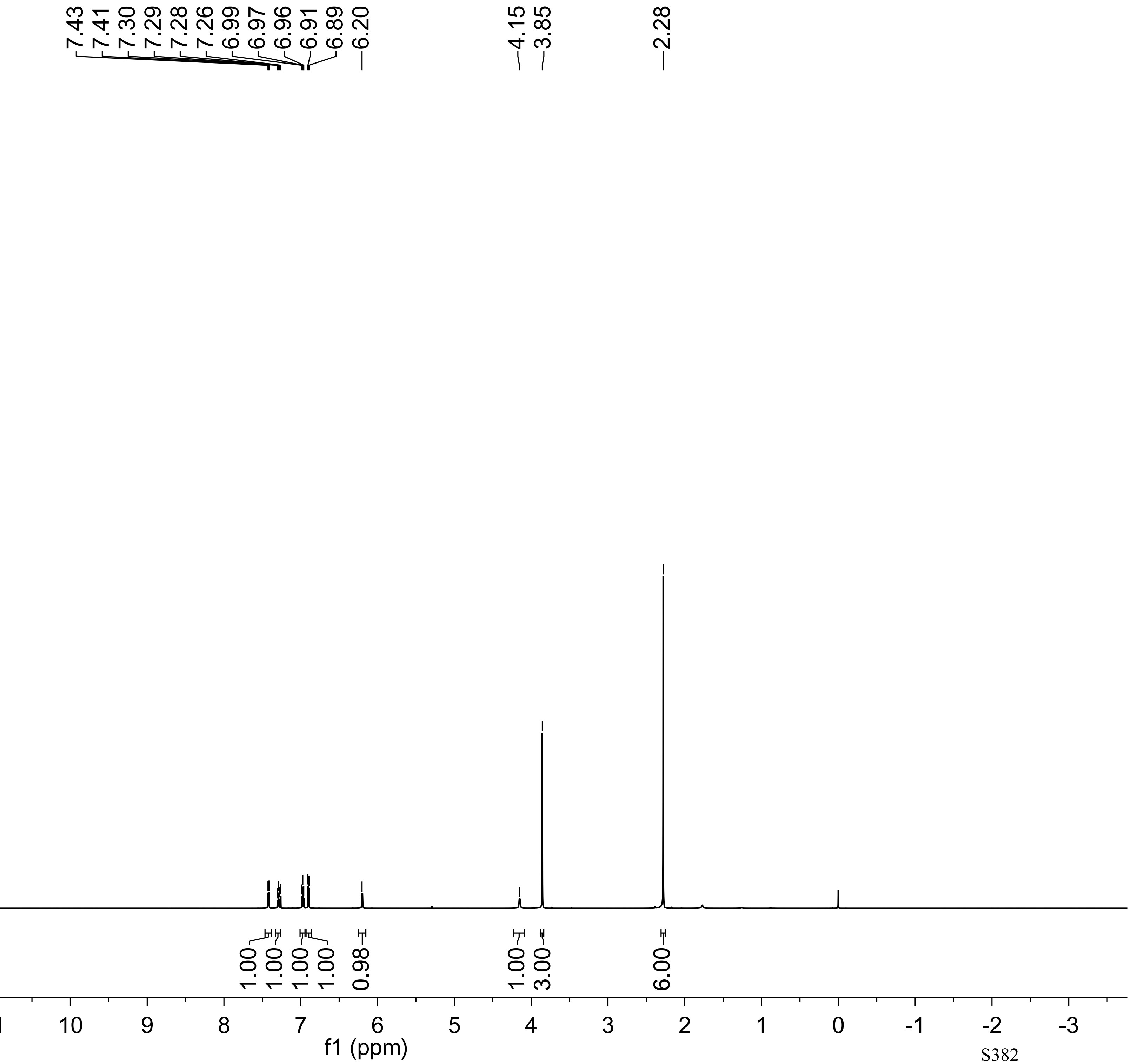

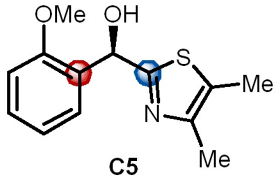

C5

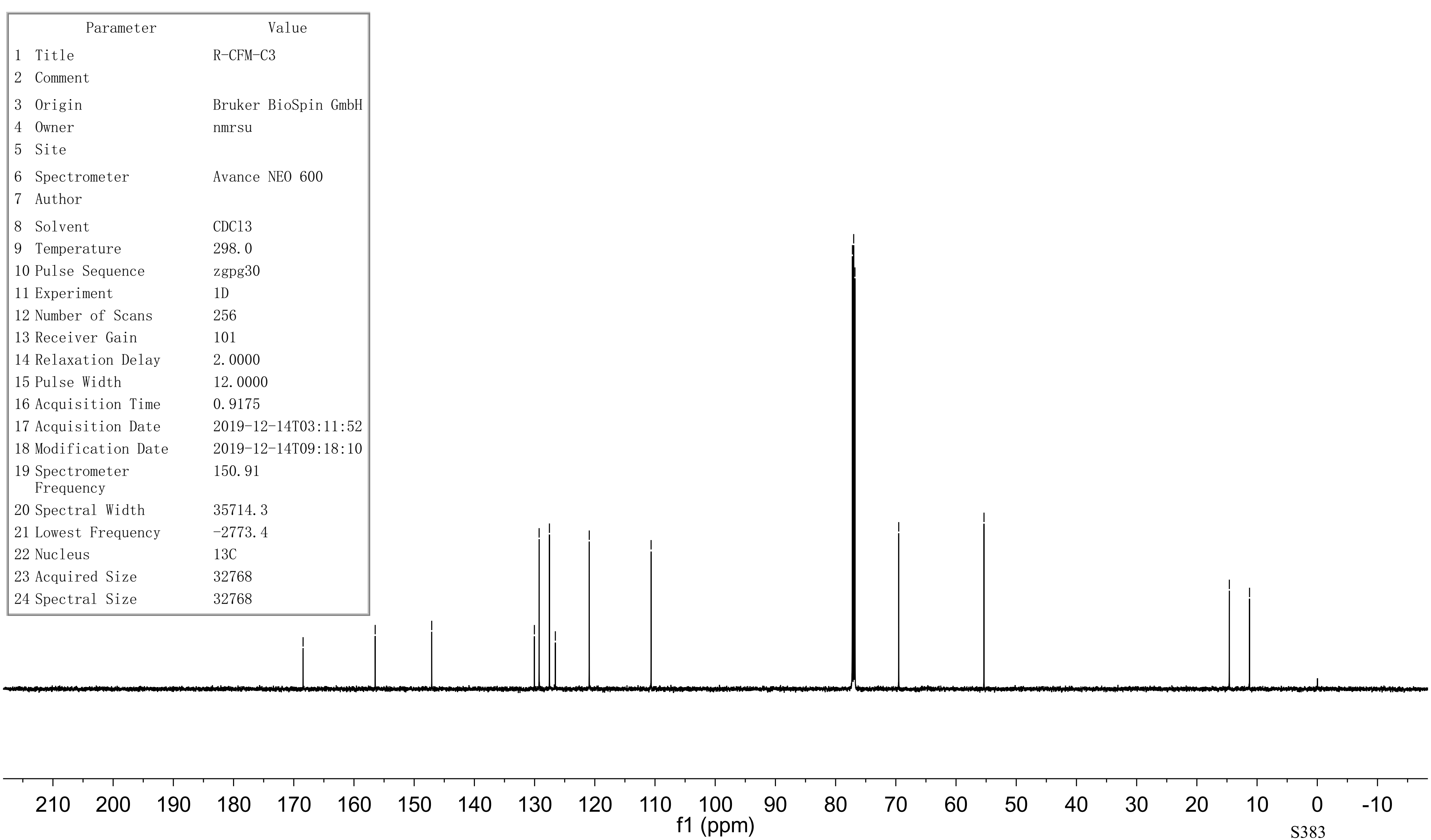

	Parameter	Value
1	Title	R-CFM-C3
2	Comment	
3	Origin	Bruker BioSpin GmbH
4	Owner	nmrsu
5	Site	
6	Spectrometer	Avance NEO 600
7	Author	
8	Solvent	CDCl3
9	Temperature	298.0
10	Pulse Sequence	zgpg30
11	Experiment	1D
12	Number of Scans	256
13	Receiver Gain	101
14	Relaxation Delay	2.0000
15	Pulse Width	12.0000
16	Acquisition Time	0.9175
17	Acquisition Date	2019-12-14T03:11:52
18	Modification Date	2019-12-14T09:18:10
19	Spectrometer Frequency	150.91
20	Spectral Width	35714.3
21	Lowest Frequency	-2773.4
22	Nucleus	13C
23	Acquired Size	32768
24	Spectral Size	32768

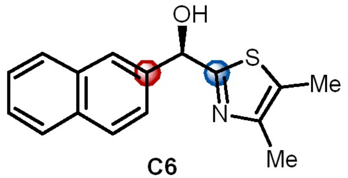

C6

	Parameter	Value
1	Title	R-CFM-C4
2	Comment	
3	Origin	Bruker BioSpin GmbH
4	Owner	nmrsu
5	Site	
6	Spectrometer	Avance NEO 600
7	Author	
8	Solvent	CDCl3
9	Temperature	297.0
10	Pulse Sequence	zg30
11	Experiment	1D
12	Number of Scans	16
13	Receiver Gain	101
14	Relaxation Delay	1.0000
15	Pulse Width	10.0000
16	Acquisition Time	2.7525
17	Acquisition Date	2019-12-14T03:18:05
18	Modification Date	2019-12-14T09:18:13
19	Spectrometer Frequency	600.15
20	Spectral Width	11904.8
21	Lowest Frequency	-2261.5
22	Nucleus	1H
23	Acquired Size	32768
24	Spectral Size	65536

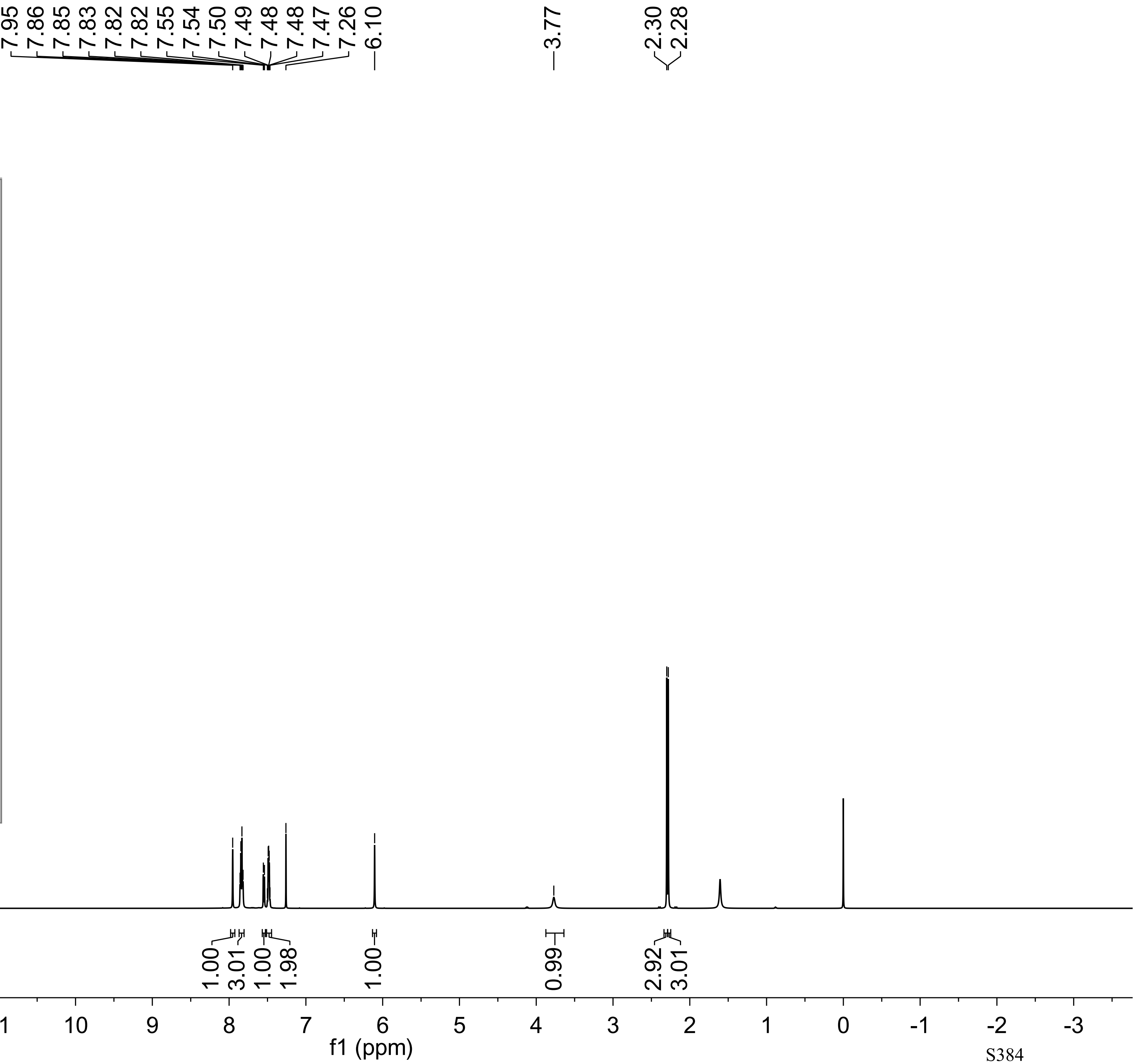

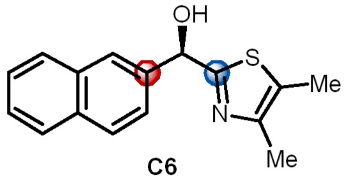

C6

Parameter	Value
1 Title	R-CFM-C4
2 Comment	
3 Origin	Bruker BioSpin GmbH
4 Owner	nmrsu
5 Site	
6 Spectrometer	Avance NEO 600
7 Author	
8 Solvent	CDCl3
9 Temperature	298.0
10 Pulse Sequence	zgpg30
11 Experiment	1D
12 Number of Scans	256
13 Receiver Gain	101
14 Relaxation Delay	2.0000
15 Pulse Width	12.0000
16 Acquisition Time	0.9175
17 Acquisition Date	2019-12-14T03:31:53
18 Modification Date	2019-12-14T09:18:14
19 Spectrometer Frequency	150.91
20 Spectral Width	35714.3
21 Lowest Frequency	-2770.4
22 Nucleus	13C
23 Acquired Size	32768
24 Spectral Size	32768

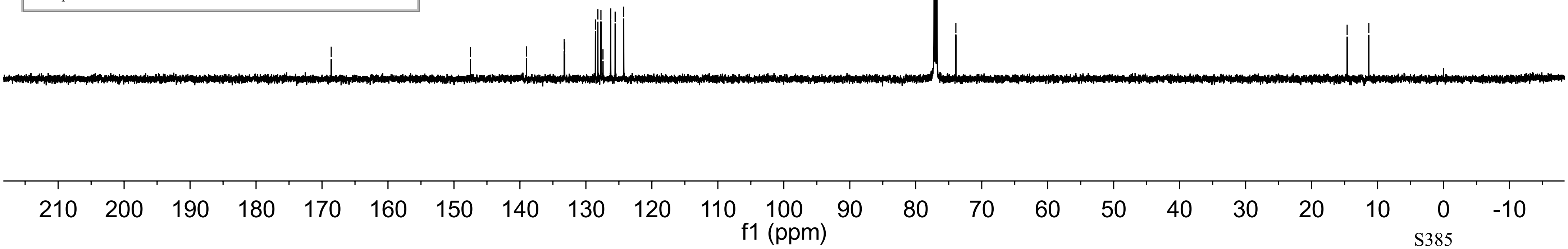

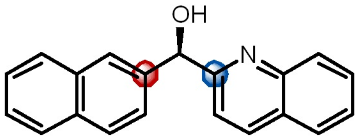

C7

Parameter	Value
1 Title	CFM-1106-6
2 Comment	
3 Origin	Bruker BioSpin GmbH
4 Owner	nmrsu
5 Site	
6 Spectrometer	Avance NEO 600
7 Author	
8 Solvent	CDCl3
9 Temperature	296.5
10 Pulse Sequence	zg30
11 Experiment	1D
12 Number of Scans	16
13 Receiver Gain	101
14 Relaxation Delay	1.0000
15 Pulse Width	10.0000
16 Acquisition Time	2.7525
17 Acquisition Date	2019-11-07T02:21:37
18 Modification Date	2019-11-07T09:53:08
19 Spectrometer Frequency	600.15
20 Spectral Width	11904.8
21 Lowest Frequency	-2261.3
22 Nucleus	1H
23 Acquired Size	32768
24 Spectral Size	65536

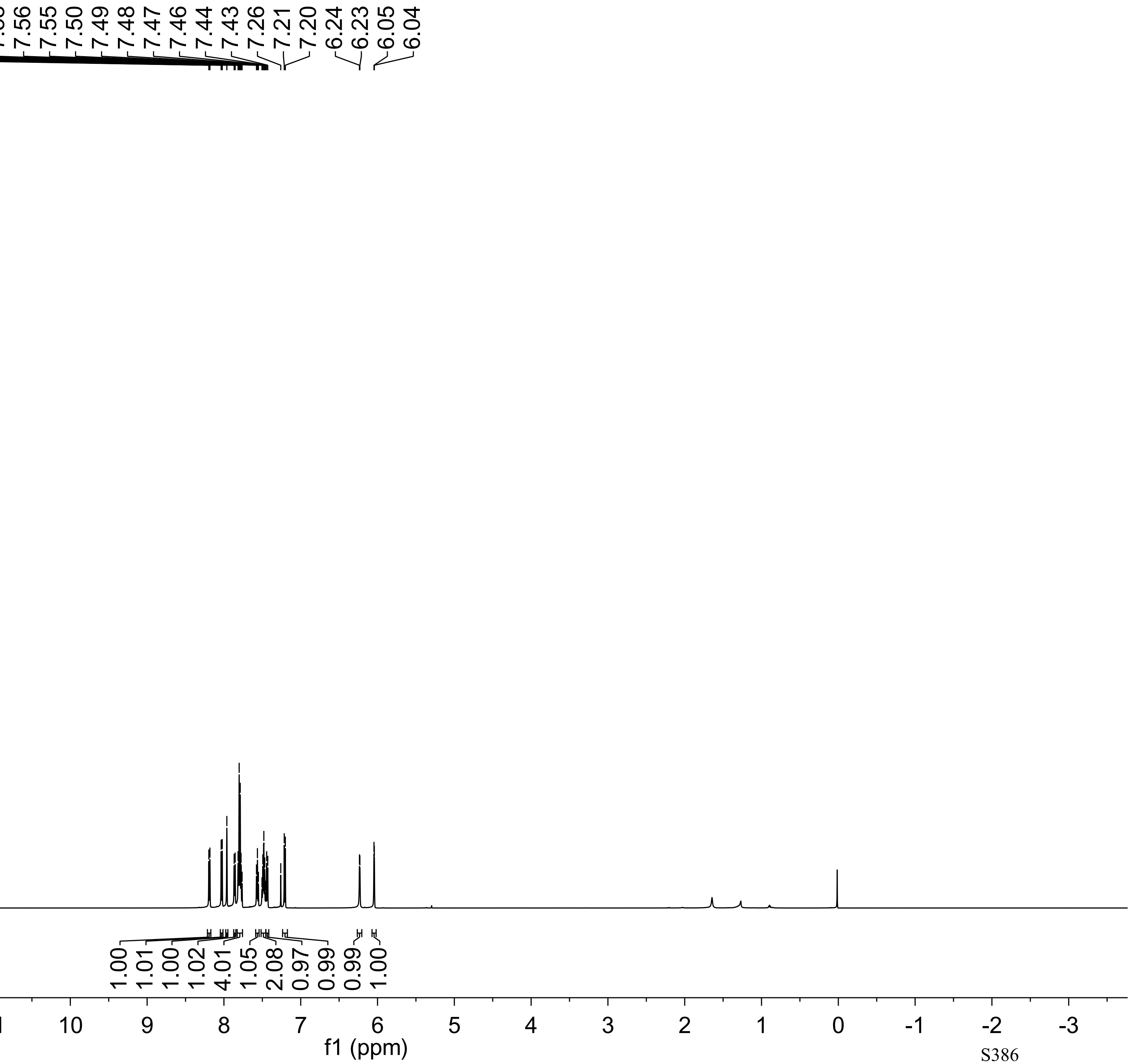

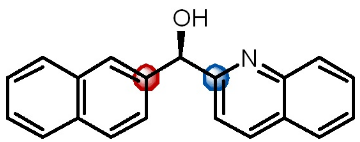

C7

Parameter		Value
1	Title	CFM-1106-6
2	Comment	
3	Origin	Bruker BioSpin GmbH
4	Owner	nmrsu
5	Site	
6	Spectrometer	Avance NEO 600
7	Author	
8	Solvent	CDCl3
9	Temperature	297.2
10	Pulse Sequence	zgpg30
11	Experiment	1D
12	Number of Scans	256
13	Receiver Gain	101
14	Relaxation Delay	2.0000
15	Pulse Width	12.0000
16	Acquisition Time	0.9175
17	Acquisition Date	2019-11-07T02:35:36
18	Modification Date	2019-11-07T09:53:09
19	Spectrometer Frequency	150.91
20	Spectral Width	35714.3
21	Lowest Frequency	-2775.1
22	Nucleus	13C
23	Acquired Size	32768
24	Spectral Size	32768

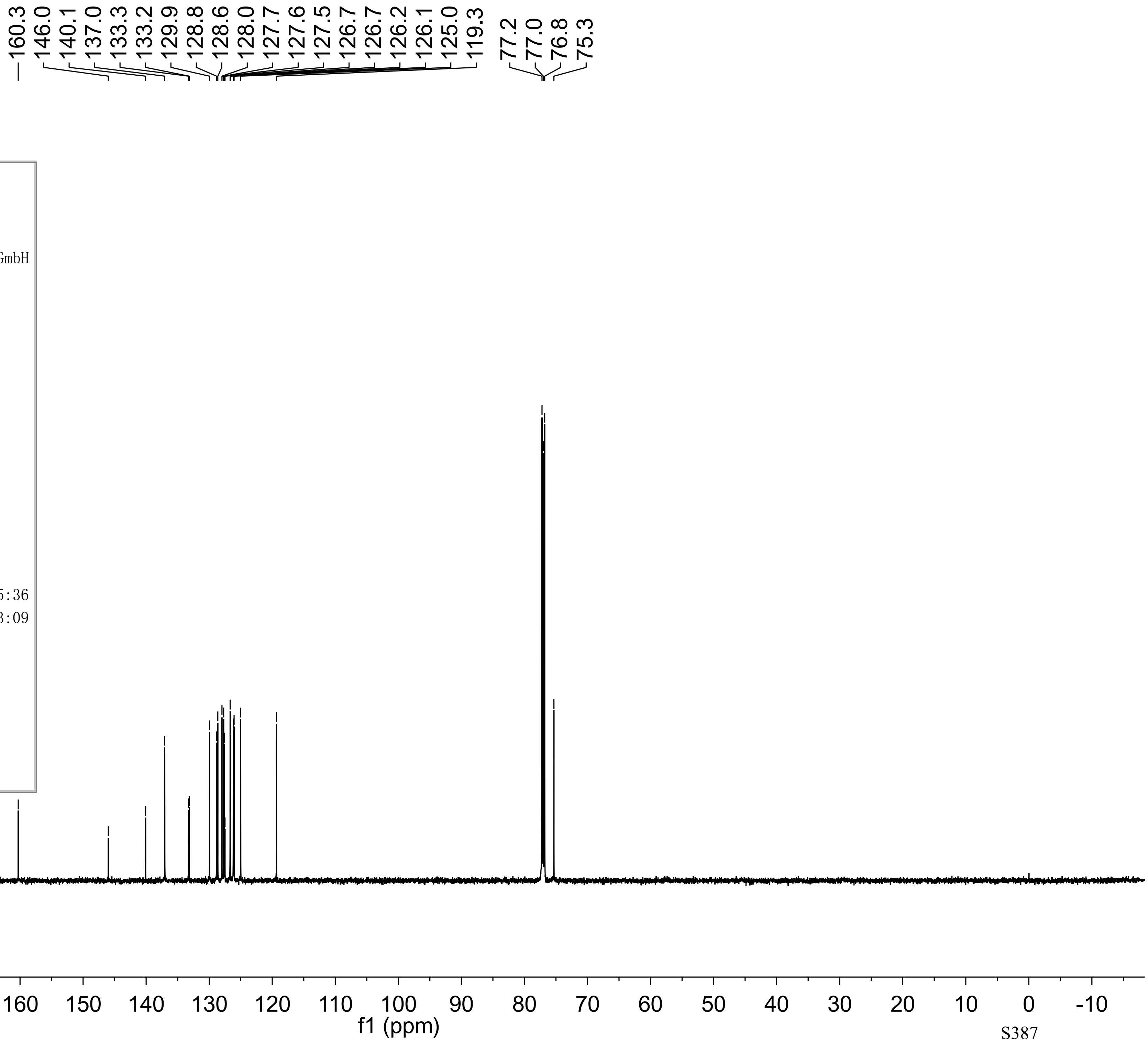

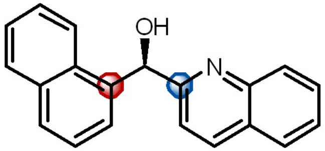

C8

Parameter		Value
1	Title	R-CFM-E10-1
2	Comment	
3	Origin	Bruker BioSpin GmbH
4	Owner	nmrsu
5	Site	
6	Spectrometer	Avance NEO 600
7	Author	
8	Solvent	CDCl3
9	Temperature	297.0
10	Pulse Sequence	zg30
11	Experiment	1D
12	Number of Scans	8
13	Receiver Gain	101
14	Relaxation Delay	1.0000
15	Pulse Width	10.0000
16	Acquisition Time	2.7525
17	Acquisition Date	2020-04-12T10:17:35
18	Modification Date	2020-04-12T14:02:36
19	Spectrometer Frequency	600.15
20	Spectral Width	11904.8
21	Lowest Frequency	-2260.7
22	Nucleus	1H
23	Acquired Size	32768
24	Spectral Size	65536

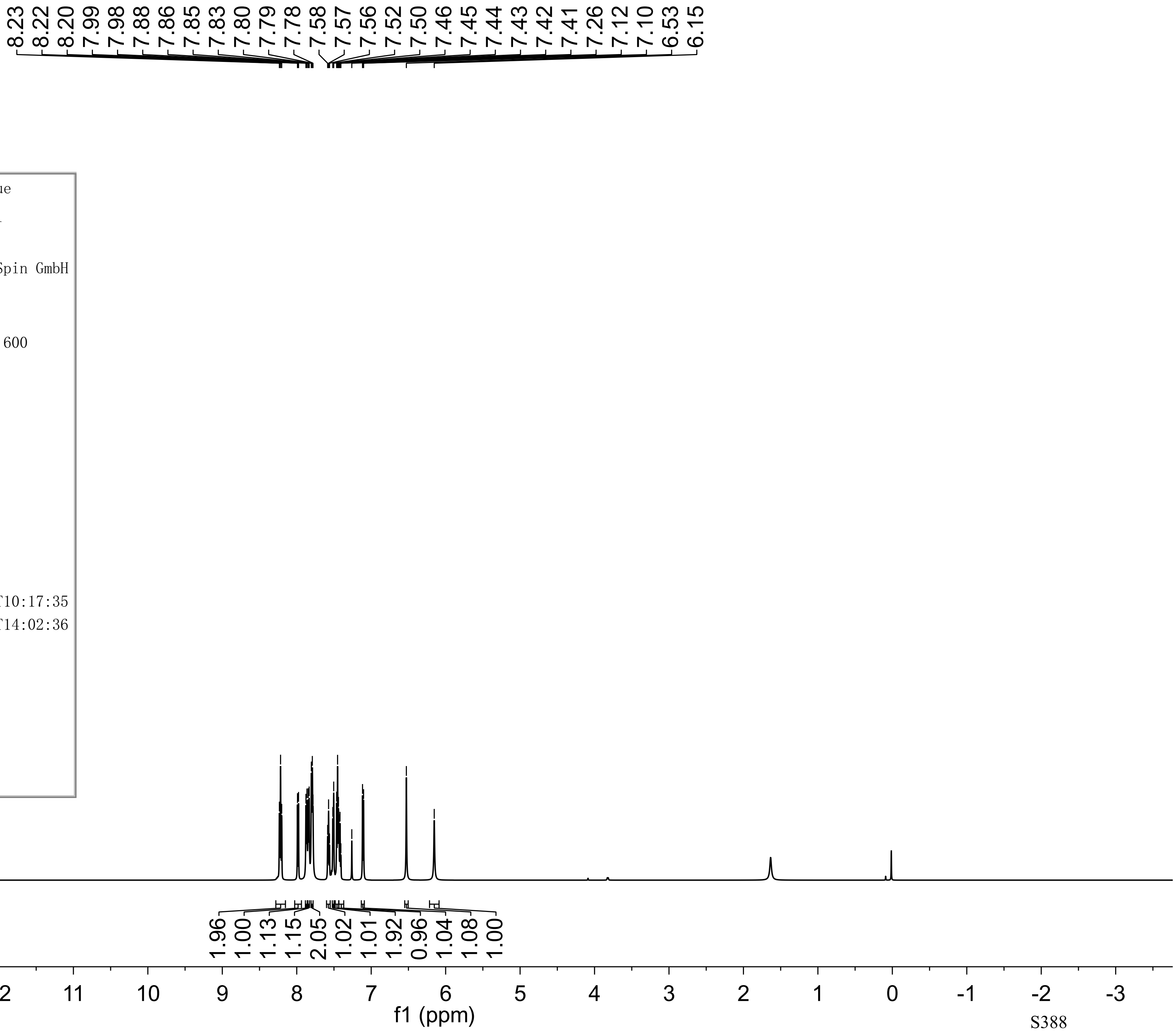

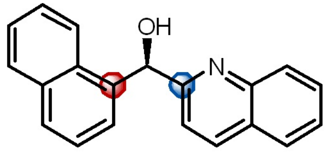

C8

	Parameter	Value
1	Title	R-CFM-E10-1
2	Comment	
3	Origin	Bruker BioSpin GmbH
4	Owner	nmrsu
5	Site	
6	Spectrometer	Avance NEO 600
7	Author	
8	Solvent	CDCl3
9	Temperature	298.3
10	Pulse Sequence	zgpg30
11	Experiment	1D
12	Number of Scans	256
13	Receiver Gain	101
14	Relaxation Delay	2.0000
15	Pulse Width	12.0000
16	Acquisition Time	0.9175
17	Acquisition Date	2020-04-12T10:31:54
18	Modification Date	2020-04-12T14:02:36
19	Spectrometer Frequency	150.91
20	Spectral Width	35714.3
21	Lowest Frequency	-2774.3
22	Nucleus	13C
23	Acquired Size	32768
24	Spectral Size	32768

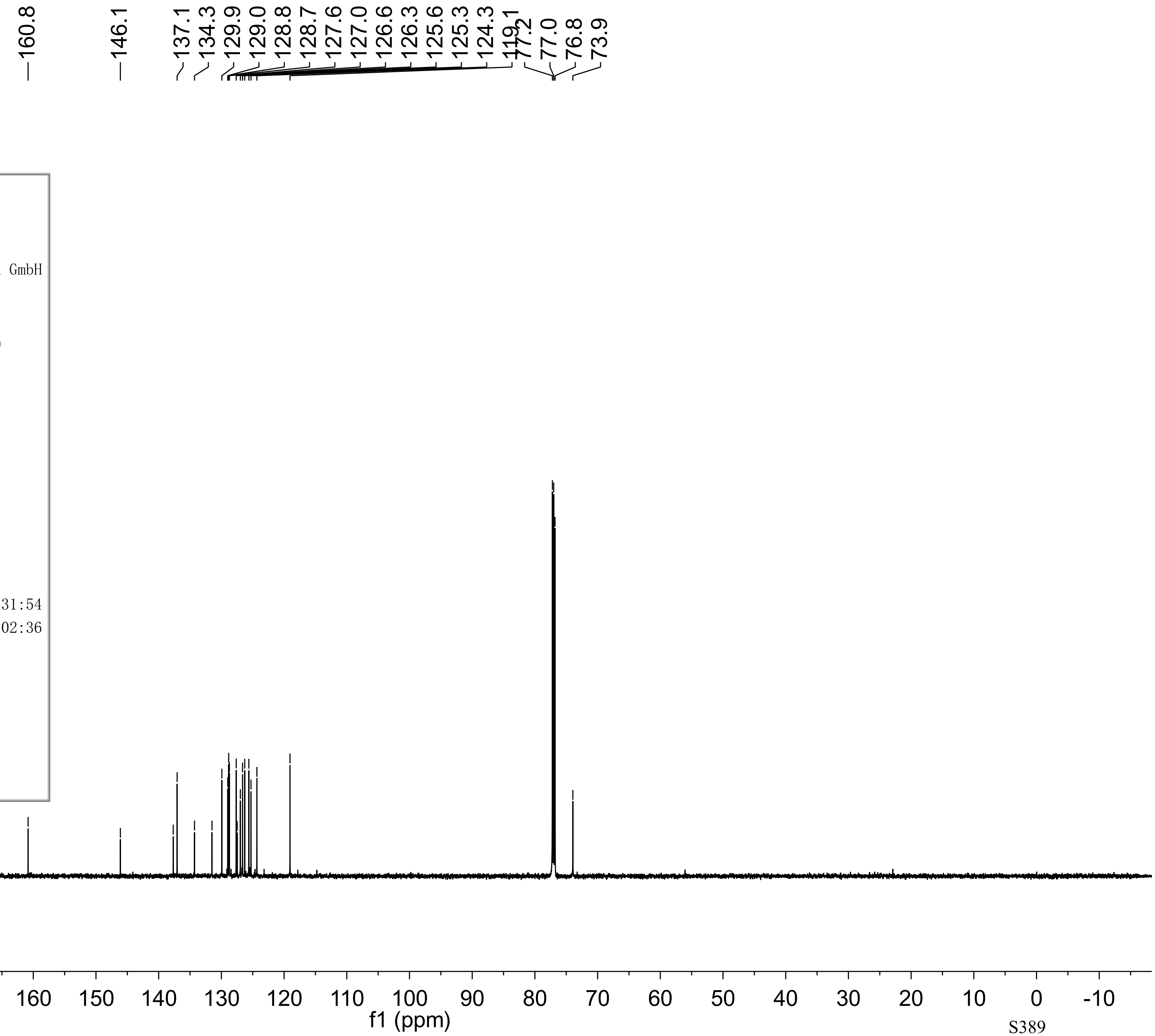

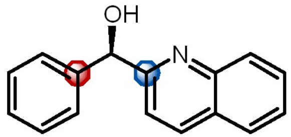

C9

	Parameter	Value
1	Title	R-CFM-E1-2
2	Comment	
3	Origin	Bruker BioSpin GmbH
4	Owner	nmrsu
5	Site	
6	Spectrometer	Avance NEO 600
7	Author	
8	Solvent	CDCl3
9	Temperature	296.7
10	Pulse Sequence	zg30
11	Experiment	1D
12	Number of Scans	8
13	Receiver Gain	101
14	Relaxation Delay	1.0000
15	Pulse Width	10.0000
16	Acquisition Time	2.7525
17	Acquisition Date	2020-04-12T09:37:29
18	Modification Date	2020-04-12T09:56:52
19	Spectrometer Frequency	600.15
20	Spectral Width	11904.8
21	Lowest Frequency	-2261.6
22	Nucleus	1H
23	Acquired Size	32768
24	Spectral Size	65536

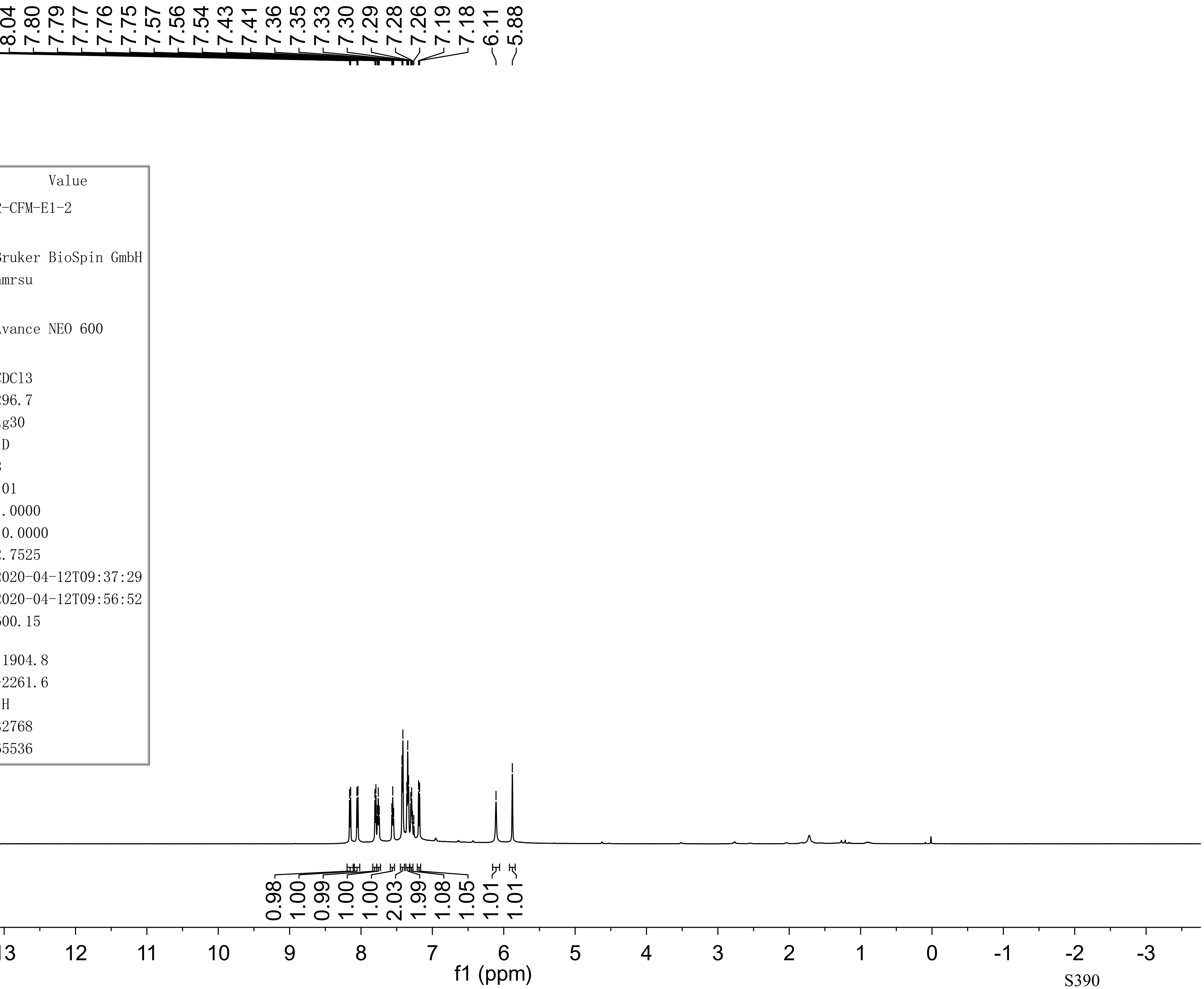

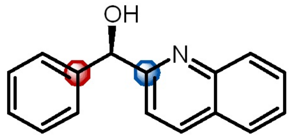

C9

Parameter	Value
1 Title	R-CFM-E1-2
2 Comment	
3 Origin	Bruker BioSpin GmbH
4 Owner	nmrsu
5 Site	
6 Spectrometer	Avance NEO 600
7 Author	
8 Solvent	CDCl3
9 Temperature	298.0
10 Pulse Sequence	zgpg30
11 Experiment	1D
12 Number of Scans	256
13 Receiver Gain	101
14 Relaxation Delay	2.0000
15 Pulse Width	12.0000
16 Acquisition Time	0.9175
17 Acquisition Date	2020-04-12T09:51:46
18 Modification Date	2020-04-12T09:56:52
19 Spectrometer Frequency	150.91
20 Spectral Width	35714.3
21 Lowest Frequency	-2776.8
22 Nucleus	13C
23 Acquired Size	32768
24 Spectral Size	32768

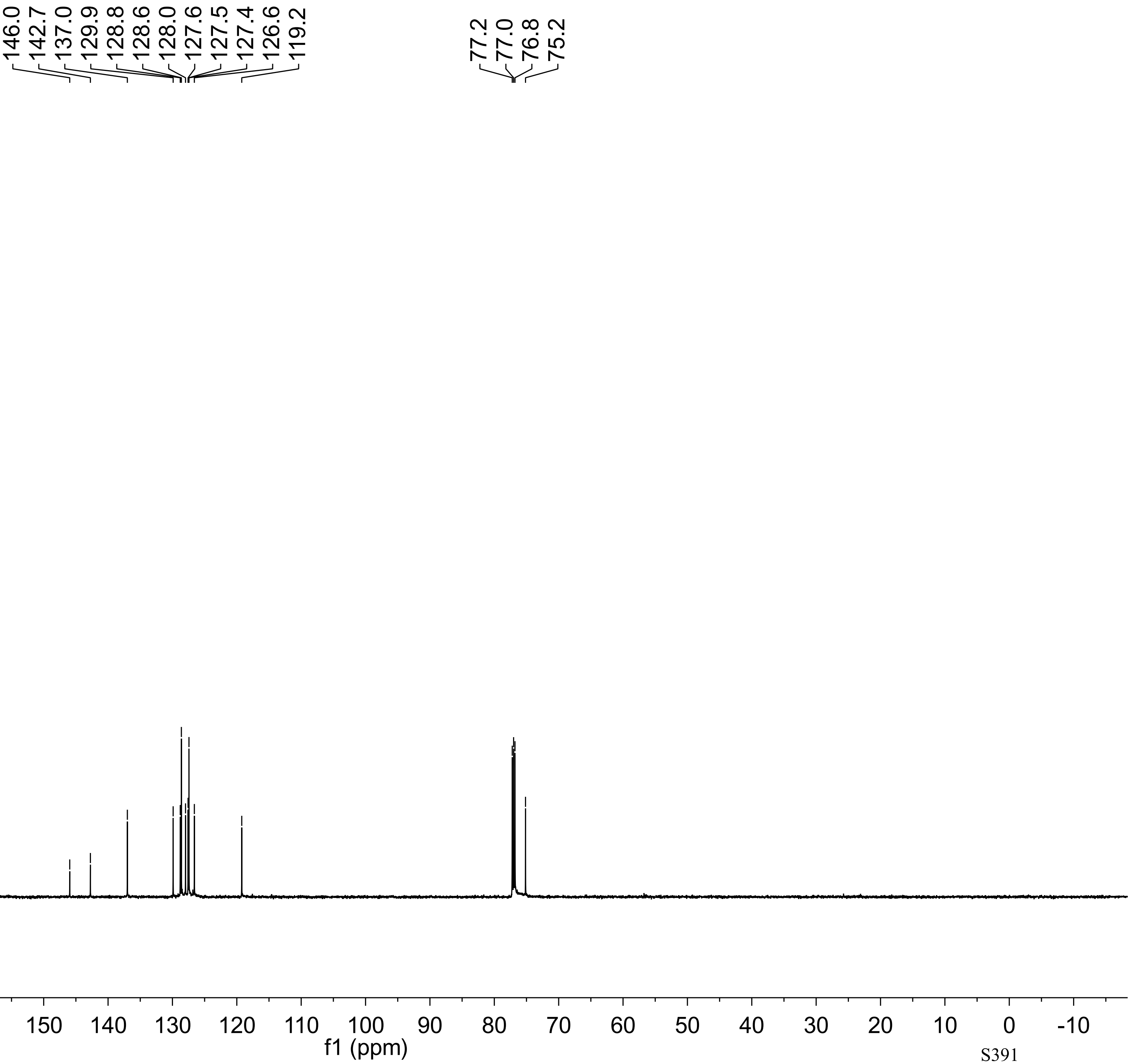

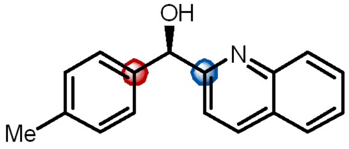

C10

Parameter	Value
1 Title	CFM-1106-4
2 Comment	
3 Origin	Bruker BioSpin GmbH
4 Owner	nmrsu
5 Site	
6 Spectrometer	Avance NEO 600
7 Author	
8 Solvent	CDCl3
9 Temperature	296.3
10 Pulse Sequence	zg30
11 Experiment	1D
12 Number of Scans	16
13 Receiver Gain	101
14 Relaxation Delay	1.0000
15 Pulse Width	10.0000
16 Acquisition Time	2.7525
17 Acquisition Date	2019-11-07T01:43:22
18 Modification Date	2019-11-07T09:53:09
19 Spectrometer Frequency	600.15
20 Spectral Width	11904.8
21 Lowest Frequency	-2260.9
22 Nucleus	1H
23 Acquired Size	32768
24 Spectral Size	65536

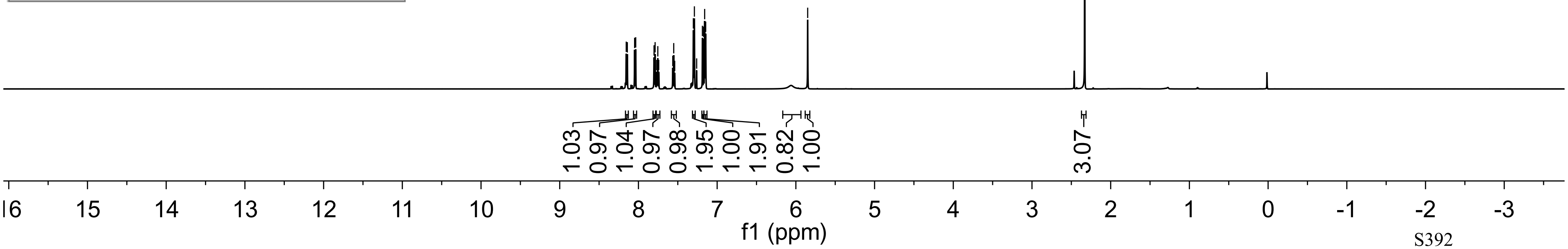

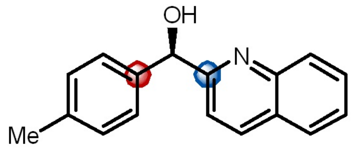

C10

Parameter	Value
1 Title	CFM-1106-4
2 Comment	
3 Origin	Bruker BioSpin GmbH
4 Owner	nmrsu
5 Site	
6 Spectrometer	Avance NEO 600
7 Author	
8 Solvent	CDCl3
9 Temperature	297.7
10 Pulse Sequence	zgpg30
11 Experiment	1D
12 Number of Scans	256
13 Receiver Gain	101
14 Relaxation Delay	2.0000
15 Pulse Width	12.0000
16 Acquisition Time	0.9175
17 Acquisition Date	2019-11-07T01:57:07
18 Modification Date	2019-11-07T09:53:09
19 Spectrometer Frequency	150.91
20 Spectral Width	35714.3
21 Lowest Frequency	-2775.9
22 Nucleus	13C
23 Acquired Size	32768
24 Spectral Size	32768

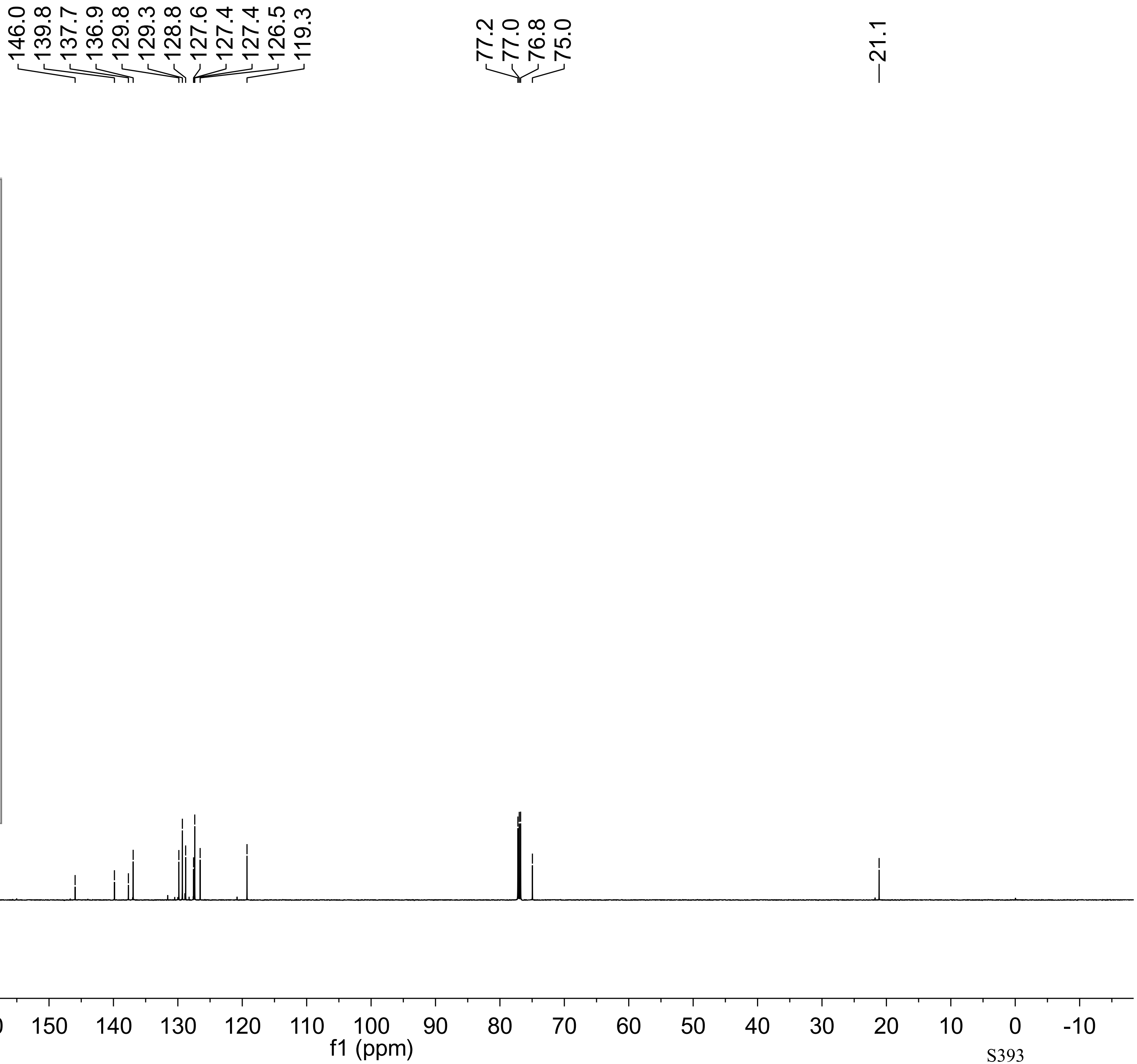

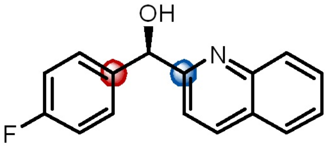

C11

Parameter	Value
1 Title	CFM-1106-2
2 Comment	
3 Origin	Bruker BioSpin GmbH
4 Owner	nmrsu
5 Site	
6 Spectrometer	Avance NEO 600
7 Author	
8 Solvent	CDCl3
9 Temperature	296.1
10 Pulse Sequence	zg30
11 Experiment	1D
12 Number of Scans	16
13 Receiver Gain	80
14 Relaxation Delay	1.0000
15 Pulse Width	10.0000
16 Acquisition Time	2.7525
17 Acquisition Date	2019-11-07T00:31:14
18 Modification Date	2019-11-07T09:53:08
19 Spectrometer Frequency	600.15
20 Spectral Width	11904.8
21 Lowest Frequency	-2261.0
22 Nucleus	1H
23 Acquired Size	32768
24 Spectral Size	65536

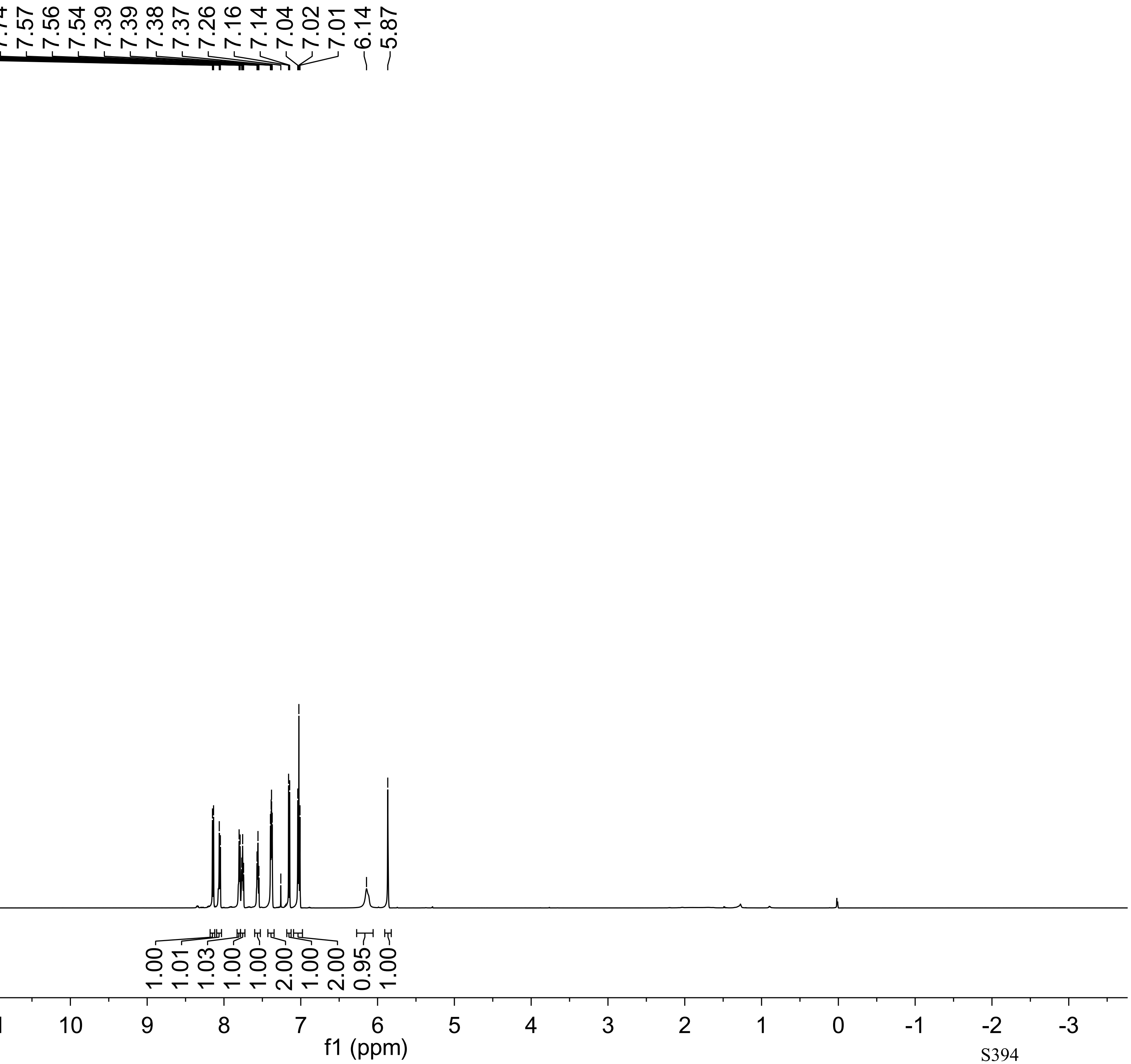

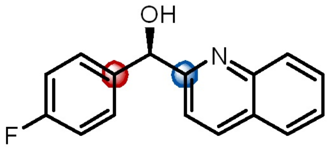

C11

Parameter	Value
1 Title	CFM-1106-2
2 Comment	
3 Origin	Bruker BioSpin GmbH
4 Owner	nmrsu
5 Site	
6 Spectrometer	Avance NEO 600
7 Author	
8 Solvent	CDCl3
9 Temperature	296.8
10 Pulse Sequence	zgpg30
11 Experiment	1D
12 Number of Scans	201
13 Receiver Gain	101
14 Relaxation Delay	2.0000
15 Pulse Width	12.0000
16 Acquisition Time	0.9175
17 Acquisition Date	2019-11-07T00:42:36
18 Modification Date	2019-11-07T09:53:08
19 Spectrometer Frequency	150.91
20 Spectral Width	35714.3
21 Lowest Frequency	-2782.5
22 Nucleus	13C
23 Acquired Size	32768
24 Spectral Size	32768

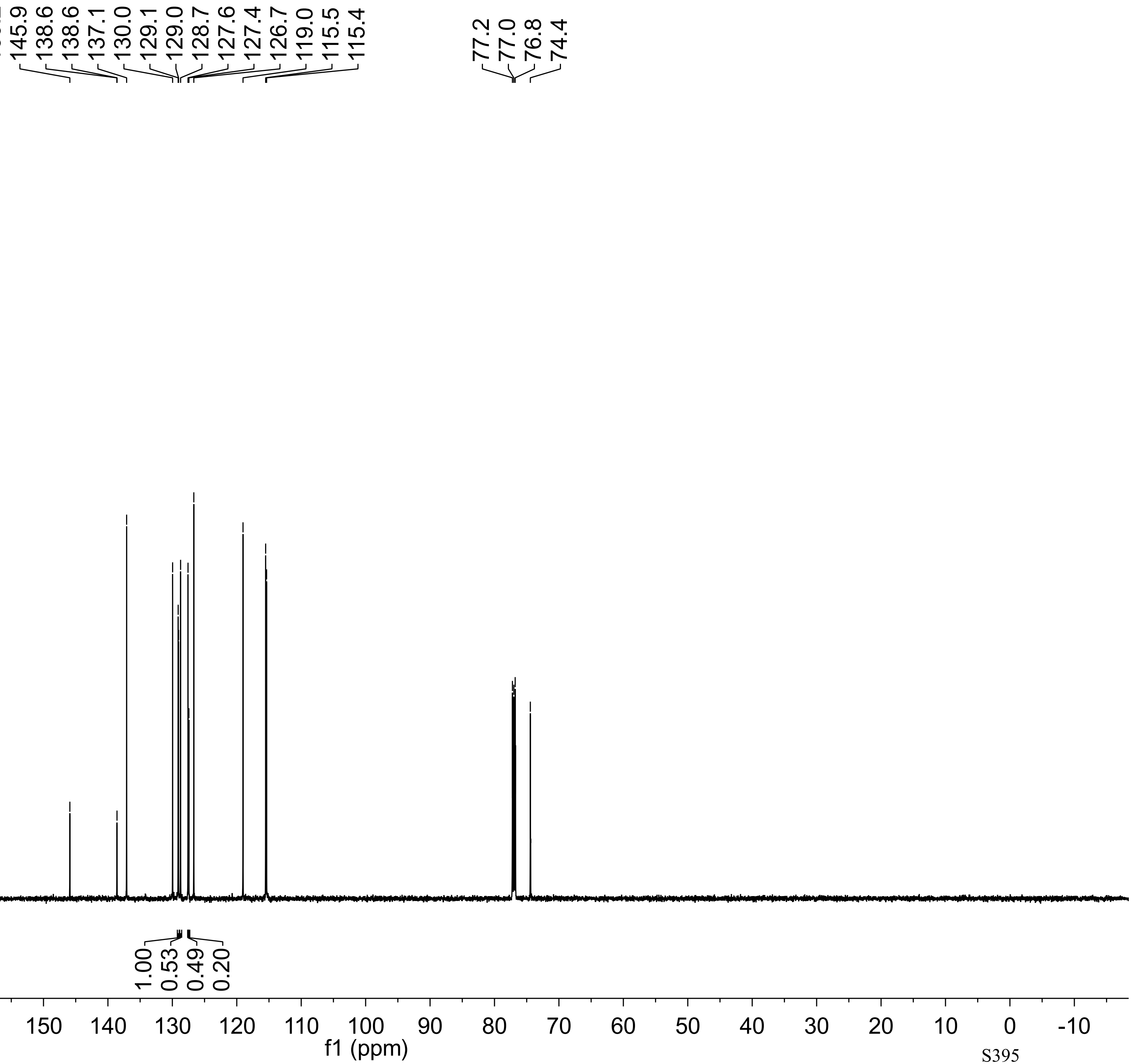

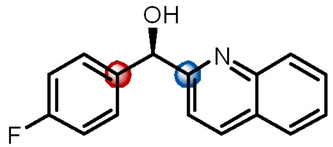

C11

Parameter	Value
1 Title	CFM-1106-2
2 Comment	
3 Origin	Bruker BioSpin GmbH
4 Owner	nmrsu
5 Site	
6 Spectrometer	Avance NEO 600
7 Author	
8 Solvent	CDCl3
9 Temperature	296.2
10 Pulse Sequence	zg
11 Experiment	1D
12 Number of Scans	16
13 Receiver Gain	101
14 Relaxation Delay	1.0000
15 Pulse Width	12.0000
16 Acquisition Time	0.4981
17 Acquisition Date	2019-11-07T00:44:07
18 Modification Date	2019-11-07T09:53:08
19 Spectrometer Frequency	564.71
20 Spectral Width	131579.0
21 Lowest Frequency	-122260.0
22 Nucleus	19F
23 Acquired Size	65536
24 Spectral Size	65536

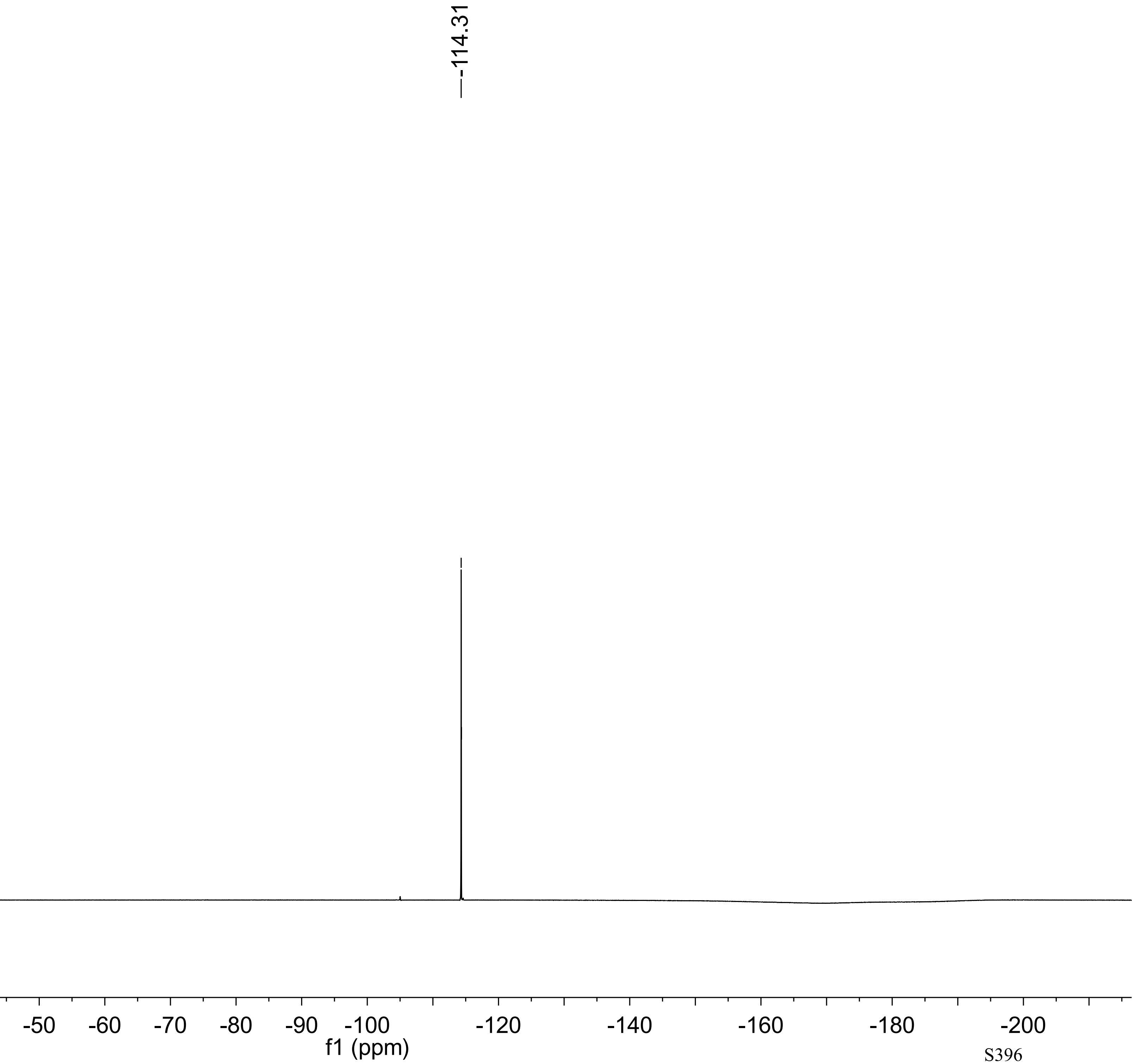

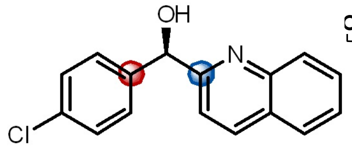

C12

Parameter	Value
1 Title	CFM-1106-3
2 Comment	
3 Origin	Bruker BioSpin GmbH
4 Owner	nmrsu
5 Site	
6 Spectrometer	Avance NEO 600
7 Author	
8 Solvent	CDCl3
9 Temperature	296.2
10 Pulse Sequence	zg30
11 Experiment	1D
12 Number of Scans	16
13 Receiver Gain	101
14 Relaxation Delay	1.0000
15 Pulse Width	10.0000
16 Acquisition Time	2.7525
17 Acquisition Date	2019-11-07T01:24:00
18 Modification Date	2019-11-07T09:53:07
19 Spectrometer Frequency	600.15
20 Spectral Width	11904.8
21 Lowest Frequency	-2260.7
22 Nucleus	1H
23 Acquired Size	32768
24 Spectral Size	65536

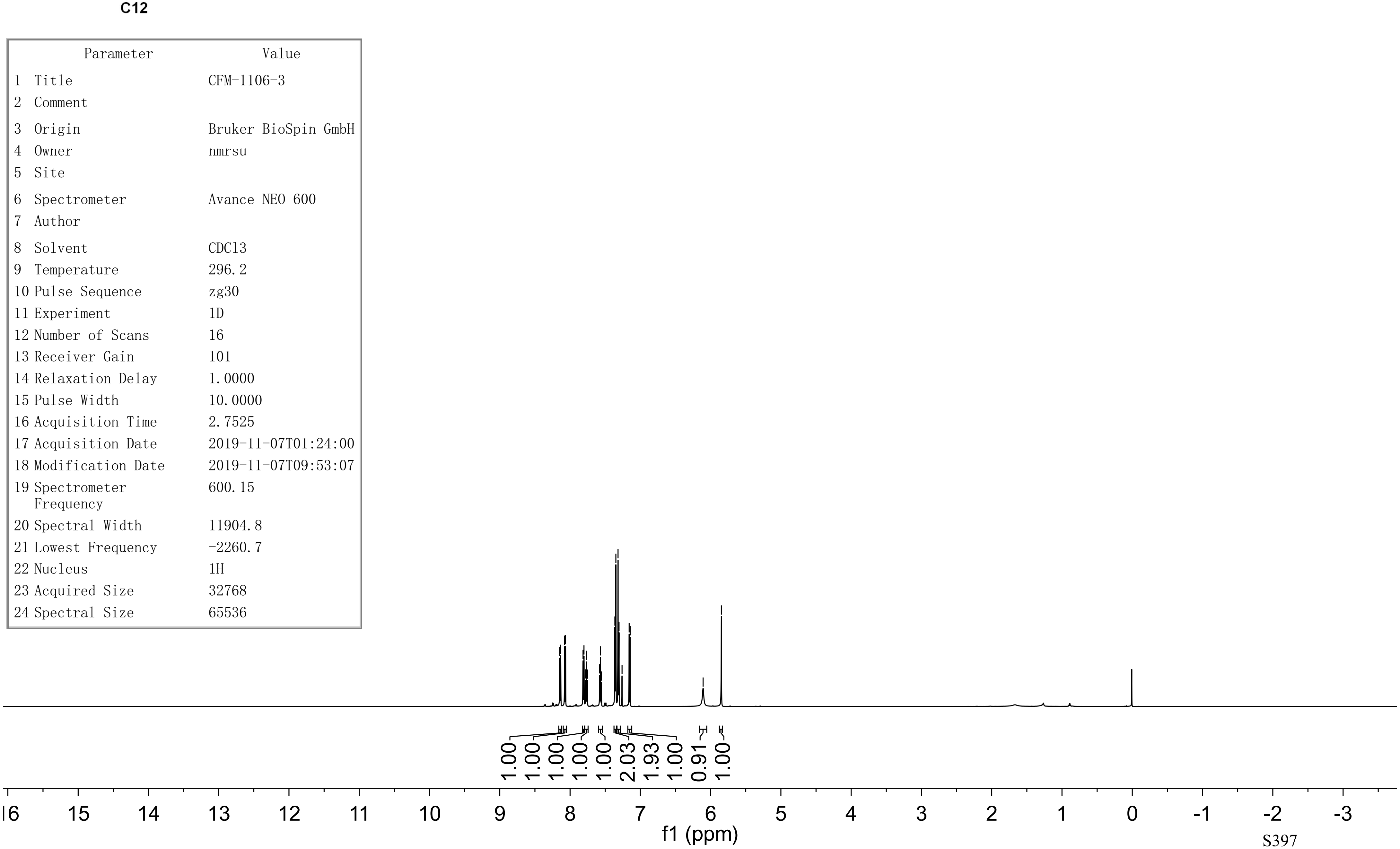

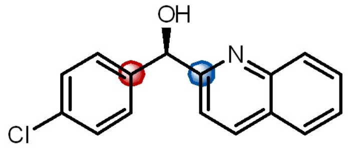

C12

Parameter	Value
1 Title	CFM-1106-3
2 Comment	
3 Origin	Bruker BioSpin GmbH
4 Owner	nmrsu
5 Site	
6 Spectrometer	Avance NEO 600
7 Author	
8 Solvent	CDCl3
9 Temperature	297.3
10 Pulse Sequence	zgpg30
11 Experiment	1D
12 Number of Scans	256
13 Receiver Gain	101
14 Relaxation Delay	2.0000
15 Pulse Width	12.0000
16 Acquisition Time	0.9175
17 Acquisition Date	2019-11-07T01:38:01
18 Modification Date	2019-11-07T09:53:07
19 Spectrometer Frequency	150.91
20 Spectral Width	35714.3
21 Lowest Frequency	-2774.5
22 Nucleus	13C
23 Acquired Size	32768
24 Spectral Size	32768

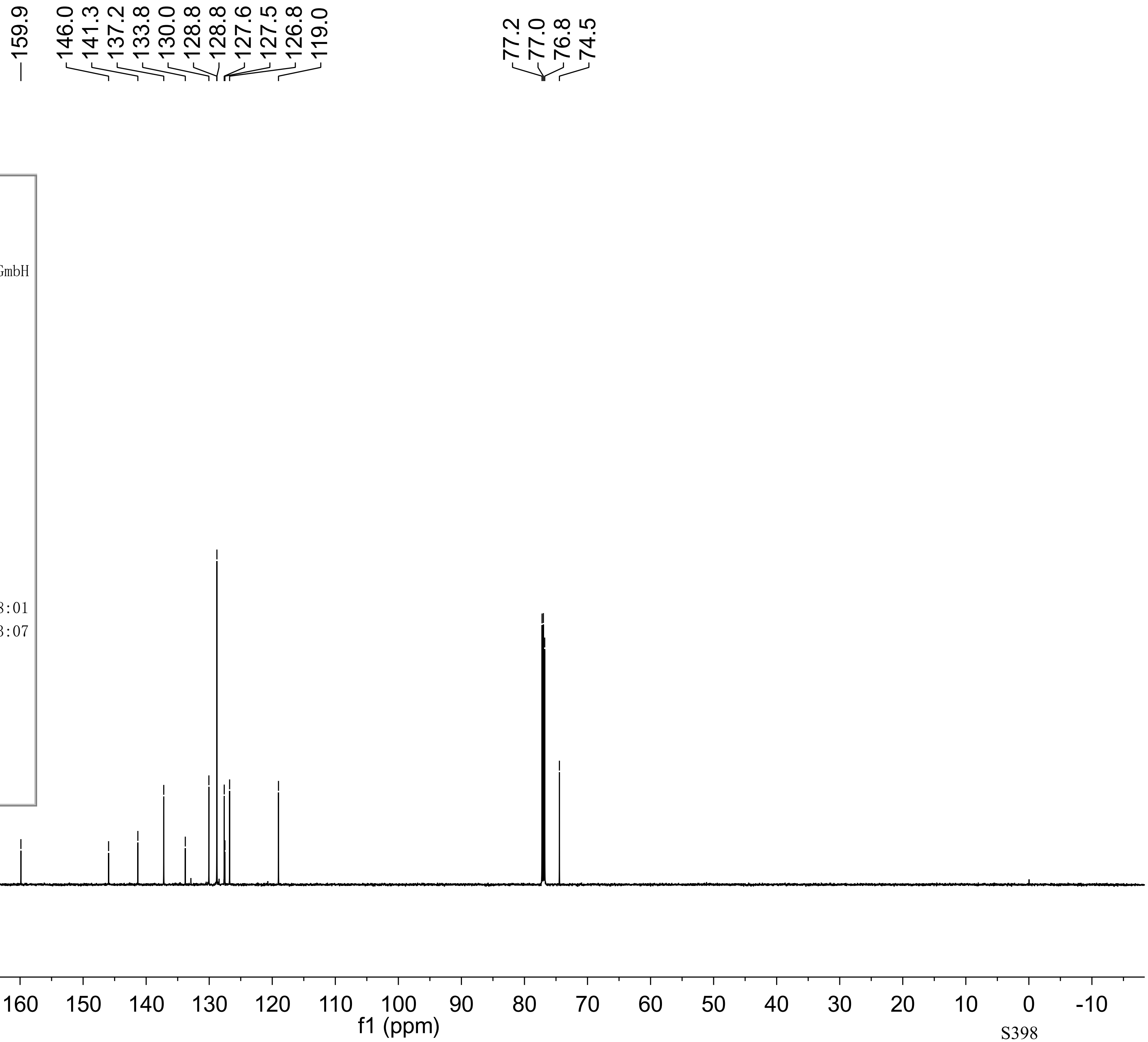

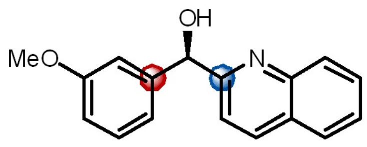

C13

Parameter	Value
1 Title	R-CFM-E7-1
2 Comment	
3 Origin	Bruker BioSpin GmbH
4 Owner	nmrsu
5 Site	
6 Spectrometer	Avance NEO 600
7 Author	
8 Solvent	CDCl3
9 Temperature	296.2
10 Pulse Sequence	zg30
11 Experiment	1D
12 Number of Scans	8
13 Receiver Gain	101
14 Relaxation Delay	1.0000
15 Pulse Width	10.0000
16 Acquisition Time	2.7525
17 Acquisition Date	2020-04-16T00:22:02
18 Modification Date	2020-04-16T10:12:03
19 Spectrometer Frequency	600.15
20 Spectral Width	11904.8
21 Lowest Frequency	-2311.8
22 Nucleus	1H
23 Acquired Size	32768
24 Spectral Size	65536

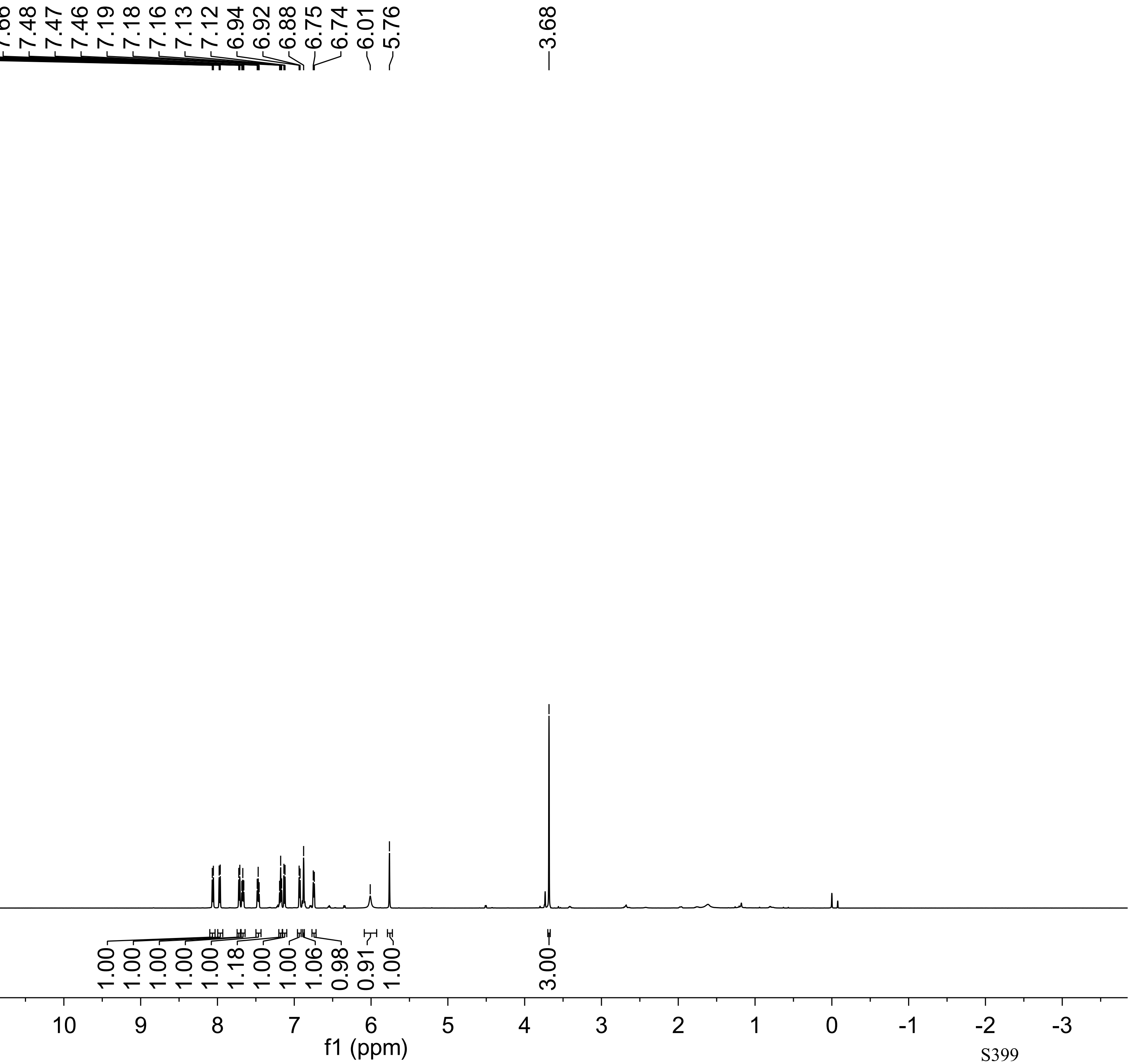

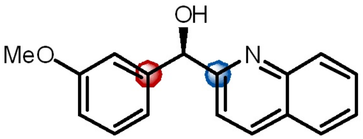

C13

Parameter	Value
1 Title	R-CFM-E7-1
2 Comment	
3 Origin	Bruker BioSpin GmbH
4 Owner	nmrsu
5 Site	
6 Spectrometer	Avance NEO 600
7 Author	
8 Solvent	CDCl3
9 Temperature	297.8
10 Pulse Sequence	zgpg30
11 Experiment	1D
12 Number of Scans	256
13 Receiver Gain	101
14 Relaxation Delay	2.0000
15 Pulse Width	12.0000
16 Acquisition Time	0.9175
17 Acquisition Date	2020-04-16T00:36:41
18 Modification Date	2020-04-16T10:12:03
19 Spectrometer Frequency	150.91
20 Spectral Width	35714.3
21 Lowest Frequency	-2775.4
22 Nucleus	13C
23 Acquired Size	32768
24 Spectral Size	32768

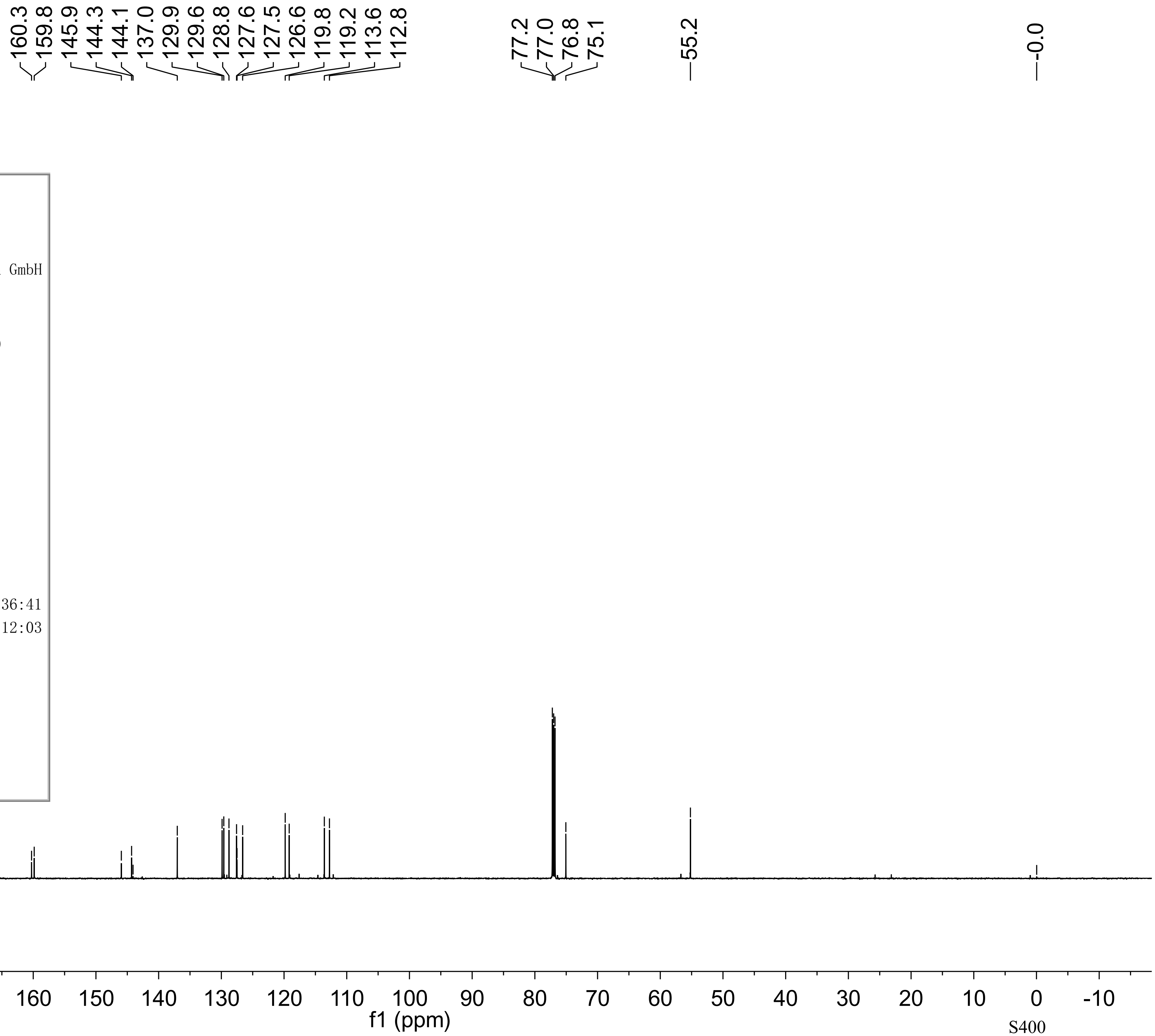

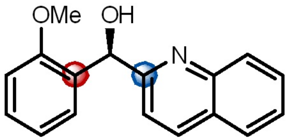

C14

Parameter	Value
1 Title	CFM-1106-5
2 Comment	
3 Origin	Bruker BioSpin GmbH
4 Owner	nmrsu
5 Site	
6 Spectrometer	Avance NEO 600
7 Author	
8 Solvent	CDCl3
9 Temperature	296.7
10 Pulse Sequence	zg30
11 Experiment	1D
12 Number of Scans	16
13 Receiver Gain	101
14 Relaxation Delay	1.0000
15 Pulse Width	10.0000
16 Acquisition Time	2.7525
17 Acquisition Date	2019-11-07T02:02:24
18 Modification Date	2019-11-07T09:53:09
19 Spectrometer Frequency	600.15
20 Spectral Width	11904.8
21 Lowest Frequency	-2261.4
22 Nucleus	1H
23 Acquired Size	32768
24 Spectral Size	65536

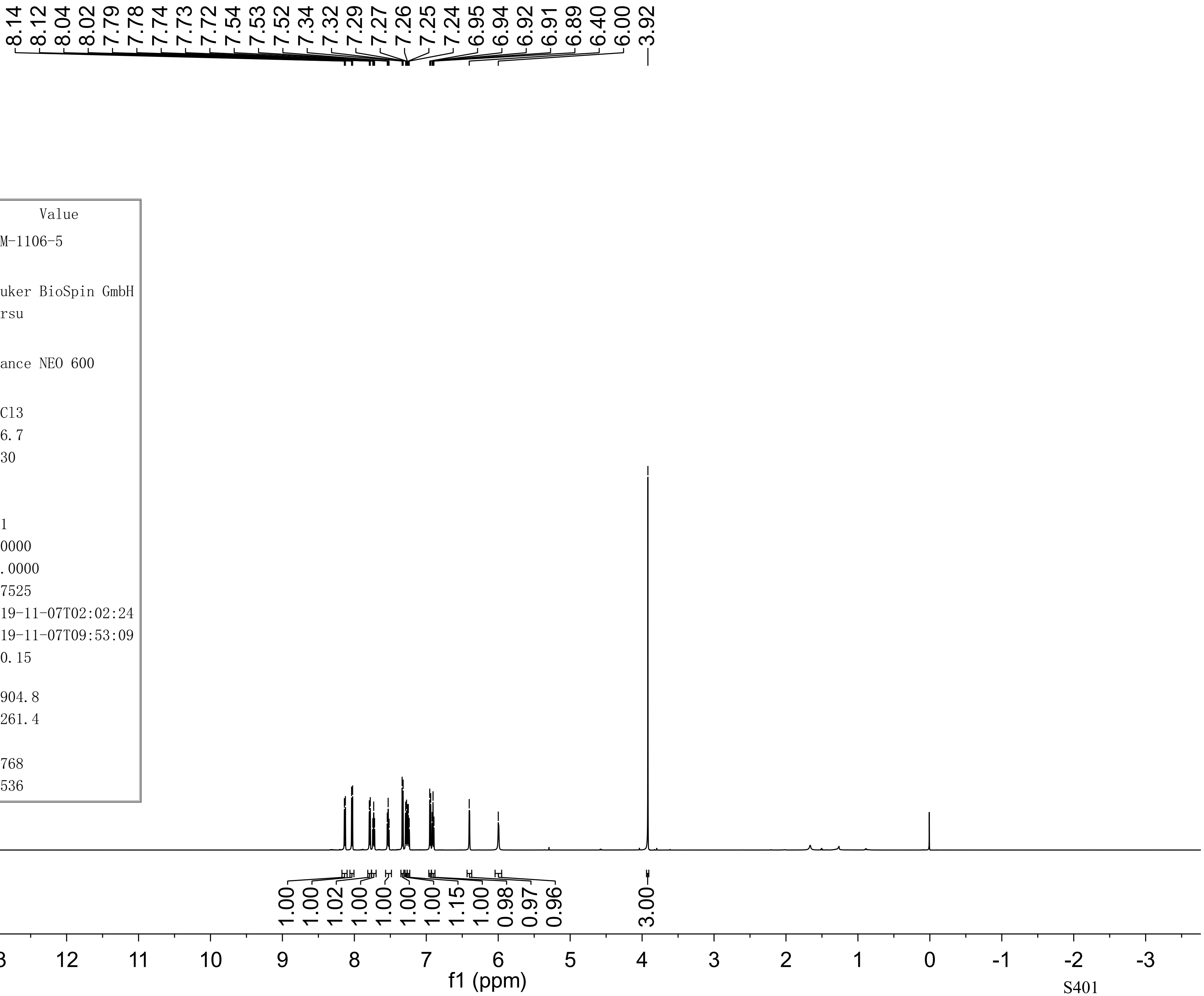

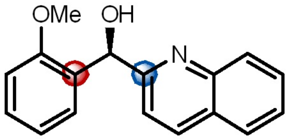

C14

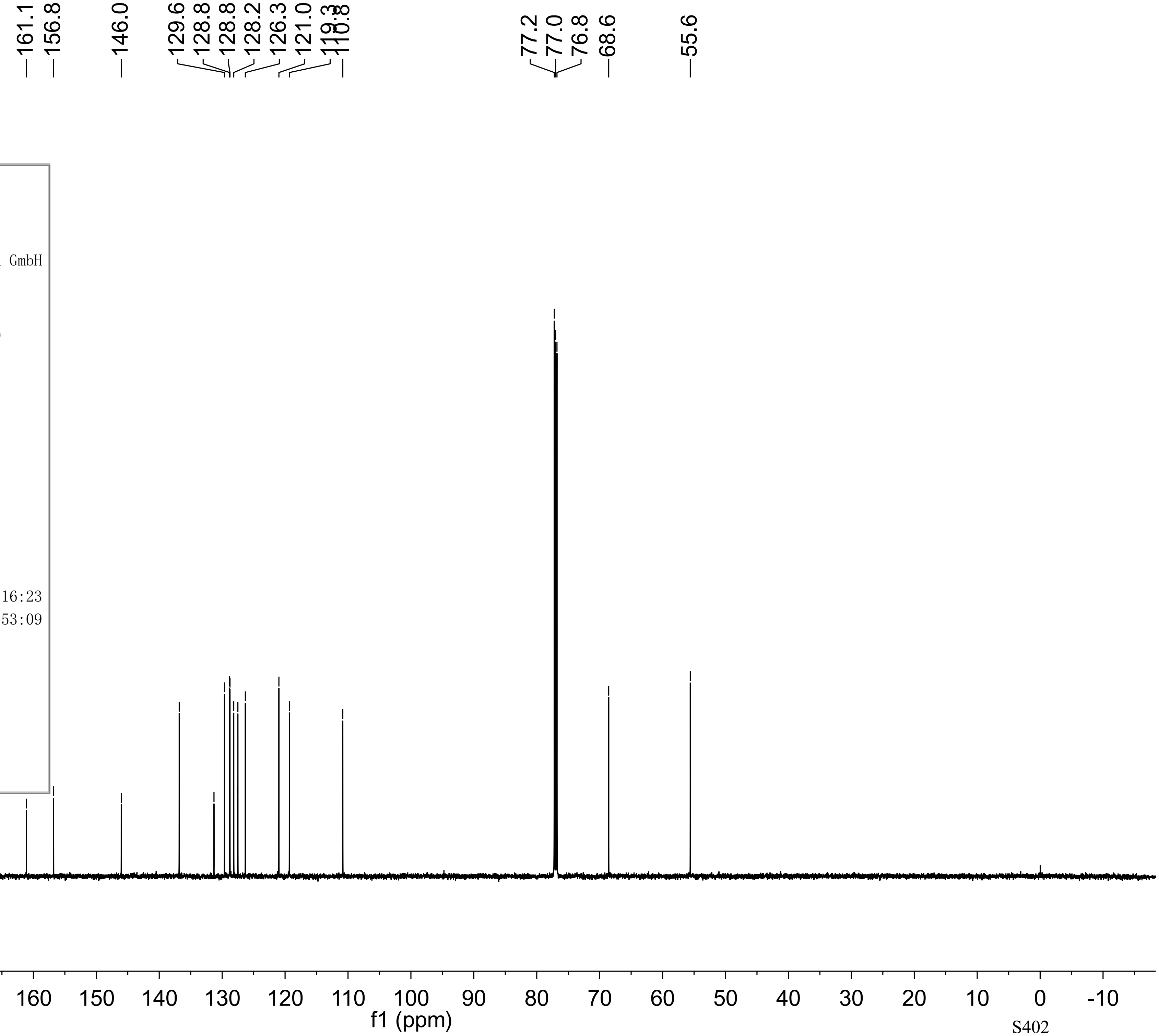

	Parameter	Value
1	Title	CFM-1106-5
2	Comment	
3	Origin	Bruker BioSpin GmbH
4	Owner	nmrsu
5	Site	
6	Spectrometer	Avance NEO 600
7	Author	
8	Solvent	CDCl3
9	Temperature	297.8
10	Pulse Sequence	zgpg30
11	Experiment	1D
12	Number of Scans	256
13	Receiver Gain	101
14	Relaxation Delay	2.0000
15	Pulse Width	12.0000
16	Acquisition Time	0.9175
17	Acquisition Date	2019-11-07T02:16:23
18	Modification Date	2019-11-07T09:53:09
19	Spectrometer Frequency	150.91
20	Spectral Width	35714.3
21	Lowest Frequency	-2774.3
22	Nucleus	13C
23	Acquired Size	32768
24	Spectral Size	32768

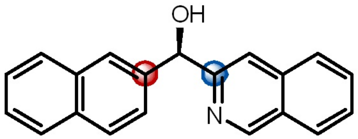

C15

	Parameter	Value
1	Title	R-CFM-A2
2	Comment	
3	Origin	Bruker BioSpin GmbH
4	Owner	nmrsu
5	Site	
6	Spectrometer	Avance NEO 600
7	Author	
8	Solvent	CDCl3
9	Temperature	297.0
10	Pulse Sequence	zg30
11	Experiment	1D
12	Number of Scans	16
13	Receiver Gain	101
14	Relaxation Delay	1.0000
15	Pulse Width	10.0000
16	Acquisition Time	2.7525
17	Acquisition Date	2019-12-14T00:00:21
18	Modification Date	2019-12-14T09:18:06
19	Spectrometer Frequency	600.15
20	Spectral Width	11904.8
21	Lowest Frequency	-2261.1
22	Nucleus	1H
23	Acquired Size	32768
24	Spectral Size	65536

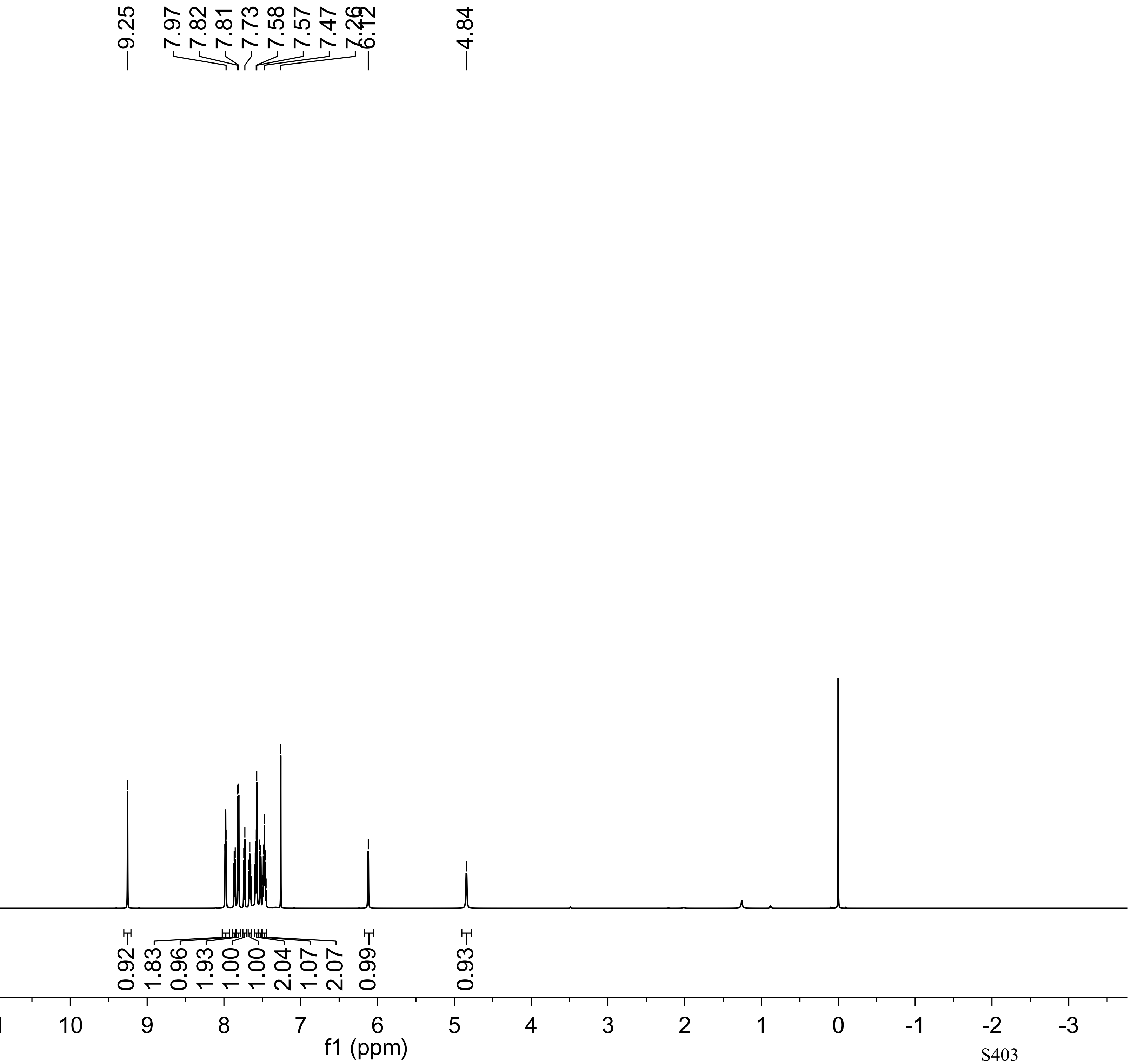

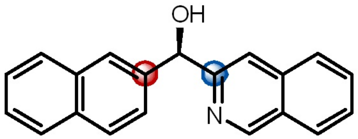

C15

Parameter	Value
1 Title	R-CFM-A2
2 Comment	
3 Origin	Bruker BioSpin GmbH
4 Owner	nmrsu
5 Site	
6 Spectrometer	Avance NEO 600
7 Author	
8 Solvent	CDCl3
9 Temperature	297.7
10 Pulse Sequence	zgpg30
11 Experiment	1D
12 Number of Scans	256
13 Receiver Gain	101
14 Relaxation Delay	2.0000
15 Pulse Width	12.0000
16 Acquisition Time	0.9175
17 Acquisition Date	2019-12-14T00:14:35
18 Modification Date	2019-12-14T09:18:07
19 Spectrometer Frequency	150.91
20 Spectral Width	35714.3
21 Lowest Frequency	-2770.6
22 Nucleus	13C
23 Acquired Size	32768
24 Spectral Size	32768

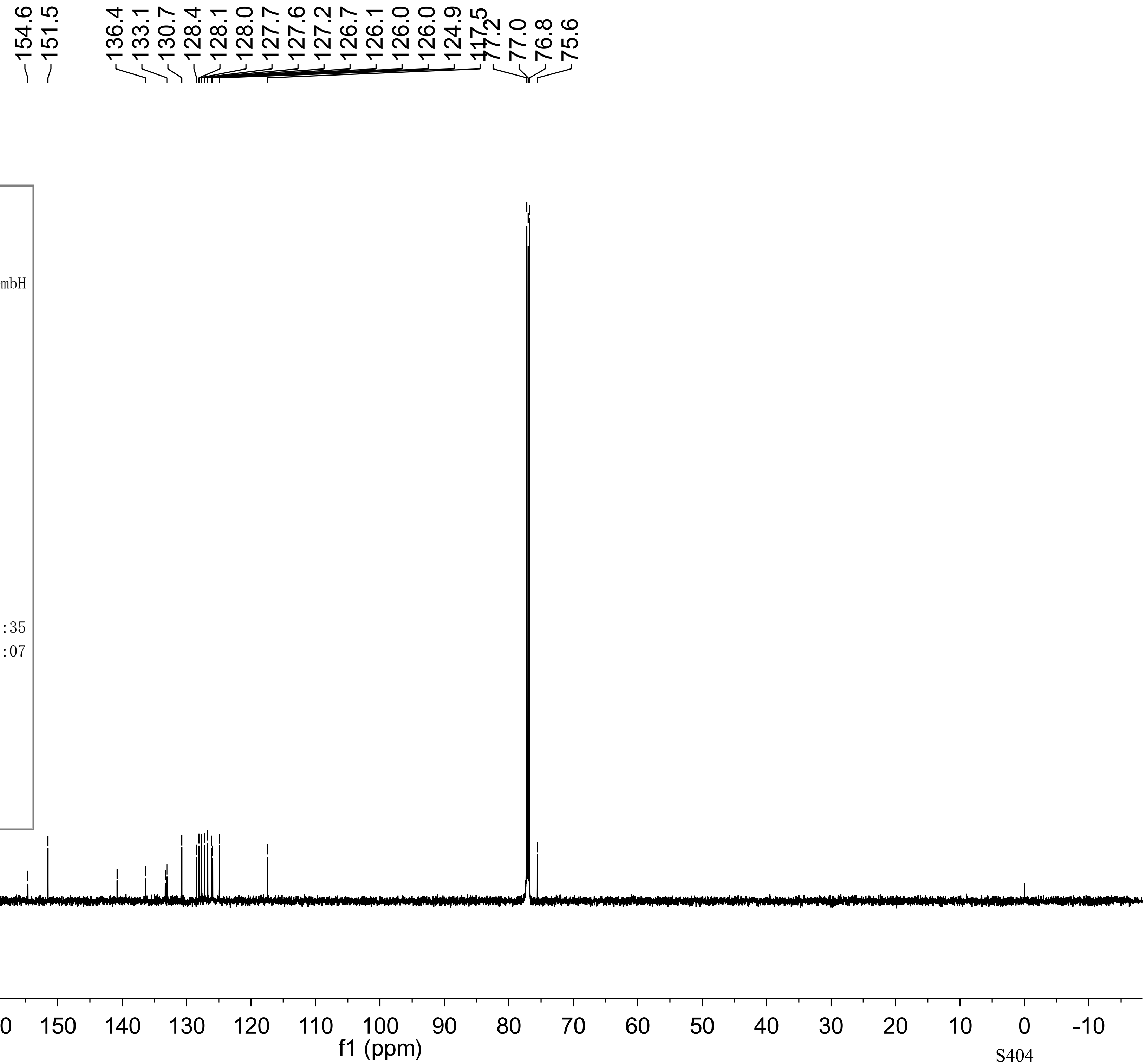

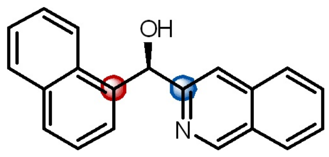

C16

	Parameter	Value
1	Title	CFM-R-A3
2	Comment	
3	Origin	Bruker BioSpin GmbH
4	Owner	nmrsu
5	Site	
6	Spectrometer	Avance NEO 600
7	Author	
8	Solvent	CDCl3
9	Temperature	297.8
10	Pulse Sequence	zg30
11	Experiment	1D
12	Number of Scans	8
13	Receiver Gain	101
14	Relaxation Delay	1.0000
15	Pulse Width	10.0000
16	Acquisition Time	2.7525
17	Acquisition Date	2020-08-05T22:53:04
18	Modification Date	2020-08-06T09:01:57
19	Spectrometer Frequency	600.15
20	Spectral Width	11904.8
21	Lowest Frequency	-2261.4
22	Nucleus	1H
23	Acquired Size	32768
24	Spectral Size	65536

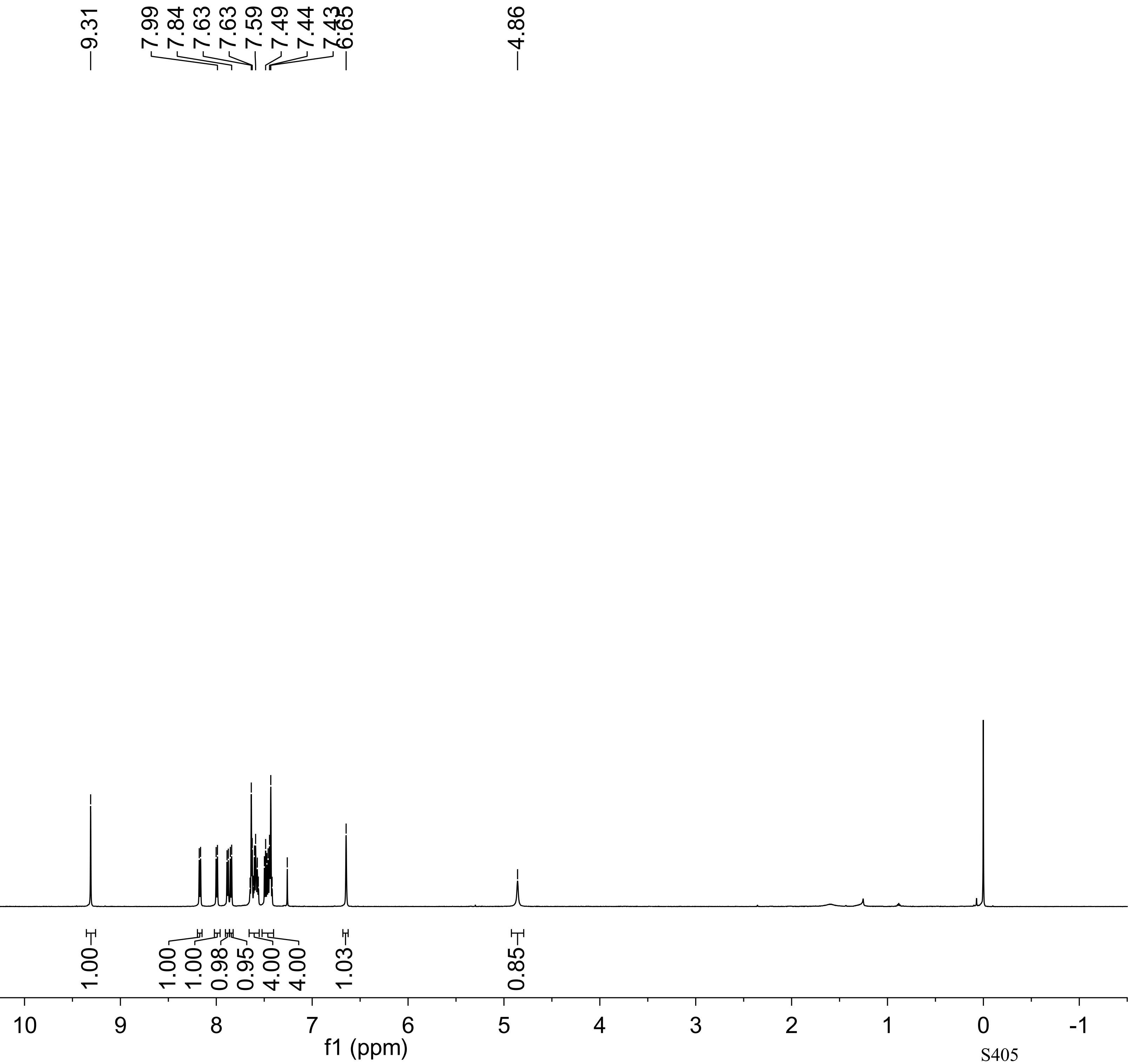

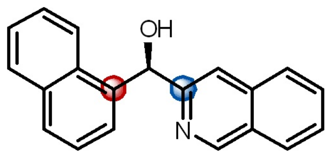

C16

	Parameter	Value
1	Title	R-CFM-A3
2	Comment	
3	Origin	Bruker BioSpin GmbH
4	Owner	nmrsu
5	Site	
6	Spectrometer	Avance NEO 600
7	Author	
8	Solvent	CDCl3
9	Temperature	297.8
10	Pulse Sequence	zgpg30
11	Experiment	1D
12	Number of Scans	256
13	Receiver Gain	101
14	Relaxation Delay	2.0000
15	Pulse Width	12.0000
16	Acquisition Time	0.9175
17	Acquisition Date	2019-12-14T00:34:57
18	Modification Date	2019-12-14T09:18:09
19	Spectrometer Frequency	150.91
20	Spectral Width	35714.3
21	Lowest Frequency	-2774.5
22	Nucleus	13C
23	Acquired Size	32768
24	Spectral Size	32768

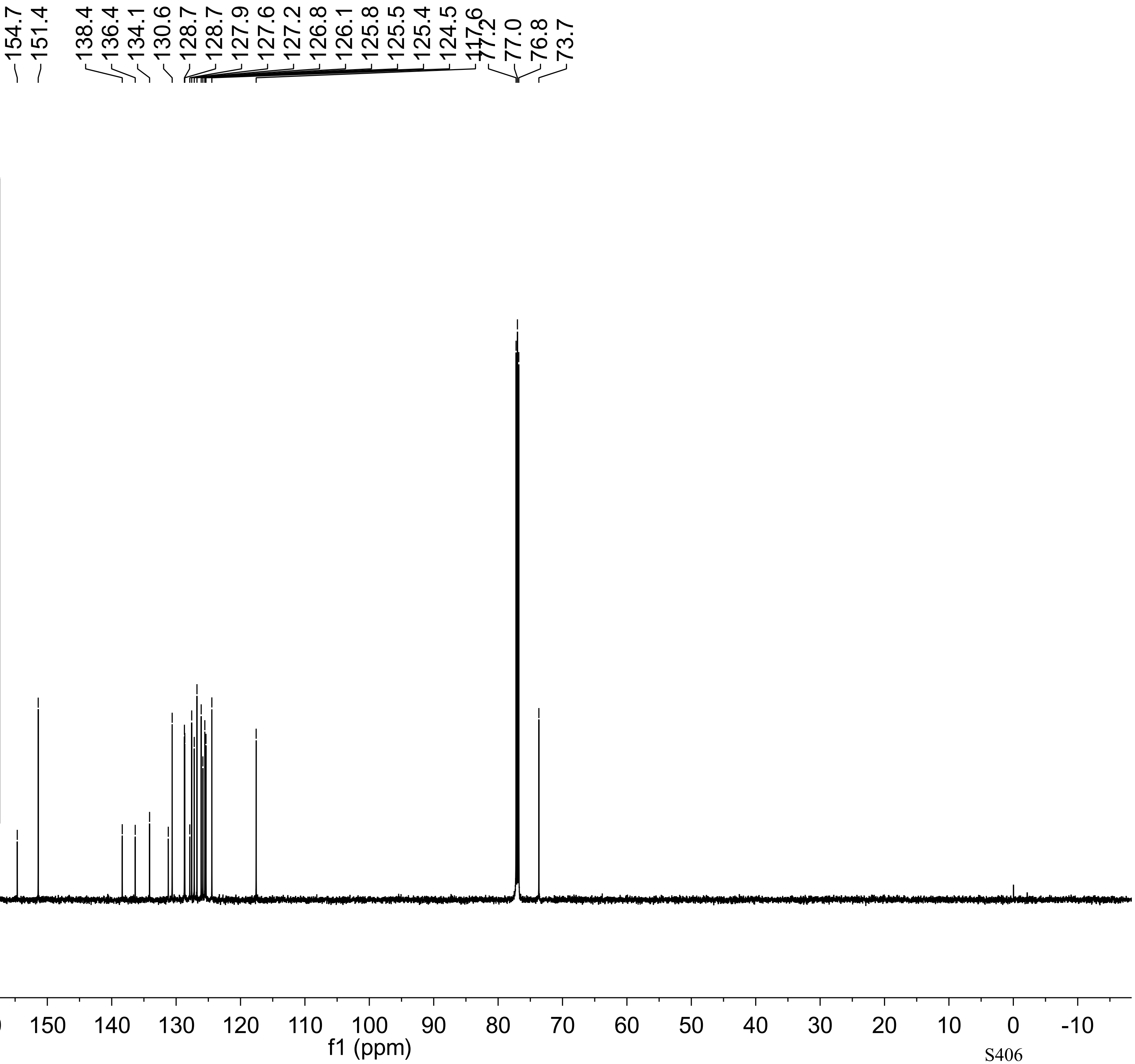

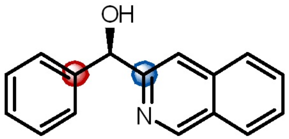

C17

Parameter	Value
1 Title	R-CFM-A1
2 Comment	
3 Origin	Bruker BioSpin GmbH
4 Owner	nmrsu
5 Site	
6 Spectrometer	Avance NEO 600
7 Author	
8 Solvent	CDCl3
9 Temperature	296.6
10 Pulse Sequence	zg30
11 Experiment	1D
12 Number of Scans	16
13 Receiver Gain	101
14 Relaxation Delay	1.0000
15 Pulse Width	10.0000
16 Acquisition Time	2.7525
17 Acquisition Date	2019-12-13T23:41:00
18 Modification Date	2019-12-14T09:18:07
19 Spectrometer Frequency	600.15
20 Spectral Width	11904.8
21 Lowest Frequency	-2260.9
22 Nucleus	1H
23 Acquired Size	32768
24 Spectral Size	65536

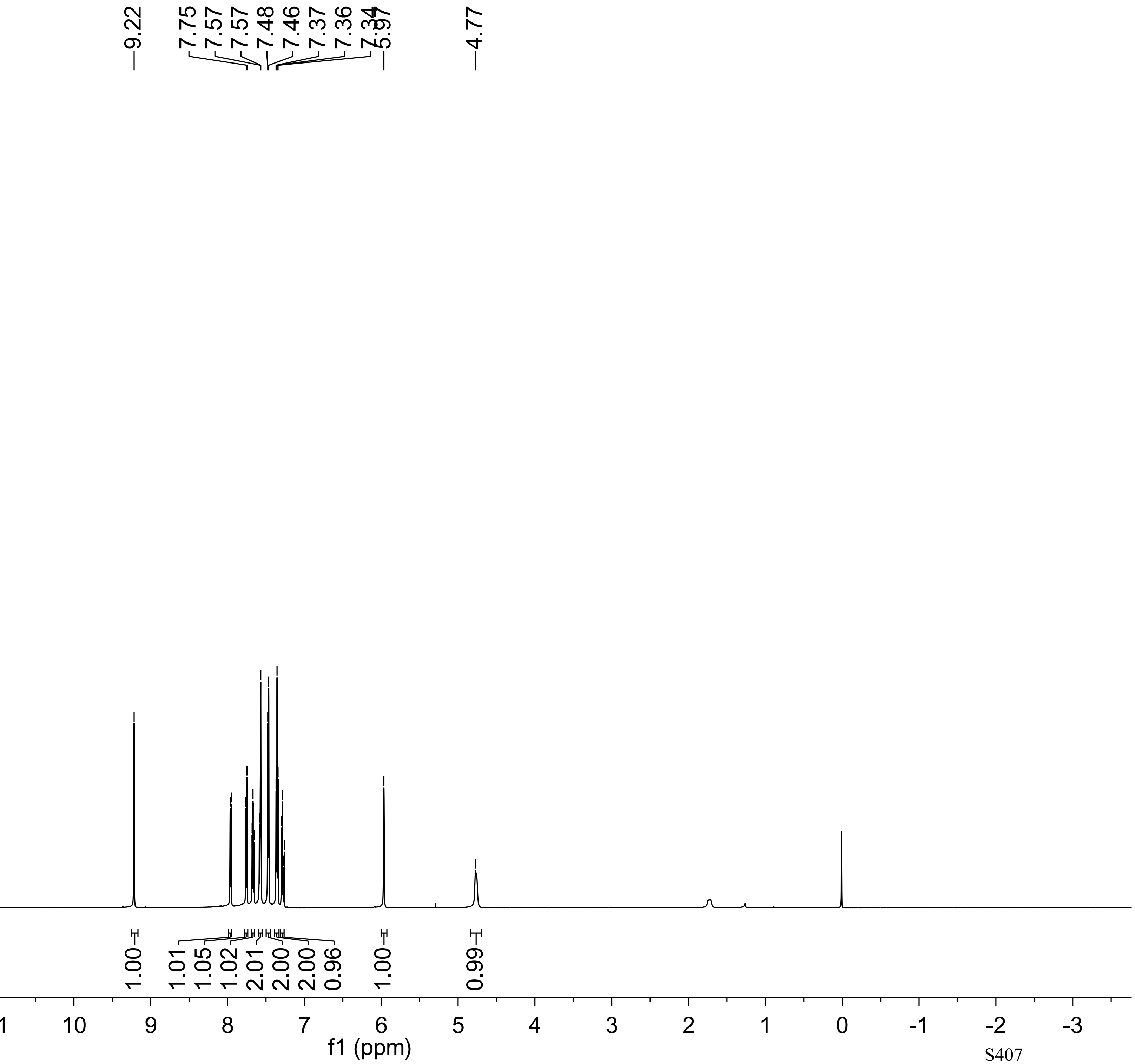

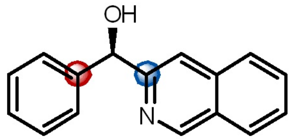

C17

Parameter	Value
1 Title	R-CFM-A1
2 Comment	
3 Origin	Bruker BioSpin GmbH
4 Owner	nmrsu
5 Site	
6 Spectrometer	Avance NEO 600
7 Author	
8 Solvent	CDCl3
9 Temperature	298.0
10 Pulse Sequence	zgpg30
11 Experiment	1D
12 Number of Scans	256
13 Receiver Gain	101
14 Relaxation Delay	2.0000
15 Pulse Width	12.0000
16 Acquisition Time	0.9175
17 Acquisition Date	2019-12-13T23:55:07
18 Modification Date	2019-12-14T09:18:07
19 Spectrometer Frequency	150.91
20 Spectral Width	35714.3
21 Lowest Frequency	-2774.8
22 Nucleus	13C
23 Acquired Size	32768
24 Spectral Size	32768

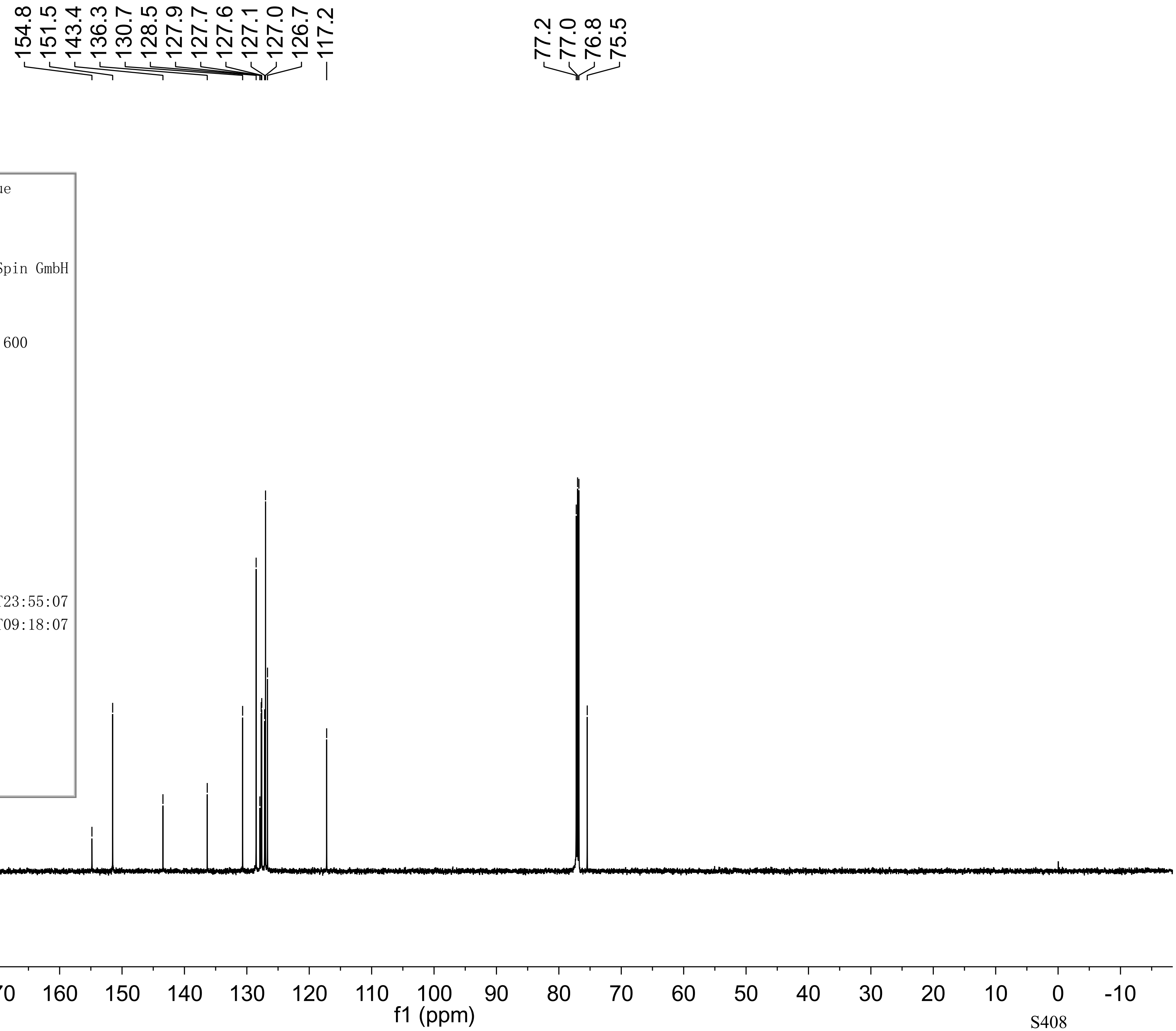

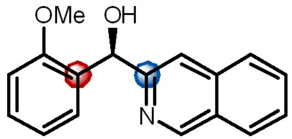

C18

	Parameter	Value
1	Title	CFM-R-A4-2
2	Comment	
3	Origin	Bruker BioSpin GmbH
4	Owner	nmrsu
5	Site	
6	Spectrometer	Avance NEO 600
7	Author	
8	Solvent	CDCl3
9	Temperature	297.7
10	Pulse Sequence	zg30
11	Experiment	1D
12	Number of Scans	8
13	Receiver Gain	101
14	Relaxation Delay	1.0000
15	Pulse Width	10.0000
16	Acquisition Time	2.7525
17	Acquisition Date	2020-08-04T13:32:25
18	Modification Date	2020-08-04T14:23:35
19	Spectrometer Frequency	600.15
20	Spectral Width	11904.8
21	Lowest Frequency	-2309.1
22	Nucleus	1H
23	Acquired Size	32768
24	Spectral Size	65536

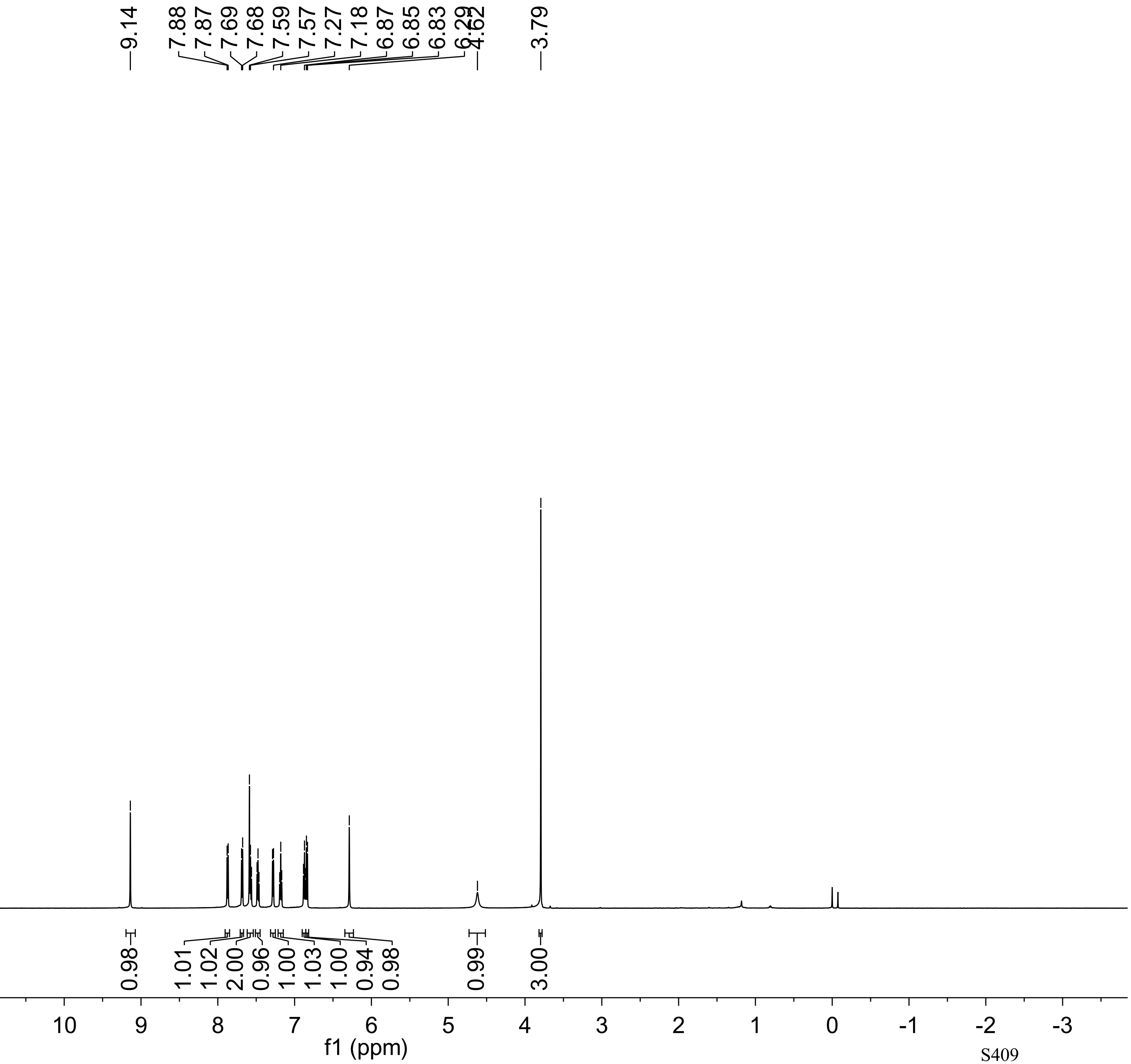

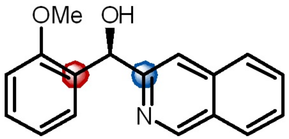

C18

	Parameter	Value
1	Title	R-CFM-A4
2	Comment	
3	Origin	Bruker BioSpin GmbH
4	Owner	nmrsu
5	Site	
6	Spectrometer	Avance NEO 600
7	Author	
8	Solvent	CDCl3
9	Temperature	298.1
10	Pulse Sequence	zgpg30
11	Experiment	1D
12	Number of Scans	256
13	Receiver Gain	101
14	Relaxation Delay	2.0000
15	Pulse Width	12.0000
16	Acquisition Time	0.9175
17	Acquisition Date	2019-12-14T00:55:13
18	Modification Date	2019-12-14T09:18:08
19	Spectrometer Frequency	150.91
20	Spectral Width	35714.3
21	Lowest Frequency	-2781.4
22	Nucleus	13C
23	Acquired Size	32768
24	Spectral Size	32768

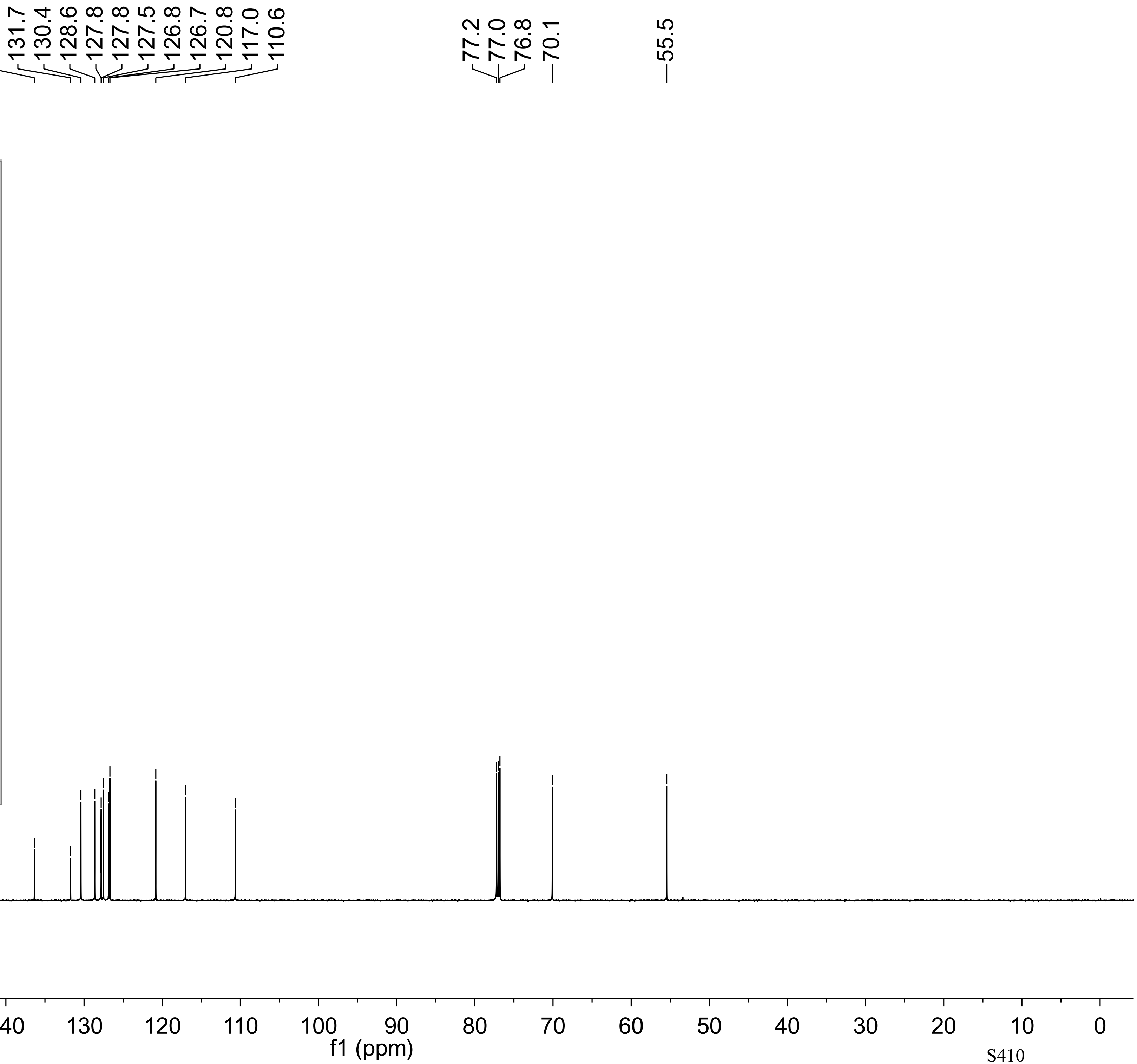

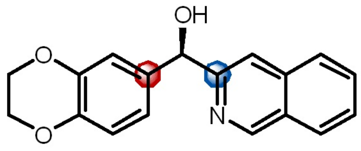

C19

	Parameter	Value
1	Title	CFM-R-A5-2
2	Comment	
3	Origin	Bruker BioSpin GmbH
4	Owner	nmrsu
5	Site	
6	Spectrometer	Avance NEO 600
7	Author	
8	Solvent	CDCl3
9	Temperature	298.1
10	Pulse Sequence	zg30
11	Experiment	1D
12	Number of Scans	8
13	Receiver Gain	101
14	Relaxation Delay	1.0000
15	Pulse Width	10.0000
16	Acquisition Time	2.7525
17	Acquisition Date	2020-08-04T14:45:16
18	Modification Date	2020-08-04T14:51:28
19	Spectrometer Frequency	600.15
20	Spectral Width	11904.8
21	Lowest Frequency	-2260.8
22	Nucleus	1H
23	Acquired Size	32768
24	Spectral Size	65536

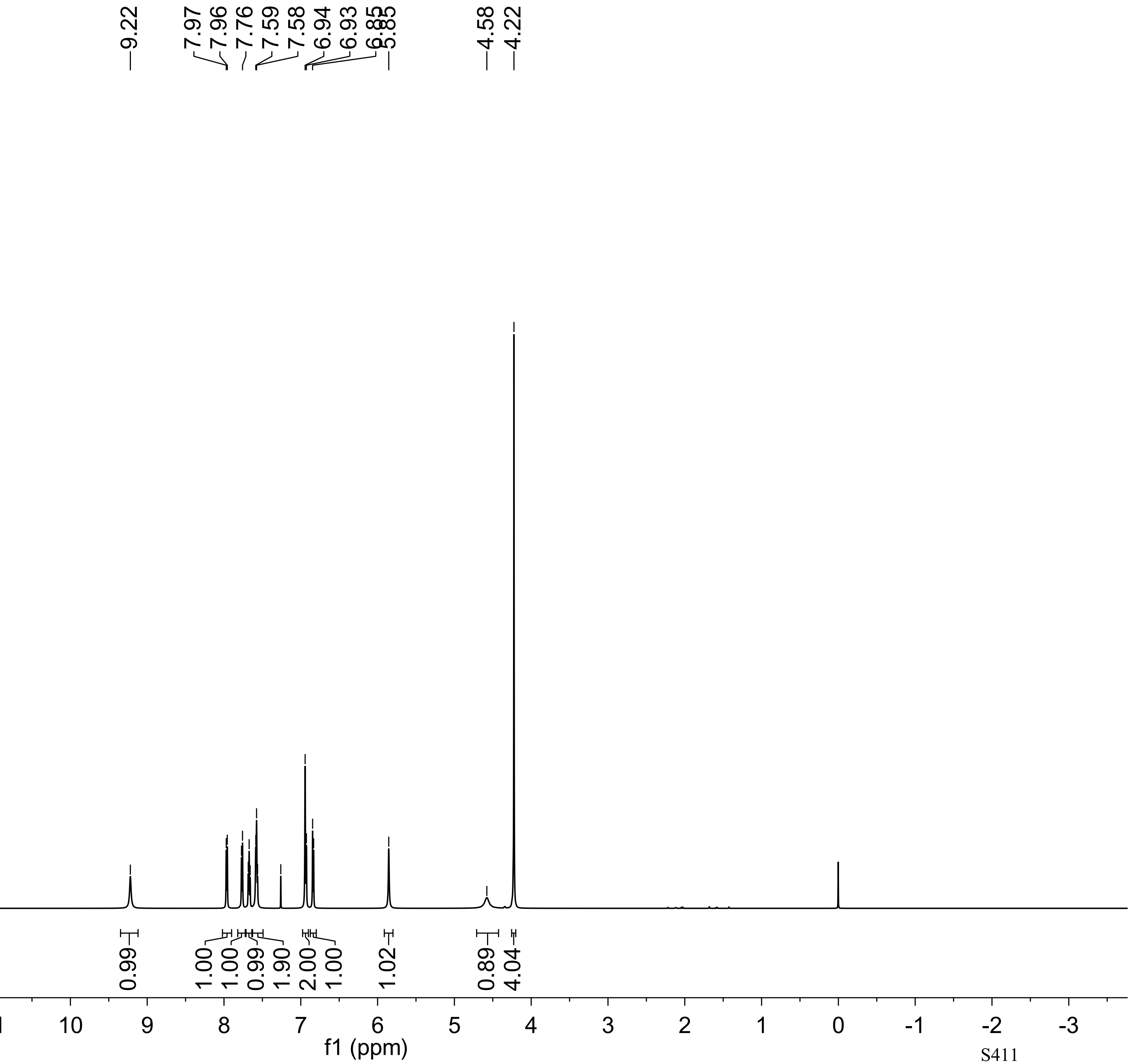

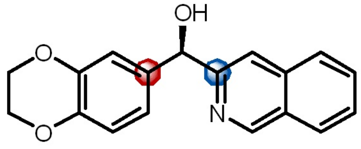

C19

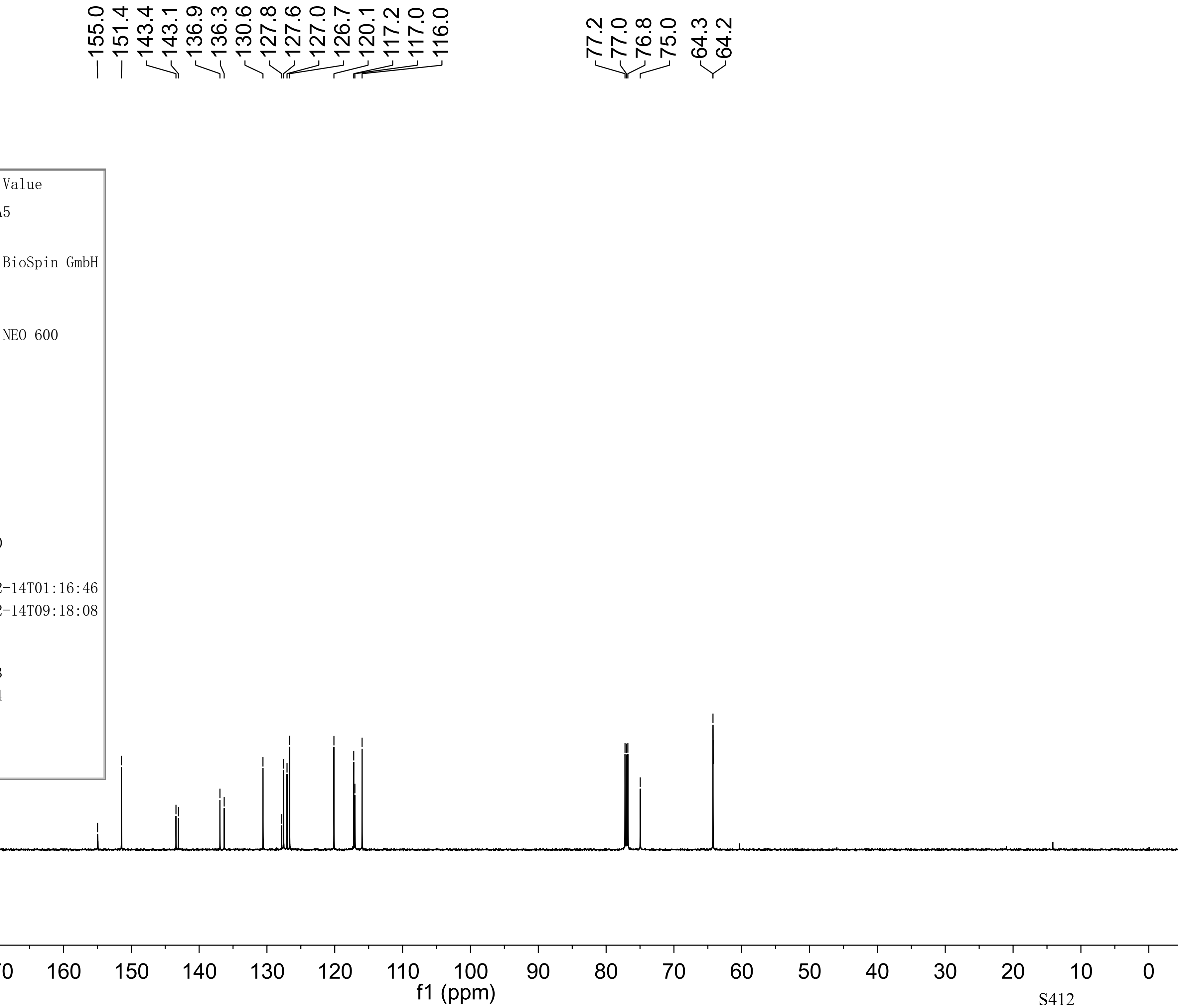

	Parameter	Value
1	Title	R-CFM-A5
2	Comment	
3	Origin	Bruker BioSpin GmbH
4	Owner	nmrsu
5	Site	
6	Spectrometer	Avance NEO 600
7	Author	
8	Solvent	CDCl3
9	Temperature	298.2
10	Pulse Sequence	zgpg30
11	Experiment	1D
12	Number of Scans	256
13	Receiver Gain	101
14	Relaxation Delay	2.0000
15	Pulse Width	12.0000
16	Acquisition Time	0.9175
17	Acquisition Date	2019-12-14T01:16:46
18	Modification Date	2019-12-14T09:18:08
19	Spectrometer Frequency	150.91
20	Spectral Width	35714.3
21	Lowest Frequency	-2780.4
22	Nucleus	13C
23	Acquired Size	32768
24	Spectral Size	32768

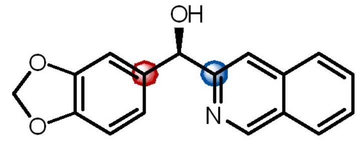

C20

	Parameter	Value
1	Title	R-CFM-A6
2	Comment	
3	Origin	Bruker BioSpin GmbH
4	Owner	nmrsu
5	Site	
6	Spectrometer	Avance NEO 600
7	Author	
8	Solvent	CDCl3
9	Temperature	296.8
10	Pulse Sequence	zg30
11	Experiment	1D
12	Number of Scans	16
13	Receiver Gain	101
14	Relaxation Delay	1.0000
15	Pulse Width	10.0000
16	Acquisition Time	2.7525
17	Acquisition Date	2019-12-14T01:21:57
18	Modification Date	2019-12-14T09:18:09
19	Spectrometer Frequency	600.15
20	Spectral Width	11904.8
21	Lowest Frequency	-2261.1
22	Nucleus	1H
23	Acquired Size	32768
24	Spectral Size	65536

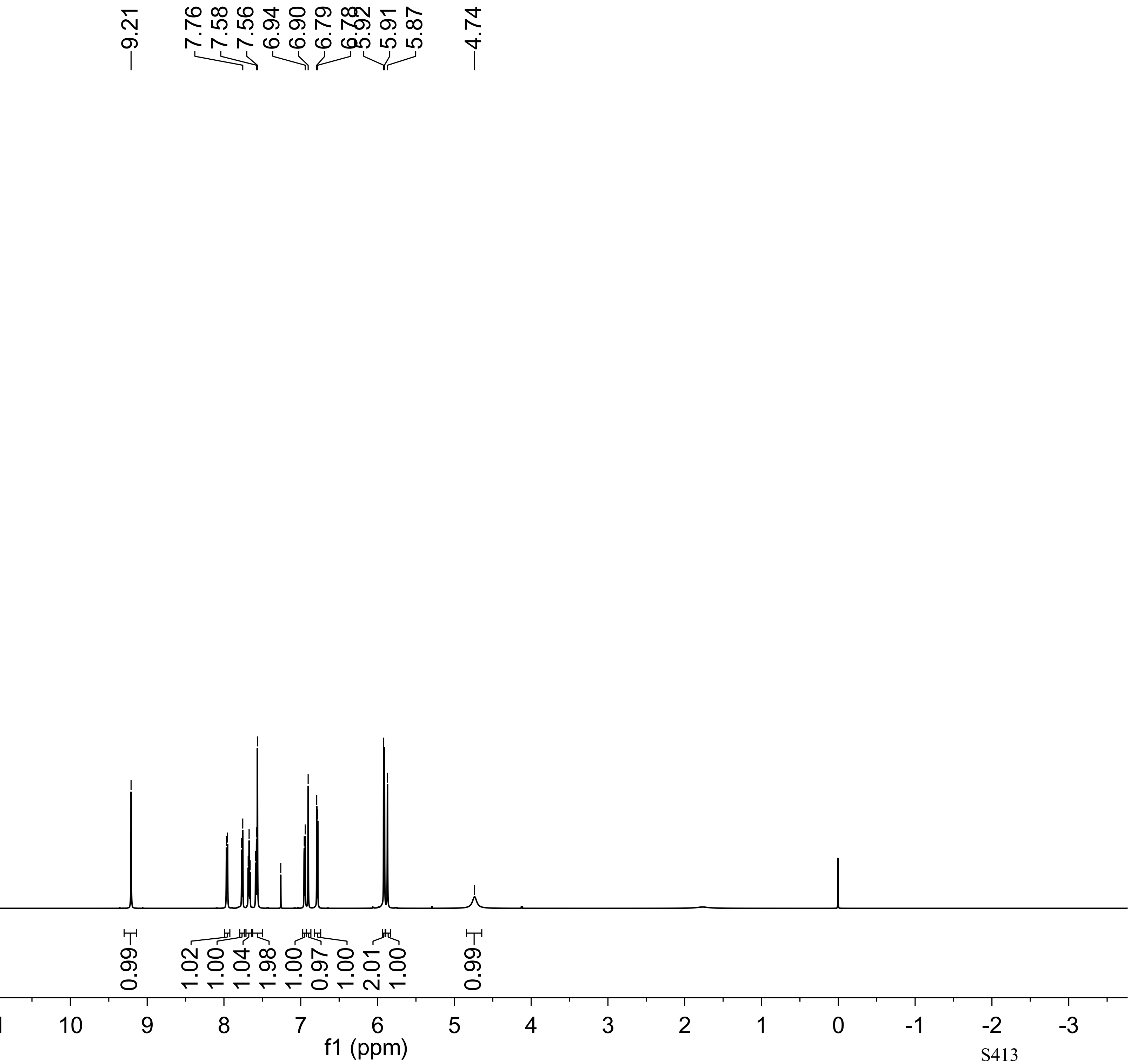

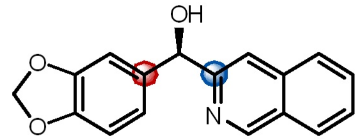

C20

	Parameter	Value
1	Title	R-CFM-A6-C
2	Comment	
3	Origin	Bruker BioSpin GmbH
4	Owner	nmrsu
5	Site	
6	Spectrometer	Avance NEO 600
7	Author	
8	Solvent	CDCl3
9	Temperature	298.0
10	Pulse Sequence	zgpg30
11	Experiment	1D
12	Number of Scans	128
13	Receiver Gain	101
14	Relaxation Delay	2.0000
15	Pulse Width	12.0000
16	Acquisition Time	0.9175
17	Acquisition Date	2019-12-14T16:26:20
18	Modification Date	2019-12-25T09:13:34
19	Spectrometer Frequency	150.91
20	Spectral Width	35714.3
21	Lowest Frequency	-2775.7
22	Nucleus	13C
23	Acquired Size	32768
24	Spectral Size	32768

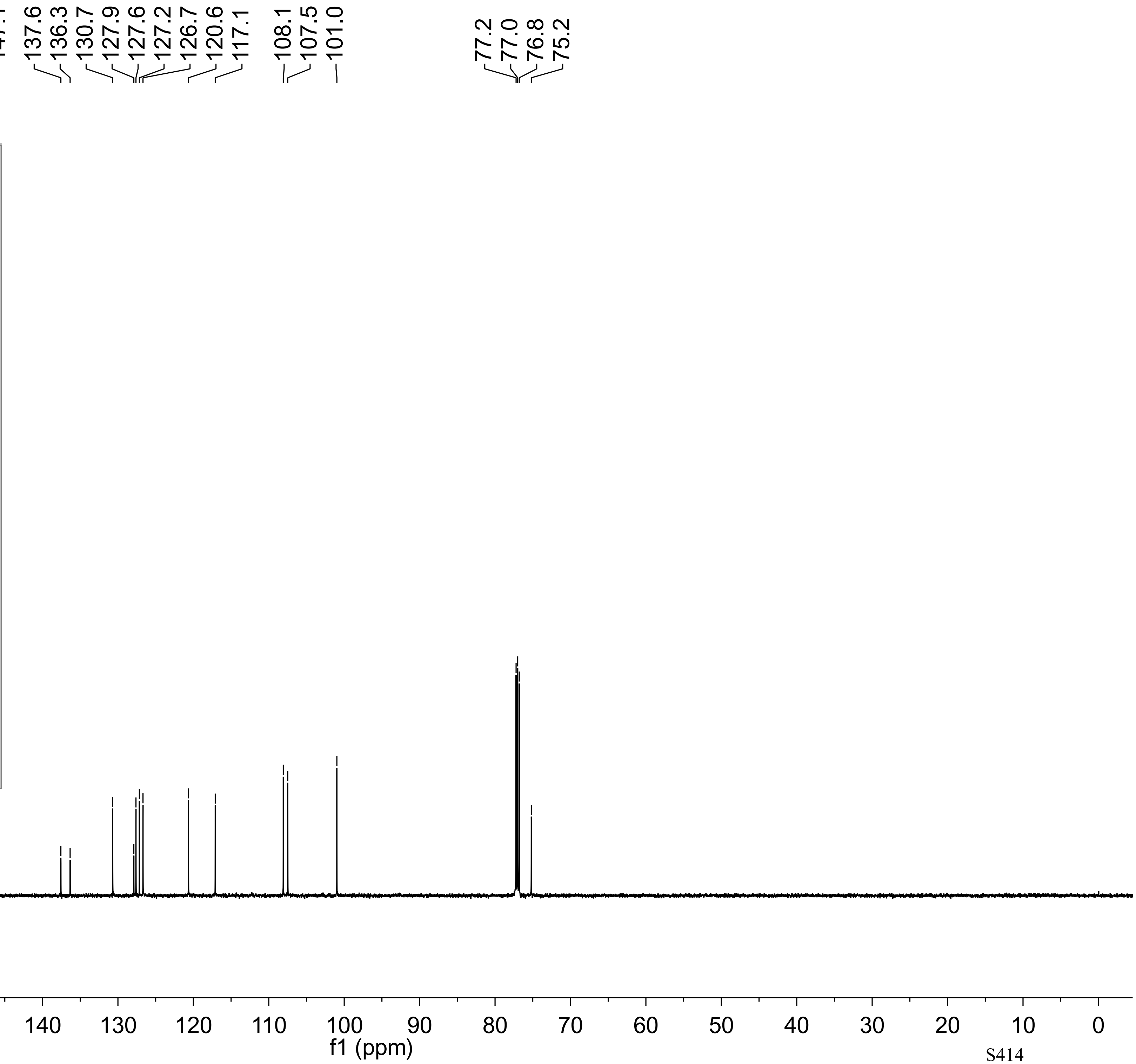

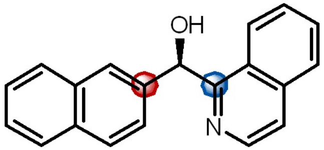

C21

Parameter	Value
1 Title	R-CFM-F2
2 Comment	
3 Origin	Bruker BioSpin GmbH
4 Owner	nmrsu
5 Site	
6 Spectrometer	Avance NEO 600
7 Author	
8 Solvent	CDCl3
9 Temperature	296.8
10 Pulse Sequence	zg30
11 Experiment	1D
12 Number of Scans	16
13 Receiver Gain	101
14 Relaxation Delay	1.0000
15 Pulse Width	10.0000
16 Acquisition Time	2.7525
17 Acquisition Date	2019-12-14T05:25:14
18 Modification Date	2019-12-14T09:18:14
19 Spectrometer Frequency	600.15
20 Spectral Width	11904.8
21 Lowest Frequency	-2261.3
22 Nucleus	1H
23 Acquired Size	32768
24 Spectral Size	65536

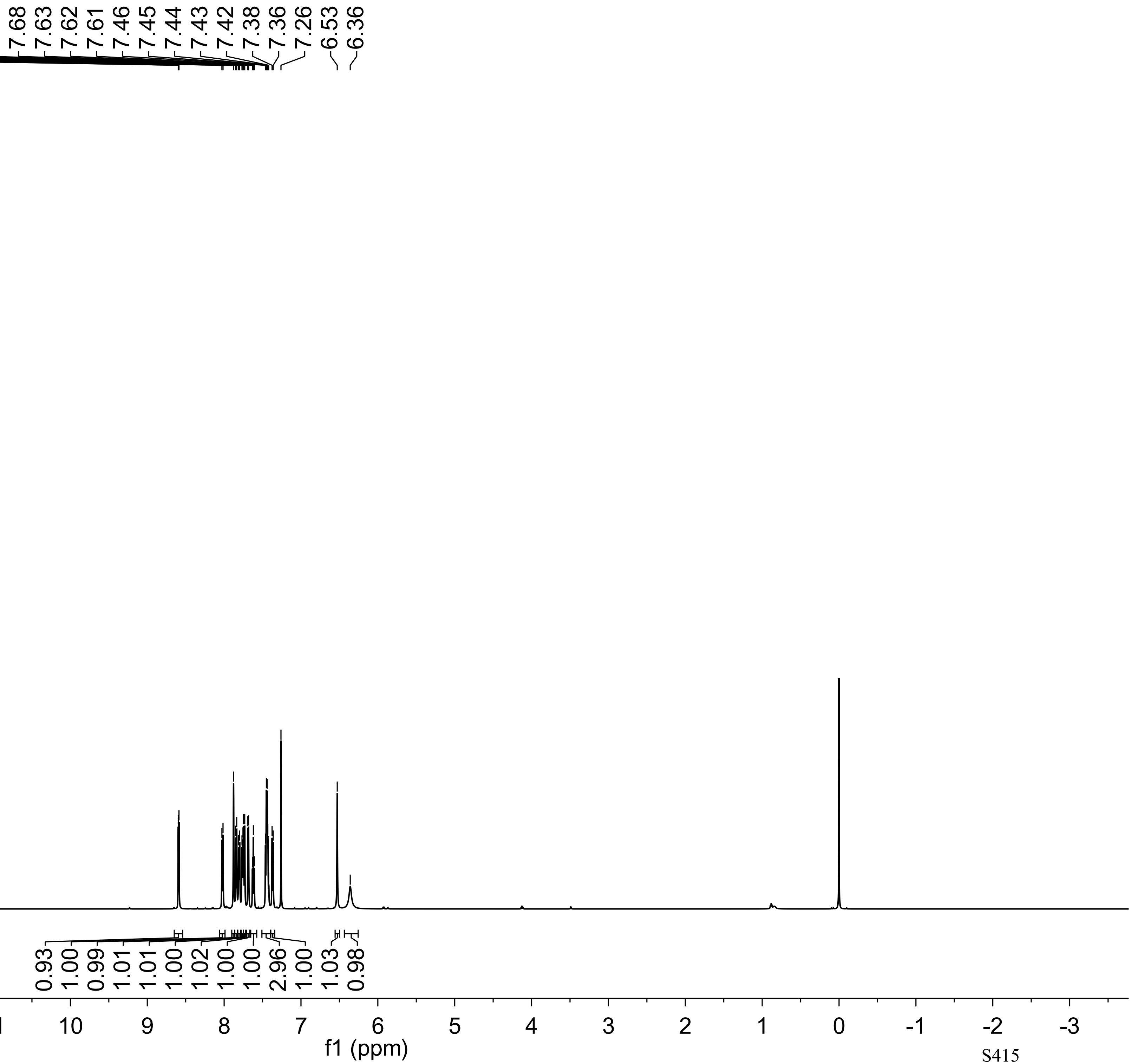

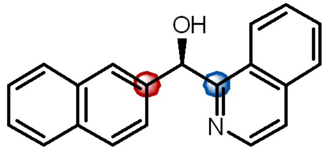

C21

Parameter		Value
1	Title	R-CFM-F2-2
2	Comment	
3	Origin	Bruker BioSpin GmbH
4	Owner	nmrsu
5	Site	
6	Spectrometer	Avance NEO 600
7	Author	
8	Solvent	CDCl3
9	Temperature	302.9
10	Pulse Sequence	zgpg30
11	Experiment	1D
12	Number of Scans	512
13	Receiver Gain	101
14	Relaxation Delay	2.0000
15	Pulse Width	12.0000
16	Acquisition Time	0.9175
17	Acquisition Date	2019-12-30T07:07:09
18	Modification Date	2019-12-30T08:57:48
19	Spectrometer Frequency	150.91
20	Spectral Width	35714.3
21	Lowest Frequency	-2771.9
22	Nucleus	13C
23	Acquired Size	32768
24	Spectral Size	32768

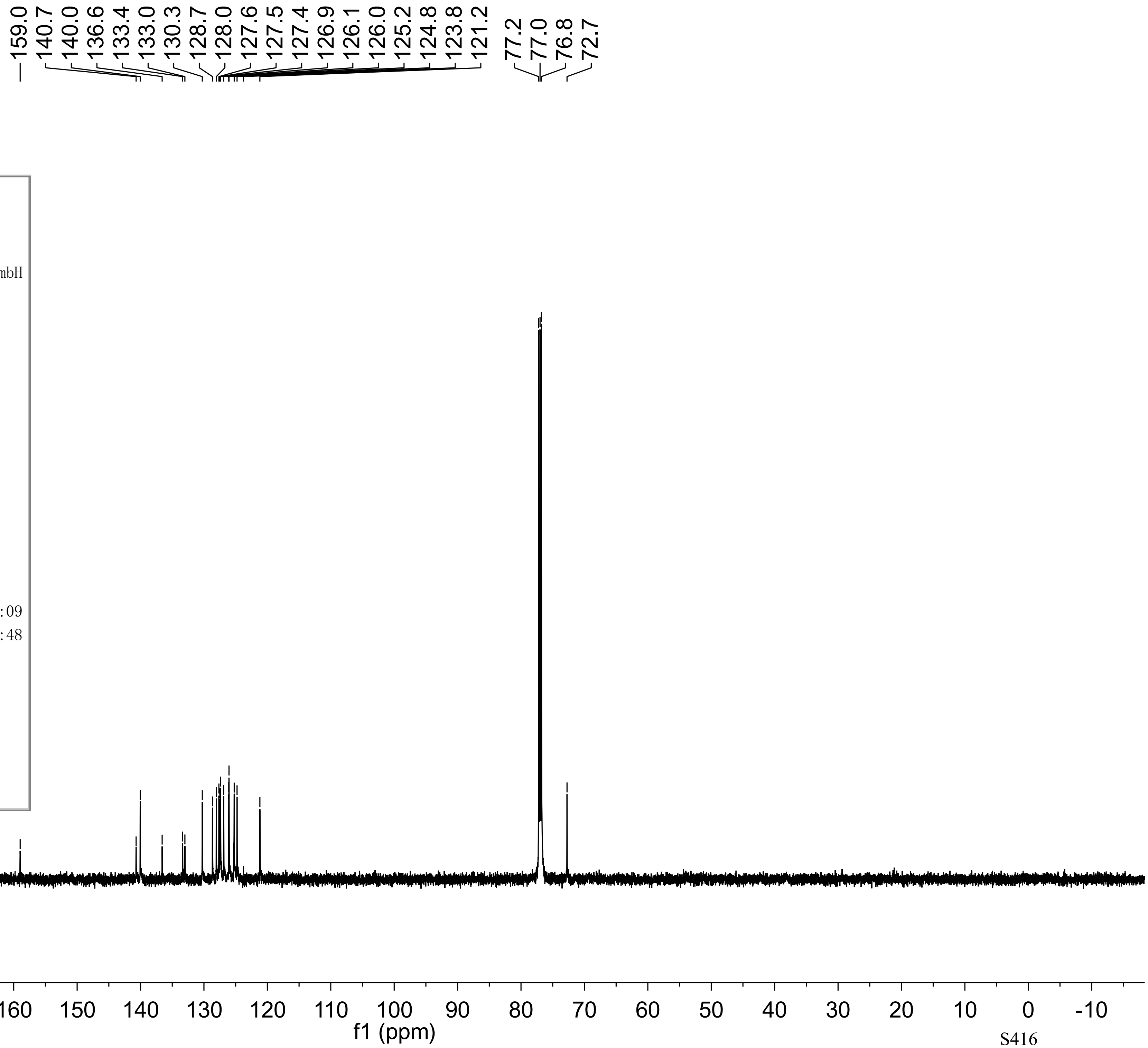

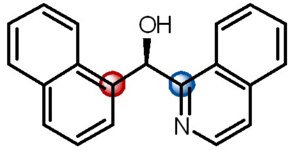

C22

Parameter		Value
1	Title	CFM-6-C22-0701
2	Comment	
3	Origin	Bruker BioSpin GmbH
4	Owner	nmrsu
5	Site	
6	Spectrometer	AVANCE NEO 400 MHZ DIGITAL NMR SPECTROMETER
7	Author	
8	Solvent	CDCl3
9	Temperature	298.2
10	Pulse Sequence	zg30
11	Experiment	1D
12	Number of Scans	8
13	Receiver Gain	101
14	Spectrometer Frequency	400.13
15	Spectral Width	8196.7
16	Lowest Frequency	-1643.6
17	Nucleus	1H
18	Acquired Size	32768
19	Spectral Size	65536

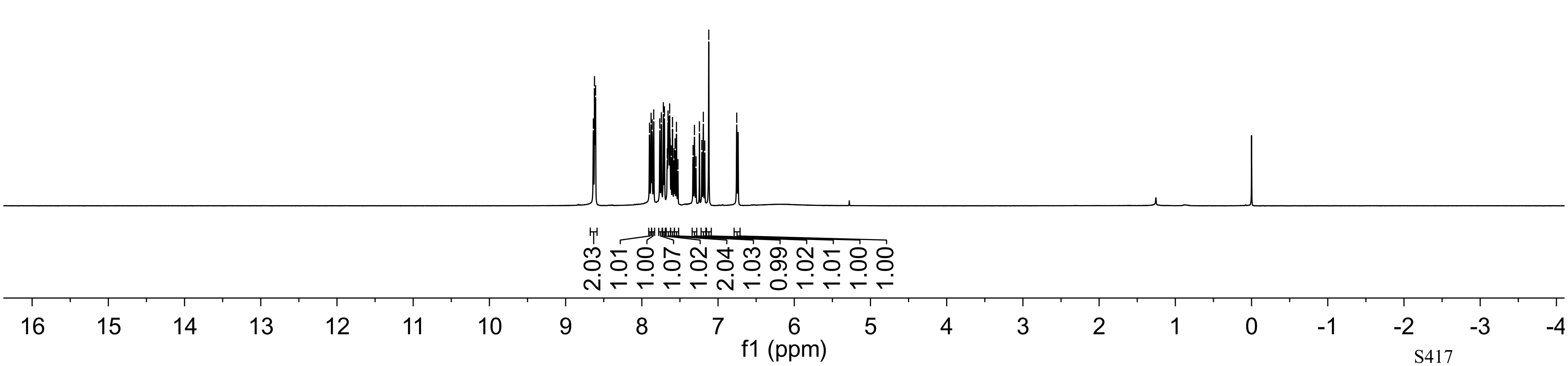

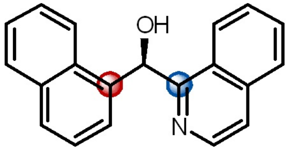

C22

Parameter	Value
1 Title	CFM-6-C22-0701
2 Comment	
3 Origin	Bruker BioSpin GmbH
4 Owner	nmrsu
5 Site	
6 Spectrometer	AVANCE NEO 400 MHZ DIGITAL NMR SPECTROMETER
7 Author	
8 Solvent	CDCl3
9 Temperature	298.2
10 Pulse Sequence	zgpg30
11 Experiment	1D
12 Number of Scans	86
13 Receiver Gain	59
14 Spectrometer Frequency	100.61
15 Spectral Width	23809.5
16 Lowest Frequency	-1848.9
17 Nucleus	13C
18 Acquired Size	32768
19 Spectral Size	32768

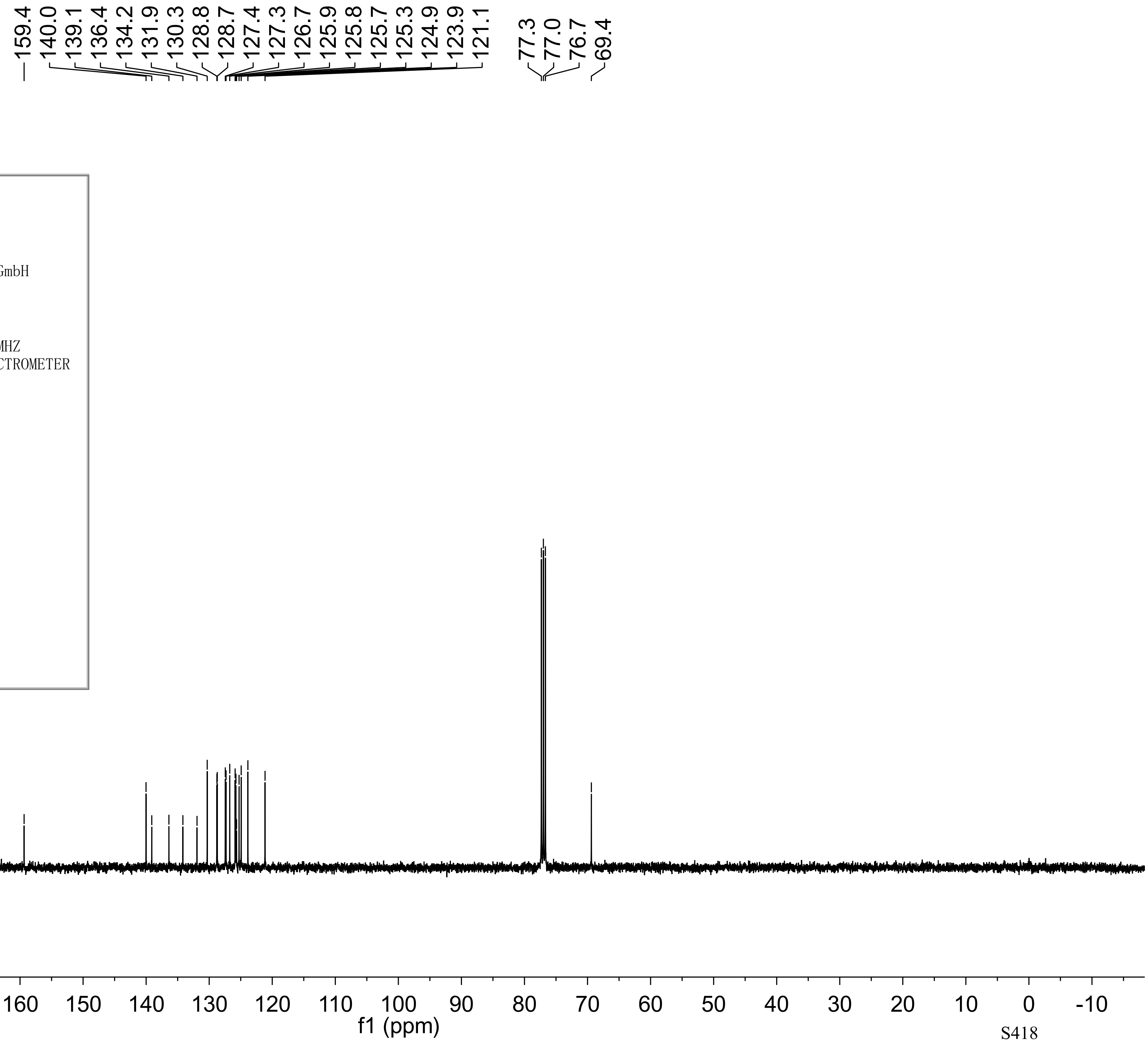

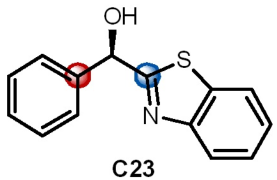

C23

	Parameter	Value
1	Title	R-CFM-D1-2
2	Comment	
3	Origin	Bruker BioSpin GmbH
4	Owner	nmrsu
5	Site	
6	Spectrometer	Avance NEO 600
7	Author	
8	Solvent	CDCl3
9	Temperature	296.3
10	Pulse Sequence	zg30
11	Experiment	1D
12	Number of Scans	8
13	Receiver Gain	85
14	Relaxation Delay	1.0000
15	Pulse Width	10.0000
16	Acquisition Time	2.7525
17	Acquisition Date	2020-04-16T11:29:58
18	Modification Date	2020-04-16T14:16:57
19	Spectrometer Frequency	600.15
20	Spectral Width	11904.8
21	Lowest Frequency	-2259.3
22	Nucleus	1H
23	Acquired Size	32768
24	Spectral Size	65536

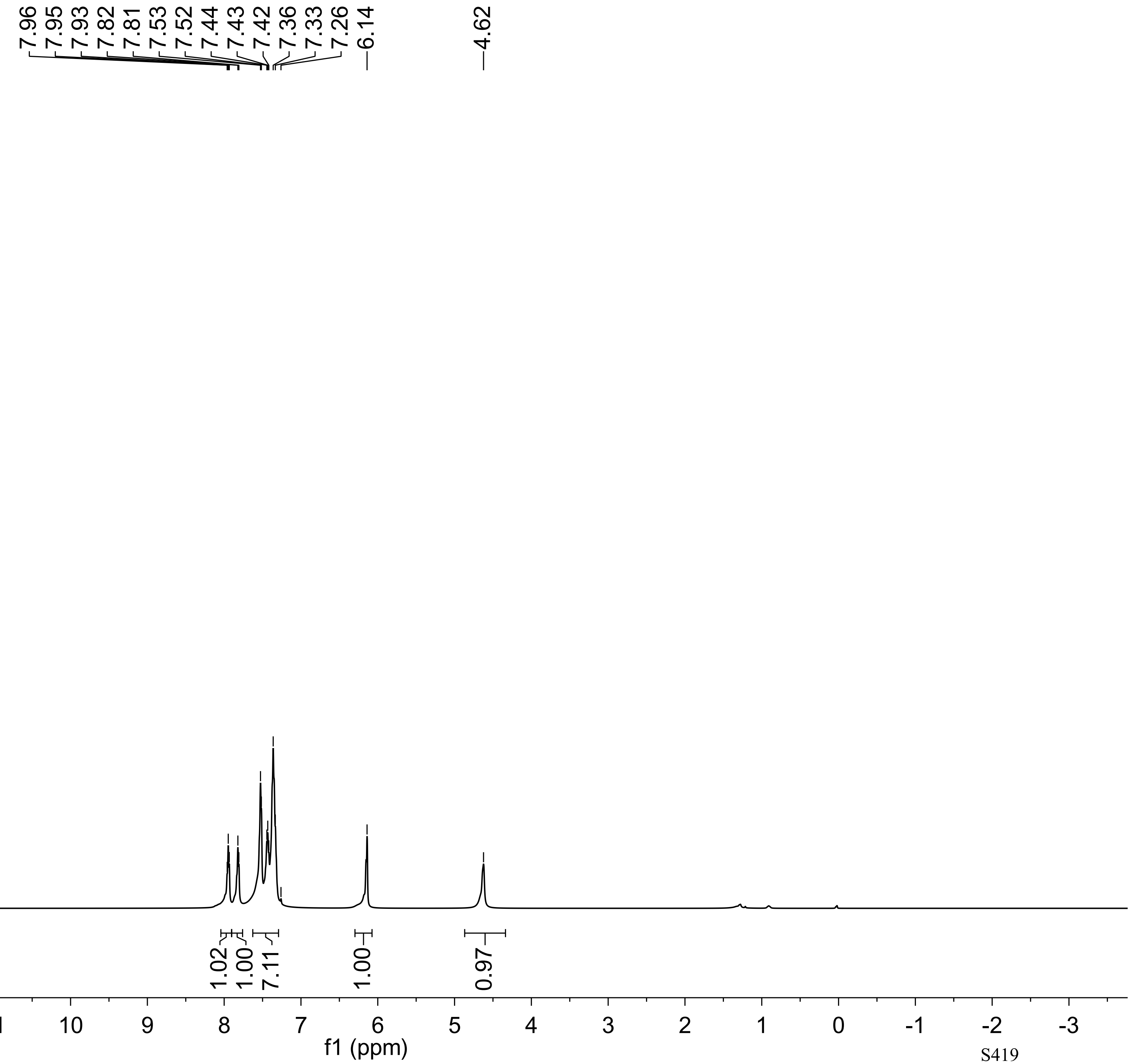

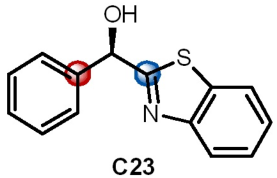

C23

Parameter	Value
1 Title	R-CFM-D1-2
2 Comment	
3 Origin	Bruker BioSpin GmbH
4 Owner	nmrsu
5 Site	
6 Spectrometer	Avance NEO 600
7 Author	
8 Solvent	CDCl3
9 Temperature	297.2
10 Pulse Sequence	zgpg30
11 Experiment	1D
12 Number of Scans	32
13 Receiver Gain	101
14 Relaxation Delay	2.0000
15 Pulse Width	12.0000
16 Acquisition Time	0.9175
17 Acquisition Date	2020-04-16T11:32:50
18 Modification Date	2020-04-16T14:16:57
19 Spectrometer Frequency	150.91
20 Spectral Width	35714.3
21 Lowest Frequency	-2780.2
22 Nucleus	13C
23 Acquired Size	32768
24 Spectral Size	32768

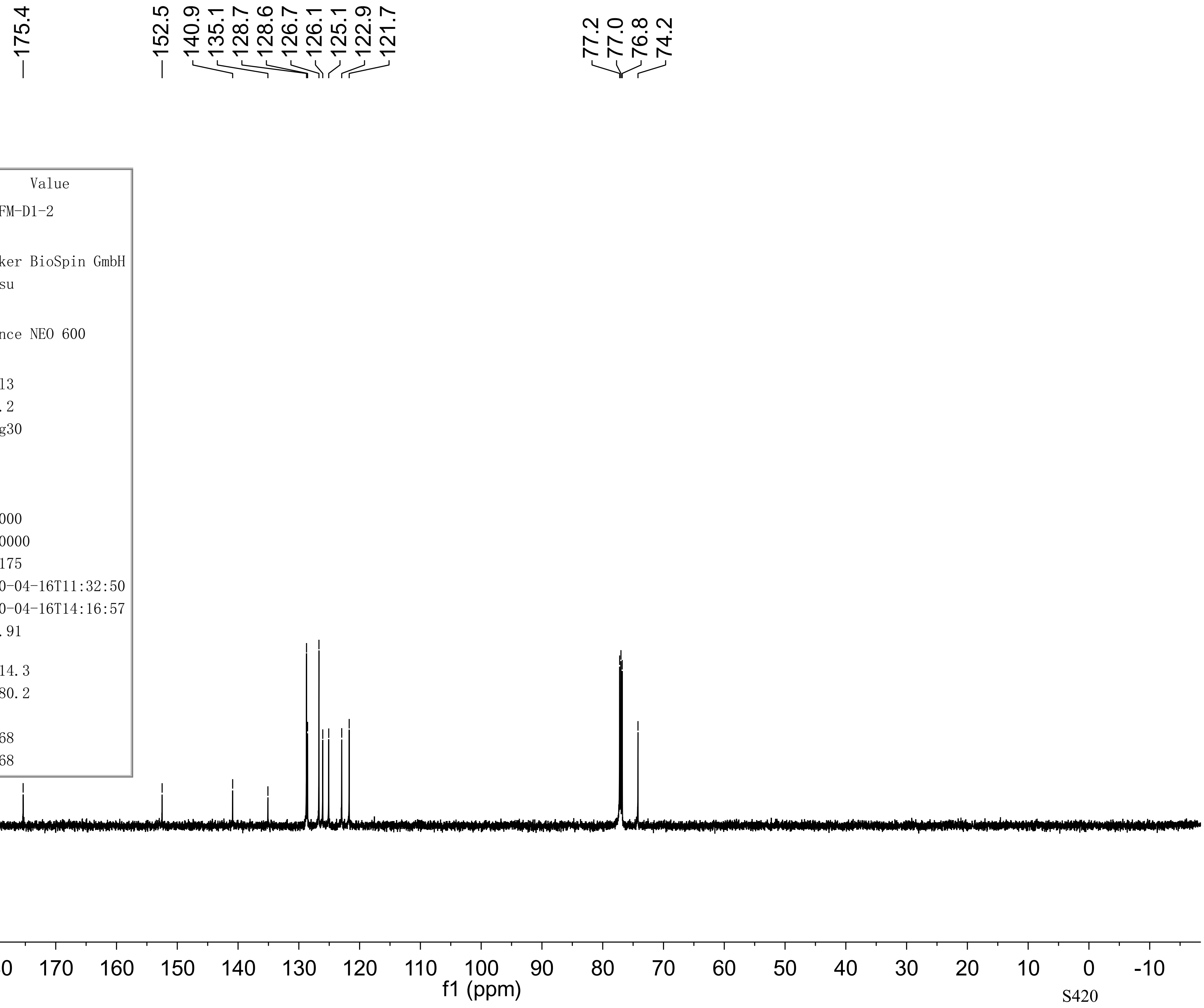

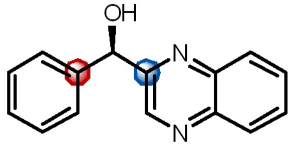

C24

	Parameter	Value
1	Title	CFM-1106-7
2	Comment	
3	Origin	Bruker BioSpin GmbH
4	Owner	nmrsu
5	Site	
6	Spectrometer	Avance NEO 600
7	Author	
8	Solvent	CDCl3
9	Temperature	296.0
10	Pulse Sequence	zg30
11	Experiment	1D
12	Number of Scans	16
13	Receiver Gain	101
14	Relaxation Delay	1.0000
15	Pulse Width	10.0000
16	Acquisition Time	2.7525
17	Acquisition Date	2019-11-07T02:40:37
18	Modification Date	2019-11-07T09:53:10
19	Spectrometer Frequency	600.15
20	Spectral Width	11904.8
21	Lowest Frequency	-2261.3
22	Nucleus	1H
23	Acquired Size	32768
24	Spectral Size	65536

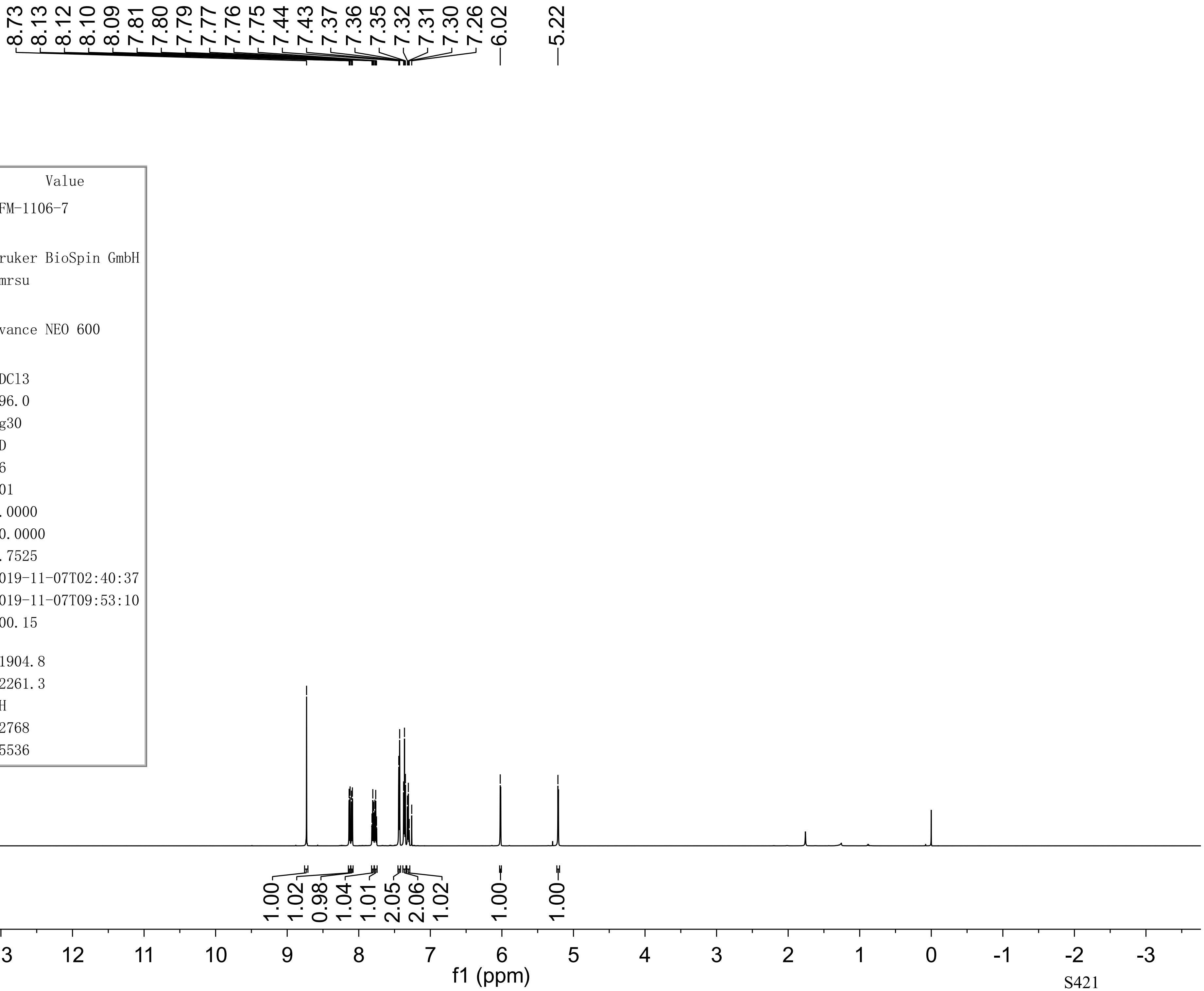

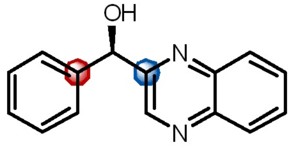

C24

Parameter	Value
1 Title	CFM-1106-7
2 Comment	
3 Origin	Bruker BioSpin GmbH
4 Owner	nmrsu
5 Site	
6 Spectrometer	Avance NEO 600
7 Author	
8 Solvent	CDCl3
9 Temperature	297.4
10 Pulse Sequence	zgpg30
11 Experiment	1D
12 Number of Scans	256
13 Receiver Gain	101
14 Relaxation Delay	2.0000
15 Pulse Width	12.0000
16 Acquisition Time	0.9175
17 Acquisition Date	2019-11-07T02:54:26
18 Modification Date	2019-11-07T09:53:11
19 Spectrometer Frequency	150.91
20 Spectral Width	35714.3
21 Lowest Frequency	-2775.0
22 Nucleus	13C
23 Acquired Size	32768
24 Spectral Size	32768

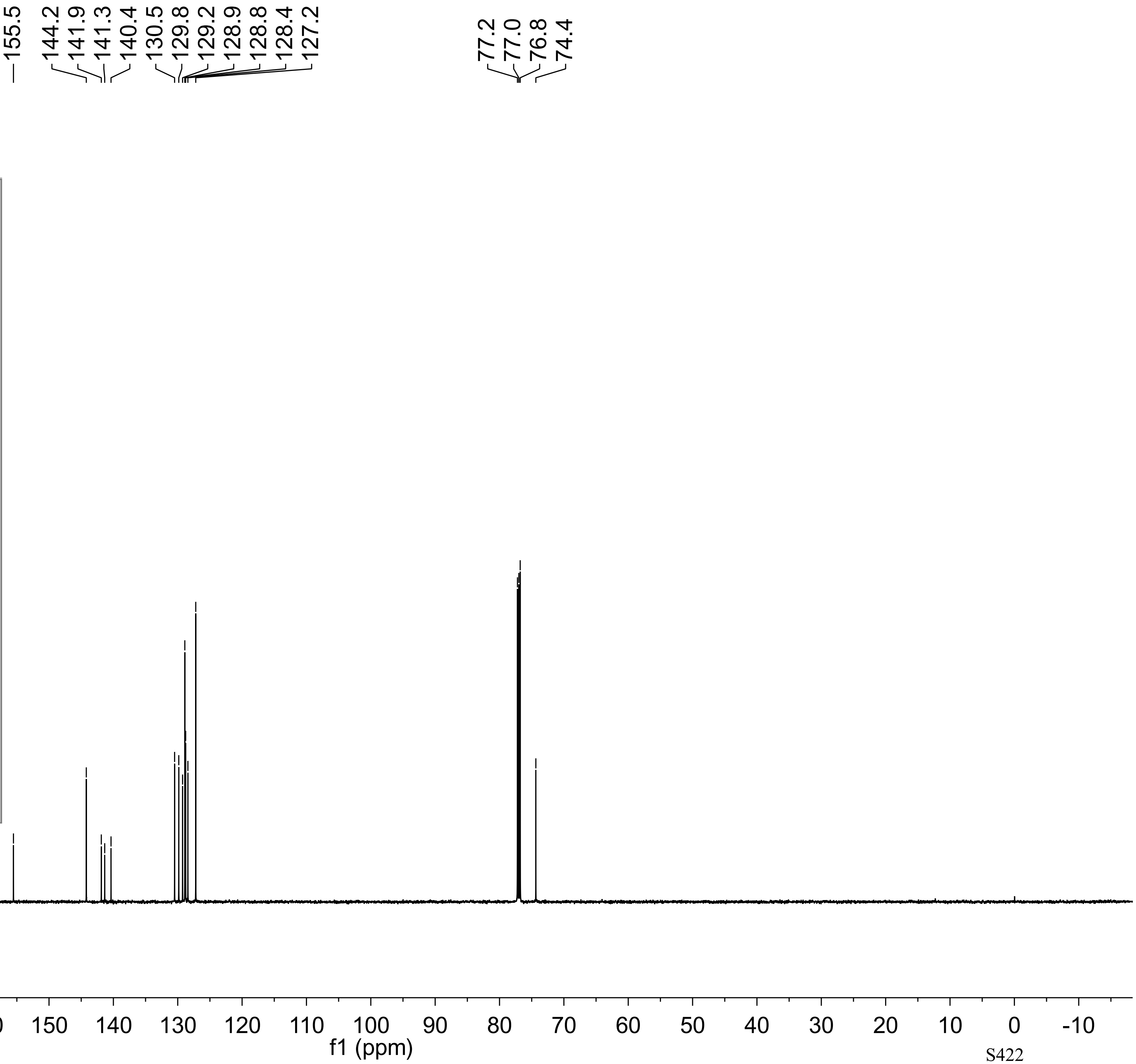

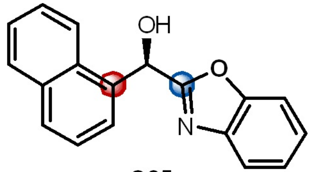

C25

Parameter	Value
1 Title	R-CFM-D4
2 Comment	
3 Origin	Bruker BioSpin GmbH
4 Owner	nmrsu
5 Site	
6 Spectrometer	Avance NEO 600
7 Author	
8 Solvent	CDCl3
9 Temperature	296.4
10 Pulse Sequence	zg30
11 Experiment	1D
12 Number of Scans	16
13 Receiver Gain	101
14 Relaxation Delay	1.0000
15 Pulse Width	10.0000
16 Acquisition Time	2.7525
17 Acquisition Date	2019-12-30T06:05:52
18 Modification Date	2019-12-30T08:57:46
19 Spectrometer Frequency	600.15
20 Spectral Width	11904.8
21 Lowest Frequency	-2261.0
22 Nucleus	1H
23 Acquired Size	32768
24 Spectral Size	65536

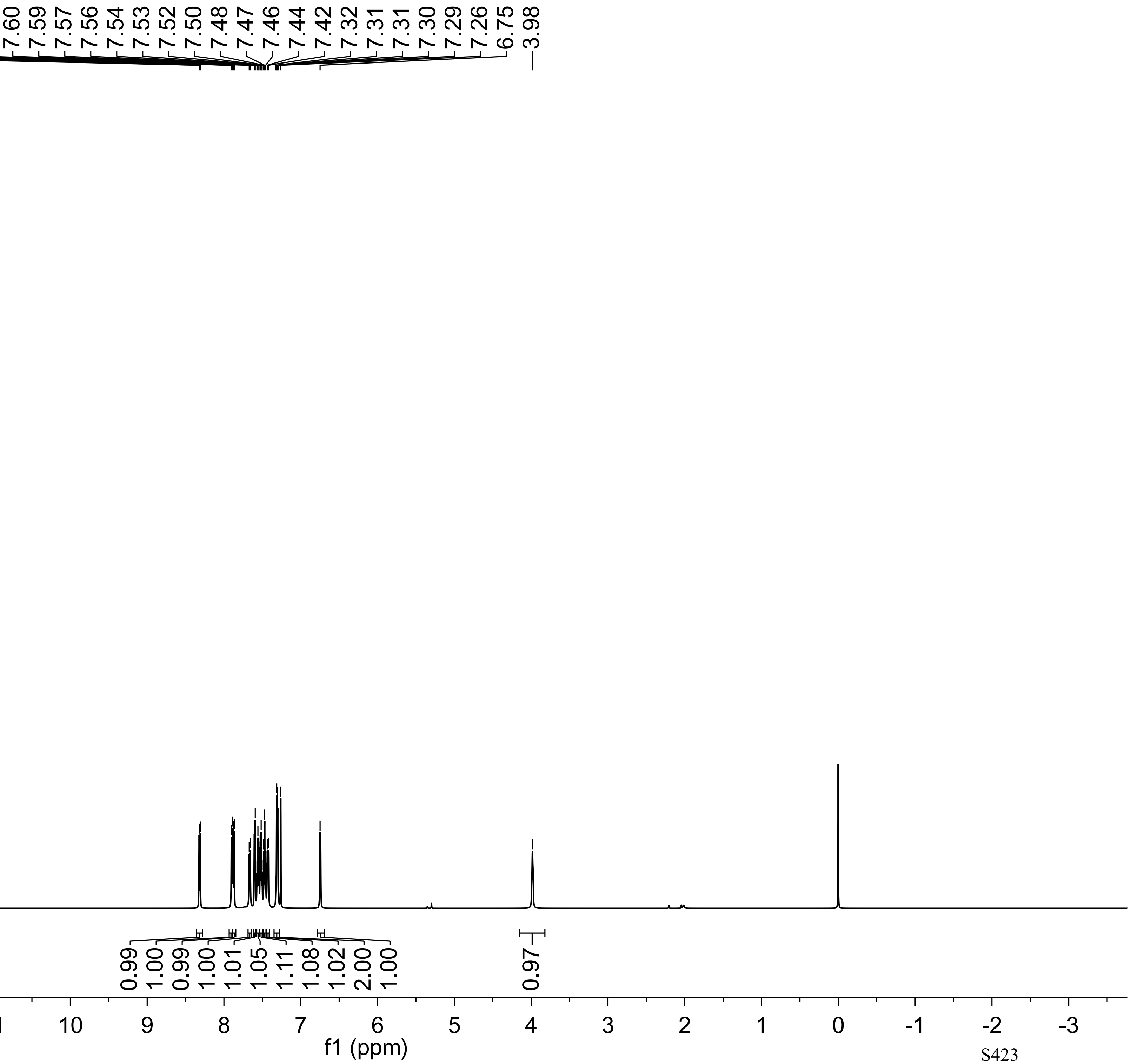

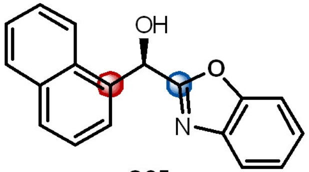

C25

Parameter	Value
1 Title	R-CFM-D4
2 Comment	
3 Origin	Bruker BioSpin GmbH
4 Owner	nmrsu
5 Site	
6 Spectrometer	Avance NEO 600
7 Author	
8 Solvent	CDCl3
9 Temperature	298.1
10 Pulse Sequence	zgpg30
11 Experiment	1D
12 Number of Scans	512
13 Receiver Gain	101
14 Relaxation Delay	2.0000
15 Pulse Width	12.0000
16 Acquisition Time	0.9175
17 Acquisition Date	2019-12-30T06:32:31
18 Modification Date	2019-12-30T08:57:46
19 Spectrometer Frequency	150.91
20 Spectral Width	35714.3
21 Lowest Frequency	-2771.2
22 Nucleus	13C
23 Acquired Size	32768
24 Spectral Size	32768

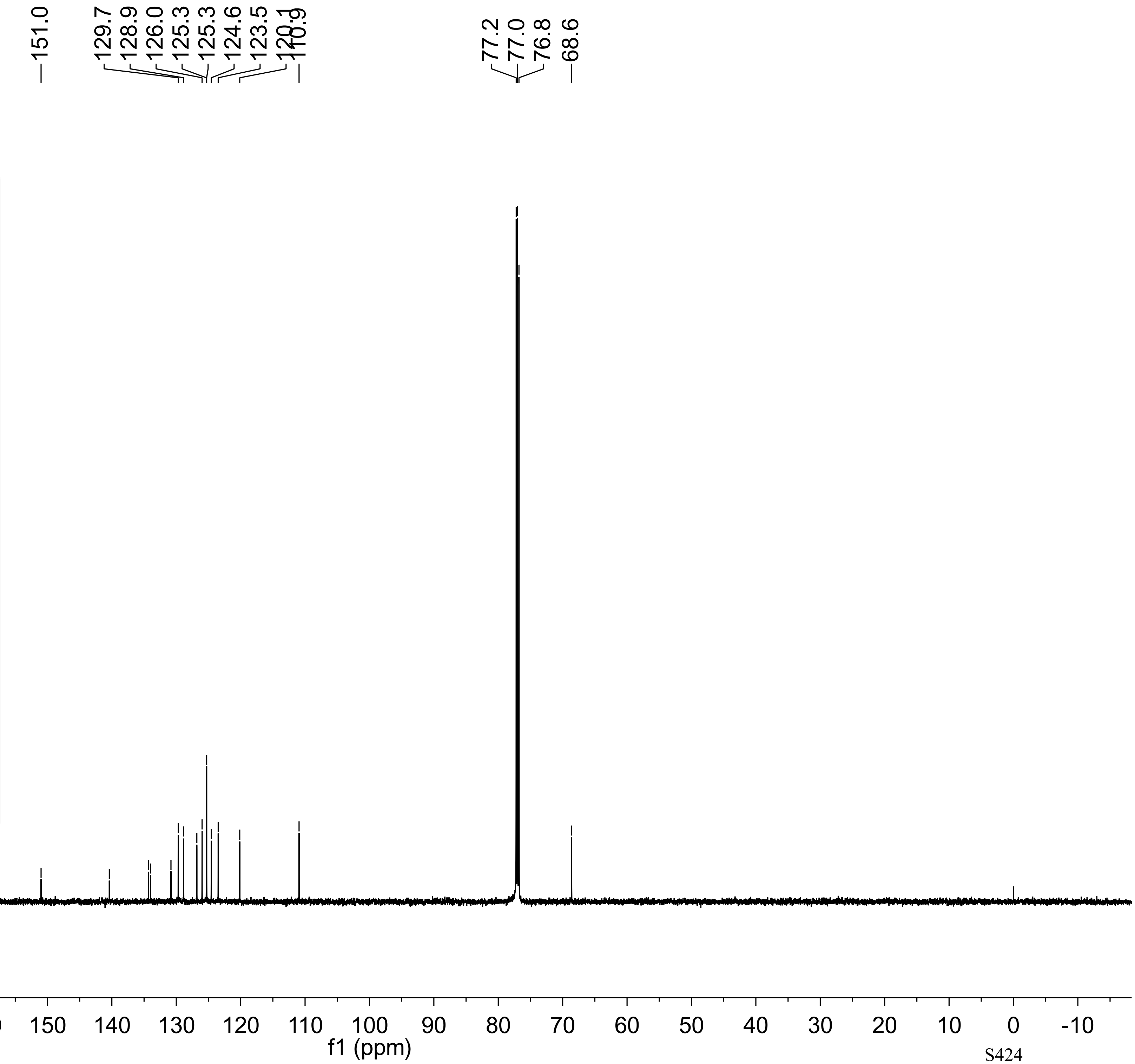

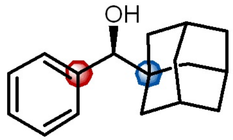

C26

	Parameter	Value
1	Title	R-CFM-G6
2	Comment	
3	Origin	Bruker BioSpin GmbH
4	Owner	nmrsu
5	Site	
6	Spectrometer	Avance NEO 600
7	Author	
8	Solvent	CDCl3
9	Temperature	296.2
10	Pulse Sequence	zg30
11	Experiment	1D
12	Number of Scans	16
13	Receiver Gain	101
14	Relaxation Delay	1.0000
15	Pulse Width	10.0000
16	Acquisition Time	2.7525
17	Acquisition Date	2019-12-25T00:27:42
18	Modification Date	2019-12-25T09:13:35
19	Spectrometer Frequency	600.15
20	Spectral Width	11904.8
21	Lowest Frequency	-2273.5
22	Nucleus	1H
23	Acquired Size	32768
24	Spectral Size	65536

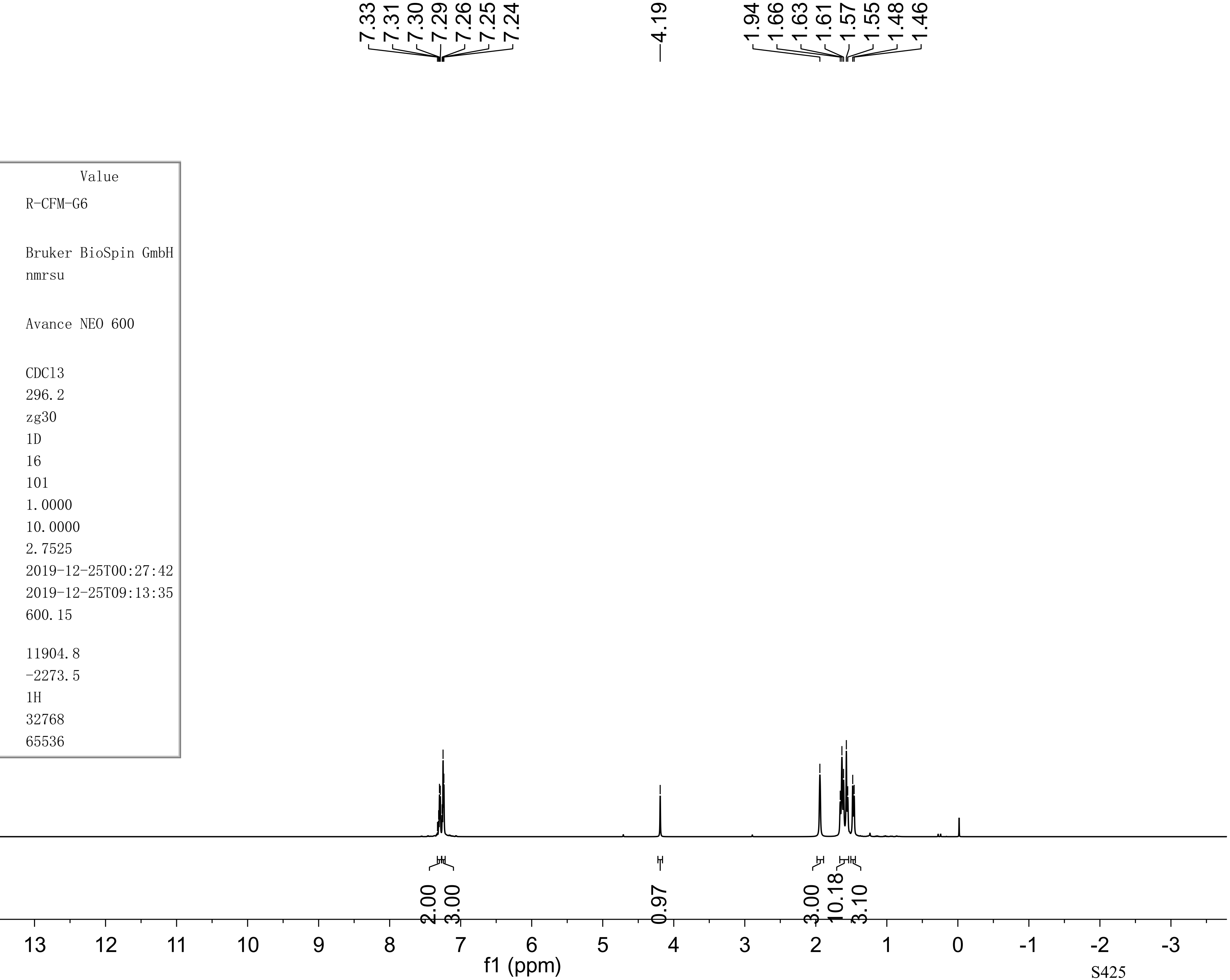

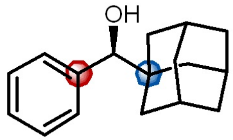

C26

	Parameter	Value
1	Title	R-CFM-G6
2	Comment	
3	Origin	Bruker BioSpin GmbH
4	Owner	nmrsu
5	Site	
6	Spectrometer	Avance NEO 600
7	Author	
8	Solvent	CDCl3
9	Temperature	297.6
10	Pulse Sequence	zgpg30
11	Experiment	1D
12	Number of Scans	256
13	Receiver Gain	101
14	Relaxation Delay	2.0000
15	Pulse Width	12.0000
16	Acquisition Time	0.9175
17	Acquisition Date	2019-12-25T00:41:45
18	Modification Date	2019-12-25T09:13:35
19	Spectrometer Frequency	150.91
20	Spectral Width	35714.3
21	Lowest Frequency	-2772.2
22	Nucleus	13C
23	Acquired Size	32768
24	Spectral Size	32768

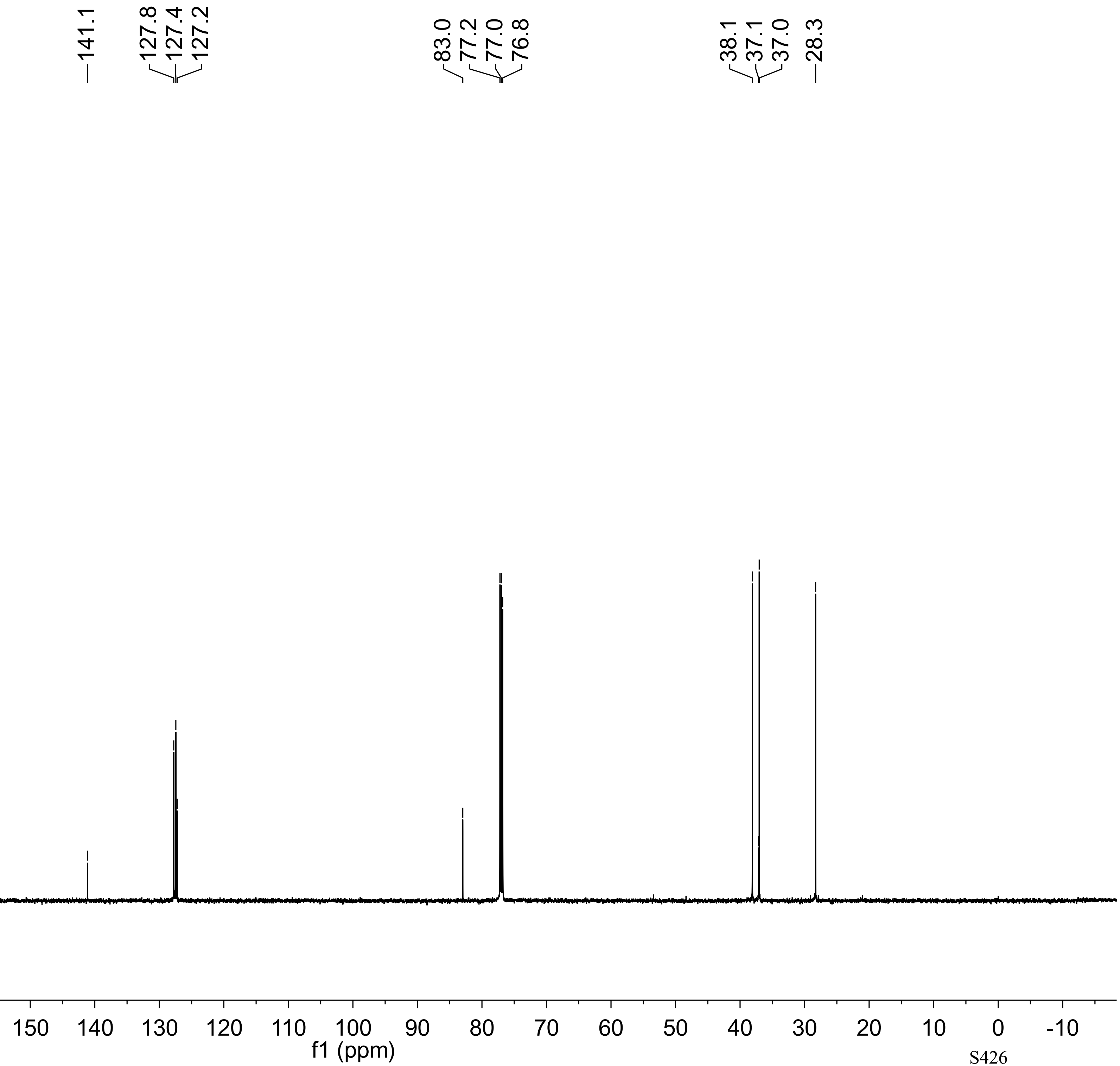

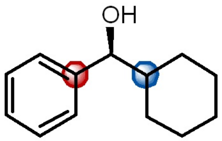

C27

Parameter	Value
1 Title	CFM-6-C27-0701
2 Comment	
3 Origin	Bruker BioSpin GmbH
4 Owner	nmrsu
5 Site	
6 Spectrometer	AVANCE NEO 400 MHZ DIGITAL NMR SPECTROMETER
7 Author	
8 Solvent	CDCl3
9 Temperature	298.1
10 Pulse Sequence	zg30
11 Experiment	1D
12 Number of Scans	8
13 Receiver Gain	78
14 Spectrometer Frequency	400.13
15 Spectral Width	8196.7
16 Lowest Frequency	-1641.8
17 Nucleus	1H
18 Acquired Size	32768
19 Spectral Size	65536

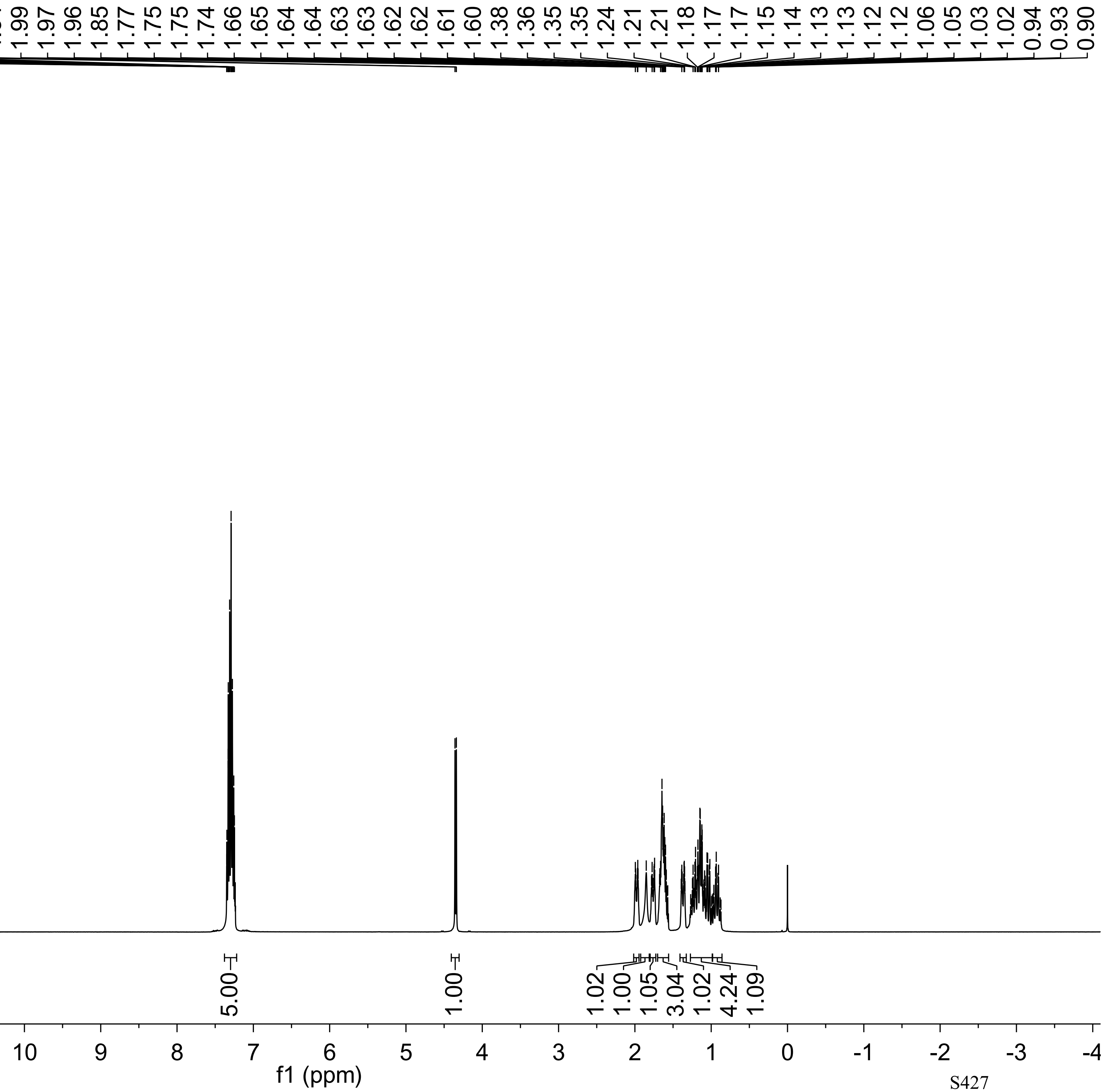

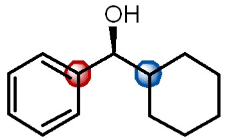

C27

Parameter	Value
1 Title	CFM-6-C27-0701
2 Comment	
3 Origin	Bruker BioSpin GmbH
4 Owner	nmrsu
5 Site	
6 Spectrometer	AVANCE NEO 400 MHZ DIGITAL NMR SPECTROMETER
7 Author	
8 Solvent	CDCl3
9 Temperature	298.2
10 Pulse Sequence	zgpg30
11 Experiment	1D
12 Number of Scans	256
13 Receiver Gain	59
14 Spectrometer Frequency	100.61
15 Spectral Width	23809.5
16 Lowest Frequency	-1849.0
17 Nucleus	13C
18 Acquired Size	32768
19 Spectral Size	32768

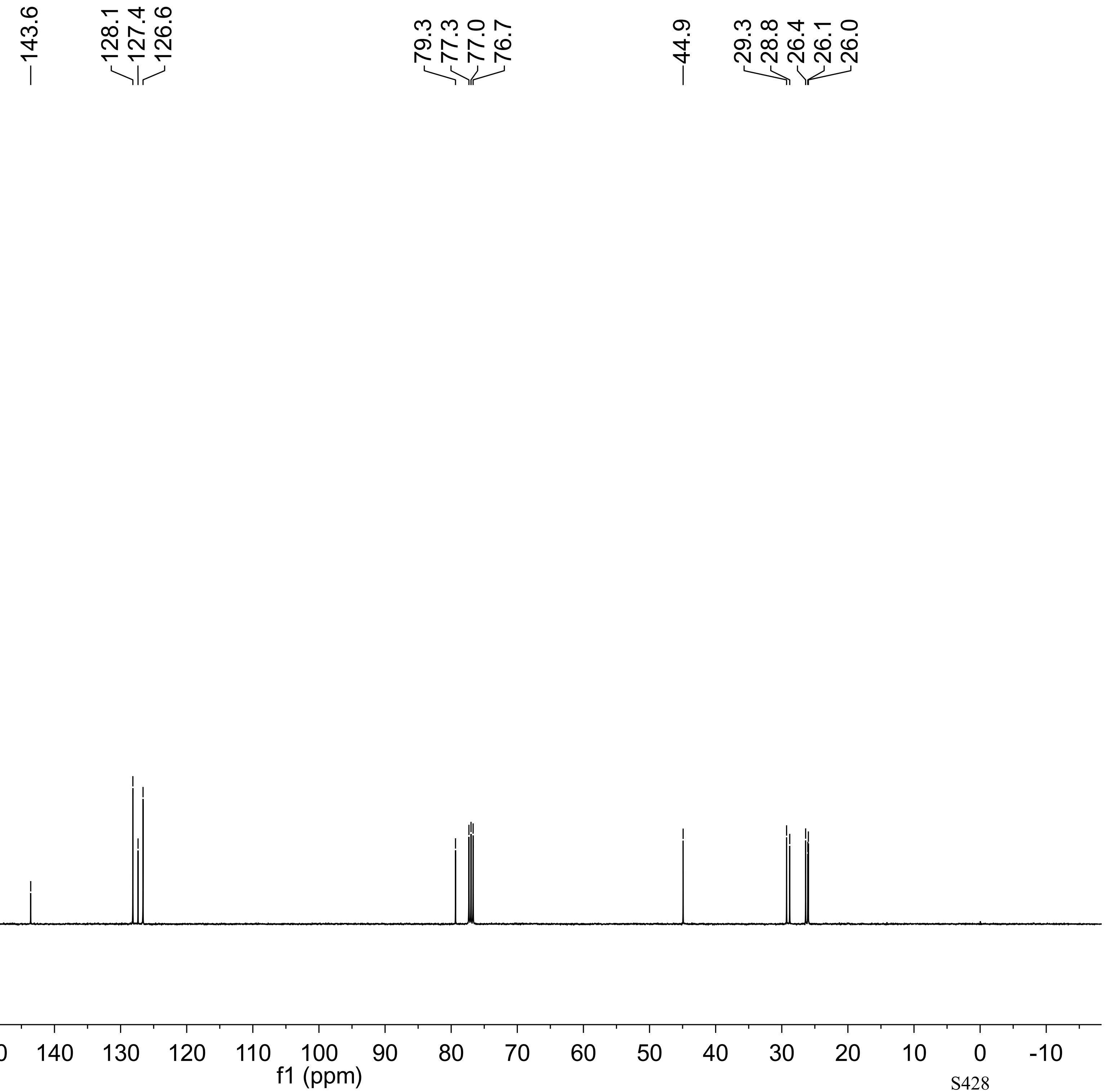

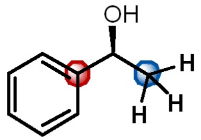

C28

	Parameter	Value
1	Title	CFM-6-C28-0701
2	Comment	
3	Origin	Bruker BioSpin GmbH
4	Owner	nmrsu
5	Site	
6	Spectrometer	AVANCE NEO 400 MHZ DIGITAL NMR SPECTROMETER
7	Author	
8	Solvent	CDCl3
9	Temperature	298.1
10	Pulse Sequence	zg30
11	Experiment	1D
12	Number of Scans	8
13	Receiver Gain	101
14	Spectrometer Frequency	400.13
15	Spectral Width	8196.7
16	Lowest Frequency	-1642.0
17	Nucleus	1H
18	Acquired Size	32768
19	Spectral Size	65536

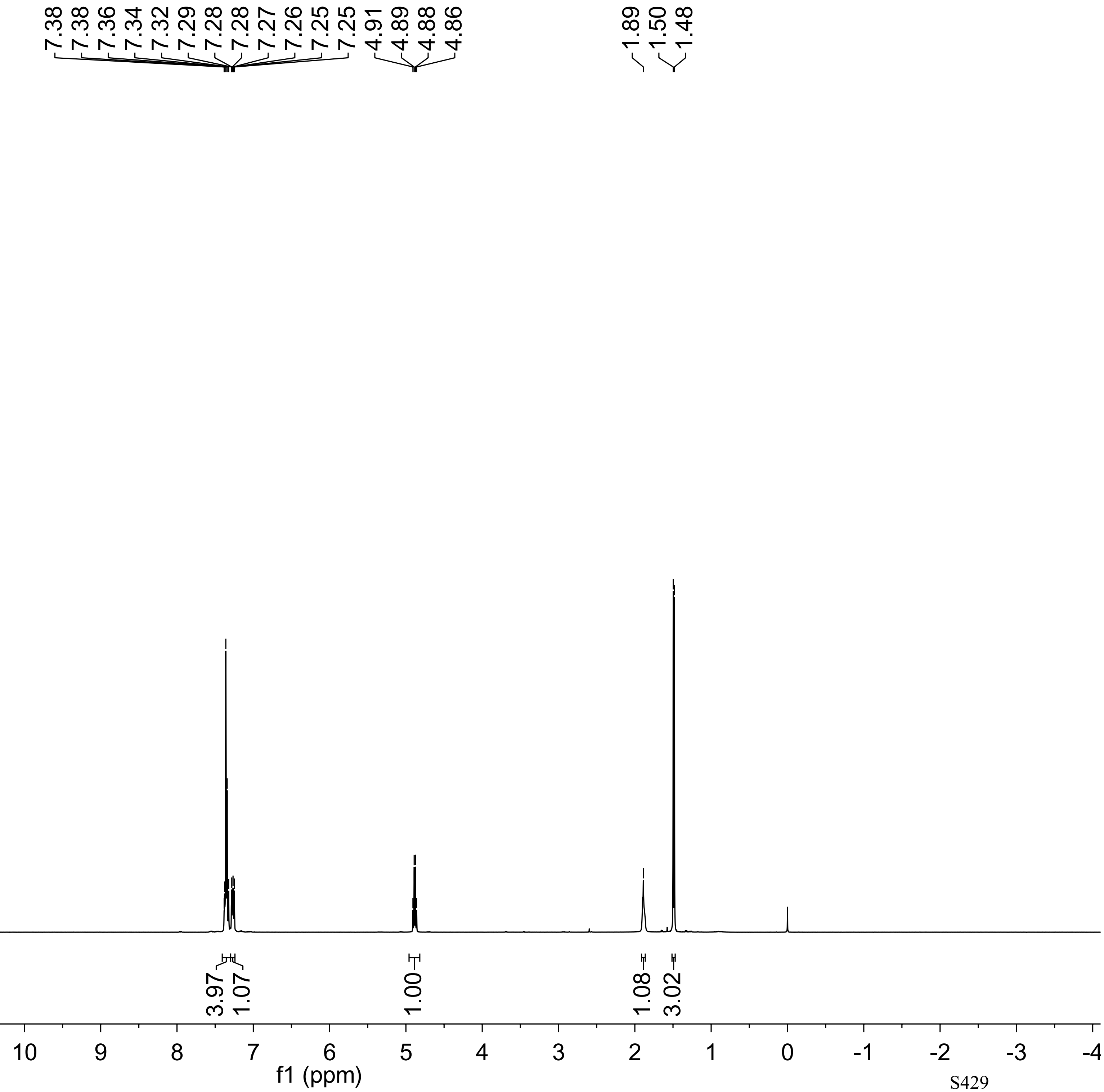

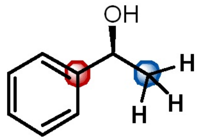

C28

Parameter	Value
1 Title	CFM-6-C28-0701
2 Comment	
3 Origin	Bruker BioSpin GmbH
4 Owner	nmrsu
5 Site	
6 Spectrometer	AVANCE NEO 400 MHZ DIGITAL NMR SPECTROMETER
7 Author	
8 Solvent	CDCl3
9 Temperature	298.2
10 Pulse Sequence	zgpg30
11 Experiment	1D
12 Number of Scans	256
13 Receiver Gain	62
14 Spectrometer Frequency	100.61
15 Spectral Width	23809.5
16 Lowest Frequency	-1848.9
17 Nucleus	13C
18 Acquired Size	32768
19 Spectral Size	32768

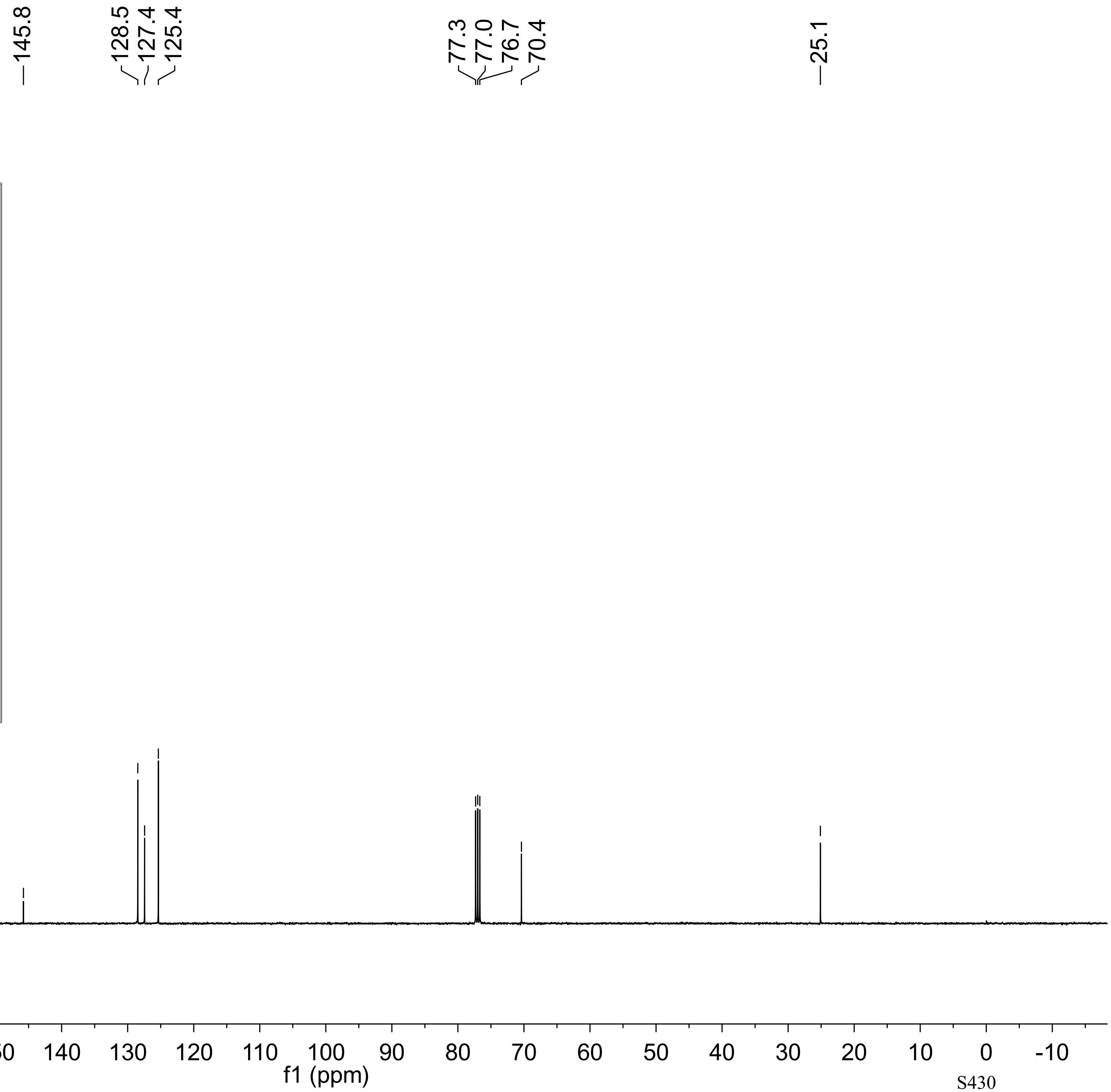

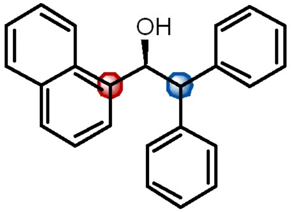

C29

Parameter	Value
1 Title	CFM-R-B3
2 Comment	
3 Origin	Bruker BioSpin GmbH
4 Owner	nmrsu
5 Site	
6 Spectrometer	Avance NEO 600
7 Author	
8 Solvent	CDCl3
9 Temperature	297.8
10 Pulse Sequence	zg30
11 Experiment	1D
12 Number of Scans	8
13 Receiver Gain	101
14 Relaxation Delay	1.0000
15 Pulse Width	10.0000
16 Acquisition Time	2.7525
17 Acquisition Date	2020-08-05T22:57:09
18 Modification Date	2020-08-06T09:01:57
19 Spectrometer Frequency	600.15
20 Spectral Width	11904.8
21 Lowest Frequency	-2251.3
22 Nucleus	1H
23 Acquired Size	32768
24 Spectral Size	65536

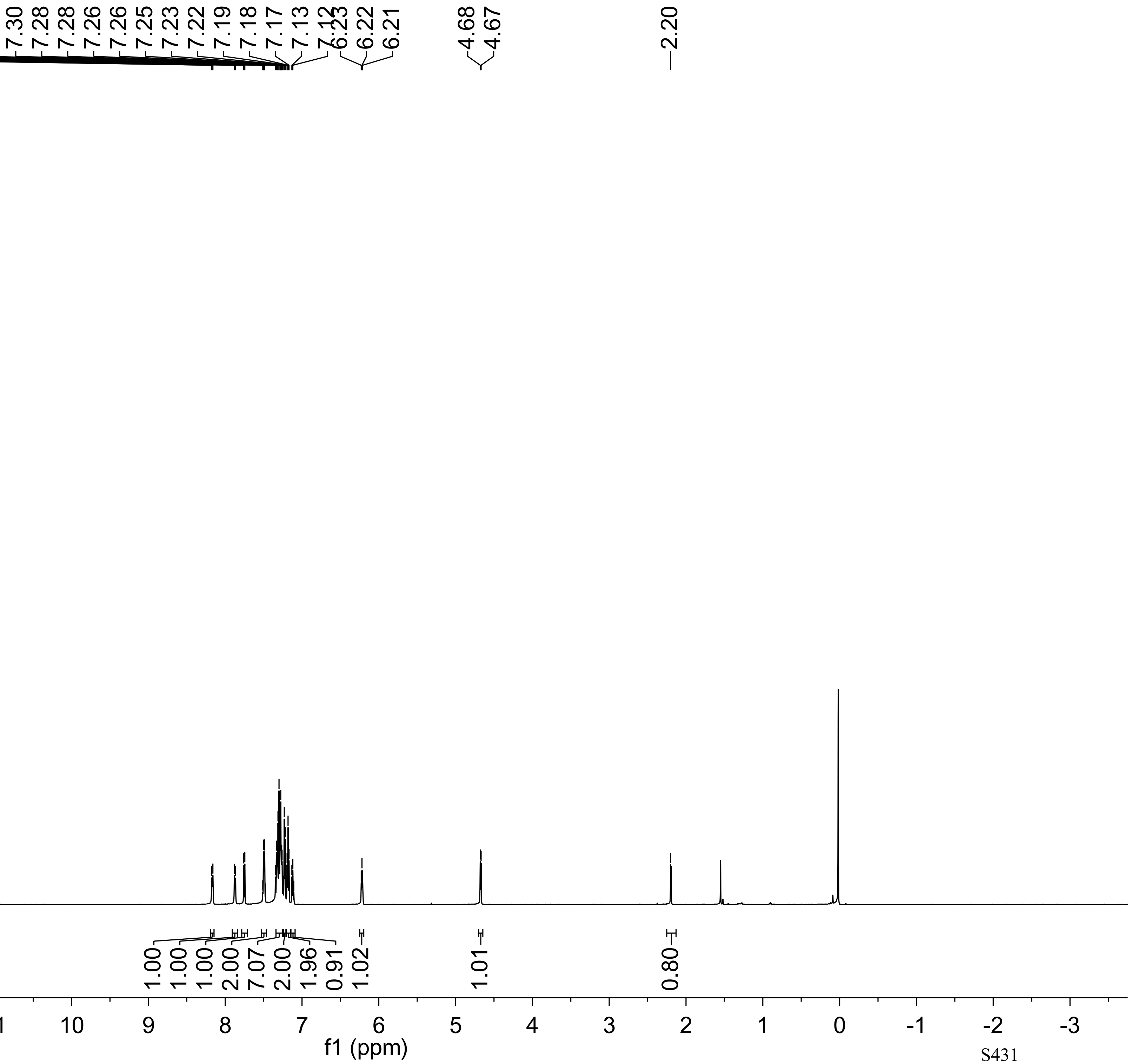

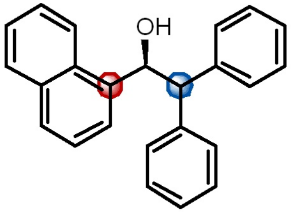

C29

Parameter	Value
1 Title	R-CFM-B3
2 Comment	
3 Origin	Bruker BioSpin GmbH
4 Owner	nmrsu
5 Site	
6 Spectrometer	Avance NEO 600
7 Author	
8 Solvent	CDCl3
9 Temperature	297.3
10 Pulse Sequence	zgpg30
11 Experiment	1D
12 Number of Scans	256
13 Receiver Gain	101
14 Relaxation Delay	2.0000
15 Pulse Width	12.0000
16 Acquisition Time	0.9175
17 Acquisition Date	2019-12-14T02:34:44
18 Modification Date	2019-12-14T09:18:11
19 Spectrometer Frequency	150.91
20 Spectral Width	35714.3
21 Lowest Frequency	-2772.7
22 Nucleus	13C
23 Acquired Size	32768
24 Spectral Size	32768

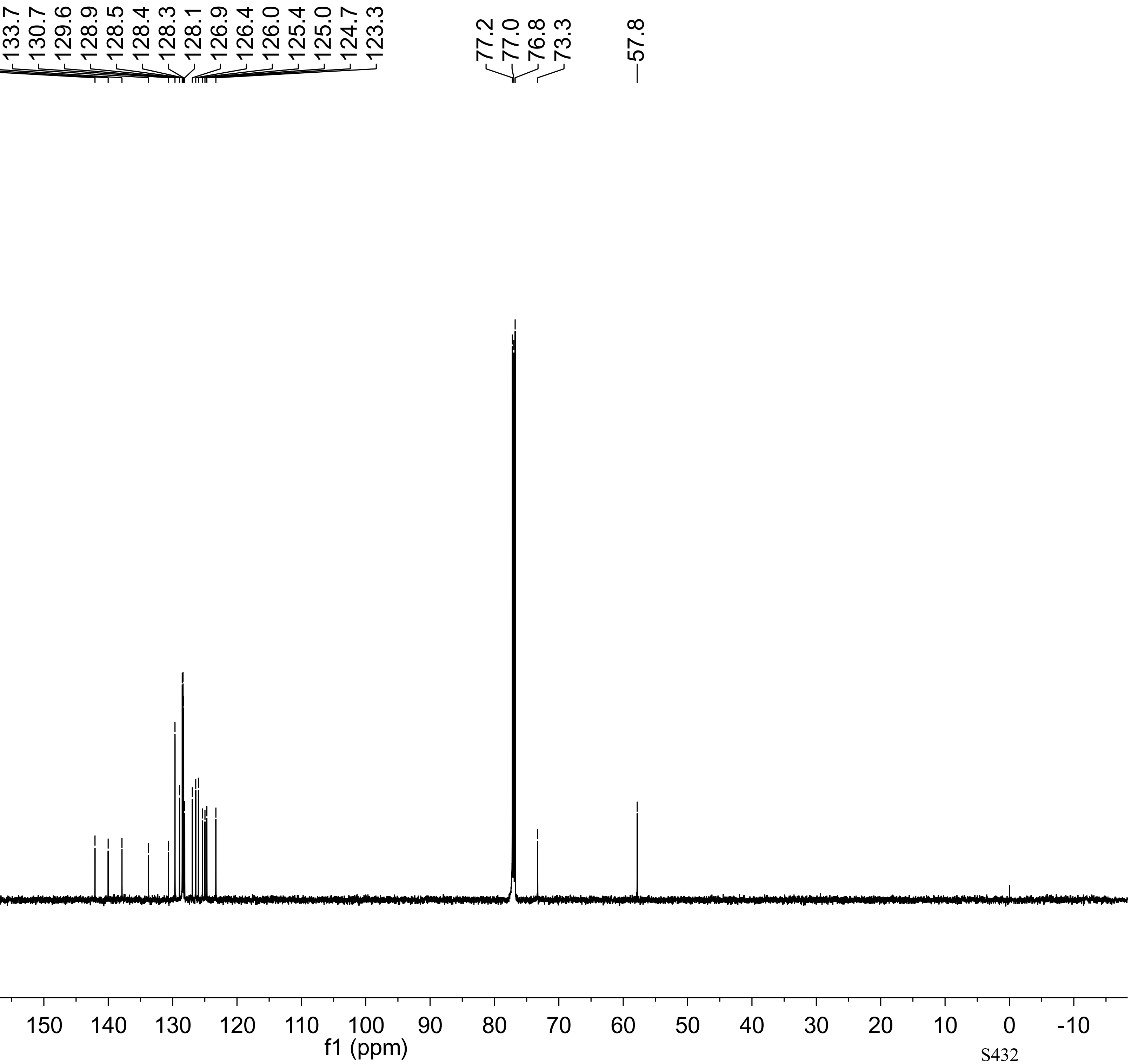

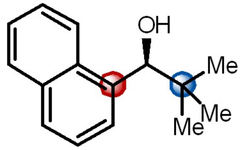

C30

Parameter	Value
1 Title	R-CFM-G4
2 Comment	
3 Origin	Bruker BioSpin GmbH
4 Owner	nmrsu
5 Site	
6 Spectrometer	Avance NEO 600
7 Author	
8 Solvent	CDCl3
9 Temperature	296.3
10 Pulse Sequence	zg30
11 Experiment	1D
12 Number of Scans	16
13 Receiver Gain	101
14 Relaxation Delay	1.0000
15 Pulse Width	10.0000
16 Acquisition Time	2.7525
17 Acquisition Date	2019-12-29T00:22:51
18 Modification Date	2019-12-29T10:16:14
19 Spectrometer Frequency	600.15
20 Spectral Width	11904.8
21 Lowest Frequency	-2260.8
22 Nucleus	1H
23 Acquired Size	32768
24 Spectral Size	65536

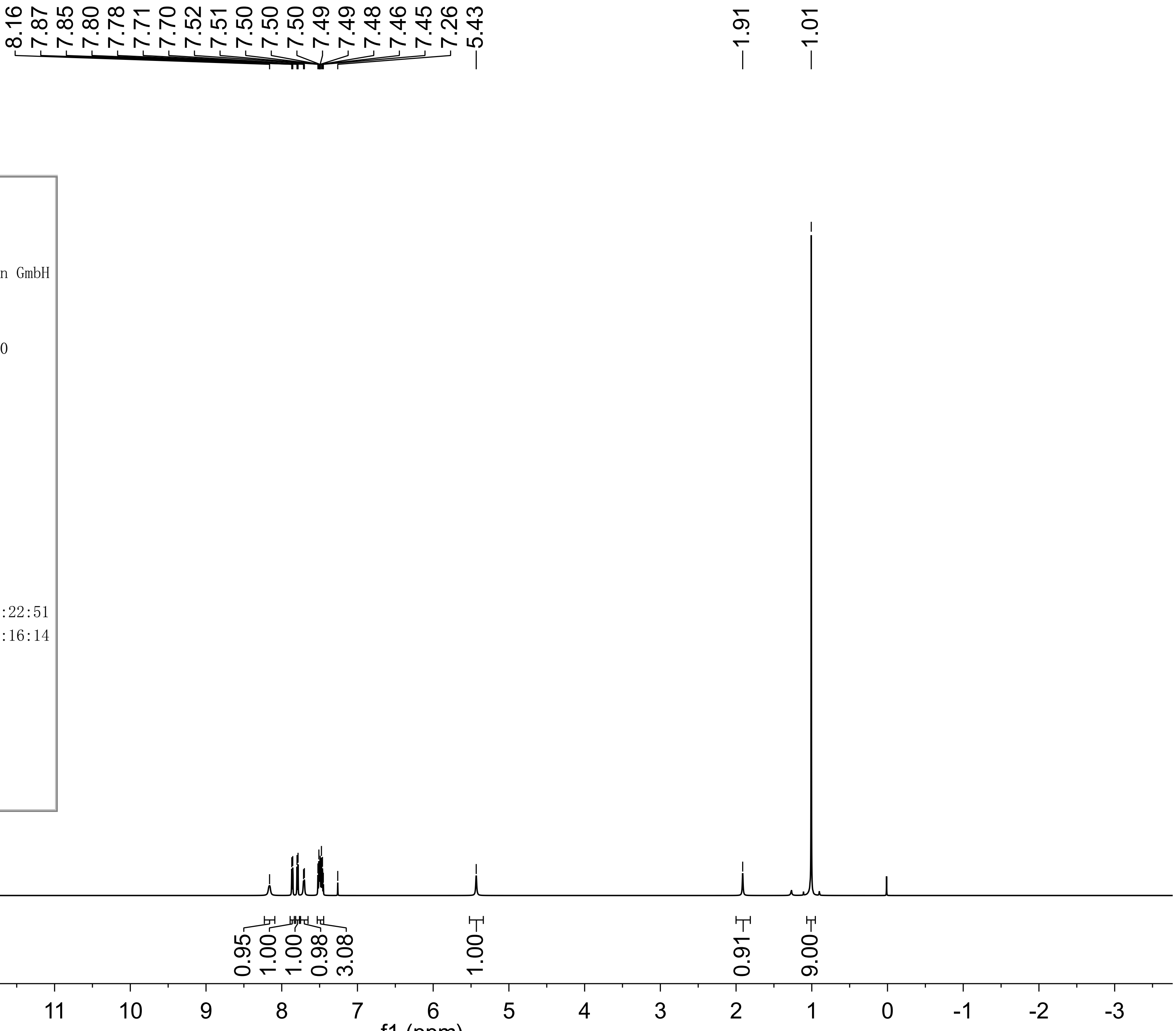

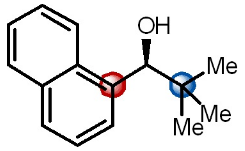

C30

Parameter	Value
1 Title	R-CFM-G4
2 Comment	
3 Origin	Bruker BioSpin GmbH
4 Owner	nmrsu
5 Site	
6 Spectrometer	Avance NEO 600
7 Author	
8 Solvent	CDCl3
9 Temperature	297.8
10 Pulse Sequence	zgpg30
11 Experiment	1D
12 Number of Scans	256
13 Receiver Gain	101
14 Relaxation Delay	2.0000
15 Pulse Width	12.0000
16 Acquisition Time	0.9175
17 Acquisition Date	2019-12-29T00:36:59
18 Modification Date	2019-12-29T10:16:15
19 Spectrometer Frequency	150.91
20 Spectral Width	35714.3
21 Lowest Frequency	-2773.1
22 Nucleus	13C
23 Acquired Size	32768
24 Spectral Size	32768

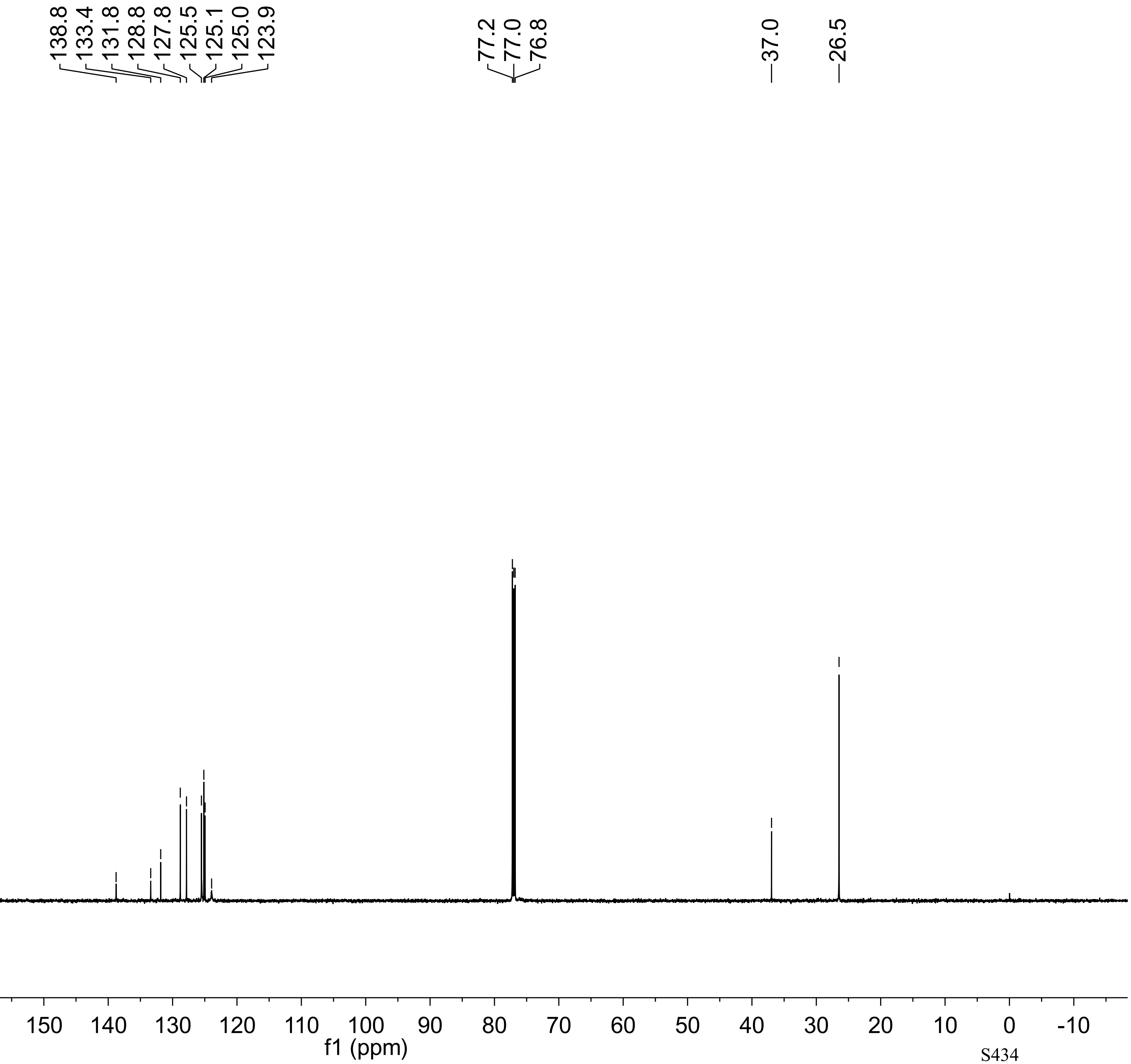

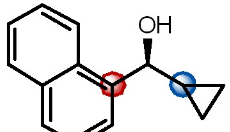

C31

Parameter	Value
1 Title	R-CFM-G3
2 Comment	
3 Origin	Bruker BioSpin GmbH
4 Owner	nmrsu
5 Site	
6 Spectrometer	Avance NEO 600
7 Author	
8 Solvent	CDCl3
9 Temperature	296.7
10 Pulse Sequence	zg30
11 Experiment	1D
12 Number of Scans	16
13 Receiver Gain	77
14 Relaxation Delay	1.0000
15 Pulse Width	10.0000
16 Acquisition Time	2.7525
17 Acquisition Date	2019-12-14T06:04:04
18 Modification Date	2019-12-14T09:18:16
19 Spectrometer Frequency	600.15
20 Spectral Width	11904.8
21 Lowest Frequency	-2261.0
22 Nucleus	1H
23 Acquired Size	32768
24 Spectral Size	65536

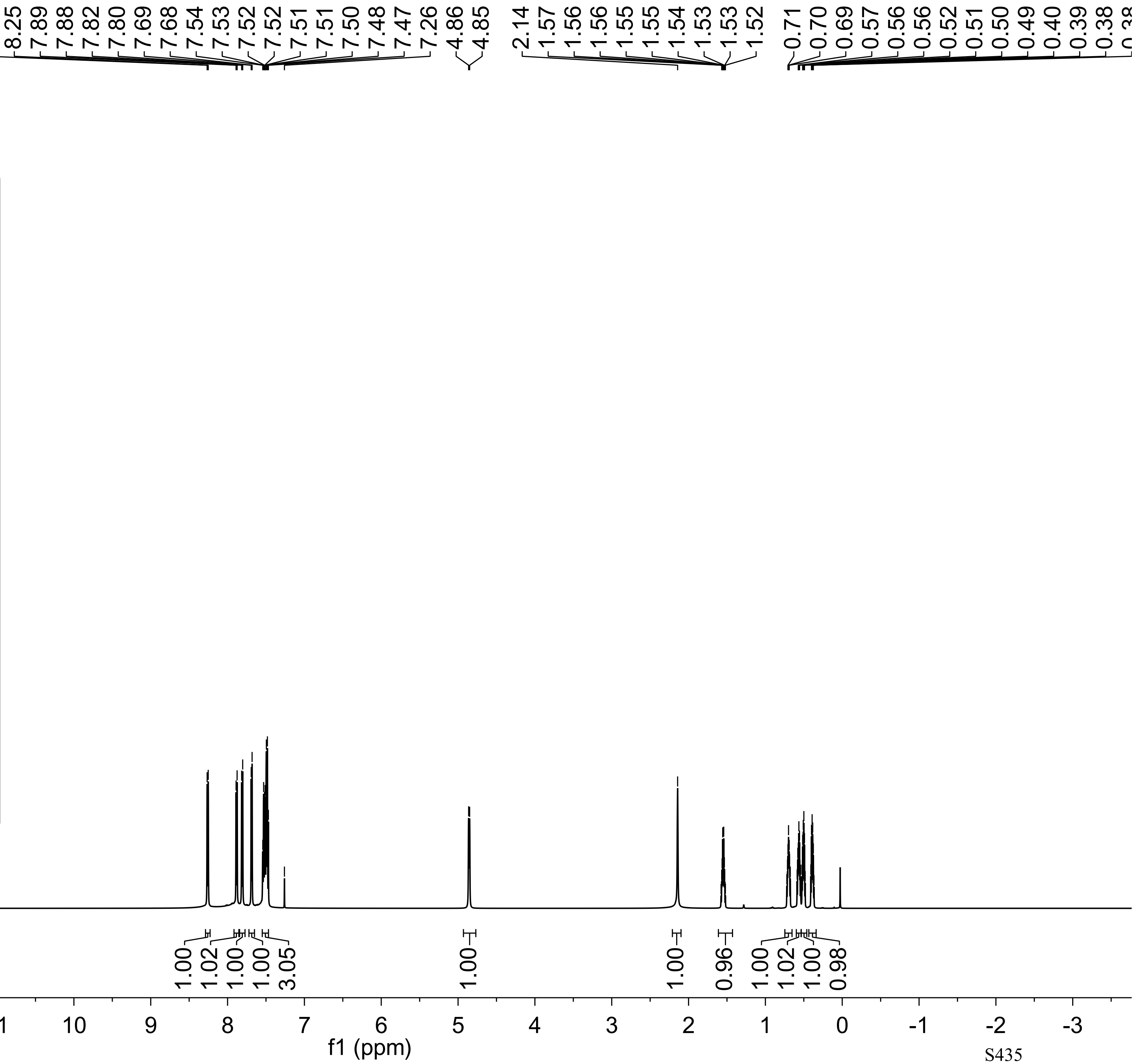

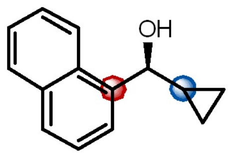

C31

Parameter	Value
1 Title	R-CFM-G3
2 Comment	
3 Origin	Bruker BioSpin GmbH
4 Owner	nmrsu
5 Site	
6 Spectrometer	Avance NEO 600
7 Author	
8 Solvent	CDCl3
9 Temperature	297.4
10 Pulse Sequence	zgpg30
11 Experiment	1D
12 Number of Scans	256
13 Receiver Gain	101
14 Relaxation Delay	2.0000
15 Pulse Width	12.0000
16 Acquisition Time	0.9175
17 Acquisition Date	2019-12-14T06:18:34
18 Modification Date	2019-12-14T09:18:16
19 Spectrometer Frequency	150.91
20 Spectral Width	35714.3
21 Lowest Frequency	-2778.7
22 Nucleus	13C
23 Acquired Size	32768
24 Spectral Size	32768

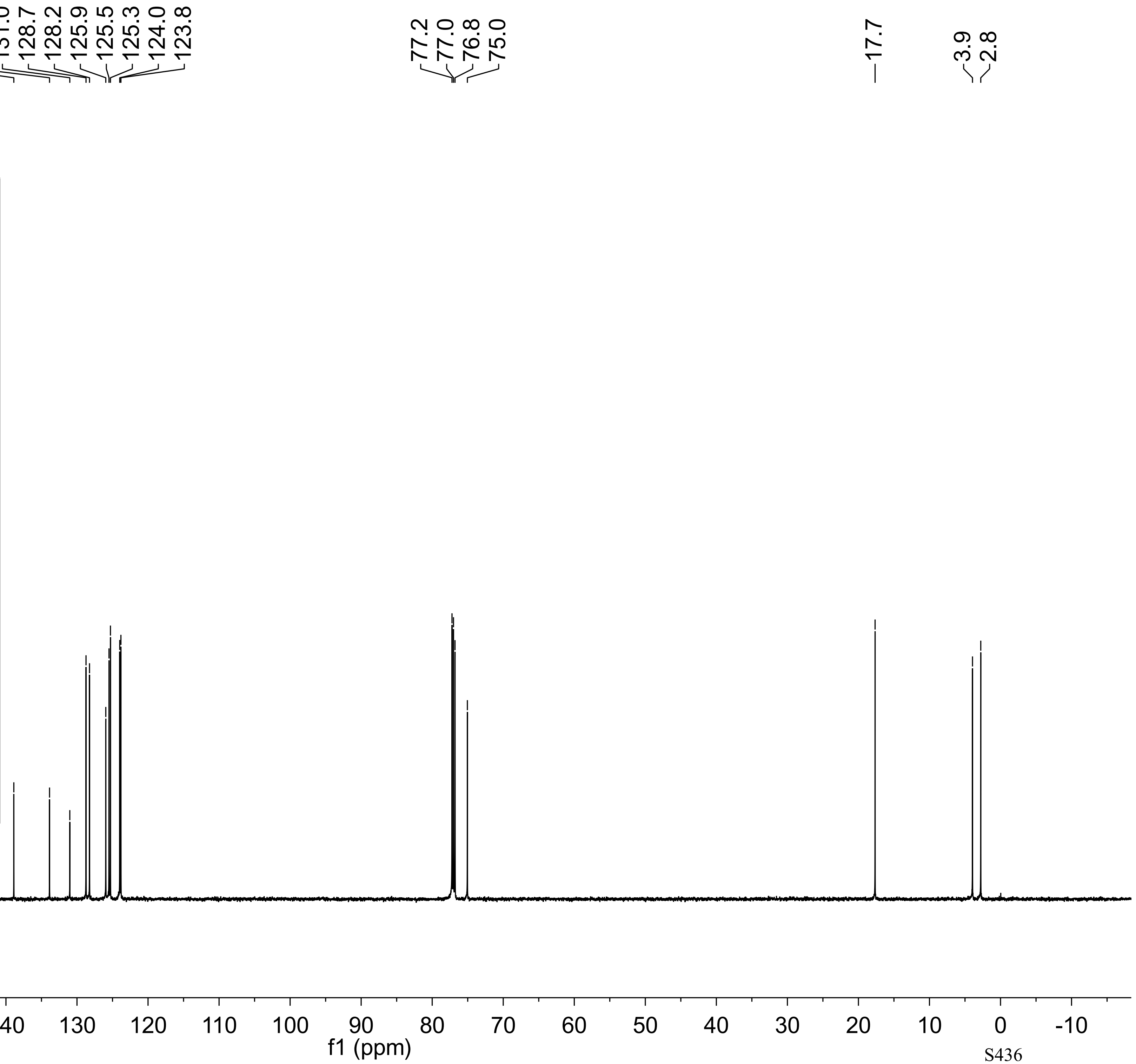

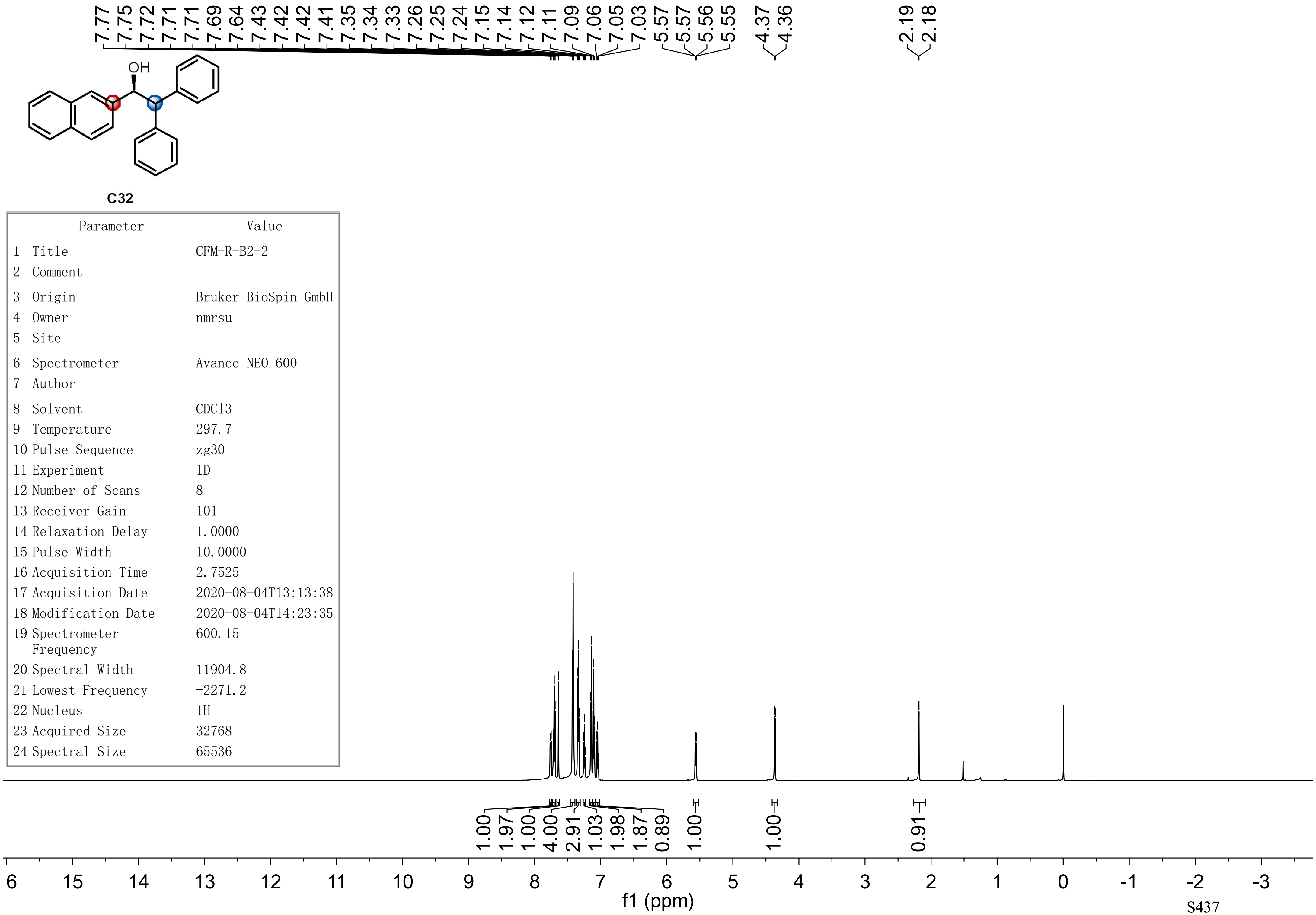

	Parameter	Value
1	Title	CFM-R-B2-2
2	Comment	
3	Origin	Bruker BioSpin GmbH
4	Owner	nmrsu
5	Site	
6	Spectrometer	Avance NEO 600
7	Author	
8	Solvent	CDCl3
9	Temperature	297.7
10	Pulse Sequence	zg30
11	Experiment	1D
12	Number of Scans	8
13	Receiver Gain	101
14	Relaxation Delay	1.0000
15	Pulse Width	10.0000
16	Acquisition Time	2.7525
17	Acquisition Date	2020-08-04T13:13:38
18	Modification Date	2020-08-04T14:23:35
19	Spectrometer Frequency	600.15
20	Spectral Width	11904.8
21	Lowest Frequency	-2271.2
22	Nucleus	1H
23	Acquired Size	32768
24	Spectral Size	65536

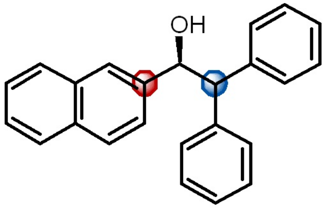

C32

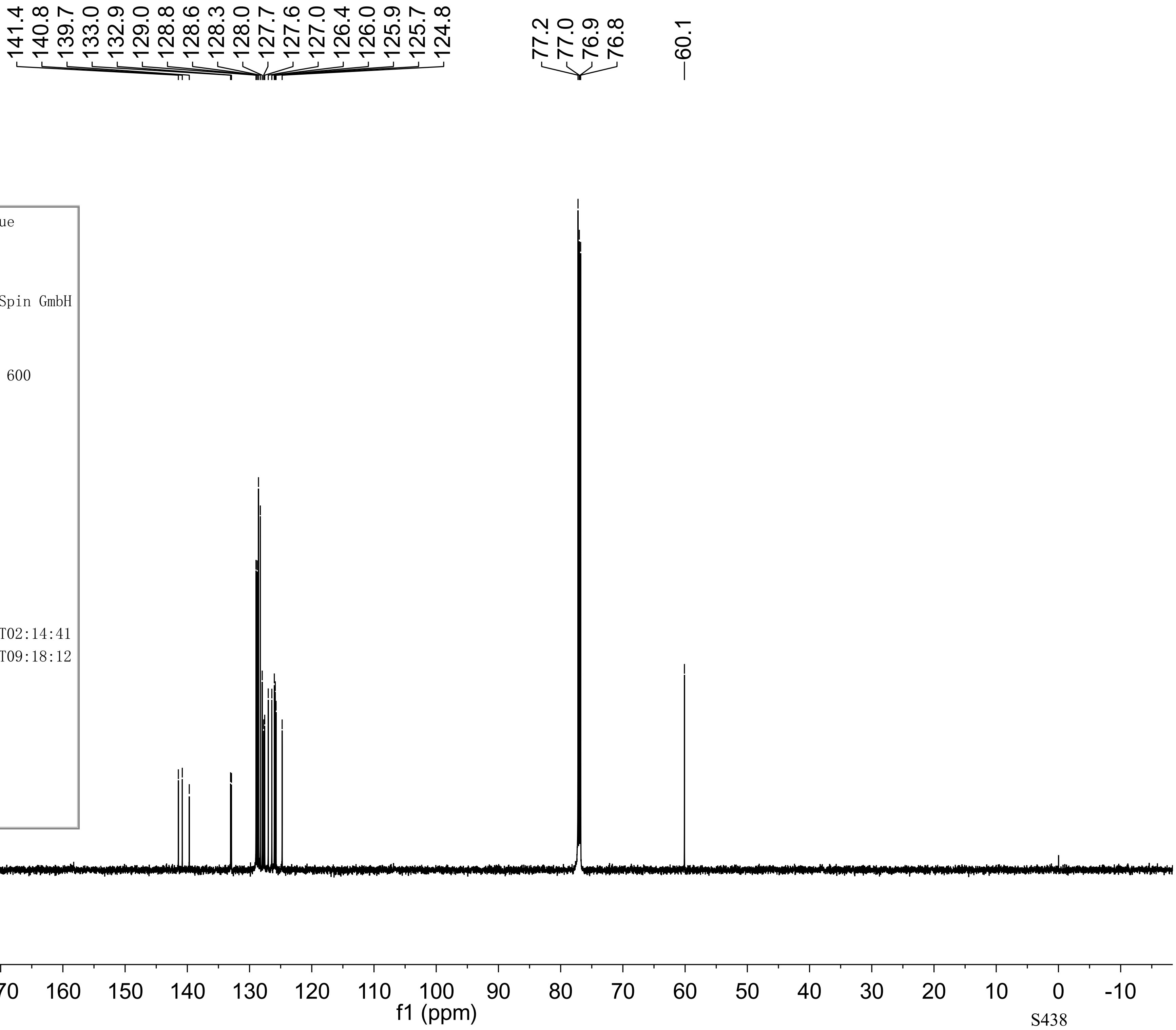

Parameter		Value
1	Title	R-CFM-B2
2	Comment	
3	Origin	Bruker BioSpin GmbH
4	Owner	nmrsu
5	Site	
6	Spectrometer	Avance NEO 600
7	Author	
8	Solvent	CDCl3
9	Temperature	298.0
10	Pulse Sequence	zgpg30
11	Experiment	1D
12	Number of Scans	256
13	Receiver Gain	101
14	Relaxation Delay	2.0000
15	Pulse Width	12.0000
16	Acquisition Time	0.9175
17	Acquisition Date	2019-12-14T02:14:41
18	Modification Date	2019-12-14T09:18:12
19	Spectrometer Frequency	150.91
20	Spectral Width	35714.3
21	Lowest Frequency	-2774.2
22	Nucleus	13C
23	Acquired Size	32768
24	Spectral Size	32768

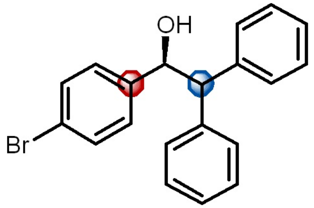

C33

Parameter	Value
1 Title	CFM-0916-4
2 Comment	
3 Origin	Bruker BioSpin GmbH
4 Owner	nmrsu
5 Site	
6 Spectrometer	Avance NEO 600
7 Author	
8 Solvent	CDCl3
9 Temperature	296.2
10 Pulse Sequence	zg30
11 Experiment	1D
12 Number of Scans	16
13 Receiver Gain	101
14 Relaxation Delay	1.0000
15 Pulse Width	10.0000
16 Acquisition Time	2.7525
17 Acquisition Date	2019-11-26T21:35:44
18 Modification Date	2019-11-26T22:04:17
19 Spectrometer Frequency	600.15
20 Spectral Width	11904.8
21 Lowest Frequency	-2261.4
22 Nucleus	1H
23 Acquired Size	32768
24 Spectral Size	65536

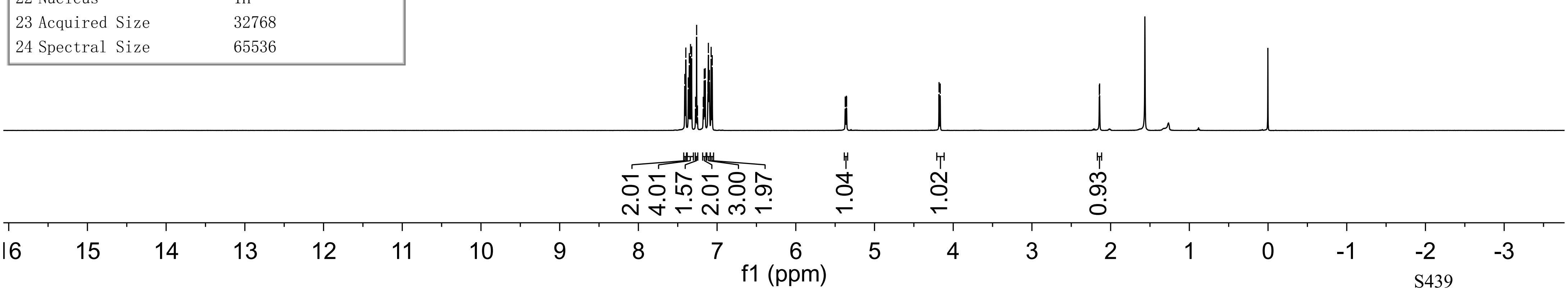

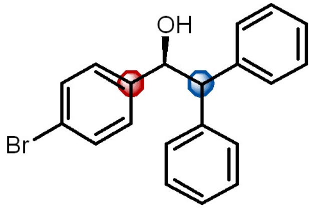

C33

Parameter	Value
1 Title	CFM-0916-4
2 Comment	
3 Origin	Bruker BioSpin GmbH
4 Owner	nmrsu
5 Site	
6 Spectrometer	Avance NEO 600
7 Author	
8 Solvent	CDCl3
9 Temperature	297.5
10 Pulse Sequence	zgpg30
11 Experiment	1D
12 Number of Scans	128
13 Receiver Gain	101
14 Relaxation Delay	2.0000
15 Pulse Width	12.0000
16 Acquisition Time	0.9175
17 Acquisition Date	2019-11-26T21:43:25
18 Modification Date	2019-11-26T22:04:17
19 Spectrometer Frequency	150.91
20 Spectral Width	35714.3
21 Lowest Frequency	-2771.5
22 Nucleus	13C
23 Acquired Size	32768
24 Spectral Size	32768

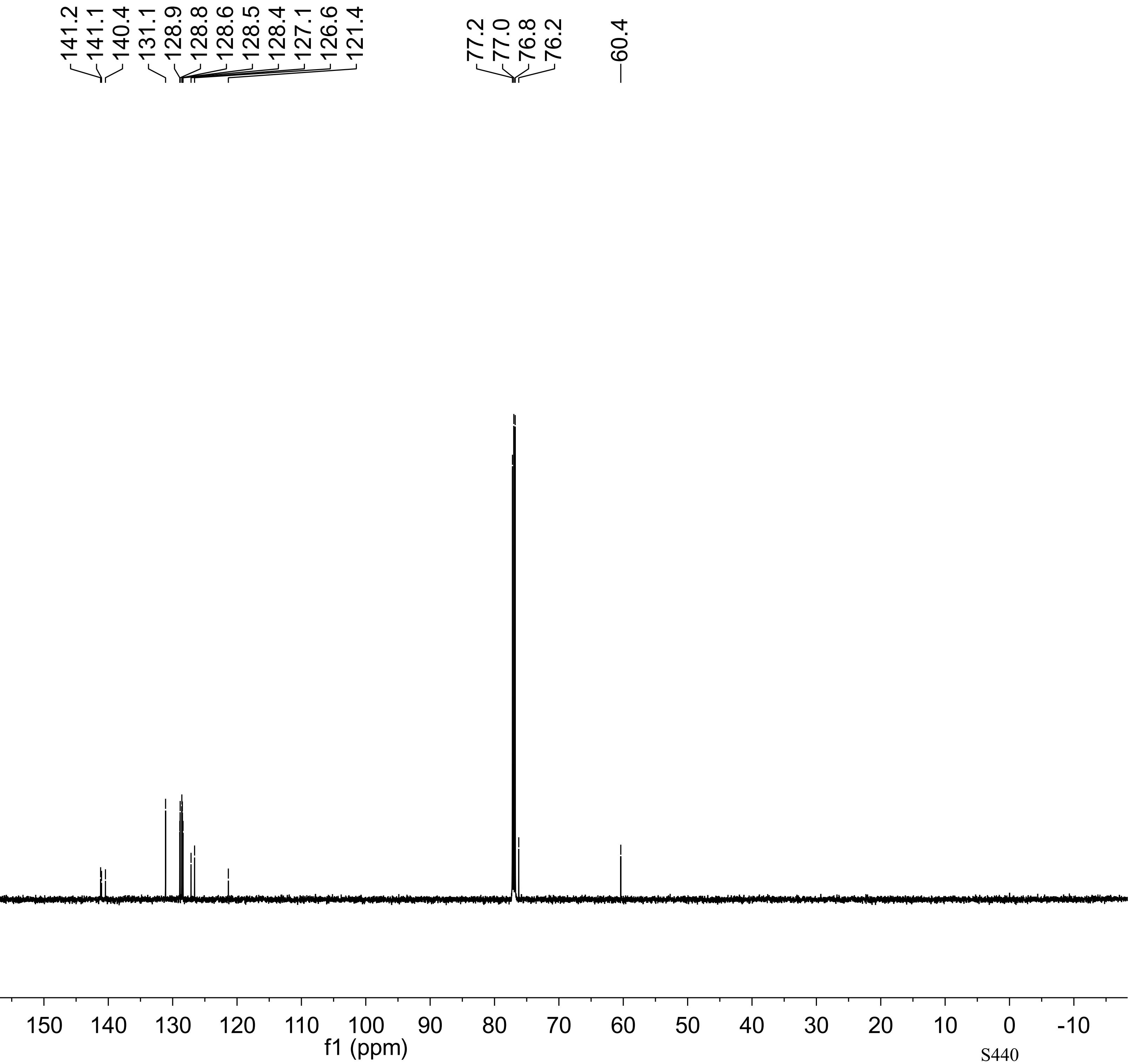

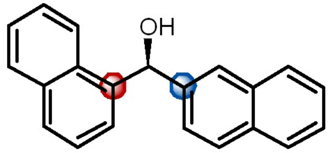

C34

Parameter	Value
1 Title	CFM-C34-0627
2 Comment	
3 Origin	Bruker BioSpin GmbH
4 Owner	nmrsu
5 Site	
6 Spectrometer	AVANCE NEO 400 MHZ DIGITAL NMR SPECTROMETER
7 Author	
8 Solvent	CDCl3
9 Temperature	298.1
10 Pulse Sequence	zg30
11 Experiment	1D
12 Number of Scans	8
13 Receiver Gain	101
14 Spectrometer Frequency	400.13
15 Spectral Width	8196.7
16 Lowest Frequency	-1637.2
17 Nucleus	1H
18 Acquired Size	32768
19 Spectral Size	65536

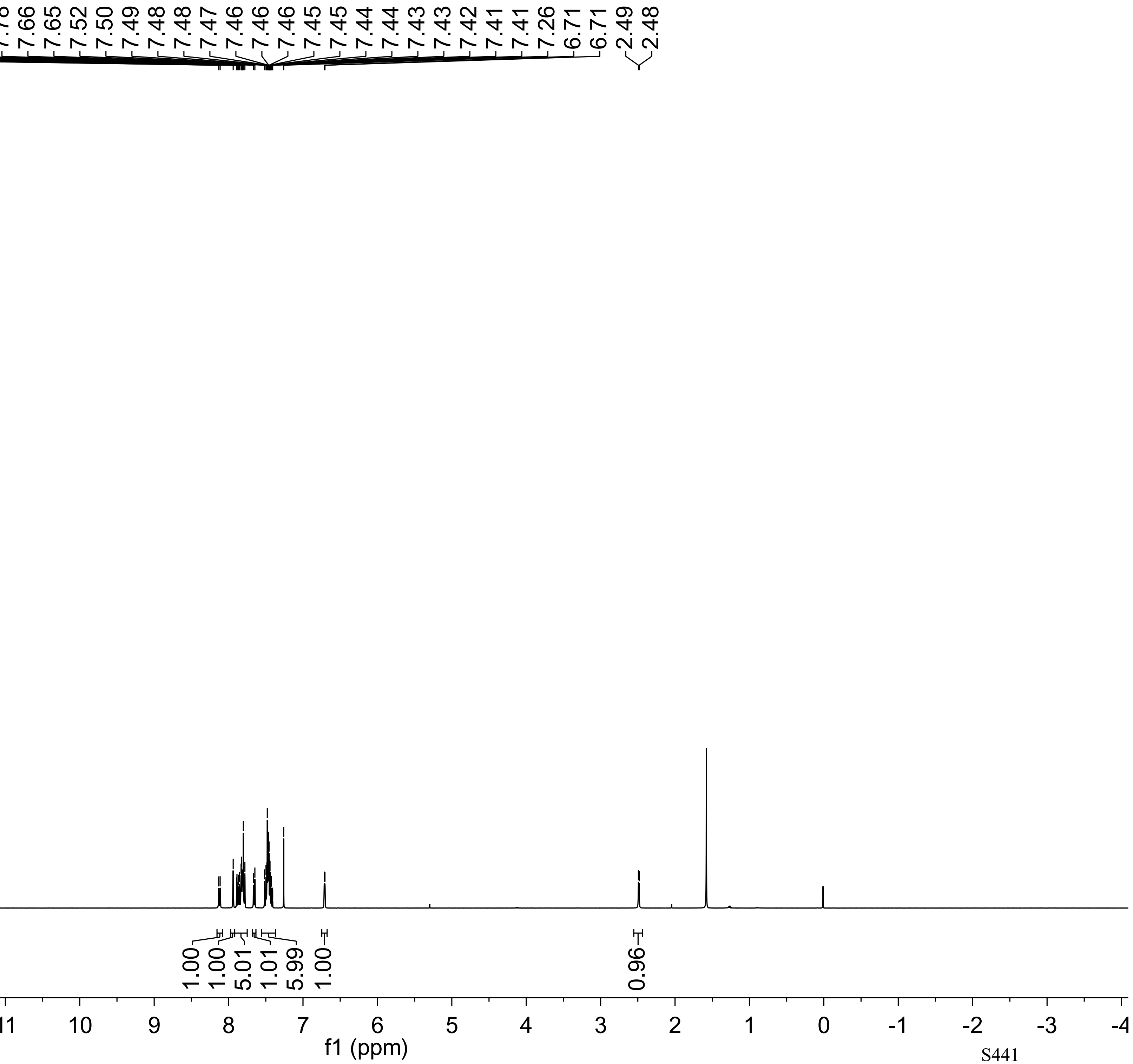

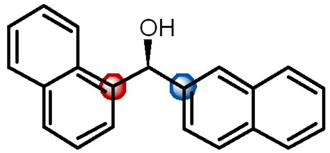

C34

Parameter	Value
1 Title	CFM-C34-0627
2 Comment	
3 Origin	Bruker BioSpin GmbH
4 Owner	nmrsu
5 Site	
6 Spectrometer	AVANCE NEO 400 MHZ DIGITAL NMR SPECTROMETER
7 Author	
8 Solvent	CDCl3
9 Temperature	298.2
10 Pulse Sequence	zgpg30
11 Experiment	1D
12 Number of Scans	256
13 Receiver Gain	59
14 Spectrometer Frequency	100.61
15 Spectral Width	23809.5
16 Lowest Frequency	-1847.7
17 Nucleus	13C
18 Acquired Size	32768
19 Spectral Size	32768

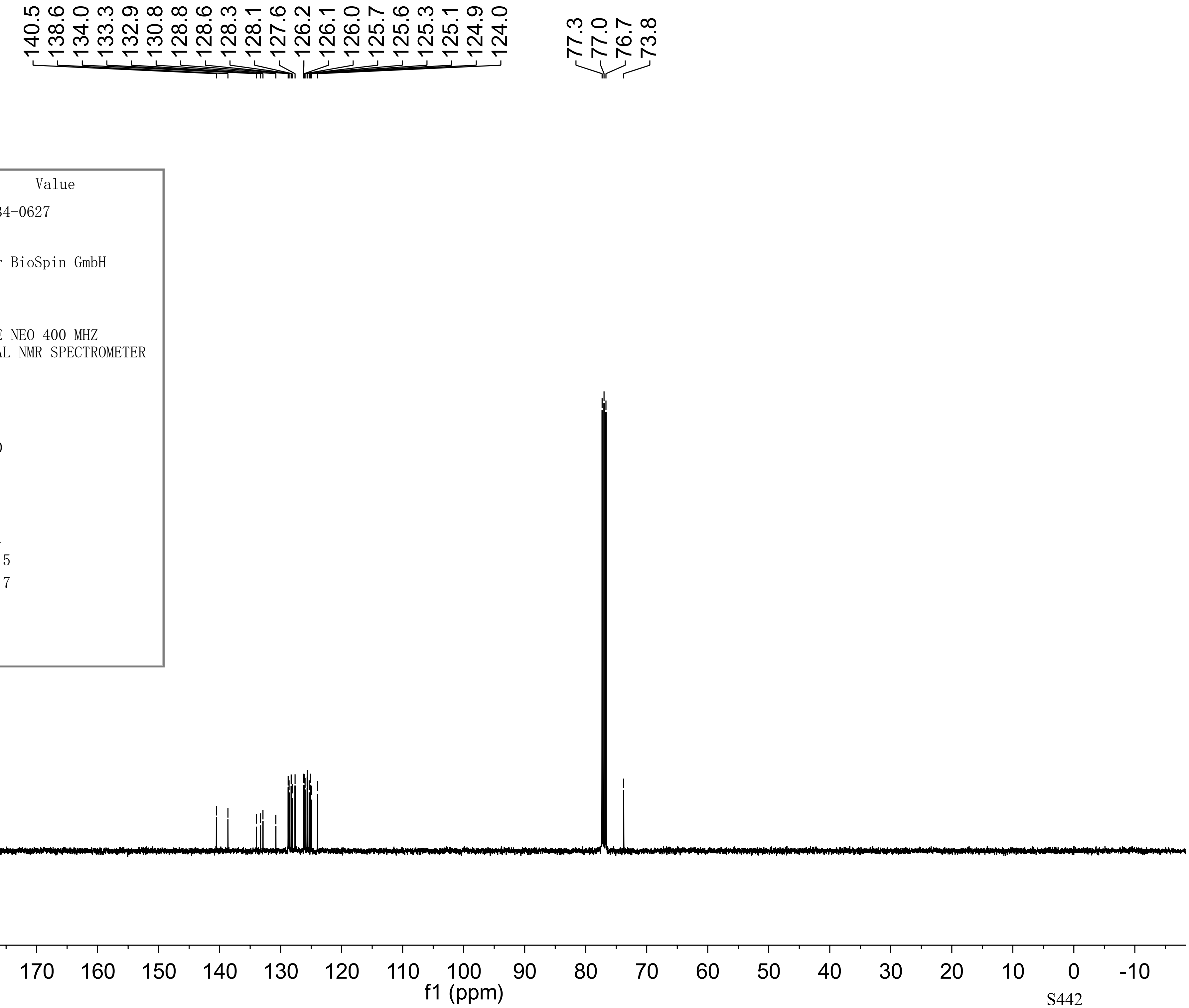

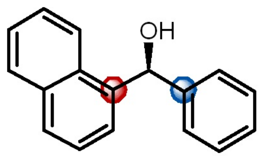

C35

Parameter	Value
1 Title	R-CFM-G8
2 Comment	
3 Origin	Bruker BioSpin GmbH
4 Owner	nmrsu
5 Site	
6 Spectrometer	Avance NEO 600
7 Author	
8 Solvent	CDCl3
9 Temperature	296.7
10 Pulse Sequence	zg30
11 Experiment	1D
12 Number of Scans	16
13 Receiver Gain	100
14 Relaxation Delay	1.0000
15 Pulse Width	10.0000
16 Acquisition Time	2.7525
17 Acquisition Date	2019-12-14T06:30:37
18 Modification Date	2019-12-14T09:18:15
19 Spectrometer Frequency	600.15
20 Spectral Width	11904.8
21 Lowest Frequency	-2260.5
22 Nucleus	1H
23 Acquired Size	32768
24 Spectral Size	65536

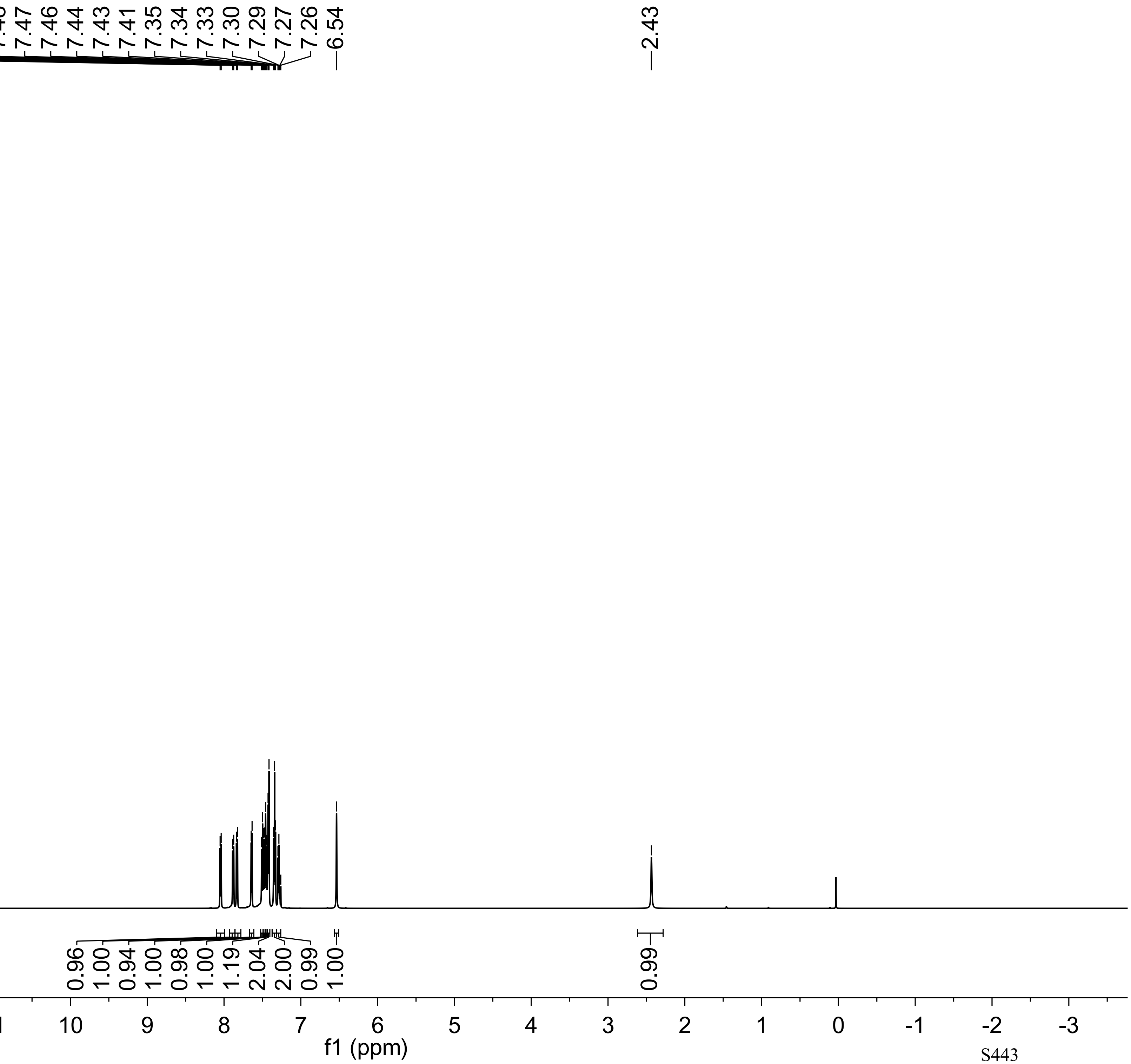

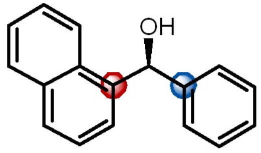

C35

Parameter		Value
1	Title	R-CFM-G8
2	Comment	
3	Origin	Bruker BioSpin GmbH
4	Owner	nmrsu
5	Site	
6	Spectrometer	Avance NEO 600
7	Author	
8	Solvent	CDCl3
9	Temperature	298.1
10	Pulse Sequence	zgpg30
11	Experiment	1D
12	Number of Scans	256
13	Receiver Gain	101
14	Relaxation Delay	2.0000
15	Pulse Width	12.0000
16	Acquisition Time	0.9175
17	Acquisition Date	2019-12-14T06:44:45
18	Modification Date	2019-12-14T09:18:15
19	Spectrometer Frequency	150.91
20	Spectral Width	35714.3
21	Lowest Frequency	-2776.9
22	Nucleus	13C
23	Acquired Size	32768
24	Spectral Size	32768

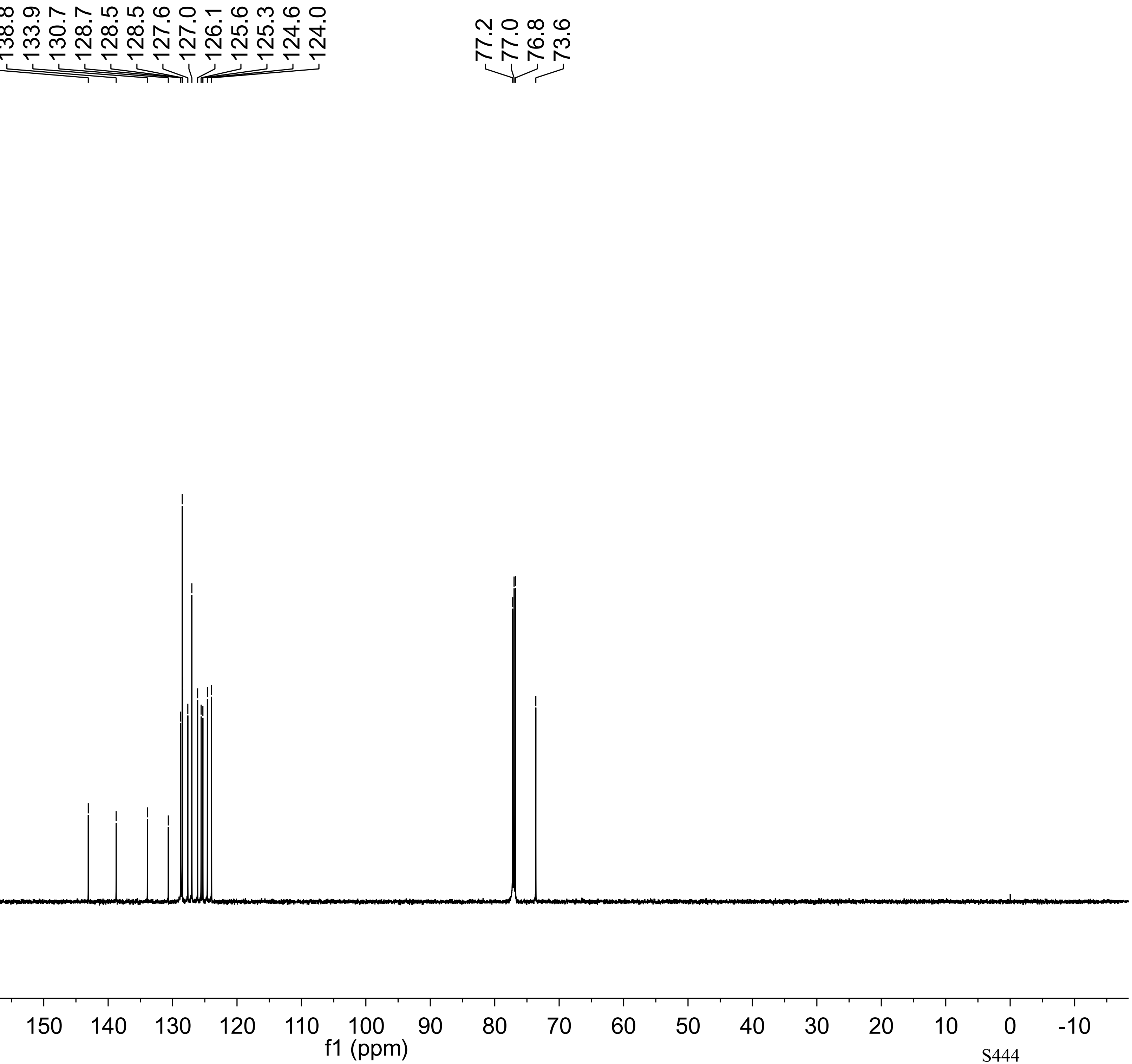

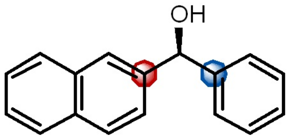

C36

Parameter	Value
1 Title	R-CFM-G9-H
2 Comment	
3 Origin	Bruker BioSpin GmbH
4 Owner	nmrsu
5 Site	
6 Spectrometer	Avance NEO 600
7 Author	
8 Solvent	CDCl3
9 Temperature	296.1
10 Pulse Sequence	zg30
11 Experiment	1D
12 Number of Scans	8
13 Receiver Gain	101
14 Relaxation Delay	1.0000
15 Pulse Width	10.0000
16 Acquisition Time	2.7525
17 Acquisition Date	2020-04-17T20:50:12
18 Modification Date	2020-04-17T20:56:44
19 Spectrometer Frequency	600.15
20 Spectral Width	11904.8
21 Lowest Frequency	-2261.2
22 Nucleus	1H
23 Acquired Size	32768
24 Spectral Size	65536

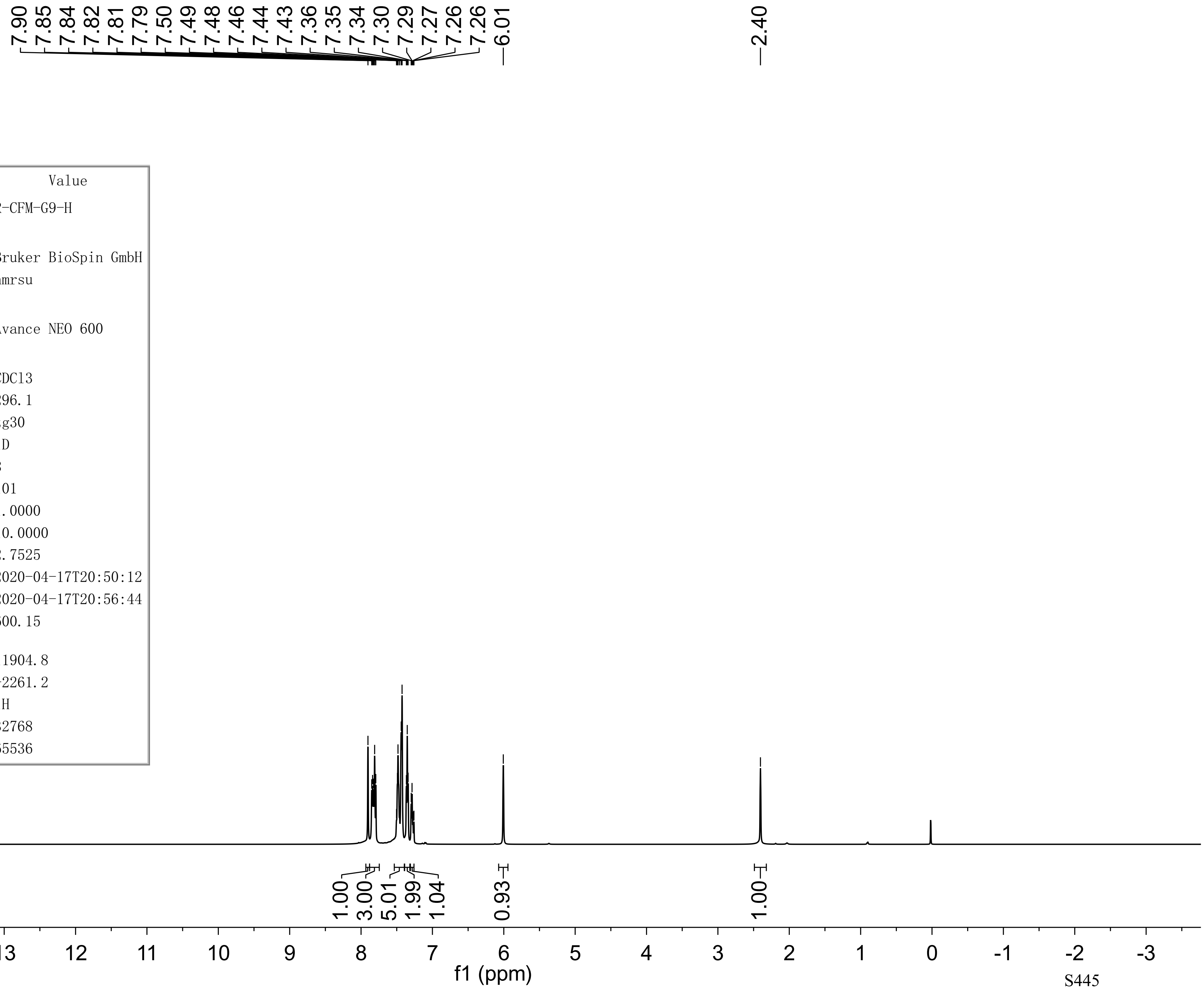

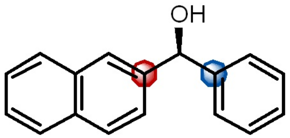

C36

Parameter	Value
1 Title	R-CFM-G9
2 Comment	
3 Origin	Bruker BioSpin GmbH
4 Owner	nmrsu
5 Site	
6 Spectrometer	Avance NEO 600
7 Author	
8 Solvent	CDCl3
9 Temperature	297.2
10 Pulse Sequence	zgpg30
11 Experiment	1D
12 Number of Scans	64
13 Receiver Gain	101
14 Relaxation Delay	2.0000
15 Pulse Width	12.0000
16 Acquisition Time	0.9175
17 Acquisition Date	2020-04-17T19:36:08
18 Modification Date	2020-04-17T19:39:14
19 Spectrometer Frequency	150.91
20 Spectral Width	35714.3
21 Lowest Frequency	-2774.5
22 Nucleus	13C
23 Acquired Size	32768
24 Spectral Size	32768

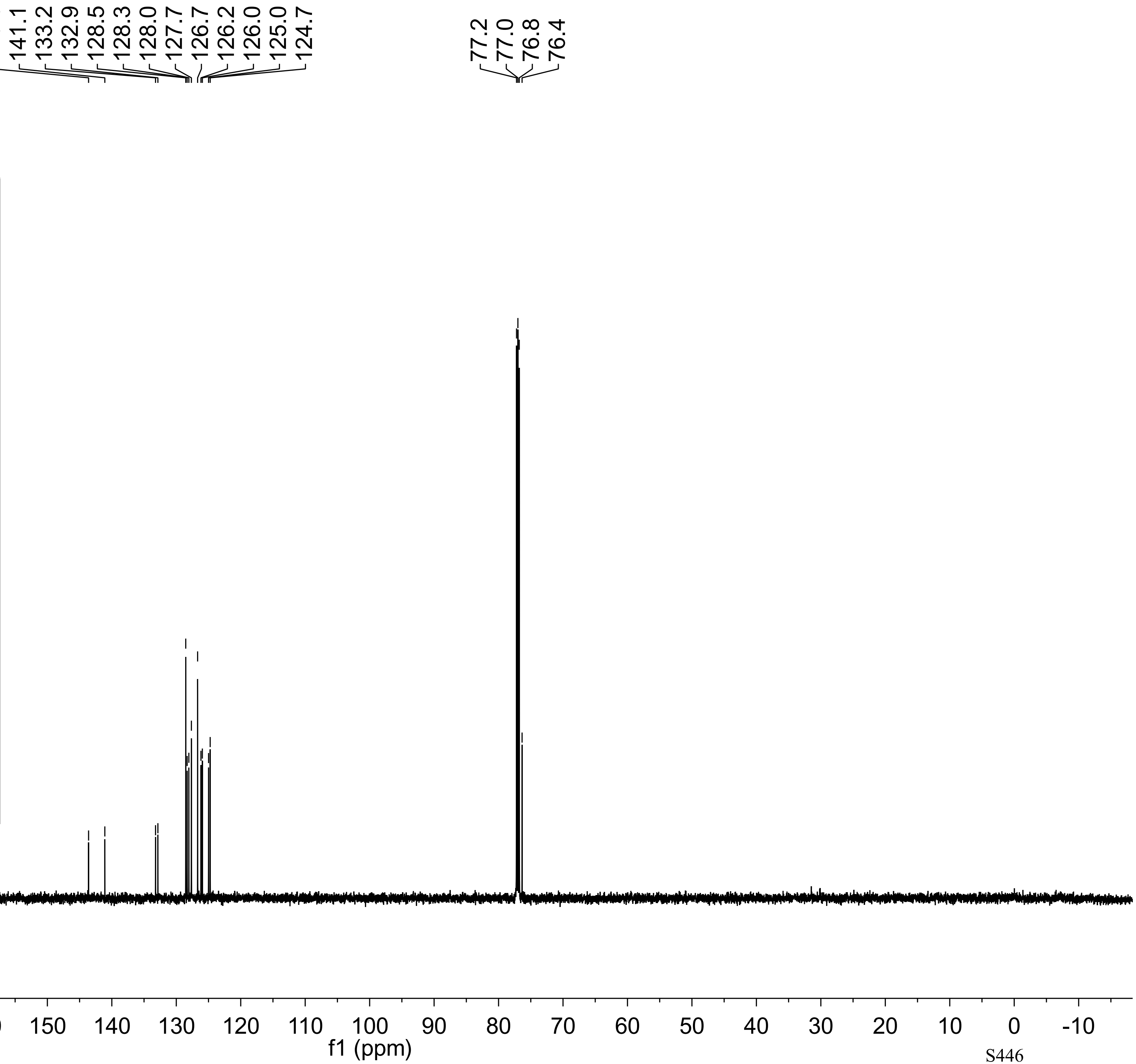

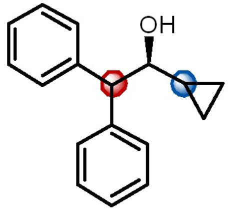

C37

	Parameter	Value
1	Title	R-CFM-G12-1
2	Comment	
3	Origin	Bruker BioSpin GmbH
4	Owner	nmrsu
5	Site	
6	Spectrometer	Avance NEO 600
7	Author	
8	Solvent	CDCl3
9	Temperature	296.2
10	Pulse Sequence	zg30
11	Experiment	1D
12	Number of Scans	8
13	Receiver Gain	85
14	Relaxation Delay	1.0000
15	Pulse Width	10.0000
16	Acquisition Time	2.7525
17	Acquisition Date	2020-04-16T22:11:23
18	Modification Date	2020-04-16T22:16:24
19	Spectrometer Frequency	600.15
20	Spectral Width	11904.8
21	Lowest Frequency	-2339.3
22	Nucleus	1H
23	Acquired Size	32768
24	Spectral Size	65536

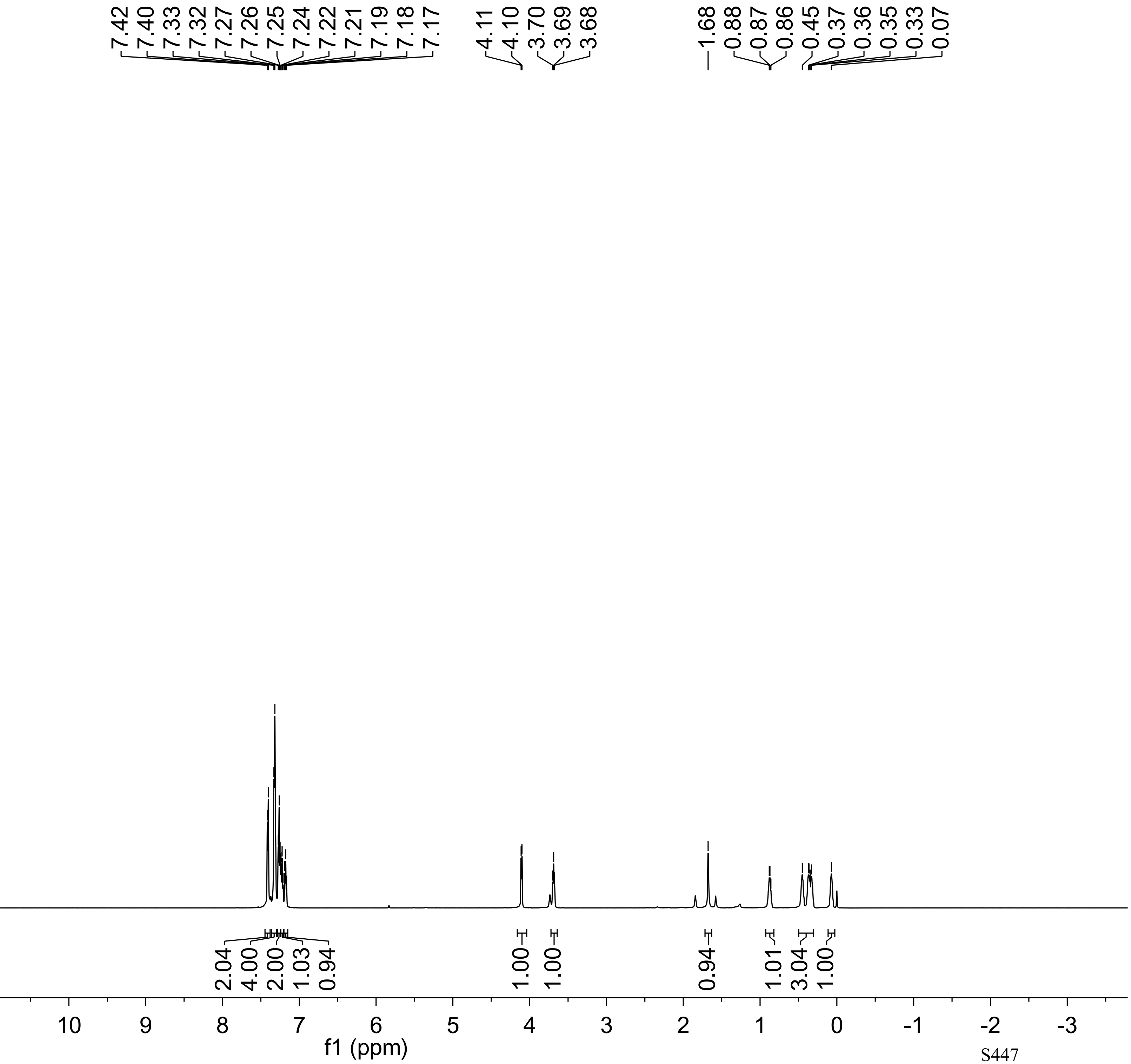

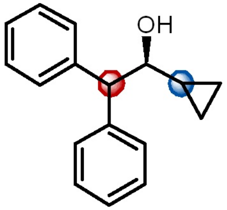

C37

Parameter	Value
1 Title	R-CFM-G12-1
2 Comment	
3 Origin	Bruker BioSpin GmbH
4 Owner	nmrsu
5 Site	
6 Spectrometer	Avance NEO 600
7 Author	
8 Solvent	CDCl3
9 Temperature	297.0
10 Pulse Sequence	zgpg30
11 Experiment	1D
12 Number of Scans	28
13 Receiver Gain	101
14 Relaxation Delay	2.0000
15 Pulse Width	12.0000
16 Acquisition Time	0.9175
17 Acquisition Date	2020-04-16T22:14:04
18 Modification Date	2020-04-16T22:16:24
19 Spectrometer Frequency	150.91
20 Spectral Width	35714.3
21 Lowest Frequency	-2777.3
22 Nucleus	13C
23 Acquired Size	32768
24 Spectral Size	32768

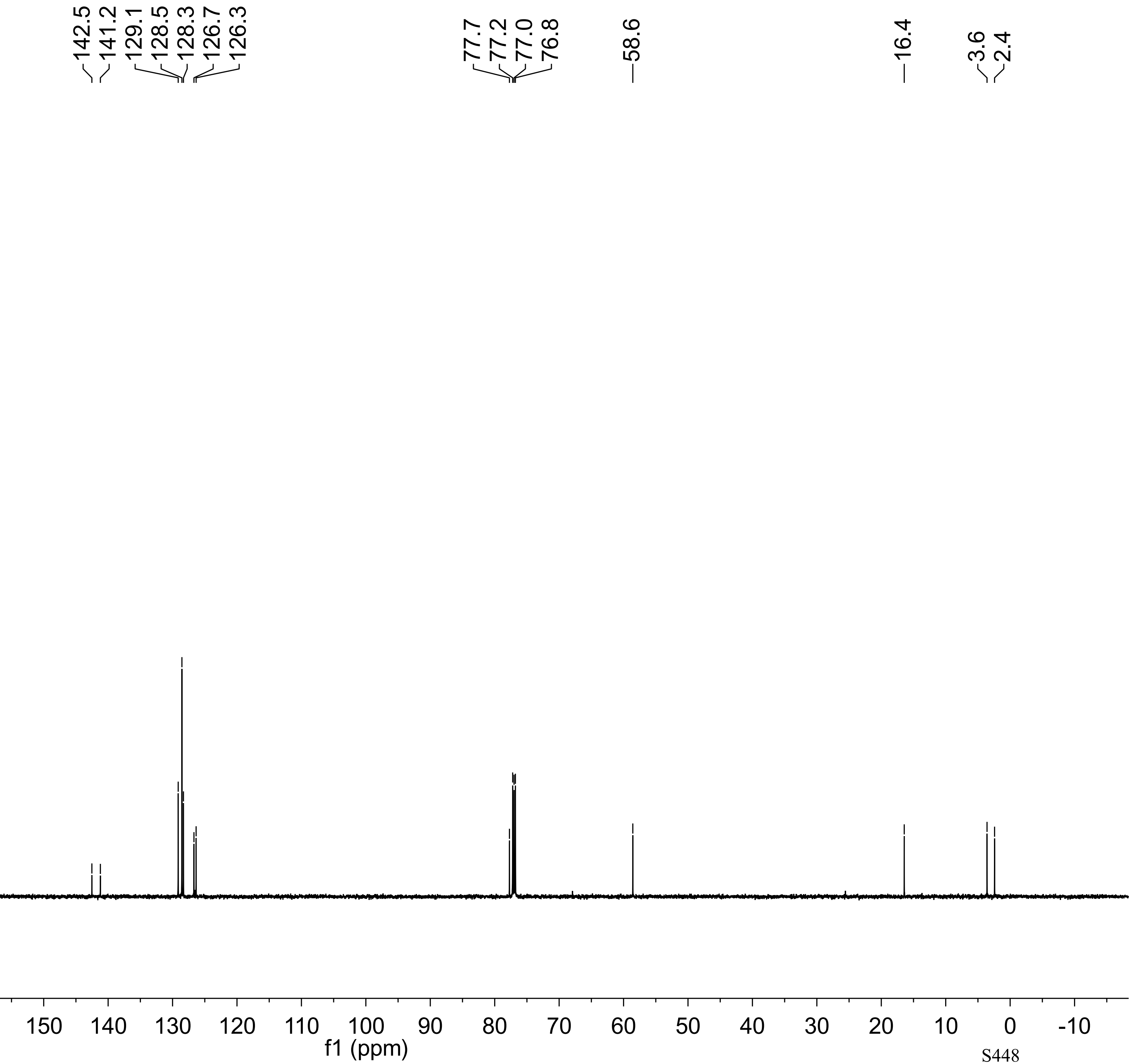

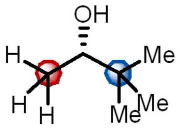

C38

	Parameter	Value
1	Title	CFM-6-53-2
2	Comment	
3	Origin	Bruker BioSpin GmbH
4	Owner	nmrsu
5	Site	
6	Spectrometer	Avance Neo 400M
7	Author	
8	Solvent	CDCl3
9	Temperature	298.2
10	Pulse Sequence	zg30
11	Experiment	1D
12	Number of Scans	6
13	Receiver Gain	101
14	Spectrometer Frequency	400.18
15	Spectral Width	8196.7
16	Lowest Frequency	-1636.9
17	Nucleus	1H
18	Acquired Size	32768
19	Spectral Size	65536

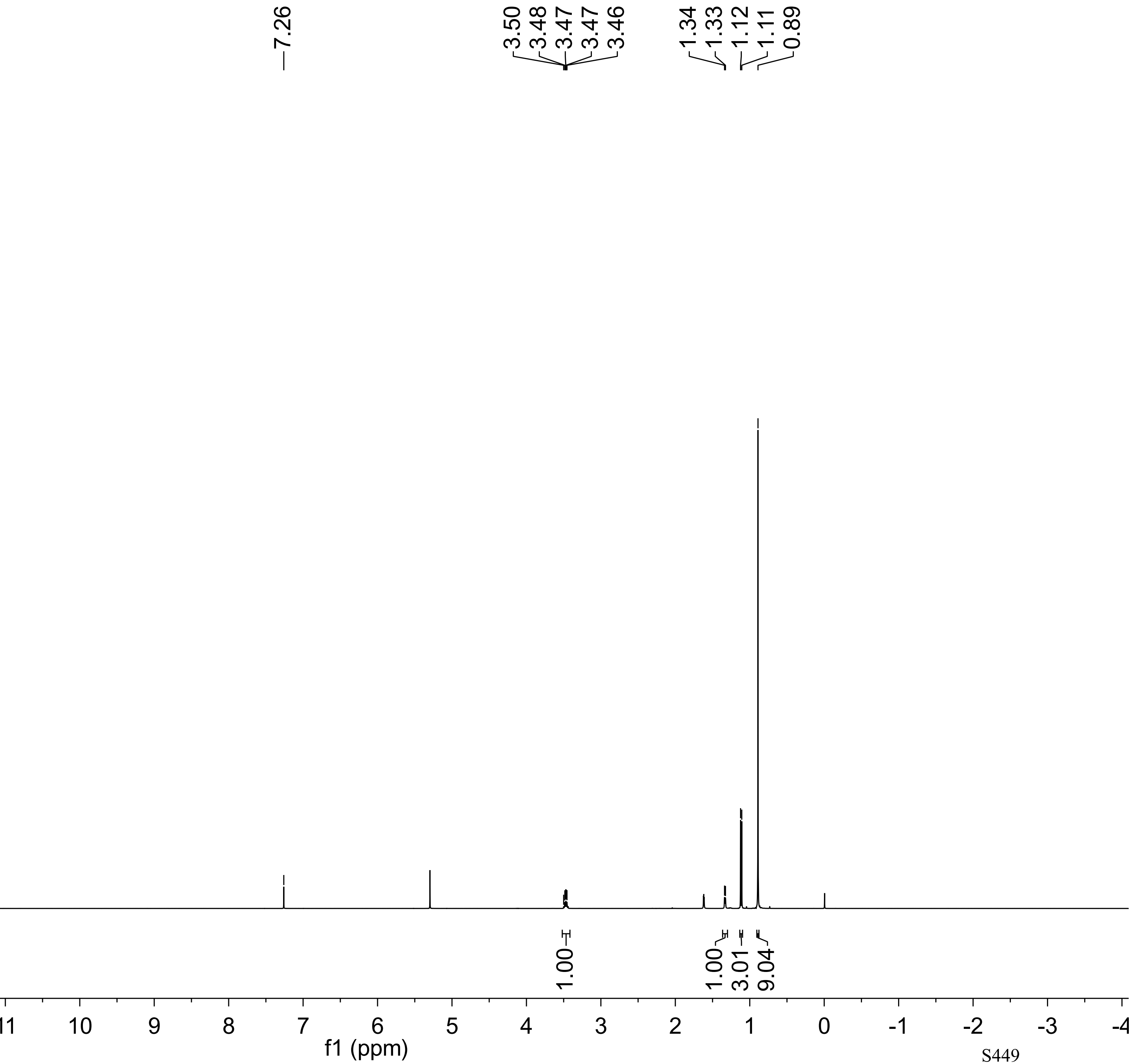

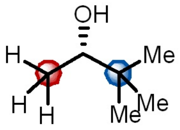

C38

	Parameter	Value
1	Title	CFM-6-52
2	Comment	
3	Origin	Bruker BioSpin GmbH
4	Owner	nmrsu
5	Site	
6	Spectrometer	Avance NEO 600
7	Author	
8	Solvent	CDCl3
9	Temperature	298.1
10	Pulse Sequence	zgpg30
11	Experiment	1D
12	Number of Scans	64
13	Receiver Gain	101
14	Spectrometer Frequency	150.91
15	Spectral Width	35714.3
16	Lowest Frequency	-2770.1
17	Nucleus	13C
18	Acquired Size	32768
19	Spectral Size	32768

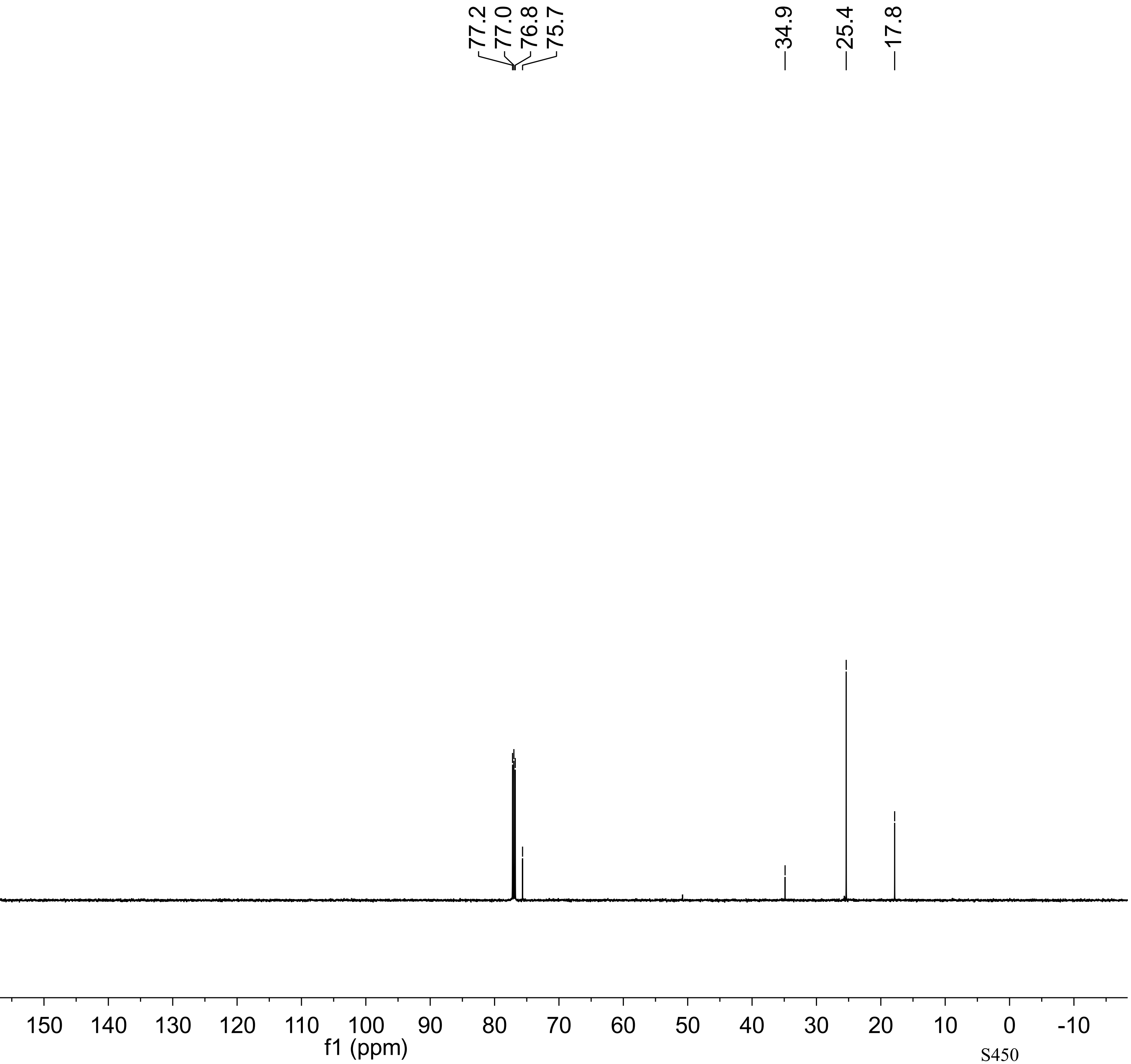

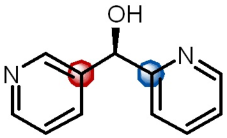

C39

Parameter		Value
1	Title	R-CFM-G14
2	Comment	
3	Origin	Bruker BioSpin GmbH
4	Owner	nmrsu
5	Site	
6	Spectrometer	Avance NEO 600
7	Author	
8	Solvent	CDCl3
9	Temperature	301.8
10	Pulse Sequence	zg30
11	Experiment	1D
12	Number of Scans	16
13	Receiver Gain	101
14	Relaxation Delay	1.0000
15	Pulse Width	10.0000
16	Acquisition Time	2.7525
17	Acquisition Date	2019-12-30T07:12:47
18	Modification Date	2019-12-30T08:57:48
19	Spectrometer Frequency	600.15
20	Spectral Width	11904.8
21	Lowest Frequency	-2260.9
22	Nucleus	1H
23	Acquired Size	32768
24	Spectral Size	65536

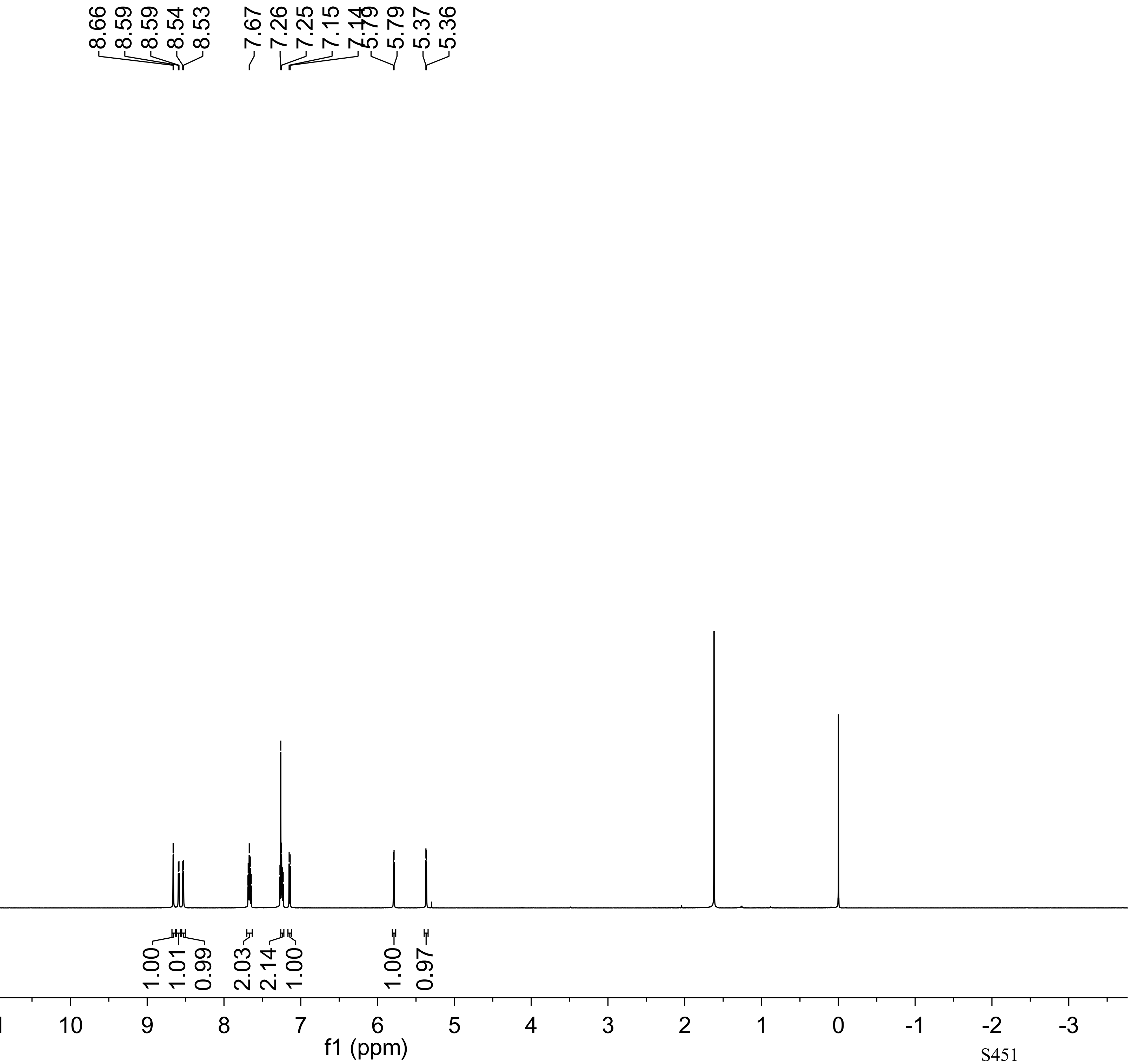

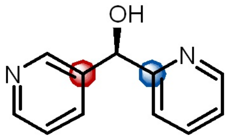

C39

Parameter	Value
1 Title	R-CFM-G14
2 Comment	
3 Origin	Bruker BioSpin GmbH
4 Owner	nmrsu
5 Site	
6 Spectrometer	Avance NEO 600
7 Author	
8 Solvent	CDCl3
9 Temperature	300.4
10 Pulse Sequence	zgpg30
11 Experiment	1D
12 Number of Scans	512
13 Receiver Gain	101
14 Relaxation Delay	2.0000
15 Pulse Width	12.0000
16 Acquisition Time	0.9175
17 Acquisition Date	2019-12-30T07:39:39
18 Modification Date	2019-12-30T08:57:48
19 Spectrometer Frequency	150.91
20 Spectral Width	35714.3
21 Lowest Frequency	-2768.3
22 Nucleus	13C
23 Acquired Size	32768
24 Spectral Size	32768

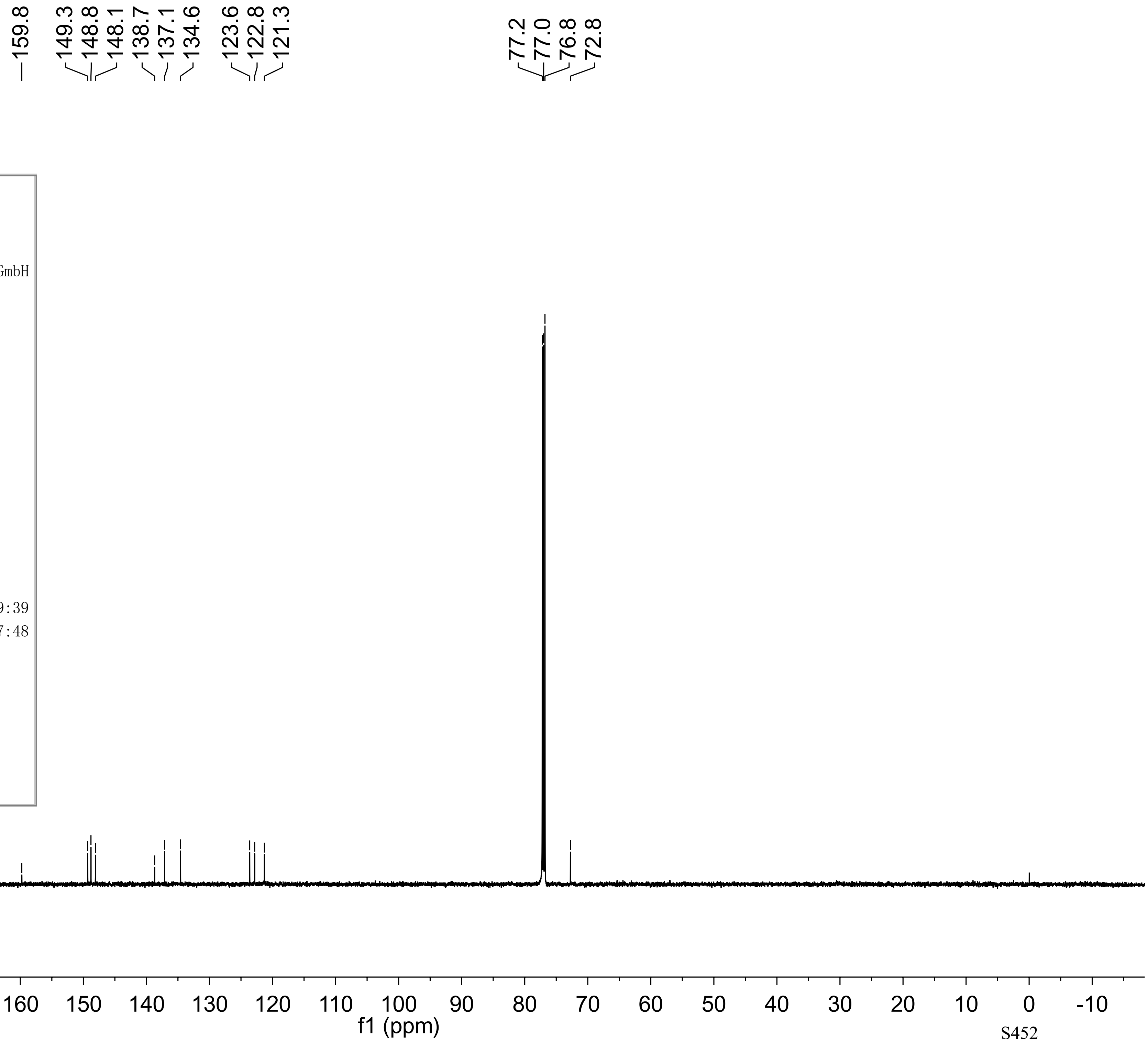

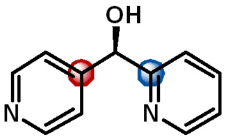

C40

Parameter	Value
1 Title	CFM-6-76-1-1
2 Comment	
3 Origin	Bruker BioSpin GmbH
4 Owner	nmrsu
5 Site	
6 Spectrometer	Avance Neo 400M
7 Author	
8 Solvent	CDCl3
9 Temperature	298.1
10 Pulse Sequence	zg30
11 Experiment	1D
12 Number of Scans	16
13 Receiver Gain	101
14 Spectrometer Frequency	400.18
15 Spectral Width	8196.7
16 Lowest Frequency	-1636.9
17 Nucleus	1H
18 Acquired Size	32768
19 Spectral Size	65536

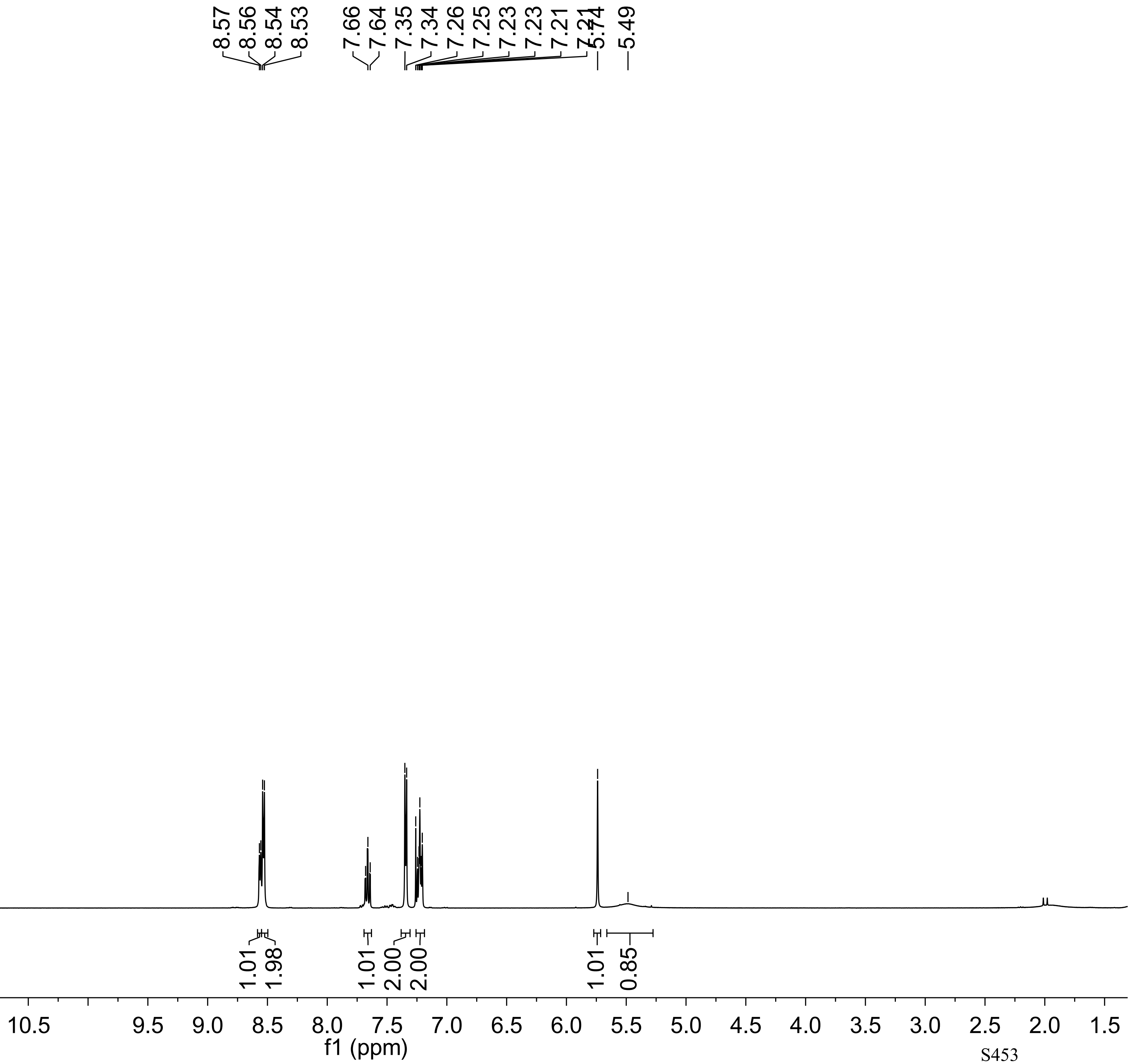

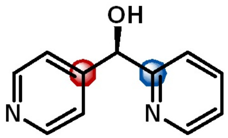

C40

	Parameter	Value
1	Title	CFM-6-76-1-1
2	Comment	
3	Origin	Bruker BioSpin GmbH
4	Owner	nmrsu
5	Site	
6	Spectrometer	Avance Neo 400M
7	Author	
8	Solvent	CDCl3
9	Temperature	298.3
10	Pulse Sequence	zgpg30
11	Experiment	1D
12	Number of Scans	107
13	Receiver Gain	33
14	Spectrometer Frequency	100.63
15	Spectral Width	23809.5
16	Lowest Frequency	-1846.5
17	Nucleus	13C
18	Acquired Size	32768
19	Spectral Size	32768

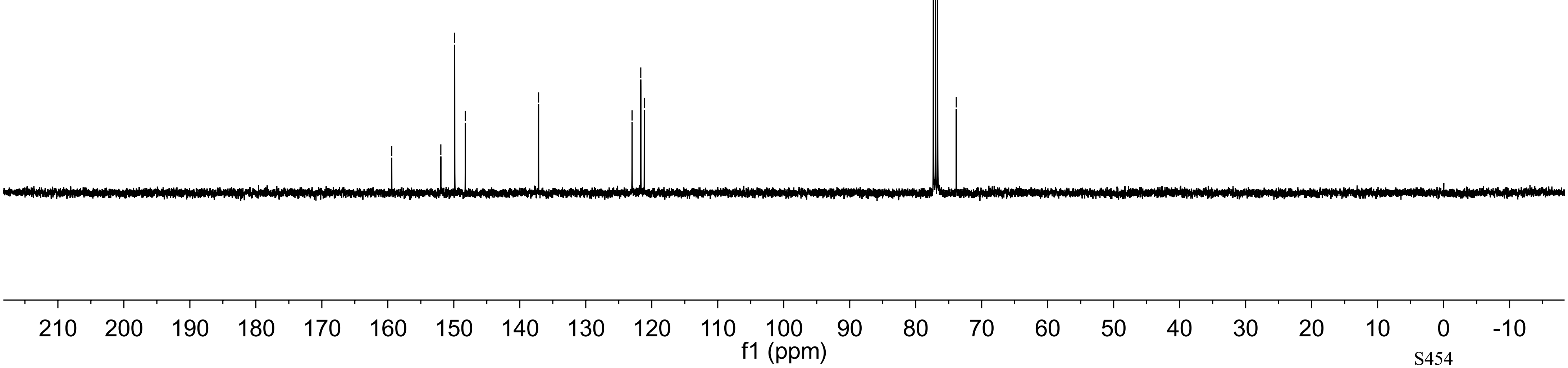

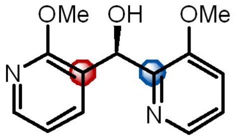

C41

Parameter		Value
1	Title	CFM-C38-0627
2	Comment	
3	Origin	Bruker BioSpin GmbH
4	Owner	nmrsu
5	Site	
6	Spectrometer	AVANCE NEO 400 MHZ DIGITAL NMR SPECTROMETER
7	Author	
8	Solvent	CDCl3
9	Temperature	298.2
10	Pulse Sequence	zg30
11	Experiment	1D
12	Number of Scans	8
13	Receiver Gain	101
14	Spectrometer Frequency	400.13
15	Spectral Width	8196.7
16	Lowest Frequency	-1621.3
17	Nucleus	1H
18	Acquired Size	32768
19	Spectral Size	65536

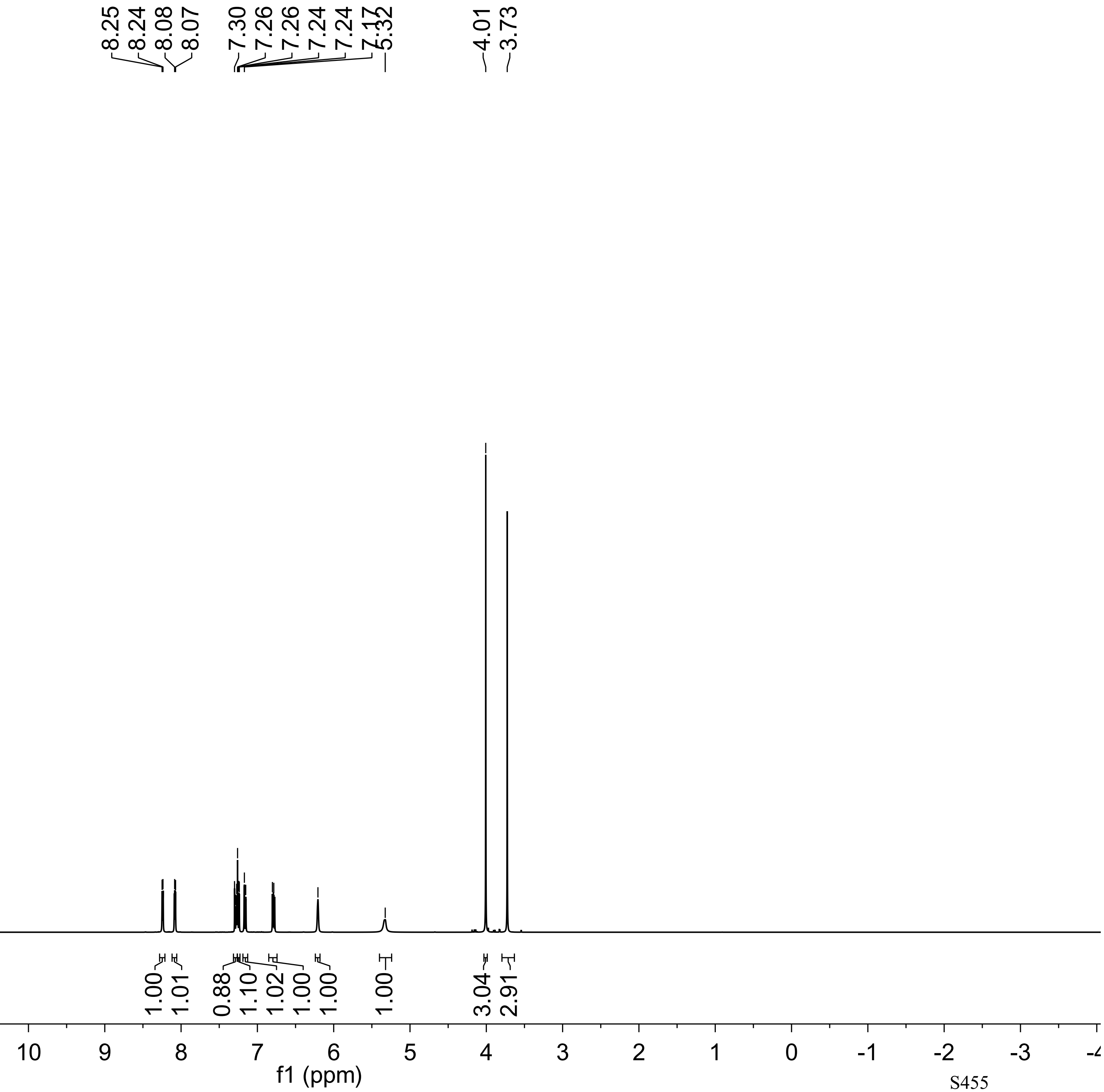

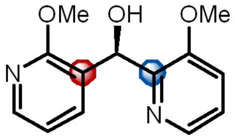

C41

	Parameter	Value
1	Title	CFM-C38-0627
2	Comment	
3	Origin	Bruker BioSpin GmbH
4	Owner	nmrsu
5	Site	
6	Spectrometer	AVANCE NEO 400 MHZ DIGITAL NMR SPECTROMETER
7	Author	
8	Solvent	CDCl3
9	Temperature	298.2
10	Pulse Sequence	zgpg30
11	Experiment	1D
12	Number of Scans	256
13	Receiver Gain	59
14	Spectrometer Frequency	100.61
15	Spectral Width	23809.5
16	Lowest Frequency	-1848.8
17	Nucleus	13C
18	Acquired Size	32768
19	Spectral Size	32768

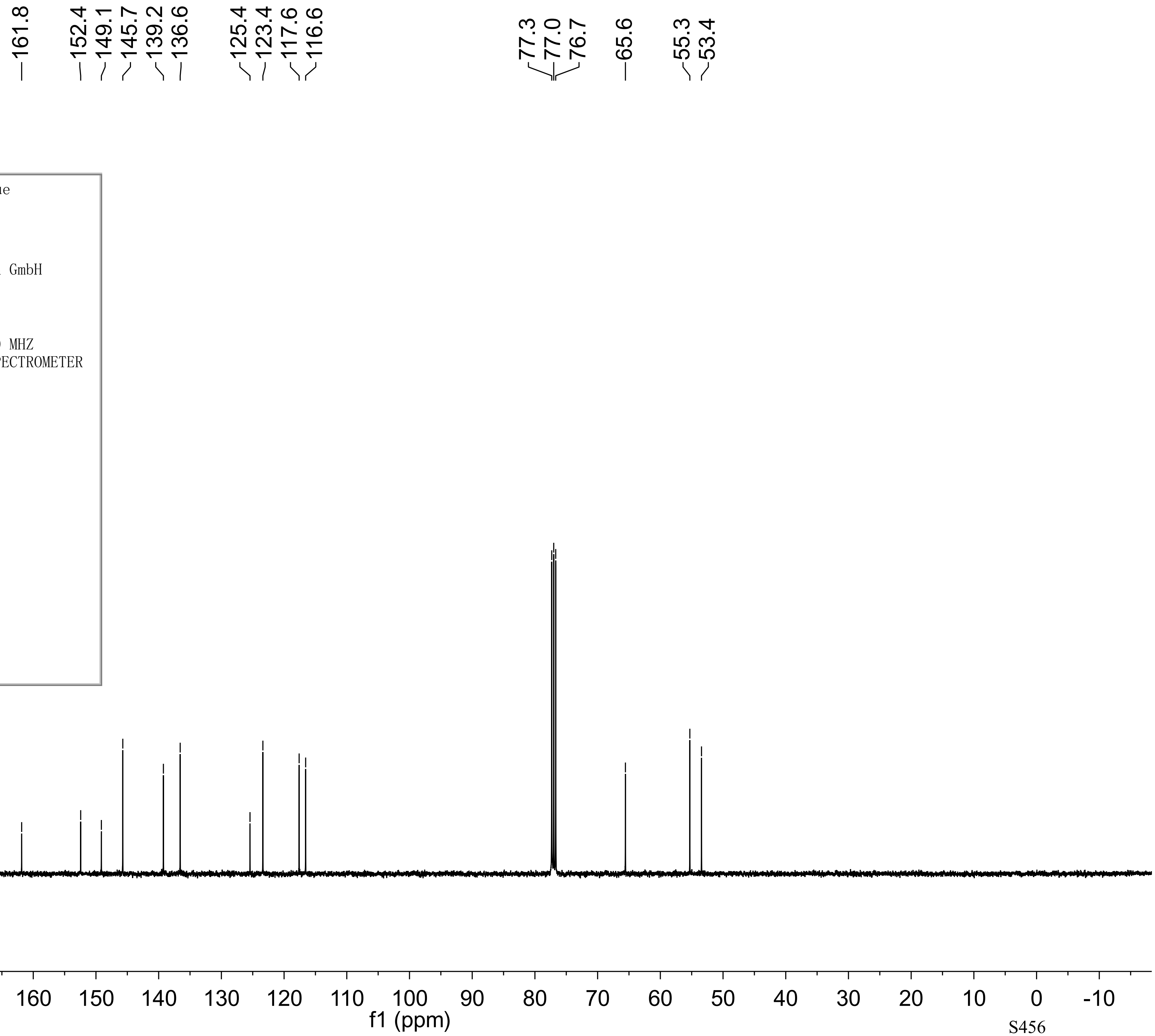

CFM-7-76-2

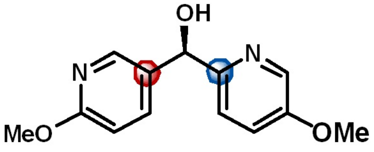

C42

Parameter	Value
1 Title	CFM-7-76-2
2 Comment	
3 Origin	Bruker BioSpin GmbH
4 Owner	nmrsu
5 Site	
6 Spectrometer	AVANCE NEO 400 MHZ DIGITAL NMR SPECTROMETER
7 Author	
8 Solvent	CDCl3
9 Temperature	298.1
10 Pulse Sequence	zg30
11 Experiment	1D
12 Number of Scans	8
13 Receiver Gain	101
14 Spectrometer Frequency	400.13
15 Spectral Width	8196.7
16 Lowest Frequency	-1636.8
17 Nucleus	1H
18 Acquired Size	32768
19 Spectral Size	65536

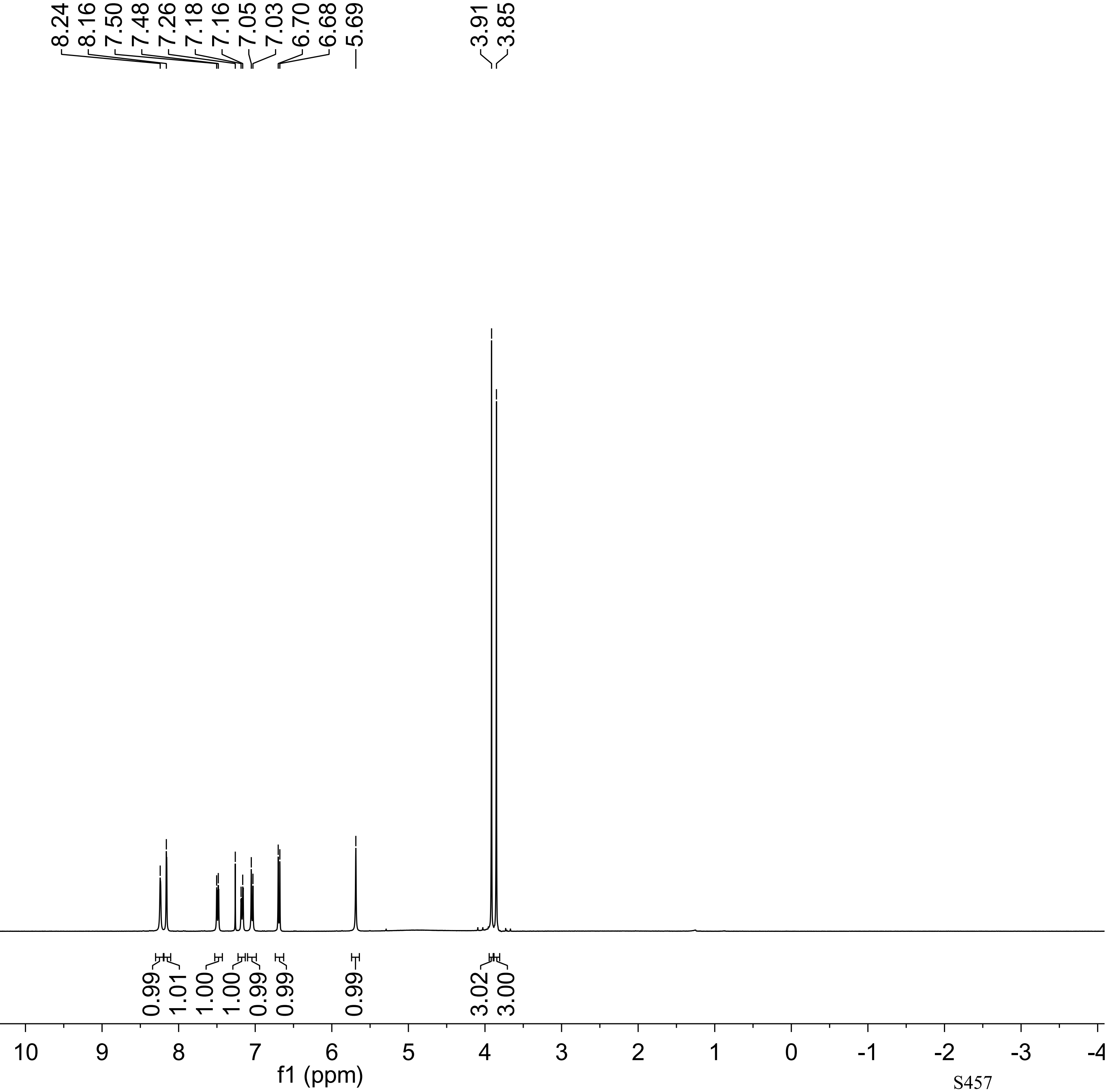

CFM-7-76-2

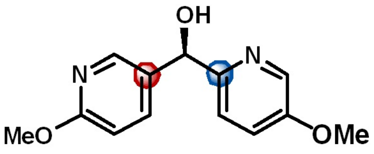

C42

Parameter	Value
1 Title	CFM-7-76-2
2 Comment	
3 Origin	Bruker BioSpin GmbH
4 Owner	nmrsu
5 Site	
6 Spectrometer	AVANCE NEO 400 MHZ DIGITAL NMR SPECTROMETER
7 Author	
8 Solvent	CDCl3
9 Temperature	298.2
10 Pulse Sequence	zgpg30
11 Experiment	1D
12 Number of Scans	512
13 Receiver Gain	64
14 Spectrometer Frequency	100.61
15 Spectral Width	23809.5
16 Lowest Frequency	-1847.3
17 Nucleus	13C
18 Acquired Size	32768
19 Spectral Size	32768

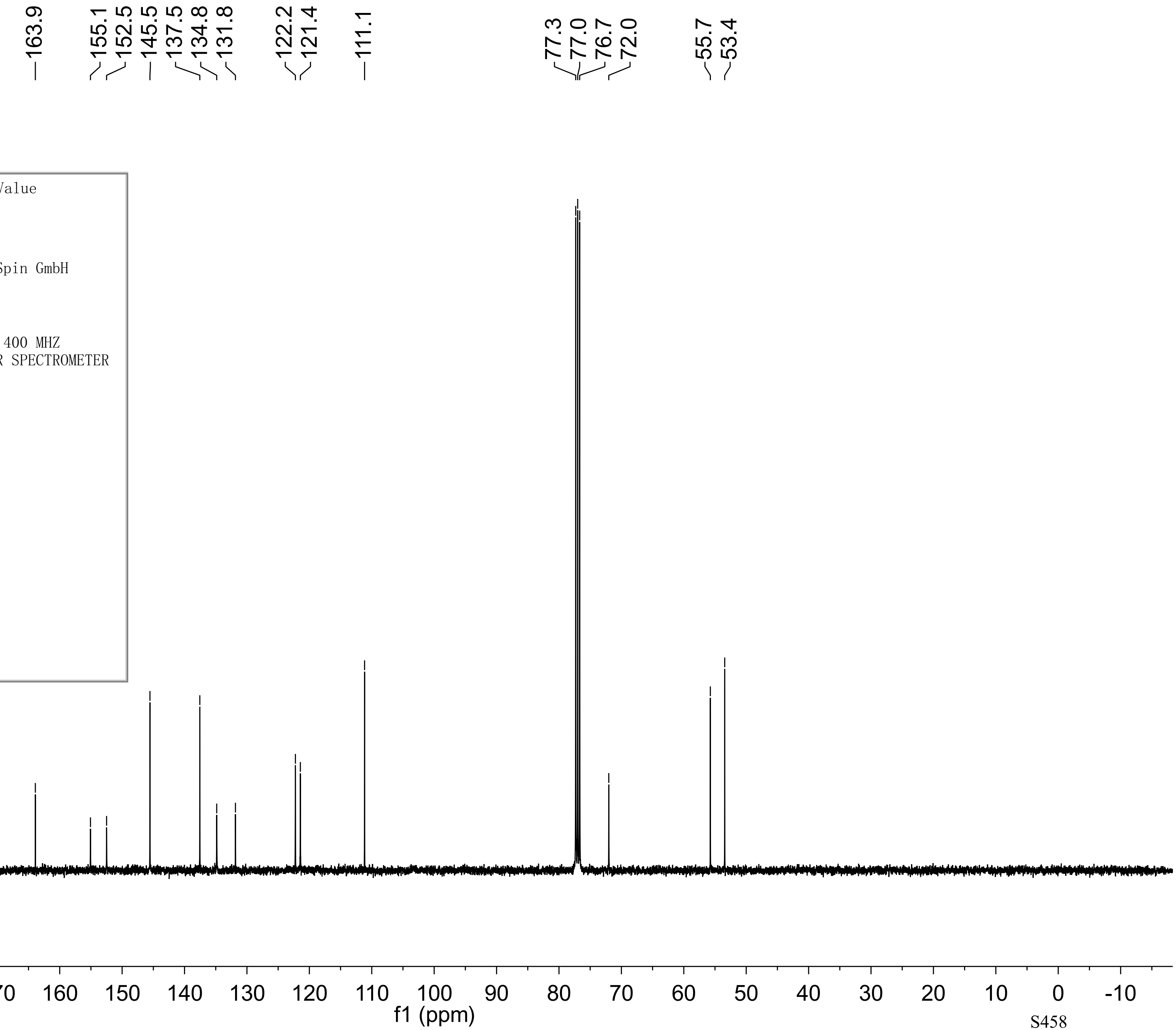

CFM-7-76-1-H

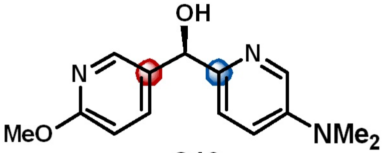

C43

	Parameter	Value
1	Title	CFM-7-76-1-H
2	Comment	
3	Origin	Bruker BioSpin GmbH
4	Owner	nmrsu
5	Site	
6	Spectrometer	AVANCE NEO 400 MHZ DIGITAL NMR SPECTROMETER
7	Author	
8	Solvent	CDCl3
9	Temperature	298.1
10	Pulse Sequence	zg30
11	Experiment	1D
12	Number of Scans	8
13	Receiver Gain	101
14	Spectrometer Frequency	400.13
15	Spectral Width	8196.7
16	Lowest Frequency	-1636.9
17	Nucleus	1H
18	Acquired Size	32768
19	Spectral Size	65536

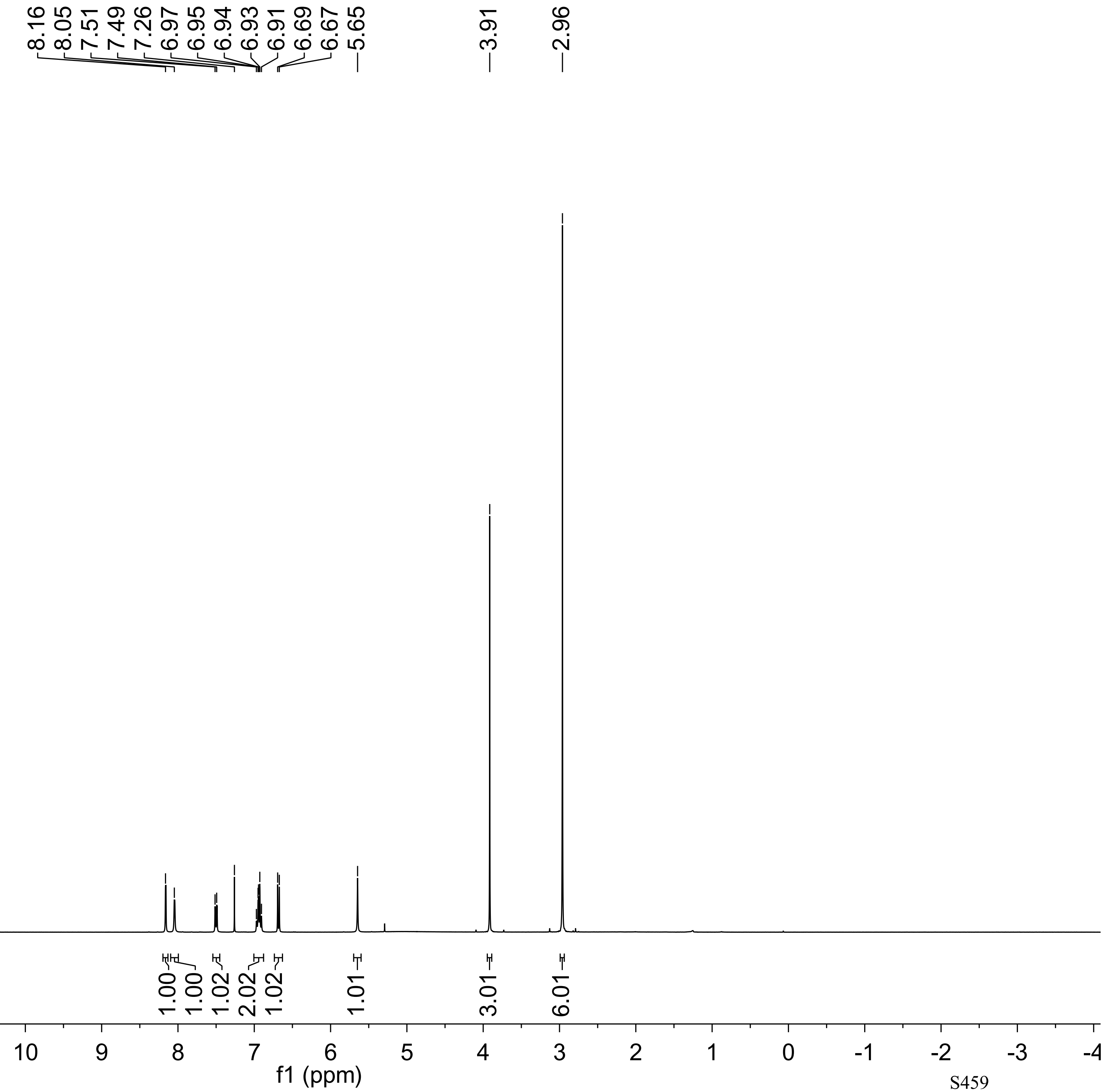

CFM-7-76-1-C

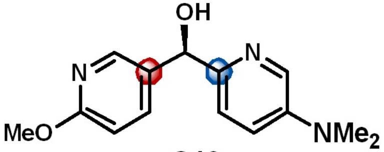

C43

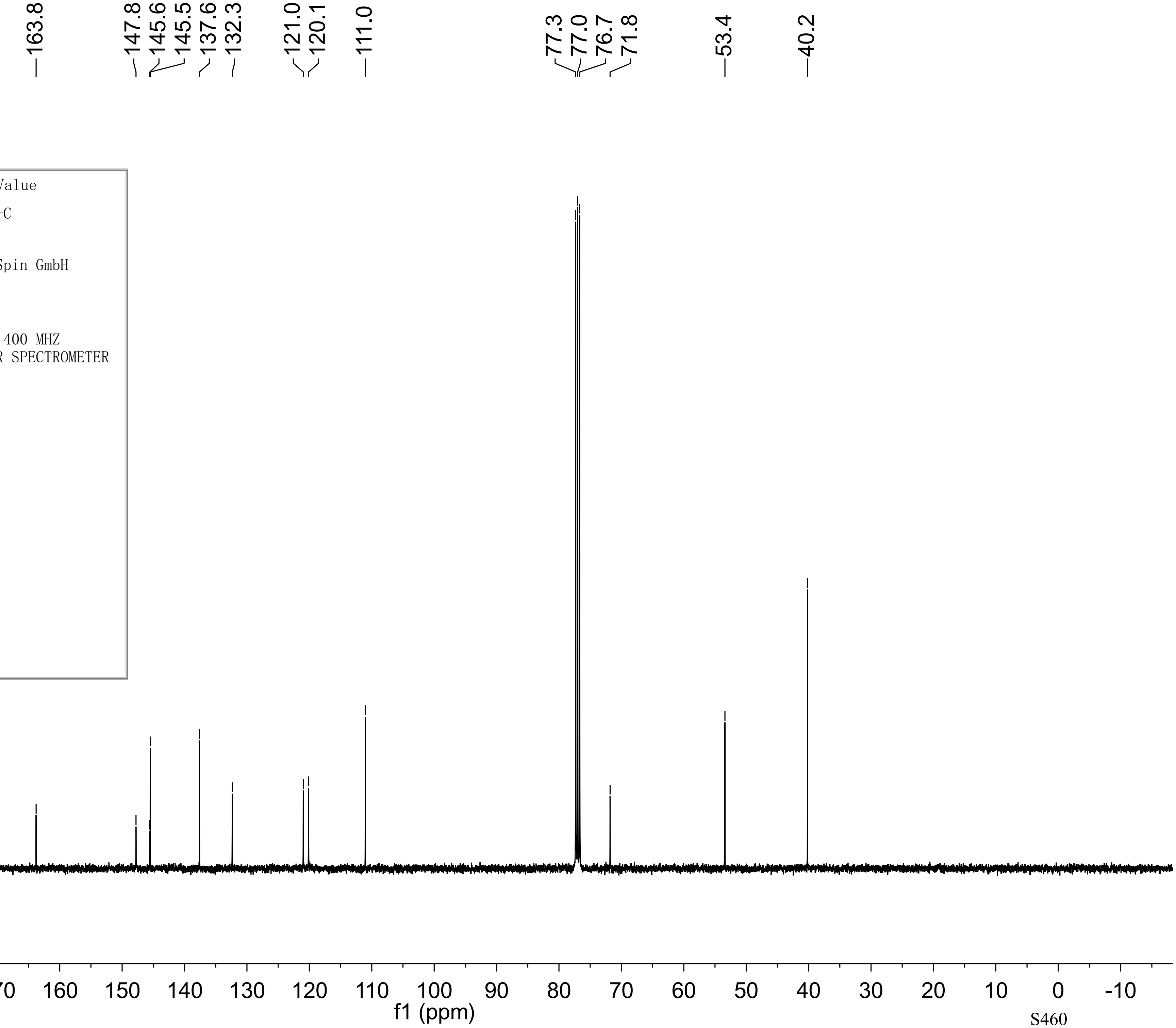

	Parameter	Value
1	Title	CFM-7-76-1-C
2	Comment	
3	Origin	Bruker BioSpin GmbH
4	Owner	nmrsu
5	Site	
6	Spectrometer	AVANCE NEO 400 MHZ DIGITAL NMR SPECTROMETER
7	Author	
8	Solvent	CDCl3
9	Temperature	298.1
10	Pulse Sequence	zgpg30
11	Experiment	1D
12	Number of Scans	512
13	Receiver Gain	64
14	Spectrometer Frequency	100.61
15	Spectral Width	23809.5
16	Lowest Frequency	-1847.3
17	Nucleus	13C
18	Acquired Size	32768
19	Spectral Size	32768

CFM-7-76-3

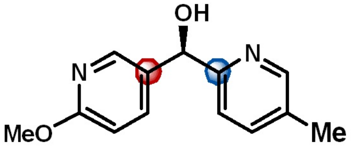

C44

	Parameter	Value
1	Title	CFM-7-76-3
2	Comment	
3	Origin	Bruker BioSpin GmbH
4	Owner	nmrsu
5	Site	
6	Spectrometer	AVANCE NEO 400 MHZ DIGITAL NMR SPECTROMETER
7	Author	
8	Solvent	CDCl3
9	Temperature	298.1
10	Pulse Sequence	zg30
11	Experiment	1D
12	Number of Scans	8
13	Receiver Gain	101
14	Spectrometer Frequency	400.13
15	Spectral Width	8196.7
16	Lowest Frequency	-1637.1
17	Nucleus	1H
18	Acquired Size	32768
19	Spectral Size	65536

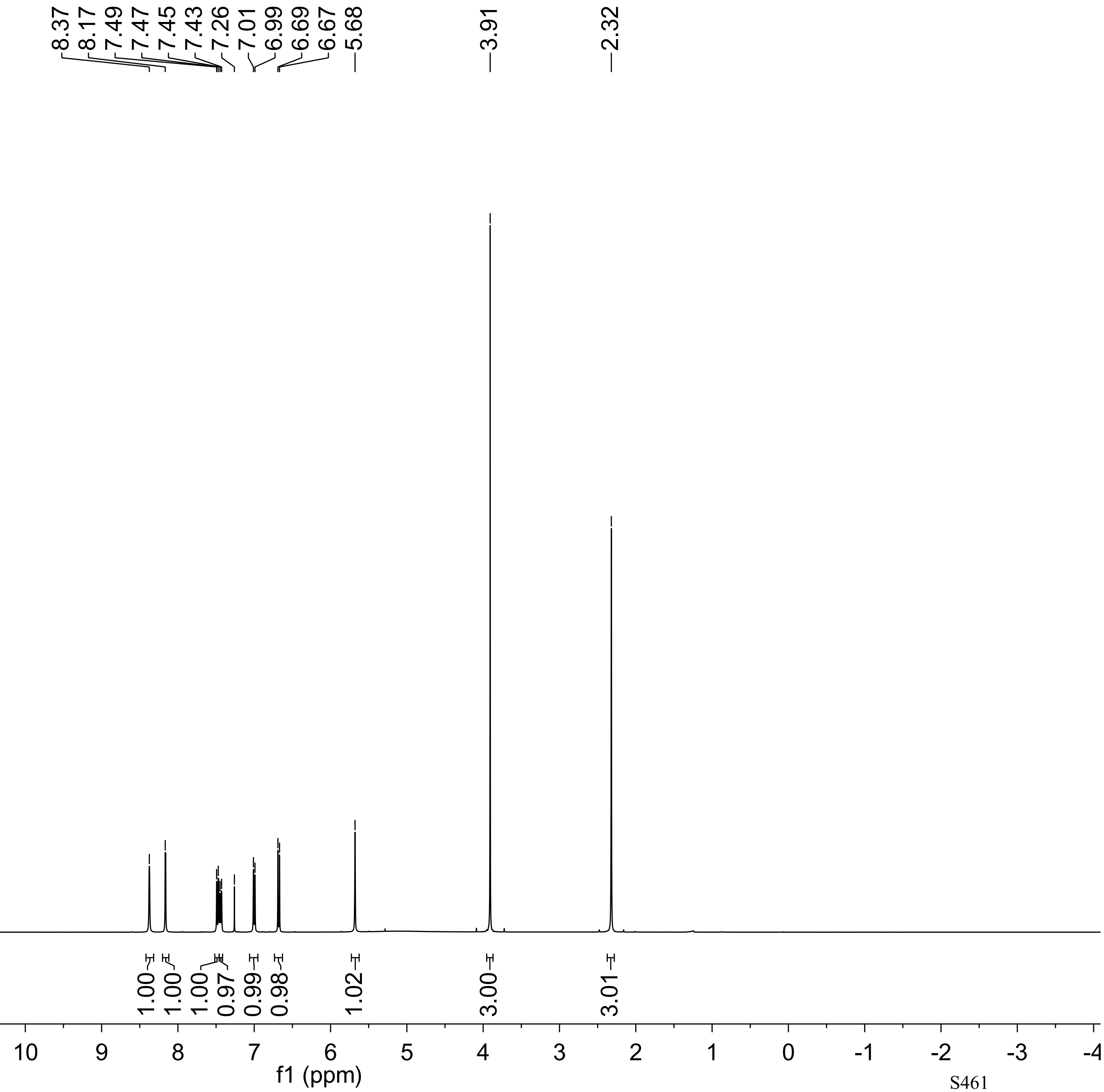

CFM-7-76-3

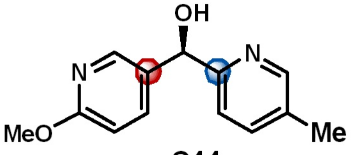

C44

Parameter	Value
1 Title	CFM-7-76-3
2 Comment	
3 Origin	Bruker BioSpin GmbH
4 Owner	nmrsu
5 Site	
6 Spectrometer	AVANCE NEO 400 MHZ DIGITAL NMR SPECTROMETER
7 Author	
8 Solvent	CDC13
9 Temperature	298.1
10 Pulse Sequence	zgpg30
11 Experiment	1D
12 Number of Scans	512
13 Receiver Gain	64
14 Spectrometer Frequency	100.61
15 Spectral Width	23809.5
16 Lowest Frequency	-1848.3
17 Nucleus	13C
18 Acquired Size	32768
19 Spectral Size	32768

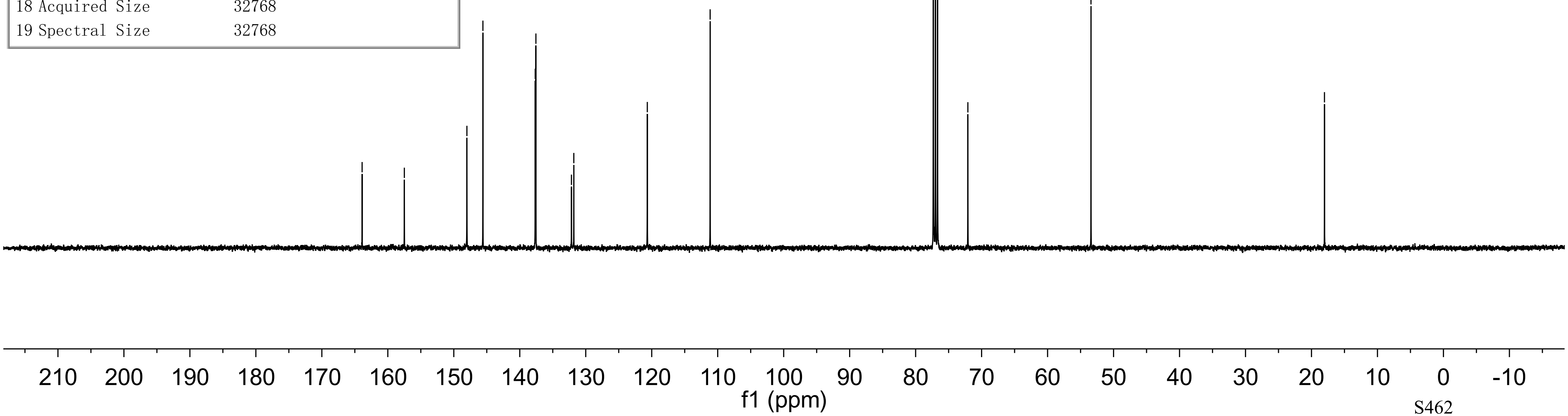

CFM-7-76-4

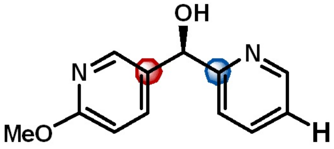

C45

Parameter	Value
1 Title	CFM-7-76-4
2 Comment	
3 Origin	Bruker BioSpin GmbH
4 Owner	nmrsu
5 Site	
6 Spectrometer	AVANCE NEO 400 MHZ DIGITAL NMR SPECTROMETER
7 Author	
8 Solvent	CDCl3
9 Temperature	298.1
10 Pulse Sequence	zg30
11 Experiment	1D
12 Number of Scans	8
13 Receiver Gain	101
14 Spectrometer Frequency	400.13
15 Spectral Width	8196.7
16 Lowest Frequency	-1637.3
17 Nucleus	1H
18 Acquired Size	32768
19 Spectral Size	65536

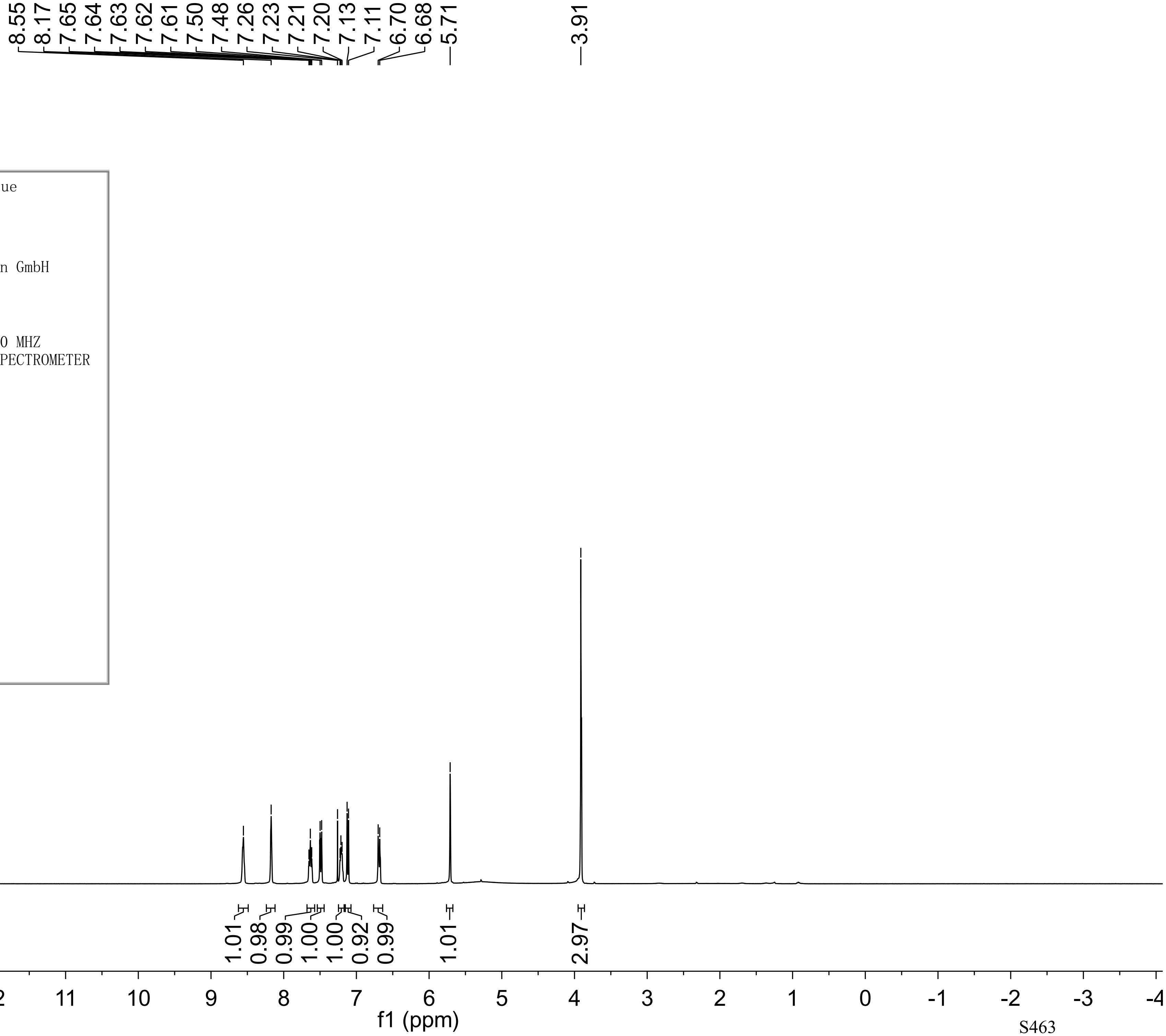

CFM-7-76-4

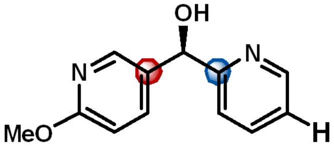

C45

Parameter	Value
1 Title	CFM-7-76-4
2 Comment	
3 Origin	Bruker BioSpin GmbH
4 Owner	nmrsu
5 Site	
6 Spectrometer	AVANCE NEO 400 MHZ DIGITAL NMR SPECTROMETER
7 Author	
8 Solvent	CDCl3
9 Temperature	298.1
10 Pulse Sequence	zgpg30
11 Experiment	1D
12 Number of Scans	512
13 Receiver Gain	57
14 Spectrometer Frequency	100.61
15 Spectral Width	23809.5
16 Lowest Frequency	-1848.0
17 Nucleus	13C
18 Acquired Size	32768
19 Spectral Size	32768

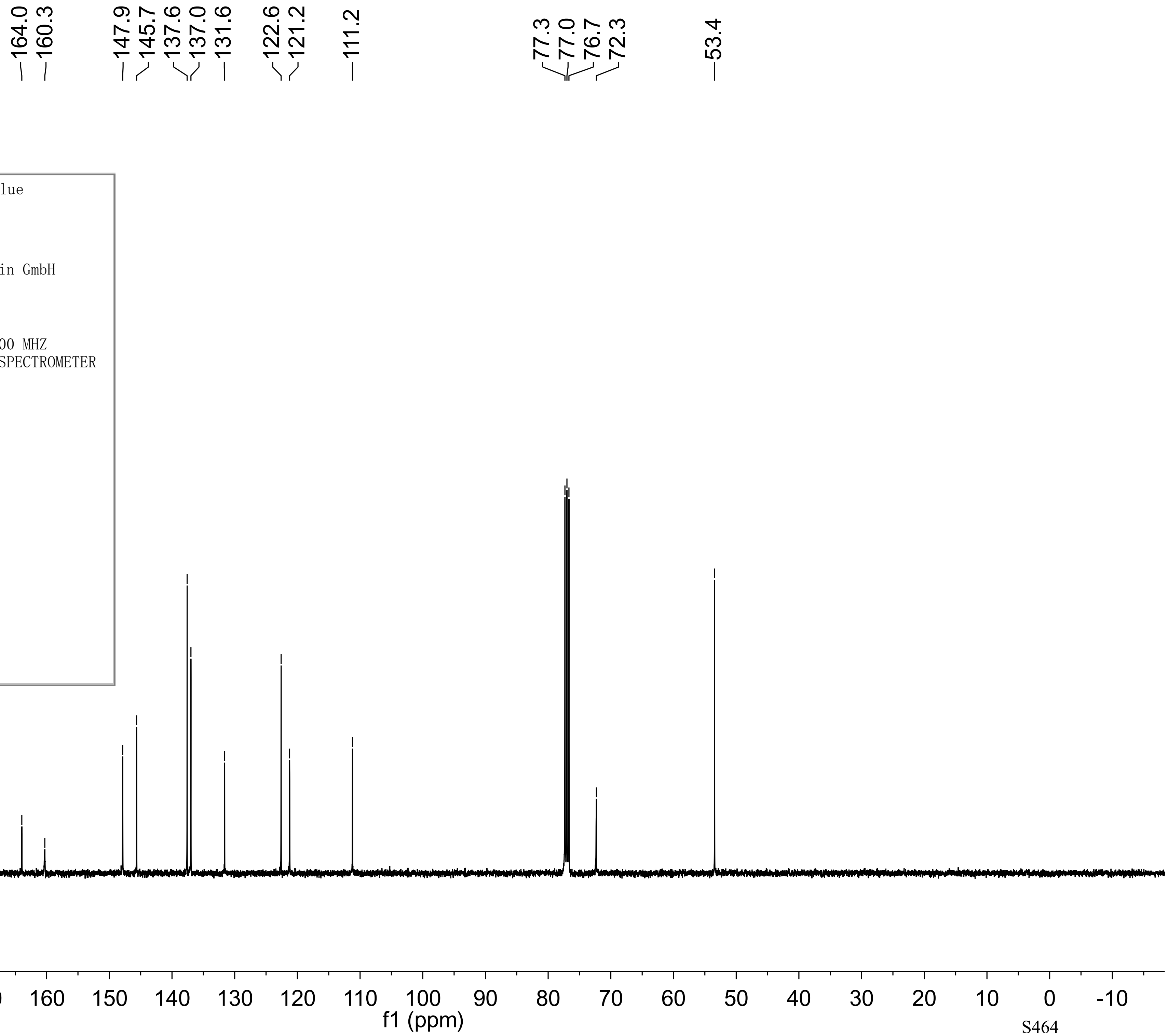

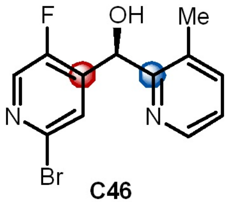

C46

	Parameter	Value
1	Title	CFM-C39-0702
2	Comment	
3	Origin	Bruker BioSpin GmbH
4	Owner	nmrsu
5	Site	
6	Spectrometer	AVANCE NEO 400 MHZ DIGITAL NMR SPECTROMETER
7	Author	
8	Solvent	CDCl3
9	Temperature	298.2
10	Pulse Sequence	zg30
11	Experiment	1D
12	Number of Scans	8
13	Receiver Gain	101
14	Spectrometer Frequency	400.13
15	Spectral Width	8196.7
16	Lowest Frequency	-1637.3
17	Nucleus	1H
18	Acquired Size	32768
19	Spectral Size	65536

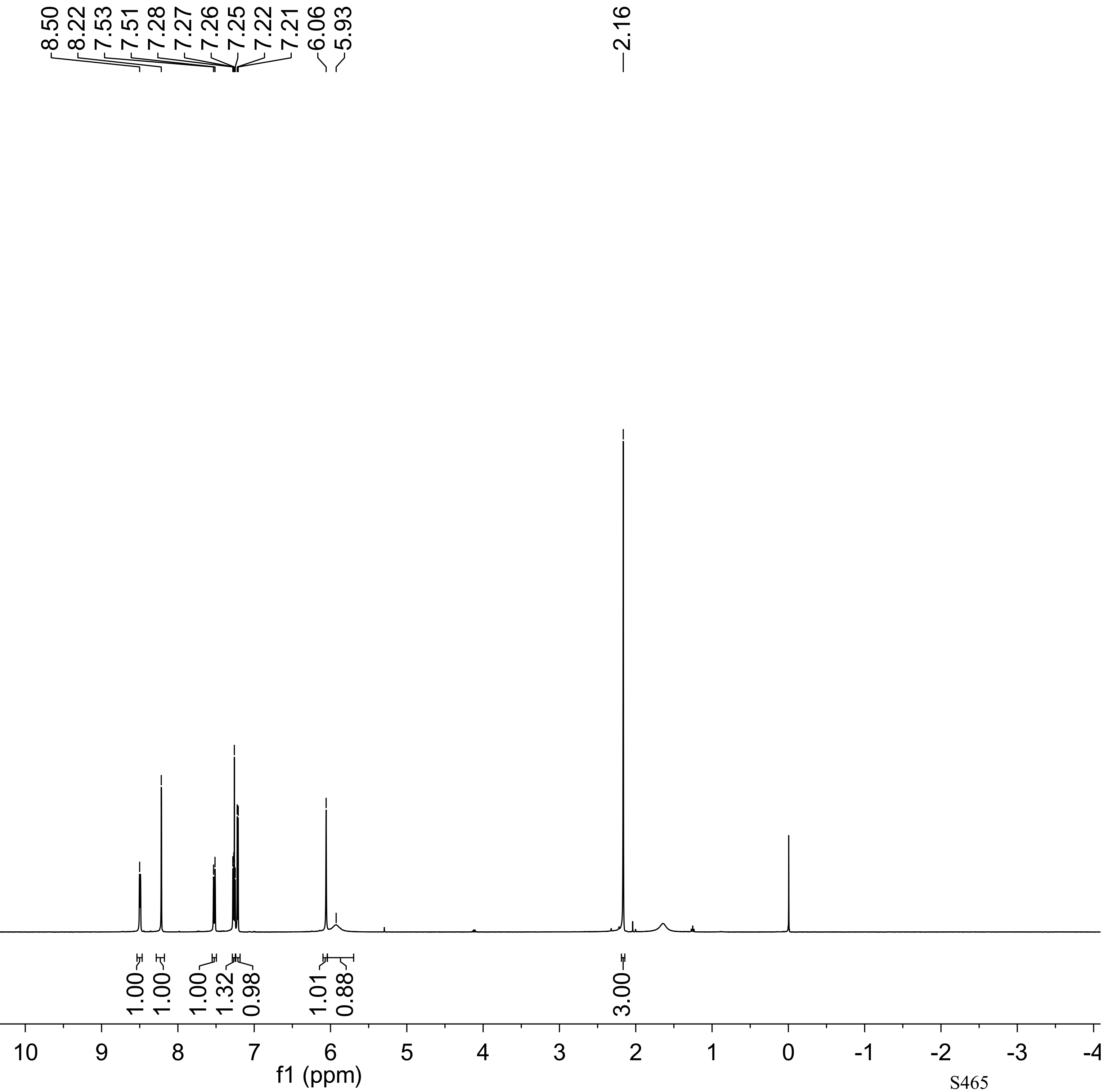

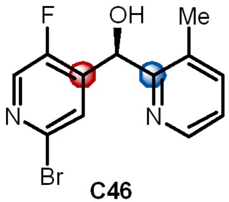

C46

Parameter	Value
1 Title	CFM-C39-H-C
2 Comment	
3 Origin	Bruker BioSpin GmbH
4 Owner	nmrsu
5 Site	
6 Spectrometer	Avance Neo 400M
7 Author	
8 Solvent	CDCl3
9 Temperature	298.3
10 Pulse Sequence	zgpg30
11 Experiment	1D
12 Number of Scans	256
13 Receiver Gain	16
14 Spectrometer Frequency	100.63
15 Spectral Width	23809.5
16 Lowest Frequency	-1845.7
17 Nucleus	13C
18 Acquired Size	32768
19 Spectral Size	32768

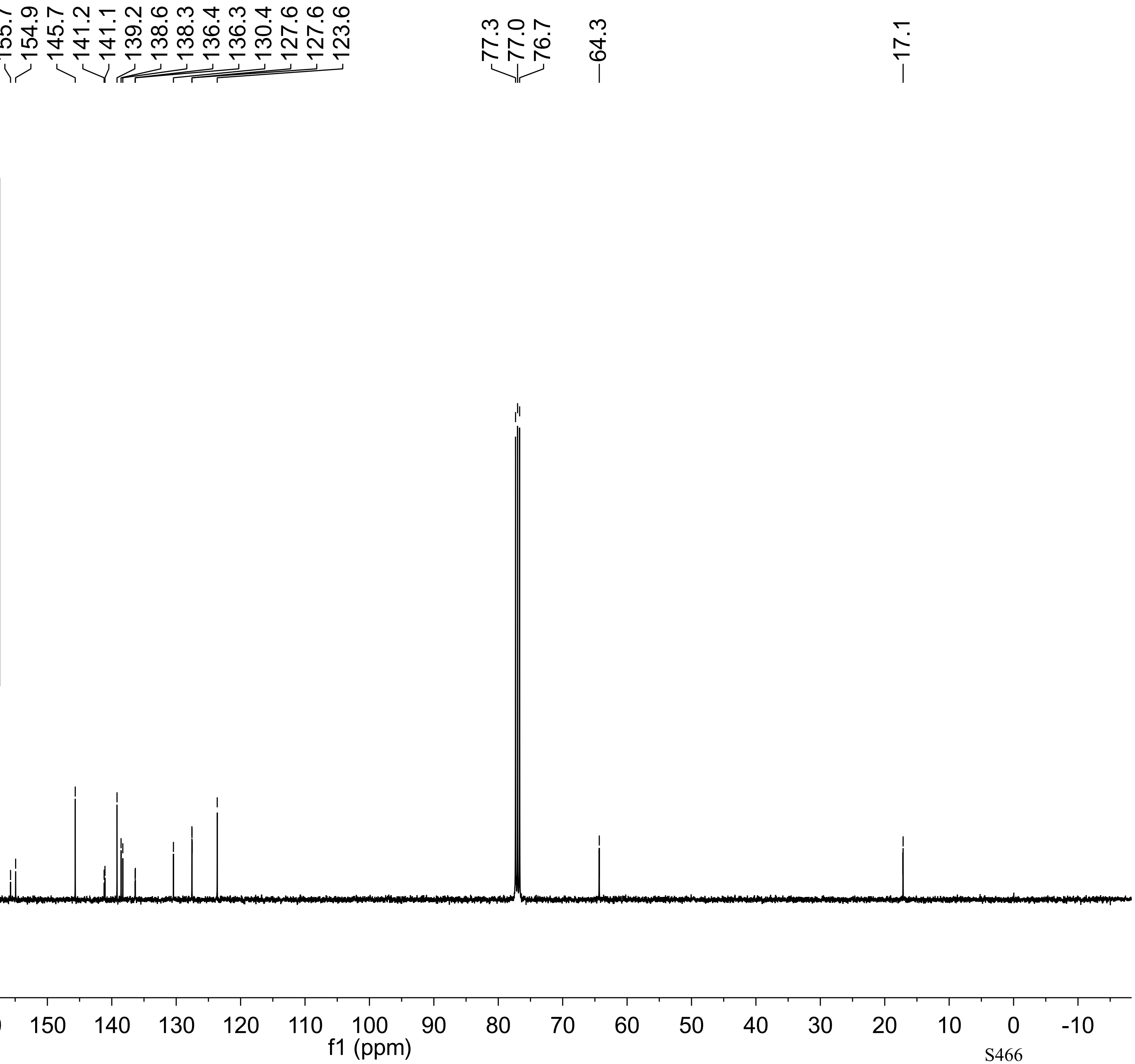

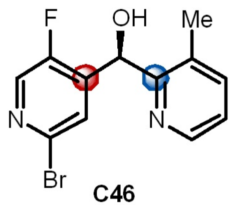

C46

Parameter	Value
1 Title	CFM-C39-0702
2 Comment	
3 Origin	Bruker BioSpin GmbH
4 Owner	nmrsu
5 Site	
6 Spectrometer	AVANCE NEO 400 MHZ DIGITAL NMR SPECTROMETER
7 Author	
8 Solvent	CDCl3
9 Temperature	298.1
10 Pulse Sequence	zg
11 Experiment	1D
12 Number of Scans	8
13 Receiver Gain	101
14 Spectrometer Frequency	376.50
15 Spectral Width	90909.1
16 Lowest Frequency	-83104.4
17 Nucleus	19F
18 Acquired Size	65536
19 Spectral Size	65536

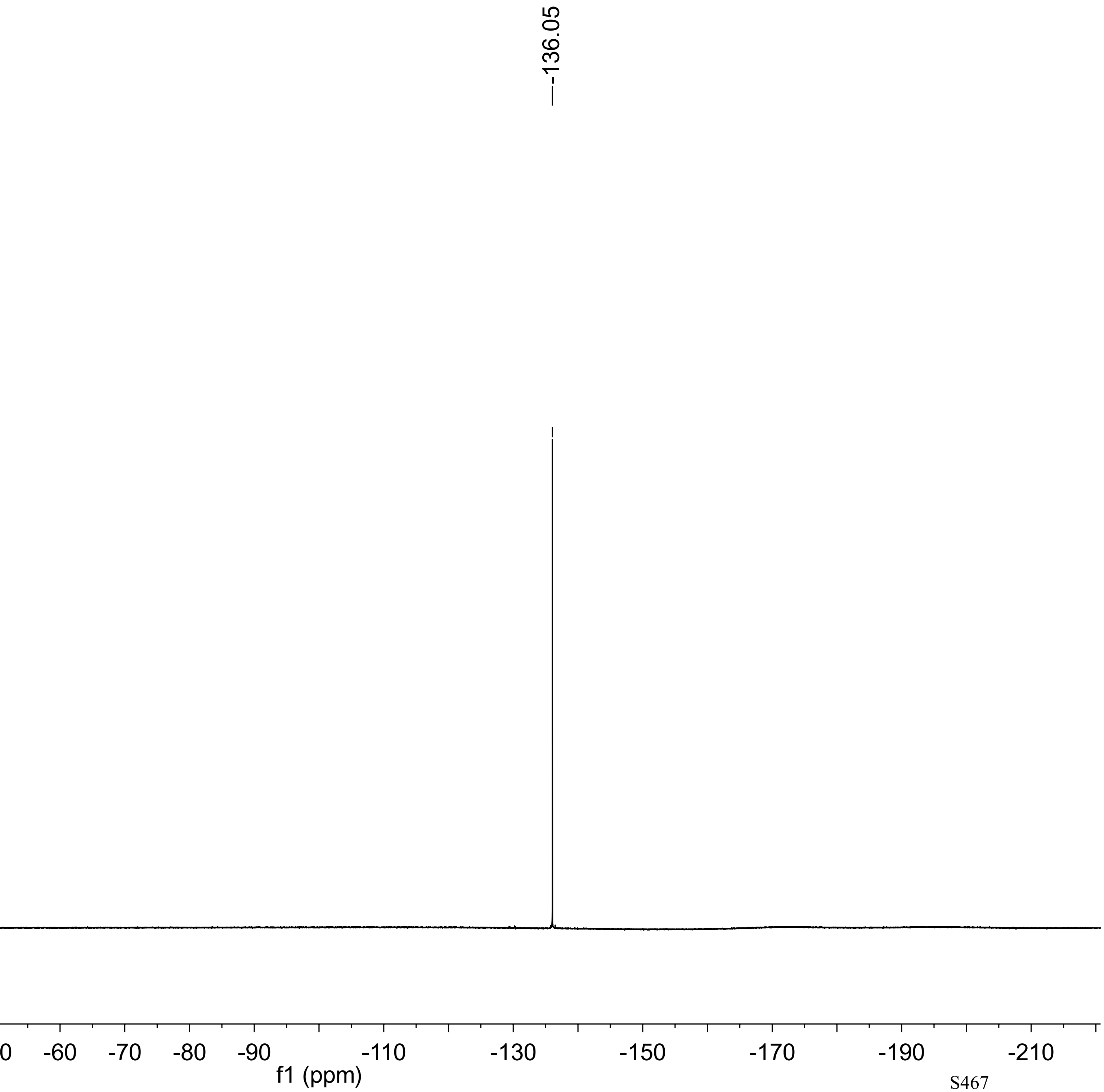

CFM-7-75-4

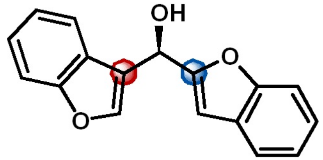

C47

	Parameter	Value
1	Title	CFM-7-75-4
2	Comment	
3	Origin	Bruker BioSpin GmbH
4	Owner	nmrsu
5	Site	
6	Spectrometer	AVANCE NEO 400 MHZ DIGITAL NMR SPECTROMETER
7	Author	
8	Solvent	CDCl3
9	Temperature	298.1
10	Pulse Sequence	zg30
11	Experiment	1D
12	Number of Scans	8
13	Receiver Gain	101
14	Spectrometer Frequency	400.13
15	Spectral Width	8196.7
16	Lowest Frequency	-1637.0
17	Nucleus	1H
18	Acquired Size	32768
19	Spectral Size	65536

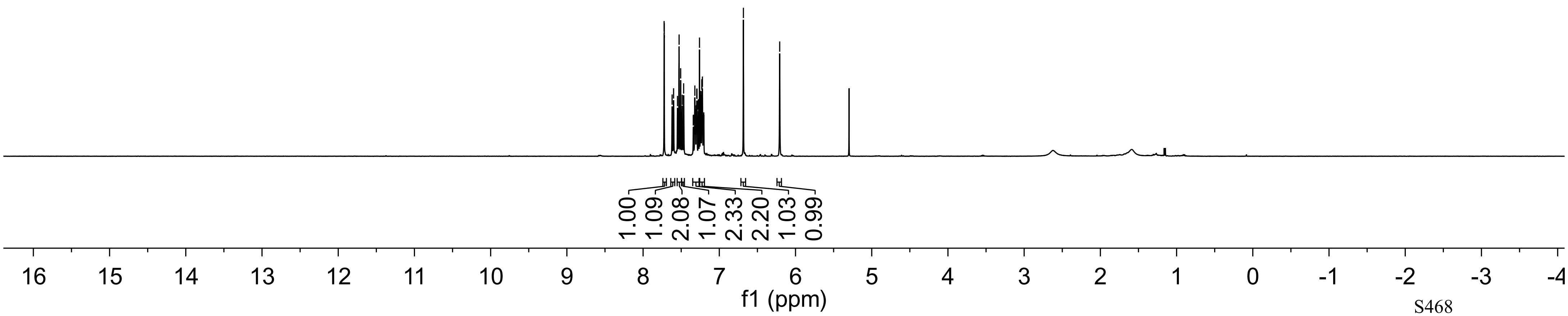

CFM-7-75-4

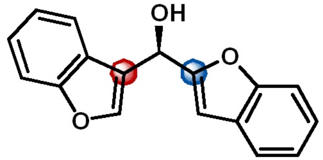

C47

Parameter	Value
1 Title	CFM-7-75-4
2 Comment	
3 Origin	Bruker BioSpin GmbH
4 Owner	nmrsu
5 Site	
6 Spectrometer	AVANCE NEO 400 MHZ DIGITAL NMR SPECTROMETER
7 Author	
8 Solvent	CDCl3
9 Temperature	298.1
10 Pulse Sequence	zgpg30
11 Experiment	1D
12 Number of Scans	512
13 Receiver Gain	57
14 Spectrometer Frequency	100.61
15 Spectral Width	23809.5
16 Lowest Frequency	-1847.7
17 Nucleus	13C
18 Acquired Size	32768
19 Spectral Size	32768

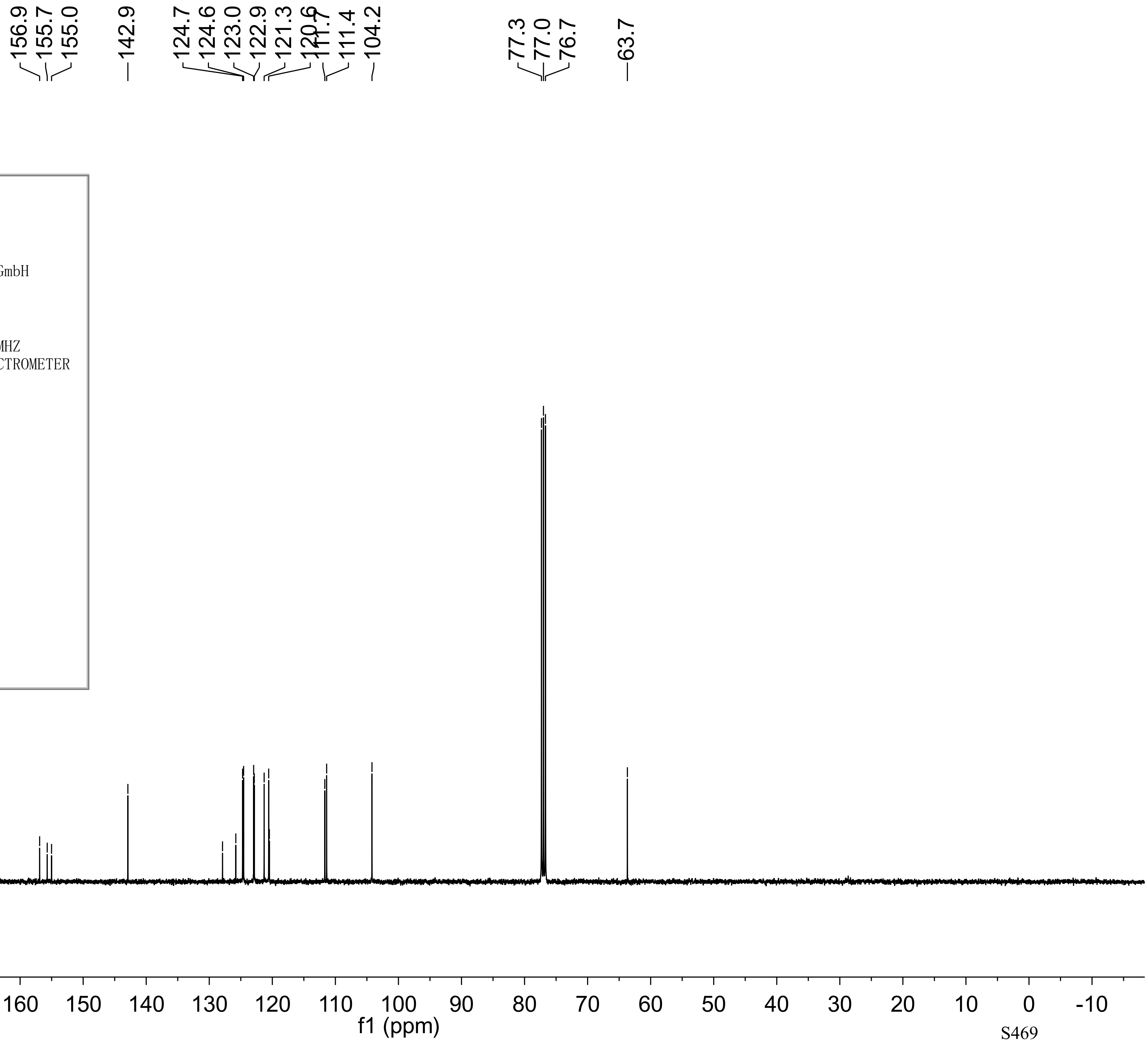

CFM-7-75-6

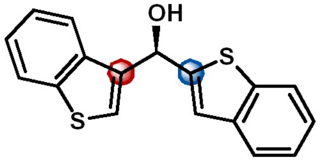

C48

	Parameter	Value
1	Title	CFM-7-75-6
2	Comment	
3	Origin	Bruker BioSpin GmbH
4	Owner	nmrsu
5	Site	
6	Spectrometer	AVANCE NEO 400 MHZ DIGITAL NMR SPECTROMETER
7	Author	
8	Solvent	CDCl3
9	Temperature	298.1
10	Pulse Sequence	zg30
11	Experiment	1D
12	Number of Scans	8
13	Receiver Gain	101
14	Spectrometer Frequency	400.13
15	Spectral Width	8196.7
16	Lowest Frequency	-1627.6
17	Nucleus	1H
18	Acquired Size	32768
19	Spectral Size	65536

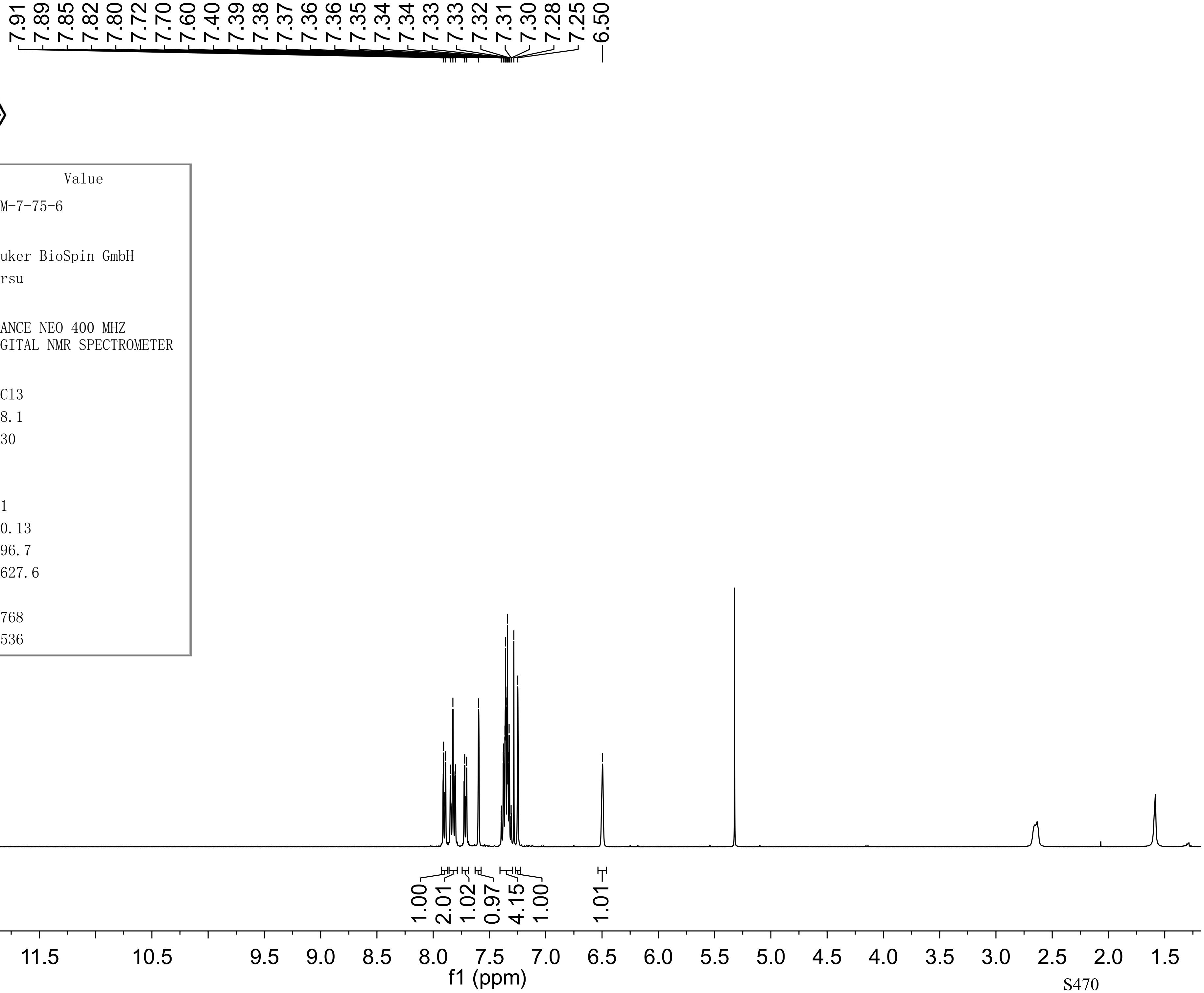

CFM-7-75-6

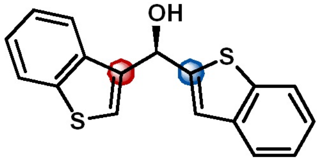

C48

Parameter	Value
1 Title	CFM-7-75-6
2 Comment	
3 Origin	Bruker BioSpin GmbH
4 Owner	nmrsu
5 Site	
6 Spectrometer	AVANCE NEO 400 MHZ DIGITAL NMR SPECTROMETER
7 Author	
8 Solvent	CDCl3
9 Temperature	298.2
10 Pulse Sequence	zgpg30
11 Experiment	1D
12 Number of Scans	512
13 Receiver Gain	64
14 Spectrometer Frequency	100.61
15 Spectral Width	23809.5
16 Lowest Frequency	-1847.4
17 Nucleus	13C
18 Acquired Size	32768
19 Spectral Size	32768

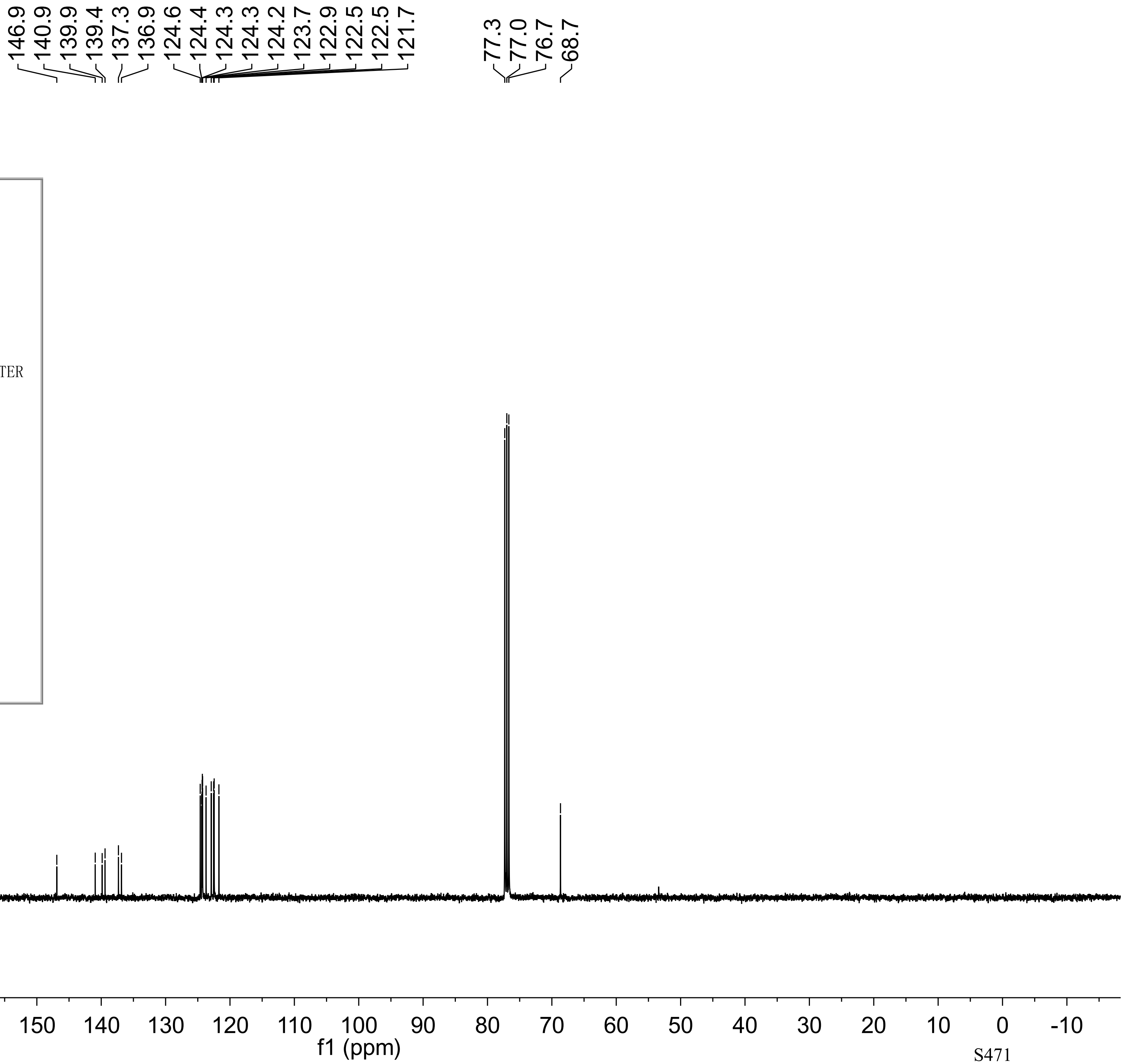

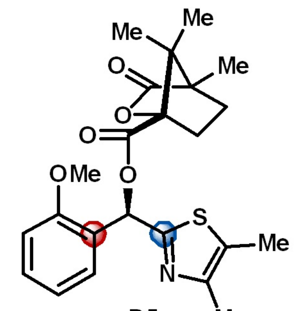

D5

	Parameter	Value
1	Title	CFM-D-C3-2
2	Comment	
3	Origin	Bruker BioSpin GmbH
4	Owner	nmrsu
5	Site	
6	Spectrometer	Avance NEO 600
7	Author	
8	Solvent	CDCl3
9	Temperature	298.1
10	Pulse Sequence	zg30
11	Experiment	1D
12	Number of Scans	8
13	Receiver Gain	101
14	Relaxation Delay	1.0000
15	Pulse Width	10.0000
16	Acquisition Time	2.7525
17	Acquisition Date	2020-08-04T14:41:20
18	Modification Date	2020-08-04T14:51:28
19	Spectrometer Frequency	600.15
20	Spectral Width	11904.8
21	Lowest Frequency	-2261.3
22	Nucleus	1H
23	Acquired Size	32768
24	Spectral Size	65536

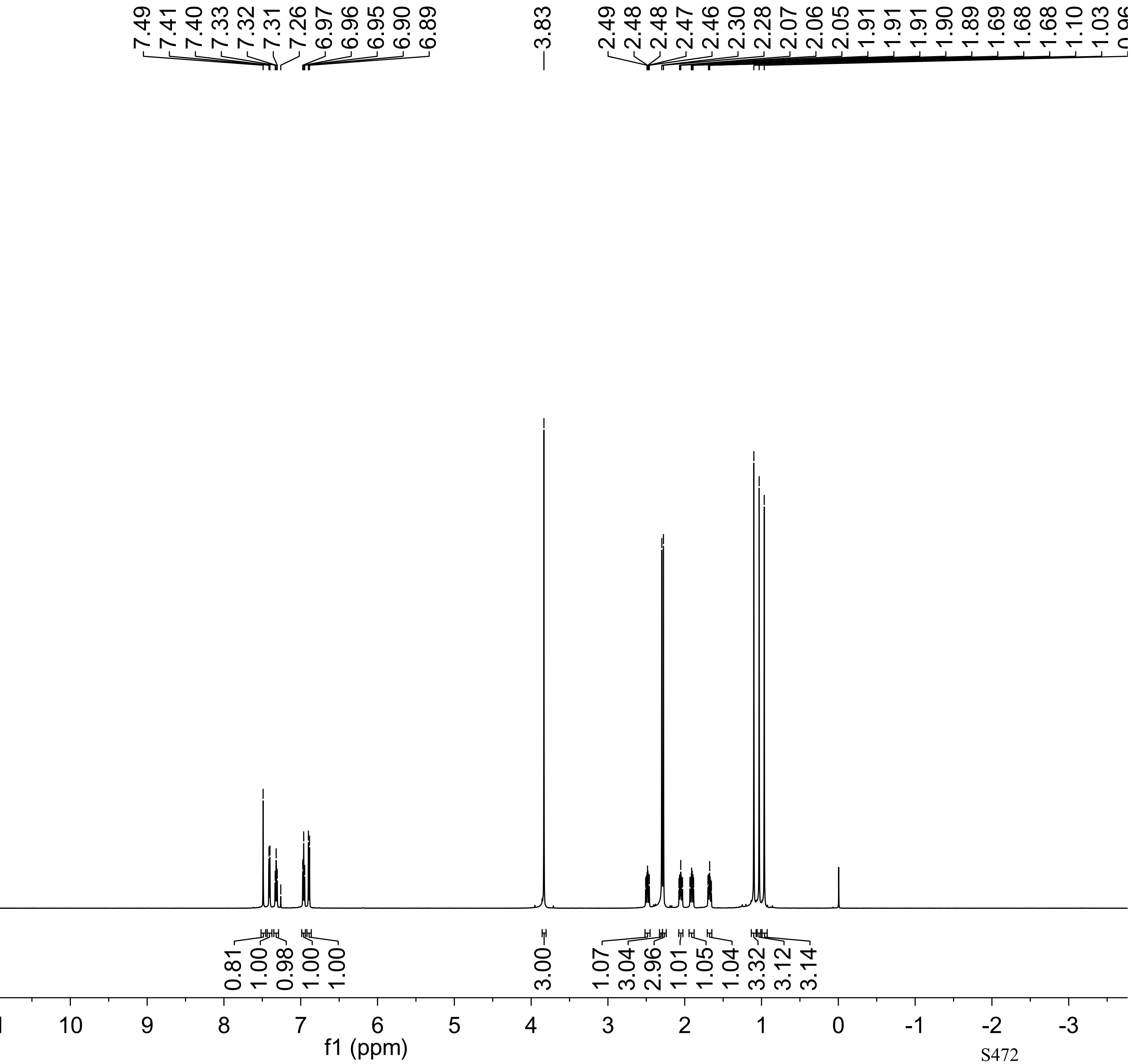

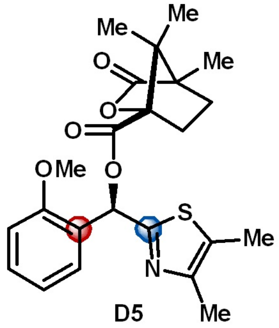

D5

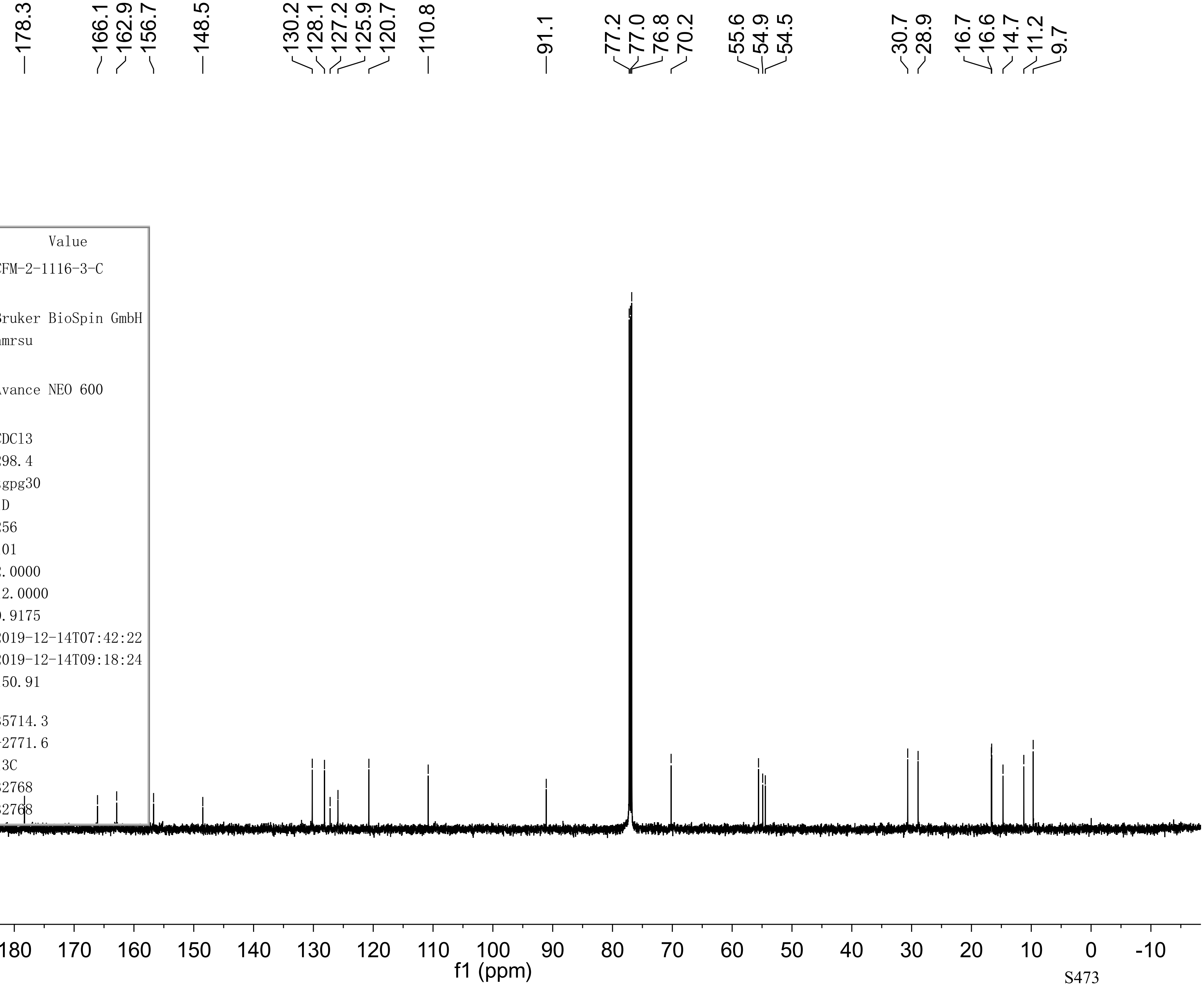

	Parameter	Value
1	Title	CFM-2-1116-3-C
2	Comment	
3	Origin	Bruker BioSpin GmbH
4	Owner	nmrsu
5	Site	
6	Spectrometer	Avance NEO 600
7	Author	
8	Solvent	CDCl3
9	Temperature	298.4
10	Pulse Sequence	zgpg30
11	Experiment	1D
12	Number of Scans	256
13	Receiver Gain	101
14	Relaxation Delay	2.0000
15	Pulse Width	12.0000
16	Acquisition Time	0.9175
17	Acquisition Date	2019-12-14T07:42:22
18	Modification Date	2019-12-14T09:18:24
19	Spectrometer Frequency	150.91
20	Spectral Width	35714.3
21	Lowest Frequency	-2771.6
22	Nucleus	13C
23	Acquired Size	32768
24	Spectral Size	32768

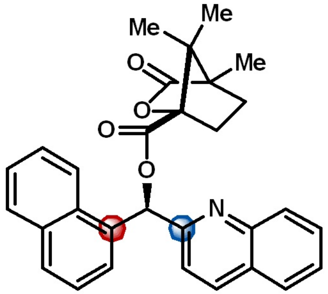

D8

Parameter		Value
1	Title	D-CFM-E10-1
2	Comment	
3	Origin	Bruker BioSpin GmbH
4	Owner	nmrsu
5	Site	
6	Spectrometer	Avance NEO 600
7	Author	
8	Solvent	CDCl3
9	Temperature	296.8
10	Pulse Sequence	zg30
11	Experiment	1D
12	Number of Scans	8
13	Receiver Gain	101
14	Relaxation Delay	1.0000
15	Pulse Width	10.0000
16	Acquisition Time	2.7525
17	Acquisition Date	2020-04-02T08:28:25
18	Modification Date	2020-04-02T09:52:36
19	Spectrometer Frequency	600.15
20	Spectral Width	11904.8
21	Lowest Frequency	-2261.0
22	Nucleus	1H
23	Acquired Size	32768
24	Spectral Size	65536

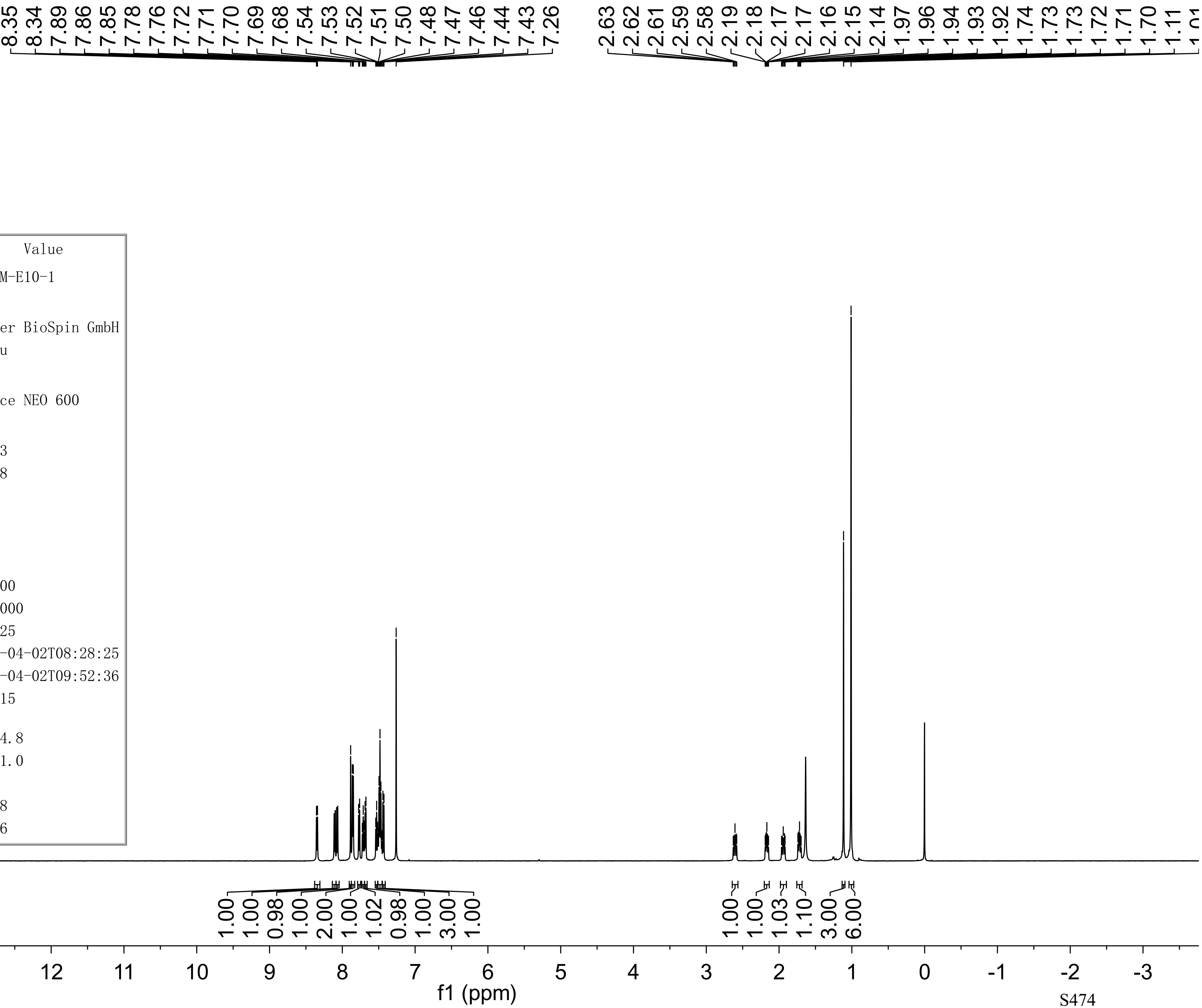

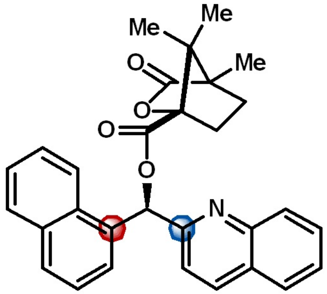

D8

	Parameter	Value
1	Title	D-CFM-E10-1
2	Comment	
3	Origin	Bruker BioSpin GmbH
4	Owner	nmrsu
5	Site	
6	Spectrometer	Avance NEO 600
7	Author	
8	Solvent	CDCl3
9	Temperature	298.0
10	Pulse Sequence	zgpg30
11	Experiment	1D
12	Number of Scans	128
13	Receiver Gain	101
14	Relaxation Delay	2.0000
15	Pulse Width	12.0000
16	Acquisition Time	0.9175
17	Acquisition Date	2020-04-02T08:35:44
18	Modification Date	2020-04-02T09:52:37
19	Spectrometer Frequency	150.91
20	Spectral Width	35714.3
21	Lowest Frequency	-2772.4
22	Nucleus	13C
23	Acquired Size	32768
24	Spectral Size	32768

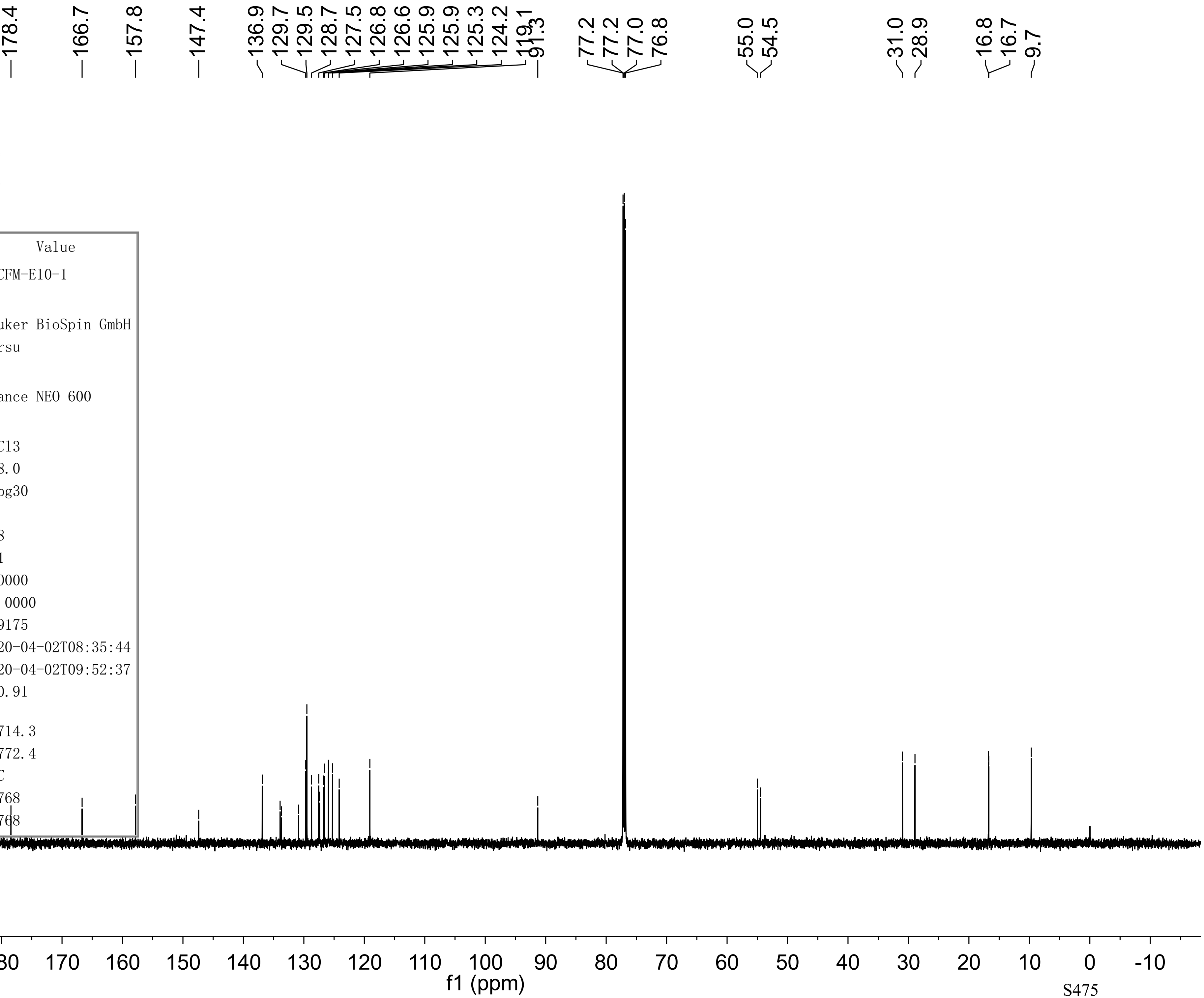

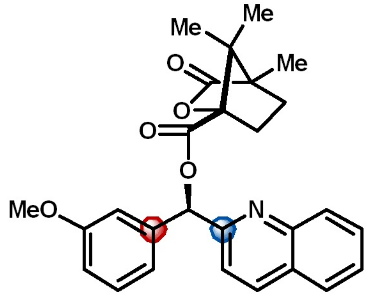

D13

	Parameter	Value
1	Title	D-CFM-E7
2	Comment	
3	Origin	Bruker BioSpin GmbH
4	Owner	nmrsu
5	Site	
6	Spectrometer	Avance NEO 600
7	Author	
8	Solvent	CDCl3
9	Temperature	296.6
10	Pulse Sequence	zg30
11	Experiment	1D
12	Number of Scans	16
13	Receiver Gain	101
14	Relaxation Delay	1.0000
15	Pulse Width	10.0000
16	Acquisition Time	2.7525
17	Acquisition Date	2019-12-30T00:39:23
18	Modification Date	2019-12-30T08:57:26
19	Spectrometer Frequency	600.15
20	Spectral Width	11904.8
21	Lowest Frequency	-2260.9
22	Nucleus	1H
23	Acquired Size	32768
24	Spectral Size	65536

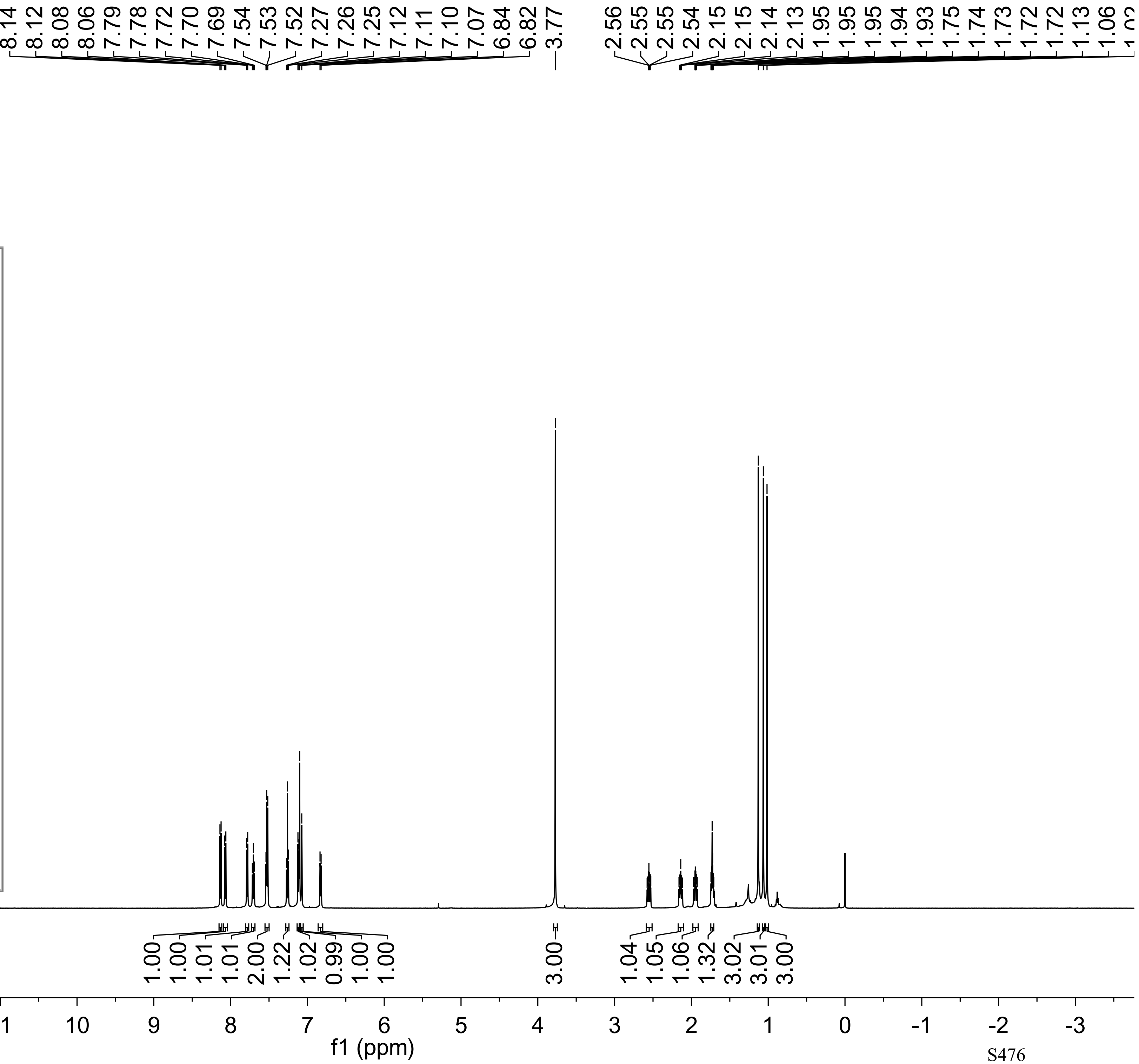

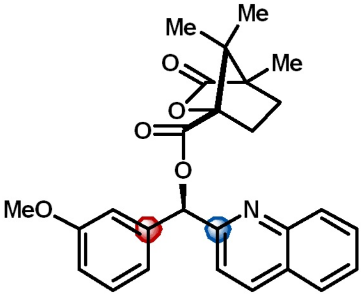

D13

Parameter	Value
1 Title	D-CFM-E7
2 Comment	
3 Origin	Bruker BioSpin GmbH
4 Owner	nmrsu
5 Site	
6 Spectrometer	Avance NEO 600
7 Author	
8 Solvent	CDCl3
9 Temperature	298.2
10 Pulse Sequence	zgpg30
11 Experiment	1D
12 Number of Scans	512
13 Receiver Gain	101
14 Relaxation Delay	2.0000
15 Pulse Width	12.0000
16 Acquisition Time	0.9175
17 Acquisition Date	2019-12-30T01:05:52
18 Modification Date	2019-12-30T08:57:27
19 Spectrometer Frequency	150.91
20 Spectral Width	35714.3
21 Lowest Frequency	-2773.9
22 Nucleus	13C
23 Acquired Size	32768
24 Spectral Size	32768

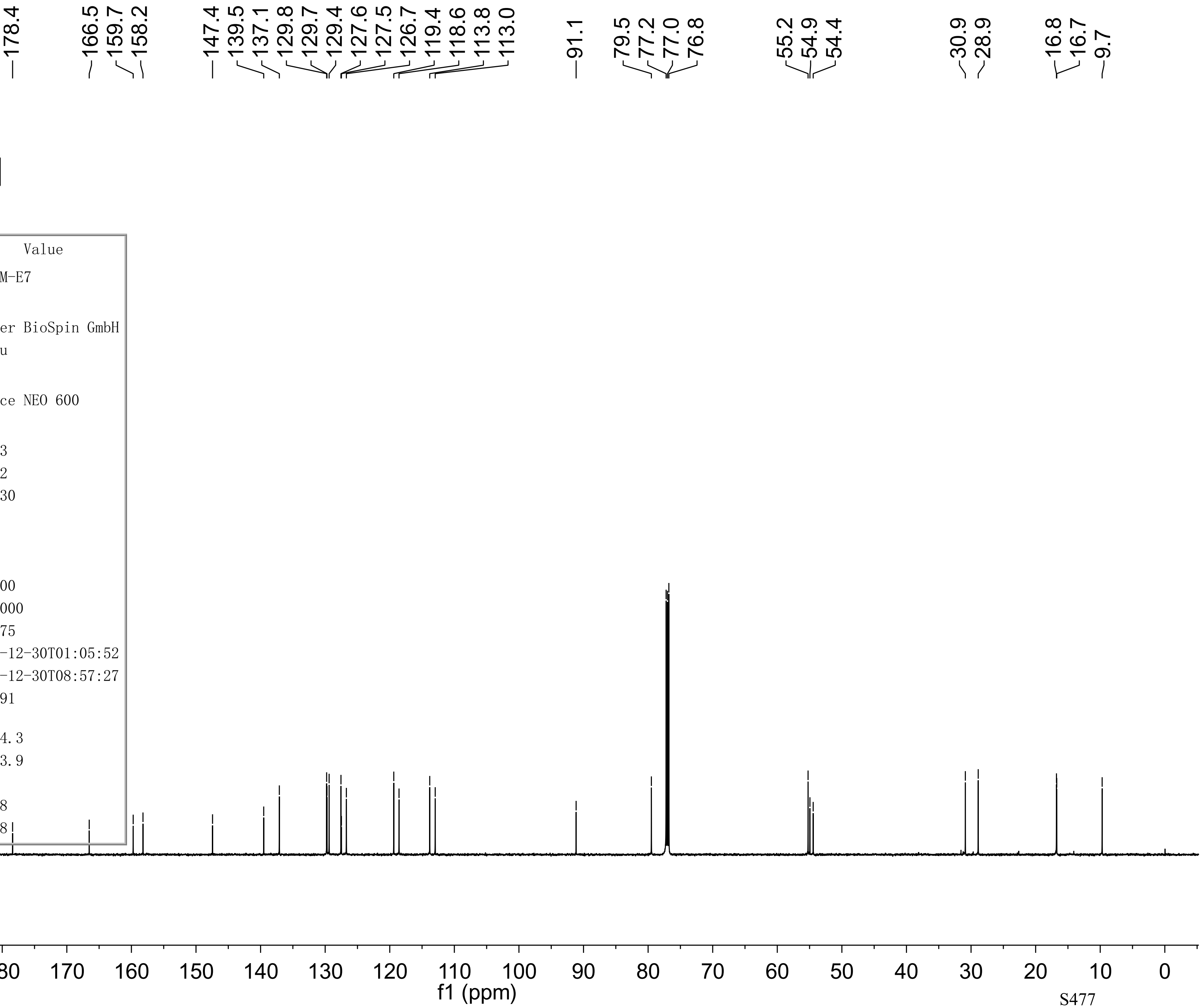

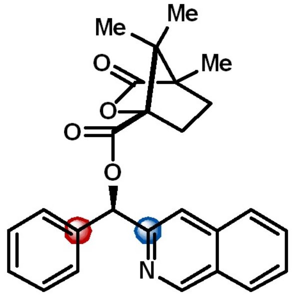

D17

	Parameter	Value
1	Title	CFM-D-A1-2
2	Comment	
3	Origin	Bruker BioSpin GmbH
4	Owner	nmrsu
5	Site	
6	Spectrometer	Avance NEO 600
7	Author	
8	Solvent	CDCl3
9	Temperature	298.3
10	Pulse Sequence	zg30
11	Experiment	1D
12	Number of Scans	8
13	Receiver Gain	101
14	Relaxation Delay	1.0000
15	Pulse Width	10.0000
16	Acquisition Time	2.7525
17	Acquisition Date	2020-08-04T14:27:25
18	Modification Date	2020-08-04T14:51:28
19	Spectrometer Frequency	600.15
20	Spectral Width	11904.8
21	Lowest Frequency	-2260.9
22	Nucleus	1H
23	Acquired Size	32768
24	Spectral Size	65536

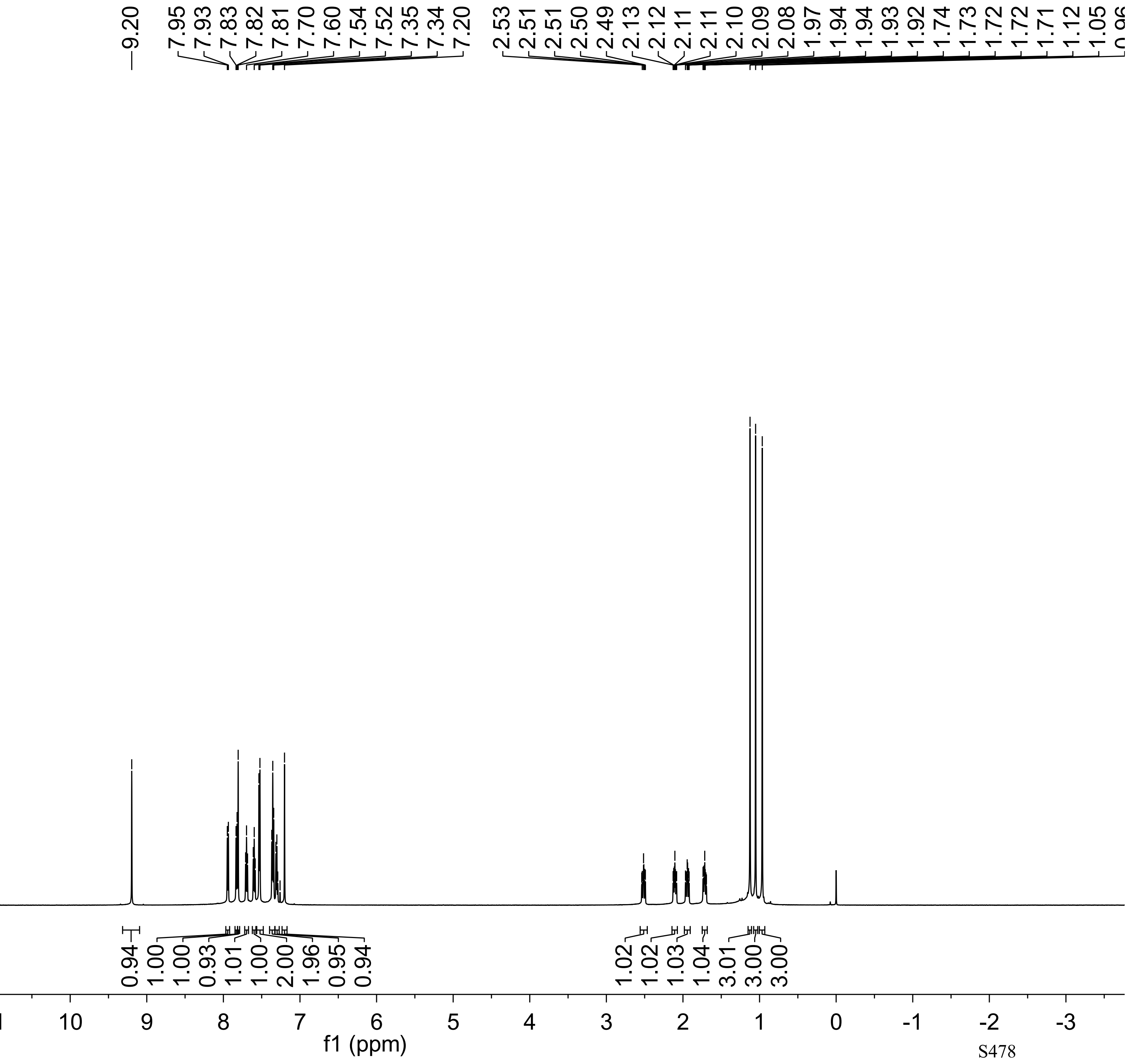

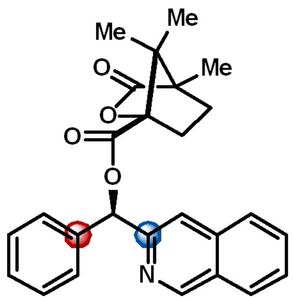

D17

Parameter	Value
1 Title	D-CFM-A1
2 Comment	
3 Origin	Bruker BioSpin GmbH
4 Owner	nmrsu
5 Site	
6 Spectrometer	Avance NEO 600
7 Author	
8 Solvent	CDCl3
9 Temperature	298.1
10 Pulse Sequence	zgpg30
11 Experiment	1D
12 Number of Scans	512
13 Receiver Gain	101
14 Relaxation Delay	2.0000
15 Pulse Width	12.0000
16 Acquisition Time	0.9175
17 Acquisition Date	2019-12-30T00:01:05
18 Modification Date	2019-12-30T08:57:26
19 Spectrometer Frequency	150.91
20 Spectral Width	35714.3
21 Lowest Frequency	-2770.3
22 Nucleus	13C
23 Acquired Size	32768
24 Spectral Size	32768

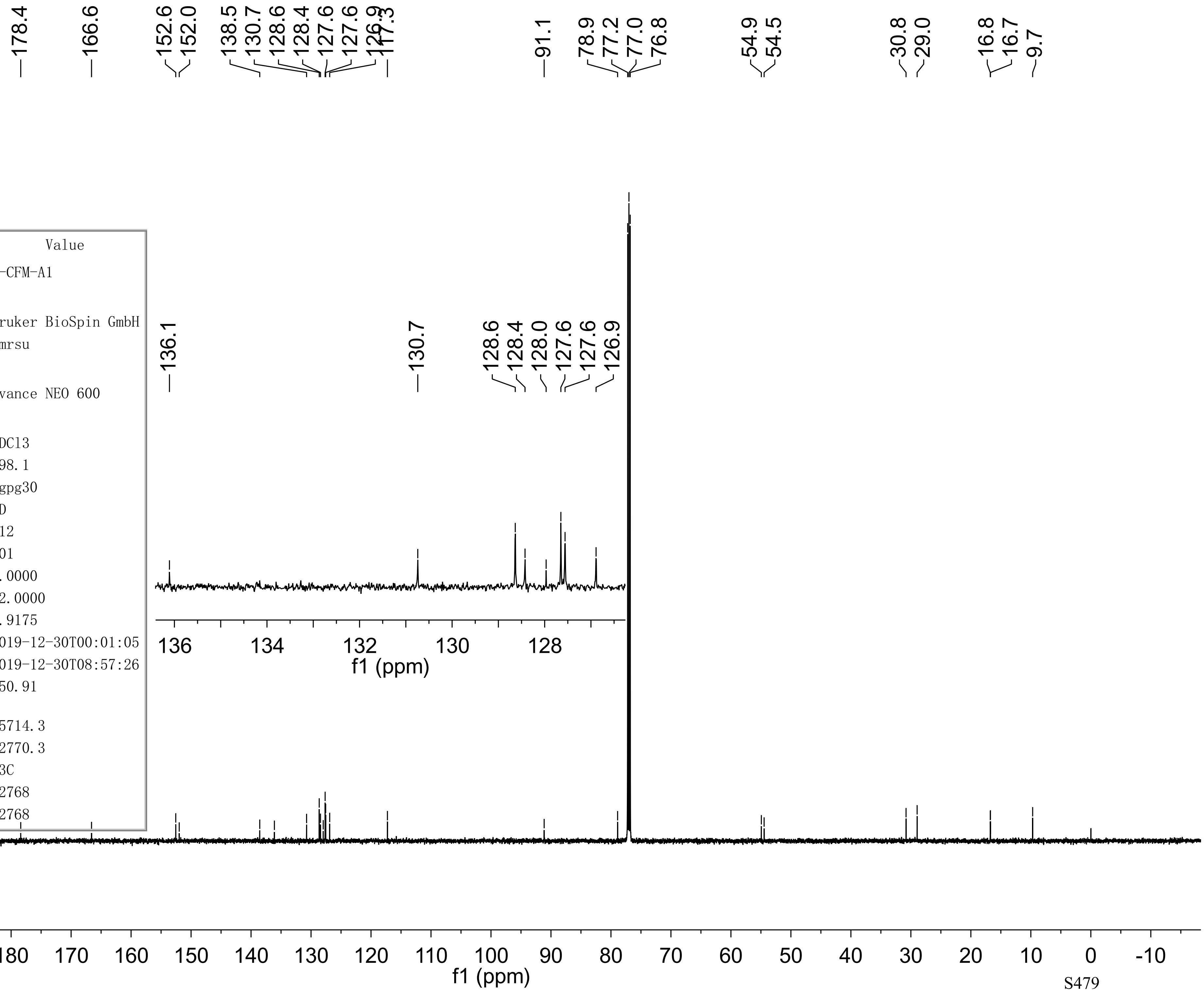

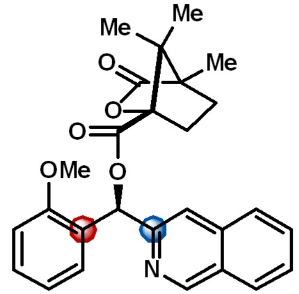

D18

Parameter	Value
1 Title	CFM-D-A4-2
2 Comment	
3 Origin	Bruker BioSpin GmbH
4 Owner	nmrsu
5 Site	
6 Spectrometer	Avance NEO 600
7 Author	
8 Solvent	CDCl3
9 Temperature	298.2
10 Pulse Sequence	zg30
11 Experiment	1D
12 Number of Scans	8
13 Receiver Gain	101
14 Relaxation Delay	1.0000
15 Pulse Width	10.0000
16 Acquisition Time	2.7525
17 Acquisition Date	2020-08-04T14:31:56
18 Modification Date	2020-08-04T14:51:27
19 Spectrometer Frequency	600.15
20 Spectral Width	11904.8
21 Lowest Frequency	-2261.2
22 Nucleus	1H
23 Acquired Size	32768
24 Spectral Size	65536

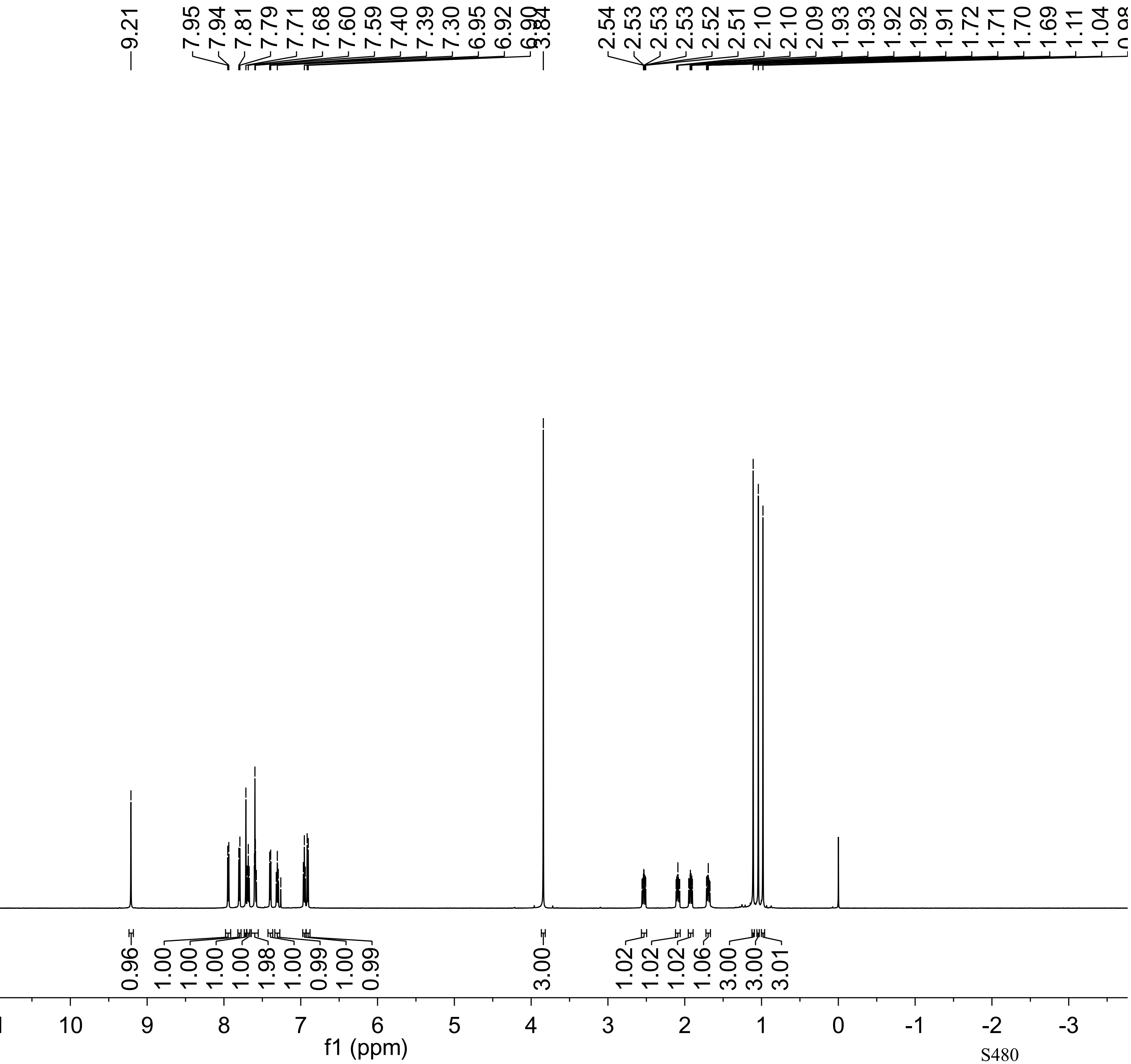

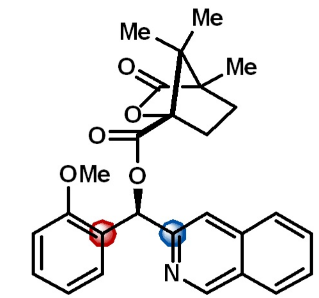

D18

Parameter	Value
1 Title	CFM-2-1116-1-C
2 Comment	
3 Origin	Bruker BioSpin GmbH
4 Owner	nmrsu
5 Site	
6 Spectrometer	Avance NEO 600
7 Author	
8 Solvent	CDCl3
9 Temperature	298.3
10 Pulse Sequence	zgpg30
11 Experiment	1D
12 Number of Scans	256
13 Receiver Gain	101
14 Relaxation Delay	2.0000
15 Pulse Width	12.0000
16 Acquisition Time	0.9175
17 Acquisition Date	2019-12-14T07:06:00
18 Modification Date	2019-12-14T09:18:17
19 Spectrometer Frequency	150.91
20 Spectral Width	35714.3
21 Lowest Frequency	-2773.1
22 Nucleus	13C
23 Acquired Size	32768
24 Spectral Size	32768

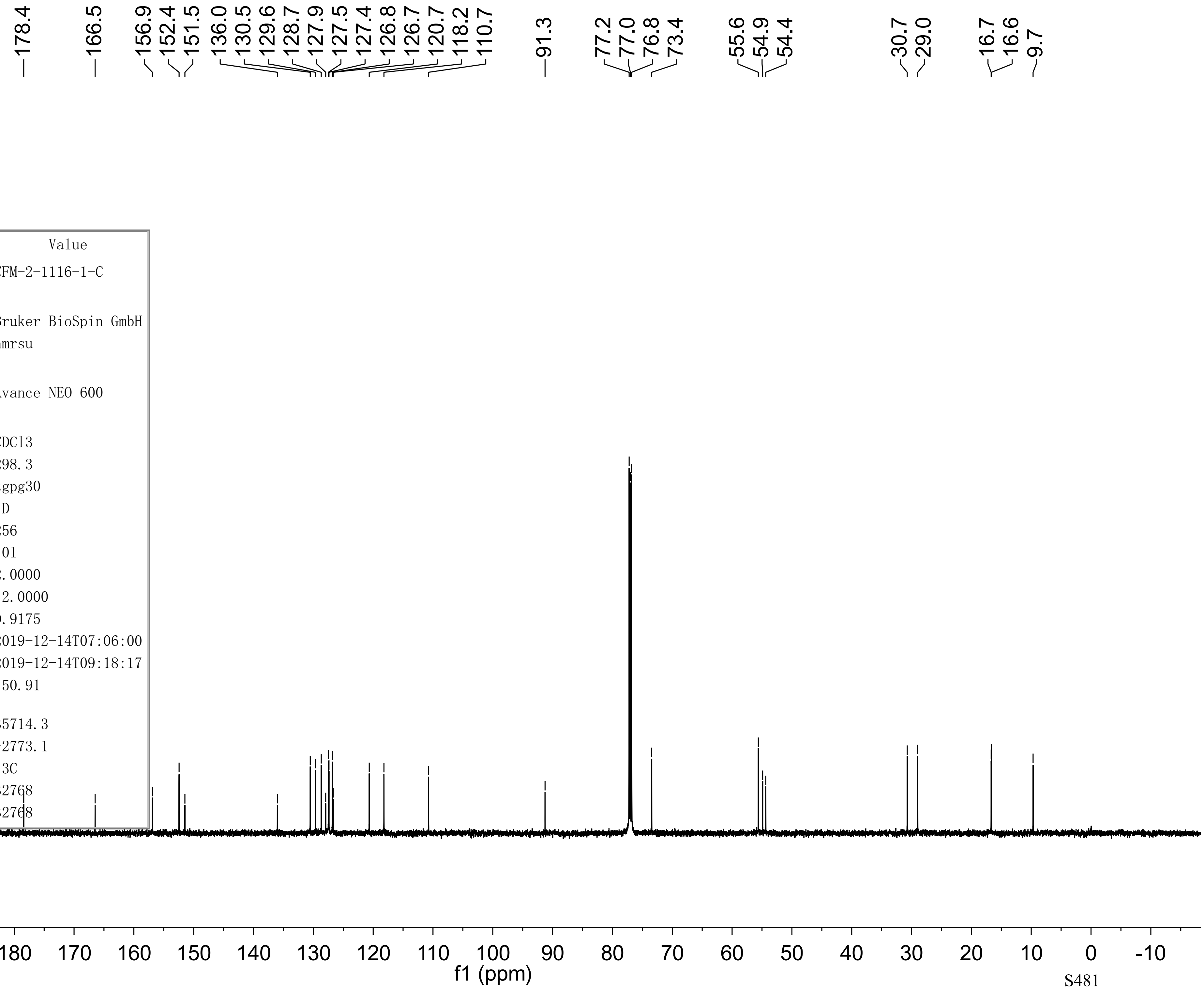

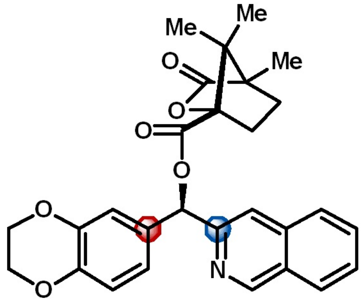

D19

	Parameter	Value
1	Title	CFM-D-A5-2
2	Comment	
3	Origin	Bruker BioSpin GmbH
4	Owner	nmrsu
5	Site	
6	Spectrometer	Avance NEO 600
7	Author	
8	Solvent	CDCl3
9	Temperature	298.1
10	Pulse Sequence	zg30
11	Experiment	1D
12	Number of Scans	8
13	Receiver Gain	101
14	Relaxation Delay	1.0000
15	Pulse Width	10.0000
16	Acquisition Time	2.7525
17	Acquisition Date	2020-08-04T14:36:59
18	Modification Date	2020-08-04T14:51:27
19	Spectrometer Frequency	600.15
20	Spectral Width	11904.8
21	Lowest Frequency	-2261.1
22	Nucleus	1H
23	Acquired Size	32768
24	Spectral Size	65536

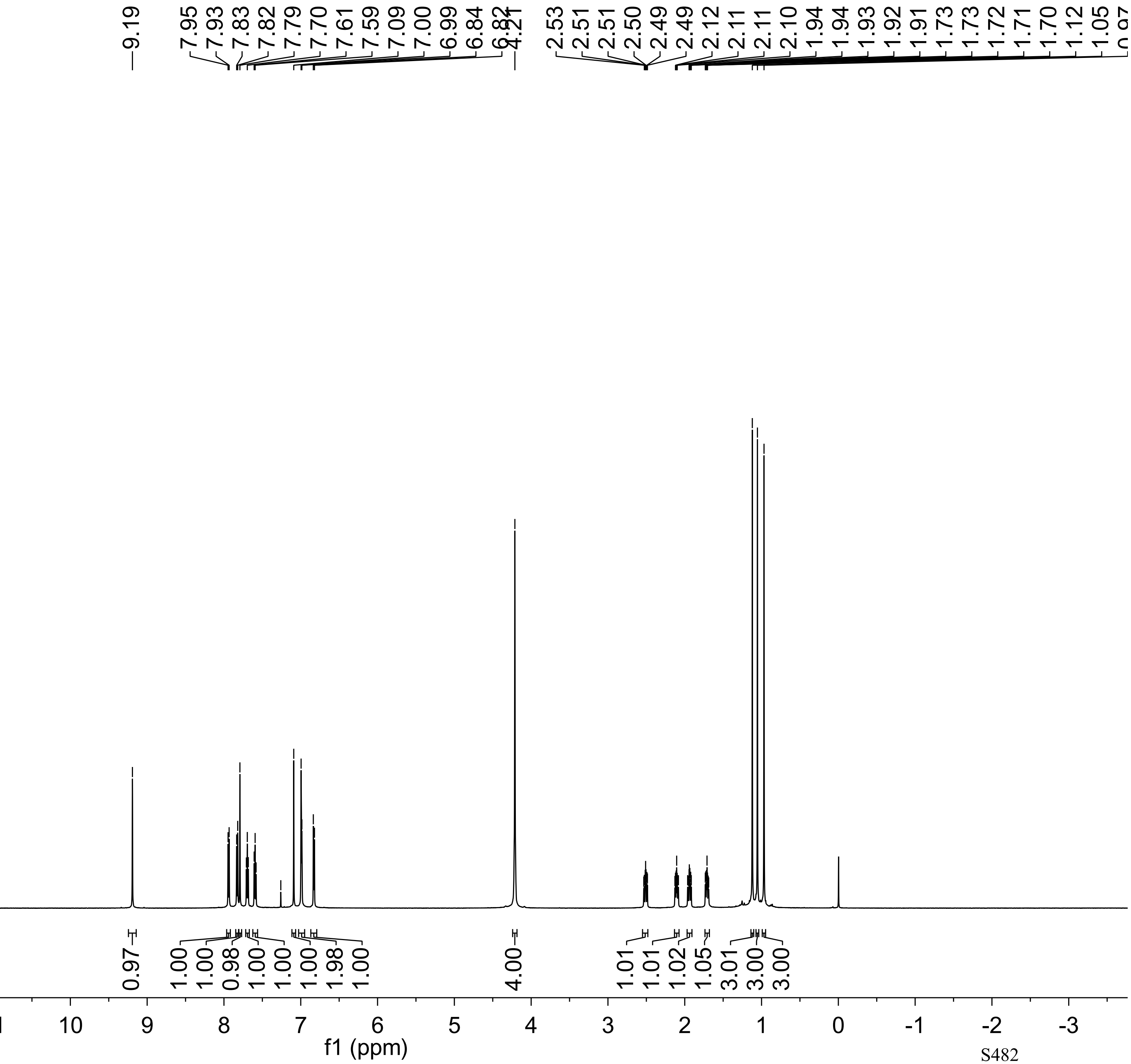

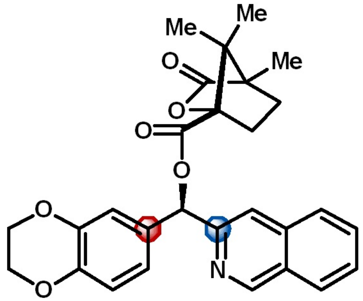

D19

	Parameter	Value
1	Title	CFM-2-1116-4-C
2	Comment	
3	Origin	Bruker BioSpin GmbH
4	Owner	nmrsu
5	Site	
6	Spectrometer	Avance NEO 600
7	Author	
8	Solvent	CDCl3
9	Temperature	298.6
10	Pulse Sequence	zgpg30
11	Experiment	1D
12	Number of Scans	256
13	Receiver Gain	101
14	Relaxation Delay	2.0000
15	Pulse Width	12.0000
16	Acquisition Time	0.9175
17	Acquisition Date	2019-12-14T08:01:35
18	Modification Date	2019-12-14T09:18:24
19	Spectrometer Frequency	150.91
20	Spectral Width	35714.3
21	Lowest Frequency	-2771.5
22	Nucleus	13C
23	Acquired Size	32768
24	Spectral Size	32768

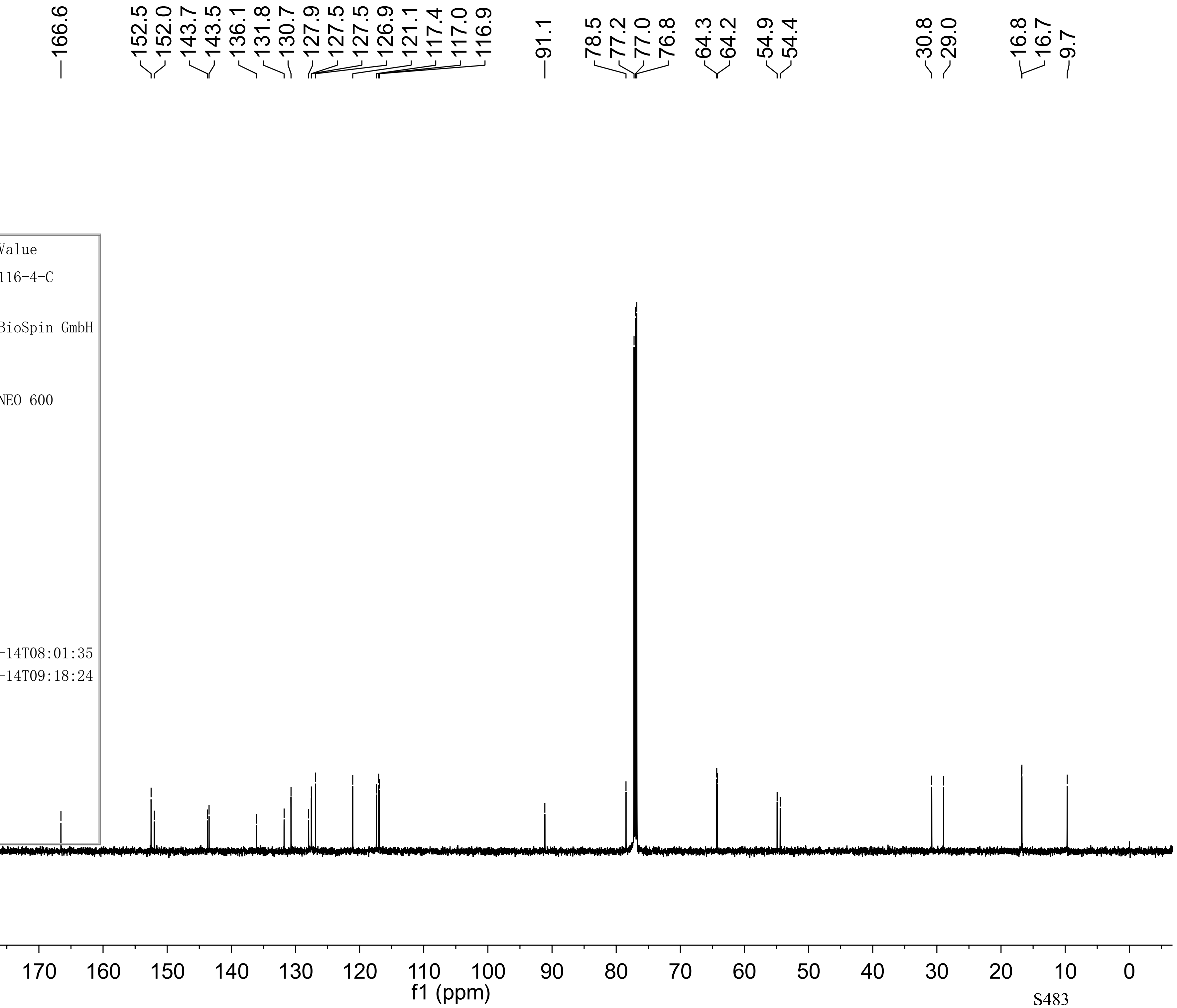

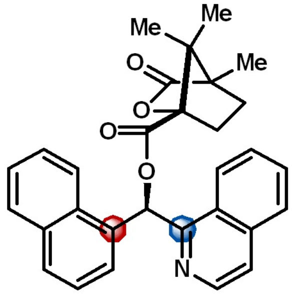

D22

	Parameter	Value
1	Title	CFM-6-D22-0701
2	Comment	
3	Origin	Bruker BioSpin GmbH
4	Owner	nmrsu
5	Site	
6	Spectrometer	AVANCE NEO 400 MHZ DIGITAL NMR SPECTROMETER
7	Author	
8	Solvent	CDCl3
9	Temperature	298.2
10	Pulse Sequence	zg30
11	Experiment	1D
12	Number of Scans	8
13	Receiver Gain	101
14	Spectrometer Frequency	400.13
15	Spectral Width	8196.7
16	Lowest Frequency	-1637.3
17	Nucleus	1H
18	Acquired Size	32768
19	Spectral Size	65536

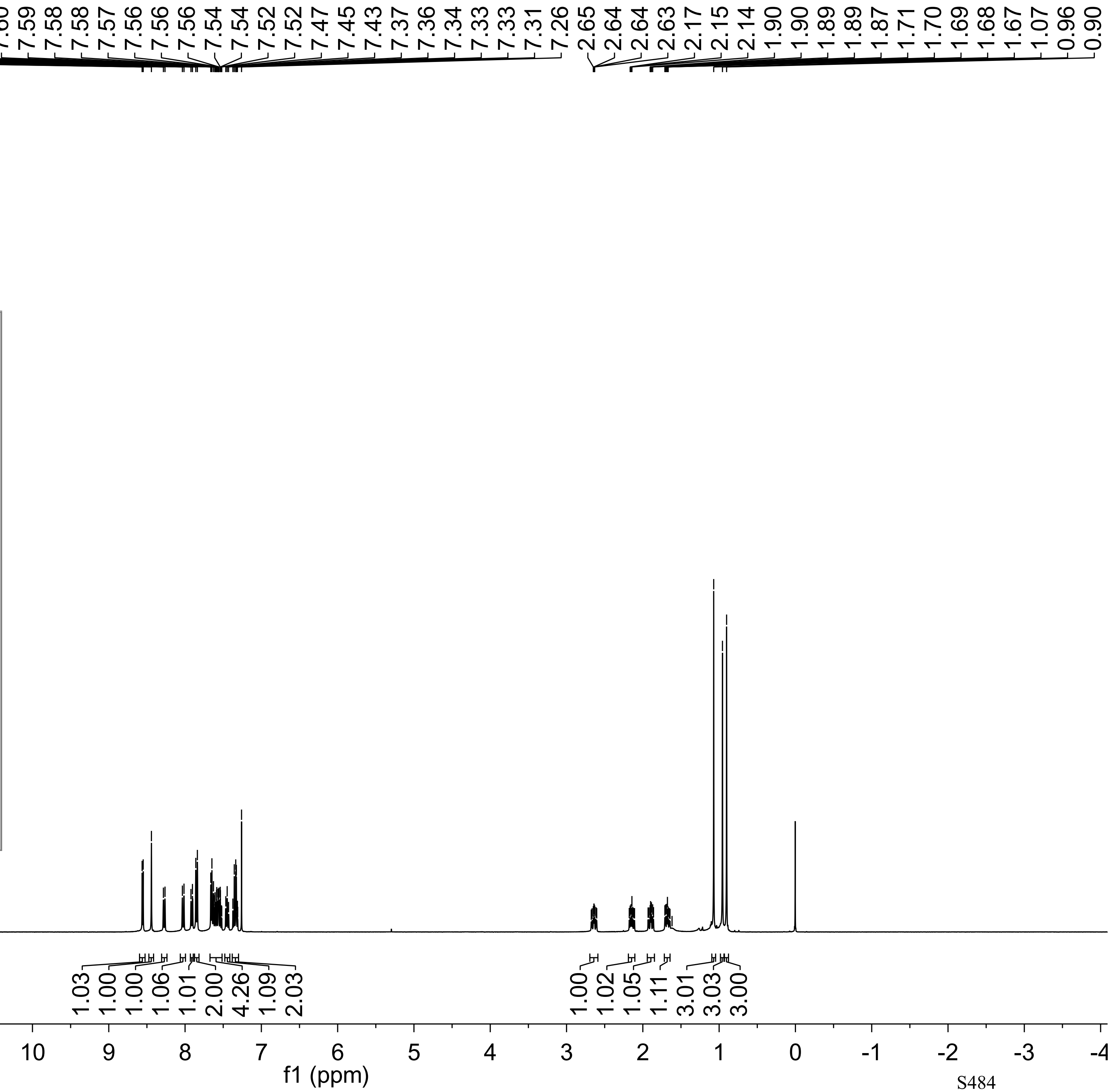

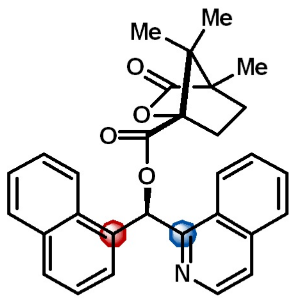

D22

Parameter	Value
1 Title	CFM-6-D22-0701
2 Comment	
3 Origin	Bruker BioSpin GmbH
4 Owner	nmrsu
5 Site	
6 Spectrometer	AVANCE NEO 400 MHZ DIGITAL NMR SPECTROMETER
7 Author	
8 Solvent	CDCl3
9 Temperature	298.2
10 Pulse Sequence	zgpg30
11 Experiment	1D
12 Number of Scans	173
13 Receiver Gain	59
14 Spectrometer Frequency	100.61
15 Spectral Width	23809.5
16 Lowest Frequency	-1847.1
17 Nucleus	13C
18 Acquired Size	32768
19 Spectral Size	32768

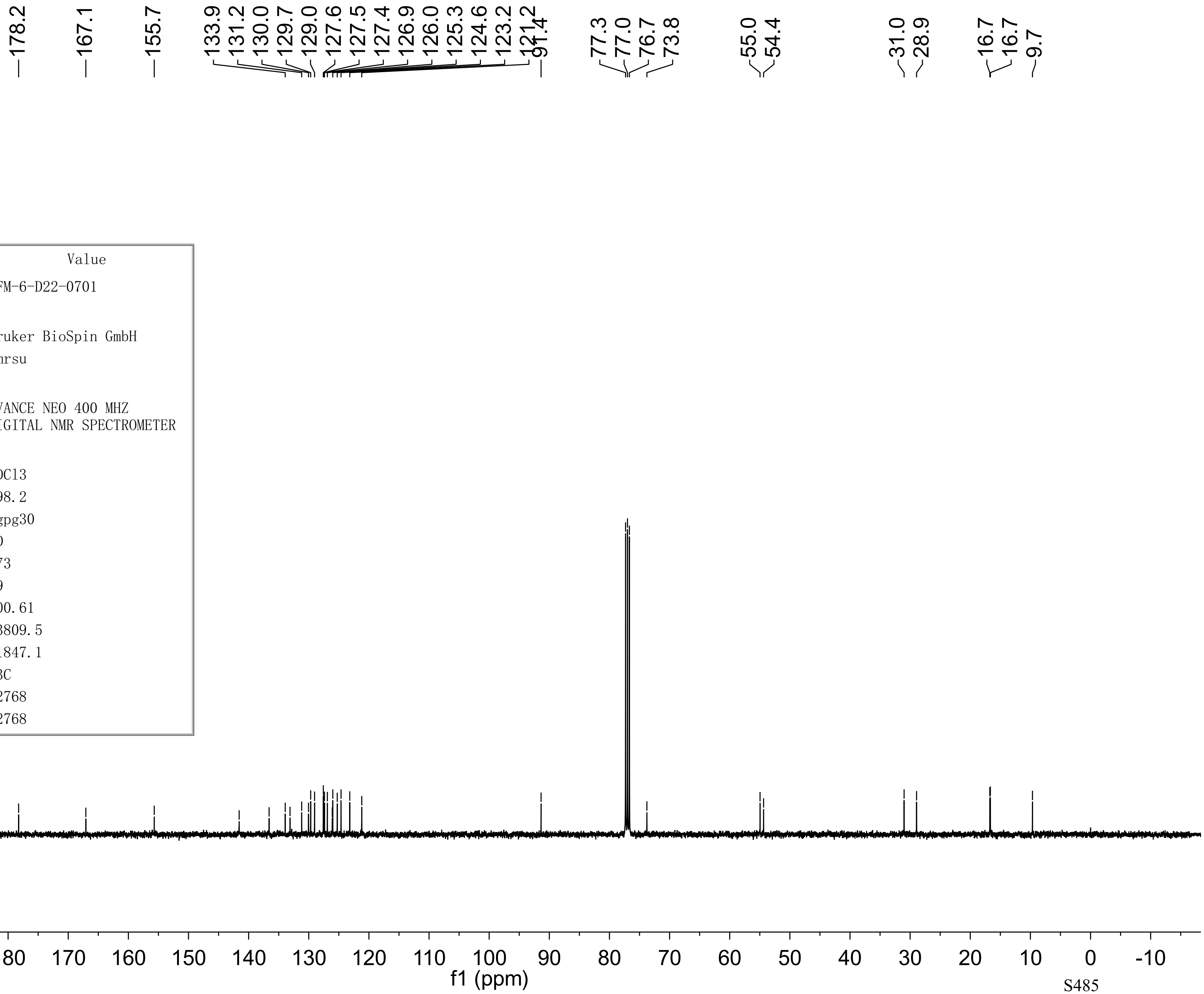

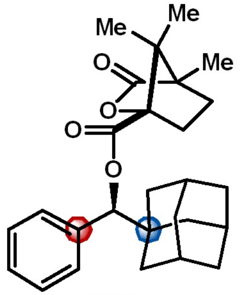

D26

Parameter	Value
1 Title	D-CFM-G6-2
2 Comment	
3 Origin	Bruker BioSpin GmbH
4 Owner	nmrsu
5 Site	
6 Spectrometer	Avance NEO 600
7 Author	
8 Solvent	CDCl3
9 Temperature	296.4
10 Pulse Sequence	zg30
11 Experiment	1D
12 Number of Scans	8
13 Receiver Gain	101
14 Relaxation Delay	1.0000
15 Pulse Width	10.0000
16 Acquisition Time	2.7525
17 Acquisition Date	2020-04-02T08:16:40
18 Modification Date	2020-04-02T09:52:32
19 Spectrometer Frequency	600.15
20 Spectral Width	11904.8
21 Lowest Frequency	-2260.8
22 Nucleus	1H
23 Acquired Size	32768
24 Spectral Size	65536

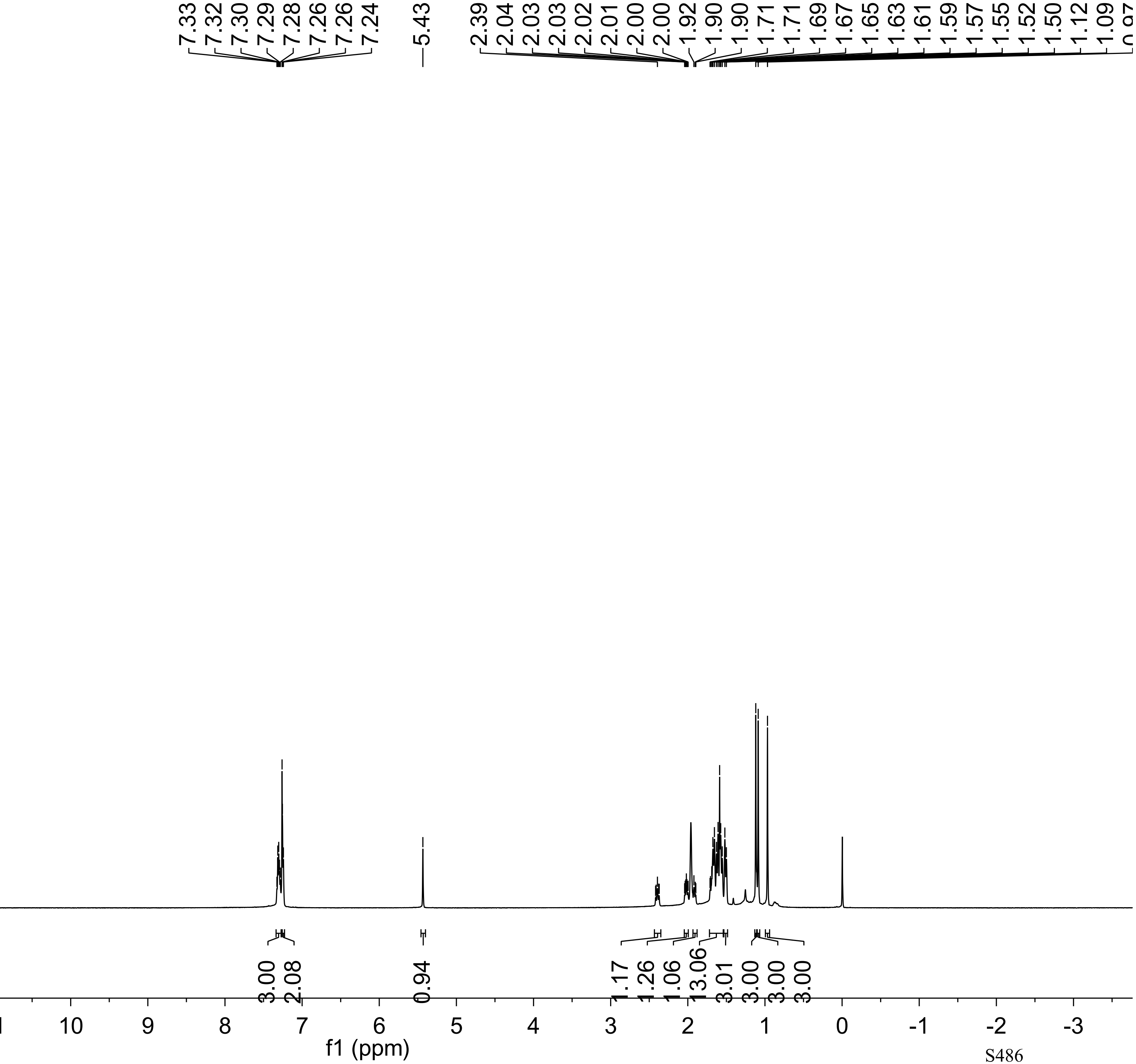

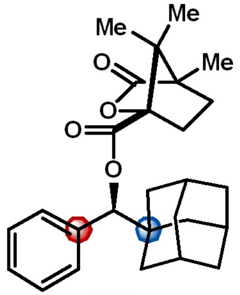

D26

Parameter	Value
1 Title	D-CFM-G6-2
2 Comment	
3 Origin	Bruker BioSpin GmbH
4 Owner	nmrsu
5 Site	
6 Spectrometer	Avance NEO 600
7 Author	
8 Solvent	CDCl3
9 Temperature	297.7
10 Pulse Sequence	zgpg30
11 Experiment	1D
12 Number of Scans	128
13 Receiver Gain	101
14 Relaxation Delay	2.0000
15 Pulse Width	12.0000
16 Acquisition Time	0.9175
17 Acquisition Date	2020-04-02T08:24:08
18 Modification Date	2020-04-02T09:52:32
19 Spectrometer Frequency	150.91
20 Spectral Width	35714.3
21 Lowest Frequency	-2771.8
22 Nucleus	13C
23 Acquired Size	32768
24 Spectral Size	32768

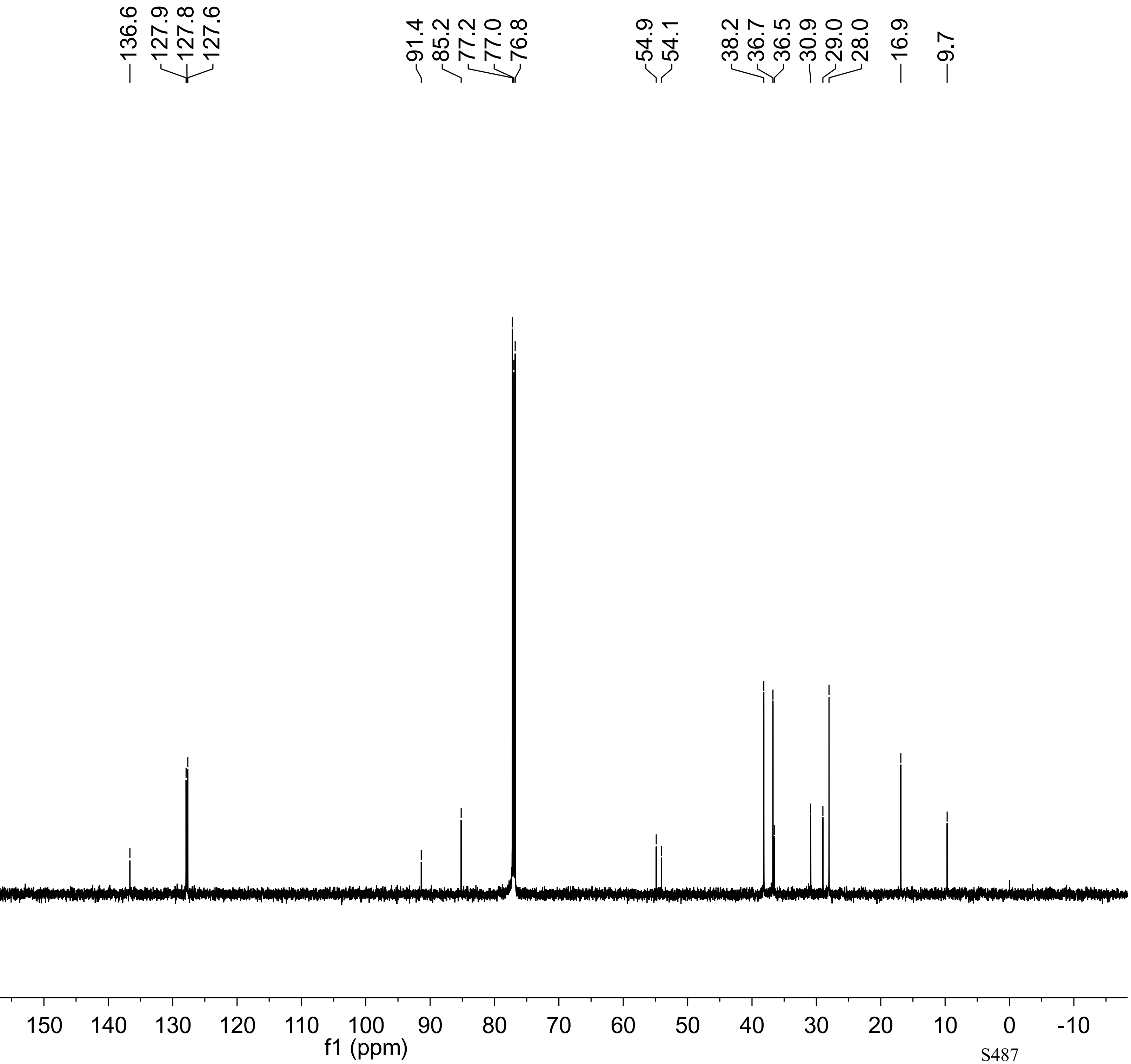

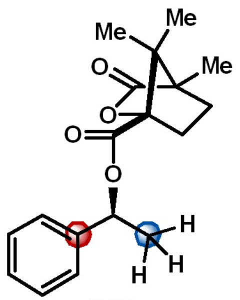

D28

Parameter		Value
1	Title	CFM-D28
2	Comment	
3	Origin	Bruker BioSpin GmbH
4	Owner	nmrsu
5	Site	
6	Spectrometer	Avance NEO 600
7	Author	
8	Solvent	CDCl3
9	Temperature	298.2
10	Pulse Sequence	zg30
11	Experiment	1D
12	Number of Scans	8
13	Receiver Gain	101
14	Spectrometer Frequency	600.15
15	Spectral Width	11904.8
16	Lowest Frequency	-2259.7
17	Nucleus	1H
18	Acquired Size	32768
19	Spectral Size	65536

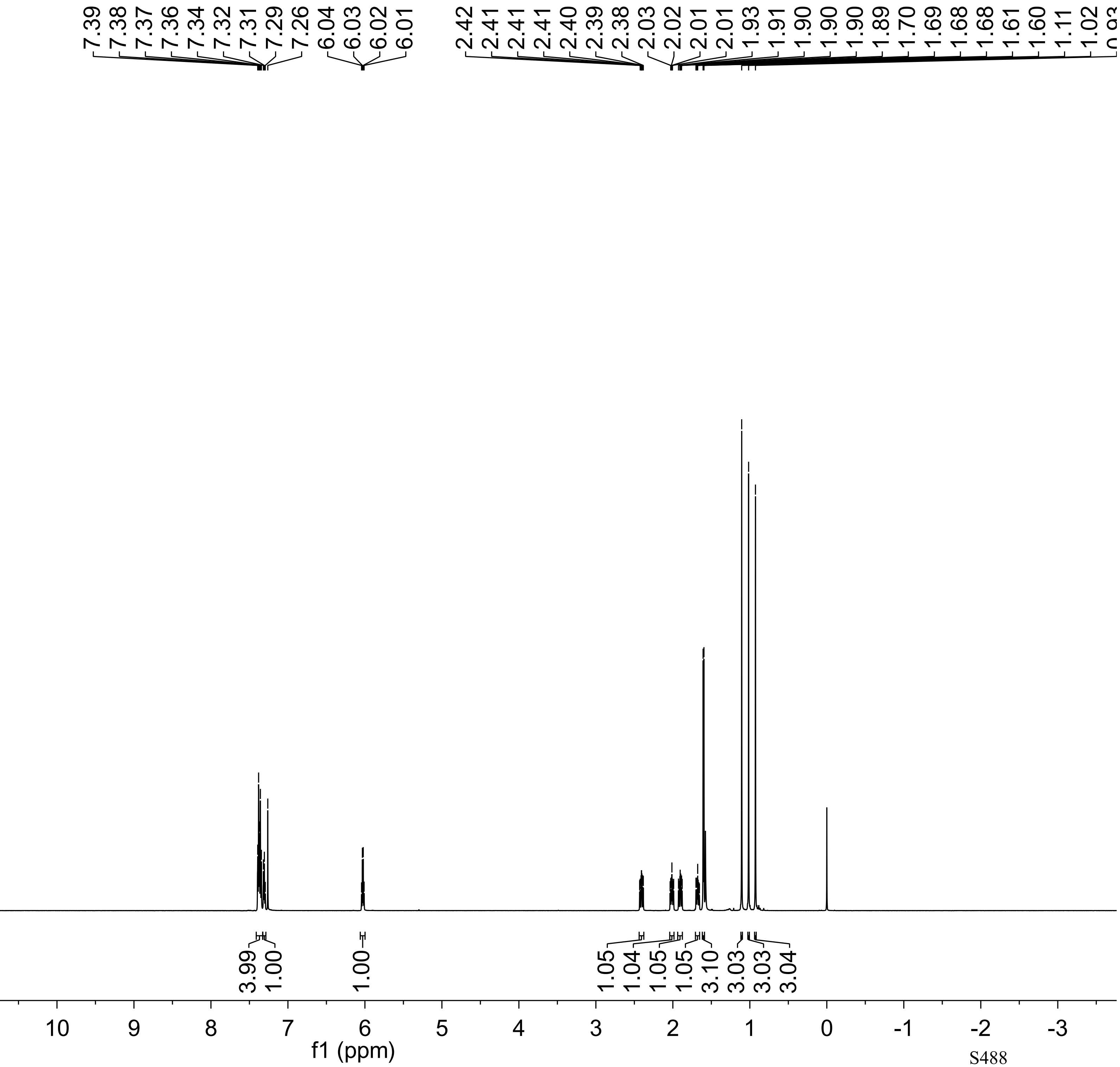

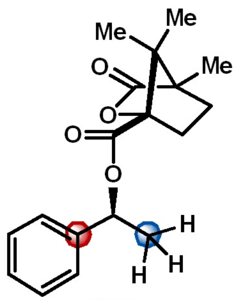

D28

Parameter	Value
1 Title	CFM-D28
2 Comment	
3 Origin	Bruker BioSpin GmbH
4 Owner	nmrsu
5 Site	
6 Spectrometer	Avance NEO 600
7 Author	
8 Solvent	CDCl3
9 Temperature	298.1
10 Pulse Sequence	zgpg30
11 Experiment	1D
12 Number of Scans	64
13 Receiver Gain	101
14 Spectrometer Frequency	150.91
15 Spectral Width	35714.3
16 Lowest Frequency	-2770.7
17 Nucleus	13C
18 Acquired Size	32768
19 Spectral Size	32768

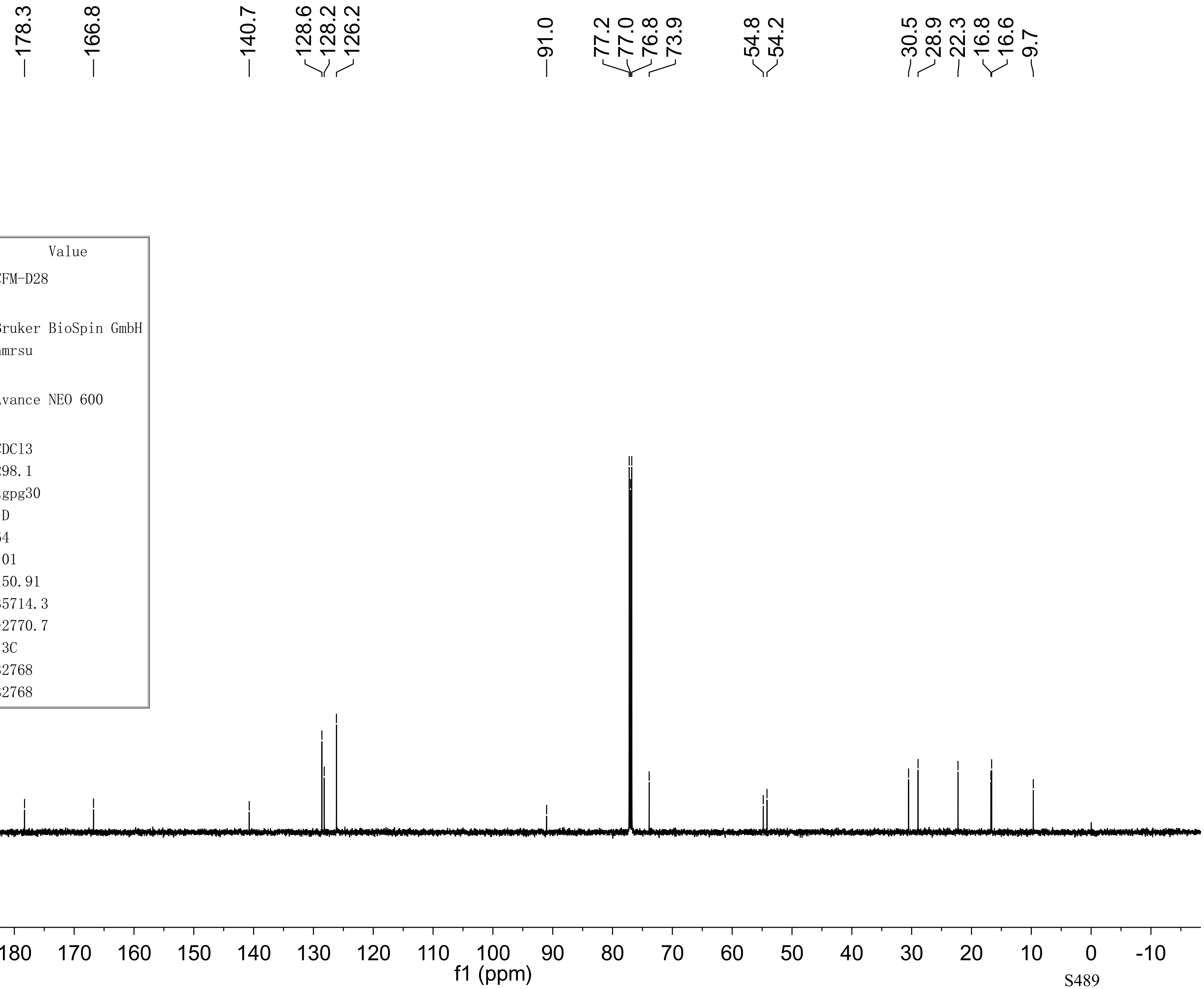

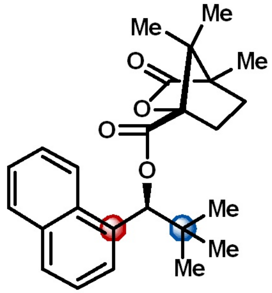

D30

	Parameter	Value
1	Title	D-CFM-G4
2	Comment	
3	Origin	Bruker BioSpin GmbH
4	Owner	nmrsu
5	Site	
6	Spectrometer	Avance NEO 600
7	Author	
8	Solvent	CDCl3
9	Temperature	296.7
10	Pulse Sequence	zg30
11	Experiment	1D
12	Number of Scans	16
13	Receiver Gain	101
14	Relaxation Delay	1.0000
15	Pulse Width	10.0000
16	Acquisition Time	2.7525
17	Acquisition Date	2019-12-29T00:41:55
18	Modification Date	2019-12-29T10:16:21
19	Spectrometer Frequency	600.15
20	Spectral Width	11904.8
21	Lowest Frequency	-2261.3
22	Nucleus	1H
23	Acquired Size	32768
24	Spectral Size	65536

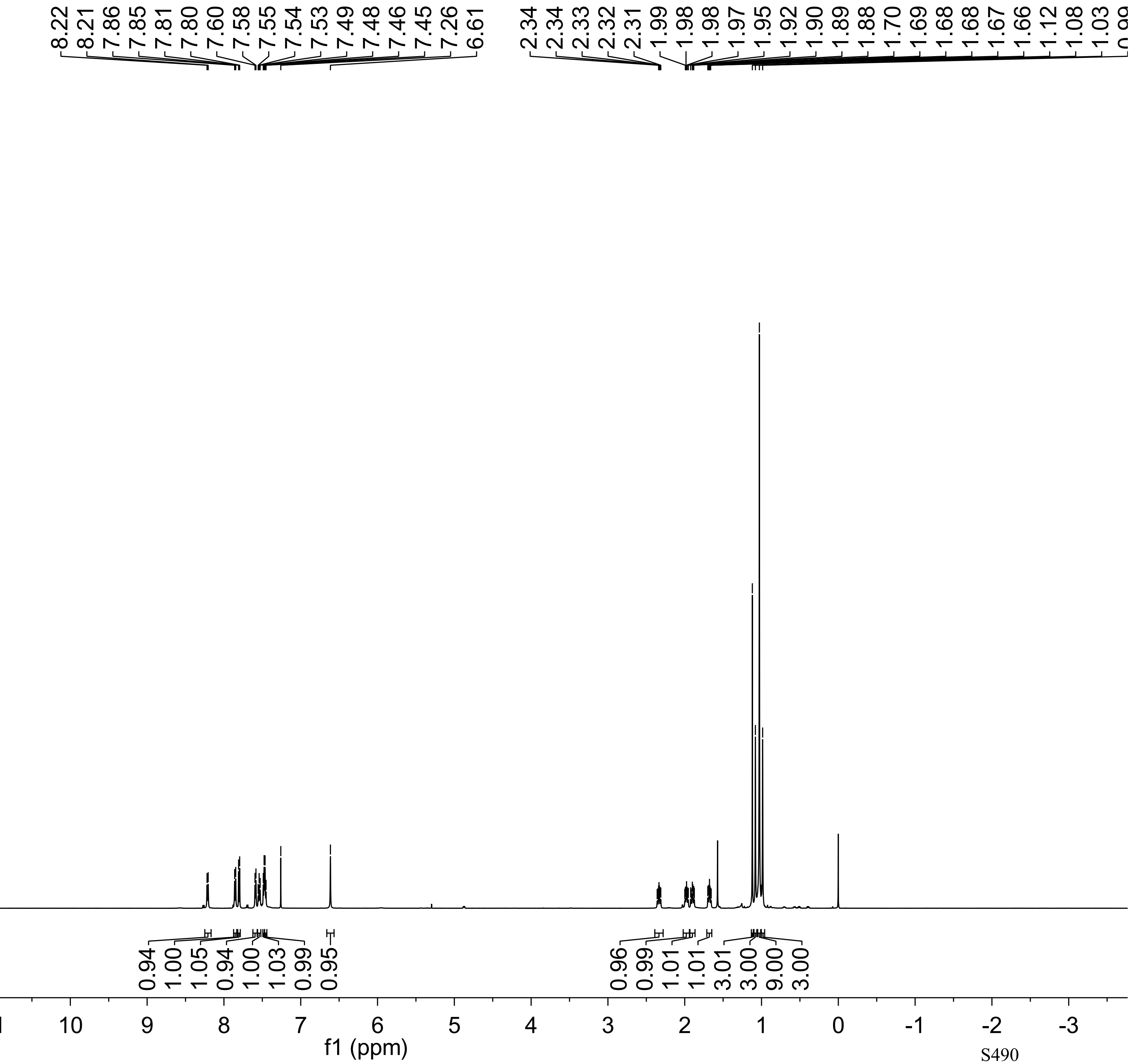

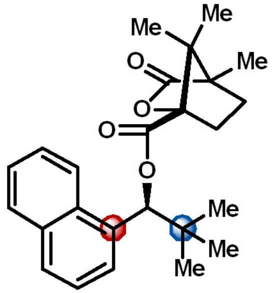

D30

	Parameter	Value
1	Title	D-CFM-G4
2	Comment	
3	Origin	Bruker BioSpin GmbH
4	Owner	nmrsu
5	Site	
6	Spectrometer	Avance NEO 600
7	Author	
8	Solvent	CDCl3
9	Temperature	297.8
10	Pulse Sequence	zgpg30
11	Experiment	1D
12	Number of Scans	256
13	Receiver Gain	101
14	Relaxation Delay	2.0000
15	Pulse Width	12.0000
16	Acquisition Time	0.9175
17	Acquisition Date	2019-12-29T00:55:52
18	Modification Date	2019-12-29T10:16:22
19	Spectrometer Frequency	150.91
20	Spectral Width	35714.3
21	Lowest Frequency	-2772.2
22	Nucleus	13C
23	Acquired Size	32768
24	Spectral Size	32768

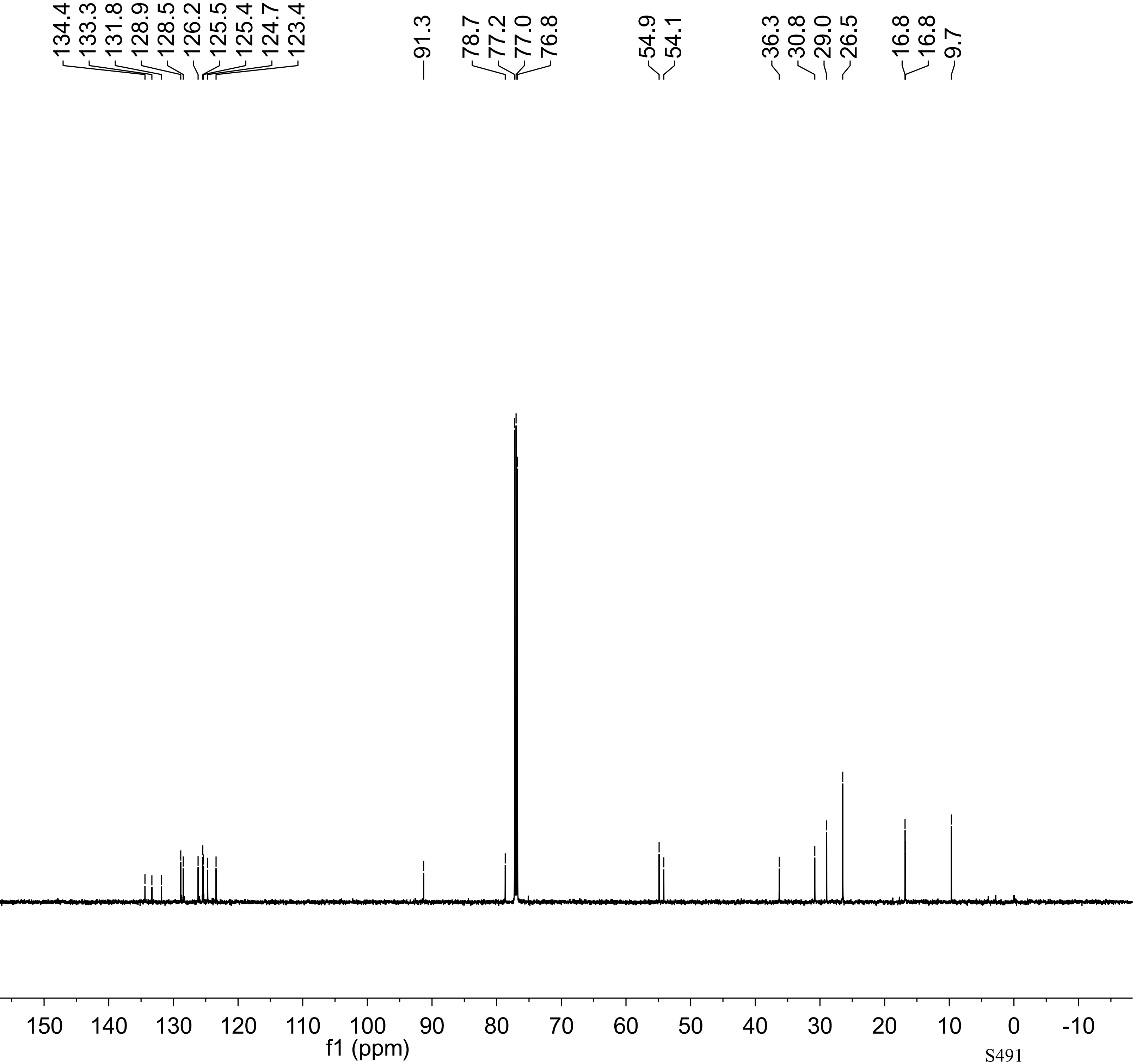

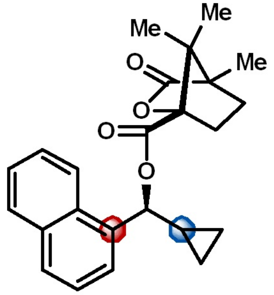

D31

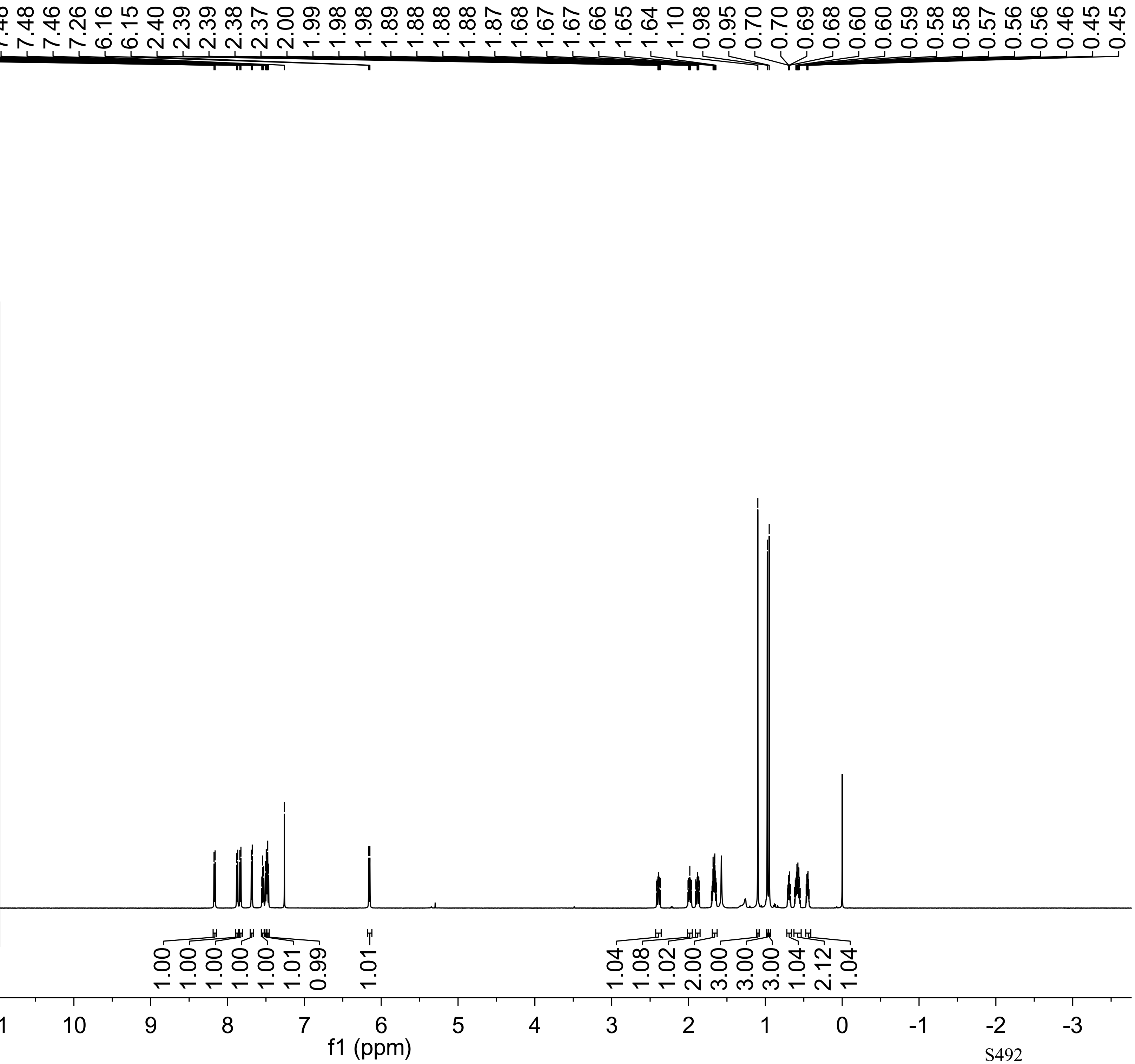

	Parameter	Value
1	Title	D-CFM-G3
2	Comment	
3	Origin	Bruker BioSpin GmbH
4	Owner	nmrsu
5	Site	
6	Spectrometer	Avance NEO 600
7	Author	
8	Solvent	CDCl3
9	Temperature	296.9
10	Pulse Sequence	zg30
11	Experiment	1D
12	Number of Scans	16
13	Receiver Gain	101
14	Relaxation Delay	1.0000
15	Pulse Width	10.0000
16	Acquisition Time	2.7525
17	Acquisition Date	2019-12-30T01:43:54
18	Modification Date	2019-12-30T08:57:28
19	Spectrometer Frequency	600.15
20	Spectral Width	11904.8
21	Lowest Frequency	-2261.2
22	Nucleus	1H
23	Acquired Size	32768
24	Spectral Size	65536

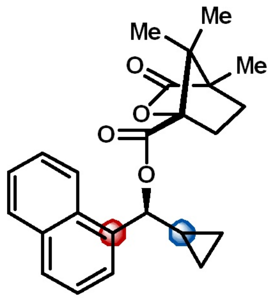

D31

	Parameter	Value
1	Title	D-CFM-G3
2	Comment	
3	Origin	Bruker BioSpin GmbH
4	Owner	nmrsu
5	Site	
6	Spectrometer	Avance NEO 600
7	Author	
8	Solvent	CDCl3
9	Temperature	298.0
10	Pulse Sequence	zgpg30
11	Experiment	1D
12	Number of Scans	512
13	Receiver Gain	101
14	Relaxation Delay	2.0000
15	Pulse Width	12.0000
16	Acquisition Time	0.9175
17	Acquisition Date	2019-12-30T02:10:28
18	Modification Date	2019-12-30T08:57:29
19	Spectrometer Frequency	150.91
20	Spectral Width	35714.3
21	Lowest Frequency	-2770.9
22	Nucleus	13C
23	Acquired Size	32768
24	Spectral Size	32768

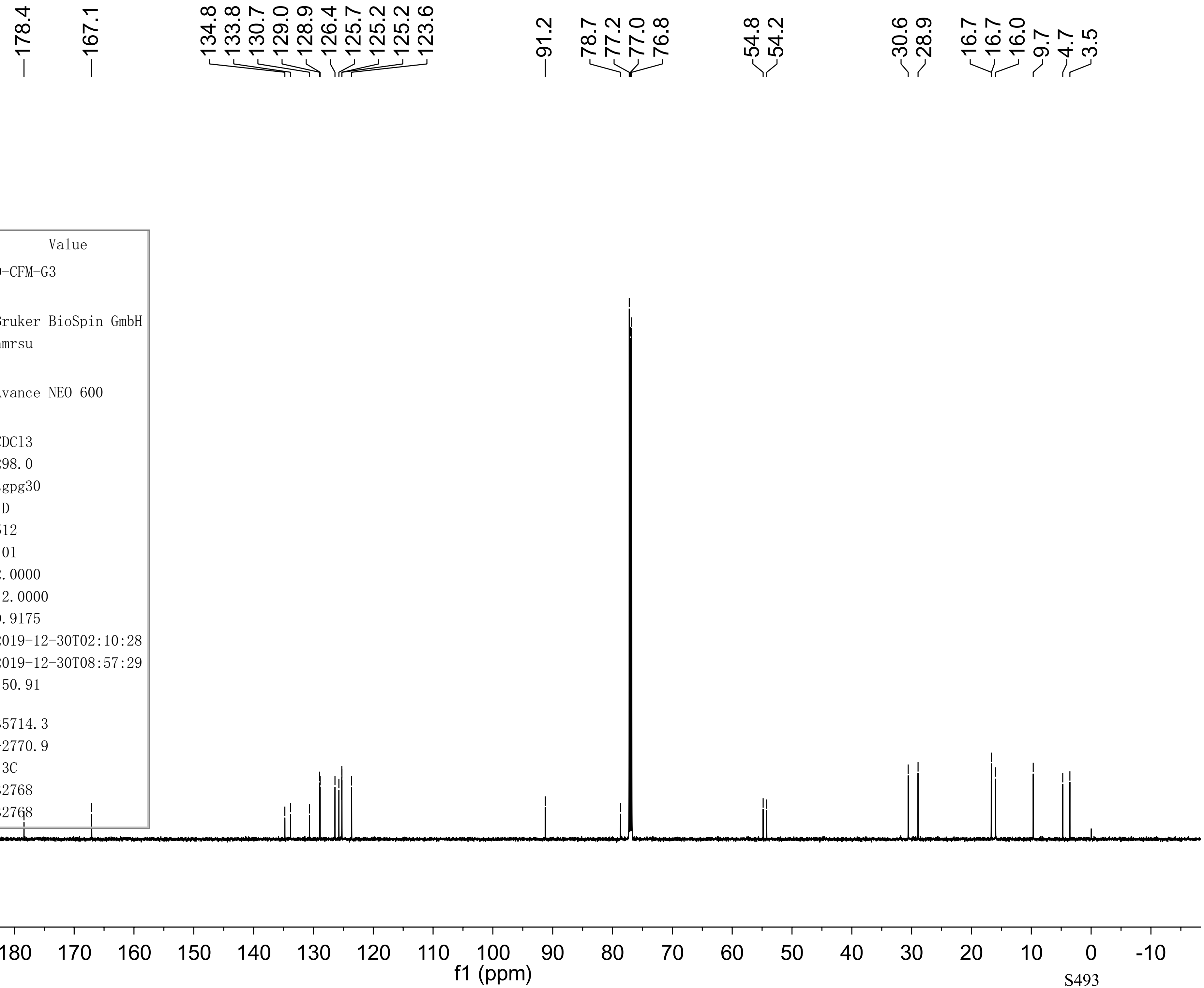

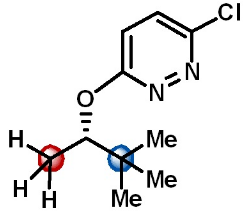

D38

Parameter	Value
1 Title	CFM-D41
2 Comment	
3 Origin	Bruker BioSpin GmbH
4 Owner	nmrsu
5 Site	
6 Spectrometer	Avance NEO 600
7 Author	
8 Solvent	CDCl3
9 Temperature	298.1
10 Pulse Sequence	zg30
11 Experiment	1D
12 Number of Scans	8
13 Receiver Gain	90
14 Spectrometer Frequency	600.15
15 Spectral Width	11904.8
16 Lowest Frequency	-2260.8
17 Nucleus	1H
18 Acquired Size	32768
19 Spectral Size	65536

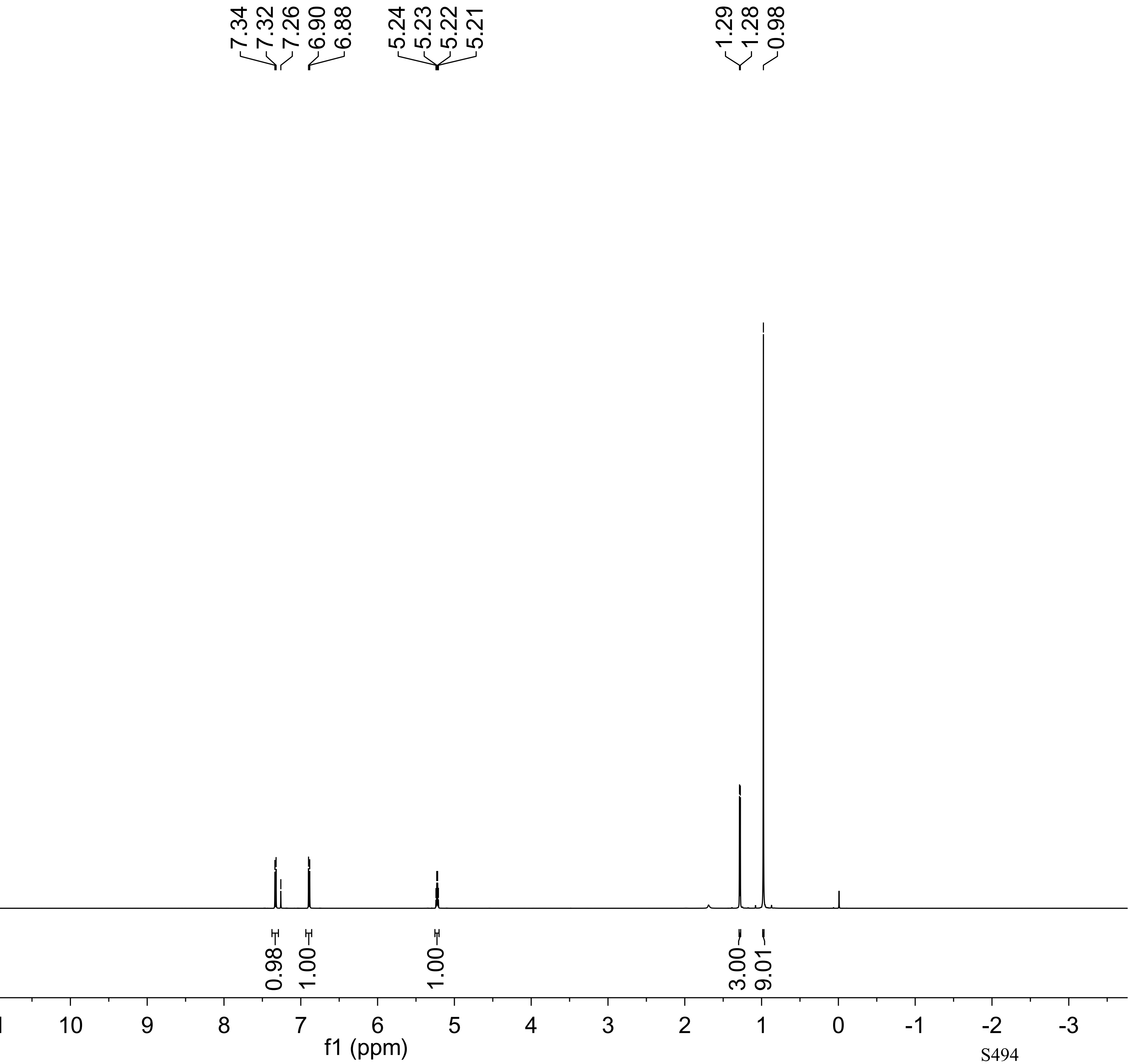

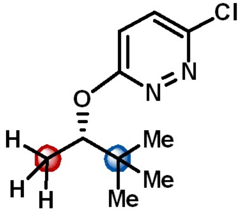

D38

	Parameter	Value
1	Title	CFM-D41
2	Comment	
3	Origin	Bruker BioSpin GmbH
4	Owner	nmrsu
5	Site	
6	Spectrometer	Avance NEO 600
7	Author	
8	Solvent	CDCl3
9	Temperature	298.2
10	Pulse Sequence	zgpg30
11	Experiment	1D
12	Number of Scans	64
13	Receiver Gain	101
14	Spectrometer Frequency	150.91
15	Spectral Width	35714.3
16	Lowest Frequency	-2770.7
17	Nucleus	13C
18	Acquired Size	32768
19	Spectral Size	32768

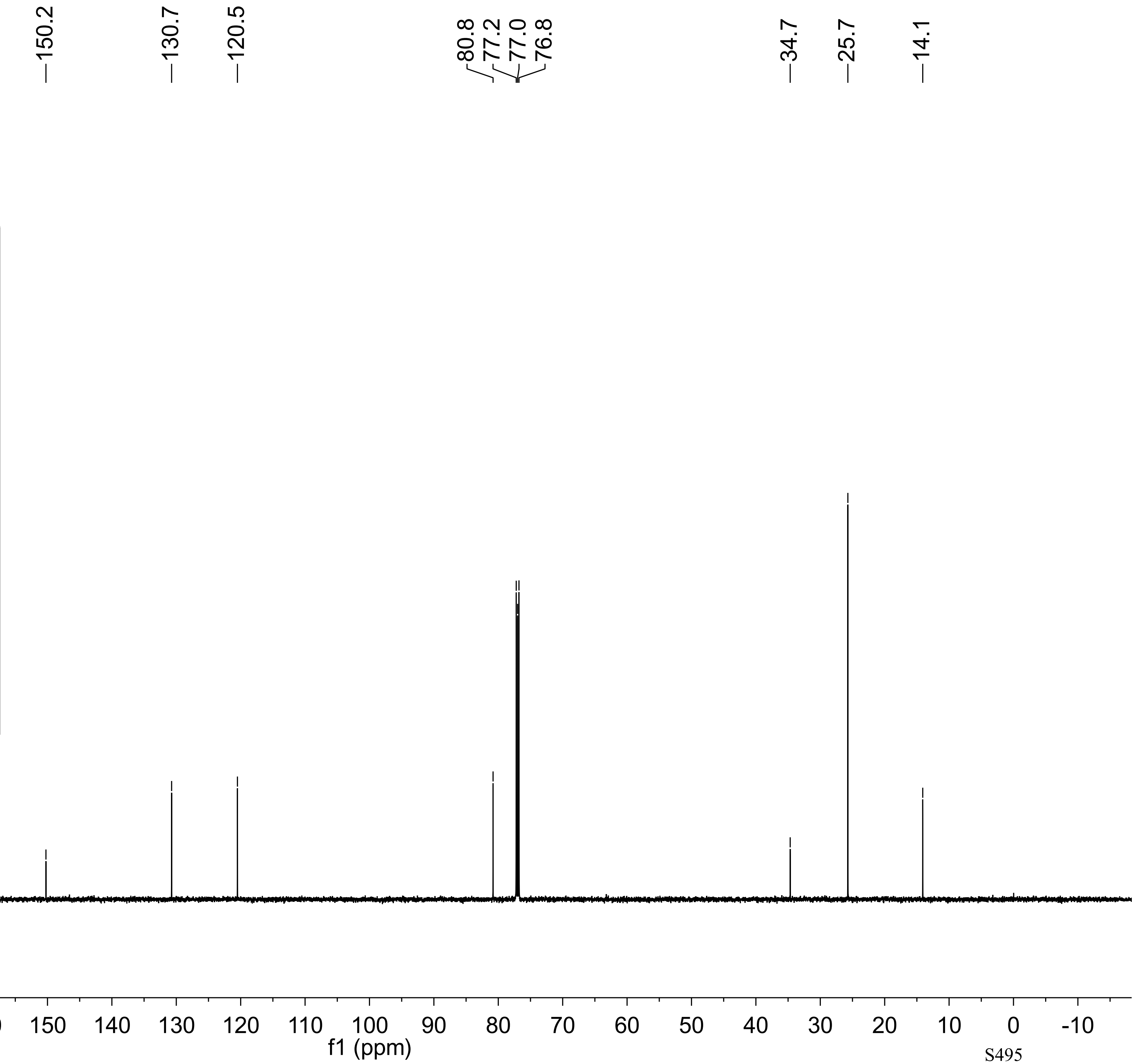

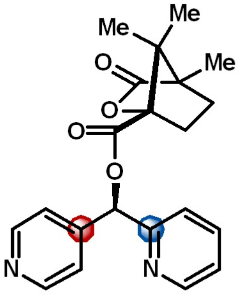

D40

	Parameter	Value
1	Title	CFM-6-80
2	Comment	
3	Origin	Bruker BioSpin GmbH
4	Owner	nmrsu
5	Site	
6	Spectrometer	Avance NEO 600
7	Author	
8	Solvent	CDCl3
9	Temperature	298.1
10	Pulse Sequence	zg30
11	Experiment	1D
12	Number of Scans	8
13	Receiver Gain	64
14	Spectrometer Frequency	600.15
15	Spectral Width	11904.8
16	Lowest Frequency	-2260.8
17	Nucleus	1H
18	Acquired Size	32768
19	Spectral Size	65536

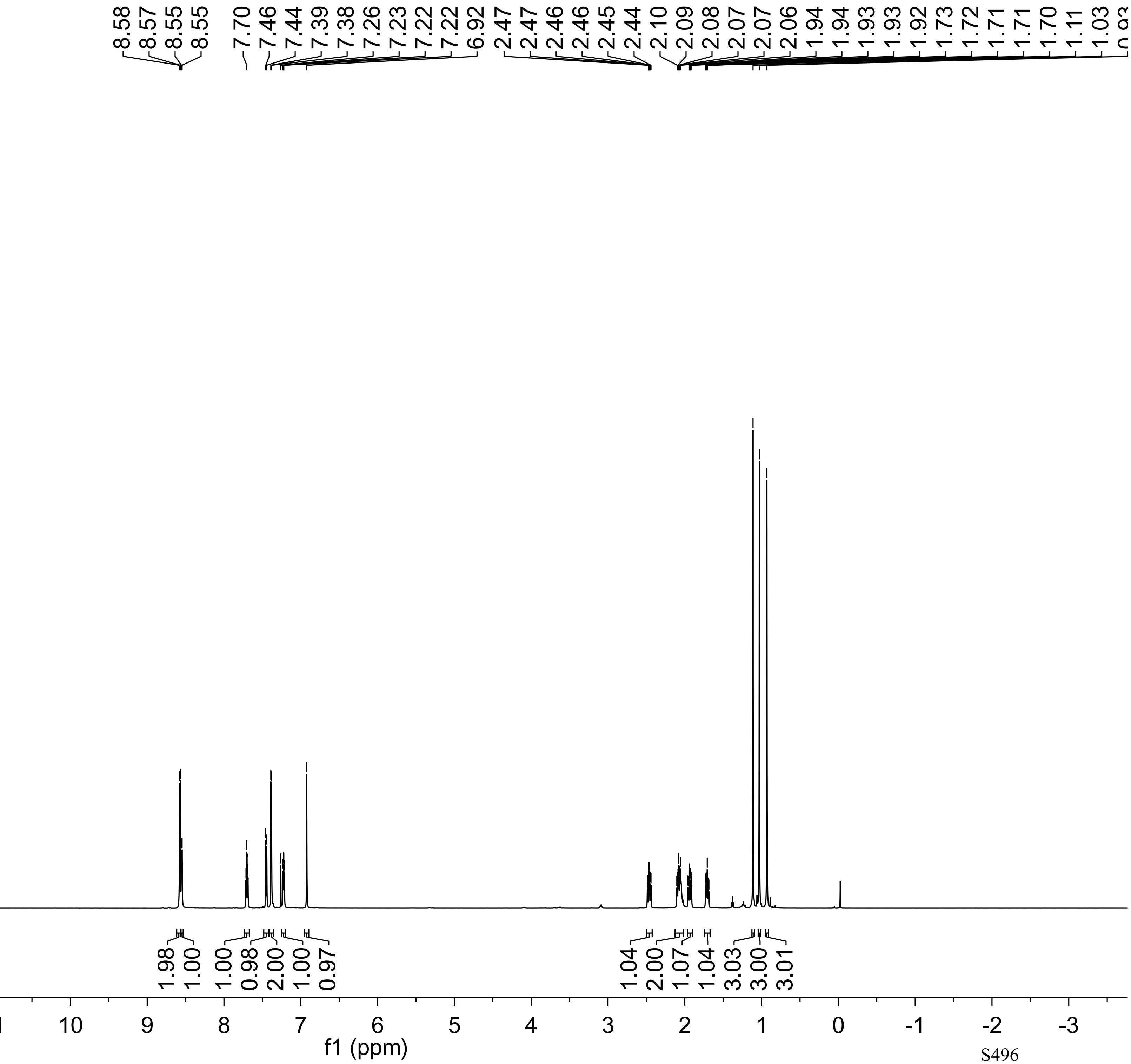

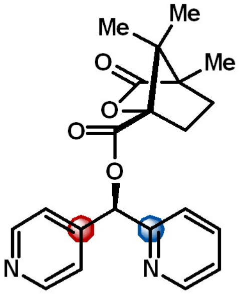

D40

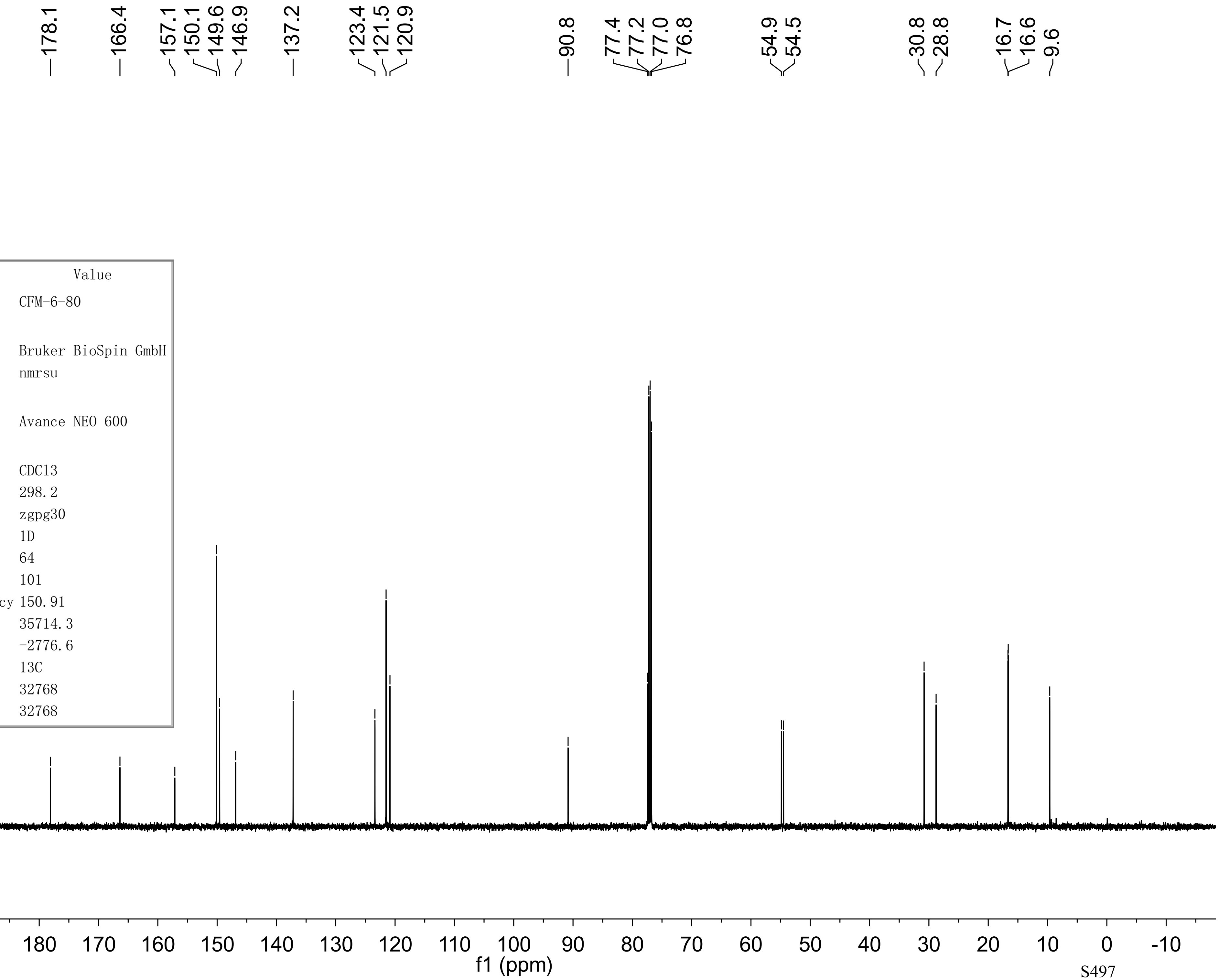

	Parameter	Value
1	Title	CFM-6-80
2	Comment	
3	Origin	Bruker BioSpin GmbH
4	Owner	nmrsu
5	Site	
6	Spectrometer	Avance NEO 600
7	Author	
8	Solvent	CDCl3
9	Temperature	298.2
10	Pulse Sequence	zgpg30
11	Experiment	1D
12	Number of Scans	64
13	Receiver Gain	101
14	Spectrometer Frequency	150.91
15	Spectral Width	35714.3
16	Lowest Frequency	-2776.6
17	Nucleus	13C
18	Acquired Size	32768
19	Spectral Size	32768

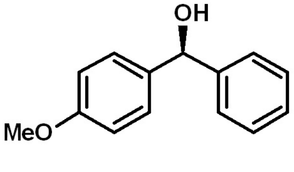

C49

Parameter	Value
1 Title	QJ-1-40A
2 Comment	QJ-1-40A
3 Origin	Bruker BioSpin GmbH
4 Owner	nmrsu
5 Site	
6 Spectrometer	Avance Neo 400M
7 Author	
8 Solvent	CDCl3
9 Temperature	298.1
10 Pulse Sequence	zg30
11 Experiment	1D
12 Number of Scans	6
13 Receiver Gain	65
14 Spectrometer Frequency	400.18
15 Spectral Width	8196.7
16 Lowest Frequency	-1627.3
17 Nucleus	1H
18 Acquired Size	32768
19 Spectral Size	65536

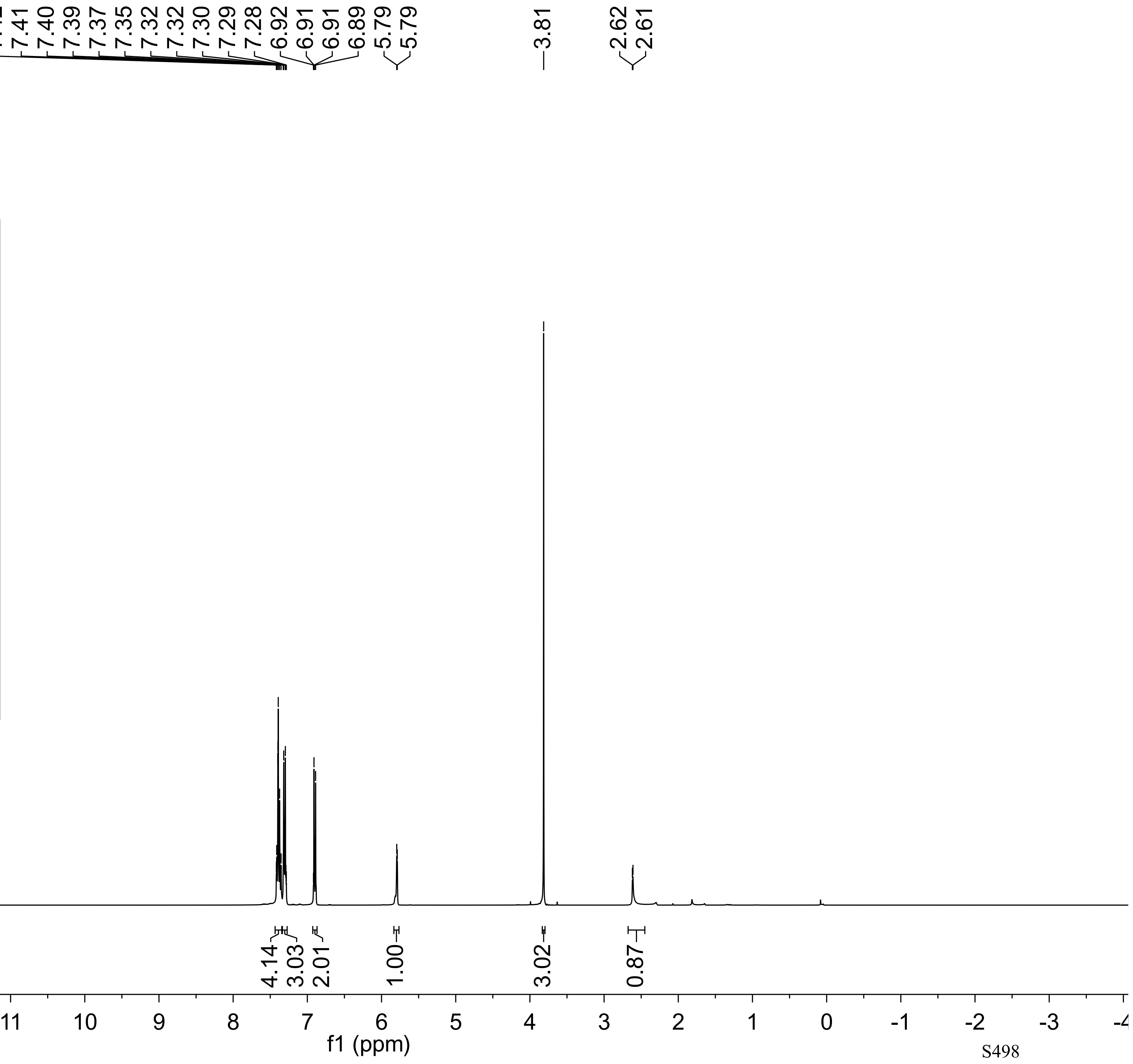

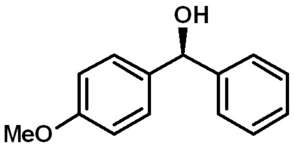

C49

	Parameter	Value
1	Title	QJ-1-40A-C
2	Comment	QJ-1-40A-C
3	Origin	Bruker BioSpin GmbH
4	Owner	nmrsu
5	Site	
6	Spectrometer	Avance Neo 400M
7	Author	
8	Solvent	CDCl3
9	Temperature	298.2
10	Pulse Sequence	zgpg30
11	Experiment	1D
12	Number of Scans	61
13	Receiver Gain	34
14	Spectrometer Frequency	100.63
15	Spectral Width	23809.5
16	Lowest Frequency	-1842.2
17	Nucleus	13C
18	Acquired Size	32768
19	Spectral Size	32768

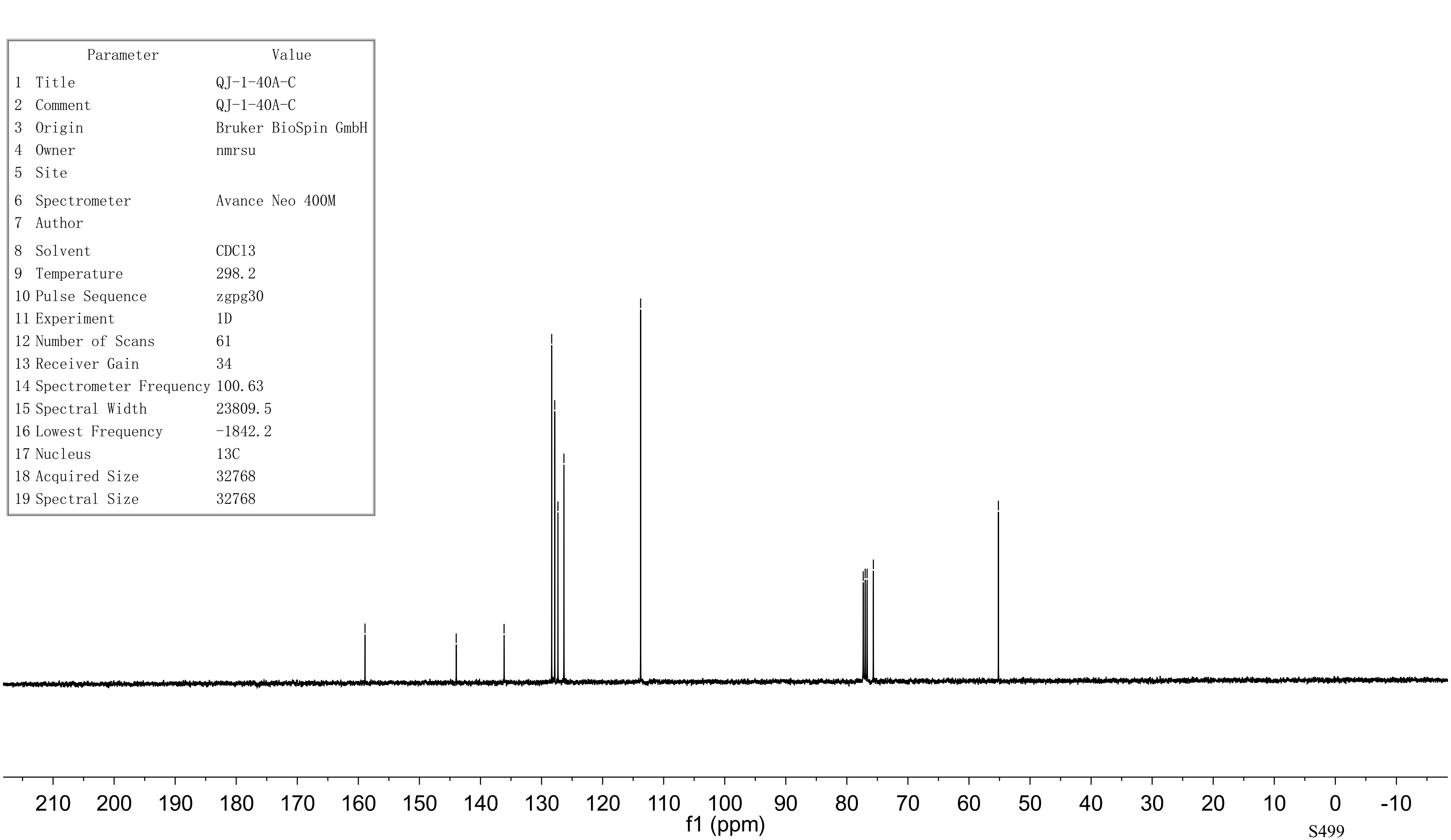

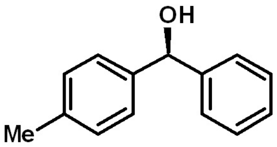

C50

	Parameter	Value
1	Title	QJ-1-43E-1
2	Comment	QJ-1-43E-1
3	Origin	Bruker BioSpin GmbH
4	Owner	nmrsu
5	Site	
6	Spectrometer	Avance Neo 400M
7	Author	
8	Solvent	CDCl3
9	Temperature	298.2
10	Pulse Sequence	zg30
11	Experiment	1D
12	Number of Scans	6
13	Receiver Gain	32
14	Spectrometer Frequency	400.18
15	Spectral Width	8196.7
16	Lowest Frequency	-1636.1
17	Nucleus	1H
18	Acquired Size	32768
19	Spectral Size	65536

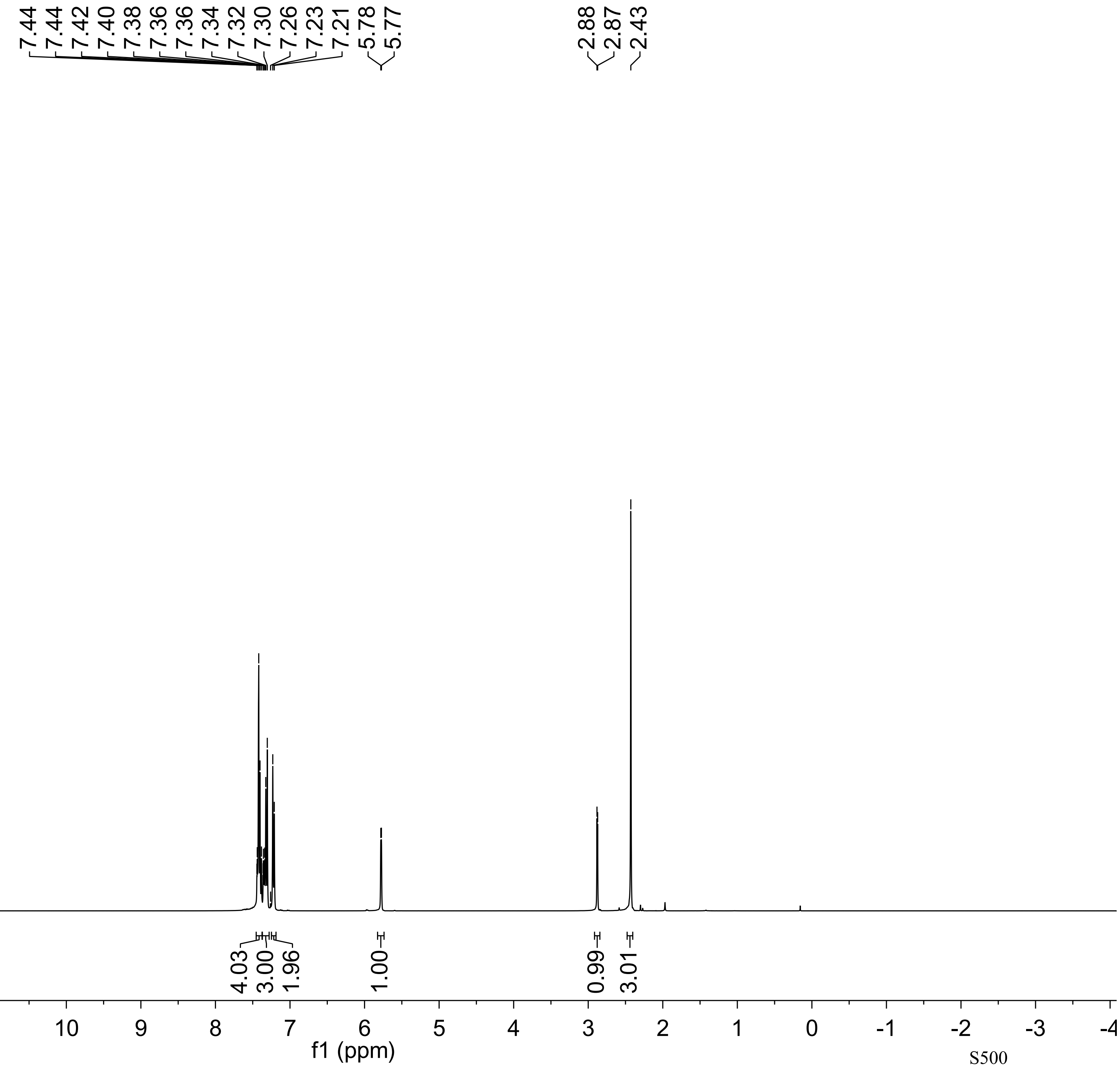

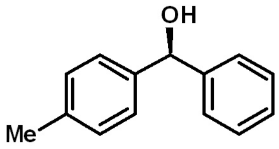

C50

	Parameter	Value
1	Title	QJ-1-43E-1
2	Comment	QJ-1-43E-1
3	Origin	Bruker BioSpin GmbH
4	Owner	nmrsu
5	Site	
6	Spectrometer	Avance Neo 400M
7	Author	
8	Solvent	CDCl3
9	Temperature	298.3
10	Pulse Sequence	zgpg30
11	Experiment	1D
12	Number of Scans	22
13	Receiver Gain	24
14	Spectrometer Frequency	100.63
15	Spectral Width	23809.5
16	Lowest Frequency	-1842.2
17	Nucleus	13C
18	Acquired Size	32768
19	Spectral Size	32768

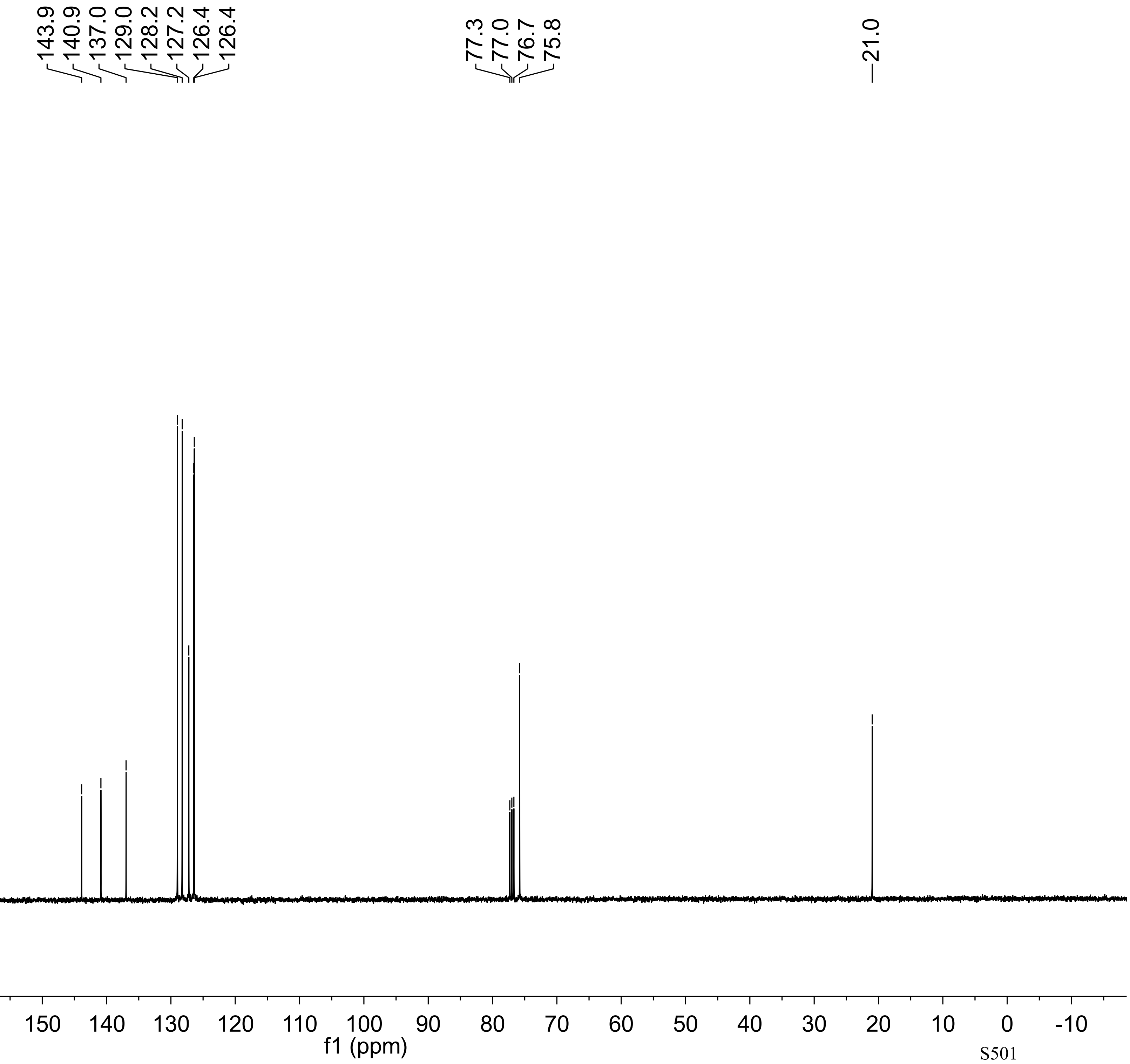

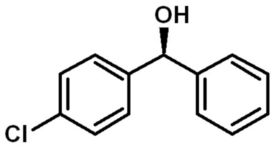

C51

Parameter	Value
1 Title	QJ-1-43D-8
2 Comment	
3 Origin	Bruker BioSpin GmbH
4 Owner	nmrsu
5 Site	
6 Spectrometer	AVANCE NEO 400 MHZ DIGITAL NMR SPECTROMETER
7 Author	
8 Solvent	CDCl3
9 Temperature	298.2
10 Pulse Sequence	zg30
11 Experiment	1D
12 Number of Scans	8
13 Receiver Gain	101
14 Spectrometer Frequency	400.13
15 Spectral Width	8196.7
16 Lowest Frequency	-1643.2
17 Nucleus	1H
18 Acquired Size	32768
19 Spectral Size	65536

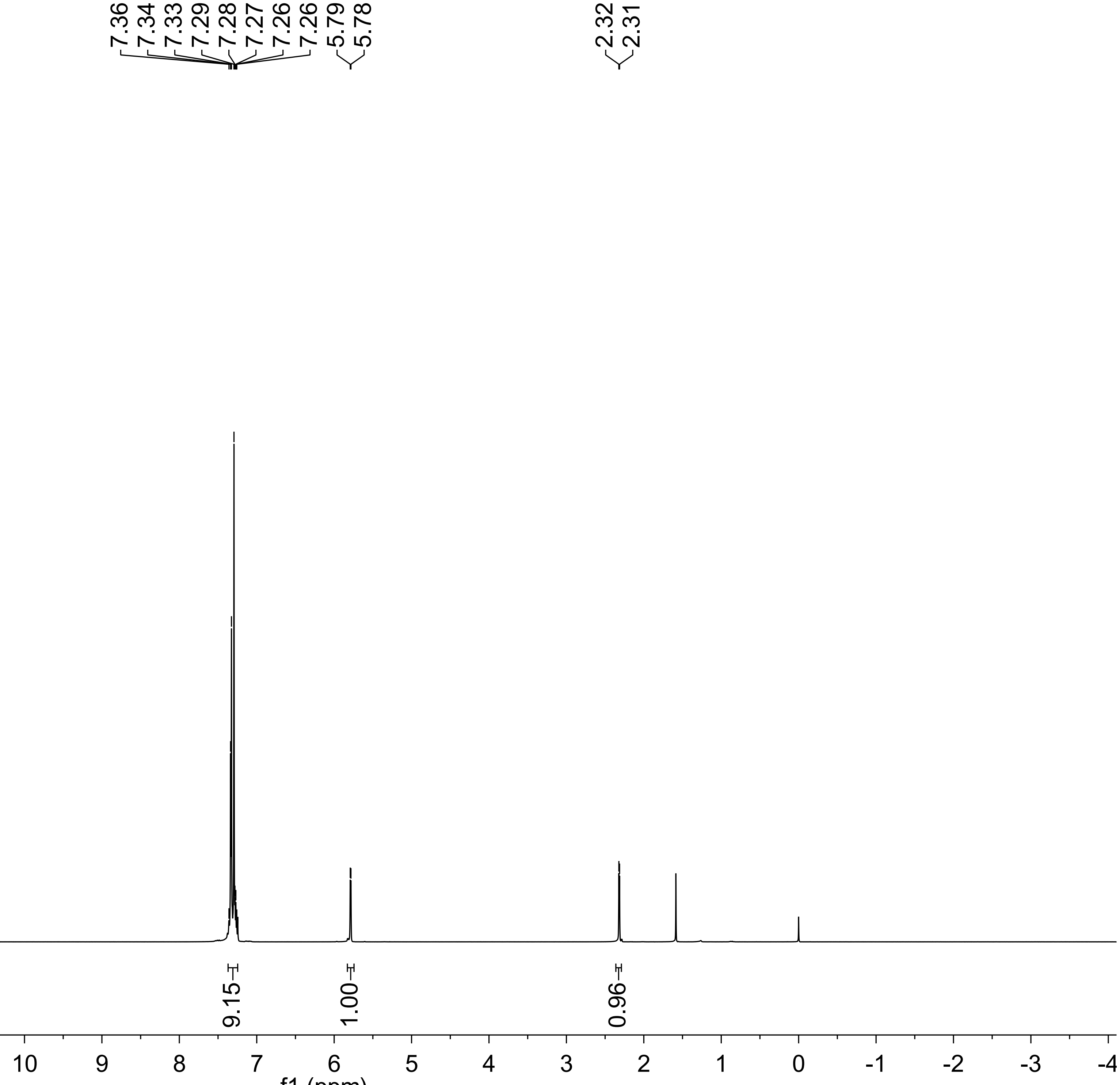

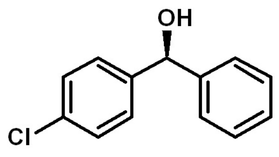

C51

	Parameter	Value
1	Title	QJ-1-43D-1
2	Comment	QJ-1-43B
3	Origin	Bruker BioSpin GmbH
4	Owner	nmrsu
5	Site	
6	Spectrometer	Avance Neo 400M
7	Author	
8	Solvent	CDCl3
9	Temperature	298.3
10	Pulse Sequence	zgpg30
11	Experiment	1D
12	Number of Scans	54
13	Receiver Gain	24
14	Spectrometer Frequency	100.63
15	Spectral Width	23809.5
16	Lowest Frequency	-1842.2
17	Nucleus	13C
18	Acquired Size	32768
19	Spectral Size	32768

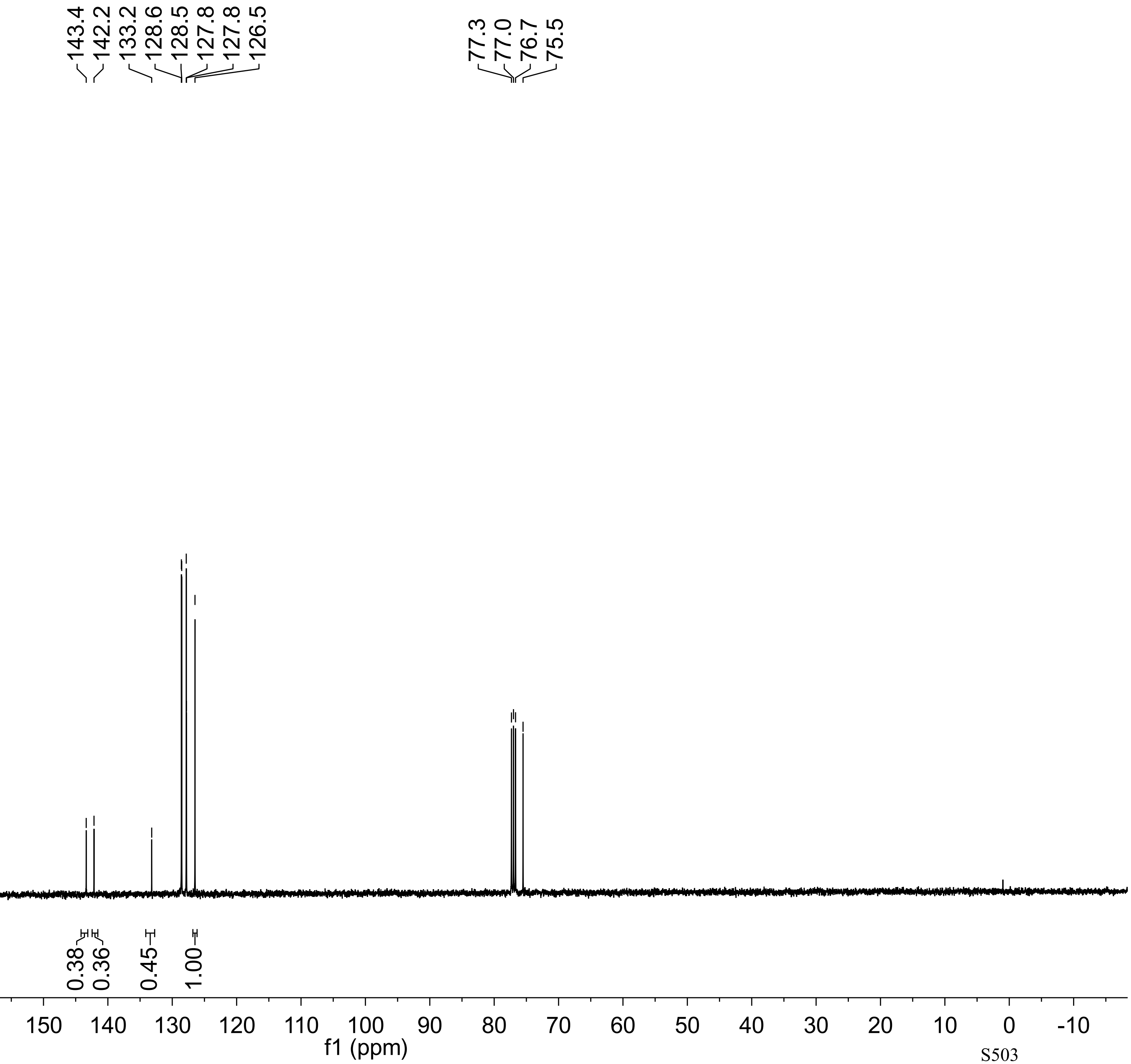

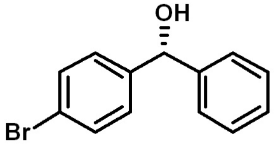

C52

	Parameter	Value
1	Title	QJ-1-43B-8
2	Comment	
3	Origin	Bruker BioSpin GmbH
4	Owner	nmrsu
5	Site	
6	Spectrometer	AVANCE NEO 400 MHZ DIGITAL NMR SPECTROMETER
7	Author	
8	Solvent	CDCl3
9	Temperature	298.1
10	Pulse Sequence	zg30
11	Experiment	1D
12	Number of Scans	8
13	Receiver Gain	101
14	Spectrometer Frequency	400.13
15	Spectral Width	8196.7
16	Lowest Frequency	-1648.8
17	Nucleus	1H
18	Acquired Size	32768
19	Spectral Size	65536

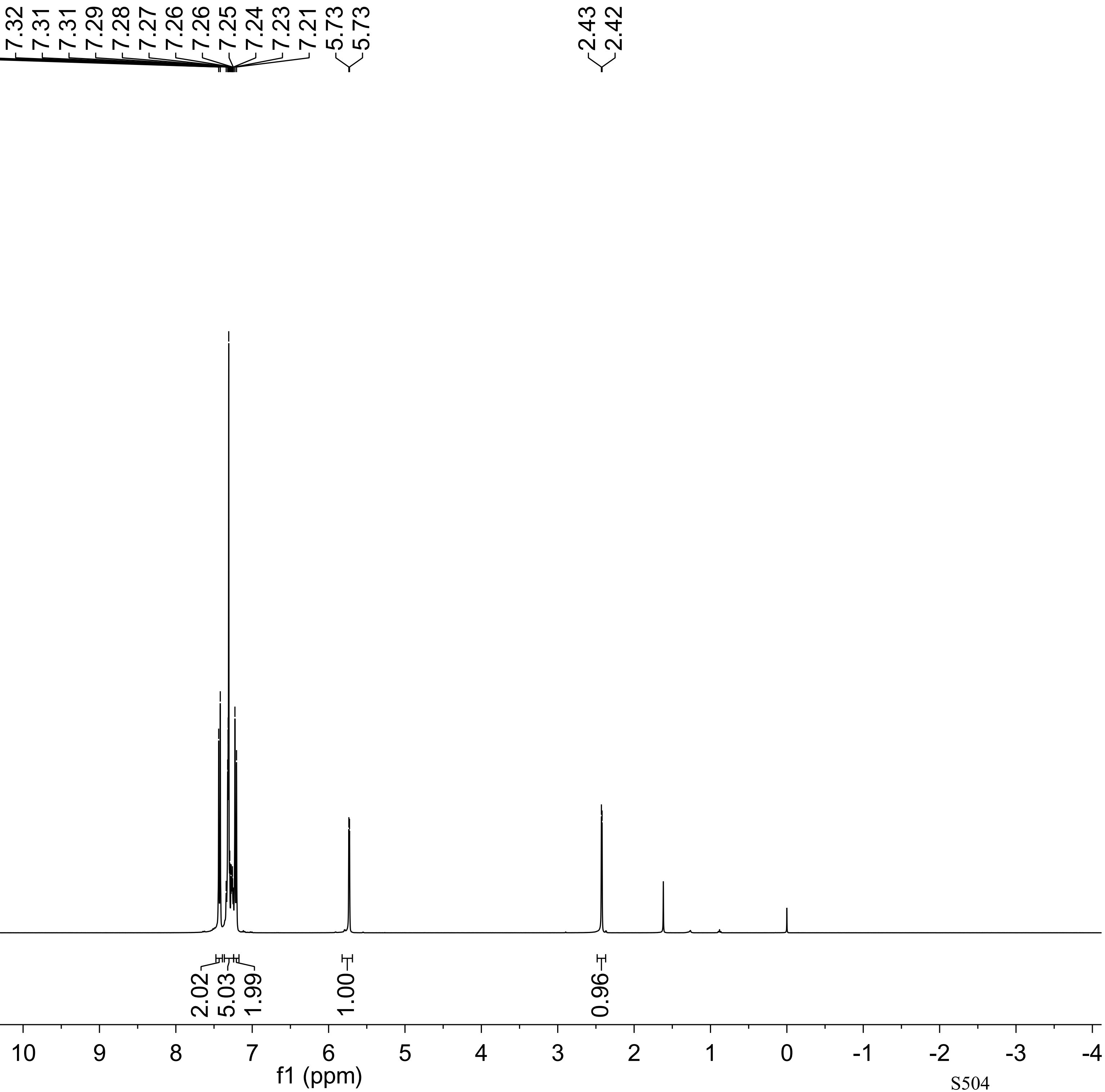

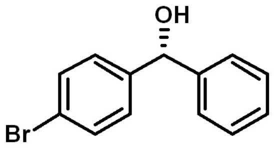

C52

Parameter	Value
1 Title	QJ-1-43B-8
2 Comment	
3 Origin	Bruker BioSpin GmbH
4 Owner	nmrsu
5 Site	
6 Spectrometer	AVANCE NEO 400 MHZ DIGITAL NMR SPECTROMETER
7 Author	
8 Solvent	CDCl3
9 Temperature	298.2
10 Pulse Sequence	zgpg30
11 Experiment	1D
12 Number of Scans	52
13 Receiver Gain	62
14 Spectrometer Frequency	100.61
15 Spectral Width	23809.5
16 Lowest Frequency	-1843.5
17 Nucleus	13C
18 Acquired Size	32768
19 Spectral Size	32768

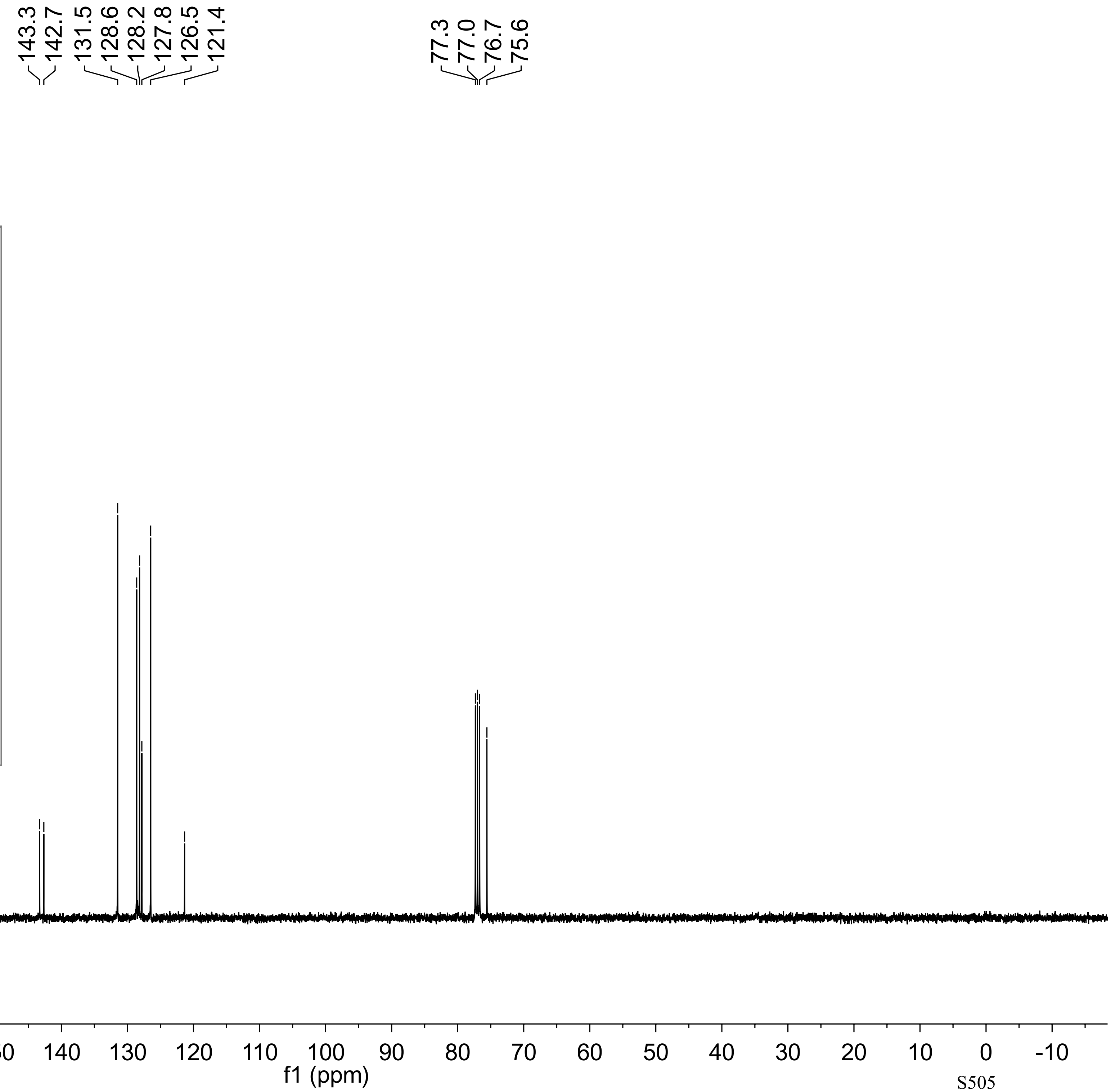

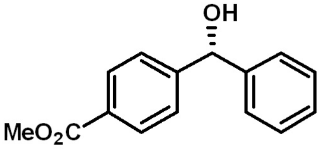

C53

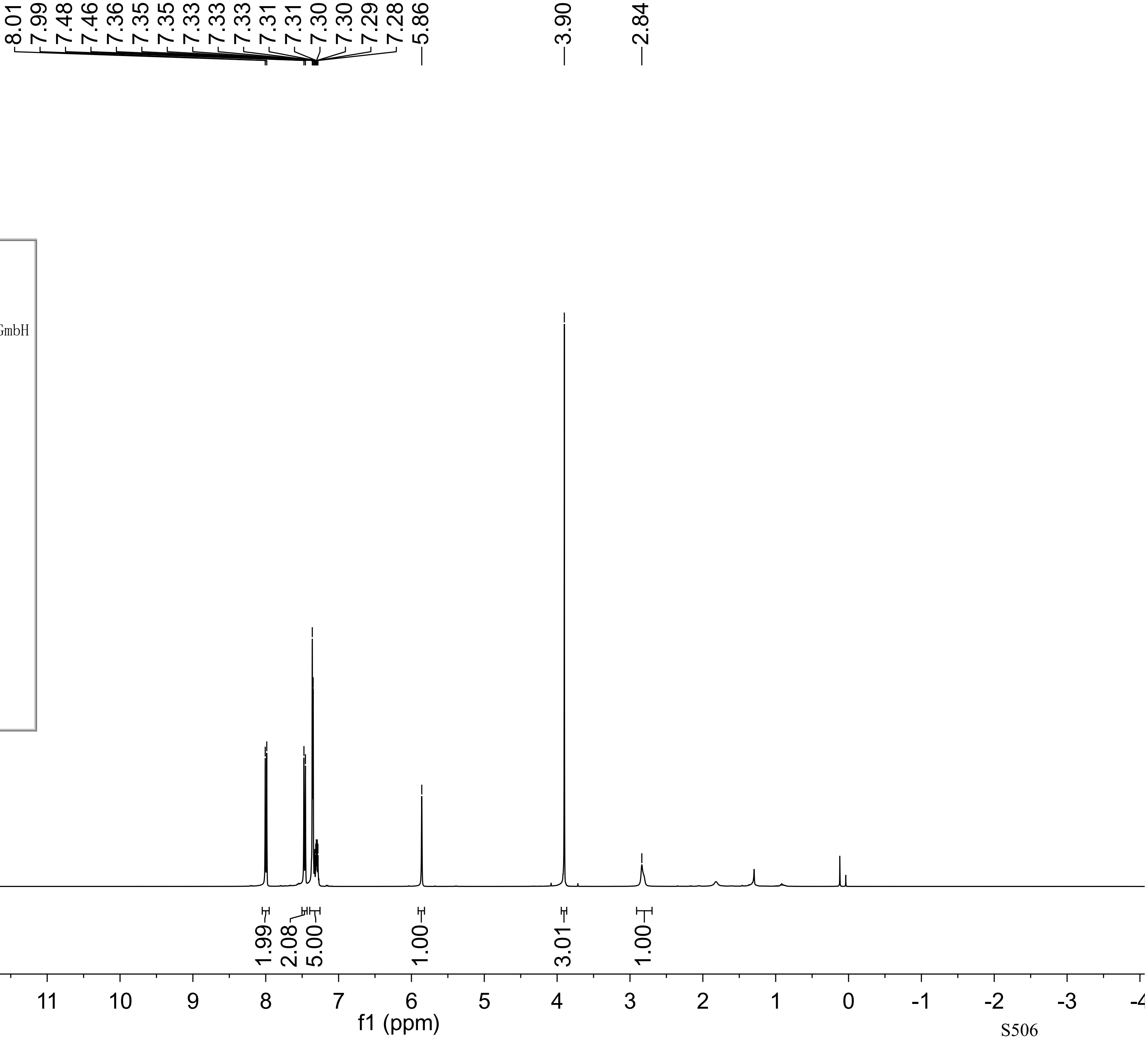

	Parameter	Value
1	Title	QJ-1-45C
2	Comment	QJ-1-45C
3	Origin	Bruker BioSpin GmbH
4	Owner	nmrsu
5	Site	
6	Spectrometer	Avance Neo 400M
7	Author	
8	Solvent	CDCl3
9	Temperature	298.1
10	Pulse Sequence	zg30
11	Experiment	1D
12	Number of Scans	7
13	Receiver Gain	88
14	Spectrometer Frequency	400.18
15	Spectral Width	8196.7
16	Lowest Frequency	-1627.3
17	Nucleus	1H
18	Acquired Size	32768
19	Spectral Size	65536

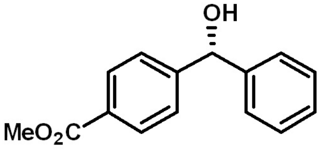

C53

Parameter		Value
1	Title	QJ-1-45C-13C
2	Comment	QJ-1-45C-13C
3	Origin	Bruker BioSpin GmbH
4	Owner	nmrsu
5	Site	
6	Spectrometer	Avance Neo 400M
7	Author	
8	Solvent	CDCl3
9	Temperature	298.2
10	Pulse Sequence	zgpg30
11	Experiment	1D
12	Number of Scans	50
13	Receiver Gain	24
14	Spectrometer Frequency	100.63
15	Spectral Width	23809.5
16	Lowest Frequency	-1852.1
17	Nucleus	13C
18	Acquired Size	32768
19	Spectral Size	32768

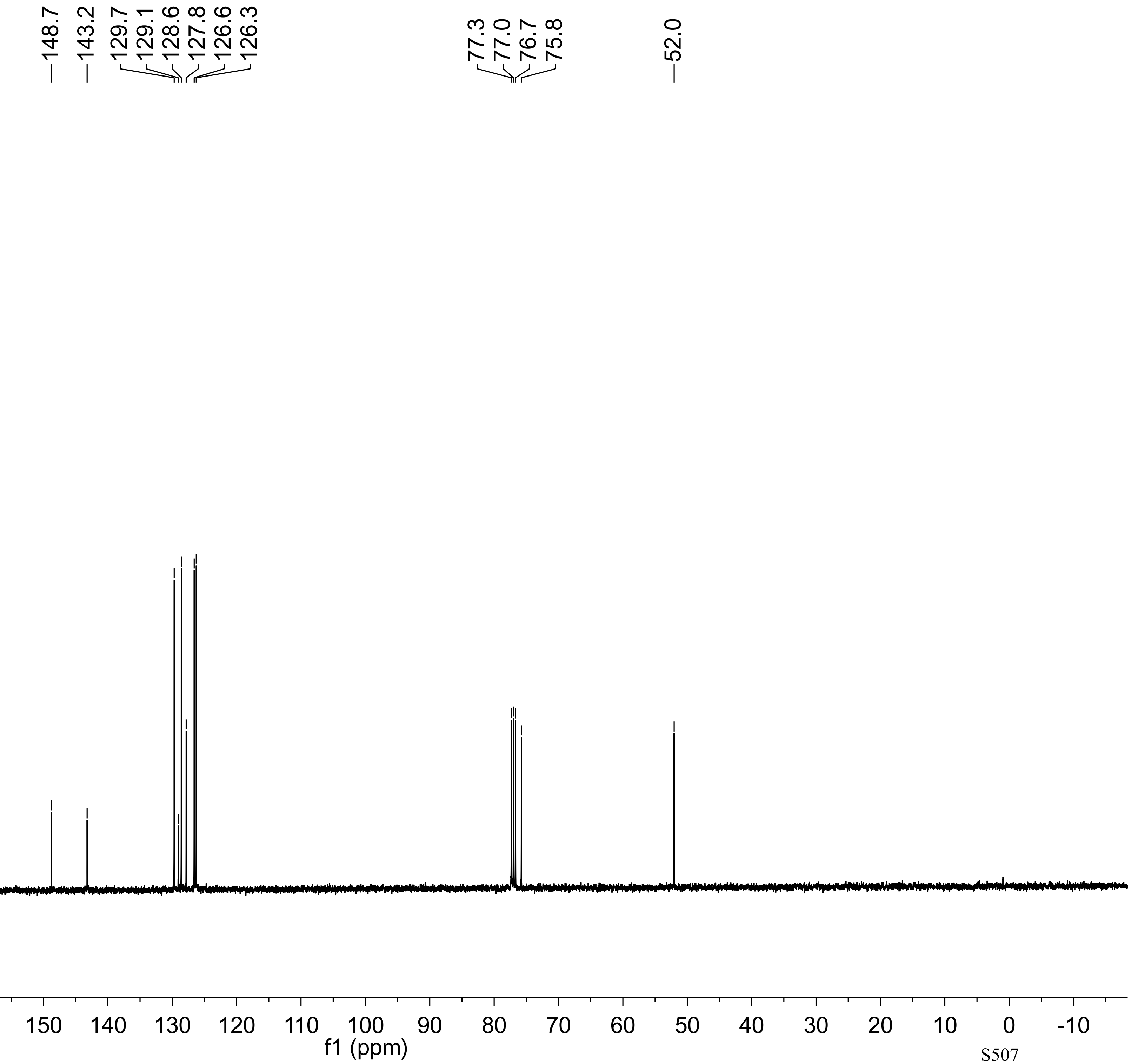

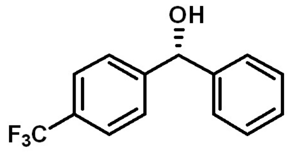

C54

Parameter	Value
1 Title	QJ-1-41A
2 Comment	QJ-1-41A-3
3 Origin	Bruker BioSpin GmbH
4 Owner	nmrsu
5 Site	
6 Spectrometer	Avance Neo 400M
7 Author	
8 Solvent	CDCl3
9 Temperature	298.2
10 Pulse Sequence	zg30
11 Experiment	1D
12 Number of Scans	7
13 Receiver Gain	101
14 Spectrometer Frequency	400.18
15 Spectral Width	8196.7
16 Lowest Frequency	-1627.3
17 Nucleus	1H
18 Acquired Size	32768
19 Spectral Size	65536

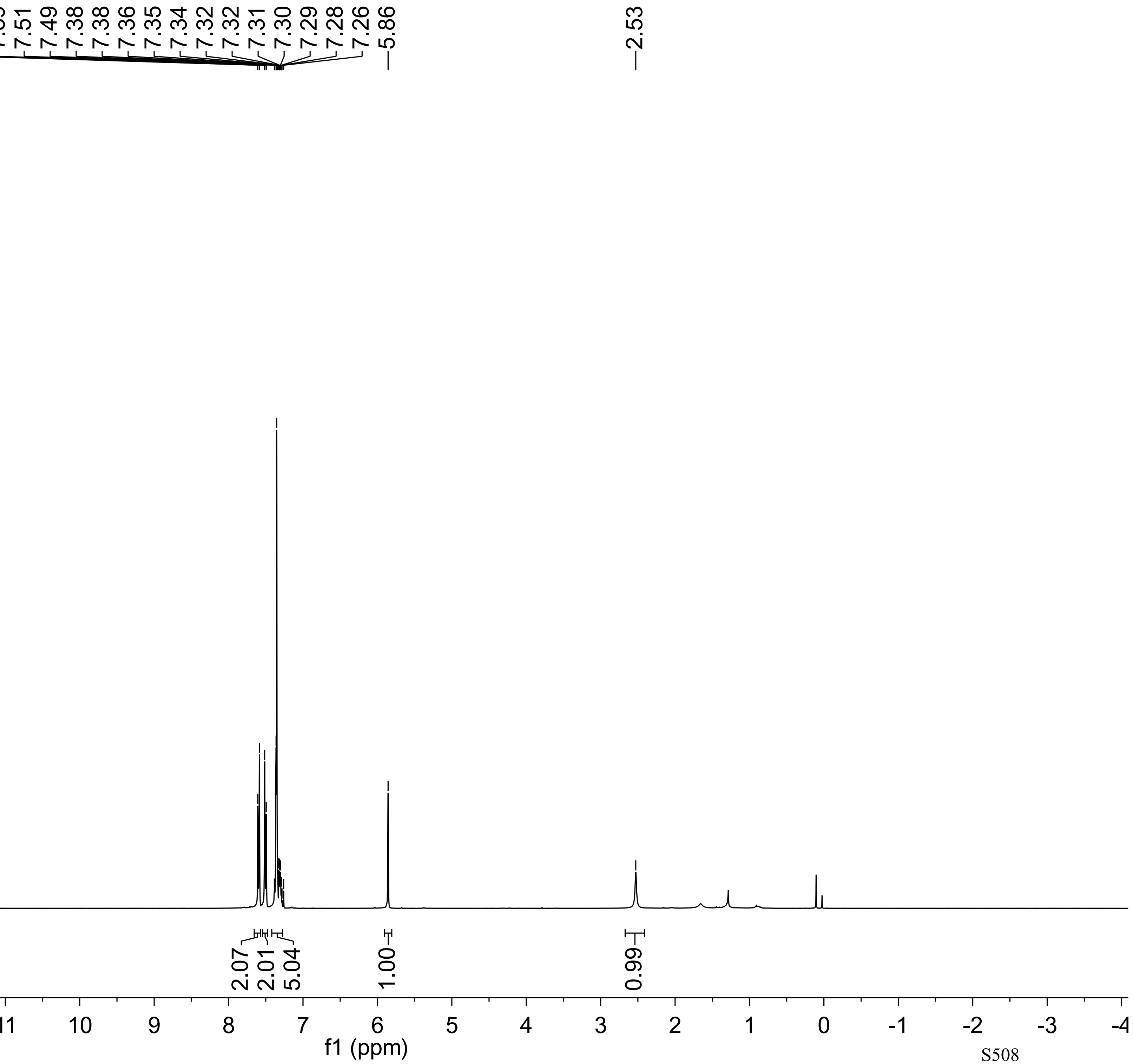

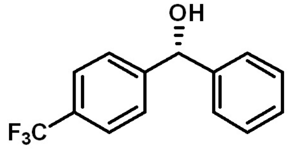

C54

Parameter	Value
1 Title	QJ-1-41A-C-3
2 Comment	
3 Origin	Bruker BioSpin GmbH
4 Owner	nmrsu
5 Site	
6 Spectrometer	Avance Neo 400M
7 Author	
8 Solvent	CDCl3
9 Temperature	298.2
10 Pulse Sequence	zgpg30
11 Experiment	1D
12 Number of Scans	95
13 Receiver Gain	33
14 Spectrometer Frequency	100.63
15 Spectral Width	23809.5
16 Lowest Frequency	-1842.2
17 Nucleus	13C
18 Acquired Size	32768
19 Spectral Size	32768

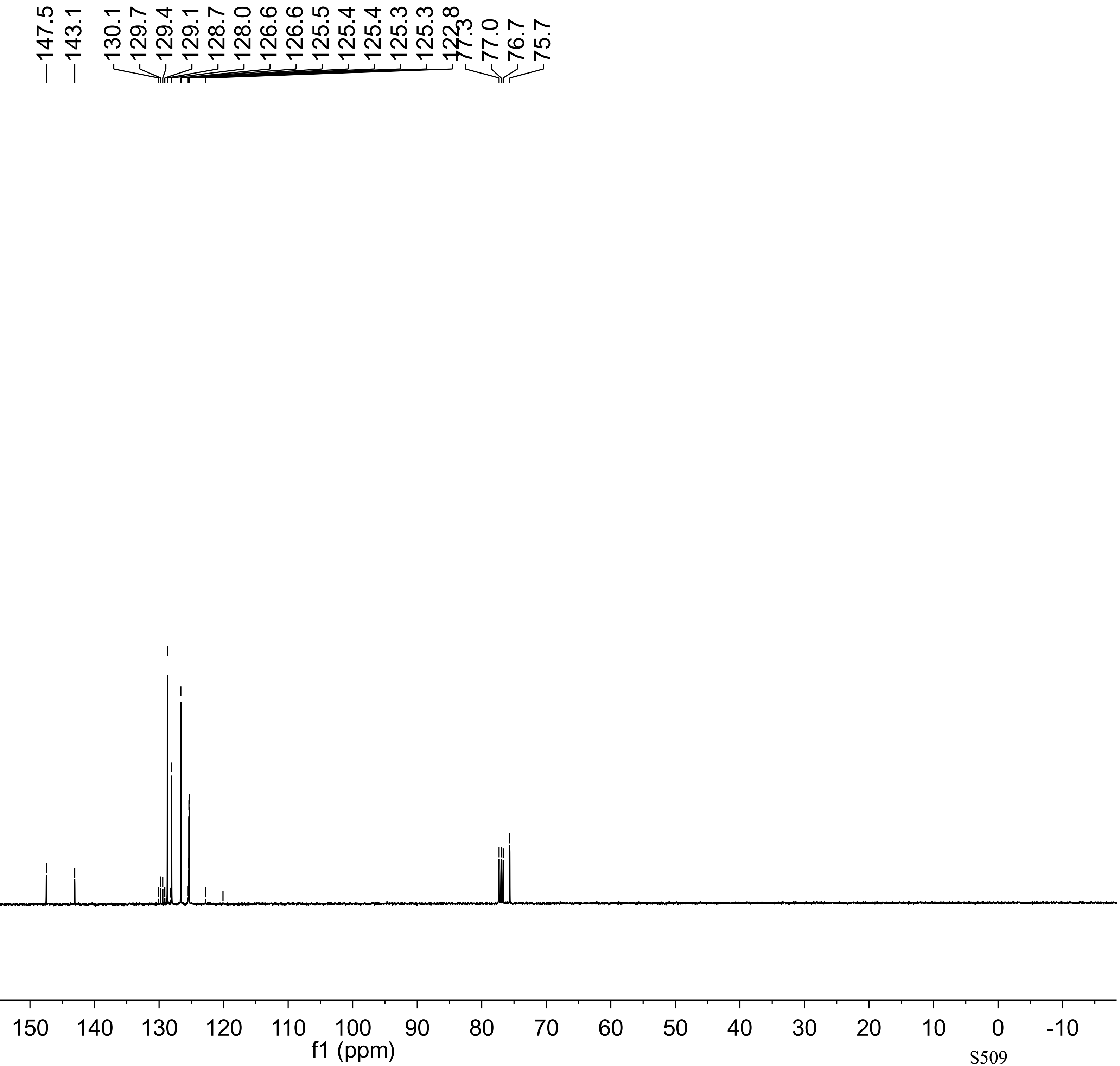

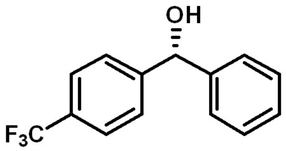

C54

	Parameter	Value
1	Title	QJ-1-41A-F
2	Comment	
3	Origin	Bruker BioSpin GmbH
4	Owner	nmrsu
5	Site	
6	Spectrometer	AVANCE NEO 400 MHZ DIGITAL NMR SPECTROMETER
7	Author	
8	Solvent	CDCl3
9	Temperature	298.1
10	Pulse Sequence	zg
11	Experiment	1D
12	Number of Scans	16
13	Receiver Gain	101
14	Spectrometer Frequency	376.50
15	Spectral Width	90909.1
16	Lowest Frequency	-83104.4
17	Nucleus	19F
18	Acquired Size	65536
19	Spectral Size	65536

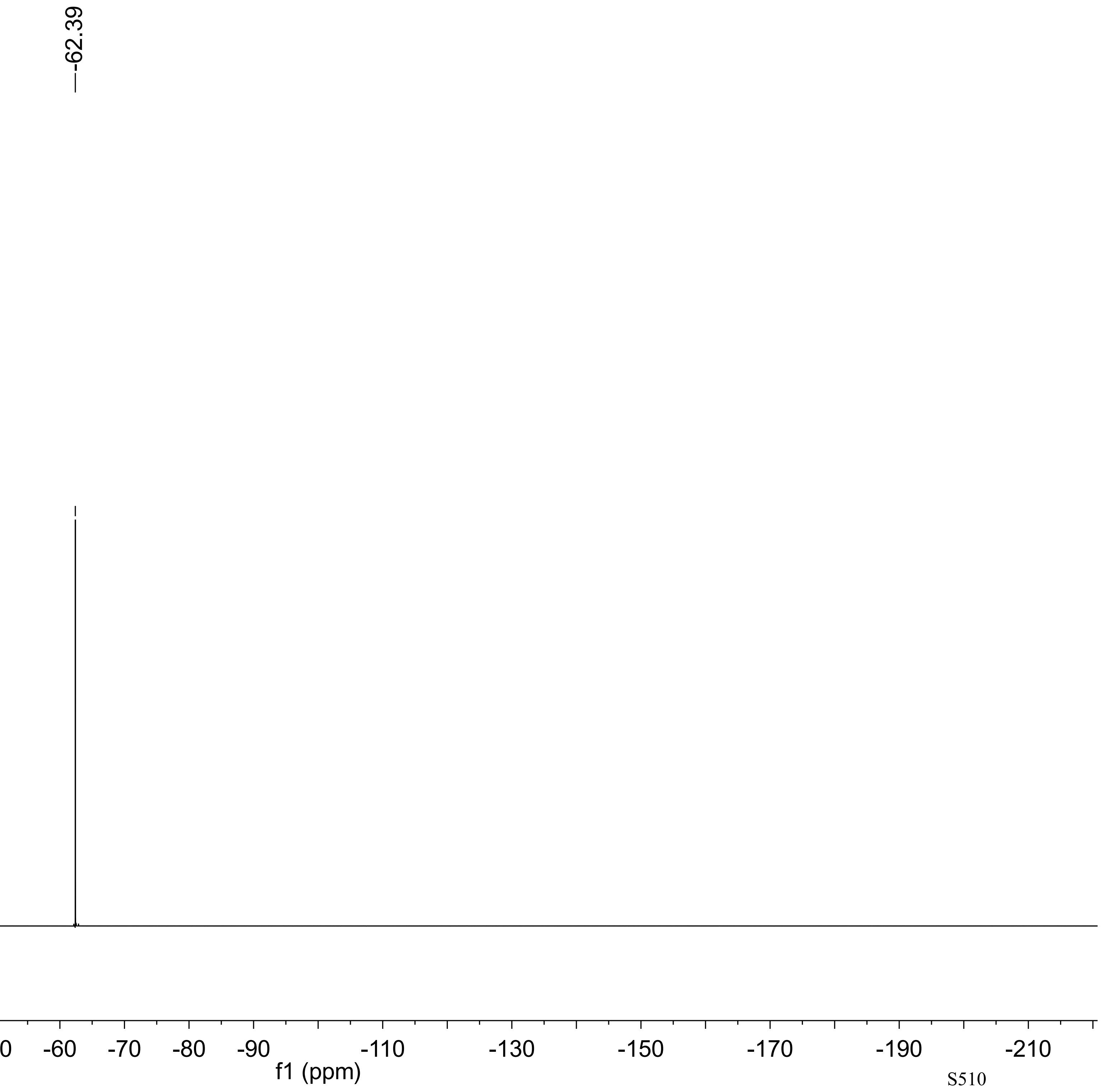

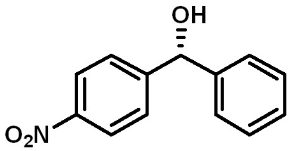

C55

Parameter	Value
1 Title	QJ-1-41B
2 Comment	QJ-1-41B
3 Origin	Bruker BioSpin GmbH
4 Owner	nmrsu
5 Site	
6 Spectrometer	Avance Neo 400M
7 Author	
8 Solvent	CDCl3
9 Temperature	298.1
10 Pulse Sequence	zg30
11 Experiment	1D
12 Number of Scans	9
13 Receiver Gain	93
14 Spectrometer Frequency	400.18
15 Spectral Width	8196.7
16 Lowest Frequency	-1636.9
17 Nucleus	1H
18 Acquired Size	32768
19 Spectral Size	65536

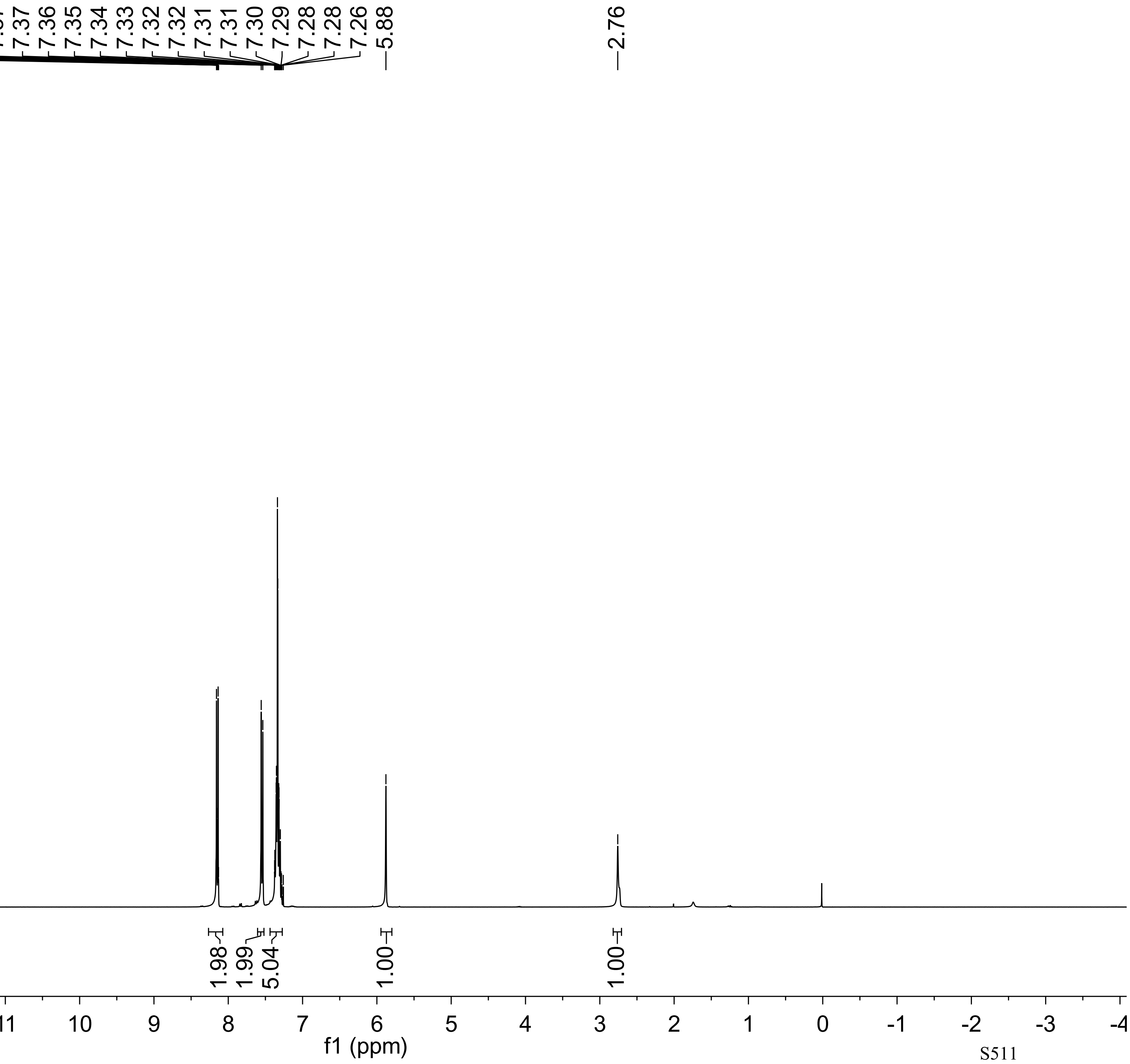

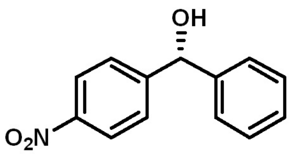

C55

Parameter	Value
1 Title	QJ-1-41B
2 Comment	QJ-1-41B
3 Origin	Bruker BioSpin GmbH
4 Owner	nmrsu
5 Site	
6 Spectrometer	Avance Neo 400M
7 Author	
8 Solvent	CDCl3
9 Temperature	298.3
10 Pulse Sequence	zgpg30
11 Experiment	1D
12 Number of Scans	53
13 Receiver Gain	38
14 Spectrometer Frequency	100.63
15 Spectral Width	23809.5
16 Lowest Frequency	-1853.3
17 Nucleus	13C
18 Acquired Size	32768
19 Spectral Size	32768

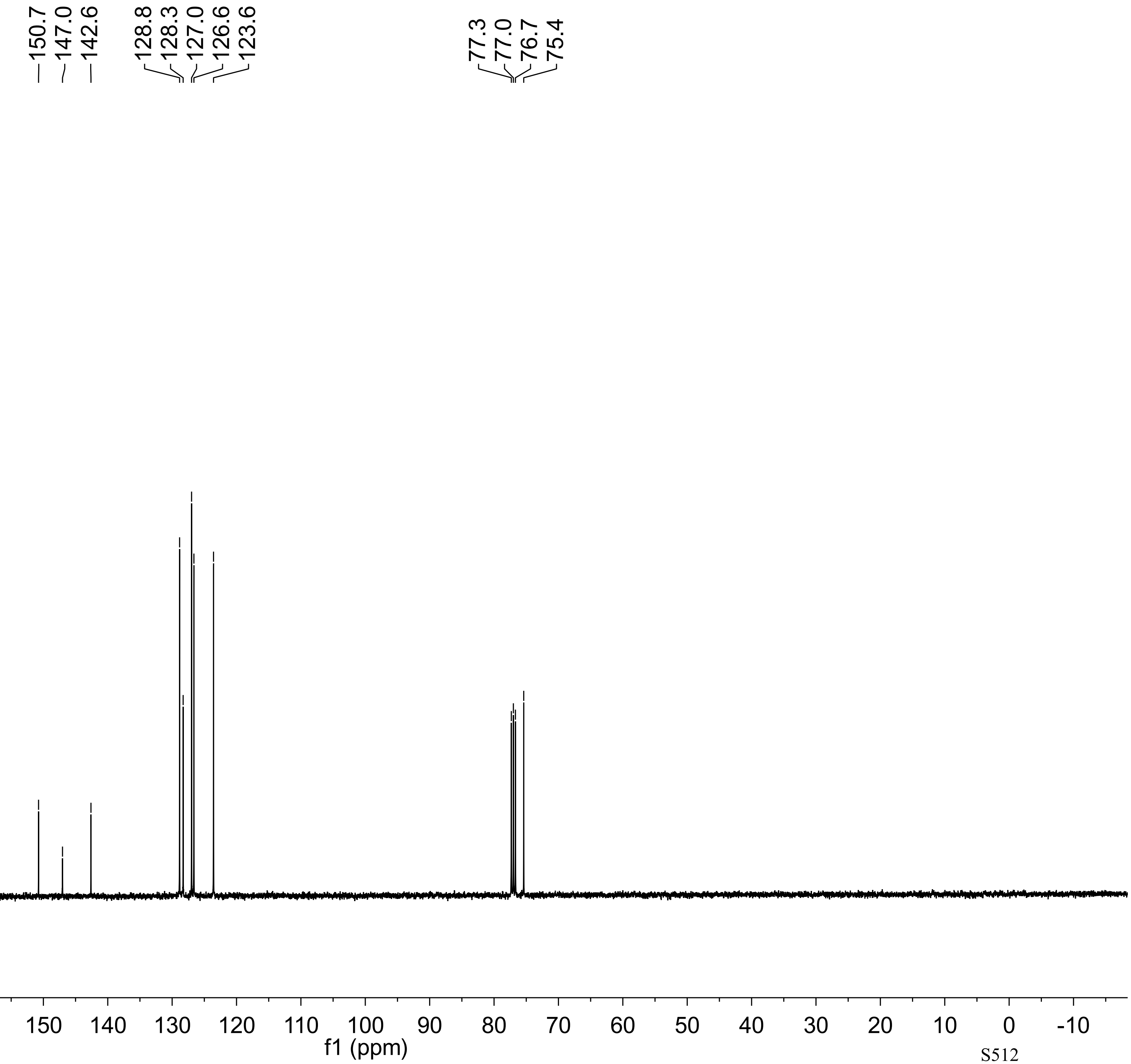

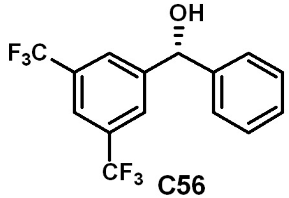

C56

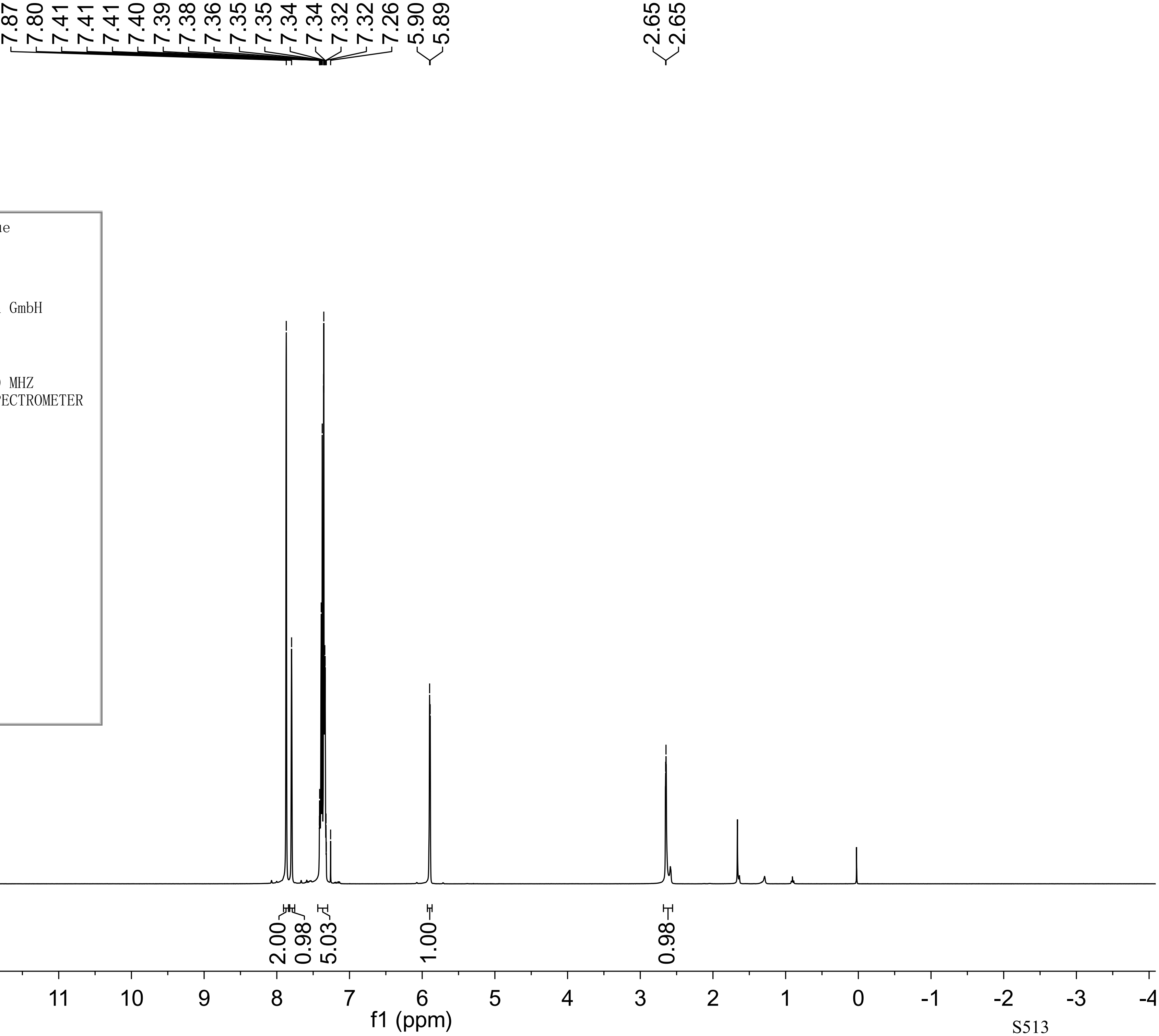

Parameter	Value
1 Title	QJ-1-45B-7
2 Comment	
3 Origin	Bruker BioSpin GmbH
4 Owner	nmrsu
5 Site	
6 Spectrometer	AVANCE NEO 400 MHZ DIGITAL NMR SPECTROMETER
7 Author	
8 Solvent	CDCl3
9 Temperature	298.1
10 Pulse Sequence	zg30
11 Experiment	1D
12 Number of Scans	8
13 Receiver Gain	101
14 Spectrometer Frequency	400.13
15 Spectral Width	8196.7
16 Lowest Frequency	-1647.3
17 Nucleus	1H
18 Acquired Size	32768
19 Spectral Size	65536

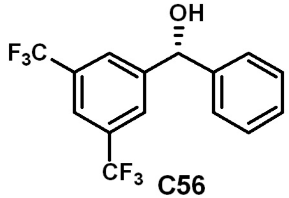

C56

Parameter	Value
1 Title	QJ-1-45B-C-3
2 Comment	
3 Origin	Bruker BioSpin GmbH
4 Owner	nmrsu
5 Site	
6 Spectrometer	Avance Neo 400M
7 Author	
8 Solvent	CDCl3
9 Temperature	298.2
10 Pulse Sequence	zgpg30
11 Experiment	1D
12 Number of Scans	32
13 Receiver Gain	33
14 Spectrometer Frequency	100.63
15 Spectral Width	23809.5
16 Lowest Frequency	-1845.4
17 Nucleus	13C
18 Acquired Size	32768
19 Spectral Size	32768

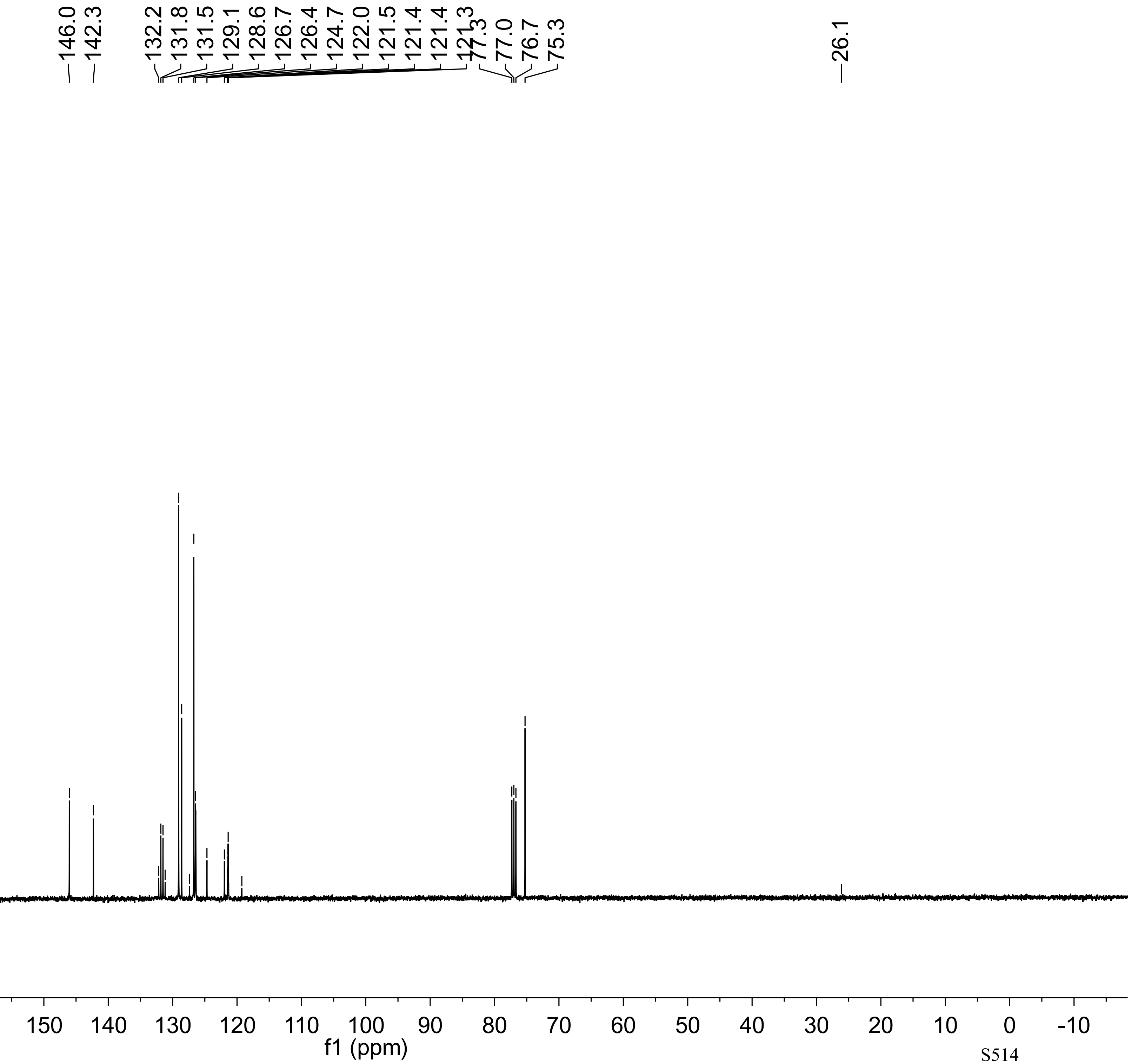

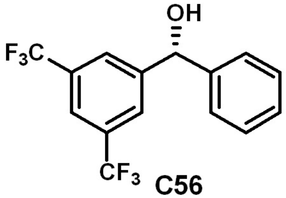

C56

	Parameter	Value
1	Title	QJ-1-45C-F
2	Comment	
3	Origin	Bruker BioSpin GmbH
4	Owner	nmrsu
5	Site	
6	Spectrometer	AVANCE NEO 400 MHZ DIGITAL NMR SPECTROMETER
7	Author	
8	Solvent	CDCl3
9	Temperature	298.1
10	Pulse Sequence	zg
11	Experiment	1D
12	Number of Scans	16
13	Receiver Gain	101
14	Spectrometer Frequency	376.50
15	Spectral Width	90909.1
16	Lowest Frequency	-83104.4
17	Nucleus	19F
18	Acquired Size	65536
19	Spectral Size	65536

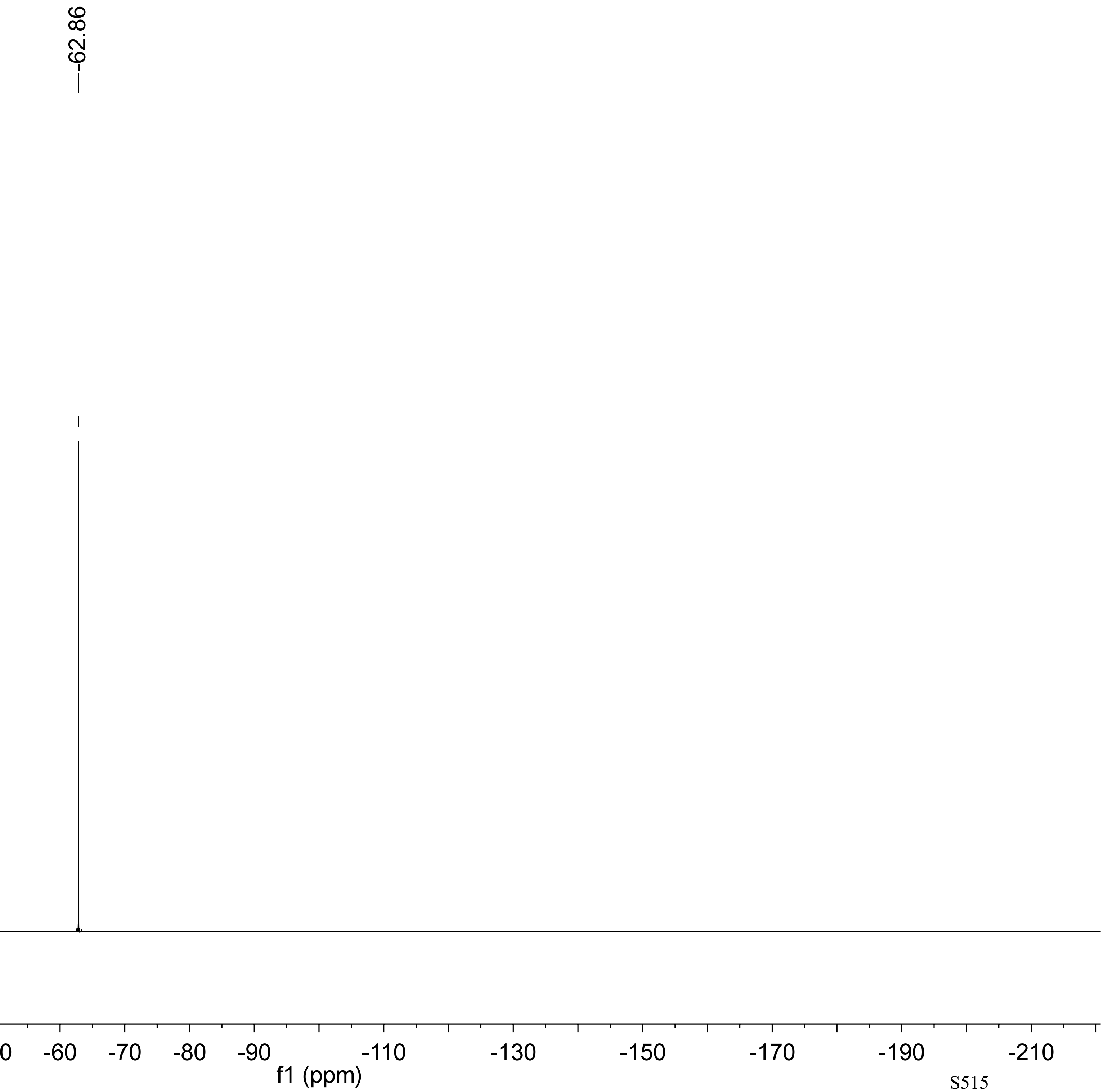

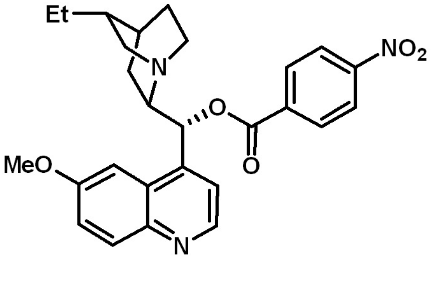

E

Parameter	Value
1 Title	cfm-6-105-10-ligand-NO2
2 Comment	
3 Origin	Bruker BioSpin GmbH
4 Owner	nmrsu
5 Site	
6 Spectrometer	Avance NEO 600
7 Author	
8 Solvent	CDCl3
9 Temperature	298.2
10 Pulse Sequence	zg30
11 Experiment	1D
12 Number of Scans	8
13 Receiver Gain	101
14 Spectrometer Frequency	600.15
15 Spectral Width	11904.8
16 Lowest Frequency	-2260.3
17 Nucleus	1H
18 Acquired Size	32768
19 Spectral Size	65536

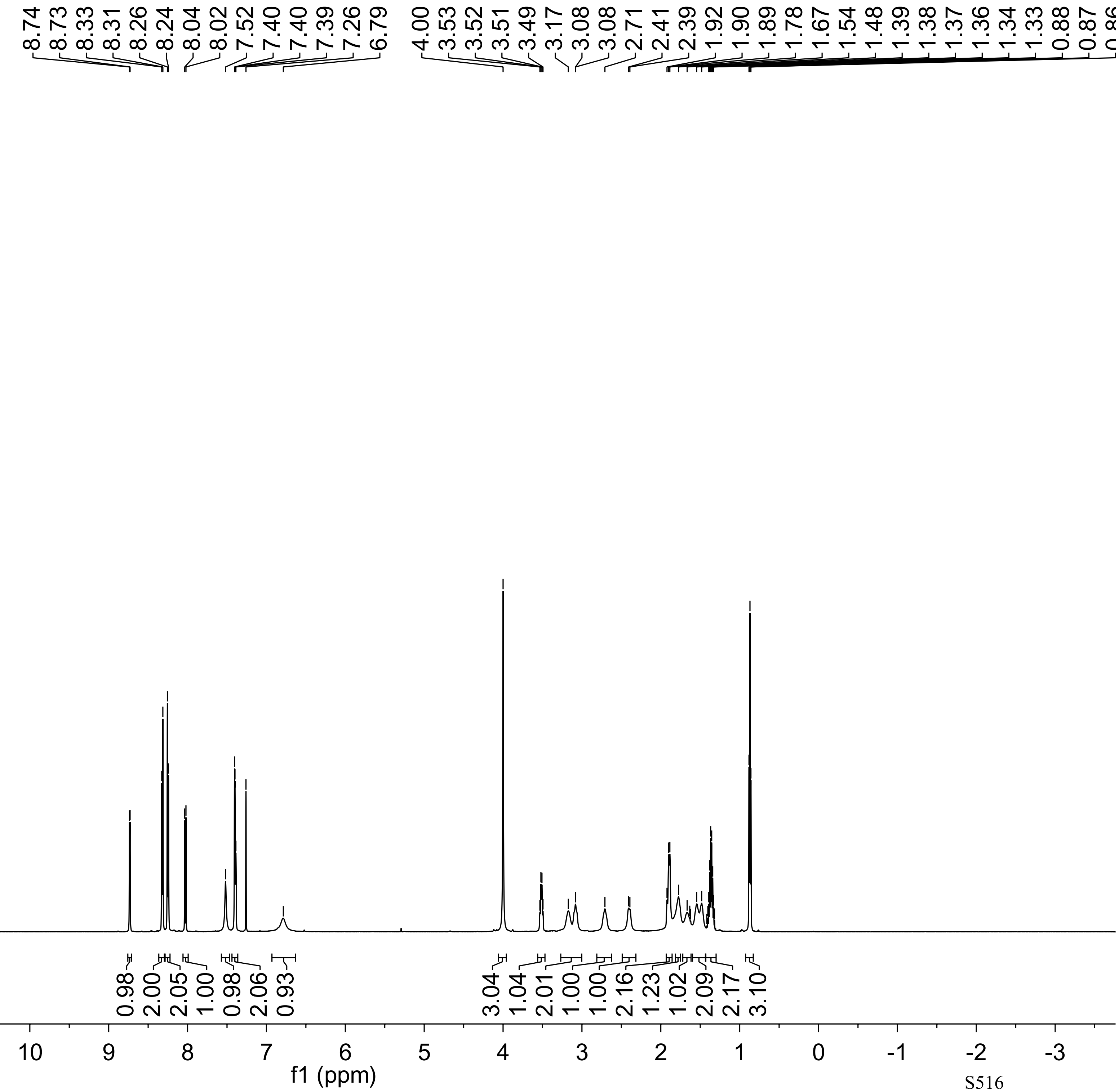

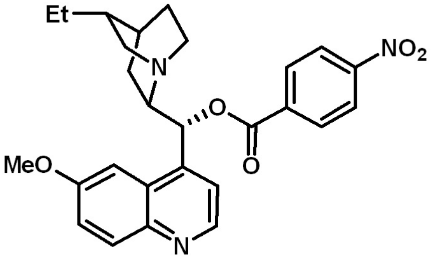

E

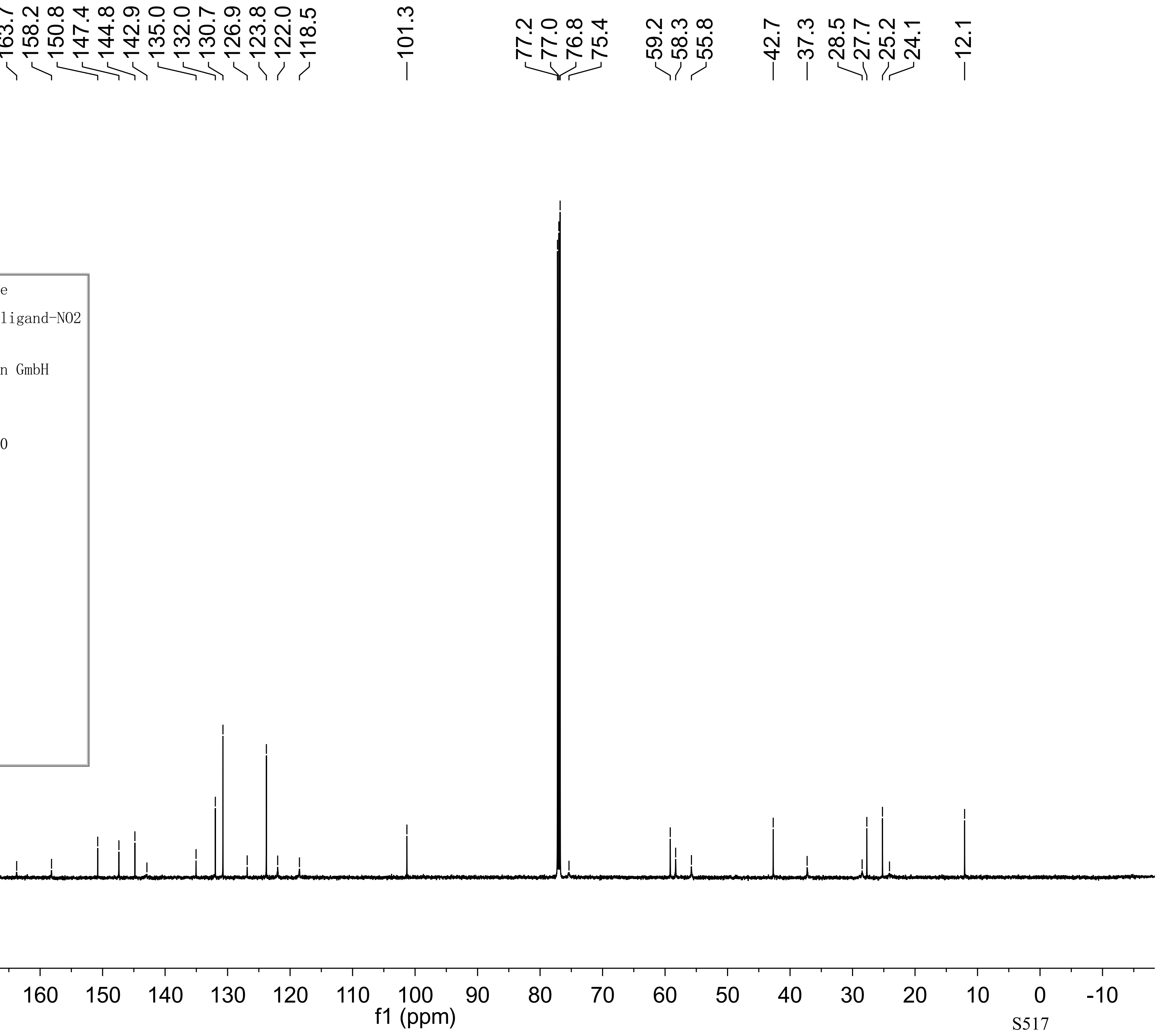

	Parameter	Value
1	Title	cfm-6-105-10-ligand-NO2
2	Comment	
3	Origin	Bruker BioSpin GmbH
4	Owner	nmrsu
5	Site	
6	Spectrometer	Avance NEO 600
7	Author	
8	Solvent	CDCl3
9	Temperature	298.2
10	Pulse Sequence	zgpg30
11	Experiment	1D
12	Number of Scans	79
13	Receiver Gain	101
14	Spectrometer Frequency	150.91
15	Spectral Width	35714.3
16	Lowest Frequency	-2771.6
17	Nucleus	13C
18	Acquired Size	32768
19	Spectral Size	32768

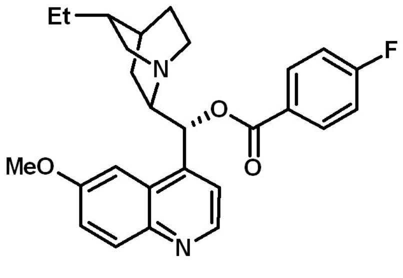

G

	Parameter	Value
1	Title	cfm-6-105-8-ligand-F
2	Comment	
3	Origin	Bruker BioSpin GmbH
4	Owner	nmrsu
5	Site	
6	Spectrometer	Avance NEO 600
7	Author	
8	Solvent	CDCl3
9	Temperature	298.1
10	Pulse Sequence	zg30
11	Experiment	1D
12	Number of Scans	8
13	Receiver Gain	101
14	Spectrometer Frequency	600.15
15	Spectral Width	11904.8
16	Lowest Frequency	-2260.3
17	Nucleus	1H
18	Acquired Size	32768
19	Spectral Size	65536

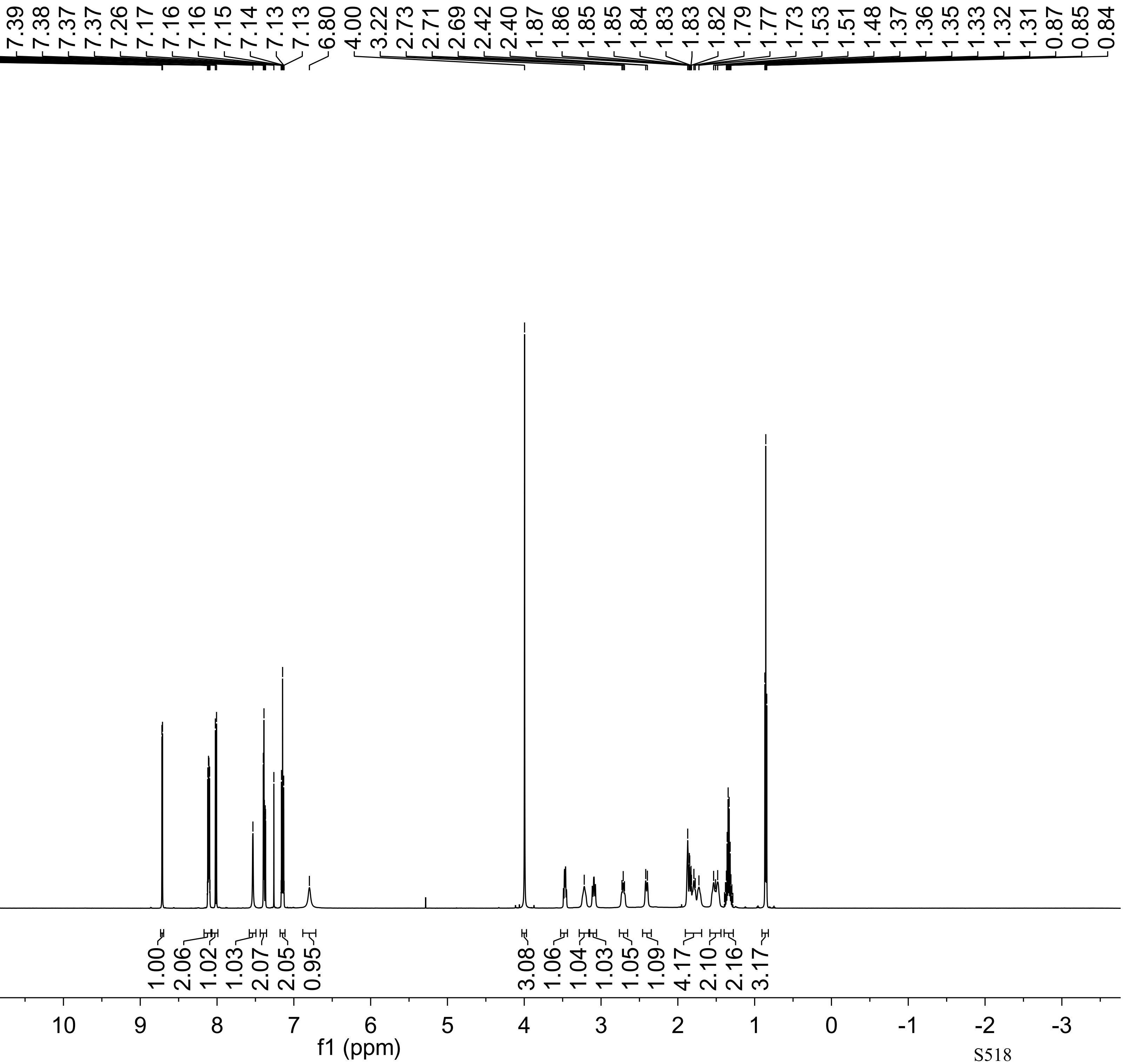

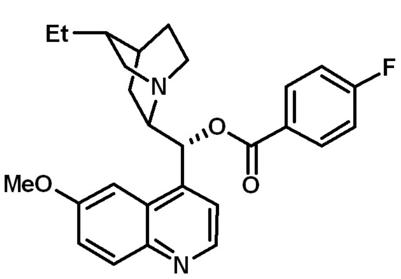

G

Parameter	Value
1 Title	cfm-6-105-8-ligand-F
2 Comment	
3 Origin	Bruker BioSpin GmbH
4 Owner	nmrsu
5 Site	
6 Spectrometer	Avance NEO 600
7 Author	
8 Solvent	CDCl3
9 Temperature	298.1
10 Pulse Sequence	zgpg30
11 Experiment	1D
12 Number of Scans	117
13 Receiver Gain	101
14 Spectrometer Frequency	150.91
15 Spectral Width	35714.3
16 Lowest Frequency	-2773.6
17 Nucleus	13C
18 Acquired Size	32768
19 Spectral Size	32768

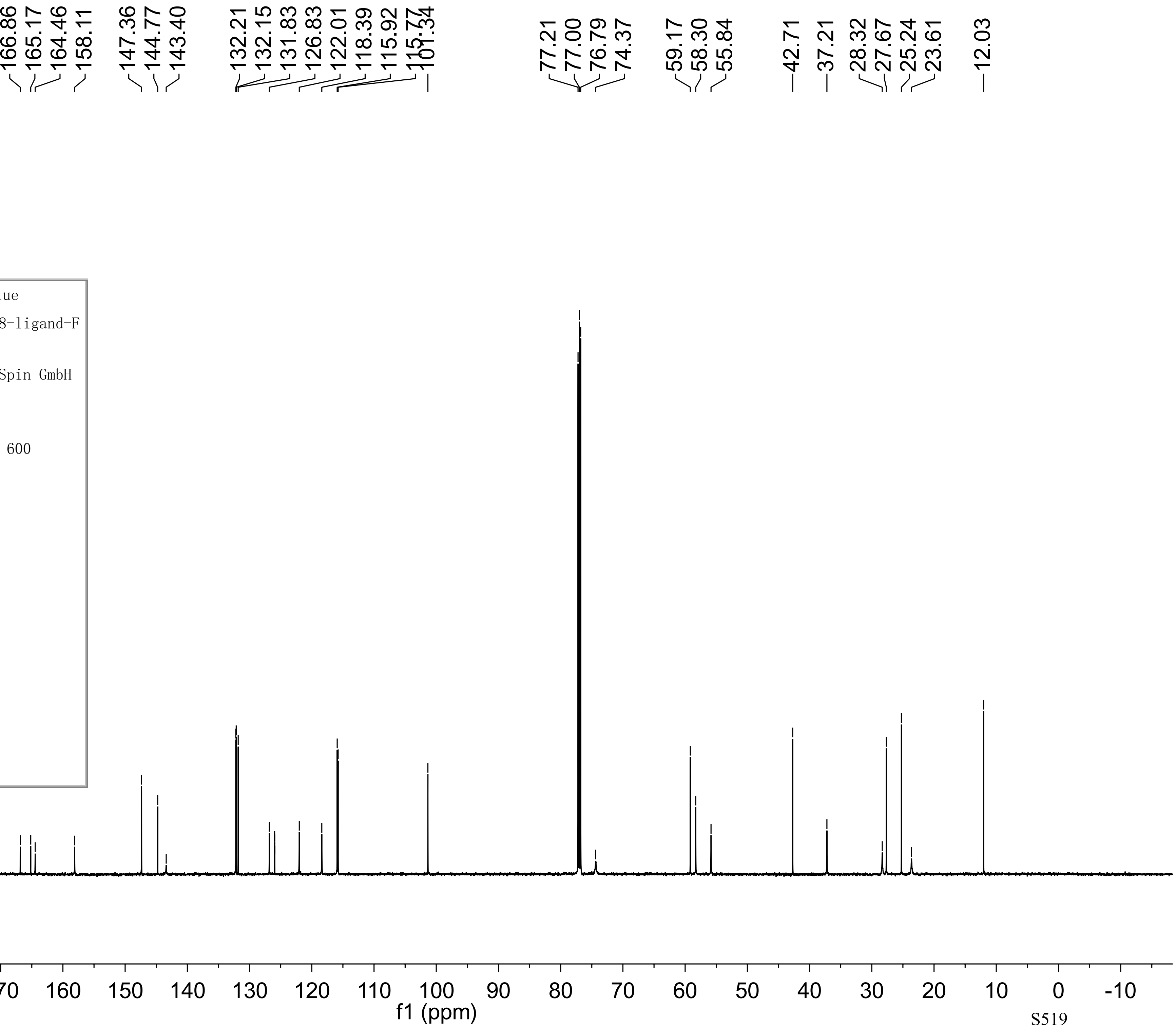

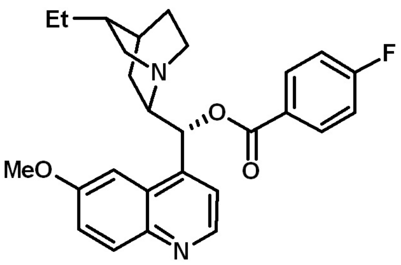

G

Parameter	Value
1 Title	CFM-6-105-8-ligand-F-1
2 Comment	
3 Origin	Bruker BioSpin GmbH
4 Owner	nmrsu
5 Site	
6 Spectrometer	AVANCE NEO 400 MHZ DIGITAL NMR SPECTROMETER
7 Author	
8 Solvent	CDCl3
9 Temperature	298.1
10 Pulse Sequence	zg
11 Experiment	1D
12 Number of Scans	16
13 Receiver Gain	101
14 Spectrometer Frequency	376.50
15 Spectral Width	90909.1
16 Lowest Frequency	-83104.4
17 Nucleus	19F
18 Acquired Size	65536
19 Spectral Size	65536

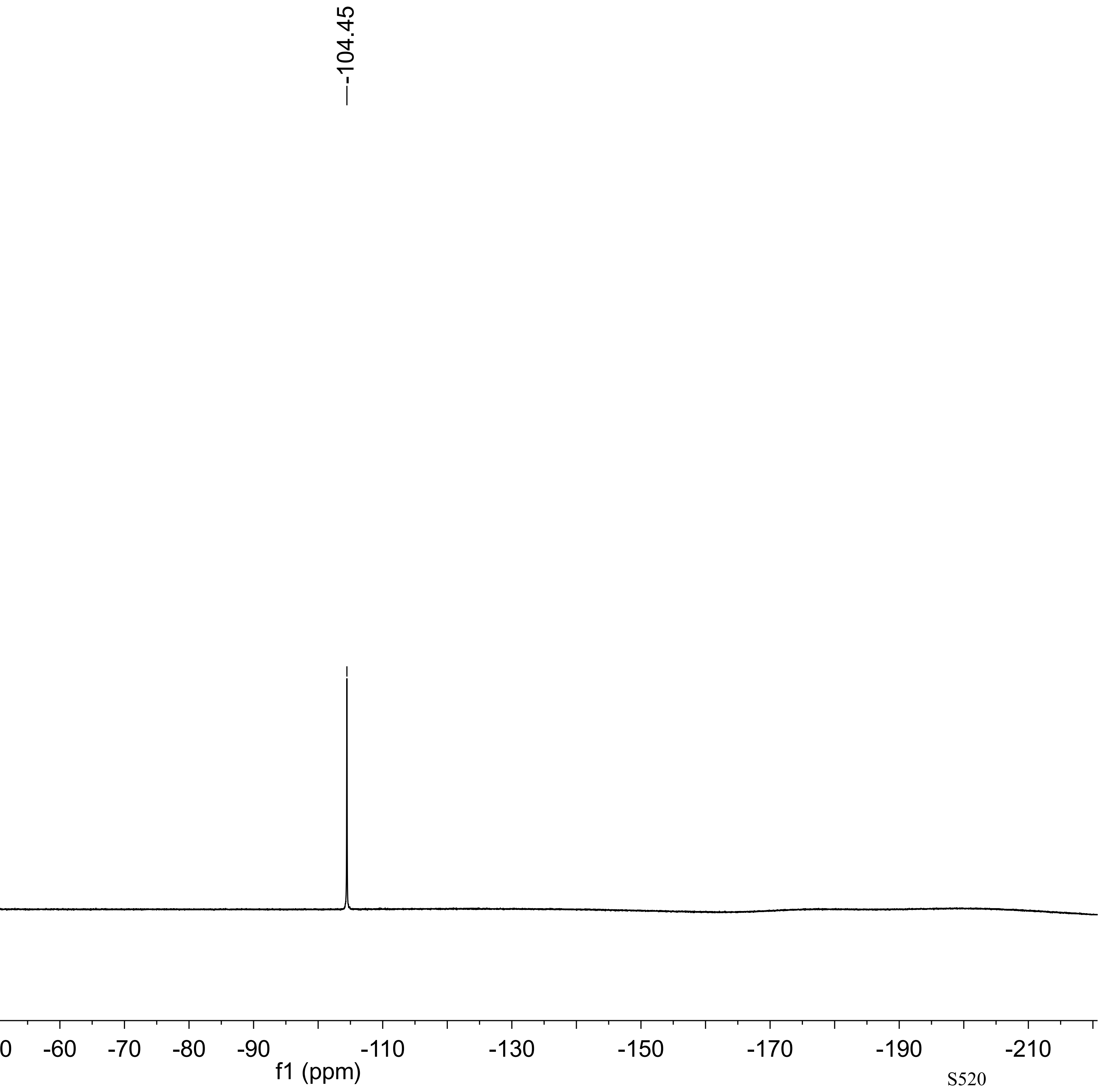

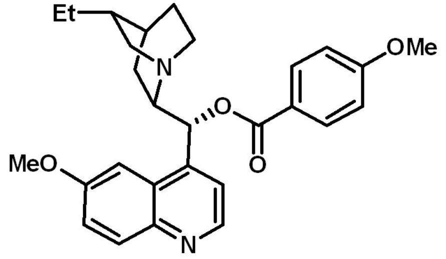

K

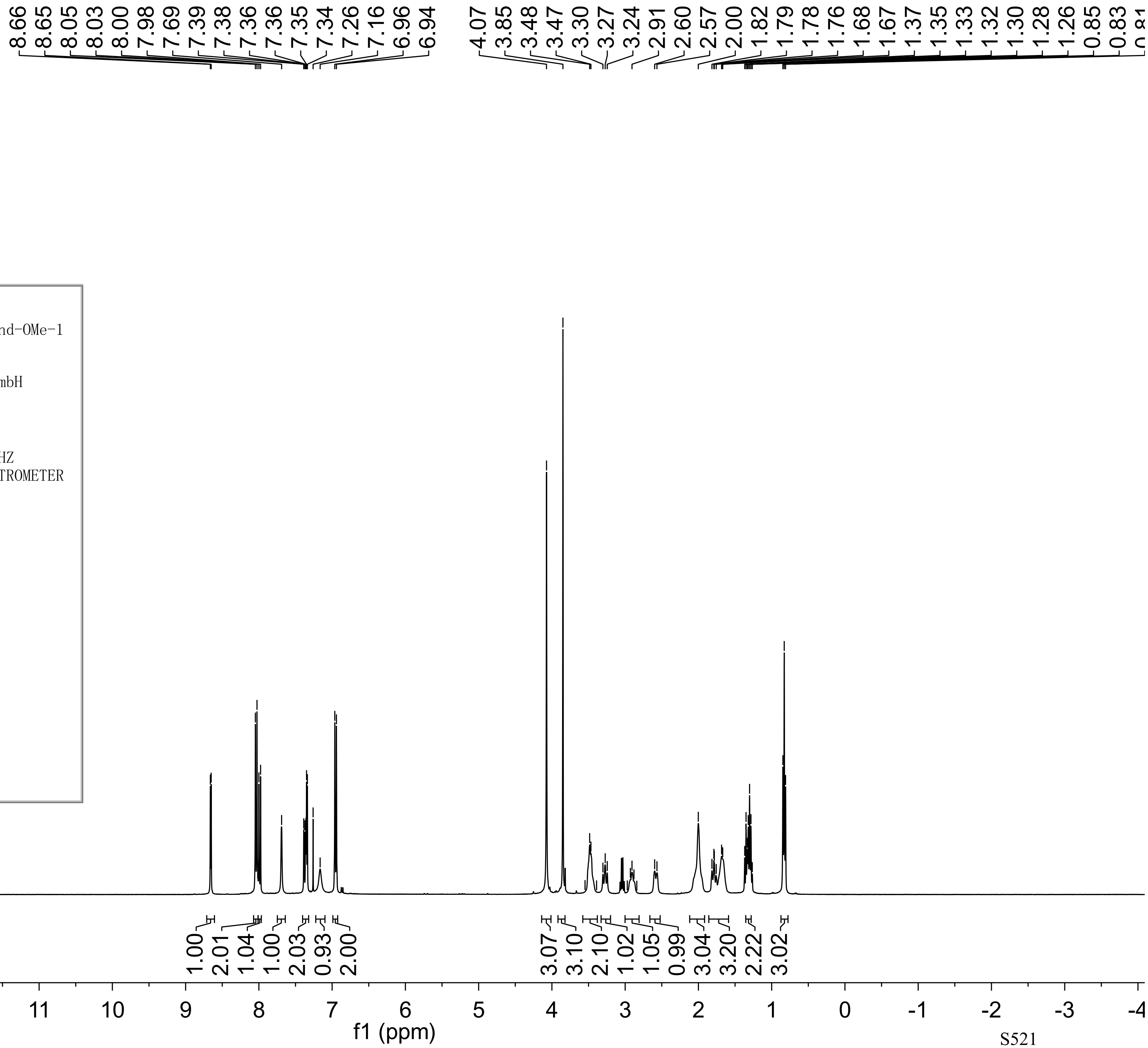

	Parameter	Value
1	Title	CFM-6-105-4-ligand-OMe-1
2	Comment	
3	Origin	Bruker BioSpin GmbH
4	Owner	nmrsu
5	Site	
6	Spectrometer	AVANCE NEO 400 MHZ DIGITAL NMR SPECTROMETER
7	Author	
8	Solvent	CDCl3
9	Temperature	298.2
10	Pulse Sequence	zg30
11	Experiment	1D
12	Number of Scans	5
13	Receiver Gain	101
14	Spectrometer Frequency	400.13
15	Spectral Width	8196.7
16	Lowest Frequency	-1637.6
17	Nucleus	1H
18	Acquired Size	32768
19	Spectral Size	65536

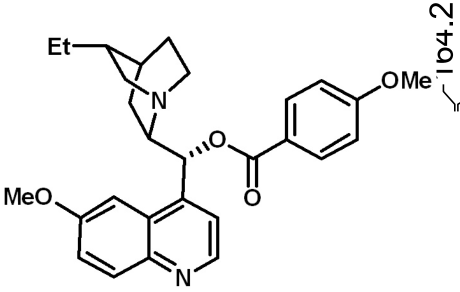

K

Parameter	Value
1 Title	CFM-6-105-4-ligand-OMe-1
2 Comment	
3 Origin	Bruker BioSpin GmbH
4 Owner	nmrsu
5 Site	
6 Spectrometer	AVANCE NEO 400 MHZ DIGITAL NMR SPECTROMETER
7 Author	
8 Solvent	CDCl3
9 Temperature	298.2
10 Pulse Sequence	zgpg30
11 Experiment	1D
12 Number of Scans	146
13 Receiver Gain	59
14 Spectrometer Frequency	100.61
15 Spectral Width	23809.5
16 Lowest Frequency	-1853.5
17 Nucleus	13C
18 Acquired Size	32768
19 Spectral Size	32768

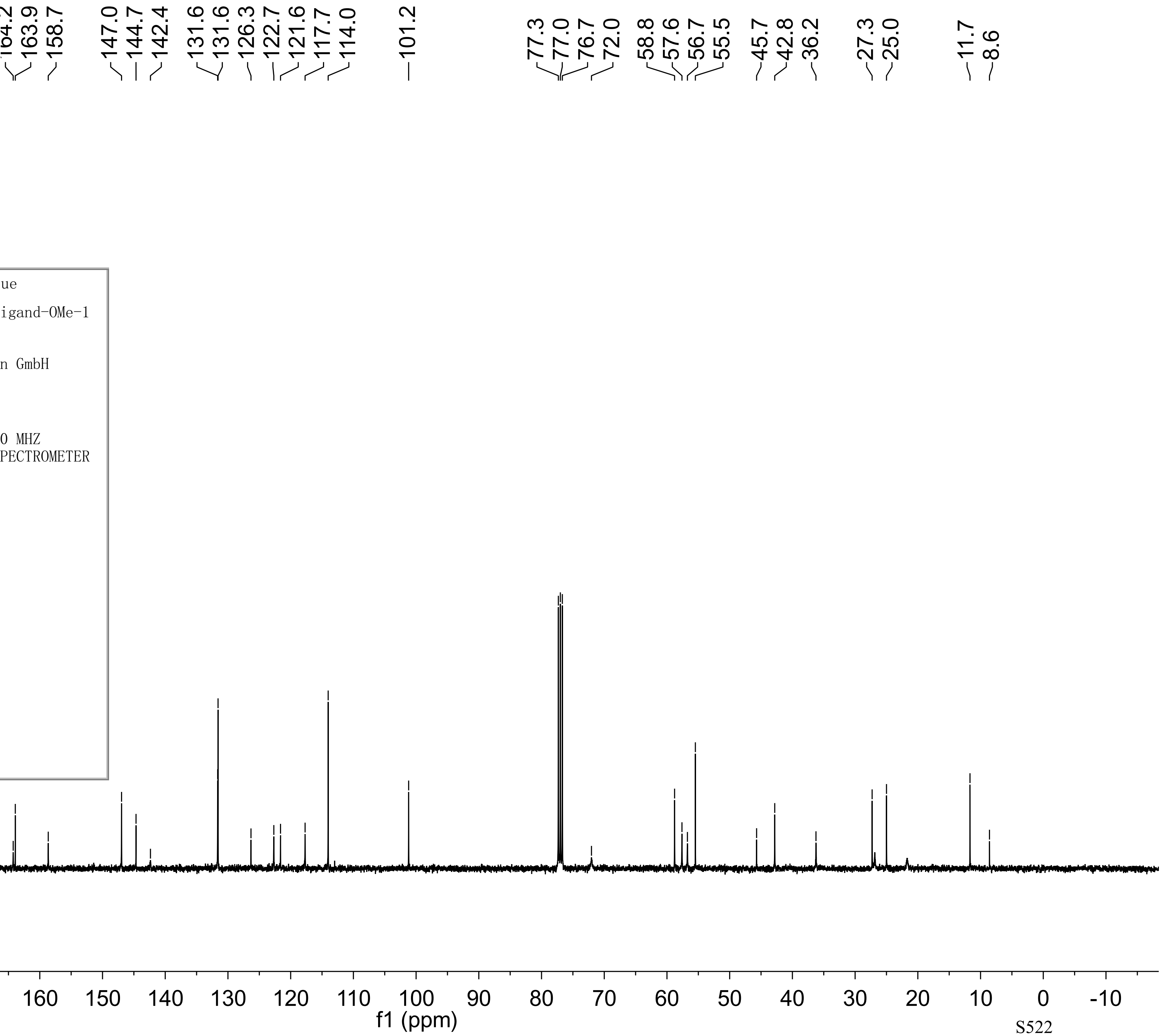

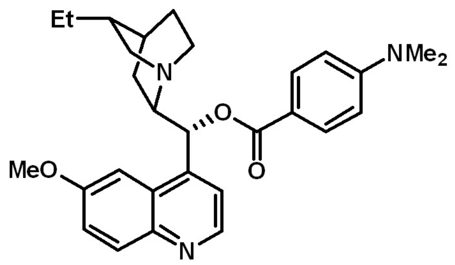

L

	Parameter	Value
1	Title	cfm-6-105-6-ligand-NMe2
2	Comment	
3	Origin	Bruker BioSpin GmbH
4	Owner	nmrsu
5	Site	
6	Spectrometer	Avance NEO 600
7	Author	
8	Solvent	CDCl3
9	Temperature	298.1
10	Pulse Sequence	zg30
11	Experiment	1D
12	Number of Scans	8
13	Receiver Gain	101
14	Spectrometer Frequency	600.15
15	Spectral Width	11904.8
16	Lowest Frequency	-2260.1
17	Nucleus	1H
18	Acquired Size	32768
19	Spectral Size	65536

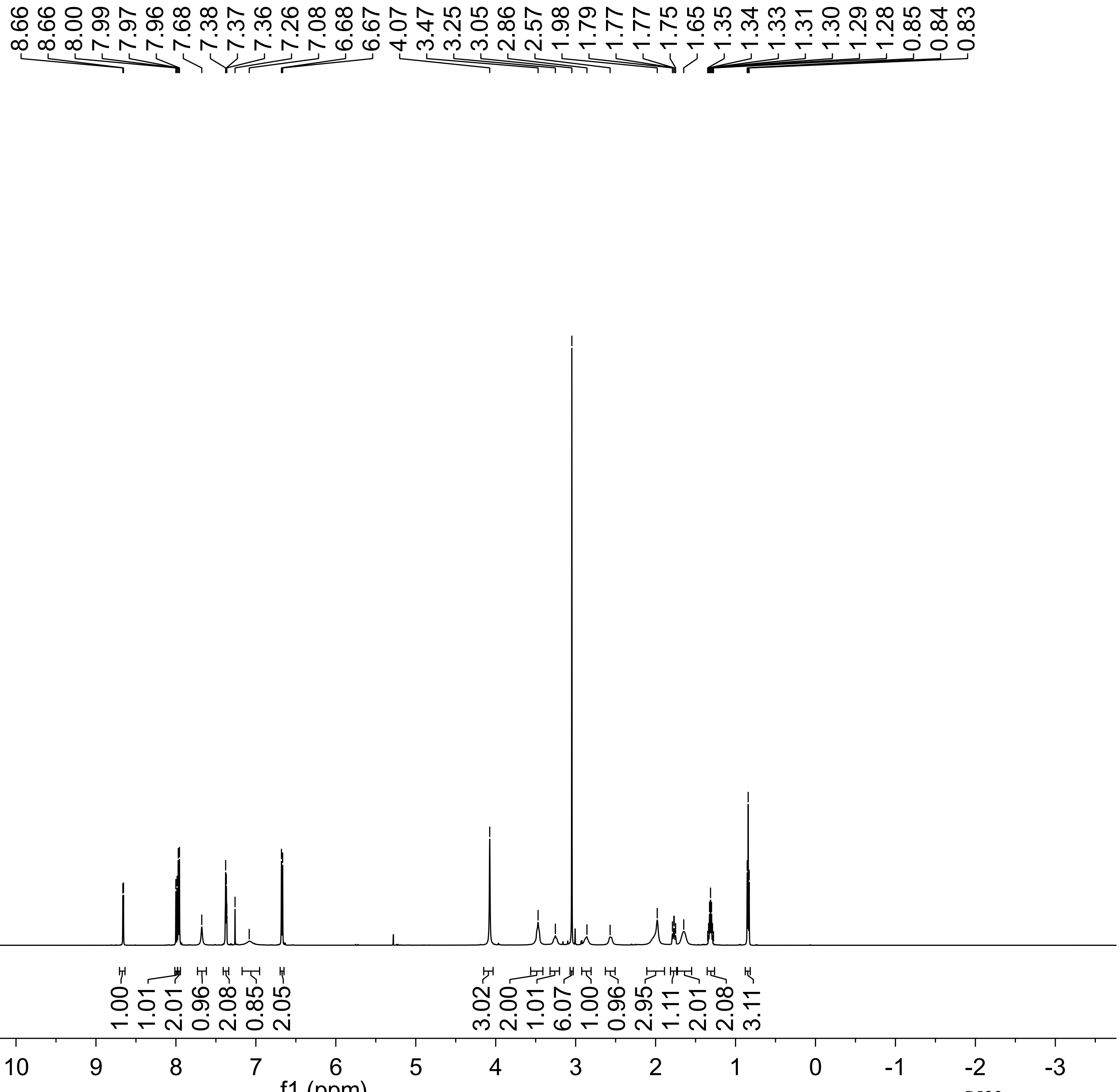

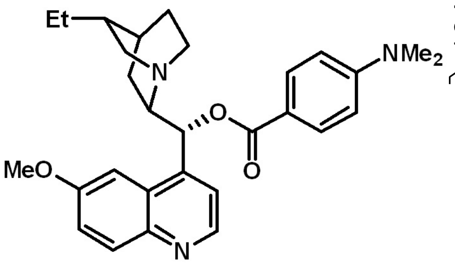

L

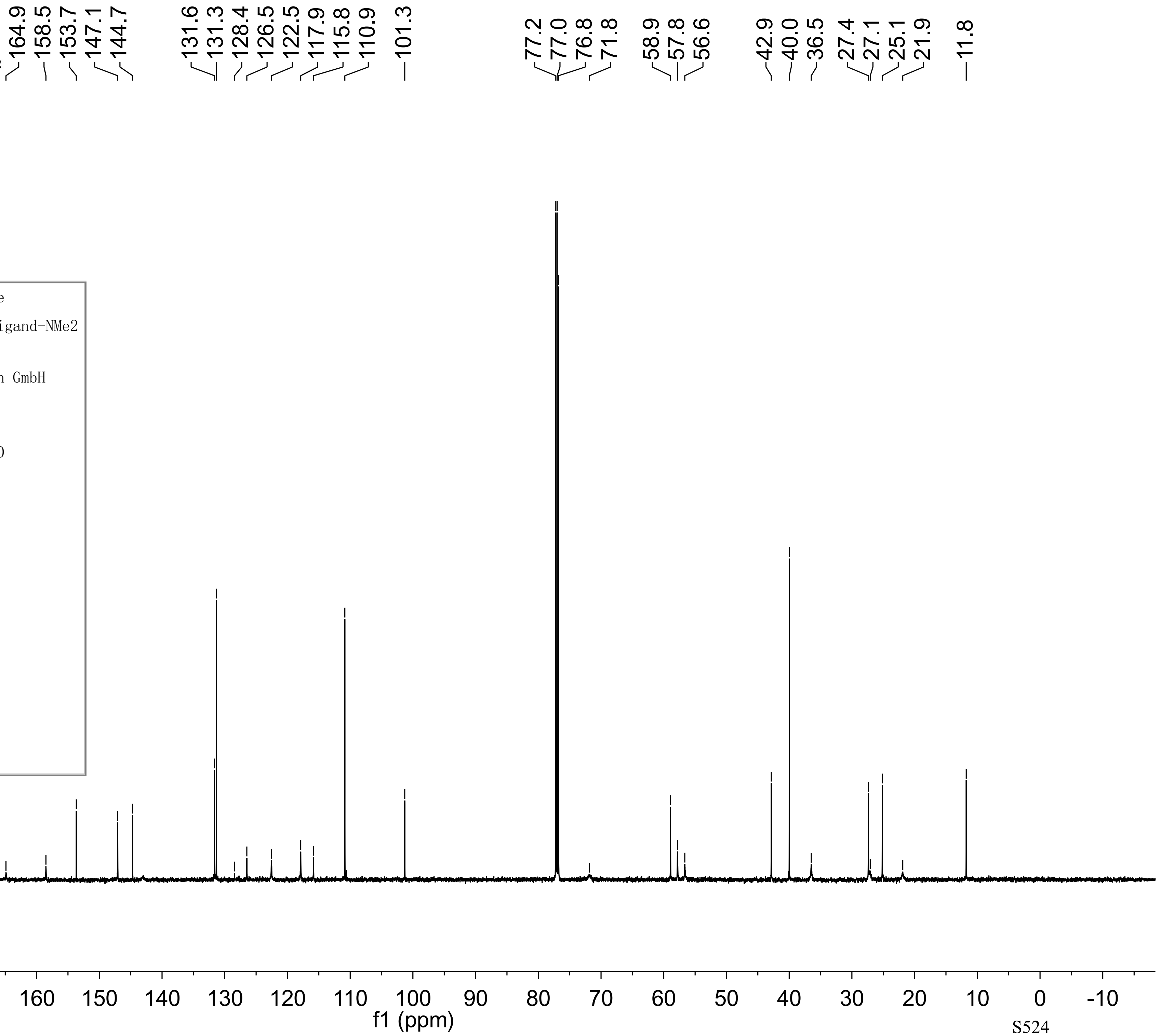

	Parameter	Value
1	Title	cfm-6-105-6-ligand-NMe2
2	Comment	
3	Origin	Bruker BioSpin GmbH
4	Owner	nmrsu
5	Site	
6	Spectrometer	Avance NEO 600
7	Author	
8	Solvent	CDCl3
9	Temperature	298.2
10	Pulse Sequence	zgpg30
11	Experiment	1D
12	Number of Scans	56
13	Receiver Gain	101
14	Spectrometer Frequency	150.91
15	Spectral Width	35714.3
16	Lowest Frequency	-2776.6
17	Nucleus	13C
18	Acquired Size	32768
19	Spectral Size	32768

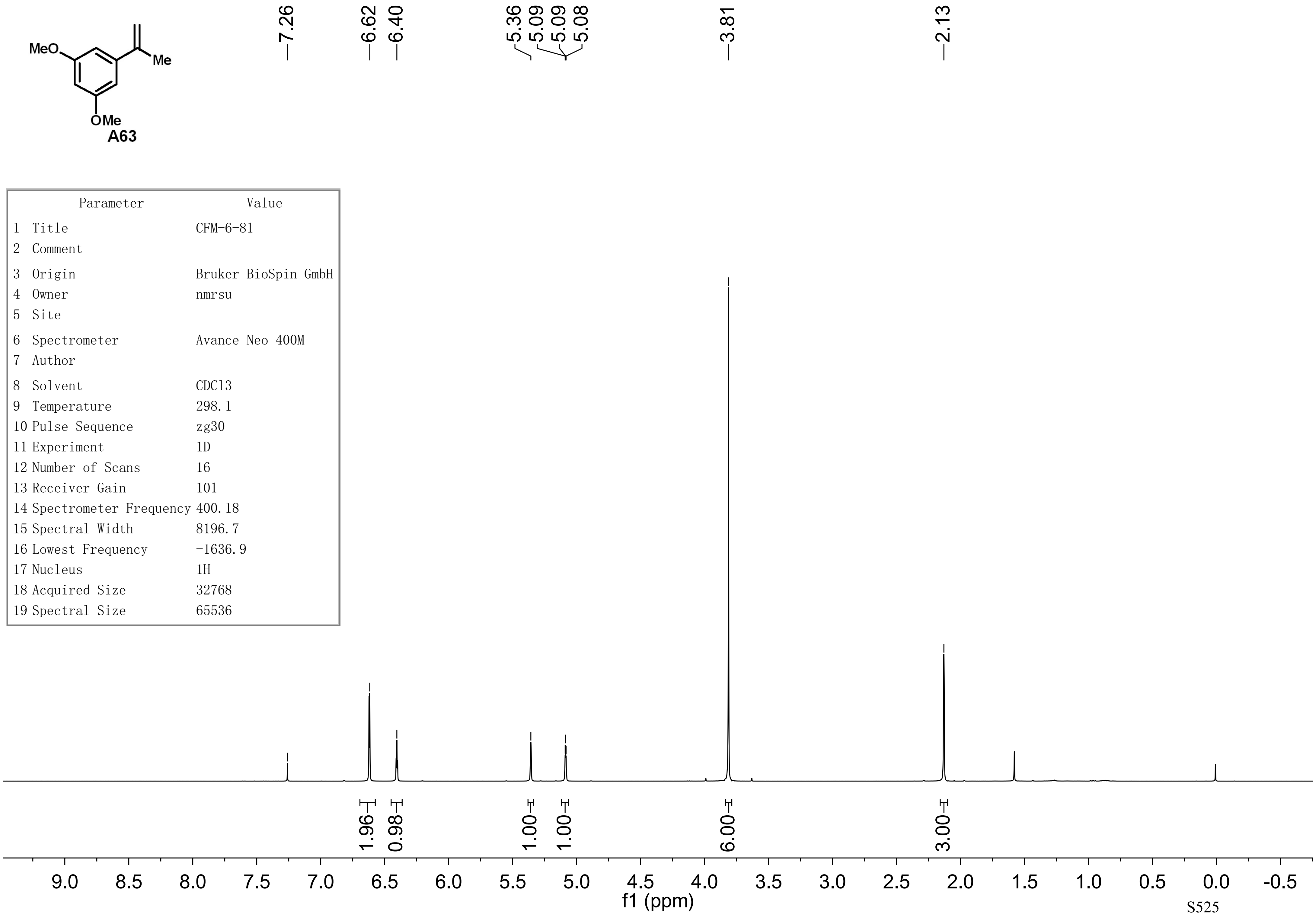

A63

	Parameter	Value
1	Title	CFM-6-81
2	Comment	
3	Origin	Bruker BioSpin GmbH
4	Owner	nmrsu
5	Site	
6	Spectrometer	Avance Neo 400M
7	Author	
8	Solvent	CDCl3
9	Temperature	298.1
10	Pulse Sequence	zg30
11	Experiment	1D
12	Number of Scans	16
13	Receiver Gain	101
14	Spectrometer Frequency	400.18
15	Spectral Width	8196.7
16	Lowest Frequency	-1636.9
17	Nucleus	1H
18	Acquired Size	32768
19	Spectral Size	65536

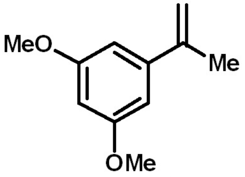

A63

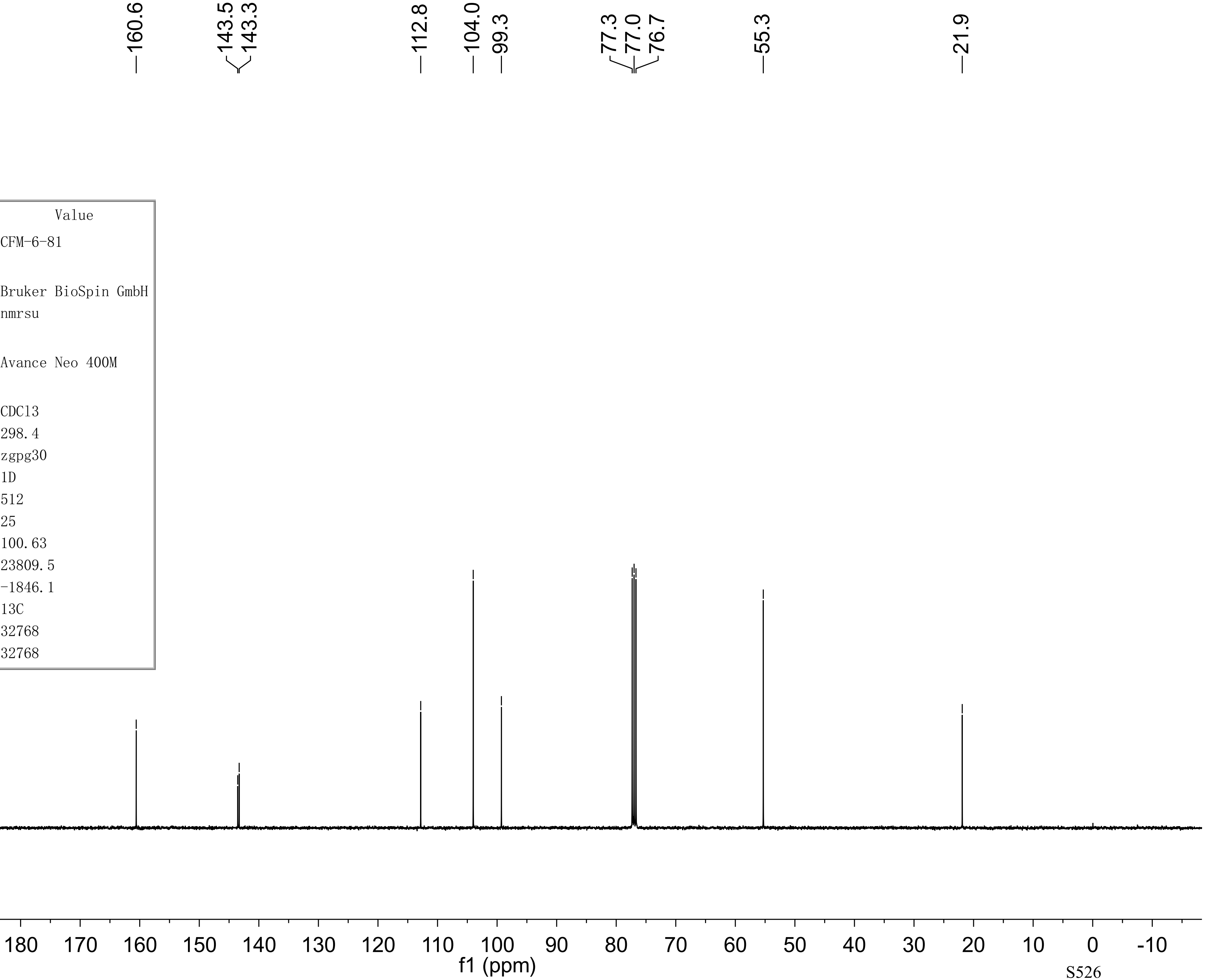

	Parameter	Value
1	Title	CFM-6-81
2	Comment	
3	Origin	Bruker BioSpin GmbH
4	Owner	nmrsu
5	Site	
6	Spectrometer	Avance Neo 400M
7	Author	
8	Solvent	CDCl3
9	Temperature	298.4
10	Pulse Sequence	zgpg30
11	Experiment	1D
12	Number of Scans	512
13	Receiver Gain	25
14	Spectrometer Frequency	100.63
15	Spectral Width	23809.5
16	Lowest Frequency	-1846.1
17	Nucleus	13C
18	Acquired Size	32768
19	Spectral Size	32768

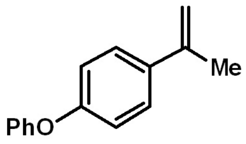

A66

	Parameter	Value
1	Title	CFM-6-94
2	Comment	
3	Origin	Bruker BioSpin GmbH
4	Owner	nmrsu
5	Site	
6	Spectrometer	Avance Neo 400M
7	Author	
8	Solvent	CDCl3
9	Temperature	298.2
10	Pulse Sequence	zg30
11	Experiment	1D
12	Number of Scans	16
13	Receiver Gain	101
14	Spectrometer Frequency	400.18
15	Spectral Width	8196.7
16	Lowest Frequency	-1636.7
17	Nucleus	1H
18	Acquired Size	32768
19	Spectral Size	65536

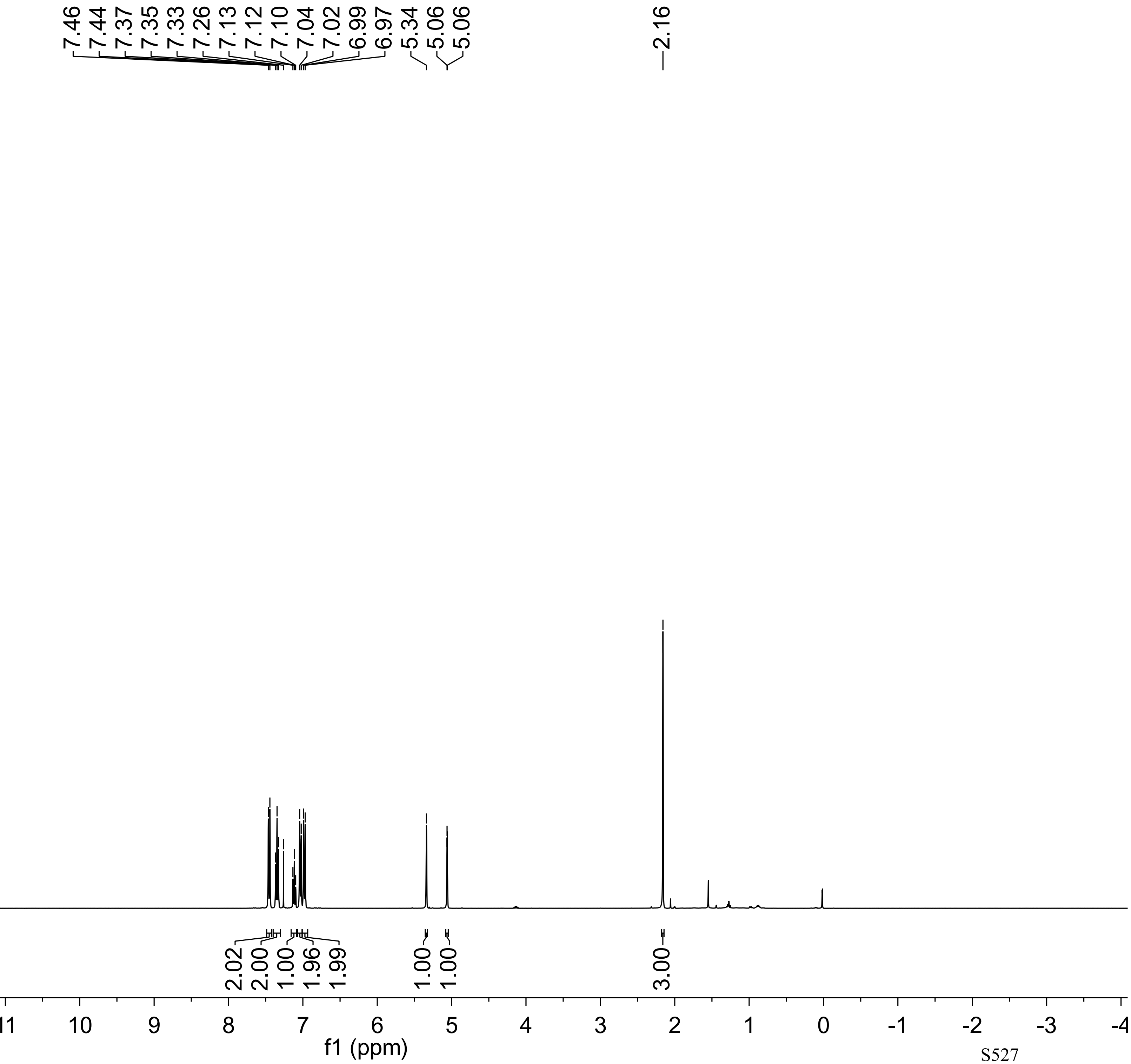

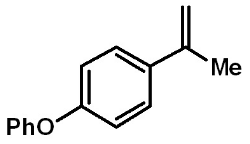

A66

Parameter	Value
1 Title	CFM-6-94
2 Comment	
3 Origin	Bruker BioSpin GmbH
4 Owner	nmrsu
5 Site	
6 Spectrometer	Avance Neo 400M
7 Author	
8 Solvent	CDCl3
9 Temperature	298.2
10 Pulse Sequence	zgpg30
11 Experiment	1D
12 Number of Scans	512
13 Receiver Gain	34
14 Spectrometer Frequency	100.63
15 Spectral Width	23809.5
16 Lowest Frequency	-1846.4
17 Nucleus	13C
18 Acquired Size	32768
19 Spectral Size	32768

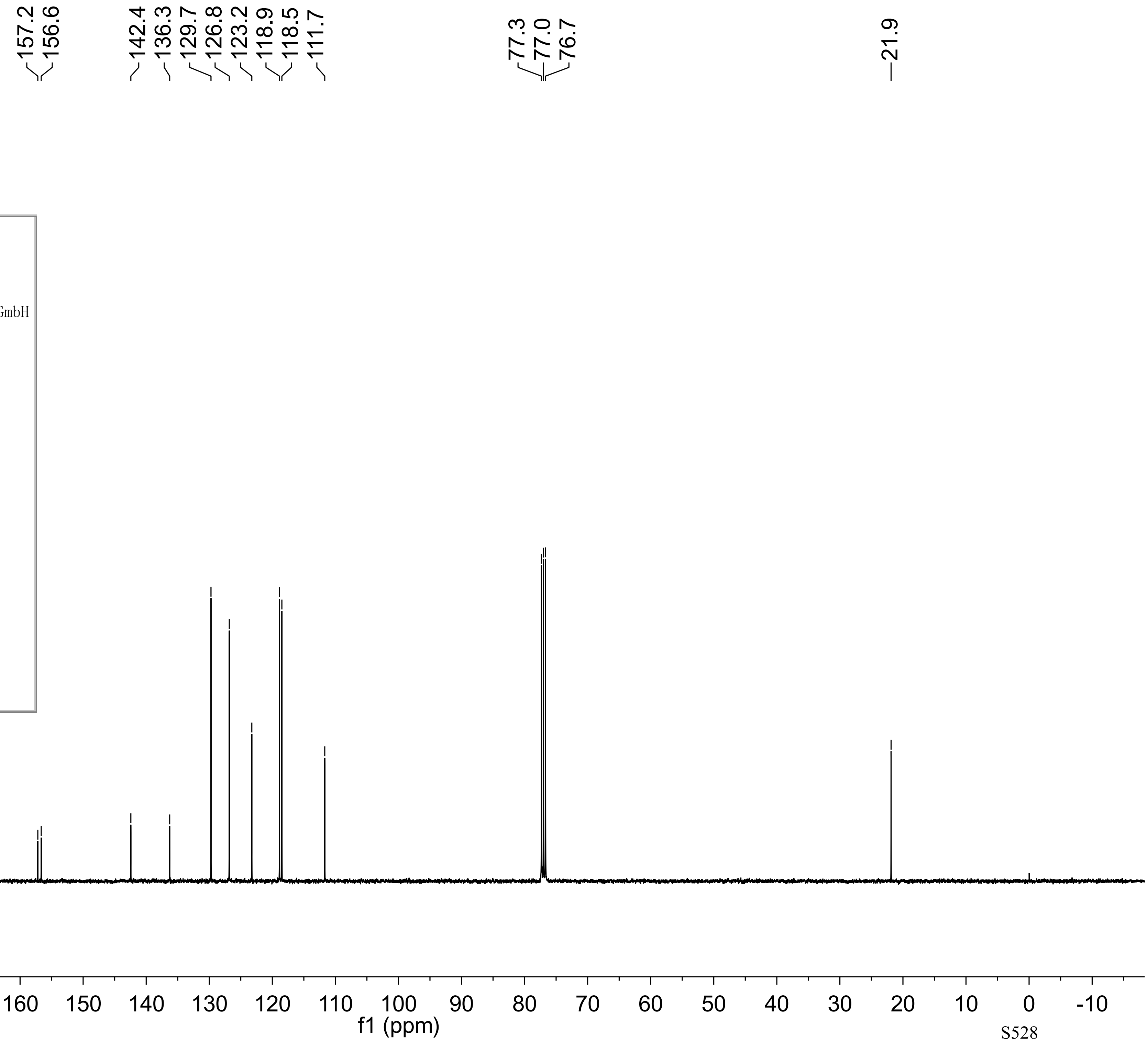

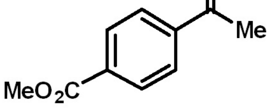

A69

	Parameter	Value
1	Title	CFM-6-66
2	Comment	
3	Origin	Bruker BioSpin GmbH
4	Owner	nmrsu
5	Site	
6	Spectrometer	AVANCE NEO 400 MHZ DIGITAL NMR SPECTROMETER
7	Author	
8	Solvent	CDCl3
9	Temperature	298.1
10	Pulse Sequence	zg30
11	Experiment	1D
12	Number of Scans	8
13	Receiver Gain	101
14	Spectrometer Frequency	400.13
15	Spectral Width	8196.7
16	Lowest Frequency	-1636.8
17	Nucleus	1H
18	Acquired Size	32768
19	Spectral Size	65536

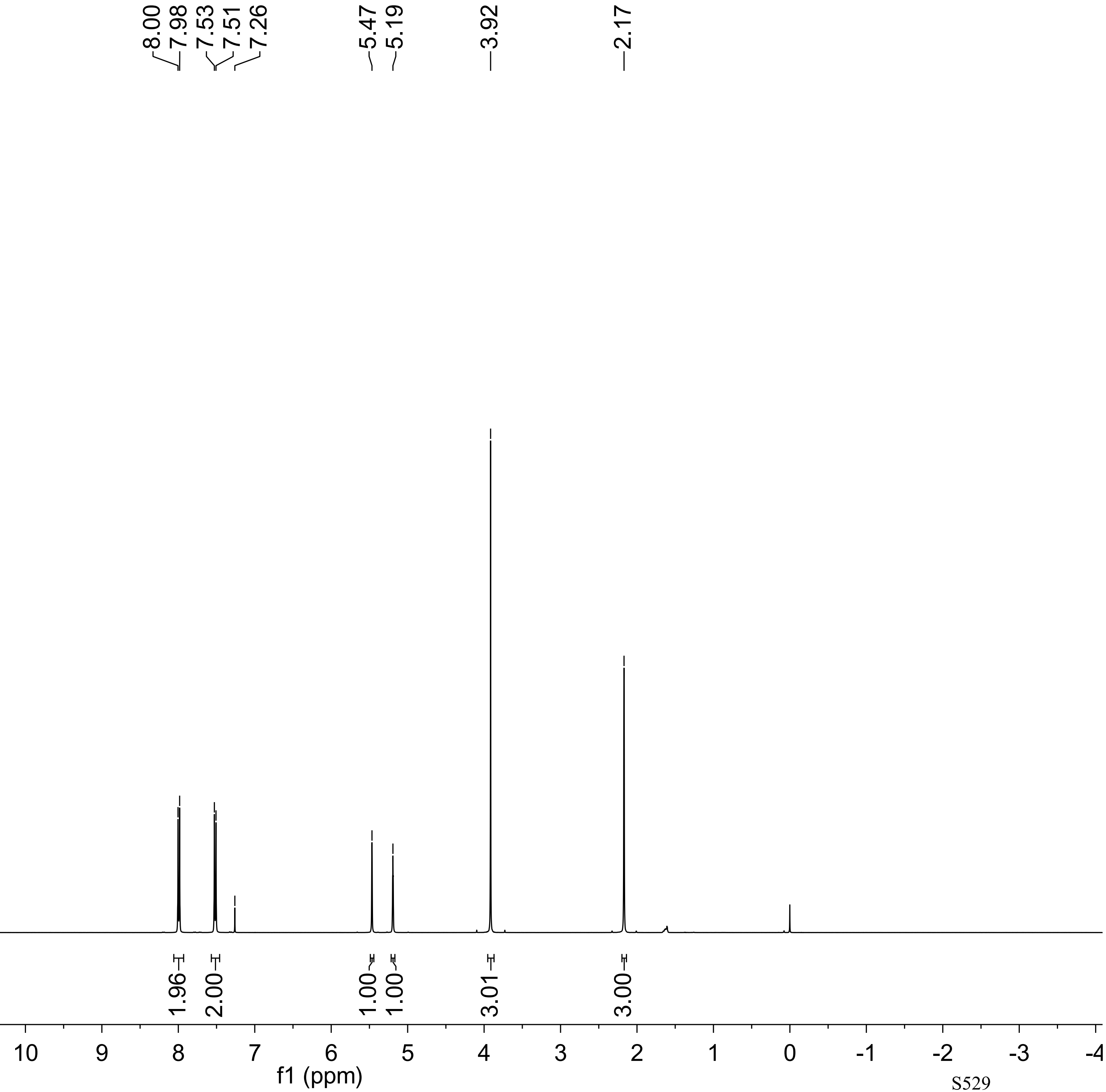

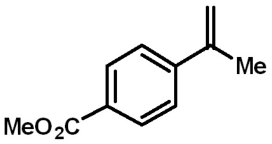

A69

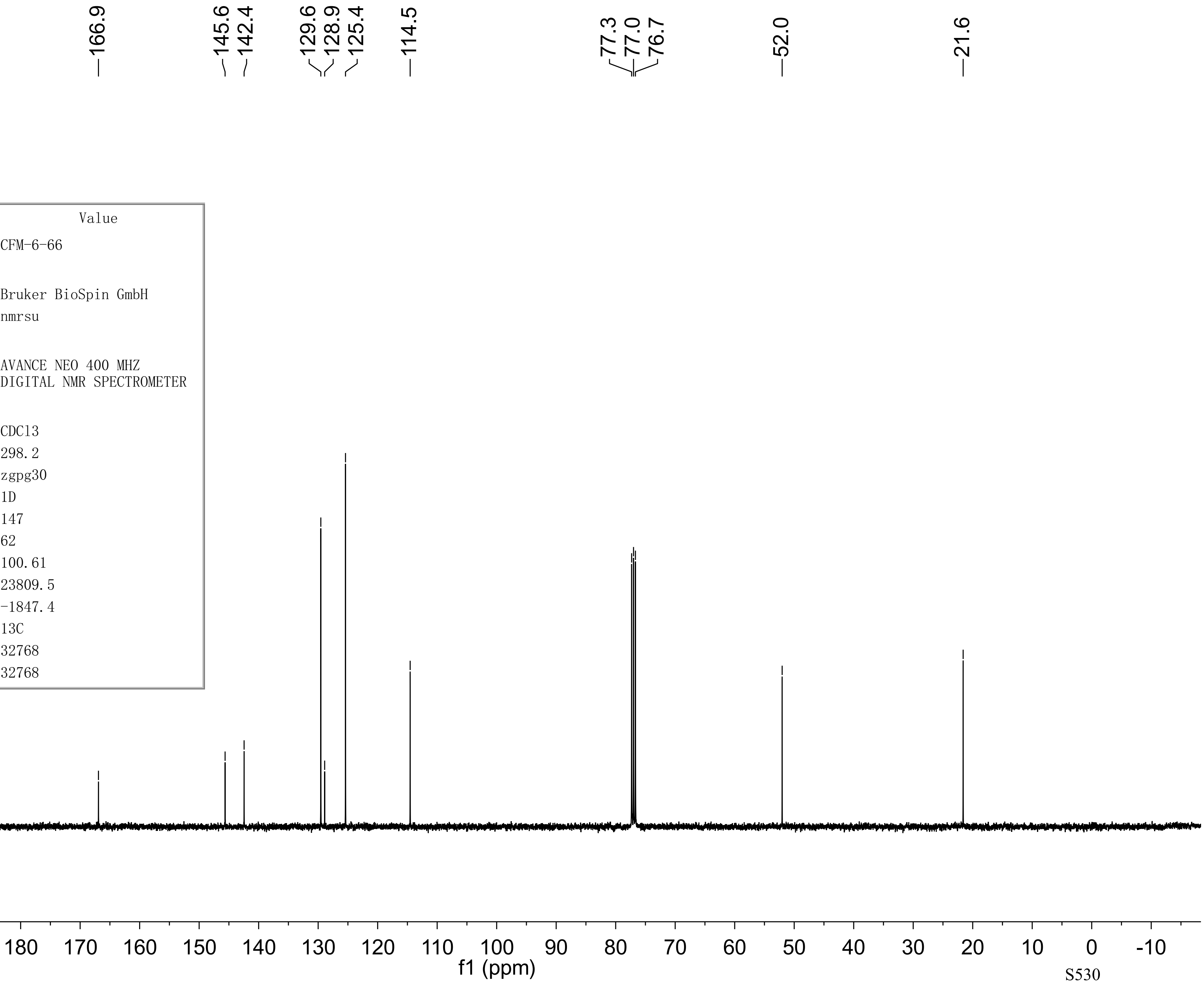

	Parameter	Value
1	Title	CFM-6-66
2	Comment	
3	Origin	Bruker BioSpin GmbH
4	Owner	nmrsu
5	Site	
6	Spectrometer	AVANCE NEO 400 MHZ DIGITAL NMR SPECTROMETER
7	Author	
8	Solvent	CDCl3
9	Temperature	298.2
10	Pulse Sequence	zgpg30
11	Experiment	1D
12	Number of Scans	147
13	Receiver Gain	62
14	Spectrometer Frequency	100.61
15	Spectral Width	23809.5
16	Lowest Frequency	-1847.4
17	Nucleus	13C
18	Acquired Size	32768
19	Spectral Size	32768

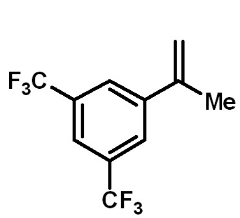

A71

Parameter	Value
1 Title	CFM-6-93
2 Comment	
3 Origin	Bruker BioSpin GmbH
4 Owner	nmrsu
5 Site	
6 Spectrometer	Avance Neo 400M
7 Author	
8 Solvent	CDCl3
9 Temperature	298.1
10 Pulse Sequence	zg30
11 Experiment	1D
12 Number of Scans	16
13 Receiver Gain	101
14 Spectrometer Frequency	400.18
15 Spectral Width	8196.7
16 Lowest Frequency	-1636.8
17 Nucleus	1H
18 Acquired Size	32768
19 Spectral Size	65536

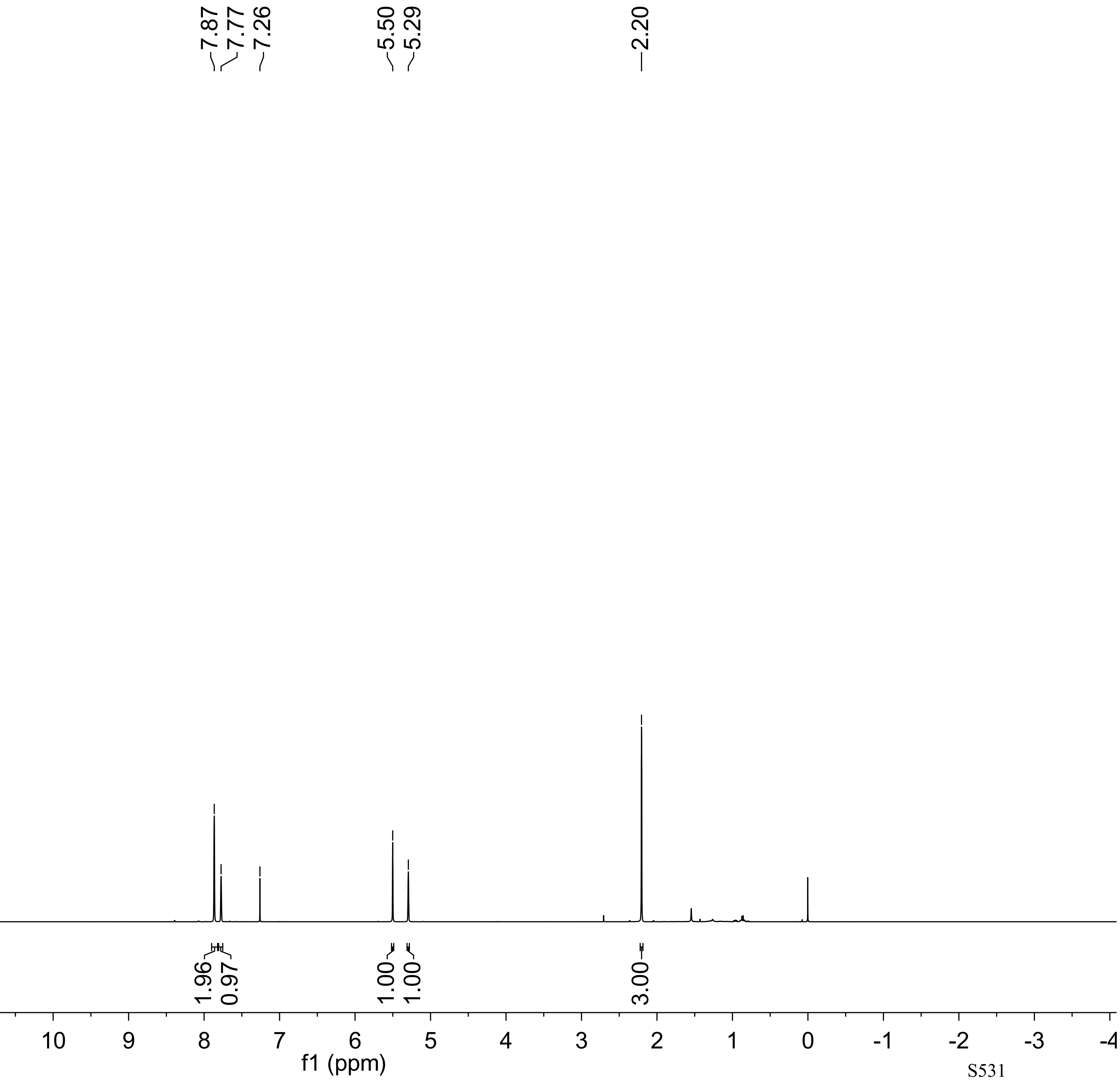

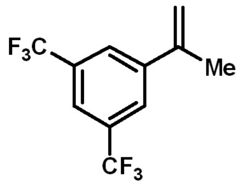

A71

Parameter		Value
1	Title	CFM-6-93
2	Comment	
3	Origin	Bruker BioSpin GmbH
4	Owner	nmrsu
5	Site	
6	Spectrometer	Avance Neo 400M
7	Author	
8	Solvent	CDCl3
9	Temperature	298.1
10	Pulse Sequence	zgpg30
11	Experiment	1D
12	Number of Scans	1024
13	Receiver Gain	32
14	Spectrometer Frequency	100.63
15	Spectral Width	23809.5
16	Lowest Frequency	-1843.0
17	Nucleus	13C
18	Acquired Size	32768
19	Spectral Size	32768

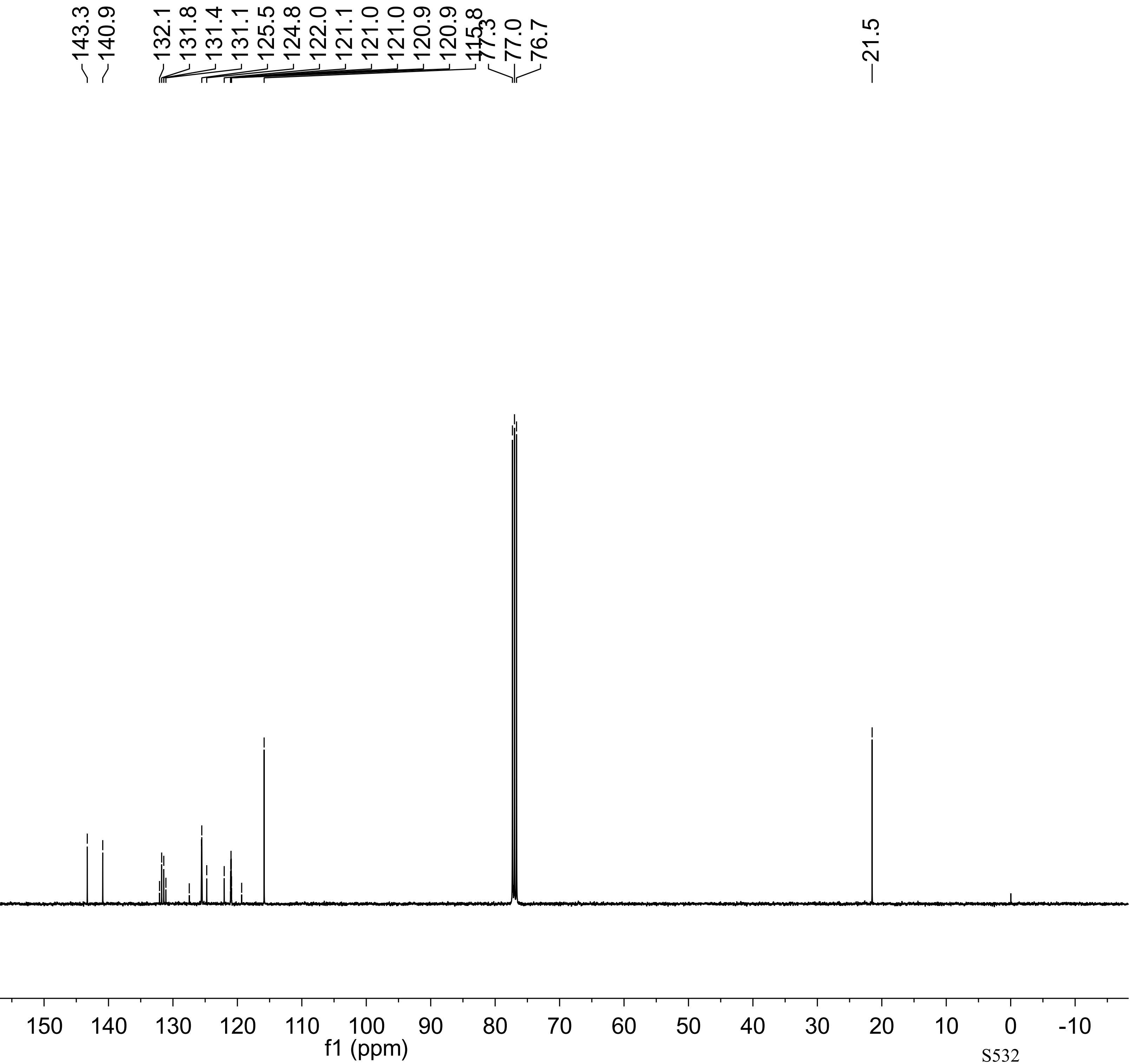

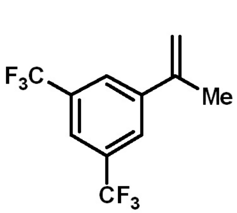

A71

Parameter	Value
1 Title	CFM-6-93
2 Comment	
3 Origin	Bruker BioSpin GmbH
4 Owner	nmrsu
5 Site	
6 Spectrometer	Avance Neo 400M
7 Author	
8 Solvent	CDCl3
9 Temperature	298.0
10 Pulse Sequence	zg
11 Experiment	1D
12 Number of Scans	16
13 Receiver Gain	101
14 Spectrometer Frequency	376.55
15 Spectral Width	90909.1
16 Lowest Frequency	-83109.1
17 Nucleus	19F
18 Acquired Size	65536
19 Spectral Size	65536

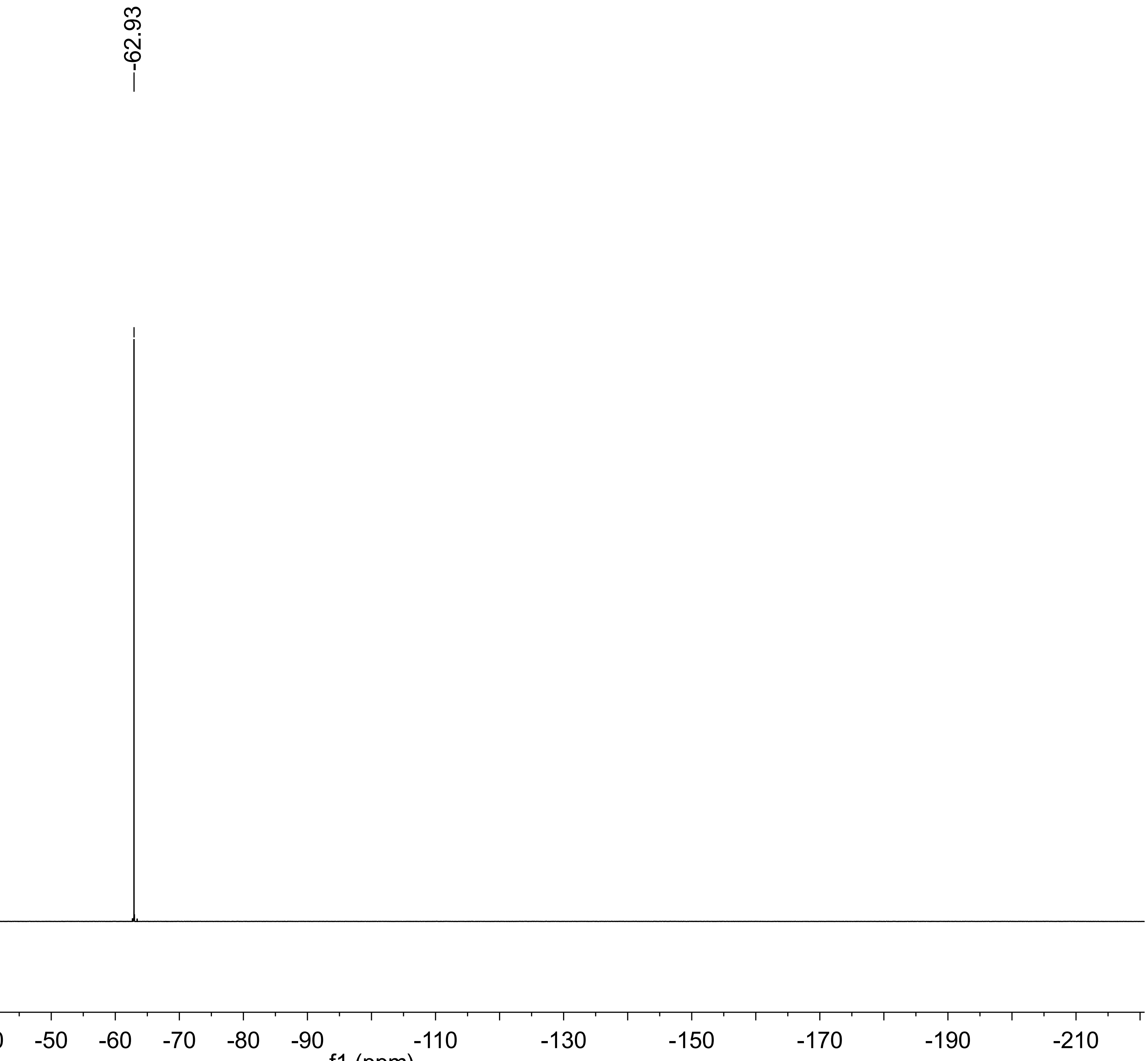

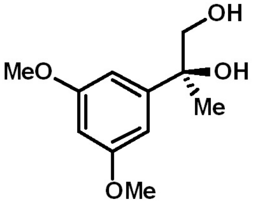

C63

Parameter	Value
1 Title	CFM-6-100-1
2 Comment	
3 Origin	Bruker BioSpin GmbH
4 Owner	nmrsu
5 Site	
6 Spectrometer	Avance Neo 400M
7 Author	
8 Solvent	CDCl3
9 Temperature	298.2
10 Pulse Sequence	zg30
11 Experiment	1D
12 Number of Scans	8
13 Receiver Gain	101
14 Spectrometer Frequency	400.18
15 Spectral Width	8196.7
16 Lowest Frequency	-1636.9
17 Nucleus	1H
18 Acquired Size	32768
19 Spectral Size	65536

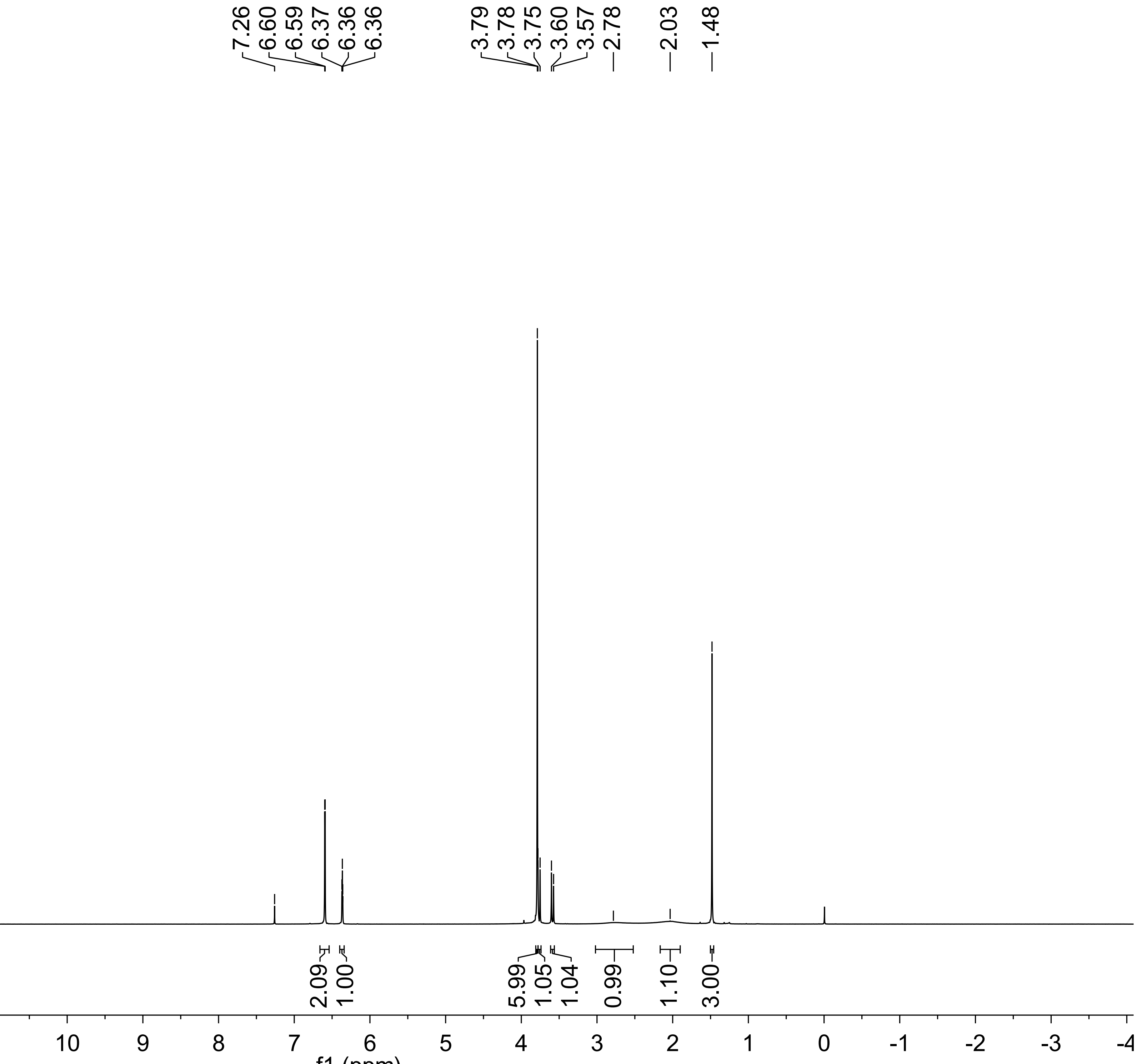

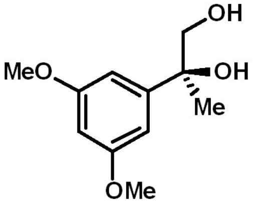

C63

Parameter	Value
1 Title	CFM-6-100-1
2 Comment	
3 Origin	Bruker BioSpin GmbH
4 Owner	nmrsu
5 Site	
6 Spectrometer	Avance Neo 400M
7 Author	
8 Solvent	CDCl3
9 Temperature	298.2
10 Pulse Sequence	zgpg30
11 Experiment	1D
12 Number of Scans	92
13 Receiver Gain	33
14 Spectrometer Frequency	100.63
15 Spectral Width	23809.5
16 Lowest Frequency	-1847.5
17 Nucleus	13C
18 Acquired Size	32768
19 Spectral Size	32768

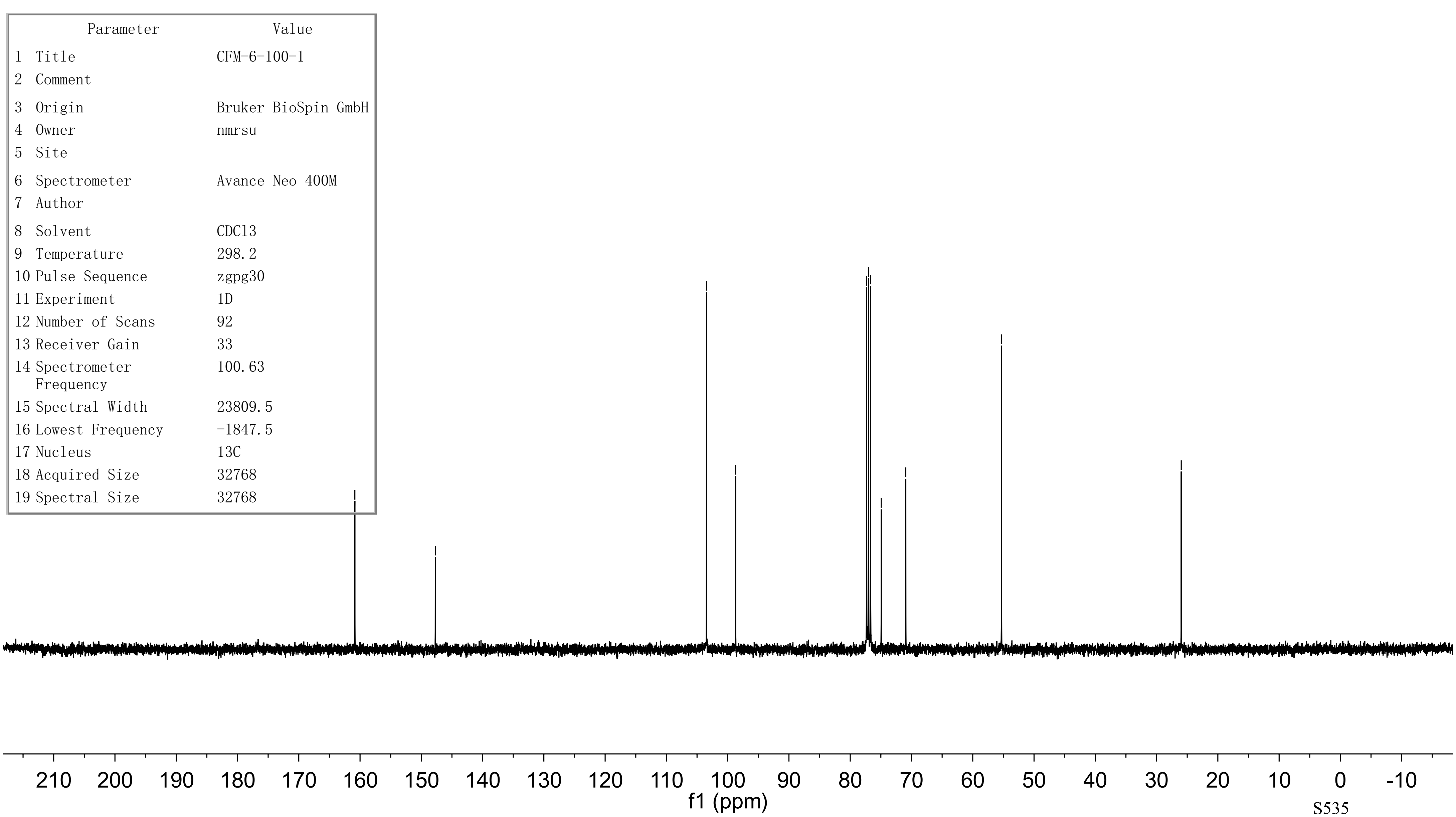

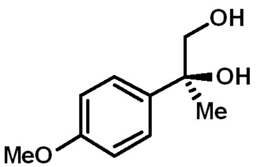

C64

Parameter	Value
1 Title	CFM-6-100-2-1006
2 Comment	
3 Origin	Bruker BioSpin GmbH
4 Owner	nmrsu
5 Site	
6 Spectrometer	Avance Neo 400M
7 Author	
8 Solvent	CDCl3
9 Temperature	298.2
10 Pulse Sequence	zg30
11 Experiment	1D
12 Number of Scans	11
13 Receiver Gain	101
14 Spectrometer Frequency	400.18
15 Spectral Width	8196.7
16 Lowest Frequency	-1636.9
17 Nucleus	1H
18 Acquired Size	32768
19 Spectral Size	65536

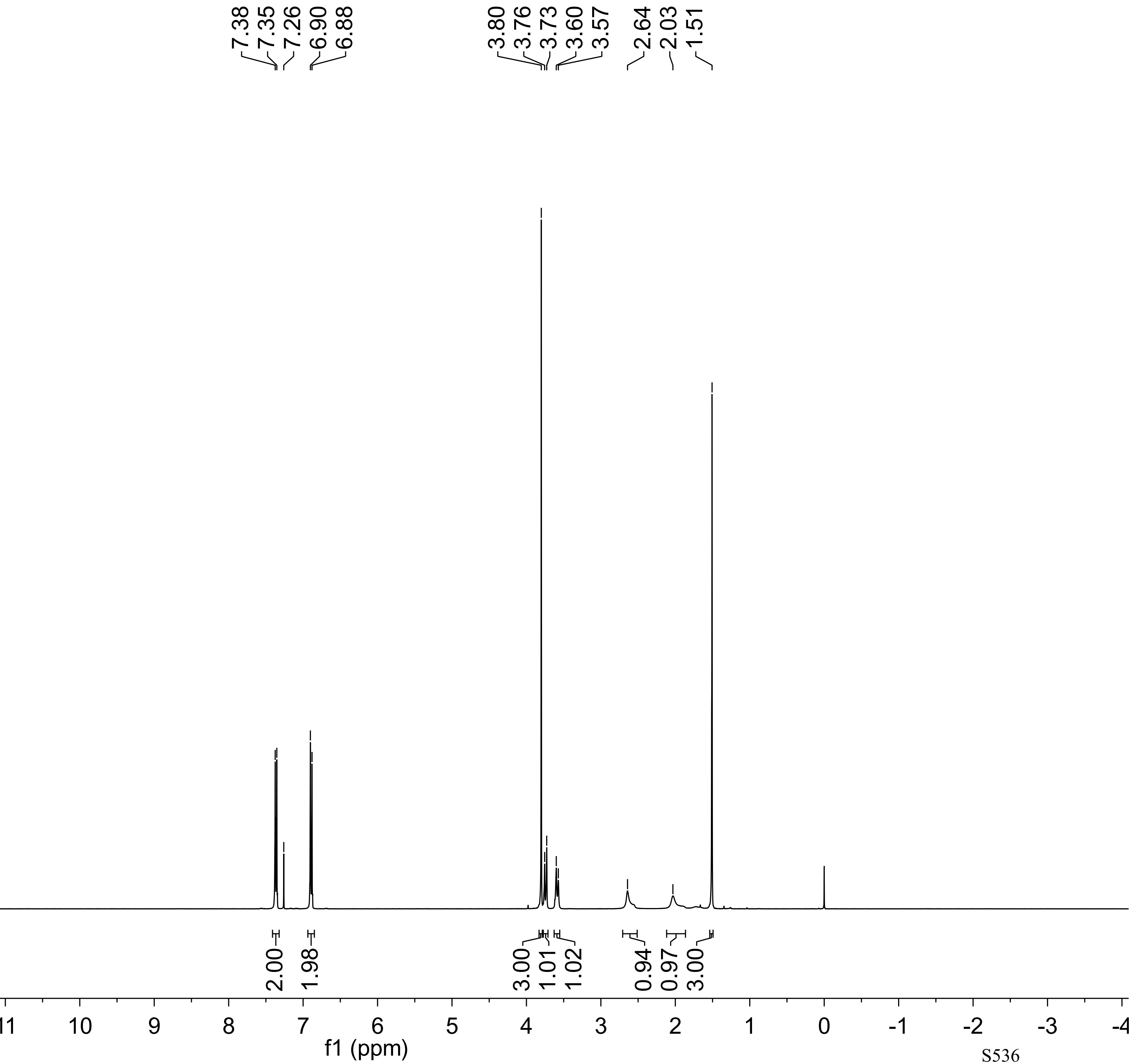

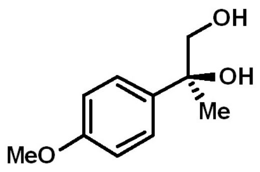

C64

Parameter		Value
1	Title	CFM-6-100-2-1006
2	Comment	
3	Origin	Bruker BioSpin GmbH
4	Owner	nmrsu
5	Site	
6	Spectrometer	Avance Neo 400M
7	Author	
8	Solvent	CDCl3
9	Temperature	298.2
10	Pulse Sequence	zgpg30
11	Experiment	1D
12	Number of Scans	35
13	Receiver Gain	33
14	Spectrometer Frequency	100.63
15	Spectral Width	23809.5
16	Lowest Frequency	-1846.9
17	Nucleus	13C
18	Acquired Size	32768
19	Spectral Size	32768

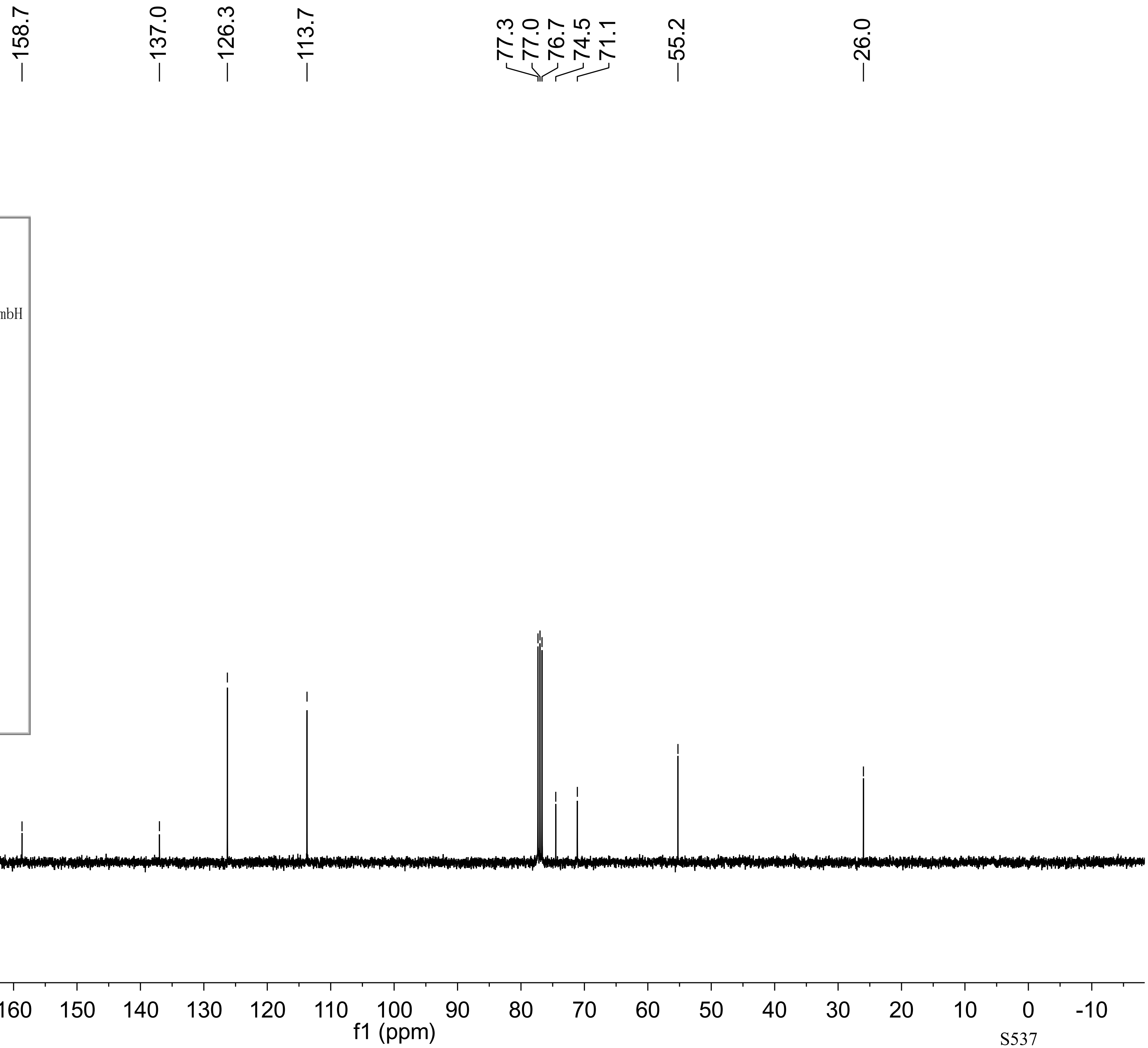

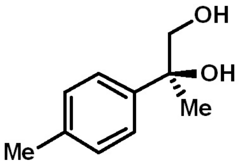

C65

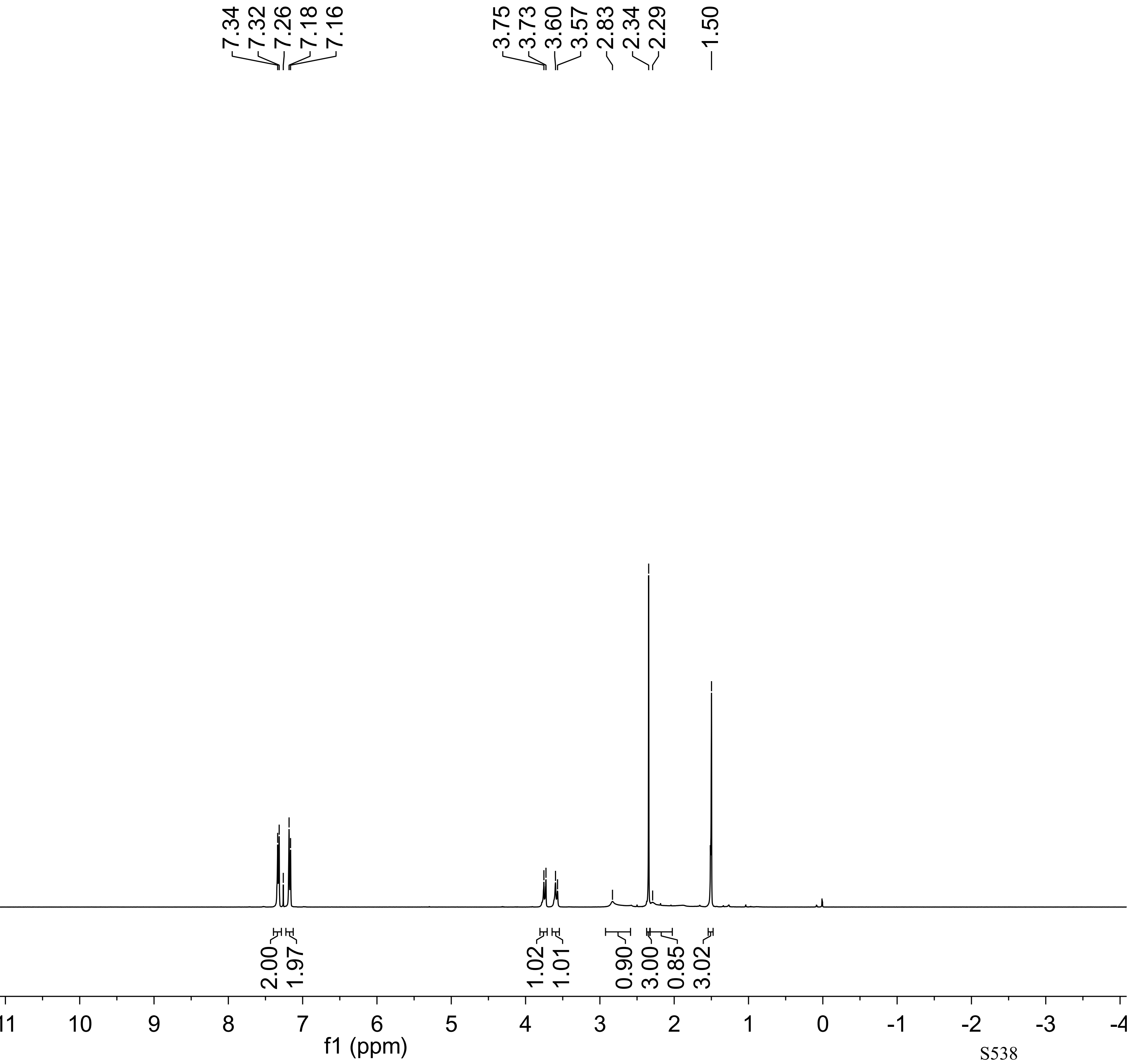

	Parameter	Value
1	Title	CFM-6-100-3-1006
2	Comment	
3	Origin	Bruker BioSpin GmbH
4	Owner	nmrsu
5	Site	
6	Spectrometer	Avance Neo 400M
7	Author	
8	Solvent	CDCl3
9	Temperature	298.1
10	Pulse Sequence	zg30
11	Experiment	1D
12	Number of Scans	7
13	Receiver Gain	101
14	Spectrometer Frequency	400.18
15	Spectral Width	8196.7
16	Lowest Frequency	-1636.5
17	Nucleus	1H
18	Acquired Size	32768
19	Spectral Size	65536

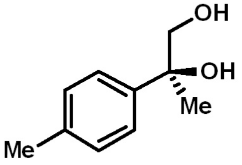

C65

Parameter	Value
1 Title	CFM-6-100-3-1006
2 Comment	
3 Origin	Bruker BioSpin GmbH
4 Owner	nmrsu
5 Site	
6 Spectrometer	Avance Neo 400M
7 Author	
8 Solvent	CDCl3
9 Temperature	298.2
10 Pulse Sequence	zgpg30
11 Experiment	1D
12 Number of Scans	42
13 Receiver Gain	32
14 Spectrometer Frequency	100.63
15 Spectral Width	23809.5
16 Lowest Frequency	-1848.8
17 Nucleus	13C
18 Acquired Size	32768
19 Spectral Size	32768

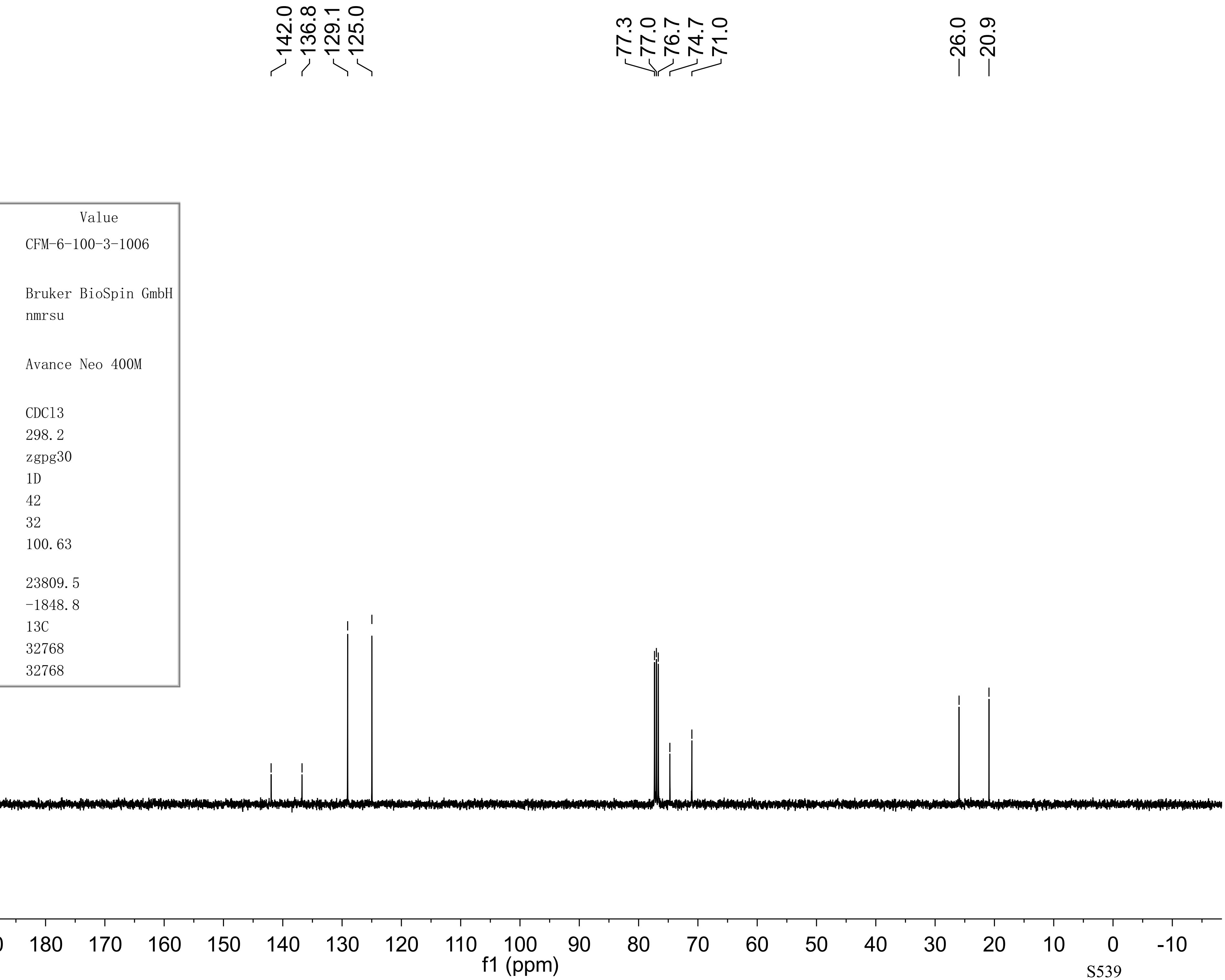

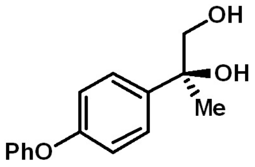

C66

Parameter	Value
1 Title	CFM-6-100-4
2 Comment	
3 Origin	Bruker BioSpin GmbH
4 Owner	nmrsu
5 Site	
6 Spectrometer	Avance Neo 400M
7 Author	
8 Solvent	CDCl3
9 Temperature	298.1
10 Pulse Sequence	zg30
11 Experiment	1D
12 Number of Scans	9
13 Receiver Gain	101
14 Spectrometer Frequency	400.18
15 Spectral Width	8196.7
16 Lowest Frequency	-1636.9
17 Nucleus	1H
18 Acquired Size	32768
19 Spectral Size	65536

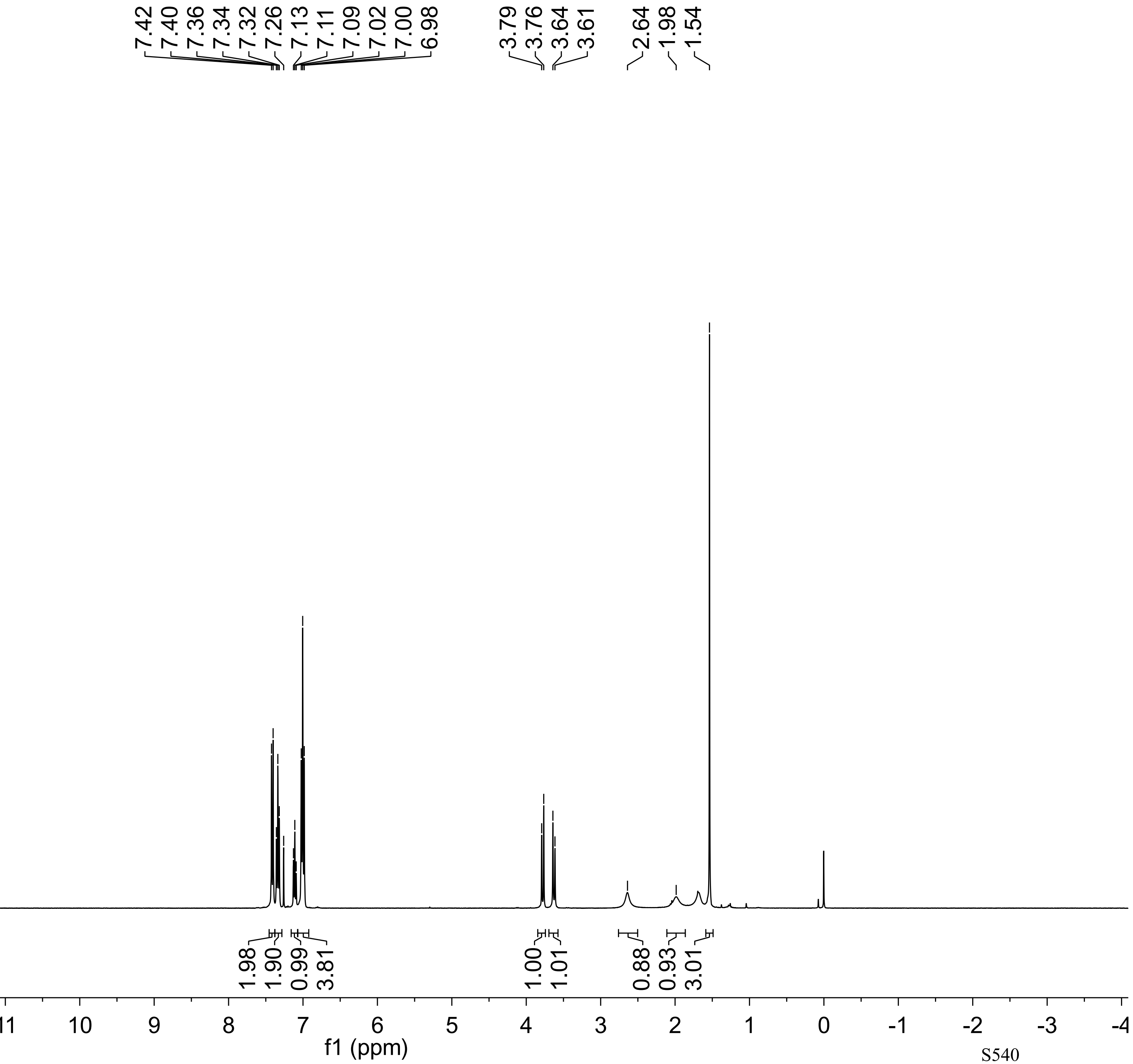

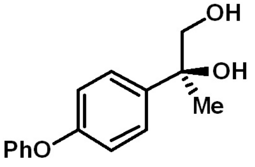

C66

Parameter	Value
1 Title	CFM-6-100-4
2 Comment	
3 Origin	Bruker BioSpin GmbH
4 Owner	nmrsu
5 Site	
6 Spectrometer	Avance Neo 400M
7 Author	
8 Solvent	CDCl3
9 Temperature	298.3
10 Pulse Sequence	zgpg30
11 Experiment	1D
12 Number of Scans	55
13 Receiver Gain	33
14 Spectrometer Frequency	100.63
15 Spectral Width	23809.5
16 Lowest Frequency	-1846.7
17 Nucleus	13C
18 Acquired Size	32768
19 Spectral Size	32768

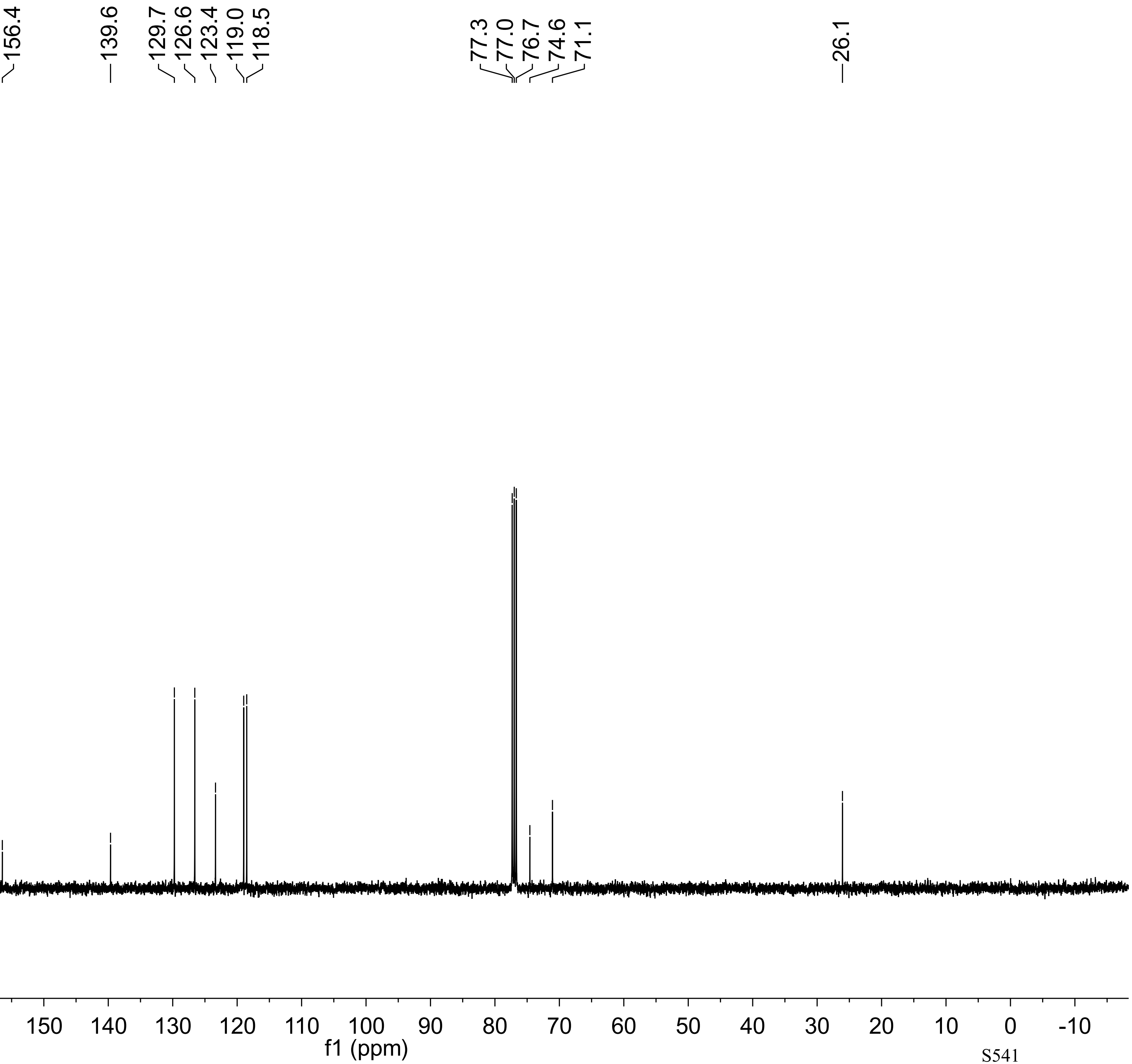

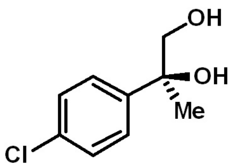

C67

	Parameter	Value
1	Title	CFM-6-100-7-1006
2	Comment	
3	Origin	Bruker BioSpin GmbH
4	Owner	nmrsu
5	Site	
6	Spectrometer	Avance NEO 600
7	Author	
8	Solvent	CDCl3
9	Temperature	298.1
10	Pulse Sequence	zg30
11	Experiment	1D
12	Number of Scans	8
13	Receiver Gain	57
14	Spectrometer Frequency	600.15
15	Spectral Width	11904.8
16	Lowest Frequency	-2260.3
17	Nucleus	1H
18	Acquired Size	32768
19	Spectral Size	65536

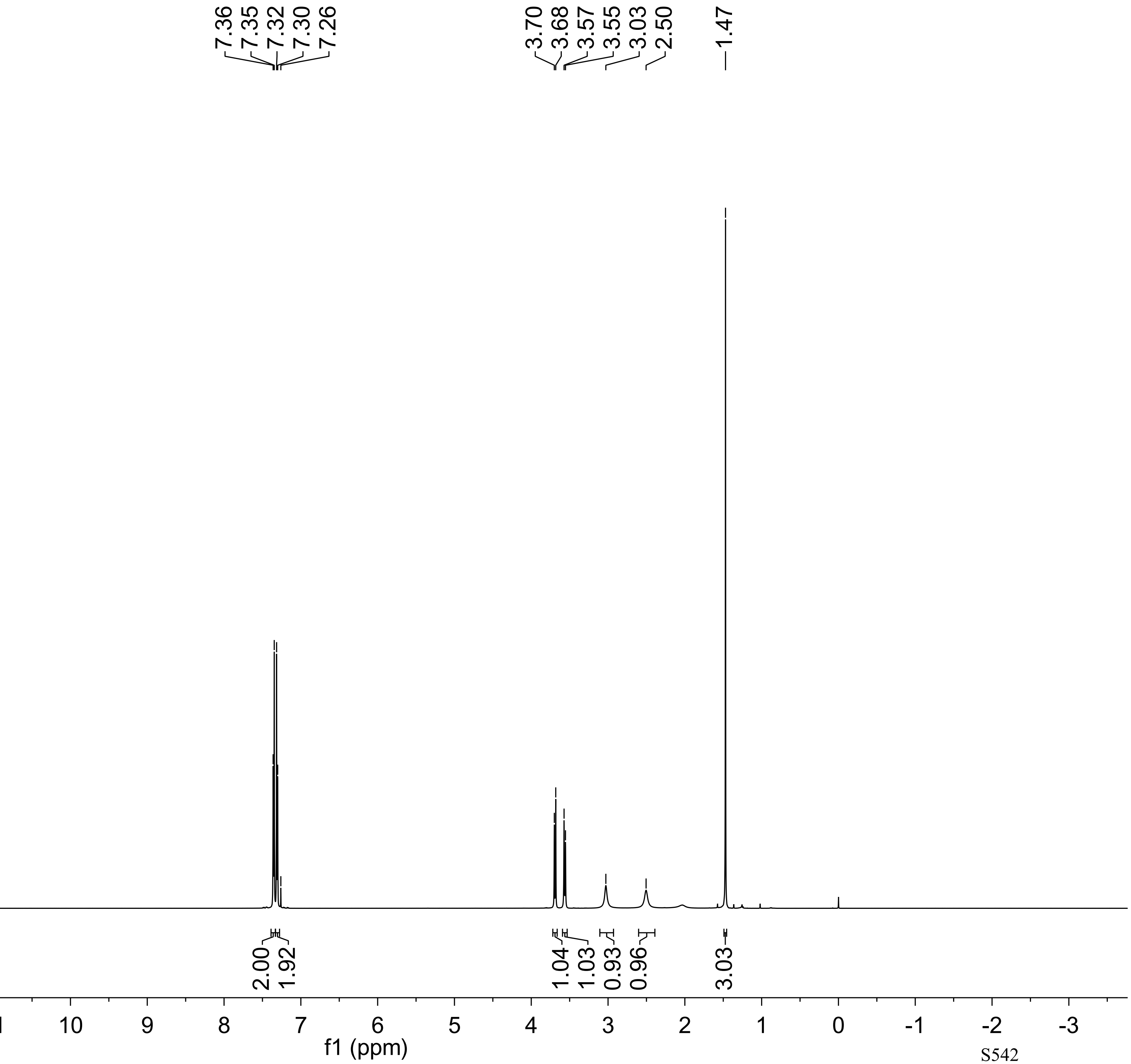

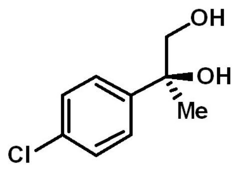

C67

Parameter	Value
1 Title	CFM-6-100-7-1006
2 Comment	
3 Origin	Bruker BioSpin GmbH
4 Owner	nmrsu
5 Site	
6 Spectrometer	Avance NEO 600
7 Author	
8 Solvent	CDCl3
9 Temperature	298.2
10 Pulse Sequence	zgpg30
11 Experiment	1D
12 Number of Scans	25
13 Receiver Gain	101
14 Spectrometer Frequency	150.91
15 Spectral Width	35714.3
16 Lowest Frequency	-2775.9
17 Nucleus	13C
18 Acquired Size	32768
19 Spectral Size	32768

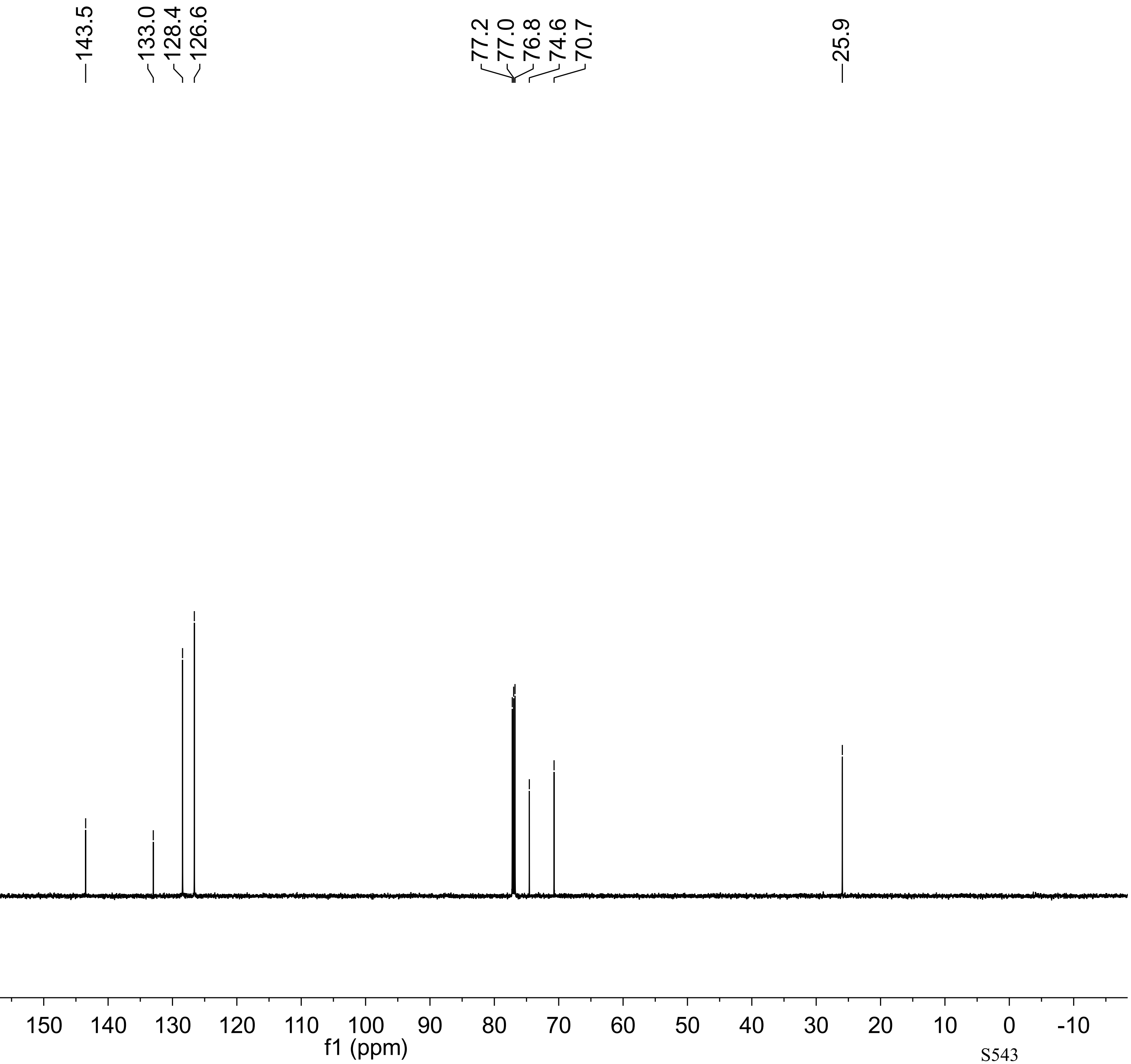

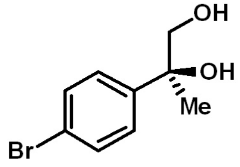

C68

	Parameter	Value
1	Title	CFM-6-100-8
2	Comment	
3	Origin	Bruker BioSpin GmbH
4	Owner	nmrsu
5	Site	
6	Spectrometer	AVANCE NEO 400 MHZ DIGITAL NMR SPECTROMETER
7	Author	
8	Solvent	CDCl3
9	Temperature	298.1
10	Pulse Sequence	zg30
11	Experiment	1D
12	Number of Scans	8
13	Receiver Gain	101
14	Spectrometer Frequency	400.13
15	Spectral Width	8196.7
16	Lowest Frequency	-1637.1
17	Nucleus	1H
18	Acquired Size	32768
19	Spectral Size	65536

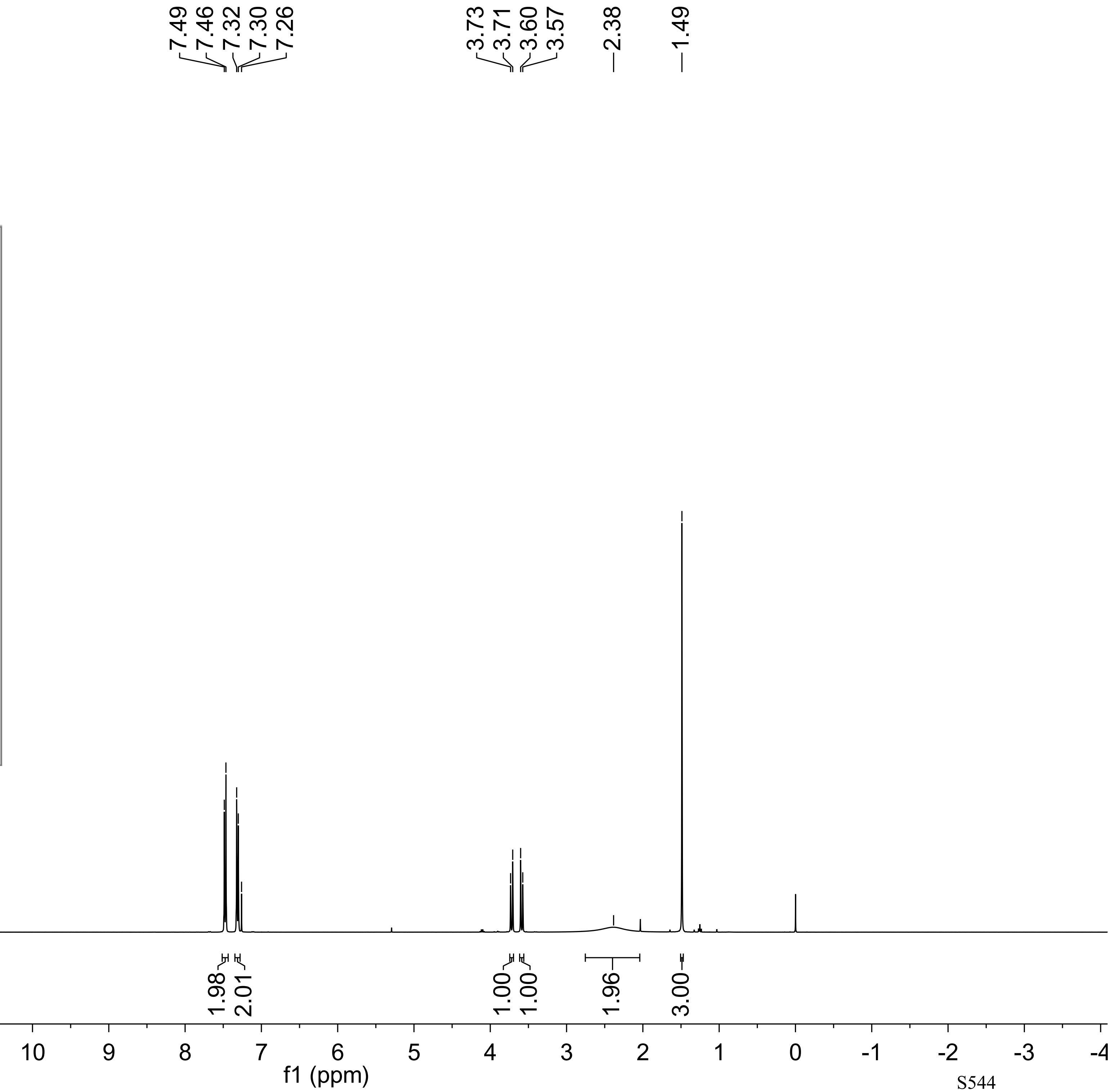

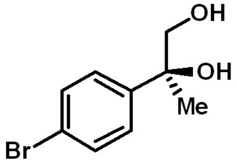

C68

	Parameter	Value
1	Title	CFM-6-100-8
2	Comment	
3	Origin	Bruker BioSpin GmbH
4	Owner	nmrsu
5	Site	
6	Spectrometer	AVANCE NEO 400 MHZ DIGITAL NMR SPECTROMETER
7	Author	
8	Solvent	CDCl3
9	Temperature	298.1
10	Pulse Sequence	zgpg30
11	Experiment	1D
12	Number of Scans	64
13	Receiver Gain	62
14	Spectrometer Frequency	100.61
15	Spectral Width	23809.5
16	Lowest Frequency	-1848.9
17	Nucleus	13C
18	Acquired Size	32768
19	Spectral Size	32768

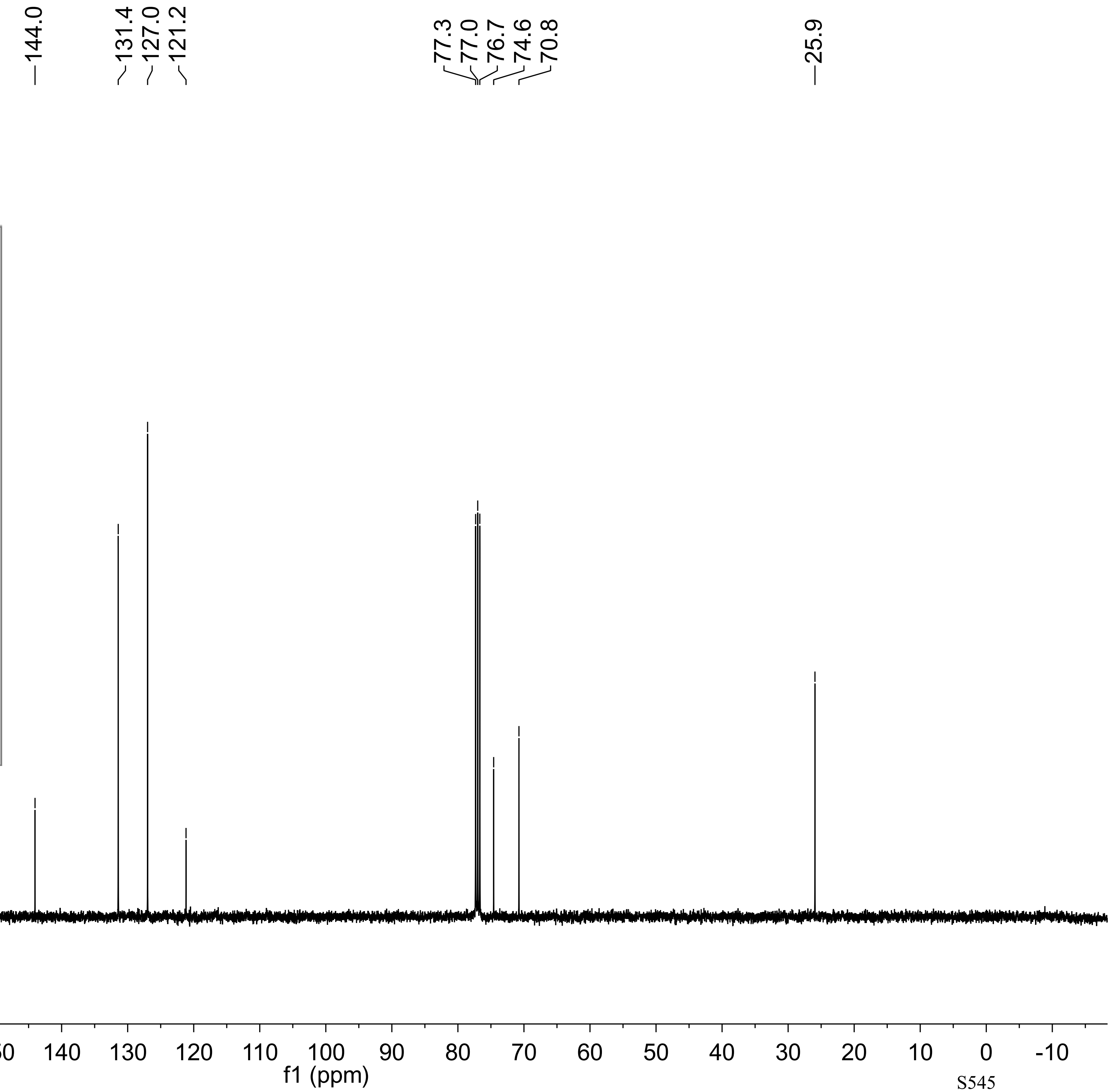

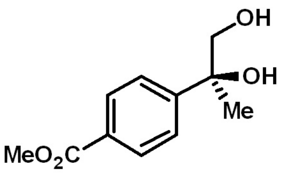

C69

Parameter	Value
1 Title	CFM-6-100-9
2 Comment	
3 Origin	Bruker BioSpin GmbH
4 Owner	nmrsu
5 Site	
6 Spectrometer	AVANCE NEO 400 MHZ DIGITAL NMR SPECTROMETER
7 Author	
8 Solvent	CDCl3
9 Temperature	298.1
10 Pulse Sequence	zg30
11 Experiment	1D
12 Number of Scans	8
13 Receiver Gain	101
14 Spectrometer Frequency	400.13
15 Spectral Width	8196.7
16 Lowest Frequency	-1637.0
17 Nucleus	1H
18 Acquired Size	32768
19 Spectral Size	65536

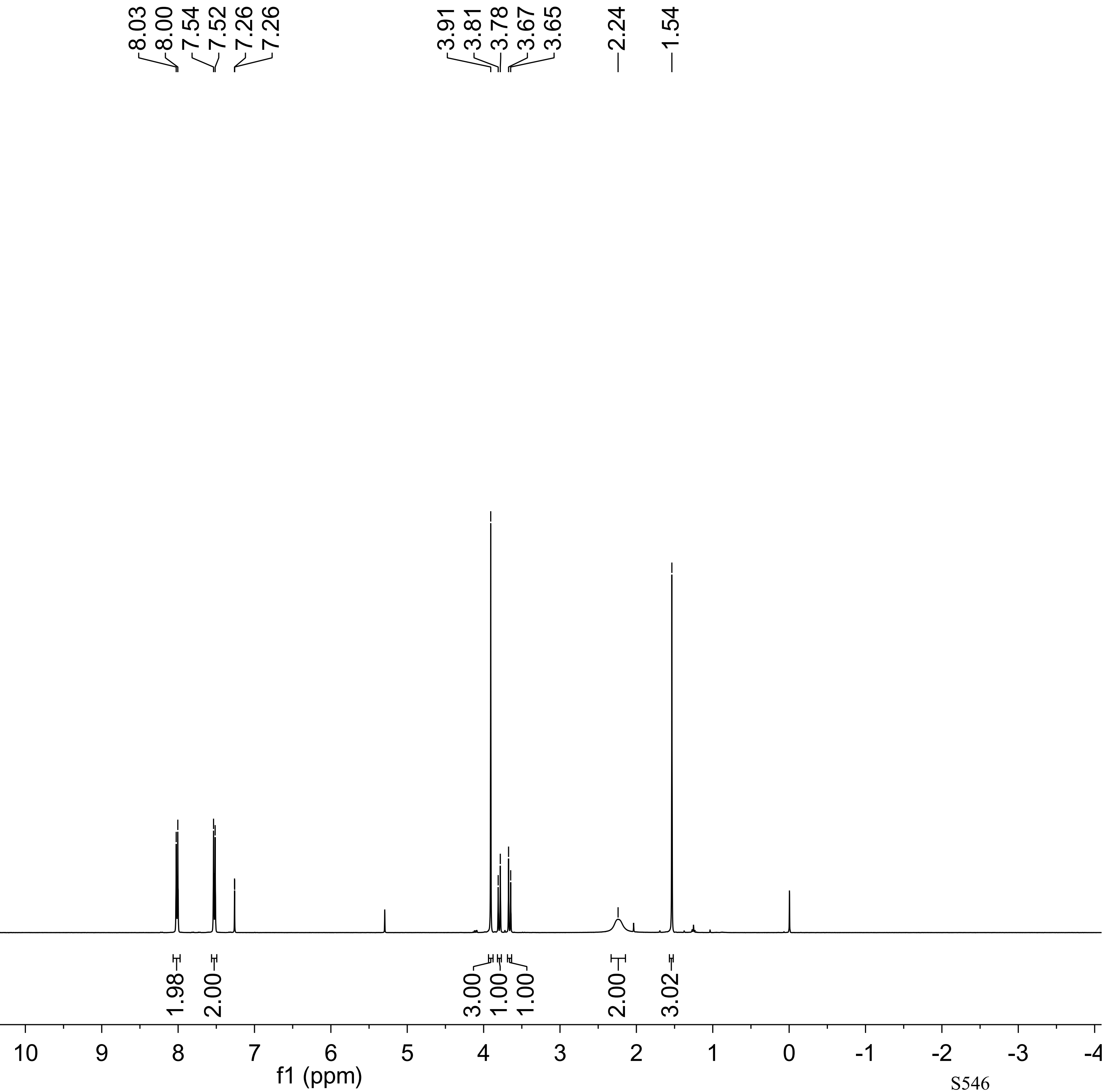

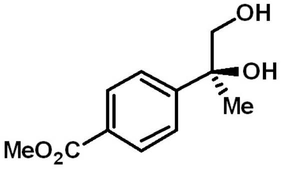

C69

	Parameter	Value
1	Title	CFM-6-100-9
2	Comment	
3	Origin	Bruker BioSpin GmbH
4	Owner	nmrsu
5	Site	
6	Spectrometer	AVANCE NEO 400 MHZ DIGITAL NMR SPECTROMETER
7	Author	
8	Solvent	CDCl3
9	Temperature	298.1
10	Pulse Sequence	zgpg30
11	Experiment	1D
12	Number of Scans	64
13	Receiver Gain	59
14	Spectrometer Frequency	100.61
15	Spectral Width	23809.5
16	Lowest Frequency	-1847.4
17	Nucleus	13C
18	Acquired Size	32768
19	Spectral Size	32768

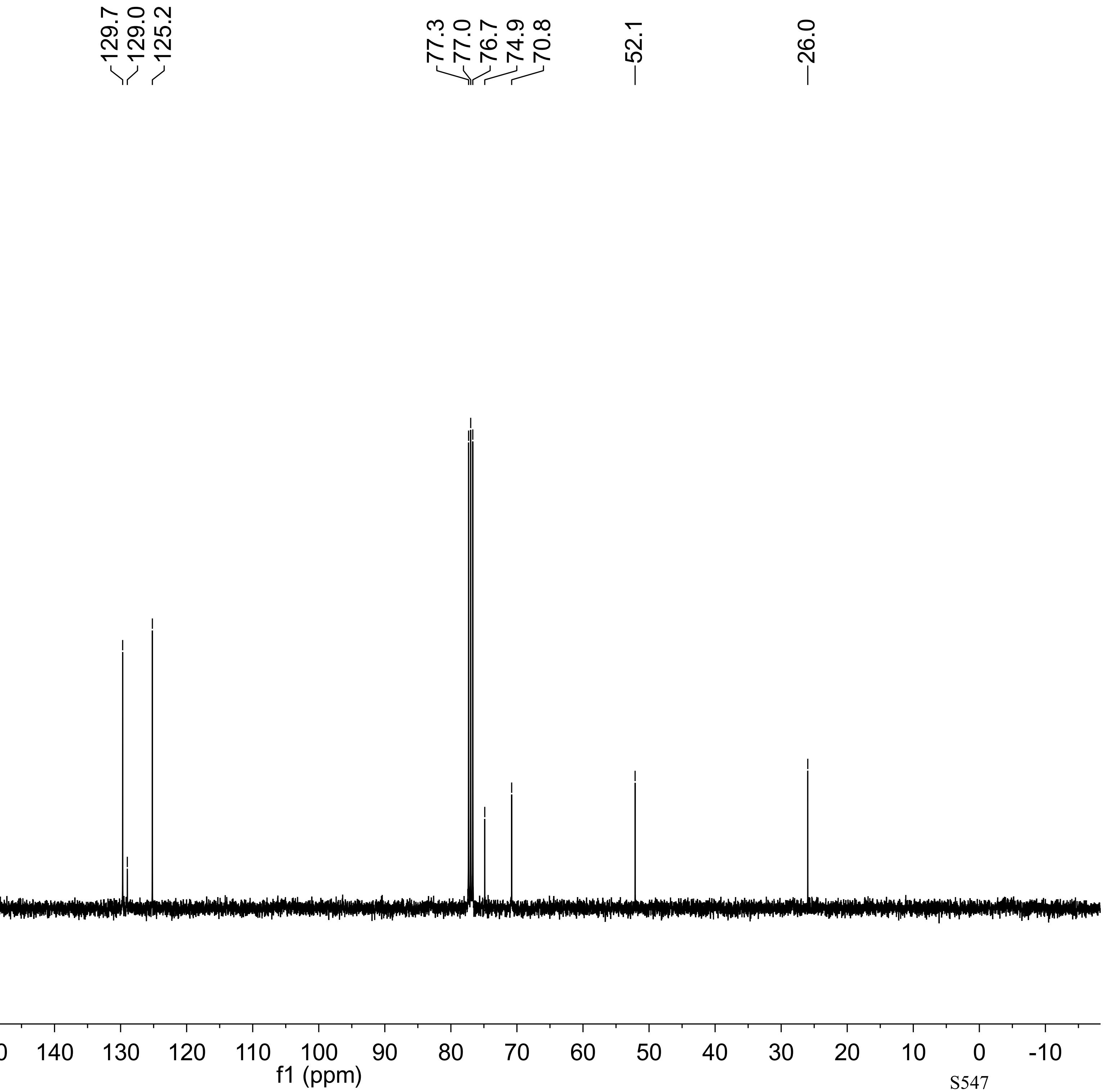

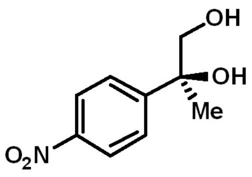

C70

	Parameter	Value
1	Title	CFM-6-100-11-1
2	Comment	
3	Origin	Bruker BioSpin GmbH
4	Owner	nmrsu
5	Site	
6	Spectrometer	AVANCE NEO 400 MHZ DIGITAL NMR SPECTROMETER
7	Author	
8	Solvent	CDCl3
9	Temperature	298.1
10	Pulse Sequence	zg30
11	Experiment	1D
12	Number of Scans	8
13	Receiver Gain	101
14	Spectrometer Frequency	400.13
15	Spectral Width	8196.7
16	Lowest Frequency	-1637.2
17	Nucleus	1H
18	Acquired Size	32768
19	Spectral Size	65536

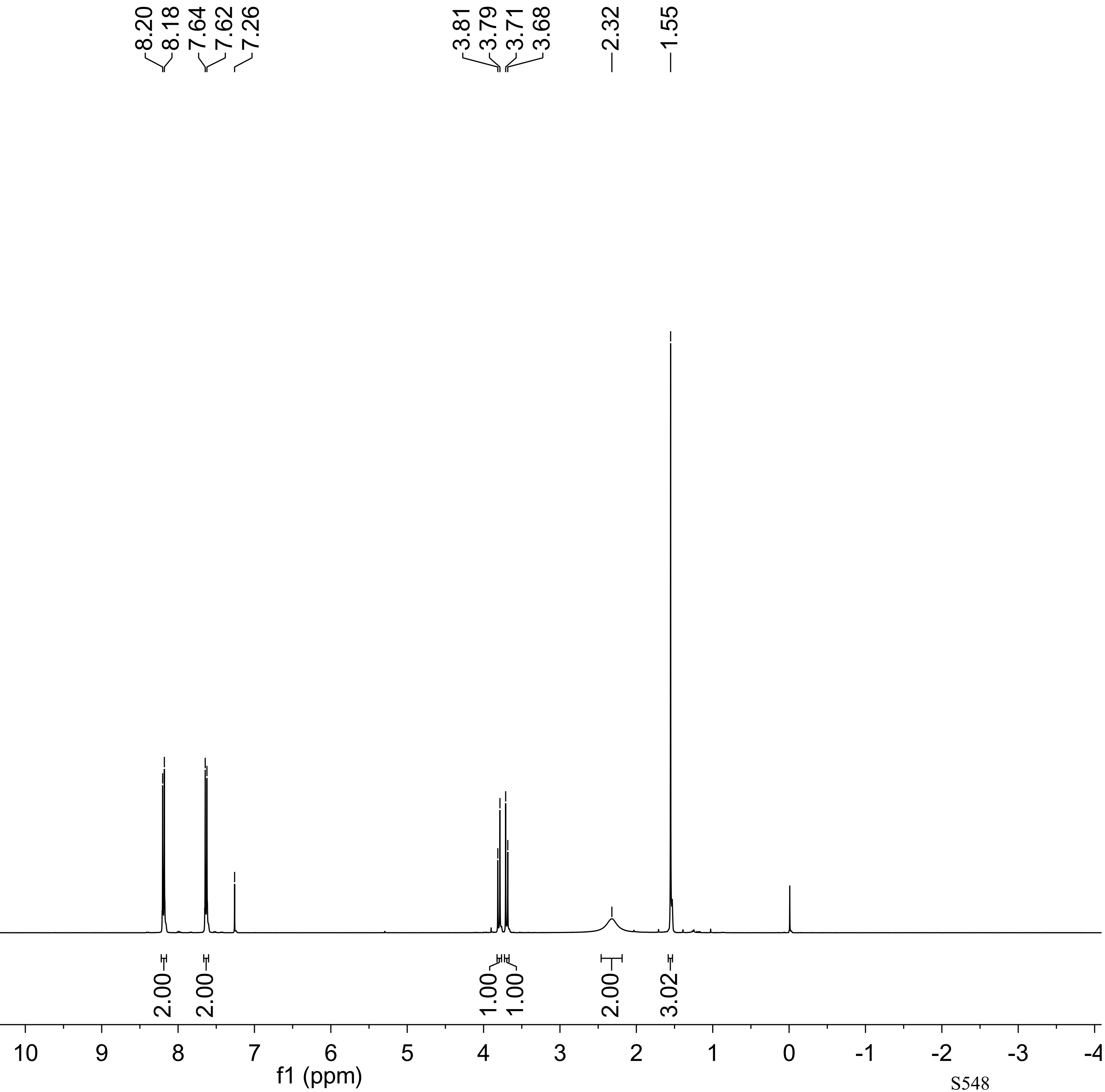

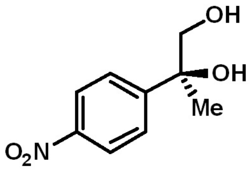

C70

	Parameter	Value
1	Title	CFM-6-100-11-1
2	Comment	
3	Origin	Bruker BioSpin GmbH
4	Owner	nmrsu
5	Site	
6	Spectrometer	AVANCE NEO 400 MHZ DIGITAL NMR SPECTROMETER
7	Author	
8	Solvent	CDCl3
9	Temperature	298.2
10	Pulse Sequence	zgpg30
11	Experiment	1D
12	Number of Scans	128
13	Receiver Gain	66
14	Spectrometer Frequency	100.61
15	Spectral Width	23809.5
16	Lowest Frequency	-1848.1
17	Nucleus	13C
18	Acquired Size	32768
19	Spectral Size	32768

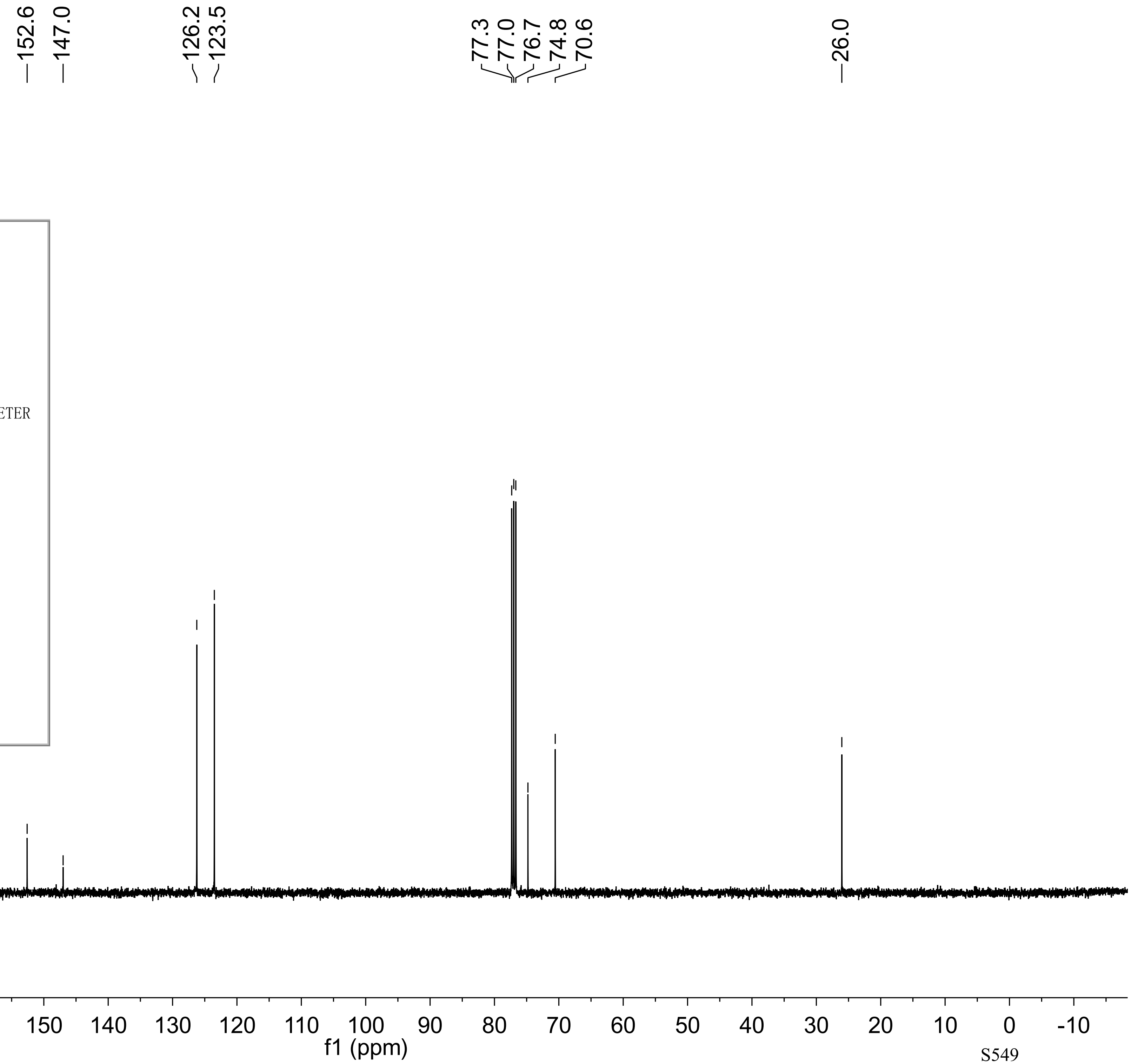

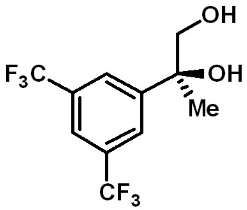

C71

	Parameter	Value
1	Title	CFM-6-100-12-1005
2	Comment	
3	Origin	Bruker BioSpin GmbH
4	Owner	nmrsu
5	Site	
6	Spectrometer	Avance NEO 600
7	Author	
8	Solvent	MeOD
9	Temperature	298.1
10	Pulse Sequence	zg30
11	Experiment	1D
12	Number of Scans	8
13	Receiver Gain	90
14	Spectrometer Frequency	600.15
15	Spectral Width	11904.8
16	Lowest Frequency	-2252.0
17	Nucleus	1H
18	Acquired Size	32768
19	Spectral Size	65536

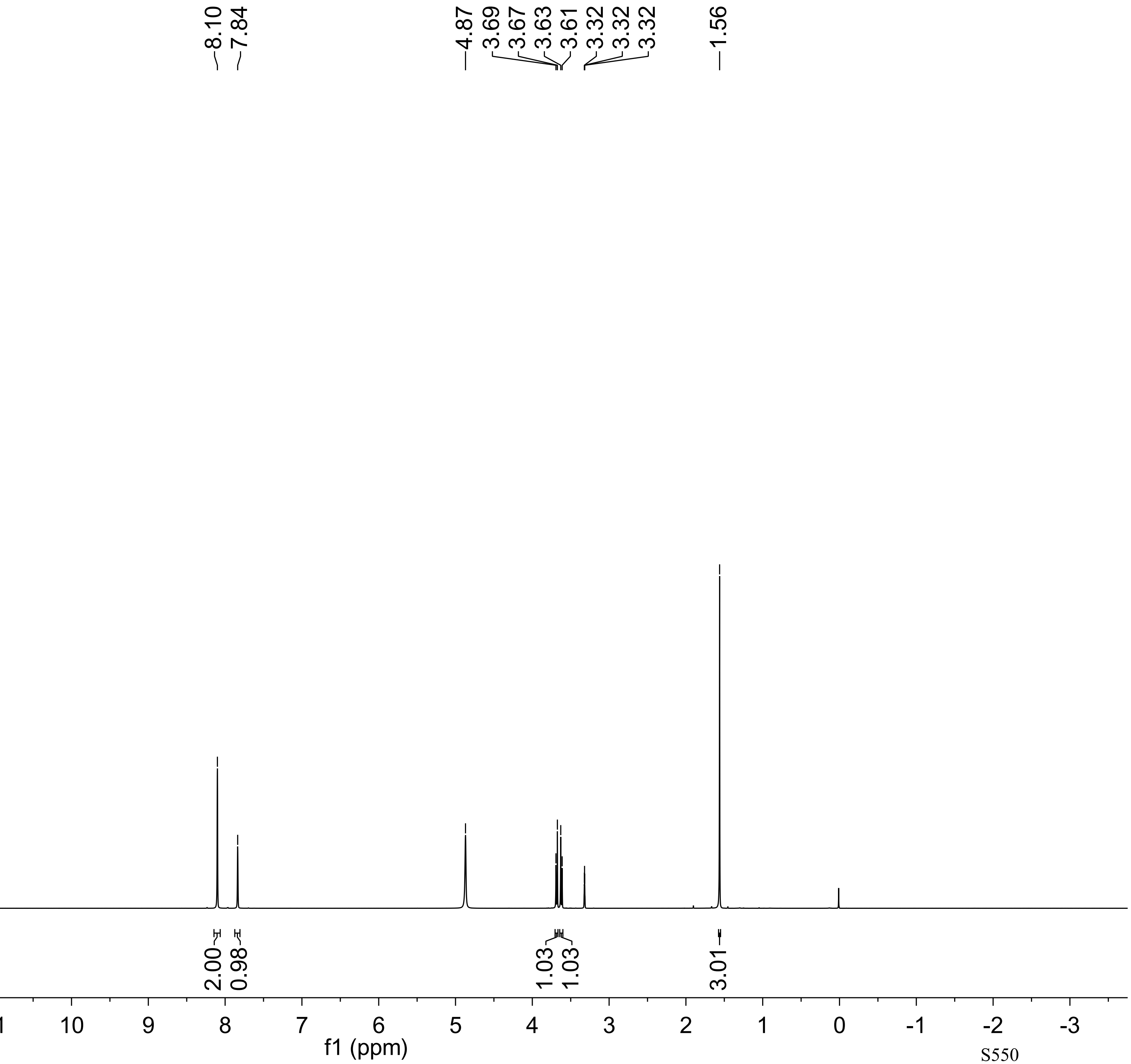

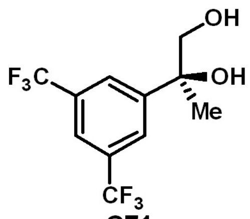

C71

Parameter	Value
1 Title	CFM-6-100-12-1005
2 Comment	
3 Origin	Bruker BioSpin GmbH
4 Owner	nmrsu
5 Site	
6 Spectrometer	Avance NEO 600
7 Author	
8 Solvent	MeOD
9 Temperature	298.2
10 Pulse Sequence	zgpg30
11 Experiment	1D
12 Number of Scans	155
13 Receiver Gain	101
14 Spectrometer Frequency	150.91
15 Spectral Width	35714.3
16 Lowest Frequency	-2554.3
17 Nucleus	13C
18 Acquired Size	32768
19 Spectral Size	32768

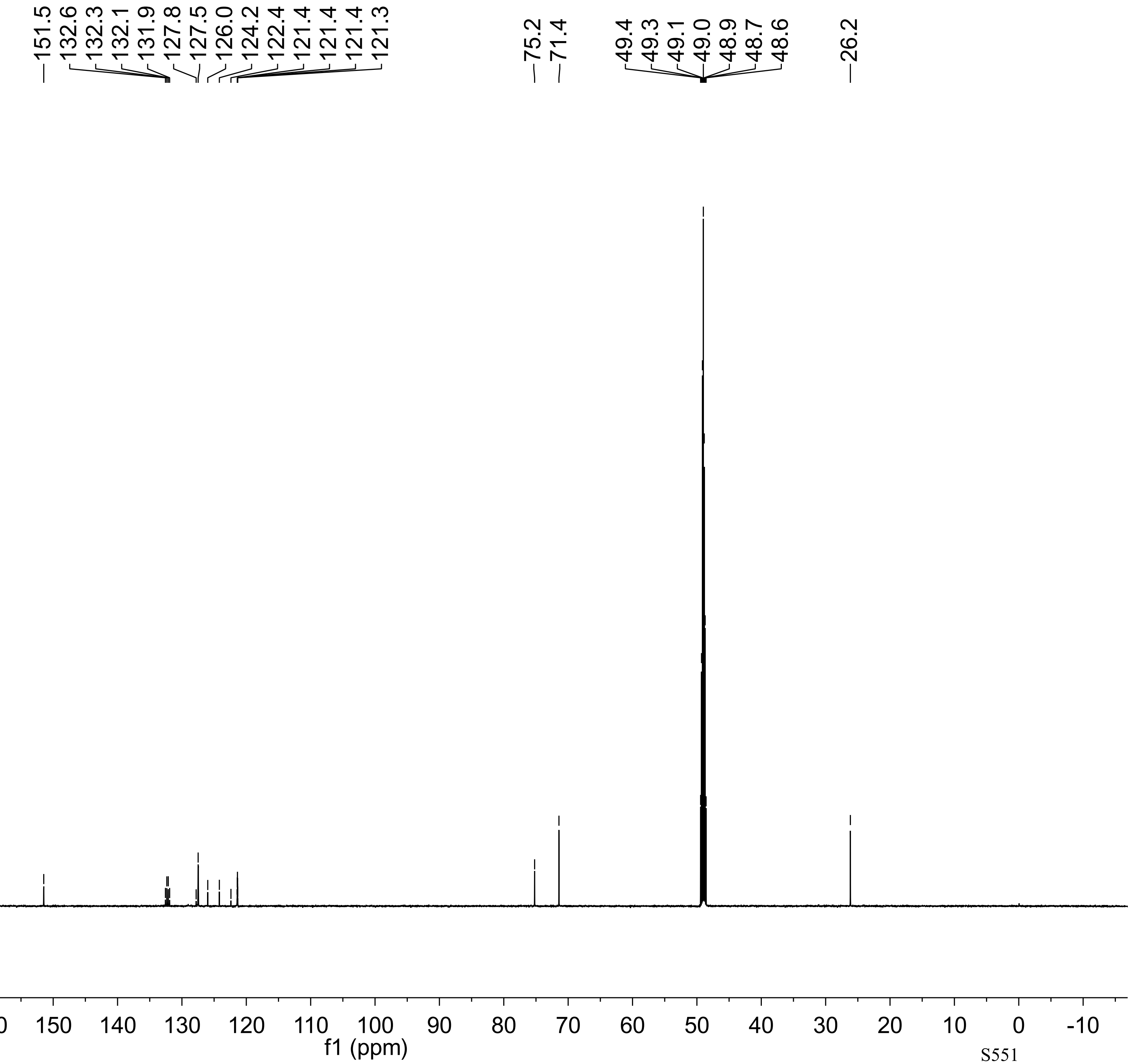

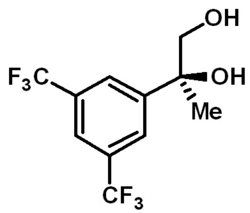

C71

Parameter	Value
1 Title	CFM-6-100-12-1005
2 Comment	
3 Origin	Bruker BioSpin GmbH
4 Owner	nmrsu
5 Site	
6 Spectrometer	Avance NEO 600
7 Author	
8 Solvent	MeOD
9 Temperature	298.1
10 Pulse Sequence	zg
11 Experiment	1D
12 Number of Scans	16
13 Receiver Gain	101
14 Spectrometer Frequency	564.71
15 Spectral Width	131579.0
16 Lowest Frequency	-122260.0
17 Nucleus	19F
18 Acquired Size	65536
19 Spectral Size	65536

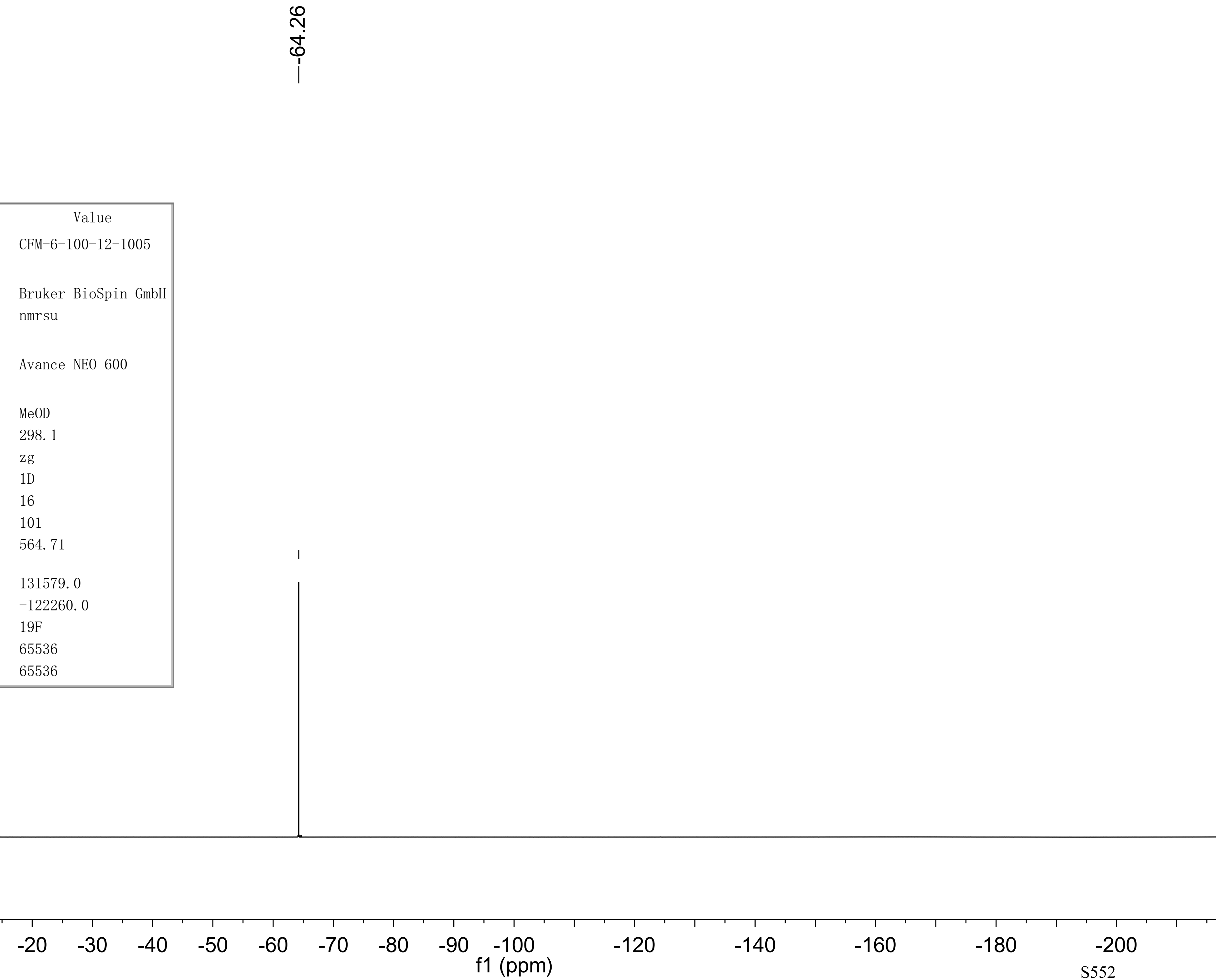

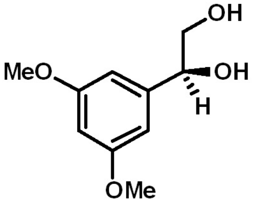

C72

Parameter	Value
1 Title	CFM-6-B1-2
2 Comment	
3 Origin	Bruker BioSpin GmbH
4 Owner	nmrsu
5 Site	
6 Spectrometer	AVANCE NEO 400 MHZ DIGITAL NMR SPECTROMETER
7 Author	
8 Solvent	CDCl3
9 Temperature	298.2
10 Pulse Sequence	zg30
11 Experiment	1D
12 Number of Scans	8
13 Receiver Gain	101
14 Spectrometer Frequency	400.13
15 Spectral Width	8196.7
16 Lowest Frequency	-1637.1
17 Nucleus	1H
18 Acquired Size	32768
19 Spectral Size	65536

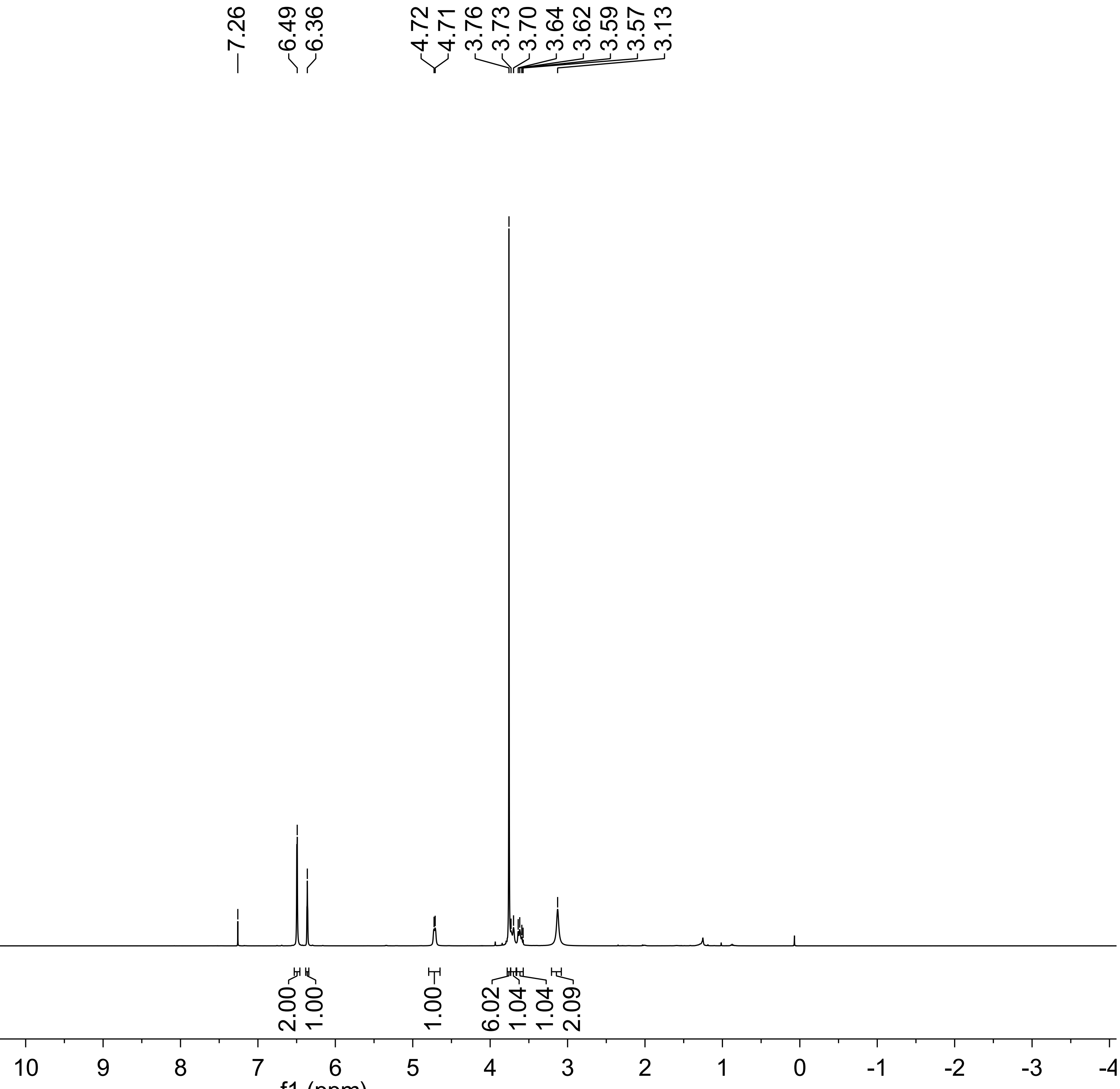

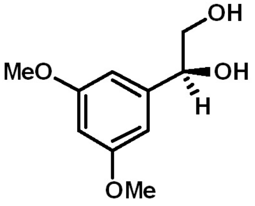

C72

Parameter	Value
1 Title	CFM-6-B1
2 Comment	
3 Origin	Bruker BioSpin GmbH
4 Owner	nmrsu
5 Site	
6 Spectrometer	Avance Neo 400M
7 Author	
8 Solvent	CDCl3
9 Temperature	298.3
10 Pulse Sequence	zgpg30
11 Experiment	1D
12 Number of Scans	73
13 Receiver Gain	33
14 Spectrometer Frequency	100.63
15 Spectral Width	23809.5
16 Lowest Frequency	-1849.6
17 Nucleus	13C
18 Acquired Size	32768
19 Spectral Size	32768

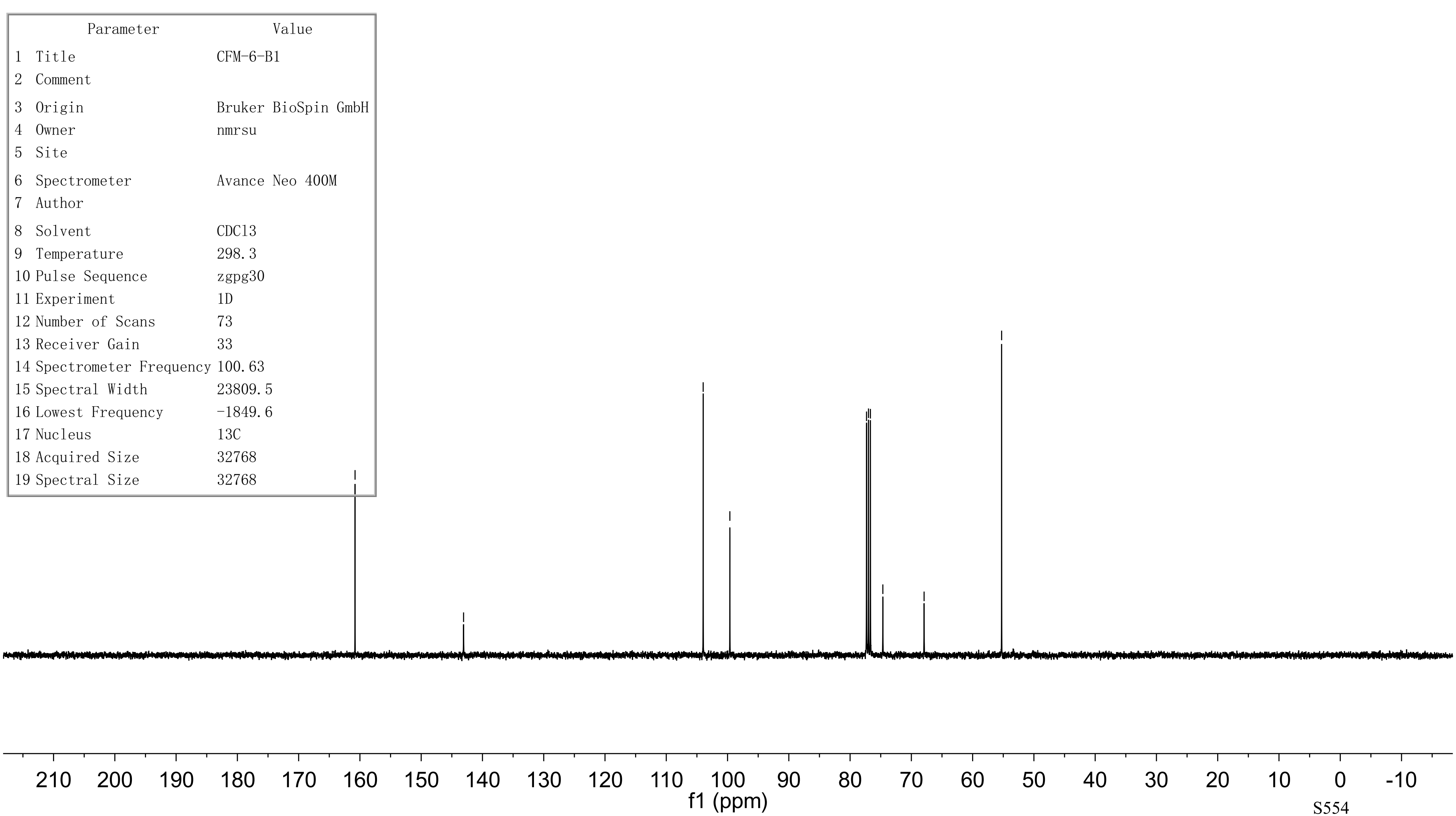

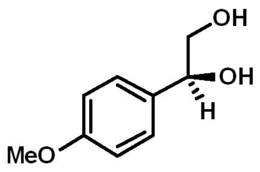

C73

	Parameter	Value
1	Title	CFM-6-102-2
2	Comment	
3	Origin	Bruker BioSpin GmbH
4	Owner	nmrsu
5	Site	
6	Spectrometer	AVANCE NEO 400 MHZ DIGITAL NMR SPECTROMETER
7	Author	
8	Solvent	CDCl3
9	Temperature	298.2
10	Pulse Sequence	zg30
11	Experiment	1D
12	Number of Scans	8
13	Receiver Gain	101
14	Spectrometer Frequency	400.13
15	Spectral Width	8196.7
16	Lowest Frequency	-1637.2
17	Nucleus	1H
18	Acquired Size	32768
19	Spectral Size	65536

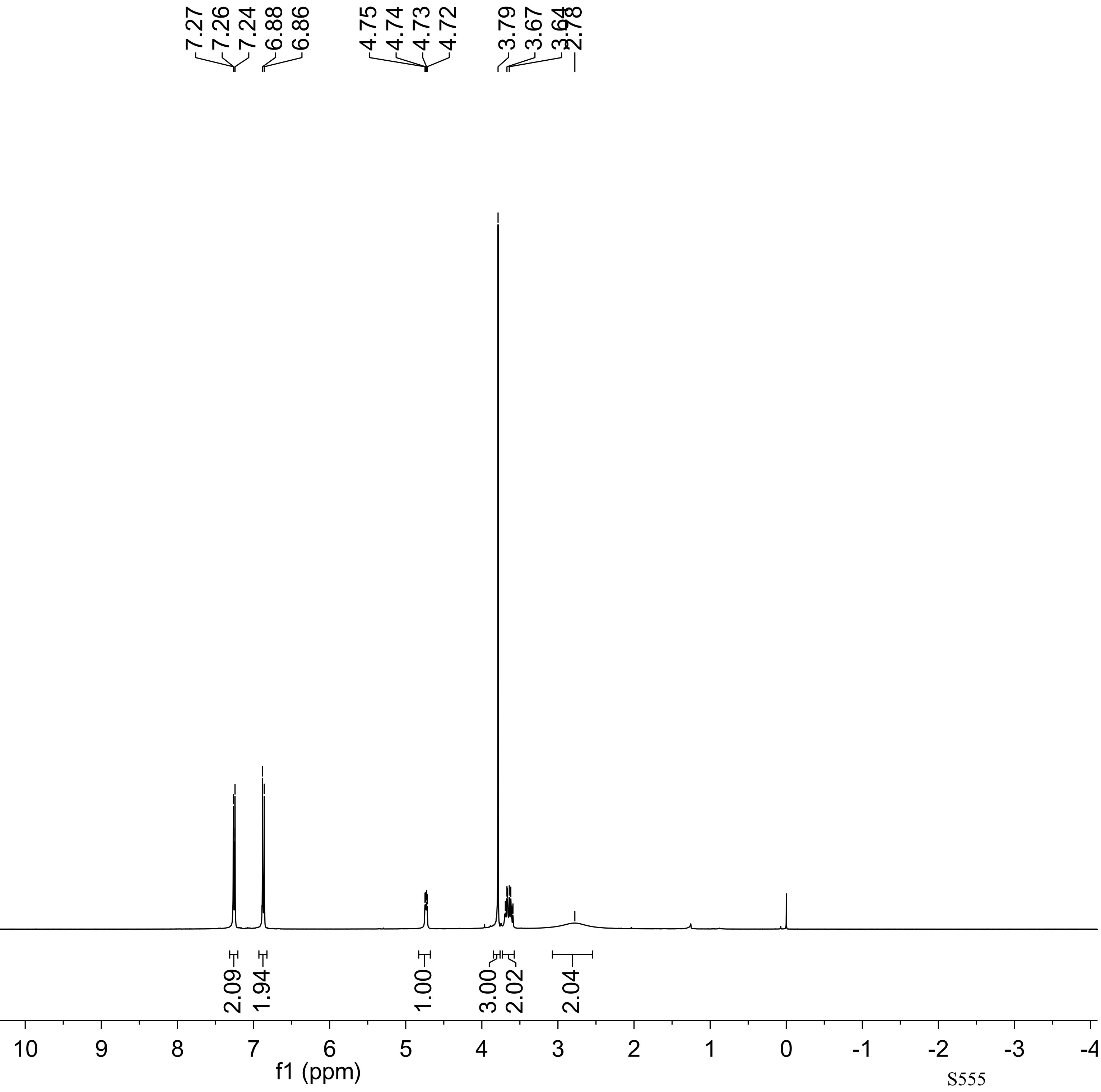

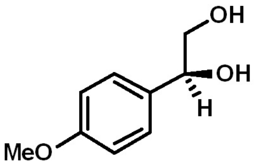

C73

Parameter	Value
1 Title	CFM-6-102-2
2 Comment	
3 Origin	Bruker BioSpin GmbH
4 Owner	nmrsu
5 Site	
6 Spectrometer	AVANCE NEO 400 MHZ DIGITAL NMR SPECTROMETER
7 Author	
8 Solvent	CDCl3
9 Temperature	298.1
10 Pulse Sequence	zgpg30
11 Experiment	1D
12 Number of Scans	128
13 Receiver Gain	62
14 Spectrometer Frequency	100.61
15 Spectral Width	23809.5
16 Lowest Frequency	-1849.3
17 Nucleus	13C
18 Acquired Size	32768
19 Spectral Size	32768

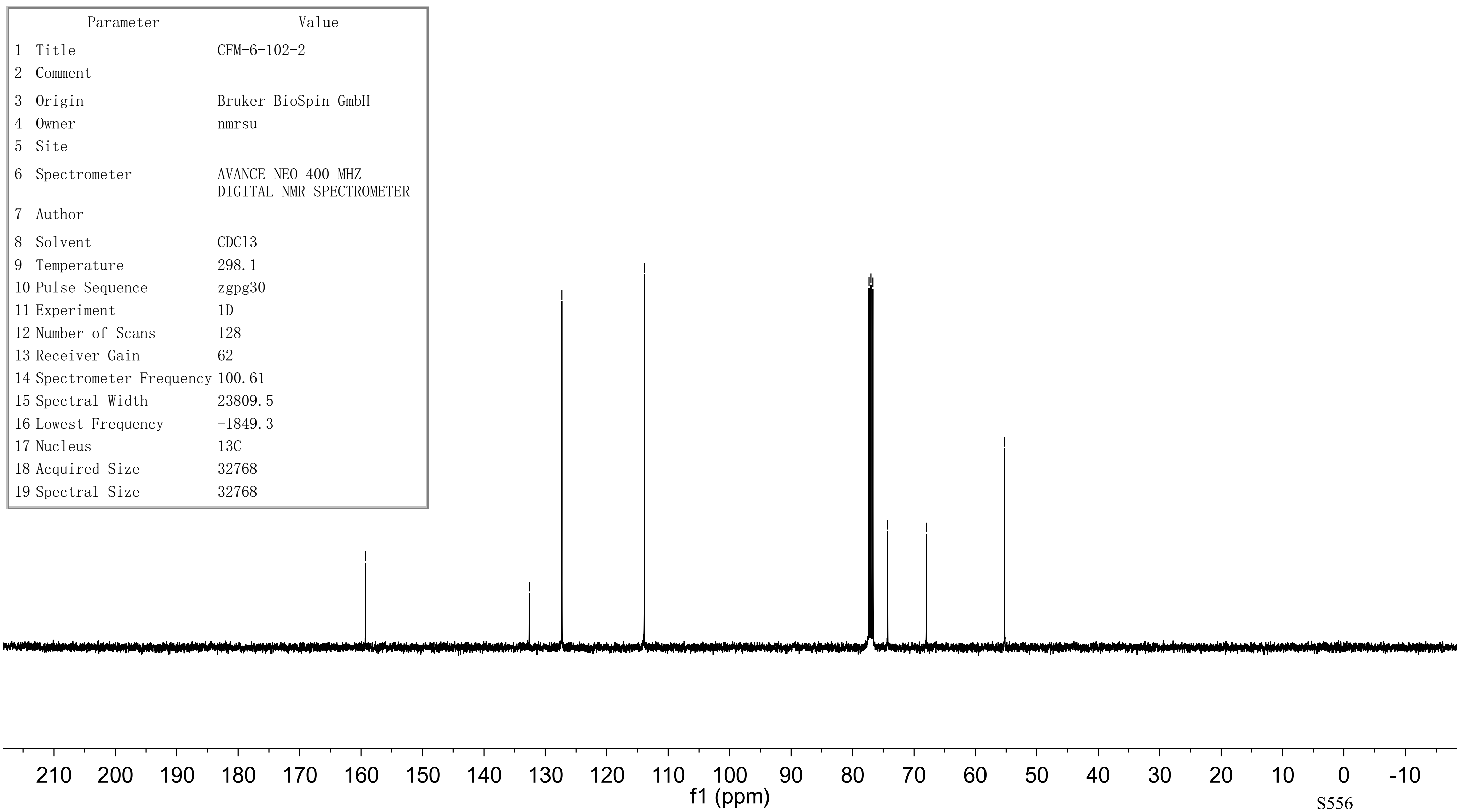

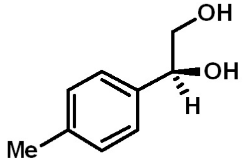

C74

Parameter	Value
1 Title	CFM-6-102-3
2 Comment	
3 Origin	Bruker BioSpin GmbH
4 Owner	nmrsu
5 Site	
6 Spectrometer	AVANCE NEO 400 MHZ DIGITAL NMR SPECTROMETER
7 Author	
8 Solvent	CDCl3
9 Temperature	298.1
10 Pulse Sequence	zg30
11 Experiment	1D
12 Number of Scans	8
13 Receiver Gain	101
14 Spectrometer Frequency	400.13
15 Spectral Width	8196.7
16 Lowest Frequency	-1637.1
17 Nucleus	1H
18 Acquired Size	32768
19 Spectral Size	65536

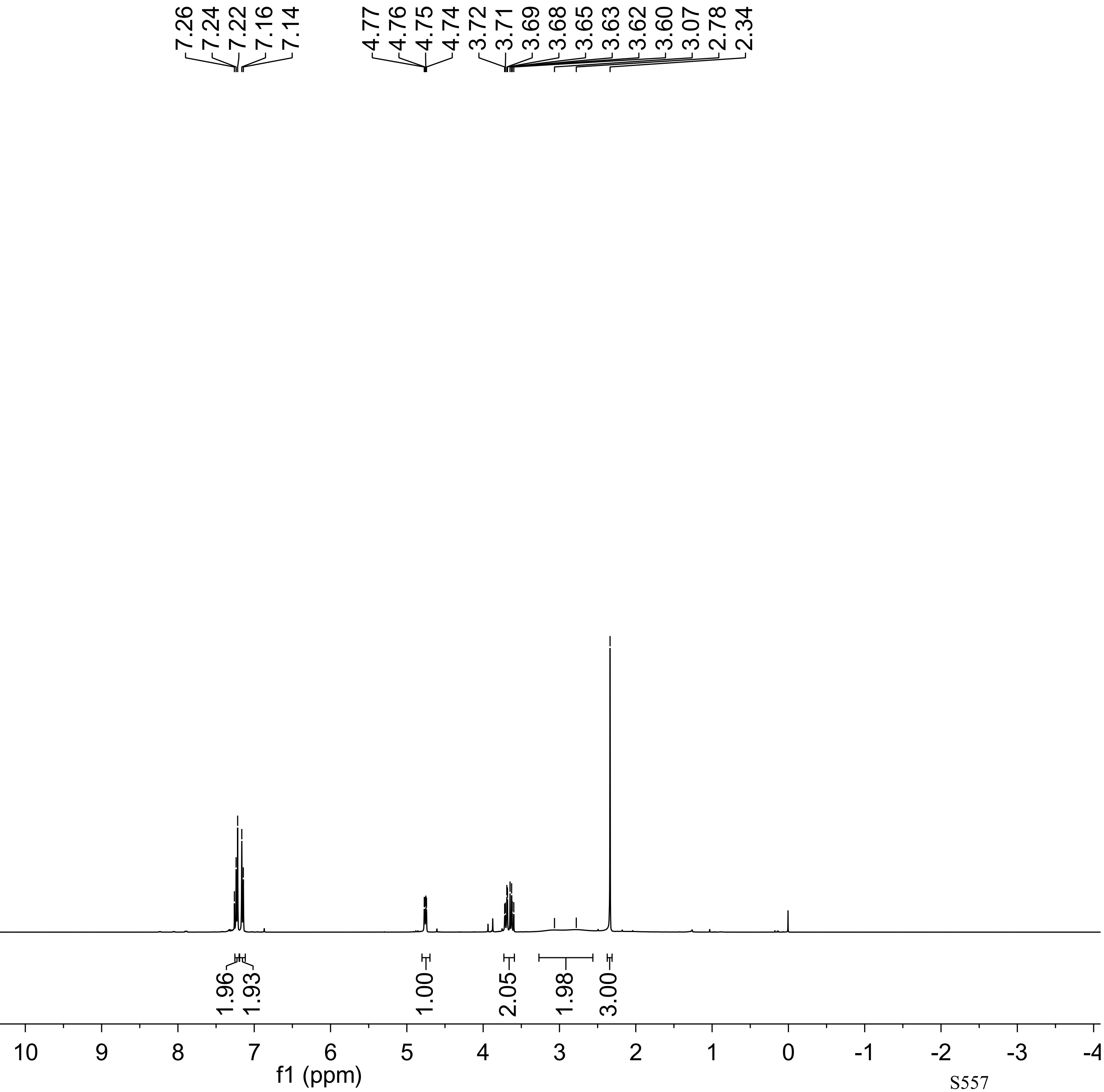

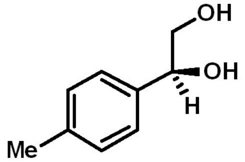

C74

Parameter	Value
1 Title	CFM-6-102-3
2 Comment	
3 Origin	Bruker BioSpin GmbH
4 Owner	nmrsu
5 Site	
6 Spectrometer	AVANCE NEO 400 MHZ DIGITAL NMR SPECTROMETER
7 Author	
8 Solvent	CDCl3
9 Temperature	298.2
10 Pulse Sequence	zgpg30
11 Experiment	1D
12 Number of Scans	128
13 Receiver Gain	62
14 Spectrometer Frequency	100.61
15 Spectral Width	23809.5
16 Lowest Frequency	-1849.2
17 Nucleus	13C
18 Acquired Size	32768
19 Spectral Size	32768

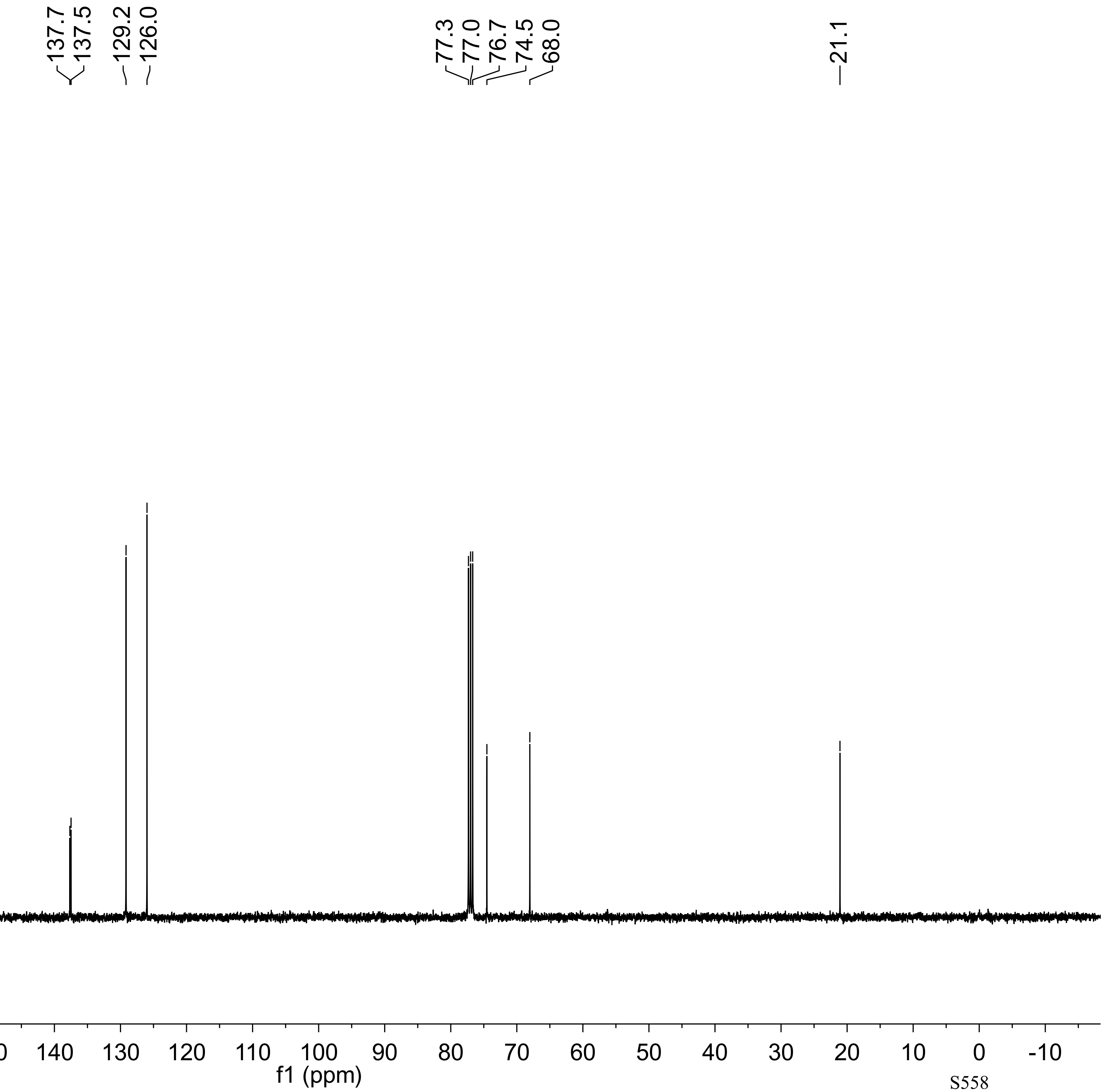

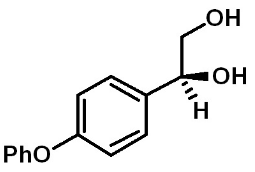

C75

	Parameter	Value
1	Title	CFM-6-102-4
2	Comment	
3	Origin	Bruker BioSpin GmbH
4	Owner	nmrsu
5	Site	
6	Spectrometer	AVANCE NEO 400 MHZ DIGITAL NMR SPECTROMETER
7	Author	
8	Solvent	CDCl3
9	Temperature	298.1
10	Pulse Sequence	zg30
11	Experiment	1D
12	Number of Scans	8
13	Receiver Gain	101
14	Spectrometer Frequency	400.13
15	Spectral Width	8196.7
16	Lowest Frequency	-1637.3
17	Nucleus	1H
18	Acquired Size	32768
19	Spectral Size	65536

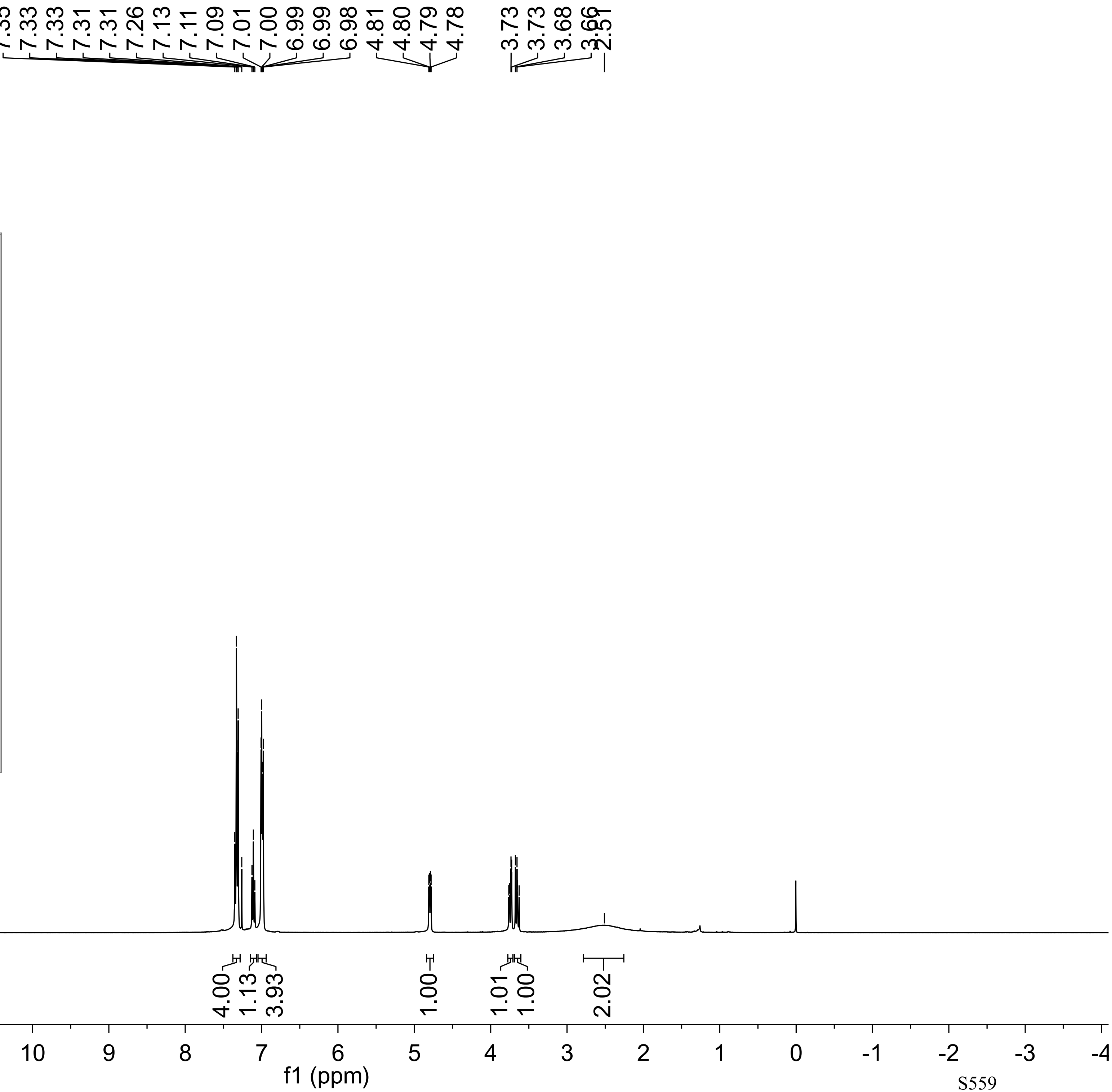

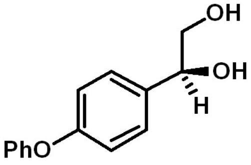

C75

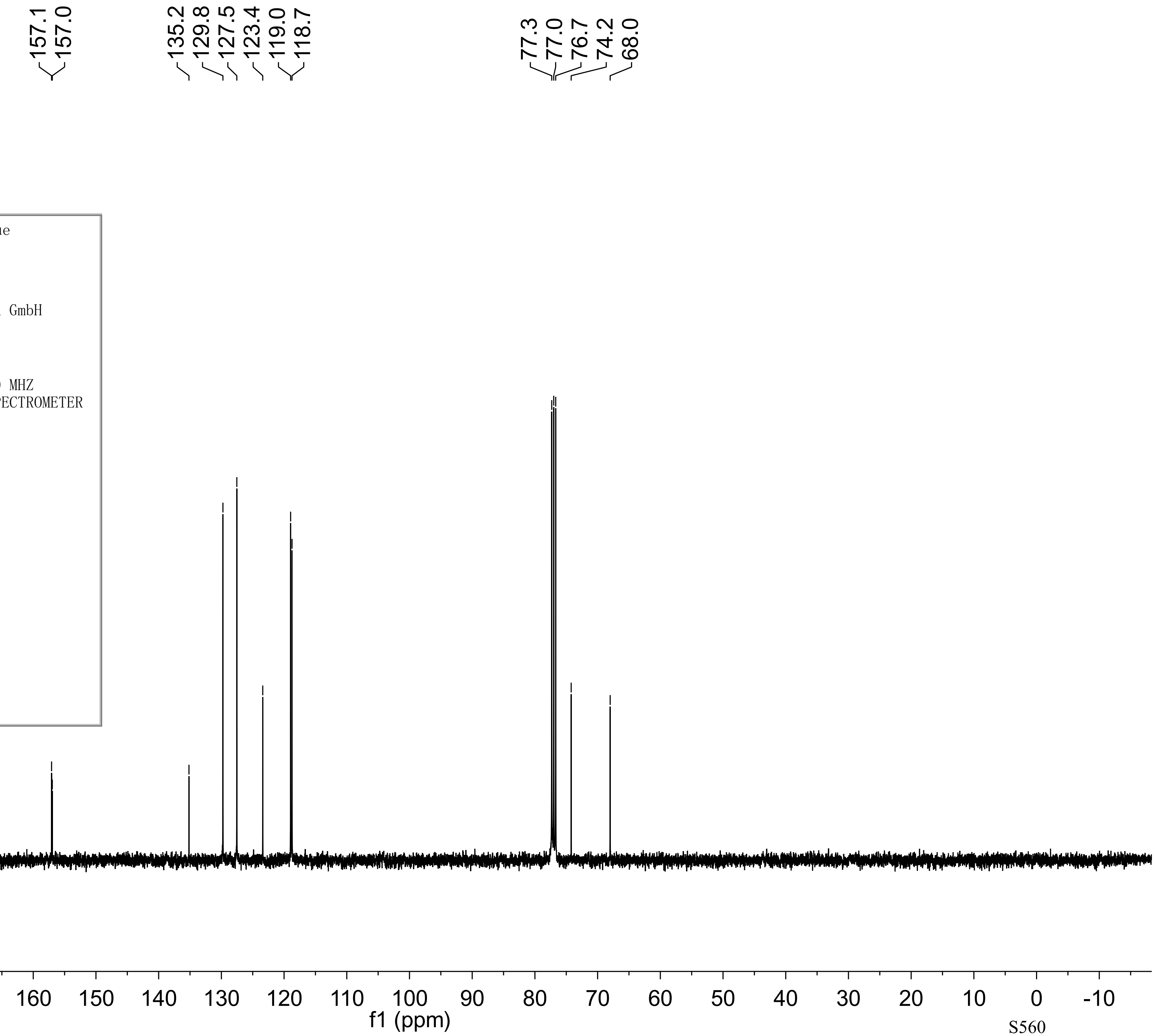

Parameter	Value
1 Title	CFM-6-102-4
2 Comment	
3 Origin	Bruker BioSpin GmbH
4 Owner	nmrsu
5 Site	
6 Spectrometer	AVANCE NEO 400 MHZ DIGITAL NMR SPECTROMETER
7 Author	
8 Solvent	CDCl3
9 Temperature	298.2
10 Pulse Sequence	zgpg30
11 Experiment	1D
12 Number of Scans	96
13 Receiver Gain	62
14 Spectrometer Frequency	100.61
15 Spectral Width	23809.5
16 Lowest Frequency	-1848.9
17 Nucleus	13C
18 Acquired Size	32768
19 Spectral Size	32768

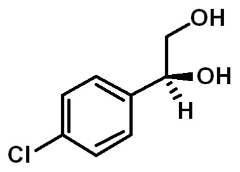

C76

Parameter	Value
1 Title	CFM-6-100-7
2 Comment	
3 Origin	Bruker BioSpin GmbH
4 Owner	nmrsu
5 Site	
6 Spectrometer	AVANCE NEO 400 MHZ DIGITAL NMR SPECTROMETER
7 Author	
8 Solvent	CDCl3
9 Temperature	298.2
10 Pulse Sequence	zg30
11 Experiment	1D
12 Number of Scans	5
13 Receiver Gain	101
14 Spectrometer Frequency	400.13
15 Spectral Width	8196.7
16 Lowest Frequency	-1637.2
17 Nucleus	1H
18 Acquired Size	32768
19 Spectral Size	65536

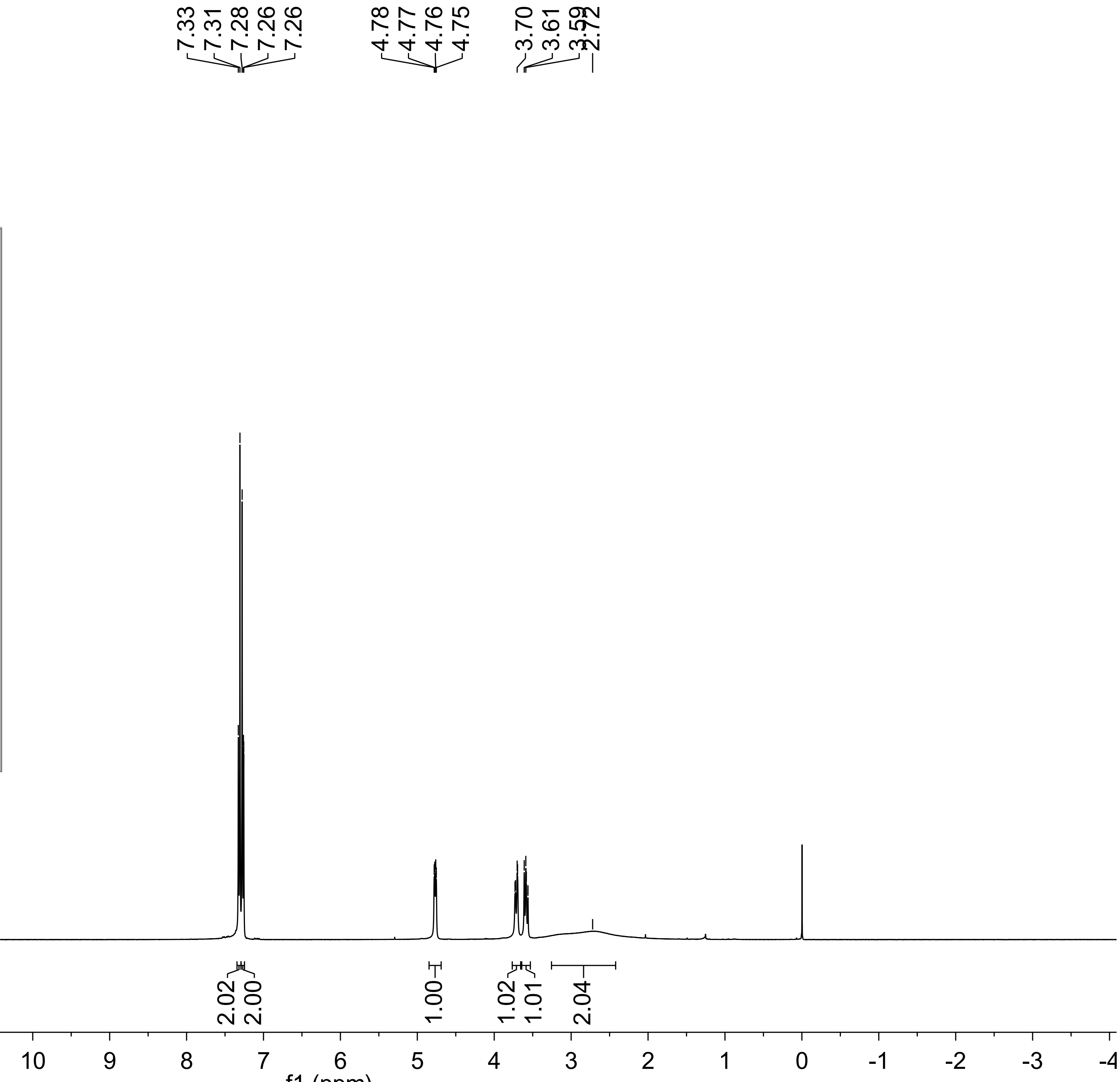

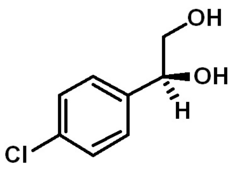

C76

Parameter	Value
1 Title	CFM-6-100-7
2 Comment	
3 Origin	Bruker BioSpin GmbH
4 Owner	nmrsu
5 Site	
6 Spectrometer	AVANCE NEO 400 MHZ DIGITAL NMR SPECTROMETER
7 Author	
8 Solvent	CDCl3
9 Temperature	298.1
10 Pulse Sequence	zgpg30
11 Experiment	1D
12 Number of Scans	64
13 Receiver Gain	62
14 Spectrometer Frequency	100.61
15 Spectral Width	23809.5
16 Lowest Frequency	-1848.3
17 Nucleus	13C
18 Acquired Size	32768
19 Spectral Size	32768

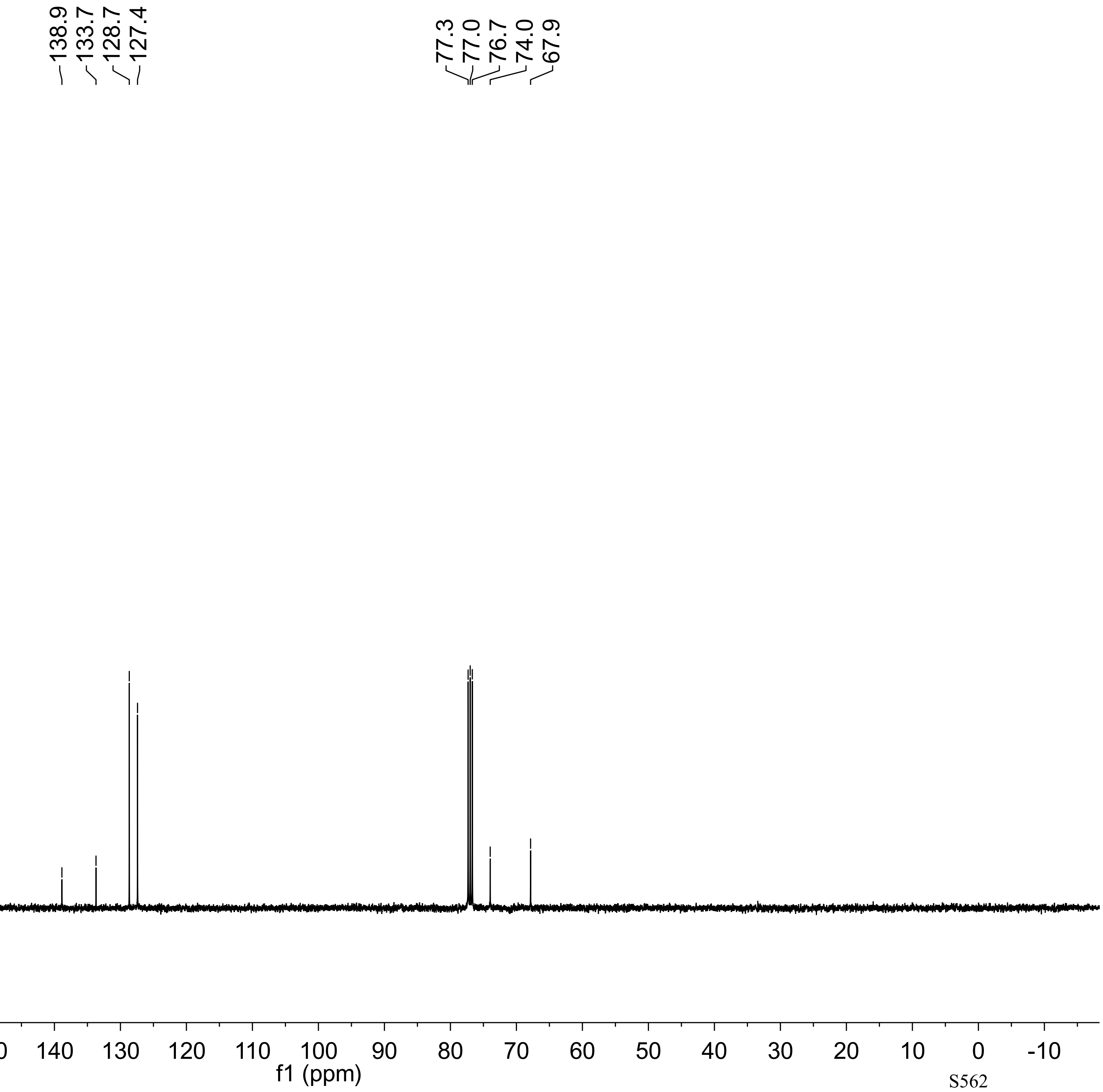

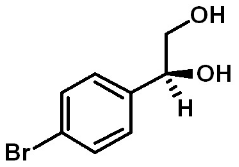

C77

	Parameter	Value
1	Title	CFM-6-102-8
2	Comment	
3	Origin	Bruker BioSpin GmbH
4	Owner	nmrsu
5	Site	
6	Spectrometer	AVANCE NEO 400 MHZ DIGITAL NMR SPECTROMETER
7	Author	
8	Solvent	CDCl3
9	Temperature	298.1
10	Pulse Sequence	zg30
11	Experiment	1D
12	Number of Scans	8
13	Receiver Gain	101
14	Spectrometer Frequency	400.13
15	Spectral Width	8196.7
16	Lowest Frequency	-1637.2
17	Nucleus	1H
18	Acquired Size	32768
19	Spectral Size	65536

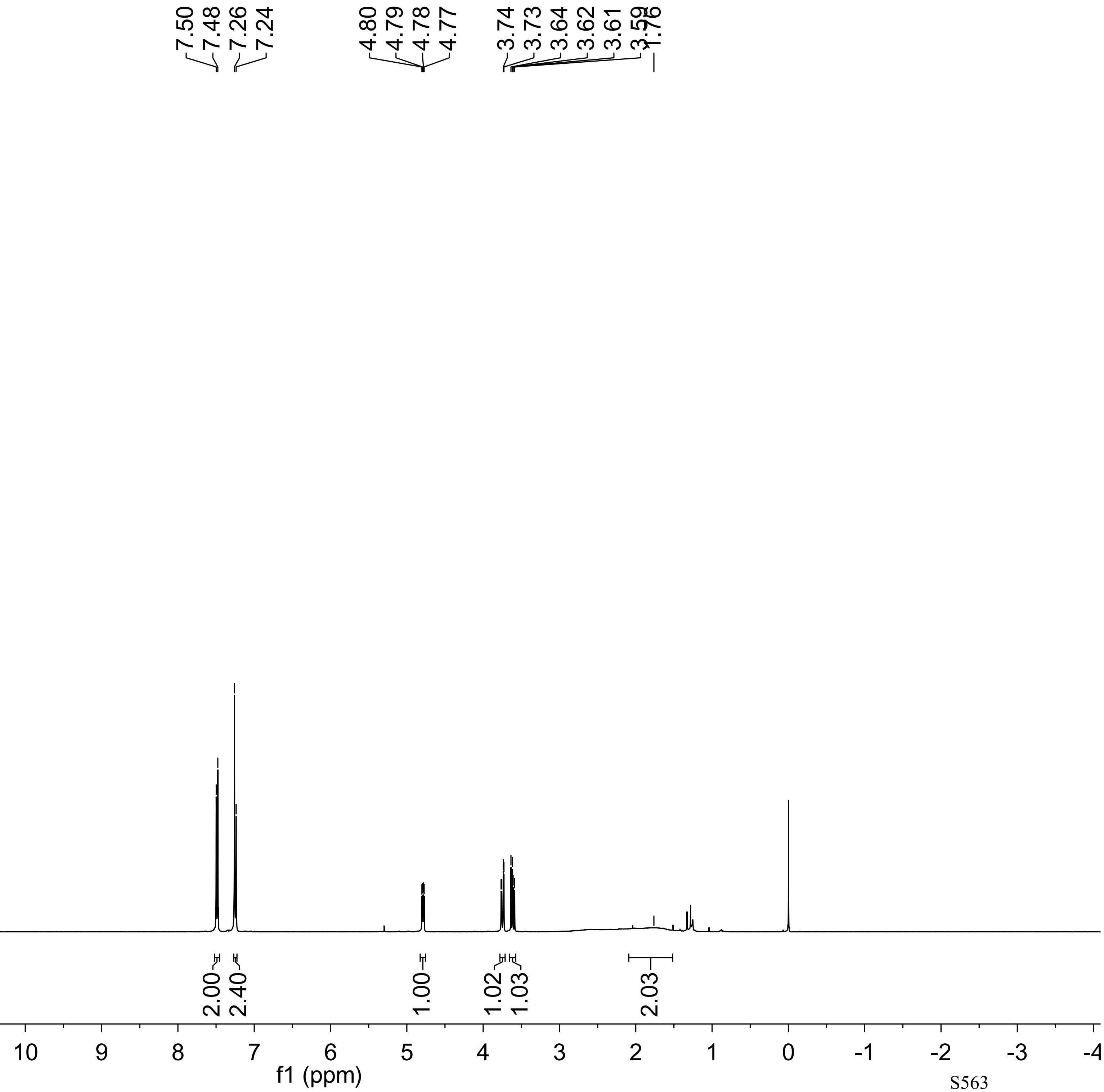

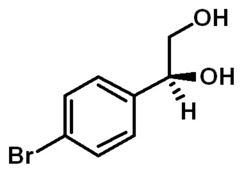

C77

Parameter	Value
1 Title	CFM-6-102-8
2 Comment	
3 Origin	Bruker BioSpin GmbH
4 Owner	nmrsu
5 Site	
6 Spectrometer	AVANCE NEO 400 MHZ DIGITAL NMR SPECTROMETER
7 Author	
8 Solvent	CDCl3
9 Temperature	298.2
10 Pulse Sequence	zgpg30
11 Experiment	1D
12 Number of Scans	128
13 Receiver Gain	62
14 Spectrometer Frequency	100.61
15 Spectral Width	23809.5
16 Lowest Frequency	-1846.6
17 Nucleus	13C
18 Acquired Size	32768
19 Spectral Size	32768

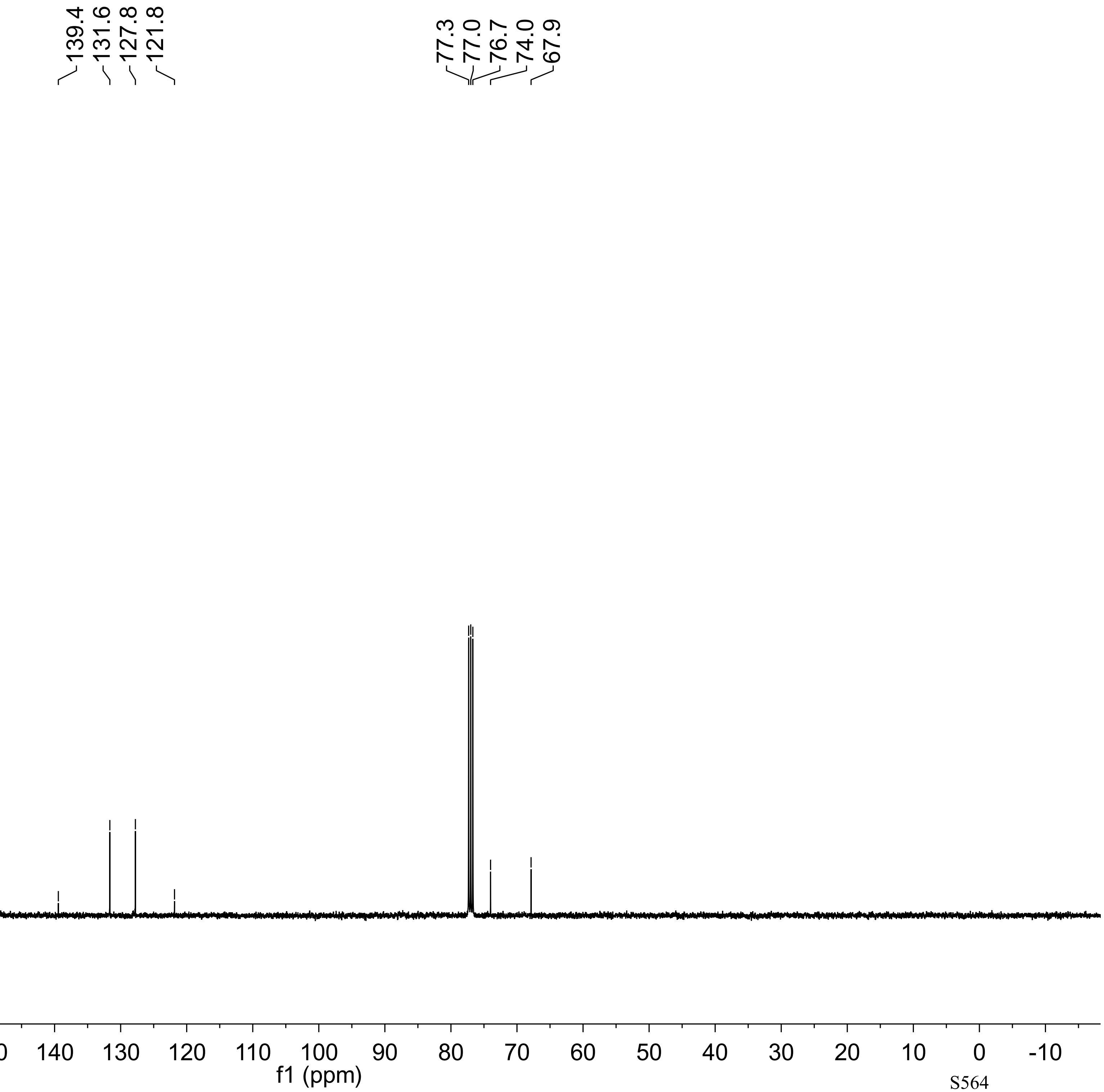

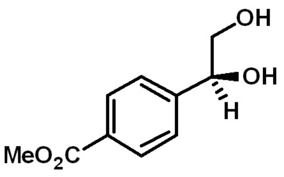

C78

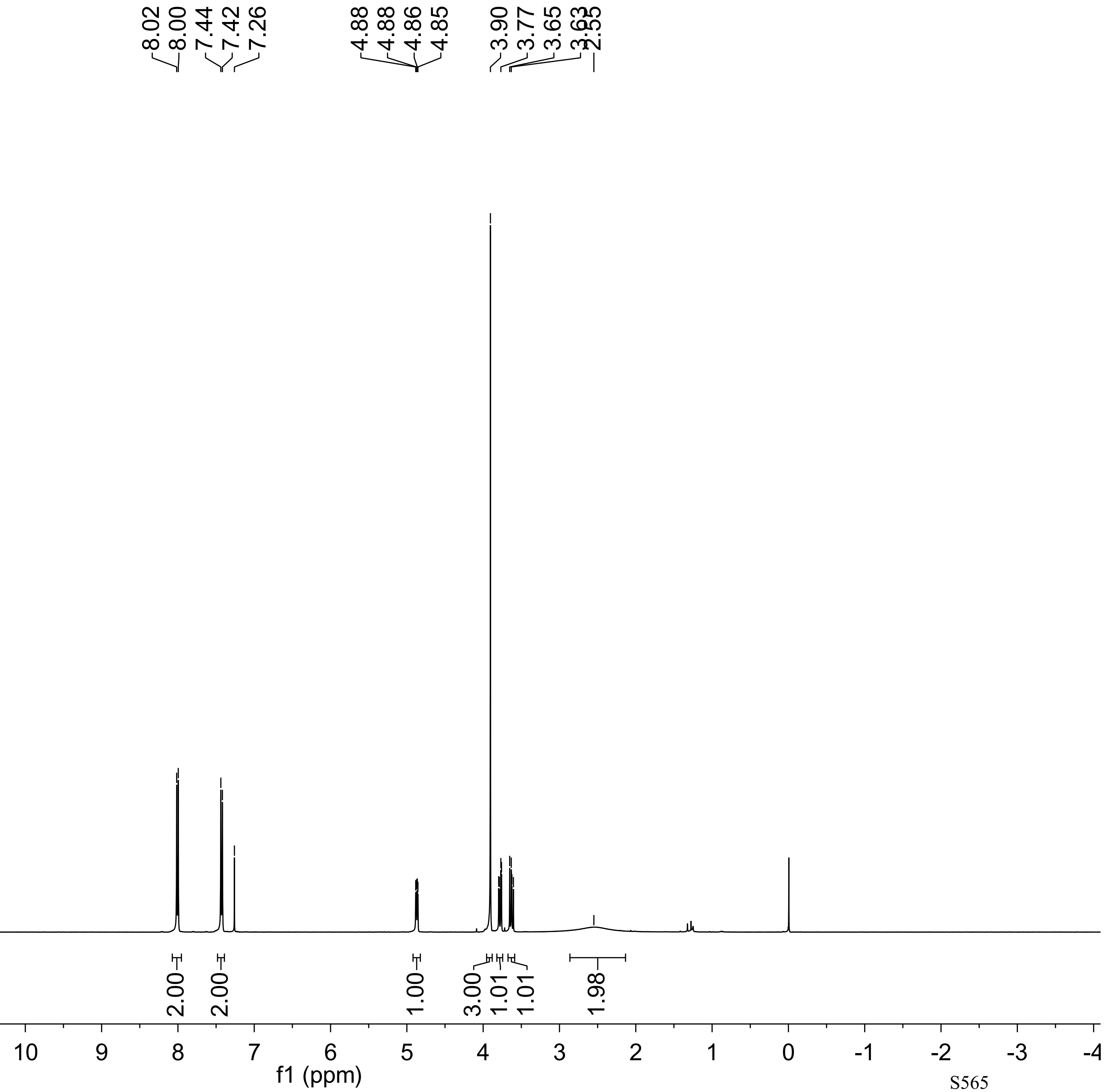

Parameter	Value
1 Title	CFM-6-102-9
2 Comment	
3 Origin	Bruker BioSpin GmbH
4 Owner	nmrsu
5 Site	
6 Spectrometer	AVANCE NEO 400 MHZ DIGITAL NMR SPECTROMETER
7 Author	
8 Solvent	CDCl3
9 Temperature	298.1
10 Pulse Sequence	zg30
11 Experiment	1D
12 Number of Scans	8
13 Receiver Gain	101
14 Spectrometer Frequency	400.13
15 Spectral Width	8196.7
16 Lowest Frequency	-1637.1
17 Nucleus	1H
18 Acquired Size	32768
19 Spectral Size	65536

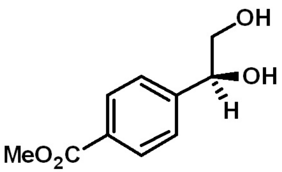

C78

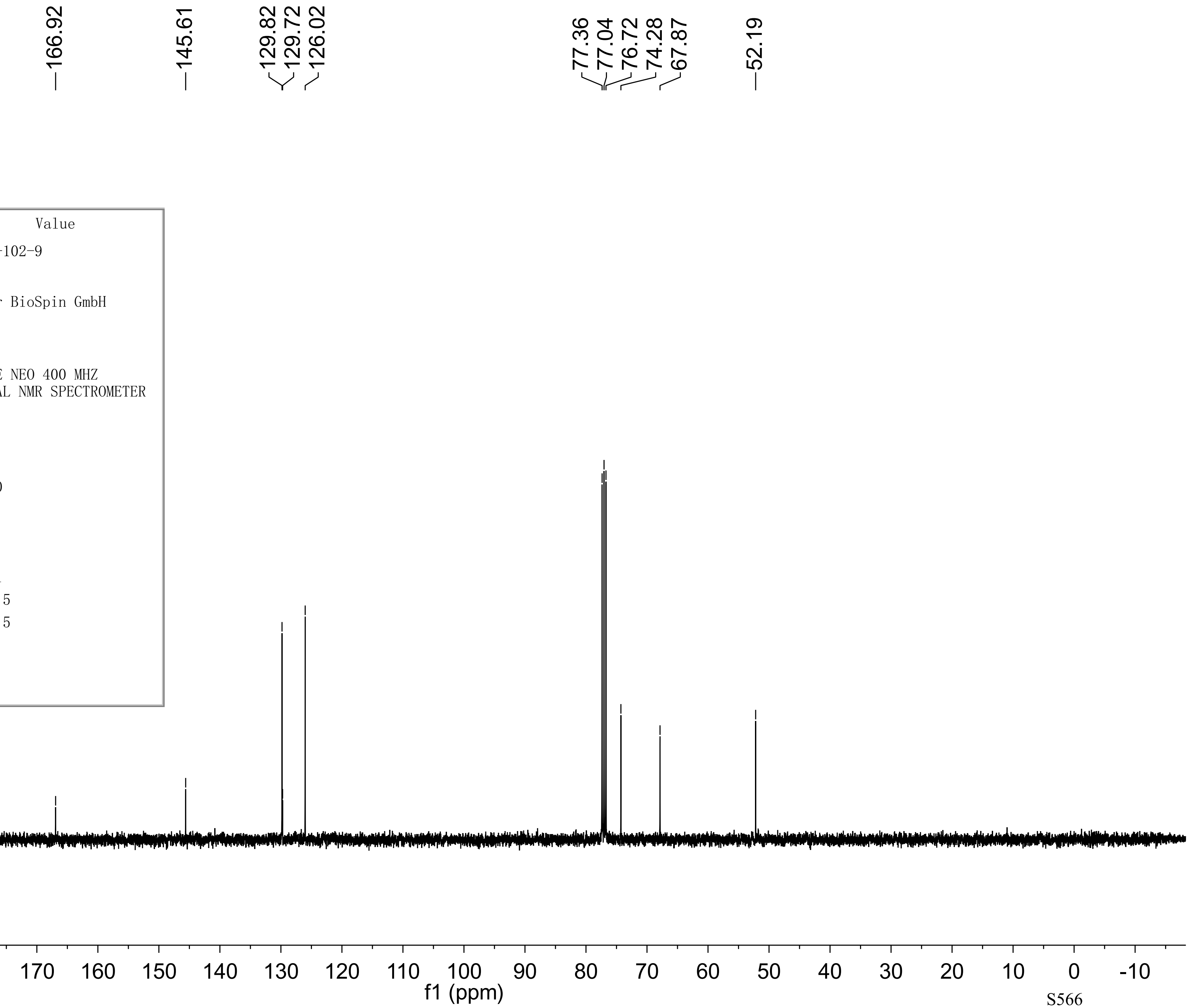

	Parameter	Value
1	Title	CFM-6-102-9
2	Comment	
3	Origin	Bruker BioSpin GmbH
4	Owner	nmrsu
5	Site	
6	Spectrometer	AVANCE NEO 400 MHZ DIGITAL NMR SPECTROMETER
7	Author	
8	Solvent	CDCl3
9	Temperature	298.1
10	Pulse Sequence	zgpg30
11	Experiment	1D
12	Number of Scans	64
13	Receiver Gain	62
14	Spectrometer Frequency	100.61
15	Spectral Width	23809.5
16	Lowest Frequency	-1843.5
17	Nucleus	13C
18	Acquired Size	32768
19	Spectral Size	32768

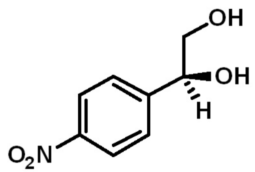

C79

	Parameter	Value
1	Title	CFM-6-102-11-1005
2	Comment	
3	Origin	Bruker BioSpin GmbH
4	Owner	nmrsu
5	Site	
6	Spectrometer	Avance NEO 600
7	Author	
8	Solvent	MeOD
9	Temperature	298.1
10	Pulse Sequence	zg30
11	Experiment	1D
12	Number of Scans	8
13	Receiver Gain	101
14	Spectrometer Frequency	600.15
15	Spectral Width	11904.8
16	Lowest Frequency	-2245.1
17	Nucleus	1H
18	Acquired Size	32768
19	Spectral Size	65536

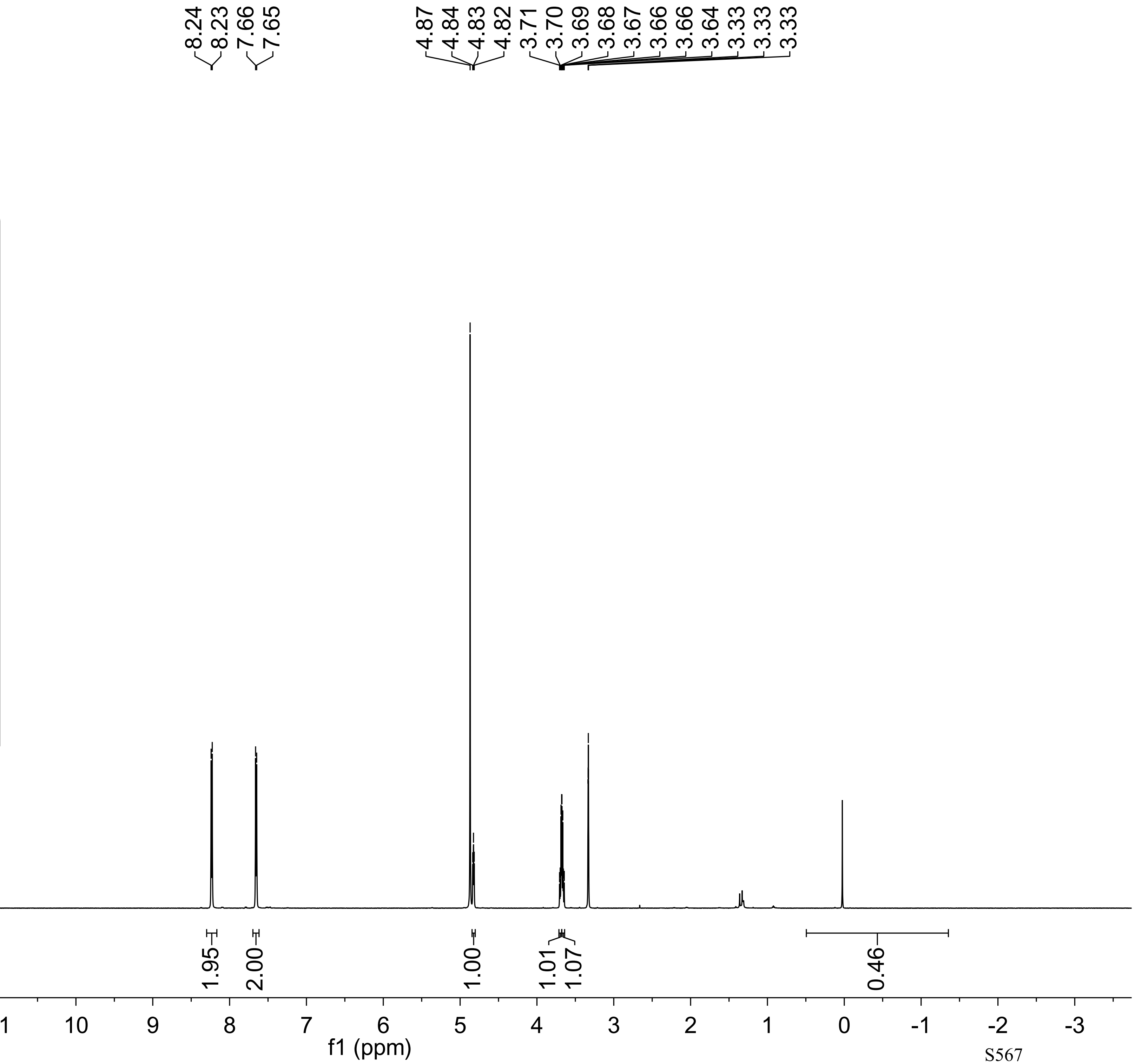

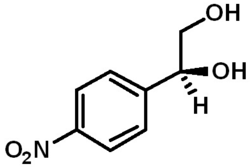

C79

Parameter	Value
1 Title	CFM-6-102-11-1005
2 Comment	
3 Origin	Bruker BioSpin GmbH
4 Owner	nmrsu
5 Site	
6 Spectrometer	Avance NEO 600
7 Author	
8 Solvent	MeOD
9 Temperature	298.1
10 Pulse Sequence	zgpg30
11 Experiment	1D
12 Number of Scans	102
13 Receiver Gain	101
14 Spectrometer Frequency	150.91
15 Spectral Width	35714.3
16 Lowest Frequency	-2555.1
17 Nucleus	13C
18 Acquired Size	32768
19 Spectral Size	32768

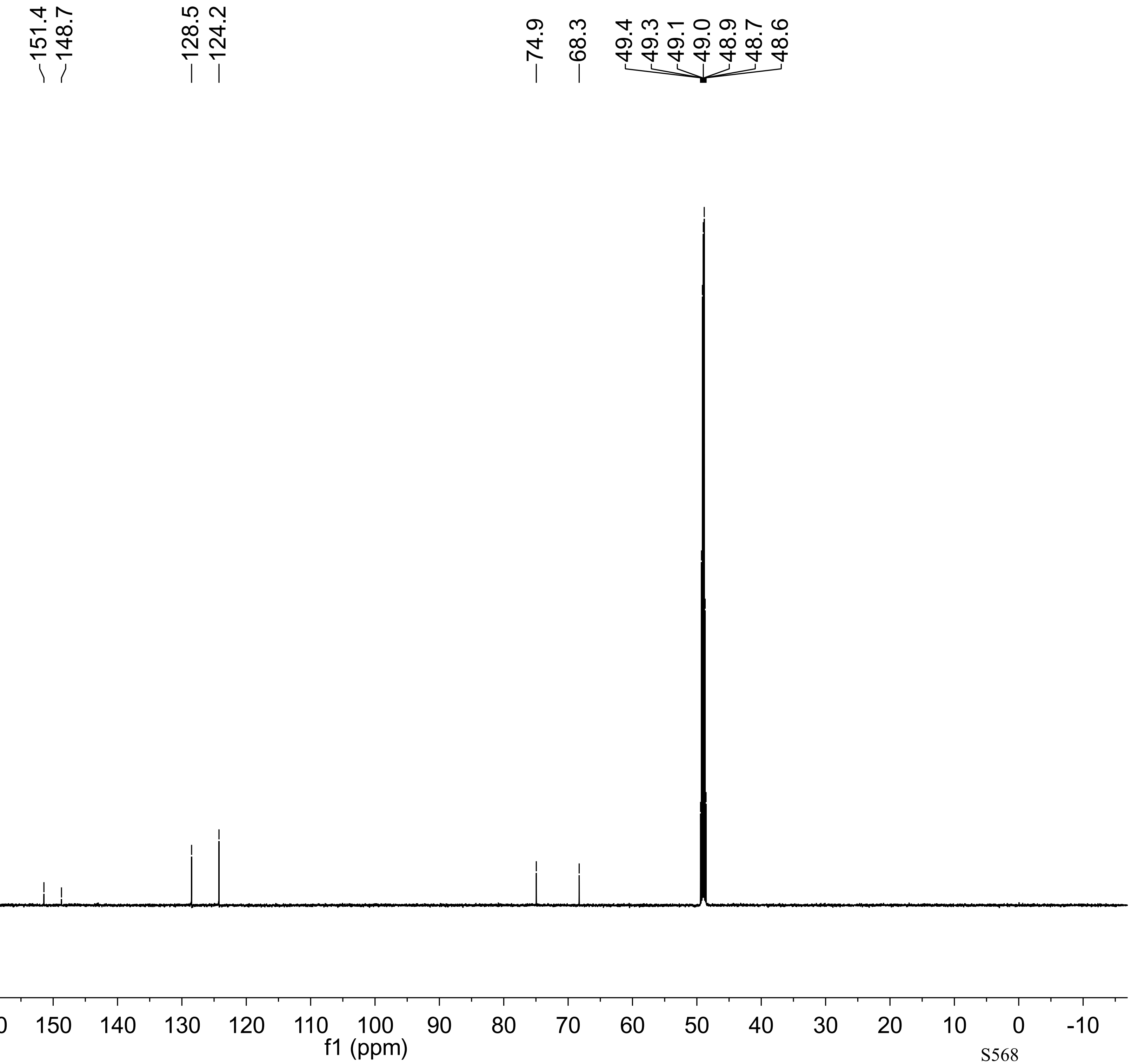

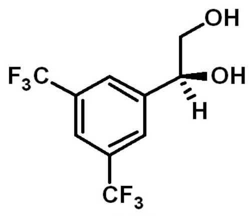

C80

Parameter	Value
1 Title	CFM-6-102-12-2
2 Comment	
3 Origin	Bruker BioSpin GmbH
4 Owner	nmrsu
5 Site	
6 Spectrometer	AVANCE NEO 400 MHZ DIGITAL NMR SPECTROMETER
7 Author	
8 Solvent	MeOD
9 Temperature	298.2
10 Pulse Sequence	zg30
11 Experiment	1D
12 Number of Scans	8
13 Receiver Gain	101
14 Spectrometer Frequency	400.13
15 Spectral Width	8196.7
16 Lowest Frequency	-1631.6
17 Nucleus	1H
18 Acquired Size	32768
19 Spectral Size	65536

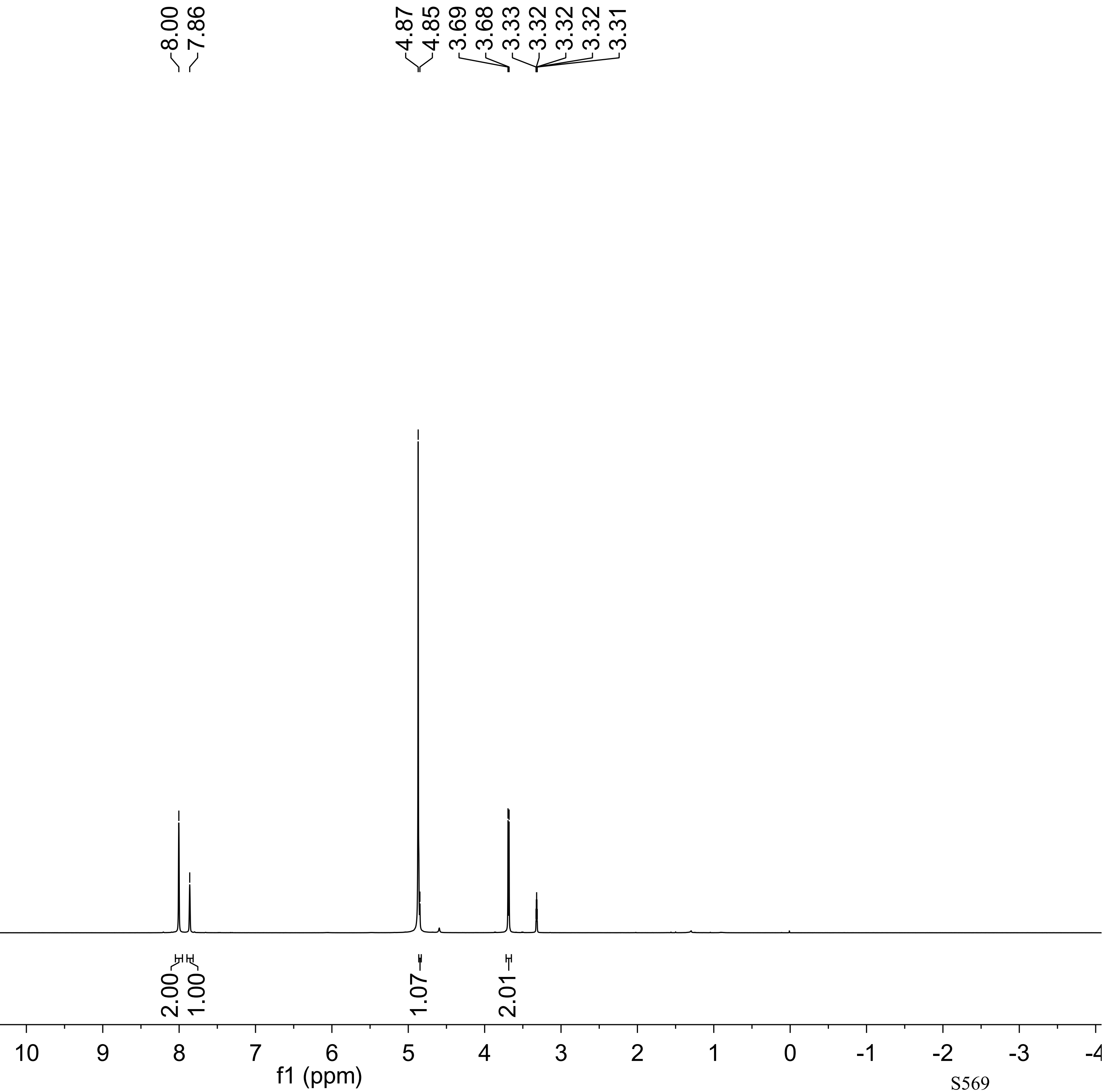

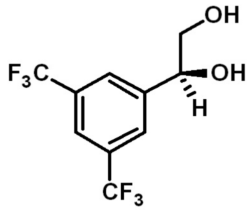

C80

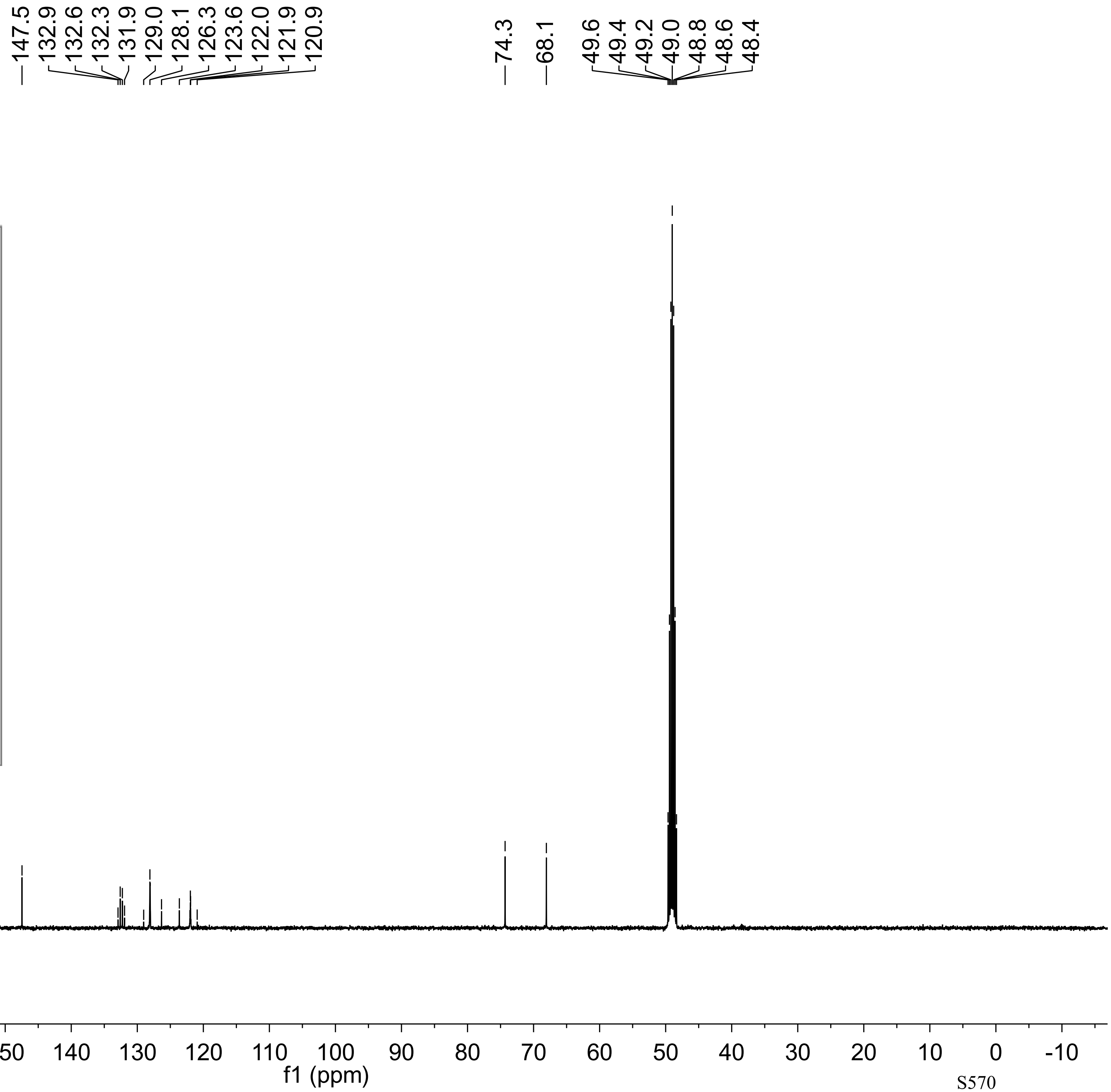

	Parameter	Value
1	Title	CFM-6-102-12-2
2	Comment	
3	Origin	Bruker BioSpin GmbH
4	Owner	nmrsu
5	Site	
6	Spectrometer	AVANCE NEO 400 MHZ DIGITAL NMR SPECTROMETER
7	Author	
8	Solvent	MeOD
9	Temperature	298.2
10	Pulse Sequence	zgpg30
11	Experiment	1D
12	Number of Scans	261
13	Receiver Gain	62
14	Spectrometer Frequency	100.61
15	Spectral Width	23809.5
16	Lowest Frequency	-1702.3
17	Nucleus	13C
18	Acquired Size	32768
19	Spectral Size	32768

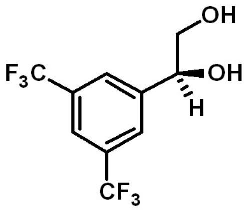

C80

Parameter		Value
1	Title	CFM-6-102-12-2
2	Comment	
3	Origin	Bruker BioSpin GmbH
4	Owner	nmrsu
5	Site	
6	Spectrometer	AVANCE NEO 400 MHZ DIGITAL NMR SPECTROMETER
7	Author	
8	Solvent	MeOD
9	Temperature	298.2
10	Pulse Sequence	zg
11	Experiment	1D
12	Number of Scans	16
13	Receiver Gain	101
14	Spectrometer Frequency	376.50
15	Spectral Width	90909.1
16	Lowest Frequency	-83104.4
17	Nucleus	19F
18	Acquired Size	65536
19	Spectral Size	65536

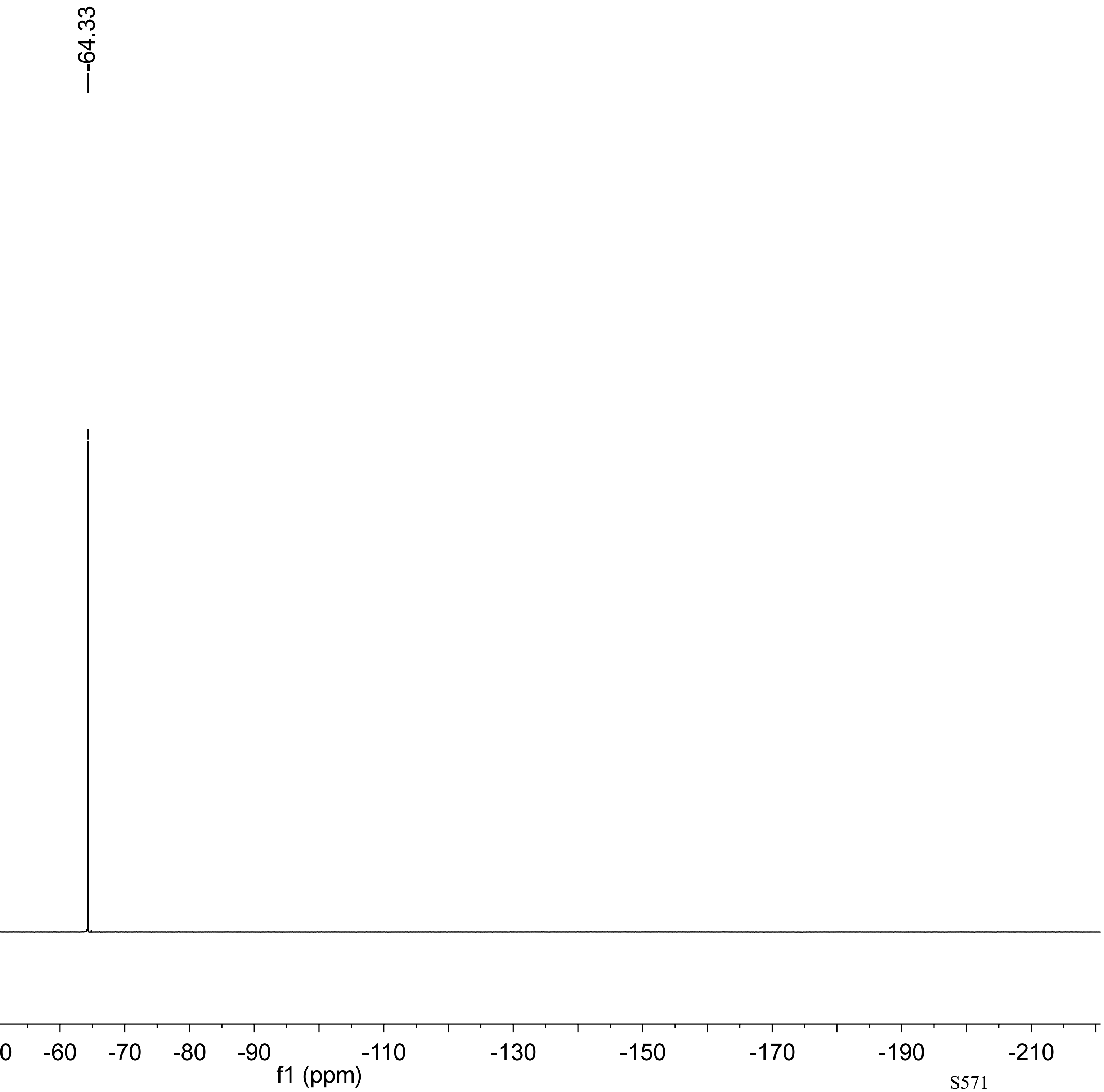

Supplement: Supplementary file 1 — Supplementary Information [file 41467_2024_47813_MOESM1_ESM.pdf]
