# Supplementary material for: Polarizability matters in enantio-selection
Source: Nat Commun. 2024 Apr 22;15:3394. doi: 10.1038/s41467-024-47813-4 (PMC11035643; doi:10.1038/s41467-024-47813-4)
Supplement: Supplementary file 3 — Description of Additional Supplementary Files [file 41467_2024_47813_MOESM3_ESM.pdf]

Description of Additional Supplementary Files

Title: Supplementary Data 1

Description: Cartesian Coordinates of computed structures
