# Supplementary material for: Polarizability matters in enantio-selection
Source: Nat Commun. 2024 Apr 22;15:3394. doi: 10.1038/s41467-024-47813-4 (PMC11035643; doi:10.1038/s41467-024-47813-4)
Supplement: Supplementary file 4 — Supplementary Data 1 [file 41467_2024_47813_MOESM4_ESM.pdf]

Cartesian coordinates of computed structures.

Coordinates of **A1:**

G_{gas} = -592.431796 Hartree

C	-0.011459	1.082612	0.002435
O	-0.036127	2.293522	0.013305
C	1.287297	0.343080	0.012890
C	1.422057	-0.955319	0.508256
C	2.418026	1.034365	-0.430602
C	2.677162	-1.551420	0.557737
H	0.553707	-1.496443	0.856830
C	3.665575	0.428848	-0.400014
H	2.297047	2.047594	-0.794372
C	3.796069	-0.865876	0.096941
H	2.780096	-2.555221	0.951860
H	4.536772	0.964852	-0.756827
H	4.770829	-1.338728	0.126906
C	-1.327365	0.337227	-0.025701
C	-2.462289	0.996265	0.446827
C	-3.682069	0.340193	0.378799
H	-2.364759	1.998422	0.843251
C	-2.535807	-1.500098	-0.627334
C	-3.723251	-0.934494	-0.173308
H	-4.585172	0.815045	0.743450
H	-2.531776	-2.490494	-1.071437
H	-4.652837	-1.483568	-0.254804
N	-1.355809	-0.888377	-0.550553

Coordinates of **A2:**

G_{gas} = -603.208376 Hartree

C	0.006418	1.119595	-0.122173
O	-0.030817	2.299594	-0.370038
C	1.320333	0.382725	-0.086825
C	2.500336	1.110013	0.044792
C	3.697749	0.409956	0.067816
H	2.453604	2.187987	0.130369
C	2.428110	-1.600608	-0.206152
C	3.663101	-0.973495	-0.059299
H	4.640574	0.931582	0.181628
H	2.366504	-2.678473	-0.316094
H	4.571885	-1.561933	-0.046367
N	1.272778	-0.942852	-0.213835
C	-1.257940	0.328722	0.071016
C	-1.557391	-0.405350	1.215661
C	-2.191555	0.368983	-0.964922

H	-1.964583	0.964049	-1.841953
C	-3.656355	-1.083529	0.264147
H	-4.582674	-1.639472	0.345769
C	-2.752645	-1.105233	1.321436
H	-2.980342	-1.659126	2.222965
I	-0.272500	-0.379232	2.913297
C	-3.380559	-0.341713	-0.878246
H	-4.090437	-0.311630	-1.695472

Coordinates of **A3:**

G_{gas} = -991.812472 Hartree

C	0.674999	-1.077479	-0.217988
O	0.684938	-2.288081	-0.287778
C	1.945901	-0.300653	-0.095724
C	2.064300	1.053802	-0.416180
C	3.302115	1.680465	-0.317974
H	1.196682	1.611685	-0.736529
C	4.417752	0.969453	0.109577
H	3.392942	2.728993	-0.574751
H	5.377925	1.465580	0.190742
C	-0.650079	-0.388599	-0.238101
C	-3.033746	0.045584	-0.355711
C	-2.242346	1.129944	-0.070829
N	-0.900923	0.865687	-0.009524
S	-2.057012	-1.358096	-0.543355
C	-2.725197	2.527178	0.167450
H	-2.337938	3.201373	-0.599660
H	-3.814011	2.577880	0.153963
H	-2.371875	2.887488	1.135329
C	-4.526687	0.007122	-0.491451
H	-5.007213	0.281754	0.450447
H	-4.861824	0.707412	-1.259514
H	-4.875893	-0.987078	-0.768482
C	3.074183	-1.014734	0.320852
H	2.965629	-2.067342	0.551834
C	4.302387	-0.381137	0.431430
H	5.170421	-0.937563	0.763756

Coordinates of **A4:**

G_{gas} = -1091.058214 Hartree

C	0.168435	-0.663303	-0.926462
O	0.003279	-1.609927	-1.660493
C	-0.969412	0.218813	-0.520884
C	-1.903167	0.581963	-1.492643

C	-2.973300	1.406805	-1.177207
H	-1.764488	0.207288	-2.499930
C	-3.132184	1.860855	0.129470
H	-3.682160	1.692409	-1.943852
H	-3.967225	2.501911	0.384825
C	1.527943	-0.292691	-0.461352
C	3.898339	-0.210217	0.016151
C	3.182837	0.916480	0.340671
N	1.845587	0.851232	0.064482
S	2.850739	-1.388829	-0.674911
C	3.743691	2.160319	0.956874
H	3.278398	2.343024	1.927608
H	4.822003	2.083370	1.096828
H	3.535034	3.022934	0.321210
C	5.365560	-0.455415	0.203850
H	5.653162	-1.444188	-0.152153
H	5.952835	0.283704	-0.345638
H	5.635848	-0.386143	1.259870
C	-2.232423	1.484435	1.119192
H	-2.344980	1.799007	2.148780
C	-1.165919	0.671755	0.778410
F	-0.341839	0.267526	1.754175

Coordinates of **A5:**

G_{gas} = -1106.289942 Hartree

C	0.039792	-0.695072	-0.754357
O	-0.161182	-1.801075	-1.196200
C	-1.029014	0.339811	-0.621614
C	-0.807860	1.638845	-1.067943
C	-1.824729	2.586908	-1.046089
H	0.172453	1.901809	-1.444143
C	-3.068630	2.231117	-0.545586
H	-1.643451	3.589595	-1.410823
H	-3.870736	2.959352	-0.515429
C	1.424727	-0.322102	-0.332691
C	3.805723	-0.340398	0.129668
C	3.124713	0.775142	0.548081
N	1.780453	0.767487	0.274479
S	2.715001	-1.443809	-0.625359
C	3.696692	1.962354	1.260803
H	4.760024	1.842204	1.463004
H	3.553929	2.866308	0.665161
H	3.179740	2.108195	2.211059
C	5.259850	-0.668686	0.276865

H	5.843262	0.239318	0.426717
H	5.429715	-1.327931	1.131814
H	5.643317	-1.169489	-0.613206
C	-3.307300	0.944812	-0.069291
H	-4.282136	0.697502	0.327198
C	-2.291604	-0.010740	-0.107087
O	-2.428588	-1.268642	0.361200
C	-3.686419	-1.660562	0.869201
H	-3.576171	-2.701387	1.163472
H	-3.966114	-1.061641	1.741466
H	-4.463074	-1.579122	0.102923

Coordinates of **A6:**

G_{gas} = -1145.386988 Hartree

C	0.652990	-1.519911	0.047080
O	0.946022	-2.697087	0.071835
C	1.773101	-0.529616	0.038423
C	3.992428	0.451267	0.070193
C	2.954304	1.328759	-0.116698
N	1.710693	0.757148	-0.131046
S	3.383693	-1.150183	0.224879
C	3.081854	2.809393	-0.300398
H	2.597054	3.340312	0.521905
H	4.127478	3.114970	-0.339649
H	2.594932	3.116719	-1.227725
C	5.458515	0.760466	0.131510
H	5.662096	1.510070	0.899006
H	6.043208	-0.128199	0.367229
H	5.811615	1.150845	-0.825893
C	-0.774773	-1.087343	0.013845
C	-1.196542	0.190741	0.296192
C	-1.730939	-2.099220	-0.284436
C	-2.577889	0.511881	0.289807
H	-0.479644	0.967980	0.522063
C	-3.063662	-1.809342	-0.308104
H	-1.369787	-3.098893	-0.489957
C	-3.527834	-0.496728	-0.020740
C	-3.033342	1.823534	0.583402
H	-3.789514	-2.580019	-0.543777
C	-4.905244	-0.163172	-0.030785
C	-4.370674	2.118004	0.566928
H	-2.300971	2.588025	0.819106
C	-5.316383	1.112412	0.255593
H	-5.628413	-0.935559	-0.269117

H	-4.710944	3.121766	0.791094
H	-6.371895	1.357263	0.245335

Coordinates of **A7:**

G_{gas} = -899.581601 Hartree

C	0.050520	2.037686	-0.034455
O	0.081266	3.248578	-0.068053
C	1.362945	1.294995	0.101277
C	2.448478	1.994878	0.694084
C	2.659076	-0.546888	-0.323160
C	3.657437	1.368465	0.781717
H	2.286823	3.002793	1.051843
C	2.765078	-1.854222	-0.863040
C	3.801751	0.057129	0.263685
H	4.512477	1.858819	1.235211
C	3.957315	-2.525201	-0.812649
H	1.879040	-2.288088	-1.310469
C	5.021895	-0.662259	0.302548
C	5.096878	-1.924620	-0.223390
H	4.035915	-3.523534	-1.226044
H	5.891344	-0.195540	0.752676
H	6.031993	-2.470745	-0.192718
N	1.453551	0.082115	-0.384977
C	-1.245693	1.304607	-0.127014
C	-1.422367	0.028062	0.350706
C	-2.352457	2.019014	-0.663495
C	-2.698495	-0.588570	0.312731
H	-0.590540	-0.525946	0.765247
C	-3.586352	1.439908	-0.728545
H	-2.186917	3.029699	-1.015289
C	-3.796904	0.121121	-0.241533
C	-2.903683	-1.902071	0.809472
H	-4.426945	1.981581	-1.148477
C	-5.067176	-0.506469	-0.285509
C	-4.142465	-2.483176	0.755338
H	-2.059687	-2.437126	1.231322
C	-5.235927	-1.776443	0.200784
H	-5.904066	0.037349	-0.710084
H	-4.291246	-3.486411	1.136093
H	-6.211469	-2.246573	0.163218

Coordinates of **A8:**

G_{gas} = -899.577219 Hartree

C	0.442414	-1.207095	-0.535917

O	0.814284	-2.356546	-0.623755
C	1.389973	-0.053862	-0.569926
C	2.690665	-0.145347	0.025565
C	1.026210	1.076035	-1.266152
C	3.129200	-1.257958	0.793947
C	3.578604	0.957057	-0.128144
C	1.924022	2.150286	-1.436729
H	0.038470	1.138903	-1.703684
C	4.375151	-1.267178	1.364504
H	2.474847	-2.108172	0.919544
C	4.864738	0.910986	0.469424
C	3.170211	2.090474	-0.876987
H	1.617205	3.018301	-2.007077
C	5.258388	-0.176458	1.199613
H	4.688741	-2.124433	1.948400
H	5.527178	1.759841	0.338163
H	3.868268	2.912741	-0.993972
H	6.241344	-0.204338	1.654334
C	-1.045314	-0.944875	-0.400651
C	-1.931645	-1.973830	-0.818095
C	-2.768630	0.377505	0.327108
C	-3.273201	-1.781540	-0.658745
H	-1.518181	-2.877963	-1.244337
C	-3.194083	1.588592	0.930906
C	-3.739288	-0.580486	-0.068059
H	-3.989630	-2.534034	-0.971330
C	-4.527514	1.829356	1.125140
H	-2.431313	2.298685	1.226316
C	-5.112430	-0.302682	0.144767
C	-5.496600	0.875516	0.727796
H	-4.850150	2.754855	1.586764
H	-5.849312	-1.038045	-0.159582
H	-6.547366	1.083553	0.889908
N	-1.434805	0.180449	0.144679

Coordinates of **A9:**

G_{gas} = -746.007384 Hartree

C	1.331247	1.353080	-0.118055
O	1.651281	2.502695	-0.324881
C	2.361236	0.275388	-0.013343
C	2.161423	-0.903223	0.708252
C	3.193826	-1.827096	0.825353
H	1.207718	-1.095835	1.179352
C	4.418166	-1.590423	0.209351

H	3.039895	-2.735432	1.395296
H	5.216408	-2.318598	0.294284
C	-0.141727	1.033311	0.020038
C	-1.009805	2.094020	0.394628
C	-1.883069	-0.437810	-0.213035
C	-2.348359	1.841984	0.475429
H	-0.586086	3.068289	0.597992
C	-2.329506	-1.743586	-0.541142
C	-2.833214	0.546650	0.165673
H	-3.049417	2.617168	0.766630
C	-3.662873	-2.048883	-0.487568
H	-1.583160	-2.472817	-0.831989
C	-4.206518	0.200772	0.213362
C	-4.611019	-1.068186	-0.105724
H	-4.001535	-3.046812	-0.738924
H	-4.927651	0.956960	0.504074
H	-5.662059	-1.328227	-0.069061
N	-0.548432	-0.177629	-0.269757
C	4.620553	-0.416954	-0.513289
H	5.574627	-0.230271	-0.991293
C	3.599850	0.516997	-0.613671
H	3.742454	1.445078	-1.153987

Coordinates of **A10:**

G_{gas} = -785.292143 Hartree

C	1.101763	-0.667920	-0.081841
O	0.948380	-1.706459	-0.687343
C	0.016896	0.353465	-0.017705
C	0.249546	1.711194	0.205639
C	-0.807939	2.612503	0.174210
H	1.252585	2.066833	0.395399
C	-2.114589	2.184074	-0.056692
H	-0.614483	3.667662	0.334990
C	-1.287648	-0.079926	-0.273152
H	-1.456343	-1.132476	-0.466873
C	-2.338163	0.822191	-0.281551
H	-3.347504	0.472197	-0.471288
C	2.424342	-0.441650	0.620150
C	3.550545	-1.164187	0.141320
C	3.630475	0.488993	2.332151
C	4.741835	-1.019149	0.790401
H	3.428576	-1.811063	-0.717018
C	4.817411	-0.175950	1.927258
C	3.662355	1.333819	3.471071

H	5.631250	-1.543044	0.456178
C	6.009240	0.023309	2.667323
C	4.828083	1.506948	4.167612
H	2.740755	1.824189	3.760221
C	6.013589	0.846205	3.762144
H	6.912913	-0.487548	2.352978
H	4.849731	2.152482	5.037423
H	6.926609	0.994557	4.326102
N	2.453425	0.350456	1.662900
C	-3.264173	3.155976	-0.045045
H	-3.995043	2.909081	-0.817065
H	-3.779261	3.126106	0.919188
H	-2.920054	4.177814	-0.208653

Coordinates of **A11:**

G_{gas} = -845.259739 Hartree

C	1.324465	-0.340549	-0.482708
O	1.350561	-1.123294	-1.406834
C	0.128635	0.520135	-0.242175
C	0.181126	1.736728	0.442734
C	-0.957968	2.521550	0.566001
H	1.110357	2.076259	0.877128
C	-2.140582	2.062934	0.013373
H	-0.943485	3.472802	1.081987
C	-1.080125	0.098494	-0.804402
H	-1.101166	-0.837937	-1.348225
C	-2.228934	0.861604	-0.671309
H	-3.178545	0.550324	-1.086958
F	-3.245159	2.812723	0.142589
C	2.529216	-0.269292	0.430254
C	3.770641	-0.725887	-0.088180
C	3.432191	0.139208	2.496790
C	4.860269	-0.720101	0.733219
H	3.814245	-1.069225	-1.112976
C	4.720030	-0.282759	2.073961
C	3.248364	0.574237	3.834309
H	5.831428	-1.050479	0.379900
C	5.795120	-0.254021	2.996579
C	4.305190	0.590054	4.704150
H	2.253990	0.884579	4.131513
C	5.591353	0.173041	4.281775
H	6.776817	-0.577022	2.667602
H	4.162436	0.922495	5.725318
H	6.415685	0.192291	4.984413

N	2.358479	0.146972	1.660655

Coordinates of **A12:**

G_{gas} = -1205.620667 Hartree

C	1.331813	-0.330371	-0.492051
O	1.358616	-1.102459	-1.424506
C	0.131363	0.524292	-0.242835
C	0.181914	1.742864	0.436440
C	-0.961370	2.520975	0.566472
H	1.112281	2.091450	0.861622
C	-2.157201	2.062415	0.030457
H	-0.930531	3.472330	1.081398
C	-1.079506	0.094225	-0.791245
H	-1.102547	-0.843949	-1.332222
C	-2.231192	0.851611	-0.648978
H	-3.175414	0.518583	-1.059712
C	2.534638	-0.263597	0.422633
C	3.776027	-0.723200	-0.092869
C	3.432398	0.141824	2.491815
C	4.863151	-0.721170	0.731935
H	3.821719	-1.065702	-1.117860
C	4.720171	-0.284038	2.072441
C	3.245932	0.577055	3.828921
H	5.834378	-1.054274	0.381410
C	5.792581	-0.259266	2.998291
C	4.300218	0.588939	4.701834
H	2.251694	0.890522	4.123254
C	5.586263	0.167857	4.283034
H	6.774201	-0.585271	2.672101
H	4.155626	0.921398	5.722726
H	6.408506	0.184072	4.988188
N	2.361252	0.152853	1.652615
Cl	-3.596382	3.026470	0.208632

Coordinates of **A13:**

G_{gas} = -860.490190 Hartree

C	1.755628	0.478533	-1.130545
O	1.973422	0.344288	-2.313906
C	0.400912	0.884218	-0.642570
C	0.209286	1.552181	0.559278
C	-1.070872	1.974550	0.925682
H	1.029372	1.761762	1.231414
C	-2.157931	1.708679	0.097016
C	-0.686209	0.630779	-1.488976

H	-0.513343	0.124961	-2.430024
C	-1.952391	1.033309	-1.108086
C	2.884897	0.214087	-0.158374
C	4.212642	0.309781	-0.655543
C	3.605523	-0.451106	1.912291
C	5.245873	0.025136	0.188935
H	4.364540	0.598416	-1.686899
C	4.967784	-0.373663	1.520299
C	3.284860	-0.854097	3.233917
H	6.276879	0.093051	-0.142163
C	5.977651	-0.697031	2.460211
C	4.281591	-1.161512	4.120408
H	2.237155	-0.906151	3.504007
C	5.641190	-1.082012	3.730787
H	7.016689	-0.635635	2.155197
H	4.034472	-1.468498	5.129576
H	6.416312	-1.329463	4.446079
N	2.585583	-0.146572	1.064546
H	-3.157751	2.018660	0.369470
O	-1.153137	2.633040	2.111708
H	-2.801443	0.830890	-1.749922
C	-2.425106	3.083951	2.525367
H	-3.117693	2.246947	2.659170
H	-2.272684	3.582688	3.479674
H	-2.846147	3.794236	1.806766

Coordinates of **A14:**

G_{gas} = -860.489410 Hartree

C	1.803415	0.625137	-1.283769
O	2.036300	0.572512	-2.469207
C	0.421049	0.841651	-0.764053
C	0.162779	1.688697	0.321757
C	-1.131430	1.792012	0.827039
C	-2.163571	1.077678	0.224910
C	-0.629672	0.166658	-1.376109
H	-0.407655	-0.461798	-2.230880
C	-1.924773	0.273971	-0.883224
C	2.919500	0.368200	-0.295714
C	4.255121	0.564522	-0.731529
C	3.589286	-0.354567	1.768670
C	5.268840	0.303311	0.144666
H	4.429712	0.913198	-1.740755
C	4.959847	-0.172415	1.443204
C	3.241523	-0.840340	3.056025

H	6.306684	0.449144	-0.136158
C	5.947553	-0.474119	2.414136
C	4.217223	-1.123361	3.973309
H	2.189150	-0.977416	3.274176
C	5.584281	-0.937884	3.650332
H	6.992425	-0.333048	2.159379
H	3.948202	-1.494428	4.955075
H	6.343025	-1.168378	4.388484
N	2.589596	-0.072068	0.892158
H	-3.167429	1.165786	0.623761
H	-2.735308	-0.268586	-1.352643
H	-1.342633	2.436243	1.670163
O	1.208965	2.416161	0.781796
C	1.148363	2.885703	2.114830
H	0.440054	3.713231	2.215035
H	0.871760	2.074228	2.794346
H	2.148329	3.239860	2.356479

Coordinates of **A15:**

G_{gas} = -899.579796 Hartree

C	0.054452	1.663929	0.127528
O	0.259720	2.854533	0.025367
C	1.238139	0.730240	0.200861
C	3.568020	0.325619	-0.242192
C	2.136159	-1.236089	0.941827
C	4.835876	0.676435	-0.773597
C	3.420218	-0.915439	0.423282
H	2.001090	-2.181620	1.463530
C	5.895814	-0.180073	-0.639388
H	4.948740	1.627397	-1.281710
C	4.531529	-1.783556	0.549613
C	5.744775	-1.421332	0.027450
H	6.863789	0.089175	-1.044948
H	4.406286	-2.730920	1.062615
H	6.597900	-2.082141	0.121846
C	2.428409	1.157303	-0.332070
H	2.481624	2.130387	-0.805698
N	1.086973	-0.465730	0.833891
C	-1.344095	1.140794	0.162466
C	-1.674516	-0.144158	-0.195115
C	-2.369845	2.067017	0.498479
C	-3.029673	-0.559676	-0.232979
H	-0.905679	-0.859806	-0.455427
C	-3.680099	1.685630	0.489220

H	-2.080626	3.077863	0.757642
C	-4.049364	0.363171	0.121416
C	-3.392135	-1.878996	-0.609863
H	-4.460207	2.390119	0.756664
C	-5.401066	-0.062889	0.091215
C	-4.706501	-2.262829	-0.631742
H	-2.607469	-2.578070	-0.878383
C	-5.721902	-1.343366	-0.276436
H	-6.177497	0.643894	0.363348
H	-4.975088	-3.271984	-0.920001
H	-6.758494	-1.658304	-0.297173

Coordinates of **A16:**

G_{gas} = -899.576019 Hartree

C	0.379979	-0.757982	-0.504895
O	0.513967	-1.907459	-0.864528
C	1.534197	0.188389	-0.430147
C	2.830811	-0.254391	-0.010762
C	1.351099	1.482240	-0.859788
C	3.094672	-1.560880	0.483613
C	3.905886	0.677981	-0.054748
C	2.428914	2.389301	-0.928528
H	0.365072	1.809395	-1.164086
C	4.352160	-1.910378	0.901163
H	2.295013	-2.286383	0.519375
C	5.197415	0.280245	0.378211
C	3.676415	1.994215	-0.531182
H	2.259104	3.395792	-1.290545
C	5.419614	-0.985759	0.845575
H	4.531475	-2.910753	1.277042
H	6.003825	1.004444	0.334655
H	4.512781	2.684195	-0.570084
H	6.408018	-1.282116	1.175776
C	-1.003709	-0.263576	-0.149965
C	-3.388943	-0.547456	-0.273158
C	-2.309190	1.208587	1.009795
C	-4.565688	-1.199958	-0.722972
C	-3.508274	0.577354	0.578637
H	-2.377077	2.070712	1.670496
C	-5.795510	-0.740988	-0.332815
H	-4.473443	-2.061927	-1.373971
C	-4.790998	1.032227	0.968368
C	-5.911606	0.384672	0.520015
H	-6.693476	-1.240769	-0.676073

H	-4.869641	1.894120	1.622046
H	-6.895051	0.729066	0.816046
C	-2.084160	-0.963395	-0.624909
H	-1.922719	-1.829645	-1.255635
N	-1.111017	0.822992	0.662984

Coordinates of **A17:**

G_{gas} = -746.005804 Hartree

C	1.239819	1.130669	-0.149886
O	1.272163	2.338739	-0.239644
C	-0.099192	0.435757	-0.163586
C	-2.481952	0.544898	0.146717
C	-1.319879	-1.435035	-0.642719
C	-3.683594	1.207674	0.506831
C	-2.542406	-0.800268	-0.291197
H	-1.343634	-2.466650	-0.988532
C	-4.880467	0.546925	0.428733
H	-3.636554	2.238106	0.840369
C	-3.792236	-1.461420	-0.363988
C	-4.937724	-0.799241	-0.010070
H	-5.797553	1.054326	0.703427
H	-3.826132	-2.491469	-0.701886
H	-5.896151	-1.301101	-0.064432
C	-1.209404	1.159224	0.192568
H	-1.097238	2.193872	0.494406
N	-0.149033	-0.860394	-0.577107
C	2.505246	0.343294	-0.024589
C	2.569569	-0.898162	0.610677
C	3.676121	0.934659	-0.505627
H	1.668966	-1.361461	0.989159
C	4.894442	0.284401	-0.373597
H	3.609796	1.906898	-0.978874
H	5.797232	0.741451	-0.760545
C	4.954841	-0.952533	0.263901
H	5.906454	-1.459536	0.373654
C	3.795233	-1.536539	0.762811
H	3.843545	-2.494429	1.266534

Coordinates of **A18:**

G_{gas} = -860.487661 Hartree

C	1.532707	0.099216	-0.885425
O	1.715974	-0.491505	-1.925575
C	0.206510	0.707580	-0.563830
C	0.095576	1.972618	0.027882

C	-1.158885	2.466997	0.378219
C	-2.295762	1.710146	0.107274
C	-0.943246	-0.015514	-0.861352
H	-0.832104	-0.979455	-1.344362
C	-2.198775	0.475380	-0.522059
C	2.627275	0.158178	0.148904
C	4.953886	-0.024170	0.721013
C	3.195428	0.378809	2.347772
C	4.579258	0.196502	2.068178
C	6.324618	-0.211343	0.408972
H	2.885618	0.534815	3.379674
C	5.570114	0.226967	3.078147
C	7.265988	-0.176829	1.403648
H	6.610781	-0.381833	-0.622734
C	6.888039	0.044317	2.750616
H	5.270535	0.394715	4.107089
H	8.312658	-0.320672	1.163218
H	7.649321	0.066391	3.520960
H	-3.267499	2.103066	0.382818
H	-3.088584	-0.101340	-0.739676
H	-1.258276	3.438777	0.843013
O	1.247144	2.672167	0.169342
C	1.268632	3.720868	1.116777
H	0.687128	4.581365	0.772980
H	0.885102	3.374945	2.081280
H	2.311551	4.013290	1.219946
C	3.921802	-0.050346	-0.246809
H	4.137124	-0.234929	-1.292752
N	2.257243	0.372840	1.441982

Coordinates of **A19:**

G_{gas} = -973.799696 Hartree

C	0.013666	1.658845	0.040750
O	0.222442	2.845206	-0.102155
C	1.197651	0.729542	0.160545
C	3.533255	0.311458	-0.237533
C	2.084304	-1.213897	0.972712
C	4.808926	0.646015	-0.760958
C	3.375436	-0.909424	0.462128
H	1.941142	-2.143659	1.519962
C	5.866326	-0.206371	-0.586698
H	4.929490	1.581635	-1.295145
C	4.484335	-1.773749	0.629495
C	5.705098	-1.427549	0.114087

H	6.840129	0.050628	-0.986271
H	4.351051	-2.705704	1.168085
H	6.556361	-2.085527	0.239986
C	2.395370	1.139849	-0.368918
H	2.455185	2.097965	-0.871369
N	1.037723	-0.446190	0.828056
C	-1.380947	1.133213	0.088199
C	-1.709134	-0.185967	-0.217959
C	-2.401378	2.047941	0.375754
C	-3.036016	-0.590830	-0.230904
H	-0.953925	-0.920666	-0.459516
C	-3.721362	1.640474	0.392838
H	-2.135515	3.075556	0.587912
C	-4.048149	0.318043	0.099743
H	-4.526874	2.322782	0.634825
O	-5.360633	-0.044541	0.134475
O	-3.315797	-1.890222	-0.560086
C	-5.570624	-1.449929	0.127952
H	-5.346577	-1.856064	1.120206
H	-6.624187	-1.605117	-0.100475
C	-4.674975	-2.097693	-0.907489
H	-4.873372	-1.664814	-1.894629
H	-4.827645	-3.175463	-0.945256

Coordinates of **A20:**

G_{gas} = -934.518654 Hartree

C	1.602512	0.453249	-0.708810
O	1.852086	0.240895	-1.876765
C	2.643008	0.094800	0.324664
C	4.951690	-0.321521	0.858737
C	3.136783	-0.594889	2.446619
C	4.528324	-0.664030	2.165691
C	6.334062	-0.378807	0.544719
H	2.787668	-0.861676	3.442301
C	5.482674	-1.056689	3.135064
C	7.238357	-0.760960	1.499400
H	6.657580	-0.118163	-0.456604
C	6.811507	-1.103013	2.806630
H	5.145937	-1.317748	4.132472
H	8.293690	-0.805004	1.257926
H	7.545311	-1.402722	3.544936
C	3.956139	0.055191	-0.071680
H	4.211709	0.302402	-1.095201
C	0.301137	1.064474	-0.311049

C	-0.733204	1.002646	-1.267844
C	0.119182	1.741089	0.894409
C	-1.916959	1.606061	-0.941989
H	-0.575206	0.503645	-2.214612
C	-1.090652	2.376027	1.199525
H	0.924015	1.778129	1.613611
C	-2.094075	2.279697	0.264616
H	-1.236027	2.914252	2.126279
C	-4.042931	2.194337	-0.777873
H	-4.672921	2.929802	-1.271702
H	-4.620846	1.348153	-0.389509
O	-3.061134	1.719171	-1.687061
O	-3.346988	2.813808	0.302243
N	2.230133	-0.225588	1.581964

Coordinates of **A21:**

G_{gas} = -899.576849 Hartree

C	0.477836	-1.178077	-0.748077
O	0.888035	-2.297479	-0.960880
C	1.457031	-0.022504	-0.722806
C	2.688565	-0.129596	-0.003516
C	3.043204	-1.243868	0.802716
C	3.580122	0.970804	-0.092355
C	1.995404	2.050025	-1.540325
C	4.232168	-1.247554	1.481982
H	2.374969	-2.091573	0.865424
C	4.806948	0.934027	0.618540
C	3.202466	2.071416	-0.899243
H	1.676882	2.876642	-2.165292
C	5.123478	-0.152020	1.387909
H	4.498083	-2.099246	2.096222
H	5.482188	1.778940	0.542207
H	3.867000	2.921744	-1.000515
H	6.060820	-0.178383	1.930912
C	-0.972075	-0.917829	-0.514864
C	-2.814703	0.439352	0.291831
C	-3.214660	-1.822395	-0.555652
C	-3.313316	1.645896	0.847797
C	-3.720912	-0.614154	-0.002565
H	-3.909503	-2.624715	-0.778910
C	-4.651029	1.798024	1.098457
H	-2.614906	2.445958	1.068184
C	-5.099814	-0.427575	0.266890
C	-5.553568	0.748644	0.804280

H	-5.024514	2.722443	1.522129
H	-5.789996	-1.233020	0.040539
H	-6.609852	0.880557	1.006658
C	-1.435252	0.260772	0.018415
H	-0.753720	1.073639	0.236805
C	-1.880533	-1.974251	-0.798352
H	-1.483087	-2.894199	-1.208708
N	1.118745	1.019253	-1.442633

Coordinates of **A22:**

G_{gas} = -899.572197 Hartree

C	-1.089459	-0.696712	-0.158491
O	-1.284871	-1.694786	-0.816110
C	-2.177213	0.248938	0.228978
C	-3.533976	-0.184802	0.396987
C	-1.861750	1.584916	0.336164
C	-4.525195	0.802995	0.661502
C	-3.939562	-1.546408	0.355106
C	-2.854202	2.557279	0.575836
H	-0.832520	1.901634	0.220819
C	-5.874812	0.410702	0.854152
C	-4.156221	2.171062	0.734337
C	-5.251231	-1.892086	0.553993
H	-3.205507	-2.313009	0.157541
H	-2.577164	3.602393	0.636451
C	-6.234355	-0.907973	0.799901
H	-6.614233	1.179360	1.051580
H	-4.930933	2.906409	0.924542
H	-5.537992	-2.936548	0.521436
H	-7.266524	-1.201159	0.949648
C	0.323766	-0.377950	0.294301
C	1.431914	-0.526813	-0.597744
C	2.722624	-0.282713	-0.060069
C	1.308482	-0.862831	-1.972553
C	1.681525	0.169083	2.057856
C	3.860607	-0.387020	-0.900444
C	2.820871	0.060366	1.310028
C	2.425509	-0.949358	-2.759990
H	0.330123	-1.060093	-2.387921
H	1.722477	0.432046	3.108785
C	3.712785	-0.713648	-2.220574
H	4.841968	-0.200558	-0.478744
H	3.793103	0.239788	1.754487
H	2.326057	-1.204545	-3.808050

H	4.582856	-0.791896	-2.861678
N	0.439275	-0.031657	1.552858

Coordinates of **A23:**

G_{gas} = -1066.824853 Hartree

C	-1.219690	-1.135532	0.196029
O	-1.363722	-2.334180	0.285361
C	-2.387165	-0.210739	0.118302
C	-2.324432	1.145883	0.445346
C	-3.478221	1.920510	0.395734
H	-1.384281	1.591468	0.735896
C	-4.687783	1.354413	0.008995
H	-3.429843	2.970301	0.658334
H	-5.581758	1.965502	-0.035064
C	0.187167	-0.610622	0.144857
C	2.612566	-0.497950	0.185149
C	1.932564	0.689177	-0.150535
C	4.008388	-0.538278	0.239744
C	2.654003	1.854810	-0.440132
C	4.703213	0.622635	-0.046480
H	4.530150	-1.451173	0.497909
C	4.032290	1.811287	-0.385038
H	2.118037	2.758854	-0.700992
H	5.785858	0.615184	-0.010558
H	4.608308	2.701498	-0.605199
N	0.556078	0.585512	-0.161267
S	1.467656	-1.767281	0.475817
C	-4.752656	0.001905	-0.319417
H	-5.694519	-0.440817	-0.619817
C	-3.609324	-0.779227	-0.255788
H	-3.640490	-1.835803	-0.492225

Coordinates of **A24:**

G_{gas} = -762.055730 Hartree

C	1.314202	1.323115	-0.111416
O	1.596225	2.482292	-0.319364
C	2.368496	0.272191	-0.007893
C	2.183161	-0.926780	0.683626
C	3.233593	-1.830520	0.795682
H	1.227926	-1.151828	1.137266
C	4.461719	-1.551719	0.205522
H	3.091314	-2.755166	1.341725
H	5.274696	-2.263757	0.286790
C	-0.146943	0.966672	0.027880

C	-1.048483	1.996073	0.441147
C	-1.895800	-0.460496	-0.219744
H	-0.654532	2.976347	0.686638
C	-2.402481	-1.736483	-0.568581
C	-2.787658	0.558290	0.202265
C	-3.748023	-1.976944	-0.491997
H	-1.696557	-2.492073	-0.891427
C	-4.173643	0.282284	0.273509
C	-4.639359	-0.959976	-0.067376
H	-4.140885	-2.950843	-0.757746
H	-4.832490	1.077821	0.599349
H	-5.700302	-1.172902	-0.014788
N	-0.556544	-0.232518	-0.290872
C	4.649603	-0.357439	-0.486736
H	5.606830	-0.139083	-0.944408
C	3.610289	0.555595	-0.583366
H	3.739588	1.498104	-1.101574
N	-2.333014	1.798745	0.538967

Coordinates of **A25:**

G_{gas} = -897.413198 Hartree

C	-0.177999	-0.797603	-0.616718
O	-0.355162	-1.938816	-0.967771
C	-1.265745	0.207249	-0.463663
C	-2.578021	-0.190880	-0.039531
C	-1.014498	1.517213	-0.806584
C	-2.912530	-1.510761	0.367272
C	-3.593399	0.805710	0.013522
C	-2.035934	2.488576	-0.774173
H	-0.020827	1.809827	-1.117180
C	-4.180263	-1.810979	0.793931
H	-2.162746	-2.287019	0.329708
C	-4.896681	0.458575	0.452967
C	-3.294352	2.138206	-0.370981
H	-1.813297	3.507207	-1.066084
C	-5.187995	-0.822119	0.834842
H	-4.413061	-2.823387	1.101945
H	-5.655633	1.232798	0.484130
H	-4.087923	2.877103	-0.334973
H	-6.184940	-1.080626	1.170774
C	1.229077	-0.367960	-0.302424
C	3.353001	-0.659710	-0.248074
C	3.010946	0.518121	0.415966
C	4.655071	-1.115024	-0.383640

C	4.000626	1.313685	0.994707
C	5.629635	-0.314667	0.195655
H	4.888848	-2.033466	-0.904882
C	5.309385	0.876921	0.872142
H	3.744198	2.227978	1.514123
H	6.667648	-0.615988	0.126335
H	6.110055	1.462895	1.306007
N	1.629559	0.666929	0.354275
O	2.204805	-1.223553	-0.703130

Coordinates of **A26:**

G_{gas} = -734.681252 Hartree

C	-0.819391	1.081393	-0.334673
O	-0.907155	2.250053	-0.638186
C	-2.103070	0.310132	-0.120092
C	-3.229351	1.070448	0.211661
C	-4.461364	0.465534	0.415370
H	-3.116123	2.143570	0.303276
C	-3.484553	-1.677233	-0.077297
C	-4.592649	-0.912329	0.268244
H	-5.320820	1.068308	0.683477
H	-3.582028	-2.748827	-0.203794
H	-5.554719	-1.387425	0.419846
C	-2.245270	-1.072756	-0.261143
H	-1.402261	-1.693036	-0.530194
C	0.562989	0.443250	-0.151515
C	0.931767	-0.385737	-1.409007
C	1.604680	1.574432	-0.018353
C	0.644539	-0.440160	1.113396
H	0.895123	0.269387	-2.286117
H	0.216637	-1.193009	-1.582485
C	2.340799	-0.977576	-1.246754
H	1.351002	2.196827	0.845959
H	1.550086	2.220706	-0.897230
C	3.017331	0.996673	0.135477
H	-0.081553	-1.254043	1.081252
H	0.401217	0.167862	1.992424
C	2.058209	-1.026299	1.251624
H	2.576045	-1.577175	-2.131059
C	3.362817	0.158224	-1.102975
C	2.382401	-1.866069	0.006163
H	3.730125	1.820818	0.231397
C	3.080398	0.110081	1.386186
H	2.092426	-1.661995	2.141198

H	3.350454	0.788505	-1.998434
H	4.372114	-0.256670	-1.007963
H	1.661441	-2.686086	-0.090580
H	3.375042	-2.317162	0.108664
H	4.087751	-0.303277	1.505603
H	2.862612	0.704826	2.279747

Coordinates of **A27:**

G_{gas} = -579.929918 Hartree

C	-1.010212	-0.626613	0.223841
O	-1.258509	-1.700670	-0.274838
C	0.424736	-0.161150	0.405095
C	1.408280	-1.330313	0.338794
C	0.759777	0.882787	-0.681046
C	2.849569	-0.839916	0.486242
H	1.281184	-1.839036	-0.621711
H	1.164678	-2.063315	1.112034
C	2.202592	1.371753	-0.539453
H	0.627792	0.407520	-1.660628
H	0.063266	1.724100	-0.639582
C	3.190306	0.204330	-0.579065
H	3.539083	-1.685480	0.421704
H	2.984962	-0.397271	1.481014
H	2.429070	2.090786	-1.331103
H	2.309407	1.904131	0.413934
H	4.212070	0.568546	-0.443741
H	3.147712	-0.266435	-1.568517
C	-2.135092	0.289348	0.631256
C	-3.439488	-0.105608	0.323166
C	-1.929260	1.496513	1.302663
C	-4.518363	0.691212	0.677814
H	-3.582040	-1.046070	-0.195160
C	-3.010847	2.294639	1.660149
H	-0.927903	1.824868	1.553344
C	-4.305166	1.893675	1.347911
H	-5.526293	0.377511	0.433860
H	-2.842366	3.228728	2.182402
H	-5.147022	2.516731	1.626282
H	0.519148	0.321457	1.385669

Coordinates of **A28:**

G_{gas} = -384.722928 Hartree

C	-1.009852	-0.575010	0.257414
O	-1.196508	-1.650307	-0.260901

C	-2.170182	0.302837	0.633939
C	-3.461940	-0.157963	0.369171
C	-1.995401	1.550807	1.233928
C	-4.562728	0.618535	0.699971
H	-3.576822	-1.129019	-0.097161
C	-3.099453	2.329433	1.565540
H	-1.000294	1.923237	1.445937
C	-4.382200	1.863816	1.298893
H	-5.562700	0.256743	0.492791
H	-2.958102	3.297335	2.031058
H	-5.242221	2.470493	1.557169
C	0.390869	-0.080784	0.543845
H	0.579398	0.859111	0.019839
H	0.520968	0.101971	1.613046
H	1.100543	-0.834760	0.211568

Coordinates of **A29:**

G_{gas} = -1000.162452 Hartree

C	0.191486	0.232833	-0.360010
O	0.163079	0.833546	-1.407533
C	-1.089539	0.079372	0.480691
H	-0.792900	-0.073980	1.520513
C	-1.927669	1.343429	0.426371
C	-1.961143	2.192635	1.530923
C	-2.657475	1.684858	-0.712559
C	-2.711388	3.364958	1.503853
H	-1.395815	1.935625	2.421101
C	-3.403454	2.856064	-0.742574
H	-2.639280	1.031333	-1.576322
C	-3.434122	3.699245	0.365367
H	-2.730084	4.013101	2.372062
H	-3.964877	3.111813	-1.633432
H	-4.020411	4.610093	0.340138
C	-1.875051	-1.151304	0.040317
C	-1.857648	-1.622239	-1.272174
C	-2.665093	-1.811066	0.984327
C	-2.617538	-2.733103	-1.630505
H	-1.250552	-1.123656	-2.019568
C	-3.422092	-2.919449	0.627048
H	-2.689991	-1.443489	2.005499
C	-3.399467	-3.384972	-0.684836
H	-2.593228	-3.089426	-2.653582
H	-4.030044	-3.419555	1.371756
H	-3.987334	-4.250450	-0.966011

C	1.440789	-0.423381	0.148183
C	2.742354	0.102567	-0.152317
C	1.320616	-1.587547	0.875113
C	3.880449	-0.616120	0.314623
C	2.961405	1.321146	-0.851876
C	2.455078	-2.303043	1.311790
H	0.337925	-1.991074	1.090891
C	5.182925	-0.115618	0.056994
C	3.705792	-1.825022	1.035915
C	4.233319	1.779543	-1.077581
H	2.115237	1.884196	-1.215857
H	2.325855	-3.227536	1.860758
C	5.359538	1.055752	-0.626300
H	6.035140	-0.680628	0.418918
H	4.588062	-2.362087	1.367758
H	4.376605	2.711638	-1.611243
H	6.356732	1.433098	-0.818540

Coordinates of **A30:**

G_{gas} = -656.122679 Hartree

C	1.439122	-0.116336	-0.791775
O	1.584379	-0.606915	-1.884234
C	2.504033	-0.248565	0.305069
C	1.854171	-0.784524	1.590114
H	1.436539	-1.783265	1.441130
H	2.612827	-0.850488	2.374335
H	1.055208	-0.127893	1.941957
C	3.110799	1.136547	0.590242
H	3.939899	1.020115	1.292965
H	3.504136	1.593876	-0.321767
H	2.382344	1.812337	1.041297
C	3.596828	-1.202156	-0.175561
H	3.183533	-2.185230	-0.409469
H	4.078681	-0.824010	-1.078387
H	4.352192	-1.317421	0.605617
C	0.163680	0.631324	-0.470214
C	-1.023630	-0.109343	-0.189351
C	0.135206	2.002742	-0.483792
C	-1.059634	-1.529764	-0.201952
C	-2.216346	0.601608	0.116049
C	-1.060797	2.704688	-0.203267
H	1.032174	2.560677	-0.726261
C	-2.215072	-2.202989	0.093286
H	-0.166498	-2.081320	-0.472190

C	-3.396402	-0.126187	0.419247
C	-2.204768	2.020606	0.101355
H	-1.060204	3.787691	-0.228626
C	-3.397114	-1.494725	0.412800
H	-2.228746	-3.286160	0.074682
H	-4.300456	0.425573	0.653488
H	-3.122831	2.551526	0.328636
H	-4.303728	-2.040743	0.644440

Coordinates of **A31:**

G_{gas} = -615.637664 Hartree

C	0.433014	-1.050827	-0.796645
O	0.793807	-2.185918	-1.025748
C	1.412087	0.076459	-0.650120
C	2.705525	-0.115900	-0.062590
C	1.062521	1.308083	-1.155049
C	3.134344	-1.344663	0.509622
C	3.595076	0.995440	-0.016913
C	1.960795	2.396085	-1.130654
H	0.088405	1.441677	-1.611321
C	4.373469	-1.452617	1.085043
H	2.477716	-2.201512	0.474868
C	4.872754	0.846609	0.582105
C	3.197859	2.240401	-0.569688
H	1.663735	3.346789	-1.555941
C	5.256950	-0.350276	1.121219
H	4.681644	-2.397707	1.516267
H	5.536698	1.703997	0.606096
H	3.897528	3.068814	-0.536808
H	6.233770	-0.456620	1.577640
C	-1.019594	-0.725612	-0.681912
C	-1.934176	-1.375919	-1.705961
C	-1.933462	-1.869678	-0.305423
H	-1.284594	0.258629	-0.323879
H	-1.435991	-1.988259	-2.446351
H	-2.773959	-0.790574	-2.055128
H	-2.774539	-1.627050	0.329427
H	-1.438657	-2.812535	-0.113802

Coordinates of **A32:**

G_{gas} = -1000.168251 Hartree

C	0.404444	-1.040254	-0.680465
O	0.902775	-1.661331	-1.587499
C	1.267895	-0.328666	0.371781

H	0.780276	-0.471149	1.340499
C	2.655796	-0.930483	0.466056
C	3.007493	-1.686368	1.581762
C	3.594259	-0.745800	-0.549485
C	4.276053	-2.250683	1.687462
H	2.283515	-1.837806	2.376194
C	4.858515	-1.310707	-0.448424
H	3.331053	-0.159026	-1.421772
C	5.204278	-2.063756	0.671189
H	4.535511	-2.833955	2.563068
H	5.577806	-1.163546	-1.245385
H	6.192698	-2.500784	0.748892
C	1.311480	1.169521	0.095987
C	1.185877	1.689918	-1.191218
C	1.519382	2.046625	1.162095
C	1.265446	3.063299	-1.406873
H	1.023365	1.024452	-2.032996
C	1.597795	3.416778	0.948326
H	1.630524	1.646142	2.165124
C	1.469676	3.929633	-0.339817
H	1.163745	3.454126	-2.412358
H	1.760800	4.084643	1.786007
H	1.528913	4.998066	-0.508893
C	-1.087242	-0.925883	-0.567903
C	-1.703859	-0.069413	0.312555
C	-1.873522	-1.736128	-1.431750
C	-3.117574	0.018446	0.373561
H	-1.125417	0.577649	0.963368
C	-3.236574	-1.679489	-1.384714
H	-1.359352	-2.396034	-2.119340
C	-3.898541	-0.802725	-0.482846
C	-3.771500	0.904766	1.268393
H	-3.835845	-2.304251	-2.038196
C	-5.311872	-0.717206	-0.413135
C	-5.138622	0.966657	1.313251
H	-3.167863	1.532732	1.914855
C	-5.916755	0.145132	0.463085
H	-5.906175	-1.346057	-1.067091
H	-5.631005	1.645535	1.998966
H	-6.997773	0.203253	0.507970

Coordinates of **A33:**

G_{gas} = -3420.180122 Hartree

C	0.409565	-0.999165	-0.704920

O	0.901919	-1.660366	-1.585292
C	1.270853	-0.293142	0.350507
H	0.761066	-0.408756	1.311938
C	2.641984	-0.925561	0.480170
C	2.950114	-1.682301	1.608108
C	3.606588	-0.770087	-0.515384
C	4.201982	-2.276092	1.745397
H	2.205525	-1.810867	2.387360
C	4.854431	-1.364263	-0.382568
H	3.377633	-0.182249	-1.396642
C	5.156734	-2.118086	0.748951
H	4.427944	-2.859631	2.630020
H	5.594706	-1.239330	-1.163906
H	6.132330	-2.578115	0.851579
C	1.349641	1.198888	0.050006
C	1.262990	1.697244	-1.249155
C	1.548391	2.091601	1.104896
C	1.371490	3.064918	-1.487814
H	1.109516	1.019353	-2.082789
C	1.655787	3.455830	0.867968
H	1.628795	1.708106	2.117421
C	1.566208	3.946863	-0.431979
H	1.300230	3.438884	-2.502257
H	1.810777	4.136282	1.696917
H	1.647762	5.010830	-0.618784
C	-1.081421	-0.826718	-0.626425
C	-1.698319	0.077681	0.239753
C	-1.870367	-1.612561	-1.469262
H	-1.108954	0.717523	0.885585
C	-3.252943	-1.510586	-1.446379
H	-1.379427	-2.307165	-2.140191
H	-3.866721	-2.123720	-2.093314
C	-3.082557	0.196849	0.267293
H	-3.563629	0.903041	0.931024
C	-3.844870	-0.602826	-0.574291
Br	-5.734874	-0.450991	-0.534716

Coordinates of **A34:**

G_{gas} = -883.525551 Hartree

C	0.29272000	-0.42551000	1.15048200
O	0.58649700	-1.17401600	2.05702100
C	1.32143400	0.46135100	0.51109600
C	2.62630400	-0.03251200	0.19070600
C	1.01418600	1.78687500	0.30800100

C	3.00692800	-1.39262000	0.35028600
C	3.57767300	0.87859200	-0.34883500
C	1.97397700	2.69008700	-0.19832500
H	0.02388500	2.14888800	0.56029800
C	4.26087900	-1.81263800	-0.00767400
H	2.30108900	-2.09473300	0.77146300
C	4.86932100	0.41049100	-0.70420000
C	3.22466200	2.24187300	-0.52297200
H	1.71177900	3.73296600	-0.32759300
C	5.20601100	-0.90487700	-0.53816000
H	4.53391100	-2.85330600	0.12051400
H	5.58289000	1.11774900	-1.11299100
H	3.96916800	2.92341800	-0.92056800
H	6.19396800	-1.25505000	-0.81221200
C	-1.12189200	-0.36087400	0.65924500
C	-2.14503700	-0.83187900	1.52633200
C	-2.78117400	0.03917100	-1.06824300
C	-3.44684100	-0.84449400	1.11588300
H	-1.86010900	-1.17726600	2.51250200
C	-3.12885700	0.46001200	-2.37790800
C	-3.80344100	-0.41114000	-0.19069000
H	-4.22999800	-1.19179400	1.78109600
C	-4.43361100	0.43907800	-2.79343800
H	-2.34210600	0.79931400	-3.04308000
C	-5.14490500	-0.42054700	-0.64864300
C	-5.45246500	-0.00551500	-1.91788300
H	-4.69177600	0.76239900	-3.79461600
H	-5.92390200	-0.76392000	0.02332800
H	-6.48101900	-0.01759500	-2.25832900
C	-1.43928500	0.06020200	-0.60971900
H	-0.66279100	0.39915000	-1.28693300

Coordinates of **A35:**

G_{gas} = -729.951088 Hartree

C	-0.759972	-0.726436	-0.631103
O	-0.601136	-1.800232	-1.168964
C	-2.121210	-0.323228	-0.142710
C	-3.233091	-0.935888	-0.725457
C	-4.511216	-0.629020	-0.281657
H	-3.072002	-1.650401	-1.523871
C	-3.581729	0.872502	1.362062
C	-4.686268	0.275249	0.764017
H	-5.372111	-1.096013	-0.744779
H	-3.716890	1.564685	2.184364

H	-5.684286	0.509893	1.115215
C	-2.300562	0.581381	0.905395
H	-1.439734	1.042961	1.374473
C	0.379346	0.235254	-0.459836
C	1.682122	-0.217337	-0.074624
C	0.168516	1.561616	-0.759053
C	1.970185	-1.564603	0.274921
C	2.733434	0.739971	-0.003528
C	1.223961	2.497976	-0.715764
H	-0.821730	1.890248	-1.053857
C	3.230820	-1.928822	0.669125
H	1.187142	-2.307114	0.214141
C	4.028520	0.327004	0.403662
C	2.475743	2.093755	-0.341469
H	1.033939	3.531591	-0.977530
C	4.274598	-0.977642	0.732848
H	3.432056	-2.960474	0.932176
H	4.818286	1.068969	0.451133
H	3.294422	2.804098	-0.296028
H	5.265810	-1.285667	1.043319

Coordinates of **A36:**

G_{gas} = -729.954315 Hartree

C	1.243317	1.064411	0.083220
O	1.231573	2.275626	0.103533
C	-0.045117	0.300841	0.064382
C	-1.413177	-1.587653	-0.612964
C	-2.405610	0.300464	0.588667
C	-1.557127	-2.849522	-1.245726
C	-2.552087	-0.971822	-0.028512
H	-3.279320	0.770462	1.027044
C	-2.775752	-3.472772	-1.289047
H	-0.683821	-3.311654	-1.693400
C	-3.800062	-1.641809	-0.087992
C	-3.909354	-2.861758	-0.702183
H	-2.877713	-4.436431	-1.773336
H	-4.667896	-1.170112	0.359979
H	-4.868144	-3.364751	-0.743155
C	-0.160581	-0.926162	-0.542967
H	0.702940	-1.397839	-0.999963
C	2.546660	0.322404	0.070249
C	2.708772	-0.911379	0.703049
C	3.643409	0.936987	-0.537535
H	1.867709	-1.378000	1.202333

C	4.881850	0.310723	-0.539272
H	3.504523	1.906535	-1.000953
H	5.727150	0.784001	-1.024210
C	5.039223	-0.921810	0.090744
H	6.008220	-1.406952	0.096619
C	3.956216	-1.526507	0.720051
H	4.082247	-2.476141	1.225975
C	-1.192679	0.926177	0.622589
H	-1.075034	1.905622	1.069792

Coordinates of **A37:**

G_{gas} = -732.281421 Hartree

C	0.387887	-1.064588	-0.614495
O	0.818828	-1.854963	-1.418214
C	1.279258	-0.340069	0.400310
H	0.769909	-0.427829	1.366891
C	2.655245	-0.955400	0.530916
C	3.030172	-1.581033	1.717277
C	3.565512	-0.907845	-0.525437
C	4.294049	-2.149715	1.853042
H	2.328265	-1.626479	2.543966
C	4.824566	-1.477705	-0.394625
H	3.283366	-0.425323	-1.454038
C	5.193853	-2.099097	0.796216
H	4.571960	-2.630810	2.783403
H	5.521139	-1.438360	-1.223725
H	6.178071	-2.540860	0.897034
C	1.336706	1.143321	0.055768
C	1.251278	1.593287	-1.261818
C	1.520596	2.077042	1.076392
C	1.350664	2.951066	-1.552403
H	1.104783	0.882143	-2.069187
C	1.619207	3.432542	0.788454
H	1.596801	1.733200	2.103087
C	1.534184	3.873617	-0.529159
H	1.282286	3.286344	-2.580564
H	1.762857	4.145135	1.592080
H	1.609881	4.930364	-0.755590
C	-1.056847	-0.703672	-0.564232
C	-1.807408	-0.725590	-1.877946
C	-2.039660	-1.810443	-0.889489
H	-1.346746	0.038584	0.168420
H	-1.211014	-0.970274	-2.747625
H	-2.570823	0.028032	-2.014331

H	-2.967139	-1.818350	-0.333475
H	-1.597134	-2.775341	-1.100224

Coordinates of **A38:**

G_{gas} = -310.896658 Hartree

C	-0.958102	-0.648909	0.591822
O	-1.070124	-1.833738	0.789742
C	0.355891	-0.072413	0.101004
H	0.210819	0.698512	-0.657171
H	0.874518	0.392050	0.944526
H	0.972480	-0.878505	-0.291425
C	-2.130028	0.314461	0.819796
C	-1.650665	1.579492	1.544402
C	-2.693797	0.690351	-0.562242
C	-3.212264	-0.385543	1.640614
H	-1.199876	1.335183	2.510238
H	-0.920467	2.138304	0.954908
H	-2.502866	2.238145	1.729754
H	-3.025765	-0.199919	-1.101597
H	-3.554447	1.352155	-0.434541
H	-1.958463	1.214570	-1.177015
H	-4.060676	0.288051	1.785253
H	-3.559497	-1.288496	1.137720
H	-2.829904	-0.679649	2.620413

Coordinates of **A39:**

G_{gas} = -608.482776 Hartree

C	0.008305	1.092883	-0.011580
O	0.020835	2.303616	-0.016377
C	1.320155	0.347086	0.018573
C	2.470517	1.020635	-0.390558
C	3.683386	0.351664	-0.320592
H	2.389906	2.040831	-0.742339
C	2.496811	-1.528607	0.559893
C	3.699986	-0.949304	0.167643
H	4.599712	0.836215	-0.635985
H	2.473121	-2.540533	0.951268
H	4.623223	-1.509349	0.246215
N	1.324680	-0.902778	0.482948
C	-1.288739	0.351172	-0.021892
C	-2.422792	1.017501	0.441113
C	-1.447769	-0.935981	-0.542934
H	-2.322891	2.025469	0.826597
H	-0.596234	-1.481704	-0.928958

C	-3.690134	-0.915043	-0.141328
H	-4.631997	-1.452412	-0.197558
C	-3.645605	0.370021	0.393333
H	-4.549521	0.844057	0.753844
N	-2.623117	-1.557522	-0.613722

Coordinates of **A40:**

G_{gas} = -608.481409 Hartree

C	-1.044603	-0.611463	-0.041602
O	-1.283384	-1.637082	-0.635368
C	-2.148407	0.362941	0.252727
C	-1.960885	1.736627	0.376526
C	-3.081009	2.540289	0.568499
H	-0.976180	2.178261	0.325723
H	-2.968040	3.616082	0.657876
C	0.369043	-0.329628	0.405044
C	1.802646	0.737495	1.819470
C	2.906333	0.089460	1.272419
H	1.925787	1.444342	2.633704
H	3.899003	0.291584	1.654445
N	0.556163	0.543852	1.395073
C	-3.447094	-0.135368	0.328500
H	-3.627318	-1.197096	0.216812
C	-4.491236	0.754287	0.543926
H	-5.511873	0.393416	0.620637
C	1.405039	-1.036482	-0.204509
H	1.173766	-1.739994	-0.993577
C	2.700987	-0.813446	0.236356
H	3.535176	-1.337015	-0.215146
N	-4.323861	2.072116	0.661097

Coordinates of **A41:**

G_{gas} = -837.454977 Hartree

C	-0.545945	-0.470169	1.043524
O	-0.575296	-0.602172	2.242736
C	0.745281	-0.614149	0.277638
C	1.596393	-1.715905	0.477517
C	2.801662	-1.737456	-0.217773
C	2.191358	0.363790	-1.197727
C	3.101340	-0.673530	-1.063154
H	3.496503	-2.559806	-0.111394
H	2.393350	1.204875	-1.851405
H	4.032594	-0.659023	-1.615605
N	1.028980	0.383668	-0.544869

C	-1.760634	-0.042177	0.286274
C	-2.693380	0.760956	0.919981
C	-2.035003	-0.422046	-1.044191
C	-3.958516	0.795995	-1.090756
H	-4.806657	1.124274	-1.683189
C	-3.818049	1.199420	0.226024
H	-4.555354	1.841540	0.688205
O	1.163036	-2.688070	1.302007
O	-1.188843	-1.282343	-1.628992
H	-2.513565	1.043218	1.951165
N	-3.094005	-0.009907	-1.716675
C	-1.438004	-1.620719	-2.989238
H	-1.431571	-0.726735	-3.613900
H	-0.628041	-2.289761	-3.270246
H	-2.402446	-2.119118	-3.092931
C	2.014408	-3.794470	1.521274
H	1.489865	-4.439933	2.221048
H	2.201484	-4.339323	0.590636
H	2.965195	-3.477391	1.960275

Coordinates of **A42:**

G_{gas} = -837.464920 Hartree

C	1.519575	0.193429	-0.798756
O	1.699060	-0.420889	-1.830819
C	0.180501	0.836942	-0.563386
C	-0.901999	0.399585	-1.318217
C	-2.139765	1.008917	-1.154947
C	-2.238005	2.053611	-0.240944
C	-1.087958	2.430433	0.470569
C	2.614468	0.307661	0.202898
C	4.965944	0.105626	0.661990
H	5.996455	-0.061283	0.379543
H	-1.159542	3.247992	1.181080
H	-2.992260	0.674159	-1.730907
C	2.425728	0.552692	1.557749
H	1.435858	0.728513	1.956599
N	0.081902	1.838953	0.319904
C	4.650133	0.372615	2.006132
O	5.675427	0.410675	2.868089
C	5.368640	0.679299	4.232981
H	4.704605	-0.086184	4.636031

H	6.325097	0.668260	4.749780
H	4.887347	1.652634	4.334716
C	3.928748	0.064407	-0.234984
H	4.097989	-0.152904	-1.282969
N	3.422948	0.577163	2.449471
H	-0.758500	-0.405616	-2.027050
O	-3.358031	2.752466	0.031431
C	-4.536356	2.405550	-0.672275
H	-4.827075	1.371824	-0.463466
H	-5.309635	3.080086	-0.314023
H	-4.402930	2.540733	-1.749584

Coordinates of **A43:**

G_{gas} = -856.860057 Hartree

C	1.466563	0.241604	-0.756448
O	1.629918	-0.349048	-1.806375
C	0.137754	0.873211	-0.474813
C	-0.960943	0.489735	-1.244644
C	-2.184950	1.091975	-1.033172
C	-2.299932	2.100983	-0.057523
C	-1.123345	2.406511	0.668600
C	2.588238	0.336572	0.221387
C	4.955462	0.160149	0.607429
H	5.980159	0.023701	0.289558
H	-1.136869	3.163476	1.443576
H	-3.040610	0.785747	-1.619426
C	2.434184	0.522580	1.589659
H	1.452670	0.663791	2.021334
N	0.041640	1.814755	0.466591
C	4.673516	0.366347	1.968968
O	5.723235	0.388281	2.804210
C	5.448978	0.592011	4.186341
H	4.809850	-0.202431	4.573634
H	6.419063	0.577058	4.677257
H	4.953159	1.550684	4.343597
C	3.893274	0.135543	-0.261386
H	4.035936	-0.037459	-1.321539
N	3.456383	0.529619	2.454122
H	-0.832869	-0.276439	-1.998501

N	-3.479558	2.756192	0.180544
C	-4.689335	2.296531	-0.473730
H	-5.518801	2.932398	-0.172328
H	-4.594319	2.363793	-1.560847
H	-4.931904	1.259572	-0.209762
C	-3.579307	3.663972	1.308362
H	-4.582720	4.082974	1.336472
H	-3.386259	3.158328	2.262343
H	-2.873528	4.492434	1.206704

Coordinates of **A44:**

G_{gas} = -762.264681 Hartree

C	1.492568	0.255720	-0.782262
O	1.663924	-0.299977	-1.847283
C	0.136980	0.851562	-0.490562
C	-0.957353	0.385156	-1.220654
C	-2.195413	0.960398	-0.992983
C	-2.315975	1.992028	-0.061502
C	-1.152787	2.382017	0.602850
C	2.602062	0.351109	0.203450
C	4.963181	0.170178	0.618083
H	5.991427	0.028985	0.314203
H	-1.193083	3.185190	1.334071
H	-3.069271	0.617392	-1.537814
C	2.431358	0.548186	1.568573
H	1.445424	0.697206	1.987806
N	0.042494	1.830667	0.407285
C	4.665047	0.386634	1.975541
O	5.703191	0.410346	2.821875
C	5.415104	0.626547	4.200230
H	4.768964	-0.162524	4.586491
H	6.380044	0.611696	4.700862
H	4.921707	1.588471	4.343677
C	3.912670	0.143757	-0.263502
H	4.068113	-0.034410	-1.320975
N	3.442281	0.558062	2.444822
H	-0.811119	-0.403629	-1.946944
C	-3.635763	2.655815	0.214859
H	-4.035758	3.115076	-0.691951

H	-3.534491	3.432649	0.973202
H	-4.369549	1.927931	0.568290

Coordinates of **A45:**

G_{gas} = -722.981386 Hartree

C	1.487467	0.236773	-0.804276
O	1.663048	-0.329147	-1.862648
C	0.126602	0.833548	-0.526246
C	-0.963917	0.331326	-1.236264
C	-2.208703	0.904575	-1.024524
C	-2.312423	1.962088	-0.128906
C	-1.162090	2.395600	0.522633
C	2.589627	0.344011	0.186980
C	4.945812	0.155359	0.624222
H	5.975542	0.001450	0.331804
H	-1.204068	3.222787	1.224015
H	-3.081554	0.537852	-1.551481
C	2.409140	0.568674	1.546624
H	1.420968	0.732173	1.955160
N	0.035572	1.843556	0.339621
C	4.638111	0.400262	1.974920
O	5.669134	0.433778	2.828723
C	5.371973	0.678945	4.200546
H	4.716663	-0.097474	4.596693
H	6.332856	0.666426	4.708889
H	4.885148	1.647313	4.321146
C	3.902603	0.118769	-0.265404
H	4.065552	-0.080439	-1.317965
N	3.412647	0.588996	2.430773
H	-0.810755	-0.480390	-1.935200
H	-3.261538	2.445856	0.064319

Coordinates of **A46:**

G_{gas} = -3320.593471 Hartree

C	-0.532955	-0.569747	-1.024067
O	-0.763788	-0.792883	-2.187130
C	0.843474	-0.670428	-0.437742
C	1.837182	-1.467876	-1.026522
C	3.084468	-1.444298	-0.400699

C	2.233196	0.106134	1.203421
C	3.294926	-0.651322	0.715699
H	3.889321	-2.052461	-0.799514
H	2.353087	0.740808	2.074994
H	4.259402	-0.620600	1.206919
N	1.031003	0.087204	0.645736
C	-1.647700	-0.101364	-0.122324
C	-2.568548	0.811022	-0.619324
C	-1.850806	-0.568288	1.169285
C	-3.601347	1.220087	0.218880
H	-2.471926	1.188015	-1.628645
N	-3.782785	0.785569	1.445686
C	-2.919555	-0.112107	1.919042
H	-3.076416	-0.485861	2.924722
F	-1.047933	-1.499217	1.692029
Br	-4.856417	2.492846	-0.436740
C	1.616110	-2.316320	-2.248509
H	0.696187	-2.897024	-2.173052
H	1.518528	-1.694237	-3.139446
H	2.456552	-2.998066	-2.382474

Coordinates of **A47:**

G_{gas} = -879.187313 Hartree

C	1.094644	-0.084853	-0.374002
O	1.208020	-0.461297	-1.523956
C	3.506327	0.545148	2.213490
C	4.411244	0.154620	1.218422
C	5.780940	0.137855	1.513856
C	6.181386	0.510654	2.784196
C	5.246432	0.897979	3.761062
C	3.886825	0.922805	3.493613
C	2.321528	0.075170	0.438750
C	3.604927	-0.147287	0.067005
H	6.504964	-0.159477	0.765257
H	7.234427	0.505950	3.036887
H	5.597316	1.183651	4.745105
H	3.156978	1.218652	4.235801
H	3.913206	-0.483594	-0.909721
C	-2.468524	0.444778	0.383637
C	-1.479109	0.073875	-0.526986
C	-1.852532	-0.309942	-1.819465
C	-3.203400	-0.301714	-2.131824
C	-4.174883	0.077152	-1.191670
C	-3.823146	0.460878	0.094478

C	-0.570916	0.631388	1.444724
C	-0.227051	0.204696	0.197295
H	-1.101231	-0.600874	-2.540491
H	-3.520238	-0.594520	-3.125373
H	-5.220171	0.070026	-1.475281
H	-4.554793	0.756459	0.834830
H	0.019600	0.861606	2.314139
O	2.233579	0.497349	1.739947
O	-1.902483	0.782864	1.582917

Coordinates of **A48:**

G_{gas} = -1525.148564 Hartree

C	0.992578	0.469856	-0.565859
O	1.123888	-0.096484	-1.631018
C	4.504553	0.891422	1.118403
C	3.553973	0.994543	2.154210
C	3.993414	1.183225	3.475721
C	5.345641	1.273464	3.731933
C	6.281613	1.176324	2.686379
C	5.875297	0.985967	1.379367
C	2.168591	0.707735	0.305793
C	2.214253	0.883660	1.654646
H	3.269528	1.257827	4.279170
H	5.694730	1.420505	4.746514
H	7.339125	1.250235	2.909482
H	6.598841	0.908507	0.577058
H	1.333981	0.898004	2.283503
C	-2.690714	1.002546	0.040298
C	-1.584824	0.321860	-0.499277
C	-1.807623	-0.814007	-1.294016
C	-3.104713	-1.235003	-1.516538
C	-4.196228	-0.545935	-0.964680
C	-4.001846	0.577567	-0.182625
C	-0.530153	2.038341	0.677528
C	-0.335157	0.937926	-0.098824
H	-0.965826	-1.333191	-1.732288
H	-3.284132	-2.109021	-2.130619
H	-5.203570	-0.895635	-1.155484
H	-4.840439	1.114049	0.244164
H	0.229218	2.700579	1.066360
S	-2.192957	2.387852	0.979533
S	3.751682	0.636772	-0.427854

Coordinates of **C47_1**

Gsol = -880.471061 Hartree

C	0.288674	1.652023	0.612673
O	0.092126	2.982554	0.162556
C	-2.994499	0.275457	0.340153
C	-2.402973	-0.645306	-0.527970
C	-3.200826	-1.629294	-1.114661
C	-4.550018	-1.648809	-0.808757
C	-5.112518	-0.709894	0.066499
C	-4.340484	0.275730	0.660231
C	-0.868411	0.783133	0.231121
C	-1.007248	-0.283449	-0.579680
H	-2.774105	-2.357107	-1.792280
H	-5.187237	-2.402386	-1.252320
H	-6.171455	-0.755112	0.283631
H	-4.760219	1.006703	1.337402
H	-0.218849	-0.756053	-1.141465
C	3.393078	-0.084178	-0.457082
C	2.235018	-0.097273	0.323071
C	1.978149	-1.198815	1.142331
C	2.892424	-2.237173	1.146752
C	4.046340	-2.195360	0.351808
C	4.318662	-1.113331	-0.469173
C	2.347514	1.785697	-0.861220
C	1.567062	1.150064	0.033327
H	1.085036	-1.241839	1.752786
H	2.715662	-3.101272	1.773421
H	4.738633	-3.026362	0.378846
H	5.204013	-1.067905	-1.088157
H	2.245642	2.741360	-1.344928
O	-2.057439	1.144910	0.808617
O	3.461131	1.065459	-1.179121
H	0.364798	1.641164	1.708779
H	-0.748138	3.300106	0.511330

Coordinates of **C47_2**

G_{sol} = -880.470701 Hartree

C	0.316555	1.223035	0.374313
O	0.611922	2.433541	-0.295513
C	-2.880098	-0.393203	0.527841

C	-3.268247	0.604460	-0.369263
C	-4.586892	0.623591	-0.826566
C	-5.455094	-0.352377	-0.368886
C	-5.033077	-1.339767	0.530995
C	-3.728865	-1.378327	0.998850
C	-1.119143	0.849744	0.165937
C	-2.088218	1.407116	-0.585767
H	-4.921780	1.382896	-1.521090
H	-6.481775	-0.355826	-0.710480
H	-5.738892	-2.087121	0.868356
H	-3.387696	-2.132700	1.694279
H	-1.985225	2.284711	-1.200565
C	3.144533	-1.053810	-0.412638
C	2.693722	0.146722	0.136232
C	3.618840	1.038878	0.680600
C	4.956124	0.684906	0.657274
C	5.378975	-0.530363	0.100656
C	4.476799	-1.426903	-0.448545
C	0.977281	-1.036054	-0.647723
C	1.257789	0.130391	-0.031321
H	3.296976	1.982356	1.101953
H	5.694043	1.357772	1.073634
H	6.433294	-0.773226	0.098341
H	4.789048	-2.366807	-0.882202
H	0.045278	-1.478724	-0.958148
O	-1.565167	-0.243056	0.854095
O	2.093383	-1.775695	-0.890727
H	0.464206	1.437520	1.437778
H	0.705521	2.242599	-1.236623

Coordinates of **C48_1**

G_{sol} = -1526.427126 Hartree

C	0.416877	-1.414870	-1.266671
O	0.375674	-2.831158	-1.242737
C	-2.745287	-0.126020	0.726387
C	-2.951898	0.184348	-0.629836
C	-4.139813	0.822252	-1.010167
C	-5.079585	1.131776	-0.050099
C	-4.857801	0.815571	1.298236

C	-3.695557	0.187084	1.698158
C	-0.857917	-0.822728	-0.727158
C	-1.842494	-0.232632	-1.441353
H	-4.310892	1.065871	-2.051314
H	-5.999493	1.624313	-0.336360
H	-5.607631	1.068311	2.036347
H	-3.524740	-0.055484	2.738799
H	-1.790724	-0.088711	-2.512900
C	3.200486	0.529155	0.405501
C	2.066980	0.391907	-0.414506
C	1.536466	1.530362	-1.036687
C	2.137425	2.755213	-0.834074
C	3.268036	2.873614	-0.013701
C	3.807518	1.767399	0.611419
C	2.387861	-1.804104	0.260212
C	1.618946	-0.977027	-0.480172
H	0.658446	1.447563	-1.665248
H	1.731687	3.637544	-1.311196
H	3.723193	3.844257	0.133047
H	4.679758	1.857343	1.245586
H	2.260432	-2.868567	0.372899
H	0.519898	-1.057620	-2.299535
H	-0.453013	-3.114650	-1.643765
S	-1.214589	-0.901428	0.972037
S	3.682478	-0.997241	1.069376

Coordinates of **C48_2**

Gsol = -1526.426092 Hartree

C	0.399715	-1.737993	-0.629091
O	0.257785	-3.091612	-0.236131
C	-3.197207	-0.128548	-0.294289
C	-2.387518	0.472295	0.686046
C	-2.948328	1.433569	1.537129
C	-4.278397	1.768298	1.397529
C	-5.072040	1.158060	0.415127
C	-4.543204	0.208408	-0.435955
C	-0.852565	-0.958249	-0.316806
C	-1.041889	-0.028288	0.645974
H	-2.334744	1.904305	2.294994

H	-4.718352	2.509860	2.051176
H	-6.113860	1.435354	0.322080
H	-5.155866	-0.261846	-1.193831
H	-0.253820	0.311618	1.304665
C	3.272832	0.567056	0.267354
C	2.106698	0.207790	-0.429706
C	1.581531	1.094479	-1.380115
C	2.219555	2.294715	-1.611358
C	3.381074	2.638522	-0.904474
C	3.916016	1.783235	0.037382
C	2.403702	-1.658001	0.912172
C	1.621806	-1.091552	-0.031745
H	0.678638	0.839497	-1.921958
H	1.819780	2.982705	-2.344496
H	3.864373	3.586824	-1.099422
H	4.812493	2.047209	0.582961
H	2.277486	-2.632170	1.357590
H	0.529969	-1.770594	-1.715543
H	-0.104252	-3.110154	0.657838
S	-2.298965	-1.277932	-1.228976
S	3.745540	-0.675349	1.379097

Coordinates of **A49:**

G_{gas} = -690.865369 Hartree

C	0.424226	0.581323	-0.036503
C	1.500879	1.315668	0.455594
C	0.670278	-0.669516	-0.617610
C	2.796152	0.817054	0.406513
H	1.308207	2.293403	0.881353
C	1.956285	-1.169915	-0.692422
H	-0.147963	-1.242733	-1.037288
C	3.025743	-0.434057	-0.171626
C	-0.932988	1.201807	0.011255
O	-1.060891	2.407545	0.029656
C	-2.147629	0.320338	0.039490
C	-3.320448	0.800968	-0.545883
C	-2.156653	-0.914665	0.690011
C	-4.481784	0.041921	-0.507788
H	-3.301595	1.773756	-1.022778
C	-3.327265	-1.663738	0.747033
H	-1.256679	-1.278372	1.172255

C	-4.486087	-1.191434	0.139817
H	-5.386191	0.412233	-0.975441
H	-3.334570	-2.614547	1.266284
H	-5.394809	-1.780708	0.176511
H	2.166479	-2.127430	-1.152149
H	3.608993	1.404948	0.810088
O	4.244056	-1.011717	-0.277220
C	5.361550	-0.302417	0.220923
H	5.266300	-0.122352	1.295764
H	6.225606	-0.935397	0.034347
H	5.487351	0.650557	-0.301260

Coordinates of **A50:**

G_{gas} = -615.663296 Hartree

C	0.833854	0.566642	-0.007550
C	1.902684	1.291435	0.526343
C	1.081745	-0.671090	-0.600576
C	3.186128	0.771980	0.492749
H	1.704683	2.264821	0.959467
C	2.376611	-1.176469	-0.649546
H	0.269218	-1.230404	-1.049611
C	3.443377	-0.471417	-0.094539
C	-0.524507	1.195841	0.007825
O	-0.641741	2.401788	0.002668
C	-1.744081	0.322051	0.029128
C	-2.910455	0.810842	-0.562862
C	-1.764283	-0.914094	0.677222
C	-4.076534	0.059101	-0.532809
H	-2.882268	1.783874	-1.038754
C	-2.939852	-1.656005	0.725731
H	-0.869327	-1.284599	1.163379
C	-4.092083	-1.175409	0.112674
H	-4.975902	0.435605	-1.005153
H	-2.955985	-2.607869	1.242802
H	-5.004646	-1.759086	0.142904
H	2.560567	-2.131677	-1.129433
H	4.007709	1.337917	0.919529
C	4.839850	-1.034112	-0.108928
H	5.572589	-0.257885	-0.336477
H	5.093992	-1.451416	0.869286
H	4.938568	-1.829365	-0.848455

Coordinates of Benzophenone:

G_{gas} = -576.380319 Hartree

C	-1.295151	0.354691	0.030094
C	-2.420209	0.959996	-0.533649
C	-1.419569	-0.885126	0.659626
C	-3.650717	0.319667	-0.493781
H	-2.308977	1.933579	-0.996021
C	-2.658339	-1.514765	0.718318
H	-0.555259	-1.346058	1.123390
C	-3.770665	-0.918456	0.133532
C	0.000000	1.110202	0.000035
O	0.000023	2.321240	0.000052
C	1.295141	0.354667	-0.030069
C	2.420251	0.960001	0.533574
C	1.419524	-0.885177	-0.659520
C	3.650754	0.319690	0.493652
H	2.309055	1.933611	0.995897
C	2.658300	-1.514836	-0.718258
H	0.555195	-1.346163	-1.123201
C	3.770653	-0.918483	-0.133601
H	4.518626	0.785956	0.944283
H	2.754855	-2.469481	-1.221184
H	4.733060	-1.415163	-0.171667
H	-2.754961	-2.469378	1.221296
H	-4.518556	0.785907	-0.944502
H	-4.733065	-1.415144	0.171576

Coordinates of **A51:**

G_{gas} = -1035.993004 Hartree

C	0.440051	0.670302	-0.004715
C	1.463794	1.448423	0.539170
C	0.749384	-0.556974	-0.592456
C	2.775160	0.999147	0.526927
H	1.214478	2.411134	0.969086
C	2.062768	-1.009045	-0.630183
H	-0.032100	-1.155737	-1.044981
C	3.060022	-0.229497	-0.059461
C	-0.949945	1.233372	-0.001478
O	-1.119127	2.432144	-0.020982
C	-2.123321	0.301459	0.024554
C	-3.311738	0.727673	-0.572012
C	-2.081307	-0.930258	0.680527
C	-4.438137	-0.082183	-0.538403
H	-3.332206	1.697683	-1.054408
C	-3.217749	-1.730265	0.732180
H	-1.170625	-1.252355	1.172078

C	-4.391724	-1.311926	0.114549
H	-5.354601	0.245188	-1.014319
H	-3.186653	-2.678354	1.255309
H	-5.273428	-1.941059	0.146958
H	2.314987	-1.952608	-1.096284
Cl	4.704480	-0.800083	-0.084660
H	3.573459	1.589005	0.958423

Coordinates of **A52:**

G_{gas} = -3149.966150 Hartree

C	-0.294093	0.777341	0.003973
C	0.681814	1.612591	0.550423
C	0.085304	-0.433645	-0.575928
C	2.016697	1.236559	0.547704
H	0.377586	2.562079	0.974533
C	1.422315	-0.813016	-0.605373
H	-0.659461	-1.076944	-1.029507
C	2.371917	0.023370	-0.033090
C	-1.712806	1.264523	-0.005936
O	-1.945482	2.452283	-0.037409
C	-2.834302	0.271180	0.020855
C	-4.039986	0.628355	-0.586421
C	-2.730458	-0.951354	0.687214
C	-5.121573	-0.240327	-0.552807
H	-4.109203	1.591721	-1.077492
C	-3.822938	-1.810402	0.739065
H	-1.806906	-1.220520	1.186551
C	-5.013709	-1.460400	0.111074
H	-6.051065	0.033637	-1.037092
H	-3.744627	-2.751076	1.270543
H	-5.860742	-2.135476	0.143790
H	1.724007	-1.743777	-1.067403
H	2.774954	1.874377	0.982777
Br	4.196292	-0.496598	-0.052913

Coordinates of **A53:**

G_{gas} = -804.206595 Hartree

C	-0.290189	0.729463	-0.080258
C	0.707425	1.556330	0.439116
C	0.050504	-0.504391	-0.638682
C	2.031515	1.145202	0.428930
H	0.425698	2.520681	0.844246
C	1.377935	-0.908718	-0.669609
H	-0.717737	-1.136600	-1.067722

C	2.366183	-0.090301	-0.127049
C	-1.700766	1.247292	-0.085551
O	-1.906672	2.436273	-0.180519
C	-2.841658	0.282837	0.024488
C	-4.064046	0.647863	-0.543778
C	-2.736252	-0.919183	0.727075
C	-5.162244	-0.192916	-0.434413
H	-4.132290	1.595258	-1.065082
C	-3.844285	-1.749915	0.853953
H	-1.797822	-1.194656	1.194030
C	-5.052937	-1.392593	0.265635
H	-6.105310	0.086527	-0.888383
H	-3.763727	-2.674795	1.412107
H	-5.912496	-2.046140	0.356936
H	1.667034	-1.855101	-1.109950
H	2.809148	1.774450	0.841544
C	3.775924	-0.581086	-0.173022
O	4.109611	-1.637270	-0.641943
O	4.639481	0.287500	0.369046
C	6.008210	-0.122889	0.353998
H	6.344191	-0.276445	-0.671521
H	6.562795	0.684353	0.824288
H	6.129234	-1.051536	0.911835

Coordinates of **A54:**

G_{gas} = -913.433528 Hartree

C	-0.226674	0.781769	-0.017401
C	0.745027	1.597689	0.563554
C	0.147890	-0.409106	-0.640329
C	2.077286	1.213980	0.551898
H	0.438694	2.533105	1.015696
C	1.484426	-0.787002	-0.674418
H	-0.599743	-1.030092	-1.119066
C	2.439268	0.022424	-0.070285
C	-1.648215	1.272266	-0.018890
O	-1.873079	2.460555	-0.053706
C	-2.769461	0.281184	0.019939
C	-3.990204	0.651600	-0.548492
C	-2.649230	-0.954288	0.659426
C	-5.071684	-0.216263	-0.502345
H	-4.070460	1.623901	-1.019821
C	-3.741245	-1.812926	0.723656
H	-1.713251	-1.235006	1.128330
C	-4.947736	-1.449070	0.134871

H	-6.013358	0.067717	-0.956280
H	-3.650231	-2.764019	1.234051
H	-5.794647	-2.123750	0.177111
H	1.785410	-1.699724	-1.173537
H	2.835531	1.838323	1.008096
C	3.874500	-0.422364	-0.042412
F	4.721882	0.614137	-0.008789
F	4.134180	-1.172134	1.042393
F	4.190936	-1.165809	-1.111112

Coordinates of **A55:**

G_{gas} = -780.870146 Hartree

C	0.167279	0.715696	-0.049974
C	0.508913	-0.516240	-0.610204
C	1.156827	1.534280	0.497678
C	1.832517	-0.936980	-0.619125
H	-0.254651	-1.139630	-1.059244
C	2.479111	1.118724	0.516111
H	0.873471	2.498245	0.901842
C	2.786349	-0.112708	-0.044497
C	-1.241509	1.246274	-0.087039
O	-1.425877	2.436370	-0.197807
C	-2.390639	0.293597	0.007543
C	-3.601868	0.670522	-0.577154
C	-2.304644	-0.909820	0.710816
C	-4.708325	-0.160896	-0.483059
H	-3.654859	1.618551	-1.099022
C	-3.421355	-1.730840	0.821935
H	-1.376470	-1.193996	1.192947
C	-4.618521	-1.361995	0.217421
H	-5.642820	0.126911	-0.949135
H	-3.356828	-2.656369	1.380912
H	-5.484954	-2.007889	0.296592
H	3.265195	1.725047	0.944829
H	2.130445	-1.879375	-1.057721
N	4.197670	-0.563394	-0.036225
O	4.442814	-1.638946	-0.536028
O	5.014685	0.171899	0.471194

Coordinates of **A56:**

G_{gas} = -1250.484196 Hartree

C	0.269512	-0.713939	-0.180752
C	-0.906583	-1.425373	0.068218
C	0.222422	0.668538	-0.317375

C	-2.105090	-0.746434	0.205893
H	-0.864715	-2.505890	0.138039
C	-0.995211	1.334490	-0.194642
H	1.121955	1.229790	-0.545062
C	-2.161894	0.640124	0.077562
C	1.538351	-1.500477	-0.380338
O	1.475331	-2.620104	-0.831963
C	2.850885	-0.880506	-0.024695
C	3.984588	-1.310153	-0.718454
C	2.980317	0.049873	1.008906
C	5.231644	-0.792477	-0.400506
H	3.866783	-2.049558	-1.501588
C	4.234557	0.550776	1.339203
H	2.110060	0.363847	1.573801
C	5.356926	0.138041	0.628898
H	6.107784	-1.116100	-0.949063
H	4.334698	1.261622	2.150211
H	6.332143	0.537553	0.880760
H	-3.105468	1.163493	0.173461
C	-3.367508	-1.492527	0.542115
C	-1.006441	2.831581	-0.334269
F	-4.426174	-0.982742	-0.101327
F	-3.285302	-2.787868	0.227017
F	-3.639261	-1.419477	1.855009
F	-2.246455	3.325680	-0.392504
F	-0.390226	3.420375	0.703595
F	-0.359595	3.225637	-1.439581

Coordinates of **A57:**

G_{gas} = -1150.478031 Hartree

C	1.269082	0.695542	-0.036155
C	2.422232	1.273765	0.490083
C	1.363682	-0.559822	-0.651382
C	3.644269	0.616185	0.441841
H	2.348461	2.255633	0.942633
C	2.576830	-1.216977	-0.725800
H	0.487094	-1.012294	-1.100040
C	3.722681	-0.637530	-0.170324
C	0.001920	1.480907	0.014675
O	0.022881	2.692368	0.059251
C	-1.314413	0.759586	0.014188
C	-2.415119	1.403688	-0.553314
C	-1.486985	-0.483360	0.624122
C	-3.666301	0.806278	-0.543229

H	-2.274176	2.381075	-0.999063
C	-2.740195	-1.082980	0.661284
H	-0.646289	-0.978200	1.096030
C	-3.814300	-0.434885	0.066259
H	-4.522487	1.291526	-0.993686
H	-2.887213	-2.039563	1.145677
H	2.671641	-2.179604	-1.212263
H	4.518579	1.084322	0.872635
O	4.858789	-1.361571	-0.278570
C	6.050270	-0.814303	0.252359
H	5.962644	-0.652880	1.330729
H	6.829178	-1.547248	0.057412
H	6.304001	0.128362	-0.241132
Cl	-5.383924	-1.189063	0.091058

Coordinates of **A58:**

G_{gas} = -895.355479 Hartree

C	-1.545892	0.716825	-0.005864
C	-2.724477	1.256807	-0.518109
C	-1.594990	-0.529427	0.633900
C	-3.927509	0.570563	-0.430383
H	-2.684731	2.230732	-0.991558
C	-2.788742	-1.214909	0.746180
H	-0.698938	-0.953405	1.071814
C	-3.960943	-0.673804	0.205999
C	-0.303130	1.529930	-0.100901
O	-0.343381	2.734930	-0.217120
C	1.037459	0.842926	-0.059762
C	2.101418	1.525386	0.532520
C	1.251195	-0.398921	-0.660022
C	3.368563	0.963471	0.556944
H	1.920066	2.501067	0.966172
C	2.519397	-0.965144	-0.663963
H	0.431755	-0.915671	-1.144808
C	3.547914	-0.274156	-0.043899
H	4.208579	1.462443	1.020335
H	2.718766	-1.918863	-1.132863
H	-2.848724	-2.170980	1.250604
H	-4.822527	1.008417	-0.850162
O	-5.074104	-1.422774	0.351888
C	-6.291994	-0.917514	-0.162835
H	-6.231655	-0.775015	-1.245577
H	-7.045717	-1.667450	0.063265
H	-6.559940	0.027006	0.319128

N	4.899089	-0.880565	-0.029573
O	5.034018	-1.956470	-0.569346
O	5.782197	-0.262750	0.522307

Coordinates of **A59:**

G_{gas} = -1099.840423 Hartree

C	1.802245	-0.833772	-0.218770
C	2.936363	-1.291215	-0.889183
C	1.956111	0.109197	0.807768
C	4.198252	-0.807880	-0.576960
H	2.813870	-2.035932	-1.666707
C	3.208029	0.584694	1.142117
H	1.096241	0.452686	1.371097
C	4.336695	0.136724	0.445227
C	0.491948	-1.427147	-0.583573
O	0.400942	-2.513176	-1.109274
C	-0.776651	-0.654850	-0.306754
C	-1.930151	-1.385131	-0.021710
C	-0.842440	0.731635	-0.416844
C	-3.118494	-0.705779	0.178339
H	-1.894960	-2.465827	0.035431
C	-2.065371	1.358904	-0.228956
H	0.028139	1.325179	-0.665532
C	-3.225263	0.671891	0.078511
H	3.347623	1.302470	1.940316
H	5.057717	-1.169829	-1.123895
O	5.512630	0.668069	0.832594
C	6.690584	0.246637	0.168619
H	6.647978	0.489757	-0.896741
H	7.506618	0.793444	0.633797
H	6.849726	-0.827641	0.297080
H	-4.166648	1.182282	0.229110
N	-4.339874	-1.481101	0.504993
O	-5.368108	-0.859021	0.641991
O	-4.220640	-2.679170	0.612957
N	-2.134200	2.833898	-0.357926
O	-1.104553	3.414173	-0.617898
O	-3.212638	3.354172	-0.191672

Coordinates of **A60:**

G_{gas} = -1495.600923 Hartree

C	-0.029458	0.389826	-0.228350
C	1.092529	1.129611	0.137104
C	0.117507	-0.942798	-0.613567

C	2.347509	0.540106	0.149608
H	0.979593	2.172033	0.408673
C	1.374602	-1.528630	-0.624221
H	-0.743741	-1.519197	-0.928342
C	2.490214	-0.796044	-0.233448
C	-1.350638	1.103191	-0.266574
O	-1.388365	2.291947	-0.487594
C	-2.609982	0.328123	-0.033284
C	-3.781109	0.784095	-0.642114
C	-2.657069	-0.776553	0.819439
C	-4.981879	0.123944	-0.423932
H	-3.729163	1.658026	-1.280551
C	-3.865842	-1.422520	1.054152
H	-1.757087	-1.115338	1.319566
C	-5.024800	-0.979690	0.425183
H	-5.886242	0.471173	-0.908726
H	-3.902860	-2.269263	1.728725
H	-5.964114	-1.490387	0.601535
H	1.506339	-2.556938	-0.934834
Cl	4.040580	-1.563294	-0.238685
Cl	3.716754	1.480844	0.637199

Coordinates of **A61:**

G_{gas} = -1240.469954 Hartree

C	-0.109423	1.117001	-0.046815
C	0.385149	-0.156677	-0.298489
C	0.780794	2.139377	0.288581
C	1.745455	-0.401419	-0.177948
H	-0.261272	-0.968175	-0.609484
C	2.137572	1.889435	0.397979
H	0.390448	3.137030	0.449975
C	2.638944	0.608392	0.171331
N	2.184167	-1.787833	-0.431597
O	1.638734	-2.370523	-1.343193
O	3.021901	-2.254449	0.302865
C	-1.563271	1.465620	-0.211748
O	-1.869252	2.590520	-0.534167
C	-2.603573	0.417049	0.016519
C	-3.802749	0.522815	-0.691871
C	-2.437371	-0.607831	0.950529
C	-4.814069	-0.404785	-0.489174
H	-3.921796	1.337895	-1.395663
C	-3.461210	-1.523220	1.167007
H	-1.523297	-0.676511	1.528979

C	-4.643511	-1.428223	0.440595
H	-5.737446	-0.329813	-1.050432
H	-3.335690	-2.308957	1.901800
H	-5.435873	-2.149320	0.602511
H	2.831116	2.681220	0.649548
Cl	4.344538	0.367169	0.261230

Coordinates of **A62:**

G_{gas} = -985.354617 Hartree

C	-0.023166	-0.617457	-0.248168
C	-1.154198	-1.405370	-0.034096
C	-0.147275	0.766483	-0.339579
C	-2.381539	-0.784960	0.113068
H	-1.070807	-2.484057	0.010456
C	-1.406242	1.333213	-0.206338
H	0.706133	1.404427	-0.531267
C	-2.547005	0.588065	0.028681
N	-3.580976	-1.620951	0.363724
O	-3.411712	-2.814010	0.457906
O	-4.642065	-1.048342	0.457743
N	-1.536641	2.805988	-0.313327
O	-0.521722	3.437594	-0.501109
O	-2.646218	3.272339	-0.202420
H	-3.517852	1.051884	0.137844
C	1.292191	-1.325586	-0.465628
O	1.285304	-2.423798	-0.968566
C	2.563517	-0.653737	-0.067141
C	3.729366	-1.010529	-0.749593
C	2.628100	0.254962	0.991657
C	4.943255	-0.441960	-0.393840
H	3.662039	-1.733564	-1.553639
C	3.849838	0.807263	1.358689
H	1.734774	0.512131	1.549133
C	5.003618	0.466938	0.660535
H	5.844175	-0.708833	-0.932553
H	3.900207	1.501723	2.188144
H	5.953203	0.906813	0.941188

Coordinates of **A63:**

G_{gas} = -577.726696 Hartree

C	3.127217	-1.982132	0.535021
C	1.930809	-2.210050	-0.010833
H	3.900504	-2.741377	0.518717
H	3.361287	-1.048913	1.033083

C	0.865443	-1.170228	-0.010194
C	1.210328	0.192469	-0.070144
C	-0.469808	-1.538397	0.043588
C	0.209330	1.151351	-0.050488
H	2.250029	0.469311	-0.165717
C	-1.470219	-0.558006	0.067982
H	-0.778353	-2.574402	0.085372
C	-1.141675	0.786904	0.023729
O	0.433403	2.490365	-0.113537
O	-2.743261	-1.024447	0.136518
H	-1.879438	1.576581	0.030591
C	1.772976	2.929492	-0.191839
C	-3.788772	-0.075780	0.165533
H	2.258935	2.556257	-1.098461
H	1.732358	4.015892	-0.223336
H	2.344577	2.610883	0.685336
H	-4.712541	-0.646683	0.224488
H	-3.705732	0.575813	1.040826
H	-3.793814	0.534039	-0.743142
C	1.597462	-3.540740	-0.634200
H	0.853792	-4.077044	-0.038675
H	1.178464	-3.408550	-1.634573
H	2.486718	-4.167339	-0.701045

Coordinates of **A64:**

G_{gas} = -463.242652 Hartree

C	3.783521	0.277890	0.428329
C	2.879540	-0.629351	0.050206
H	4.840565	0.039290	0.439520
H	3.499404	1.270958	0.754960
C	1.428727	-0.314935	-0.002246
C	0.971915	0.991327	-0.241086
C	0.466430	-1.306853	0.175134
C	-0.375464	1.290551	-0.270111
H	1.690182	1.779829	-0.432108
C	-0.898895	-1.023315	0.151882
H	0.772406	-2.331510	0.349090
C	-1.324345	0.283519	-0.067199
H	-0.726673	2.296785	-0.463223
H	-1.607531	-1.826041	0.304207
O	-2.624207	0.673284	-0.114447
C	-3.612752	-0.316838	0.073010
H	-3.523779	-0.782470	1.059536
H	-4.569926	0.194127	-0.000859

H	-3.551069	-1.088355	-0.700951
C	3.297674	-2.027836	-0.327123
H	2.956527	-2.753688	0.416489
H	2.867474	-2.319102	-1.288642
H	4.383257	-2.101261	-0.388657

Coordinates of **A65:**

G_{gas} = -388.042041 Hartree

C	-3.327875	-0.463929	0.392448
C	-2.493663	0.497116	-0.010748
H	-4.401279	-0.315558	0.371333
H	-2.967701	-1.412400	0.772592
C	-1.020393	0.298089	-0.013028
C	-0.458927	-0.966721	-0.228322
C	-0.146201	1.369283	0.185718
C	0.914961	-1.152245	-0.213938
H	-1.109177	-1.808303	-0.437003
C	1.231786	1.178472	0.203223
H	-0.539158	2.367210	0.340000
C	1.787104	-0.083782	0.009390
H	1.322132	-2.142297	-0.393712
H	1.885418	2.028787	0.368030
C	3.277297	-0.300668	0.044502
H	3.815793	0.642979	-0.051243
H	3.578550	-0.764485	0.987784
H	3.597404	-0.960988	-0.763928
C	-3.010858	1.837408	-0.467237
H	-2.751318	2.622764	0.248112
H	-2.575401	2.117022	-1.429789
H	-4.096487	1.819160	-0.561629

Coordinates of **A66:**

G_{gas} = -654.908113 Hartree

C	-5.395554	-0.125129	-0.205965
C	-4.319322	-0.910994	-0.285935
H	-6.394736	-0.541253	-0.260212
H	-5.313679	0.950559	-0.105869
C	-2.943317	-0.359405	-0.181659
C	-2.673042	0.774431	0.593723
C	-1.871519	-0.960216	-0.849367
C	-1.393608	1.300618	0.682064
H	-3.473745	1.234986	1.160116
C	-0.585011	-0.439776	-0.778668
H	-2.038963	-1.841673	-1.456493

C	-0.350241	0.693455	-0.008129
H	-1.180917	2.172684	1.287926
H	0.230259	-0.908463	-1.316426
O	0.881896	1.297345	0.066821
C	2.018431	0.521489	0.068227
C	2.112181	-0.646398	0.820063
C	3.105744	0.991019	-0.657980
C	3.310499	-1.350359	0.830025
H	1.255960	-0.992360	1.386757
C	4.300851	0.280972	-0.633590
H	2.997655	1.906669	-1.225858
C	4.407522	-0.892690	0.105553
H	3.387678	-2.258861	1.415472
H	5.149871	0.646623	-1.198706
H	5.338466	-1.445616	0.120011
C	-4.453570	-2.396825	-0.501873
H	-4.100233	-2.682972	-1.496475
H	-3.859735	-2.953421	0.227487
H	-5.495591	-2.704971	-0.418584

Coordinates of **A67:**

G_{gas} = -808.372294 Hartree

C	-3.714951	-0.509898	0.366187
C	-2.897974	0.456318	-0.058129
H	-4.790772	-0.383136	0.335558
H	-3.338919	-1.440245	0.774824
C	-1.421528	0.282499	-0.044542
C	-0.840344	-0.975695	-0.239085
C	-0.570023	1.373975	0.151686
C	0.535940	-1.148606	-0.209712
H	-1.474642	-1.830352	-0.441452
C	0.810513	1.217640	0.187082
H	-0.982899	2.365243	0.293424
C	1.352400	-0.046429	0.008818
H	0.977304	-2.124287	-0.368172
H	1.461615	2.066839	0.350611
Cl	3.083702	-0.253375	0.042206
C	-3.433980	1.774679	-0.554342
H	-3.198631	2.582951	0.143658
H	-2.993560	2.037882	-1.519193
H	-4.517746	1.733017	-0.660160

Coordinates of **A68:**

G_{gas} = -2922.345626 Hartree

C	-4.381139	-0.556045	0.376332
C	-3.585543	0.427086	-0.049690
H	-5.459376	-0.450081	0.352035
H	-3.984868	-1.480003	0.780279
C	-2.105980	0.281712	-0.043336
C	-1.501550	-0.964197	-0.244975
C	-1.274939	1.388718	0.153067
C	-0.121648	-1.111301	-0.221960
H	-2.119915	-1.830418	-0.447884
C	0.108926	1.259376	0.182066
H	-1.706011	2.371494	0.300162
C	0.674207	0.006106	-0.002561
H	0.333047	-2.079728	-0.385877
H	0.740160	2.123119	0.345838
Br	2.564017	-0.182388	0.026178
C	-4.149259	1.736120	-0.539634
H	-3.925657	2.547086	0.159091
H	-3.719140	2.010065	-1.506157
H	-5.232560	1.673947	-0.639883

Coordinates of **A69:**

G_{gas} = -576.586779 Hartree

C	4.477936	0.337752	0.439077
C	3.632407	-0.576339	-0.041361
H	5.547744	0.164325	0.433716
H	4.130672	1.268854	0.870571
C	2.165064	-0.337657	-0.052331
C	1.645791	0.953808	-0.214719
C	1.265846	-1.400630	0.087943
C	0.279296	1.177745	-0.206750
H	2.323653	1.783600	-0.374035
C	-0.105179	-1.182029	0.099595
H	1.637733	-2.411274	0.202818
C	-0.603988	0.110571	-0.044963
H	-0.125314	2.174222	-0.337186
H	-0.791809	-2.009977	0.219967
C	-2.063722	0.403961	-0.046658
O	-2.537133	1.503224	-0.173861
O	-2.811787	-0.699129	0.111863
C	-4.222842	-0.480884	0.121616
H	-4.672112	-1.460935	0.257527
H	-4.496590	0.185732	0.939620
H	-4.543339	-0.036868	-0.821029
C	4.123932	-1.898375	-0.572741

H	3.840794	-2.719460	0.091543
H	3.693524	-2.109695	-1.554687
H	5.210473	-1.897803	-0.656216

Coordinates of **A70:**

G_{gas} = -553.251662 Hartree

C	-3.965142	-0.517090	0.382635
C	-3.165926	0.446204	-0.079890
H	-5.042705	-0.404673	0.359719
H	-3.573421	-1.428200	0.818841
C	-1.688005	0.289079	-0.060945
C	-1.096730	-0.970990	-0.223956
C	-0.853835	1.400861	0.107775
C	0.279252	-1.127755	-0.188849
H	-1.724174	-1.834638	-0.405162
C	0.526227	1.263475	0.147198
H	-1.283477	2.387791	0.222585
C	1.068495	-0.002935	0.001638
H	0.746112	-2.094637	-0.318517
H	1.179259	2.113896	0.288239
N	2.535791	-0.158510	0.035422
O	3.201976	0.840876	0.198774
O	2.984074	-1.276381	-0.100873
C	-3.718876	1.741228	-0.616651
H	-3.501407	2.573070	0.058889
H	-3.278555	1.983288	-1.586917
H	-4.800957	1.677921	-0.726962

Coordinates of **A71:**

G_{gas} = -1022.866409 Hartree

C	-1.417185	3.925119	0.435487
C	-0.316992	3.355730	-0.059350
H	-1.542513	5.001147	0.415111
H	-2.210375	3.346823	0.894089
C	-0.146414	1.878661	-0.048542
C	-1.250378	1.021595	-0.133964
C	1.122432	1.305073	0.033672
C	-1.082178	-0.353524	-0.107500
H	-2.245126	1.433543	-0.251787
C	1.277321	-0.077560	0.062544
H	2.004388	1.932286	0.088340
C	0.182969	-0.923785	-0.005007
C	-2.289400	-1.249088	-0.147120
C	2.671316	-0.636805	0.125688

H	0.308661	-1.998838	0.007516
F	-2.733289	-1.526649	1.089713
F	-3.309001	-0.687914	-0.809354
F	-2.022193	-2.421402	-0.736156
F	3.399689	-0.036931	1.078929
F	2.680686	-1.949443	0.378861
F	3.326510	-0.450405	-1.031701
C	0.812226	4.176939	-0.625316
H	1.689906	4.142576	0.026253
H	1.116765	3.804118	-1.606365
H	0.513515	5.220010	-0.722795

Coordinates of **A72:**

G_{gas} = -538.447046 Hartree

C	0.707192	-1.614724	-8.060242
C	0.772412	-0.516083	-7.310762
H	1.601264	-2.039829	-8.498618
H	-0.227707	-2.120495	-8.272877
H	1.740928	-0.047137	-7.157994
C	-0.357502	0.172301	-6.658200
C	-1.635195	-0.411382	-6.580650
C	-0.136738	1.426356	-6.112543
C	-2.661578	0.287785	-5.967283
H	-1.798731	-1.400315	-6.982813
C	-1.184826	2.123030	-5.496423
H	0.839413	1.893921	-6.155281
C	-2.448724	1.562681	-5.421118
O	-3.930519	-0.179145	-5.832558
O	-0.865301	3.344393	-4.999696
H	-3.287613	2.057417	-4.952690
C	-4.219896	-1.457486	-6.357267
C	-1.889422	4.083040	-4.367164
H	-3.615935	-2.229833	-5.871162
H	-5.272411	-1.636884	-6.150236
H	-4.050289	-1.487911	-7.438036
H	-1.433418	5.016432	-4.045496
H	-2.707809	4.296472	-5.061618
H	-2.280714	3.548314	-3.496246

Coordinates of **A73:**

G_{gas} = -423.963356 Hartree

C	0.689295	-0.862474	-5.925760
C	0.636555	-1.996810	-5.228627
H	-0.188446	-0.485320	-6.434887

H	1.595067	-0.274083	-6.016518
H	-0.309317	-2.531898	-5.186708
C	1.735973	-2.634616	-4.486844
C	3.025138	-2.083788	-4.403283
C	1.507194	-3.840876	-3.830209
C	4.028212	-2.715211	-3.698223
H	3.245657	-1.144893	-4.897111
C	2.507936	-4.494809	-3.112613
H	0.519964	-4.288674	-3.877257
C	3.777476	-3.928876	-3.045917
H	5.023600	-2.293596	-3.631019
H	2.282560	-5.430410	-2.619339
O	4.828561	-4.470090	-2.379869
C	4.620299	-5.694916	-1.709163
H	4.329356	-6.484028	-2.409534
H	5.571054	-5.952280	-1.248277
H	3.854623	-5.595168	-0.933472

Coordinates of **A74:**

G_{gas} = -348.762167 Hartree

C	-0.666687	-2.273731	-6.210230
C	-0.690843	-3.310231	-5.374171
H	-1.584383	-1.872823	-6.621491
H	0.255971	-1.796004	-6.519258
H	-1.653190	-3.750239	-5.123661
C	0.469552	-3.960206	-4.740204
C	1.766692	-3.436476	-4.805965
C	0.282583	-5.149911	-4.034199
C	2.827660	-4.089750	-4.200643
H	1.947421	-2.502048	-5.324222
C	1.349936	-5.804190	-3.428327
H	-0.715341	-5.569773	-3.960647
C	2.641031	-5.288500	-3.504311
H	3.823169	-3.661387	-4.260733
H	1.174597	-6.727612	-2.886446
C	3.809069	-5.999094	-2.872716
H	3.474254	-6.737631	-2.143342
H	4.399905	-6.520635	-3.630806
H	4.471485	-5.293861	-2.366962

Coordinates of **A75:**

G_{gas} = -615.628447 Hartree

C	-0.098267	0.112000	-8.500410
C	-0.527381	0.729001	-7.400570

H	0.928958	0.213440	-8.826641
H	-0.746326	-0.503089	-9.114267
H	0.175765	1.345755	-6.845964
C	-1.887458	0.684253	-6.837314
C	-2.877068	-0.188957	-7.305207
C	-2.219707	1.545000	-5.788097
C	-4.145853	-0.196025	-6.750999
H	-2.651291	-0.884768	-8.104438
C	-3.489874	1.556016	-5.224700
H	-1.469443	2.231465	-5.410121
C	-4.451627	0.677472	-5.710135
H	-4.914616	-0.871386	-7.105417
H	-3.732148	2.237261	-4.418300
O	-5.745075	0.659961	-5.249413
C	-5.996561	0.933287	-3.923609
C	-5.241486	0.363730	-2.902348
C	-7.086426	1.745343	-3.637860
C	-5.584500	0.624763	-1.581218
H	-4.399801	-0.273482	-3.146529
C	-7.423410	1.992745	-2.311491
H	-7.654343	2.165188	-4.458706
C	-6.673371	1.438284	-1.279704
H	-5.000388	0.182118	-0.783164
H	-8.274149	2.624741	-2.086405
H	-6.935917	1.635490	-0.247816

Coordinates of **A76:**

G_{gas} = -769.092163 Hartree

C	0.113916	-2.527524	-6.998944
C	0.062642	-3.465163	-6.054860
H	-0.790295	-2.170271	-7.475105
H	1.048475	-2.094629	-7.337462
H	-0.905002	-3.879266	-5.782996
C	1.211724	-4.037830	-5.331412
C	2.477379	-3.440616	-5.332462
C	1.042354	-5.222373	-4.609785
C	3.541949	-4.015883	-4.656059
H	2.633723	-2.504583	-5.855066
C	2.098582	-5.812955	-3.927076
H	0.066692	-5.695690	-4.587757
C	3.343644	-5.202896	-3.959680
H	4.518608	-3.549108	-4.656378
H	1.960853	-6.733493	-3.374580
Cl	4.679660	-5.927740	-3.106033

Coordinates of **A77:**

G_{gas} = -2883.065437 Hartree

C	-0.058424	-2.246292	-7.112361
C	0.012048	-3.139948	-6.127848
H	-0.999776	-2.036464	-7.604065
H	0.810596	-1.705217	-7.469307
H	-0.892215	-3.669032	-5.837890
C	1.223451	-3.518943	-5.379038
C	2.399309	-2.761044	-5.412445
C	1.207975	-4.677405	-4.598102
C	3.527303	-3.155280	-4.708670
H	2.434722	-1.840578	-5.982822
C	2.329121	-5.088445	-3.886888
H	0.303317	-5.273953	-4.550514
C	3.483054	-4.321345	-3.951975
H	4.431423	-2.561127	-4.737369
H	2.306087	-5.990475	-3.289457
Br	5.023775	-4.863356	-2.983422

Coordinates of **A78:**

G_{gas} = -537.306497 Hartree

C	0.754041	0.025430	-6.863401
C	0.713011	-1.039091	-6.064419
H	-0.136797	0.371307	-7.372274
H	1.668544	0.577401	-7.048323
H	-0.229533	-1.567251	-5.945114
C	1.849712	-1.612328	-5.321949
C	3.047729	-0.913694	-5.121960
C	1.732396	-2.901082	-4.791812
C	4.097628	-1.495730	-4.433519
H	3.151675	0.098331	-5.493986
C	2.781896	-3.491065	-4.101134
H	0.806107	-3.447801	-4.931047
C	3.971459	-2.788588	-3.922151
H	5.026140	-0.961415	-4.272385
H	2.684309	-4.491008	-3.698824
C	5.130827	-3.365270	-3.187059
O	6.173630	-2.792834	-3.003966
O	4.892153	-4.608709	-2.742231
C	5.968975	-5.216957	-2.028420
H	5.614563	-6.204291	-1.744951
H	6.850391	-5.291605	-2.665554
H	6.217433	-4.627998	-1.145354

Coordinates of **A79:**

G_{gas} = -513.971097 Hartree

C	1.782678	-0.818238	-6.935382
C	1.609273	-1.819462	-6.075373
H	0.943612	-0.402477	-7.478611
H	2.757957	-0.391114	-7.139734
H	0.612251	-2.229503	-5.939061
C	2.670243	-2.470907	-5.287019
C	3.919193	-1.872926	-5.073504
C	2.422930	-3.729051	-4.726352
C	4.902310	-2.519749	-4.344079
H	4.119114	-0.883926	-5.466412
C	3.394978	-4.392737	-3.992579
H	1.456036	-4.195914	-4.875330
C	4.622200	-3.773849	-3.818300
H	5.870044	-2.070182	-4.168230
H	3.218385	-5.367701	-3.559324
N	5.664911	-4.466659	-3.036500
O	5.395465	-5.559943	-2.588018
O	6.726495	-3.899721	-2.892289

Coordinates of **A80:**

G_{gas} = -983.585809 Hartree

C	-0.034562	0.829614	-9.170578
C	0.046263	1.735145	-8.198899
H	0.846073	0.537289	-9.728027
H	-0.969369	0.359346	-9.454013
H	1.008364	2.191111	-7.982526
C	-1.074968	2.219203	-7.373231
C	-2.306219	1.554375	-7.312270
C	-0.912360	3.376211	-6.613754
C	-3.333927	2.052607	-6.530201
H	-2.456415	0.631526	-7.858679
C	-1.954273	3.867645	-5.832599
H	0.036672	3.902080	-6.631547
C	-3.175588	3.217845	-5.782826
C	-4.666043	1.354838	-6.513211
C	-1.720707	5.128037	-5.047139
H	-3.981528	3.594591	-5.166103
F	-5.511415	1.892748	-7.407068
F	-4.556592	0.054599	-6.812976
F	-5.258619	1.444617	-5.315832
F	-1.496770	6.174506	-5.857161

F	-2.760599	5.441953	-4.268553
F	-0.643275	5.018842	-4.255112

Coordinates of C_2H_6:

G_{gas} = -79.743369 Hartree

C	0.000000	0.000000	0.763511
H	0.508620	0.881510	1.159054
H	0.509100	-0.881233	1.159054
H	-1.017720	-0.000277	1.159054
C	0.000000	0.000000	-0.763511
H	1.017720	-0.000277	-1.159054
H	-0.509100	-0.881233	-1.159054
H	-0.508620	0.881510	-1.159054

Coordinates of C_2H_4:

G_{gas} = -78.533753 Hartree

C	0.000000	-0.662880	0.000000
H	0.000000	-1.230825	-0.923529
H	0.000000	-1.230825	0.923529
C	0.000000	0.662880	0.000000
H	0.000000	1.230825	0.923529
H	0.000000	1.230825	-0.923529

Coordinates of C_2H_2:

G_{gas} = -77.307672 Hartree

C	0.000000	0.000000	0.598516
H	0.000000	0.000000	1.662954
C	0.000000	0.000000	-0.598516
H	0.000000	0.000000	-1.662954

Coordinates of C_6H_6:

G_{gas} = -232.124668 Hartree

C	1.346002	-0.353813	0.000001
C	0.979397	0.988773	-0.000013
C	-0.366561	1.342554	0.000013
C	-1.346018	0.353752	-0.000002
C	-0.979442	-0.988729	-0.000011
C	0.366621	-1.342537	0.000010
H	2.394084	-0.629252	-0.000001
H	1.741983	1.758705	-0.000007
H	-0.652205	2.387899	0.000010
H	-2.394063	0.629331	0.000006
H	-1.741929	-1.758760	-0.000012
H	0.652130	-2.387919	0.000020

Coordinates of **TS1:**

G_{sol} = -3452.510648 Hartree

Ru	-0.040929	0.025912	-0.727164
P	1.716329	-1.160654	-1.675318
P	-1.421776	-1.785083	-0.824965
C	-1.328785	-2.854582	0.673122
N	0.554968	-0.063294	1.298636
H	-0.156520	0.512272	1.791307
H	0.520651	-0.984928	1.724586
N	1.270924	1.718731	-0.482769
C	1.215240	-2.287393	-3.050223
H	0.706002	-1.675025	-3.801221
H	2.115234	-2.720502	-3.501141
C	0.274023	-3.400366	-2.567967
H	0.719273	-3.915557	-1.707331
H	0.207553	-4.150369	-3.365537
C	-1.156114	-2.939319	-2.254621
H	-1.818016	-3.804772	-2.129149
H	-1.544354	-2.372784	-3.109917
C	-3.242600	-1.605953	-1.030177
C	-3.735470	-0.553591	-1.807101
H	-3.053479	0.210820	-2.167485
C	-5.087802	-0.499645	-2.130058
H	-5.457853	0.319490	-2.739171
C	-5.963626	-1.475651	-1.663269
H	-7.019788	-1.425674	-1.913528
C	-5.483947	-2.509307	-0.864227
H	-6.162330	-3.270031	-0.487810
C	-4.128490	-2.578180	-0.555326
H	-3.762074	-3.398625	0.055641
C	-0.999853	-4.211898	0.656137
H	-0.853518	-4.729426	-0.286413
C	-0.838582	-4.920927	1.846186
H	-0.573346	-5.973662	1.811864
C	-1.013846	-4.284596	3.070826
H	-0.883864	-4.836646	3.997004
C	-1.366680	-2.935788	3.101583
H	-1.518846	-2.430399	4.051141
C	-1.522982	-2.229171	1.913676
H	-1.779965	-1.172428	1.947420
C	2.976616	-0.062908	-2.435775
C	4.065518	0.399394	-1.686899
H	4.246986	0.005042	-0.690673

C	4.926027	1.363177	-2.200636
H	5.761474	1.712239	-1.600650
C	4.712647	1.881306	-3.475341
H	5.383887	2.634204	-3.878254
C	3.631040	1.432705	-4.226874
H	3.449831	1.837364	-5.218303
C	2.764338	0.473201	-3.710078
H	1.897883	0.173529	-4.289240
C	2.751485	-2.334178	-0.691081
C	2.229057	-2.886347	0.481034
H	1.238043	-2.599325	0.810826
C	2.943661	-3.829085	1.216482
H	2.505468	-4.246346	2.118507
C	4.203983	-4.231134	0.787052
H	4.769711	-4.961203	1.358492
C	4.735116	-3.698384	-0.386767
H	5.714964	-4.014527	-0.732745
C	4.013115	-2.764007	-1.122020
H	4.439284	-2.356475	-2.034624
C	1.899138	0.505817	1.510050
H	2.601207	-0.238021	1.106367
C	2.035418	1.728655	0.627311
C	2.951257	2.745746	0.863701
H	3.554502	2.737675	1.764320
C	3.087186	3.768742	-0.066981
H	3.796997	4.572363	0.103376
C	2.306935	3.741560	-1.212364
H	2.377571	4.514703	-1.968237
C	1.406675	2.700185	-1.380839
H	0.771634	2.614547	-2.255490
H	-1.397185	0.941303	-0.140488
C	-0.374119	5.655941	-0.105893
C	-1.054576	4.878995	-1.043683
C	-1.562124	3.635246	-0.692181
C	-1.417757	3.163575	0.616678
C	-0.725488	3.936828	1.546571
C	-0.198057	5.175881	1.187169
H	0.025204	6.626666	-0.387642
H	-1.175513	5.236433	-2.062574
H	-2.046060	3.012676	-1.434406
H	-0.619560	3.559780	2.558182
H	0.338587	5.768492	1.923188
C	-2.006784	1.858949	1.103996
O	-1.584469	1.360871	2.180985

C	-3.475414	1.596593	0.785932
C	-4.138400	0.585993	1.486333
C	-5.417886	2.251767	-0.228756
C	-5.501349	0.421125	1.295538
H	-3.587084	-0.033889	2.180575
C	-6.164959	1.280449	0.426198
H	-5.894577	2.940824	-0.923367
H	-6.038761	-0.364126	1.818386
H	-7.232014	1.194083	0.249045
N	-4.105683	2.410936	-0.061131
C	2.262896	0.695034	2.994138
C	1.442901	1.772820	3.716762
C	2.178704	-0.641493	3.749916
H	3.321325	0.998082	3.020992
C	1.890758	1.922351	5.174019
H	0.377490	1.513323	3.681703
H	1.538034	2.731528	3.194928
C	2.637184	-0.490398	5.202552
H	1.138453	-0.998790	3.754000
H	2.775856	-1.404406	3.232502
C	1.824582	0.588868	5.923691
H	1.270311	2.672023	5.677974
H	2.924317	2.298192	5.200272
H	2.547993	-1.449958	5.725073
H	3.702042	-0.217681	5.220596
H	2.183412	0.711149	6.952282
H	0.775889	0.265668	5.988580
Cl	-0.697903	0.676815	-3.037081

Coordinates of **TS1'**:

G_{sol} = -3452.508038 Hartree

Ru	-0.048297	0.040901	-0.739128
P	1.711594	-1.164404	-1.667674
P	-1.428083	-1.774218	-0.821254
C	-1.334744	-2.823563	0.691244
N	0.545337	-0.025734	1.291343
H	-0.162957	0.556516	1.778884
H	0.502437	-0.943935	1.723980
N	1.257888	1.737794	-0.508763
C	1.216982	-2.304389	-3.033694
H	0.709492	-1.699093	-3.791369
H	2.119134	-2.741179	-3.476726
C	0.274933	-3.412412	-2.542560
H	0.719420	-3.920571	-1.677363

H	0.208714	-4.169157	-3.333841
C	-1.154738	-2.947332	-2.234486
H	-1.815893	-3.811580	-2.097419
H	-1.543879	-2.392697	-3.097067
C	-3.245177	-1.600599	-1.033607
C	-3.741536	-0.528471	-1.778564
H	-3.065081	0.255104	-2.106597
C	-5.093114	-0.476778	-2.105247
H	-5.469475	0.361495	-2.682940
C	-5.961374	-1.474833	-1.674409
H	-7.017410	-1.423633	-1.924964
C	-5.476748	-2.531636	-0.908713
H	-6.150699	-3.309007	-0.559353
C	-4.123289	-2.597352	-0.595017
H	-3.751784	-3.432724	-0.007481
C	-0.993582	-4.177913	0.694954
H	-0.837252	-4.706926	-0.239607
C	-0.832871	-4.869130	1.895564
H	-0.558575	-5.919997	1.877271
C	-1.020588	-4.217416	3.110228
H	-0.891531	-4.755545	4.044758
C	-1.386654	-2.871728	3.120030
H	-1.551437	-2.355199	4.061499
C	-1.543152	-2.182946	1.921648
H	-1.814293	-1.129252	1.938827
C	2.979789	-0.078338	-2.434567
C	4.075397	0.378807	-1.692451
H	4.256883	-0.010406	-0.694207
C	4.946125	1.328063	-2.216675
H	5.787933	1.671371	-1.622104
C	4.736140	1.837333	-3.495355
H	5.416042	2.577679	-3.907074
C	3.647154	1.394829	-4.240064
H	3.468369	1.792326	-5.234867
C	2.770744	0.449885	-3.713066
H	1.898888	0.155336	-4.286681
C	2.743055	-2.332691	-0.671625
C	2.219025	-2.869465	0.506725
H	1.230719	-2.572042	0.835512
C	2.927269	-3.811431	1.249516
H	2.486788	-4.217051	2.155747
C	4.183412	-4.227655	0.821658
H	4.744102	-4.957389	1.398496
C	4.716491	-3.710062	-0.358149

H	5.692880	-4.037678	-0.703311
C	4.000386	-2.777099	-1.100815
H	4.427642	-2.382650	-2.018665
C	1.897828	0.529051	1.482309
H	2.583393	-0.221528	1.063690
C	2.036672	1.747457	0.591892
C	2.972333	2.752836	0.803332
H	3.588121	2.745598	1.695621
C	3.121907	3.754139	-0.148843
H	3.855918	4.541019	-0.004028
C	2.326031	3.725667	-1.284480
H	2.411356	4.480385	-2.057680
C	1.397055	2.704713	-1.422879
H	0.740524	2.618804	-2.282760
H	-1.401809	0.977902	-0.176427
C	-0.288858	5.560365	-0.077529
C	-1.099834	4.814477	-0.928761
C	-1.420454	3.173150	0.633431
C	-0.045253	5.057477	1.194520
H	0.137523	6.501828	-0.408525
H	-1.314342	5.165473	-1.936156
H	0.581262	5.600391	1.896764
C	-2.066465	1.869426	1.090750
O	-1.625220	1.371563	2.159966
C	-3.528207	1.629103	0.770748
C	-5.543495	0.414459	1.360717
C	-5.656301	2.220783	-0.220428
C	-6.291713	1.220991	0.507127
H	-6.024770	-0.379096	1.924571
H	-6.228702	2.850713	-0.895854
H	-7.361477	1.062342	0.403209
C	2.298035	0.705668	2.958755
C	1.502062	1.780637	3.710154
C	2.223768	-0.637316	3.704780
H	3.358370	1.003123	2.963497
C	1.976525	1.912103	5.160894
H	0.434045	1.527120	3.692891
H	1.600113	2.744860	3.198741
C	2.711453	-0.502835	5.149417
H	1.182817	-0.991684	3.725878
H	2.807763	-1.396775	3.168441
C	1.917133	0.571337	5.897510
H	1.369671	2.659981	5.683832
H	3.012396	2.282036	5.171213

H	2.628493	-1.467540	5.663129
H	3.777539	-0.234340	5.149801
H	2.295418	0.681756	6.920372
H	0.868391	0.252176	5.978507
C	-4.179226	0.628455	1.499634
H	-3.604024	0.033847	2.198757
C	-4.283345	2.417354	-0.103407
H	-3.795320	3.178773	-0.695853
N	-1.648696	3.649882	-0.593440
C	-0.625309	3.849074	1.559446
H	-0.492314	3.422129	2.544709
Cl	-0.679245	0.639214	-3.065730

Coordinates of **TS2:**

G_{sol} = -2924.869584 Hartree

S	1.553254	1.186036	-1.316912
O	1.691395	2.312486	-0.396661
O	0.707598	1.347534	-2.505213
N	1.072568	-0.161243	-0.520867
N	-0.442984	-1.676044	1.057470
C	1.511729	-0.227870	0.885042
C	0.979994	-1.551066	1.445645
C	3.019597	-0.183820	1.071380
C	3.842289	-1.126509	0.449805
C	5.218547	-1.098837	0.633656
C	5.792076	-0.125091	1.449598
C	4.980132	0.817099	2.072801
C	3.600382	0.786592	1.882796
C	1.233920	-1.656727	2.937570
C	0.587814	-0.808502	3.842861
C	0.860395	-0.903606	5.203656
C	1.782922	-1.835260	5.675635
C	2.433495	-2.676503	4.778522
C	2.156097	-2.586333	3.417228
C	3.195863	0.836102	-1.931957
C	3.392818	-0.217768	-2.817489
C	4.678338	-0.521849	-3.244795
C	5.776661	0.220299	-2.798991
C	5.549638	1.289089	-1.929930
C	4.267640	1.602277	-1.493770
C	7.178992	-0.139822	-3.219124
C	-1.101558	-2.857856	1.646284
H	1.060598	0.593925	1.456966
H	1.533582	-2.357646	0.947870

H	3.402504	-1.862555	-0.218490
H	5.846383	-1.827499	0.128735
H	6.868495	-0.098332	1.592936
H	5.419577	1.582177	2.706114
H	2.966578	1.531135	2.355451
H	-0.139810	-0.086465	3.479646
H	0.350704	-0.242969	5.898888
H	1.995209	-1.902601	6.738646
H	3.159491	-3.400912	5.135836
H	2.679685	-3.231695	2.715398
H	2.546552	-0.804015	-3.161971
H	4.834517	-1.347829	-3.934422
H	6.390886	1.878240	-1.574421
H	4.092207	2.413483	-0.796547
H	7.692462	-0.689457	-2.420758
H	7.772512	0.754776	-3.433069
H	7.182165	-0.771451	-4.112554
H	-2.157046	-2.821299	1.364543
H	-1.062341	-2.749117	2.737520
H	-1.059481	-0.776999	1.580427
H	-1.535524	0.348246	-0.055208
C	-0.549720	-4.243068	1.317441
H	0.548646	-4.256197	1.357880
H	-0.908874	-4.928211	2.090172
C	-0.293347	-4.454125	-1.053494
H	0.781890	-4.335784	-0.847120
H	-0.409282	-5.283366	-1.761616
C	-0.814091	-3.196561	-1.710197
C	0.035290	-2.417871	-2.542323
C	-2.154693	-2.759848	-1.497994
C	-0.449467	-1.229336	-3.164949
H	1.076374	-2.701572	-2.661713
C	-2.582137	-1.530231	-2.028270
H	-2.791168	-3.310361	-0.816278
C	-1.743266	-0.751202	-2.896243
H	0.231005	-0.601796	-3.726707
H	-3.556343	-1.142640	-1.746887
O	-0.995679	-4.815138	0.099059
Ru	-0.687303	-1.192427	-0.946567
C	-2.149541	0.788784	0.862393
O	-1.800466	0.156086	1.989255
C	-1.765218	2.260178	0.839640
C	-1.378613	2.902894	-0.334575
C	-1.814313	2.978852	2.036250

C	-1.057272	4.253877	-0.331124
H	-1.274066	2.336298	-1.255357
C	-1.499676	4.328980	2.058647
H	-2.094291	2.460486	2.946669
C	-1.130643	4.947264	0.868140
C	-3.599546	0.528461	0.437009
C	-4.247801	-0.588201	0.952681
C	-5.532958	0.936380	-0.969386
C	-5.514234	-0.969410	0.510573
H	-3.743376	-1.159756	1.724641
C	-6.160543	-0.208865	-0.466875
H	-6.058981	1.534740	-1.706364
C	-2.229593	0.546480	-3.474212
H	-1.401273	1.251249	-3.569960
H	-2.665123	0.368793	-4.464501
H	-3.004408	0.983710	-2.839978
C	-4.272873	1.299445	-0.515873
H	-3.812471	2.201375	-0.908653
H	-1.532170	4.906175	2.974385
H	-0.728896	4.758436	-1.230592
H	-5.985835	-1.844729	0.943612
O	-7.387200	-0.485354	-0.977702
N	-0.794052	6.373393	0.885647
O	-0.818444	6.951471	1.962889
O	-0.512818	6.906642	-0.177267
C	-8.073139	-1.609873	-0.477821
H	-9.025144	-1.643257	-1.008962
H	-7.519875	-2.538925	-0.670063
H	-8.263090	-1.520421	0.599588

Coordinates of **TS2'**:

G_{sol} = -2924.867837 Hartree

S	1.607390	1.185090	-1.449434
O	1.767647	2.383616	-0.631376
O	0.728409	1.239590	-2.622809
N	1.159879	-0.093512	-0.528865
N	-0.282684	-1.508699	1.201873
C	1.635663	-0.038976	0.865638
C	1.145873	-1.322978	1.543537
C	3.146973	0.043261	1.008422
C	3.970322	-0.936494	0.447994
C	5.349835	-0.874549	0.595569
C	5.926434	0.172193	1.312878
C	5.113975	1.152184	1.874147

C	3.730867	1.086598	1.721095
C	1.436556	-1.305044	3.032371
C	0.796453	-0.398262	3.883594
C	1.101820	-0.382069	5.240688
C	2.051199	-1.258936	5.761796
C	2.696115	-2.157605	4.917840
C	2.386017	-2.179166	3.560825
C	3.235141	0.791417	-2.081160
C	3.416728	-0.331189	-2.881403
C	4.692332	-0.662700	-3.318160
C	5.796613	0.120463	-2.967419
C	5.584811	1.256909	-2.184692
C	4.312830	1.597588	-1.739802
C	7.189506	-0.265917	-3.395670
C	-0.898472	-2.655980	1.896778
H	1.183264	0.818750	1.380849
H	1.704428	-2.155785	1.097627
H	3.527536	-1.732208	-0.145766
H	5.977276	-1.634373	0.138359
H	7.005290	0.225681	1.427666
H	5.555817	1.973730	2.430473
H	3.095845	1.859155	2.144523
H	0.047294	0.280621	3.482677
H	0.595900	0.322475	5.894358
H	2.288373	-1.239635	6.821521
H	3.442557	-2.840300	5.313128
H	2.904579	-2.869581	2.899289
H	2.566344	-0.948609	-3.153255
H	4.835801	-1.542370	-3.940940
H	6.430784	1.878665	-1.903602
H	4.149964	2.462713	-1.107359
H	7.725034	-0.755617	-2.573119
H	7.774003	0.612124	-3.688461
H	7.172873	-0.960423	-4.241005
H	-1.960767	-2.665936	1.639483
H	-0.836025	-2.461924	2.974952
H	-0.902449	-0.590026	1.670762
H	-1.451123	0.409027	-0.029182
C	-0.322293	-4.050051	1.660392
H	0.776723	-4.036391	1.670824
H	-0.644865	-4.680515	2.493363
C	-0.122632	-4.441669	-0.693069
H	0.954983	-4.287390	-0.526670
H	-0.240179	-5.325632	-1.331194

C	-0.686228	-3.249261	-1.430933
C	0.125951	-2.519990	-2.341411
C	-2.030408	-2.824770	-1.219765
C	-0.398108	-1.392745	-3.041231
H	1.169670	-2.792039	-2.464545
C	-2.495340	-1.647919	-1.832264
H	-2.639819	-3.335657	-0.484403
C	-1.694499	-0.919667	-2.776678
H	0.255709	-0.795976	-3.664855
H	-3.472374	-1.261561	-1.560979
O	-0.786968	-4.723990	0.502863
Ru	-0.584630	-1.189057	-0.827362
C	-2.034032	0.913798	0.877597
O	-1.657525	0.358757	2.035078
C	-1.685896	2.383727	0.734388
C	-1.349738	2.955562	-0.494310
C	-1.730870	3.198393	1.860316
C	-1.088584	4.311223	-0.600597
H	-1.244315	2.327006	-1.375275
C	-1.470141	4.563875	1.771250
H	-1.970175	2.752527	2.820578
C	-1.152687	5.126030	0.532747
C	-3.491250	0.588242	0.509037
C	-4.098547	-0.503430	1.134051
C	-5.472142	0.880249	-0.856918
C	-5.365570	-0.929133	0.760377
H	-3.553955	-1.001960	1.927842
C	-6.030210	-0.236138	-0.245863
H	-6.031124	1.411091	-1.617951
C	-2.219369	0.323203	-3.435761
H	-1.406430	1.032353	-3.603560
H	-2.677661	0.066277	-4.397903
H	-2.983723	0.796019	-2.814345
C	-4.201951	1.290136	-0.468368
H	-3.762400	2.169678	-0.927750
H	-1.514094	5.170463	2.668824
H	-0.797011	4.753448	-1.546924
H	-5.844743	-1.777827	1.233482
O	-0.881038	6.442485	0.331093
C	-0.893107	7.297061	1.448944
H	-0.639971	8.289725	1.073529
H	-1.883911	7.332631	1.921629
H	-0.149311	6.993730	2.197705
N	-7.359552	-0.688570	-0.665310

O	-7.824101	-1.674813	-0.113029
O	-7.922834	-0.057950	-1.547343

Coordinates of **TS3:**

G_{sol} = -1687.906943 Hartree

C	-2.182940	0.254104	-0.169776
C	-1.471963	0.112865	1.236943
H	-0.732716	0.910597	1.330918
C	-1.947374	1.659436	-0.731628
C	-1.673418	1.813417	-2.090630
C	-1.984522	2.797137	0.078286
C	-1.427743	3.073667	-2.625029
H	-1.626070	0.927311	-2.713043
C	-1.747685	4.061366	-0.456033
H	-2.176985	2.704225	1.144443
C	-1.463030	4.203568	-1.810756
H	-1.202957	3.173804	-3.682965
H	-1.778885	4.933053	0.191775
H	-1.271787	5.187174	-2.230069
C	-3.695064	-0.043886	-0.155816
C	-4.628963	0.866389	0.346671
C	-4.162823	-1.258521	-0.659019
C	-5.983498	0.553423	0.388506
H	-4.305846	1.840682	0.699693
C	-5.518460	-1.572796	-0.623727
H	-3.450622	-1.949892	-1.093711
C	-6.435009	-0.672697	-0.090796
H	-6.688267	1.276110	0.789582
H	-5.857642	-2.525383	-1.020856
H	-7.492668	-0.917453	-0.060836
O	-1.567097	-0.705607	-0.990783
B	-0.421654	-1.321537	-0.389111
N	-0.718759	-1.178033	1.176689
C	-2.343987	0.004364	2.483862
H	-3.233038	0.634873	2.432618
H	-1.757151	0.319002	3.349279
C	-2.692547	-1.492478	2.584243
H	-3.720869	-1.677594	2.262909
H	-2.590077	-1.848481	3.612056
C	-1.708519	-2.210691	1.641822
H	-2.230357	-2.618828	0.774144
H	-1.162272	-3.018281	2.126936
B	0.531676	-1.176323	2.158435
H	1.005485	-2.286466	2.214302

H	0.239757	-0.702421	3.235907
H	1.384208	-0.398735	1.652623
O	0.728509	-0.247488	-0.597016
C	1.869626	-0.152115	-0.038580
C	2.388717	1.217757	0.151023
C	3.700903	1.472029	0.580526
C	1.549840	2.301300	-0.122454
C	4.145053	2.767908	0.750121
H	4.380223	0.650067	0.777550
C	1.986826	3.607267	0.036424
C	3.290339	3.846707	0.482522
H	1.304036	4.416369	-0.193963
H	0.544767	2.117048	-0.480261
H	5.155530	2.977857	1.083360
C	2.841970	-1.288434	-0.135717
C	3.051173	-1.805119	-1.416024
C	3.565436	-1.795756	0.942966
C	3.959359	-2.839760	-1.610718
H	2.491742	-1.401765	-2.253723
C	4.470342	-2.831501	0.744665
H	3.378400	-1.412331	1.939997
C	4.665282	-3.359128	-0.530052
H	4.106793	-3.245654	-2.606620
H	5.012716	-3.238968	1.592040
C	-0.120796	-2.766113	-1.030895
C	0.628544	-3.848694	-0.244279
H	0.408842	-2.590050	-1.978810
H	-1.093401	-3.179786	-1.337172
C	1.014590	-5.050479	-1.106841
H	1.534793	-3.446048	0.215780
H	0.008565	-4.197740	0.592195
C	1.807426	-6.101222	-0.332866
H	0.109828	-5.503088	-1.536045
H	1.614016	-4.696837	-1.957271
H	2.077056	-6.954043	-0.965622
H	2.732891	-5.666847	0.063854
H	1.230325	-6.483220	0.517551
H	5.364209	-4.176514	-0.679181
O	3.817988	5.072990	0.676470
C	3.003869	6.199059	0.419815
H	3.620104	7.070004	0.643786
H	2.691154	6.234939	-0.630856
H	2.116440	6.205446	1.064342

Coordinates of **TS3'**:

G_{sol} = -1687.906179 Hartree

C	-2.397419	-0.433795	-0.197727
C	-1.782615	-0.211846	1.243334
H	-1.564440	0.850777	1.364657
C	-2.891408	0.901229	-0.762298
C	-2.666154	1.199411	-2.105948
C	-3.551211	1.841958	0.032450
C	-3.078095	2.415869	-2.639621
H	-2.137332	0.476224	-2.715969
C	-3.972070	3.057333	-0.501635
H	-3.722129	1.643737	1.087899
C	-3.731930	3.350842	-1.840475
H	-2.882498	2.637475	-3.684819
H	-4.479297	3.777294	0.134397
H	-4.051132	4.301154	-2.258302
C	-3.537737	-1.468960	-0.254177
C	-4.830669	-1.177240	0.189117
C	-3.287105	-2.744255	-0.762304
C	-5.829935	-2.144483	0.169321
H	-5.074043	-0.180438	0.543102
C	-4.286228	-3.713126	-0.788637
H	-2.300214	-2.963892	-1.152178
C	-5.560501	-3.421550	-0.313790
H	-6.825146	-1.893837	0.525030
H	-4.065691	-4.698908	-1.188357
H	-6.340550	-4.176949	-0.331924
O	-1.336284	-0.926531	-0.977372
B	-0.070410	-0.882945	-0.312357
N	-0.470567	-0.930729	1.233681
C	-2.533286	-0.772599	2.447125
H	-3.616337	-0.690338	2.343006
H	-2.236309	-0.212964	3.336418
C	-2.064969	-2.236263	2.549401
H	-2.834528	-2.920116	2.181788
H	-1.842012	-2.502708	3.585199
C	-0.808699	-2.332369	1.663718
H	-1.004888	-2.938708	0.777426
H	0.051886	-2.750272	2.184045
B	0.552921	-0.304539	2.278399
H	1.532655	-1.008004	2.354726
H	0.009116	-0.089047	3.340717
H	0.895400	0.817229	1.822303

O	0.377397	0.643636	-0.476080
C	1.285366	1.290818	0.134425
C	1.017701	2.736101	0.362416
C	2.010103	3.601459	0.834733
C	-0.254731	3.238700	0.069996
C	1.722054	4.945712	1.036008
H	3.008045	3.228944	1.037615
C	-0.535428	4.583461	0.264440
C	0.449929	5.438138	0.754044
H	-1.526404	4.958239	0.028275
H	0.229887	6.490439	0.908606
H	-1.012430	2.572946	-0.323466
H	2.495021	5.611718	1.406611
C	2.692573	0.798819	0.097386
C	3.188548	0.431552	-1.150786
C	3.530882	0.711853	1.213745
C	4.482959	-0.057359	-1.293316
H	2.550719	0.511701	-2.024869
C	4.814911	0.219024	1.086412
H	3.145930	0.970430	2.194105
C	5.293170	-0.190033	-0.165394
H	4.831947	-0.351217	-2.275897
H	5.460128	0.104623	1.950519
C	0.954491	-1.964153	-0.922776
C	2.117256	-2.530255	-0.096959
H	1.358321	-1.533710	-1.850826
H	0.339058	-2.809789	-1.265113
C	3.091364	-3.357963	-0.937017
H	2.675345	-1.732753	0.400108
H	1.727065	-3.159641	0.713776
C	4.294506	-3.853834	-0.137087
H	2.561300	-4.210917	-1.383047
H	3.442414	-2.743073	-1.777790
H	4.973027	-4.452861	-0.755507
H	4.864258	-3.011306	0.273293
H	3.977136	-4.476354	0.707619
O	6.542233	-0.708337	-0.175605
C	7.012048	-1.279110	-1.379086
H	7.120173	-0.523706	-2.167533
H	7.991115	-1.699621	-1.148475
H	6.344041	-2.077930	-1.724933

Coordinates of **TS4**:

G_{sol} = -2326.466762 Hartree

C	1.700852	-1.939239	-3.238529
C	2.283063	-1.052995	-2.359742
H	2.094490	-2.940700	-3.361239
H	1.059229	-1.570844	-4.030114
H	2.119391	0.009375	-2.514971
C	3.328763	-1.426103	-1.398460
C	4.228953	-0.461420	-0.938899
C	3.418619	-2.721793	-0.870372
C	5.195727	-0.776913	0.007833
H	4.170042	0.549091	-1.331952
C	4.383385	-3.031283	0.078100
H	2.711169	-3.486200	-1.181334
C	5.289853	-2.067304	0.532876
H	5.880900	-0.005996	0.353587
H	4.427484	-4.039383	0.483095
C	6.347172	-2.420057	1.547696
H	5.953947	-3.095378	2.314685
H	7.193721	-2.926904	1.068858
H	6.736753	-1.527015	2.045542
O	0.099811	-2.892354	-2.257436
Os	-0.400646	-2.139866	-0.759239
O	-2.034446	-1.687693	-1.086025
O	0.747682	-0.827800	-0.845153
O	0.016673	-3.335219	0.402969
N	-1.007731	-0.967545	1.408663
C	-1.874044	-1.891312	2.183701
C	-1.766644	0.312497	1.278618
C	0.261898	-0.790364	2.156347
C	-2.303696	-1.277872	3.545923
H	-1.311881	-2.817983	2.315452
H	-2.742292	-2.121245	1.556121
C	-1.830805	1.029692	2.646149
H	-2.775192	-0.000888	0.995270
C	-1.337616	1.240224	0.132817
H	0.777790	-1.753578	2.133293
H	0.870458	-0.085307	1.595173
C	-0.000177	-0.324401	3.607291
C	-1.489521	0.014365	3.741878
H	-2.013228	-1.973226	4.345238
C	-3.819046	-1.064722	3.655059
H	-1.109919	1.854716	2.681117
H	-2.821602	1.469750	2.787916
H	-1.318815	0.694955	-0.810581

O	-0.008730	1.713188	0.386881
C	-2.304901	2.403717	0.024210
H	0.275341	-1.107104	4.323116
H	0.607827	0.556779	3.842813
H	-1.696298	0.438488	4.730148
H	-4.028983	-0.461625	4.548165
H	-4.180457	-0.481061	2.797766
C	-4.601237	-2.375409	3.738039
C	0.703588	2.069716	-0.692366
C	-3.593458	2.225120	-0.560450
C	-1.984624	3.639860	0.529175
H	-5.676818	-2.187749	3.813033
H	-4.436851	-3.001934	2.853739
H	-4.298003	-2.956376	4.616605
O	0.267700	2.104991	-1.818344
C	2.114970	2.416111	-0.346704
C	-4.031885	1.000367	-1.137754
C	-4.467515	3.346928	-0.551798
C	-2.928473	4.686880	0.470487
H	-1.013249	3.820608	0.976317
C	2.897386	3.001113	-1.345169
C	2.666370	2.129301	0.902558
C	-5.298859	0.904921	-1.668425
H	-3.362354	0.149445	-1.175387
C	-5.768479	3.206812	-1.109432
N	-4.130690	4.560211	-0.037984
H	-2.669977	5.665294	0.871813
H	2.445569	3.215914	-2.307348
C	4.010558	2.379465	1.142911
H	2.051725	1.691792	1.679482
C	-6.176821	2.022898	-1.648038
O	-5.816996	-0.204326	-2.237656
H	-6.417143	4.076147	-1.089424
H	4.473781	2.129565	2.088919
H	-7.165841	1.901941	-2.077494
C	-5.004492	-1.360534	-2.288312
H	-5.606616	-2.128298	-2.775027
H	-4.090998	-1.184158	-2.867994
H	-4.721672	-1.693792	-1.282687
C	4.234731	3.285566	-1.107503
H	4.864969	3.738874	-1.862447
C	4.770083	2.948143	0.129891
N	6.202853	3.177519	0.366061
O	6.698217	2.663999	1.358149

O	6.807539	3.856552	-0.446128

Coordinates of **TS4'**:

G_{sol} = -2326.464997 Hartree

C	2.412875	-0.279369	-2.019525
C	2.922297	-1.551916	-2.145894
H	1.678760	0.091313	-2.725608
H	2.932734	0.469389	-1.433287
H	2.623851	-2.148062	-3.001689
C	4.036698	-2.062647	-1.344124
C	4.353330	-1.520228	-0.090413
C	4.794134	-3.143434	-1.807653
C	5.406328	-2.030148	0.655204
H	3.757834	-0.705425	0.312975
C	5.850456	-3.645857	-1.058957
H	4.555211	-3.588817	-2.770013
C	6.174578	-3.099786	0.185162
O	1.059987	-0.331519	-0.445222
Os	0.439356	-1.907249	0.011751
O	1.065873	-2.482499	1.503265
O	1.371227	-2.794686	-1.161544
O	-1.183732	-2.223083	-0.483573
N	-0.885137	-0.495433	1.674849
C	-2.022145	0.290289	1.107416
C	0.086390	0.365730	2.392613
C	-1.474250	-1.435557	2.661618
C	-2.575868	1.264932	2.172248
H	-2.783452	-0.461262	0.881974
C	-1.758992	0.968966	-0.245162
H	0.910213	-0.283247	2.701665
H	0.483720	1.080769	1.675218
C	-0.571622	1.058443	3.608503
C	-2.334663	-0.700633	3.726706
H	-0.643264	-1.977966	3.117697
H	-2.074157	-2.162922	2.103142
C	-2.084166	0.808841	3.549281
H	-2.222193	2.284818	1.981350
H	-3.667861	1.295157	2.115375
H	-1.383841	0.244211	-0.967664
O	-0.745039	1.963817	-0.064045
C	-3.035228	1.593775	-0.772939
H	-0.162747	0.668964	4.547726
H	-0.369097	2.135678	3.591709
H	-1.968225	-0.979768	4.723975

C	-3.811013	-1.114537	3.650270
H	-2.585159	1.375489	4.339744
C	0.028457	2.231483	-1.126473
C	-4.058231	0.782095	-1.346317
C	-3.256410	2.945002	-0.669857
H	-4.202764	-0.932419	2.640479
H	-3.863201	-2.201187	3.798246
C	-4.706915	-0.422526	4.675823
O	-0.168875	1.805675	-2.239460
C	1.180462	3.110861	-0.763924
C	-3.935386	-0.623582	-1.532051
C	-5.254569	1.438855	-1.746161
C	-4.479628	3.490554	-1.113451
H	-2.500475	3.598712	-0.248728
H	-5.718862	-0.838627	4.653418
H	-4.313786	-0.549524	5.691505
H	-4.789241	0.652292	4.481196
C	1.346654	3.623547	0.524754
C	2.115015	3.396684	-1.761055
C	-4.976237	-1.338154	-2.081776
H	-3.017040	-1.128406	-1.257090
C	-6.306944	0.664342	-2.308044
N	-5.455022	2.778513	-1.624817
H	-4.650898	4.562374	-1.029891
H	0.611919	3.407703	1.291770
C	3.224339	4.179882	-1.475200
H	1.960011	2.994323	-2.756081
C	-6.177261	-0.683185	-2.468707
O	-4.967811	-2.669965	-2.302356
H	-7.210116	1.187817	-2.603307
C	3.370632	4.671543	-0.184227
H	3.967269	4.412203	-2.227803
H	-6.971017	-1.286102	-2.897271
C	-3.807016	-3.391570	-1.939116
H	-3.602321	-3.304673	-0.865524
H	-4.010325	-4.432393	-2.192502
H	-2.927580	-3.041471	-2.492056
C	7.294867	-3.668682	1.017840
H	6.907538	-4.382985	1.754544
H	8.025506	-4.197199	0.398030
H	7.821172	-2.882736	1.568992
H	5.632108	-1.596506	1.626331
H	6.431304	-4.479837	-1.444798
C	2.450197	4.411926	0.822575

H	2.606178	4.822426	1.812232
N	4.545652	5.500838	0.129705
O	4.650293	5.919839	1.271068
O	5.340052	5.715639	-0.770775
